# Genome-Wide Analysis of Long Noncoding RNAs and Their Responses to Drought Stress in Cotton (*Gossypium hirsutum* L.)

**DOI:** 10.1371/journal.pone.0156723

**Published:** 2016-06-13

**Authors:** Xuke Lu, Xiugui Chen, Min Mu, Junjuan Wang, Xiaoge Wang, Delong Wang, Zujun Yin, Weili Fan, Shuai Wang, Lixue Guo, Wuwei Ye

**Affiliations:** 1 State Key Laboratory of Cotton Biology, Institute of Cotton Research of Chinese Academy of Agricultural Sciences, Anyang, 455000, Henan, China; 2 College of Agronomy, Xinjiang Agricultural University, Urumqi, 830052, China; Kunming University of Science and Technology, CHINA

## Abstract

Recent researches on long noncoding RNAs (lncRNAs) have expanded our horizon of gene regulation and the cellular complexity. However, the number, characteristics and expression patterns of lncRNAs remain poorly characterized and how these lncRNAs biogenesis are regulated in response to drought stress in cotton are still largely unclear. In the study, using a reproducibility-based RNA-sequencing and bioinformatics strategy to analyze the lncRNAs of 9 samples under three different environment stresses (control, drought stress and re-watering, three replications), we totally identified 10,820 lncRNAs of high-confidence through five strict steps filtration, of which 9,989 were lincRNAs, 153 were inronic lncRNAs, 678 were anti-sense lncRNAs. Coding function analysis showed 6,470 lncRNAs may have the ability to code proteins. Small RNAs precursor analysis revealed that 196 lncRNAs may be the precursors to small RNAs, most of which (35.7%, 70) were miRNAs. Expression patterns analysis showed that most of lncRNAs were expressed at a low level and most inronic lncRNAs (75.95%) had a consistent expression pattern with their adjacent protein-coding genes. Further analysis of transcriptome data uncovered that lncRNAs XLOC_063105 and XLOC_115463 probably function in regulating two adjacent coding genes CotAD_37096 and CotAD_12502, respectively. Investigations of the content of plant hormones and proteomics analysis under drought stress also complemented the prediction. We analyzed the characteristics and the expression patterns of lncRNAs under drought stress and re-watering treatment, and found lncRNAs may be likely to involve in regulating plant hormones pathway in response to drought stress.

## Introduction

The discovery of long noncoding (lnc RNAs) provides a new insight into genome regulation [1]. Generally, lncRNAs are transcripts with at least 200bp in length possessing no coding capacity, but are involved in the regulation of various biological processes, including plant growth and development, epigenetics, and the response to the stress, *etc* [2,3]. Based on the position of protein-coding genes and lnc RNAs, lnc RNAs can be classified into long intergenic noncoding RNAs (lincRNAs), long noncoding natural antisense transcripts (lncNATs), long intronic noncoding RNAs and overlapping lncRNAs [4]. Today, lncRNAs have been regarded as a cryptic, but crucial regulator in genetic regulatory code [1].

Now, the rapid advances in sequencing technology enable the identification of various RNAs possible. Studies have uncovered quite a number of noncoding RNAs in human (~61%–72%) [5,6], mouse (72%) [6], *Drosophila melanogaster* (16.8%) [7], and *Arabidopsis thaliana* (7.4%) [8]. Wang *et al*. have identified 30,550 lincRNAs and 4,718 lncNATs, and lncNATs are mainly enriched in repetitive sequences in fiber development of cotton [3]. In maize, 20,163 putative lncRNAs were identified and characterized and more than 90% were predicted to be the precursors of small RNAs [9]. In *Arabidopsis thaliana*, 6,480 lincRNAs have been characterized with custom microarrays and RNA sequencing [10]. Numerous lncRNAs have been identified in various species. For example, approximately 10,000 human lncRNAs were discovered by the GENCODE consortium [4] and some lncRNAs have been proved to be significant in influencing plant development, human disease and other biological process [11–15].

Emerging evidence supports the view that Noncoding RNAs play important roles in regulating responses to a variety of abiotic and biotic stress [16,17]. More than 1000 NAT pairs involved in response to light in seedling in a spatial and development-specific manner are found [18]. Some lincRNAs showed organ-specific and some others were responsive to biotic or abiotic stresses in *Arabidopsis thaliana* [19]. In addition, several stress-responsive lncRNAs have been functionally characterized in plant signaling pathways, e.g. *COLDAIR* [20], *COOLAIR* [21], *At4/IPS1* [22], *npc48* [23], and *npc536* [24]. However, up to now, comprehensive surveys of lncRNAs response to drought stress are still missing.

Cotton (*Gossypium* spp.) is the most important fiber crop and is also a very important oilseed crop and has been widely cultivated around the world. Besides, cotton has been regarded as a pioneer crop in the saline-alkali fields for its stronger tolerance to various stresses. Now most studies on noncoding RNAs in cotton have been limited to small RNAs, for example, Gong *et al*. have demonstrated 33 microRNA families with similar copy numbers and average evolutionary rates are conserved in the two congeneric cottons *G*. *arboretum* and *G*. *raimondii* [25]. 257 novel low-abundance miRNAs in elongating cotton fiber cells have been discovered and a potential regulatory network of nine sRNAs important for fiber elongation was revealed in cotton [26]. A number of 31 miRNA families, including 27 conserved and 4 novel miRNA families, have been characterized in developing cotton ovules with a high-throughput sequencing technology [27].

Here, in order to decipher the regulation of long non-coding RNAs in response to drought stress, we analyzed lncRNA differences under three different environment stress (control, drought and re-watering) with a new generation RNA sequencing method. We identified a total of 10,820 lncRNAs of high-confidence through five steps filtration, of which 9,989 were lincRNAs, 153 were inronic lncRNAs, 678 were anti-sense lncRNAs. Along with previous published proteomics data, we observed a fair chance of lncRNAs to regulate plant hormones in response to drought stress.

## Results and Discussion

### Identification and characterization of lncRNAs in *Gossypium hirsutum* L.

**Fig 1:**
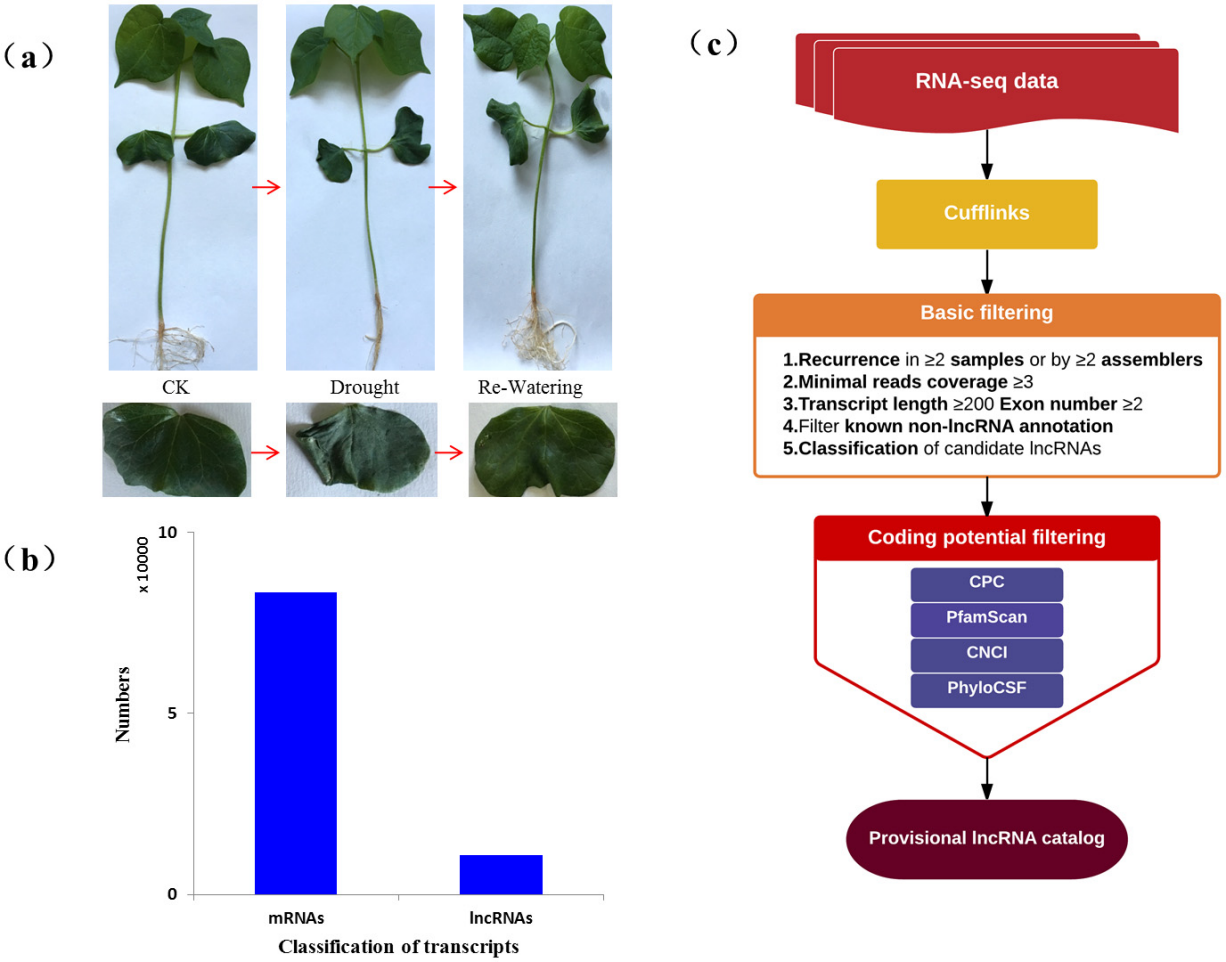
Morphological changes of seedlings after treatments and identification of long noncoding RNAs (lncRNAs). (a) Morphological changes of cotton seedlings after drought and re-watering treatments at trefoil stage. (b) The classification of transcripts by RNA-seq. (c) The Pipeline for the identification of lncRNAs in *Gossypium hirsutum* L.

The greatly improved RNA-seq technologies make it possible for us to detect the change of various RNAs in response to stresses, which could help us to better understand the regulation mechanism of RNAs. In the case of *Gossypium hirsutum* L., much work about the identification of mRNA and microRNAs has been done and a large number of RNAs data sets were available from various experiments conducted by different laboratories. However, these data sets have not yet been utilized to explore and study lncRNAs. Cotton is one of the important fiber crops with stronger stress resistance and the regulation mechanism answering the drought stress was largely unknown. Therefore, three environments, control(C), drought(D), re-watering (Re-W), were used to discover novel lncRNAs in relation to drought stress. After the treatments, the morphology of seedlings dramatically changed (Fig 1a). Under drought stress (the relative water content dropped to nearly 7%), cotyledons of the seedlings begun to grow soft while the control seedlings were still very strong. But when the drought-stressed seedlings were subjected to re-watered, the cotyledons were re-strong again. Then true leaves within different stages were sampled and used to following researches. Using a next-generation RNA sequencing strategy, we mapped the RNA-seq data to the reference genome of *Gossypium hirsutum* L. [28]. The work was conducted with three replicates (S1 Fig). Lastly, we totally discovered 83,414 transcripts and most of the transcripts (89.60%) were mRNAs (Fig 1b). Among which, 15,789 transcripts (18.93%) were annotated as novel isoforms (S1 Table). A total of 2,824 isoforms (3.39%) were annotated with Swiss-Prot database (S2 Table).

**Fig 2:**
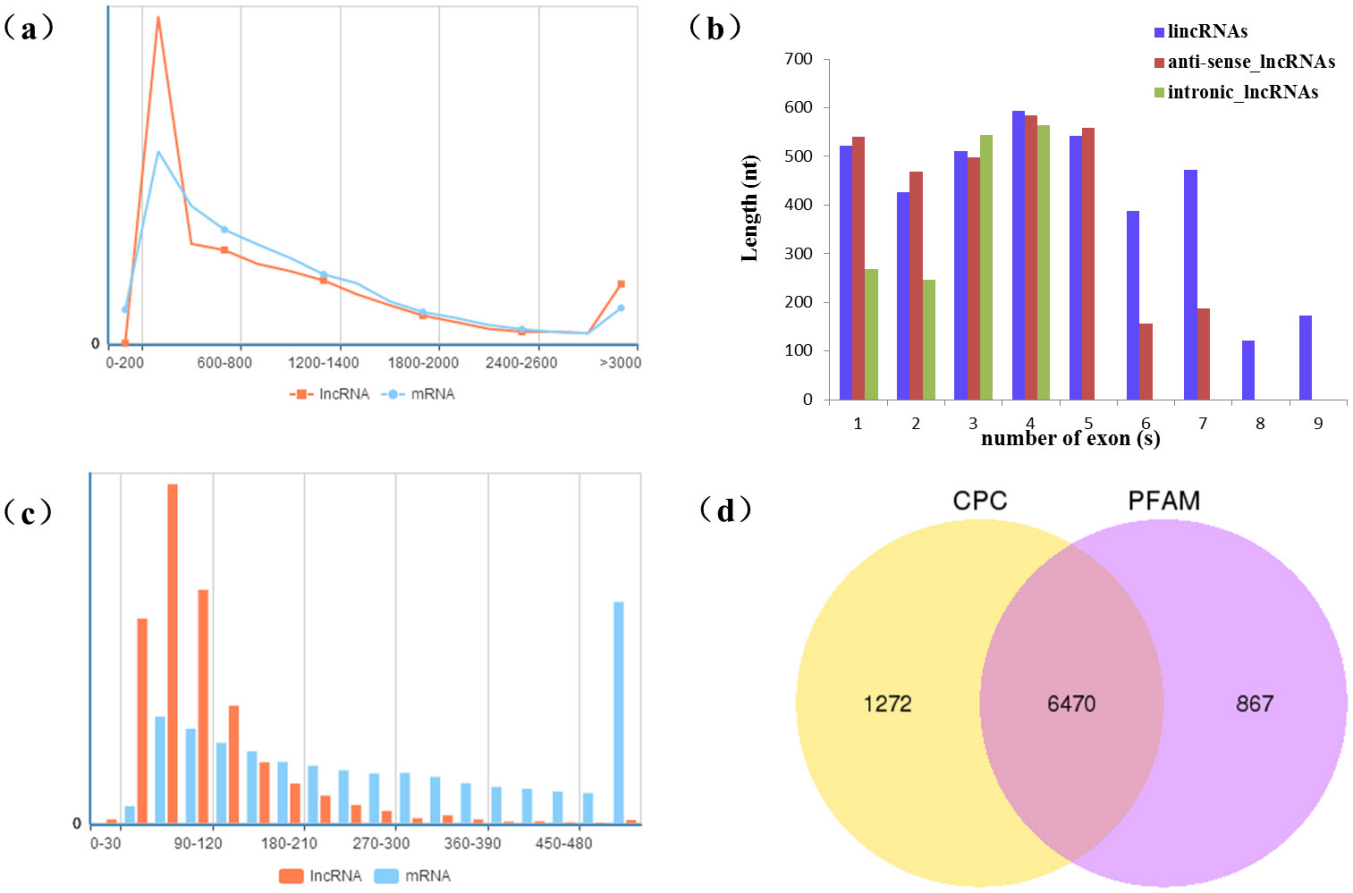
Characterization analysis of lncRNAs. (a) The length compassion analysis of lncRNA and mRNA. X axis indicates the length of lncRNAs and mRNAs (bp), while Y axis represents the frequent count. (b) The features of different types of lncRNAs, including lincRNAs, anti-sense_lncRNAs and intronic_lncRNAs. (c) The ORF compasion analysis of lncRNA and mRNA. X axis indicates the number of ORFs, while Y axis represents the frequent count. (d) Coding potential prediction of lncRNAs by CPC and PFAM software. Venn diagrams show number of lncRNAs with coding potential.

LncRNAs assembly was realized using Cufflinks in order to uncover all lncRNAs in cotton in response to drought. Five steps were conducted to retain 10,820 long non-coding RNAs of high-confidence (Fig 1c) and a great majority (92.3%) were lincRNAs. The length of lncRNAs varies from 200 to 12,057 nt with an average length of 686 nt. Attributing lncRNAs to subgenomes showed that the number of lncRNAs in At subgenome was 4,771, fewer than that in Dt genome. The exon number of each lncRNAs differs greatly from 1 to 9 with a majority was only one exon, which showed *G*. *hirsutum* L. genome encoded 46.0% single-exonic lincRNAs and 45.8% single-exonic lncNATs. We also discovered 76,943 mRNAs and the length of mRNAs varies greatly from 150 nt to 32,759 nt, slightly shorter than lncRNAs (Fig 2a). Compare the length of lincRNAs, anti-sense_lncRNAs and inronic lncRNAs (Fig 2b), we found that inronic lncRNAs containing one or two exons was typically shorter than that lincRNAs and anti-sense_lncRNAs containing the same number exons, while there was no significant difference between lincRNAs and anti-sense_lncRNAs from one to five exons except six to nine exons. From the result of ORF comparison between lncRNAs and mRNAs (Fig 2c), the ORF length of most lncRNAs was all no longer than 300 nt, while the ORF length of mRNAs were significantly longer than lncRNAs, which should be related with the coding function of mRNAs.

An overwhelming majority of lncRNAs (79.67%) may have the ability to code proteins (Fig 2d) through the prediction analysis and this observation suggests another layer of regulatory complexity on gene expression. Among all the lncRNAs identified, only a fraction (6.27%) was anti-sense lncRNAs. And more than half of those lncRNAs were forecasted to encode proteins with great possibilities, suggesting that anti-sense lncRNAs may also involve in gene regulatory network, but the specific function of anti-sense lncRNAs remains unclear and requires further investigation. In total, a number of 9,989 lincRNAs were identified, which were regarded as playing important roles in key biological processes [10].

These 10,820 lncRNAs may contain precursors to small molecules, such as miRNAs, siRNAs, tRNAs and short hairpin (shRNAs) [29]. The putative lncRNAs were predicted with rfam-scan program in rfam 11 database (Evalue≤1E-05). We totally obtained 196 lncRNAs which may be the precursors to small RNAs, of which 35.7% (70) were miRNAs and 9.7% (19) were tRNAs (S3 Table). So we speculated that these lncRNAs may function normally by degrading into miRNAs in response to drought stress.

### Characterization and expression analysis of cotton lncRNAs in response to the drought stress

**Fig 3:**
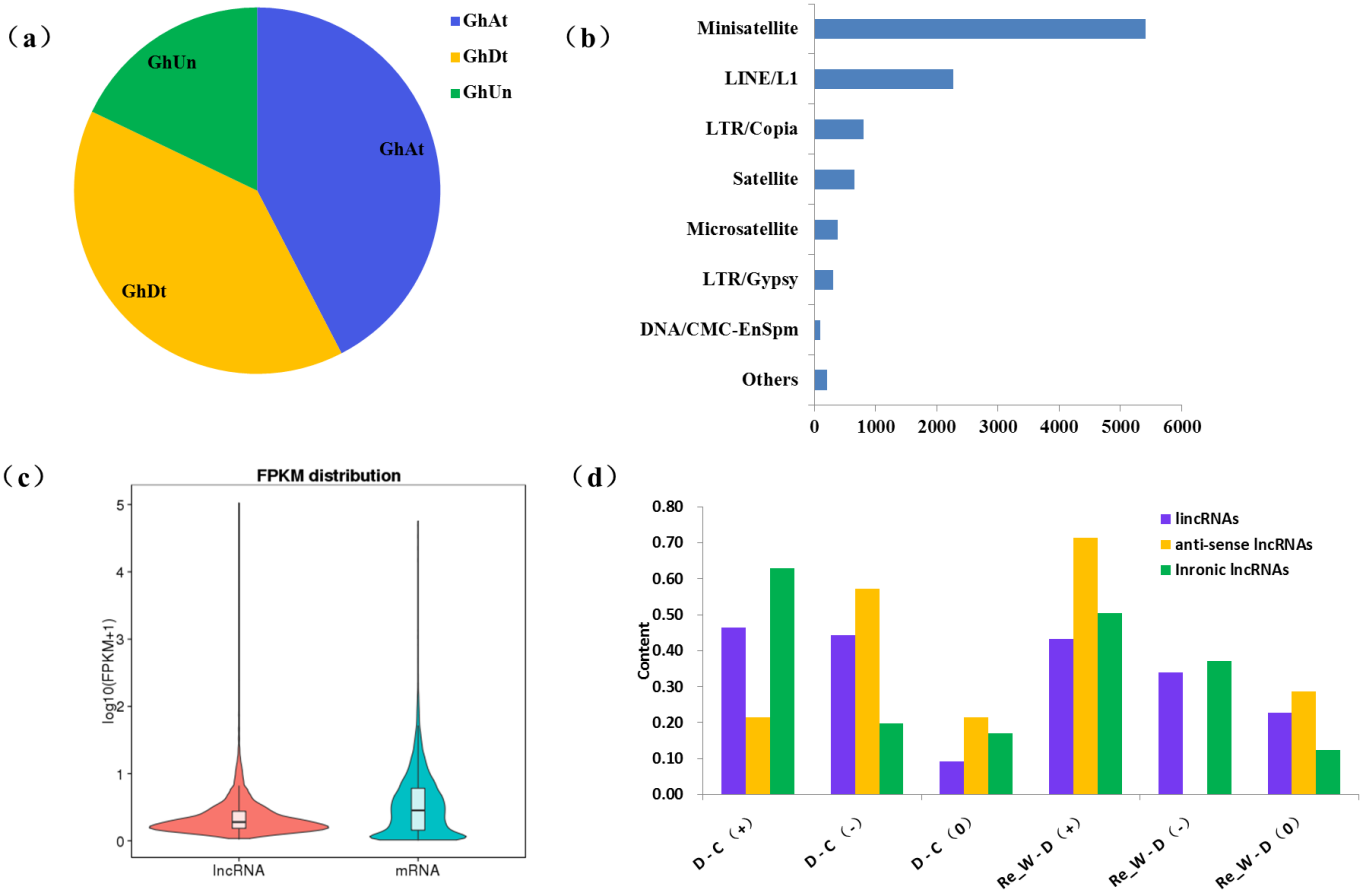
Transposable elements (TEs) prediction and expression patterns analysis of lncRNAs. (a) The results of repeat sequences and transposable elements (TEs) prediction. GhAt, GhDt and GhUn represent A subgroup, D subgroup and ungrouped, respectively. (b) The faction of lncRNAs with different characteristics. (c) Overall expression levels difference between lncRNAs and mRNAs. (d) Expression patterns analysis of different lncRNAs in response to the drought stress. D-C, Re_W-D means drought vs control, Re-Watering vs drought, respectively and +, - represents up-regulated and down-regulated, respectively.

Plant genomes were reported to be massively invaded by transposable elements (TEs), and research showed as much as 60% of the *G*. *hirsutum* genome was composed of TEs [28]. By overlapping the coordinates of lncRNAs with the repeat sequences and transposable elements (TEs) predicted with RepeatMasker software, we found approximately 53.29% of lncRNAs contained Mini-satellites, of which 42.38% in the At subgenome, 39.72% in the Dt subgenome and 17.90% in the ungrouped scaffolds (Fig 3a). The faction of LTR/Gypsy-containing lncRNAs was far less than that of LTR/Copia-containing lncRNAs (Fig 3b). It has been reported that *Copia* elements were remarkably more active than *Gypsy*, with higher proportions of *Copia* located near coding genes than *Gypsy*-type [28].

**Fig 4:**
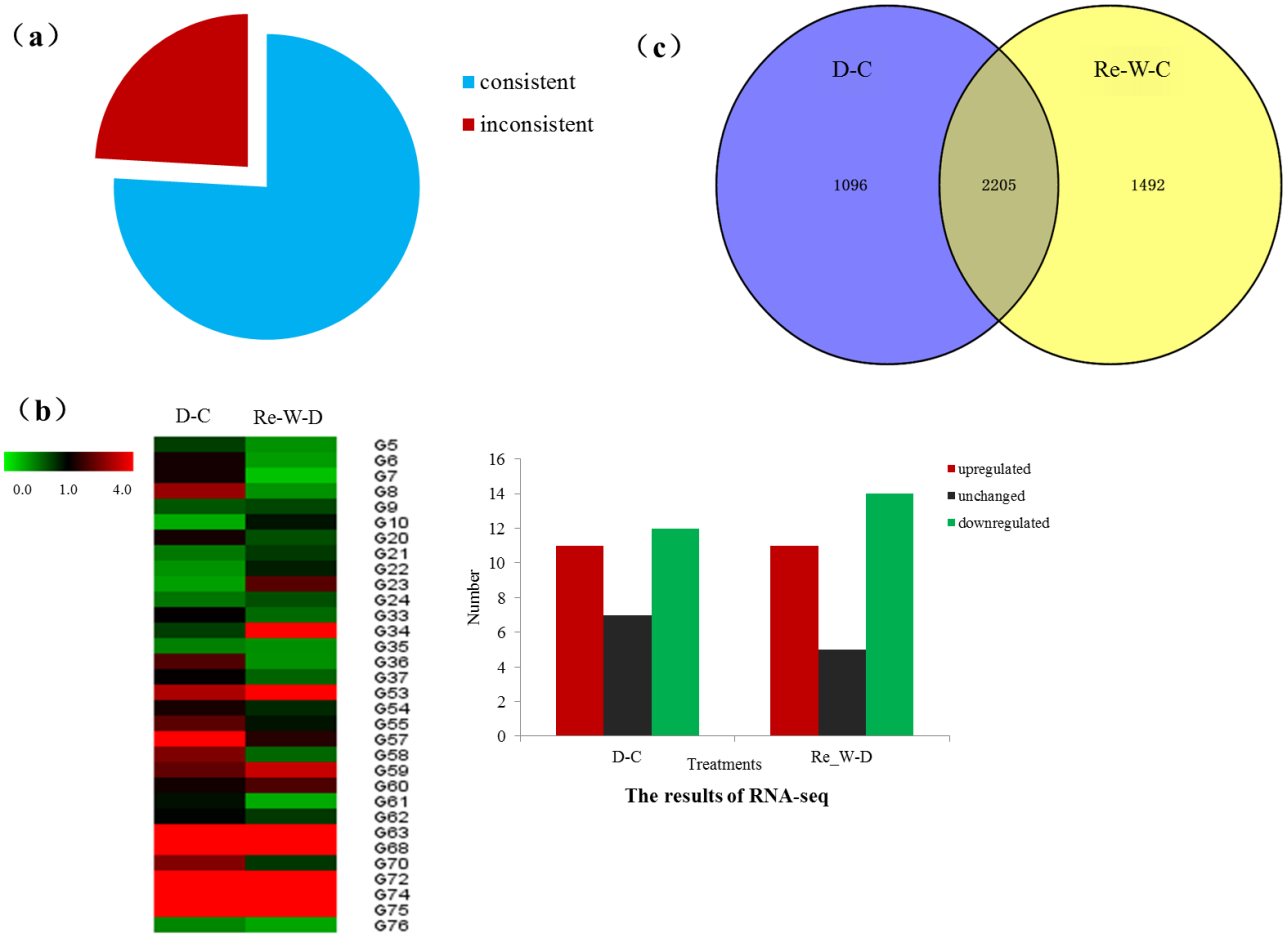
Differentially expressed lncRNAs and expression validation of lncRNAs in cotton with qRT-PCR. (a) Expression pattern analysis of lncRNAs-PCgene pairs. (b) Heatmap showed the real-time (RT)-PCR validation of the expression of 32 lncRNAs, including 16 lincRNAs, 10 anti-sense lncRNAs and 6 intronic lncRNAs. Histogram showed the FPKM value of 32 lncRNAs from the result of RNA-seq. (c) Differentially expressed genes (DEGs) analysis under drought and re-watering stress.

All transcripts identified including lncRNAs and mRNAs were used to systematically explore the expression difference in response to different environments. The results showed that overall expression difference of lncRNAs was relatively significant and most of lncRNAs were usually expressed at low levels while the expression alterations of mRNAs were uniform between the maximum and least FPKMs (Fig 3c) calculated with Cuffdiff (v2.1.1) [30], which may be connected with their specialized functions [31]. Based on the fact that different lncRNAs would have different expression patterns and various functions for their different locations in genome, we calculated the number of different lncRNAs in response to drought stress (Fig 3d). The results indicated that approximately 63% intronic lncRNAs all presented an up-regulated pattern while only about 21% anti-sense lncRNAs were up-regulated. Similar to coding-protein genes, lncRNAs also could be induced to be down-regulated by drought stress. Almost 44% lincRNAs, 57% anti-sense lncRNAs and 20% intronic lncRNAs were down-regulated in our research. But after the re-watering, nearly 71% anti-sense lncRNAs showed a re-up-regulated pattern, so we inferred that most anti-sense lncRNAs may only play a part in the process of drought and when the stress was removed, the regulating roles would disappear. Interestingly, we compared the expression pattern of inronic lncRNAs and the protein-coding genes to which intronic lncRNAs belonged, named as intronic lncRNAs-PCgene pairs (Fig 4a), we found that most of intronic lncRNAs-PCgene pairs (75.95%) have a similar expression pattern, which may be related with the locations of intronic lncRNAs (between genes) and they may function in regulating adjacent protein-coding genes.

RT-PCR was utilized to validate the expression for 32 lncRNAs (Fig 4b), including 16 lincRNAs, 10 anti-sense lncRNAs and 6 intronic lncRNAs, and the results of RT-PCR were largely consistent (30/32) with the RNA-seq data. Differentially expressed genes under drought and re-watering stress were also computed compared with control among 6,470 protein-coding lncRNAs (Fig 4c). We totally found 3,301 and 3,697 lncRNAs were up-regulated at drought stress and re-watering conditions, respectively. 2,205 lncRNAs were detected at both conditions, showing an acquired expression pattern (up-regulated) for the induction of drought. And 1,096 lncRNAs were only discovered under drought stress, when the stress was removed, these lncRNAs would recover to normal expression level as control, suggesting a drought-specific up-regulation pattern. So we speculated these lncRNAs may be closely related to the drought stress. Besides, 1,492 lncRNAs were only found after re-watering treatment, which may be the result of delayed up-regulation lured by drought stress. These results indicated that a large number of lncRNAs were expressed preferentially in response to drought stress.

### GO and KEGG enrichment analysis of lncRNAs target genes (LTGs)

**Fig 5:**
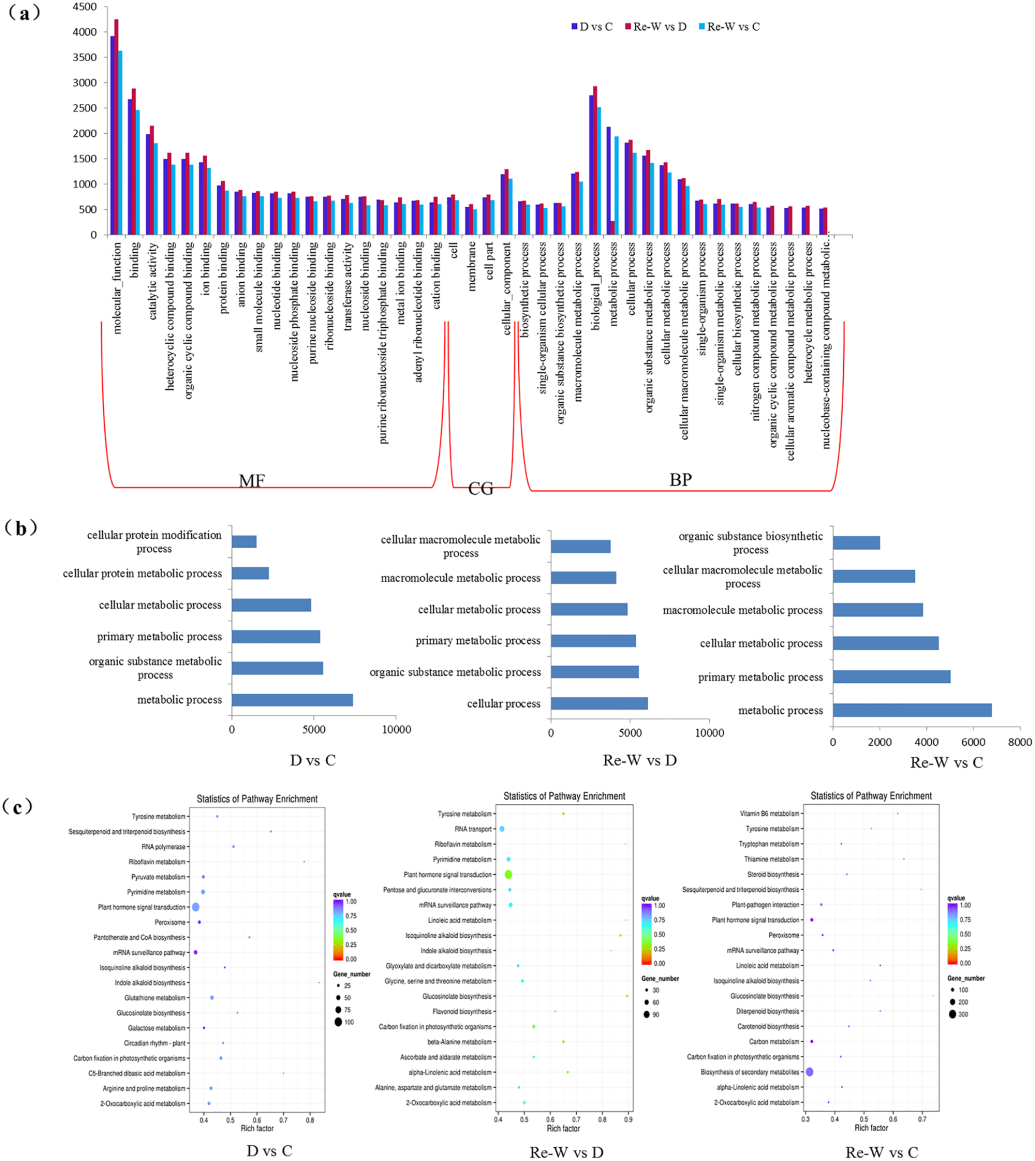
Gene ontology (GO) enrichment of lncRNAs and KEGG enrichment of lncRNA-targets. (a) The results of GO analysis based on the differently expressed lncRNAs. (b) Main enrichments of differentially expressed lncRNAs based on Gene ontology (GO) analysis. (c) Statistical KEGG enrichment of lncRNA-targets genes using KOBAS software.

Gene ontology (GO) analysis was performed for functional categorization based on differentially expressed lncRNAs under drought stress and the methods were given in “Materials and Methods” section. In this part, we investigated the lncRNA target genes enrichment by *cis*-acting or *trans*-acting. The results of *cis*-acting analysis indicated most LTGs were enriched in molecular function (molecular function, binding and catalytic activity) and biological process section (biological process, metabolic process and cellular process), while only a small number of LTGs were appeared in cellular component (cellular component, cell part, membrane and cell) (Fig 5a). We screened items by counting the number of LTGs (>500) contained in each item. Interestingly, we found that the number of LTGs in metabolic process decreased dramatically after re-watering treatment, that is to say, nearly 1,865 lncRNAs were involved in the metabolic process to regulate relevant genes. Besides, some biological processes, like organic cyclic compound metabolic process, cellular aromatic compound metabolic process, heterocycle metabolic process, anion binding, metal ion binding, cat ion binding, were all related with the concentration of chemical compound, which contributed to the osmotic potential of cell under drought stress. When the drought stress was removed (re-watering was conducted), the number of LTGs would decline, even returned to the control level, such as LTGs in the process of organic cyclic compound metabolic process, cellular aromatic compound metabolic process and heterocycle metabolic process, were found only a few LTGs between recovery and control while so much (>500) between drought and control or between recovery and drought. Another process, nucleobase-containing compound metabolic process associated with nucleobase-containing small molecular metabolic, nucleobase-containing compound biosynthetic process, nucleic acid metabolic process and genetic imprinting, was also detected to be closely related to drought stress like organic cyclic compound metabolic process, cellular aromatic compound metabolic process and heterocycle metabolic process. Based on the target genes prediction by *trans*-acting (Fig 5b), cellular metabolic process, primary metabolic process and organic substance metabolic process were discovered in different treatments, showing these lncRNAs may be conserved in the process of fundamental growth and development, and the response to the stresses.

Statistical enrichment of lncRNA targets was performed in KEGG pathways using KOBAS software (Fig 5c). Expression bias of lncRNAs was extensive in plant hormone signal transduction pathway under drought and recovery compared with control. Besides, pathways like mRNA surveillance, pyrimidine metabolic and carbon fixation in photosynthesis pathway were also found under drought stress compared to control. Bias-expressed lncRNAs in RNA transport pathway showed that the directed movement of RNA out or within a cell or between cells played a vital role after re-watering following drought stress. But compared to control, biosynthesis of secondary metabolites pathway could be induced by drought stress, which could be proved by the result that most lncRNA targets with bias expression were enriched in the pathway. The results of *trans*-acting prediction indicated protein processing in endoplasmic reticulum, mRNA surveillance, purine/pyrimidine metabolism, RNA transport and ribosome biogenesis were main pathways related to drought stress (S2 Fig). Genes encoding cell components and various plant hormones such as auxin, ABA and ETH, always serve as regulators of plant growth and the responses to stresses [32]. Therefore, we concluded that lncRNAs with bias expression were enriched in various pathways by *cis*-acting or *trans*-acting, but mainly focused on plant hormone signal transduction, mRNA surveillance, protein biogenesis and processing, RNA transport and purine/pyrimidine metabolism, which played a prominent role in response to drought stress.

### Functional lncRNA candidates and their targets in response to drought stress

**Fig 6:**
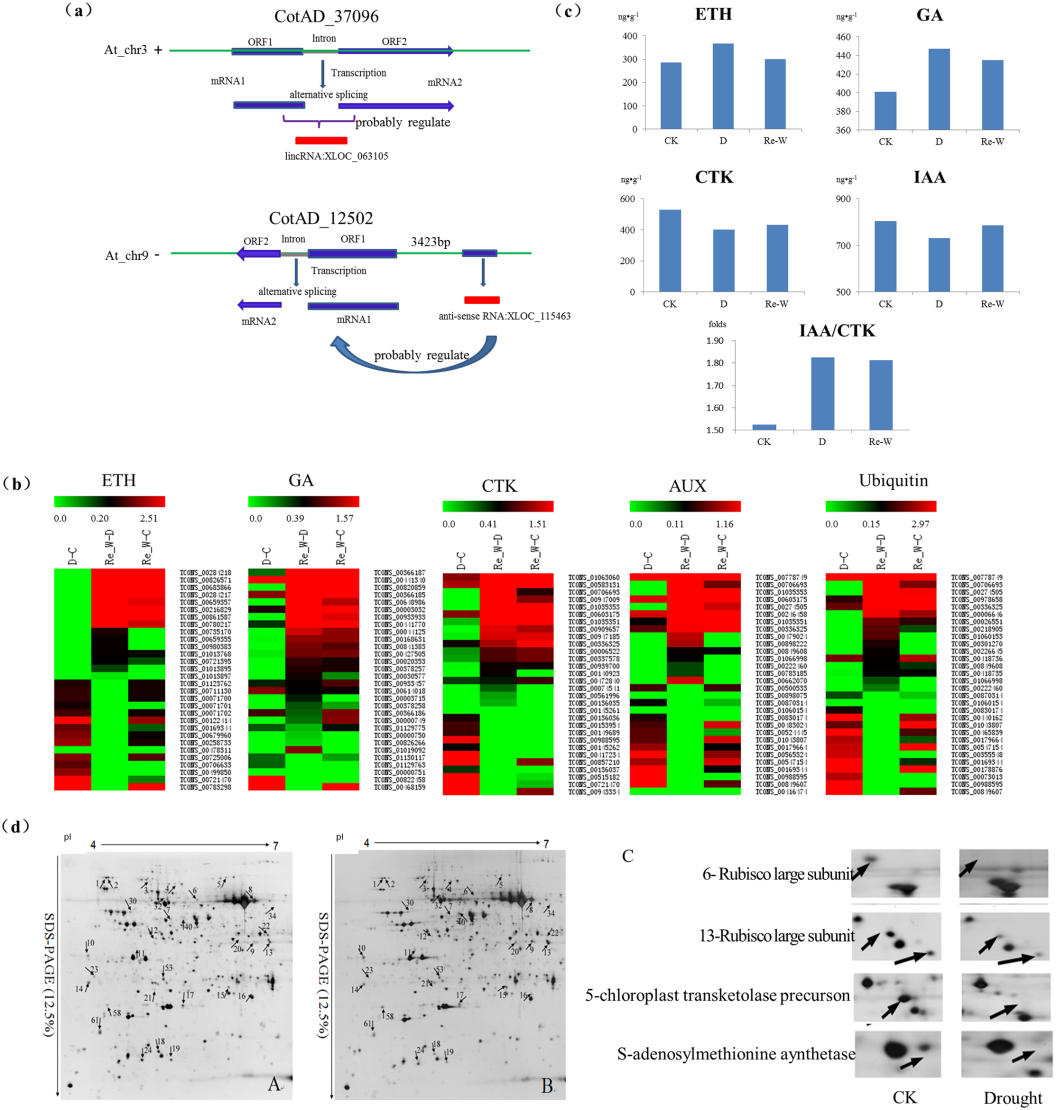
Regulation mechanism prediction of functional lncRNAs predicted by *cis*-acting and *tran*s-acting and relative analysis. (a) Regulation mechanism prediction of functional lncRNA-targets predicted by *cis*-acting and *trans*-acting. (b) The expression analysis of 30 randomly selected lncRNAs associated with each kind of hormones. (c) The content variations of plant hormones under drought stress and re-watering treatment. (d) The 2-DE image of total protein in leaves of ZhongH177 at trefoil stage and several differentially expressed proteins were enlarged. A represents proteins in ZhongH177-CK, B represents ZhongH177-Drought and C represents the enlargement of differentially expressed proteins.

Cotton is the world’s most important fiber crop and a model polyploid crop [33,34], and also a pioneer crop for its strong resistance to adversity. The process of responding to adversity in plants is complex that depends upon types of adversity, duration of adversity, developmental stage of plant and time of a day [35], involving multiple pathways of biological process. In the research, targets prediction of functional candidates was conducted and numerous lnRNA-mRNA pairs of different function annotations were identified with *cis*-acting or *trans*-acting (S4 Table). Based on the enrichment analysis of differentially expressed genes, we found plant hormone signal transduction was closely related to drought stress. In total, we found 9,247,20,28 lncRNAs were associated with ethylene (ETH), auxin (IAA), gibberellin (GA), cytokinin (CTK) by *cis*-acting, respectively. In ETH metabolic pathway, interestingly we found two lncRNAs XLOC_063105 and XLOC_115463 probably function in regulating two adjacent coding genes CotAD_37096 and CotAD_12502, respectively (Fig 6a). Of which, XLOC_063105, a type of lincRNA, was transcribed together with the localized gene at the same expression level. After the transcription, alternative splicing worked to make gene CotAD_37096 into two mRNAs. The effect of adjacent location also helped confirm the regulation between lncRNA XLOC_063105 and gene CotAD_37096. Another lncRNA XLOC_115463, a type of anti-sense RNA, was localized at—strand in At_chr9, may playing an vital role in regulating its adjacent genes in—strand in the same chromosome. In *Arabidopsis*, approximately 70% of annotated mRNAs were found to associated with antisense transcripts [18]. Therefore, we speculated that anti-sense lncRNA XLOC_115463 may also regulate cotton gene CotAD_12502 or some other genes in a way. Besides, we also identified approximately 407 lncRNAs linked with ubiquitin, which may take part in signal transduction and the degradation of protein in response to stress. 4

By *trans*-acting, about 167, 3,086, 239, 334 and 2,621 lncRNA-target pairs were associated with ethylene (ETH), auxin (IAA), gibberellin (GA), cytokinin (CTK) and ubiquitin, respectively. To investigate the function of these lncRNAs and the putative functional lncRNAs regulating hormones levels in response to drought stress, the expression of 30 randomly selected lncRNAs associated with each kind of hormones have been identified was determined (Fig 6b). Big expression differences of these lncRNAs were found between drought and control, re-watering and drought, re-watering and control, which showed these lncRNAs all played a significant role in the process. We also observed the great content variations of plant hormones under drought stress and re-watering treatments (Fig 6c).

By lncRNA-target prediction, we also discovered 15 and 186 lncRNA-targets associated with photosynthesis by *cis*-acting and *trans*-acting, respectively and most of these lncRNAs were about Rubisco subunits. Comparative analysis of proteomics in upland cotton leaves previously conducted in our lab showed Rubisco enzyme was a protein complexes closely related to the photosynthesis in chloroplast and could be induced to partial degradation under drought stress (Fig 6d). So we speculated that drought stress could affect the photosynthesis of leaves by the lncRNA-mediated degradation of photosynthesis-related Rubisco enzyme.

## Discussions

Understanding the mechanism of gene regulation will provide molecular basis for the resistance research of cotton, contributing to make cotton better adapt to drought stress. One of the biggest surprises of the post-genome era is the vast amount of transcription emanating from the noncoding regions of the genome. The existence of non-coding genes, including short non-coding genes (such as small interfering RNAs and miRNAs) and long non-protein coding genes revealed the complexity of genome expression. However, the short non-protein coding RNAs were relatively well characterized and their important roles in transcriptional and post-transcriptional regulation of other genes was well understood [36]. Long non-coding RNAs (lncRNAs, 200bp or longer and non-protein coding RNAs) has been emerged as new advent in recent years. Increasing numbers of functional lncRNAs identified, together with functional protein-coding genes and small noncoding RNAs are revealing the high level of complexity of eukaryotic transcriptomes [37]. In contrast, lncRNAs have not been comprehensively identified or studied in many plant species, especially in cotton. In the research, drought-resistant upland cotton ZhongH177 was used to identify drought-related lncRNAs under three different environment stresses (CK, drought and re-watering) with a new generation RNA sequencing method. Based on five strict screen criteria, we totally identified a number of 10,820 high-confidence lncRNAs, of which 9,989 were lincRNAs, 153 were inronic lncRNAs, 678 were anti-sense lncRNAs, which were far less than Wang *et al*. [38], which may be due to the rigorous filtration criteria we used to identify lncRNAs. In view of not quite clear functions of lncRNAs, coding potential of lncRNAs was conducted and approximately 6,470 lncRNAs were found to have the potential to code proteins.

Our analysis generated a relatively robust list of potential lncRNAs for cotton, which will likely be useful for functional genomics research or the functional difference analysis among cotton varieties. The lncRNAs including lincRNAs, inronic lncRNAs, anti-sense lncRNAs, were identified with different numbers of exons and varying length of ORF in the research, which may be due to their locations in the genome and different specialized functions. We have provided annotation files as supplemental tables (S5 Table) to enable the use and display of these lncRNAs by other researchers. Our study also analyzed the expression patterns of lncRNAs, and we found most lncRNAs were expressed at low levels compared with mRNAs. Based on the special functions of lncRNAs, selective transcription and alternative splicing probably worked to generate different expressions of lncRNAs and mRNAs. LncRNAs do not appear to encode proteins, but could often result in functional RNA molecules, and the regulation mechanism is frequently sequence homology dependent [39]. In the research, 196 lncRNAs were identified as the precursors to small RNAs, of which 35.7% (70) were miRNAs and 9.7% (19) were tRNAs and others were other RNAs. Additionally, a distinctive pathway in plants utilizing lncRNAs through RNAs has been recently discovered, also confirmed by RNA molecules pathway statistically enriched with differentially expressed lncRNAs. Our study sheds light on the features and expression patterns of lncRNAs in cotton, and also complements the reference genome annotation of cotton, which might further aid the research of functional lncRNAs and trigger more comprehensive studies on gene regulation in cotton.

One important class of noncoding RNAs is lncRNAs generated from the opposite strand of coding or noncoding genes, the so-called anti-sense lncRNAs. In the study, we found most anti-sense lncRNAs showed a down-regulated pattern compared to the control, but represented a re-up-regulated pattern after the re-watering. This observation indicated these lncRNAs may be very important to the gene regulation in response to drought stress. Studies documented showed that anti-sense lncRNAs were involved in regulating responses to various abiotic stresses [16,17,40]. Anti-sense lncRNAs have been shown to deploy diverse mechanisms to regulate the expression of sense transcripts at the transcriptional or post-transcriptional level. Previous studies reported that anti-sense lncRNAs-directed chromatin remodeling at target loci has emerged as an important mode of action at transcriptional level [41–43]. Histone modifications have also been shown to be important for plant development and responses to various stresses [44,45]. According to the huge changes of anti-sense lncRNAs pre- and post- drought stress, we speculated that these anti-sense lncRNAs may involve in the gene regulation by the ways of chromatin modeling, histone modifications or some other mechanisms.

GO and KEGG enrichment analysis of lncRNAs target genes (LTGs) could help us understand the functions of lncRNAs effectively. In the study, we found metabolic process, RNA molecules, proteins/amino acid metabolism, plant hormones metabolism were closely related with drought stress. Plant hormones regulate numerous growth, developmental process and responses to various stresses [46]. Based on the prediction, we obtained some lncRNAs (in the results section), which may be the regulators of genes regulating plant hormones. Under drought stress, the content of gibberellin acid (GA) and ethylene (ETH) increased, higher than that in control, this could stimulate the synthesis of proteins and nucleic acid to change the utilization of water, contributing to the resistance to drought stress. The content of cytokinin (CTK) and auxin (IAA), were found decreased in some degree. Plants need reduce their energy and growth regulating substances demanded in the process to resist various stresses. The ratio of IAA/CTK was slightly higher under drought stress compared with control, and the increase of IAA could stimulate the growth and extension of roots, which could help plants absorb more water from sand. 191 lncRNAs-targets were found by prediction and most targets were about the subunits of Rubisco enzymes, a protein complex regulating plant photosynthesis by controlling CO_2_ fixation and emission. Rubisco enzymes could be partially degraded in response to drought stress, which was confirmed by our previous proteomics analysis. So we speculated that lncRNAs-directed Rubisco degradation would be a helpful way to resist drought stress for cotton. Although we have identified 10,820 lncRNAs, it is likely that additional cotton lncRNAs exit, which will be discovered with creative, diverse and collaborative multifaceted approaches.

## Materials and Methods

### Plant material, growth conditions and drought treatment

Drought-tolerant upland cotton ZhongH177 seeds were sterilized with 0.1% HgCl_2_ and placed in a sterile culture dish to accelerate germination. Cotton seedlings of uniform size were selected and transplanted into a sand container (10 seedlings per container) in the greenhouse (14 h/day, 30°C and 10 h/night, 24°C) of the Institute of Cotton Research of Chinese Academy of Agricultural Sciences. At trefoil stage, drought stress was performed by withholding watering till the relative water content (RWC) reaches to about 7% in pots and drooping effects on plant leaves became evident, while the control pots were watered as before. Then the second and third true leaves on each plant were harvested, snap frozen with liquid nitrogen, and stored at –80°C until use.

### RNA isolation, library construction and sequencing

High-quality total RNA was extracted as the method reported by Zhao *et al*.[47]. RNA degradation and contamination was monitored on 1% agarose gels and RNA purity was checked using the NanoPhotometer^®^ spectrophotometer (IMPLEN, CA, USA). RNA concentration was measured using Qubit^®^ RNA Assay Kit in Qubit^®^ 2.0 Flurometer (Life Technologies, CA, USA) and RNA integrity was assessed using the RNA Nano 6000 Assay Kit of the Bioanalyzer 2100 system (Agilent Technologies, CA, USA).

A total amount of 3 μg RNA per sample was used as input material for the RNA sample preparations. Firstly, ribosomal RNA was removed by Epicentre Ribo-zero^™^ rRNA Removal Kit (Epicentre, USA), and rRNA free residue was cleaned up by ethanol precipitation. Subsequently, sequencing libraries were generated using the rRNA-depleted RNA by NEBNext^®^ Ultra^™^ Directional RNA Library Prep Kit for Illumina^®^ (NEB, USA) following manufacturer’s recommendations. Briefly, fragmentation was carried out using divalent cations under elevated temperature in NEBNext First Strand Synthesis Reaction Buffer (5X). First strand cDNA was synthesized using random hexamer primer and M-MuLV Reverse Transcriptase (RNaseH-). Second strand cDNA synthesis was subsequently performed using DNA polymerase I and RNase H. In the reaction buffer, dNTPswith dTTP were replaced by dUTP. Remaining overhangs were converted into blunt ends via exonuclease/ polymerase activities. After adenylation of 3’ ends of DNA fragments, NEBNext Adaptor with hairpin loop structure were ligated to prepare for hybridization. In order to select cDNA fragments of preferentially 150~200 bp in length, the library fragments were purified with AMPure XP system (Beckman Coulter, Beverly, USA). Then 3μL USER Enzyme (NEB,USA) was used with size-selected, adaptor-ligated cDNA at 37°C for 15 min followed by 5 min at 95°C before PCR. Then PCR was performed with Phusion High-Fidelity DNA polymerase, Universal PCR primers and Index (X) Primer. At last, products were purified (AMPure XP system) and library quality was assessed on the Agilent Bioanalyzer 2100 system.

The clustering of the index-coded samples was performed on a cBot Cluster Generation System using TruSeq PE Cluster Kit v3-cBot-HS (Illumia) according to the manufacturer’s instructions. After cluster generation, the libraries were sequenced on an Illumina Hiseq 2000 platform and 100 bp paired-end reads were generated.

### Raw data quality control and Mapping to the reference genome

Raw data (raw reads) of fastq format were firstly processed through in-house perl scripts. In this step, clean data (clean reads) were obtained by removing reads containing adapter, reads on containing ploy-N and low quality reads from raw data. At the same time, Q20, Q30 and GC content of the clean data were calculated. All the downstream analyses were based on the clean data with high quality. Reference genome and gene model annotation files were downloaded from genome website (http://cgp.genomics.org.cn/page/species/index.jsp) directly. Index of the reference genome was built using Bowtie v2.0.6 and paired-end clean reads were aligned to the reference genome using TopHat v2.0.9. Sequencing work and raw data analysis were conducted by Novogene (Beijing).

### Transcriptome assembly

The mapped reads of each sample were assembled by both Scripture (beta2) [48] and Cufflinks (v2.1.1) [49] in a reference-based approach. Both methods use spliced reads to determine exons connectivity, but with two different approaches. Scripture uses a statistical segmentation model to distinguish expressed loci from experimental noise and uses spliced reads to assemble expressed segments. It reports all statistically expressed isoforms in a given locus. Cufflinks uses a probabilistic model to simultaneously assemble and quantify the expression level of a minimal set of isoforms that provides a maximum likelihood explanation of the expression data in a given locus [50]. Scripture was run with default parameters, Cufflinks was run with ‘min-frags-per-transfrag = 0’ and ‘—library-type’, other parameters were set as default.

### Coding potential analysis

CPC (Coding Potential Calculator) (0.9-r2) mainly through assess the extent and quality of the ORF in a transcript and search the sequences with known protein sequence database to clarify the coding and non-coding transcripts [51]. We used the NCBI eukaryotes' protein database and set the e-value ‘1e-10’in our analysis. We translated each transcript in all three possible frames and used Pfam Scan (v1.3) to identify occurrence of any of the known protein family domains documented in the Pfam database (release 27; used both Pfam A and Pfam B) [52]. Any transcript with a Pfam hit would be excluded in following steps. Pfam searches use default parameters of -E 0.001—domE 0.001[53].

### Conservative analysis

Phast (v1.3) is a software package containing much of statistical programs and most were used in phylogenetic analysis [54], and phastCons is a conservation scoring and identification program of conserved elements. We used phyloFit to compute phylogenetic models for conserved and non-conserved regions among species and then gave the model and HMM transition parameters to phastConsto compute a set of conservation scores of lncRNA and coding genes.

### Target gene prediction

Cis-role is lncRNA acting on neighboring target genes. We searched coding genes 10k/100k upstream and downstream of lncRNA and then analyzed their function next. Trans-role is lncRNA to identify each other by the expression level. While there were no more than 25 samples, we calculated the expressed correlation between lncRNAs and coding genes with custom scripts; otherwise, we clustered the genes from different samples with WGCNA [55] to search common expression modules and then analyzed their function through functional enrichment analysis.

### Quantification of gene expression level

Cuffdiff (v2.1.1) was used to calculate FPKMs of both lncRNAs and coding genes in each sample [30]. Gene FPKMs were computed by summing the FPKMs of transcripts in each gene group. FPKM means fragments per kilo-base of exon per million fragments mapped, calculated based on the length of the fragments and reads count mapped to this fragment.

### Differential expression analysis

Cuffdiff provides statistical routines for determining differential expression in digital transcript or gene expression data using a model based on the negative binomial distribution [30]. For biological replicates, transcripts or genes with an P-adjust < 0.05 were assigned as differentially expressed. For non-biological replicates, P-adjust < 0.05 and the absolute value of log_2_ (fold change) < 1 were set as the threshold for significantly differential expression.

### GO and KEGG enrichment analysis

Gene Ontology (GO) enrichment analyses of differentially expressed genes or lncRNA target genes were implemented by the GO seq R package, in which gene length bias was corrected. GO terms with corrected P-value less than 0.05 were considered significantly enriched by differential expressed genes.

KEGG is a database resource for understanding high-level functions and utilities of the biological system, such as the cell, the organism and the ecosystem, from molecular-level information, especially large-scale molecular datasets generated by genome sequencing and other high-throughput experimental technologies (http://www.genome.jp/kegg/). We used KOBAS software to test the statistical enrichment of differential expression genes or lncRNAs target genes in KEGG pathways.

### PPI analysis

PPI analysis of differentially expressed genes was based on the STRING database, which known and predicted Protein-Protein Interactions. For the species existing in the database, we construct the networks by extract the target gene list from the database; Otherwise, Blastx (v2.2.28) was used to align the target gene sequences to the selected reference protein sequences, and then the networks was built according to the known interaction of selected reference species.

### Alternative splicing analysis and SNP analysis

Alternative splicing events were classified to 12 basic types by the software Asprofile v1.0. The number of AS events in each sample was estimated, separately. Picard-tools v1.96 and samtools v0.1.18 were used to sort, mark duplicated reads and reorder the bam alignment results of each sample. GATK2 software was used to perform SNP calling.

### Determination of plant hormones in cotton

Plant hormones ethylene (ETH), auxin (IAA), gibberellin (GA), cytokinin (CTK) was measured with enzyme-linked immunosorbent assay (ELISA). 1.0g samples were used to conduct the experiment and the main steps were follows: grinding the samples, centrifugation, extraction, constant volume, antigen coating, board-washing, competition, board-washing again, adding the second antibody, developing and measurement. ELISA kits were purchased from Shanghai Enzyme-linked Biotechnology. Each kind of hormones was determined with three replications.

## Supporting Information

S1 Fig Three replicates for RNA-sequencing. (PNG)

S2 Fig Statistics analysis of pathway enrichment by trans-acting prediction. (PNG)

S1 Table Annotation of novel isoforms (PDF). (PDF)

S2 Table Isoforms were annotated with Swiss-Prot database (PDF). (PDF)

S3 Table Precursors prediction of small RNA molecules (PDF). (PDF)

S4 Table Targets of lncRNAs predicted by cis-acting and trans-acting (XLS). (XLSX)

S5 Table Annotation for lncRNAs identified. (GTF)
