# Supplementary figures and images for: Genome-Wide Analysis of Long Noncoding RNAs and Their Responses to Drought Stress in Cotton (Gossypium hirsutum L.)
Source: PLoS One. 2016 Jun 13;11(6):e0156723. doi: 10.1371/journal.pone.0156723 (PMC4905672; doi:10.1371/journal.pone.0156723)

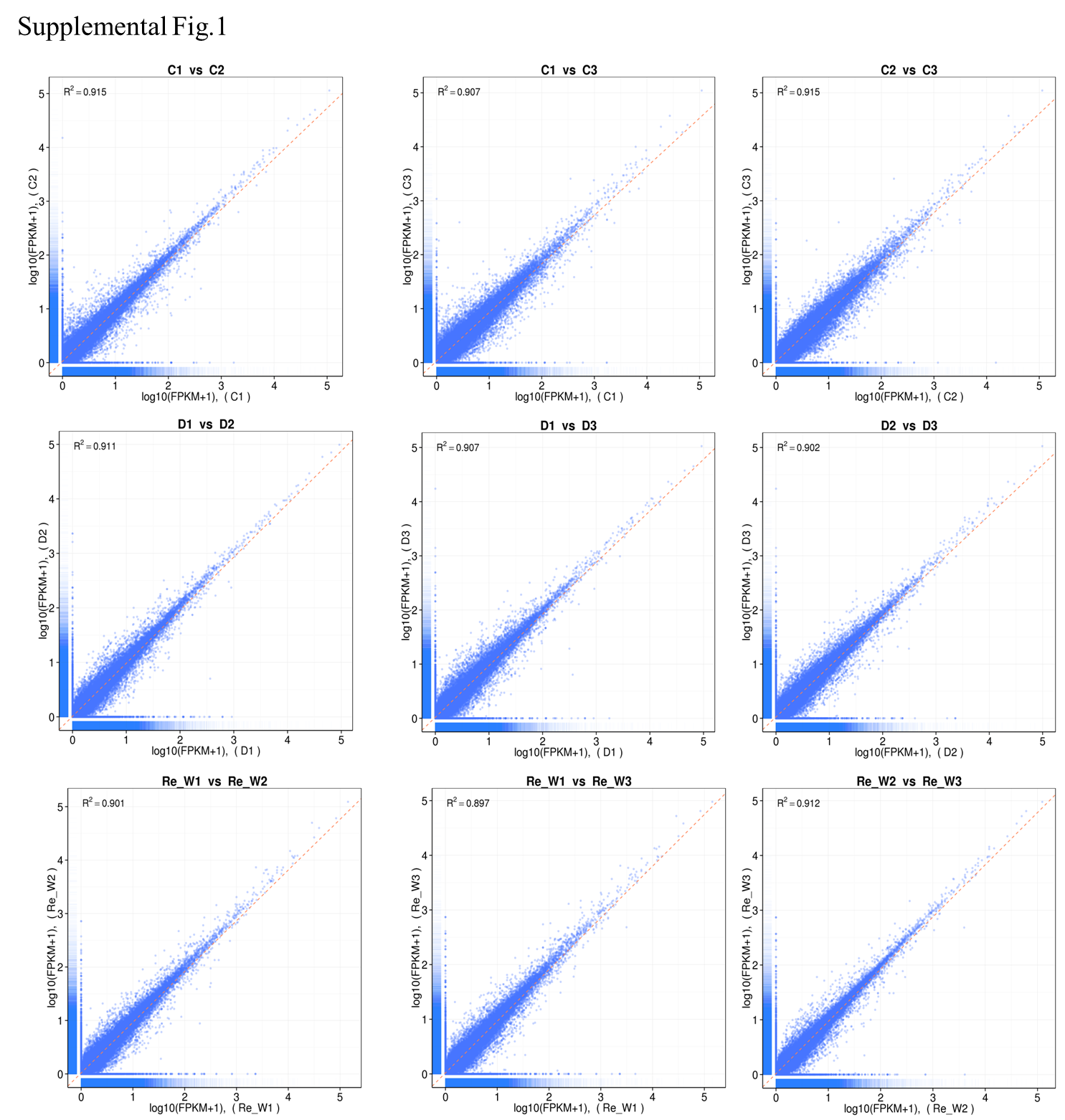

Supplement: S1 Fig — (PNG) [file pone.0156723.s001.png]

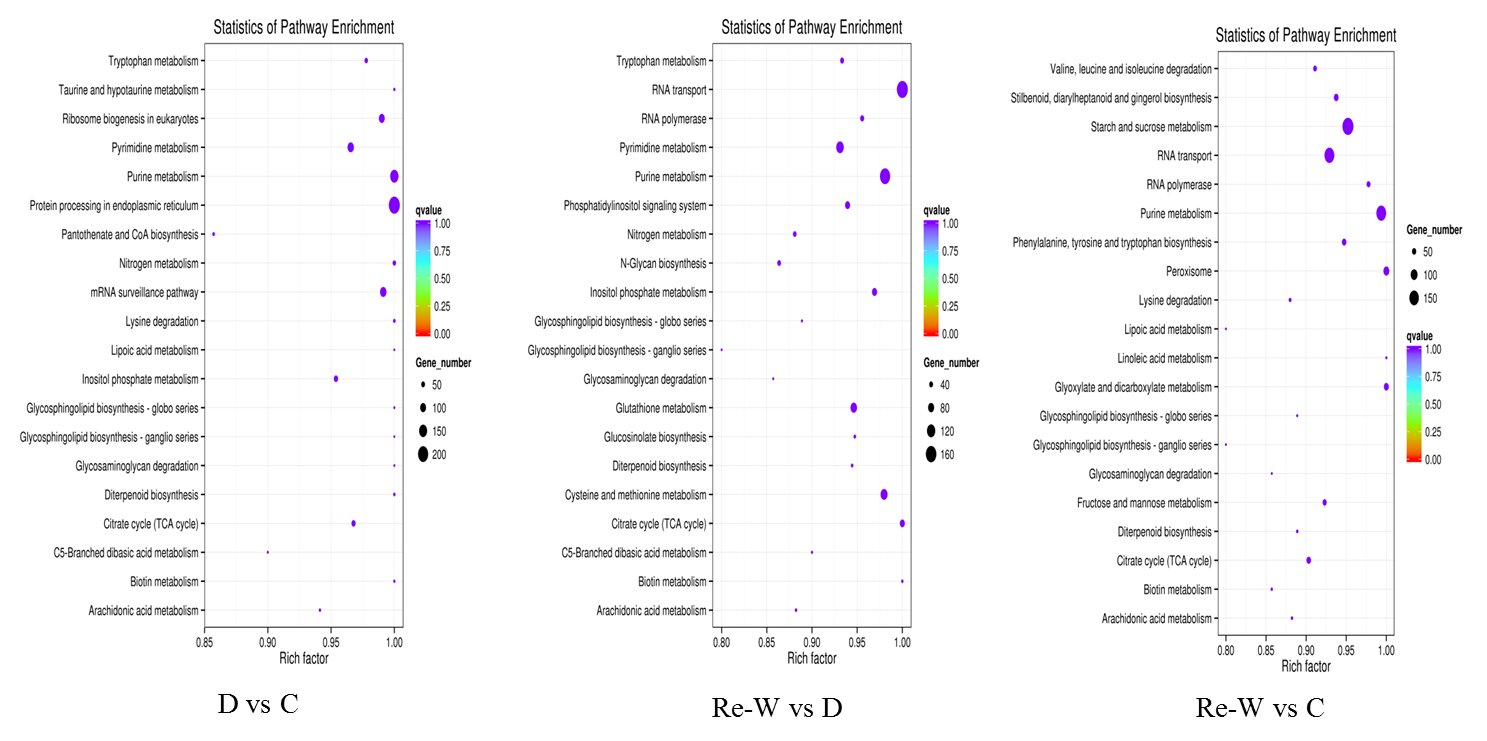

Supplement: S2 Fig — (PNG) [file pone.0156723.s002.png]
