# Supplementary material for: Genome-Wide Analysis of Long Noncoding RNAs and Their Responses to Drought Stress in Cotton (Gossypium hirsutum L.)
Source: PLoS One. 2016 Jun 13;11(6):e0156723. doi: 10.1371/journal.pone.0156723 (PMC4905672; doi:10.1371/journal.pone.0156723)
Supplement: S2 Table — (PDF) [file pone.0156723.s004.pdf]

S2 Table Isoforms were annotated with Swiss-Prot database

<table>
<tr><th>Gene_id</th><th>Blast swiss prot</th></tr>
<tr><td>XLOC_0000</td><td>sp|C0LGT6|EFR_ARATH LRR receptor-like serine/threonine-protein kinase EFR OS=Arabidopsis thaliana GN=EFR PE=1 SV=1//4</td></tr>
<tr><td>XLOC_0000</td><td>-//-</td></tr>
<tr><td>XLOC_0000</td><td>-//-</td></tr>
<tr><td>XLOC_0001</td><td>sp|Q9FJI1|AAK6_ARATH Adenylate kinase isoenzyme 6 OS=Arabidopsis thaliana GN=AAK6 PE=1 SV=1//1.93463e-75</td></tr>
<tr><td>XLOC_0001</td><td>sp|Q08069|RS8_MAIZE 40S ribosomal protein S8 OS=Zea mays GN=RPS8 PE=2 SV=2//1.12816e-116</td></tr>
<tr><td>XLOC_0001</td><td>sp|Q93ZB9|FACR3_ARATH Fatty acyl-CoA reductase 3 OS=Arabidopsis thaliana GN=FAR3 PE=2 SV=1//1.43466e-76</td></tr>
<tr><td>XLOC_0001</td><td>sp|Q9ZVI6|LOR8_ARATH Protein LURP-one-related 8 OS=Arabidopsis thaliana GN=At2g38640 PE=2 SV=1//1.95763e-59</td></tr>
<tr><td>XLOC_0002</td><td>sp|P04323|POL3_DROME Retrovirus-related Pol polyprotein from transposon 17.6 OS=Drosophila melanogaster GN=pol PE=4 S</td></tr>
<tr><td>XLOC_0002</td><td>-//-</td></tr>
<tr><td>XLOC_0002</td><td>sp|Q9SY75|PPR30_ARATH Putative pentatricopeptide repeat-containing protein At1g10330 OS=Arabidopsis thaliana GN=PCMP-</td></tr>
<tr><td>XLOC_0002</td><td>sp|Q9SE43|REV_ARATH Homeobox-leucine zipper protein REVOLUTA OS=Arabidopsis thaliana GN=REV PE=1 SV=2//5.87213e-16</td></tr>
<tr><td>XLOC_0002</td><td>sp|C0LGP9|IMK3_ARATH Probable leucine-rich repeat receptor-like protein kinase IMK3 OS=Arabidopsis thaliana GN=IMK3 P</td></tr>
<tr><td>XLOC_0003</td><td>-//-</td></tr>
<tr><td>XLOC_0003</td><td>sp|O04350|TBCA_ARATH Tubulin-specific chaperone A OS=Arabidopsis thaliana GN=At2g30410 PE=1 SV=2//3.10261e-23</td></tr>
<tr><td>XLOC_0003</td><td>sp|Q9SZD5|LRK59_ARATH L-type lectin-domain containing receptor kinase V.9 OS=Arabidopsis thaliana GN=LECRK59 PE=2 SV=</td></tr>
<tr><td>XLOC_0004</td><td>sp|O82500|Y4117_ARATH Putative disease resistance protein At4g11170 OS=Arabidopsis thaliana GN=At4g11170 PE=2 SV=1//6</td></tr>
<tr><td>XLOC_0004</td><td>sp|Q40392|TMVRN_NICGU TMV resistance protein N OS=Nicotiana glutinosa GN=N PE=1 SV=1//4.85908e-66</td></tr>
<tr><td>XLOC_0004</td><td>sp|Q40392|TMVRN_NICGU TMV resistance protein N OS=Nicotiana glutinosa GN=N PE=1 SV=1//8.30539e-30</td></tr>
<tr><td>XLOC_0005</td><td>-//-</td></tr>
<tr><td>XLOC_0005</td><td>sp|C0LGN2|Y3148_ARATH Probable leucine-rich repeat receptor-like serine/threonine-protein kinase At3g14840 OS=Arabido</td></tr>
<tr><td>XLOC_0005</td><td>sp|C0LGE0|Y1765_ARATH Probable LRR receptor-like serine/threonine-protein kinase At1g07650 OS=Arabidopsis thaliana GN</td></tr>
<tr><td>XLOC_0005</td><td>sp|O65493|XCP1_ARATH Xylem cysteine proteinase 1 OS=Arabidopsis thaliana GN=XCP1 PE=1 SV=1//1.13329e-98</td></tr>
<tr><td>XLOC_0005</td><td>sp|Q9AUR8|COPA1_ORYSJ Coatomer subunit alpha-1 OS=Oryza sativa subsp. japonica GN=Os03g0711400 PE=2 SV=1//1.41985e-41</td></tr>
<tr><td>XLOC_0005</td><td>sp|C0LGN2|Y3148_ARATH Probable leucine-rich repeat receptor-like serine/threonine-protein kinase At3g14840 OS=Arabido</td></tr>
<tr><td>XLOC_0006</td><td>sp|Q9H063|MAF1_HUMAN Repressor of RNA polymerase III transcription MAF1 homolog OS=Homo sapiens GN=MAF1 PE=1 SV=2//2.</td></tr>
<tr><td>XLOC_0006</td><td>sp|Q8GY61|BH063_ARATH Transcription factor bHLH63 OS=Arabidopsis thaliana GN=BHLH63 PE=2 SV=1//2.81342e-21</td></tr>
<tr><td>XLOC_0006</td><td>-//-</td></tr>
<tr><td>XLOC_0006</td><td>-//-</td></tr>
<tr><td>XLOC_0006</td><td>sp|P08770|TRA1_MAIZE Putative AC transposase OS=Zea mays PE=2 SV=2//7.39915e-101</td></tr>
<tr><td>XLOC_0006</td><td>sp|Q9SX38|DRL4_ARATH Putative disease resistance protein At1g50180 OS=Arabidopsis thaliana GN=At1g50180 PE=2 SV=2//5.</td></tr>
<tr><td>XLOC_0006</td><td>sp|Q9SX38|DRL4_ARATH Putative disease resistance protein At1g50180 OS=Arabidopsis thaliana GN=At1g50180 PE=2 SV=2//6.</td></tr>
<tr><td>XLOC_0006</td><td>sp|Q9FID6|Y5392_ARATH Probable receptor-like protein kinase At5g39020 OS=Arabidopsis thaliana GN=At5g39020 PE=2 SV=1/</td></tr>
<tr><td>XLOC_0006</td><td>sp|Q9LHN8|F6H1_ARATH Feruloyl CoA ortho-hydroxylase 1 OS=Arabidopsis thaliana GN=F6'H1 PE=1 SV=1//8.40698e-87</td></tr>
</table>

XLOC_0006 sp|Q9S7I6|RPK2_ARATH LRR receptor-like serine/threonine-protein kinase RPK2 OS=Arabidopsis thaliana GN=RPK2 PE=1 SV=1
XLOC_0008 sp|Q40392|TMVRN_NICGU TMV resistance protein N OS=Nicotiana glutinosa GN=N PE=1 SV=1//9.04209e-123
XLOC_0008 -//-
XLOC_0008 sp|O80492|P2C05_ARATH Probable protein phosphatase 2C 5 OS=Arabidopsis thaliana GN=At1g09160 PE=2 SV=1//2.02849e-112
XLOC_0009 sp|P93293|M300_ARATH Uncharacterized mitochondrial protein AtMg00300 OS=Arabidopsis thaliana GN=AtMg00300 PE=4 SV=1//
XLOC_0010 sp|Q8GXK5|EDL14_ARATH Sugar transporter ERD6-like 14 OS=Arabidopsis thaliana GN=At4g04750 PE=2 SV=2//3.45616e-13
XLOC_0012 -//-
XLOC_0012 sp|Q9SQZ9|PITH1_ARATH PITH domain-containing protein At3g04780 OS=Arabidopsis thaliana GN=At3g04780 PE=1 SV=2//3.5594
XLOC_0013 sp|Q41387|PSBW_SPIOL Photosystem II reaction center W protein, chloroplastic OS=Spinacia oleracea GN=psbW PE=1 SV=1//
XLOC_0015 sp|Q9LYT3|TT12_ARATH Protein TRANSPARENT TESTA 12 OS=Arabidopsis thaliana GN=TT12 PE=2 SV=1//1.41997e-79
XLOC_0015 sp|P81392|MYB06_ANTMA Myb-related protein 306 OS=Antirrhinum majus GN=MYB306 PE=2 SV=1//6.04934e-76
XLOC_0017 -//-
XLOC_0017 -//-
XLOC_0018 -//-
XLOC_0020 sp|Q08747|UAF30_YEAST Upstream activation factor subunit UAF30 OS=Saccharomyces cerevisiae (strain ATCC 204508 / S288
XLOC_0024 -//-
XLOC_0025 sp|Q8LB81|GDL79_ARATH GDSL esterase/lipase At5g33370 OS=Arabidopsis thaliana GN=At5g33370 PE=2 SV=1//0
XLOC_0029 sp|Q2QNM3|PUS1_ORYSJ RNA pseudourine synthase 1 OS=Oryza sativa subsp. japonica GN=Os12g0560500 PE=2 SV=1//1.48239e-1
XLOC_0032 sp|P36907|CHIX_PEA Endochitinase OS=Pisum sativum PE=2 SV=1//2.67389e-140
XLOC_0032 sp|Q9LX20|ASPL1_ARATH Aspartic proteinase-like protein 1 OS=Arabidopsis thaliana GN=At5g10080 PE=1 SV=1//2.39723e-14
XLOC_0036 -//-
XLOC_0036 -//-
XLOC_0036 -//-
XLOC_0037 -//-
XLOC_0038 sp|P92792|TOM20_SOLTU Mitochondrial import receptor subunit TOM20 OS=Solanum tuberosum GN=TOM20 PE=1 SV=1//3.52687e-1
XLOC_0040 sp|P42802|INO1_CITPA Inositol-3-phosphate synthase OS=Citrus paradisi PE=3 SV=1//9.85967e-104
XLOC_0044 -//-
XLOC_0045 sp|Q943I6|STOP1_ORYSJ Zinc finger protein STOP1 homolog OS=Oryza sativa subsp. japonica GN=Os01g0871200 PE=2 SV=1//5.
XLOC_0049 sp|Q9SVB7|WRK13_ARATH Probable WRKY transcription factor 13 OS=Arabidopsis thaliana GN=WRKY13 PE=2 SV=1//4.17883e-42
XLOC_0053 -//-
XLOC_0054 sp|Q93Y94|RPOT1_NICSY DNA-directed RNA polymerase 1, mitochondrial OS=Nicotiana sylvestris GN=RPOT1 PE=2 SV=1//1.4229
XLOC_0055 sp|P08770|TRA1_MAIZE Putative AC transposase OS=Zea mays PE=2 SV=2//3.93543e-31
XLOC_0055 sp|Q09353|SENP_CAEEL Sentrin-specific protease OS=Caenorhabditis elegans GN=ulp-1 PE=2 SV=3//1.15447e-10
XLOC_0057 sp|Q94F47|UBC28_ARATH Ubiquitin-conjugating enzyme E2 28 OS=Arabidopsis thaliana GN=UBC28 PE=2 SV=1//2.08606e-103
XLOC_0057 -//-

XLOC_0057 sp|Q9LY77|ACA12_ARATH Calcium-transporting ATPase 12, plasma membrane-type OS=Arabidopsis thaliana GN=ACA12 PE=2 SV=1
XLOC_0058 sp|Q9LUW0|LOX5_ARATH Linoleate 9S-lipoxygenase 5, chloroplastic OS=Arabidopsis thaliana GN=LOX5 PE=1 SV=2//1.38067e-9
XLOC_0058 sp|Q8RXE8|BASS3_ARATH Probable sodium/metabolite cotransporter BASS3, chloroplastic OS=Arabidopsis thaliana GN=BASS3
XLOC_0058 sp|P38416|LOXB_SOLLC Linoleate 9S-lipoxygenase B OS=Solanum lycopersicum GN=LOX1.2 PE=2 SV=1//6.58458e-18
XLOC_0058 sp|Q6UNT2|RL5_CUCSA 60S ribosomal protein L5 OS=Cucumis sativus GN=RPL5 PE=2 SV=1//2.03385e-21
XLOC_0058 sp|Q9ZUW3|PP172_ARATH Pentatricopeptide repeat-containing protein At2g27610 OS=Arabidopsis thaliana GN=PCMP-H60 PE=2
XLOC_0059 -//-
XLOC_0059 sp|Q9LEL6|6OMT_COPJA (RS)-norcoclaurine 6-O-methyltransferase OS=Coptis japonica PE=1 SV=1//8.18996e-11
XLOC_0059 sp|Q9FL28|FLS2_ARATH LRR receptor-like serine/threonine-protein kinase FLS2 OS=Arabidopsis thaliana GN=FLS2 PE=1 SV=1
XLOC_0059 sp|O50003|RL12_PRUAR 60S ribosomal protein L12 OS=Prunus armeniaca GN=RPL12 PE=2 SV=1//2.10468e-61
XLOC_0060 sp|Q9SKN5|ARFJ_ARATH Auxin response factor 10 OS=Arabidopsis thaliana GN=ARF10 PE=2 SV=1//9.0081e-90
XLOC_0060 -//-
XLOC_0060 sp|Q9SL29|CNG15_ARATH Putative cyclic nucleotide-gated ion channel 15 OS=Arabidopsis thaliana GN=CNGC15 PE=2 SV=1//2.
XLOC_0060 sp|Q9SL29|CNG15_ARATH Putative cyclic nucleotide-gated ion channel 15 OS=Arabidopsis thaliana GN=CNGC15 PE=2 SV=1//6.
XLOC_0060 sp|Q40392|TMVRN_NICGU TMV resistance protein N OS=Nicotiana glutinosa GN=N PE=1 SV=1//3.21547e-23
XLOC_0060 sp|Q40392|TMVRN_NICGU TMV resistance protein N OS=Nicotiana glutinosa GN=N PE=1 SV=1//9.24308e-43
XLOC_0061 sp|Q9SZ67|WRK19_ARATH Probable WRKY transcription factor 19 OS=Arabidopsis thaliana GN=WRKY19 PE=2 SV=1//4.61048e-14
XLOC_0061 sp|Q40392|TMVRN_NICGU TMV resistance protein N OS=Nicotiana glutinosa GN=N PE=1 SV=1//2.05793e-24
XLOC_0061 -//-
XLOC_0061 sp|Q40392|TMVRN_NICGU TMV resistance protein N OS=Nicotiana glutinosa GN=N PE=1 SV=1//1.44641e-94
XLOC_0061 sp|Q40392|TMVRN_NICGU TMV resistance protein N OS=Nicotiana glutinosa GN=N PE=1 SV=1//1.65526e-11
XLOC_0061 sp|Q04062|RPN9_YEAST 26S proteasome regulatory subunit RPN9 OS=Saccharomyces cerevisiae (strain ATCC 204508 / S288c)
XLOC_0062 sp|Q40392|TMVRN_NICGU TMV resistance protein N OS=Nicotiana glutinosa GN=N PE=1 SV=1//5.00223e-120
XLOC_0062 -//-
XLOC_0062 sp|Q40392|TMVRN_NICGU TMV resistance protein N OS=Nicotiana glutinosa GN=N PE=1 SV=1//3.10402e-27
XLOC_0062 sp|P10978|POLX_TOBAC Retrovirus-related Pol polyprotein from transposon TNT 1-94 OS=Nicotiana tabacum PE=2 SV=1//9.83
XLOC_0063 sp|C0LGP4|Y3475_ARATH Probable LRR receptor-like serine/threonine-protein kinase At3g47570 OS=Arabidopsis thaliana GN
XLOC_0063 sp|Q9FZ59|PEPR2_ARATH Leucine-rich repeat receptor-like protein kinase PEPR2 OS=Arabidopsis thaliana GN=PEPR2 PE=1 SV
XLOC_0063 sp|Q9C9H7|RLP12_ARATH Receptor-like protein 12 OS=Arabidopsis thaliana GN=RLP12 PE=2 SV=2//5.99818e-115
XLOC_0064 -//-
XLOC_0064 sp|Q9LUC3|MPK19_ARATH Mitogen-activated protein kinase 19 OS=Arabidopsis thaliana GN=MPK19 PE=2 SV=2//0
XLOC_0064 sp|Q9LUD7|CALS8_ARATH Putative callose synthase 8 OS=Arabidopsis thaliana GN=CALS8 PE=2 SV=2//1.95313e-77
XLOC_0064 -//-
XLOC_0065 -//-
XLOC_0065 sp|Q8BG09|T184B_MOUSE Transmembrane protein 184B OS=Mus musculus GN=Tmem184b PE=2 SV=1//3.80116e-65

XLOC_0066-//-
XLOC_0067-//-
XLOC_0068-//-
XLOC_0069sp|Q9SAK5|APL_ARATH Myb family transcription factor APL OS=Arabidopsis thaliana GN=APL PE=2 SV=2//3.25882e-17
XLOC_0071-//-
XLOC_0074sp|Q9AT00|AB13I_ARATH Protein TRIGALACTOSYLDIACYLGLYCEROL 3, chloroplastic OS=Arabidopsis thaliana GN=TGD3 PE=1 SV=1/
XLOC_0074sp|Q9CSU0|RPR1B_MOUSE Regulation of nuclear pre-mRNA domain-containing protein 1B OS=Mus musculus GN=Rprd1b PE=1 SV=2
XLOC_0074sp|O64668|PSNA_ARATH Presenilin-like protein At1g08700 OS=Arabidopsis thaliana GN=At1g08700 PE=2 SV=1//1.44269e-176
XLOC_0076sp|P00054|CYC_SESIN Cytochrome c OS=Sesamum indicum PE=1 SV=1//4.40323e-71
XLOC_0081-//-
XLOC_0083sp|Q651X6|CSLE6_ORYSJ Cellulose synthase-like protein E6 OS=Oryza sativa subsp. japonica GN=CSLE6 PE=2 SV=1//2.00798e
XLOC_0083sp|P51418|R18A2_ARATH 60S ribosomal protein L18a-2 OS=Arabidopsis thaliana GN=RPL18AB PE=1 SV=2//2.52381e-46
XLOC_0084-//-
XLOC_0086sp|P08770|TRA1_MAIZE Putative AC transposase OS=Zea mays PE=2 SV=2//4.35641e-11
XLOC_0088sp|Q8GWL2|LOR7_ARATH Protein LURP-one-related 7 OS=Arabidopsis thaliana GN=At2g30270 PE=2 SV=1//2.40375e-26
XLOC_0089sp|Q8VZT0|NLAL1_ARATH Putative H/ACA ribonucleoprotein complex subunit 1-like protein 1 OS=Arabidopsis thaliana GN=At
XLOC_0090sp|P10978|POLX_TOBAC Retrovirus-related Pol polyprotein from transposon TNT 1-94 OS=Nicotiana tabacum PE=2 SV=1//3.23
XLOC_0091sp|Q7XA86|ZDH11_ARATH Probable S-acyltransferase At3g51390 OS=Arabidopsis thaliana GN=At3g51390 PE=2 SV=1//3.67563e-1
XLOC_0091-//-
XLOC_0091-//-
XLOC_0091sp|Q35638|RHO1_PEA Rac-like GTP-binding protein RHO1 OS=Pisum sativum GN=RHO1 PE=2 SV=1//4.76948e-89
XLOC_0093sp|Q6FWI1|GYP7_CANGA GTPase-activating protein GYP7 OS=Candida glabrata (strain ATCC 2001 / CBS 138 / JCM 3761 / NBRC
XLOC_0094sp|Q7LHG5|YI31B_YEAST Transposon Ty3-I Gag-Pol polyprotein OS=Saccharomyces cerevisiae (strain ATCC 204508 / S288c) G
XLOC_0096sp|Q84R21|Y1559_ARATH DUF21 domain-containing protein At1g55930, chloroplastic OS=Arabidopsis thaliana GN=CBSDUFCH2 P
XLOC_0096sp|Q9LK64|AB3C_ARATH ABC transporter C family member 3 OS=Arabidopsis thaliana GN=ABCC3 PE=1 SV=1//6.16473e-07
XLOC_0096sp|Q9LK64|AB3C_ARATH ABC transporter C family member 3 OS=Arabidopsis thaliana GN=ABCC3 PE=1 SV=1//6.15934e-09
XLOC_0096sp|Q10KF0|PSA2_ORYSJ Proteasome subunit alpha type-2 OS=Oryza sativa subsp. japonica GN=PAB1 PE=2 SV=1//3.28924e-47
XLOC_0096sp|O48923|C71DA_SOYBN Cytochrome P450 71D10 OS=Glycine max GN=CYP71D10 PE=2 SV=1//3.72293e-117
XLOC_0096sp|B6SFA4|MAA3_ARATH Probable helicase MAGATAMA 3 OS=Arabidopsis thaliana GN=MAA3 PE=2 SV=1//1.12561e-07
XLOC_0097-//-
XLOC_0097-//-
XLOC_0097-//-
XLOC_0102sp|Q8S1Z1|UTP11_ORYSJ Probable U3 small nucleolar RNA-associated protein 11 OS=Oryza sativa subsp. japonica GN=Os01g0
XLOC_0102sp|P10978|POLX_TOBAC Retrovirus-related Pol polyprotein from transposon TNT 1-94 OS=Nicotiana tabacum PE=2 SV=1//9.93
XLOC_0106-//-

XLOC_0106 -//-
XLOC_0106 sp|Q9SXA1|P4KA1_ARATH Phosphatidylinositol 4-kinase alpha OS=Arabidopsis thaliana GN=PI4KALPHA1 PE=1 SV=2//8.35573e-2
XLOC_0108 sp|Q10137|SEC14_SCHPO Sec14 cytosolic factor OS=Schizosaccharomyces pombe (strain 972 / ATCC 24843) GN=sec14 PE=2 SV=
XLOC_0108 sp|P10978|POLX_TOBAC Retrovirus-related Pol polyprotein from transposon TNT 1-94 OS=Nicotiana tabacum PE=2 SV=1//5.13
XLOC_0110 sp|Q9XIQ7|FBT7_ARATH Probable folate-biopterin transporter 7 OS=Arabidopsis thaliana GN=At1g64890 PE=2 SV=1//1.76642e
XLOC_0111 sp|Q9C104|GDE1_SCHPO Glycerophosphodiester phosphodiesterase gde1 OS=Schizosaccharomyces pombe (strain 972 / ATCC 248
XLOC_0112 sp|P27572|NU4M_WHEAT NADH-ubiquinone oxidoreductase chain 4 OS=Triticum aestivum GN=ND4 PE=2 SV=2//2.1306e-32
XLOC_0113 -//-
XLOC_0113 sp|Q40392|TMVRN_NICGU TMV resistance protein N OS=Nicotiana glutinosa GN=N PE=1 SV=1//5.58841e-96
XLOC_0114 sp|Q8L7H1|FBL75_ARATH F-box/LRR-repeat protein At4g14103 OS=Arabidopsis thaliana GN=At4g14103 PE=2 SV=1//1.87904e-29
XLOC_0119 -//-
XLOC_0120 sp|P83877|TXN4A_MOUSE Thioredoxin-like protein 4A OS=Mus musculus GN=Txnl4a PE=2 SV=1//8.77141e-14
XLOC_0121 sp|Q8GWP0|MYB39_ARATH Transcription factor MYB39 OS=Arabidopsis thaliana GN=MYB39 PE=2 SV=1//3.76479e-50
XLOC_0122 sp|Q2L8Y7|RR16_GOSHI 30S ribosomal protein S16, chloroplastic OS=Gossypium hirsutum GN=rps16 PE=3 SV=1//9.73126e-38
XLOC_0126 sp|O64702|HMG14_ARATH High mobility group B protein 14 OS=Arabidopsis thaliana GN=HMGB14 PE=2 SV=2//9.85449e-07
XLOC_0126 sp|P04323|POL3_DROME Retrovirus-related Pol polyprotein from transposon 17.6 OS=Drosophila melanogaster GN=pol PE=4 S
XLOC_0127 sp|Q9FVC1|SVP_ARATH MADS-box protein SVP OS=Arabidopsis thaliana GN=SVP PE=1 SV=1//9.41402e-13
XLOC_0127 -//-
XLOC_0127 sp|O82244|ALG3_ARATH Dol-P-Man:Man(5)GlcNAc(2)-PP-Dol alpha-1,3-mannosyltransferase OS=Arabidopsis thaliana GN=ALG3 P
XLOC_0127 -//-
XLOC_0130 sp|Q05654|RTF21_SCHPO Retrotransposable element Tf2 155 kDa protein type 1 OS=Schizosaccharomyces pombe (strain 972 /
XLOC_0131 sp|Q6R2J8|SRF8_ARATH Protein STRUBBELIG-RECEPTOR FAMILY 8 OS=Arabidopsis thaliana GN=SRF8 PE=2 SV=1//5.00937e-61
XLOC_0135 sp|P93306|NDUS2_ARATH NADH dehydrogenase [ubiquinone] iron-sulfur protein 2 OS=Arabidopsis thaliana GN=NAD7 PE=1 SV=2
XLOC_0135 sp|P14680|YAK1_YEAST Dual specificity protein kinase YAK1 OS=Saccharomyces cerevisiae (strain ATCC 204508 / S288c) GN
XLOC_0135 sp|O65351|SUBL_ARATH Subtilisin-like protease OS=Arabidopsis thaliana GN=ARA12 PE=1 SV=1//1.40332e-07
XLOC_0136 sp|Q9C5H6|CSTR3_ARATH CMP-sialic acid transporter 3 OS=Arabidopsis thaliana GN=UTR6 PE=2 SV=1//1.61448e-21
XLOC_0137 -//-
XLOC_0138 -//-
XLOC_0141 -//-
XLOC_0142 sp|Q8LBV4|Y1814_ARATH Uncharacterized methyltransferase At1g78140, chloroplastic OS=Arabidopsis thaliana GN=At1g78140
XLOC_0144 -//-
XLOC_0147 sp|O80366|ARR9_ARATH Two-component response regulator ARR9 OS=Arabidopsis thaliana GN=ARR9 PE=1 SV=1//1.43635e-12
XLOC_0148 sp|Q0INZ4|SPS3_ORYSJ Probable solanesyl-diphosphate synthase 3, chloroplastic (Fragment) OS=Oryza sativa subsp. japon
XLOC_0149 sp|Q949Y3|PPA26_ARATH Bifunctional purple acid phosphatase 26 OS=Arabidopsis thaliana GN=PAP26 PE=1 SV=1//2.19659e-13
XLOC_0149 sp|P08770|TRA1_MAIZE Putative AC transposase OS=Zea mays PE=2 SV=2//1.87459e-75

XLOC_0150-//-
XLOC_0151sp|P53632|PAP2_YEAST Poly(A) RNA polymerase protein 2 OS=Saccharomyces cerevisiae (strain ATCC 204508 / S288c) GN=PAP
XLOC_0152sp|P0C2F6|RNHX1_ARATH Putative ribonuclease H protein At1g65750 OS=Arabidopsis thaliana GN=At1g65750 PE=3 SV=1//2.201
XLOC_0152sp|Q6NQK2|NAC8_ARATH NAC domain-containing protein 8 OS=Arabidopsis thaliana GN=NAC008 PE=2 SV=1//5.70739e-68
XLOC_0153-//-
XLOC_0154sp|Q6DBP8|GAT11_ARATH GATA transcription factor 11 OS=Arabidopsis thaliana GN=GATA11 PE=2 SV=1//1.32204e-46
XLOC_0154sp|Q9SKN3|CSPL6_ARATH CASP-like protein At2g28370 OS=Arabidopsis thaliana GN=At2g28370 PE=2 SV=1//6.21616e-85
XLOC_0154sp|Q6K215|PIP22_ORYSJ Probable aquaporin PIP2-2 OS=Oryza sativa subsp. japonica GN=PIP2-2 PE=2 SV=1//5.1345e-157
XLOC_0154sp|F4K0E8|ISPG_ARATH 4-hydroxy-3-methylbut-2-en-1-yl diphosphate synthase, chloroplastic OS=Arabidopsis thaliana GN=I
XLOC_0154sp|Q10N21|APX1_ORYSJ L-ascorbate peroxidase 1, cytosolic OS=Oryza sativa subsp. japonica GN=APX1 PE=1 SV=1//1.23177e-
XLOC_0154sp|Q6ZRS2|SRCAP_HUMAN Helicase SRCAP OS=Homo sapiens GN=SRCAP PE=1 SV=3//2.0933e-11
XLOC_0154sp|Q6ICB0|DESI1_HUMAN Desumoylating isopeptidase 1 OS=Homo sapiens GN=DESI1 PE=1 SV=1//2.40015e-11
XLOC_0158sp|Q9SSQ4|FRS6_ARATH Protein FAR1-RELATED SEQUENCE 6 OS=Arabidopsis thaliana GN=FRS6 PE=2 SV=1//0
XLOC_0158sp|Q9LJI5|VA0D1_ARATH V-type proton ATPase subunit d1 OS=Arabidopsis thaliana GN=VHA-D1 PE=2 SV=1//4.53314e-11
XLOC_0160sp|Q9SSB8|CX5B2_ARATH Cytochrome c oxidase subunit 5b-2, mitochondrial OS=Arabidopsis thaliana GN=COX5B-2 PE=2 SV=1//
XLOC_0160sp|Q9SJJ5|CKS2_ARATH Cyclin-dependent kinases regulatory subunit 2 OS=Arabidopsis thaliana GN=CKS2 PE=2 SV=1//2.93914
XLOC_0160-//-
XLOC_0163-//-
XLOC_0163sp|Q6NPP4|CMTA2_ARATH Calmodulin-binding transcription activator 2 OS=Arabidopsis thaliana GN=CMTA2 PE=1 SV=1//6.0023
XLOC_0166sp|Q9SJA6|RZ22A_ARATH Serine/arginine-rich splicing factor RSZ22A OS=Arabidopsis thaliana GN=RSZ22A PE=1 SV=1//2.1979
XLOC_0168sp|P08770|TRA1_MAIZE Putative AC transposase OS=Zea mays PE=2 SV=2//1.10553e-14
XLOC_0171sp|P34756|FAB1_YEAST 1-phosphatidylinositol 3-phosphate 5-kinase FAB1 OS=Saccharomyces cerevisiae (strain ATCC 204508
XLOC_0171-//-
XLOC_0171-//-
XLOC_0172sp|Q93VA3|CYC6_ARATH Cytochrome c6, chloroplastic OS=Arabidopsis thaliana GN=petJ PE=1 SV=1//5.53018e-16
XLOC_0172sp|Q9SA16|P2A09_ARATH Protein PHLOEM PROTEIN 2-LIKE A9 OS=Arabidopsis thaliana GN=PP2A9 PE=2 SV=1//6.87214e-28
XLOC_0172-//-
XLOC_0174sp|Q8LB81|GDL79_ARATH GDSL esterase/lipase At5g33370 OS=Arabidopsis thaliana GN=At5g33370 PE=2 SV=1//0
XLOC_0176sp|Q9P281|BAHC1_HUMAN BAH and coiled-coil domain-containing protein 1 OS=Homo sapiens GN=BAHCC1 PE=1 SV=3//1.37074e-1
XLOC_0176sp|Q9C7B1|TRP3_ARATH Telomere repeat-binding protein 3 OS=Arabidopsis thaliana GN=TRP3 PE=1 SV=1//2.50916e-125
XLOC_0179-//-
XLOC_0183sp|Q9LU10|DEGP8_ARATH Protease Do-like 8, chloroplastic OS=Arabidopsis thaliana GN=DEGP8 PE=1 SV=1//3.89639e-06
XLOC_0186sp|Q4FTX1|NDK_PSYA2 Nucleoside diphosphate kinase OS=Psychrobacter arcticus (strain DSM 17307 / 273-4) GN=ndk PE=3 SV
XLOC_0186sp|Q9ZPB7|AL7A1_MALDO Aldehyde dehydrogenase family 7 member A1 OS=Malus domestica PE=1 SV=3//2.14056e-89
XLOC_0193sp|P0C5D6|SAPK3_ORYSJ Serine/threonine-protein kinase SAPK3 OS=Oryza sativa subsp. japonica GN=SAPK3 PE=1 SV=1//6.790

XLOC_0195	sp|Q9FLQ7|FH20_ARATH Formin-like protein 20 OS=Arabidopsis thaliana GN=FH20 PE=2 SV=3//3.78298e-13
XLOC_0196	sp|Q9ASQ5|CRCK3_ARATH Calmodulin-binding receptor-like cytoplasmic kinase 3 OS=Arabidopsis thaliana GN=CRCK3 PE=2 SV=
XLOC_0196	sp|P08770|TRA1_MAIZE Putative AC transposase OS=Zea mays PE=2 SV=2//1.01712e-74
XLOC_0197	sp|Q2L942|RR8_GOSHI 30S ribosomal protein S8, chloroplastic OS=Gossypium hirsutum GN=rps8 PE=3 SV=1//1.57751e-23
XLOC_0202	sp|C0LGV0|Y5487_ARATH Probable LRR receptor-like serine/threonine-protein kinase At5g48740 OS=Arabidopsis thaliana GN
XLOC_0202	sp|Q9FKB5|KRP3_ARATH Cyclin-dependent kinase inhibitor 3 OS=Arabidopsis thaliana GN=KRP3 PE=1 SV=1//9.97715e-45
XLOC_0202	sp|Q8VWQ5|WRK50_ARATH Probable WRKY transcription factor 50 OS=Arabidopsis thaliana GN=WRKY50 PE=2 SV=1//2.56476e-40
XLOC_0207	-//-
XLOC_0211	-//-
XLOC_0211	-//-
XLOC_0213	sp|Q42202|RL40B_ARATH Ubiquitin-60S ribosomal protein L40-2 OS=Arabidopsis thaliana GN=RPL40B PE=1 SV=2//8.45941e-13
XLOC_0213	sp|P69326|UBIQ_WHEAT Ubiquitin OS=Triticum aestivum PE=3 SV=2//4.55546e-36
XLOC_0213	-//-
XLOC_0213	sp|O24160|TGA21_TOBAC TGACG-sequence-specific DNA-binding protein TGA-2.1 OS=Nicotiana tabacum GN=TGA21 PE=1 SV=1//2.
XLOC_0215	-//-
XLOC_0217	-//-
XLOC_0219	sp|P17642|PRS2_SOLTU Pathogenesis-related protein STH-2 OS=Solanum tuberosum GN=STH-2 PE=2 SV=1//8.24309e-41
XLOC_0219	sp|P15494|BEV1A_BETPN Major pollen allergen Bet v 1-A OS=Betula pendula GN=BETVIA PE=1 SV=2//9.50573e-47
XLOC_0220	sp|P10978|POLX_TOBAC Retrovirus-related Pol polyprotein from transposon TNT 1-94 OS=Nicotiana tabacum PE=2 SV=1//1.57
XLOC_0220	-//-
XLOC_0222	-//-
XLOC_0222	sp|Q93Y94|RPOT1_NICSY DNA-directed RNA polymerase 1, mitochondrial OS=Nicotiana sylvestris GN=RPOT1 PE=2 SV=1//3.9627
XLOC_0225	sp|Q9FN48|CAS_ARATH Calcium sensing receptor, chloroplastic OS=Arabidopsis thaliana GN=CAS PE=1 SV=1//1.58101e-11
XLOC_0225	-//-
XLOC_0225	sp|P46299|RS4_GOSHI 40S ribosomal protein S4 OS=Gossypium hirsutum GN=RPS4 PE=2 SV=1//6.46481e-166
XLOC_0225	sp|P27054|CHI4_PHAVU Endochitinase PR4 OS=Phaseolus vulgaris GN=CHI4 PE=2 SV=1//2.47633e-90
XLOC_0225	sp|Q8LRK9|TAF1A_ARATH Transcription initiation factor TFIID subunit 1-A OS=Arabidopsis thaliana GN=HAF1 PE=2 SV=1//7.
XLOC_0226	sp|Q8LRK9|TAF1A_ARATH Transcription initiation factor TFIID subunit 1-A OS=Arabidopsis thaliana GN=HAF1 PE=2 SV=1//4.
XLOC_0226	sp|Q8LRK9|TAF1A_ARATH Transcription initiation factor TFIID subunit 1-A OS=Arabidopsis thaliana GN=HAF1 PE=2 SV=1//1.
XLOC_0226	sp|C0LGQ5|GSO1_ARATH LRR receptor-like serine/threonine-protein kinase GSO1 OS=Arabidopsis thaliana GN=GSO1 PE=2 SV=1
XLOC_0226	sp|Q9LFN6|RH56_ARATH DEAD-box ATP-dependent RNA helicase 56 OS=Arabidopsis thaliana GN=RH56 PE=2 SV=2//3.01242e-10
XLOC_0226	sp|Q9LFP7|Y5158_ARATH Probable receptor-like protein kinase At5g15080 OS=Arabidopsis thaliana GN=At5g15080 PE=1 SV=1/
XLOC_0228	sp|Q54Y55|SHKC_DICDI Dual specificity protein kinase shkC OS=Dictyostelium discoideum GN=shkC PE=3 SV=1//7.79749e-37
XLOC_0229	-//-
XLOC_0230	-//-

XLOC_0235	sp|O65351|SUBL_ARATH Subtilisin-like protease OS=Arabidopsis thaliana GN=ARA12 PE=1 SV=1//0
XLOC_0235	-//-
XLOC_0236	sp|Q10295|PAP_SCHPO Poly(A) polymerase pla1 OS=Schizosaccharomyces pombe (strain 972 / ATCC 24843) GN=pla1 PE=2 SV=1/
XLOC_0238	-//-
XLOC_0242	-//-
XLOC_0243	sp|P40035|PIC2_YEAST Mitochondrial phosphate carrier protein 2 OS=Saccharomyces cerevisiae (strain ATCC 204508 / S288
XLOC_0244	sp|Q9LUV2|POP3_ARATH Probable protein Pop3 OS=Arabidopsis thaliana GN=At3g17210 PE=1 SV=1//4.8782e-59
XLOC_0246	sp|P68428|H32_WHEAT Histone H3.2 OS=Triticum aestivum PE=1 SV=2//4.91541e-76
XLOC_0252	sp|O64702|HMG14_ARATH High mobility group B protein 14 OS=Arabidopsis thaliana GN=HMGB14 PE=2 SV=2//1.70311e-10
XLOC_0256	-//-
XLOC_0258	sp|C7J4U3|OST4A_ORYSJ Dolichyl-diphosphooligosaccharide--protein glycosyltransferase subunit 4A OS=Oryza sativa subsp
XLOC_0260	-//-
XLOC_0260	-//-
XLOC_0261	-//-
XLOC_0262	-//-
XLOC_0263	sp|Q6EIK6|MATK_GOSHI Maturase K OS=Gossypium hirsutum GN=matK PE=3 SV=1//1.4413e-35
XLOC_0264	sp|Q8I7P9|POL5_DROME Retrovirus-related Pol polyprotein from transposon opus OS=Drosophila melanogaster GN=pol PE=4 S
XLOC_0265	-//-
XLOC_0267	sp|Q9C9K2|CP123_ARATH Calvin cycle protein CP12-3, chloroplastic OS=Arabidopsis thaliana GN=CP12-3 PE=1 SV=1//2.23622
XLOC_0269	sp|Q8LEM8|RL373_ARATH 60S ribosomal protein L37-3 OS=Arabidopsis thaliana GN=RPL37C PE=3 SV=1//7.81384e-16
XLOC_0271	sp|Q5QM99|RL37A_ORYSJ 60S ribosomal protein L37a OS=Oryza sativa subsp. japonica GN=Os01g0679700 PE=1 SV=1//2.8407e-5
XLOC_0271	sp|P83877|TXN4A_MOUSE Thioredoxin-like protein 4A OS=Mus musculus GN=Txnl4a PE=2 SV=1//1.38332e-17
XLOC_0273	sp|Q9LSP9|CTL2_ARATH Chitinase-like protein 2 OS=Arabidopsis thaliana GN=CTL2 PE=2 SV=1//0
XLOC_0273	sp|O04834|SAR1A_ARATH GTP-binding protein SAR1A OS=Arabidopsis thaliana GN=SAR1A PE=2 SV=1//2.95388e-107
XLOC_0273	sp|Q9S746|HTH_ARATH Protein HOTHEAD OS=Arabidopsis thaliana GN=HTH PE=1 SV=1//3.04061e-160
XLOC_0273	-//-
XLOC_0274	sp|Q9SY66|FRS11_ARATH Protein FAR1-RELATED SEQUENCE 11 OS=Arabidopsis thaliana GN=FRS11 PE=2 SV=1//4.97896e-97
XLOC_0274	sp|P10978|POLX_TOBAC Retrovirus-related Pol polyprotein from transposon TNT 1-94 OS=Nicotiana tabacum PE=2 SV=1//1.02
XLOC_0275	-//-
XLOC_0276	sp|Q2L907|PSAA_GOSHI Photosystem I P700 chlorophyll a apoprotein A1 OS=Gossypium hirsutum GN=psaA PE=3 SV=1//8.50313e
XLOC_0276	sp|Q8S403|PHO1_ARATH Phosphate transporter PHO1 OS=Arabidopsis thaliana GN=PHO1 PE=2 SV=1//1.37852e-36
XLOC_0279	-//-
XLOC_0279	-//-
XLOC_0280	-//-
XLOC_0280	sp|Q9FFF9|APC1_ARATH Anaphase-promoting complex subunit 1 OS=Arabidopsis thaliana GN=APC1 PE=2 SV=1//1.0753e-08

XLOC_0280 -//-

XLOC_0283 sp|Q54XK2|SC61A_DICDI Protein transport protein Sec61 subunit alpha OS=Dictyostelium discoideum GN=sec61a PE=3 SV=1//

XLOC_0287 -//-

XLOC_0289 sp|P10978|POLX_TOBAC Retrovirus-related Pol polyprotein from transposon TNT 1-94 OS=Nicotiana tabacum PE=2 SV=1//4.09

XLOC_0289 sp|Q9M7Q4|AI5L5_ARATH ABSCISIC ACID-INSENSITIVE 5-like protein 5 OS=Arabidopsis thaliana GN=ABF2 PE=1 SV=1//6.56748e-

XLOC_0291 -//-

XLOC_0293 sp|Q9S6Z8|TPK5_ARATH Two-pore potassium channel 5 OS=Arabidopsis thaliana GN=TPK5 PE=1 SV=1//1.10341e-08

XLOC_0294 -//-

XLOC_0296 sp|P04146|COPIA_DROME Copia protein OS=Drosophila melanogaster GN=GIP PE=1 SV=3//5.55786e-28

XLOC_0297 sp|Q9SSQ4|FRS6_ARATH Protein FAR1-RELATED SEQUENCE 6 OS=Arabidopsis thaliana GN=FRS6 PE=2 SV=1//0

XLOC_0297 sp|Q940Z2|ARP5_ARATH Actin-related protein 5 OS=Arabidopsis thaliana GN=ARP5 PE=1 SV=2//0

XLOC_0299 -//-

XLOC_0299 sp|Q93YW0|EXEC1_ARATH Protein EXECUTER 1, chloroplastic OS=Arabidopsis thaliana GN=EX1 PE=1 SV=1//3.30948e-32

XLOC_0302 -//-

XLOC_0302 -//-

XLOC_0305 -//-

XLOC_0305 sp|Q58E77|WD82B_XENLA WD repeat-containing protein 82-B OS=Xenopus laevis GN=wdr82-b PE=2 SV=1//8.01641e-37

XLOC_0306 sp|Q8GUM5|NICA_ARATH Nicastrin OS=Arabidopsis thaliana GN=At3g52640/At3g52650 PE=2 SV=1//2.05084e-09

XLOC_0306 sp|Q0ZIW7|PSAC_VITVI Photosystem I iron-sulfur center OS=Vitis vinifera GN=psaC PE=3 SV=1//1.30027e-39

XLOC_0309 sp|Q2L955|NU4C_GOSHI NAD(P)H-quinone oxidoreductase chain 4, chloroplastic OS=Gossypium hirsutum GN=ndhD PE=3 SV=1//2

XLOC_0310 sp|B0BN95|HARB1_RAT Putative nuclease HARBI1 OS=Rattus norvegicus GN=Harbi1 PE=2 SV=1//7.26925e-18

XLOC_0311 -//-

XLOC_0311 sp|P31843|RRPO_OENBE RNA-directed DNA polymerase homolog OS=Oenothera berteriana PE=4 SV=1//6.7319e-36

XLOC_0312 sp|Q8GWS0|GRXC5_ARATH Glutaredoxin-C5, chloroplastic OS=Arabidopsis thaliana GN=GRXC5 PE=1 SV=1//2.46157e-10

XLOC_0312 sp|Q9LJ98|PFD2_ARATH Probable prefoldin subunit 2 OS=Arabidopsis thaliana GN=At3g22480 PE=2 SV=1//5.07663e-64

XLOC_0314 sp|P93293|M300_ARATH Uncharacterized mitochondrial protein AtMg00300 OS=Arabidopsis thaliana GN=AtMg00300 PE=4 SV=1//

XLOC_0315 sp|Q56X72|RTNLS_ARATH Reticulon-like protein B21 OS=Arabidopsis thaliana GN=RTNLB21 PE=2 SV=2//1.52223e-28

XLOC_0315 sp|O49434|AAH_ARATH Allantoate deiminase, chloroplastic OS=Arabidopsis thaliana GN=AAH PE=1 SV=2//2.03219e-22

XLOC_0316 sp|P10978|POLX_TOBAC Retrovirus-related Pol polyprotein from transposon TNT 1-94 OS=Nicotiana tabacum PE=2 SV=1//6.21

XLOC_0317 sp|Q2MHE4|HT1_ARATH Serine/threonine-protein kinase HT1 OS=Arabidopsis thaliana GN=HT1 PE=1 SV=1//9.50234e-17

XLOC_0317 sp|Q84JC0|RRP8_ARATH Ribosomal RNA-processing protein 8 OS=Arabidopsis thaliana GN=At5g40530 PE=2 SV=1//9.18233e-13

XLOC_0320 sp|P10978|POLX_TOBAC Retrovirus-related Pol polyprotein from transposon TNT 1-94 OS=Nicotiana tabacum PE=2 SV=1//1.70

XLOC_0320 -//-

XLOC_0322 sp|Q8GW72|FUCO1_ARATH Alpha-L-fucosidase 1 OS=Arabidopsis thaliana GN=FUC1 PE=1 SV=2//0

XLOC_0322 sp|P10978|POLX_TOBAC Retrovirus-related Pol polyprotein from transposon TNT 1-94 OS=Nicotiana tabacum PE=2 SV=1//4.11

XLOC_0324sp|P08770|TRA1_MAIZE Putative AC transposase OS=Zea mays PE=2 SV=2//3.61485e-10
XLOC_0331-//-
XLOC_0331sp|P35685|RL7A_ORYSJ 60S ribosomal protein L7a OS=Oryza sativa subsp. japonica GN=RPL7A PE=2 SV=1//1.18605e-16
XLOC_0332-//-
XLOC_0334sp|P10978|POLX_TOBAC Retrovirus-related Pol polyprotein from transposon TNT 1-94 OS=Nicotiana tabacum PE=2 SV=1//2.08
XLOC_0336sp|Q9LU60|PUS7_ARATH RNA pseudourine synthase 7 OS=Arabidopsis thaliana GN=At5g51140 PE=2 SV=1//8.65973e-19
XLOC_0336sp|P10978|POLX_TOBAC Retrovirus-related Pol polyprotein from transposon TNT 1-94 OS=Nicotiana tabacum PE=2 SV=1//1.99
XLOC_0338-//-
XLOC_0340-//-
XLOC_0340sp|Q55BK0|UFD1_DICDI Ubiquitin fusion degradation protein 1 homolog OS=Dictyostelium discoideum GN=ufd1 PE=3 SV=1//2.
XLOC_0343-//-
XLOC_0343sp|Q0WM29|MMSA_ARATH Methylmalonate-semialdehyde dehydrogenase [acylating], mitochondrial OS=Arabidopsis thaliana GN=
XLOC_0344-//-
XLOC_0349-//-
XLOC_0349sp|P48578|PP2A3_ARATH Serine/threonine-protein phosphatase PP2A-3 catalytic subunit OS=Arabidopsis thaliana GN=PP2A3
XLOC_0350sp|Q653P0|KOR1_ORYSJ Potassium channel KOR1 OS=Oryza sativa subsp. japonica GN=Os06g0250600 PE=2 SV=1//7.1831e-41
XLOC_0353-//-
XLOC_0354sp|Q9LDX3|UTR4_ARATH UDP-galactose/UDP-glucose transporter 4 OS=Arabidopsis thaliana GN=UTR4 PE=2 SV=1//1.41886e-69
XLOC_0357sp|Q9MAK9|PS10B_ARATH 26S protease regulatory subunit S10B homolog B OS=Arabidopsis thaliana GN=RPT4B PE=2 SV=1//1.90
XLOC_0357sp|P24280|SEC14_YEAST SEC14 cytosolic factor OS=Saccharomyces cerevisiae (strain ATCC 204508 / S288c) GN=SEC14 PE=1 S
XLOC_0358sp|Q40170|AGL8_SOLLC Agamous-like MADS-box protein AGL8 homolog OS=Solanum lycopersicum GN=TDR4 PE=2 SV=1//4.13451e-4
XLOC_0359sp|Q05654|RTF21_SCHPO Retrotransposable element Tf2 155 kDa protein type 1 OS=Schizosaccharomyces pombe (strain 972 /
XLOC_0359-//-
XLOC_0360sp|P60498|NU6M_BRACM NADH-ubiquinone oxidoreductase chain 6 OS=Brassica campestris GN=ND6 PE=2 SV=1//2.21798e-124
XLOC_0361-//-
XLOC_0364-//-
XLOC_0367sp|Q8L8B8|LOG3_ARATH Cytokinin riboside 5'-monophosphate phosphoribohydrolase LOG3 OS=Arabidopsis thaliana GN=LC
XLOC_0367sp|P10978|POLX_TOBAC Retrovirus-related Pol polyprotein from transposon TNT 1-94 OS=Nicotiana tabacum PE=2 SV=1//5.27
XLOC_0371-//-
XLOC_0375sp|P27489|CB23_SOLLC Chlorophyll a-b binding protein 13, chloroplastic OS=Solanum lycopersicum GN=CAB13 PE=1 SV=1//2.
XLOC_0375sp|O96860|DYL2_DROME Dynein light chain 2, cytoplasmic OS=Drosophila melanogaster GN=Cdlc2 PE=3 SV=1//8.33801e-23
XLOC_0377-//-
XLOC_0378-//-
XLOC_0378sp|Q9SXD8|PPR90_ARATH Pentatricopeptide repeat-containing protein At1g62590 OS=Arabidopsis thaliana GN=At1g62590 PE=2
XLOC_0379-//-

XLOC_0384 -//-
XLOC_0385 sp|P10978|POLX_TOBAC Retrovirus-related Pol polyprotein from transposon TNT 1-94 OS=Nicotiana tabacum PE=2 SV=1//1.13
XLOC_0385 sp|F4KD71|DUR3_ARATH Urea-proton symporter DUR3 OS=Arabidopsis thaliana GN=DUR3 PE=1 SV=1//9.59264e-11
XLOC_0390 -//-
XLOC_0395 sp|O23145|KSG2_ARATH Shaggy-related protein kinase beta OS=Arabidopsis thaliana GN=ASK2 PE=2 SV=1//1.07425e-23
XLOC_0395 -//-
XLOC_0395 sp|P93740|NUD23_ARATH Nudix hydrolase 23, chloroplastic OS=Arabidopsis thaliana GN=NUDT23 PE=1 SV=2//7.89041e-15
XLOC_0398 -//-
XLOC_0399 -//-
XLOC_0400 sp|Q93YW0|EXEC1_ARATH Protein EXECUTER 1, chloroplastic OS=Arabidopsis thaliana GN=EX1 PE=1 SV=1//2.04744e-12
XLOC_0408 sp|Q39231|SUC2_ARATH Sucrose transport protein SUC2 OS=Arabidopsis thaliana GN=SUC2 PE=1 SV=2//1.69072e-29
XLOC_0412 sp|Q9FJK3|AGL80_ARATH Agamous-like MADS-box protein AGL80 OS=Arabidopsis thaliana GN=AGL80 PE=1 SV=1//1.63005e-21
XLOC_0414 sp|Q9M9M6|NDUS6_ARATH NADH dehydrogenase [ubiquinone] iron-sulfur protein 6, mitochondrial OS=Arabidopsis thaliana GN
XLOC_0414 -//-
XLOC_0417 sp|Q9M291|SC24C_ARATH Protein transport protein Sec24-like CEF OS=Arabidopsis thaliana GN=CEF PE=1 SV=3//1.53926e-36
XLOC_0417 sp|Q9M081|SC24B_ARATH Protein transport protein Sec24-like At4g32640 OS=Arabidopsis thaliana GN=At4g32640 PE=1 SV=3//
XLOC_0417 sp|Q2RAX3|CIPKX_ORYSJ CBL-interacting protein kinase 33 OS=Oryza sativa subsp. japonica GN=CIPK33 PE=2 SV=1//2.98606e
XLOC_0421 sp|P10978|POLX_TOBAC Retrovirus-related Pol polyprotein from transposon TNT 1-94 OS=Nicotiana tabacum PE=2 SV=1//5.72
XLOC_0422 sp|Q8H5T6|LTI6A_ORYSJ Hydrophobic protein LTI6A OS=Oryza sativa subsp. japonica GN=LTI6A PE=2 SV=1//8.6772e-07
XLOC_0422 -//-
XLOC_0422 -//-
XLOC_0422 sp|Q0IQN5|RTOR1_ORYSJ Regulatory-associated protein of TOR 1 OS=Oryza sativa subsp. japonica GN=RAPTOR1 PE=2 SV=2//3.
XLOC_0422 -//-
XLOC_0424 -//-
XLOC_0425 -//-
XLOC_0425 sp|Q42429|AGL8_SOLTU Agamous-like MADS-box protein AGL8 homolog OS=Solanum tuberosum PE=2 SV=1//3.14775e-07
XLOC_0429 sp|Q9LKR4|FRS10_ARATH Putative protein FAR1-RELATED SEQUENCE 10 OS=Arabidopsis thaliana GN=FRS10 PE=2 SV=2//0
XLOC_0430 sp|C7G304|GOLS2_SOLLC Galactinol synthase 2 OS=Solanum lycopersicum GN=GOLS2 PE=2 SV=1//8.83671e-172
XLOC_0430 -//-
XLOC_0430 -//-
XLOC_0433 -//-
XLOC_0437 -//-
XLOC_0440 sp|Q6R8G7|PHO13_ARATH Phosphate transporter PHO1 homolog 3 OS=Arabidopsis thaliana GN=PHO1;H3 PE=2 SV=2//1.61269e-08
XLOC_0441 sp|Q9SZL8|FRS5_ARATH Protein FAR1-RELATED SEQUENCE 5 OS=Arabidopsis thaliana GN=FRS5 PE=2 SV=1//0
XLOC_0443 sp|P08770|TRA1_MAIZE Putative AC transposase OS=Zea mays PE=2 SV=2//4.85368e-21

XLOC_0443 sp|Q2PGG3|SRR_ARATH Serine racemase OS=Arabidopsis thaliana GN=SR PE=1 SV=1//1.01096e-12
XLOC_0443 sp|Q2L942|RR8_GOSHI 30S ribosomal protein S8, chloroplastic OS=Gossypium hirsutum GN=rps8 PE=3 SV=1//1.56745e-24
XLOC_0444 -//-
XLOC_0447 sp|P93398|GBB2_TOBAC Guanine nucleotide-binding protein subunit beta-2 OS=Nicotiana tabacum PE=2 SV=1//0
XLOC_0447 sp|Q9LS94|RAG3F_ARATH Ras-related protein RABG3f OS=Arabidopsis thaliana GN=RABG3F PE=2 SV=1//1.22313e-21
XLOC_0447 sp|Q75HP9|AKT2_ORYSJ Potassium channel AKT2 OS=Oryza sativa subsp. japonica GN=Os05g0428700 PE=2 SV=1//7.55758e-76
XLOC_0448 sp|Q9C952|CPSF3_ARATH Cleavage and polyadenylation specificity factor subunit 3-I OS=Arabidopsis thaliana GN=CPSF73-I
XLOC_0448 sp|Q8W3M7|Y4598_ARATH Uncharacterized protein At4g06598 OS=Arabidopsis thaliana GN=At4g06598 PE=2 SV=2//1.19525e-41
XLOC_0448 -//-
XLOC_0449 -//-
XLOC_0453 -//-
XLOC_0454 sp|Q9LDW3|PUM11_ARATH Pumilio homolog 11 OS=Arabidopsis thaliana GN=APUM11 PE=2 SV=2//3.11888e-06
XLOC_0454 sp|Q9FWX2|NAC7_ARATH NAC domain-containing protein 7 OS=Arabidopsis thaliana GN=NAC007 PE=2 SV=2//3.25574e-29
XLOC_0455 sp|Q9FNP8|RS193_ARATH 40S ribosomal protein S19-3 OS=Arabidopsis thaliana GN=RPS19C PE=2 SV=1//2.20694e-86
XLOC_0456 sp|Q9FF99|AAP7_ARATH Probable amino acid permease 7 OS=Arabidopsis thaliana GN=AAP7 PE=1 SV=1//2.51087e-21
XLOC_0456 sp|P69322|UBIQP_PEA Polyubiquitin OS=Pisum sativum GN=PU1 PE=2 SV=2//0
XLOC_0457 sp|Q9ZW88|FBL34_ARATH F-box/LRR-repeat protein At1g67190 OS=Arabidopsis thaliana GN=At1g67190 PE=2 SV=1//5.23607e-15
XLOC_0458 sp|Q62785|HAP28_RAT 28 kDa heat- and acid-stable phosphoprotein OS=Rattus norvegicus GN=Pdap1 PE=1 SV=1//1.08102e-19
XLOC_0458 sp|Q9FJK3|AGL80_ARATH Agamous-like MADS-box protein AGL80 OS=Arabidopsis thaliana GN=AGL80 PE=1 SV=1//1.21726e-15
XLOC_0460 -//-
XLOC_0461 sp|P08770|TRA1_MAIZE Putative AC transposase OS=Zea mays PE=2 SV=2//1.52558e-81
XLOC_0462 -//-
XLOC_0463 -//-
XLOC_0463 sp|F4K5T2|CGL_ARATH Bifunctional cystathionine gamma-lyase/cysteine synthase OS=Arabidopsis thaliana GN=DES1 PE=1 SV=
XLOC_0464 sp|P07463|CALM_PARTE Calmodulin OS=Paramecium tetraurelia GN=CAM PE=1 SV=3//3.7776e-08
XLOC_0464 sp|P32294|AX22B_VIGRR Auxin-induced protein 22B OS=Vigna radiata var. radiata GN=AUX22B PE=2 SV=1//2.82768e-24
XLOC_0464 sp|Q8LG10|GAT15_ARATH GATA transcription factor 15 OS=Arabidopsis thaliana GN=GATA15 PE=2 SV=2//2.81461e-13
XLOC_0465 -//-
XLOC_0468 sp|Q6K9C3|RZP23_ORYSJ Serine/arginine-rich splicing factor RSZ23 OS=Oryza sativa subsp. japonica GN=RSZ23 PE=2 SV=1//
XLOC_0468 sp|P46087|NOP2_HUMAN Putative ribosomal RNA methyltransferase NOP2 OS=Homo sapiens GN=NOP2 PE=1 SV=2//1.05336e-26
XLOC_0469 sp|Q6NUB2|CW15A_XENLA Protein CWC15 homolog A OS=Xenopus laevis GN=cwc15-a PE=2 SV=1//3.51664e-07
XLOC_0469 -//-
XLOC_0469 sp|Q9SAK5|APL_ARATH Myb family transcription factor APL OS=Arabidopsis thaliana GN=APL PE=2 SV=2//4.77278e-117
XLOC_0469 sp|Q9LY87|RGLG2_ARATH E3 ubiquitin-protein ligase RGLG2 OS=Arabidopsis thaliana GN=RGLG2 PE=1 SV=1//2.01926e-147
XLOC_0470 sp|O64518|MCA5_ARATH Metacaspase-5 OS=Arabidopsis thaliana GN=AMC5 PE=1 SV=1//7.92534e-19

XLOC_0470 sp|Q8W206|CSN6A_ARATH COP9 signalosome complex subunit 6a OS=Arabidopsis thaliana GN=CSN6A PE=1 SV=2//2.28904e-73
XLOC_0470 sp|Q42962|PGKY_TOBAC Phosphoglycerate kinase, cytosolic OS=Nicotiana tabacum PE=2 SV=1//2.56528e-26
XLOC_0470 sp|Q20263|GOT1_CAEEL Probable Golgi transport protein 1 OS=Caenorhabditis elegans GN=F41C3.4 PE=2 SV=2//2.50415e-15
XLOC_0471 sp|Q9S9Z7|P2C10_ARATH Probable protein phosphatase 2C 10 OS=Arabidopsis thaliana GN=At1g34750 PE=1 SV=1//1.15987e-12
XLOC_0472 sp|Q75H77|SAPKA_ORYSJ Serine/threonine-protein kinase SAPK10 OS=Oryza sativa subsp. japonica GN=SAPK10 PE=2 SV=1//5.0
XLOC_0477 sp|Q2L934|PSBH_GOSHI Photosystem II reaction center protein H OS=Gossypium hirsutum GN=psbH PE=3 SV=1//2.01731e-19
XLOC_0478 -//-
XLOC_0480 -//-
XLOC_0481 sp|Q8LB81|GDL79_ARATH GDSL esterase/lipase At5g33370 OS=Arabidopsis thaliana GN=At5g33370 PE=2 SV=1//0
XLOC_0488 -//-
XLOC_0489 sp|Q9CA90|HPR2_ARATH Glyoxylate/hydroxypyruvate reductase A HPR2 OS=Arabidopsis thaliana GN=HPR2 PE=1 SV=1//8.18956e-
XLOC_0489 sp|O48786|C734A_ARATH Cytochrome P450 734A1 OS=Arabidopsis thaliana GN=CYP734A1 PE=2 SV=1//8.96681e-21
XLOC_0492 -//-
XLOC_0493 sp|Q42400|AAP1_ARATH Amino acid permease 1 OS=Arabidopsis thaliana GN=AAP1 PE=1 SV=1//5.44879e-21
XLOC_0495 -//-
XLOC_0495 sp|Q8L840|RQL4A_ARATH ATP-dependent DNA helicase Q-like 4A OS=Arabidopsis thaliana GN=RECQL4A PE=2 SV=1//2.91347e-26
XLOC_0496 sp|P42731|PABP2_ARATH Polyadenylate-binding protein 2 OS=Arabidopsis thaliana GN=PAB2 PE=1 SV=1//1.49008e-19
XLOC_0499 sp|Q8GW29|LOG7_ARATH Cytokinin riboside 5'-monophosphate phosphoribohydrolase LOG7 OS=Arabidopsis thaliana GN=LC
XLOC_0500 -//-
XLOC_0502 sp|Q8L5Y9|PANK2_ARATH Pantothenate kinase 2 OS=Arabidopsis thaliana GN=At4g32180 PE=1 SV=2//2.77758e-38
XLOC_0505 sp|O81832|Y4729_ARATH G-type lectin S-receptor-like serine/threonine-protein kinase At4g27290 OS=Arabidopsis thaliana
XLOC_0506 sp|P0CH27|RL402_TRYCR Ubiquitin-60S ribosomal protein L40 OS=Trypanosoma cruzi PE=2 SV=1//7.41173e-35
XLOC_0506 sp|P0CG71|UBIQ1_CAEEL Polyubiquitin-A OS=Caenorhabditis elegans GN=ubq-1 PE=3 SV=1//0
XLOC_0508 -//-
XLOC_0508 sp|Q94EG9|ZIP11_ARATH Zinc transporter 11 OS=Arabidopsis thaliana GN=ZIP11 PE=2 SV=1//4.25366e-134
XLOC_0509 -//-
XLOC_0511 -//-
XLOC_0517 sp|P42844|ZIM17_YEAST Mitochondrial protein import protein ZIM17 OS=Saccharomyces cerevisiae (strain ATCC 204508 / S2
XLOC_0518 sp|Q9MAM1|CIPK9_ARATH CBL-interacting serine/threonine-protein kinase 9 OS=Arabidopsis thaliana GN=CIPK9 PE=1 SV=2//1
XLOC_0521 sp|P08770|TRA1_MAIZE Putative AC transposase OS=Zea mays PE=2 SV=2//3.36773e-63
XLOC_0521 sp|Q9SPM5|APY2_ARATH Apyrase 2 OS=Arabidopsis thaliana GN=APY2 PE=1 SV=1//2.91978e-14
XLOC_0523 sp|P0C2F6|RNHX1_ARATH Putative ribonuclease H protein At1g65750 OS=Arabidopsis thaliana GN=At1g65750 PE=3 SV=1//3.372
XLOC_0526 -//-
XLOC_0531 sp|Q9LSG3|GAUT8_ARATH Galacturonosyltransferase 8 OS=Arabidopsis thaliana GN=GAUT8 PE=1 SV=1//5.8509e-98
XLOC_0532 sp|P81393|MYB08_ANTMA Myb-related protein 308 OS=Antirrhinum majus GN=MYB308 PE=2 SV=1//1.10869e-119

XLOC_0532	sp|P03010|TRAC9_MAIZE Putative AC9 transposase OS=Zea mays PE=4 SV=1//4.37812e-07
XLOC_0533	sp|Q9ZPI6|AIM1_ARATH Peroxisomal fatty acid beta-oxidation multifunctional protein AIM1 OS=Arabidopsis thaliana GN=AI
XLOC_0535	sp|P27489|CB23_SOLLC Chlorophyll a-b binding protein 13, chloroplastic OS=Solanum lycopersicum GN=CAB13 PE=1 SV=1//1.
XLOC_0535	sp|Q02909|CAPP1_SOYBN Phosphoenolpyruvate carboxylase, housekeeping isozyme OS=Glycine max GN=PPC16 PE=2 SV=1//7.1531
XLOC_0536	sp|Q9C9H7|RLP12_ARATH Receptor-like protein 12 OS=Arabidopsis thaliana GN=RLP12 PE=2 SV=2//4.88504e-15
XLOC_0538	sp|Q62785|HAP28_RAT 28 kDa heat- and acid-stable phosphoprotein OS=Rattus norvegicus GN=Pdap1 PE=1 SV=1//9.41414e-13
XLOC_0538	sp|Q96386|MT3_CARPA Metallothionein-like protein type 3 OS=Carica papaya PE=3 SV=1//6.98043e-07
XLOC_0538	sp|Q40392|TMVRN_NICGU TMV resistance protein N OS=Nicotiana glutinosa GN=N PE=1 SV=1//2.66225e-31
XLOC_0541	sp|Q8VY88|LTD_ARATH Protein LHCP TRANSLOCATION DEFECT OS=Arabidopsis thaliana GN=LTD PE=1 SV=1//7.12087e-80
XLOC_0541	sp|P10978|POLX_TOBAC Retrovirus-related Pol polyprotein from transposon TNT 1-94 OS=Nicotiana tabacum PE=2 SV=1//4.72
XLOC_0541	-//-
XLOC_0545	sp|Q9LM71|FKB18_ARATH Peptidyl-prolyl cis-trans isomerase FKBP18, chloroplastic OS=Arabidopsis thaliana GN=FKBP18 PE=
XLOC_0545	sp|P92555|M1250_ARATH Uncharacterized mitochondrial protein AtMg01250 OS=Arabidopsis thaliana GN=AtMg01250 PE=4 SV=1/
XLOC_0547	-//-
XLOC_0548	sp|P08770|TRA1_MAIZE Putative AC transposase OS=Zea mays PE=2 SV=2//1.13662e-72
XLOC_0548	sp|P92934|AAP6_ARATH Amino acid permease 6 OS=Arabidopsis thaliana GN=AAP6 PE=1 SV=1//1.25837e-55
XLOC_0548	sp|P92934|AAP6_ARATH Amino acid permease 6 OS=Arabidopsis thaliana GN=AAP6 PE=1 SV=1//1.52565e-56
XLOC_0548	sp|Q9M0E0|R15A5_ARATH 40S ribosomal protein S15a-5 OS=Arabidopsis thaliana GN=RPS15AE PE=2 SV=1//7.03187e-41
XLOC_0550	-//-
XLOC_0555	sp|P69322|UBIQP_PEA Polyubiquitin OS=Pisum sativum GN=PU1 PE=2 SV=2//0
XLOC_0559	-//-
XLOC_0563	-//-
XLOC_0564	sp|P29610|CY12_SOLTU Cytochrome c1-2, heme protein, mitochondrial (Fragment) OS=Solanum tuberosum GN=CYCL PE=1 SV=1//
XLOC_0565	sp|Q8LBA6|PIS1_ARATH CDP-diacylglycerol--inositol 3-phosphatidyltransferase 1 OS=Arabidopsis thaliana GN=PIS1 PE=1 SV
XLOC_0570	sp|Q8S628|PDR13_ORYSJ Pleiotropic drug resistance protein 13 OS=Oryza sativa subsp. japonica GN=PDR13 PE=3 SV=1//2.96
XLOC_0571	sp|P08548|LIN1_NYCCO LINE-1 reverse transcriptase homolog OS=Nycticebus coucang PE=1 SV=1//3.67696e-09
XLOC_0574	sp|Q2L953|NDHH_GOSHI NAD(P)H-quinone oxidoreductase subunit H, chloroplastic OS=Gossypium hirsutum GN=ndhH PE=3 SV=1/
XLOC_0575	-//-
XLOC_0575	-//-
XLOC_0578	sp|Q9F234|AGL2_BACTQ Alpha-glucosidase 2 OS=Bacillus thermoamyloliquefaciens PE=3 SV=1//1.98621e-98
XLOC_0578	sp|Q6F2U9|SYK_ORYSJ Lysine--tRNA ligase OS=Oryza sativa subsp. japonica GN=Os03g0586800 PE=2 SV=1//1.60709e-22
XLOC_0578	sp|Q2L913|YCF4_GOSHI Photosystem I assembly protein Ycf4 OS=Gossypium hirsutum GN=ycf4 PE=3 SV=1//4.21266e-33
XLOC_0578	sp|A0A361|PSBB_COFAR Photosystem II CP47 chlorophyll apoprotein OS=Coffea arabica GN=psbB PE=3 SV=1//1.13008e-22
XLOC_0578	sp|Q09WZ1|PSBB_MORIN Photosystem II CP47 chlorophyll apoprotein OS=Morus indica GN=psbB PE=3 SV=1//5.95932e-19
XLOC_0580	-//-

XLOC_0582	sp|Q89VX9|GLND_BRAJA [Protein-PII] uridylyltransferase OS=Bradyrhizobium japonicum (strain USDA 110) GN=glnD PE=3 SV=
XLOC_0582	sp|O49286|SKP2B_ARATH F-box protein SKP2B OS=Arabidopsis thaliana GN=SKP2B PE=1 SV=1//4.57419e-09
XLOC_0583	sp|Q9SYL9|RK13_ARATH 50S ribosomal protein L13, chloroplastic OS=Arabidopsis thaliana GN=RPL13 PE=1 SV=1//5.19782e-12
XLOC_0586	sp|Q84MA2|IP5P1_ARATH Type I inositol 1,4,5-trisphosphate 5-phosphatase 1 OS=Arabidopsis thaliana GN=IP5P1 PE=1 SV=2/
XLOC_0586	-//-
XLOC_0587	sp|Q42962|PGKY_TOBAC Phosphoglycerate kinase, cytosolic OS=Nicotiana tabacum PE=2 SV=1//3.86339e-23
XLOC_0589	sp|P49636|RL40_NICSY Ubiquitin-60S ribosomal protein L40 OS=Nicotiana sylvestris GN=UBICEP52-7 PE=2 SV=2//1.0956e-86
XLOC_0591	-//-
XLOC_0591	sp|Q42783|BCCP_SOYBN Biotin carboxyl carrier protein of acetyl-CoA carboxylase, chloroplastic OS=Glycine max GN=ACCB-
XLOC_0592	-//-
XLOC_0598	sp|Q55E69|SYS1_DICDI Protein SYS1 homolog OS=Dictyostelium discoideum GN=sys1 PE=3 SV=1//2.90558e-06
XLOC_0598	sp|O74985|NAT1_SCHPO N-terminal acetyltransferase A complex subunit nat1 OS=Schizosaccharomyces pombe (strain 972 / A
XLOC_0599	-//-
XLOC_0599	sp|P10978|POLX_TOBAC Retrovirus-related Pol polyprotein from transposon TNT 1-94 OS=Nicotiana tabacum PE=2 SV=1//4.05
XLOC_0599	sp|P86387|PLDA1_CARPA Phospholipase D alpha 1 OS=Carica papaya GN=PLD1 PE=1 SV=1//2.78517e-16
XLOC_0602	sp|P27057|GAST1_SOLLC Protein GAST1 OS=Solanum lycopersicum GN=GAST1 PE=2 SV=1//8.96252e-32
XLOC_0603	sp|Q93Y09|SCP45_ARATH Serine carboxypeptidase-like 45 OS=Arabidopsis thaliana GN=SCPL45 PE=2 SV=1//1.26314e-24
XLOC_0603	sp|O49292|PPD4_ARATH PsbP domain-containing protein 4, chloroplastic OS=Arabidopsis thaliana GN=PPD4 PE=1 SV=2//1.179
XLOC_0603	sp|Q9FZ36|M3K2_ARATH Mitogen-activated protein kinase kinase kinase 2 OS=Arabidopsis thaliana GN=ANP2 PE=2 SV=1//1.51
XLOC_0604	sp|Q9FL28|FLS2_ARATH LRR receptor-like serine/threonine-protein kinase FLS2 OS=Arabidopsis thaliana GN=FLS2 PE=1 SV=1
XLOC_0606	-//-
XLOC_0607	sp|O64477|Y2913_ARATH G-type lectin S-receptor-like serine/threonine-protein kinase At2g19130 OS=Arabidopsis thaliana
XLOC_0607	sp|Q0WPW4|ACCO5_ARATH 1-aminocyclopropane-1-carboxylate oxidase 5 OS=Arabidopsis thaliana GN=At1g77330 PE=2 SV=1//3.8
XLOC_0612	sp|Q1LY77|SE1BA_DANRE Histone-lysine N-methyltransferase SETD1B-A OS=Danio rerio GN=setd1ba PE=1 SV=2//3.05687e-57
XLOC_0613	sp|Q8LC03|ATB13_ARATH Homeobox-leucine zipper protein ATHB-13 OS=Arabidopsis thaliana GN=ATHB-13 PE=2 SV=2//1.42634e-
XLOC_0618	sp|Q8CP71|DING_STAES Probable ATP-dependent helicase DinG homolog OS=Staphylococcus epidermidis (strain ATCC 12228) G
XLOC_0618	sp|P08770|TRA1_MAIZE Putative AC transposase OS=Zea mays PE=2 SV=2//3.07807e-13
XLOC_0621	-//-
XLOC_0623	sp|P20825|POL2_DROME Retrovirus-related Pol polyprotein from transposon 297 OS=Drosophila melanogaster GN=pol PE=4 SV
XLOC_0624	-//-
XLOC_0625	sp|Q6NPR7|PMTO_ARATH Probable methyltransferase PMT24 OS=Arabidopsis thaliana GN=At1g29470 PE=1 SV=1//0
XLOC_0625	-//-
XLOC_0625	sp|Q43636|TRXH_RICCO Thioredoxin H-type OS=Ricinus communis PE=3 SV=1//4.93945e-58
XLOC_0625	-//-
XLOC_0627	-//-

XLOC_0627 sp|Q9I8D1|MYO6_CHICK Unconventional myosin-VI OS=Gallus gallus GN=MYO6 PE=1 SV=1//4.57223e-13
XLOC_0627 -//-
XLOC_0629 sp|Q9LQ73|PX11C_ARATH Peroxisomal membrane protein 11C OS=Arabidopsis thaliana GN=PEX11C PE=1 SV=1//3.08898e-141
XLOC_0630 sp|Q00962|POL_CAMVN Enzymatic polyprotein OS=Cauliflower mosaic virus (strain NY8153) GN=ORF V PE=3 SV=1//4.02417e-10
XLOC_0631 -//-
XLOC_0633 sp|Q8GYN9|MENB_ARATH 1,4-Dihydroxy-2-naphthoyl-CoA synthase, peroxisomal OS=Arabidopsis thaliana GN=MENB PE=1 SV=2//5
XLOC_0634 -//-
XLOC_0639 sp|P92540|M1060_ARATH Uncharacterized mitochondrial protein AtMg01060 OS=Arabidopsis thaliana GN=AtMg01060 PE=4 SV=1/
XLOC_0639 -//-
XLOC_0645 sp|Q9STN3|SPT51_ARATH Putative transcription elongation factor SPT5 homolog 1 OS=Arabidopsis thaliana GN=At4g08350 PE
XLOC_0646 sp|P07591|TRXM_SPIOL Thioredoxin M-type, chloroplastic OS=Spinacia oleracea PE=1 SV=2//1.76432e-55
XLOC_0648 sp|F4HPR5|DRP5A_ARATH Dynamin-related protein 5A OS=Arabidopsis thaliana GN=DRP5A PE=2 SV=1//3.40957e-31
XLOC_0648 -//-
XLOC_0649 sp|Q9FMA3|PEX5_ARATH Peroxisome biogenesis protein 5 OS=Arabidopsis thaliana GN=PEX5 PE=1 SV=1//1.62249e-19
XLOC_0651 -//-
XLOC_0651 sp|P0C2F6|RNHX1_ARATH Putative ribonuclease H protein At1g65750 OS=Arabidopsis thaliana GN=At1g65750 PE=3 SV=1//1.170
XLOC_0652 sp|Q29Q28|UTR2_ARATH UDP-galactose/UDP-glucose transporter 2 OS=Arabidopsis thaliana GN=UTR2 PE=2 SV=1//2.247e-24
XLOC_0654 -//-
XLOC_0655 -//-
XLOC_0655 sp|Q850K7|EXLB1_ORYSJ Expansin-like B1 OS=Oryza sativa subsp. japonica GN=EXLB1 PE=2 SV=2//4.02615e-11
XLOC_0657 sp|Q6PHQ8|NAA35_MOUSE N-alpha-acetyltransferase 35, NatC auxiliary subunit OS=Mus musculus GN=Naa35 PE=1 SV=1//7.0199
XLOC_0659 -//-
XLOC_0659 sp|Q42560|ACO1_ARATH Aconitate hydratase 1 OS=Arabidopsis thaliana GN=ACO1 PE=1 SV=2//7.6177e-22
XLOC_0660 sp|Q54RR9|Y9844_DICDI Probable serine/threonine-protein kinase DDB_G0282963 OS=Dictyostelium discoideum GN=DDB_G02829
XLOC_0660 sp|Q292F9|GDAP2_DROPS Protein GDAP2 homolog OS=Drosophila pseudoobscura pseudoobscura GN=GA15091 PE=3 SV=1//2.19921e-
XLOC_0660 sp|Q7XA86|ZDH11_ARATH Probable S-acyltransferase At3g51390 OS=Arabidopsis thaliana GN=At3g51390 PE=2 SV=1//8.61437e-9
XLOC_0660 sp|Q5ZIF1|APMAP_CHICK Adipocyte plasma membrane-associated protein OS=Gallus gallus GN=APMAP PE=2 SV=1//1.29702e-42
XLOC_0664 -//-
XLOC_0666 -//-
XLOC_0666 sp|Q9FV71|E2FB_ARATH Transcription factor E2FB OS=Arabidopsis thaliana GN=E2FB PE=1 SV=1//7.75412e-58
XLOC_0669 -//-
XLOC_0670 sp|Q2JS42|PYRH_SYNJA Uridylate kinase OS=Synechococcus sp. (strain JA-3-3Ab) GN=pyrH PE=3 SV=1//4.17217e-61
XLOC_0670 sp|P10978|POLX_TOBAC Retrovirus-related Pol polyprotein from transposon TNT 1-94 OS=Nicotiana tabacum PE=2 SV=1//1.65
XLOC_0672 -//-
XLOC_0674 sp|Q39255|SKP1A_ARATH SKP1-like protein 1A OS=Arabidopsis thaliana GN=SKP1A PE=1 SV=1//9.71046e-73

XLOC_0675 -//-
XLOC_0679 sp|O64789|DRL18_ARATH Probable disease resistance protein At1g61310 OS=Arabidopsis thaliana GN=At1g61310 PE=2 SV=1//4
XLOC_0680 -//-
XLOC_0681 sp|F4IQV7|SCY2_ARATH Preprotein translocase subunit SCY2, chloroplastic OS=Arabidopsis thaliana GN=SCY2 PE=2 SV=1//3.
XLOC_0681 sp|P70698|PYRG1_MOUSE CTP synthase 1 OS=Mus musculus GN=Ctps1 PE=1 SV=2//4.419e-30
XLOC_0681 sp|P0C8Z0|Y8359_ORYSI Uncharacterized protein OsI_027940 OS=Oryza sativa subsp. indica GN=OsI_027940 PE=1 SV=2//4.629
XLOC_0682 -//-
XLOC_0682 sp|Q8LBI9|EDL16_ARATH Sugar transporter ERD6-like 16 OS=Arabidopsis thaliana GN=At5g18840 PE=2 SV=2//4.01047e-42
XLOC_0682 sp|Q8LBI9|EDL16_ARATH Sugar transporter ERD6-like 16 OS=Arabidopsis thaliana GN=At5g18840 PE=2 SV=2//1.0452e-21
XLOC_0682 sp|Q9LUG9|MD33A_ARATH Mediator of RNA polymerase II transcription subunit 33A OS=Arabidopsis thaliana GN=MED33A PE=1
XLOC_0682 sp|Q8H100|AGD8_ARATH Probable ADP-ribosylation factor GTPase-activating protein AGD8 OS=Arabidopsis thaliana GN=AGD8
XLOC_0682 sp|Q42840|HEM6_HORVU Coproporphyrinogen-III oxidase, chloroplastic OS=Hordeum vulgare GN=CPX PE=2 SV=1//1.5607e-41
XLOC_0683 -//-
XLOC_0684 sp|Q9LM02|SMT1_ARATH Cycloartenol-C-24-methyltransferase OS=Arabidopsis thaliana GN=SMT1 PE=2 SV=1//0
XLOC_0690 sp|Q04062|RPN9_YEAST 26S proteasome regulatory subunit RPN9 OS=Saccharomyces cerevisiae (strain ATCC 204508 / S288c)
XLOC_0690 sp|P0CG71|UBIQ1_CAEEL Polyubiquitin-A OS=Caenorhabditis elegans GN=ubq-1 PE=3 SV=1//5.04817e-36
XLOC_0690 sp|P0CH05|UBI2P_PETCR Polyubiquitin OS=Petroselinum crispum GN=PCUBI4-2 PE=3 SV=1//0
XLOC_0691 sp|A4GYU7|RK14_POPTR 50S ribosomal protein L14, chloroplastic OS=Populus trichocarpa GN=rpl14 PE=3 SV=1//4.6354e-25
XLOC_0691 sp|Q8L6Y1|UBP14_ARATH Ubiquitin carboxyl-terminal hydrolase 14 OS=Arabidopsis thaliana GN=UBP14 PE=1 SV=1//6.67603e-1
XLOC_0692 -//-
XLOC_0694 sp|O65717|CNGC1_ARATH Cyclic nucleotide-gated ion channel 1 OS=Arabidopsis thaliana GN=CNGC1 PE=1 SV=1//1.25559e-39
XLOC_0694 sp|P0C2F6|RNHX1_ARATH Putative ribonuclease H protein At1g65750 OS=Arabidopsis thaliana GN=At1g65750 PE=3 SV=1//1.848
XLOC_0697 -//-
XLOC_0697 -//-
XLOC_0700 sp|Q8RXD4|BRCA1_ARATH Protein BREAST CANCER SUSCEPTIBILITY 1 homolog OS=Arabidopsis thaliana GN=BRCA1 PE=2 SV=1//3.85
XLOC_0700 -//-
XLOC_0700 -//-
XLOC_0700 sp|P10978|POLX_TOBAC Retrovirus-related Pol polyprotein from transposon TNT 1-94 OS=Nicotiana tabacum PE=2 SV=1//7.76
XLOC_0701 sp|P42791|RL182_ARATH 60S ribosomal protein L18-2 OS=Arabidopsis thaliana GN=RPL18B PE=1 SV=2//4.44841e-112
XLOC_0702 sp|Q9LX82|MYB48_ARATH Transcription factor MYB48 OS=Arabidopsis thaliana GN=MYB48 PE=2 SV=1//5.06086e-62
XLOC_0703 sp|Q9XGI9|AGUB_SOLLC N-carbamoylputrescine amidase OS=Solanum lycopersicum GN=CPA PE=2 SV=1//1.64254e-15
XLOC_0703 sp|Q0WPH8|NEK5_ARATH Serine/threonine-protein kinase Nek5 OS=Arabidopsis thaliana GN=NEK5 PE=1 SV=1//3.18009e-72
XLOC_0703 -//-
XLOC_0703 sp|P34913|HYES_HUMAN Bifunctional epoxide hydrolase 2 OS=Homo sapiens GN=EPHX2 PE=1 SV=2//3.14926e-40
XLOC_0704 -//-

XLOC_0704 sp|O23116|EIL3_ARATH ETHYLENE INSENSITIVE 3-like 3 protein OS=Arabidopsis thaliana GN=EIL3 PE=1 SV=1//2.30449e-138
XLOC_0705 sp|Q93W93|FBK22_ARATH F-box/kelch-repeat protein At1g55270 OS=Arabidopsis thaliana GN=At1g55270 PE=2 SV=1//0
XLOC_0707 -//-
XLOC_0707 sp|Q9CA90|HPR2_ARATH Glyoxylate/hydroxypyruvate reductase A HPR2 OS=Arabidopsis thaliana GN=HPR2 PE=1 SV=1//1.32852e-
XLOC_0707 sp|P0C2F6|RNHX1_ARATH Putative ribonuclease H protein At1g65750 OS=Arabidopsis thaliana GN=At1g65750 PE=3 SV=1//1.613
XLOC_0711 sp|Q9Y5Q8|TF3C5_HUMAN General transcription factor 3C polypeptide 5 OS=Homo sapiens GN=GTF3C5 PE=1 SV=2//3.96468e-27
XLOC_0712 -//-
XLOC_0713 sp|Q07123|AMO2_ARTS1 Copper methylamine oxidase OS=Arthrobacter sp. (strain P1) GN=maoII PE=1 SV=1//3.72682e-23
XLOC_0714 -//-
XLOC_0719 sp|Q9C9L5|WAKLH_ARATH Wall-associated receptor kinase-like 9 OS=Arabidopsis thaliana GN=WAKL9 PE=2 SV=1//2.03629e-09
XLOC_0719 -//-
XLOC_0720 sp|A7QEU4|PER5_VITVI Peroxidase 5 OS=Vitis vinifera GN=GSVIVT00037159001 PE=1 SV=2//8.67384e-89
XLOC_0720 -//-
XLOC_0720 -//-
XLOC_0720 sp|P22242|DRPE_CRAPL Desiccation-related protein PCC13-62 OS=Craterostigma plantagineum PE=2 SV=1//1.36631e-92
XLOC_0720 sp|Q8TE76|MORC4_HUMAN MORC family CW-type zinc finger protein 4 OS=Homo sapiens GN=MORC4 PE=1 SV=2//5.01182e-08
XLOC_0720 -//-
XLOC_0720 -//-
XLOC_0720 sp|P08770|TRA1_MAIZE Putative AC transposase OS=Zea mays PE=2 SV=2//9.62734e-78
XLOC_0722 sp|O65572|CCD1_ARATH Carotenoid 9,10(9',10')-cleavage dioxygenase 1 OS=Arabidopsis thaliana GN=CCD1 PE=1 SV
XLOC_0723 sp|Q8W471|AAE15_ARATH Long-chain-fatty-acid--[acyl-carrier-protein] ligase AEE15, chloroplastic OS=Arabidopsis thalia
XLOC_0724 sp|P10978|POLX_TOBAC Retrovirus-related Pol polyprotein from transposon TNT 1-94 OS=Nicotiana tabacum PE=2 SV=1//7.92
XLOC_0725 -//-
XLOC_0725 sp|Q9ZT50|RHA2A_ARATH E3 ubiquitin-protein ligase RHA2A OS=Arabidopsis thaliana GN=RHA2A PE=1 SV=1//5.94929e-12
XLOC_0728 -//-
XLOC_0730 sp|P37392|TBB1_LUPAL Tubulin beta-1 chain OS=Lupinus albus GN=TUBB1 PE=3 SV=1//0
XLOC_0730 sp|Q6VAF4|TBB9_GOSHI Tubulin beta-9 chain OS=Gossypium hirsutum PE=2 SV=1//0
XLOC_0730 sp|Q7XSQ9|PIP12_ORYSJ Probable aquaporin PIP1-2 OS=Oryza sativa subsp. japonica GN=PIP1-2 PE=2 SV=3//3.53958e-180
XLOC_0731 sp|Q9LVM1|AB25B_ARATH ABC transporter B family member 25, mitochondrial OS=Arabidopsis thaliana GN=ABCB25 PE=1 SV=1//
XLOC_0733 sp|P10978|POLX_TOBAC Retrovirus-related Pol polyprotein from transposon TNT 1-94 OS=Nicotiana tabacum PE=2 SV=1//1.03
XLOC_0734 sp|O04885|LGUL_BRAJU Lactoylglutathione lyase OS=Brassica juncea GN=GLY I PE=2 SV=1//6.31902e-94
XLOC_0734 sp|Q9SJL9|XTH32_ARATH Probable xyloglucan endotransglucosylase/hydrolase protein 32 OS=Arabidopsis thaliana GN=XTH32
XLOC_0734 -//-
XLOC_0734 -//-
XLOC_0737 sp|Q9ZW33|TIM10_ARATH Mitochondrial import inner membrane translocase subunit TIM10 OS=Arabidopsis thaliana GN=TIM10

XLOC_0737-//-
XLOC_0738sp|Q94EI3|PE192_ARATH Peroxisome biogenesis protein 19-2 OS=Arabidopsis thaliana GN=PEX19-2 PE=1 SV=1//7.63146e-66
XLOC_0739sp|P53997|SET_DROME Protein SET OS=Drosophila melanogaster GN=Set PE=1 SV=2//1.67098e-47
XLOC_0741sp|C7J4U3|OST4A_ORYSJ Dolichyl-diphosphooligosaccharide--protein glycosyltransferase subunit 4A OS=Oryza sativa subsp
XLOC_0741-//-
XLOC_0742sp|P08770|TRA1_MAIZE Putative AC transposase OS=Zea mays PE=2 SV=2//1.96444e-97
XLOC_0743sp|Q9LQU4|PCR2_ARATH Protein PLANT CADMIUM RESISTANCE 2 OS=Arabidopsis thaliana GN=PCR2 PE=1 SV=1//4.44787e-60
XLOC_0743sp|Q7PC82|AB42G_ARATH ABC transporter G family member 42 OS=Arabidopsis thaliana GN=ABCG42 PE=2 SV=1//1.44692e-08
XLOC_0746sp|Q8BGE5|FANCM_MOUSE Fanconi anemia group M protein homolog OS=Mus musculus GN=Fancm PE=1 SV=3//2.43096e-09
XLOC_0747-//-
XLOC_0748-//-
XLOC_0752sp|Q8GTB6|THCAS_CANSA Tetrahydrocannabinolic acid synthase OS=Cannabis sativa PE=1 SV=1//2.45285e-48
XLOC_0752sp|Q7GB25|AB5C_ARATH ABC transporter C family member 5 OS=Arabidopsis thaliana GN=ABCC5 PE=2 SV=2//5.77499e-49
XLOC_0752sp|Q7GB25|AB5C_ARATH ABC transporter C family member 5 OS=Arabidopsis thaliana GN=ABCC5 PE=2 SV=2//3.48881e-44
XLOC_0752sp|Q8VZZ4|AB6C_ARATH ABC transporter C family member 6 OS=Arabidopsis thaliana GN=ABCC6 PE=2 SV=3//1.52381e-54
XLOC_0752sp|Q7FAH2|G3PC2_ORYSJ Glyceraldehyde-3-phosphate dehydrogenase 2, cytosolic OS=Oryza sativa subsp. japonica GN=GAPC2
XLOC_0753sp|Q9LYT3|TT12_ARATH Protein TRANSPARENT TESTA 12 OS=Arabidopsis thaliana GN=TT12 PE=2 SV=1//4.08827e-15
XLOC_0755sp|P0C2F6|RNHX1_ARATH Putative ribonuclease H protein At1g65750 OS=Arabidopsis thaliana GN=At1g65750 PE=3 SV=1//1.977
XLOC_0756sp|P31165|RK15_PEA 50S ribosomal protein L15, chloroplastic (Fragment) OS=Pisum sativum GN=RPL15 PE=2 SV=1//3.9359e-4
XLOC_0756sp|Q941R6|MLP31_ARATH MLP-like protein 31 OS=Arabidopsis thaliana GN=MLP31 PE=1 SV=2//1.16588e-18
XLOC_0757-//-
XLOC_0759-//-
XLOC_0763sp|Q9LT17|BBR_ARATH E3 ubiquitin ligase BIG BROTHER-related OS=Arabidopsis thaliana GN=BBR PE=2 SV=1//3.02093e-17
XLOC_0764-//-
XLOC_0764sp|O80977|VSR3_ARATH Vacuolar-sorting receptor 3 OS=Arabidopsis thaliana GN=VSR3 PE=2 SV=1//6.25066e-61
XLOC_0764sp|Q0WL52|NPY5_ARATH BTB/POZ domain-containing protein NPY5 OS=Arabidopsis thaliana GN=NPY5 PE=2 SV=2//1.6636e-79
XLOC_0764sp|O64818|Y2309_ARATH Uncharacterized protein At2g23090 OS=Arabidopsis thaliana GN=At2g23090 PE=1 SV=1//1.31124e-16
XLOC_0764sp|A4FVI0|ZSWM7_DANRE Zinc finger SWIM domain-containing protein 7 OS=Danio rerio GN=zswim7 PE=2 SV=2//9.80263e-20
XLOC_0768sp|Q93ZB1|LOL1_ARATH Protein LOL1 OS=Arabidopsis thaliana GN=LOL1 PE=2 SV=1//3.80161e-18
XLOC_0769sp|Q6VAF5|TBB7_GOSHI Tubulin beta-7 chain OS=Gossypium hirsutum PE=2 SV=1//0
XLOC_0769sp|Q39024|MPK4_ARATH Mitogen-activated protein kinase 4 OS=Arabidopsis thaliana GN=MPK4 PE=1 SV=2//1.49884e-74
XLOC_0770sp|Q9SG92|MES17_ARATH Methylesterase 17 OS=Arabidopsis thaliana GN=MES17 PE=1 SV=1//3.18179e-28
XLOC_0770sp|Q9C562|CIPKA_ARATH CBL-interacting serine/threonine-protein kinase 10 OS=Arabidopsis thaliana GN=CIPK10 PE=1 SV=1/
XLOC_0771sp|Q0JBP5|LOGL6_ORYSJ Probable cytokinin riboside 5'-monophosphate phosphoribohydrolase LOGL6 OS=Oryza sativa su
XLOC_0771-//-

XLOC_0772-//-

XLOC_0773sp|F4ISV6|THG2_ARATH tRNA(His) guanylyltransferase 2 OS=Arabidopsis thaliana GN=THG2 PE=1 SV=1//2.40072e-07

XLOC_0777sp|P53997|SET_DROME Protein SET OS=Drosophila melanogaster GN=Set PE=1 SV=2//9.58607e-53

XLOC_0779-//-

XLOC_0780-//-

XLOC_0782-//-

XLOC_0787sp|Q6TAW2|SERP2_MOUSE Stress-associated endoplasmic reticulum protein 2 OS=Mus musculus GN=Serp2 PE=2 SV=2//9.10479e-

XLOC_0789sp|Q9SAF0|ORP1D_ARATH Oxysterol-binding protein-related protein 1D OS=Arabidopsis thaliana GN=ORP1D PE=2 SV=1//2.5557

XLOC_0789sp|Q9SAF0|ORP1D_ARATH Oxysterol-binding protein-related protein 1D OS=Arabidopsis thaliana GN=ORP1D PE=2 SV=1//1.0426

XLOC_0789sp|Q5PQN1|HERC4_RAT Probable E3 ubiquitin-protein ligase HERC4 OS=Rattus norvegicus GN=Herc4 PE=2 SV=1//8.62193e-09

XLOC_0789-//-

XLOC_0789-//-

XLOC_0789-//-

XLOC_0789-//-

XLOC_0789sp|C0LGI2|Y1677_ARATH Probable LRR receptor-like serine/threonine-protein kinase At1g67720 OS=Arabidopsis thaliana GN

XLOC_0789sp|Q8LF36|2A5T_ARATH Serine/threonine protein phosphatase 2A 57 kDa regulatory subunit B' theta isoform OS=Arabi

XLOC_0789sp|Q9FT92|Y5843_ARATH Uncharacterized protein At5g08430 OS=Arabidopsis thaliana GN=At5g08430 PE=1 SV=2//2.05394e-62

XLOC_0789-//-

XLOC_0789sp|Q9CAF9|IOJAM_ARATH Protein Iojap-related, mitochondrial OS=Arabidopsis thaliana GN=At1g67620 PE=2 SV=1//2.43906e-4

XLOC_0791sp|Q14FC6|PETD_POPAL Cytochrome b6-f complex subunit 4 OS=Populus alba GN=petD PE=3 SV=1//2.36237e-104

XLOC_0793-//-

XLOC_0795-//-

XLOC_0797-//-

XLOC_0797sp|P0C2F6|RNHX1_ARATH Putative ribonuclease H protein At1g65750 OS=Arabidopsis thaliana GN=At1g65750 PE=3 SV=1//1.597

XLOC_0800sp|P29675|SF3_HELAN Pollen-specific protein SF3 OS=Helianthus annuus GN=SF3 PE=2 SV=1//9.93272e-14

XLOC_0800-//-

XLOC_0800sp|O64818|Y2309_ARATH Uncharacterized protein At2g23090 OS=Arabidopsis thaliana GN=At2g23090 PE=1 SV=1//2.52443e-42

XLOC_0801-//-

XLOC_0801sp|P93312|YMF16_ARATH Uncharacterized tatC-like protein ymf16 OS=Arabidopsis thaliana GN=YMF16 PE=2 SV=2//3.63004e-37

XLOC_0809sp|Q921Q3|ALG1_MOUSE Chitobiosyldiphosphodolichol beta-mannosyltransferase OS=Mus musculus GN=Alg1 PE=2 SV=3//3.94891

XLOC_0810sp|Q9MAB9|MUB1_ARATH Membrane-anchored ubiquitin-fold protein 1 OS=Arabidopsis thaliana GN=MUB1 PE=1 SV=1//3.80885e-4

XLOC_0810sp|Q8LBM4|ISAM1_ARATH Iron-sulfur assembly protein IscA-like 1, mitochondrial OS=Arabidopsis thaliana GN=At2g16710 PE

XLOC_0810sp|Q9SZ67|WRK19_ARATH Probable WRKY transcription factor 19 OS=Arabidopsis thaliana GN=WRKY19 PE=2 SV=1//4.38338e-35

XLOC_0812sp|Q8VZG8|Y4885_ARATH Probable LRR receptor-like serine/threonine-protein kinase At4g08850 OS=Arabidopsis thaliana GN

XLOC_0812sp|Q9VR59|VIP1_DROME Inositol hexakisphosphate and diphosphoinositol-pentakisphosphate kinase OS=Drosophila melanogas

XLOC_0813 sp|P10978|POLX_TOBAC Retrovirus-related Pol polyprotein from transposon TNT 1-94 OS=Nicotiana tabacum PE=2 SV=1//4.69
XLOC_0814 sp|P26520|G3PC_PETHY Glyceraldehyde-3-phosphate dehydrogenase, cytosolic OS=Petunia hybrida GN=GAPC PE=2 SV=1//1.6878
XLOC_0815 sp|Q9C9A9|COL7_ARATH Zinc finger protein CONSTANS-LIKE 7 OS=Arabidopsis thaliana GN=COL7 PE=2 SV=1//2.57046e-17
XLOC_0815 -//-
XLOC_0815 sp|Q9SZL8|FRS5_ARATH Protein FAR1-RELATED SEQUENCE 5 OS=Arabidopsis thaliana GN=FRS5 PE=2 SV=1//9.49594e-15
XLOC_0817 -//-
XLOC_0820 -//-
XLOC_0820 -//-
XLOC_0821 sp|P10978|POLX_TOBAC Retrovirus-related Pol polyprotein from transposon TNT 1-94 OS=Nicotiana tabacum PE=2 SV=1//1.75
XLOC_0821 sp|Q8LBC0|CCT13_ARATH Cyclin-T1-3 OS=Arabidopsis thaliana GN=CYCT1-3 PE=1 SV=2//3.63738e-17
XLOC_0823 sp|Q94AR8|LEUC_ARATH 3-isopropylmalate dehydratase OS=Arabidopsis thaliana GN=IIL1 PE=2 SV=1//8.76971e-15
XLOC_0825 sp|P14381|YTX2_XENLA Transposon TX1 uncharacterized 149 kDa protein OS=Xenopus laevis PE=4 SV=1//1.01226e-14
XLOC_0827 -//-
XLOC_0827 sp|P40940|ARF3_ARATH ADP-ribosylation factor 3 OS=Arabidopsis thaliana GN=ARF3 PE=1 SV=2//4.57016e-13
XLOC_0829 sp|Q55E69|SYS1_DICDI Protein SYS1 homolog OS=Dictyostelium discoideum GN=sys1 PE=3 SV=1//2.03946e-09
XLOC_0831 -//-
XLOC_0832 sp|Q9M1E7|TET3_ARATH Tetraspanin-3 OS=Arabidopsis thaliana GN=TET3 PE=2 SV=1//5.44835e-133
XLOC_0832 sp|Q9SK08|LBD11_ARATH LOB domain-containing protein 11 OS=Arabidopsis thaliana GN=LBD11 PE=2 SV=2//1.75777e-50
XLOC_0832 -//-
XLOC_0835 -//-
XLOC_0838 sp|P0C2F6|RNHX1_ARATH Putative ribonuclease H protein At1g65750 OS=Arabidopsis thaliana GN=At1g65750 PE=3 SV=1//1.084
XLOC_0840 sp|P10978|POLX_TOBAC Retrovirus-related Pol polyprotein from transposon TNT 1-94 OS=Nicotiana tabacum PE=2 SV=1//1.33
XLOC_0840 sp|Q2L951|YCF1_GOSHI Putative membrane protein ycf1 OS=Gossypium hirsutum GN=ycf1 PE=3 SV=1//2.87815e-36
XLOC_0840 -//-
XLOC_0840 sp|P0CH33|UBQ11_ARATH Polyubiquitin 11 OS=Arabidopsis thaliana GN=UBQ11 PE=1 SV=1//6.96845e-100
XLOC_0840 -//-
XLOC_0840 -//-
XLOC_0841 -//-
XLOC_0841 sp|P0CI03|PTR28_ARATH Putative peptide/nitrate transporter At2g37900 OS=Arabidopsis thaliana GN=At2g37900 PE=2 SV=1//
XLOC_0841 sp|Q9M331|PTR45_ARATH Probable peptide/nitrate transporter At3g53960 OS=Arabidopsis thaliana GN=At3g53960 PE=2 SV=2//
XLOC_0841 sp|Q945M8|CSPLI_ARATH CASP-like protein At3g53850 OS=Arabidopsis thaliana GN=At3g53850 PE=2 SV=1//4.09268e-57
XLOC_0841 sp|O81081|LAC2_ARATH Laccase-2 OS=Arabidopsis thaliana GN=LAC2 PE=2 SV=1//4.69308e-14
XLOC_0841 sp|Q9FJD5|LAC17_ARATH Laccase-17 OS=Arabidopsis thaliana GN=LAC17 PE=2 SV=1//2.85645e-36
XLOC_0841 sp|Q9FHR7|WRK49_ARATH Probable WRKY transcription factor 49 OS=Arabidopsis thaliana GN=WRKY49 PE=2 SV=1//7.14302e-16
XLOC_0841 sp|P10978|POLX_TOBAC Retrovirus-related Pol polyprotein from transposon TNT 1-94 OS=Nicotiana tabacum PE=2 SV=1//8.05

XLOC_0841 sp|Q40392|TMVRN_NICGU TMV resistance protein N OS=Nicotiana glutinosa GN=N PE=1 SV=1//3.39095e-08
XLOC_0842 -//-
XLOC_0842 sp|Q40392|TMVRN_NICGU TMV resistance protein N OS=Nicotiana glutinosa GN=N PE=1 SV=1//8.1041e-74
XLOC_0844 sp|Q54MZ3|CDC20_DICDI Anaphase-promoting complex subunit cdc20 OS=Dictyostelium discoideum GN=cdc20 PE=1 SV=1//1.7524
XLOC_0845 sp|Q8LG10|GAT15_ARATH GATA transcription factor 15 OS=Arabidopsis thaliana GN=GATA15 PE=2 SV=2//5.93077e-14
XLOC_0845 -//-
XLOC_0846 sp|Q8H5T6|LTI6A_ORYSJ Hydrophobic protein LTI6A OS=Oryza sativa subsp. japonica GN=LTI6A PE=2 SV=1//6.28954e-15
XLOC_0847 sp|P04323|POL3_DROME Retrovirus-related Pol polyprotein from transposon 17.6 OS=Drosophila melanogaster GN=pol PE=4 S
XLOC_0853 -//-
XLOC_0856 sp|Q0WPX7|BAG2_ARATH BAG family molecular chaperone regulator 2 OS=Arabidopsis thaliana GN=BAG2 PE=2 SV=1//1.95954e-2
XLOC_0856 sp|P29516|TBB8_ARATH Tubulin beta-8 chain OS=Arabidopsis thaliana GN=TUBB8 PE=2 SV=2//0
XLOC_0858 sp|Q9M8K6|MUTE_ARATH Transcription factor MUTE OS=Arabidopsis thaliana GN=MUTE PE=2 SV=1//2.61994e-74
XLOC_0859 sp|Q9LIQ6|ING1_ARATH PHD finger protein ING1 OS=Arabidopsis thaliana GN=ING1 PE=1 SV=1//9.26545e-32
XLOC_0860 -//-
XLOC_0862 sp|P10978|POLX_TOBAC Retrovirus-related Pol polyprotein from transposon TNT 1-94 OS=Nicotiana tabacum PE=2 SV=1//1.94
XLOC_0864 sp|Q9SQ57|PXG_SESIN Peroxygenase OS=Sesamum indicum GN=SOP1 PE=1 SV=1//8.98053e-97
XLOC_0864 sp|O22788|PXG3_ARATH Probable peroxygenase 3 OS=Arabidopsis thaliana GN=PXG3 PE=1 SV=1//6.93241e-34
XLOC_0864 sp|Q93Y94|RPOT1_NICSY DNA-directed RNA polymerase 1, mitochondrial OS=Nicotiana sylvestris GN=RPOT1 PE=2 SV=1//2.6676
XLOC_0864 sp|Q84WG1|NHX3_ARATH Sodium/hydrogen exchanger 3 OS=Arabidopsis thaliana GN=NHX3 PE=2 SV=2//1.22629e-36
XLOC_0865 -//-
XLOC_0867 sp|P08770|TRA1_MAIZE Putative AC transposase OS=Zea mays PE=2 SV=2//4.51457e-50
XLOC_0867 -//-
XLOC_0867 sp|A8FRR2|ARNA_SHESH Bifunctional polymyxin resistance protein ArnA OS=Shewanella sediminis (strain HAW-EB3) GN=arnA
XLOC_0868 sp|O80874|NDA2_ARATH Alternative NAD(P)H dehydrogenase 2, mitochondrial OS=Arabidopsis thaliana GN=NDA2 PE=1 SV=1//2.
XLOC_0868 sp|P49078|ASNS1_ARATH Asparagine synthetase [glutamine-hydrolyzing] 1 OS=Arabidopsis thaliana GN=ASN1 PE=2 SV=2//8.86
XLOC_0868 sp|O24661|ASNS_TRIVS Asparagine synthetase [glutamine-hydrolyzing] OS=Triphysaria versicolor GN=AS PE=2 SV=3//4.66226
XLOC_0868 sp|P49078|ASNS1_ARATH Asparagine synthetase [glutamine-hydrolyzing] 1 OS=Arabidopsis thaliana GN=ASN1 PE=2 SV=2//2.87
XLOC_0868 sp|O43918|AIRE_HUMAN Autoimmune regulator OS=Homo sapiens GN=AIRE PE=1 SV=1//6.37906e-11
XLOC_0868 -//-
XLOC_0868 sp|P05621|H2B2_WHEAT Histone H2B.2 OS=Triticum aestivum PE=1 SV=2//2.9806e-07
XLOC_0869 sp|P93295|M310_ARATH Uncharacterized mitochondrial protein AtMg00310 OS=Arabidopsis thaliana GN=AtMg00310 PE=4 SV=1//
XLOC_0869 sp|P93295|M310_ARATH Uncharacterized mitochondrial protein AtMg00310 OS=Arabidopsis thaliana GN=AtMg00310 PE=4 SV=1//
XLOC_0869 sp|Q42962|PGKY_TOBAC Phosphoglycerate kinase, cytosolic OS=Nicotiana tabacum PE=2 SV=1//3.1343e-15
XLOC_0869 sp|Q9S793|FRS8_ARATH Protein FAR1-RELATED SEQUENCE 8 OS=Arabidopsis thaliana GN=FRS8 PE=2 SV=2//0
XLOC_0872 sp|Q9FIJ2|NDUA2_ARATH NADH dehydrogenase [ubiquinone] 1 alpha subcomplex subunit 2 OS=Arabidopsis thaliana GN=At5g478

XLOC_0872 sp|P53811|PIPNB_MOUSE Phosphatidylinositol transfer protein beta isoform OS=Mus musculus GN=Pitpnb PE=1 SV=2//1.08964

XLOC_0873 -//-

XLOC_0873 sp|Q9LHN8|F6H1_ARATH Feruloyl CoA ortho-hydroxylase 1 OS=Arabidopsis thaliana GN=F6'H1 PE=1 SV=1//2.29895e-11

XLOC_0873 sp|Q9SKN3|CSPL6_ARATH CASP-like protein At2g28370 OS=Arabidopsis thaliana GN=At2g28370 PE=2 SV=1//1.42919e-14

XLOC_0874 sp|Q24K03|THUM1_BOVIN THUMP domain-containing protein 1 OS=Bos taurus GN=THUMPD1 PE=2 SV=1//8.12521e-11

XLOC_0874 sp|Q7LHG5|YI31B_YEAST Transposon Ty3-I Gag-Pol polyprotein OS=Saccharomyces cerevisiae (strain ATCC 204508 / S288c) G

XLOC_0875 sp|Q8BYR1|LCMT2_MOUSE Leucine carboxyl methyltransferase 2 OS=Mus musculus GN=Lcmt2 PE=2 SV=4//2.13771e-13

XLOC_0878 -//-

XLOC_0878 -//-

XLOC_0879 -//-

XLOC_0880 sp|Q03033|EF1A_WHEAT Elongation factor 1-alpha OS=Triticum aestivum GN=TEF1 PE=2 SV=1//6.72522e-30

XLOC_0881 -//-

XLOC_0881 sp|Q0PGJ6|AKRC9_ARATH Aldo-keto reductase family 4 member C9 OS=Arabidopsis thaliana GN=AKR4C9 PE=1 SV=1//1.80502e-15

XLOC_0882 sp|C7J4U3|OST4A_ORYSJ Dolichyl-diphosphooligosaccharide--protein glycosyltransferase subunit 4A OS=Oryza sativa subsp

XLOC_0882 -//-

XLOC_0882 sp|P25862|TBB1_AVESA Tubulin beta-1 chain (Fragment) OS=Avena sativa GN=TUBB1 PE=2 SV=1//1.83314e-62

XLOC_0886 -//-

XLOC_0886 sp|Q9SHJ8|HIBC8_ARATH 3-hydroxyisobutyryl-CoA hydrolase-like protein 5 OS=Arabidopsis thaliana GN=At1g06550 PE=2 SV=2

XLOC_0887 sp|Q7TS68|NSUN6_MOUSE Putative methyltransferase NSUN6 OS=Mus musculus GN=Nsun6 PE=2 SV=2//3.62167e-07

XLOC_0887 -//-

XLOC_0889 sp|Q5XF59|HIBC4_ARATH 3-hydroxyisobutyryl-CoA hydrolase-like protein 1, mitochondrial OS=Arabidopsis thaliana GN=At3g

XLOC_0890 sp|Q93ZR1|ELP3_ARATH Elongator complex protein 3 OS=Arabidopsis thaliana GN=HAG3 PE=1 SV=1//0

XLOC_0890 sp|A5PK39|TPP2_BOVIN Tripeptidyl-peptidase 2 OS=Bos taurus GN=TPP2 PE=2 SV=1//5.85208e-25

XLOC_0890 sp|Q9LYP4|BAG3_ARATH BAG family molecular chaperone regulator 3 OS=Arabidopsis thaliana GN=BAG3 PE=1 SV=1//2.71143e-1

XLOC_0890 sp|Q9FJ90|ERF25_ARATH Ethylene-responsive transcription factor ERF025 OS=Arabidopsis thaliana GN=ERF025 PE=2 SV=1//5.

XLOC_0890 sp|Q8VYB5|AMSH1_ARATH AMSH-like ubiquitin thioesterase 1 OS=Arabidopsis thaliana GN=AMSH1 PE=2 SV=1//1.24464e-44

XLOC_0891 sp|Q0IU52|ASP1_ORYSJ Aspartic proteinase Asp1 OS=Oryza sativa subsp. japonica GN=ASP1 PE=2 SV=1//1.16384e-85

XLOC_0894 sp|O81148|PSA4_ARATH Proteasome subunit alpha type-4 OS=Arabidopsis thaliana GN=PAC1 PE=1 SV=1//1.95129e-159

XLOC_0894 sp|P37707|B2_DAUCA B2 protein OS=Daucus carota PE=2 SV=1//8.55382e-90

XLOC_0895 -//-

XLOC_0897 sp|Q8VZJ9|CRCK2_ARATH Calmodulin-binding receptor-like cytoplasmic kinase 2 OS=Arabidopsis thaliana GN=CRCK2 PE=2 SV=

XLOC_0898 sp|Q9FPW6|POB1_ARATH BTB/POZ domain-containing protein POB1 OS=Arabidopsis thaliana GN=POB1 PE=2 SV=2//2.07834e-73

XLOC_0899 sp|Q9C671|P4K2B_ARATH Probable phosphatidylinositol 4-kinase type 2-beta At1g26270 OS=Arabidopsis thaliana GN=At1g262

XLOC_0899 sp|Q84JU4|IBR5_ARATH Protein-tyrosine-phosphatase IBR5 OS=Arabidopsis thaliana GN=IBR5 PE=1 SV=1//4.1338e-18

XLOC_0901 -//-

XLOC_0902	-//-
XLOC_0904	sp|P25093|FAAA_RAT Fumarylacetoacetase OS=Rattus norvegicus GN=Fah PE=1 SV=1//6.72224e-13
XLOC_0904	sp|P10978|POLX_TOBAC Retrovirus-related Pol polyprotein from transposon TNT 1-94 OS=Nicotiana tabacum PE=2 SV=1//5.15
XLOC_0905	sp|Q8LDV3|Y4320_ARATH Uncharacterized protein At4g13200, chloroplastic OS=Arabidopsis thaliana GN=At4g13200 PE=1 SV=2
XLOC_0909	sp|Q3E6Q1|PPR32_ARATH Pentatricopeptide repeat-containing protein At1g11290 OS=Arabidopsis thaliana GN=PCMP-H40 PE=2
XLOC_0910	-//-
XLOC_0910	-//-
XLOC_0912	sp|P93295|M310_ARATH Uncharacterized mitochondrial protein AtMg00310 OS=Arabidopsis thaliana GN=AtMg00310 PE=4 SV=1//
XLOC_0915	-//-
XLOC_0916	sp|Q8H1U4|APC5_ARATH Anaphase-promoting complex subunit 5 OS=Arabidopsis thaliana GN=APC5 PE=2 SV=1//2.60373e-74
XLOC_0916	sp|Q38942|RAE1L_ARATH Rae1-like protein At1g80670 OS=Arabidopsis thaliana GN=At1g80670 PE=2 SV=2//6.63155e-33
XLOC_0918	-//-
XLOC_0919	sp|A6P6V9|CBDAS_CANSA Cannabidiolic acid synthase OS=Cannabis sativa GN=CBDAS PE=1 SV=1//5.48004e-170
XLOC_0919	sp|Q8W234|SEUSS_ARATH Transcriptional corepressor SEUSS OS=Arabidopsis thaliana GN=SEU PE=1 SV=1//1.69617e-94
XLOC_0919	-//-
XLOC_0919	sp|Q9SIA1|MATE8_ARATH MATE efflux family protein 8 OS=Arabidopsis thaliana GN=DTXL4 PE=2 SV=2//1.14672e-09
XLOC_0921	sp|Q08467|CSK21_ARATH Casein kinase II subunit alpha-1 OS=Arabidopsis thaliana GN=CKA1 PE=1 SV=3//1.25878e-84
XLOC_0924	sp|P10978|POLX_TOBAC Retrovirus-related Pol polyprotein from transposon TNT 1-94 OS=Nicotiana tabacum PE=2 SV=1//1.98
XLOC_0925	sp|Q7XVA8|RIP1_ORYSJ Putative ripening-related protein 1 OS=Oryza sativa subsp. japonica GN=Os04g0364800 PE=3 SV=2//6
XLOC_0927	sp|Q0DBU5|RH31_ORYSJ DEAD-box ATP-dependent RNA helicase 31 OS=Oryza sativa subsp. japonica GN=Os06g0526600 PE=3 SV=2
XLOC_0927	-//-
XLOC_0928	-//-
XLOC_0928	-//-
XLOC_0928	-//-
XLOC_0928	sp|Q9FZ19|PPR5_ARATH Putative pentatricopeptide repeat-containing protein At1g02420 OS=Arabidopsis thaliana GN=At1g02
XLOC_0929	sp|O35900|LSM2_MOUSE U6 snRNA-associated Sm-like protein LSm2 OS=Mus musculus GN=Lsm2 PE=2 SV=1//1.41262e-24
XLOC_0931	sp|Q8VZU9|DERL1_ARATH Derlin-1 OS=Arabidopsis thaliana GN=DER1 PE=2 SV=1//9.93508e-95
XLOC_0933	sp|Q9SF24|LOR10_ARATH Protein LURP-one-related 10 OS=Arabidopsis thaliana GN=At3g11740 PE=2 SV=2//1.1769e-10
XLOC_0934	sp|Q8LDV3|Y4320_ARATH Uncharacterized protein At4g13200, chloroplastic OS=Arabidopsis thaliana GN=At4g13200 PE=1 SV=2
XLOC_0938	sp|Q6GQ69|FAF2B_XENLA FAS-associated factor 2-B OS=Xenopus laevis GN=faf2-b PE=2 SV=1//5.68193e-11
XLOC_0938	sp|P0C2F6|RNHX1_ARATH Putative ribonuclease H protein At1g65750 OS=Arabidopsis thaliana GN=At1g65750 PE=3 SV=1//2.738
XLOC_0938	sp|Q9M612|NACA_PINTA Nascent polypeptide-associated complex subunit alpha-like protein OS=Pinus taeda PE=2 SV=1//1.02
XLOC_0939	sp|Q9UQ35|SRRM2_HUMAN Serine/arginine repetitive matrix protein 2 OS=Homo sapiens GN=SRRM2 PE=1 SV=2//2.87384e-12
XLOC_0939	sp|P49105|G6PI_MAIZE Glucose-6-phosphate isomerase, cytosolic OS=Zea mays GN=PHI1 PE=2 SV=1//6.97446e-11
XLOC_0945	-//-

XLOC_0948	-//-
XLOC_0948	-//-
XLOC_0948	-//-
XLOC_0953	-//-
XLOC_0954	-//-
XLOC_0955	sp|Q2L907|PSAA_GOSHI Photosystem I P700 chlorophyll a apoprotein A1 OS=Gossypium hirsutum GN=psaA PE=3 SV=1//1.61302e
XLOC_0955	-//-
XLOC_0958	sp|Q9LLC1|BCCP2_ARATH Biotin carboxyl carrier protein of acetyl-CoA carboxylase 2, chloroplastic OS=Arabidopsis thali
XLOC_0960	sp|Q5E964|PSD13_BOVIN 26S proteasome non-ATPase regulatory subunit 13 OS=Bos taurus GN=PSMD13 PE=2 SV=1//1.40819e-10
XLOC_0961	-//-
XLOC_0963	sp|P93295|M310_ARATH Uncharacterized mitochondrial protein AtMg00310 OS=Arabidopsis thaliana GN=AtMg00310 PE=4 SV=1//
XLOC_0965	-//-
XLOC_0965	sp|Q9LK39|AAE16_ARATH Probable acyl-activating enzyme 16, chloroplastic OS=Arabidopsis thaliana GN=AAE16 PE=2 SV=1//2
XLOC_0965	sp|P42856|ZB14_MAIZE 14 kDa zinc-binding protein OS=Zea mays GN=ZBP14 PE=1 SV=1//3.10597e-09
XLOC_0965	sp|Q8W471|AAE15_ARATH Long-chain-fatty-acid--[acyl-carrier-protein] ligase AEE15, chloroplastic OS=Arabidopsis thalia
XLOC_0968	sp|P93732|PIP_ARATH Proline iminopeptidase OS=Arabidopsis thaliana GN=PIP PE=2 SV=3//1.23826e-150
XLOC_0968	sp|P33154|PR1_ARATH Pathogenesis-related protein 1 OS=Arabidopsis thaliana GN=At2g14610 PE=1 SV=1//3.86737e-72
XLOC_0968	sp|Q9FKS4|ATR_ARATH Serine/threonine-protein kinase ATR OS=Arabidopsis thaliana GN=ATR PE=2 SV=2//1.17767e-15
XLOC_0968	-//-
XLOC_0968	sp|Q5TAQ9|DCAF8_HUMAN DDB1- and CUL4-associated factor 8 OS=Homo sapiens GN=DCAF8 PE=1 SV=1//3.1793e-21
XLOC_0968	-//-
XLOC_0968	sp|Q9ZUP2|RECA3_ARATH DNA repair protein recA homolog 3, mitochondrial OS=Arabidopsis thaliana GN=At2g19490 PE=2 SV=2
XLOC_0968	sp|P81393|MYB08_ANTMA Myb-related protein 308 OS=Antirrhinum majus GN=MYB308 PE=2 SV=1//1.30539e-48
XLOC_0968	-//-
XLOC_0969	sp|Q38899|ORC2_ARATH Origin recognition complex subunit 2 OS=Arabidopsis thaliana GN=ORC2 PE=1 SV=1//1.43292e-07
XLOC_0970	-//-
XLOC_0970	sp|P84752|PERA_ALOVR Peroxidase A (Fragments) OS=Aloe vera PE=1 SV=1//1.29806e-08
XLOC_0970	sp|Q9M086|DCAF1_ARATH DDB1- and CUL4-associated factor homolog 1 OS=Arabidopsis thaliana GN=DCAF1 PE=1 SV=2//1.99699e
XLOC_0971	sp|Q8L649|BB_ARATH E3 ubiquitin ligase BIG BROTHER OS=Arabidopsis thaliana GN=BB PE=1 SV=1//4.18349e-08
XLOC_0972	-//-
XLOC_0974	sp|O80507|CSK2E_ARATH Putative casein kinase II subunit beta-4 OS=Arabidopsis thaliana GN=At2g44680 PE=2 SV=1//8.1753
XLOC_0974	sp|P40228|CSK2B_ARATH Casein kinase II subunit beta OS=Arabidopsis thaliana GN=CKB1 PE=1 SV=1//7.31049e-64
XLOC_0976	sp|O04895|BADH_AMAHP Betaine aldehyde dehydrogenase, chloroplastic OS=Amaranthus hypochondriacus GN=BADH4 PE=3 SV=1//
XLOC_0978	sp|Q5RCV3|NBN_PONAB Nibrin OS=Pongo abelii GN=NBN PE=2 SV=2//1.92424e-08
XLOC_0978	-//-

XLOC_0979 -//-
XLOC_0983 -//-
XLOC_0984 -//-
XLOC_0985 sp|Q9M2C9|SKIP4_ARATH F-box/kelch-repeat protein SKIP4 OS=Arabidopsis thaliana GN=SKIP4 PE=1 SV=1//2.80676e-120
XLOC_0985 sp|Q93ZQ5|SPXM3_ARATH SPX domain-containing membrane protein At4g22990 OS=Arabidopsis thaliana GN=At4g22990 PE=2 SV=2
XLOC_0986 -//-
XLOC_0987 -//-
XLOC_0990 sp|Q9FH99|FB302_ARATH F-box protein At5g67140 OS=Arabidopsis thaliana GN=At5g67140 PE=2 SV=1//4.8701e-110
XLOC_0991 sp|Q9C5W3|NADK2_ARATH NAD kinase 2, chloroplastic OS=Arabidopsis thaliana GN=NADK2 PE=1 SV=1//4.23124e-19
XLOC_0991 -//-
XLOC_0994 -//-
XLOC_0994 sp|Q9M2F1|RS272_ARATH 40S ribosomal protein S27-2 OS=Arabidopsis thaliana GN=RPS27B PE=2 SV=2//2.89239e-43
XLOC_0996 sp|Q7YJX2|PSAA_CALFG Photosystem I P700 chlorophyll a apoprotein A1 OS=Calycanthus floridus var. glaucus GN=psaA PE=3
XLOC_0997 -//-
XLOC_0998 -//-
XLOC_0998 sp|P11369|POL2_MOUSE Retrovirus-related Pol polyprotein LINE-1 OS=Mus musculus GN=Pol PE=1 SV=2//2.33245e-24
XLOC_0999 sp|Q2V3H0|PP322_ARATH Pentatricopeptide repeat-containing protein At4g18975, chloroplastic OS=Arabidopsis thaliana GN
XLOC_1008 -//-
XLOC_1009 sp|P80093|PDS_CAPAN 15-cis-phytoene desaturase, chloroplastic/chromoplastic OS=Capsicum annuum GN=PDS PE=1 SV=1//1.49
XLOC_1010 sp|Q8LFH6|ADF12_ARATH Actin-depolymerizing factor 12 OS=Arabidopsis thaliana GN=ADF12 PE=2 SV=2//4.29836e-14
XLOC_1010 sp|Q8L4H4|NORK_MEDTR Nodulation receptor kinase OS=Medicago truncatula GN=NORK PE=1 SV=2//2.99491e-48
XLOC_1010 sp|Q8LKZ1|NORK_PEA Nodulation receptor kinase OS=Pisum sativum GN=NORK PE=1 SV=1//3.27163e-65
XLOC_1011 sp|P28188|RAD2A_ARATH Ras-related protein RABD2a OS=Arabidopsis thaliana GN=RABD2A PE=1 SV=3//1.1752e-47
XLOC_1012 -//-
XLOC_1013 sp|Q7XLX6|S1FA2_ORYSJ DNA-binding protein S1FA2 OS=Oryza sativa subsp. japonica GN=S1FA2 PE=3 SV=2//4.58663e-06
XLOC_1013 sp|P69322|UBIQP_PEA Polyubiquitin OS=Pisum sativum GN=PU1 PE=2 SV=2//0
XLOC_1015 -//-
XLOC_1016 -//-
XLOC_1016 -//-
XLOC_1018 sp|O22187|Y2232_ARATH Probable receptor-like protein kinase At2g23200 OS=Arabidopsis thaliana GN=At2g23200 PE=2 SV=1/
XLOC_1018 -//-
XLOC_1018 sp|P10978|POLX_TOBAC Retrovirus-related Pol polyprotein from transposon TNT 1-94 OS=Nicotiana tabacum PE=2 SV=1//2.96
XLOC_1020 sp|Q9ZTN2|ERD2_PETHY ER lumen protein retaining receptor OS=Petunia hybrida GN=ERD2 PE=2 SV=1//1.18494e-106
XLOC_1021 sp|Q39676|DCAM1_DIACA S-adenosylmethionine decarboxylase proenzyme 1 OS=Dianthus caryophyllus GN=SAMDC1 PE=2 SV=1//3.
XLOC_1023 sp|P81392|MYB06_ANTMA Myb-related protein 306 OS=Antirrhinum majus GN=MYB306 PE=2 SV=1//2.90288e-78

XLOC_1023 sp|P10978|POLX_TOBAC Retrovirus-related Pol polyprotein from transposon TNT 1-94 OS=Nicotiana tabacum PE=2 SV=1//1.01
XLOC_1023 -//-
XLOC_1024 sp|P10978|POLX_TOBAC Retrovirus-related Pol polyprotein from transposon TNT 1-94 OS=Nicotiana tabacum PE=2 SV=1//5.23
XLOC_1025 sp|Q9JIX0|ENY2_MOUSE Enhancer of yellow 2 transcription factor homolog OS=Mus musculus GN=Eny2 PE=2 SV=1//6.86511e-20
XLOC_1027 sp|P38385|SC61G_ORYSJ Protein transport protein Sec61 subunit gamma OS=Oryza sativa subsp. japonica GN=Os02g0178400 P
XLOC_1029 -//-
XLOC_1029 sp|Q9SK82|U85A1_ARATH UDP-glycosyltransferase 85A1 OS=Arabidopsis thaliana GN=UGT85A1 PE=1 SV=1//1.42199e-78
XLOC_1029 -//-
XLOC_1029 -//-
XLOC_1030 sp|Q04050|NU4M_BRACM NADH-ubiquinone oxidoreductase chain 4 OS=Brassica campestris GN=ND4 PE=3 SV=1//2.80486e-56
XLOC_1030 sp|A9X7L0|ANMT_RUTGR Anthranilate N-methyltransferase OS=Ruta graveolens PE=1 SV=1//4.20658e-10
XLOC_1030 sp|P0C2F6|RNHX1_ARATH Putative ribonuclease H protein At1g65750 OS=Arabidopsis thaliana GN=At1g65750 PE=3 SV=1//2.627
XLOC_1033 sp|Q9LK39|AAE16_ARATH Probable acyl-activating enzyme 16, chloroplastic OS=Arabidopsis thaliana GN=AAE16 PE=2 SV=1//0
XLOC_1034 sp|Q9C9H7|RLP12_ARATH Receptor-like protein 12 OS=Arabidopsis thaliana GN=RLP12 PE=2 SV=2//1.29148e-08
XLOC_1035 sp|O64644|SAP18_ARATH Histone deacetylase complex subunit SAP18 OS=Arabidopsis thaliana GN=At2g45640 PE=1 SV=1//3.329
XLOC_1035 -//-
XLOC_1035 sp|P10978|POLX_TOBAC Retrovirus-related Pol polyprotein from transposon TNT 1-94 OS=Nicotiana tabacum PE=2 SV=1//1.70
XLOC_1037 -//-
XLOC_1037 sp|Q8L736|SKI11_ARATH F-box/kelch-repeat protein SKIP11 OS=Arabidopsis thaliana GN=SKIP11 PE=1 SV=2//1.89485e-171
XLOC_1040 -//-
XLOC_1041 -//-
XLOC_1041 sp|O04057|ASPR_CUCPE Aspartic proteinase OS=Cucurbita pepo PE=2 SV=1//6.98998e-23
XLOC_1041 sp|Q7K4W1|CDKAL_DROME Threonylcarbamoyladenosine tRNA methylthiotransferase OS=Drosophila melanogaster GN=CG6550 PE=2
XLOC_1045 sp|Q67YU0|CKX5_ARATH Cytokinin dehydrogenase 5 OS=Arabidopsis thaliana GN=CKX5 PE=2 SV=1//1.24299e-73
XLOC_1045 sp|Q84J37|LAC15_ARATH Laccase-15 OS=Arabidopsis thaliana GN=TT10 PE=1 SV=1//1.21299e-10
XLOC_1045 sp|P0C8Z0|Y8359_ORYSI Uncharacterized protein OsI_027940 OS=Oryza sativa subsp. indica GN=OsI_027940 PE=1 SV=2//1.167
XLOC_1050 sp|Q9FIG9|ARC6_ARATH Protein ACCUMULATION AND REPLICATION OF CHLOROPLASTS 6, chloroplastic OS=Arabidopsis thaliana GN
XLOC_1052 -//-
XLOC_1053 sp|Q9M7Q4|AI5L5_ARATH ABSCISIC ACID-INSENSITIVE 5-like protein 5 OS=Arabidopsis thaliana GN=ABF2 PE=1 SV=1//2.89329e-
XLOC_1055 sp|Q6VAF6|TBB6_GOSHI Tubulin beta-6 chain OS=Gossypium hirsutum PE=2 SV=1//0
XLOC_1056 sp|Q9FH37|ILR3_ARATH Transcription factor ILR3 OS=Arabidopsis thaliana GN=ILR3 PE=1 SV=1//1.92792e-35
XLOC_1057 sp|P42620|YQJG_ECOLI Glutathionyl-hydroquinone reductase YqjG OS=Escherichia coli (strain K12) GN=yqjG PE=1 SV=1//7.7
XLOC_1058 -//-
XLOC_1058 -//-
XLOC_1058 sp|Q93Y37|MNS3_ARATH Mannosyl-oligosaccharide 1,2-alpha-mannosidase MNS3 OS=Arabidopsis thaliana GN=MNS3 PE=2 SV=1//6

XLOC_1058 sp|Q93Y37|MNS3_ARATH Mannosyl-oligosaccharide 1,2-alpha-mannosidase MNS3 OS=Arabidopsis thaliana GN=MNS3 PE=2 SV=1//2
XLOC_1061 -//-
XLOC_1062 sp|P42055|VDAC1_SOLTU Mitochondrial outer membrane protein porin of 34 kDa OS=Solanum tuberosum PE=1 SV=2//1.62231e-1
XLOC_1064 sp|Q93ZT6|IF4G1_ARATH Eukaryotic translation initiation factor isoform 4G-1 OS=Arabidopsis thaliana GN=EIF(ISO)4G1 PE
XLOC_1064 sp|Q9LDX1|SGS3_ARATH Protein SUPPRESSOR OF GENE SILENCING 3 OS=Arabidopsis thaliana GN=SGS3 PE=1 SV=1//8.60482e-174
XLOC_1064 sp|Q9SUV1|BRT1_ARATH Adenine nucleotide transporter BT1, chloroplastic/mitochondrial OS=Arabidopsis thaliana GN=BT1 P
XLOC_1064 sp|Q5SMK6|P2C54_ORYSJ Probable protein phosphatase 2C 54 OS=Oryza sativa subsp. japonica GN=Os06g0179700 PE=2 SV=1//3
XLOC_1064 -//-
XLOC_1064 sp|Q9SZE1|3HID1_ARATH Probable 3-hydroxyisobutyrate dehydrogenase-like 1, mitochondrial OS=Arabidopsis thaliana GN=At
XLOC_1066 sp|Q9LY41|ATL4_ARATH E3 ubiquitin-protein ligase ATL4 OS=Arabidopsis thaliana GN=ATL4 PE=1 SV=1//6.25306e-09
XLOC_1066 sp|P93022|ARFG_ARATH Auxin response factor 7 OS=Arabidopsis thaliana GN=ARF7 PE=1 SV=2//0
XLOC_1068 -//-
XLOC_1069 sp|Q0JBP5|LOGL6_ORYSJ Probable cytokinin riboside 5'-monophosphate phosphoribohydrolase LOGL6 OS=Oryza sativa su
XLOC_1073 sp|Q40412|ABA2_NICPL Zeaxanthin epoxidase, chloroplastic OS=Nicotiana plumbaginifolia GN=ABA2 PE=1 SV=1//3.95127e-08
XLOC_1075 sp|Q9ZUX4|UMP2_ARATH Uncharacterized protein At2g27730, mitochondrial OS=Arabidopsis thaliana GN=At2g27730 PE=1 SV=1/
XLOC_1075 sp|P28644|ROC1_SPIOL 28 kDa ribonucleoprotein, chloroplastic OS=Spinacia oleracea PE=1 SV=1//3.65772e-06
XLOC_1075 -//-
XLOC_1076 sp|Q9S9K4|ASPL2_ARATH Aspartic proteinase-like protein 2 OS=Arabidopsis thaliana GN=At1g65240 PE=1 SV=2//3.74336e-37
XLOC_1076 -//-
XLOC_1076 sp|Q9ZVR5|PP2B2_ARATH Putative F-box protein PP2-B2 OS=Arabidopsis thaliana GN=PP2B2 PE=2 SV=2//7.67769e-50
XLOC_1076 sp|Q3E6P4|FB95_ARATH F-box protein At2g02240 OS=Arabidopsis thaliana GN=At2g02240 PE=2 SV=1//1.20853e-39
XLOC_1076 sp|Q9SCQ2|UPL7_ARATH E3 ubiquitin-protein ligase UPL7 OS=Arabidopsis thaliana GN=UPL7 PE=2 SV=1//4.12866e-42
XLOC_1076 -//-
XLOC_1076 sp|Q6BQ20|ATG13_DEBHA Autophagy-related protein 13 OS=Debaryomyces hansenii (strain ATCC 36239 / CBS 767 / JCM 1990 /
XLOC_1077 sp|Q39202|RLK1_ARATH G-type lectin S-receptor-like serine/threonine-protein kinase RLK1 OS=Arabidopsis thaliana GN=RL
XLOC_1077 sp|Q39202|RLK1_ARATH G-type lectin S-receptor-like serine/threonine-protein kinase RLK1 OS=Arabidopsis thaliana GN=RL
XLOC_1079 -//-
XLOC_1080 sp|P0C2F6|RNHX1_ARATH Putative ribonuclease H protein At1g65750 OS=Arabidopsis thaliana GN=At1g65750 PE=3 SV=1//5.052
XLOC_1081 sp|Q8GXN6|VP201_ARATH Vacuolar protein sorting-associated protein 20 homolog 1 OS=Arabidopsis thaliana GN=VPS20.1 PE=
XLOC_1081 sp|Q93WV4|WRK71_ARATH Probable WRKY transcription factor 71 OS=Arabidopsis thaliana GN=WRKY71 PE=2 SV=1//9.72917e-49
XLOC_1081 sp|Q7DM39|PSBP1_TOBAC Oxygen-evolving enhancer protein 2-1, chloroplastic OS=Nicotiana tabacum GN=PSBP1 PE=3 SV=2//7.
XLOC_1081 -//-
XLOC_1082 sp|Q9XGM1|VATD_ARATH V-type proton ATPase subunit D OS=Arabidopsis thaliana GN=VHA-D PE=1 SV=2//8.51853e-33
XLOC_1083 sp|P08770|TRA1_MAIZE Putative AC transposase OS=Zea mays PE=2 SV=2//4.84006e-91
XLOC_1083 -//-

XLOC_1084	sp|P0C031|RUB2_ORYSJ Ubiquitin-NEDD8-like protein RUB2 OS=Oryza sativa subsp. japonica GN=RUB2 PE=2 SV=2//8.67301e-16
XLOC_1084	sp|P0C073|RUB1_DESAN Ubiquitin-NEDD8-like protein RUB1 OS=Deschampsia antarctica GN=RUB1 PE=2 SV=2//1.43338e-16
XLOC_1086	sp|P93295|M310_ARATH Uncharacterized mitochondrial protein AtMg00310 OS=Arabidopsis thaliana GN=AtMg00310 PE=4 SV=1//
XLOC_1088	sp|P04146|COPIA_DROME Copia protein OS=Drosophila melanogaster GN=GIP PE=1 SV=3//9.0029e-12
XLOC_1088	sp|Q9SCX9|GPDA1_ARATH Glycerol-3-phosphate dehydrogenase [NAD(+)] 1, chloroplastic OS=Arabidopsis thaliana GN=DHAPRD
XLOC_1090	sp|Q0WMN5|Y3913_ARATH Uncharacterized protein At3g49140 OS=Arabidopsis thaliana GN=At3g49140 PE=1 SV=2//5.2295e-09
XLOC_1094	sp|Q9C9F4|COLX_ARATH Putative zinc finger protein At1g68190 OS=Arabidopsis thaliana GN=At1g68190 PE=2 SV=1//1.02331e-
XLOC_1094	sp|Q9LNG5|PPP7L_ARATH Serine/threonine-protein phosphatase 7 long form homolog OS=Arabidopsis thaliana GN=At1g48120 P
XLOC_1096	sp|P0C2F6|RNHX1_ARATH Putative ribonuclease H protein At1g65750 OS=Arabidopsis thaliana GN=At1g65750 PE=3 SV=1//5.625
XLOC_1097	sp|Q9VTY4|KXDL1_DROME KxDL motif-containing protein CG10681 OS=Drosophila melanogaster GN=CG10681 PE=2 SV=1//3.30051e
XLOC_1097	sp|Q8GYB1|NUD15_ARATH Nudix hydrolase 15, mitochondrial OS=Arabidopsis thaliana GN=NUDT15 PE=1 SV=2//1.70777e-80
XLOC_1099	-//-
XLOC_1099	sp|P10978|POLX_TOBAC Retrovirus-related Pol polyprotein from transposon TNT 1-94 OS=Nicotiana tabacum PE=2 SV=1//2.79
XLOC_1100	sp|Q9SHS7|BSL3_ARATH Serine/threonine-protein phosphatase BSL3 OS=Arabidopsis thaliana GN=BSL3 PE=1 SV=2//3.4444e-18
XLOC_1101	sp|P37224|MAOM_AMAHP NAD-dependent malic enzyme 65 kDa isoform, mitochondrial OS=Amaranthus hypochondriacus PE=1 SV=1
XLOC_1101	sp|Q8LBZ4|OTU_ARATH OTU domain-containing protein At3g57810 OS=Arabidopsis thaliana GN=At3g57810 PE=2 SV=1//2.45467e-
XLOC_1101	-//-
XLOC_1104	-//-
XLOC_1108	-//-
XLOC_1112	sp|B3GS44|LRE_ARATH GPI-anchored protein LORELEI OS=Arabidopsis thaliana GN=LRE PE=2 SV=1//4.85468e-21
XLOC_1117	sp|Q6R8G7|PHO13_ARATH Phosphate transporter PHO1 homolog 3 OS=Arabidopsis thaliana GN=PHO1;H3 PE=2 SV=2//0
XLOC_1117	-//-
XLOC_1117	sp|Q2L955|NU4C_GOSHI NAD(P)H-quinone oxidoreductase chain 4, chloroplastic OS=Gossypium hirsutum GN=ndhD PE=3 SV=1//1
XLOC_1117	-//-
XLOC_1119	-//-
XLOC_1119	sp|P10978|POLX_TOBAC Retrovirus-related Pol polyprotein from transposon TNT 1-94 OS=Nicotiana tabacum PE=2 SV=1//5.07
XLOC_1119	-//-
XLOC_1120	sp|Q94BR4|PR19A_ARATH Pre-mRNA-processing factor 19 homolog 1 OS=Arabidopsis thaliana GN=PRP19A PE=1 SV=1//2.26419e-0
XLOC_1120	-//-
XLOC_1123	sp|O23712|PSA1B_ARATH Proteasome subunit alpha type-1-B OS=Arabidopsis thaliana GN=PAF2 PE=1 SV=2//2.36057e-155
XLOC_1124	sp|Q9M0X9|4CLL7_ARATH 4-coumarate--CoA ligase-like 7 OS=Arabidopsis thaliana GN=4CLL7 PE=1 SV=1//8.58044e-38
XLOC_1124	sp|P74038|RSMI_SYNY3 Ribosomal RNA small subunit methyltransferase I OS=Synechocystis sp. (strain PCC 6803 / Kazusa)
XLOC_1125	-//-
XLOC_1126	sp|Q9SYT0|ANXD1_ARATH Annexin D1 OS=Arabidopsis thaliana GN=ANN1 PE=1 SV=1//5.38625e-151
XLOC_1131	sp|C7J4U3|OST4A_ORYSJ Dolichyl-diphosphooligosaccharide--protein glycosyltransferase subunit 4A OS=Oryza sativa subsp

XLOC_1132	sp|P10978|POLX_TOBAC Retrovirus-related Pol polyprotein from transposon TNT 1-94 OS=Nicotiana tabacum PE=2 SV=1//1.57
XLOC_1135	sp|Q8L8B8|LOG3_ARATH Cytokinin riboside 5'-monophosphate phosphoribohydrolase LOG3 OS=Arabidopsis thaliana GN=LO
XLOC_1138	-//-
XLOC_1139	-//-
XLOC_1140	-//-
XLOC_1140	-//-
XLOC_1142	-//-
XLOC_1144	sp|Q6AT26|CESA1_ORYSJ Probable cellulose synthase A catalytic subunit 1 [UDP-forming] OS=Oryza sativa subsp. japonica
XLOC_1144	sp|O48946|CESA1_ARATH Cellulose synthase A catalytic subunit 1 [UDP-forming] OS=Arabidopsis thaliana GN=CESA1 PE=1 SV
XLOC_1144	sp|O22969|Y2416_ARATH Uncharacterized protein At2g34160 OS=Arabidopsis thaliana GN=At2g34160 PE=1 SV=1//2.76133e-50
XLOC_1144	sp|Q9XI33|WIN1_ARATH Ethylene-responsive transcription factor WIN1 OS=Arabidopsis thaliana GN=WIN1 PE=2 SV=1//3.48496
XLOC_1146	-//-
XLOC_1149	sp|Q53YF3|SPC3B_ARATH Signal peptidase complex subunit 3B OS=Arabidopsis thaliana GN=At5g27430 PE=2 SV=1//6.78868e-93
XLOC_1150	sp|P28186|RAE1C_ARATH Ras-related protein RABE1c OS=Arabidopsis thaliana GN=RABE1C PE=1 SV=1//2.75165e-27
XLOC_1152	-//-
XLOC_1153	sp|Q9M9M9|NDUAC_ARATH Probable NADH dehydrogenase [ubiquinone] 1 alpha subcomplex subunit 12 OS=Arabidopsis thaliana
XLOC_1156	-//-
XLOC_1156	sp|Q9LUN2|YSL5_ARATH Probable metal-nicotianamine transporter YSL5 OS=Arabidopsis thaliana GN=YSL5 PE=1 SV=1//1.51845
XLOC_1156	sp|Q9V3X4|BSCL2_DROME Seipin OS=Drosophila melanogaster GN=Seipin PE=2 SV=1//2.84671e-06
XLOC_1156	sp|Q9FFD0|PIN8_ARATH Putative auxin efflux carrier component 8 OS=Arabidopsis thaliana GN=PIN8 PE=2 SV=2//6.19844e-17
XLOC_1156	sp|Q9AT00|AB13I_ARATH Protein TRIGALACTOSYLDIACYLGLYCEROL 3, chloroplastic OS=Arabidopsis thaliana GN=TGD3 PE=1 SV=1/
XLOC_1156	sp|Q9FZ87|GH317_ARATH Indole-3-acetic acid-amido synthetase GH3.17 OS=Arabidopsis thaliana GN=GH3.17 PE=1 SV=1//1.841
XLOC_1156	sp|O81028|PP171_ARATH Pentatricopeptide repeat-containing protein At2g26790, mitochondrial OS=Arabidopsis thaliana GN
XLOC_1157	-//-
XLOC_1159	sp|Q24733|ACT_DICVI Actin (Fragment) OS=Dictyocaulus viviparus PE=3 SV=1//9.25161e-08
XLOC_1160	sp|Q9SJG2|Y2296_ARATH Probable receptor-like protein kinase At2g42960 OS=Arabidopsis thaliana GN=At2g42960 PE=2 SV=1/
XLOC_1161	sp|Q94A68|Y1669_ARATH Uncharacterized oxidoreductase At1g06690, chloroplastic OS=Arabidopsis thaliana GN=At1g06690 PE
XLOC_1162	sp|P08770|TRA1_MAIZE Putative AC transposase OS=Zea mays PE=2 SV=2//5.93813e-90
XLOC_1165	sp|Q15751|HERC1_HUMAN Probable E3 ubiquitin-protein ligase HERC1 OS=Homo sapiens GN=HERC1 PE=1 SV=2//2.11674e-06
XLOC_1167	sp|Q6J163|5NG4_PINTA Auxin-induced protein 5NG4 OS=Pinus taeda PE=2 SV=1//1.25659e-06
XLOC_1167	sp|Q6AZB8|HARB1_DANRE Putative nuclease HARBI1 OS=Danio rerio GN=harbi1 PE=2 SV=1//4.03962e-29
XLOC_1170	sp|Q64FQ2|PID2_ARATH Protein kinase PINOID 2 OS=Arabidopsis thaliana GN=PID2 PE=1 SV=1//2.07607e-29
XLOC_1171	sp|Q940T9|COL4_ARATH Zinc finger protein CONSTANS-LIKE 4 OS=Arabidopsis thaliana GN=COL4 PE=2 SV=2//4.11251e-06
XLOC_1172	sp|Q93Y94|RPOT1_NICSY DNA-directed RNA polymerase 1, mitochondrial OS=Nicotiana sylvestris GN=RPOT1 PE=2 SV=1//3.1620
XLOC_1174	-//-

XLOC_1178 sp|P48011|RPAB4_SCHPO DNA-directed RNA polymerases I, II, and III subunit RPABC4 OS=Schizosaccharomyces pombe (strain
XLOC_1180 sp|P50346|RLA0_SOYBN 60S acidic ribosomal protein P0 OS=Glycine max PE=2 SV=1//0
XLOC_1180 sp|P0C8Q9|Y5902_ARATH Uncharacterized protein At5g19025 OS=Arabidopsis thaliana GN=At5g19025 PE=2 SV=3//6.92735e-19
XLOC_1181 sp|Q9LPK5|CML44_ARATH Probable calcium-binding protein CML44 OS=Arabidopsis thaliana GN=CML44 PE=2 SV=2//2.82035e-31
XLOC_1186 sp|Q9SZL8|FRS5_ARATH Protein FAR1-RELATED SEQUENCE 5 OS=Arabidopsis thaliana GN=FRS5 PE=2 SV=1//4.12966e-19
XLOC_1189 -//-
XLOC_1190 sp|Q09020|PR4_PHAVU Wound-induced basic protein OS=Phaseolus vulgaris GN=PR4 PE=2 SV=1//1.31989e-23
XLOC_1191 sp|O82662|SUCB_ARATH Succinyl-CoA ligase [ADP-forming] subunit beta, mitochondrial OS=Arabidopsis thaliana GN=At2g204
XLOC_1194 sp|P52855|RLA1_MAIZE 60S acidic ribosomal protein P1 OS=Zea mays GN=RPP1A PE=1 SV=1//4.73096e-20
XLOC_1194 -//-
XLOC_1196 -//-
XLOC_1196 sp|Q500U8|TKPR1_ARATH Tetraketide alpha-pyrone reductase 1 OS=Arabidopsis thaliana GN=TKPR1 PE=2 SV=1//4.57126e-48
XLOC_1196 -//-
XLOC_1199 -//-
XLOC_1200 sp|P10978|POLX_TOBAC Retrovirus-related Pol polyprotein from transposon TNT 1-94 OS=Nicotiana tabacum PE=2 SV=1//4.16
XLOC_1207 sp|Q5A3M6|MCD4_CANAL GPI ethanolamine phosphate transferase 1 OS=Candida albicans (strain SC5314 / ATCC MYA-2876) GN=
XLOC_1208 sp|Q8N2Z9|CENPS_HUMAN Centromere protein S OS=Homo sapiens GN=APITD1 PE=1 SV=1//6.39422e-07
XLOC_1209 -//-
XLOC_1209 -//-
XLOC_1209 sp|Q9C8Z4|HEAT1_ARATH Uncharacterized protein At3g06530 OS=Arabidopsis thaliana GN=At3g06530 PE=2 SV=3//7.80349e-81
XLOC_1209 sp|Q9C8Z4|HEAT1_ARATH Uncharacterized protein At3g06530 OS=Arabidopsis thaliana GN=At3g06530 PE=2 SV=3//3.08013e-48
XLOC_1209 sp|Q91WD1|RPC4_MOUSE DNA-directed RNA polymerase III subunit RPC4 OS=Mus musculus GN=Polr3d PE=2 SV=2//2.36469e-08
XLOC_1210 sp|Q9LUJ5|EBP2_ARATH Probable rRNA-processing protein EBP2 homolog OS=Arabidopsis thaliana GN=At3g22660 PE=2 SV=1//8.
XLOC_1210 sp|Q9SWW6|CESA7_ARATH Cellulose synthase A catalytic subunit 7 [UDP-forming] OS=Arabidopsis thaliana GN=CESA7 PE=1 SV
XLOC_1211 sp|Q8GW46|YAB5_ARATH Axial regulator YABBY 5 OS=Arabidopsis thaliana GN=YAB5 PE=2 SV=1//3.06374e-50
XLOC_1211 sp|Q940T9|COL4_ARATH Zinc finger protein CONSTANS-LIKE 4 OS=Arabidopsis thaliana GN=COL4 PE=2 SV=2//1.50535e-92
XLOC_1212 -//-
XLOC_1212 -//-
XLOC_1212 sp|P14009|14KD_DAUCA 14 kDa proline-rich protein DC2.15 OS=Daucus carota PE=2 SV=1//2.12388e-28
XLOC_1212 -//-
XLOC_1213 -//-
XLOC_1213 sp|Q13231|CHIT1_HUMAN Chitotriosidase-1 OS=Homo sapiens GN=CHIT1 PE=1 SV=1//4.5683e-16
XLOC_1213 sp|Q8VZ59|YUC6_ARATH Flavin-containing monooxygenase YUCCA6 OS=Arabidopsis thaliana GN=YUC6 PE=2 SV=1//1.93566e-10
XLOC_1213 sp|Q8LEM8|RL373_ARATH 60S ribosomal protein L37-3 OS=Arabidopsis thaliana GN=RPL37C PE=3 SV=1//5.43463e-44
XLOC_1213 sp|Q9S7G7|MB3R1_ARATH Myb-related protein 3R-1 OS=Arabidopsis thaliana GN=MYB3R-1 PE=2 SV=1//1.24467e-87

XLOC_1213 -//-
XLOC_1213 -//-
XLOC_1213 sp|Q5SRX1|TM1L2_MOUSE TOM1-like protein 2 OS=Mus musculus GN=Tom1l2 PE=1 SV=1//1.96532e-10
XLOC_1213 sp|Q8LGF7|PEX4_ARATH Protein PEROXIN-4 OS=Arabidopsis thaliana GN=PEX4 PE=1 SV=1//8.64546e-40
XLOC_1213 -//-
XLOC_1213 sp|P09523|POL_FMVD Enzymatic polyprotein OS=Figwort mosaic virus (strain DxS) GN=ORF V PE=3 SV=1//3.03325e-132
XLOC_1213 sp|Q07469|BSPA_POPDE Bark storage protein A OS=Populus deltoides GN=BSPA PE=2 SV=1//5.15866e-27
XLOC_1214 sp|Q9ZU11|ATB15_ARATH Homeobox-leucine zipper protein ATHB-15 OS=Arabidopsis thaliana GN=ATHB-15 PE=1 SV=1//1.99343e-
XLOC_1214 sp|Q9ZU11|ATB15_ARATH Homeobox-leucine zipper protein ATHB-15 OS=Arabidopsis thaliana GN=ATHB-15 PE=1 SV=1//6.44771e-
XLOC_1214 sp|D7STK2|GATC_VITVI Glutamyl-tRNA(Gln) amidotransferase subunit C, chloroplastic/mitochondrial OS=Vitis vinifera GN=
XLOC_1214 sp|O23969|SF21_HELAN Pollen-specific protein SF21 OS=Helianthus annuus GN=SF21 PE=2 SV=1//2.1777e-10
XLOC_1214 sp|O80983|FTSH4_ARATH ATP-dependent zinc metalloprotease FTSH 4, mitochondrial OS=Arabidopsis thaliana GN=FTSH4 PE=1
XLOC_1215 sp|Q9SXJ6|CLPP3_ARATH ATP-dependent Clp protease proteolytic subunit 3, chloroplastic OS=Arabidopsis thaliana GN=CLPF
XLOC_1222 sp|O32472|PHAJ_AERPU (R)-specific enoyl-CoA hydratase OS=Aeromonas punctata GN=phaJ PE=1 SV=1//7.59853e-22
XLOC_1224 -//-
XLOC_1225 -//-
XLOC_1225 -//-
XLOC_1226 sp|Q8GU89|PDR4_ORYSJ Pleiotropic drug resistance protein 4 OS=Oryza sativa subsp. japonica GN=PDR4 PE=2 SV=1//1.03298
XLOC_1226 sp|P24922|IF5A2_NICPL Eukaryotic translation initiation factor 5A-2 OS=Nicotiana plumbaginifolia GN=EIF-5A2 PE=2 SV=1
XLOC_1229 sp|Q40353|MMK2_MEDSA Mitogen-activated protein kinase homolog MMK2 OS=Medicago sativa GN=MMK2 PE=2 SV=1//5.27339e-14
XLOC_1231 sp|P10978|POLX_TOBAC Retrovirus-related Pol polyprotein from transposon TNT 1-94 OS=Nicotiana tabacum PE=2 SV=1//2.07
XLOC_1231 -//-
XLOC_1233 sp|Q9SRR7|CML3_ARATH Calmodulin-like protein 3 OS=Arabidopsis thaliana GN=CML3 PE=2 SV=1//3.50755e-20
XLOC_1237 -//-
XLOC_1237 -//-
XLOC_1238 -//-
XLOC_1239 sp|Q811Q9|PCY1B_MOUSE Choline-phosphate cytidylyltransferase B OS=Mus musculus GN=Pcyt1b PE=1 SV=2//3.70586e-67
XLOC_1239 -//-
XLOC_1240 -//-
XLOC_1242 sp|Q9LRR4|R13L1_ARATH Putative disease resistance RPP13-like protein 1 OS=Arabidopsis thaliana GN=RPPL1 PE=2 SV=1//2.
XLOC_1247 sp|P10978|POLX_TOBAC Retrovirus-related Pol polyprotein from transposon TNT 1-94 OS=Nicotiana tabacum PE=2 SV=1//1.52
XLOC_1249 -//-
XLOC_1253 sp|Q9SRT3|CSLC6_ARATH Probable xyloglucan glycosyltransferase 6 OS=Arabidopsis thaliana GN=CSLC6 PE=1 SV=1//2.58924e-
XLOC_1254 sp|Q9SZL8|FRS5_ARATH Protein FAR1-RELATED SEQUENCE 5 OS=Arabidopsis thaliana GN=FRS5 PE=2 SV=1//9.04738e-28
XLOC_1254 sp|P55034|PSMD4_ARATH 26S proteasome non-ATPase regulatory subunit 4 OS=Arabidopsis thaliana GN=MBP1 PE=1 SV=1//4.931

XLOC_1261	sp|P93295|M310_ARATH Uncharacterized mitochondrial protein AtMg00310 OS=Arabidopsis thaliana GN=AtMg00310 PE=4 SV=1//
XLOC_1263	sp|Q9H0Z9|RBM38_HUMAN RNA-binding protein 38 OS=Homo sapiens GN=RBM38 PE=1 SV=2//1.71871e-30
XLOC_1264	sp|Q8LAC4|URH2_ARATH Probable uridine nucleosidase 2 OS=Arabidopsis thaliana GN=URH2 PE=2 SV=1//9.14411e-11
XLOC_1269	sp|P0C2F6|RNHX1_ARATH Putative ribonuclease H protein At1g65750 OS=Arabidopsis thaliana GN=At1g65750 PE=3 SV=1//2.983
XLOC_1270	sp|Q9ZPI6|AIM1_ARATH Peroxisomal fatty acid beta-oxidation multifunctional protein AIM1 OS=Arabidopsis thaliana GN=AI
XLOC_1272	sp|Q54DD7|UNC50_DICDI Protein unc-50 homolog OS=Dictyostelium discoideum GN=DDB_G0292320 PE=3 SV=1//6.63244e-07
XLOC_1277	-//-
XLOC_1277	-//-
XLOC_1277	sp|Q5WWK6|Y1447_LEGPL Uncharacterized protein lpl1447 OS=Legionella pneumophila (strain Lens) GN=lpl1447 PE=3 SV=1//6
XLOC_1277	sp|B0JQV0|PHS_MICAN Putative pterin-4-alpha-carbinolamine dehydratase OS=Microcystis aeruginosa (strain NIES-843) GN=
XLOC_1278	-//-
XLOC_1278	-//-
XLOC_1278	sp|Q40479|ERF2_TOBAC Ethylene-responsive transcription factor 2 OS=Nicotiana tabacum GN=ERF2 PE=2 SV=1//8.0506e-56
XLOC_1278	sp|P27061|PPA1_SOLLC Acid phosphatase 1 OS=Solanum lycopersicum GN=APS1 PE=2 SV=1//5.99201e-111
XLOC_1278	sp|Q07423|HEX6_RICCO Hexose carrier protein HEX6 OS=Ricinus communis GN=HEX6 PE=2 SV=1//9.65919e-174
XLOC_1278	sp|Q9C5Y4|SMC21_ARATH Structural maintenance of chromosomes protein 2-1 OS=Arabidopsis thaliana GN=SMC2-1 PE=2 SV=2//
XLOC_1279	-//-
XLOC_1279	sp|Q9FHM4|MA653_ARATH 65-kDa microtubule-associated protein 3 OS=Arabidopsis thaliana GN=MAP65-3 PE=1 SV=1//1.3995e-3
XLOC_1279	sp|Q9LI74|CHUP1_ARATH Protein CHUP1, chloroplastic OS=Arabidopsis thaliana GN=CHUP1 PE=1 SV=1//1.01818e-11
XLOC_1279	sp|O04716|MSH6_ARATH DNA mismatch repair protein MSH6 OS=Arabidopsis thaliana GN=MSH6 PE=1 SV=2//1.89085e-06
XLOC_1279	sp|Q9SKK0|EBF1_ARATH EIN3-binding F-box protein 1 OS=Arabidopsis thaliana GN=EBF1 PE=1 SV=1//0
XLOC_1279	sp|Q9SZY4|PTR27_ARATH Nitrate transporter 1.4 OS=Arabidopsis thaliana GN=NRT1.4 PE=1 SV=1//0
XLOC_1280	sp|Q9C5V5|GLR28_ARATH Glutamate receptor 2.8 OS=Arabidopsis thaliana GN=GLR2.8 PE=2 SV=2//0
XLOC_1280	sp|O81772|PER46_ARATH Peroxidase 46 OS=Arabidopsis thaliana GN=PER46 PE=2 SV=1//5.24362e-149
XLOC_1280	sp|O81770|MGDG1_ARATH Monogalactosyldiacylglycerol synthase 1, chloroplastic OS=Arabidopsis thaliana GN=MGD1 PE=1 SV=
XLOC_1280	sp|Q9SK27|ENL1_ARATH Early nodulin-like protein 1 OS=Arabidopsis thaliana GN=At2g25060 PE=1 SV=2//7.78771e-54
XLOC_1280	sp|Q9LK40|RPD2A_ARATH DNA-directed RNA polymerase D subunit 2a OS=Arabidopsis thaliana GN=NRPD2a PE=1 SV=1//0
XLOC_1280	sp|Q9C5T4|WRK18_ARATH WRKY transcription factor 18 OS=Arabidopsis thaliana GN=WRKY18 PE=1 SV=2//5.73601e-35
XLOC_1280	sp|Q9C5T4|WRK18_ARATH WRKY transcription factor 18 OS=Arabidopsis thaliana GN=WRKY18 PE=1 SV=2//2.53661e-75
XLOC_1280	sp|Q04264|PDS5_YEAST Sister chromatid cohesion protein PDS5 OS=Saccharomyces cerevisiae (strain ATCC 204508 / S288c)
XLOC_1281	sp|Q8VY97|Y4213_ARATH ER membrane protein complex subunit 7 homolog OS=Arabidopsis thaliana GN=At4g32130 PE=1 SV=1//1
XLOC_1281	sp|P86387|PLDA1_CARPA Phospholipase D alpha 1 OS=Carica papaya GN=PLD1 PE=1 SV=1//6.33975e-163
XLOC_1281	sp|P81406|GAPN_PEA NADP-dependent glyceraldehyde-3-phosphate dehydrogenase OS=Pisum sativum GN=GAPN PE=1 SV=1//0
XLOC_1282	sp|P37833|AATC_ORYSJ Aspartate aminotransferase, cytoplasmic OS=Oryza sativa subsp. japonica GN=Os01g0760600 PE=2 SV=
XLOC_1282	sp|Q5RCZ7|RCBT2_PONAB RCC1 and BTB domain-containing protein 2 OS=Pongo abelii GN=RCBTB2 PE=2 SV=1//1.49542e-06

XLOC_1282 sp|Q54J73|PAP_DICDI Poly(A) polymerase OS=Dictyostelium discoideum GN=papA PE=3 SV=1//9.32881e-47
XLOC_1282 sp|Q02921|NO93_SOYBN Early nodulin-93 OS=Glycine max PE=2 SV=1//1.87478e-10
XLOC_1282 sp|Q9M077|AUR1_ARATH Serine/threonine-protein kinase Aurora-1 OS=Arabidopsis thaliana GN=AUR1 PE=1 SV=1//2.524e-58
XLOC_1282 sp|Q941R6|MLP31_ARATH MLP-like protein 31 OS=Arabidopsis thaliana GN=MLP31 PE=1 SV=2//5.08778e-27
XLOC_1282 sp|O82804|ELF3_ARATH Protein EARLY FLOWERING 3 OS=Arabidopsis thaliana GN=ELF3 PE=1 SV=1//1.78897e-43
XLOC_1283 -//-
XLOC_1283 sp|P49043|VPE_CITSI Vacuolar-processing enzyme OS=Citrus sinensis PE=2 SV=1//0
XLOC_1283 sp|Q9FPR3|EDR1_ARATH Serine/threonine-protein kinase EDR1 OS=Arabidopsis thaliana GN=EDR1 PE=1 SV=1//4.4982e-46
XLOC_1283 sp|P55786|PSA_HUMAN Puromycin-sensitive aminopeptidase OS=Homo sapiens GN=NPEPPS PE=1 SV=2//2.8377e-41
XLOC_1283 sp|Q8VWQ5|WRK50_ARATH Probable WRKY transcription factor 50 OS=Arabidopsis thaliana GN=WRKY50 PE=2 SV=1//5.44294e-36
XLOC_1283 -//-
XLOC_1283 -//-
XLOC_1284 -//-
XLOC_1284 sp|P93147|C81E1_GLYEC Isoflavone 2'-hydroxylase OS=Glycyrrhiza echinata GN=CYP81E1 PE=1 SV=2//1.48556e-39
XLOC_1286 sp|Q84JU4|IBR5_ARATH Protein-tyrosine-phosphatase IBR5 OS=Arabidopsis thaliana GN=IBR5 PE=1 SV=1//7.81282e-57
XLOC_1287 -//-
XLOC_1287 -//-
XLOC_1287 sp|P08770|TRA1_MAIZE Putative AC transposase OS=Zea mays PE=2 SV=2//4.19813e-82
XLOC_1292 sp|Q8LEM8|RL373_ARATH 60S ribosomal protein L37-3 OS=Arabidopsis thaliana GN=RPL37C PE=3 SV=1//3.60387e-28
XLOC_1292 -//-
XLOC_1292 -//-
XLOC_1293 sp|Q9SB51|UBP16_ARATH Ubiquitin carboxyl-terminal hydrolase 16 OS=Arabidopsis thaliana GN=UBP16 PE=2 SV=1//2.15488e-2
XLOC_1294 sp|Q9LRR4|R13L1_ARATH Putative disease resistance RPP13-like protein 1 OS=Arabidopsis thaliana GN=RPPL1 PE=2 SV=1//4.
XLOC_1294 -//-
XLOC_1295 sp|P52855|RLA1_MAIZE 60S acidic ribosomal protein P1 OS=Zea mays GN=RPP1A PE=1 SV=1//1.33273e-20
XLOC_1295 sp|Q39639|PLSB_CUCSA Glycerol-3-phosphate acyltransferase, chloroplastic OS=Cucumis sativus PE=2 SV=1//6.08749e-22
XLOC_1298 sp|Q9M2E2|SDR1_ARATH (+)-neomenthol dehydrogenase OS=Arabidopsis thaliana GN=SDR1 PE=1 SV=1//2.34409e-97
XLOC_1300 -//-
XLOC_1301 sp|Q8H2T0|P2C65_ORYSJ Probable protein phosphatase 2C 65 OS=Oryza sativa subsp. japonica GN=Os07g0646100 PE=2 SV=1//2
XLOC_1301 -//-
XLOC_1302 sp|Q9SZL8|FRS5_ARATH Protein FAR1-RELATED SEQUENCE 5 OS=Arabidopsis thaliana GN=FRS5 PE=2 SV=1//2.23987e-30
XLOC_1302 sp|Q09020|PR4_PHAVU Wound-induced basic protein OS=Phaseolus vulgaris GN=PR4 PE=2 SV=1//1.36373e-23
XLOC_1307 -//-
XLOC_1307 sp|Q96361|ARF1_BRARP ADP-ribosylation factor 1 OS=Brassica rapa subsp. pekinensis GN=ARF1 PE=2 SV=3//1.03474e-21
XLOC_1308 sp|P0C2F6|RNHX1_ARATH Putative ribonuclease H protein At1g65750 OS=Arabidopsis thaliana GN=At1g65750 PE=3 SV=1//5.186

XLOC_1308 sp|C0LGQ5|GSO1_ARATH LRR receptor-like serine/threonine-protein kinase GSO1 OS=Arabidopsis thaliana GN=GSO1 PE=2 SV=1
XLOC_1310 sp|Q96MD7|CI085_HUMAN Uncharacterized protein C9orf85 OS=Homo sapiens GN=C9orf85 PE=1 SV=1//1.23472e-18
XLOC_1311 sp|Q9SI37|WRKY1_ARATH WRKY transcription factor 1 OS=Arabidopsis thaliana GN=WRKY1 PE=1 SV=1//3.49884e-85
XLOC_1312 sp|P10978|POLX_TOBAC Retrovirus-related Pol polyprotein from transposon TNT 1-94 OS=Nicotiana tabacum PE=2 SV=1//2.83
XLOC_1317 sp|Q9FPT1|UBP12_ARATH Ubiquitin carboxyl-terminal hydrolase 12 OS=Arabidopsis thaliana GN=UBP12 PE=1 SV=2//3.43026e-1
XLOC_1319 -//-
XLOC_1321 sp|Q9LN63|BZR2_ARATH Protein BRASSINAZOLE-RESISTANT 2 OS=Arabidopsis thaliana GN=BZR2 PE=1 SV=1//3.76574e-24
XLOC_1323 -//-
XLOC_1324 sp|P10978|POLX_TOBAC Retrovirus-related Pol polyprotein from transposon TNT 1-94 OS=Nicotiana tabacum PE=2 SV=1//2.53
XLOC_1324 -//-
XLOC_1328 sp|P03010|TRAC9_MAIZE Putative AC9 transposase OS=Zea mays PE=4 SV=1//2.51705e-29
XLOC_1329 sp|O49668|PAM68_ARATH Protein PAM68, chloroplastic OS=Arabidopsis thaliana GN=PAM68 PE=1 SV=1//7.61798e-46
XLOC_1329 sp|P44731|POTD2_HAEIN Spermidine/putrescine-binding periplasmic protein 2 OS=Haemophilus influenzae (strain ATCC 5190
XLOC_1330 sp|Q9FNA2|PAO1_ARATH Polyamine oxidase 1 OS=Arabidopsis thaliana GN=PAO1 PE=1 SV=1//2.40617e-102
XLOC_1331 sp|P10978|POLX_TOBAC Retrovirus-related Pol polyprotein from transposon TNT 1-94 OS=Nicotiana tabacum PE=2 SV=1//1.27
XLOC_1331 sp|P04323|POL3_DROME Retrovirus-related Pol polyprotein from transposon 17.6 OS=Drosophila melanogaster GN=pol PE=4 S
XLOC_1331 sp|Q5A3M6|MCD4_CANAL GPI ethanolamine phosphate transferase 1 OS=Candida albicans (strain SC5314 / ATCC MYA-2876) GN=
XLOC_1331 sp|O04369|RAC1_LOTJA Rac-like GTP-binding protein RAC1 OS=Lotus japonicus GN=RAC1 PE=2 SV=1//1.08104e-30
XLOC_1332 -//-
XLOC_1335 -//-
XLOC_1337 -//-
XLOC_1337 sp|Q945L0|CX6B2_ARATH Cytochrome c oxidase subunit 6b-2 OS=Arabidopsis thaliana GN=COX6B-2 PE=2 SV=1//1.20268e-22
XLOC_1339 sp|P10978|POLX_TOBAC Retrovirus-related Pol polyprotein from transposon TNT 1-94 OS=Nicotiana tabacum PE=2 SV=1//8.06
XLOC_1340 sp|Q6NQK2|NAC8_ARATH NAC domain-containing protein 8 OS=Arabidopsis thaliana GN=NAC008 PE=2 SV=1//5.26528e-66
XLOC_1340 -//-
XLOC_1340 sp|Q9CXS4|CENPV_MOUSE Centromere protein V OS=Mus musculus GN=Cenpv PE=2 SV=2//7.12256e-13
XLOC_1341 sp|Q54LT8|STRAP_DICDI Serine-threonine kinase receptor-associated protein OS=Dictyostelium discoideum GN=strap PE=3 S
XLOC_1342 sp|P20825|POL2_DROME Retrovirus-related Pol polyprotein from transposon 297 OS=Drosophila melanogaster GN=pol PE=4 SV
XLOC_1342 sp|O02414|DYL1_HELCR Dynein light chain LC6, flagellar outer arm OS=Heliocidaris crassispina PE=3 SV=1//1.09631e-23
XLOC_1343 sp|Q8GW61|STP14_ARATH Sugar transport protein 14 OS=Arabidopsis thaliana GN=STP14 PE=2 SV=2//4.28381e-66
XLOC_1343 -//-
XLOC_1344 sp|P08770|TRA1_MAIZE Putative AC transposase OS=Zea mays PE=2 SV=2//4.38957e-75
XLOC_1347 sp|Q5A3M6|MCD4_CANAL GPI ethanolamine phosphate transferase 1 OS=Candida albicans (strain SC5314 / ATCC MYA-2876) GN=
XLOC_1348 sp|P08770|TRA1_MAIZE Putative AC transposase OS=Zea mays PE=2 SV=2//4.64649e-72
XLOC_1350 sp|Q8W593|LGUC_ARATH Probable lactoylglutathione lyase, chloroplast OS=Arabidopsis thaliana GN=At1g67280 PE=2 SV=1//2

XLOC_135(-//-
XLOC_1351 sp|C0LGK9|Y2242_ARATH Probable LRR receptor-like serine/threonine-protein kinase At2g24230 OS=Arabidopsis thaliana GN
XLOC_1352 sp|Q5RDY2|TM9S4_PONAB Transmembrane 9 superfamily member 4 OS=Pongo abelii GN=TM9SF4 PE=2 SV=1//4.4171e-85
XLOC_1352 sp|O82232|RC22_ARATH UPF0057 membrane protein At2g24040 OS=Arabidopsis thaliana GN=At2g24040 PE=2 SV=1//7.41999e-23
XLOC_1352 sp|Q9FG13|CXE15_ARATH Probable carboxylesterase 15 OS=Arabidopsis thaliana GN=CXE15 PE=2 SV=1//1.19167e-10
XLOC_1354 -//-
XLOC_1354 sp|P08770|TRA1_MAIZE Putative AC transposase OS=Zea mays PE=2 SV=2//7.22828e-15
XLOC_1356 sp|F4HVJ3|POD1_ARATH Protein POLLEN DEFECTIVE IN GUIDANCE 1 OS=Arabidopsis thaliana GN=POD1 PE=1 SV=1//2.35927e-39
XLOC_1357 sp|Q42438|CDPK8_ARATH Calcium-dependent protein kinase 8 OS=Arabidopsis thaliana GN=CPK8 PE=1 SV=1//4.42455e-41
XLOC_1362 sp|P0CG83|UBIQP_HORVU Polyubiquitin (Fragment) OS=Hordeum vulgare PE=2 SV=1//1.54246e-45
XLOC_1371 sp|Q9FFT9|RH32_ARATH DEAD-box ATP-dependent RNA helicase 32 OS=Arabidopsis thaliana GN=RH32 PE=2 SV=1//4.12967e-07
XLOC_1373 sp|P08770|TRA1_MAIZE Putative AC transposase OS=Zea mays PE=2 SV=2//5.16443e-41
XLOC_1374 -//-
XLOC_1375 sp|Q9FLF7|MYST1_ARATH Probable MYST-like histone acetyltransferase 1 OS=Arabidopsis thaliana GN=HAG4 PE=2 SV=1//2.304
XLOC_1376 -//-
XLOC_1378 -//-
XLOC_1379 sp|Q9LT17|BBR_ARATH E3 ubiquitin ligase BIG BROTHER-related OS=Arabidopsis thaliana GN=BBR PE=2 SV=1//4.63406e-77
XLOC_1381 sp|O82531|PSB1_PETHY Proteasome subunit beta type-1 OS=Petunia hybrida GN=PBF1 PE=2 SV=1//2.76388e-06
XLOC_1382 -//-
XLOC_1383 -//-
XLOC_1384 sp|Q8H156|RAN3_ARATH GTP-binding nuclear protein Ran-3 OS=Arabidopsis thaliana GN=RAN3 PE=1 SV=2//6.44554e-148
XLOC_1386 sp|P58854|GCP3_MOUSE Gamma-tubulin complex component 3 OS=Mus musculus GN=Tubgcp3 PE=2 SV=2//5.02791e-09
XLOC_1386 sp|Q54YQ9|MVD1_DICDI Diphosphomevalonate decarboxylase OS=Dictyostelium discoideum GN=mvd PE=3 SV=1//4.1998e-16
XLOC_1387 -//-
XLOC_1387 sp|P08927|RUBB_PEA RuBisCO large subunit-binding protein subunit beta, chloroplastic OS=Pisum sativum PE=1 SV=2//2.28
XLOC_1387 sp|P08770|TRA1_MAIZE Putative AC transposase OS=Zea mays PE=2 SV=2//3.36544e-76
XLOC_139(sp|Q09912|PSI1_SCHPO Protein psi1 OS=Schizosaccharomyces pombe (strain 972 / ATCC 24843) GN=psi1 PE=2 SV=2//7.07087e-
XLOC_139(-//-
XLOC_139(-//-
XLOC_139(-//-
XLOC_1391 sp|Q95ZI6|PITH1_CAEEL PITH domain-containing protein ZK353.9 OS=Caenorhabditis elegans GN=ZK353.9 PE=2 SV=1//2.28666e
XLOC_1391 sp|C0LGX3|HSL2_ARATH LRR receptor-like serine/threonine-protein kinase HSL2 OS=Arabidopsis thaliana GN=HSL2 PE=2 SV=1
XLOC_1391 sp|Q9LU15|AHP4_ARATH Histidine-containing phosphotransfer protein 4 OS=Arabidopsis thaliana GN=AHP4 PE=1 SV=2//4.748(
XLOC_1391 sp|Q8YXE4|SYW_NOSS1 Tryptophan--tRNA ligase OS=Nostoc sp. (strain PCC 7120 / UTEX 2576) GN=trpS PE=3 SV=1//4.07404e-2
XLOC_1391 sp|P51615|MAOX_VITVI NADP-dependent malic enzyme OS=Vitis vinifera PE=2 SV=1//1.95313e-40

XLOC_1392 sp|Q9ZU11|ATB15_ARATH Homeobox-leucine zipper protein ATHB-15 OS=Arabidopsis thaliana GN=ATHB-15 PE=1 SV=1//5.69755e-
XLOC_1392 sp|Q8L493|BCAL3_ARATH Branched-chain-amino-acid aminotransferase-like protein 3, chloroplastic OS=Arabidopsis thalian
XLOC_1392 sp|Q940P5|TET19_ARATH Tetraspanin-19 OS=Arabidopsis thaliana GN=At2g20740 PE=2 SV=1//3.19819e-07
XLOC_1393 sp|Q0UYV9|YD156_PHANO WD repeat-containing protein SNOG_03055 OS=Phaeosphaeria nodorum (strain SN15 / ATCC MYA-4574 /
XLOC_1393 -//-
XLOC_1393 sp|Q56X72|RTNLS_ARATH Reticulon-like protein B21 OS=Arabidopsis thaliana GN=RTNLB21 PE=2 SV=2//2.75181e-105
XLOC_1393 sp|Q7VG78|GUAA_HELHP Probable GMP synthase [glutamine-hydrolyzing] OS=Helicobacter hepaticus (strain ATCC 51449 / 3B1
XLOC_1393 sp|Q9DBG7|SRPR_MOUSE Signal recognition particle receptor subunit alpha OS=Mus musculus GN=Srpr PE=1 SV=1//4.19103e-3
XLOC_1394 sp|Q84ZT9|WVD2_ARATH Protein WAVE-DAMPENED 2 OS=Arabidopsis thaliana GN=WVD2 PE=2 SV=1//1.0072e-21
XLOC_1394 sp|Q8GY91|APX6_ARATH Putative L-ascorbate peroxidase 6 OS=Arabidopsis thaliana GN=APX6 PE=2 SV=1//1.60498e-36
XLOC_1395 sp|P43291|SRK2A_ARATH Serine/threonine-protein kinase SRK2A OS=Arabidopsis thaliana GN=SRK2A PE=1 SV=1//3.80174e-20
XLOC_1400 -//-
XLOC_1400 -//-
XLOC_1401 sp|Q9LNG5|PPP7L_ARATH Serine/threonine-protein phosphatase 7 long form homolog OS=Arabidopsis thaliana GN=At1g48120 P
XLOC_1403 sp|Q6UNT2|RL5_CUCSA 60S ribosomal protein L5 OS=Cucumis sativus GN=RPL5 PE=2 SV=1//1.10394e-14
XLOC_1404 sp|Q2L952|NU1C_GOSHI NAD(P)H-quinone oxidoreductase subunit 1, chloroplastic OS=Gossypium hirsutum GN=ndhA PE=3 SV=1/
XLOC_1408 sp|Q4V3C1|MED8_ARATH Mediator of RNA polymerase II transcription subunit 8 OS=Arabidopsis thaliana GN=MED8 PE=1 SV=1/
XLOC_1410 sp|A0T0I9|RK14_PHATC 50S ribosomal protein L14, chloroplastic OS=Phaeodactylum tricornutum (strain CCAP 1055/1) GN=rp
XLOC_1411 sp|Q43128|PMA10_ARATH ATPase 10, plasma membrane-type OS=Arabidopsis thaliana GN=AHA10 PE=2 SV=2//5.06166e-17
XLOC_1411 sp|Q9ATF5|RL18A_CASSA 60S ribosomal protein L18a OS=Castanea sativa GN=RPL18A PE=2 SV=1//5.79939e-20
XLOC_1412 sp|P08770|TRA1_MAIZE Putative AC transposase OS=Zea mays PE=2 SV=2//2.21045e-56
XLOC_1415 sp|Q53FA7|QORX_HUMAN Quinone oxidoreductase PIG3 OS=Homo sapiens GN=TP53I3 PE=1 SV=2//3.9395e-18
XLOC_1416 sp|P0C8Z0|Y8359_ORYSI Uncharacterized protein OsI_027940 OS=Oryza sativa subsp. indica GN=OsI_027940 PE=1 SV=2//1.163
XLOC_1418 sp|Q9SSQ4|FRS6_ARATH Protein FAR1-RELATED SEQUENCE 6 OS=Arabidopsis thaliana GN=FRS6 PE=2 SV=1//0
XLOC_1421 sp|Q9LK94|MDAR2_ARATH Probable monodehydroascorbate reductase, cytoplasmic isoform 2 OS=Arabidopsis thaliana GN=At3g2
XLOC_1423 -//-
XLOC_1424 -//-
XLOC_1427 -//-
XLOC_1429 sp|Q9Y305|ACOT9_HUMAN Acyl-coenzyme A thioesterase 9, mitochondrial OS=Homo sapiens GN=ACOT9 PE=1 SV=2//6.12366e-32
XLOC_1429 -//-
XLOC_1435 -//-
XLOC_1436 sp|Q9SR02|MED14_ARATH Mediator of RNA polymerase II transcription subunit 14 OS=Arabidopsis thaliana GN=MED14 PE=1 SV
XLOC_1438 -//-
XLOC_1439 sp|Q9SKZ5|FBT1_ARATH Folate-biopterin transporter 1, chloroplastic OS=Arabidopsis thaliana GN=At2g32040 PE=2 SV=2//9.
XLOC_1439 sp|Q3E9C3|RH58_ARATH DEAD-box ATP-dependent RNA helicase 58, chloroplastic OS=Arabidopsis thaliana GN=RH58 PE=2 SV=1/

XLOC_1439-//-
XLOC_1441-//-
XLOC_1443sp|Q67XQ0|Y4424_ARATH DUF21 domain-containing protein At4g14240 OS=Arabidopsis thaliana GN=CBSDUF1 PE=2 SV=1//3.07096
XLOC_1444-//-
XLOC_1444sp|P34091|RL6_MESCR 60S ribosomal protein L6 OS=Mesembryanthemum crystallinum GN=RPL6 PE=2 SV=1//1.67152e-12
XLOC_1444sp|Q42910|PPDK_MESCR Pyruvate, phosphate dikinase, chloroplastic OS=Mesembryanthemum crystallinum GN=PPD PE=2 SV=1//2
XLOC_1445sp|Q94BZ5|HSFA5_ARATH Heat stress transcription factor A-5 OS=Arabidopsis thaliana GN=HSFA5 PE=2 SV=1//1.09597e-13
XLOC_1446sp|P10978|POLX_TOBAC Retrovirus-related Pol polyprotein from transposon TNT 1-94 OS=Nicotiana tabacum PE=2 SV=1//1.35
XLOC_1447sp|Q9M612|NACA_PINTA Nascent polypeptide-associated complex subunit alpha-like protein OS=Pinus taeda PE=2 SV=1//1.08
XLOC_1448sp|Q9SUJ1|SYP43_ARATH Syntaxin-43 OS=Arabidopsis thaliana GN=SYP43 PE=2 SV=2//3.07905e-21
XLOC_1451sp|Q80Z37|TOPRS_MOUSE E3 ubiquitin-protein ligase Topors OS=Mus musculus GN=Topors PE=1 SV=1//1.44114e-13
XLOC_1453sp|Q9FPR3|EDR1_ARATH Serine/threonine-protein kinase EDR1 OS=Arabidopsis thaliana GN=EDR1 PE=1 SV=1//8.89966e-19
XLOC_1453sp|Q07123|AMO2_ARTS1 Copper methylamine oxidase OS=Arthrobacter sp. (strain P1) GN=maoII PE=1 SV=1//2.27899e-24
XLOC_1454sp|P92519|M810_ARATH Uncharacterized mitochondrial protein AtMg00810 OS=Arabidopsis thaliana GN=AtMg00810 PE=4 SV=1//
XLOC_1454sp|Q9SBJ1|PDK_ARATH [Pyruvate dehydrogenase (acetyl-transferring)] kinase, mitochondrial OS=Arabidopsis thaliana GN=P
XLOC_1456-//-
XLOC_1458sp|Q9LJH5|GLO4_ARATH Peroxisomal (S)-2-hydroxy-acid oxidase GLO4 OS=Arabidopsis thaliana GN=GLO4 PE=2 SV=1//5.87684e-
XLOC_1458sp|Q24JJ8|GLO3_ARATH Peroxisomal (S)-2-hydroxy-acid oxidase GLO3 OS=Arabidopsis thaliana GN=GLO3 PE=2 SV=1//6.24168e-
XLOC_1459sp|P62981|RS27A_SOLTU Ubiquitin-40S ribosomal protein S27a OS=Solanum tuberosum GN=UBI3 PE=2 SV=2//7.15626e-84
XLOC_1461sp|Q42551|SCE1_ARATH SUMO-conjugating enzyme SCE1 OS=Arabidopsis thaliana GN=SCE1 PE=1 SV=1//8.06287e-12
XLOC_1463-//-
XLOC_1463sp|Q8LDR0|DOF54_ARATH Dof zinc finger protein DOF5.4 OS=Arabidopsis thaliana GN=DOF5.4 PE=2 SV=2//1.47631e-28
XLOC_1467-//-
XLOC_1468-//-
XLOC_1469-//-
XLOC_1469sp|Q9LUV2|POP3_ARATH Probable protein Pop3 OS=Arabidopsis thaliana GN=At3g17210 PE=1 SV=1//2.39299e-47
XLOC_1469-//-
XLOC_1470-//-
XLOC_1472sp|P10978|POLX_TOBAC Retrovirus-related Pol polyprotein from transposon TNT 1-94 OS=Nicotiana tabacum PE=2 SV=1//4.49
XLOC_1474-//-
XLOC_1474sp|O82528|RL15_PETHY 60S ribosomal protein L15 OS=Petunia hybrida GN=RPL15 PE=2 SV=1//5.36282e-73
XLOC_1475sp|Q7XA39|RGA4_SOLBU Putative disease resistance protein RGA4 OS=Solanum bulbocastanum GN=RGA4 PE=2 SV=1//2.87317e-10
XLOC_1480-//-
XLOC_1481sp|P04323|POL3_DROME Retrovirus-related Pol polyprotein from transposon 17.6 OS=Drosophila melanogaster GN=pol PE=4 S
XLOC_1481-//-

XLOC_148[illegible] sp|Q9SCY5|KINB2_ARATH SNF1-related protein kinase regulatory subunit beta-2 OS=Arabidopsis thaliana GN=KINB2 PE=1 SV=
XLOC_148[illegible] sp|Q9SQH6|PIN6_ARATH Probable auxin efflux carrier component 6 OS=Arabidopsis thaliana GN=PIN6 PE=2 SV=2//6.11381e-42
XLOC_148[illegible] sp|Q56YJ8|FAMA_ARATH Transcription factor FAMA OS=Arabidopsis thaliana GN=FMA PE=1 SV=1//1.09377e-133
XLOC_148[illegible] sp|Q6ZGL9|SIP1_ORYSJ Signal peptide peptidase 1 OS=Oryza sativa subsp. japonica GN=SPP1 PE=2 SV=1//2.88419e-17
XLOC_148[illegible] -//-
XLOC_149[illegible] sp|Q6UNT2|RL5_CUCSA 60S ribosomal protein L5 OS=Cucumis sativus GN=RPL5 PE=2 SV=1//4.17562e-24
XLOC_1491 sp|Q8GXK5|EDL14_ARATH Sugar transporter ERD6-like 14 OS=Arabidopsis thaliana GN=At4g04750 PE=2 SV=2//5.65134e-09
XLOC_1491 sp|Q96MB7|HARB1_HUMAN Putative nuclease HARBI1 OS=Homo sapiens GN=HARBI1 PE=1 SV=1//6.98608e-18
XLOC_1491 -//-
XLOC_149[illegible] -//-
XLOC_149[illegible] -//-
XLOC_149[illegible] sp|O80366|ARR9_ARATH Two-component response regulator ARR9 OS=Arabidopsis thaliana GN=ARR9 PE=1 SV=1//1.27442e-60
XLOC_149[illegible] sp|P46286|RL81_ARATH 60S ribosomal protein L8-1 OS=Arabidopsis thaliana GN=RPL8A PE=1 SV=2//1.41406e-54
XLOC_149[illegible] -//-
XLOC_149[illegible] sp|Q9LHP4|RCH2_ARATH Receptor-like protein kinase 2 OS=Arabidopsis thaliana GN=RCH2 PE=1 SV=1//7.87871e-06
XLOC_149[illegible] -//-
XLOC_149[illegible] -//-
XLOC_149[illegible] sp|Q39688|EP1G_DAUCA Epidermis-specific secreted glycoprotein EP1 OS=Daucus carota GN=EP1 PE=1 SV=1//3.07704e-61
XLOC_150[illegible] -//-
XLOC_150[illegible] sp|Q9S746|HTH_ARATH Protein HOTHEAD OS=Arabidopsis thaliana GN=HTH PE=1 SV=1//3.37268e-45
XLOC_1511 -//-
XLOC_1511 -//-
XLOC_151[illegible] -//-
XLOC_151[illegible] -//-
XLOC_151[illegible] sp|Q93Z66|KPRS3_ARATH Ribose-phosphate pyrophosphokinase 3, chloroplastic OS=Arabidopsis thaliana GN=PRS3 PE=2 SV=1//
XLOC_151[illegible] sp|Q9LMX8|6PGL1_ARATH Probable 6-phosphogluconolactonase 1 OS=Arabidopsis thaliana GN=At1g13700 PE=2 SV=1//2.59461e-4
XLOC_151[illegible] sp|O80944|AKRC8_ARATH Aldo-keto reductase family 4 member C8 OS=Arabidopsis thaliana GN=AKR4C8 PE=1 SV=2//2.95166e-97
XLOC_151[illegible] sp|P08770|TRA1_MAIZE Putative AC transposase OS=Zea mays PE=2 SV=2//1.32241e-81
XLOC_151[illegible] sp|P68855|PSBN_WHEAT Protein PsbN OS=Triticum aestivum GN=psbN PE=3 SV=1//5.38161e-21
XLOC_151[illegible] sp|Q9SZL7|FRS9_ARATH Protein FAR1-RELATED SEQUENCE 9 OS=Arabidopsis thaliana GN=FRS9 PE=2 SV=2//0
XLOC_151[illegible] sp|Q0WNR6|ARID5_ARATH AT-rich interactive domain-containing protein 5 OS=Arabidopsis thaliana GN=ARID5 PE=2 SV=1//1.2
XLOC_1521 sp|P29685|ATPBM_HEVBR ATP synthase subunit beta, mitochondrial OS=Hevea brasiliensis GN=ATPB PE=2 SV=1//2.36324e-31
XLOC_1521 -//-
XLOC_152[illegible] sp|Q9ATF5|RL18A_CASSA 60S ribosomal protein L18a OS=Castanea sativa GN=RPL18A PE=2 SV=1//1.41211e-27
XLOC_152[illegible] sp|Q680C0|GDL62_ARATH GDSL esterase/lipase At4g10955 OS=Arabidopsis thaliana GN=At4g10955 PE=2 SV=1//4.79805e-17

XLOC_1523-//-

XLOC_1524sp|Q8L8A5|GIF1_ARATH GRF1-interacting factor 1 OS=Arabidopsis thaliana GN=GIF1 PE=1 SV=1//4.11633e-30

XLOC_1524sp|Q9M8Y5|LTL1_ARATH GDSL esterase/lipase LTL1 OS=Arabidopsis thaliana GN=LTL1 PE=2 SV=1//0

XLOC_1524sp|Q8LB81|GDL79_ARATH GDSL esterase/lipase At5g33370 OS=Arabidopsis thaliana GN=At5g33370 PE=2 SV=1//2.56232e-149

XLOC_1524sp|Q5N749|MOR1_ORYSJ Protein MOR1 OS=Oryza sativa subsp. japonica GN=MOR1 PE=2 SV=1//2.8637e-18

XLOC_1530sp|Q3B724|CALS5_ARATH Callose synthase 5 OS=Arabidopsis thaliana GN=CALS5 PE=1 SV=1//1.21313e-19

XLOC_1531sp|P17784|ALF_ORYSJ Fructose-bisphosphate aldolase cytoplasmic isozyme OS=Oryza sativa subsp. japonica GN=FBA PE=1 SV

XLOC_1533-//-

XLOC_1535sp|O26373|Y273_METTH UPF0098 protein MTH_273 OS=Methanothermobacter thermautotrophicus (strain ATCC 29096 / DSM 1053

XLOC_1535sp|Q9LM55|FBK8_ARATH F-box/kelch-repeat protein At1g22040 OS=Arabidopsis thaliana GN=At1g22040 PE=2 SV=1//0

XLOC_1536sp|Q6S591|JAG_ARATH Zinc finger protein JAGGED OS=Arabidopsis thaliana GN=JAG PE=2 SV=1//2.3384e-12

XLOC_1536sp|P10978|POLX_TOBAC Retrovirus-related Pol polyprotein from transposon TNT 1-94 OS=Nicotiana tabacum PE=2 SV=1//1.36

XLOC_1537sp|Q8L8B8|LOG3_ARATH Cytokinin riboside 5'-monophosphate phosphoribohydrolase LOG3 OS=Arabidopsis thaliana GN=LC

XLOC_1537sp|P0CH32|UBQ4_ARATH Polyubiquitin 4 OS=Arabidopsis thaliana GN=UBQ4 PE=1 SV=1//0

XLOC_1539sp|Q8MR62|VIAF1_DROME Viral IAP-associated factor homolog OS=Drosophila melanogaster GN=viaf PE=1 SV=1//4.62682e-27

XLOC_1540sp|P08770|TRA1_MAIZE Putative AC transposase OS=Zea mays PE=2 SV=2//2.29002e-07

XLOC_1544sp|P10978|POLX_TOBAC Retrovirus-related Pol polyprotein from transposon TNT 1-94 OS=Nicotiana tabacum PE=2 SV=1//7.52

XLOC_1544sp|P10978|POLX_TOBAC Retrovirus-related Pol polyprotein from transposon TNT 1-94 OS=Nicotiana tabacum PE=2 SV=1//5.82

XLOC_1544-//-

XLOC_1545-//-

XLOC_1546sp|Q2PS26|ULP1D_ARATH Ubiquitin-like-specific protease 1D OS=Arabidopsis thaliana GN=ULP1D PE=1 SV=1//1.41021e-36

XLOC_1546sp|P10978|POLX_TOBAC Retrovirus-related Pol polyprotein from transposon TNT 1-94 OS=Nicotiana tabacum PE=2 SV=1//1.27

XLOC_1546sp|P42856|ZB14_MAIZE 14 kDa zinc-binding protein OS=Zea mays GN=ZBP14 PE=1 SV=1//6.7458e-10

XLOC_1547sp|Q6NLY8|HA22K_ARATH HVA22-like protein k OS=Arabidopsis thaliana GN=HVA22K PE=2 SV=1//1.13079e-65

XLOC_1548sp|Q9LXA5|LRK91_ARATH L-type lectin-domain containing receptor kinase IX.1 OS=Arabidopsis thaliana GN=LECRK91 PE=2 SV

XLOC_1548sp|Q54UU1|TPPC4_DICDI Trafficking protein particle complex subunit 4 OS=Dictyostelium discoideum GN=trappc4 PE=3 SV=1

XLOC_1550sp|Q8H5X5|SCAM1_ORYSJ Secretory carrier-associated membrane protein 1 OS=Oryza sativa subsp. japonica GN=SCAMP1 PE=2

XLOC_1552sp|Q8S857|H2AV2_ORYSJ Probable histone H2A variant 2 OS=Oryza sativa subsp. japonica GN=Os10g0418000 PE=2 SV=1//9.494

XLOC_1552sp|O82798|ARR4_ARATH Two-component response regulator ARR4 OS=Arabidopsis thaliana GN=ARR4 PE=1 SV=1//2.14425e-67

XLOC_1553sp|Q9M391|ATX3H_ARATH Ataxin-3 homolog OS=Arabidopsis thaliana GN=At3g54130 PE=2 SV=1//7.98781e-17

XLOC_1553sp|Q9M2Z4|MSBP2_ARATH Membrane steroid-binding protein 2 OS=Arabidopsis thaliana GN=MSBP2 PE=1 SV=1//8.09486e-54

XLOC_1554sp|Q9LNG5|PPP7L_ARATH Serine/threonine-protein phosphatase 7 long form homolog OS=Arabidopsis thaliana GN=At1g48120 P

XLOC_1557sp|Q8S8M5|ABIL1_ARATH Protein ABIL1 OS=Arabidopsis thaliana GN=ABIL1 PE=1 SV=1//2.9622e-39

XLOC_1558sp|Q39433|RB1BV_BETVU Ras-related protein RAB1BV OS=Beta vulgaris GN=RAB1BV PE=2 SV=1//5.01956e-19

XLOC_1558sp|P08770|TRA1_MAIZE Putative AC transposase OS=Zea mays PE=2 SV=2//3.09541e-42

XLOC_1560 -//-

XLOC_1560 sp|P08770|TRA1_MAIZE Putative AC transposase OS=Zea mays PE=2 SV=2//8.83103e-85

XLOC_1560 -//-

XLOC_1561 sp|Q6NKX5|POT1B_ARATH Protection of telomeres protein 1b OS=Arabidopsis thaliana GN=POT1B PE=1 SV=1//3.31089e-11

XLOC_1564 sp|Q9ZVC9|FRS3_ARATH Protein FAR1-RELATED SEQUENCE 3 OS=Arabidopsis thaliana GN=FRS3 PE=2 SV=2//6.21094e-13

XLOC_1567 sp|Q9C5R8|BAS1B_ARATH 2-Cys peroxiredoxin BAS1-like, chloroplastic OS=Arabidopsis thaliana GN=At5g06290 PE=2 SV=3//1.

XLOC_1567 -//-

XLOC_1567 sp|Q9UI26|IPO11_HUMAN Importin-11 OS=Homo sapiens GN=IPO11 PE=1 SV=1//1.28859e-25

XLOC_1567 -//-

XLOC_1567 sp|Q9M6N7|AMT2_ARATH Ammonium transporter 2 OS=Arabidopsis thaliana GN=AMT2 PE=1 SV=2//0

XLOC_1569 sp|P08770|TRA1_MAIZE Putative AC transposase OS=Zea mays PE=2 SV=2//3.96992e-21

XLOC_1569 sp|A2QVV2|CREC_ASPNC Probable catabolite repression protein creC OS=Aspergillus niger (strain CBS 513.88 / FGSC A1513

XLOC_1572 sp|P10978|POLX_TOBAC Retrovirus-related Pol polyprotein from transposon TNT 1-94 OS=Nicotiana tabacum PE=2 SV=1//4.48

XLOC_1575 -//-

XLOC_1575 sp|P33079|A10A5_SOYBN Auxin-induced protein 10A5 OS=Glycine max PE=2 SV=1//1.07298e-22

XLOC_1577 sp|P0C2F6|RNHX1_ARATH Putative ribonuclease H protein At1g65750 OS=Arabidopsis thaliana GN=At1g65750 PE=3 SV=1//3.380

XLOC_1577 sp|Q5VTL8|PR38B_HUMAN Pre-mRNA-splicing factor 38B OS=Homo sapiens GN=PRPF38B PE=1 SV=1//1.27971e-12

XLOC_1579 sp|Q9SWI1|PKS1_ARATH Protein PHYTOCHROME KINASE SUBSTRATE 1 OS=Arabidopsis thaliana GN=PKS1 PE=1 SV=2//4.32349e-39

XLOC_1582 sp|Q6NUB2|CW15A_XENLA Protein CWC15 homolog A OS=Xenopus laevis GN=cwc15-a PE=2 SV=1//1.16179e-08

XLOC_1584 sp|Q8LB81|GDL79_ARATH GDSL esterase/lipase At5g33370 OS=Arabidopsis thaliana GN=At5g33370 PE=2 SV=1//0

XLOC_1584 sp|Q5S2C3|PIR_ARATH Protein PIR OS=Arabidopsis thaliana GN=PIR PE=1 SV=2//7.1092e-20

XLOC_1585 sp|P10978|POLX_TOBAC Retrovirus-related Pol polyprotein from transposon TNT 1-94 OS=Nicotiana tabacum PE=2 SV=1//2.71

XLOC_1587 sp|Q93XY5|TET18_ARATH Tetraspanin-18 OS=Arabidopsis thaliana GN=At2g20230 PE=2 SV=1//7.88789e-11

XLOC_1590 -//-

XLOC_1591 sp|Q93W34|RP45C_ARATH Polyadenylate-binding protein RBP45C OS=Arabidopsis thaliana GN=RBP45C PE=2 SV=1//1.33826e-99

XLOC_1592 sp|P25093|FAAA_RAT Fumarylacetoacetase OS=Rattus norvegicus GN=Fah PE=1 SV=1//3.25854e-12

XLOC_1593 sp|P20825|POL2_DROME Retrovirus-related Pol polyprotein from transposon 297 OS=Drosophila melanogaster GN=pol PE=4 SV

XLOC_1594 sp|P69322|UBIQP_PEA Polyubiquitin OS=Pisum sativum GN=PU1 PE=2 SV=2//0

XLOC_1594 sp|O82569|SUI1_PIMBR Protein translation factor SUI1 homolog OS=Pimpinella brachycarpa PE=3 SV=1//2.84359e-44

XLOC_1594 sp|P98183|C71DC_CATRO Tabersonine 16-hydroxylase (Fragment) OS=Catharanthus roseus GN=CYP71D12 PE=1 SV=1//4.92671e-95

XLOC_1594 -//-

XLOC_1595 sp|P10978|POLX_TOBAC Retrovirus-related Pol polyprotein from transposon TNT 1-94 OS=Nicotiana tabacum PE=2 SV=1//4.47

XLOC_1595 sp|C7G304|GOLS2_SOLLC Galactinol synthase 2 OS=Solanum lycopersicum GN=GOLS2 PE=2 SV=1//1.7656e-169

XLOC_1602 sp|P36183|ENPL_HORVU Endoplasmin homolog OS=Hordeum vulgare PE=2 SV=1//1.62954e-06

XLOC_1602 sp|P49310|GRP1_SINAL Glycine-rich RNA-binding protein GRP1A OS=Sinapis alba PE=2 SV=1//1.78465e-26

XLOC_1603 sp|Q9S9Z7|P2C10_ARATH Probable protein phosphatase 2C 10 OS=Arabidopsis thaliana GN=At1g34750 PE=1 SV=1//2.37901e-38
XLOC_1604 sp|Q6R8G7|PHO13_ARATH Phosphate transporter PHO1 homolog 3 OS=Arabidopsis thaliana GN=PHO1;H3 PE=2 SV=2//3.60529e-33
XLOC_1607 -//-
XLOC_1608 sp|Q9M0Y3|ENT3_ARATH Equilibrative nucleotide transporter 3 OS=Arabidopsis thaliana GN=ENT3 PE=1 SV=1//2.9072e-34
XLOC_1611 -//-
XLOC_1612 -//-
XLOC_1613 sp|Q9C6D2|MTK_ARATH Methylthioribose kinase OS=Arabidopsis thaliana GN=MTK PE=1 SV=1//1.736e-31
XLOC_1614 sp|P51362|TRPG_PORPU Anthranilate synthase component II OS=Porphyra purpurea GN=trpG PE=4 SV=1//9.57381e-08
XLOC_1616 sp|Q0J709|UP12_ORYSJ Unknown protein DS12 from 2D-PAGE of leaf, chloroplastic OS=Oryza sativa subsp. japonica GN=Os08
XLOC_1616 sp|Q0J709|UP12_ORYSJ Unknown protein DS12 from 2D-PAGE of leaf, chloroplastic OS=Oryza sativa subsp. japonica GN=Os08
XLOC_1618 sp|P42158|KC1D_ARATH Casein kinase I isoform delta-like OS=Arabidopsis thaliana GN=At4g26100 PE=2 SV=2//3.43608e-12
XLOC_1620 -//-
XLOC_1621 -//-
XLOC_1621 sp|B5BSX1|BAMO_GLYUR Beta-amyrin 11-oxidase OS=Glycyrrhiza uralensis GN=CYP88D6 PE=1 SV=1//7.78366e-21
XLOC_1622 -//-
XLOC_1624 sp|Q8GW78|CLP41_ARATH Clp protease-related protein At4g12060, chloroplastic OS=Arabidopsis thaliana GN=At4g12060 PE=1
XLOC_1625 sp|A7Y3C4|PSBD_IPOPU Photosystem II D2 protein OS=Ipomoea purpurea GN=psbD PE=3 SV=1//5.8817e-106
XLOC_1625 sp|A3EWL3|REV1_ARATH DNA repair protein REV1 OS=Arabidopsis thaliana GN=REV1 PE=2 SV=1//9.28241e-12
XLOC_1625 -//-
XLOC_1626 -//-
XLOC_1626 sp|Q8W3Z4|CAS1_BETPL Cycloartenol synthase OS=Betula platyphylla GN=CASBPX1 PE=1 SV=1//4.32591e-15
XLOC_1627 -//-
XLOC_1628 -//-
XLOC_1629 -//-
XLOC_1631 -//-
XLOC_1631 -//-
XLOC_1632 sp|Q62785|HAP28_RAT 28 kDa heat- and acid-stable phosphoprotein OS=Rattus norvegicus GN=Pdap1 PE=1 SV=1//7.41298e-21
XLOC_1634 sp|Q54HF6|LSM7_DICDI Probable U6 snRNA-associated Sm-like protein LSm7 OS=Dictyostelium discoideum GN=lsm7 PE=3 SV=1/
XLOC_1634 sp|P10978|POLX_TOBAC Retrovirus-related Pol polyprotein from transposon TNT 1-94 OS=Nicotiana tabacum PE=2 SV=1//1.29
XLOC_1635 sp|P0A3U1|LTRA_LACLM Group II intron-encoded protein LtrA OS=Lactococcus lactis subsp. cremoris (strain MG1363) GN=lt
XLOC_1636 sp|O82768|HIS2_ARATH Histidine biosynthesis bifunctional protein hisIE, chloroplastic OS=Arabidopsis thaliana GN=At1g
XLOC_1636 sp|Q7XSK0|BGL18_ORYSJ Beta-glucosidase 18 OS=Oryza sativa subsp. japonica GN=BGLU18 PE=2 SV=2//0
XLOC_1637 sp|Q8LBL1|TPK1_ARATH Two-pore potassium channel 1 OS=Arabidopsis thaliana GN=TPK1 PE=1 SV=2//1.35491e-71
XLOC_1637 sp|P83877|TXN4A_MOUSE Thioredoxin-like protein 4A OS=Mus musculus GN=Txnl4a PE=2 SV=1//8.09449e-08
XLOC_1638 sp|Q9SZL8|FRS5_ARATH Protein FAR1-RELATED SEQUENCE 5 OS=Arabidopsis thaliana GN=FRS5 PE=2 SV=1//3.69011e-07

XLOC_1639 sp|P00054|CYC_SESIN Cytochrome c OS=Sesamum indicum PE=1 SV=1//9.84578e-63
XLOC_1642 sp|P0C2F6|RNHX1_ARATH Putative ribonuclease H protein At1g65750 OS=Arabidopsis thaliana GN=At1g65750 PE=3 SV=1//5.656
XLOC_1643 -//-
XLOC_1644 -//-
XLOC_1646 sp|Q80TA1|EPT1_MOUSE Ethanolaminephosphotransferase 1 OS=Mus musculus GN=Ept1 PE=2 SV=3//1.57236e-08
XLOC_1651 sp|Q42064|RL83_ARATH 60S ribosomal protein L8-3 OS=Arabidopsis thaliana GN=RPL8C PE=1 SV=2//1.03725e-58
XLOC_1655 sp|Q5VNM3|RH2_ORYSJ DEAD-box ATP-dependent RNA helicase 2 OS=Oryza sativa subsp. japonica GN=Os01g0639100 PE=2 SV=1//
XLOC_1655 -//-
XLOC_1656 sp|Q9LSL5|LRK92_ARATH L-type lectin-domain containing receptor kinase IX.2 OS=Arabidopsis thaliana GN=LECRK92 PE=2 SV
XLOC_1657 sp|P80269|NDUS8_SOLTU NADH dehydrogenase [ubiquinone] iron-sulfur protein 8, mitochondrial OS=Solanum tuberosum PE=1
XLOC_1657 -//-
XLOC_1658 sp|Q9C6C3|AGD2_ARATH ADP-ribosylation factor GTPase-activating protein AGD2 OS=Arabidopsis thaliana GN=AGD2 PE=2 SV=1
XLOC_1658 sp|Q01667|CAB6_ARATH Chlorophyll a-b binding protein 6, chloroplastic OS=Arabidopsis thaliana GN=LHCA1 PE=1 SV=1//1.5
XLOC_1658 sp|Q9SFB5|SCP27_ARATH Serine carboxypeptidase-like 27 OS=Arabidopsis thaliana GN=SCPL27 PE=2 SV=1//3.02552e-83
XLOC_1658 -//-
XLOC_1659 sp|Q8VYA5|RSZ33_ARATH Serine/arginine-rich splicing factor RS2Z33 OS=Arabidopsis thaliana GN=RS2Z33 PE=1 SV=1//1.7211
XLOC_1660 -//-
XLOC_1660 -//-
XLOC_1661 sp|P51851|PDC2_PEA Pyruvate decarboxylase isozyme 2 (Fragment) OS=Pisum sativum GN=PDC2 PE=2 SV=1//1.41173e-115
XLOC_1663 sp|Q93Y22|COPD_ARATH Coatomer subunit delta OS=Arabidopsis thaliana GN=At5g05010 PE=1 SV=2//0
XLOC_1664 sp|Q7XLY8|ATL41_ORYSJ E3 ubiquitin-protein ligase Os04g0590900 OS=Oryza sativa subsp. japonica GN=Os04g0590900 PE=2 S
XLOC_1665 -//-
XLOC_1665 sp|P10708|CB12_SOLLC Chlorophyll a-b binding protein 7, chloroplastic OS=Solanum lycopersicum GN=CAB7 PE=3 SV=1//1.27
XLOC_1665 -//-
XLOC_1668 sp|P10978|POLX_TOBAC Retrovirus-related Pol polyprotein from transposon TNT 1-94 OS=Nicotiana tabacum PE=2 SV=1//9.88
XLOC_1668 sp|Q10EJ2|CSPLU_ORYSJ CASP-like protein Os03g0767900 OS=Oryza sativa subsp. japonica GN=Os03g0767900 PE=2 SV=1//9.777
XLOC_1668 -//-
XLOC_1668 -//-
XLOC_1668 sp|Q42669|ACOC_CUCMC Aconitate hydratase (Fragment) OS=Cucumis melo var. conomon GN=ACO PE=2 SV=1//1.36741e-16
XLOC_1669 sp|Q9LN95|BH055_ARATH Transcription factor bHLH55 OS=Arabidopsis thaliana GN=BHLH55 PE=2 SV=1//4.4584e-18
XLOC_1669 sp|Q9FYA6|BCAT5_ARATH Branched-chain-amino-acid aminotransferase 5, chloroplastic OS=Arabidopsis thaliana GN=BCAT5 PE
XLOC_1669 sp|Q9M439|BCAT2_ARATH Branched-chain-amino-acid aminotransferase 2, chloroplastic OS=Arabidopsis thaliana GN=BCAT2 PE
XLOC_1669 sp|P46488|MDHG_CUCSA Malate dehydrogenase, glyoxysomal OS=Cucumis sativus GN=MDHG PE=2 SV=1//0
XLOC_1669 -//-
XLOC_1669 sp|Q69U54|TLP12_ORYSJ Tubby-like F-box protein 12 OS=Oryza sativa subsp. japonica GN=TULP12 PE=2 SV=1//3.34247e-54

XLOC_1669 -//-
XLOC_1670 sp|Q9LMA1|FMO1_ARATH Probable flavin-containing monooxygenase 1 OS=Arabidopsis thaliana GN=FMO1 PE=2 SV=1//8.33214e-1
XLOC_1670 -//-
XLOC_1670 -//-
XLOC_1670 sp|O65718|CNGC2_ARATH Cyclic nucleotide-gated ion channel 2 OS=Arabidopsis thaliana GN=CNGC2 PE=1 SV=1//5.07148e-125
XLOC_1670 sp|Q39785|CHI2_GOSHI Endochitinase 2 (Fragment) OS=Gossypium hirsutum PE=2 SV=2//1.49931e-54
XLOC_1670 sp|Q39799|CHI1_GOSHI Endochitinase 1 OS=Gossypium hirsutum PE=3 SV=1//3.4713e-157
XLOC_1670 sp|Q9SX98|LHTL8_ARATH Lysine histidine transporter-like 8 OS=Arabidopsis thaliana GN=AATL1 PE=1 SV=1//1.7182e-60
XLOC_1670 sp|P29162|ASOL_TOBAC L-ascorbate oxidase homolog OS=Nicotiana tabacum PE=2 SV=1//1.50422e-11
XLOC_1670 sp|Q00624|ASOL_BRANA L-ascorbate oxidase homolog OS=Brassica napus GN=Bp10 PE=2 SV=1//6.6664e-104
XLOC_1671 -//-
XLOC_1671 -//-
XLOC_1671 -//-
XLOC_1671 -//-
XLOC_1671 sp|Q9SZN7|HIP26_ARATH Heavy metal-associated isoprenylated plant protein 26 OS=Arabidopsis thaliana GN=HIPP26 PE=1 SV
XLOC_1671 sp|Q42564|APX3_ARATH L-ascorbate peroxidase 3, peroxisomal OS=Arabidopsis thaliana GN=APX3 PE=1 SV=1//4.70652e-10
XLOC_1671 sp|P81393|MYB08_ANTMA Myb-related protein 308 OS=Antirrhinum majus GN=MYB308 PE=2 SV=1//5.06218e-108
XLOC_1672 -//-
XLOC_1672 sp|Q8L883|LAX5_MEDTR Auxin transporter-like protein 5 OS=Medicago truncatula GN=LAX5 PE=2 SV=1//1.20783e-32
XLOC_1672 -//-
XLOC_1673 sp|Q9LRR4|R13L1_ARATH Putative disease resistance RPP13-like protein 1 OS=Arabidopsis thaliana GN=RPPL1 PE=2 SV=1//1.
XLOC_1674 -//-
XLOC_1675 -//-
XLOC_1675 sp|Q9LFN6|RH56_ARATH DEAD-box ATP-dependent RNA helicase 56 OS=Arabidopsis thaliana GN=RH56 PE=2 SV=2//2.8812e-11
XLOC_1676 -//-
XLOC_1676 sp|Q6ZGL9|SIP1_ORYSJ Signal peptide peptidase 1 OS=Oryza sativa subsp. japonica GN=SPP1 PE=2 SV=1//4.88633e-25
XLOC_1677 -//-
XLOC_1677 -//-
XLOC_1680 sp|P69322|UBIQP_PEA Polyubiquitin OS=Pisum sativum GN=PU1 PE=2 SV=2//0
XLOC_1681 sp|Q9LNG5|PPP7L_ARATH Serine/threonine-protein phosphatase 7 long form homolog OS=Arabidopsis thaliana GN=At1g48120 P
XLOC_1682 sp|Q945S8|ASHH3_ARATH Histone-lysine N-methyltransferase ASHH3 OS=Arabidopsis thaliana GN=ASHH3 PE=2 SV=2//4.0867e-07
XLOC_1682 sp|F4I0P8|VP35B_ARATH Vacuolar protein sorting-associated protein 35B OS=Arabidopsis thaliana GN=VPS35B PE=1 SV=1//7.
XLOC_1684 -//-
XLOC_1686 sp|Q8GW61|STP14_ARATH Sugar transport protein 14 OS=Arabidopsis thaliana GN=STP14 PE=2 SV=2//4.71703e-42
XLOC_1688 sp|Q8H103|G6PIP_ARATH Glucose-6-phosphate isomerase 1, chloroplastic OS=Arabidopsis thaliana GN=PGI1 PE=1 SV=1//1.042

XLOC_1688 sp|Q87SU3|RL27_VIBPA 50S ribosomal protein L27 OS=Vibrio parahaemolyticus serotype O3:K6 (strain RIMD 2210633) GN=rpm
XLOC_1689 sp|Q8LBB7|LOG5_ARATH Cytokinin riboside 5'-monophosphate phosphoribohydrolase LOG5 OS=Arabidopsis thaliana GN=LC
XLOC_1691 -//-
XLOC_1692 -//-
XLOC_1692 sp|O81312|GAE3_ARATH UDP-glucuronate 4-epimerase 3 OS=Arabidopsis thaliana GN=GAE3 PE=2 SV=1//0
XLOC_1692 sp|O82531|PSB1_PETHY Proteasome subunit beta type-1 OS=Petunia hybrida GN=PBF1 PE=2 SV=1//4.68392e-154
XLOC_1694 sp|Q9AR19|GCN5_ARATH Histone acetyltransferase GCN5 OS=Arabidopsis thaliana GN=HAG1 PE=1 SV=1//3.88893e-26
XLOC_1695 sp|P33082|AXX15_SOYBN Auxin-induced protein X15 OS=Glycine max PE=2 SV=1//1.40286e-10
XLOC_1696 -//-
XLOC_1698 sp|Q9CA28|TKPR2_ARATH Tetraketide alpha-pyrone reductase 2 OS=Arabidopsis thaliana GN=TKPR2 PE=2 SV=1//1.26814e-38
XLOC_1699 sp|Q6NNI8|SAP1_ARATH Zinc finger A20 and AN1 domain-containing stress-associated protein 1 OS=Arabidopsis thaliana GN
XLOC_1702 sp|Q84K16|AP1G1_ARATH AP-1 complex subunit gamma-1 OS=Arabidopsis thaliana GN=GAMMA-ADR PE=1 SV=1//3.16903e-26
XLOC_1705 sp|Q9M2Y6|Y3972_ARATH Uncharacterized protein At3g49720 OS=Arabidopsis thaliana GN=At3g49720 PE=1 SV=1//1.66106e-71
XLOC_1706 sp|Q9LJM4|IKU2_ARATH Receptor-like protein kinase HAIKU2 OS=Arabidopsis thaliana GN=IKU2 PE=1 SV=1//0
XLOC_1706 -//-
XLOC_1706 sp|P00303|BABL_CUCSA Basic blue protein OS=Cucumis sativus PE=1 SV=1//1.04087e-38
XLOC_1706 sp|Q9LDX1|SGS3_ARATH Protein SUPPRESSOR OF GENE SILENCING 3 OS=Arabidopsis thaliana GN=SGS3 PE=1 SV=1//1.47446e-119
XLOC_1707 sp|P04146|COPIA_DROME Copia protein OS=Drosophila melanogaster GN=GIP PE=1 SV=3//2.00117e-14
XLOC_1707 sp|Q8N5L8|RP25L_HUMAN Ribonuclease P protein subunit p25-like protein OS=Homo sapiens GN=RPP25L PE=1 SV=1//2.08079e-0
XLOC_1707 -//-
XLOC_1707 sp|O49354|COQ3_ARATH Hexaprenyldihydroxybenzoate methyltransferase, mitochondrial OS=Arabidopsis thaliana GN=COQ3 PE=
XLOC_1707 sp|O65718|CNGC2_ARATH Cyclic nucleotide-gated ion channel 2 OS=Arabidopsis thaliana GN=CNGC2 PE=1 SV=1//0
XLOC_1708 -//-
XLOC_1708 sp|O80905|S40A1_ARATH Solute carrier family 40 member 1 OS=Arabidopsis thaliana GN=IREG1 PE=2 SV=1//1.41718e-19
XLOC_1708 sp|F4KGN5|S40A2_ARATH Solute carrier family 40 member 2 OS=Arabidopsis thaliana GN=IREG2 PE=2 SV=1//3.92041e-42
XLOC_1708 sp|O08863|BIRC3_MOUSE Baculoviral IAP repeat-containing protein 3 OS=Mus musculus GN=Birc3 PE=1 SV=2//2.56335e-06
XLOC_1709 -//-
XLOC_1709 -//-
XLOC_1710 -//-
XLOC_1710 -//-
XLOC_1711 -//-
XLOC_1712 sp|P93295|M310_ARATH Uncharacterized mitochondrial protein AtMg00310 OS=Arabidopsis thaliana GN=AtMg00310 PE=4 SV=1//
XLOC_1712 -//-
XLOC_1712 -//-
XLOC_1712 sp|Q9ZU90|SKI28_ARATH F-box protein SKIP28 OS=Arabidopsis thaliana GN=SKIP28 PE=1 SV=1//4.24378e-64

XLOC_1713	-//-
XLOC_1715	sp|P48631|FD6E2_SOYBN Omega-6 fatty acid desaturase, endoplasmic reticulum isozyme 2 OS=Glycine max GN=FAD2-2 PE=2 SV
XLOC_1718	-//-
XLOC_1723	sp|Q9SE97|FH1_ARATH Formin-like protein 1 OS=Arabidopsis thaliana GN=FH1 PE=1 SV=1//2.58864e-11
XLOC_1723	sp|O23278|PP310_ARATH Pentatricopeptide repeat-containing protein At4g14190, chloroplastic OS=Arabidopsis thaliana GN
XLOC_1725	sp|P03010|TRAC9_MAIZE Putative AC9 transposase OS=Zea mays PE=4 SV=1//8.11531e-28
XLOC_1728	sp|Q9C944|H2AV3_ARATH Probable histone H2A variant 3 OS=Arabidopsis thaliana GN=At1g52740 PE=1 SV=1//7.86525e-21
XLOC_1728	sp|P62302|RS13_SOYBN 40S ribosomal protein S13 OS=Glycine max GN=RPS13 PE=2 SV=1//3.26306e-101
XLOC_1729	sp|O80739|POT12_ARATH Putative potassium transporter 12 OS=Arabidopsis thaliana GN=POT12 PE=1 SV=2//1.48414e-31
XLOC_1733	sp|P10978|POLX_TOBAC Retrovirus-related Pol polyprotein from transposon TNT 1-94 OS=Nicotiana tabacum PE=2 SV=1//4.76
XLOC_1736	sp|Q9FQ03|XRN3_ARATH 5'-3' exoribonuclease 3 OS=Arabidopsis thaliana GN=XRN3 PE=2 SV=1//1.1796e-11
XLOC_1736	-//-
XLOC_1742	sp|Q42539|PIMT_ARATH Protein-L-isoaspartate O-methyltransferase OS=Arabidopsis thaliana GN=PCM PE=2 SV=3//3.533e-69
XLOC_1745	sp|P53997|SET_DROME Protein SET OS=Drosophila melanogaster GN=Set PE=1 SV=2//3.56724e-53
XLOC_1745	sp|Q9S7V4|HA22A_ARATH HVA22-like protein a OS=Arabidopsis thaliana GN=HVA22A PE=2 SV=1//9.40393e-60
XLOC_1745	-//-
XLOC_1749	sp|Q39065|COPT1_ARATH Copper transporter 1 OS=Arabidopsis thaliana GN=COPT1 PE=2 SV=2//9.54306e-33
XLOC_1749	-//-
XLOC_1749	sp|Q8LGG8|USPAL_ARATH Universal stress protein A-like protein OS=Arabidopsis thaliana GN=At3g01520 PE=1 SV=2//1.04199
XLOC_1751	sp|P42855|ZB14_BRAJU 14 kDa zinc-binding protein (Fragment) OS=Brassica juncea PE=3 SV=1//1.00364e-29
XLOC_1751	-//-
XLOC_1751	sp|Q9FMA3|PEX5_ARATH Peroxisome biogenesis protein 5 OS=Arabidopsis thaliana GN=PEX5 PE=1 SV=1//1.07466e-34
XLOC_1751	sp|Q93YQ1|RTOR1_ARATH Regulatory-associated protein of TOR 1 OS=Arabidopsis thaliana GN=RAPTOR1 PE=1 SV=1//9.56661e-1
XLOC_1752	-//-
XLOC_1752	-//-
XLOC_1753	sp|Q71H73|H33_VITVI Histone H3.3 OS=Vitis vinifera PE=2 SV=3//1.34847e-92
XLOC_1756	sp|P83877|TXN4A_MOUSE Thioredoxin-like protein 4A OS=Mus musculus GN=Txnl4a PE=2 SV=1//7.00915e-20
XLOC_1757	-//-
XLOC_1757	-//-
XLOC_1757	-//-
XLOC_1766	-//-
XLOC_1768	sp|Q9LW86|SUT34_ARATH Probable sulfate transporter 3.4 OS=Arabidopsis thaliana GN=SULTR3;4 PE=2 SV=1//1.85093e-08
XLOC_1768	-//-
XLOC_1770	sp|O22666|RGP3_ARATH UDP-arabinopyranose mutase 3 OS=Arabidopsis thaliana GN=RGP3 PE=1 SV=2//3.45578e-09
XLOC_1771	-//-

XLOC_1772 sp|Q9SB67|NRT32_ARATH High-affinity nitrate transporter 3.2 OS=Arabidopsis thaliana GN=NRT3.2 PE=2 SV=1//1.06039e-61
XLOC_1774 sp|P53997|SET_DROME Protein SET OS=Drosophila melanogaster GN=Set PE=1 SV=2//2.21181e-49
XLOC_1775 -//-
XLOC_1776 -//-
XLOC_1778 -//-
XLOC_1779 -//-
XLOC_1780 sp|Q6K833|MCES2_ORYSJ mRNA cap guanine-N7 methyltransferase 2 OS=Oryza sativa subsp. japonica GN=Os02g0780600 PE=2 SV
XLOC_1781 sp|O48922|C98A2_SOYBN Cytochrome P450 98A2 OS=Glycine max GN=CYP98A2 PE=2 SV=1//0
XLOC_1781 sp|O65780|GALE1_CYATE UDP-glucose 4-epimerase GEPI42 OS=Cyamopsis tetragonoloba PE=2 SV=1//1.75558e-24
XLOC_1781 sp|C0LGS2|Y4361_ARATH Probable LRR receptor-like serine/threonine-protein kinase At4g36180 OS=Arabidopsis thaliana GN
XLOC_1783 -//-
XLOC_1783 sp|Q7Z9L3|EXGA_ASPOR Glucan 1,3-beta-glucosidase A OS=Aspergillus oryzae (strain ATCC 42149 / RIB 40) GN=exgA PE=1 SV
XLOC_1785 sp|Q9SUG3|ITPK2_ARATH Inositol-tetrakisphosphate 1-kinase 2 OS=Arabidopsis thaliana GN=ITPK2 PE=2 SV=2//6.41585e-08
XLOC_1785 sp|Q9LX65|VATH_ARATH V-type proton ATPase subunit H OS=Arabidopsis thaliana GN=VHA-H PE=2 SV=1//4.50147e-53
XLOC_1785 -//-
XLOC_1785 sp|Q84KP0|DFRA_PYRCO Bifunctional dihydroflavonol 4-reductase/flavanone 4-reductase OS=Pyrus communis GN=DFR PE=1 SV=
XLOC_1786 sp|Q8W206|CSN6A_ARATH COP9 signalosome complex subunit 6a OS=Arabidopsis thaliana GN=CSN6A PE=1 SV=2//2.45539e-10
XLOC_1786 sp|P83877|TXN4A_MOUSE Thioredoxin-like protein 4A OS=Mus musculus GN=Txnl4a PE=2 SV=1//1.42844e-15
XLOC_1786 sp|F4I933|STR8_ARATH Rhodanese-like domain-containing protein 8, chloroplastic OS=Arabidopsis thaliana GN=STR8 PE=2 S
XLOC_1790 -//-
XLOC_1790 sp|Q8LPT1|AB6B_ARATH ABC transporter B family member 6 OS=Arabidopsis thaliana GN=ABCB6 PE=1 SV=2//4.92393e-19
XLOC_1791 -//-
XLOC_1797 sp|P93286|CCMF_ARATH Putative cytochrome c biogenesis ccmF-like mitochondrial protein OS=Arabidopsis thaliana GN=CCMF
XLOC_1797 sp|P31839|NU1M_OENBE NADH-ubiquinone oxidoreductase chain 1 OS=Oenothera berteriana GN=ND1 PE=2 SV=1//2.90188e-22
XLOC_1797 sp|Q2L926|CLPP_GOSHI ATP-dependent Clp protease proteolytic subunit OS=Gossypium hirsutum GN=clpP PE=3 SV=1//5.06215e
XLOC_1798 -//-
XLOC_1798 sp|Q9DBB4|NAA16_MOUSE N-alpha-acetyltransferase 16, NatA auxiliary subunit OS=Mus musculus GN=Naa16 PE=2 SV=1//9.1368
XLOC_1799 sp|Q9SR02|MED14_ARATH Mediator of RNA polymerase II transcription subunit 14 OS=Arabidopsis thaliana GN=MED14 PE=1 SV
XLOC_1799 sp|Q9S6Z8|TPK5_ARATH Two-pore potassium channel 5 OS=Arabidopsis thaliana GN=TPK5 PE=1 SV=1//1.12248e-08
XLOC_1800 -//-
XLOC_1801 sp|Q688R1|MTP1_ORYSJ Metal tolerance protein 1 OS=Oryza sativa subsp. japonica GN=MTP1 PE=2 SV=1//7.49933e-32
XLOC_1802 sp|Q8LBI9|EDL16_ARATH Sugar transporter ERD6-like 16 OS=Arabidopsis thaliana GN=At5g18840 PE=2 SV=2//6.23547e-07
XLOC_1802 -//-
XLOC_1802 sp|P08770|TRA1_MAIZE Putative AC transposase OS=Zea mays PE=2 SV=2//2.76135e-75
XLOC_1803 -//-

XLOC_1805 sp|Q9Y5Q8|TF3C5_HUMAN General transcription factor 3C polypeptide 5 OS=Homo sapiens GN=GTF3C5 PE=1 SV=2//3.21161e-29
XLOC_1806 sp|Q9SA27|RH36_ARATH DEAD-box ATP-dependent RNA helicase 36 OS=Arabidopsis thaliana GN=RH36 PE=2 SV=1//4.09769e-22
XLOC_1807 sp|P42791|RL182_ARATH 60S ribosomal protein L18-2 OS=Arabidopsis thaliana GN=RPL18B PE=1 SV=2//2.51229e-117
XLOC_1807 sp|O05000|NU2M_ARATH NADH-ubiquinone oxidoreductase chain 2 OS=Arabidopsis thaliana GN=ND2 PE=2 SV=2//6.21062e-23
XLOC_1807 sp|Q5HZ60|MCES2_ARATH mRNA cap guanine-N7 methyltransferase 2 OS=Arabidopsis thaliana GN=At3g52210 PE=2 SV=1//2.15822
XLOC_1809 sp|Q9KG32|Y283_BACHD Uncharacterized isomerase BH0283 OS=Bacillus halodurans (strain ATCC BAA-125 / DSM 18197 / FERM
XLOC_1809 sp|Q8RVH5|7SBG2_SOYBN Basic 7S globulin 2 OS=Glycine max PE=1 SV=1//9.29323e-10
XLOC_1809 -//-
XLOC_1809 sp|Q9JKB8|PRP18_RAT Pre-mRNA-splicing factor 18 OS=Rattus norvegicus GN=Prpf18 PE=2 SV=1//5.55812e-51
XLOC_1810 sp|P32869|PSAD_CUCSA Photosystem I reaction center subunit II, chloroplastic OS=Cucumis sativus GN=psaD PE=1 SV=1//6.
XLOC_1810 -//-
XLOC_1810 -//-
XLOC_1810 sp|Q8H7Y6|HFA2D_ORYSJ Heat stress transcription factor A-2d OS=Oryza sativa subsp. japonica GN=HSFA2D PE=2 SV=2//4.07
XLOC_1814 -//-
XLOC_1816 sp|Q94AH9|MD36A_ARATH Mediator of RNA polymerase II transcription subunit 36a OS=Arabidopsis thaliana GN=MED36A PE=1
XLOC_1818 sp|Q945L0|CX6B2_ARATH Cytochrome c oxidase subunit 6b-2 OS=Arabidopsis thaliana GN=COX6B-2 PE=2 SV=1//3.36382e-43
XLOC_1820 sp|P51428|RT10_PEA Ribosomal protein S10, mitochondrial OS=Pisum sativum GN=RPS10 PE=2 SV=1//1.07466e-31
XLOC_1820 -//-
XLOC_1821 -//-
XLOC_1821 -//-
XLOC_1825 sp|O04300|UPTG_PEA Alpha-1,4-glucan-protein synthase [UDP-forming] OS=Pisum sativum GN=UPTG PE=1 SV=1//4.57915e-09
XLOC_1828 sp|Q40153|LE14B_LITER LEC14B protein OS=Lithospermum erythrorhizon PE=2 SV=1//2.53009e-13
XLOC_1828 sp|Q6ZGL9|SIP1_ORYSJ Signal peptide peptidase 1 OS=Oryza sativa subsp. japonica GN=SPP1 PE=2 SV=1//8.81913e-18
XLOC_1830 -//-
XLOC_1832 -//-
XLOC_1832 -//-
XLOC_1833 sp|P61600|NAA20_MOUSE N-alpha-acetyltransferase 20 OS=Mus musculus GN=Naa20 PE=2 SV=1//1.48414e-16
XLOC_1833 sp|Q9FUM9|HMT2_MAIZE Homocysteine S-methyltransferase 2 OS=Zea mays GN=HMT-2 PE=2 SV=1//8.95792e-25
XLOC_1833 sp|Q9M1W4|HMT2_ARATH Homocysteine S-methyltransferase 2 OS=Arabidopsis thaliana GN=HMT-2 PE=1 SV=1//1.16548e-20
XLOC_1835 sp|Q94IR2|CCD1_PHAVU Carotenoid 9,10(9',10')-cleavage dioxygenase 1 OS=Phaseolus vulgaris GN=CCD1 PE=1 SV=1
XLOC_1836 sp|Q0WPZ6|PP158_ARATH Pentatricopeptide repeat-containing protein At2g17140 OS=Arabidopsis thaliana GN=At2g17140 PE=2
XLOC_1839 -//-
XLOC_1841 sp|Q653P0|KOR1_ORYSJ Potassium channel KOR1 OS=Oryza sativa subsp. japonica GN=Os06g0250600 PE=2 SV=1//9.94711e-60
XLOC_1842 sp|Q94F30|ESD4_ARATH Ubiquitin-like-specific protease ESD4 OS=Arabidopsis thaliana GN=ESD4 PE=1 SV=1//7.42661e-14
XLOC_1845 sp|Q9SUI5|PSAK_ARATH Photosystem I reaction center subunit psaK, chloroplastic OS=Arabidopsis thaliana GN=PSAK PE=2 S

XLOC_1846 sp|P10978|POLX_TOBAC Retrovirus-related Pol polyprotein from transposon TNT 1-94 OS=Nicotiana tabacum PE=2 SV=1//1.61
XLOC_1846 -//-
XLOC_1851 sp|A6UQ90|GUAAA_METVS GMP synthase [glutamine-hydrolyzing] subunit A OS=Methanococcus vannielii (strain SB / ATCC 350
XLOC_1852 sp|O04287|FKB12_VICFA Peptidyl-prolyl cis-trans isomerase FKBP12 OS=Vicia faba GN=FKBP12 PE=1 SV=1//9.00162e-57
XLOC_1853 -//-
XLOC_1858 sp|A7ZE19|RF2_CAMC1 Peptide chain release factor 2 OS=Campylobacter concisus (strain 13826) GN=prfB PE=3 SV=1//2.7647
XLOC_1859 sp|Q2MY58|PATA3_SOLTU Patatin group A-3 OS=Solanum tuberosum PE=2 SV=1//9.52321e-26
XLOC_1859 sp|Q86K80|U420_DICDI UPF0420 protein OS=Dictyostelium discoideum GN=DDB_G0277179 PE=3 SV=1//1.95692e-23
XLOC_1860 sp|Q9SCQ7|AN32_ARATH Acidic leucine-rich nuclear phosphoprotein 32-related protein OS=Arabidopsis thaliana GN=At3g506
XLOC_1864 sp|Q941R6|MLP31_ARATH MLP-like protein 31 OS=Arabidopsis thaliana GN=MLP31 PE=1 SV=2//9.50432e-59
XLOC_1865 sp|P20825|POL2_DROME Retrovirus-related Pol polyprotein from transposon 297 OS=Drosophila melanogaster GN=pol PE=4 SV
XLOC_1865 sp|P10978|POLX_TOBAC Retrovirus-related Pol polyprotein from transposon TNT 1-94 OS=Nicotiana tabacum PE=2 SV=1//1.78
XLOC_1870 sp|Q9C671|P4K2B_ARATH Probable phosphatidylinositol 4-kinase type 2-beta At1g26270 OS=Arabidopsis thaliana GN=At1g262
XLOC_1872 sp|Q6TAW2|SERP2_MOUSE Stress-associated endoplasmic reticulum protein 2 OS=Mus musculus GN=Serp2 PE=2 SV=2//1.04232e-
XLOC_1872 -//-
XLOC_1873 sp|O64459|UGPA_PYRPY UTP--glucose-1-phosphate uridylyltransferase OS=Pyrus pyrifolia PE=2 SV=1//1.52336e-13
XLOC_1876 -//-
XLOC_1877 sp|Q6R2J8|SRF8_ARATH Protein STRUBBELIG-RECEPTOR FAMILY 8 OS=Arabidopsis thaliana GN=SRF8 PE=2 SV=1//0
XLOC_1878 -//-
XLOC_1881 sp|Q94DM8|UFM1_ORYSJ Ubiquitin-fold modifier 1 OS=Oryza sativa subsp. japonica GN=Os01g0962400 PE=3 SV=1//2.84637e-13
XLOC_1882 sp|Q99315|YG31B_YEAST Transposon Ty3-G Gag-Pol polyprotein OS=Saccharomyces cerevisiae (strain ATCC 204508 / S288c) G
XLOC_1884 sp|Q9BZV1|UBXN6_HUMAN UBX domain-containing protein 6 OS=Homo sapiens GN=UBXN6 PE=1 SV=1//3.41319e-09
XLOC_1886 sp|Q6DBN1|Y4845_ARATH BTB/POZ domain-containing protein At4g08455 OS=Arabidopsis thaliana GN=At4g08455 PE=1 SV=1//2.2
XLOC_1887 sp|Q9M291|SC24C_ARATH Protein transport protein Sec24-like CEF OS=Arabidopsis thaliana GN=CEF PE=1 SV=3//3.98286e-29
XLOC_1888 -//-
XLOC_1890 -//-
XLOC_1891 sp|Q9LKR4|FRS10_ARATH Putative protein FAR1-RELATED SEQUENCE 10 OS=Arabidopsis thaliana GN=FRS10 PE=2 SV=2//0
XLOC_1893 sp|Q6VAF7|TBB5_GOSHI Tubulin beta-5 chain OS=Gossypium hirsutum PE=2 SV=1//4.32264e-142
XLOC_1900 sp|Q2V3H0|PP322_ARATH Pentatricopeptide repeat-containing protein At4g18975, chloroplastic OS=Arabidopsis thaliana GN
XLOC_1900 sp|Q05737|YPTM2_MAIZE GTP-binding protein YPTM2 OS=Zea mays GN=YPTM2 PE=2 SV=1//2.71937e-16
XLOC_1900 sp|B0BN95|HARB1_RAT Putative nuclease HARBI1 OS=Rattus norvegicus GN=Harbi1 PE=2 SV=1//1.80824e-14
XLOC_1901 -//-
XLOC_1901 -//-
XLOC_1903 sp|P02982|TCR1_ECOLX Tetracycline resistance protein, class A OS=Escherichia coli GN=tetA PE=3 SV=2//2.45498e-08
XLOC_1911 sp|Q9SGW3|RP12A_ARATH 26S proteasome non-ATPase regulatory subunit RPN12A OS=Arabidopsis thaliana GN=RPN12A PE=1 SV=1

XLOC_1911-//-
XLOC_1911sp|Q6NPT8|PP2B1_ARATH F-box protein PP2-B1 OS=Arabidopsis thaliana GN=PP2B1 PE=1 SV=1//3.45836e-73
XLOC_1912sp|Q84TF5|RHA4A_ARATH RING-H2 zinc finger protein RHA4a OS=Arabidopsis thaliana GN=RHA4A PE=2 SV=1//2.99102e-16
XLOC_1913sp|Q05047|C72A1_CATRO Secologanin synthase OS=Catharanthus roseus GN=CYP72A1 PE=2 SV=1//7.90983e-24
XLOC_1913sp|P08770|TRA1_MAIZE Putative AC transposase OS=Zea mays PE=2 SV=2//5.66235e-95
XLOC_1915sp|F4K1B1|RPAP2_ARATH Putative RNA polymerase II subunit B1 CTD phosphatase RPAP2 homolog OS=Arabidopsis thaliana GN=
XLOC_1916sp|Q9C566|CYP40_ARATH Peptidyl-prolyl cis-trans isomerase CYP40 OS=Arabidopsis thaliana GN=CYP40 PE=2 SV=1//8.61282e-
XLOC_1919-//-
XLOC_1923-//-
XLOC_1926sp|O04616|Y4115_ARATH Uncharacterized protein At4g01150, chloroplastic OS=Arabidopsis thaliana GN=At4g01150 PE=1 SV=1
XLOC_1927sp|Q9SRX2|RL191_ARATH 60S ribosomal protein L19-1 OS=Arabidopsis thaliana GN=RPL19A PE=2 SV=1//1.04631e-12
XLOC_1928sp|Q9LQU4|PCR2_ARATH Protein PLANT CADMIUM RESISTANCE 2 OS=Arabidopsis thaliana GN=PCR2 PE=1 SV=1//1.04716e-58
XLOC_1930-//-
XLOC_1930sp|Q8W425|PSMD6_ORYSJ 26S proteasome non-ATPase regulatory subunit 6 OS=Oryza sativa subsp. japonica GN=RPN7 PE=2 SV=
XLOC_1933sp|Q9SU30|CPR30_ARATH F-box protein CPR30 OS=Arabidopsis thaliana GN=CPR30 PE=1 SV=2//2.62299e-128
XLOC_1936sp|Q9SZL8|FRS5_ARATH Protein FAR1-RELATED SEQUENCE 5 OS=Arabidopsis thaliana GN=FRS5 PE=2 SV=1//8.83064e-19
XLOC_1936sp|P08770|TRA1_MAIZE Putative AC transposase OS=Zea mays PE=2 SV=2//1.61683e-62
XLOC_1937sp|P24280|SEC14_YEAST SEC14 cytosolic factor OS=Saccharomyces cerevisiae (strain ATCC 204508 / S288c) GN=SEC14 PE=1 S
XLOC_1937-//-
XLOC_1938sp|Q75LR7|SAPK1_ORYSJ Serine/threonine-protein kinase SAPK1 OS=Oryza sativa subsp. japonica GN=SAPK1 PE=1 SV=1//0
XLOC_1938-//-
XLOC_1939sp|P46299|RS4_GOSHI 40S ribosomal protein S4 OS=Gossypium hirsutum GN=RPS4 PE=2 SV=1//5.83319e-110
XLOC_1941sp|P10978|POLX_TOBAC Retrovirus-related Pol polyprotein from transposon TNT 1-94 OS=Nicotiana tabacum PE=2 SV=1//9.55
XLOC_1943-//-
XLOC_1944sp|P14009|14KD_DAUCA 14 kDa proline-rich protein DC2.15 OS=Daucus carota PE=2 SV=1//1.2088e-13
XLOC_1947sp|F4I933|STR8_ARATH Rhodanese-like domain-containing protein 8, chloroplastic OS=Arabidopsis thaliana GN=STR8 PE=2 S
XLOC_1953sp|Q12955|ANK3_HUMAN Ankyrin-3 OS=Homo sapiens GN=ANK3 PE=1 SV=3//9.90342e-06
XLOC_1954sp|Q8LCL3|RL272_ARATH 60S ribosomal protein L27-2 OS=Arabidopsis thaliana GN=RPL27B PE=2 SV=2//3.05814e-21
XLOC_1955sp|Q93Z27|CML45_ARATH Probable calcium-binding protein CML45 OS=Arabidopsis thaliana GN=CML45 PE=1 SV=1//8.40589e-39
XLOC_1955-//-
XLOC_1956-//-
XLOC_1958sp|Q9XHE4|RL37A_GOSHI 60S ribosomal protein L37a OS=Gossypium hirsutum GN=RPL37A PE=3 SV=1//1.18659e-56
XLOC_1961sp|Q9M2Z4|MSBP2_ARATH Membrane steroid-binding protein 2 OS=Arabidopsis thaliana GN=MSBP2 PE=1 SV=1//1.07453e-54
XLOC_1962-//-
XLOC_1962sp|P10978|POLX_TOBAC Retrovirus-related Pol polyprotein from transposon TNT 1-94 OS=Nicotiana tabacum PE=2 SV=1//1.39

XLOC_1964 sp|Q8S9H7|DIV_ANTMA Transcription factor DIVARICATA OS=Antirrhinum majus GN=DIVARICATA PE=2 SV=1//8.01962e-21
XLOC_1965 sp|C7J4U3|OST4A_ORYSJ Dolichyl-diphosphooligosaccharide--protein glycosyltransferase subunit 4A OS=Oryza sativa subsp
XLOC_1966 sp|O64937|EF1A_ORYSJ Elongation factor 1-alpha OS=Oryza sativa subsp. japonica GN=REFA1 PE=2 SV=2//5.82335e-23
XLOC_1967 sp|Q8VYA5|RSZ33_ARATH Serine/arginine-rich splicing factor RS2Z33 OS=Arabidopsis thaliana GN=RS2Z33 PE=1 SV=1//1.7038
XLOC_1967 sp|Q54GS1|CL16A_DICDI Protein CLEC16A homolog OS=Dictyostelium discoideum GN=DDB_G0289943 PE=3 SV=1//6.65234e-07
XLOC_1968 -//-
XLOC_1971 -//-
XLOC_1971 sp|Q43155|GLTB_SPIOL Ferredoxin-dependent glutamate synthase, chloroplastic OS=Spinacia oleracea PE=1 SV=3//5.30473e-
XLOC_1973 sp|Q9LHN8|F6H1_ARATH Feruloyl CoA ortho-hydroxylase 1 OS=Arabidopsis thaliana GN=F6'H1 PE=1 SV=1//1.83083e-124
XLOC_1974 sp|Q9SY66|FRS11_ARATH Protein FAR1-RELATED SEQUENCE 11 OS=Arabidopsis thaliana GN=FRS11 PE=2 SV=1//0
XLOC_1974 -//-
XLOC_1975 sp|O24412|PSD7_ARATH Probable 26S proteasome non-ATPase regulatory subunit 7 OS=Arabidopsis thaliana GN=RPN8 PE=2 SV=
XLOC_1981 sp|Q2L934|PSBH_GOSHI Photosystem II reaction center protein H OS=Gossypium hirsutum GN=psbH PE=3 SV=1//2.02122e-38
XLOC_1984 sp|P42055|VDAC1_SOLTU Mitochondrial outer membrane protein porin of 34 kDa OS=Solanum tuberosum PE=1 SV=2//6.28848e-9
XLOC_1984 sp|Q9SD53|Y3720_ARATH UPF0481 protein At3g47200 OS=Arabidopsis thaliana GN=At3g47200 PE=1 SV=1//2.60196e-13
XLOC_1984 sp|Q6NQ88|DDB2_ARATH Protein DAMAGED DNA-BINDING 2 OS=Arabidopsis thaliana GN=DDB2 PE=1 SV=1//3.2898e-93
XLOC_1984 sp|Q9UT06|YLWB_SCHPO Uncharacterized GTP-binding protein P8A3.11c, mitochondrial OS=Schizosaccharomyces pombe (strain
XLOC_1988 sp|Q9SHH7|GSTUP_ARATH Glutathione S-transferase U25 OS=Arabidopsis thaliana GN=GSTU25 PE=2 SV=1//7.47124e-46
XLOC_1990 sp|Q6NQK2|NAC8_ARATH NAC domain-containing protein 8 OS=Arabidopsis thaliana GN=NAC008 PE=2 SV=1//6.83904e-27
XLOC_1991 sp|Q9FN09|NPY3_ARATH BTB/POZ domain-containing protein NPY3 OS=Arabidopsis thaliana GN=NPY3 PE=2 SV=1//1.78305e-07
XLOC_1993 sp|O49339|PTI12_ARATH PTI1-like tyrosine-protein kinase 2 OS=Arabidopsis thaliana GN=PTI12 PE=1 SV=1//5.74454e-16
XLOC_1997 sp|Q6R8G6|PHO14_ARATH Phosphate transporter PHO1 homolog 4 OS=Arabidopsis thaliana GN=PHO1-H4 PE=2 SV=1//4.31823e-94
XLOC_1997 sp|Q9ATY5|UVH3_ARATH DNA repair protein UVH3 OS=Arabidopsis thaliana GN=UVH3 PE=2 SV=1//1.37427e-28
XLOC_1997 sp|P53536|PHSL_VICFA Alpha-1,4 glucan phosphorylase L isozyme, chloroplastic/amyloplastic OS=Vicia faba GN=PHO1 PE=2
XLOC_1999 sp|Q9FL28|FLS2_ARATH LRR receptor-like serine/threonine-protein kinase FLS2 OS=Arabidopsis thaliana GN=FLS2 PE=1 SV=1
XLOC_1999 sp|Q9M8K6|MUTE_ARATH Transcription factor MUTE OS=Arabidopsis thaliana GN=MUTE PE=2 SV=1//2.22794e-71
XLOC_2000 sp|Q9P2Y5|UVRAG_HUMAN UV radiation resistance-associated gene protein OS=Homo sapiens GN=UVRAG PE=1 SV=1//1.64753e-12
XLOC_2002 -//-
XLOC_2003 sp|Q9LTF2|RS103_ARATH 40S ribosomal protein S10-3 OS=Arabidopsis thaliana GN=RPS10C PE=2 SV=2//9.50464e-52
XLOC_2004 -//-
XLOC_2018 -//-
XLOC_2020 -//-
XLOC_2021 sp|Q9C5Y0|PLDD1_ARATH Phospholipase D delta OS=Arabidopsis thaliana GN=PLDDELTA PE=1 SV=2//1.06676e-12
XLOC_2021 -//-
XLOC_2023 -//-

XLOC_2025 sp|Q9SHI2|Y1723_ARATH Leucine-rich repeat receptor-like serine/threonine-protein kinase At1g17230 OS=Arabidopsis thal
XLOC_2028 sp|Q84MB3|ACCH1_ARATH 1-aminocyclopropane-1-carboxylate oxidase homolog 1 OS=Arabidopsis thaliana GN=At1g06620 PE=2 S
XLOC_2030 -//-
XLOC_2030 sp|O23016|KCAB_ARATH Probable voltage-gated potassium channel subunit beta OS=Arabidopsis thaliana GN=KAB1 PE=1 SV=1/
XLOC_2032 sp|Q5R8J8|DNJB4_PONAB DnaJ homolog subfamily B member 4 OS=Pongo abelii GN=DNAJB4 PE=2 SV=1//7.29239e-08
XLOC_2034 sp|Q6NQJ8|SDG40_ARATH Protein SET DOMAIN GROUP 40 OS=Arabidopsis thaliana GN=SDG40 PE=2 SV=1//2.32403e-07
XLOC_2034 -//-
XLOC_2035 sp|Q8GW61|STP14_ARATH Sugar transport protein 14 OS=Arabidopsis thaliana GN=STP14 PE=2 SV=2//2.89517e-10
XLOC_2035 sp|Q9FPS7|UBP20_ARATH Ubiquitin carboxyl-terminal hydrolase 20 OS=Arabidopsis thaliana GN=UBP20 PE=2 SV=1//2.14868e-1
XLOC_2036 sp|Q8S8M5|ABIL1_ARATH Protein ABIL1 OS=Arabidopsis thaliana GN=ABIL1 PE=1 SV=1//7.61688e-11
XLOC_2037 -//-
XLOC_2038 -//-
XLOC_2038 -//-
XLOC_2039 sp|Q9SZL8|FRS5_ARATH Protein FAR1-RELATED SEQUENCE 5 OS=Arabidopsis thaliana GN=FRS5 PE=2 SV=1//8.68141e-15
XLOC_2039 sp|Q8GW61|STP14_ARATH Sugar transport protein 14 OS=Arabidopsis thaliana GN=STP14 PE=2 SV=2//7.77892e-40
XLOC_2040 sp|P49215|RS17_SOLLC 40S ribosomal protein S17 OS=Solanum lycopersicum GN=RPS17 PE=2 SV=3//2.40726e-36
XLOC_2040 sp|P81391|MYB05_ANTMA Myb-related protein 305 OS=Antirrhinum majus GN=MYB305 PE=2 SV=1//1.80859e-14
XLOC_2040 sp|Q9SXA1|P4KA1_ARATH Phosphatidylinositol 4-kinase alpha OS=Arabidopsis thaliana GN=PI4KALPHA1 PE=1 SV=2//3.08705e-2
XLOC_2041 sp|Q9LFQ3|SNL2_ARATH Paired amphipathic helix protein Sin3-like 2 OS=Arabidopsis thaliana GN=SNL2 PE=1 SV=2//3.01968e
XLOC_2042 sp|Q9LFQ3|SNL2_ARATH Paired amphipathic helix protein Sin3-like 2 OS=Arabidopsis thaliana GN=SNL2 PE=1 SV=2//3.00322e
XLOC_2045 -//-
XLOC_2046 sp|Q9M2D2|YU88_ARATH UPF0187 protein At3g61320, chloroplastic OS=Arabidopsis thaliana GN=At3g61320 PE=2 SV=2//2.13114
XLOC_2046 -//-
XLOC_2047 sp|Q32LE3|CETN1_BOVIN Centrin-1 OS=Bos taurus GN=CETN1 PE=2 SV=1//1.67678e-07
XLOC_2048 sp|Q9T068|EPFL2_ARATH EPIDERMAL PATTERNING FACTOR-like protein 2 OS=Arabidopsis thaliana GN=EPFL2 PE=2 SV=1//1.55424e
XLOC_2048 sp|C0LGT1|Y5129_ARATH Probable LRR receptor-like serine/threonine-protein kinase At5g10290 OS=Arabidopsis thaliana GN
XLOC_2048 sp|C0LGT1|Y5129_ARATH Probable LRR receptor-like serine/threonine-protein kinase At5g10290 OS=Arabidopsis thaliana GN
XLOC_2048 sp|Q9ASU1|DGAT2_ARATH Diacylglycerol O-acyltransferase 2 OS=Arabidopsis thaliana GN=DGAT2 PE=2 SV=1//1.31256e-47
XLOC_2049 sp|Q0IU52|ASP1_ORYSJ Aspartic proteinase Asp1 OS=Oryza sativa subsp. japonica GN=ASP1 PE=2 SV=1//2.37931e-75
XLOC_2049 sp|O81148|PSA4_ARATH Proteasome subunit alpha type-4 OS=Arabidopsis thaliana GN=PAC1 PE=1 SV=1//4.83865e-159
XLOC_2050 sp|Q8H184|PT106_ARATH Probable sugar phosphate/phosphate translocator At1g06470 OS=Arabidopsis thaliana GN=At1g06470
XLOC_2050 sp|Q9FUS9|GSTUI_ARATH Glutathione S-transferase U18 OS=Arabidopsis thaliana GN=GSTU18 PE=2 SV=1//8.30945e-61
XLOC_2050 sp|P37707|B2_DAUCA B2 protein OS=Daucus carota PE=2 SV=1//7.4242e-90
XLOC_2050 -//-
XLOC_2051 -//-

XLOC_2051sp|P29136|MEP1_SOYBN Metalloendoproteinase 1 OS=Glycine max PE=1 SV=2//2.22482e-69
XLOC_2051sp|P54001|P4HA1_RAT Prolyl 4-hydroxylase subunit alpha-1 OS=Rattus norvegicus GN=P4ha1 PE=2 SV=2//1.19058e-29
XLOC_2051sp|Q9LFQ3|SNL2_ARATH Paired amphipathic helix protein Sin3-like 2 OS=Arabidopsis thaliana GN=SNL2 PE=1 SV=2//1.80174e
XLOC_2051sp|Q8GW61|STP14_ARATH Sugar transport protein 14 OS=Arabidopsis thaliana GN=STP14 PE=2 SV=2//3.44088e-14
XLOC_2051sp|O04235|SSRP1_VICFA FACT complex subunit SSRP1 OS=Vicia faba GN=SSRP1 PE=2 SV=1//2.56811e-31
XLOC_2051sp|P47082|AVT1_YEAST Vacuolar amino acid transporter 1 OS=Saccharomyces cerevisiae (strain ATCC 204508 / S288c) GN=AV
XLOC_2051sp|Q93Z27|CML45_ARATH Probable calcium-binding protein CML45 OS=Arabidopsis thaliana GN=CML45 PE=1 SV=1//2.35119e-31
XLOC_2051-//-
XLOC_2051sp|Q9SGA8|U83A1_ARATH UDP-glycosyltransferase 83A1 OS=Arabidopsis thaliana GN=UGT83A1 PE=2 SV=1//7.77322e-135
XLOC_2052sp|Q5R4F4|SC31A_PONAB Protein transport protein Sec31A OS=Pongo abelii GN=SEC31A PE=2 SV=1//2.5651e-09
XLOC_2052-//-
XLOC_2052sp|Q10422|YDC1_SCHPO Uncharacterized RNA-binding protein C25G10.01 OS=Schizosaccharomyces pombe (strain 972 / ATCC 24
XLOC_2052sp|Q94JU3|CSN7_ARATH COP9 signalosome complex subunit 7 OS=Arabidopsis thaliana GN=CSN7 PE=1 SV=1//6.40657e-99
XLOC_2052-//-
XLOC_2052-//-
XLOC_2052sp|A0JP85|CNOT1_XENTR CCR4-NOT transcription complex subunit 1 OS=Xenopus tropicalis GN=cnot1 PE=2 SV=1//2.91458e-20
XLOC_2052sp|Q8GZ38|UNE10_ARATH Transcription factor UNE10 OS=Arabidopsis thaliana GN=UNE10 PE=2 SV=1//3.63985e-99
XLOC_2052-//-
XLOC_2052sp|G5EFL0|GLD4_CAEEL Poly(A) RNA polymerase gld-4 OS=Caenorhabditis elegans GN=gld-4 PE=1 SV=1//3.12067e-07
XLOC_2053sp|Q9SXD5|GSXL3_ARATH Flavin-containing monooxygenase FMO GS-OX-like 3 OS=Arabidopsis thaliana GN=At1g62620 PE=2 SV=2
XLOC_2053-//-
XLOC_2053-//-
XLOC_2053sp|Q9LPC5|BIG3_ARATH Brefeldin A-inhibited guanine nucleotide-exchange protein 3 OS=Arabidopsis thaliana GN=BIG3 PE=1
XLOC_2053sp|Q50EK3|C04C1_PINTA Cytochrome P450 704C1 OS=Pinus taeda GN=CYP704C1 PE=2 SV=1//2.96355e-09
XLOC_2053sp|P93733|PLDB1_ARATH Phospholipase D beta 1 OS=Arabidopsis thaliana GN=PLDBETA1 PE=2 SV=4//1.38808e-153
XLOC_2053sp|P93733|PLDB1_ARATH Phospholipase D beta 1 OS=Arabidopsis thaliana GN=PLDBETA1 PE=2 SV=4//6.86514e-116
XLOC_2053sp|A0MDQ1|URM11_ARATH Ubiquitin-related modifier 1 homolog 1 OS=Arabidopsis thaliana GN=URM1-1 PE=3 SV=2//1.09554e-11
XLOC_2053-//-
XLOC_2053sp|P45432|CSN1_ARATH COP9 signalosome complex subunit 1 OS=Arabidopsis thaliana GN=CSN1 PE=1 SV=2//9.69043e-97
XLOC_2053sp|Q0WV12|ANL2_ARATH Homeobox-leucine zipper protein ANTHOCYANINLESS 2 OS=Arabidopsis thaliana GN=ANL2 PE=2 SV=1//1.2
XLOC_2054sp|B2X050|MNR1_CAPAN (+)-neomenthol dehydrogenase OS=Capsicum annuum GN=MNR1 PE=1 SV=1//2.25333e-44
XLOC_2054sp|P29675|SF3_HELAN Pollen-specific protein SF3 OS=Helianthus annuus GN=SF3 PE=2 SV=1//8.85758e-09
XLOC_2056sp|Q93ZR1|ELP3_ARATH Elongator complex protein 3 OS=Arabidopsis thaliana GN=HAG3 PE=1 SV=1//0
XLOC_2056sp|Q3TZ87|MARH9_MOUSE E3 ubiquitin-protein ligase MARCH9 OS=Mus musculus GN=March9 PE=2 SV=1//5.67145e-07
XLOC_2057-//-

XLOC_2058	sp|P08770|TRA1_MAIZE Putative AC transposase OS=Zea mays PE=2 SV=2//4.45503e-16
XLOC_2058	sp|P18549|CEFD_STRC2 Isopenicillin N epimerase OS=Streptomyces clavuligerus (strain ATCC 27064 / DSM 738 / JCM 4710 /
XLOC_2058	sp|Q9S6Z8|TPK5_ARATH Two-pore potassium channel 5 OS=Arabidopsis thaliana GN=TPK5 PE=1 SV=1//4.43246e-144
XLOC_2059	-//-
XLOC_2059	sp|Q9FZ29|MAN1_ARATH Mannan endo-1,4-beta-mannosidase 1 OS=Arabidopsis thaliana GN=MAN1 PE=2 SV=1//2.21269e-94
XLOC_2059	sp|P81391|MYB05_ANTMA Myb-related protein 305 OS=Antirrhinum majus GN=MYB305 PE=2 SV=1//5.66365e-22
XLOC_2059	sp|Q94A16|CP21C_ARATH Peptidyl-prolyl cis-trans isomerase CYP21-3, mitochondrial OS=Arabidopsis thaliana GN=CYP21-3 P
XLOC_2060	sp|Q9C7F5|NTF2_ARATH Nuclear transport factor 2 OS=Arabidopsis thaliana GN=NTF2 PE=2 SV=1//7.17821e-24
XLOC_2061	sp|P11369|POL2_MOUSE Retrovirus-related Pol polyprotein LINE-1 OS=Mus musculus GN=Pol PE=1 SV=2//5.62931e-37
XLOC_2062	-//-
XLOC_2063	-//-
XLOC_2066	sp|Q9C0R2|RTF22_SCHPO Retrotransposable element Tf2 155 kDa protein type 2 OS=Schizosaccharomyces pombe (strain 972 /
XLOC_2066	sp|Q84P52|GATP3_SOLLC Gamma aminobutyrate transaminase 3, chloroplastic OS=Solanum lycopersicum GN=GABA-TP3 PE=1 SV=1
XLOC_2071	sp|Q94K01|DBR1_ARATH Lariat debranching enzyme OS=Arabidopsis thaliana GN=DBR1 PE=2 SV=1//6.47005e-16
XLOC_2071	sp|P08770|TRA1_MAIZE Putative AC transposase OS=Zea mays PE=2 SV=2//8.36233e-69
XLOC_2073	-//-
XLOC_2076	sp|Q9LVQ8|P2C80_ARATH Probable protein phosphatase 2C 80 OS=Arabidopsis thaliana GN=At5g66720 PE=2 SV=1//3.29044e-51
XLOC_2079	sp|Q8S4P6|EZ1_MAIZE Histone-lysine N-methyltransferase EZ1 OS=Zea mays GN=EZ1 PE=2 SV=1//2.802e-20
XLOC_2082	sp|Q0J3L4|GRS10_ORYSJ Monothiol glutaredoxin-S10 OS=Oryza sativa subsp. japonica GN=GRXS10 PE=2 SV=2//1.68273e-11
XLOC_2084	-//-
XLOC_2084	sp|Q75LR7|SAPK1_ORYSJ Serine/threonine-protein kinase SAPK1 OS=Oryza sativa subsp. japonica GN=SAPK1 PE=1 SV=1//5.191
XLOC_2087	sp|Q9LIH7|ZDHC7_ARATH Probable S-acyltransferase At3g18620 OS=Arabidopsis thaliana GN=At3g18620 PE=2 SV=1//1.97285e-1
XLOC_2088	sp|Q8GX29|SKI25_ARATH F-box/kelch-repeat protein SKIP25 OS=Arabidopsis thaliana GN=SKIP25 PE=1 SV=1//1.78254e-88
XLOC_2088	sp|O22808|LYK5_ARATH Protein LYK5 OS=Arabidopsis thaliana GN=LYK5 PE=1 SV=1//1.46783e-07
XLOC_2090	sp|Q9ZNT1|NB5R1_ARATH NADH--cytochrome b5 reductase 1 OS=Arabidopsis thaliana GN=CBR1 PE=1 SV=1//5.64872e-15
XLOC_2091	-//-
XLOC_2094	-//-
XLOC_2095	sp|Q39237|TGA1_ARATH Transcription factor TGA1 OS=Arabidopsis thaliana GN=TGA1 PE=1 SV=2//6.67412e-80
XLOC_2095	sp|P0C605|KGP1_MOUSE cGMP-dependent protein kinase 1 OS=Mus musculus GN=Prkg1 PE=1 SV=1//3.09184e-08
XLOC_2095	sp|Q0WMJ8|SIPL4_ARATH Signal peptide peptidase-like 4 OS=Arabidopsis thaliana GN=SPPL4 PE=2 SV=1//5.04256e-17
XLOC_2097	-//-
XLOC_2098	sp|Q2R8Z5|ADH1_ORYSJ Alcohol dehydrogenase 1 OS=Oryza sativa subsp. japonica GN=ADH1 PE=2 SV=2//4.34473e-117
XLOC_2098	sp|Q94502|GANAB_DICDI Neutral alpha-glucosidase AB OS=Dictyostelium discoideum GN=modA PE=3 SV=1//1.92054e-43
XLOC_2099	-//-
XLOC_2099	-//-

XLOC_2099 sp|Q03200|LIRP1_ORYSJ Light-regulated protein OS=Oryza sativa subsp. japonica GN=LIR1 PE=2 SV=1//6.66745e-20
XLOC_2099 -//-
XLOC_2099 sp|Q9CX11|UTP23_MOUSE rRNA-processing protein UTP23 homolog OS=Mus musculus GN=Utp23 PE=2 SV=1//9.57413e-26
XLOC_2099 sp|B2RXR6|ANR44_MOUSE Serine/threonine-protein phosphatase 6 regulatory ankyrin repeat subunit B OS=Mus musculus GN=A
XLOC_2099 sp|P57758|CTNS_ARATH Cystinosin homolog OS=Arabidopsis thaliana GN=At5g40670 PE=2 SV=1//5.51639e-35
XLOC_2099 -//-
XLOC_2100 sp|Q9FL12|DEGP9_ARATH Protease Do-like 9 OS=Arabidopsis thaliana GN=DEGP9 PE=2 SV=1//3.96958e-178
XLOC_2100 sp|P29136|MEP1_SOYBN Metalloendoproteinase 1 OS=Glycine max PE=1 SV=2//5.98846e-75
XLOC_2100 sp|P92958|KIN11_ARATH SNF1-related protein kinase catalytic subunit alpha KIN11 OS=Arabidopsis thaliana GN=KIN11 PE=1
XLOC_2101 sp|Q8RY89|PI5K8_ARATH Phosphatidylinositol 4-phosphate 5-kinase 8 OS=Arabidopsis thaliana GN=PIP5K8 PE=2 SV=1//6.1768
XLOC_2101 -//-
XLOC_2101 sp|Q5XF59|HIBC4_ARATH 3-hydroxyisobutyryl-CoA hydrolase-like protein 1, mitochondrial OS=Arabidopsis thaliana GN=At3g
XLOC_2101 sp|Q7XT42|SPL7_ORYSJ Squamosa promoter-binding-like protein 7 OS=Oryza sativa subsp. japonica GN=SPL7 PE=2 SV=2//1.34
XLOC_2101 -//-
XLOC_2101 sp|O22152|YAB1_ARATH Axial regulator YABBY 1 OS=Arabidopsis thaliana GN=YAB1 PE=1 SV=1//3.56834e-43
XLOC_2102 -//-
XLOC_2102 sp|Q50EK3|C04C1_PINTA Cytochrome P450 704C1 OS=Pinus taeda GN=CYP704C1 PE=2 SV=1//6.64489e-09
XLOC_2102 -//-
XLOC_2102 -//-
XLOC_2102 -//-
XLOC_2102 -//-
XLOC_2102 sp|O64647|TCP9_ARATH Transcription factor TCP9 OS=Arabidopsis thaliana GN=TCP9 PE=2 SV=1//4.14162e-68
XLOC_2103 sp|Q6DW75|DGDG2_SOYBN Digalactosyldiacylglycerol synthase 2, chloroplastic OS=Glycine max GN=DGD2 PE=2 SV=1//4.3066e-
XLOC_2103 sp|Q6H5X0|RIP2_ORYSJ Putative ripening-related protein 2 OS=Oryza sativa subsp. japonica GN=Os02g0637000 PE=3 SV=1//5
XLOC_2105 -//-
XLOC_2105 -//-
XLOC_2108 sp|P52855|RLA1_MAIZE 60S acidic ribosomal protein P1 OS=Zea mays GN=RPP1A PE=1 SV=1//3.24025e-11
XLOC_2108 -//-
XLOC_2108 sp|Q9SIC9|PP178_ARATH Pentatricopeptide repeat-containing protein At2g31400, chloroplastic OS=Arabidopsis thaliana GN
XLOC_2108 sp|O64747|WRK35_ARATH Probable WRKY transcription factor 35 OS=Arabidopsis thaliana GN=WRKY35 PE=2 SV=1//5.56493e-31
XLOC_2108 -//-
XLOC_2108 sp|P93295|M310_ARATH Uncharacterized mitochondrial protein AtMg00310 OS=Arabidopsis thaliana GN=AtMg00310 PE=4 SV=1//
XLOC_2110 sp|Q8L735|ISOA2_ARATH Isoamylase 2, chloroplastic OS=Arabidopsis thaliana GN=ISA2 PE=1 SV=2//0
XLOC_2112 sp|Q3SWX2|ACOT9_BOVIN Acyl-coenzyme A thioesterase 9, mitochondrial OS=Bos taurus GN=ACOT9 PE=2 SV=1//1.0783e-35
XLOC_2113 sp|Q09020|PR4_PHAVU Wound-induced basic protein OS=Phaseolus vulgaris GN=PR4 PE=2 SV=1//6.71167e-22

XLOC_2116 sp|Q8W1X2|PDXK_ARATH Pyridoxal kinase OS=Arabidopsis thaliana GN=PK PE=1 SV=2//6.8751e-32

XLOC_2117 sp|P23919|KTHY_HUMAN Thymidylate kinase OS=Homo sapiens GN=DTYMK PE=1 SV=4//4.52699e-54

XLOC_2123 -//-

XLOC_2124 -//-

XLOC_2124 sp|P10978|POLX_TOBAC Retrovirus-related Pol polyprotein from transposon TNT 1-94 OS=Nicotiana tabacum PE=2 SV=1//1.05

XLOC_2124 -//-

XLOC_2125 -//-

XLOC_2125 -//-

XLOC_2128 -//-

XLOC_2128 sp|P0C2F6|RNHX1_ARATH Putative ribonuclease H protein At1g65750 OS=Arabidopsis thaliana GN=At1g65750 PE=3 SV=1//1.013

XLOC_2129 sp|Q94B08|GCP1_ARATH Germination-specific cysteine protease 1 OS=Arabidopsis thaliana GN=GCP1 PE=2 SV=2//2.41904e-56

XLOC_2129 -//-

XLOC_2130 -//-

XLOC_2132 sp|Q5RC69|DCTD_PONAB Deoxycytidylate deaminase OS=Pongo abelii GN=DCTD PE=2 SV=1//2.65944e-60

XLOC_2134 -//-

XLOC_2134 sp|F4HVJ3|POD1_ARATH Protein POLLEN DEFECTIVE IN GUIDANCE 1 OS=Arabidopsis thaliana GN=POD1 PE=1 SV=1//1.86707e-63

XLOC_2134 -//-

XLOC_2135 sp|P10978|POLX_TOBAC Retrovirus-related Pol polyprotein from transposon TNT 1-94 OS=Nicotiana tabacum PE=2 SV=1//5.49

XLOC_2136 sp|Q05654|RTF21_SCHPO Retrotransposable element Tf2 155 kDa protein type 1 OS=Schizosaccharomyces pombe (strain 972 /

XLOC_2137 -//-

XLOC_2138 sp|P46440|GSTF2_TOBAC Glutathione S-transferase APIC OS=Nicotiana tabacum GN=APIC PE=2 SV=1//2.48839e-18

XLOC_2139 sp|Q9FN09|NPY3_ARATH BTB/POZ domain-containing protein NPY3 OS=Arabidopsis thaliana GN=NPY3 PE=2 SV=1//6.68925e-13

XLOC_2142 sp|Q5RET6|T184C_PONAB Transmembrane protein 184C OS=Pongo abelii GN=TMEM184C PE=2 SV=1//1.32677e-21

XLOC_2144 -//-

XLOC_2151 -//-

XLOC_2153 sp|Q42551|SCE1_ARATH SUMO-conjugating enzyme SCE1 OS=Arabidopsis thaliana GN=SCE1 PE=1 SV=1//8.22903e-06

XLOC_2154 sp|Q9FJP9|DHSB3_ARATH Succinate dehydrogenase [ubiquinone] iron-sulfur subunit 3, mitochondrial OS=Arabidopsis thalia

XLOC_2155 sp|P08770|TRA1_MAIZE Putative AC transposase OS=Zea mays PE=2 SV=2//5.73268e-39

XLOC_2156 sp|P10978|POLX_TOBAC Retrovirus-related Pol polyprotein from transposon TNT 1-94 OS=Nicotiana tabacum PE=2 SV=1//1.12

XLOC_2156 -//-

XLOC_2158 -//-

XLOC_2161 sp|Q9ASX5|Y5520_ARATH Uncharacterized aarF domain-containing protein kinase At5g05200, chloroplastic OS=Arabidopsis t

XLOC_2161 sp|F4IZC4|BASS4_ARATH Probable sodium/metabolite cotransporter BASS4, chloroplastic OS=Arabidopsis thaliana GN=BASS4

XLOC_2164 -//-

XLOC_2164 sp|O81062|SIP_ARATH Signal peptide peptidase OS=Arabidopsis thaliana GN=SPP PE=2 SV=1//5.8481e-12

XLOC_2165 -//-
XLOC_2165 sp|Q8L5Y5|ZDH17_ARATH Probable S-acyltransferase At4g15080 OS=Arabidopsis thaliana GN=At4g15080 PE=1 SV=1//0
XLOC_2166 -//-
XLOC_2167 -//-
XLOC_2168 sp|Q9LZI6|NDS5B_ARATH NADH dehydrogenase [ubiquinone] iron-sulfur protein 5-B OS=Arabidopsis thaliana GN=At3g62790 PE
XLOC_2168 sp|Q9SRX9|BAH1_ARATH E3 ubiquitin-protein ligase BAH1 OS=Arabidopsis thaliana GN=BAH1 PE=1 SV=1//3.11638e-123
XLOC_2169 -//-
XLOC_2171 sp|P10978|POLX_TOBAC Retrovirus-related Pol polyprotein from transposon TNT 1-94 OS=Nicotiana tabacum PE=2 SV=1//1.26
XLOC_2174 sp|P08548|LIN1_NYCCO LINE-1 reverse transcriptase homolog OS=Nycticebus coucang PE=1 SV=1//3.6051e-17
XLOC_2174 sp|Q6Z1D5|ATG8C_ORYSJ Autophagy-related protein 8C OS=Oryza sativa subsp. japonica GN=ATG8C PE=2 SV=1//1.72857e-08
XLOC_2175 sp|Q6R8G7|PHO13_ARATH Phosphate transporter PHO1 homolog 3 OS=Arabidopsis thaliana GN=PHO1;H3 PE=2 SV=2//0
XLOC_2176 -//-
XLOC_2177 sp|Q93ZF5|PHO11_ARATH Phosphate transporter PHO1 homolog 1 OS=Arabidopsis thaliana GN=PHO1-H1 PE=2 SV=1//0
XLOC_2178 sp|Q43472|GRP_HORVU Glycine-rich RNA-binding protein blt801 OS=Hordeum vulgare GN=blt801 PE=1 SV=1//1.48164e-22
XLOC_2180 sp|P10978|POLX_TOBAC Retrovirus-related Pol polyprotein from transposon TNT 1-94 OS=Nicotiana tabacum PE=2 SV=1//2.20
XLOC_2181 -//-
XLOC_2182 sp|O95677|EYA4_HUMAN Eyes absent homolog 4 OS=Homo sapiens GN=EYA4 PE=1 SV=2//5.57386e-41
XLOC_2182 -//-
XLOC_2183 sp|Q9ZQG4|U73B5_ARATH UDP-glycosyltransferase 73B5 OS=Arabidopsis thaliana GN=UGT73B5 PE=2 SV=1//6.26288e-42
XLOC_2183 sp|P03876|AI2M_YEAST Putative COX1/OXI3 intron 2 protein OS=Saccharomyces cerevisiae (strain ATCC 204508 / S288c) GN=
XLOC_2184 sp|Q07G10|ALKB8_XENTR Alkylated DNA repair protein alkB homolog 8 OS=Xenopus tropicalis GN=alkbh8 PE=2 SV=2//6.93856e
XLOC_2184 -//-
XLOC_2184 -//-
XLOC_2186 sp|Q69IL4|RF2A_ORYSJ Transcription factor RF2a OS=Oryza sativa subsp. japonica GN=RF2a PE=1 SV=1//1.06147e-33
XLOC_2187 sp|Q9FPW6|POB1_ARATH BTB/POZ domain-containing protein POB1 OS=Arabidopsis thaliana GN=POB1 PE=2 SV=2//4.57919e-16
XLOC_2190 sp|Q14974|IMB1_HUMAN Importin subunit beta-1 OS=Homo sapiens GN=KPNB1 PE=1 SV=2//9.68868e-61
XLOC_2192 sp|Q86V88|MGDP1_HUMAN Magnesium-dependent phosphatase 1 OS=Homo sapiens GN=MDP1 PE=1 SV=1//9.99249e-31
XLOC_2194 -//-
XLOC_2194 -//-
XLOC_2196 sp|Q680H3|PP170_ARATH Pentatricopeptide repeat-containing protein At2g25580 OS=Arabidopsis thaliana GN=PCMP-H75 PE=2
XLOC_2197 sp|Q5Z627|EF1G3_ORYSJ Elongation factor 1-gamma 3 OS=Oryza sativa subsp. japonica GN=Os06g0571400 PE=2 SV=1//8.31013e
XLOC_2200 sp|P84752|PERA_ALOVR Peroxidase A (Fragments) OS=Aloe vera PE=1 SV=1//3.94775e-08
XLOC_2201 -//-
XLOC_2202 -//-
XLOC_2204 sp|O22941|PXM16_ARATH Zinc-metallopeptidase, peroxisomal OS=Arabidopsis thaliana GN=PXM16 PE=2 SV=1//5.52059e-35

XLOC_2204-//-
XLOC_2207sp|P10978|POLX_TOBAC Retrovirus-related Pol polyprotein from transposon TNT 1-94 OS=Nicotiana tabacum PE=2 SV=1//7.00
XLOC_2207sp|P10978|POLX_TOBAC Retrovirus-related Pol polyprotein from transposon TNT 1-94 OS=Nicotiana tabacum PE=2 SV=1//3.96
XLOC_2209sp|Q76E23|IF4G_ARATH Eukaryotic translation initiation factor 4G OS=Arabidopsis thaliana GN=EIF4G PE=1 SV=2//1.94142e
XLOC_2210-//-
XLOC_2210sp|O80739|POT12_ARATH Putative potassium transporter 12 OS=Arabidopsis thaliana GN=POT12 PE=1 SV=2//1.3399e-34
XLOC_2210sp|Q96361|ARF1_BRARP ADP-ribosylation factor 1 OS=Brassica rapa subsp. pekinensis GN=ARF1 PE=2 SV=3//5.83193e-12
XLOC_2211-//-
XLOC_2213-//-
XLOC_2213-//-
XLOC_2213-//-
XLOC_2215sp|Q9M886|LBD41_ARATH LOB domain-containing protein 41 OS=Arabidopsis thaliana GN=LBD41 PE=2 SV=1//3.38103e-94
XLOC_2216-//-
XLOC_2216sp|Q0DJA3|DRB3_ORYSJ Double-stranded RNA-binding protein 3 OS=Oryza sativa subsp. japonica GN=DRB3 PE=2 SV=1//4.02776
XLOC_2217sp|P33079|A10A5_SOYBN Auxin-induced protein 10A5 OS=Glycine max PE=2 SV=1//1.19751e-11
XLOC_2217-//-
XLOC_2217sp|P92973|CCA1_ARATH Protein CCA1 OS=Arabidopsis thaliana GN=CCA1 PE=1 SV=1//1.28968e-22
XLOC_2218sp|O34598|GUAD_BACSU Guanine deaminase OS=Bacillus subtilis (strain 168) GN=guaD PE=1 SV=1//2.37189e-07
XLOC_2221sp|Q9ZRU9|LSM4_FAGSY Probable U6 snRNA-associated Sm-like protein LSm4 OS=Fagus sylvatica GN=LSM4 PE=2 SV=1//1.57891e
XLOC_2221sp|O81062|SIP_ARATH Signal peptide peptidase OS=Arabidopsis thaliana GN=SPP PE=2 SV=1//1.00241e-12
XLOC_2223-//-
XLOC_2227sp|O67716|DHAS_AQUAE Aspartate-semialdehyde dehydrogenase OS=Aquifex aeolicus (strain VF5) GN=asd PE=3 SV=1//2.93995e
XLOC_2229sp|Q8H157|PTR19_ARATH Nitrate transporter 1.2 OS=Arabidopsis thaliana GN=NRT1.2 PE=1 SV=1//3.90833e-14
XLOC_2229sp|Q9LS01|AOC3_ARATH Allene oxide cyclase 3, chloroplastic OS=Arabidopsis thaliana GN=AOC3 PE=2 SV=1//8.78019e-82
XLOC_2231sp|Q96361|ARF1_BRARP ADP-ribosylation factor 1 OS=Brassica rapa subsp. pekinensis GN=ARF1 PE=2 SV=3//2.78944e-06
XLOC_2231-//-
XLOC_2231sp|F1NTD6|ASCC3_CHICK Activating signal cointegrator 1 complex subunit 3 OS=Gallus gallus GN=ascc3 PE=3 SV=2//3.11155
XLOC_2232-//-
XLOC_2232sp|Q0WWT7|STR11_ARATH Rhodanese-like domain-containing protein 11, chloroplastic OS=Arabidopsis thaliana GN=STR11 PE=
XLOC_2232sp|O65039|CYSEP_RICCO Vignain OS=Ricinus communis GN=CYSEP PE=1 SV=1//2.38321e-58
XLOC_2233sp|Q2V6J9|UFOG7_FRAAN UDP-glucose flavonoid 3-O-glucosyltransferase 7 OS=Fragaria ananassa GN=GT7 PE=1 SV=1//2.00095e
XLOC_2236sp|Q5F4C4|SHOC2_CHICK Leucine-rich repeat protein SHOC-2 OS=Gallus gallus GN=SHOC2 PE=2 SV=1//3.81238e-11
XLOC_2236sp|Q9UTE6|MAK16_SCHPO Protein mak16 OS=Schizosaccharomyces pombe (strain 972 / ATCC 24843) GN=mak16 PE=2 SV=1//9.9845
XLOC_2238sp|B8JMH0|CAF17_DANRE Putative transferase CAF17 homolog, mitochondrial OS=Danio rerio GN=iba57 PE=3 SV=1//3.23313e-4
XLOC_2238sp|Q55720|YC49L_SYNY3 Ycf49-like protein OS=Synechocystis sp. (strain PCC 6803 / Kazusa) GN=sll0608 PE=3 SV=1//5.5324

XLOC_2239 -//-
XLOC_2240 sp|Q9FGM1|PYL8_ARATH Abscisic acid receptor PYL8 OS=Arabidopsis thaliana GN=PYL8 PE=1 SV=1//1.97761e-97
XLOC_2241 sp|Q339W7|LHP1_ORYSJ Probable chromo domain-containing protein LHP1 OS=Oryza sativa subsp. japonica GN=LHP1 PE=3 SV=1
XLOC_2241 -//-
XLOC_2241 -//-
XLOC_2243 sp|A2SY66|VICHY_VICSN Vicianin hydrolase (Fragment) OS=Vicia sativa subsp. nigra PE=1 SV=1//1.99483e-22
XLOC_2244 -//-
XLOC_2248 sp|F4I933|STR8_ARATH Rhodanese-like domain-containing protein 8, chloroplastic OS=Arabidopsis thaliana GN=STR8 PE=2 S
XLOC_2248 -//-
XLOC_2248 sp|Q9FMA3|PEX5_ARATH Peroxisome biogenesis protein 5 OS=Arabidopsis thaliana GN=PEX5 PE=1 SV=1//1.44435e-19
XLOC_2250 -//-
XLOC_2250 sp|Q9C9H7|RLP12_ARATH Receptor-like protein 12 OS=Arabidopsis thaliana GN=RLP12 PE=2 SV=2//1.12206e-10
XLOC_2253 -//-
XLOC_2254 sp|O82318|Y2579_ARATH Probably inactive leucine-rich repeat receptor-like protein kinase At2g25790 OS=Arabidopsis tha
XLOC_2254 sp|P93293|M300_ARATH Uncharacterized mitochondrial protein AtMg00300 OS=Arabidopsis thaliana GN=AtMg00300 PE=4 SV=1//
XLOC_2259 sp|Q5R9C7|SC11A_PONAB Signal peptidase complex catalytic subunit SEC11A OS=Pongo abelii GN=SEC11A PE=2 SV=1//3.2712e-
XLOC_2263 sp|Q3ZBU9|UBXN4_BOVIN UBX domain-containing protein 4 OS=Bos taurus GN=UBXN4 PE=2 SV=2//3.26873e-08
XLOC_2263 sp|Q54ZW0|PHG1B_DICDI Putative phagocytic receptor 1b OS=Dictyostelium discoideum GN=phg1b PE=2 SV=1//2.72004e-13
XLOC_2264 -//-
XLOC_2264 -//-
XLOC_2264 sp|Q550W1|CAPTC_DICDI Uncharacterized CDP-alcohol phosphatidyltransferase class-I family protein 3 OS=Dictyostelium d
XLOC_2264 sp|Q9LFS4|NIK1_ARATH Protein NSP-INTERACTING KINASE 1 OS=Arabidopsis thaliana GN=NIK1 PE=1 SV=1//4.9471e-50
XLOC_2264 sp|Q9CAA4|BIM2_ARATH Transcription factor BIM2 OS=Arabidopsis thaliana GN=BIM2 PE=1 SV=1//9.67355e-18
XLOC_2264 sp|Q5CZL1|GDAP2_XENTR Ganglioside-induced differentiation-associated protein 2 OS=Xenopus tropicalis PE=2 SV=1//2.293
XLOC_2264 sp|Q6R8G7|PHO13_ARATH Phosphate transporter PHO1 homolog 3 OS=Arabidopsis thaliana GN=PHO1;H3 PE=2 SV=2//4.08983e-12
XLOC_2266 -//-
XLOC_2268 -//-
XLOC_2268 sp|Q8L736|SKI11_ARATH F-box/kelch-repeat protein SKIP11 OS=Arabidopsis thaliana GN=SKIP11 PE=1 SV=2//2.36796e-172
XLOC_2274 sp|Q54P70|Y4757_DICDI OTU domain-containing protein DDB_G0284757 OS=Dictyostelium discoideum GN=DDB_G0284757 PE=4 SV=
XLOC_2275 -//-
XLOC_2277 sp|Q9LN77|P2A12_ARATH F-box protein PP2-A12 OS=Arabidopsis thaliana GN=P2A12 PE=2 SV=1//3.35512e-84
XLOC_2279 sp|Q9T096|YIPL6_ARATH Protein yippee-like At4g27745 OS=Arabidopsis thaliana GN=At4g27745 PE=3 SV=2//3.02372e-59
XLOC_2280 sp|Q9STN8|SINA4_ARATH E3 ubiquitin-protein ligase SINAT4 OS=Arabidopsis thaliana GN=SINAT4 PE=2 SV=1//0
XLOC_2280 sp|Q6PBM8|RBM18_DANRE Probable RNA-binding protein 18 OS=Danio rerio GN=rbm18 PE=2 SV=1//1.78507e-12
XLOC_2281 sp|P08770|TRA1_MAIZE Putative AC transposase OS=Zea mays PE=2 SV=2//5.59968e-29

XLOC_2285 -//-
XLOC_2288 sp|Q9SB51|UBP16_ARATH Ubiquitin carboxyl-terminal hydrolase 16 OS=Arabidopsis thaliana GN=UBP16 PE=2 SV=1//7.84308e-3
XLOC_2289 sp|Q55CU8|RSC5_DICDI Random slug protein 5 OS=Dictyostelium discoideum GN=rsc5 PE=2 SV=1//4.81514e-08
XLOC_2289 sp|Q8VYI9|NLTL5_ARATH Non-specific lipid-transfer protein-like protein At5g64080 OS=Arabidopsis thaliana GN=At5g6408C
XLOC_2289 -//-
XLOC_2289 -//-
XLOC_2289 -//-
XLOC_2289 -//-
XLOC_2289 sp|Q71H73|H33_VITVI Histone H3.3 OS=Vitis vinifera PE=2 SV=3//2.88196e-86
XLOC_2289 sp|A2YNT8|SAPK2_ORYSI Serine/threonine-protein kinase SAPK2 OS=Oryza sativa subsp. indica GN=SAPK2 PE=2 SV=2//5.0895e
XLOC_2289 sp|Q9ZWJ3|U85A2_ARATH UDP-glycosyltransferase 85A2 OS=Arabidopsis thaliana GN=UGT85A2 PE=2 SV=1//0
XLOC_2290 sp|Q766C2|NEP2_NEPGR Aspartic proteinase nepenthesin-2 OS=Nepenthes gracilis GN=nep2 PE=1 SV=1//6.95818e-26
XLOC_2292 sp|Q56YP2|PI5K1_ARATH Phosphatidylinositol 4-phosphate 5-kinase 1 OS=Arabidopsis thaliana GN=PIP5K1 PE=1 SV=1//8.0824
XLOC_2294 sp|Q9FFN2|GLYT3_ARATH Probable glycosyltransferase At5g03795 OS=Arabidopsis thaliana GN=At5g03795 PE=3 SV=2//6.40781e
XLOC_2294 sp|P10978|POLX_TOBAC Retrovirus-related Pol polyprotein from transposon TNT 1-94 OS=Nicotiana tabacum PE=2 SV=1//6.96
XLOC_2295 sp|Q9STG6|DUT_ARATH Deoxyuridine 5'-triphosphate nucleotidohydrolase OS=Arabidopsis thaliana GN=DUT PE=1 SV=1//3
XLOC_2296 -//-
XLOC_2296 sp|Q8L8B8|LOG3_ARATH Cytokinin riboside 5'-monophosphate phosphoribohydrolase LOG3 OS=Arabidopsis thaliana GN=LC
XLOC_2296 -//-
XLOC_2299 -//-
XLOC_2302 -//-
XLOC_2303 sp|Q9BTZ2|DHRS4_HUMAN Dehydrogenase/reductase SDR family member 4 OS=Homo sapiens GN=DHRS4 PE=1 SV=3//1.06923e-07
XLOC_2303 sp|P54260|GCST_SOLTU Aminomethyltransferase, mitochondrial OS=Solanum tuberosum GN=GDCST PE=2 SV=1//0
XLOC_2303 sp|P12301|PSBQ_SPIOL Oxygen-evolving enhancer protein 3, chloroplastic OS=Spinacia oleracea GN=PSBQ PE=1 SV=1//7.7721
XLOC_2304 sp|Q1RHN1|RL14_RICBR 50S ribosomal protein L14 OS=Rickettsia bellii (strain RML369-C) GN=rplN PE=3 SV=1//5.30152e-32
XLOC_2304 sp|Q94F00|IMPL1_ARATH Phosphatase IMPL1, chloroplastic OS=Arabidopsis thaliana GN=IMPL1 PE=1 SV=2//4.42199e-39
XLOC_2305 sp|Q9SYG1|HS174_ARATH 17.4 kDa class III heat shock protein OS=Arabidopsis thaliana GN=HSP17.4B PE=2 SV=1//4.87275e-3
XLOC_2305 sp|Q93ZH2|NFYA3_ARATH Nuclear transcription factor Y subunit A-3 OS=Arabidopsis thaliana GN=NFYA3 PE=2 SV=2//1.90073e
XLOC_2306 -//-
XLOC_2308 sp|Q53YF3|SPC3B_ARATH Signal peptidase complex subunit 3B OS=Arabidopsis thaliana GN=At5g27430 PE=2 SV=1//3.64863e-96
XLOC_2309 -//-
XLOC_2309 sp|Q6VAF7|TBB5_GOSHI Tubulin beta-5 chain OS=Gossypium hirsutum PE=2 SV=1//0
XLOC_2310 -//-
XLOC_2312 sp|F4I171|MD15A_ARATH Mediator of RNA polymerase II transcription subunit 15a OS=Arabidopsis thaliana GN=MED15A PE=1
XLOC_2312 sp|Q9SSQ4|FRS6_ARATH Protein FAR1-RELATED SEQUENCE 6 OS=Arabidopsis thaliana GN=FRS6 PE=2 SV=1//0

XLOC_2313	sp|P09189|HSP7C_PETHY Heat shock cognate 70 kDa protein OS=Petunia hybrida GN=HSP70 PE=2 SV=1//5.99022e-161
XLOC_2316	sp|Q6NQJ7|FRS4_ARATH Protein FAR1-RELATED SEQUENCE 4 OS=Arabidopsis thaliana GN=FRS4 PE=2 SV=2//0
XLOC_2316	sp|Q28BZ1|RBM8A_XENTR RNA-binding protein 8A OS=Xenopus tropicalis GN=rbm8a PE=2 SV=1//4.3782e-32
XLOC_2317	sp|Q8LAW2|AFR_ARATH F-box protein AFR OS=Arabidopsis thaliana GN=AFR PE=1 SV=2//6.26488e-136
XLOC_2318	-//-
XLOC_2319	sp|P49212|RL37_SOLLC 60S ribosomal protein L37 (Fragment) OS=Solanum lycopersicum GN=RPL37 PE=3 SV=1//5.60419e-19
XLOC_2320	sp|Q84KI6|SQD1_SPIOL UDP-sulfoquinovose synthase, chloroplastic OS=Spinacia oleracea GN=SQD1 PE=1 SV=1//0
XLOC_2322	sp|Q8RWQ9|ALEUL_ARATH Thiol protease aleurain-like OS=Arabidopsis thaliana GN=At3g45310 PE=2 SV=1//1.36139e-141
XLOC_2324	-//-
XLOC_2326	sp|Q5EAR5|TRPT1_DANRE tRNA 2'-phosphotransferase 1 OS=Danio rerio GN=trpt1 PE=2 SV=2//2.81323e-17
XLOC_2327	-//-
XLOC_2331	sp|P35135|UBC4_SOLLC Ubiquitin-conjugating enzyme E2-17 kDa OS=Solanum lycopersicum PE=2 SV=1//2.0912e-17
XLOC_2332	sp|Q8LPB4|PSKR1_DAUCA Phytosulfokine receptor 1 OS=Daucus carota GN=PSKR PE=1 SV=1//4.24168e-10
XLOC_2333	sp|Q71N54|LCAT4_ARATH Lecithine-cholesterol acyltransferase-like 4 OS=Arabidopsis thaliana GN=LCAT4 PE=2 SV=1//3.7269
XLOC_2333	sp|P0CH33|UBQ11_ARATH Polyubiquitin 11 OS=Arabidopsis thaliana GN=UBQ11 PE=1 SV=1//1.25042e-104
XLOC_2333	sp|P0CG71|UBIQ1_CAEEL Polyubiquitin-A OS=Caenorhabditis elegans GN=ubq-1 PE=3 SV=1//0
XLOC_2335	-//-
XLOC_2335	sp|Q8L6Y1|UBP14_ARATH Ubiquitin carboxyl-terminal hydrolase 14 OS=Arabidopsis thaliana GN=UBP14 PE=1 SV=1//3.70706e-1
XLOC_2339	sp|Q9SYL0|BT3_ARATH BTB/POZ and TAZ domain-containing protein 3 OS=Arabidopsis thaliana GN=BT3 PE=1 SV=2//2.01288e-21
XLOC_2340	sp|P92519|M810_ARATH Uncharacterized mitochondrial protein AtMg00810 OS=Arabidopsis thaliana GN=AtMg00810 PE=4 SV=1//
XLOC_2341	sp|Q8L6Y1|UBP14_ARATH Ubiquitin carboxyl-terminal hydrolase 14 OS=Arabidopsis thaliana GN=UBP14 PE=1 SV=1//1.07171e-1
XLOC_2342	sp|P10978|POLX_TOBAC Retrovirus-related Pol polyprotein from transposon TNT 1-94 OS=Nicotiana tabacum PE=2 SV=1//4.18
XLOC_2343	-//-
XLOC_2343	sp|Q923G2|RPAB3_MOUSE DNA-directed RNA polymerases I, II, and III subunit RPABC3 OS=Mus musculus GN=Polr2h PE=2 SV=3/
XLOC_2345	-//-
XLOC_2346	-//-
XLOC_2347	-//-
XLOC_2348	-//-
XLOC_2348	-//-
XLOC_2349	-//-
XLOC_2349	-//-
XLOC_2350	sp|Q54UU1|TPPC4_DICDI Trafficking protein particle complex subunit 4 OS=Dictyostelium discoideum GN=trappc4 PE=3 SV=1
XLOC_2350	sp|Q6R8G7|PHO13_ARATH Phosphate transporter PHO1 homolog 3 OS=Arabidopsis thaliana GN=PHO1;H3 PE=2 SV=2//3.589e-64
XLOC_2352	sp|Q940Z2|ARP5_ARATH Actin-related protein 5 OS=Arabidopsis thaliana GN=ARP5 PE=1 SV=2//1.33874e-62
XLOC_2358	-//-

XLOC_2361 sp|P49636|RL40_NICSY Ubiquitin-60S ribosomal protein L40 OS=Nicotiana sylvestris GN=UBICEP52-7 PE=2 SV=2//2.10125e-86
XLOC_2361 sp|Q9FYR7|SCL8_ARATH Scarecrow-like protein 8 OS=Arabidopsis thaliana GN=SCL8 PE=2 SV=1//1.49692e-20
XLOC_2363 -//-
XLOC_2366 -//-
XLOC_2367 sp|O23712|PSA1B_ARATH Proteasome subunit alpha type-1-B OS=Arabidopsis thaliana GN=PAF2 PE=1 SV=2//3.30822e-158
XLOC_2367 -//-
XLOC_2367 sp|P10978|POLX_TOBAC Retrovirus-related Pol polyprotein from transposon TNT 1-94 OS=Nicotiana tabacum PE=2 SV=1//1.24
XLOC_2367 sp|Q8VYI3|ECH2_ARATH Enoyl-CoA hydratase 2, peroxisomal OS=Arabidopsis thaliana GN=ECH2 PE=1 SV=1//5.69356e-41
XLOC_2367 sp|Q8LFG1|AMY2_ARATH Probable alpha-amylase 2 OS=Arabidopsis thaliana GN=AMY2 PE=2 SV=1//6.4506e-17
XLOC_2369 -//-
XLOC_2369 sp|Q8H103|G6PIP_ARATH Glucose-6-phosphate isomerase 1, chloroplastic OS=Arabidopsis thaliana GN=PGI1 PE=1 SV=1//8.530
XLOC_2369 sp|P93009|CUTA_ARATH Protein CutA, chloroplastic OS=Arabidopsis thaliana GN=CUTA PE=1 SV=1//7.58431e-45
XLOC_2369 sp|Q8S8F8|GEM_ARATH GLABRA2 expression modulator OS=Arabidopsis thaliana GN=GEM PE=1 SV=1//1.67695e-79
XLOC_2369 sp|Q9FZ95|PUP3_ARATH Purine permease 3 OS=Arabidopsis thaliana GN=PUP3 PE=2 SV=1//9.37933e-24
XLOC_2369 sp|Q9M0S5|ISOA3_ARATH Isoamylase 3, chloroplastic OS=Arabidopsis thaliana GN=ISA3 PE=1 SV=2//3.95603e-148
XLOC_2369 sp|P93343|1433C_TOBAC 14-3-3-like protein C OS=Nicotiana tabacum PE=1 SV=1//1.02789e-17
XLOC_2369 sp|Q9M9E8|FB92_ARATH F-box protein At1g78280 OS=Arabidopsis thaliana GN=At1g78280 PE=2 SV=3//5.39729e-52
XLOC_2370 sp|Q9LME4|P2C09_ARATH Probable protein phosphatase 2C 9 OS=Arabidopsis thaliana GN=At1g22280 PE=1 SV=1//3.5508e-116
XLOC_2370 sp|Q93Y91|STP5_ARATH Sugar transport protein 5 OS=Arabidopsis thaliana GN=STP5 PE=2 SV=1//3.43068e-116
XLOC_2370 -//-
XLOC_2370 sp|Q6L0S6|DNAJ_PICTO Chaperone protein DnaJ OS=Picrophilus torridus (strain ATCC 700027 / DSM 9790 / JCM 10055 / NBRC
XLOC_2372 sp|P08770|TRA1_MAIZE Putative AC transposase OS=Zea mays PE=2 SV=2//5.38131e-94
XLOC_2372 sp|O65819|H2B3_SOLLC Histone H2B.3 (Fragment) OS=Solanum lycopersicum GN=H2B-3 PE=2 SV=1//5.4018e-23
XLOC_2373 sp|P46297|RS23_FRAAN 40S ribosomal protein S23 OS=Fragaria ananassa GN=RPS23 PE=2 SV=1//2.15825e-94
XLOC_2377 -//-
XLOC_2379 sp|Q9SGS2|HMGB9_ARATH High mobility group B protein 9 OS=Arabidopsis thaliana GN=HMGB9 PE=2 SV=1//2.76947e-117
XLOC_2379 -//-
XLOC_2379 sp|Q9ASS4|Y5838_ARATH Probably inactive leucine-rich repeat receptor-like protein kinase At5g48380 OS=Arabidopsis tha
XLOC_2379 sp|O60942|MCE1_HUMAN mRNA-capping enzyme OS=Homo sapiens GN=RNGTT PE=1 SV=1//4.37458e-08
XLOC_2379 sp|Q39102|FTSH1_ARATH ATP-dependent zinc metalloprotease FTSH 1, chloroplastic OS=Arabidopsis thaliana GN=FTSH1 PE=1
XLOC_2379 sp|Q8LBB7|LOG5_ARATH Cytokinin riboside 5'-monophosphate phosphoribohydrolase LOG5 OS=Arabidopsis thaliana GN=LO
XLOC_2381 sp|Q8L9A0|RM21_ARATH 50S ribosomal protein L21, mitochondrial OS=Arabidopsis thaliana GN=RPL21M PE=2 SV=1//5.17166e-1
XLOC_2381 sp|P93014|RR5_ARATH 30S ribosomal protein S5, chloroplastic OS=Arabidopsis thaliana GN=rps5 PE=1 SV=1//4.86487e-92
XLOC_2382 -//-
XLOC_2386 -//-

XLOC_2386 sp|Q9LQI7|CIA30_ARATH Probable complex I intermediate-associated protein 30 OS=Arabidopsis thaliana GN=At1g17350 PE=1
XLOC_2386 -//-
XLOC_2386 sp|Q5RD67|S2544_PONAB Solute carrier family 25 member 44 OS=Pongo abelii GN=SLC25A44 PE=2 SV=2//1.22519e-28
XLOC_2390 sp|Q2G6X5|EFP_NOVAD Elongation factor P OS=Novosphingobium aromaticivorans (strain DSM 12444) GN=efp PE=3 SV=1//1.877
XLOC_2392 -//-
XLOC_2393 -//-
XLOC_2398 -//-
XLOC_2398 sp|Q3S4A7|AHK5_ARATH Histidine kinase 5 OS=Arabidopsis thaliana GN=AHK5 PE=2 SV=1//4.29445e-28
XLOC_2398 sp|Q9SZ52|PP344_ARATH Pentatricopeptide repeat-containing protein At4g31850, chloroplastic OS=Arabidopsis thaliana GN
XLOC_2399 sp|Q3SZ19|PSMD9_BOVIN 26S proteasome non-ATPase regulatory subunit 9 OS=Bos taurus GN=PSMD9 PE=1 SV=1//9.00468e-28
XLOC_2400 sp|Q8VZU9|DERL1_ARATH Derlin-1 OS=Arabidopsis thaliana GN=DER1 PE=2 SV=1//7.23788e-16
XLOC_2404 sp|Q8LBH4|SPX1_ARATH SPX domain-containing protein 1 OS=Arabidopsis thaliana GN=SPX1 PE=2 SV=2//8.20906e-125
XLOC_2405 -//-
XLOC_2405 -//-
XLOC_2405 sp|A6SRX6|MOCOS_BOTFB Molybdenum cofactor sulfurase OS=Botryotinia fuckeliana (strain B05.10) GN=BC1G_15280 PE=3 SV=1
XLOC_2405 -//-
XLOC_2406 sp|P92519|M810_ARATH Uncharacterized mitochondrial protein AtMg00810 OS=Arabidopsis thaliana GN=AtMg00810 PE=4 SV=1//
XLOC_2406 -//-
XLOC_2407 sp|P10401|POLY_DROME Retrovirus-related Pol polyprotein from transposon gypsy OS=Drosophila melanogaster GN=pol PE=4
XLOC_2415 sp|Q2MHE4|HT1_ARATH Serine/threonine-protein kinase HT1 OS=Arabidopsis thaliana GN=HT1 PE=1 SV=1//4.15952e-58
XLOC_2415 sp|Q8LBO2|DHSB2_ARATH Succinate dehydrogenase [ubiquinone] iron-sulfur subunit 2, mitochondrial OS=Arabidopsis thalia
XLOC_2415 sp|Q7FAH2|G3PC2_ORYSJ Glyceraldehyde-3-phosphate dehydrogenase 2, cytosolic OS=Oryza sativa subsp. japonica GN=GAPC2
XLOC_2416 sp|Q9ZUU1|KADC1_ARATH Probable adenylate kinase 1, chloroplastic OS=Arabidopsis thaliana GN=At2g37250 PE=2 SV=1//3.62
XLOC_2416 -//-
XLOC_2417 -//-
XLOC_2418 sp|Q9ZUU1|KADC1_ARATH Probable adenylate kinase 1, chloroplastic OS=Arabidopsis thaliana GN=At2g37250 PE=2 SV=1//1.29
XLOC_2419 sp|Q8LBO2|DHSB2_ARATH Succinate dehydrogenase [ubiquinone] iron-sulfur subunit 2, mitochondrial OS=Arabidopsis thalia
XLOC_2419 sp|Q2MHE4|HT1_ARATH Serine/threonine-protein kinase HT1 OS=Arabidopsis thaliana GN=HT1 PE=1 SV=1//4.48914e-63
XLOC_2423 -//-
XLOC_2423 -//-
XLOC_2424 -//-
XLOC_2426 -//-
XLOC_2426 -//-
XLOC_2426 sp|Q0IQN5|RTOR1_ORYSJ Regulatory-associated protein of TOR 1 OS=Oryza sativa subsp. japonica GN=RAPTOR1 PE=2 SV=2//1.
XLOC_2426 sp|P10978|POLX_TOBAC Retrovirus-related Pol polyprotein from transposon TNT 1-94 OS=Nicotiana tabacum PE=2 SV=1//1.64

XLOC_2427-//-

XLOC_2428sp|P95245|PHLC_MYCTU Phospholipase C 3 OS=Mycobacterium tuberculosis GN=plcC PE=3 SV=2//1.53269e-19

XLOC_2429sp|P10978|POLX_TOBAC Retrovirus-related Pol polyprotein from transposon TNT 1-94 OS=Nicotiana tabacum PE=2 SV=1//1.00

XLOC_2430-//-

XLOC_2430-//-

XLOC_2432sp|Q9ST27|PHOT2_ORYSJ Phototropin-2 OS=Oryza sativa subsp. japonica GN=PHOT2 PE=1 SV=1//1.89307e-13

XLOC_2434sp|O48916|FAH1_ARATH Fatty acid 2-hydroxylase 1 OS=Arabidopsis thaliana GN=FAH1 PE=1 SV=1//1.13696e-39

XLOC_2434sp|P10978|POLX_TOBAC Retrovirus-related Pol polyprotein from transposon TNT 1-94 OS=Nicotiana tabacum PE=2 SV=1//1.93

XLOC_2434sp|Q9SVG4|RETOL_ARATH Reticuline oxidase-like protein OS=Arabidopsis thaliana GN=At4g20830 PE=1 SV=2//0

XLOC_2434sp|Q9FWQ5|HAC12_ARATH Histone acetyltransferase HAC12 OS=Arabidopsis thaliana GN=HAC12 PE=2 SV=2//1.23108e-15

XLOC_2435sp|Q0V7T5|Y1864_ARATH Probable receptor-like protein kinase At1g80640 OS=Arabidopsis thaliana GN=At1g80640 PE=2 SV=1/

XLOC_2435sp|O82146|BAMS2_PANGI Beta-Amyrin Synthase 2 OS=Panax ginseng GN=OSCPNY2 PE=2 SV=1//2.35775e-16

XLOC_2436-//-

XLOC_2438sp|Q9CAA4|BIM2_ARATH Transcription factor BIM2 OS=Arabidopsis thaliana GN=BIM2 PE=1 SV=1//7.1818e-94

XLOC_2439sp|P04146|COPIA_DROME Copia protein OS=Drosophila melanogaster GN=GIP PE=1 SV=3//4.56502e-30

XLOC_2442sp|Q9SA25|WAKLG_ARATH Wall-associated receptor kinase-like 8 OS=Arabidopsis thaliana GN=WAKL8 PE=2 SV=1//2.45004e-14

XLOC_2442-//-

XLOC_2442sp|Q8L612|MACP1_ARATH MACPF domain-containing protein At1g14780 OS=Arabidopsis thaliana GN=At1g14780 PE=2 SV=1//1.179

XLOC_2442sp|Q8L612|MACP1_ARATH MACPF domain-containing protein At1g14780 OS=Arabidopsis thaliana GN=At1g14780 PE=2 SV=1//2.425

XLOC_2442sp|Q9SA25|WAKLG_ARATH Wall-associated receptor kinase-like 8 OS=Arabidopsis thaliana GN=WAKL8 PE=2 SV=1//2.04366e-13

XLOC_2442sp|Q9LN59|WAKLK_ARATH Putative wall-associated receptor kinase-like 11 OS=Arabidopsis thaliana GN=WAKL11 PE=3 SV=2//1

XLOC_2445sp|P25794|PIP2_PEA Probable aquaporin PIP-type 7a OS=Pisum sativum GN=TRG-31 PE=2 SV=2//5.60294e-172

XLOC_2445sp|P25794|PIP2_PEA Probable aquaporin PIP-type 7a OS=Pisum sativum GN=TRG-31 PE=2 SV=2//1.69066e-141

XLOC_2445sp|Q946J2|SUVR1_ARATH Histone-lysine N-methyltransferase SUVR1 OS=Arabidopsis thaliana GN=SUVR1 PE=2 SV=2//4.07746e-0

XLOC_2445sp|O23530|SNC1_ARATH Protein SUPPRESSOR OF npr1-1, CONSTITUTIVE 1 OS=Arabidopsis thaliana GN=SNC1 PE=1 SV=3//2.33366e

XLOC_2445sp|Q40392|TMVRN_NICGU TMV resistance protein N OS=Nicotiana glutinosa GN=N PE=1 SV=1//8.48886e-31

XLOC_2445sp|Q40392|TMVRN_NICGU TMV resistance protein N OS=Nicotiana glutinosa GN=N PE=1 SV=1//1.1376e-62

XLOC_2445sp|Q9FH83|WRK52_ARATH Probable WRKY transcription factor 52 OS=Arabidopsis thaliana GN=WRKY52 PE=2 SV=3//9.26015e-53

XLOC_2446-//-

XLOC_2447sp|O81767|PP348_ARATH Pentatricopeptide repeat-containing protein At4g33990 OS=Arabidopsis thaliana GN=EMB2758 PE=2 S

XLOC_2448-//-

XLOC_2450sp|Q9SSQ4|FRS6_ARATH Protein FAR1-RELATED SEQUENCE 6 OS=Arabidopsis thaliana GN=FRS6 PE=2 SV=1//1.41811e-07

XLOC_2450sp|Q9FN09|NPY3_ARATH BTB/POZ domain-containing protein NPY3 OS=Arabidopsis thaliana GN=NPY3 PE=2 SV=1//2.89001e-09

XLOC_2451sp|P83877|TXN4A_MOUSE Thioredoxin-like protein 4A OS=Mus musculus GN=Txnl4a PE=2 SV=1//1.18883e-06

XLOC_2451sp|P37225|MAON_SOLTU NAD-dependent malic enzyme 59 kDa isoform, mitochondrial OS=Solanum tuberosum PE=1 SV=1//2.69943

XLOC_2452 sp|Q944H2|AB12I_ARATH Protein ABCI12, chloroplastic OS=Arabidopsis thaliana GN=ABCI12 PE=1 SV=1//1.91477e-09
XLOC_2453 sp|Q6NYW6|CLAP2_DANRE CLIP-associating protein 2 OS=Danio rerio GN=clasp2 PE=2 SV=1//1.44997e-12
XLOC_2453 sp|Q9FKJ0|FK132_ARATH F-box/kelch-repeat protein At5g60570 OS=Arabidopsis thaliana GN=At5g60570 PE=2 SV=1//8.1499e-09
XLOC_2454 sp|C0LGX3|HSL2_ARATH LRR receptor-like serine/threonine-protein kinase HSL2 OS=Arabidopsis thaliana GN=HSL2 PE=2 SV=1
XLOC_2454 sp|O82531|PSB1_PETHY Proteasome subunit beta type-1 OS=Petunia hybrida GN=PBF1 PE=2 SV=1//5.20596e-06
XLOC_2456 sp|Q8GUM5|NICA_ARATH Nicastrin OS=Arabidopsis thaliana GN=At3g52640/At3g52650 PE=2 SV=1//6.23962e-09
XLOC_2458 sp|O23169|PP353_ARATH Pentatricopeptide repeat-containing protein At4g37170 OS=Arabidopsis thaliana GN=PCMP-H5 PE=2 S
XLOC_2461 sp|B0BN95|HARB1_RAT Putative nuclease HARBI1 OS=Rattus norvegicus GN=Harbi1 PE=2 SV=1//8.56866e-19
XLOC_2462 sp|G5E8K5|ANK3_MOUSE Ankyrin-3 OS=Mus musculus GN=Ank3 PE=1 SV=1//5.33087e-06
XLOC_2464 -//-
XLOC_2466 -//-
XLOC_2466 sp|Q9SR00|PP213_ARATH Pentatricopeptide repeat-containing protein At3g04760, chloroplastic OS=Arabidopsis thaliana GN
XLOC_2467 sp|Q05762|DRTS1_ARATH Bifunctional dihydrofolate reductase-thymidylate synthase 1 OS=Arabidopsis thaliana GN=THY-1 PE
XLOC_2467 -//-
XLOC_2467 sp|P26569|H12_ARATH Histone H1.2 OS=Arabidopsis thaliana GN=At2g30620 PE=1 SV=1//8.08108e-24
XLOC_2467 -//-
XLOC_2467 sp|Q4KRV1|GPI8_PIG GPI-anchor transamidase OS=Sus scrofa GN=PIGK PE=2 SV=1//4.12327e-11
XLOC_2468 -//-
XLOC_2468 sp|Q96JB2|COG3_HUMAN Conserved oligomeric Golgi complex subunit 3 OS=Homo sapiens GN=COG3 PE=1 SV=3//4.69272e-07
XLOC_2468 sp|P09762|WIN2_SOLTU Wound-induced protein WIN2 OS=Solanum tuberosum GN=WIN2 PE=2 SV=1//5.07178e-86
XLOC_2468 sp|C6TBN2|AKR1_SOYBN Probable aldo-keto reductase 1 OS=Glycine max GN=AKR1 PE=2 SV=1//5.06916e-98
XLOC_2469 sp|P50134|DCOR_DATST Ornithine decarboxylase OS=Datura stramonium PE=2 SV=1//4.50608e-57
XLOC_2469 sp|P50134|DCOR_DATST Ornithine decarboxylase OS=Datura stramonium PE=2 SV=1//2.2214e-12
XLOC_2469 -//-
XLOC_2469 sp|Q21890|YF64_CAEEL Uncharacterized protein R102.4 OS=Caenorhabditis elegans GN=R102.4 PE=2 SV=3//1.17705e-26
XLOC_2469 sp|O52177|RELA_MYXXA GTP pyrophosphokinase OS=Myxococcus xanthus GN=relA PE=3 SV=1//6.95866e-17
XLOC_2469 -//-
XLOC_2470 sp|Q41387|PSBW_SPIOL Photosystem II reaction center W protein, chloroplastic OS=Spinacia oleracea GN=psbW PE=1 SV=1//
XLOC_2470 sp|Q84ZT9|WVD2_ARATH Protein WAVE-DAMPENED 2 OS=Arabidopsis thaliana GN=WVD2 PE=2 SV=1//4.84044e-27
XLOC_2470 sp|P02877|HEVE_HEVBR Pro-hevein OS=Hevea brasiliensis GN=HEV1 PE=1 SV=2//1.17627e-86
XLOC_2470 sp|Q9SQZ9|PITH1_ARATH PITH domain-containing protein At3g04780 OS=Arabidopsis thaliana GN=At3g04780 PE=1 SV=2//5.7569
XLOC_2472 sp|P10978|POLX_TOBAC Retrovirus-related Pol polyprotein from transposon TNT 1-94 OS=Nicotiana tabacum PE=2 SV=1//3.59
XLOC_2474 sp|P10978|POLX_TOBAC Retrovirus-related Pol polyprotein from transposon TNT 1-94 OS=Nicotiana tabacum PE=2 SV=1//1.40
XLOC_2474 sp|Q8LG53|UN932_ARATH UNC93-like protein 2 OS=Arabidopsis thaliana GN=At1g18010 PE=2 SV=2//2.08919e-164
XLOC_2475 sp|P08770|TRA1_MAIZE Putative AC transposase OS=Zea mays PE=2 SV=2//1.23887e-10

XLOC_2475 sp|Q9LTD9|Y5278_ARATH Uncharacterized protein PAM68-like OS=Arabidopsis thaliana GN=At5g52780 PE=2 SV=1//6.49958e-32
XLOC_2477 sp|Q8W474|DRL7_ARATH Probable disease resistance protein At1g58390 OS=Arabidopsis thaliana GN=At1g58390 PE=2 SV=4//3.
XLOC_2477 -//-
XLOC_2478 sp|O04017|NAC98_ARATH Protein CUP-SHAPED COTYLEDON 2 OS=Arabidopsis thaliana GN=NAC098 PE=1 SV=1//5.82897e-104
XLOC_2478 sp|Q9C9H7|RLP12_ARATH Receptor-like protein 12 OS=Arabidopsis thaliana GN=RLP12 PE=2 SV=2//1.10636e-15
XLOC_2478 sp|Q9FRS6|PXL1_ARATH Leucine-rich repeat receptor-like protein kinase PXL1 OS=Arabidopsis thaliana GN=PXL1 PE=2 SV=1/
XLOC_2479 sp|Q0WRC9|SKI17_ARATH F-box protein SKIP17 OS=Arabidopsis thaliana GN=SKIP17 PE=1 SV=1//3.78326e-06
XLOC_2481 -//-
XLOC_2481 sp|O65426|LOL2_ARATH Protein LOL2 OS=Arabidopsis thaliana GN=LOL2 PE=2 SV=1//1.22579e-06
XLOC_2487 -//-
XLOC_2489 sp|A9GP90|UREG_SORC5 Urease accessory protein UreG OS=Sorangium cellulosum (strain So ce56) GN=ureG PE=3 SV=1//4.3648
XLOC_2490 -//-
XLOC_2490 sp|Q94F47|UBC28_ARATH Ubiquitin-conjugating enzyme E2 28 OS=Arabidopsis thaliana GN=UBC28 PE=2 SV=1//2.98918e-104
XLOC_2495 sp|Q2L910|NDHJ_GOSHI NAD(P)H-quinone oxidoreductase subunit J, chloroplastic OS=Gossypium hirsutum GN=ndhJ PE=3 SV=1/
XLOC_2495 sp|Q2PMQ5|CYB6_SOYBN Cytochrome b6 OS=Glycine max GN=petB PE=3 SV=1//2.22863e-46
XLOC_2496 sp|O80365|ARR8_ARATH Two-component response regulator ARR8 OS=Arabidopsis thaliana GN=ARR8 PE=1 SV=1//2.70157e-70
XLOC_2497 sp|P92519|M810_ARATH Uncharacterized mitochondrial protein AtMg00810 OS=Arabidopsis thaliana GN=AtMg00810 PE=4 SV=1//
XLOC_2497 sp|Q8S403|PHO1_ARATH Phosphate transporter PHO1 OS=Arabidopsis thaliana GN=PHO1 PE=2 SV=1//3.06276e-12
XLOC_2500 sp|P0C2F6|RNHX1_ARATH Putative ribonuclease H protein At1g65750 OS=Arabidopsis thaliana GN=At1g65750 PE=3 SV=1//1.127
XLOC_2501 sp|Q9LQ10|1A110_ARATH Probable aminotransferase ACS10 OS=Arabidopsis thaliana GN=ACS10 PE=2 SV=1//7.30906e-47
XLOC_2502 sp|Q9T0D3|HFB2B_ARATH Heat stress transcription factor B-2b OS=Arabidopsis thaliana GN=HSFB2B PE=2 SV=1//1.30896e-78
XLOC_2502 -//-
XLOC_2503 sp|P35130|UBC2_MEDSA Ubiquitin-conjugating enzyme E2 2 OS=Medicago sativa GN=UBC2 PE=2 SV=1//1.3275e-104
XLOC_2504 -//-
XLOC_2507 sp|Q39024|MPK4_ARATH Mitogen-activated protein kinase 4 OS=Arabidopsis thaliana GN=MPK4 PE=1 SV=2//6.30372e-18
XLOC_2509 -//-
XLOC_2511 sp|P10978|POLX_TOBAC Retrovirus-related Pol polyprotein from transposon TNT 1-94 OS=Nicotiana tabacum PE=2 SV=1//1.16
XLOC_2511 -//-
XLOC_2514 sp|Q96MB7|HARB1_HUMAN Putative nuclease HARBI1 OS=Homo sapiens GN=HARBI1 PE=1 SV=1//1.38104e-20
XLOC_2515 sp|Q5RE03|PRP18_PONAB Pre-mRNA-splicing factor 18 OS=Pongo abelii GN=PRPF18 PE=2 SV=1//2.3785e-54
XLOC_2519 sp|Q9SXJ6|CLPP3_ARATH ATP-dependent Clp protease proteolytic subunit 3, chloroplastic OS=Arabidopsis thaliana GN=CLPF
XLOC_2519 sp|P10978|POLX_TOBAC Retrovirus-related Pol polyprotein from transposon TNT 1-94 OS=Nicotiana tabacum PE=2 SV=1//1.18
XLOC_2519 sp|P0C8S1|RP8L2_ARATH Probable disease resistance RPP8-like protein 2 OS=Arabidopsis thaliana GN=RPP8L2 PE=1 SV=1//1.
XLOC_2520 sp|Q0WSN2|DAR2_ARATH Protein DA1-related 2 OS=Arabidopsis thaliana GN=DAR2 PE=2 SV=1//2.29568e-143
XLOC_2520 -//-

XLOC_2521sp|Q8GYB1|NUD15_ARATH Nudix hydrolase 15, mitochondrial OS=Arabidopsis thaliana GN=NUDT15 PE=1 SV=2//2.52579e-52
XLOC_2521sp|Q6VAF4|TBB9_GOSHI Tubulin beta-9 chain OS=Gossypium hirsutum PE=2 SV=1//0
XLOC_2522-//-
XLOC_2525-//-
XLOC_2525sp|P42791|RL182_ARATH 60S ribosomal protein L18-2 OS=Arabidopsis thaliana GN=RPL18B PE=1 SV=2//4.2347e-105
XLOC_2527sp|Q500W7|PIGM_ARATH GPI mannosyltransferase 1 OS=Arabidopsis thaliana GN=PIGM PE=2 SV=1//2.64439e-51
XLOC_2528-//-
XLOC_2528-//-
XLOC_2528sp|B6IPJ8|RL20_RHOCS 50S ribosomal protein L20 OS=Rhodospirillum centenum (strain ATCC 51521 / SW) GN=rplT PE=3 SV=1/
XLOC_2528sp|P49636|RL40_NICSY Ubiquitin-60S ribosomal protein L40 OS=Nicotiana sylvestris GN=UBICEP52-7 PE=2 SV=2//5.23355e-86
XLOC_2531sp|Q8RXC8|RBK2_ARATH Receptor-like cytosolic serine/threonine-protein kinase RBK2 OS=Arabidopsis thaliana GN=RBK2 PE=
XLOC_2534sp|Q9SZL8|FRS5_ARATH Protein FAR1-RELATED SEQUENCE 5 OS=Arabidopsis thaliana GN=FRS5 PE=2 SV=1//2.71472e-18
XLOC_2534sp|Q00IB6|CPL4_ARATH RNA polymerase II C-terminal domain phosphatase-like 4 OS=Arabidopsis thaliana GN=CPL4 PE=1 SV=1
XLOC_2534sp|P08770|TRA1_MAIZE Putative AC transposase OS=Zea mays PE=2 SV=2//1.28002e-39
XLOC_2534-//-
XLOC_2535sp|P24397|HY6H_HYONI Hyoscyamine 6-dioxygenase OS=Hyoscyamus niger GN=H6H PE=1 SV=1//5.53113e-71
XLOC_2536sp|Q9SZL8|FRS5_ARATH Protein FAR1-RELATED SEQUENCE 5 OS=Arabidopsis thaliana GN=FRS5 PE=2 SV=1//8.35928e-22
XLOC_2537sp|Q8L7H1|FBL75_ARATH F-box/LRR-repeat protein At4g14103 OS=Arabidopsis thaliana GN=At4g14103 PE=2 SV=1//2.74163e-33
XLOC_2539sp|Q94F30|ESD4_ARATH Ubiquitin-like-specific protease ESD4 OS=Arabidopsis thaliana GN=ESD4 PE=1 SV=1//3.47574e-12
XLOC_2539sp|Q9FID6|Y5392_ARATH Probable receptor-like protein kinase At5g39020 OS=Arabidopsis thaliana GN=At5g39020 PE=2 SV=1/
XLOC_2540sp|Q9FMA3|PEX5_ARATH Peroxisome biogenesis protein 5 OS=Arabidopsis thaliana GN=PEX5 PE=1 SV=1//1.71568e-16
XLOC_2541sp|Q6AZB8|HARB1_DANRE Putative nuclease HARBI1 OS=Danio rerio GN=harbi1 PE=2 SV=1//7.80414e-27
XLOC_2543sp|Q84W27|SCP43_ARATH Serine carboxypeptidase-like 43 OS=Arabidopsis thaliana GN=SCPL43 PE=2 SV=1//5.22127e-18
XLOC_2545-//-
XLOC_2545sp|Q9ZVC9|FRS3_ARATH Protein FAR1-RELATED SEQUENCE 3 OS=Arabidopsis thaliana GN=FRS3 PE=2 SV=2//0
XLOC_2546sp|O08997|ATOX1_MOUSE Copper transport protein ATOX1 OS=Mus musculus GN=Atox1 PE=2 SV=1//4.0928e-08
XLOC_2546-//-
XLOC_2548sp|A2XFI3|PDC2_ORYSI Pyruvate decarboxylase isozyme 2 OS=Oryza sativa subsp. indica GN=PDC2 PE=2 SV=2//0
XLOC_2549-//-
XLOC_2551sp|P0C073|RUB1_DESAN Ubiquitin-NEDD8-like protein RUB1 OS=Deschampsia antarctica GN=RUB1 PE=2 SV=2//1.11077e-16
XLOC_2551sp|Q9FFN4|BGAL6_ARATH Beta-galactosidase 6 OS=Arabidopsis thaliana GN=BGAL6 PE=2 SV=1//1.00615e-39
XLOC_2555sp|Q8W486|Y1491_ARATH Uncharacterized protein At1g04910 OS=Arabidopsis thaliana GN=At1g04910 PE=1 SV=1//2.3291e-09
XLOC_2558sp|Q9SSR1|Y1259_ARATH DCC family protein At1g52590, chloroplastic OS=Arabidopsis thaliana GN=At1g52590 PE=1 SV=1//4.9
XLOC_2562sp|P62981|RS27A_SOLTU Ubiquitin-40S ribosomal protein S27a OS=Solanum tuberosum GN=UBI3 PE=2 SV=2//2.78356e-83
XLOC_2562sp|P10978|POLX_TOBAC Retrovirus-related Pol polyprotein from transposon TNT 1-94 OS=Nicotiana tabacum PE=2 SV=1//1.92

XLOC_2567 sp|P32110|GSTX6_SOYBN Probable glutathione S-transferase OS=Glycine max GN=HSP26-A PE=2 SV=1//6.60987e-11
XLOC_2570 sp|Q8LBB7|LOG5_ARATH Cytokinin riboside 5'-monophosphate phosphoribohydrolase LOG5 OS=Arabidopsis thaliana GN=LC
XLOC_2571 -//-
XLOC_2572 sp|P53849|GIS2_YEAST Zinc finger protein GIS2 OS=Saccharomyces cerevisiae (strain ATCC 204508 / S288c) GN=GIS2 PE=1 S
XLOC_2573 sp|Q9XER8|RAB7_GOSHI Ras-related protein Rab7 OS=Gossypium hirsutum GN=RAB7 PE=2 SV=1//7.99813e-28
XLOC_2574 -//-
XLOC_2575 sp|P33080|AX10A_SOYBN Auxin-induced protein X10A OS=Glycine max PE=2 SV=1//9.81719e-25
XLOC_2579 -//-
XLOC_2579 sp|P10978|POLX_TOBAC Retrovirus-related Pol polyprotein from transposon TNT 1-94 OS=Nicotiana tabacum PE=2 SV=1//2.16
XLOC_2580 sp|Q8S1Z1|UTP11_ORYSJ Probable U3 small nucleolar RNA-associated protein 11 OS=Oryza sativa subsp. japonica GN=Os01g0
XLOC_2581 sp|Q9S7U9|M2K2_ARATH Mitogen-activated protein kinase kinase 2 OS=Arabidopsis thaliana GN=MKK2 PE=1 SV=2//6.48932e-67
XLOC_2581 -//-
XLOC_2581 sp|P61166|TM258_MOUSE Transmembrane protein 258 OS=Mus musculus GN=Tmem258 PE=2 SV=1//1.45719e-06
XLOC_2581 sp|Q9SZI6|GAT22_ARATH Putative GATA transcription factor 22 OS=Arabidopsis thaliana GN=GATA22 PE=2 SV=1//1.89096e-30
XLOC_2582 sp|Q42484|RPS2_ARATH Disease resistance protein RPS2 OS=Arabidopsis thaliana GN=RPS2 PE=1 SV=1//3.3944e-88
XLOC_2582 sp|Q38932|LCYE_ARATH Lycopene epsilon cyclase, chloroplastic OS=Arabidopsis thaliana GN=LUT2 PE=1 SV=2//1.45944e-14
XLOC_2582 -//-
XLOC_2582 sp|Q9LTS4|BH041_ARATH Putative transcription factor bHLH041 OS=Arabidopsis thaliana GN=BHLH41 PE=4 SV=1//6.72271e-25
XLOC_2582 -//-
XLOC_2582 sp|Q9ZUN5|TET2_ARATH Tetraspanin-2 OS=Arabidopsis thaliana GN=TET2 PE=2 SV=1//6.28936e-105
XLOC_2582 sp|Q8S9H7|DIV_ANTMA Transcription factor DIVARICATA OS=Antirrhinum majus GN=DIVARICATA PE=2 SV=1//2.97457e-29
XLOC_2583 sp|Q9NPC4|A4GAT_HUMAN Lactosylceramide 4-alpha-galactosyltransferase OS=Homo sapiens GN=A4GALT PE=2 SV=1//5.31925e-22
XLOC_2584 sp|Q9FXF2|RKF1_ARATH Probable LRR receptor-like serine/threonine-protein kinase RFK1 OS=Arabidopsis thaliana GN=RKF1
XLOC_2585 -//-
XLOC_2586 -//-
XLOC_2586 sp|Q40392|TMVRN_NICGU TMV resistance protein N OS=Nicotiana glutinosa GN=N PE=1 SV=1//2.64651e-27
XLOC_2586 sp|Q40392|TMVRN_NICGU TMV resistance protein N OS=Nicotiana glutinosa GN=N PE=1 SV=1//9.62991e-12
XLOC_2587 sp|Q9LRR4|R13L1_ARATH Putative disease resistance RPP13-like protein 1 OS=Arabidopsis thaliana GN=RPPL1 PE=2 SV=1//3.
XLOC_2587 sp|Q40392|TMVRN_NICGU TMV resistance protein N OS=Nicotiana glutinosa GN=N PE=1 SV=1//4.49245e-118
XLOC_2587 -//-
XLOC_2591 sp|P10978|POLX_TOBAC Retrovirus-related Pol polyprotein from transposon TNT 1-94 OS=Nicotiana tabacum PE=2 SV=1//4.16
XLOC_2593 -//-
XLOC_2594 -//-
XLOC_2594 -//-
XLOC_2595 -//-

XLOC_2595 -//-
XLOC_2596 sp|C7J4U3|OST4A_ORYSJ Dolichyl-diphosphooligosaccharide--protein glycosyltransferase subunit 4A OS=Oryza sativa subsp
XLOC_2597 sp|Q93ZF5|PHO11_ARATH Phosphate transporter PHO1 homolog 1 OS=Arabidopsis thaliana GN=PHO1-H1 PE=2 SV=1//6.48401e-52
XLOC_2597 -//-
XLOC_2600 -//-
XLOC_2600 sp|A1B4F0|DNAJ_PARDP Chaperone protein DnaJ OS=Paracoccus denitrificans (strain Pd 1222) GN=dnaJ PE=3 SV=1//3.38462e-
XLOC_2600 sp|Q6AWY3|GRF6_ORYSJ Growth-regulating factor 6 OS=Oryza sativa subsp. japonica GN=GRF6 PE=2 SV=2//5.07052e-12
XLOC_2600 -//-
XLOC_2600 -//-
XLOC_2600 sp|O48651|ERG1_PANGI Squalene monooxygenase OS=Panax ginseng PE=2 SV=1//7.60373e-132
XLOC_2600 -//-
XLOC_2601 sp|Q6QNM1|KC1_TOXGO Casein kinase I OS=Toxoplasma gondii PE=2 SV=1//1.3493e-10
XLOC_2604 sp|Q93ZM9|TIF9_ARATH Protein TIFY 9 OS=Arabidopsis thaliana GN=TIFY9 PE=1 SV=1//8.02587e-36
XLOC_2604 sp|Q9LYV6|GEML5_ARATH GEM-like protein 5 OS=Arabidopsis thaliana GN=At5g13200 PE=1 SV=1//1.4226e-85
XLOC_2604 sp|Q94AG2|SERK1_ARATH Somatic embryogenesis receptor kinase 1 OS=Arabidopsis thaliana GN=SERK1 PE=1 SV=2//9.6527e-50
XLOC_2604 sp|Q9FY94|SWT15_ARATH Bidirectional sugar transporter SWEET15 OS=Arabidopsis thaliana GN=SWEET15 PE=2 SV=1//1.10278e-
XLOC_2604 sp|Q9SIV3|GLK1_ARATH Transcription activator GLK1 OS=Arabidopsis thaliana GN=GLK1 PE=2 SV=2//5.07712e-75
XLOC_2604 -//-
XLOC_2604 sp|Q9FMA3|PEX5_ARATH Peroxisome biogenesis protein 5 OS=Arabidopsis thaliana GN=PEX5 PE=1 SV=1//1.16589e-44
XLOC_2604 sp|Q9SUI5|PSAK_ARATH Photosystem I reaction center subunit psaK, chloroplastic OS=Arabidopsis thaliana GN=PSAK PE=2 S
XLOC_2605 sp|Q9Y5A9|YTHD2_HUMAN YTH domain family protein 2 OS=Homo sapiens GN=YTHDF2 PE=1 SV=2//1.3549e-50
XLOC_2605 -//-
XLOC_2608 sp|Q8VY88|LTD_ARATH Protein LHCP TRANSLOCATION DEFECT OS=Arabidopsis thaliana GN=LTD PE=1 SV=1//1.61164e-79
XLOC_2609 -//-
XLOC_2610 sp|P04146|COPIA_DROME Copia protein OS=Drosophila melanogaster GN=GIP PE=1 SV=3//7.9769e-10
XLOC_2610 sp|P19892|RAA5E_ARATH Ras-related protein RABA5e OS=Arabidopsis thaliana GN=RABA5E PE=2 SV=1//2.75341e-124
XLOC_2610 sp|Q9SZL8|FRS5_ARATH Protein FAR1-RELATED SEQUENCE 5 OS=Arabidopsis thaliana GN=FRS5 PE=2 SV=1//2.79536e-23
XLOC_2611 sp|P14381|YTX2_XENLA Transposon TX1 uncharacterized 149 kDa protein OS=Xenopus laevis PE=4 SV=1//3.82771e-12
XLOC_2615 -//-
XLOC_2615 -//-
XLOC_2615 -//-
XLOC_2615 sp|Q9M0Z3|KEA3_ARATH K(+) efflux antiporter 3, chloroplastic OS=Arabidopsis thaliana GN=KEA3 PE=1 SV=2//3.37552e-94
XLOC_2616 sp|A5YVF1|SGS3_SOLLC Protein SUPPRESSOR OF GENE SILENCING 3 OS=Solanum lycopersicum GN=SGS3 PE=1 SV=1//2.37816e-15
XLOC_2616 sp|Q93VG8|PPDEX_ARATH DeSI-like protein At4g17486 OS=Arabidopsis thaliana GN=At4g17486 PE=2 SV=1//4.04167e-55
XLOC_2620 -//-

XLOC_2620 -//-
XLOC_2620 sp|Q93X23|MYRS_QUEIL Myrcene synthase, chloroplastic OS=Quercus ilex PE=1 SV=1//1.65969e-150
XLOC_2625 sp|Q9T048|DRL27_ARATH Disease resistance protein At4g27190 OS=Arabidopsis thaliana GN=At4g27190 PE=2 SV=1//3.05399e-0
XLOC_2629 -//-
XLOC_2634 sp|O64967|HMDH2_GOSHI 3-hydroxy-3-methylglutaryl-coenzyme A reductase 2 OS=Gossypium hirsutum GN=HMG2 PE=3 SV=1//0
XLOC_2634 sp|P29058|HMDH2_HEVBR 3-hydroxy-3-methylglutaryl-coenzyme A reductase 2 (Fragment) OS=Hevea brasiliensis GN=HMGR2 PE=
XLOC_2635 sp|P08770|TRA1_MAIZE Putative AC transposase OS=Zea mays PE=2 SV=2//4.63275e-96
XLOC_2637 -//-
XLOC_2638 -//-
XLOC_2640 sp|Q9ZVH3|ORC6_ARATH Origin recognition complex subunit 6 OS=Arabidopsis thaliana GN=ORC6 PE=1 SV=2//9.66726e-41
XLOC_2641 sp|O80365|ARR8_ARATH Two-component response regulator ARR8 OS=Arabidopsis thaliana GN=ARR8 PE=1 SV=1//8.93149e-71
XLOC_2642 sp|Q7X843|ATL48_ARATH RING-H2 finger protein ATL48 OS=Arabidopsis thaliana GN=ATL48 PE=2 SV=2//2.16325e-41
XLOC_2642 sp|Q8L8T2|GRXC1_ARATH Glutaredoxin-C1 OS=Arabidopsis thaliana GN=GRXC1 PE=2 SV=2//1.2586e-39
XLOC_2643 sp|P16016|CAHC_SPIOL Carbonic anhydrase, chloroplastic OS=Spinacia oleracea PE=1 SV=2//1.56424e-11
XLOC_2646 sp|O23342|SECE1_ARATH Preprotein translocase subunit SECE1 OS=Arabidopsis thaliana GN=SECE1 PE=1 SV=1//4.30287e-34
XLOC_2647 -//-
XLOC_2648 -//-
XLOC_2649 sp|P10978|POLX_TOBAC Retrovirus-related Pol polyprotein from transposon TNT 1-94 OS=Nicotiana tabacum PE=2 SV=1//2.52
XLOC_2652 sp|Q28BZ1|RBM8A_XENTR RNA-binding protein 8A OS=Xenopus tropicalis GN=rbm8a PE=2 SV=1//6.28146e-43
XLOC_2653 sp|Q7LHG5|YI31B_YEAST Transposon Ty3-I Gag-Pol polyprotein OS=Saccharomyces cerevisiae (strain ATCC 204508 / S288c) G
XLOC_2653 -//-
XLOC_2655 sp|P48504|QCR6_SOLTU Cytochrome b-c1 complex subunit 6 OS=Solanum tuberosum PE=1 SV=2//4.08297e-08
XLOC_2658 sp|P69322|UBIQP_PEA Polyubiquitin OS=Pisum sativum GN=PU1 PE=2 SV=2//0
XLOC_2658 sp|P0CH32|UBQ4_ARATH Polyubiquitin 4 OS=Arabidopsis thaliana GN=UBQ4 PE=1 SV=1//0
XLOC_2660 sp|O04378|SYP23_ARATH Syntaxin-23 OS=Arabidopsis thaliana GN=SYP23 PE=2 SV=1//2.05465e-09
XLOC_2660 -//-
XLOC_2662 -//-
XLOC_2664 sp|Q9NPC4|A4GAT_HUMAN Lactosylceramide 4-alpha-galactosyltransferase OS=Homo sapiens GN=A4GALT PE=2 SV=1//7.65382e-20
XLOC_2672 sp|P10978|POLX_TOBAC Retrovirus-related Pol polyprotein from transposon TNT 1-94 OS=Nicotiana tabacum PE=2 SV=1//4.50
XLOC_2673 -//-
XLOC_2676 -//-
XLOC_2677 sp|Q7X843|ATL48_ARATH RING-H2 finger protein ATL48 OS=Arabidopsis thaliana GN=ATL48 PE=2 SV=2//2.15275e-41
XLOC_2680 sp|Q40392|TMVRN_NICGU TMV resistance protein N OS=Nicotiana glutinosa GN=N PE=1 SV=1//3.71455e-19
XLOC_2681 -//-
XLOC_2681 sp|P25349|YCP4_YEAST Flavoprotein-like protein YCP4 OS=Saccharomyces cerevisiae (strain ATCC 204508 / S288c) GN=YCP4

XLOC_2682 -//-
XLOC_2684 -//-
XLOC_2684 sp|C7J4U3|OST4A_ORYSJ Dolichyl-diphosphooligosaccharide--protein glycosyltransferase subunit 4A OS=Oryza sativa subsp
XLOC_2685 sp|P24397|HY6H_HYONI Hyoscyamine 6-dioxygenase OS=Hyoscyamus niger GN=H6H PE=1 SV=1//3.74957e-59
XLOC_2685 sp|O04921|HEMH2_ARATH Ferrochelatase-2, chloroplastic OS=Arabidopsis thaliana GN=At2g30390 PE=2 SV=1//5.89428e-07
XLOC_2686 sp|Q56YJ8|FAMA_ARATH Transcription factor FAMA OS=Arabidopsis thaliana GN=FMA PE=1 SV=1//2.26437e-136
XLOC_2687 -//-
XLOC_2690 sp|Q9LT17|BBR_ARATH E3 ubiquitin ligase BIG BROTHER-related OS=Arabidopsis thaliana GN=BBR PE=2 SV=1//3.87195e-76
XLOC_2695 sp|Q9ZRD6|YKT61_ARATH VAMP-like protein YKT61 OS=Arabidopsis thaliana GN=YKT61 PE=2 SV=1//1.79937e-17
XLOC_2697 sp|Q4R4E4|MON1B_MACFA Vacuolar fusion protein MON1 homolog B OS=Macaca fascicularis GN=MON1B PE=2 SV=1//2.10269e-07
XLOC_2697 -//-
XLOC_2697 sp|Q8NI08|NCOA7_HUMAN Nuclear receptor coactivator 7 OS=Homo sapiens GN=NCOA7 PE=1 SV=2//1.66605e-14
XLOC_2698 sp|P0C2F6|RNHX1_ARATH Putative ribonuclease H protein At1g65750 OS=Arabidopsis thaliana GN=At1g65750 PE=3 SV=1//5.168
XLOC_2705 sp|Q96MB7|HARB1_HUMAN Putative nuclease HARBI1 OS=Homo sapiens GN=HARBI1 PE=1 SV=1//9.97087e-21
XLOC_2706 sp|Q9SX38|DRL4_ARATH Putative disease resistance protein At1g50180 OS=Arabidopsis thaliana GN=At1g50180 PE=2 SV=2//3.
XLOC_2708 -//-
XLOC_2709 sp|P04146|COPIA_DROME Copia protein OS=Drosophila melanogaster GN=GIP PE=1 SV=3//4.59338e-105
XLOC_2709 sp|P37392|TBB1_LUPAL Tubulin beta-1 chain OS=Lupinus albus GN=TUBB1 PE=3 SV=1//0
XLOC_2709 sp|P37392|TBB1_LUPAL Tubulin beta-1 chain OS=Lupinus albus GN=TUBB1 PE=3 SV=1//0
XLOC_2711 sp|Q9LTD9|Y5278_ARATH Uncharacterized protein PAM68-like OS=Arabidopsis thaliana GN=At5g52780 PE=2 SV=1//2.41306e-20
XLOC_2715 -//-
XLOC_2716 sp|P42745|UBC2_ARATH Ubiquitin-conjugating enzyme E2 2 OS=Arabidopsis thaliana GN=UBC2 PE=2 SV=1//2.18913e-11
XLOC_2717 sp|Q9MAM1|CIPK9_ARATH CBL-interacting serine/threonine-protein kinase 9 OS=Arabidopsis thaliana GN=CIPK9 PE=1 SV=2//1
XLOC_2718 sp|Q42551|SCE1_ARATH SUMO-conjugating enzyme SCE1 OS=Arabidopsis thaliana GN=SCE1 PE=1 SV=1//1.53313e-49
XLOC_2719 sp|P08770|TRA1_MAIZE Putative AC transposase OS=Zea mays PE=2 SV=2//1.31688e-10
XLOC_2719 sp|P35130|UBC2_MEDSA Ubiquitin-conjugating enzyme E2 2 OS=Medicago sativa GN=UBC2 PE=2 SV=1//8.56705e-100
XLOC_2722 sp|P10978|POLX_TOBAC Retrovirus-related Pol polyprotein from transposon TNT 1-94 OS=Nicotiana tabacum PE=2 SV=1//6.80
XLOC_2724 -//-
XLOC_2725 sp|P49636|RL40_NICSY Ubiquitin-60S ribosomal protein L40 OS=Nicotiana sylvestris GN=UBICEP52-7 PE=2 SV=2//1.01471e-71
XLOC_2728 -//-
XLOC_2728 sp|P0C2F6|RNHX1_ARATH Putative ribonuclease H protein At1g65750 OS=Arabidopsis thaliana GN=At1g65750 PE=3 SV=1//1.300
XLOC_2730 sp|Q9SKT3|SCAM1_ARATH Secretory carrier-associated membrane protein 1 OS=Arabidopsis thaliana GN=SCAMP1 PE=1 SV=1//2.
XLOC_2733 -//-
XLOC_2733 sp|P10978|POLX_TOBAC Retrovirus-related Pol polyprotein from transposon TNT 1-94 OS=Nicotiana tabacum PE=2 SV=1//3.25
XLOC_2736 sp|Q9ZQI7|ALD1_ARATH Aminotransferase ALD1 OS=Arabidopsis thaliana GN=ALD1 PE=2 SV=2//3.55335e-19

XLOC_2736	sp|Q9SZN7|HIP26_ARATH Heavy metal-associated isoprenylated plant protein 26 OS=Arabidopsis thaliana GN=HIPP26 PE=1 SV
XLOC_2736	sp|Q84N64|ARC5_ARATH Dynamin-like protein ARC5 OS=Arabidopsis thaliana GN=ARC5 PE=1 SV=2//4.25804e-26
XLOC_2736	sp|Q9SZ67|WRK19_ARATH Probable WRKY transcription factor 19 OS=Arabidopsis thaliana GN=WRKY19 PE=2 SV=1//2.58121e-41
XLOC_2736	sp|Q9LM55|FBK8_ARATH F-box/kelch-repeat protein At1g22040 OS=Arabidopsis thaliana GN=At1g22040 PE=2 SV=1//0
XLOC_2737	-//-
XLOC_2738	sp|Q8VYA5|RSZ33_ARATH Serine/arginine-rich splicing factor RS2Z33 OS=Arabidopsis thaliana GN=RS2Z33 PE=1 SV=1//2.1297
XLOC_2739	sp|P10978|POLX_TOBAC Retrovirus-related Pol polyprotein from transposon TNT 1-94 OS=Nicotiana tabacum PE=2 SV=1//5.92
XLOC_2739	-//-
XLOC_2743	sp|Q40392|TMVRN_NICGU TMV resistance protein N OS=Nicotiana glutinosa GN=N PE=1 SV=1//4.16406e-42
XLOC_2743	sp|Q40392|TMVRN_NICGU TMV resistance protein N OS=Nicotiana glutinosa GN=N PE=1 SV=1//4.20024e-16
XLOC_2743	sp|Q9FL92|WRK16_ARATH Probable WRKY transcription factor 16 OS=Arabidopsis thaliana GN=WRKY16 PE=2 SV=1//2.61561e-13
XLOC_2744	sp|Q9LKL2|APRR1_ARATH Two-component response regulator-like APRR1 OS=Arabidopsis thaliana GN=APRR1 PE=1 SV=1//2.85735
XLOC_2744	sp|P49636|RL40_NICSY Ubiquitin-60S ribosomal protein L40 OS=Nicotiana sylvestris GN=UBICEP52-7 PE=2 SV=2//2.71694e-88
XLOC_2745	-//-
XLOC_2746	sp|P10978|POLX_TOBAC Retrovirus-related Pol polyprotein from transposon TNT 1-94 OS=Nicotiana tabacum PE=2 SV=1//1.64
XLOC_2746	-//-
XLOC_2751	-//-
XLOC_2752	sp|P10978|POLX_TOBAC Retrovirus-related Pol polyprotein from transposon TNT 1-94 OS=Nicotiana tabacum PE=2 SV=1//4.54
XLOC_2753	-//-
XLOC_2753	-//-
XLOC_2756	sp|Q8RYC8|ARFS_ARATH Auxin response factor 19 OS=Arabidopsis thaliana GN=ARF19 PE=1 SV=2//6.2762e-134
XLOC_2759	-//-
XLOC_2760	sp|Q7X8C5|WAKLB_ARATH Wall-associated receptor kinase-like 2 OS=Arabidopsis thaliana GN=WAKL2 PE=2 SV=1//1.62739e-88
XLOC_2760	sp|Q8RY17|WAKLI_ARATH Wall-associated receptor kinase-like 22 OS=Arabidopsis thaliana GN=WAKL22 PE=2 SV=1//1.05571e-0
XLOC_2760	sp|Q9LHP4|RCH2_ARATH Receptor-like protein kinase 2 OS=Arabidopsis thaliana GN=RCH2 PE=1 SV=1//2.12108e-40
XLOC_2760	-//-
XLOC_2761	sp|O34508|AEEP_BACSU L-Ala-D/L-Glu epimerase OS=Bacillus subtilis (strain 168) GN=ykfB PE=1 SV=1//3.63575e-51
XLOC_2762	sp|Q93ZT5|EDL3_ARATH EID1-like F-box protein 3 OS=Arabidopsis thaliana GN=EDL3 PE=2 SV=1//4.346e-62
XLOC_2765	sp|Q9FZJ1|IRX10_ARATH Probable beta-1,4-xylosyltransferase IRX10 OS=Arabidopsis thaliana GN=IRX10 PE=2 SV=1//3.75295e
XLOC_2766	sp|Q940Z2|ARP5_ARATH Actin-related protein 5 OS=Arabidopsis thaliana GN=ARP5 PE=1 SV=2//0
XLOC_2767	sp|Q9T0D3|HFB2B_ARATH Heat stress transcription factor B-2b OS=Arabidopsis thaliana GN=HSFB2B PE=2 SV=1//5.62389e-15
XLOC_2768	-//-
XLOC_2770	sp|P09394|GLPQ_ECOLI Glycerophosphoryl diester phosphodiesterase OS=Escherichia coli (strain K12) GN=glpQ PE=1 SV=2//
XLOC_2770	sp|Q9SVM8|GRP2_ARATH Glycine-rich RNA-binding protein 2, mitochondrial OS=Arabidopsis thaliana GN=GRP2 PE=1 SV=1//5.3
XLOC_2770	-//-

XLOC_2770 sp|P42730|CLPB1_ARATH Chaperone protein ClpB1 OS=Arabidopsis thaliana GN=CLPB1 PE=1 SV=2//0
XLOC_2770 sp|P27880|HSP12_MEDSA 18.2 kDa class I heat shock protein OS=Medicago sativa GN=HSP18.2 PE=2 SV=1//5.13548e-70
XLOC_2770 -//-
XLOC_2772 -//-
XLOC_2774 sp|P42856|ZB14_MAIZE 14 kDa zinc-binding protein OS=Zea mays GN=ZBP14 PE=1 SV=1//4.23784e-07
XLOC_2775 sp|Q8RWM3|MED27_ARATH Mediator of RNA polymerase II transcription subunit 27 OS=Arabidopsis thaliana GN=MED27 PE=1 SV
XLOC_2775 sp|Q8VYA5|RSZ33_ARATH Serine/arginine-rich splicing factor RS2Z33 OS=Arabidopsis thaliana GN=RS2Z33 PE=1 SV=1//3.9944
XLOC_2776 sp|F4I933|STR8_ARATH Rhodanese-like domain-containing protein 8, chloroplastic OS=Arabidopsis thaliana GN=STR8 PE=2 S
XLOC_2780 sp|P08770|TRA1_MAIZE Putative AC transposase OS=Zea mays PE=2 SV=2//5.40918e-75
XLOC_2782 sp|Q9SHZ8|PP168_ARATH Pentatricopeptide repeat-containing protein At2g22070 OS=Arabidopsis thaliana GN=PCMP-H41 PE=2
XLOC_2783 sp|Q43019|NLTP3_PRUDU Non-specific lipid-transfer protein 3 OS=Prunus dulcis PE=2 SV=1//2.48252e-22
XLOC_2783 sp|Q9LX82|MYB48_ARATH Transcription factor MYB48 OS=Arabidopsis thaliana GN=MYB48 PE=2 SV=1//8.81409e-15
XLOC_2783 -//-
XLOC_2784 -//-
XLOC_2785 sp|P49689|RS30_ARATH 40S ribosomal protein S30 OS=Arabidopsis thaliana GN=RPS30A PE=1 SV=3//6.01282e-12
XLOC_2785 sp|P48631|FD6E2_SOYBN Omega-6 fatty acid desaturase, endoplasmic reticulum isozyme 2 OS=Glycine max GN=FAD2-2 PE=2 SV
XLOC_2793 sp|A2RRP1|NBAS_HUMAN Neuroblastoma-amplified sequence OS=Homo sapiens GN=NBAS PE=1 SV=2//7.53347e-07
XLOC_2793 sp|P52855|RLA1_MAIZE 60S acidic ribosomal protein P1 OS=Zea mays GN=RPP1A PE=1 SV=1//7.75368e-25
XLOC_2794 -//-
XLOC_2795 -//-
XLOC_2796 -//-
XLOC_2797 sp|Q54WM0|T1843_DICDI Transmembrane protein 184 homolog DDB_G0279555 OS=Dictyostelium discoideum GN=tmem184C PE=3 SV=
XLOC_2798 sp|P10978|POLX_TOBAC Retrovirus-related Pol polyprotein from transposon TNT 1-94 OS=Nicotiana tabacum PE=2 SV=1//8.22
XLOC_2798 -//-
XLOC_2798 sp|Q9SHI3|RLP2_ARATH Receptor-like protein 2 OS=Arabidopsis thaliana GN=RLP2 PE=2 SV=1//3.00223e-14
XLOC_2798 -//-
XLOC_2798 sp|Q9C9H7|RLP12_ARATH Receptor-like protein 12 OS=Arabidopsis thaliana GN=RLP12 PE=2 SV=2//1.00199e-06
XLOC_2798 sp|Q9C9H7|RLP12_ARATH Receptor-like protein 12 OS=Arabidopsis thaliana GN=RLP12 PE=2 SV=2//1.73348e-15
XLOC_2799 sp|Q9XIA7|TOM6_ARATH Mitochondrial import receptor subunit TOM6 homolog OS=Arabidopsis thaliana GN=TOM6 PE=1 SV=1//1.
XLOC_2803 sp|P04323|POL3_DROME Retrovirus-related Pol polyprotein from transposon 17.6 OS=Drosophila melanogaster GN=pol PE=4 S
XLOC_2806 sp|Q9M2Y6|Y3972_ARATH Uncharacterized protein At3g49720 OS=Arabidopsis thaliana GN=At3g49720 PE=1 SV=1//6.6336e-111
XLOC_2807 -//-
XLOC_2808 sp|Q9LRR4|R13L1_ARATH Putative disease resistance RPP13-like protein 1 OS=Arabidopsis thaliana GN=RPPL1 PE=2 SV=1//4.
XLOC_2808 sp|Q9LRR5|DRL21_ARATH Putative disease resistance protein At3g14460 OS=Arabidopsis thaliana GN=At3g14460 PE=2 SV=1//4
XLOC_2809 sp|Q56XU4|C3H6_ARATH Zinc finger CCCH domain-containing protein 6 OS=Arabidopsis thaliana GN=At1g19860 PE=2 SV=1//3.7

XLOC_2811-//-
XLOC_2813sp|A9BIJ7|SYV_PETMO Valine--tRNA ligase OS=Petrotoga mobilis (strain DSM 10674 / SJ95) GN=valS PE=3 SV=1//2.47688e-08
XLOC_2813sp|Q84JK8|MAKR6_ARATH Probable membrane-associated kinase regulator 6 OS=Arabidopsis thaliana GN=MAKR6 PE=2 SV=1//1.9
XLOC_2814-//-
XLOC_2814sp|O15050|TRNK1_HUMAN TPR and ankyrin repeat-containing protein 1 OS=Homo sapiens GN=TRANK1 PE=2 SV=4//1.26304e-19
XLOC_2815sp|Q9FNA2|PAO1_ARATH Polyamine oxidase 1 OS=Arabidopsis thaliana GN=PAO1 PE=1 SV=1//0
XLOC_2816sp|Q94A94|DCDA2_ARATH Diaminopimelate decarboxylase 2, chloroplastic OS=Arabidopsis thaliana GN=LYSA2 PE=2 SV=1//8.82
XLOC_2816sp|Q84P24|4CLL6_ARATH 4-coumarate--CoA ligase-like 6 OS=Arabidopsis thaliana GN=4CLL6 PE=2 SV=2//9.20256e-39
XLOC_2816sp|P10978|POLX_TOBAC Retrovirus-related Pol polyprotein from transposon TNT 1-94 OS=Nicotiana tabacum PE=2 SV=1//6.77
XLOC_2817sp|O55055|TRDMT_MOUSE tRNA (cytosine(38)-C(5))-methyltransferase OS=Mus musculus GN=Trdmt1 PE=2 SV=2//7.66568e-08
XLOC_2817sp|Q0WPT7|Y2104_ARATH Uncharacterized methyltransferase At2g41040, chloroplastic OS=Arabidopsis thaliana GN=At2g41040
XLOC_2817sp|O22969|Y2416_ARATH Uncharacterized protein At2g34160 OS=Arabidopsis thaliana GN=At2g34160 PE=1 SV=1//1.06865e-15
XLOC_2818sp|P24922|IF5A2_NICPL Eukaryotic translation initiation factor 5A-2 OS=Nicotiana plumbaginifolia GN=EIF-5A2 PE=2 SV=1
XLOC_2818sp|Q9FLQ7|FH20_ARATH Formin-like protein 20 OS=Arabidopsis thaliana GN=FH20 PE=2 SV=3//2.99995e-85
XLOC_2818sp|Q9FLQ7|FH20_ARATH Formin-like protein 20 OS=Arabidopsis thaliana GN=FH20 PE=2 SV=3//3.41601e-80
XLOC_2818sp|Q9FLQ7|FH20_ARATH Formin-like protein 20 OS=Arabidopsis thaliana GN=FH20 PE=2 SV=3//1.90826e-16
XLOC_2819sp|Q94EI3|PE192_ARATH Peroxisome biogenesis protein 19-2 OS=Arabidopsis thaliana GN=PEX19-2 PE=1 SV=1//9.54472e-62
XLOC_2821-//-
XLOC_2822-//-
XLOC_2825-//-
XLOC_2826-//-
XLOC_2828sp|Q2QV94|EXOC5_ORYSJ Exocyst complex component 5 OS=Oryza sativa subsp. japonica GN=SEC10 PE=2 SV=1//9.94192e-12
XLOC_2831sp|P52855|RLA1_MAIZE 60S acidic ribosomal protein P1 OS=Zea mays GN=RPP1A PE=1 SV=1//1.18287e-23
XLOC_2833-//-
XLOC_2833sp|P10978|POLX_TOBAC Retrovirus-related Pol polyprotein from transposon TNT 1-94 OS=Nicotiana tabacum PE=2 SV=1//7.53
XLOC_2839sp|Q9SJM6|SAP4_ARATH Zinc finger A20 and AN1 domain-containing stress-associated protein 4 OS=Arabidopsis thaliana GN
XLOC_2840sp|Q9LNG5|PPP7L_ARATH Serine/threonine-protein phosphatase 7 long form homolog OS=Arabidopsis thaliana GN=At1g48120 P
XLOC_2841-//-
XLOC_2843sp|Q8L4B2|NFYC9_ARATH Nuclear transcription factor Y subunit C-9 OS=Arabidopsis thaliana GN=NFYC9 PE=2 SV=1//1.83554e
XLOC_2843sp|Q9XJ27|RR9_ARATH 30S ribosomal protein S9, chloroplastic OS=Arabidopsis thaliana GN=RPS9 PE=2 SV=1//7.61447e-77
XLOC_2846-//-
XLOC_2847sp|Q9FPW6|POB1_ARATH BTB/POZ domain-containing protein POB1 OS=Arabidopsis thaliana GN=POB1 PE=2 SV=2//6.20491e-126
XLOC_2848sp|F4IQJ2|ELP5_ARATH Elongator complex protein 5 OS=Arabidopsis thaliana GN=ELP5 PE=1 SV=1//8.48616e-15
XLOC_2849sp|Q91YT2|RN185_MOUSE E3 ubiquitin-protein ligase RNF185 OS=Mus musculus GN=Rnf185 PE=2 SV=1//8.24175e-28
XLOC_2850-//-

XLOC_2851 sp|Q8RWS9|CNGC5_ARATH Probable cyclic nucleotide-gated ion channel 5 OS=Arabidopsis thaliana GN=CNGC5 PE=2 SV=1//2.21
XLOC_2856 -//-
XLOC_2858 sp|P49608|ACOC_CUCMA Aconitate hydratase, cytoplasmic OS=Cucurbita maxima PE=2 SV=1//0
XLOC_2859 sp|Q93VY3|NAC72_ARATH NAC domain-containing protein 72 OS=Arabidopsis thaliana GN=NAC072 PE=2 SV=1//3.65344e-130
XLOC_2860 -//-
XLOC_2861 sp|O49169|EF1A_MANES Elongation factor 1-alpha OS=Manihot esculenta GN=EF1 PE=3 SV=1//1.02615e-14
XLOC_2865 -//-
XLOC_2866 sp|Q9SXB4|Y1130_ARATH G-type lectin S-receptor-like serine/threonine-protein kinase At1g11300 OS=Arabidopsis thaliana
XLOC_2867 sp|Q9LP24|Y1571_ARATH Probable leucine-rich repeat receptor-like protein kinase At1g35710 OS=Arabidopsis thaliana GN=
XLOC_2868 sp|Q39002|TLC1_ARATH ADP,ATP carrier protein 1, chloroplastic OS=Arabidopsis thaliana GN=AATP1 PE=1 SV=2//1.10318e-12
XLOC_2869 -//-
XLOC_2871 sp|P55217|METB_ARATH Cystathionine gamma-synthase, chloroplastic OS=Arabidopsis thaliana GN=CGS1 PE=2 SV=3//4.16474e-
XLOC_2871 sp|Q94A21|SPX4_ARATH SPX domain-containing protein 4 OS=Arabidopsis thaliana GN=SPX4 PE=2 SV=1//5.72214e-75
XLOC_2871 sp|Q9FKA5|Y5957_ARATH Uncharacterized protein At5g39570 OS=Arabidopsis thaliana GN=At5g39570 PE=1 SV=1//2.12486e-09
XLOC_2871 sp|P92958|KIN11_ARATH SNF1-related protein kinase catalytic subunit alpha KIN11 OS=Arabidopsis thaliana GN=KIN11 PE=1
XLOC_2873 sp|Q9P6K0|YLA3_SCHPO Uncharacterized HTH La-type RNA-binding protein C1527.03 OS=Schizosaccharomyces pombe (strain 97
XLOC_2873 -//-
XLOC_2879 sp|Q0WVK7|PPR12_ARATH Pentatricopeptide repeat-containing protein At1g05670, mitochondrial OS=Arabidopsis thaliana GN
XLOC_2880 sp|Q55CU8|RSC5_DICDI Random slug protein 5 OS=Dictyostelium discoideum GN=rsc5 PE=2 SV=1//2.44438e-07
XLOC_2882 sp|Q9ZW34|ENO3_ARATH Cytosolic enolase 3 OS=Arabidopsis thaliana GN=ENO3 PE=2 SV=1//2.116e-33
XLOC_2883 sp|Q5R580|ZRAB2_PONAB Zinc finger Ran-binding domain-containing protein 2 OS=Pongo abelii GN=ZRANB2 PE=2 SV=1//3.3144
XLOC_2883 sp|P10978|POLX_TOBAC Retrovirus-related Pol polyprotein from transposon TNT 1-94 OS=Nicotiana tabacum PE=2 SV=1//1.92
XLOC_2883 sp|Q9SLH3|RGA_ARATH DELLA protein RGA OS=Arabidopsis thaliana GN=RGA PE=1 SV=1//1.11065e-29
XLOC_2883 -//-
XLOC_2884 sp|Q9FJ41|GDL85_ARATH GDSL esterase/lipase At5g45950 OS=Arabidopsis thaliana GN=At5g45950 PE=2 SV=1//2.4593e-18
XLOC_2886 -//-
XLOC_2887 sp|Q7TP47|HNRPQ_RAT Heterogeneous nuclear ribonucleoprotein Q OS=Rattus norvegicus GN=Syncrip PE=2 SV=1//4.04802e-48
XLOC_2889 sp|Q9LUJ5|EBP2_ARATH Probable rRNA-processing protein EBP2 homolog OS=Arabidopsis thaliana GN=At3g22660 PE=2 SV=1//2.
XLOC_2890 sp|Q9M0N8|MZT1B_ARATH Mitotic-spindle organizing protein 1B OS=Arabidopsis thaliana GN=At4g09550 PE=1 SV=1//6.05533e-
XLOC_2892 -//-
XLOC_2892 sp|Q86JJ0|TRM61_DICDI tRNA (adenine(58)-N(1))-methyltransferase catalytic subunit trmt61a OS=Dictyostelium discoideum
XLOC_2894 sp|Q9FMA3|PEX5_ARATH Peroxisome biogenesis protein 5 OS=Arabidopsis thaliana GN=PEX5 PE=1 SV=1//2.41269e-24
XLOC_2894 -//-
XLOC_2894 sp|Q6NRP2|PSME4_XENLA Proteasome activator complex subunit 4 OS=Xenopus laevis GN=psme4 PE=2 SV=1//1.64674e-25
XLOC_2895 sp|O81635|ATK4_ARATH Kinesin-4 OS=Arabidopsis thaliana GN=ATK4 PE=1 SV=2//4.03206e-65

XLOC_2895	sp|Q9LTH2|U76E2_ARATH UDP-glycosyltransferase 76E2 OS=Arabidopsis thaliana GN=UGT76E2 PE=2 SV=1//1.64817e-118
XLOC_2895	sp|Q9FM66|PLY21_ARATH Putative pectate lyase 21 OS=Arabidopsis thaliana GN=At5g55720 PE=2 SV=1//2.79883e-159
XLOC_2895	-//-
XLOC_2895	-//-
XLOC_2896	sp|Q9LK39|AAE16_ARATH Probable acyl-activating enzyme 16, chloroplastic OS=Arabidopsis thaliana GN=AAE16 PE=2 SV=1//1
XLOC_2896	-//-
XLOC_2896	sp|Q9FMA3|PEX5_ARATH Peroxisome biogenesis protein 5 OS=Arabidopsis thaliana GN=PEX5 PE=1 SV=1//8.29744e-28
XLOC_2896	sp|P35135|UBC4_SOLLC Ubiquitin-conjugating enzyme E2-17 kDa OS=Solanum lycopersicum PE=2 SV=1//2.52323e-99
XLOC_2896	sp|Q84VX0|RFS1_ARATH Probable galactinol--sucrose galactosyltransferase 1 OS=Arabidopsis thaliana GN=RFS1 PE=2 SV=1//
XLOC_2896	sp|Q9LRR5|DRL21_ARATH Putative disease resistance protein At3g14460 OS=Arabidopsis thaliana GN=At3g14460 PE=2 SV=1//4
XLOC_2896	-//-
XLOC_2896	sp|C0LGR3|Y4265_ARATH Probable LRR receptor-like serine/threonine-protein kinase At4g26540 OS=Arabidopsis thaliana GN
XLOC_2898	sp|Q07488|BCB1_ARATH Blue copper protein OS=Arabidopsis thaliana GN=BCB PE=1 SV=2//8.54821e-12
XLOC_2899	sp|P10978|POLX_TOBAC Retrovirus-related Pol polyprotein from transposon TNT 1-94 OS=Nicotiana tabacum PE=2 SV=1//1.0
XLOC_2899	sp|Q6AWT8|GG3_ARATH Guanine nucleotide-binding protein subunit gamma 3 OS=Arabidopsis thaliana GN=GG3 PE=2 SV=1//7.44
XLOC_2900	sp|Q9LZI6|NDS5B_ARATH NADH dehydrogenase [ubiquinone] iron-sulfur protein 5-B OS=Arabidopsis thaliana GN=At3g62790 PE
XLOC_2901	sp|Q9ZRU9|LSM4_FAGSY Probable U6 snRNA-associated Sm-like protein LSm4 OS=Fagus sylvatica GN=LSM4 PE=2 SV=1//6.85437e
XLOC_2902	sp|Q55535|Y328_SYNY3 Putative low molecular weight protein-tyrosine-phosphatase slr0328 OS=Synechocystis sp. (strain
XLOC_2902	sp|F4HW04|DPOE1_ARATH DNA polymerase epsilon catalytic subunit A OS=Arabidopsis thaliana GN=POL2A PE=1 SV=1//4.20715e
XLOC_2902	sp|F4HW04|DPOE1_ARATH DNA polymerase epsilon catalytic subunit A OS=Arabidopsis thaliana GN=POL2A PE=1 SV=1//8.05516e
XLOC_2903	sp|P10978|POLX_TOBAC Retrovirus-related Pol polyprotein from transposon TNT 1-94 OS=Nicotiana tabacum PE=2 SV=1//1.31
XLOC_2904	sp|Q4HXT6|PMP3_GIBZE Plasma membrane proteolipid 3 OS=Gibberella zeae (strain PH-1 / ATCC MYA-4620 / FGSC 9075 / NRRL
XLOC_2906	sp|Q700D2|JKD_ARATH Zinc finger protein JACKDAW OS=Arabidopsis thaliana GN=JKD PE=1 SV=1//7.68935e-20
XLOC_2906	sp|Q700D2|JKD_ARATH Zinc finger protein JACKDAW OS=Arabidopsis thaliana GN=JKD PE=1 SV=1//5.28828e-09
XLOC_2910	sp|Q9C9D0|SOT16_ARATH Cytosolic sulfotransferase 16 OS=Arabidopsis thaliana GN=SOT16 PE=1 SV=1//1.12198e-43
XLOC_2910	-//-
XLOC_2910	sp|O49858|C82A3_SOYBN Cytochrome P450 82A3 OS=Glycine max GN=CYP82A3 PE=2 SV=1//2.69748e-117
XLOC_2911	-//-
XLOC_2911	-//-
XLOC_2911	sp|P86221|TBB4B_MESAU Tubulin beta-4B chain (Fragments) OS=Mesocricetus auratus GN=TUBB4B PE=1 SV=1//8.94407e-27
XLOC_2912	sp|O65154|KIWI_ARATH RNA polymerase II transcriptional coactivator KIWI OS=Arabidopsis thaliana GN=KIWI PE=1 SV=1//1.
XLOC_2912	sp|B1XJY4|MNMG_SYNP2 tRNA uridine 5-carboxymethylaminomethyl modification enzyme MnmG OS=Synechococcus sp. (strain AT
XLOC_2912	sp|P33083|AX6B_SOYBN Auxin-induced protein 6B OS=Glycine max PE=2 SV=1//8.17368e-12
XLOC_2912	-//-
XLOC_2913	-//-

XLOC_2915-//-
XLOC_2915sp|O23266|PP308_ARATH Pentatricopeptide repeat-containing protein At4g14050, mitochondrial OS=Arabidopsis thaliana GN
XLOC_2917sp|Q8LB81|GDL79_ARATH GDSL esterase/lipase At5g33370 OS=Arabidopsis thaliana GN=At5g33370 PE=2 SV=1//3.25153e-131
XLOC_2920sp|Q9NVW2|RNF12_HUMAN E3 ubiquitin-protein ligase RLIM OS=Homo sapiens GN=RLIM PE=1 SV=3//1.04026e-13
XLOC_2922-//-
XLOC_2923sp|Q9SEC2|MSRA_LACSA Peptide methionine sulfoxide reductase OS=Lactuca sativa PE=2 SV=1//1.95566e-58
XLOC_2923sp|Q9FGW9|GTE10_ARATH Transcription factor GTE10 OS=Arabidopsis thaliana GN=GTE10 PE=1 SV=2//3.45142e-10
XLOC_2924sp|Q9FNP8|RS193_ARATH 40S ribosomal protein S19-3 OS=Arabidopsis thaliana GN=RPS19C PE=2 SV=1//2.9596e-85
XLOC_2924sp|Q9SSQ4|FRS6_ARATH Protein FAR1-RELATED SEQUENCE 6 OS=Arabidopsis thaliana GN=FRS6 PE=2 SV=1//2.02491e-07
XLOC_2924sp|Q84W92|ANM13_ARATH Probable histone-arginine methyltransferase 1.3 OS=Arabidopsis thaliana GN=PRMT13 PE=2 SV=3//0
XLOC_2925-//-
XLOC_2926sp|P0C2F6|RNHX1_ARATH Putative ribonuclease H protein At1g65750 OS=Arabidopsis thaliana GN=At1g65750 PE=3 SV=1//4.478
XLOC_2928sp|O65837|LCYE_SOLLC Lycopene epsilon cyclase, chloroplastic OS=Solanum lycopersicum GN=CRTL-E-1 PE=2 SV=1//3.35599e-
XLOC_2928-//-
XLOC_2930sp|Q9SZR0|CHMO_ARATH Choline monooxygenase, chloroplastic OS=Arabidopsis thaliana GN=At4g29890 PE=2 SV=2//9.57009e-39
XLOC_2933-//-
XLOC_2934sp|Q24307|IAP2_DROME Apoptosis 2 inhibitor OS=Drosophila melanogaster GN=Iap2 PE=2 SV=3//1.50706e-06
XLOC_2938sp|Q54SN4|RPAC1_DICDI DNA-directed RNA polymerases I and III subunit rpac1 OS=Dictyostelium discoideum GN=polr1c PE=3
XLOC_2938sp|P49042|VPE_RICCO Vacuolar-processing enzyme OS=Ricinus communis PE=1 SV=1//3.27082e-107
XLOC_2945-//-
XLOC_2946sp|O82201|AP4S_ARATH AP-4 complex subunit sigma OS=Arabidopsis thaliana GN=At2g19790 PE=2 SV=1//1.5702e-42
XLOC_2948sp|Q05654|RTF21_SCHPO Retrotransposable element Tf2 155 kDa protein type 1 OS=Schizosaccharomyces pombe (strain 972 /
XLOC_2948-//-
XLOC_2949-//-
XLOC_2949sp|Q8NI08|NCOA7_HUMAN Nuclear receptor coactivator 7 OS=Homo sapiens GN=NCOA7 PE=1 SV=2//2.33925e-14
XLOC_2951sp|P93401|NU2M_OENBE NADH-ubiquinone oxidoreductase chain 2 OS=Oenothera berteriana GN=ND2 PE=2 SV=2//4.24204e-13
XLOC_2954-//-
XLOC_2955-//-
XLOC_2957sp|Q8W486|Y1491_ARATH Uncharacterized protein At1g04910 OS=Arabidopsis thaliana GN=At1g04910 PE=1 SV=1//6.87926e-09
XLOC_2957sp|Q40392|TMVRN_NICGU TMV resistance protein N OS=Nicotiana glutinosa GN=N PE=1 SV=1//1.20761e-79
XLOC_2957-//-
XLOC_2957sp|Q9FL92|WRK16_ARATH Probable WRKY transcription factor 16 OS=Arabidopsis thaliana GN=WRKY16 PE=2 SV=1//5.78116e-28
XLOC_2957-//-
XLOC_2957-//-
XLOC_2958sp|Q40392|TMVRN_NICGU TMV resistance protein N OS=Nicotiana glutinosa GN=N PE=1 SV=1//5.55396e-27

XLOC_2958 -//-

XLOC_2958 -//-

XLOC_2958 sp|Q40392|TMVRN_NICGU TMV resistance protein N OS=Nicotiana glutinosa GN=N PE=1 SV=1//5.07392e-44

XLOC_2962 sp|O81117|C94A1_VICSA Cytochrome P450 94A1 OS=Vicia sativa GN=CYP94A1 PE=2 SV=2//3.00519e-156

XLOC_2962 -//-

XLOC_2962 sp|Q940Q3|ZTP29_ARATH Zinc transporter ZTP29 OS=Arabidopsis thaliana GN=ZTP29 PE=2 SV=1//3.87363e-19

XLOC_2963 sp|Q9FPW4|VIP2_ARATH Probable NOT transcription complex subunit VIP2 OS=Arabidopsis thaliana GN=VIP2 PE=1 SV=2//9.646

XLOC_2963 sp|O22644|RL23A_FRIAG 60S ribosomal protein L23A OS=Fritillaria agrestis GN=RPL23A PE=2 SV=1//1.80447e-61

XLOC_2963 sp|Q9M2U1|DOF36_ARATH Dof zinc finger protein DOF3.6 OS=Arabidopsis thaliana GN=DOF3.6 PE=1 SV=2//1.15619e-47

XLOC_2963 sp|P21820|TPIS_COPJA Triosephosphate isomerase, cytosolic OS=Coptis japonica PE=2 SV=1//3.27329e-14

XLOC_2963 sp|Q9ZW09|LRK31_ARATH Probable inactive L-type lectin-domain containing receptor kinase III.1 OS=Arabidopsis thaliana

XLOC_2964 -//-

XLOC_2964 sp|Q9LX66|HERK_ARATH Receptor-like protein kinase HERK 1 OS=Arabidopsis thaliana GN=HERK1 PE=1 SV=1//0

XLOC_2964 sp|P41247|PLPL4_HUMAN Patatin-like phospholipase domain-containing protein 4 OS=Homo sapiens GN=PNPLA4 PE=1 SV=3//9.9

XLOC_2964 sp|Q9M5L0|RL35_EUPES 60S ribosomal protein L35 OS=Euphorbia esula GN=RPL35 PE=2 SV=1//4.13425e-73

XLOC_2964 sp|Q9LZ46|H2A4_ARATH Probable histone H2A.4 OS=Arabidopsis thaliana GN=At5g02560 PE=1 SV=1//1.98746e-51

XLOC_2964 sp|Q9LZ46|H2A4_ARATH Probable histone H2A.4 OS=Arabidopsis thaliana GN=At5g02560 PE=1 SV=1//5.08662e-52

XLOC_2964 -//-

XLOC_2964 sp|P0CG89|H4_SOYBN Histone H4 OS=Glycine max PE=3 SV=1//5.37575e-49

XLOC_2964 -//-

XLOC_2965 sp|Q652L2|HIRA_ORYSJ Protein HIRA OS=Oryza sativa subsp. japonica GN=Os09g0567700 PE=2 SV=1//6.06823e-52

XLOC_2965 sp|Q9FLB4|DRL31_ARATH Putative disease resistance protein At5g05400 OS=Arabidopsis thaliana GN=At5g05400 PE=2 SV=1//1

XLOC_2966 sp|Q42798|C93A1_SOYBN Cytochrome P450 93A1 OS=Glycine max GN=CYP93A1 PE=2 SV=1//1.17858e-17

XLOC_2966 sp|P10978|POLX_TOBAC Retrovirus-related Pol polyprotein from transposon TNT 1-94 OS=Nicotiana tabacum PE=2 SV=1//1.43

XLOC_2968 sp|Q6AZB8|HARB1_DANRE Putative nuclease HARBI1 OS=Danio rerio GN=harbi1 PE=2 SV=1//1.24351e-28

XLOC_2970 -//-

XLOC_2970 -//-

XLOC_2970 sp|O64459|UGPA_PYRPY UTP--glucose-1-phosphate uridylyltransferase OS=Pyrus pyrifolia PE=2 SV=1//2.22626e-24

XLOC_2971 -//-

XLOC_2971 sp|O22193|PUB4_ARATH U-box domain-containing protein 4 OS=Arabidopsis thaliana GN=PUB4 PE=1 SV=3//1.77151e-27

XLOC_2972 sp|Q9LNV5|C3H4_ARATH Zinc finger CCCH domain-containing protein 4 OS=Arabidopsis thaliana GN=At1g07360 PE=2 SV=1//1.5

XLOC_2973 -//-

XLOC_2974 sp|P57078|RIPK4_HUMAN Receptor-interacting serine/threonine-protein kinase 4 OS=Homo sapiens GN=RIPK4 PE=1 SV=1//3.43

XLOC_2975 -//-

XLOC_2977 sp|Q8IZ81|ELMD2_HUMAN ELMO domain-containing protein 2 OS=Homo sapiens GN=ELMOD2 PE=1 SV=1//1.41201e-13

XLOC_2977-//-
XLOC_2979sp|Q93W34|RP45C_ARATH Polyadenylate-binding protein RBP45C OS=Arabidopsis thaliana GN=RBP45C PE=2 SV=1//3.36961e-41
XLOC_2982sp|Q55680|Y005_SYNY3 Uncharacterized protein sll0005 OS=Synechocystis sp. (strain PCC 6803 / Kazusa) GN=sll0005 PE=3
XLOC_2983-//-
XLOC_2986sp|P10978|POLX_TOBAC Retrovirus-related Pol polyprotein from transposon TNT 1-94 OS=Nicotiana tabacum PE=2 SV=1//6.38
XLOC_2986-//-
XLOC_2988-//-
XLOC_2991sp|Q6R8G7|PHO13_ARATH Phosphate transporter PHO1 homolog 3 OS=Arabidopsis thaliana GN=PHO1;H3 PE=2 SV=2//3.65096e-25
XLOC_2991-//-
XLOC_2992sp|P33081|AX15A_SOYBN Auxin-induced protein 15A OS=Glycine max PE=2 SV=1//1.11255e-15
XLOC_2994sp|Q9LSF8|C82G1_ARATH Cytochrome P450 82G1 OS=Arabidopsis thaliana GN=CYP82G1 PE=1 SV=1//0
XLOC_2994sp|O95628|CNOT4_HUMAN CCR4-NOT transcription complex subunit 4 OS=Homo sapiens GN=CNOT4 PE=1 SV=3//2.73878e-45
XLOC_2995sp|Q9LSG3|GAUT8_ARATH Galacturonosyltransferase 8 OS=Arabidopsis thaliana GN=GAUT8 PE=1 SV=1//0
XLOC_2995sp|Q09020|PR4_PHAVU Wound-induced basic protein OS=Phaseolus vulgaris GN=PR4 PE=2 SV=1//9.38649e-08
XLOC_2995sp|Q6AWW5|Y5262_ARATH Ankyrin repeat-containing protein At5g02620 OS=Arabidopsis thaliana GN=At5g02620 PE=1 SV=1//1.4
XLOC_2997sp|Q96522|PER45_ARATH Peroxidase 45 OS=Arabidopsis thaliana GN=PER45 PE=1 SV=1//9.36598e-35
XLOC_2999sp|Q9XI05|PSB3A_ARATH Proteasome subunit beta type-3-A OS=Arabidopsis thaliana GN=PBC1 PE=1 SV=2//5.79751e-77
XLOC_2999sp|Q84K11|PPP5_SOLLC Serine/threonine-protein phosphatase 5 OS=Solanum lycopersicum GN=PP5 PE=1 SV=1//1.05374e-20
XLOC_3003sp|P0C1I7|CYP5_RHIO9 Peptidyl-prolyl cis-trans isomerase cyp5 OS=Rhizopus delemar (strain RA 99-880 / ATCC MYA-4621 /
XLOC_3003sp|Q40518|MSK1_TOBAC Shaggy-related protein kinase NtK-1 OS=Nicotiana tabacum GN=NTK-1 PE=2 SV=1//3.97668e-23
XLOC_3003sp|Q6K846|IAA9_ORYSJ Auxin-responsive protein IAA9 OS=Oryza sativa subsp. japonica GN=IAA9 PE=2 SV=1//1.8714e-13
XLOC_3004sp|P08770|TRA1_MAIZE Putative AC transposase OS=Zea mays PE=2 SV=2//2.89065e-79
XLOC_3007-//-
XLOC_3007-//-
XLOC_3008sp|Q94B55|XB31_ARATH Putative E3 ubiquitin-protein ligase XBAT31 OS=Arabidopsis thaliana GN=XBAT31 PE=2 SV=1//5.23344
XLOC_3009sp|Q8GWR3|QKIL5_ARATH KH domain-containing protein At1g09660 OS=Arabidopsis thaliana GN=At1g09660 PE=2 SV=1//1.92419e
XLOC_3009sp|P00054|CYC_SESIN Cytochrome c OS=Sesamum indicum PE=1 SV=1//5.63884e-51
XLOC_3009sp|Q9FYJ2|OSB4_ARATH Protein OSB4, chloroplastic OS=Arabidopsis thaliana GN=OSB4 PE=2 SV=1//9.12265e-42
XLOC_3009-//-
XLOC_3010sp|Q9SQ64|COR2_PAPSO Non-functional NADPH-dependent codeinone reductase 2 OS=Papaver somniferum GN=COR2 PE=1 SV=1//1.
XLOC_3010sp|Q9SAD9|PPR40_ARATH Pentatricopeptide repeat-containing protein At1g13040, mitochondrial OS=Arabidopsis thaliana GN
XLOC_3010sp|P23548|GUN_PAEPO Endoglucanase OS=Paenibacillus polymyxa PE=3 SV=2//1.11842e-07
XLOC_3010-//-
XLOC_3010sp|Q7GB25|AB5C_ARATH ABC transporter C family member 5 OS=Arabidopsis thaliana GN=ABCC5 PE=2 SV=2//1.15113e-20
XLOC_3010sp|Q3MG79|RSGA_ANAVT Putative ribosome biogenesis GTPase RsgA OS=Anabaena variabilis (strain ATCC 29413 / PCC 7937) G

XLOC_3011sp|Q0WPE9|NAT7_ARATH Nucleobase-ascorbate transporter 7 OS=Arabidopsis thaliana GN=NAT7 PE=2 SV=2//2.43172e-89

XLOC_3011sp|Q8RWW0|ALE2_ARATH Receptor-like serine/threonine-protein kinase ALE2 OS=Arabidopsis thaliana GN=ALE2 PE=1 SV=1//3.

XLOC_3011-//-

XLOC_3011sp|Q8W4I4|POT6_ARATH Potassium transporter 6 OS=Arabidopsis thaliana GN=POT6 PE=2 SV=1//0

XLOC_3011sp|O80575|RISB_ARATH 6,7-dimethyl-8-ribityllumazine synthase, chloroplastic OS=Arabidopsis thaliana GN=At2g44050 PE=2

XLOC_3011sp|Q8W486|Y1491_ARATH Uncharacterized protein At1g04910 OS=Arabidopsis thaliana GN=At1g04910 PE=1 SV=1//9.36482e-65

XLOC_3011sp|P09886|HS21C_PEA Small heat shock protein, chloroplastic OS=Pisum sativum GN=HSP21 PE=2 SV=1//5.50667e-35

XLOC_3011-//-

XLOC_3011sp|Q9LU89|2A5N_ARATH Serine/threonine protein phosphatase 2A 59 kDa regulatory subunit B' eta isoform OS=Arabido

XLOC_3012-//-

XLOC_3012sp|Q9SD34|C3H44_ARATH Zinc finger CCCH domain-containing protein 44 OS=Arabidopsis thaliana GN=At3g51120 PE=2 SV=3//2

XLOC_3012sp|P10978|POLX_TOBAC Retrovirus-related Pol polyprotein from transposon TNT 1-94 OS=Nicotiana tabacum PE=2 SV=1//2.24

XLOC_3013sp|Q93W34|RP45C_ARATH Polyadenylate-binding protein RBP45C OS=Arabidopsis thaliana GN=RBP45C PE=2 SV=1//1.89089e-47

XLOC_3014sp|P33543|TMKL1_ARATH Putative kinase-like protein TMKL1 OS=Arabidopsis thaliana GN=TMKL1 PE=1 SV=1//3.56335e-56

XLOC_3019sp|Q9LNG5|PPP7L_ARATH Serine/threonine-protein phosphatase 7 long form homolog OS=Arabidopsis thaliana GN=At1g48120 P

XLOC_3020-//-

XLOC_3020sp|Q6J163|5NG4_PINTA Auxin-induced protein 5NG4 OS=Pinus taeda PE=2 SV=1//2.53074e-19

XLOC_3020-//-

XLOC_3020sp|Q93X17|SNAK2_SOLTU Snakin-2 OS=Solanum tuberosum GN=SN2 PE=1 SV=1//6.93324e-21

XLOC_3020-//-

XLOC_3020sp|Q7G8V2|ARR15_ARATH Two-component response regulator ARR15 OS=Arabidopsis thaliana GN=ARR15 PE=1 SV=1//4.44715e-57

XLOC_3020sp|O04922|GPX2_ARATH Probable glutathione peroxidase 2 OS=Arabidopsis thaliana GN=GPX2 PE=1 SV=1//4.63717e-34

XLOC_3021sp|Q9FKE9|RHD32_ARATH Protein ROOT HAIR DEFECTIVE 3 homolog 2 OS=Arabidopsis thaliana GN=At5g45160 PE=2 SV=1//3.61038

XLOC_3021sp|A2RVM0|TIC32_ARATH Short-chain dehydrogenase TIC 32, chloroplastic OS=Arabidopsis thaliana GN=TIC32 PE=2 SV=1//5.1

XLOC_3021sp|Q9M3G7|ATM_ARATH Serine/threonine-protein kinase ATM OS=Arabidopsis thaliana GN=ATM PE=2 SV=1//2.42332e-156

XLOC_3021sp|Q9M3G7|ATM_ARATH Serine/threonine-protein kinase ATM OS=Arabidopsis thaliana GN=ATM PE=2 SV=1//2.53163e-154

XLOC_3021sp|Q9M3G7|ATM_ARATH Serine/threonine-protein kinase ATM OS=Arabidopsis thaliana GN=ATM PE=2 SV=1//7.3981e-20

XLOC_3021sp|Q9M3G7|ATM_ARATH Serine/threonine-protein kinase ATM OS=Arabidopsis thaliana GN=ATM PE=2 SV=1//3.02124e-68

XLOC_3023-//-

XLOC_3024sp|Q6NWJ4|DNJC2_DANRE DnaJ homolog subfamily C member 2 OS=Danio rerio GN=dnajc2 PE=2 SV=1//2.29888e-11

XLOC_3024sp|Q9CPW7|ZMAT2_MOUSE Zinc finger matrin-type protein 2 OS=Mus musculus GN=Zmat2 PE=2 SV=1//5.16266e-40

XLOC_3025sp|Q8GWL2|LOR7_ARATH Protein LURP-one-related 7 OS=Arabidopsis thaliana GN=At2g30270 PE=2 SV=1//1.4132e-36

XLOC_3025sp|C0LGQ4|MRH1_ARATH Probable LRR receptor-like serine/threonine-protein kinase MRH1 OS=Arabidopsis thaliana GN=MRH1

XLOC_3025sp|F4K1B1|RPAP2_ARATH Putative RNA polymerase II subunit B1 CTD phosphatase RPAP2 homolog OS=Arabidopsis thaliana GN=

XLOC_3025sp|O04834|SAR1A_ARATH GTP-binding protein SAR1A OS=Arabidopsis thaliana GN=SAR1A PE=2 SV=1//1.40913e-112

XLOC_3026	sp|Q9SYJ2|GPAT3_ARATH Probable glycerol-3-phosphate acyltransferase 3 OS=Arabidopsis thaliana GN=GPAT3 PE=2 SV=1//2.8
XLOC_3026	sp|Q9ZPV5|NOC2L_ARATH Nucleolar complex protein 2 homolog OS=Arabidopsis thaliana GN=At2g18220 PE=2 SV=2//1.44304e-31
XLOC_3026	sp|P42211|ASPRX_ORYSJ Aspartic proteinase OS=Oryza sativa subsp. japonica GN=RAP PE=2 SV=2//7.15719e-31
XLOC_3026	-//-
XLOC_3026	-//-
XLOC_3027	-//-
XLOC_3028	sp|Q99315|YG31B_YEAST Transposon Ty3-G Gag-Pol polyprotein OS=Saccharomyces cerevisiae (strain ATCC 204508 / S288c) G
XLOC_3030	-//-
XLOC_3032	sp|O82204|RL281_ARATH 60S ribosomal protein L28-1 OS=Arabidopsis thaliana GN=RPL28A PE=1 SV=1//9.29148e-66
XLOC_3032	-//-
XLOC_3037	sp|P21240|CPNB1_ARATH Chaperonin 60 subunit beta 1, chloroplastic OS=Arabidopsis thaliana GN=CPN60B1 PE=1 SV=3//3.572
XLOC_3039	sp|Q9SEI0|WER_ARATH Transcription factor WER OS=Arabidopsis thaliana GN=WER PE=1 SV=1//7.23107e-49
XLOC_3039	sp|Q0DJS1|TOR_ORYSJ Serine/threonine-protein kinase TOR OS=Oryza sativa subsp. japonica GN=TOR PE=2 SV=3//2.33086e-53
XLOC_3040	sp|Q84WF5|VAMPL_ARATH Probable VAMP-like protein At1g33475 OS=Arabidopsis thaliana GN=At1g33475 PE=2 SV=1//2.26712e-6
XLOC_3041	sp|O49169|EF1A_MANES Elongation factor 1-alpha OS=Manihot esculenta GN=EF1 PE=3 SV=1//7.47641e-83
XLOC_3042	-//-
XLOC_3042	sp|Q17RB8|LONF1_HUMAN LON peptidase N-terminal domain and RING finger protein 1 OS=Homo sapiens GN=LONRF1 PE=2 SV=2//
XLOC_3043	-//-
XLOC_3043	sp|Q552P9|Y5933_DICDI Uncharacterized protein DDB_G0275933 OS=Dictyostelium discoideum GN=DDB_G0275933 PE=3 SV=1//9.3
XLOC_3043	sp|Q8JFV4|HTR5A_DANRE HEAT repeat-containing protein 5A OS=Danio rerio GN=heatr5a PE=2 SV=1//5.94844e-15
XLOC_3043	sp|Q56ZZ7|PLST4_ARATH Plastidic glucose transporter 4 OS=Arabidopsis thaliana GN=At5g16150 PE=1 SV=2//0
XLOC_3044	sp|P10978|POLX_TOBAC Retrovirus-related Pol polyprotein from transposon TNT 1-94 OS=Nicotiana tabacum PE=2 SV=1//1.08
XLOC_3044	sp|P54769|TYDC2_PAPSO Tyrosine/DOPA decarboxylase 2 OS=Papaver somniferum GN=TYDC2 PE=2 SV=1//0
XLOC_3044	-//-
XLOC_3047	-//-

:.22512e-15

;V=1//7.11135e-69

-E71 PE=3 SV=1//1.033e-107

'E=1 SV=1//2.4849e-09

=1//2.14183e-08
i.62268e-17

)psis thaliana GN=LRR-RLK PE=1 SV=1//1.39587e-24
I=At1g07650 PE=1 SV=1//6.10941e-55

)psis thaliana GN=LRR-RLK PE=1 SV=1//3.24384e-92
80381e-08

2204e-105
86128e-16
'/2.63367e-98

//5.7876e-06

'5.64002e-13

8e-09
'5.7541e-19

c) GN=UAF30 PE=1 SV=1//5.02143e-13

07

6

54026e-67

8e-14

//0
01
PE=2 SV=1//5.10143e-150

SV=1//1.37728e-09

//9.10969e-06

54156e-126
07944e-10

GN=RPN9 PE=1 SV=1//1.33228e-06

[illegible]174e-13
[illegible]=At3g47570 PE=1 SV=1//5.42214e-15
[illegible]=1//8.8443e-07

/7.86657e-10
//1.48611e-28

-88

3g03920 PE=2 SV=1//1.83341e-35
353e-13
0

0622 / NRRL Y-65) GN=GYP7 PE=3 SV=1//1.56911e-10
N=TY3B-I PE=3 SV=2//5.2901e-38
E=2 SV=2//1.22995e-20

810000 PE=2 SV=2//1.12546e-32
841e-82

:3
=1//1.36239e-62
3926e-23
e-16
343) GN=gde1 PE=1 SV=1//1.32699e-06

SV=1//1.15245e-10

PE=1 SV=1//2.06111e-21

' ATCC 24843) GN=Tf2-1 PE=2 SV=1//3.0132e-83

2//4.44732e-26
N=YAK1 PE=1 SV=1//2.68279e-10

) PE=1 SV=1//1.78968e-14

aica GN=SPS3 PE=3 SV=1//2.34935e-15
3

?2 PE=1 SV=1//1.82791e-09
.3e-06

.SPG PE=1 SV=1//0
-28

/4.06219e-15
.e-09

39e-21
)7e-40

3 / S288c) GN=FAB1 PE=1 SV=3//8.71155e-22

0

7=1//2.75955e-06

)68e-10

1//1.81036e-67

=At5g48740 PE=2 SV=1//3.55784e-61

90251e-52

396e-20

5e-13

31215e-22
87e-19
35446e-53
//2.55193e-06

/4.42685e-61

/1.12498e-86

c) GN=PIC2 PE=1 SV=1//1.7792e-07

. japonica GN=OST4A PE=3 SV=1//2.06968e-12

V=1//2.32348e-55

e-24

7

253e-36

-38

'9.28652e-12

)086e-33
-81

2.7789e-43

'1.98625e-10

.718e-38

)896e-12

.944e-08

3103e-40

)164e-18

09994e-16

=ALDH6B2 PE=2 SV=2//1.54699e-40

PE=2 SV=1//3.09237e-06

)699e-110
SV=3//1.42173e-57
:2
' ATCC 24843) GN=Tf2-1 PE=2 SV=1//2.31734e-96

)G3 PE=1 SV=1//5.59628e-07
'64e-13

80025e-175

! SV=1//1.89859e-13

369e-10

=At3g03070 PE=3 SV=1//3.34736e-23

1.65288e-49
-09
667e-66

17843e-12

PE=1 SV=1//0

1//8.02087e-51

4.34922e-36

)2357e-08

-12

)G7 PE=1 SV=2//7.838e-112

ι GN=At4g27290 PE=2 SV=4//2.19393e-22

288c) GN=ZIM17 PE=1 SV=2//1.71561e-07
.08294e-27

201e-33

M1 PE=1 SV=1//2.4238e-14
30225e-173
8e-07

017e-20

1 SV=2//7.01221e-80
/6.20845e-07

3.33972e-12
=2//6.5811e-124
08e-18

/3.16105e-27

2//1.83555e-06

/3.35183e-06

1 PE=1 SV=1//6.20852e-14

TCC 24843) GN=nat1 PE=2 SV=1//5.07446e-08

153e-08

31e-08
302e-26
//1.38696e-07

GN=At2g19130 PE=2 SV=1//3.57244e-10
8574e-25

113
N=dinG PE=3 SV=1//1.12528e-07

=1//1.10887e-67

)

5.01138e-27

7/2.12622e-07

E=1 SV=2//1.64077e-82

)95e-33

)6e-19

)63 PE=3 SV=1//1.6892e-07
-07
)8

5362e-40

.94059e-110

49944e-09

83e-45

SV=1//0
PE=1 SV=1//3.43521e-33

GN=RPN9 PE=1 SV=1//2.29762e-06

3

65e-36

374e-13

289e-09

-10
805e-07

V=2//3.43234e-30
ana GN=AAE15 PE=1 SV=1//4.62013e-08
2338e-07

/2.8851e-80
8054e-13

PE=2 SV=1//2.18012e-48

PE=1 SV=1//7.18405e-45

). japonica GN=OST4A PE=3 SV=1//1.50194e-13

PE=1 SV=1//0

'46e-49
:5

'/0
ibsp. japonica GN=LOGL6 PE=2 SV=1//1.59898e-54

-10
79e-06
59e-64

I=At1g67720 PE=2 SV=1//8.20937e-65
dopsis thaliana GN=B'THETA PE=2 SV=2//4.72219e-62

:3

72e-36

,

e-36
:4
=2 SV=2//5.20951e-09

I=At4g08850 PE=1 SV=3//2.29343e-38
ster GN=l(1)G0196 PE=1 SV=2//6.11864e-35

)651e-25
34e-15

5093e-39

:77e-07
3601e-09

'1. 5657e-91
'9. 14337e-132

5869e-98

:2e-121

;V=1//4.65851e-51

!0

:252e-16

;5e-11

PE=3 SV=1//3.03115e-09
04469e-47
;852e-114
;e-155
'669e-49

'5.85168e-26
'1.91513e-06

}90 PE=2 SV=1//2.73015e-32

e-25

N=TY3B-I PE=3 SV=2//3.17964e-51

. japonica GN=OST4A PE=3 SV=1//6.52255e-14

//8.23371e-10

60510 PE=2 SV=1//0

01
18817e-26

1//1.09758e-158

70 PE=1 SV=1//4.39309e-53

534e-39
//2.54432e-18
SV=1//0

1.72927e-22

065e-76
.75383e-22
//4.83479e-69

420 PE=3 SV=2//3.51722e-06

//1.06198e-18

11e-55
599e-77

e-39

ana GN=BCCP2 PE=1 SV=1//1.04105e-19

/2.01934e-22

2.59737e-12

ana GN=AAE15 PE=1 SV=1//1.94462e-07

2//8.86e-51

e-08

8e-44

/6.86116e-12

2//2.73105e-19

3 SV=1//8.17176e-28

I=At4g18975 PE=2 SV=2//2.82043e-15

0594e-10

//0

5559e-46

77897e-16

[illegible]885e-13

[illegible]455e-42
[illegible]
[illegible]E=3 SV=1//1.29265e-24

[illegible]75e-58
[illegible]

[illegible]66e-60

[illegible]406e-51

[illegible] SV=1//6.4641e-66

[illegible]6e-25
[illegible]=ARC6 PE=1 SV=1//3.41494e-31

[illegible]82

[illegible]5396e-100

[illegible].54416e-16

2.5119e-165

158
E=1 SV=1//0

PE=1 SV=1//4.75021e-08
6.59499e-16

At4g29120 PE=2 SV=1//1.12966e-34

ubsp. japonica GN=LOGL6 PE=2 SV=1//8.94697e-54

//7.64215e-22

/ NBRC 0083 / IGC 2968) GN=ATG13 PE=3 SV=2//1.57398e-07
LK1 PE=2 SV=2//1.95183e-128
LK1 PE=2 SV=2//1.45803e-109

252e-14
=1 SV=1//1.37635e-25

.88863e-137

;

'1.30716e-10

PE=1 SV=1//1.46008e-39

:34
PE=2 SV=1//1.22105e-19
557e-11
-09

0375e-16

//5.50728e-62
-53

.22001e-64

'952e-09

07

GN=rsmI PE=3 SV=1//7.26617e-11

. japonica GN=OST4A PE=3 SV=1//1.72759e-13

288e-19
G3 PE=1 SV=1//2.65371e-112

GN=CESA1 PE=2 SV=1//9.79728e-86
=1//2.15901e-111

e-61

GN=At3g03100 PE=1 SV=1//6.33778e-13

e-17

3
/4.7135e-38
39e-27
=At2g26790 PE=2 SV=1//5.4794e-06

/0
=1 SV=1//1.22064e-15

8e-19

ı 972 / ATCC 24843) GN=rpc10 PE=1 SV=2//8.95767e-15

:20 PE=1 SV=1//2.18172e-09

ʲ478e-23
=MCD4 PE=3 SV=1//5.58502e-13

93191e-84
'=1//7.69274e-80

-15
-83
=GATC PE=3 SV=1//1.43321e-54

SV=2//1.98822e-31
?3 PE=1 SV=1//5.32208e-143

3e-16
//3.97898e-12

7612e-08

45908e-16
2302e-12

-17

04e-09

/2.14466e-18

329e-08
M1 PE=1 SV=1//4.193e-15

5.35538e-09
=MAE_39970 PE=3 SV=1//9.30123e-17

/9.59097e-23

35

=1//0

GN=PDS5 PE=1 SV=1//1.28751e-15
.21584e-76

=1//1.83962e-16

26
4135e-16

2.91424e-11

532e-36

//2.60133e-06

382e-14
2

979e-42

7 / DSM 11121 / KW20 / Rd) GN=potD-A PE=3 SV=2//5.17495e-15

588e-27
V=1//6.29573e-71
MCD4 PE=3 SV=1//1.25185e-07

721e-09

V=1//2.37293e-28
=1//2.76995e-12

MCD4 PE=3 SV=1//1.00368e-10

.45595e-10

l=At2g24230 PE=2 SV=1//4.09754e-15

:4e-18

3869e-17

-29

e-17
//3.24651e-87
)5e-52
:2

-55
ıa GN=At5g57850 PE=2 SV=1//2.20824e-149

' FGSC 10173) GN=SNOG_03055 PE=3 SV=1//1.50605e-14

) GN=guaA PE=3 SV=1//2.02006e-44
;1

'E=2 SV=1//2.70399e-16

'/2.49727e-42
'/3.58295e-20
ı114 PE=3 SV=1//3.24875e-11

;78e-24

!7820 PE=2 SV=1//0

'=1//1.43995e-15

31008e-18
'/3.43444e-54

;e-23

2.02211e-135

;993e-28
3209e-75

'9.3227e-48
PDK PE=1 SV=1//2.92193e-37

-27
-66

)225e-14

)

SV=1//4.94665e-33

1//1.51976e-20

0

9

6062e-118

'=2//0

/ JCM 10044 / NBRC 100330 / Delta H) GN=MTH_273 PE=3 SV=1//3.28469e-29

008e-52
)G3 PE=1 SV=1//1.49528e-81

202e-13
882e-25

'674e-28

'=1//1.06413e-70
//7.14818e-16
SV=1//3.81987e-37
88e-19

'E=2 SV=1//6.50794e-32

73295e-24

) GN=creC PE=3 SV=1//1.07653e-60
259e-28

7e-27

871e-42

=1//2.16083e-49

073e-07

3g0242700 PE=2 SV=2//7.00869e-08
3g0242700 PE=2 SV=2//1.03847e-46

SV=1//2.09608e-19

/4.53449e-33
0351e-31
rA PE=1 SV=1//1.34231e-18
31860 PE=2 SV=1//5.18283e-22

585e-35

/6.30331e-34

/=1//5.17342e-77
SV=2//3.10605e-34

1//2.99011e-36
52819e-81

.4e-78

SV=2//9.60975e-10

7237e-152

3534e-22
716e-29

C=1 SV=1//7.09107e-36
C=1 SV=1//6.88527e-19

09

=1//8.73942e-85

18244e-14

E=2 SV=1//1.46834e-26

93431e-12

26e-19

ıA PE=3 SV=1//2.36638e-20
)G5 PE=1 SV=1//2.97587e-106

I=SAP1 PE=1 SV=1//1.52294e-08

)9

=2 SV=2//4.0745e-53

'3.04227e-10

'=1//0

I=At4g14190 PE=2 SV=2//7.2494e-08

5025e-19

)e-08

.6

=1//9.01935e-11

=At4g36180 PE=1 SV=1//5.86173e-07

=1//7.69861e-06

1//1.18081e-21

V=1//4.38504e-24

C PE=1 SV=2//7.73476e-29

-95

7e-07
=1//4.6824e-17

?e-06
7344 / JCM 9153 / C-125) GN=BH0283 PE=3 SV=1//2.09708e-39

88299e-72

'1e-08

SV=2//9.75722e-146

//2.78726e-39
? SV=1//5.01436e-13

SV=2//1.2929e-55

378e-31

)89 / DSM 1224) GN=guaAA PE=3 SV=1//1.29738e-08

7e-12

590 PE=2 SV=1//9.29779e-89

=1//5.24424e-82
3724e-27
270 PE=1 SV=1//1.53335e-52
-11

3
N=TY3B-G PE=1 SV=3//4.24438e-116

21468e-30

=At4g18975 PE=2 SV=2//1.921e-15

//2.698e-146

:At5g26760 PE=2 SV=1//5.72345e-58
:37

//3.30855e-48

:1//1.63058e-24

SV=3//2.70455e-58

5563e-35

SV=1//3.56323e-27

0289e-28

). japonica GN=OST4A PE=3 SV=1//4.58587e-14

38e-77

-15

=1//1.37582e-19

)0

ı 972 / ATCC 24843) GN=SPAP8A3.11c PE=2 SV=1//1.06359e-11

SV=2//8.70359e-135
//1.46256e-06

2

iana GN=At1g17230 PE=2 SV=2//3.33773e-07
SV=1//1.37431e-62

/1.73941e-44

2

28
e-21
e-13

e-164

e-25
=At5g10290 PE=1 SV=1//5.86947e-48
=At5g10290 PE=1 SV=1//1.89088e-15

PE=2 SV=1//3.87709e-83

-58

T1 PE=1 SV=1//1.02678e-34

843) GN=SPAC25G10.01 PE=1 SV=1//1.40009e-06

//8.63797e-42

SV=1//1.71099e-175

4337e-32

' NBRC 13307 / NCIMB 12785 / NRRL 3585 / VKM Ac-602) GN=cefD PE=1 SV=3//1.3162e-23

'E=2 SV=2//2.92379e-78

' ATCC 24843) GN=Tf2-7 PE=2 SV=1//1.96525e-13
//0

95e-135
44

ınkrd44 PE=2 SV=1//5.61998e-08

. SV=1//3.82802e-24
33e-09

;60510 PE=2 SV=1//5.49773e-164
:363e-24

-94
ì.90726e-53

I=At2g31400 PE=2 SV=1//0

'3.90311e-18

591e-13

51e-47

5e-14
ATCC 24843) GN=Tf2-1 PE=2 SV=1//1.69567e-06

na GN=SDH2-3 PE=2 SV=1//1.8068e-38

129e-16

haliana GN=At5g05200 PE=1 SV=1//1.1595e-08
PE=3 SV=1//8.05901e-30

:=2 SV=1//7.02769e-45

i803e-14

)51e-58

:aI2 PE=1 SV=2//4.60338e-10
:-09

SV=2//2.88906e-46
:-49

)41e-34
5753e-25
e-72

5e-10

e-60

e-27

5e-23

:2 SV=1//4.3643e-133

e-41

52e-21
:2
:8e-21

//1.10916e-08

V=1//1.7015e-28

liana GN=At2g25790 PE=1 SV=1//2.25355e-10
4.19134e-16
11

liscoideum GN=captC PE=2 SV=1//1.98908e-43

41e-07

2//1.76905e-10

;2

) PE=1 SV=1//1.65493e-09

e-134

1e-07
e-57
5207e-146
3.08731e-79

OG3 PE=1 SV=1//3.15719e-114

3e-75

57
e-47

5

SV=1//1.78796e-08

)7e-47

.0

/1.89056e-54
.1
3748e-24

//3.49396e-12

.//5.63327e-14

5

:64e-55

066e-98

: 100828) GN=dnaJ PE=3 SV=1//7.23361e-08

aliana GN=At5g48380 PE=1 SV=1//8.32888e-28

SV=2//0
0G5 PE=1 SV=1//1.31399e-98
1 0

. SV=2//9.24908e-124

'79e-16

I=PGR3 PE=1 SV=1//6.68992e-08

.//3.03245e-07

'5.07456e-18

SV=1//3.89071e-13

ına GN=SDH2-2 PE=1 SV=2//3.2638e-171
PE=1 SV=1//0
:208e-40

)927e-34
ına GN=SDH2-2 PE=1 SV=2//1.52066e-169

59767e-18
:278e-27

)499e-34

;824e-11

//5.42706e-28

)96e-104
;4e-28

.09585e-124

)6
:-22

;V=2//5.13283e-10

;e-14

)
//3.18399e-11

SV=1//1.11096e-07

I=At3g04760 PE=2 SV=1//8.72649e-07
E=2 SV=2//1.19326e-105

/3.67591e-18

)3e-11
)62e-06
)215e-103

36104e-112

/8.76952e-06

1e-13

/2.97053e-46

8.26983e-07

95e-07

853e-25

3 PE=1 SV=1//6.13075e-146
222e-58
53775e-06

'/7.41992e-40
5
=1 SV=1//4.62056e-87

.//1.94777e-38

'/3.09475e-15

)6811e-32

2165e-17

[illegible]G5 PE=1 SV=1//2.39716e-104

[illegible]V=1//2.01269e-14

[illegible]155e-29
[illegible]810000 PE=2 SV=2//2.33432e-16
[illegible]

[illegible]
[illegible] PE=1 SV=1//1.55977e-41

[illegible]22801e-12

[illegible]591e-28

). japonica GN=OST4A PE=3 SV=1//6.51994e-13

-11

-122

;V=2//1.37337e-56

)6

=2 SV=1//1.57568e-37

277e-39

GN=TY3B-I PE=3 SV=2//1.28299e-15

)

)227e-17

PE=1 SV=1//1.18312e-28

). japonica GN=OST4A PE=3 SV=1//1.54463e-14

338e-08

.33552e-10

1.32341e-16

0093e-15

013e-10
.77668e-19

5309e-79

=1//1.21392e-06

2e-08
994e-28

e-08

236e-21

684e-14

6

-42

2.66755e-10
9028e-31

'=1//5.45515e-13
:1e-77
SV=1//4.28802e-29

SV=1//1.02108e-19

'=1//0

=1//9.02278e-32
2674e-23

36517e-21
SV=1//3.05745e-38

37262e-102
:.93764e-10
'3019e-21

3
)0034e-12

2499e-56

7958e-11

) PE=1 SV=1//3.98806e-76

1//1.6507e-55

3897e-53
N=SAP4 PE=1 SV=1//2.21952e-47
PE=2 SV=1//3.46471e-09

e-68

238e-29

ı GN=At1g11300 PE=2 SV=1//8.28914e-30
=At1g35710 PE=2 SV=1//4.82326e-14
21

-10

. SV=1//6.11237e-24
'2 / ATCC 24843) GN=SPAC1527.03 PE=1 SV=1//2.82352e-09

I=At1g05670 PE=2 SV=1//1.05408e-06

9e-06
2188e-46

93083e-42
-12

ı GN=trmt61a PE=1 SV=1//3.21666e-12

.44612e-08

'4.58546e-12
:.75752e-88

I=At4g26540 PE=1 SV=1//1.95997e-11

)491e-41
:585e-13
;=2 SV=1//7.78524e-45
:-59
PCC 6803 / Kazusa) GN=slr0328 PE=3 SV=1//6.50287e-23
:-21
:-45
474e-38
, 31084) GN=PMP3 PE=3 SV=2//2.60285e-07

28375e-14
'CC 27264 / PCC 7002 / PR-6) GN=mnmG PE=3 SV=1//1.80712e-38

[illegible]=PCMP-H13 PE=2 SV=3//3.65259e-28

[illegible]306e-06
[illegible]26

[illegible]

[illegible] SV=1//1.60662e-14

[illegible] ATCC 24843) GN=Tf2-1 PE=2 SV=1//1.59854e-85

i6e-30

ı GN=LECRK31 PE=2 SV=1//1.60994e-19

)3927e-14

..00028e-13

i519e-19

i6321e-34

i914e-08

SV=1//3.26343e-11

3109e-07

1444e-178

/ FGSC 9543 / NRRL 43880) GN=cyp5 PE=3 SV=1//2.01412e-25

1e-12
e-29

03263e-77
N=At1g13040 PE=2 SV=1//4.99596e-16

GN=rsgA PE=3 SV=1//7.97875e-52

10699e-09

SV=1//9.39434e-13

psis thaliana GN=B'ETA PE=2 SV=1//0

.19295e-15
15e-34

E=2 SV=1//3.50476e-11

e-23
0625e-06

PE=2 SV=1//1.55782e-06
At5g26760 PE=2 SV=1//4.48922e-18

[illegible]6495e-170

[illegible]N=TY3B-G PE=1 SV=3//8.51343e-114

[illegible]93e-26

[illegible]
[illegible]2

[illegible]8.60055e-09

[illegible]9433e-09

[illegible]27e-48
