# Supplementary material for: Genome-Wide Analysis of Long Noncoding RNAs and Their Responses to Drought Stress in Cotton (Gossypium hirsutum L.)
Source: PLoS One. 2016 Jun 13;11(6):e0156723. doi: 10.1371/journal.pone.0156723 (PMC4905672; doi:10.1371/journal.pone.0156723)
Supplement: S3 Table — (PDF) [file pone.0156723.s005.pdf]

S3 Table Precursors prediction of small RNA molecules

LncRNA_II	source	start	end	strand	E_value	Rfam_accessio	Rfam-id
TCONS_009	Rfam	2	216	+	1.31E-16	RF01848	ACEA_U3
TCONS_007	Rfam	191	443	+	1.02E-19	RF01845	enod40
TCONS_000	Rfam	16	301	+	3.31E-13	RF00028	Intron_gpI
TCONS_002	Rfam	183	261	+	1.42E-09	RF00029	Intron_gpII
TCONS_010	Rfam	7	69	+	6.95E-09	RF00029	Intron_gpII
TCONS_003	Rfam	2490	2566	+	1.10E-06	RF00029	Intron_gpII
TCONS_003	Rfam	417	493	+	1.77E-15	RF01419	IsrR
TCONS_003	Rfam	286	367	+	1.47E-19	RF00073	mir-156
TCONS_003	Rfam	285	366	+	1.47E-19	RF00073	mir-156
TCONS_001	Rfam	551	633	+	1.58E-19	RF00073	mir-156
TCONS_001	Rfam	534	616	+	1.58E-19	RF00073	mir-156
TCONS_004	Rfam	296	380	+	5.16E-17	RF00073	mir-156
TCONS_005	Rfam	270	354	+	5.16E-17	RF00073	mir-156
TCONS_004	Rfam	296	380	-	6.99E-14	RF00073	mir-156
TCONS_005	Rfam	270	354	-	6.99E-14	RF00073	mir-156
TCONS_001	Rfam	551	633	-	2.10E-10	RF00073	mir-156
TCONS_001	Rfam	534	616	-	2.10E-10	RF00073	mir-156
TCONS_003	Rfam	286	367	-	9.10E-09	RF00073	mir-156
TCONS_003	Rfam	285	366	-	9.10E-09	RF00073	mir-156
TCONS_003	Rfam	1038	1238	+	1.29E-40	RF00638	MIR159
TCONS_005	Rfam	973	1162	+	5.69E-29	RF00638	MIR159
TCONS_003	Rfam	1036	1236	-	1.65E-27	RF00638	MIR159
TCONS_005	Rfam	971	1160	-	8.50E-13	RF00638	MIR159
TCONS_009	Rfam	65	153	+	2.38E-18	RF00640	MIR167_1
TCONS_009	Rfam	99	205	+	3.10E-18	RF00640	MIR167_1
TCONS_000	Rfam	106	212	+	3.61E-18	RF00640	MIR167_1
TCONS_000	Rfam	110	216	+	3.61E-18	RF00640	MIR167_1
TCONS_000	Rfam	100	206	+	3.61E-18	RF00640	MIR167_1
TCONS_000	Rfam	107	213	-	2.68E-16	RF00640	MIR167_1
TCONS_000	Rfam	111	217	-	2.68E-16	RF00640	MIR167_1
TCONS_000	Rfam	101	207	-	2.68E-16	RF00640	MIR167_1
TCONS_009	Rfam	100	206	-	4.08E-15	RF00640	MIR167_1
TCONS_009	Rfam	66	154	-	6.84E-14	RF00640	MIR167_1
TCONS_005	Rfam	102	334	+	1.33E-13	RF00640	MIR167_1
TCONS_005	Rfam	103	335	-	1.58E-08	RF00640	MIR167_1
TCONS_010	Rfam	99	263	+	6.01E-35	RF00677	MIR168
TCONS_010	Rfam	98	262	-	8.10E-16	RF00677	MIR168
TCONS_009	Rfam	197	291	-	2.59E-18	RF00645	MIR169_2
TCONS_009	Rfam	199	293	+	9.33E-13	RF00645	MIR169_2
TCONS_009	Rfam	191	299	+	5.97E-14	RF00865	MIR169_5
TCONS_009	Rfam	192	300	-	6.24E-06	RF00865	MIR169_5
TCONS_002	Rfam	813	910	+	2.95E-24	RF00452	mir-172
TCONS_002	Rfam	325	422	+	2.95E-24	RF00452	mir-172
TCONS_009	Rfam	807	904	+	4.47E-23	RF00452	mir-172
TCONS_002	Rfam	22	186	+	7.24E-23	RF00452	mir-172
TCONS_008	Rfam	200	365	+	7.12E-22	RF00452	mir-172
TCONS_002	Rfam	813	910	-	3.64E-20	RF00452	mir-172
TCONS_002	Rfam	325	422	-	3.64E-20	RF00452	mir-172
TCONS_010	Rfam	50	157	+	7.61E-19	RF00452	mir-172
TCONS_009	Rfam	807	904	-	8.01E-19	RF00452	mir-172
TCONS_002	Rfam	22	186	-	3.19E-18	RF00452	mir-172
TCONS_008	Rfam	200	365	-	6.98E-17	RF00452	mir-172

TCONS_01(	Rfam	50	157	-	1.82E-15	RF00452	mir-172
TCONS_00(	Rfam	1	120	+	8.20E-24	RF00689	MIR390
TCONS_00(	Rfam	1	120	-	1.11E-15	RF00689	MIR390
TCONS_00(	Rfam	114	223	+	8.68E-23	RF00688	MIR394
TCONS_00(	Rfam	114	223	-	2.55E-15	RF00688	MIR394
TCONS_00(	Rfam	97	223	+	2.11E-14	RF00451	mir-395
TCONS_00(	Rfam	97	223	-	2.91E-10	RF00451	mir-395
TCONS_00(	Rfam	52	188	+	4.26E-17	RF00445	mir-399
TCONS_00(	Rfam	14	112	+	2.04E-15	RF00445	mir-399
TCONS_00(	Rfam	23	118	+	1.53E-14	RF00445	mir-399
TCONS_00(	Rfam	23	118	-	2.36E-13	RF00445	mir-399
TCONS_00(	Rfam	15	112	-	2.49E-12	RF00445	mir-399
TCONS_00(	Rfam	52	188	-	2.42E-11	RF00445	mir-399
TCONS_007	Rfam	235	338	+	1.54E-21	RF00842	MIR403
TCONS_007	Rfam	233	336	-	5.90E-19	RF00842	MIR403
TCONS_007	Rfam	1428	1527	+	1.10E-22	RF00714	MIR535
TCONS_01(	Rfam	1221	1320	+	8.42E-21	RF00714	MIR535
TCONS_01(	Rfam	1190	1289	+	8.42E-21	RF00714	MIR535
TCONS_01(	Rfam	1292	1391	+	8.42E-21	RF00714	MIR535
TCONS_01(	Rfam	1200	1299	+	8.42E-21	RF00714	MIR535
TCONS_01(	Rfam	1219	1318	-	2.65E-18	RF00714	MIR535
TCONS_01(	Rfam	1188	1287	-	2.65E-18	RF00714	MIR535
TCONS_01(	Rfam	1290	1389	-	2.65E-18	RF00714	MIR535
TCONS_01(	Rfam	1198	1297	-	2.65E-18	RF00714	MIR535
TCONS_007	Rfam	1426	1525	-	4.50E-17	RF00714	MIR535
TCONS_00(	Rfam	1	304	+	7.66E-38	RF01855	Plant_SRP
TCONS_00(	Rfam	1	303	+	1.09E-37	RF01855	Plant_SRP
TCONS_01(	Rfam	1	307	+	1.78E-37	RF01855	Plant_SRP
TCONS_00(	Rfam	2	307	+	3.44E-37	RF01855	Plant_SRP
TCONS_01(	Rfam	1	309	+	8.46E-36	RF01855	Plant_SRP
TCONS_01(	Rfam	1	303	+	1.19E-34	RF01855	Plant_SRP
TCONS_007	Rfam	2	309	+	2.79E-34	RF01855	Plant_SRP
TCONS_01(	Rfam	1	300	+	2.35E-33	RF01855	Plant_SRP
TCONS_00(	Rfam	740	1049	+	1.23E-32	RF01855	Plant_SRP
TCONS_00(	Rfam	2	301	+	1.76E-30	RF01855	Plant_SRP
TCONS_00(	Rfam	1	300	+	7.24E-28	RF01855	Plant_SRP
TCONS_00(	Rfam	1	259	+	1.73E-22	RF01855	Plant_SRP
TCONS_01(	Rfam	1	273	+	1.39E-21	RF01855	Plant_SRP
TCONS_00(	Rfam	2	216	+	4.46E-55	RF01847	Plant_U3
TCONS_00(	Rfam	1	218	+	2.36E-54	RF01847	Plant_U3
TCONS_00(	Rfam	24	238	+	2.33E-47	RF01847	Plant_U3
TCONS_00(	Rfam	1	211	+	1.24E-39	RF01847	Plant_U3
TCONS_00(	Rfam	28	214	+	3.61E-32	RF01847	Plant_U3
TCONS_00(	Rfam	1	203	+	7.29E-30	RF01847	Plant_U3
TCONS_00(	Rfam	24	211	+	2.93E-29	RF01847	Plant_U3
TCONS_00(	Rfam	15	200	+	7.61E-27	RF01847	Plant_U3
TCONS_00(	Rfam	28	208	+	5.66E-26	RF01847	Plant_U3
TCONS_001	Rfam	80	321	+	2.09E-10	RF00030	RNase_MRP
TCONS_00(	Rfam	2405	2509	+	4.35E-18	RF01213	snoR103
TCONS_00(	Rfam	322	428	+	1.30E-16	RF01213	snoR103
TCONS_00(	Rfam	322	428	+	1.30E-16	RF01213	snoR103
TCONS_001	Rfam	283	384	+	1.32E-20	RF01420	snoR113
TCONS_00(	Rfam	162	263	+	2.78E-19	RF01420	snoR113
TCONS_00(	Rfam	428	573	+	5.28E-36	RF01292	snoR2

TCONS_009	Rfam	437	582	+	5.28E-36	RF01292	snoR2
TCONS_004	Rfam	428	573	+	7.68E-36	RF01292	snoR2
TCONS_003	Rfam	300	390	+	9.50E-14	RF01193	snoR20a
TCONS_008	Rfam	306	398	+	8.33E-13	RF01193	snoR20a
TCONS_003	Rfam	135	227	+	8.44E-13	RF01193	snoR20a
TCONS_001	Rfam	449	541	+	8.44E-13	RF01193	snoR20a
TCONS_002	Rfam	104	204	+	9.88E-13	RF00353	snoR31_Z110_Z27
TCONS_004	Rfam	106	197	+	2.66E-17	RF00356	snoR32_R81
TCONS_011	Rfam	295	375	-	1.77E-21	RF01281	snoR35
TCONS_011	Rfam	277	357	-	1.77E-21	RF01281	snoR35
TCONS_011	Rfam	295	375	-	1.77E-21	RF01281	snoR35
TCONS_011	Rfam	295	375	-	1.77E-21	RF01281	snoR35
TCONS_007	Rfam	571	668	+	9.09E-22	RF00267	snoR64
TCONS_003	Rfam	165	242	+	5.15E-11	RF01163	snoR64a
TCONS_001	Rfam	311	391	+	6.21E-11	RF01163	snoR64a
TCONS_008	Rfam	168	248	+	6.62E-11	RF01163	snoR64a
TCONS_003	Rfam	2	77	+	2.40E-09	RF01163	snoR64a
TCONS_010	Rfam	1352	1458	-	4.72E-14	RF00097	snoR71
TCONS_003	Rfam	10013	10119	+	2.12E-13	RF00097	snoR71
TCONS_005	Rfam	3407	3513	-	1.96E-12	RF00097	snoR71
TCONS_002	Rfam	4315	4421	-	4.18E-12	RF00097	snoR71
TCONS_002	Rfam	4070	4176	-	4.18E-12	RF00097	snoR71
TCONS_004	Rfam	1044	1150	-	4.61E-11	RF00097	snoR71
TCONS_004	Rfam	1186	1292	-	4.61E-11	RF00097	snoR71
TCONS_005	Rfam	1202	1308	-	4.28E-10	RF00097	snoR71
TCONS_007	Rfam	4164	4270	-	6.43E-10	RF00097	snoR71
TCONS_004	Rfam	594	686	+	2.84E-17	RF01178	snoR77Y
TCONS_009	Rfam	594	686	+	3.47E-14	RF01178	snoR77Y
TCONS_009	Rfam	603	695	+	3.47E-14	RF01178	snoR77Y
TCONS_007	Rfam	257	334	+	1.33E-11	RF00093	SNORD18
TCONS_007	Rfam	259	335	+	3.16E-07	RF01159	snoU18
TCONS_009	Rfam	155	302	+	8.65E-22	RF01236	snoU19
TCONS_000	Rfam	162	309	+	3.80E-21	RF01236	snoU19
TCONS_004	Rfam	176	240	+	1.14E-17	RF01300	snoU49
TCONS_009	Rfam	198	262	+	1.06E-15	RF01300	snoU49
TCONS_009	Rfam	207	271	+	1.06E-15	RF01300	snoU49
TCONS_004	Rfam	2	57	+	3.94E-10	RF01300	snoU49
TCONS_009	Rfam	197	404	+	4.97E-15	RF00337	snoZ112
TCONS_009	Rfam	206	413	+	4.97E-15	RF00337	snoZ112
TCONS_004	Rfam	179	388	+	7.22E-12	RF00337	snoZ112
TCONS_004	Rfam	761	846	+	2.28E-12	RF00343	snoZ122
TCONS_004	Rfam	764	849	+	2.17E-11	RF00343	snoZ122
TCONS_007	Rfam	722	814	+	2.41E-10	RF00343	snoZ122
TCONS_003	Rfam	763	853	+	1.25E-08	RF00343	snoZ122
TCONS_007	Rfam	97	179	+	2.41E-17	RF00200	snoZ199
TCONS_001	Rfam	420	535	+	3.21E-15	RF00332	snoZ266
TCONS_001	Rfam	1919	1992	+	2.90E-12	RF00005	tRNA
TCONS_003	Rfam	451	522	+	2.90E-12	RF00005	tRNA
TCONS_000	Rfam	177	250	-	3.66E-12	RF00005	tRNA
TCONS_000	Rfam	154	226	-	4.40E-12	RF00005	tRNA
TCONS_010	Rfam	3	76	-	4.55E-12	RF00005	tRNA
TCONS_003	Rfam	2717	2788	+	6.33E-12	RF00005	tRNA
TCONS_000	Rfam	56	127	+	7.04E-12	RF00005	tRNA
TCONS_009	Rfam	44	115	-	1.53E-11	RF00005	tRNA

TCONS_001	Rfam	36	109	+	2.51E-11	RF00005	tRNA
TCONS_007	Rfam	136	208	+	2.69E-11	RF00005	tRNA
TCONS_005	Rfam	2672	2743	-	4.16E-11	RF00005	tRNA
TCONS_005	Rfam	1610	1681	-	4.16E-11	RF00005	tRNA
TCONS_000	Rfam	275	348	+	9.89E-11	RF00005	tRNA
TCONS_005	Rfam	518	589	-	4.27E-09	RF00005	tRNA
TCONS_005	Rfam	553	624	-	4.27E-09	RF00005	tRNA
TCONS_005	Rfam	3255	3324	-	2.16E-08	RF00005	tRNA
TCONS_003	Rfam	2588	2659	-	4.69E-08	RF00005	tRNA
TCONS_007	Rfam	38	123	-	1.19E-06	RF00005	tRNA
TCONS_009	Rfam	2346	2412	-	3.20E-06	RF00005	tRNA
TCONS_006	Rfam	2	141	+	8.11E-20	RF00003	U1
TCONS_000	Rfam	2	193	+	5.53E-50	RF00004	U2
TCONS_010	Rfam	1	184	+	6.74E-44	RF00004	U2
TCONS_006	Rfam	1	186	+	1.09E-43	RF00004	U2
TCONS_004	Rfam	1	184	+	1.34E-43	RF00004	U2
TCONS_011	Rfam	1	185	+	1.08E-41	RF00004	U2
TCONS_006	Rfam	1	185	+	3.71E-41	RF00004	U2
TCONS_006	Rfam	3	177	+	5.94E-36	RF00004	U2
TCONS_009	Rfam	2	215	+	1.00E-16	RF00012	U3
TCONS_007	Rfam	1	136	+	1.92E-18	RF00015	U4
TCONS_008	Rfam	1	135	+	6.33E-18	RF00015	U4
TCONS_008	Rfam	1	135	+	6.33E-18	RF00015	U4
TCONS_008	Rfam	1	135	+	6.33E-18	RF00015	U4
TCONS_010	Rfam	1466	1582	-	3.00E-19	RF00020	U5
TCONS_010	Rfam	122	213	+	6.35E-18	RF00020	U5
TCONS_007	Rfam	442	531	+	1.85E-12	RF00206	U54
TCONS_007	Rfam	25	127	+	4.61E-31	RF00026	U6
TCONS_000	Rfam	2	94	+	2.83E-27	RF00026	U6
TCONS_000	Rfam	1	83	+	4.20E-26	RF00026	U6
TCONS_009	Rfam	4	96	+	4.07E-25	RF00026	U6
TCONS_000	Rfam	1	73	+	4.40E-21	RF00026	U6
TCONS_010	Rfam	1	78	+	1.55E-20	RF00026	U6
TCONS_000	Rfam	623	732	-	1.03E-15	RF00026	U6
TCONS_000	Rfam	853	962	-	1.03E-15	RF00026	U6
TCONS_011	Rfam	1	106	+	1.04E-13	RF00619	U6atac
