# Supplementary material for: Genome-Wide Analysis of Long Noncoding RNAs and Their Responses to Drought Stress in Cotton (Gossypium hirsutum L.)
Source: PLoS One. 2016 Jun 13;11(6):e0156723. doi: 10.1371/journal.pone.0156723 (PMC4905672; doi:10.1371/journal.pone.0156723)
Supplement: S1 Table — (PDF) [file pone.0156723.s003.pdf]

S1 Table Annotation of novel isoforms

At_chr1	Cufflinks	exon	150469	151207	.	+	.	gene_id "XLOC_000019"; transcript_id "Novel_000001"; exon_number "1"; gene_type "
At_chr1	Cufflinks	exon	155018	155265	.	+	.	gene_id "XLOC_000019"; transcript_id "Novel_000001"; exon_number "2"; gene_type "
At_chr1	Cufflinks	exon	250659	251335	.	+	.	gene_id "XLOC_000068"; transcript_id "Novel_000002"; exon_number "1"; gene_type "
At_chr1	Cufflinks	exon	252436	252512	.	+	.	gene_id "XLOC_000068"; transcript_id "Novel_000002"; exon_number "2"; gene_type "
At_chr1	Cufflinks	exon	252626	252866	.	+	.	gene_id "XLOC_000068"; transcript_id "Novel_000002"; exon_number "3"; gene_type "
At_chr1	Cufflinks	exon	252951	253530	.	+	.	gene_id "XLOC_000068"; transcript_id "Novel_000002"; exon_number "4"; gene_type "
At_chr1	Cufflinks	exon	250763	251335	.	+	.	gene_id "XLOC_000068"; transcript_id "Novel_000003"; exon_number "1"; gene_type "
At_chr1	Cufflinks	exon	252436	252512	.	+	.	gene_id "XLOC_000068"; transcript_id "Novel_000003"; exon_number "2"; gene_type "
At_chr1	Cufflinks	exon	252626	252866	.	+	.	gene_id "XLOC_000068"; transcript_id "Novel_000003"; exon_number "3"; gene_type "
At_chr1	Cufflinks	exon	252951	253033	.	+	.	gene_id "XLOC_000068"; transcript_id "Novel_000003"; exon_number "4"; gene_type "
At_chr1	Cufflinks	exon	253111	253689	.	+	.	gene_id "XLOC_000068"; transcript_id "Novel_000003"; exon_number "5"; gene_type "
At_chr1	Cufflinks	exon	319812	320076	.	+	.	gene_id "XLOC_000078"; transcript_id "Novel_000004"; exon_number "1"; gene_type "
At_chr1	Cufflinks	exon	320341	321820	.	+	.	gene_id "XLOC_000078"; transcript_id "Novel_000004"; exon_number "2"; gene_type "
At_chr1	Cufflinks	exon	738853	739155	.	+	.	gene_id "XLOC_000139"; transcript_id "Novel_000005"; exon_number "1"; gene_type "
At_chr1	Cufflinks	exon	739877	740022	.	+	.	gene_id "XLOC_000139"; transcript_id "Novel_000005"; exon_number "2"; gene_type "
At_chr1	Cufflinks	exon	740416	741239	.	+	.	gene_id "XLOC_000139"; transcript_id "Novel_000005"; exon_number "3"; gene_type "
At_chr1	Cufflinks	exon	787719	787949	.	+	.	gene_id "XLOC_000143"; transcript_id "Novel_000006"; exon_number "1"; gene_type "
At_chr1	Cufflinks	exon	788923	789253	.	+	.	gene_id "XLOC_000143"; transcript_id "Novel_000006"; exon_number "2"; gene_type "
At_chr1	Cufflinks	exon	789475	789647	.	+	.	gene_id "XLOC_000143"; transcript_id "Novel_000006"; exon_number "3"; gene_type "
At_chr1	Cufflinks	exon	790044	790719	.	+	.	gene_id "XLOC_000143"; transcript_id "Novel_000006"; exon_number "4"; gene_type "
At_chr1	Cufflinks	exon	787744	787949	.	+	.	gene_id "XLOC_000143"; transcript_id "Novel_000007"; exon_number "1"; gene_type "
At_chr1	Cufflinks	exon	788923	788999	.	+	.	gene_id "XLOC_000143"; transcript_id "Novel_000007"; exon_number "2"; gene_type "
At_chr1	Cufflinks	exon	832040	832083	.	+	.	gene_id "XLOC_000143"; transcript_id "Novel_000007"; exon_number "3"; gene_type "
At_chr1	Cufflinks	exon	787758	787949	.	+	.	gene_id "XLOC_000143"; transcript_id "Novel_000008"; exon_number "1"; gene_type "
At_chr1	Cufflinks	exon	788923	789253	.	+	.	gene_id "XLOC_000143"; transcript_id "Novel_000008"; exon_number "2"; gene_type "
At_chr1	Cufflinks	exon	789475	789647	.	+	.	gene_id "XLOC_000143"; transcript_id "Novel_000008"; exon_number "3"; gene_type "
At_chr1	Cufflinks	exon	833092	833136	.	+	.	gene_id "XLOC_000143"; transcript_id "Novel_000008"; exon_number "4"; gene_type "
At_chr1	Cufflinks	exon	1080501	1080688	.	+	.	gene_id "XLOC_000161"; transcript_id "Novel_000009"; exon_number "1"; gene_type "
At_chr1	Cufflinks	exon	1080778	1080881	.	+	.	gene_id "XLOC_000161"; transcript_id "Novel_000009"; exon_number "2"; gene_type "
At_chr1	Cufflinks	exon	1080966	1081168	.	+	.	gene_id "XLOC_000161"; transcript_id "Novel_000009"; exon_number "3"; gene_type "
At_chr1	Cufflinks	exon	1081256	1081356	.	+	.	gene_id "XLOC_000161"; transcript_id "Novel_000009"; exon_number "4"; gene_type "
At_chr1	Cufflinks	exon	1082155	1082347	.	+	.	gene_id "XLOC_000161"; transcript_id "Novel_000009"; exon_number "5"; gene_type "
At_chr1	Cufflinks	exon	1084207	1084368	.	+	.	gene_id "XLOC_000161"; transcript_id "Novel_000009"; exon_number "6"; gene_type "
At_chr1	Cufflinks	exon	1084506	1084587	.	+	.	gene_id "XLOC_000161"; transcript_id "Novel_000009"; exon_number "7"; gene_type "

At_chr1	Cufflinks	exon	1084685	1085493	.	+	.	gene_id "XLOC_000161"; transcript_id "Novel_000009"; exon_number "8"; gene_type "
At_chr1	Cufflinks	exon	1080507	1080688	.	+	.	gene_id "XLOC_000161"; transcript_id "Novel_000010"; exon_number "1"; gene_type "
At_chr1	Cufflinks	exon	1080778	1081168	.	+	.	gene_id "XLOC_000161"; transcript_id "Novel_000010"; exon_number "2"; gene_type "
At_chr1	Cufflinks	exon	1081256	1081356	.	+	.	gene_id "XLOC_000161"; transcript_id "Novel_000010"; exon_number "3"; gene_type "
At_chr1	Cufflinks	exon	1082155	1082472	.	+	.	gene_id "XLOC_000161"; transcript_id "Novel_000010"; exon_number "4"; gene_type "
At_chr1	Cufflinks	exon	1080510	1080688	.	+	.	gene_id "XLOC_000161"; transcript_id "Novel_000011"; exon_number "1"; gene_type "
At_chr1	Cufflinks	exon	1080778	1080881	.	+	.	gene_id "XLOC_000161"; transcript_id "Novel_000011"; exon_number "2"; gene_type "
At_chr1	Cufflinks	exon	1080966	1081168	.	+	.	gene_id "XLOC_000161"; transcript_id "Novel_000011"; exon_number "3"; gene_type "
At_chr1	Cufflinks	exon	1081256	1081356	.	+	.	gene_id "XLOC_000161"; transcript_id "Novel_000011"; exon_number "4"; gene_type "
At_chr1	Cufflinks	exon	1082155	1082347	.	+	.	gene_id "XLOC_000161"; transcript_id "Novel_000011"; exon_number "5"; gene_type "
At_chr1	Cufflinks	exon	1084207	1084368	.	+	.	gene_id "XLOC_000161"; transcript_id "Novel_000011"; exon_number "6"; gene_type "
At_chr1	Cufflinks	exon	1084506	1084587	.	+	.	gene_id "XLOC_000161"; transcript_id "Novel_000011"; exon_number "7"; gene_type "
At_chr1	Cufflinks	exon	1084685	1085169	.	+	.	gene_id "XLOC_000161"; transcript_id "Novel_000011"; exon_number "8"; gene_type "
At_chr1	Cufflinks	exon	1085253	1086502	.	+	.	gene_id "XLOC_000161"; transcript_id "Novel_000011"; exon_number "9"; gene_type "
At_chr1	Cufflinks	exon	1194255	1194637	.	+	.	gene_id "XLOC_000171"; transcript_id "Novel_000012"; exon_number "1"; gene_type "
At_chr1	Cufflinks	exon	1194724	1195491	.	+	.	gene_id "XLOC_000171"; transcript_id "Novel_000012"; exon_number "2"; gene_type "
At_chr1	Cufflinks	exon	1668327	1668471	.	+	.	gene_id "XLOC_000200"; transcript_id "Novel_000013"; exon_number "1"; gene_type "
At_chr1	Cufflinks	exon	1668917	1669028	.	+	.	gene_id "XLOC_000200"; transcript_id "Novel_000013"; exon_number "2"; gene_type "
At_chr1	Cufflinks	exon	1669433	1672359	.	+	.	gene_id "XLOC_000200"; transcript_id "Novel_000013"; exon_number "3"; gene_type "
At_chr1	Cufflinks	exon	1882013	1883092	.	+	.	gene_id "XLOC_000213"; transcript_id "Novel_000014"; exon_number "1"; gene_type "
At_chr1	Cufflinks	exon	1883229	1883926	.	+	.	gene_id "XLOC_000213"; transcript_id "Novel_000014"; exon_number "2"; gene_type "
At_chr1	Cufflinks	exon	1882080	1882643	.	+	.	gene_id "XLOC_000213"; transcript_id "Novel_000015"; exon_number "1"; gene_type "
At_chr1	Cufflinks	exon	1882823	1883231	.	+	.	gene_id "XLOC_000213"; transcript_id "Novel_000015"; exon_number "2"; gene_type "
At_chr1	Cufflinks	exon	1883214	1884237	.	+	.	gene_id "XLOC_000213"; transcript_id "Novel_000016"; exon_number "1"; gene_type "
At_chr1	Cufflinks	exon	1884707	1884839	.	+	.	gene_id "XLOC_000213"; transcript_id "Novel_000016"; exon_number "2"; gene_type "
At_chr1	Cufflinks	exon	1884970	1885386	.	+	.	gene_id "XLOC_000213"; transcript_id "Novel_000016"; exon_number "3"; gene_type "
At_chr1	Cufflinks	exon	1906899	1906961	.	+	.	gene_id "XLOC_000218"; transcript_id "Novel_000017"; exon_number "1"; gene_type "
At_chr1	Cufflinks	exon	1908243	1908462	.	+	.	gene_id "XLOC_000218"; transcript_id "Novel_000017"; exon_number "2"; gene_type "
At_chr1	Cufflinks	exon	1908533	1910566	.	+	.	gene_id "XLOC_000218"; transcript_id "Novel_000017"; exon_number "3"; gene_type "
At_chr1	Cufflinks	exon	1906915	1906961	.	+	.	gene_id "XLOC_000218"; transcript_id "Novel_000018"; exon_number "1"; gene_type "
At_chr1	Cufflinks	exon	1907368	1908462	.	+	.	gene_id "XLOC_000218"; transcript_id "Novel_000018"; exon_number "2"; gene_type "
At_chr1	Cufflinks	exon	1908533	1910092	.	+	.	gene_id "XLOC_000218"; transcript_id "Novel_000018"; exon_number "3"; gene_type "
At_chr1	Cufflinks	exon	1934909	1936993	.	+	.	gene_id "XLOC_000220"; transcript_id "Novel_000019"; exon_number "1"; gene_type "
At_chr1	Cufflinks	exon	1937071	1937154	.	+	.	gene_id "XLOC_000220"; transcript_id "Novel_000019"; exon_number "2"; gene_type "
At_chr1	Cufflinks	exon	1937403	1937558	.	+	.	gene_id "XLOC_000220"; transcript_id "Novel_000019"; exon_number "3"; gene_type "

At_chr1	Cufflinks	exon	1937643	1937717	.	+	.	gene_id "XLOC_000220"; transcript_id "Novel_000019"; exon_number "4"; gene_type "
At_chr1	Cufflinks	exon	1937889	1937995	.	+	.	gene_id "XLOC_000220"; transcript_id "Novel_000019"; exon_number "5"; gene_type "
At_chr1	Cufflinks	exon	1938161	1938344	.	+	.	gene_id "XLOC_000220"; transcript_id "Novel_000019"; exon_number "6"; gene_type "
At_chr1	Cufflinks	exon	1938601	1938789	.	+	.	gene_id "XLOC_000220"; transcript_id "Novel_000019"; exon_number "7"; gene_type "
At_chr1	Cufflinks	exon	1938899	1938979	.	+	.	gene_id "XLOC_000220"; transcript_id "Novel_000019"; exon_number "8"; gene_type "
At_chr1	Cufflinks	exon	1939254	1939388	.	+	.	gene_id "XLOC_000220"; transcript_id "Novel_000019"; exon_number "9"; gene_type "
At_chr1	Cufflinks	exon	1939623	1939957	.	+	.	gene_id "XLOC_000220"; transcript_id "Novel_000019"; exon_number "10"; gene_type
At_chr1	Cufflinks	exon	1940063	1940168	.	+	.	gene_id "XLOC_000220"; transcript_id "Novel_000019"; exon_number "11"; gene_type
At_chr1	Cufflinks	exon	1940259	1940408	.	+	.	gene_id "XLOC_000220"; transcript_id "Novel_000019"; exon_number "12"; gene_type
At_chr1	Cufflinks	exon	1940493	1940768	.	+	.	gene_id "XLOC_000220"; transcript_id "Novel_000019"; exon_number "13"; gene_type
At_chr1	Cufflinks	exon	1935021	1936393	.	+	.	gene_id "XLOC_000220"; transcript_id "Novel_000020"; exon_number "1"; gene_type "
At_chr1	Cufflinks	exon	1936485	1936575	.	+	.	gene_id "XLOC_000220"; transcript_id "Novel_000020"; exon_number "2"; gene_type "
At_chr1	Cufflinks	exon	1936694	1937154	.	+	.	gene_id "XLOC_000220"; transcript_id "Novel_000020"; exon_number "3"; gene_type "
At_chr1	Cufflinks	exon	1937403	1937558	.	+	.	gene_id "XLOC_000220"; transcript_id "Novel_000020"; exon_number "4"; gene_type "
At_chr1	Cufflinks	exon	1937643	1937717	.	+	.	gene_id "XLOC_000220"; transcript_id "Novel_000020"; exon_number "5"; gene_type "
At_chr1	Cufflinks	exon	1937889	1937995	.	+	.	gene_id "XLOC_000220"; transcript_id "Novel_000020"; exon_number "6"; gene_type "
At_chr1	Cufflinks	exon	1938161	1938344	.	+	.	gene_id "XLOC_000220"; transcript_id "Novel_000020"; exon_number "7"; gene_type "
At_chr1	Cufflinks	exon	1938601	1938789	.	+	.	gene_id "XLOC_000220"; transcript_id "Novel_000020"; exon_number "8"; gene_type "
At_chr1	Cufflinks	exon	1938899	1938979	.	+	.	gene_id "XLOC_000220"; transcript_id "Novel_000020"; exon_number "9"; gene_type "
At_chr1	Cufflinks	exon	1939254	1939388	.	+	.	gene_id "XLOC_000220"; transcript_id "Novel_000020"; exon_number "10"; gene_type
At_chr1	Cufflinks	exon	1939623	1939957	.	+	.	gene_id "XLOC_000220"; transcript_id "Novel_000020"; exon_number "11"; gene_type
At_chr1	Cufflinks	exon	1940063	1940168	.	+	.	gene_id "XLOC_000220"; transcript_id "Novel_000020"; exon_number "12"; gene_type
At_chr1	Cufflinks	exon	1940259	1940408	.	+	.	gene_id "XLOC_000220"; transcript_id "Novel_000020"; exon_number "13"; gene_type
At_chr1	Cufflinks	exon	1940493	1940740	.	+	.	gene_id "XLOC_000220"; transcript_id "Novel_000020"; exon_number "14"; gene_type
At_chr1	Cufflinks	exon	1935965	1936393	.	+	.	gene_id "XLOC_000220"; transcript_id "Novel_000021"; exon_number "1"; gene_type "
At_chr1	Cufflinks	exon	1936485	1936575	.	+	.	gene_id "XLOC_000220"; transcript_id "Novel_000021"; exon_number "2"; gene_type "
At_chr1	Cufflinks	exon	1936694	1936993	.	+	.	gene_id "XLOC_000220"; transcript_id "Novel_000021"; exon_number "3"; gene_type "
At_chr1	Cufflinks	exon	1937071	1937154	.	+	.	gene_id "XLOC_000220"; transcript_id "Novel_000021"; exon_number "4"; gene_type "
At_chr1	Cufflinks	exon	1937403	1937558	.	+	.	gene_id "XLOC_000220"; transcript_id "Novel_000021"; exon_number "5"; gene_type "
At_chr1	Cufflinks	exon	1937643	1937717	.	+	.	gene_id "XLOC_000220"; transcript_id "Novel_000021"; exon_number "6"; gene_type "
At_chr1	Cufflinks	exon	1937889	1937995	.	+	.	gene_id "XLOC_000220"; transcript_id "Novel_000021"; exon_number "7"; gene_type "
At_chr1	Cufflinks	exon	1938161	1938344	.	+	.	gene_id "XLOC_000220"; transcript_id "Novel_000021"; exon_number "8"; gene_type "
At_chr1	Cufflinks	exon	1938601	1938789	.	+	.	gene_id "XLOC_000220"; transcript_id "Novel_000021"; exon_number "9"; gene_type "
At_chr1	Cufflinks	exon	1938899	1938979	.	+	.	gene_id "XLOC_000220"; transcript_id "Novel_000021"; exon_number "10"; gene_type
At_chr1	Cufflinks	exon	1939254	1939388	.	+	.	gene_id "XLOC_000220"; transcript_id "Novel_000021"; exon_number "11"; gene_type

At_chr1	Cufflinks	exon	1939623	1939957	.	+	.	gene_id "XLOC_000220"; transcript_id "Novel_000021"; exon_number "12"; gene_type
At_chr1	Cufflinks	exon	1940063	1940168	.	+	.	gene_id "XLOC_000220"; transcript_id "Novel_000021"; exon_number "13"; gene_type
At_chr1	Cufflinks	exon	1940259	1940408	.	+	.	gene_id "XLOC_000220"; transcript_id "Novel_000021"; exon_number "14"; gene_type
At_chr1	Cufflinks	exon	1940493	1940806	.	+	.	gene_id "XLOC_000220"; transcript_id "Novel_000021"; exon_number "15"; gene_type
At_chr1	Cufflinks	exon	1938864	1939957	.	+	.	gene_id "XLOC_000220"; transcript_id "Novel_000022"; exon_number "1"; gene_type "
At_chr1	Cufflinks	exon	1940063	1940168	.	+	.	gene_id "XLOC_000220"; transcript_id "Novel_000022"; exon_number "2"; gene_type "
At_chr1	Cufflinks	exon	1940259	1940408	.	+	.	gene_id "XLOC_000220"; transcript_id "Novel_000022"; exon_number "3"; gene_type "
At_chr1	Cufflinks	exon	1940493	1940741	.	+	.	gene_id "XLOC_000220"; transcript_id "Novel_000022"; exon_number "4"; gene_type "
At_chr1	Cufflinks	exon	2471459	2471672	.	+	.	gene_id "XLOC_000265"; transcript_id "Novel_000023"; exon_number "1"; gene_type "
At_chr1	Cufflinks	exon	2999462	2999926	.	+	.	gene_id "XLOC_000326"; transcript_id "Novel_000024"; exon_number "1"; gene_type "
At_chr1	Cufflinks	exon	3304340	3304409	.	+	.	gene_id "XLOC_000336"; transcript_id "Novel_000025"; exon_number "1"; gene_type "
At_chr1	Cufflinks	exon	3304696	3305268	.	+	.	gene_id "XLOC_000336"; transcript_id "Novel_000025"; exon_number "2"; gene_type "
At_chr1	Cufflinks	exon	3305936	3306584	.	+	.	gene_id "XLOC_000336"; transcript_id "Novel_000025"; exon_number "3"; gene_type "
At_chr1	Cufflinks	exon	3580897	3581145	.	+	.	gene_id "XLOC_000368"; transcript_id "Novel_000026"; exon_number "1"; gene_type "
At_chr1	Cufflinks	exon	3581437	3583075	.	+	.	gene_id "XLOC_000368"; transcript_id "Novel_000026"; exon_number "2"; gene_type "
At_chr1	Cufflinks	exon	4263363	4263597	.	+	.	gene_id "XLOC_000440"; transcript_id "Novel_000027"; exon_number "1"; gene_type "
At_chr1	Cufflinks	exon	4508397	4508968	.	+	.	gene_id "XLOC_000441"; transcript_id "Novel_000028"; exon_number "1"; gene_type "
At_chr1	Cufflinks	exon	4517497	4517995	.	+	.	gene_id "XLOC_000441"; transcript_id "Novel_000028"; exon_number "2"; gene_type "
At_chr1	Cufflinks	exon	4436677	4437044	.	+	.	gene_id "XLOC_000464"; transcript_id "Novel_000029"; exon_number "1"; gene_type "
At_chr1	Cufflinks	exon	5211094	5212663	.	+	.	gene_id "XLOC_000548"; transcript_id "Novel_000030"; exon_number "1"; gene_type "
At_chr1	Cufflinks	exon	5212870	5212941	.	+	.	gene_id "XLOC_000548"; transcript_id "Novel_000030"; exon_number "2"; gene_type "
At_chr1	Cufflinks	exon	5246960	5247111	.	+	.	gene_id "XLOC_000554"; transcript_id "Novel_000031"; exon_number "1"; gene_type "
At_chr1	Cufflinks	exon	5248991	5249060	.	+	.	gene_id "XLOC_000554"; transcript_id "Novel_000031"; exon_number "2"; gene_type "
At_chr1	Cufflinks	exon	5249177	5249248	.	+	.	gene_id "XLOC_000554"; transcript_id "Novel_000031"; exon_number "3"; gene_type "
At_chr1	Cufflinks	exon	5249356	5249427	.	+	.	gene_id "XLOC_000554"; transcript_id "Novel_000031"; exon_number "4"; gene_type "
At_chr1	Cufflinks	exon	5249553	5249769	.	+	.	gene_id "XLOC_000554"; transcript_id "Novel_000031"; exon_number "5"; gene_type "
At_chr1	Cufflinks	exon	5254512	5254865	.	+	.	gene_id "XLOC_000562"; transcript_id "Novel_000032"; exon_number "1"; gene_type "
At_chr1	Cufflinks	exon	5254990	5255061	.	+	.	gene_id "XLOC_000562"; transcript_id "Novel_000032"; exon_number "2"; gene_type "
At_chr1	Cufflinks	exon	5255167	5255437	.	+	.	gene_id "XLOC_000562"; transcript_id "Novel_000032"; exon_number "3"; gene_type "
At_chr1	Cufflinks	exon	5254619	5254684	.	+	.	gene_id "XLOC_000562"; transcript_id "Novel_000033"; exon_number "1"; gene_type "
At_chr1	Cufflinks	exon	5254794	5254865	.	+	.	gene_id "XLOC_000562"; transcript_id "Novel_000033"; exon_number "2"; gene_type "
At_chr1	Cufflinks	exon	5254990	5255061	.	+	.	gene_id "XLOC_000562"; transcript_id "Novel_000033"; exon_number "3"; gene_type "
At_chr1	Cufflinks	exon	5255167	5255212	.	+	.	gene_id "XLOC_000562"; transcript_id "Novel_000033"; exon_number "4"; gene_type "
At_chr1	Cufflinks	exon	5255729	5255951	.	+	.	gene_id "XLOC_000562"; transcript_id "Novel_000034"; exon_number "1"; gene_type "
At_chr1	Cufflinks	exon	5256196	5256264	.	+	.	gene_id "XLOC_000562"; transcript_id "Novel_000034"; exon_number "2"; gene_type "

At_chr1	Cufflinks	exon	5256349	5256414	.	+	.	gene_id "XLOC_000562"; transcript_id "Novel_000034"; exon_number "3"; gene_type "
At_chr1	Cufflinks	exon	5360663	5360722	.	+	.	gene_id "XLOC_000562"; transcript_id "Novel_000034"; exon_number "4"; gene_type "
At_chr1	Cufflinks	exon	5360818	5360853	.	+	.	gene_id "XLOC_000562"; transcript_id "Novel_000034"; exon_number "5"; gene_type "
At_chr1	Cufflinks	exon	5360927	5360973	.	+	.	gene_id "XLOC_000562"; transcript_id "Novel_000034"; exon_number "6"; gene_type "
At_chr1	Cufflinks	exon	5361057	5361433	.	+	.	gene_id "XLOC_000562"; transcript_id "Novel_000034"; exon_number "7"; gene_type "
At_chr1	Cufflinks	exon	5361563	5361761	.	+	.	gene_id "XLOC_000562"; transcript_id "Novel_000034"; exon_number "8"; gene_type "
At_chr1	Cufflinks	exon	5363374	5364948	.	+	.	gene_id "XLOC_000562"; transcript_id "Novel_000034"; exon_number "9"; gene_type "
At_chr1	Cufflinks	exon	5256773	5257315	.	+	.	gene_id "XLOC_000562"; transcript_id "Novel_000035"; exon_number "1"; gene_type "
At_chr1	Cufflinks	exon	5257454	5257823	.	+	.	gene_id "XLOC_000562"; transcript_id "Novel_000035"; exon_number "2"; gene_type "
At_chr1	Cufflinks	exon	5380109	5380244	.	+	.	gene_id "XLOC_000562"; transcript_id "Novel_000036"; exon_number "1"; gene_type "
At_chr1	Cufflinks	exon	5380498	5380557	.	+	.	gene_id "XLOC_000562"; transcript_id "Novel_000036"; exon_number "2"; gene_type "
At_chr1	Cufflinks	exon	5380655	5380690	.	+	.	gene_id "XLOC_000562"; transcript_id "Novel_000036"; exon_number "3"; gene_type "
At_chr1	Cufflinks	exon	5380764	5380810	.	+	.	gene_id "XLOC_000562"; transcript_id "Novel_000036"; exon_number "4"; gene_type "
At_chr1	Cufflinks	exon	5399962	5400338	.	+	.	gene_id "XLOC_000562"; transcript_id "Novel_000036"; exon_number "5"; gene_type "
At_chr1	Cufflinks	exon	5400483	5400681	.	+	.	gene_id "XLOC_000562"; transcript_id "Novel_000036"; exon_number "6"; gene_type "
At_chr1	Cufflinks	exon	5400801	5400947	.	+	.	gene_id "XLOC_000562"; transcript_id "Novel_000036"; exon_number "7"; gene_type "
At_chr1	Cufflinks	exon	5401146	5401264	.	+	.	gene_id "XLOC_000562"; transcript_id "Novel_000036"; exon_number "8"; gene_type "
At_chr1	Cufflinks	exon	5401349	5401559	.	+	.	gene_id "XLOC_000562"; transcript_id "Novel_000036"; exon_number "9"; gene_type "
At_chr1	Cufflinks	exon	5401671	5401908	.	+	.	gene_id "XLOC_000562"; transcript_id "Novel_000036"; exon_number "10"; gene_type
At_chr1	Cufflinks	exon	5401997	5402147	.	+	.	gene_id "XLOC_000562"; transcript_id "Novel_000036"; exon_number "11"; gene_type
At_chr1	Cufflinks	exon	5402219	5402725	.	+	.	gene_id "XLOC_000562"; transcript_id "Novel_000036"; exon_number "12"; gene_type
At_chr1	Cufflinks	exon	5400956	5401264	.	+	.	gene_id "XLOC_000562"; transcript_id "Novel_000037"; exon_number "1"; gene_type "
At_chr1	Cufflinks	exon	5401349	5401559	.	+	.	gene_id "XLOC_000562"; transcript_id "Novel_000037"; exon_number "2"; gene_type "
At_chr1	Cufflinks	exon	5401671	5401908	.	+	.	gene_id "XLOC_000562"; transcript_id "Novel_000037"; exon_number "3"; gene_type "
At_chr1	Cufflinks	exon	5401997	5402147	.	+	.	gene_id "XLOC_000562"; transcript_id "Novel_000037"; exon_number "4"; gene_type "
At_chr1	Cufflinks	exon	5402219	5402791	.	+	.	gene_id "XLOC_000562"; transcript_id "Novel_000037"; exon_number "5"; gene_type "
At_chr1	Cufflinks	exon	5344930	5345381	.	+	.	gene_id "XLOC_000571"; transcript_id "Novel_000038"; exon_number "1"; gene_type "
At_chr1	Cufflinks	exon	5345501	5346298	.	+	.	gene_id "XLOC_000571"; transcript_id "Novel_000038"; exon_number "2"; gene_type "
At_chr1	Cufflinks	exon	5347994	5348571	.	+	.	gene_id "XLOC_000572"; transcript_id "Novel_000039"; exon_number "1"; gene_type "
At_chr1	Cufflinks	exon	5375239	5375486	.	+	.	gene_id "XLOC_000586"; transcript_id "Novel_000040"; exon_number "1"; gene_type "
At_chr1	Cufflinks	exon	5375575	5375746	.	+	.	gene_id "XLOC_000586"; transcript_id "Novel_000040"; exon_number "2"; gene_type "
At_chr1	Cufflinks	exon	5397416	5397487	.	+	.	gene_id "XLOC_000586"; transcript_id "Novel_000040"; exon_number "3"; gene_type "
At_chr1	Cufflinks	exon	5397596	5397667	.	+	.	gene_id "XLOC_000586"; transcript_id "Novel_000040"; exon_number "4"; gene_type "
At_chr1	Cufflinks	exon	5397804	5397875	.	+	.	gene_id "XLOC_000586"; transcript_id "Novel_000040"; exon_number "5"; gene_type "
At_chr1	Cufflinks	exon	5397986	5398057	.	+	.	gene_id "XLOC_000586"; transcript_id "Novel_000040"; exon_number "6"; gene_type "

At_chr1	Cufflinks	exon	5398166	5398237	.	+	.	gene_id "XLOC_000586"; transcript_id "Novel_000040"; exon_number "7"; gene_type "
At_chr1	Cufflinks	exon	5398388	5398462	.	+	.	gene_id "XLOC_000586"; transcript_id "Novel_000040"; exon_number "8"; gene_type "
At_chr1	Cufflinks	exon	5398573	5398644	.	+	.	gene_id "XLOC_000586"; transcript_id "Novel_000040"; exon_number "9"; gene_type "
At_chr1	Cufflinks	exon	5398753	5398833	.	+	.	gene_id "XLOC_000586"; transcript_id "Novel_000040"; exon_number "10"; gene_type
At_chr1	Cufflinks	exon	5398933	5399004	.	+	.	gene_id "XLOC_000586"; transcript_id "Novel_000040"; exon_number "11"; gene_type
At_chr1	Cufflinks	exon	5399098	5399133	.	+	.	gene_id "XLOC_000586"; transcript_id "Novel_000040"; exon_number "12"; gene_type
At_chr1	Cufflinks	exon	5394280	5394591	.	+	.	gene_id "XLOC_000586"; transcript_id "Novel_000041"; exon_number "1"; gene_type "
At_chr1	Cufflinks	exon	5394680	5394851	.	+	.	gene_id "XLOC_000586"; transcript_id "Novel_000041"; exon_number "2"; gene_type "
At_chr1	Cufflinks	exon	5397416	5397487	.	+	.	gene_id "XLOC_000586"; transcript_id "Novel_000041"; exon_number "3"; gene_type "
At_chr1	Cufflinks	exon	5397596	5397667	.	+	.	gene_id "XLOC_000586"; transcript_id "Novel_000041"; exon_number "4"; gene_type "
At_chr1	Cufflinks	exon	5397804	5397875	.	+	.	gene_id "XLOC_000586"; transcript_id "Novel_000041"; exon_number "5"; gene_type "
At_chr1	Cufflinks	exon	5397986	5398057	.	+	.	gene_id "XLOC_000586"; transcript_id "Novel_000041"; exon_number "6"; gene_type "
At_chr1	Cufflinks	exon	5398166	5398237	.	+	.	gene_id "XLOC_000586"; transcript_id "Novel_000041"; exon_number "7"; gene_type "
At_chr1	Cufflinks	exon	5398388	5398462	.	+	.	gene_id "XLOC_000586"; transcript_id "Novel_000041"; exon_number "8"; gene_type "
At_chr1	Cufflinks	exon	5398573	5398644	.	+	.	gene_id "XLOC_000586"; transcript_id "Novel_000041"; exon_number "9"; gene_type "
At_chr1	Cufflinks	exon	5398753	5398833	.	+	.	gene_id "XLOC_000586"; transcript_id "Novel_000041"; exon_number "10"; gene_type
At_chr1	Cufflinks	exon	5398933	5399004	.	+	.	gene_id "XLOC_000586"; transcript_id "Novel_000041"; exon_number "11"; gene_type
At_chr1	Cufflinks	exon	5399098	5399175	.	+	.	gene_id "XLOC_000586"; transcript_id "Novel_000041"; exon_number "12"; gene_type
At_chr1	Cufflinks	exon	5448974	5449064	.	+	.	gene_id "XLOC_000601"; transcript_id "Novel_000042"; exon_number "1"; gene_type "
At_chr1	Cufflinks	exon	5449148	5449219	.	+	.	gene_id "XLOC_000601"; transcript_id "Novel_000042"; exon_number "2"; gene_type "
At_chr1	Cufflinks	exon	5449311	5449456	.	+	.	gene_id "XLOC_000601"; transcript_id "Novel_000042"; exon_number "3"; gene_type "
At_chr1	Cufflinks	exon	5449986	5450097	.	+	.	gene_id "XLOC_000602"; transcript_id "Novel_000043"; exon_number "1"; gene_type "
At_chr1	Cufflinks	exon	5451060	5451192	.	+	.	gene_id "XLOC_000602"; transcript_id "Novel_000043"; exon_number "2"; gene_type "
At_chr1	Cufflinks	exon	5543440	5543844	.	+	.	gene_id "XLOC_000612"; transcript_id "Novel_000044"; exon_number "1"; gene_type "
At_chr1	Cufflinks	exon	5544314	5544365	.	+	.	gene_id "XLOC_000612"; transcript_id "Novel_000044"; exon_number "2"; gene_type "
At_chr1	Cufflinks	exon	5598791	5598962	.	+	.	gene_id "XLOC_000620"; transcript_id "Novel_000045"; exon_number "1"; gene_type "
At_chr1	Cufflinks	exon	5599365	5599482	.	+	.	gene_id "XLOC_000620"; transcript_id "Novel_000045"; exon_number "2"; gene_type "
At_chr1	Cufflinks	exon	5602938	5603083	.	+	.	gene_id "XLOC_000623"; transcript_id "Novel_000046"; exon_number "1"; gene_type "
At_chr1	Cufflinks	exon	5604816	5607262	.	+	.	gene_id "XLOC_000623"; transcript_id "Novel_000046"; exon_number "2"; gene_type "
At_chr1	Cufflinks	exon	5602938	5603083	.	+	.	gene_id "XLOC_000623"; transcript_id "Novel_000047"; exon_number "1"; gene_type "
At_chr1	Cufflinks	exon	5604816	5606804	.	+	.	gene_id "XLOC_000623"; transcript_id "Novel_000047"; exon_number "2"; gene_type "
At_chr1	Cufflinks	exon	5606896	5607524	.	+	.	gene_id "XLOC_000623"; transcript_id "Novel_000047"; exon_number "3"; gene_type "
At_chr1	Cufflinks	exon	5602938	5603083	.	+	.	gene_id "XLOC_000623"; transcript_id "Novel_000048"; exon_number "1"; gene_type "
At_chr1	Cufflinks	exon	5604816	5606804	.	+	.	gene_id "XLOC_000623"; transcript_id "Novel_000048"; exon_number "2"; gene_type "
At_chr1	Cufflinks	exon	5606896	5606970	.	+	.	gene_id "XLOC_000623"; transcript_id "Novel_000048"; exon_number "3"; gene_type "

At_chr1	Cufflinks	exon	5607048	5607524	.	+	.	gene_id "XLOC_000623"; transcript_id "Novel_000048"; exon_number "4"; gene_type "
At_chr1	Cufflinks	exon	5672329	5673516	.	+	.	gene_id "XLOC_000641"; transcript_id "Novel_000049"; exon_number "1"; gene_type "
At_chr1	Cufflinks	exon	5673592	5673657	.	+	.	gene_id "XLOC_000641"; transcript_id "Novel_000049"; exon_number "2"; gene_type "
At_chr1	Cufflinks	exon	5738958	5738998	.	+	.	gene_id "XLOC_000641"; transcript_id "Novel_000049"; exon_number "3"; gene_type "
At_chr1	Cufflinks	exon	5822888	5822906	.	+	.	gene_id "XLOC_000641"; transcript_id "Novel_000049"; exon_number "4"; gene_type "
At_chr1	Cufflinks	exon	5672329	5673516	.	+	.	gene_id "XLOC_000641"; transcript_id "Novel_000050"; exon_number "1"; gene_type "
At_chr1	Cufflinks	exon	5673592	5673657	.	+	.	gene_id "XLOC_000641"; transcript_id "Novel_000050"; exon_number "2"; gene_type "
At_chr1	Cufflinks	exon	5738958	5738998	.	+	.	gene_id "XLOC_000641"; transcript_id "Novel_000050"; exon_number "3"; gene_type "
At_chr1	Cufflinks	exon	5839298	5839316	.	+	.	gene_id "XLOC_000641"; transcript_id "Novel_000050"; exon_number "4"; gene_type "
At_chr1	Cufflinks	exon	5672334	5673516	.	+	.	gene_id "XLOC_000641"; transcript_id "Novel_000051"; exon_number "1"; gene_type "
At_chr1	Cufflinks	exon	5673592	5673657	.	+	.	gene_id "XLOC_000641"; transcript_id "Novel_000051"; exon_number "2"; gene_type "
At_chr1	Cufflinks	exon	5677727	5677894	.	+	.	gene_id "XLOC_000641"; transcript_id "Novel_000051"; exon_number "3"; gene_type "
At_chr1	Cufflinks	exon	5739126	5739294	.	+	.	gene_id "XLOC_000641"; transcript_id "Novel_000051"; exon_number "4"; gene_type "
At_chr1	Cufflinks	exon	5672443	5673516	.	+	.	gene_id "XLOC_000641"; transcript_id "Novel_000052"; exon_number "1"; gene_type "
At_chr1	Cufflinks	exon	5673592	5673657	.	+	.	gene_id "XLOC_000641"; transcript_id "Novel_000052"; exon_number "2"; gene_type "
At_chr1	Cufflinks	exon	5738958	5739293	.	+	.	gene_id "XLOC_000641"; transcript_id "Novel_000052"; exon_number "3"; gene_type "
At_chr1	Cufflinks	exon	5672459	5673516	.	+	.	gene_id "XLOC_000641"; transcript_id "Novel_000053"; exon_number "1"; gene_type "
At_chr1	Cufflinks	exon	5673592	5673657	.	+	.	gene_id "XLOC_000641"; transcript_id "Novel_000053"; exon_number "2"; gene_type "
At_chr1	Cufflinks	exon	5677727	5677989	.	+	.	gene_id "XLOC_000641"; transcript_id "Novel_000053"; exon_number "3"; gene_type "
At_chr1	Cufflinks	exon	5721404	5721669	.	+	.	gene_id "XLOC_000647"; transcript_id "Novel_000054"; exon_number "1"; gene_type "
At_chr1	Cufflinks	exon	5894229	5894243	.	+	.	gene_id "XLOC_000676"; transcript_id "Novel_000055"; exon_number "1"; gene_type "
At_chr1	Cufflinks	exon	5894341	5895473	.	+	.	gene_id "XLOC_000676"; transcript_id "Novel_000055"; exon_number "2"; gene_type "
At_chr1	Cufflinks	exon	5930321	5930654	.	+	.	gene_id "XLOC_000680"; transcript_id "Novel_000056"; exon_number "1"; gene_type "
At_chr1	Cufflinks	exon	5932645	5932990	.	+	.	gene_id "XLOC_000680"; transcript_id "Novel_000056"; exon_number "2"; gene_type "
At_chr1	Cufflinks	exon	5933349	5933554	.	+	.	gene_id "XLOC_000680"; transcript_id "Novel_000056"; exon_number "3"; gene_type "
At_chr1	Cufflinks	exon	5963345	5963605	.	+	.	gene_id "XLOC_000685"; transcript_id "Novel_000057"; exon_number "1"; gene_type "
At_chr1	Cufflinks	exon	7021160	7021813	.	+	.	gene_id "XLOC_000835"; transcript_id "Novel_000058"; exon_number "1"; gene_type "
At_chr1	Cufflinks	exon	7022402	7023500	.	+	.	gene_id "XLOC_000835"; transcript_id "Novel_000058"; exon_number "2"; gene_type "
At_chr1	Cufflinks	exon	7024071	7024382	.	+	.	gene_id "XLOC_000835"; transcript_id "Novel_000058"; exon_number "3"; gene_type "
At_chr1	Cufflinks	exon	7028302	7028584	.	+	.	gene_id "XLOC_000835"; transcript_id "Novel_000058"; exon_number "4"; gene_type "
At_chr1	Cufflinks	exon	7021169	7021813	.	+	.	gene_id "XLOC_000835"; transcript_id "Novel_000059"; exon_number "1"; gene_type "
At_chr1	Cufflinks	exon	7022402	7023500	.	+	.	gene_id "XLOC_000835"; transcript_id "Novel_000059"; exon_number "2"; gene_type "
At_chr1	Cufflinks	exon	7024071	7025113	.	+	.	gene_id "XLOC_000835"; transcript_id "Novel_000059"; exon_number "3"; gene_type "
At_chr1	Cufflinks	exon	7307420	7307455	.	+	.	gene_id "XLOC_000866"; transcript_id "Novel_000060"; exon_number "1"; gene_type "
At_chr1	Cufflinks	exon	7307593	7307756	.	+	.	gene_id "XLOC_000866"; transcript_id "Novel_000060"; exon_number "2"; gene_type "

At_chr1	Cufflinks	exon	7308319	7308434	.	+	.	gene_id "XLOC_000866"; transcript_id "Novel_000060"; exon_number "3"; gene_type "
At_chr1	Cufflinks	exon	7308925	7310096	.	+	.	gene_id "XLOC_000866"; transcript_id "Novel_000060"; exon_number "4"; gene_type "
At_chr1	Cufflinks	exon	7307449	7307756	.	+	.	gene_id "XLOC_000866"; transcript_id "Novel_000061"; exon_number "1"; gene_type "
At_chr1	Cufflinks	exon	7308319	7308434	.	+	.	gene_id "XLOC_000866"; transcript_id "Novel_000061"; exon_number "2"; gene_type "
At_chr1	Cufflinks	exon	7308925	7309932	.	+	.	gene_id "XLOC_000866"; transcript_id "Novel_000061"; exon_number "3"; gene_type "
At_chr1	Cufflinks	exon	7434785	7435028	.	+	.	gene_id "XLOC_000886"; transcript_id "Novel_000062"; exon_number "1"; gene_type "
At_chr1	Cufflinks	exon	7435709	7436016	.	+	.	gene_id "XLOC_000886"; transcript_id "Novel_000062"; exon_number "2"; gene_type "
At_chr1	Cufflinks	exon	7436277	7436448	.	+	.	gene_id "XLOC_000886"; transcript_id "Novel_000062"; exon_number "3"; gene_type "
At_chr1	Cufflinks	exon	7436540	7436746	.	+	.	gene_id "XLOC_000886"; transcript_id "Novel_000062"; exon_number "4"; gene_type "
At_chr1	Cufflinks	exon	7880990	7881306	.	+	.	gene_id "XLOC_000938"; transcript_id "Novel_000063"; exon_number "1"; gene_type "
At_chr1	Cufflinks	exon	9318470	9318569	.	+	.	gene_id "XLOC_001068"; transcript_id "Novel_000064"; exon_number "1"; gene_type "
At_chr1	Cufflinks	exon	9318659	9318860	.	+	.	gene_id "XLOC_001068"; transcript_id "Novel_000064"; exon_number "2"; gene_type "
At_chr1	Cufflinks	exon	11185502	11185772	.	+	.	gene_id "XLOC_001212"; transcript_id "Novel_000065"; exon_number "1"; gene_type "
At_chr1	Cufflinks	exon	11185908	11186020	.	+	.	gene_id "XLOC_001212"; transcript_id "Novel_000065"; exon_number "2"; gene_type "
At_chr1	Cufflinks	exon	11187899	11187937	.	+	.	gene_id "XLOC_001212"; transcript_id "Novel_000065"; exon_number "3"; gene_type "
At_chr1	Cufflinks	exon	11188769	11188863	.	+	.	gene_id "XLOC_001212"; transcript_id "Novel_000065"; exon_number "4"; gene_type "
At_chr1	Cufflinks	exon	11189928	11190990	.	+	.	gene_id "XLOC_001212"; transcript_id "Novel_000065"; exon_number "5"; gene_type "
At_chr1	Cufflinks	exon	11308656	11309089	.	+	.	gene_id "XLOC_001225"; transcript_id "Novel_000066"; exon_number "1"; gene_type "
At_chr1	Cufflinks	exon	11309340	11309890	.	+	.	gene_id "XLOC_001225"; transcript_id "Novel_000066"; exon_number "2"; gene_type "
At_chr1	Cufflinks	exon	11310140	11310263	.	+	.	gene_id "XLOC_001225"; transcript_id "Novel_000066"; exon_number "3"; gene_type "
At_chr1	Cufflinks	exon	11310468	11310516	.	+	.	gene_id "XLOC_001225"; transcript_id "Novel_000066"; exon_number "4"; gene_type "
At_chr1	Cufflinks	exon	11310590	11310647	.	+	.	gene_id "XLOC_001225"; transcript_id "Novel_000066"; exon_number "5"; gene_type "
At_chr1	Cufflinks	exon	11310734	11310780	.	+	.	gene_id "XLOC_001225"; transcript_id "Novel_000066"; exon_number "6"; gene_type "
At_chr1	Cufflinks	exon	11310875	11310952	.	+	.	gene_id "XLOC_001225"; transcript_id "Novel_000066"; exon_number "7"; gene_type "
At_chr1	Cufflinks	exon	11311630	11312399	.	+	.	gene_id "XLOC_001225"; transcript_id "Novel_000066"; exon_number "8"; gene_type "
At_chr1	Cufflinks	exon	12985755	12986149	.	+	.	gene_id "XLOC_001362"; transcript_id "Novel_000067"; exon_number "1"; gene_type "
At_chr1	Cufflinks	exon	12986292	12986547	.	+	.	gene_id "XLOC_001362"; transcript_id "Novel_000067"; exon_number "2"; gene_type "
At_chr1	Cufflinks	exon	15648610	15648891	.	+	.	gene_id "XLOC_001560"; transcript_id "Novel_000068"; exon_number "1"; gene_type "
At_chr1	Cufflinks	exon	15648974	15649515	.	+	.	gene_id "XLOC_001560"; transcript_id "Novel_000068"; exon_number "2"; gene_type "
At_chr1	Cufflinks	exon	15649611	15649697	.	+	.	gene_id "XLOC_001560"; transcript_id "Novel_000068"; exon_number "3"; gene_type "
At_chr1	Cufflinks	exon	15649790	15649846	.	+	.	gene_id "XLOC_001560"; transcript_id "Novel_000068"; exon_number "4"; gene_type "
At_chr1	Cufflinks	exon	15650006	15650244	.	+	.	gene_id "XLOC_001560"; transcript_id "Novel_000068"; exon_number "5"; gene_type "
At_chr1	Cufflinks	exon	15650317	15650435	.	+	.	gene_id "XLOC_001560"; transcript_id "Novel_000068"; exon_number "6"; gene_type "
At_chr1	Cufflinks	exon	15650552	15650638	.	+	.	gene_id "XLOC_001560"; transcript_id "Novel_000068"; exon_number "7"; gene_type "
At_chr1	Cufflinks	exon	15650726	15651004	.	+	.	gene_id "XLOC_001560"; transcript_id "Novel_000068"; exon_number "8"; gene_type "

At_chr1	Cufflinks	exon	15648611	15649515	.	+	.	gene_id "XLOC_001560"; transcript_id "Novel_000069"; exon_number "1"; gene_type "
At_chr1	Cufflinks	exon	15649611	15649697	.	+	.	gene_id "XLOC_001560"; transcript_id "Novel_000069"; exon_number "2"; gene_type "
At_chr1	Cufflinks	exon	15649790	15649846	.	+	.	gene_id "XLOC_001560"; transcript_id "Novel_000069"; exon_number "3"; gene_type "
At_chr1	Cufflinks	exon	15650006	15650244	.	+	.	gene_id "XLOC_001560"; transcript_id "Novel_000069"; exon_number "4"; gene_type "
At_chr1	Cufflinks	exon	15650317	15650435	.	+	.	gene_id "XLOC_001560"; transcript_id "Novel_000069"; exon_number "5"; gene_type "
At_chr1	Cufflinks	exon	15650552	15650638	.	+	.	gene_id "XLOC_001560"; transcript_id "Novel_000069"; exon_number "6"; gene_type "
At_chr1	Cufflinks	exon	15650726	15651243	.	+	.	gene_id "XLOC_001560"; transcript_id "Novel_000069"; exon_number "7"; gene_type "
At_chr1	Cufflinks	exon	16976488	16976848	.	+	.	gene_id "XLOC_001594"; transcript_id "Novel_000070"; exon_number "1"; gene_type "
At_chr1	Cufflinks	exon	16976956	16977085	.	+	.	gene_id "XLOC_001594"; transcript_id "Novel_000070"; exon_number "2"; gene_type "
At_chr1	Cufflinks	exon	16977188	16978352	.	+	.	gene_id "XLOC_001594"; transcript_id "Novel_000070"; exon_number "3"; gene_type "
At_chr1	Cufflinks	exon	16976560	16976848	.	+	.	gene_id "XLOC_001594"; transcript_id "Novel_000071"; exon_number "1"; gene_type "
At_chr1	Cufflinks	exon	16976956	16977078	.	+	.	gene_id "XLOC_001594"; transcript_id "Novel_000071"; exon_number "2"; gene_type "
At_chr1	Cufflinks	exon	16977188	16978384	.	+	.	gene_id "XLOC_001594"; transcript_id "Novel_000071"; exon_number "3"; gene_type "
At_chr1	Cufflinks	exon	16976573	16977085	.	+	.	gene_id "XLOC_001594"; transcript_id "Novel_000072"; exon_number "1"; gene_type "
At_chr1	Cufflinks	exon	16977188	16978247	.	+	.	gene_id "XLOC_001594"; transcript_id "Novel_000072"; exon_number "2"; gene_type "
At_chr1	Cufflinks	exon	23716824	23717130	.	+	.	gene_id "XLOC_001768"; transcript_id "Novel_000073"; exon_number "1"; gene_type "
At_chr1	Cufflinks	exon	23717241	23718017	.	+	.	gene_id "XLOC_001768"; transcript_id "Novel_000073"; exon_number "2"; gene_type "
At_chr1	Cufflinks	exon	23716951	23717130	.	+	.	gene_id "XLOC_001768"; transcript_id "Novel_000074"; exon_number "1"; gene_type "
At_chr1	Cufflinks	exon	23717241	23717310	.	+	.	gene_id "XLOC_001768"; transcript_id "Novel_000074"; exon_number "2"; gene_type "
At_chr1	Cufflinks	exon	23717414	23718017	.	+	.	gene_id "XLOC_001768"; transcript_id "Novel_000074"; exon_number "3"; gene_type "
At_chr1	Cufflinks	exon	23980696	23981082	.	+	.	gene_id "XLOC_001780"; transcript_id "Novel_000075"; exon_number "1"; gene_type "
At_chr1	Cufflinks	exon	23981900	23981959	.	+	.	gene_id "XLOC_001780"; transcript_id "Novel_000075"; exon_number "2"; gene_type "
At_chr1	Cufflinks	exon	23982027	23984334	.	+	.	gene_id "XLOC_001780"; transcript_id "Novel_000075"; exon_number "3"; gene_type "
At_chr1	Cufflinks	exon	24789379	24789833	.	+	.	gene_id "XLOC_001820"; transcript_id "Novel_000076"; exon_number "1"; gene_type "
At_chr1	Cufflinks	exon	24790993	24791141	.	+	.	gene_id "XLOC_001820"; transcript_id "Novel_000076"; exon_number "2"; gene_type "
At_chr1	Cufflinks	exon	30676981	30677445	.	+	.	gene_id "XLOC_002016"; transcript_id "Novel_000077"; exon_number "1"; gene_type "
At_chr1	Cufflinks	exon	30679363	30680451	.	+	.	gene_id "XLOC_002016"; transcript_id "Novel_000077"; exon_number "2"; gene_type "
At_chr1	Cufflinks	exon	40201293	40201580	.	+	.	gene_id "XLOC_002406"; transcript_id "Novel_000078"; exon_number "1"; gene_type "
At_chr1	Cufflinks	exon	40201778	40201843	.	+	.	gene_id "XLOC_002406"; transcript_id "Novel_000078"; exon_number "2"; gene_type "
At_chr1	Cufflinks	exon	40202577	40203016	.	+	.	gene_id "XLOC_002406"; transcript_id "Novel_000078"; exon_number "3"; gene_type "
At_chr1	Cufflinks	exon	40201318	40201571	.	+	.	gene_id "XLOC_002406"; transcript_id "Novel_000079"; exon_number "1"; gene_type "
At_chr1	Cufflinks	exon	40201778	40201843	.	+	.	gene_id "XLOC_002406"; transcript_id "Novel_000079"; exon_number "2"; gene_type "
At_chr1	Cufflinks	exon	40202577	40202694	.	+	.	gene_id "XLOC_002406"; transcript_id "Novel_000079"; exon_number "3"; gene_type "
At_chr1	Cufflinks	exon	40203718	40203769	.	+	.	gene_id "XLOC_002406"; transcript_id "Novel_000079"; exon_number "4"; gene_type "
At_chr1	Cufflinks	exon	40203875	40204311	.	+	.	gene_id "XLOC_002406"; transcript_id "Novel_000079"; exon_number "5"; gene_type "

At_chr1	Cufflinks	exon	40201349	40201580	.	+	.	gene_id "XLOC_002406"; transcript_id "Novel_000080"; exon_number "1"; gene_type "
At_chr1	Cufflinks	exon	40201778	40201843	.	+	.	gene_id "XLOC_002406"; transcript_id "Novel_000080"; exon_number "2"; gene_type "
At_chr1	Cufflinks	exon	40202577	40202694	.	+	.	gene_id "XLOC_002406"; transcript_id "Novel_000080"; exon_number "3"; gene_type "
At_chr1	Cufflinks	exon	40203718	40203769	.	+	.	gene_id "XLOC_002406"; transcript_id "Novel_000080"; exon_number "4"; gene_type "
At_chr1	Cufflinks	exon	40203875	40204072	.	+	.	gene_id "XLOC_002406"; transcript_id "Novel_000080"; exon_number "5"; gene_type "
At_chr1	Cufflinks	exon	43178845	43179249	.	+	.	gene_id "XLOC_002529"; transcript_id "Novel_000081"; exon_number "1"; gene_type "
At_chr1	Cufflinks	exon	43179344	43179471	.	+	.	gene_id "XLOC_002529"; transcript_id "Novel_000081"; exon_number "2"; gene_type "
At_chr1	Cufflinks	exon	43179578	43179820	.	+	.	gene_id "XLOC_002529"; transcript_id "Novel_000081"; exon_number "3"; gene_type "
At_chr1	Cufflinks	exon	43179911	43180163	.	+	.	gene_id "XLOC_002529"; transcript_id "Novel_000081"; exon_number "4"; gene_type "
At_chr1	Cufflinks	exon	43180586	43180989	.	+	.	gene_id "XLOC_002529"; transcript_id "Novel_000081"; exon_number "5"; gene_type "
At_chr1	Cufflinks	exon	52074297	52074645	.	+	.	gene_id "XLOC_002979"; transcript_id "Novel_000082"; exon_number "1"; gene_type "
At_chr1	Cufflinks	exon	52079658	52081050	.	+	.	gene_id "XLOC_002979"; transcript_id "Novel_000082"; exon_number "2"; gene_type "
At_chr1	Cufflinks	exon	54086836	54087469	.	+	.	gene_id "XLOC_003223"; transcript_id "Novel_000083"; exon_number "1"; gene_type "
At_chr1	Cufflinks	exon	54087967	54088461	.	+	.	gene_id "XLOC_003223"; transcript_id "Novel_000083"; exon_number "2"; gene_type "
At_chr1	Cufflinks	exon	54437877	54437975	.	+	.	gene_id "XLOC_003261"; transcript_id "Novel_000084"; exon_number "1"; gene_type "
At_chr1	Cufflinks	exon	54438531	54438656	.	+	.	gene_id "XLOC_003261"; transcript_id "Novel_000084"; exon_number "2"; gene_type "
At_chr1	Cufflinks	exon	61428978	61429397	.	+	.	gene_id "XLOC_003639"; transcript_id "Novel_000085"; exon_number "1"; gene_type "
At_chr1	Cufflinks	exon	61429908	61430014	.	+	.	gene_id "XLOC_003639"; transcript_id "Novel_000085"; exon_number "2"; gene_type "
At_chr1	Cufflinks	exon	61430677	61431861	.	+	.	gene_id "XLOC_003641"; transcript_id "Novel_000086"; exon_number "1"; gene_type "
At_chr1	Cufflinks	exon	61432031	61433320	.	+	.	gene_id "XLOC_003641"; transcript_id "Novel_000086"; exon_number "2"; gene_type "
At_chr1	Cufflinks	exon	61430677	61431593	.	+	.	gene_id "XLOC_003641"; transcript_id "Novel_000087"; exon_number "1"; gene_type "
At_chr1	Cufflinks	exon	61432031	61433320	.	+	.	gene_id "XLOC_003641"; transcript_id "Novel_000087"; exon_number "2"; gene_type "
At_chr1	Cufflinks	exon	62903659	62903913	.	+	.	gene_id "XLOC_003687"; transcript_id "Novel_000088"; exon_number "1"; gene_type "
At_chr1	Cufflinks	exon	65799268	65799653	.	+	.	gene_id "XLOC_003775"; transcript_id "Novel_000089"; exon_number "1"; gene_type "
At_chr1	Cufflinks	exon	65799732	65800107	.	+	.	gene_id "XLOC_003775"; transcript_id "Novel_000089"; exon_number "2"; gene_type "
At_chr1	Cufflinks	exon	67974745	67974900	.	+	.	gene_id "XLOC_003874"; transcript_id "Novel_000090"; exon_number "1"; gene_type "
At_chr1	Cufflinks	exon	67975012	67975547	.	+	.	gene_id "XLOC_003874"; transcript_id "Novel_000090"; exon_number "2"; gene_type "
At_chr1	Cufflinks	exon	73129878	73130276	.	+	.	gene_id "XLOC_004041"; transcript_id "Novel_000091"; exon_number "1"; gene_type "
At_chr1	Cufflinks	exon	73130371	73130506	.	+	.	gene_id "XLOC_004041"; transcript_id "Novel_000091"; exon_number "2"; gene_type "
At_chr1	Cufflinks	exon	73130596	73130710	.	+	.	gene_id "XLOC_004041"; transcript_id "Novel_000091"; exon_number "3"; gene_type "
At_chr1	Cufflinks	exon	83334317	83334534	.	+	.	gene_id "XLOC_004462"; transcript_id "Novel_000092"; exon_number "1"; gene_type "
At_chr1	Cufflinks	exon	85419262	85420812	.	+	.	gene_id "XLOC_004586"; transcript_id "Novel_000093"; exon_number "1"; gene_type "
At_chr1	Cufflinks	exon	90690136	90690478	.	+	.	gene_id "XLOC_004912"; transcript_id "Novel_000094"; exon_number "1"; gene_type "
At_chr1	Cufflinks	exon	90690599	90690817	.	+	.	gene_id "XLOC_004912"; transcript_id "Novel_000094"; exon_number "2"; gene_type "
At_chr1	Cufflinks	exon	90694958	90695399	.	+	.	gene_id "XLOC_004912"; transcript_id "Novel_000094"; exon_number "3"; gene_type "

At_chr1	Cufflinks	exon	1.02E+08	1.02E+08	.	+	.	gene_id	"XLOC_005370";	transcript_id	"Novel_000095";	exon_number	"1";	gene_type	"
At_chr1	Cufflinks	exon	1.05E+08	1.05E+08	.	+	.	gene_id	"XLOC_005487";	transcript_id	"Novel_000096";	exon_number	"1";	gene_type	"
At_chr1	Cufflinks	exon	1.05E+08	1.05E+08	.	+	.	gene_id	"XLOC_005487";	transcript_id	"Novel_000096";	exon_number	"2";	gene_type	"
At_chr1	Cufflinks	exon	1.05E+08	1.05E+08	.	+	.	gene_id	"XLOC_005487";	transcript_id	"Novel_000096";	exon_number	"3";	gene_type	"
At_chr1	Cufflinks	exon	1.05E+08	1.05E+08	.	+	.	gene_id	"XLOC_005487";	transcript_id	"Novel_000096";	exon_number	"4";	gene_type	"
At_chr1	Cufflinks	exon	1.05E+08	1.05E+08	.	+	.	gene_id	"XLOC_005487";	transcript_id	"Novel_000097";	exon_number	"1";	gene_type	"
At_chr1	Cufflinks	exon	1.05E+08	1.05E+08	.	+	.	gene_id	"XLOC_005487";	transcript_id	"Novel_000097";	exon_number	"2";	gene_type	"
At_chr1	Cufflinks	exon	1.05E+08	1.05E+08	.	+	.	gene_id	"XLOC_005487";	transcript_id	"Novel_000097";	exon_number	"3";	gene_type	"
At_chr1	Cufflinks	exon	1.05E+08	1.05E+08	.	+	.	gene_id	"XLOC_005487";	transcript_id	"Novel_000097";	exon_number	"4";	gene_type	"
At_chr1	Cufflinks	exon	1.06E+08	1.06E+08	.	+	.	gene_id	"XLOC_005547";	transcript_id	"Novel_000098";	exon_number	"1";	gene_type	"
At_chr1	Cufflinks	exon	1.06E+08	1.06E+08	.	+	.	gene_id	"XLOC_005549";	transcript_id	"Novel_000099";	exon_number	"1";	gene_type	"
At_chr1	Cufflinks	exon	1.06E+08	1.06E+08	.	+	.	gene_id	"XLOC_005549";	transcript_id	"Novel_000099";	exon_number	"2";	gene_type	"
At_chr1	Cufflinks	exon	1.07E+08	1.07E+08	.	+	.	gene_id	"XLOC_005704";	transcript_id	"Novel_000100";	exon_number	"1";	gene_type	"
At_chr1	Cufflinks	exon	1.07E+08	1.07E+08	.	+	.	gene_id	"XLOC_005704";	transcript_id	"Novel_000100";	exon_number	"2";	gene_type	"
At_chr1	Cufflinks	exon	1.07E+08	1.07E+08	.	+	.	gene_id	"XLOC_005704";	transcript_id	"Novel_000100";	exon_number	"3";	gene_type	"
At_chr1	Cufflinks	exon	1.07E+08	1.07E+08	.	+	.	gene_id	"XLOC_005704";	transcript_id	"Novel_000100";	exon_number	"4";	gene_type	"
At_chr1	Cufflinks	exon	1.07E+08	1.07E+08	.	+	.	gene_id	"XLOC_005704";	transcript_id	"Novel_000100";	exon_number	"5";	gene_type	"
At_chr1	Cufflinks	exon	1.07E+08	1.07E+08	.	+	.	gene_id	"XLOC_005728";	transcript_id	"Novel_000101";	exon_number	"1";	gene_type	"
At_chr1	Cufflinks	exon	1.07E+08	1.07E+08	.	+	.	gene_id	"XLOC_005728";	transcript_id	"Novel_000101";	exon_number	"2";	gene_type	"
At_chr1	Cufflinks	exon	1.07E+08	1.07E+08	.	+	.	gene_id	"XLOC_005728";	transcript_id	"Novel_000101";	exon_number	"3";	gene_type	"
At_chr1	Cufflinks	exon	382805	386666	.	-	.	gene_id	"XLOC_005793";	transcript_id	"Novel_000102";	exon_number	"1";	gene_type	"
At_chr1	Cufflinks	exon	798668	799535	.	-	.	gene_id	"XLOC_005852";	transcript_id	"Novel_000103";	exon_number	"1";	gene_type	"
At_chr1	Cufflinks	exon	799631	799927	.	-	.	gene_id	"XLOC_005852";	transcript_id	"Novel_000103";	exon_number	"2";	gene_type	"
At_chr1	Cufflinks	exon	801475	801763	.	-	.	gene_id	"XLOC_005852";	transcript_id	"Novel_000104";	exon_number	"1";	gene_type	"
At_chr1	Cufflinks	exon	801860	802103	.	-	.	gene_id	"XLOC_005852";	transcript_id	"Novel_000104";	exon_number	"2";	gene_type	"
At_chr1	Cufflinks	exon	802215	802543	.	-	.	gene_id	"XLOC_005852";	transcript_id	"Novel_000104";	exon_number	"3";	gene_type	"
At_chr1	Cufflinks	exon	802774	803068	.	-	.	gene_id	"XLOC_005852";	transcript_id	"Novel_000104";	exon_number	"4";	gene_type	"
At_chr1	Cufflinks	exon	836013	836601	.	-	.	gene_id	"XLOC_005853";	transcript_id	"Novel_000105";	exon_number	"1";	gene_type	"
At_chr1	Cufflinks	exon	836719	836966	.	-	.	gene_id	"XLOC_005853";	transcript_id	"Novel_000105";	exon_number	"2";	gene_type	"
At_chr1	Cufflinks	exon	837061	837367	.	-	.	gene_id	"XLOC_005853";	transcript_id	"Novel_000105";	exon_number	"3";	gene_type	"
At_chr1	Cufflinks	exon	837477	837572	.	-	.	gene_id	"XLOC_005853";	transcript_id	"Novel_000105";	exon_number	"4";	gene_type	"
At_chr1	Cufflinks	exon	838871	839349	.	-	.	gene_id	"XLOC_005853";	transcript_id	"Novel_000105";	exon_number	"5";	gene_type	"
At_chr1	Cufflinks	exon	867162	867237	.	-	.	gene_id	"XLOC_005861";	transcript_id	"Novel_000106";	exon_number	"1";	gene_type	"
At_chr1	Cufflinks	exon	867349	867647	.	-	.	gene_id	"XLOC_005861";	transcript_id	"Novel_000106";	exon_number	"2";	gene_type	"
At_chr1	Cufflinks	exon	889020	889486	.	-	.	gene_id	"XLOC_005866";	transcript_id	"Novel_000107";	exon_number	"1";	gene_type	"

At_chr1	Cufflinks	exon	889591	889670	.	-	.	gene_id "XLOC_005866"; transcript_id "Novel_000107"; exon_number "2"; gene_type "
At_chr1	Cufflinks	exon	889761	890256	.	-	.	gene_id "XLOC_005866"; transcript_id "Novel_000107"; exon_number "3"; gene_type "
At_chr1	Cufflinks	exon	890345	890414	.	-	.	gene_id "XLOC_005866"; transcript_id "Novel_000107"; exon_number "4"; gene_type "
At_chr1	Cufflinks	exon	891279	891442	.	-	.	gene_id "XLOC_005866"; transcript_id "Novel_000107"; exon_number "5"; gene_type "
At_chr1	Cufflinks	exon	1019781	1020001	.	-	.	gene_id "XLOC_005875"; transcript_id "Novel_000108"; exon_number "1"; gene_type "
At_chr1	Cufflinks	exon	1332934	1335909	.	-	.	gene_id "XLOC_005924"; transcript_id "Novel_000109"; exon_number "1"; gene_type "
At_chr1	Cufflinks	exon	1337359	1337590	.	-	.	gene_id "XLOC_005924"; transcript_id "Novel_000109"; exon_number "2"; gene_type "
At_chr1	Cufflinks	exon	1332934	1335694	.	-	.	gene_id "XLOC_005924"; transcript_id "Novel_000110"; exon_number "1"; gene_type "
At_chr1	Cufflinks	exon	1335795	1335909	.	-	.	gene_id "XLOC_005924"; transcript_id "Novel_000110"; exon_number "2"; gene_type "
At_chr1	Cufflinks	exon	1337359	1337590	.	-	.	gene_id "XLOC_005924"; transcript_id "Novel_000110"; exon_number "3"; gene_type "
At_chr1	Cufflinks	exon	1383901	1384110	.	-	.	gene_id "XLOC_005937"; transcript_id "Novel_000111"; exon_number "1"; gene_type "
At_chr1	Cufflinks	exon	1489820	1493084	.	-	.	gene_id "XLOC_005953"; transcript_id "Novel_000112"; exon_number "1"; gene_type "
At_chr1	Cufflinks	exon	1805153	1805509	.	-	.	gene_id "XLOC_005992"; transcript_id "Novel_000113"; exon_number "1"; gene_type "
At_chr1	Cufflinks	exon	2006032	2006604	.	-	.	gene_id "XLOC_006005"; transcript_id "Novel_000114"; exon_number "1"; gene_type "
At_chr1	Cufflinks	exon	2006698	2007009	.	-	.	gene_id "XLOC_006005"; transcript_id "Novel_000114"; exon_number "2"; gene_type "
At_chr1	Cufflinks	exon	2040784	2041262	.	-	.	gene_id "XLOC_006014"; transcript_id "Novel_000115"; exon_number "1"; gene_type "
At_chr1	Cufflinks	exon	2041373	2042797	.	-	.	gene_id "XLOC_006014"; transcript_id "Novel_000115"; exon_number "2"; gene_type "
At_chr1	Cufflinks	exon	2134698	2135004	.	-	.	gene_id "XLOC_006040"; transcript_id "Novel_000116"; exon_number "1"; gene_type "
At_chr1	Cufflinks	exon	2135083	2135194	.	-	.	gene_id "XLOC_006040"; transcript_id "Novel_000116"; exon_number "2"; gene_type "
At_chr1	Cufflinks	exon	2135345	2135637	.	-	.	gene_id "XLOC_006040"; transcript_id "Novel_000116"; exon_number "3"; gene_type "
At_chr1	Cufflinks	exon	2135748	2135797	.	-	.	gene_id "XLOC_006040"; transcript_id "Novel_000116"; exon_number "4"; gene_type "
At_chr1	Cufflinks	exon	2136044	2136068	.	-	.	gene_id "XLOC_006040"; transcript_id "Novel_000116"; exon_number "5"; gene_type "
At_chr1	Cufflinks	exon	2136409	2136605	.	-	.	gene_id "XLOC_006041"; transcript_id "Novel_000117"; exon_number "1"; gene_type "
At_chr1	Cufflinks	exon	2136724	2137211	.	-	.	gene_id "XLOC_006041"; transcript_id "Novel_000117"; exon_number "2"; gene_type "
At_chr1	Cufflinks	exon	2742476	2743913	.	-	.	gene_id "XLOC_006087"; transcript_id "Novel_000118"; exon_number "1"; gene_type "
At_chr1	Cufflinks	exon	2744905	2744945	.	-	.	gene_id "XLOC_006087"; transcript_id "Novel_000118"; exon_number "2"; gene_type "
At_chr1	Cufflinks	exon	2746658	2747327	.	-	.	gene_id "XLOC_006087"; transcript_id "Novel_000118"; exon_number "3"; gene_type "
At_chr1	Cufflinks	exon	2775310	2776221	.	-	.	gene_id "XLOC_006097"; transcript_id "Novel_000119"; exon_number "1"; gene_type "
At_chr1	Cufflinks	exon	2870379	2870759	.	-	.	gene_id "XLOC_006128"; transcript_id "Novel_000120"; exon_number "1"; gene_type "
At_chr1	Cufflinks	exon	2871285	2871597	.	-	.	gene_id "XLOC_006128"; transcript_id "Novel_000120"; exon_number "2"; gene_type "
At_chr1	Cufflinks	exon	2877189	2879339	.	-	.	gene_id "XLOC_006129"; transcript_id "Novel_000121"; exon_number "1"; gene_type "
At_chr1	Cufflinks	exon	3441495	3441708	.	-	.	gene_id "XLOC_006178"; transcript_id "Novel_000122"; exon_number "1"; gene_type "
At_chr1	Cufflinks	exon	3535165	3536313	.	-	.	gene_id "XLOC_006191"; transcript_id "Novel_000123"; exon_number "1"; gene_type "
At_chr1	Cufflinks	exon	3536575	3537143	.	-	.	gene_id "XLOC_006191"; transcript_id "Novel_000123"; exon_number "2"; gene_type "
At_chr1	Cufflinks	exon	3550179	3551482	.	-	.	gene_id "XLOC_006198"; transcript_id "Novel_000124"; exon_number "1"; gene_type "

At_chr1	Cufflinks	exon	3551620	3552140	.	-	.	gene_id "XLOC_006198"; transcript_id "Novel_000124"; exon_number "2"; gene_type "
At_chr1	Cufflinks	exon	3550179	3551861	.	-	.	gene_id "XLOC_006198"; transcript_id "Novel_000125"; exon_number "1"; gene_type "
At_chr1	Cufflinks	exon	3552511	3552551	.	-	.	gene_id "XLOC_006198"; transcript_id "Novel_000125"; exon_number "2"; gene_type "
At_chr1	Cufflinks	exon	3554278	3555185	.	-	.	gene_id "XLOC_006198"; transcript_id "Novel_000125"; exon_number "3"; gene_type "
At_chr1	Cufflinks	exon	3550304	3551861	.	-	.	gene_id "XLOC_006198"; transcript_id "Novel_000126"; exon_number "1"; gene_type "
At_chr1	Cufflinks	exon	3552511	3555210	.	-	.	gene_id "XLOC_006198"; transcript_id "Novel_000126"; exon_number "2"; gene_type "
At_chr1	Cufflinks	exon	3552356	3552551	.	-	.	gene_id "XLOC_006198"; transcript_id "Novel_000127"; exon_number "1"; gene_type "
At_chr1	Cufflinks	exon	3554278	3555107	.	-	.	gene_id "XLOC_006198"; transcript_id "Novel_000127"; exon_number "2"; gene_type "
At_chr1	Cufflinks	exon	3557092	3557695	.	-	.	gene_id "XLOC_006199"; transcript_id "Novel_000128"; exon_number "1"; gene_type "
At_chr1	Cufflinks	exon	3558889	3558969	.	-	.	gene_id "XLOC_006199"; transcript_id "Novel_000128"; exon_number "2"; gene_type "
At_chr1	Cufflinks	exon	3559835	3559985	.	-	.	gene_id "XLOC_006199"; transcript_id "Novel_000128"; exon_number "3"; gene_type "
At_chr1	Cufflinks	exon	3574112	3575274	.	-	.	gene_id "XLOC_006201"; transcript_id "Novel_000129"; exon_number "1"; gene_type "
At_chr1	Cufflinks	exon	3575514	3576195	.	-	.	gene_id "XLOC_006201"; transcript_id "Novel_000129"; exon_number "2"; gene_type "
At_chr1	Cufflinks	exon	3602762	3602970	.	-	.	gene_id "XLOC_006220"; transcript_id "Novel_000130"; exon_number "1"; gene_type "
At_chr1	Cufflinks	exon	3638163	3638245	.	-	.	gene_id "XLOC_006233"; transcript_id "Novel_000131"; exon_number "1"; gene_type "
At_chr1	Cufflinks	exon	3638894	3641430	.	-	.	gene_id "XLOC_006233"; transcript_id "Novel_000131"; exon_number "2"; gene_type "
At_chr1	Cufflinks	exon	3638732	3639661	.	-	.	gene_id "XLOC_006233"; transcript_id "Novel_000132"; exon_number "1"; gene_type "
At_chr1	Cufflinks	exon	3639795	3641381	.	-	.	gene_id "XLOC_006233"; transcript_id "Novel_000132"; exon_number "2"; gene_type "
At_chr1	Cufflinks	exon	3642211	3644330	.	-	.	gene_id "XLOC_006233"; transcript_id "Novel_000133"; exon_number "1"; gene_type "
At_chr1	Cufflinks	exon	4421991	4422308	.	-	.	gene_id "XLOC_006298"; transcript_id "Novel_000134"; exon_number "1"; gene_type "
At_chr1	Cufflinks	exon	4973969	4973999	.	-	.	gene_id "XLOC_006356"; transcript_id "Novel_000135"; exon_number "1"; gene_type "
At_chr1	Cufflinks	exon	4983061	4983922	.	-	.	gene_id "XLOC_006356"; transcript_id "Novel_000135"; exon_number "2"; gene_type "
At_chr1	Cufflinks	exon	5013740	5013855	.	-	.	gene_id "XLOC_006362"; transcript_id "Novel_000136"; exon_number "1"; gene_type "
At_chr1	Cufflinks	exon	5025735	5025918	.	-	.	gene_id "XLOC_006362"; transcript_id "Novel_000136"; exon_number "2"; gene_type "
At_chr1	Cufflinks	exon	5276695	5277761	.	-	.	gene_id "XLOC_006394"; transcript_id "Novel_000137"; exon_number "1"; gene_type "
At_chr1	Cufflinks	exon	5278992	5279282	.	-	.	gene_id "XLOC_006394"; transcript_id "Novel_000137"; exon_number "2"; gene_type "
At_chr1	Cufflinks	exon	5279593	5279811	.	-	.	gene_id "XLOC_006394"; transcript_id "Novel_000137"; exon_number "3"; gene_type "
At_chr1	Cufflinks	exon	5279892	5280541	.	-	.	gene_id "XLOC_006394"; transcript_id "Novel_000137"; exon_number "4"; gene_type "
At_chr1	Cufflinks	exon	5277254	5277761	.	-	.	gene_id "XLOC_006394"; transcript_id "Novel_000138"; exon_number "1"; gene_type "
At_chr1	Cufflinks	exon	5278992	5280543	.	-	.	gene_id "XLOC_006394"; transcript_id "Novel_000138"; exon_number "2"; gene_type "
At_chr1	Cufflinks	exon	5575068	5575119	.	-	.	gene_id "XLOC_006407"; transcript_id "Novel_000139"; exon_number "1"; gene_type "
At_chr1	Cufflinks	exon	5575589	5576002	.	-	.	gene_id "XLOC_006407"; transcript_id "Novel_000139"; exon_number "2"; gene_type "
At_chr1	Cufflinks	exon	5624977	5625648	.	-	.	gene_id "XLOC_006419"; transcript_id "Novel_000140"; exon_number "1"; gene_type "
At_chr1	Cufflinks	exon	5625847	5626001	.	-	.	gene_id "XLOC_006419"; transcript_id "Novel_000140"; exon_number "2"; gene_type "
At_chr1	Cufflinks	exon	5626106	5626207	.	-	.	gene_id "XLOC_006419"; transcript_id "Novel_000140"; exon_number "3"; gene_type "

At_chr1	Cufflinks	exon	5626321	5626532	.	-	.	gene_id "XLOC_006419"; transcript_id "Novel_000140"; exon_number "4"; gene_type "
At_chr1	Cufflinks	exon	5626666	5626821	.	-	.	gene_id "XLOC_006419"; transcript_id "Novel_000140"; exon_number "5"; gene_type "
At_chr1	Cufflinks	exon	5626919	5627068	.	-	.	gene_id "XLOC_006419"; transcript_id "Novel_000140"; exon_number "6"; gene_type "
At_chr1	Cufflinks	exon	5627589	5627648	.	-	.	gene_id "XLOC_006419"; transcript_id "Novel_000140"; exon_number "7"; gene_type "
At_chr1	Cufflinks	exon	5627758	5627885	.	-	.	gene_id "XLOC_006419"; transcript_id "Novel_000140"; exon_number "8"; gene_type "
At_chr1	Cufflinks	exon	5628116	5628524	.	-	.	gene_id "XLOC_006419"; transcript_id "Novel_000140"; exon_number "9"; gene_type "
At_chr1	Cufflinks	exon	5629756	5630259	.	-	.	gene_id "XLOC_006419"; transcript_id "Novel_000140"; exon_number "10"; gene_type
At_chr1	Cufflinks	exon	5624978	5625670	.	-	.	gene_id "XLOC_006419"; transcript_id "Novel_000141"; exon_number "1"; gene_type "
At_chr1	Cufflinks	exon	5625847	5626001	.	-	.	gene_id "XLOC_006419"; transcript_id "Novel_000141"; exon_number "2"; gene_type "
At_chr1	Cufflinks	exon	5626106	5626207	.	-	.	gene_id "XLOC_006419"; transcript_id "Novel_000141"; exon_number "3"; gene_type "
At_chr1	Cufflinks	exon	5626321	5626532	.	-	.	gene_id "XLOC_006419"; transcript_id "Novel_000141"; exon_number "4"; gene_type "
At_chr1	Cufflinks	exon	5626666	5626821	.	-	.	gene_id "XLOC_006419"; transcript_id "Novel_000141"; exon_number "5"; gene_type "
At_chr1	Cufflinks	exon	5626919	5627223	.	-	.	gene_id "XLOC_006419"; transcript_id "Novel_000141"; exon_number "6"; gene_type "
At_chr1	Cufflinks	exon	5625075	5625648	.	-	.	gene_id "XLOC_006419"; transcript_id "Novel_000142"; exon_number "1"; gene_type "
At_chr1	Cufflinks	exon	5625847	5626001	.	-	.	gene_id "XLOC_006419"; transcript_id "Novel_000142"; exon_number "2"; gene_type "
At_chr1	Cufflinks	exon	5626106	5626207	.	-	.	gene_id "XLOC_006419"; transcript_id "Novel_000142"; exon_number "3"; gene_type "
At_chr1	Cufflinks	exon	5626321	5626532	.	-	.	gene_id "XLOC_006419"; transcript_id "Novel_000142"; exon_number "4"; gene_type "
At_chr1	Cufflinks	exon	5626666	5626821	.	-	.	gene_id "XLOC_006419"; transcript_id "Novel_000142"; exon_number "5"; gene_type "
At_chr1	Cufflinks	exon	5626919	5627580	.	-	.	gene_id "XLOC_006419"; transcript_id "Novel_000142"; exon_number "6"; gene_type "
At_chr1	Cufflinks	exon	5749359	5749732	.	-	.	gene_id "XLOC_006432"; transcript_id "Novel_000143"; exon_number "1"; gene_type "
At_chr1	Cufflinks	exon	5749893	5750078	.	-	.	gene_id "XLOC_006432"; transcript_id "Novel_000143"; exon_number "2"; gene_type "
At_chr1	Cufflinks	exon	5750191	5750313	.	-	.	gene_id "XLOC_006432"; transcript_id "Novel_000143"; exon_number "3"; gene_type "
At_chr1	Cufflinks	exon	5851994	5852493	.	-	.	gene_id "XLOC_006432"; transcript_id "Novel_000144"; exon_number "1"; gene_type "
At_chr1	Cufflinks	exon	5852601	5852830	.	-	.	gene_id "XLOC_006432"; transcript_id "Novel_000144"; exon_number "2"; gene_type "
At_chr1	Cufflinks	exon	5852923	5853035	.	-	.	gene_id "XLOC_006432"; transcript_id "Novel_000144"; exon_number "3"; gene_type "
At_chr1	Cufflinks	exon	5853448	5853531	.	-	.	gene_id "XLOC_006432"; transcript_id "Novel_000144"; exon_number "4"; gene_type "
At_chr1	Cufflinks	exon	5853982	5854097	.	-	.	gene_id "XLOC_006432"; transcript_id "Novel_000144"; exon_number "5"; gene_type "
At_chr1	Cufflinks	exon	5854174	5854293	.	-	.	gene_id "XLOC_006432"; transcript_id "Novel_000144"; exon_number "6"; gene_type "
At_chr1	Cufflinks	exon	5855164	5855275	.	-	.	gene_id "XLOC_006432"; transcript_id "Novel_000144"; exon_number "7"; gene_type "
At_chr1	Cufflinks	exon	5855370	5855538	.	-	.	gene_id "XLOC_006432"; transcript_id "Novel_000144"; exon_number "8"; gene_type "
At_chr1	Cufflinks	exon	5855675	5855743	.	-	.	gene_id "XLOC_006432"; transcript_id "Novel_000144"; exon_number "9"; gene_type "
At_chr1	Cufflinks	exon	5855848	5856018	.	-	.	gene_id "XLOC_006432"; transcript_id "Novel_000144"; exon_number "10"; gene_type
At_chr1	Cufflinks	exon	5856303	5856409	.	-	.	gene_id "XLOC_006432"; transcript_id "Novel_000144"; exon_number "11"; gene_type
At_chr1	Cufflinks	exon	5857155	5857306	.	-	.	gene_id "XLOC_006432"; transcript_id "Novel_000144"; exon_number "12"; gene_type
At_chr1	Cufflinks	exon	5857531	5857678	.	-	.	gene_id "XLOC_006432"; transcript_id "Novel_000144"; exon_number "13"; gene_type

At_chr1	Cufflinks	exon	5857766	5857860	.	-	.	gene_id "XLOC_006432"; transcript_id "Novel_000144"; exon_number "14"; gene_type
At_chr1	Cufflinks	exon	5857965	5858088	.	-	.	gene_id "XLOC_006432"; transcript_id "Novel_000144"; exon_number "15"; gene_type
At_chr1	Cufflinks	exon	5858277	5858437	.	-	.	gene_id "XLOC_006432"; transcript_id "Novel_000144"; exon_number "16"; gene_type
At_chr1	Cufflinks	exon	5858517	5858598	.	-	.	gene_id "XLOC_006432"; transcript_id "Novel_000144"; exon_number "17"; gene_type
At_chr1	Cufflinks	exon	5858680	5858771	.	-	.	gene_id "XLOC_006432"; transcript_id "Novel_000144"; exon_number "18"; gene_type
At_chr1	Cufflinks	exon	5858861	5858980	.	-	.	gene_id "XLOC_006432"; transcript_id "Novel_000144"; exon_number "19"; gene_type
At_chr1	Cufflinks	exon	5859042	5859128	.	-	.	gene_id "XLOC_006432"; transcript_id "Novel_000144"; exon_number "20"; gene_type
At_chr1	Cufflinks	exon	5859502	5859551	.	-	.	gene_id "XLOC_006432"; transcript_id "Novel_000144"; exon_number "21"; gene_type
At_chr1	Cufflinks	exon	5859635	5859716	.	-	.	gene_id "XLOC_006432"; transcript_id "Novel_000144"; exon_number "22"; gene_type
At_chr1	Cufflinks	exon	5860547	5860607	.	-	.	gene_id "XLOC_006432"; transcript_id "Novel_000144"; exon_number "23"; gene_type
At_chr1	Cufflinks	exon	5860693	5860745	.	-	.	gene_id "XLOC_006432"; transcript_id "Novel_000144"; exon_number "24"; gene_type
At_chr1	Cufflinks	exon	5861446	5861774	.	-	.	gene_id "XLOC_006432"; transcript_id "Novel_000144"; exon_number "25"; gene_type
At_chr1	Cufflinks	exon	5862168	5862285	.	-	.	gene_id "XLOC_006432"; transcript_id "Novel_000144"; exon_number "26"; gene_type
At_chr1	Cufflinks	exon	5862359	5862484	.	-	.	gene_id "XLOC_006432"; transcript_id "Novel_000144"; exon_number "27"; gene_type
At_chr1	Cufflinks	exon	5863120	5863276	.	-	.	gene_id "XLOC_006432"; transcript_id "Novel_000144"; exon_number "28"; gene_type
At_chr1	Cufflinks	exon	5863393	5863469	.	-	.	gene_id "XLOC_006432"; transcript_id "Novel_000144"; exon_number "29"; gene_type
At_chr1	Cufflinks	exon	5863540	5863677	.	-	.	gene_id "XLOC_006432"; transcript_id "Novel_000144"; exon_number "30"; gene_type
At_chr1	Cufflinks	exon	5864552	5864678	.	-	.	gene_id "XLOC_006432"; transcript_id "Novel_000144"; exon_number "31"; gene_type
At_chr1	Cufflinks	exon	5864946	5865037	.	-	.	gene_id "XLOC_006432"; transcript_id "Novel_000144"; exon_number "32"; gene_type
At_chr1	Cufflinks	exon	5865124	5865216	.	-	.	gene_id "XLOC_006432"; transcript_id "Novel_000144"; exon_number "33"; gene_type
At_chr1	Cufflinks	exon	5865353	5865472	.	-	.	gene_id "XLOC_006432"; transcript_id "Novel_000144"; exon_number "34"; gene_type
At_chr1	Cufflinks	exon	5865716	5865823	.	-	.	gene_id "XLOC_006432"; transcript_id "Novel_000144"; exon_number "35"; gene_type
At_chr1	Cufflinks	exon	5865932	5866013	.	-	.	gene_id "XLOC_006432"; transcript_id "Novel_000144"; exon_number "36"; gene_type
At_chr1	Cufflinks	exon	5866132	5866253	.	-	.	gene_id "XLOC_006432"; transcript_id "Novel_000144"; exon_number "37"; gene_type
At_chr1	Cufflinks	exon	5867117	5867217	.	-	.	gene_id "XLOC_006432"; transcript_id "Novel_000144"; exon_number "38"; gene_type
At_chr1	Cufflinks	exon	5867560	5868068	.	-	.	gene_id "XLOC_006432"; transcript_id "Novel_000144"; exon_number "39"; gene_type
At_chr1	Cufflinks	exon	5972004	5972257	.	-	.	gene_id "XLOC_006462"; transcript_id "Novel_000145"; exon_number "1"; gene_type "
At_chr1	Cufflinks	exon	6483855	6485611	.	-	.	gene_id "XLOC_006522"; transcript_id "Novel_000146"; exon_number "1"; gene_type "
At_chr1	Cufflinks	exon	6486034	6488027	.	-	.	gene_id "XLOC_006522"; transcript_id "Novel_000146"; exon_number "2"; gene_type "
At_chr1	Cufflinks	exon	6488161	6488510	.	-	.	gene_id "XLOC_006522"; transcript_id "Novel_000146"; exon_number "3"; gene_type "
At_chr1	Cufflinks	exon	6488921	6489109	.	-	.	gene_id "XLOC_006522"; transcript_id "Novel_000146"; exon_number "4"; gene_type "
At_chr1	Cufflinks	exon	6486088	6488027	.	-	.	gene_id "XLOC_006522"; transcript_id "Novel_000147"; exon_number "1"; gene_type "
At_chr1	Cufflinks	exon	6488161	6488510	.	-	.	gene_id "XLOC_006522"; transcript_id "Novel_000147"; exon_number "2"; gene_type "
At_chr1	Cufflinks	exon	6488921	6488974	.	-	.	gene_id "XLOC_006522"; transcript_id "Novel_000147"; exon_number "3"; gene_type "
At_chr1	Cufflinks	exon	6489186	6489353	.	-	.	gene_id "XLOC_006522"; transcript_id "Novel_000147"; exon_number "4"; gene_type "

At_chr1	Cufflinks	exon	6521418	6522649	.	-	.	gene_id "XLOC_006526"; transcript_id "Novel_000148"; exon_number "1"; gene_type "
At_chr1	Cufflinks	exon	6522742	6522975	.	-	.	gene_id "XLOC_006526"; transcript_id "Novel_000148"; exon_number "2"; gene_type "
At_chr1	Cufflinks	exon	6523501	6523786	.	-	.	gene_id "XLOC_006526"; transcript_id "Novel_000148"; exon_number "3"; gene_type "
At_chr1	Cufflinks	exon	6525089	6525238	.	-	.	gene_id "XLOC_006526"; transcript_id "Novel_000148"; exon_number "4"; gene_type "
At_chr1	Cufflinks	exon	6525523	6525596	.	-	.	gene_id "XLOC_006526"; transcript_id "Novel_000148"; exon_number "5"; gene_type "
At_chr1	Cufflinks	exon	6525683	6526350	.	-	.	gene_id "XLOC_006526"; transcript_id "Novel_000148"; exon_number "6"; gene_type "
At_chr1	Cufflinks	exon	7217512	7218099	.	-	.	gene_id "XLOC_006617"; transcript_id "Novel_000149"; exon_number "1"; gene_type "
At_chr1	Cufflinks	exon	7218578	7218716	.	-	.	gene_id "XLOC_006617"; transcript_id "Novel_000149"; exon_number "2"; gene_type "
At_chr1	Cufflinks	exon	7219298	7221049	.	-	.	gene_id "XLOC_006617"; transcript_id "Novel_000149"; exon_number "3"; gene_type "
At_chr1	Cufflinks	exon	7221154	7221212	.	-	.	gene_id "XLOC_006617"; transcript_id "Novel_000149"; exon_number "4"; gene_type "
At_chr1	Cufflinks	exon	7221339	7221449	.	-	.	gene_id "XLOC_006617"; transcript_id "Novel_000149"; exon_number "5"; gene_type "
At_chr1	Cufflinks	exon	7221710	7222176	.	-	.	gene_id "XLOC_006617"; transcript_id "Novel_000149"; exon_number "6"; gene_type "
At_chr1	Cufflinks	exon	7880513	7880730	.	-	.	gene_id "XLOC_006714"; transcript_id "Novel_000150"; exon_number "1"; gene_type "
At_chr1	Cufflinks	exon	8938149	8938387	.	-	.	gene_id "XLOC_006822"; transcript_id "Novel_000151"; exon_number "1"; gene_type "
At_chr1	Cufflinks	exon	9984291	9985330	.	-	.	gene_id "XLOC_006928"; transcript_id "Novel_000152"; exon_number "1"; gene_type "
At_chr1	Cufflinks	exon	9985410	9985479	.	-	.	gene_id "XLOC_006928"; transcript_id "Novel_000152"; exon_number "2"; gene_type "
At_chr1	Cufflinks	exon	9985576	9985660	.	-	.	gene_id "XLOC_006928"; transcript_id "Novel_000152"; exon_number "3"; gene_type "
At_chr1	Cufflinks	exon	9985782	9986151	.	-	.	gene_id "XLOC_006928"; transcript_id "Novel_000152"; exon_number "4"; gene_type "
At_chr1	Cufflinks	exon	9984292	9985324	.	-	.	gene_id "XLOC_006928"; transcript_id "Novel_000153"; exon_number "1"; gene_type "
At_chr1	Cufflinks	exon	9985410	9985479	.	-	.	gene_id "XLOC_006928"; transcript_id "Novel_000153"; exon_number "2"; gene_type "
At_chr1	Cufflinks	exon	9985576	9985660	.	-	.	gene_id "XLOC_006928"; transcript_id "Novel_000153"; exon_number "3"; gene_type "
At_chr1	Cufflinks	exon	9985782	9985983	.	-	.	gene_id "XLOC_006928"; transcript_id "Novel_000153"; exon_number "4"; gene_type "
At_chr1	Cufflinks	exon	9986092	9986366	.	-	.	gene_id "XLOC_006928"; transcript_id "Novel_000153"; exon_number "5"; gene_type "
At_chr1	Cufflinks	exon	9984292	9985330	.	-	.	gene_id "XLOC_006928"; transcript_id "Novel_000154"; exon_number "1"; gene_type "
At_chr1	Cufflinks	exon	9985410	9985479	.	-	.	gene_id "XLOC_006928"; transcript_id "Novel_000154"; exon_number "2"; gene_type "
At_chr1	Cufflinks	exon	9985576	9985660	.	-	.	gene_id "XLOC_006928"; transcript_id "Novel_000154"; exon_number "3"; gene_type "
At_chr1	Cufflinks	exon	9985782	9985983	.	-	.	gene_id "XLOC_006928"; transcript_id "Novel_000154"; exon_number "4"; gene_type "
At_chr1	Cufflinks	exon	9986092	9986366	.	-	.	gene_id "XLOC_006928"; transcript_id "Novel_000154"; exon_number "5"; gene_type "
At_chr1	Cufflinks	exon	9984314	9985479	.	-	.	gene_id "XLOC_006928"; transcript_id "Novel_000155"; exon_number "1"; gene_type "
At_chr1	Cufflinks	exon	9985576	9985660	.	-	.	gene_id "XLOC_006928"; transcript_id "Novel_000155"; exon_number "2"; gene_type "
At_chr1	Cufflinks	exon	9985782	9986154	.	-	.	gene_id "XLOC_006928"; transcript_id "Novel_000155"; exon_number "3"; gene_type "
At_chr1	Cufflinks	exon	9984314	9985479	.	-	.	gene_id "XLOC_006928"; transcript_id "Novel_000156"; exon_number "1"; gene_type "
At_chr1	Cufflinks	exon	9985576	9985660	.	-	.	gene_id "XLOC_006928"; transcript_id "Novel_000156"; exon_number "2"; gene_type "
At_chr1	Cufflinks	exon	9985782	9985983	.	-	.	gene_id "XLOC_006928"; transcript_id "Novel_000156"; exon_number "3"; gene_type "
At_chr1	Cufflinks	exon	9986092	9986307	.	-	.	gene_id "XLOC_006928"; transcript_id "Novel_000156"; exon_number "4"; gene_type "

At_chr1	Cufflinks	exon	12676510	12679466	.	-	.	gene_id "XLOC_007167"; transcript_id "Novel_000157"; exon_number "1"; gene_type "
At_chr1	Cufflinks	exon	12680268	12680603	.	-	.	gene_id "XLOC_007167"; transcript_id "Novel_000157"; exon_number "2"; gene_type "
At_chr1	Cufflinks	exon	15691276	15691866	.	-	.	gene_id "XLOC_007418"; transcript_id "Novel_000158"; exon_number "1"; gene_type "
At_chr1	Cufflinks	exon	15692240	15692368	.	-	.	gene_id "XLOC_007418"; transcript_id "Novel_000158"; exon_number "2"; gene_type "
At_chr1	Cufflinks	exon	15692702	15692822	.	-	.	gene_id "XLOC_007418"; transcript_id "Novel_000158"; exon_number "3"; gene_type "
At_chr1	Cufflinks	exon	15693167	15693168	.	-	.	gene_id "XLOC_007418"; transcript_id "Novel_000158"; exon_number "4"; gene_type "
At_chr1	Cufflinks	exon	15796786	15798128	.	-	.	gene_id "XLOC_007431"; transcript_id "Novel_000159"; exon_number "1"; gene_type "
At_chr1	Cufflinks	exon	15799067	15799102	.	-	.	gene_id "XLOC_007431"; transcript_id "Novel_000159"; exon_number "2"; gene_type "
At_chr1	Cufflinks	exon	15799198	15799337	.	-	.	gene_id "XLOC_007431"; transcript_id "Novel_000159"; exon_number "3"; gene_type "
At_chr1	Cufflinks	exon	15801324	15801480	.	-	.	gene_id "XLOC_007431"; transcript_id "Novel_000159"; exon_number "4"; gene_type "
At_chr1	Cufflinks	exon	15801632	15801802	.	-	.	gene_id "XLOC_007431"; transcript_id "Novel_000159"; exon_number "5"; gene_type "
At_chr1	Cufflinks	exon	15803381	15803628	.	-	.	gene_id "XLOC_007431"; transcript_id "Novel_000159"; exon_number "6"; gene_type "
At_chr1	Cufflinks	exon	15805860	15805966	.	-	.	gene_id "XLOC_007431"; transcript_id "Novel_000159"; exon_number "7"; gene_type "
At_chr1	Cufflinks	exon	15807698	15807800	.	-	.	gene_id "XLOC_007431"; transcript_id "Novel_000159"; exon_number "8"; gene_type "
At_chr1	Cufflinks	exon	15796797	15797812	.	-	.	gene_id "XLOC_007431"; transcript_id "Novel_000160"; exon_number "1"; gene_type "
At_chr1	Cufflinks	exon	15798032	15798128	.	-	.	gene_id "XLOC_007431"; transcript_id "Novel_000160"; exon_number "2"; gene_type "
At_chr1	Cufflinks	exon	15799067	15799102	.	-	.	gene_id "XLOC_007431"; transcript_id "Novel_000160"; exon_number "3"; gene_type "
At_chr1	Cufflinks	exon	15799198	15799337	.	-	.	gene_id "XLOC_007431"; transcript_id "Novel_000160"; exon_number "4"; gene_type "
At_chr1	Cufflinks	exon	15801324	15801480	.	-	.	gene_id "XLOC_007431"; transcript_id "Novel_000160"; exon_number "5"; gene_type "
At_chr1	Cufflinks	exon	15801632	15801802	.	-	.	gene_id "XLOC_007431"; transcript_id "Novel_000160"; exon_number "6"; gene_type "
At_chr1	Cufflinks	exon	15803381	15803628	.	-	.	gene_id "XLOC_007431"; transcript_id "Novel_000160"; exon_number "7"; gene_type "
At_chr1	Cufflinks	exon	15805860	15805966	.	-	.	gene_id "XLOC_007431"; transcript_id "Novel_000160"; exon_number "8"; gene_type "
At_chr1	Cufflinks	exon	15807698	15807799	.	-	.	gene_id "XLOC_007431"; transcript_id "Novel_000160"; exon_number "9"; gene_type "
At_chr1	Cufflinks	exon	15802007	15803628	.	-	.	gene_id "XLOC_007431"; transcript_id "Novel_000161"; exon_number "1"; gene_type "
At_chr1	Cufflinks	exon	15805860	15805966	.	-	.	gene_id "XLOC_007431"; transcript_id "Novel_000161"; exon_number "2"; gene_type "
At_chr1	Cufflinks	exon	15807698	15807839	.	-	.	gene_id "XLOC_007431"; transcript_id "Novel_000161"; exon_number "3"; gene_type "
At_chr1	Cufflinks	exon	16075674	16077245	.	-	.	gene_id "XLOC_007446"; transcript_id "Novel_000162"; exon_number "1"; gene_type "
At_chr1	Cufflinks	exon	16077878	16078268	.	-	.	gene_id "XLOC_007446"; transcript_id "Novel_000162"; exon_number "2"; gene_type "
At_chr1	Cufflinks	exon	23931280	23931575	.	-	.	gene_id "XLOC_007669"; transcript_id "Novel_000163"; exon_number "1"; gene_type "
At_chr1	Cufflinks	exon	23931964	23932097	.	-	.	gene_id "XLOC_007669"; transcript_id "Novel_000163"; exon_number "2"; gene_type "
At_chr1	Cufflinks	exon	23932179	23932352	.	-	.	gene_id "XLOC_007669"; transcript_id "Novel_000163"; exon_number "3"; gene_type "
At_chr1	Cufflinks	exon	36199426	36200174	.	-	.	gene_id "XLOC_008151"; transcript_id "Novel_000164"; exon_number "1"; gene_type "
At_chr1	Cufflinks	exon	36200252	36200323	.	-	.	gene_id "XLOC_008151"; transcript_id "Novel_000164"; exon_number "2"; gene_type "
At_chr1	Cufflinks	exon	36200585	36201097	.	-	.	gene_id "XLOC_008151"; transcript_id "Novel_000164"; exon_number "3"; gene_type "
At_chr1	Cufflinks	exon	36201254	36202155	.	-	.	gene_id "XLOC_008151"; transcript_id "Novel_000164"; exon_number "4"; gene_type "

At_chr1	Cufflinks	exon	40083254	40084260	.	-	.	gene_id "XLOC_008318"; transcript_id "Novel_000165"; exon_number "1"; gene_type "
At_chr1	Cufflinks	exon	40084472	40084650	.	-	.	gene_id "XLOC_008318"; transcript_id "Novel_000165"; exon_number "2"; gene_type "
At_chr1	Cufflinks	exon	40525710	40526476	.	-	.	gene_id "XLOC_008350"; transcript_id "Novel_000166"; exon_number "1"; gene_type "
At_chr1	Cufflinks	exon	40527979	40528292	.	-	.	gene_id "XLOC_008350"; transcript_id "Novel_000166"; exon_number "2"; gene_type "
At_chr1	Cufflinks	exon	40525714	40527330	.	-	.	gene_id "XLOC_008350"; transcript_id "Novel_000167"; exon_number "1"; gene_type "
At_chr1	Cufflinks	exon	40527979	40528038	.	-	.	gene_id "XLOC_008350"; transcript_id "Novel_000167"; exon_number "2"; gene_type "
At_chr1	Cufflinks	exon	40525869	40526476	.	-	.	gene_id "XLOC_008350"; transcript_id "Novel_000168"; exon_number "1"; gene_type "
At_chr1	Cufflinks	exon	40526636	40526704	.	-	.	gene_id "XLOC_008350"; transcript_id "Novel_000168"; exon_number "2"; gene_type "
At_chr1	Cufflinks	exon	40527261	40527330	.	-	.	gene_id "XLOC_008350"; transcript_id "Novel_000168"; exon_number "3"; gene_type "
At_chr1	Cufflinks	exon	40527979	40528322	.	-	.	gene_id "XLOC_008350"; transcript_id "Novel_000168"; exon_number "4"; gene_type "
At_chr1	Cufflinks	exon	40525879	40526476	.	-	.	gene_id "XLOC_008350"; transcript_id "Novel_000169"; exon_number "1"; gene_type "
At_chr1	Cufflinks	exon	40526636	40528098	.	-	.	gene_id "XLOC_008350"; transcript_id "Novel_000169"; exon_number "2"; gene_type "
At_chr1	Cufflinks	exon	40525879	40526476	.	-	.	gene_id "XLOC_008350"; transcript_id "Novel_000170"; exon_number "1"; gene_type "
At_chr1	Cufflinks	exon	40526636	40526704	.	-	.	gene_id "XLOC_008350"; transcript_id "Novel_000170"; exon_number "2"; gene_type "
At_chr1	Cufflinks	exon	40527261	40528281	.	-	.	gene_id "XLOC_008350"; transcript_id "Novel_000170"; exon_number "3"; gene_type "
At_chr1	Cufflinks	exon	44821702	44822562	.	-	.	gene_id "XLOC_008467"; transcript_id "Novel_000171"; exon_number "1"; gene_type "
At_chr1	Cufflinks	exon	44822646	44822768	.	-	.	gene_id "XLOC_008467"; transcript_id "Novel_000171"; exon_number "2"; gene_type "
At_chr1	Cufflinks	exon	44822880	44822962	.	-	.	gene_id "XLOC_008467"; transcript_id "Novel_000171"; exon_number "3"; gene_type "
At_chr1	Cufflinks	exon	44823130	44823348	.	-	.	gene_id "XLOC_008467"; transcript_id "Novel_000171"; exon_number "4"; gene_type "
At_chr1	Cufflinks	exon	44824466	44824692	.	-	.	gene_id "XLOC_008467"; transcript_id "Novel_000171"; exon_number "5"; gene_type "
At_chr1	Cufflinks	exon	44825063	44825316	.	-	.	gene_id "XLOC_008467"; transcript_id "Novel_000171"; exon_number "6"; gene_type "
At_chr1	Cufflinks	exon	44821702	44822562	.	-	.	gene_id "XLOC_008467"; transcript_id "Novel_000172"; exon_number "1"; gene_type "
At_chr1	Cufflinks	exon	44822661	44822768	.	-	.	gene_id "XLOC_008467"; transcript_id "Novel_000172"; exon_number "2"; gene_type "
At_chr1	Cufflinks	exon	44822880	44822971	.	-	.	gene_id "XLOC_008467"; transcript_id "Novel_000172"; exon_number "3"; gene_type "
At_chr1	Cufflinks	exon	44823130	44823348	.	-	.	gene_id "XLOC_008467"; transcript_id "Novel_000172"; exon_number "4"; gene_type "
At_chr1	Cufflinks	exon	44824466	44824692	.	-	.	gene_id "XLOC_008467"; transcript_id "Novel_000172"; exon_number "5"; gene_type "
At_chr1	Cufflinks	exon	44825063	44825316	.	-	.	gene_id "XLOC_008467"; transcript_id "Novel_000172"; exon_number "6"; gene_type "
At_chr1	Cufflinks	exon	44821889	44822562	.	-	.	gene_id "XLOC_008467"; transcript_id "Novel_000173"; exon_number "1"; gene_type "
At_chr1	Cufflinks	exon	44822646	44822768	.	-	.	gene_id "XLOC_008467"; transcript_id "Novel_000173"; exon_number "2"; gene_type "
At_chr1	Cufflinks	exon	44822880	44822971	.	-	.	gene_id "XLOC_008467"; transcript_id "Novel_000173"; exon_number "3"; gene_type "
At_chr1	Cufflinks	exon	44823130	44823348	.	-	.	gene_id "XLOC_008467"; transcript_id "Novel_000173"; exon_number "4"; gene_type "
At_chr1	Cufflinks	exon	44824466	44824692	.	-	.	gene_id "XLOC_008467"; transcript_id "Novel_000173"; exon_number "5"; gene_type "
At_chr1	Cufflinks	exon	44825063	44825317	.	-	.	gene_id "XLOC_008467"; transcript_id "Novel_000173"; exon_number "6"; gene_type "
At_chr1	Cufflinks	exon	48760324	48762299	.	-	.	gene_id "XLOC_008653"; transcript_id "Novel_000174"; exon_number "1"; gene_type "
At_chr1	Cufflinks	exon	48763424	48763727	.	-	.	gene_id "XLOC_008653"; transcript_id "Novel_000174"; exon_number "2"; gene_type "

At_chr1	Cufflinks	exon	51732502	51732801	.	-	.	gene_id "XLOC_008823"; transcript_id "Novel_000175"; exon_number "1"; gene_type "
At_chr1	Cufflinks	exon	51733243	51733544	.	-	.	gene_id "XLOC_008823"; transcript_id "Novel_000175"; exon_number "2"; gene_type "
At_chr1	Cufflinks	exon	51733168	51733455	.	-	.	gene_id "XLOC_008823"; transcript_id "Novel_000176"; exon_number "1"; gene_type "
At_chr1	Cufflinks	exon	51733629	51733927	.	-	.	gene_id "XLOC_008823"; transcript_id "Novel_000176"; exon_number "2"; gene_type "
At_chr1	Cufflinks	exon	52767005	52767053	.	-	.	gene_id "XLOC_008970"; transcript_id "Novel_000177"; exon_number "1"; gene_type "
At_chr1	Cufflinks	exon	52767142	52767293	.	-	.	gene_id "XLOC_008970"; transcript_id "Novel_000177"; exon_number "2"; gene_type "
At_chr1	Cufflinks	exon	53194370	53195428	.	-	.	gene_id "XLOC_009033"; transcript_id "Novel_000178"; exon_number "1"; gene_type "
At_chr1	Cufflinks	exon	53197424	53198276	.	-	.	gene_id "XLOC_009033"; transcript_id "Novel_000178"; exon_number "2"; gene_type "
At_chr1	Cufflinks	exon	54154622	54155650	.	-	.	gene_id "XLOC_009137"; transcript_id "Novel_000179"; exon_number "1"; gene_type "
At_chr1	Cufflinks	exon	54155786	54155851	.	-	.	gene_id "XLOC_009137"; transcript_id "Novel_000179"; exon_number "2"; gene_type "
At_chr1	Cufflinks	exon	54155988	54156071	.	-	.	gene_id "XLOC_009137"; transcript_id "Novel_000179"; exon_number "3"; gene_type "
At_chr1	Cufflinks	exon	54156287	54156373	.	-	.	gene_id "XLOC_009137"; transcript_id "Novel_000179"; exon_number "4"; gene_type "
At_chr1	Cufflinks	exon	54156716	54156825	.	-	.	gene_id "XLOC_009137"; transcript_id "Novel_000179"; exon_number "5"; gene_type "
At_chr1	Cufflinks	exon	54157218	54157251	.	-	.	gene_id "XLOC_009137"; transcript_id "Novel_000179"; exon_number "6"; gene_type "
At_chr1	Cufflinks	exon	54157639	54157775	.	-	.	gene_id "XLOC_009137"; transcript_id "Novel_000179"; exon_number "7"; gene_type "
At_chr1	Cufflinks	exon	54157923	54157992	.	-	.	gene_id "XLOC_009137"; transcript_id "Novel_000179"; exon_number "8"; gene_type "
At_chr1	Cufflinks	exon	54158103	54158177	.	-	.	gene_id "XLOC_009137"; transcript_id "Novel_000179"; exon_number "9"; gene_type "
At_chr1	Cufflinks	exon	54158516	54158630	.	-	.	gene_id "XLOC_009137"; transcript_id "Novel_000179"; exon_number "10"; gene_type
At_chr1	Cufflinks	exon	54158896	54159274	.	-	.	gene_id "XLOC_009137"; transcript_id "Novel_000179"; exon_number "11"; gene_type
At_chr1	Cufflinks	exon	54154662	54155650	.	-	.	gene_id "XLOC_009137"; transcript_id "Novel_000180"; exon_number "1"; gene_type "
At_chr1	Cufflinks	exon	54155786	54155851	.	-	.	gene_id "XLOC_009137"; transcript_id "Novel_000180"; exon_number "2"; gene_type "
At_chr1	Cufflinks	exon	54155988	54156071	.	-	.	gene_id "XLOC_009137"; transcript_id "Novel_000180"; exon_number "3"; gene_type "
At_chr1	Cufflinks	exon	54156287	54157176	.	-	.	gene_id "XLOC_009137"; transcript_id "Novel_000180"; exon_number "4"; gene_type "
At_chr1	Cufflinks	exon	54158074	54158630	.	-	.	gene_id "XLOC_009137"; transcript_id "Novel_000181"; exon_number "1"; gene_type "
At_chr1	Cufflinks	exon	54158896	54159259	.	-	.	gene_id "XLOC_009137"; transcript_id "Novel_000181"; exon_number "2"; gene_type "
At_chr1	Cufflinks	exon	54249218	54250572	.	-	.	gene_id "XLOC_009141"; transcript_id "Novel_000182"; exon_number "1"; gene_type "
At_chr1	Cufflinks	exon	54250648	54250701	.	-	.	gene_id "XLOC_009141"; transcript_id "Novel_000182"; exon_number "2"; gene_type "
At_chr1	Cufflinks	exon	54250825	54251145	.	-	.	gene_id "XLOC_009141"; transcript_id "Novel_000182"; exon_number "3"; gene_type "
At_chr1	Cufflinks	exon	54253705	54253715	.	-	.	gene_id "XLOC_009141"; transcript_id "Novel_000183"; exon_number "1"; gene_type "
At_chr1	Cufflinks	exon	54254222	54254272	.	-	.	gene_id "XLOC_009141"; transcript_id "Novel_000183"; exon_number "2"; gene_type "
At_chr1	Cufflinks	exon	54254355	54254465	.	-	.	gene_id "XLOC_009141"; transcript_id "Novel_000183"; exon_number "3"; gene_type "
At_chr1	Cufflinks	exon	54254757	54254811	.	-	.	gene_id "XLOC_009141"; transcript_id "Novel_000183"; exon_number "4"; gene_type "
At_chr1	Cufflinks	exon	54255038	54255157	.	-	.	gene_id "XLOC_009141"; transcript_id "Novel_000183"; exon_number "5"; gene_type "
At_chr1	Cufflinks	exon	54255284	54255381	.	-	.	gene_id "XLOC_009141"; transcript_id "Novel_000183"; exon_number "6"; gene_type "
At_chr1	Cufflinks	exon	54255514	54255628	.	-	.	gene_id "XLOC_009141"; transcript_id "Novel_000183"; exon_number "7"; gene_type "

At_chr1	Cufflinks	exon	54255958	54256329	.	-	.	gene_id "XLOC_009141"; transcript_id "Novel_000183"; exon_number "8"; gene_type "
At_chr1	Cufflinks	exon	54254179	54254465	.	-	.	gene_id "XLOC_009141"; transcript_id "Novel_000184"; exon_number "1"; gene_type "
At_chr1	Cufflinks	exon	54254757	54254811	.	-	.	gene_id "XLOC_009141"; transcript_id "Novel_000184"; exon_number "2"; gene_type "
At_chr1	Cufflinks	exon	54255038	54255770	.	-	.	gene_id "XLOC_009141"; transcript_id "Novel_000184"; exon_number "3"; gene_type "
At_chr1	Cufflinks	exon	54254532	54255157	.	-	.	gene_id "XLOC_009141"; transcript_id "Novel_000185"; exon_number "1"; gene_type "
At_chr1	Cufflinks	exon	54255284	54255381	.	-	.	gene_id "XLOC_009141"; transcript_id "Novel_000185"; exon_number "2"; gene_type "
At_chr1	Cufflinks	exon	54255514	54255628	.	-	.	gene_id "XLOC_009141"; transcript_id "Novel_000185"; exon_number "3"; gene_type "
At_chr1	Cufflinks	exon	54255958	54256267	.	-	.	gene_id "XLOC_009141"; transcript_id "Novel_000185"; exon_number "4"; gene_type "
At_chr1	Cufflinks	exon	54254845	54255381	.	-	.	gene_id "XLOC_009141"; transcript_id "Novel_000186"; exon_number "1"; gene_type "
At_chr1	Cufflinks	exon	54255514	54255628	.	-	.	gene_id "XLOC_009141"; transcript_id "Novel_000186"; exon_number "2"; gene_type "
At_chr1	Cufflinks	exon	54255958	54256288	.	-	.	gene_id "XLOC_009141"; transcript_id "Novel_000186"; exon_number "3"; gene_type "
At_chr1	Cufflinks	exon	54300114	54300368	.	-	.	gene_id "XLOC_009145"; transcript_id "Novel_000187"; exon_number "1"; gene_type "
At_chr1	Cufflinks	exon	54300459	54300794	.	-	.	gene_id "XLOC_009145"; transcript_id "Novel_000187"; exon_number "2"; gene_type "
At_chr1	Cufflinks	exon	54615848	54616989	.	-	.	gene_id "XLOC_009183"; transcript_id "Novel_000188"; exon_number "1"; gene_type "
At_chr1	Cufflinks	exon	54617095	54617158	.	-	.	gene_id "XLOC_009183"; transcript_id "Novel_000188"; exon_number "2"; gene_type "
At_chr1	Cufflinks	exon	54617270	54617379	.	-	.	gene_id "XLOC_009183"; transcript_id "Novel_000188"; exon_number "3"; gene_type "
At_chr1	Cufflinks	exon	54617499	54617586	.	-	.	gene_id "XLOC_009183"; transcript_id "Novel_000188"; exon_number "4"; gene_type "
At_chr1	Cufflinks	exon	54618221	54618721	.	-	.	gene_id "XLOC_009183"; transcript_id "Novel_000188"; exon_number "5"; gene_type "
At_chr1	Cufflinks	exon	54615904	54616639	.	-	.	gene_id "XLOC_009183"; transcript_id "Novel_000189"; exon_number "1"; gene_type "
At_chr1	Cufflinks	exon	54616725	54616869	.	-	.	gene_id "XLOC_009183"; transcript_id "Novel_000189"; exon_number "2"; gene_type "
At_chr1	Cufflinks	exon	54616892	54617158	.	-	.	gene_id "XLOC_009183"; transcript_id "Novel_000190"; exon_number "1"; gene_type "
At_chr1	Cufflinks	exon	54617270	54617379	.	-	.	gene_id "XLOC_009183"; transcript_id "Novel_000190"; exon_number "2"; gene_type "
At_chr1	Cufflinks	exon	54617499	54617586	.	-	.	gene_id "XLOC_009183"; transcript_id "Novel_000190"; exon_number "3"; gene_type "
At_chr1	Cufflinks	exon	54618221	54618710	.	-	.	gene_id "XLOC_009183"; transcript_id "Novel_000190"; exon_number "4"; gene_type "
At_chr1	Cufflinks	exon	56371593	56372200	.	-	.	gene_id "XLOC_009313"; transcript_id "Novel_000191"; exon_number "1"; gene_type "
At_chr1	Cufflinks	exon	56372466	56372546	.	-	.	gene_id "XLOC_009313"; transcript_id "Novel_000191"; exon_number "2"; gene_type "
At_chr1	Cufflinks	exon	56373478	56373627	.	-	.	gene_id "XLOC_009313"; transcript_id "Novel_000191"; exon_number "3"; gene_type "
At_chr1	Cufflinks	exon	56374049	56374117	.	-	.	gene_id "XLOC_009313"; transcript_id "Novel_000191"; exon_number "4"; gene_type "
At_chr1	Cufflinks	exon	56374210	56374250	.	-	.	gene_id "XLOC_009313"; transcript_id "Novel_000191"; exon_number "5"; gene_type "
At_chr1	Cufflinks	exon	56374339	56374705	.	-	.	gene_id "XLOC_009313"; transcript_id "Novel_000191"; exon_number "6"; gene_type "
At_chr1	Cufflinks	exon	56371769	56374250	.	-	.	gene_id "XLOC_009313"; transcript_id "Novel_000192"; exon_number "1"; gene_type "
At_chr1	Cufflinks	exon	56374339	56374739	.	-	.	gene_id "XLOC_009313"; transcript_id "Novel_000192"; exon_number "2"; gene_type "
At_chr1	Cufflinks	exon	56371801	56372546	.	-	.	gene_id "XLOC_009313"; transcript_id "Novel_000193"; exon_number "1"; gene_type "
At_chr1	Cufflinks	exon	56373478	56373627	.	-	.	gene_id "XLOC_009313"; transcript_id "Novel_000193"; exon_number "2"; gene_type "
At_chr1	Cufflinks	exon	56374049	56374117	.	-	.	gene_id "XLOC_009313"; transcript_id "Novel_000193"; exon_number "3"; gene_type "

At_chr1	Cufflinks	exon	56374210	56374250	.	-	.	gene_id "XLOC_009313"; transcript_id "Novel_000193"; exon_number "4"; gene_type "
At_chr1	Cufflinks	exon	56374339	56374705	.	-	.	gene_id "XLOC_009313"; transcript_id "Novel_000193"; exon_number "5"; gene_type "
At_chr1	Cufflinks	exon	56371801	56372200	.	-	.	gene_id "XLOC_009313"; transcript_id "Novel_000194"; exon_number "1"; gene_type "
At_chr1	Cufflinks	exon	56372466	56372546	.	-	.	gene_id "XLOC_009313"; transcript_id "Novel_000194"; exon_number "2"; gene_type "
At_chr1	Cufflinks	exon	56373478	56373627	.	-	.	gene_id "XLOC_009313"; transcript_id "Novel_000194"; exon_number "3"; gene_type "
At_chr1	Cufflinks	exon	56374049	56374112	.	-	.	gene_id "XLOC_009313"; transcript_id "Novel_000194"; exon_number "4"; gene_type "
At_chr1	Cufflinks	exon	56374210	56374250	.	-	.	gene_id "XLOC_009313"; transcript_id "Novel_000194"; exon_number "5"; gene_type "
At_chr1	Cufflinks	exon	56374339	56374705	.	-	.	gene_id "XLOC_009313"; transcript_id "Novel_000194"; exon_number "6"; gene_type "
At_chr1	Cufflinks	exon	57270195	57283857	.	-	.	gene_id "XLOC_009405"; transcript_id "Novel_000195"; exon_number "1"; gene_type "
At_chr1	Cufflinks	exon	57283936	57284159	.	-	.	gene_id "XLOC_009405"; transcript_id "Novel_000195"; exon_number "2"; gene_type "
At_chr1	Cufflinks	exon	57284316	57284440	.	-	.	gene_id "XLOC_009405"; transcript_id "Novel_000195"; exon_number "3"; gene_type "
At_chr1	Cufflinks	exon	57284747	57284934	.	-	.	gene_id "XLOC_009405"; transcript_id "Novel_000195"; exon_number "4"; gene_type "
At_chr1	Cufflinks	exon	61493157	61493827	.	-	.	gene_id "XLOC_009611"; transcript_id "Novel_000196"; exon_number "1"; gene_type "
At_chr1	Cufflinks	exon	61494499	61495226	.	-	.	gene_id "XLOC_009611"; transcript_id "Novel_000196"; exon_number "2"; gene_type "
At_chr1	Cufflinks	exon	61496219	61496751	.	-	.	gene_id "XLOC_009614"; transcript_id "Novel_000197"; exon_number "1"; gene_type "
At_chr1	Cufflinks	exon	61496833	61497072	.	-	.	gene_id "XLOC_009614"; transcript_id "Novel_000197"; exon_number "2"; gene_type "
At_chr1	Cufflinks	exon	61497203	61497266	.	-	.	gene_id "XLOC_009614"; transcript_id "Novel_000197"; exon_number "3"; gene_type "
At_chr1	Cufflinks	exon	61497367	61498334	.	-	.	gene_id "XLOC_009614"; transcript_id "Novel_000197"; exon_number "4"; gene_type "
At_chr1	Cufflinks	exon	61498509	61498673	.	-	.	gene_id "XLOC_009614"; transcript_id "Novel_000197"; exon_number "5"; gene_type "
At_chr1	Cufflinks	exon	61498751	61499386	.	-	.	gene_id "XLOC_009614"; transcript_id "Novel_000197"; exon_number "6"; gene_type "
At_chr1	Cufflinks	exon	61499482	61499568	.	-	.	gene_id "XLOC_009614"; transcript_id "Novel_000197"; exon_number "7"; gene_type "
At_chr1	Cufflinks	exon	61499778	61502293	.	-	.	gene_id "XLOC_009614"; transcript_id "Novel_000197"; exon_number "8"; gene_type "
At_chr1	Cufflinks	exon	61529255	61529791	.	-	.	gene_id "XLOC_009620"; transcript_id "Novel_000198"; exon_number "1"; gene_type "
At_chr1	Cufflinks	exon	61529868	61529954	.	-	.	gene_id "XLOC_009620"; transcript_id "Novel_000198"; exon_number "2"; gene_type "
At_chr1	Cufflinks	exon	61530114	61530629	.	-	.	gene_id "XLOC_009620"; transcript_id "Novel_000198"; exon_number "3"; gene_type "
At_chr1	Cufflinks	exon	61624148	61624762	.	-	.	gene_id "XLOC_009626"; transcript_id "Novel_000199"; exon_number "1"; gene_type "
At_chr1	Cufflinks	exon	61625053	61626255	.	-	.	gene_id "XLOC_009626"; transcript_id "Novel_000199"; exon_number "2"; gene_type "
At_chr1	Cufflinks	exon	61626678	61626742	.	-	.	gene_id "XLOC_009626"; transcript_id "Novel_000199"; exon_number "3"; gene_type "
At_chr1	Cufflinks	exon	61626847	61626923	.	-	.	gene_id "XLOC_009626"; transcript_id "Novel_000199"; exon_number "4"; gene_type "
At_chr1	Cufflinks	exon	61627202	61627335	.	-	.	gene_id "XLOC_009626"; transcript_id "Novel_000199"; exon_number "5"; gene_type "
At_chr1	Cufflinks	exon	61624148	61624762	.	-	.	gene_id "XLOC_009626"; transcript_id "Novel_000200"; exon_number "1"; gene_type "
At_chr1	Cufflinks	exon	61625053	61625212	.	-	.	gene_id "XLOC_009626"; transcript_id "Novel_000200"; exon_number "2"; gene_type "
At_chr1	Cufflinks	exon	61625378	61625453	.	-	.	gene_id "XLOC_009626"; transcript_id "Novel_000200"; exon_number "3"; gene_type "
At_chr1	Cufflinks	exon	61626209	61626255	.	-	.	gene_id "XLOC_009626"; transcript_id "Novel_000200"; exon_number "4"; gene_type "
At_chr1	Cufflinks	exon	61626678	61626742	.	-	.	gene_id "XLOC_009626"; transcript_id "Novel_000200"; exon_number "5"; gene_type "

At_chr1	Cufflinks	exon	61626847	61626923	.	-	.	gene_id "XLOC_009626"; transcript_id "Novel_000200"; exon_number "6"; gene_type "
At_chr1	Cufflinks	exon	61627202	61627335	.	-	.	gene_id "XLOC_009626"; transcript_id "Novel_000200"; exon_number "7"; gene_type "
At_chr1	Cufflinks	exon	61624207	61624762	.	-	.	gene_id "XLOC_009626"; transcript_id "Novel_000201"; exon_number "1"; gene_type "
At_chr1	Cufflinks	exon	61625053	61625453	.	-	.	gene_id "XLOC_009626"; transcript_id "Novel_000201"; exon_number "2"; gene_type "
At_chr1	Cufflinks	exon	61625563	61625661	.	-	.	gene_id "XLOC_009626"; transcript_id "Novel_000201"; exon_number "3"; gene_type "
At_chr1	Cufflinks	exon	61626209	61626255	.	-	.	gene_id "XLOC_009626"; transcript_id "Novel_000201"; exon_number "4"; gene_type "
At_chr1	Cufflinks	exon	61626678	61626742	.	-	.	gene_id "XLOC_009626"; transcript_id "Novel_000201"; exon_number "5"; gene_type "
At_chr1	Cufflinks	exon	61626847	61626923	.	-	.	gene_id "XLOC_009626"; transcript_id "Novel_000201"; exon_number "6"; gene_type "
At_chr1	Cufflinks	exon	61627207	61627310	.	-	.	gene_id "XLOC_009626"; transcript_id "Novel_000201"; exon_number "7"; gene_type "
At_chr1	Cufflinks	exon	61624207	61624762	.	-	.	gene_id "XLOC_009626"; transcript_id "Novel_000202"; exon_number "1"; gene_type "
At_chr1	Cufflinks	exon	61625053	61625453	.	-	.	gene_id "XLOC_009626"; transcript_id "Novel_000202"; exon_number "2"; gene_type "
At_chr1	Cufflinks	exon	61626209	61626255	.	-	.	gene_id "XLOC_009626"; transcript_id "Novel_000202"; exon_number "3"; gene_type "
At_chr1	Cufflinks	exon	61626678	61626742	.	-	.	gene_id "XLOC_009626"; transcript_id "Novel_000202"; exon_number "4"; gene_type "
At_chr1	Cufflinks	exon	61626847	61626923	.	-	.	gene_id "XLOC_009626"; transcript_id "Novel_000202"; exon_number "5"; gene_type "
At_chr1	Cufflinks	exon	61627202	61627310	.	-	.	gene_id "XLOC_009626"; transcript_id "Novel_000202"; exon_number "6"; gene_type "
At_chr1	Cufflinks	exon	61624292	61624762	.	-	.	gene_id "XLOC_009626"; transcript_id "Novel_000203"; exon_number "1"; gene_type "
At_chr1	Cufflinks	exon	61625053	61625219	.	-	.	gene_id "XLOC_009626"; transcript_id "Novel_000203"; exon_number "2"; gene_type "
At_chr1	Cufflinks	exon	61625378	61625453	.	-	.	gene_id "XLOC_009626"; transcript_id "Novel_000203"; exon_number "3"; gene_type "
At_chr1	Cufflinks	exon	61626209	61626255	.	-	.	gene_id "XLOC_009626"; transcript_id "Novel_000203"; exon_number "4"; gene_type "
At_chr1	Cufflinks	exon	61626678	61626742	.	-	.	gene_id "XLOC_009626"; transcript_id "Novel_000203"; exon_number "5"; gene_type "
At_chr1	Cufflinks	exon	61626847	61626923	.	-	.	gene_id "XLOC_009626"; transcript_id "Novel_000203"; exon_number "6"; gene_type "
At_chr1	Cufflinks	exon	61627202	61627285	.	-	.	gene_id "XLOC_009626"; transcript_id "Novel_000203"; exon_number "7"; gene_type "
At_chr1	Cufflinks	exon	61625852	61626742	.	-	.	gene_id "XLOC_009626"; transcript_id "Novel_000204"; exon_number "1"; gene_type "
At_chr1	Cufflinks	exon	61626847	61626923	.	-	.	gene_id "XLOC_009626"; transcript_id "Novel_000204"; exon_number "2"; gene_type "
At_chr1	Cufflinks	exon	61627202	61627316	.	-	.	gene_id "XLOC_009626"; transcript_id "Novel_000204"; exon_number "3"; gene_type "
At_chr1	Cufflinks	exon	61634361	61635147	.	-	.	gene_id "XLOC_009629"; transcript_id "Novel_000205"; exon_number "1"; gene_type "
At_chr1	Cufflinks	exon	61635255	61635476	.	-	.	gene_id "XLOC_009629"; transcript_id "Novel_000205"; exon_number "2"; gene_type "
At_chr1	Cufflinks	exon	63469803	63469872	.	-	.	gene_id "XLOC_009676"; transcript_id "Novel_000206"; exon_number "1"; gene_type "
At_chr1	Cufflinks	exon	63469987	63470151	.	-	.	gene_id "XLOC_009676"; transcript_id "Novel_000206"; exon_number "2"; gene_type "
At_chr1	Cufflinks	exon	66107413	66107505	.	-	.	gene_id "XLOC_009790"; transcript_id "Novel_000207"; exon_number "1"; gene_type "
At_chr1	Cufflinks	exon	66108355	66108439	.	-	.	gene_id "XLOC_009790"; transcript_id "Novel_000207"; exon_number "2"; gene_type "
At_chr1	Cufflinks	exon	66108829	66108937	.	-	.	gene_id "XLOC_009790"; transcript_id "Novel_000207"; exon_number "3"; gene_type "
At_chr1	Cufflinks	exon	66109454	66109590	.	-	.	gene_id "XLOC_009790"; transcript_id "Novel_000207"; exon_number "4"; gene_type "
At_chr1	Cufflinks	exon	66111266	66111610	.	-	.	gene_id "XLOC_009790"; transcript_id "Novel_000207"; exon_number "5"; gene_type "
At_chr1	Cufflinks	exon	66107460	66107505	.	-	.	gene_id "XLOC_009790"; transcript_id "Novel_000208"; exon_number "1"; gene_type "

At_chr1	Cufflinks	exon	66108355	66108439	.	-	.	gene_id "XLOC_009790"; transcript_id "Novel_000208"; exon_number "2"; gene_type "
At_chr1	Cufflinks	exon	66108829	66108937	.	-	.	gene_id "XLOC_009790"; transcript_id "Novel_000208"; exon_number "3"; gene_type "
At_chr1	Cufflinks	exon	66109454	66110722	.	-	.	gene_id "XLOC_009790"; transcript_id "Novel_000208"; exon_number "4"; gene_type "
At_chr1	Cufflinks	exon	66129899	66130801	.	-	.	gene_id "XLOC_009792"; transcript_id "Novel_000209"; exon_number "1"; gene_type "
At_chr1	Cufflinks	exon	66131115	66131275	.	-	.	gene_id "XLOC_009792"; transcript_id "Novel_000209"; exon_number "2"; gene_type "
At_chr1	Cufflinks	exon	66129899	66130801	.	-	.	gene_id "XLOC_009792"; transcript_id "Novel_000210"; exon_number "1"; gene_type "
At_chr1	Cufflinks	exon	66131120	66131275	.	-	.	gene_id "XLOC_009792"; transcript_id "Novel_000210"; exon_number "2"; gene_type "
At_chr1	Cufflinks	exon	66152507	66152760	.	-	.	gene_id "XLOC_009798"; transcript_id "Novel_000211"; exon_number "1"; gene_type "
At_chr1	Cufflinks	exon	66153019	66153063	.	-	.	gene_id "XLOC_009798"; transcript_id "Novel_000211"; exon_number "2"; gene_type "
At_chr1	Cufflinks	exon	66153688	66153892	.	-	.	gene_id "XLOC_009798"; transcript_id "Novel_000211"; exon_number "3"; gene_type "
At_chr1	Cufflinks	exon	66152509	66152760	.	-	.	gene_id "XLOC_009798"; transcript_id "Novel_000212"; exon_number "1"; gene_type "
At_chr1	Cufflinks	exon	66153019	66153788	.	-	.	gene_id "XLOC_009798"; transcript_id "Novel_000212"; exon_number "2"; gene_type "
At_chr1	Cufflinks	exon	66154585	66154662	.	-	.	gene_id "XLOC_009798"; transcript_id "Novel_000212"; exon_number "3"; gene_type "
At_chr1	Cufflinks	exon	66154971	66155048	.	-	.	gene_id "XLOC_009798"; transcript_id "Novel_000212"; exon_number "4"; gene_type "
At_chr1	Cufflinks	exon	66155133	66155244	.	-	.	gene_id "XLOC_009798"; transcript_id "Novel_000212"; exon_number "5"; gene_type "
At_chr1	Cufflinks	exon	66155482	66155541	.	-	.	gene_id "XLOC_009798"; transcript_id "Novel_000212"; exon_number "6"; gene_type "
At_chr1	Cufflinks	exon	66155616	66155695	.	-	.	gene_id "XLOC_009798"; transcript_id "Novel_000212"; exon_number "7"; gene_type "
At_chr1	Cufflinks	exon	66156163	66156266	.	-	.	gene_id "XLOC_009798"; transcript_id "Novel_000212"; exon_number "8"; gene_type "
At_chr1	Cufflinks	exon	66156449	66156719	.	-	.	gene_id "XLOC_009798"; transcript_id "Novel_000212"; exon_number "9"; gene_type "
At_chr1	Cufflinks	exon	66153944	66154662	.	-	.	gene_id "XLOC_009798"; transcript_id "Novel_000213"; exon_number "1"; gene_type "
At_chr1	Cufflinks	exon	66154971	66155048	.	-	.	gene_id "XLOC_009798"; transcript_id "Novel_000213"; exon_number "2"; gene_type "
At_chr1	Cufflinks	exon	66155482	66155541	.	-	.	gene_id "XLOC_009798"; transcript_id "Novel_000213"; exon_number "3"; gene_type "
At_chr1	Cufflinks	exon	66155616	66155695	.	-	.	gene_id "XLOC_009798"; transcript_id "Novel_000213"; exon_number "4"; gene_type "
At_chr1	Cufflinks	exon	66156163	66156266	.	-	.	gene_id "XLOC_009798"; transcript_id "Novel_000213"; exon_number "5"; gene_type "
At_chr1	Cufflinks	exon	66156449	66156719	.	-	.	gene_id "XLOC_009798"; transcript_id "Novel_000213"; exon_number "6"; gene_type "
At_chr1	Cufflinks	exon	66155290	66155541	.	-	.	gene_id "XLOC_009798"; transcript_id "Novel_000214"; exon_number "1"; gene_type "
At_chr1	Cufflinks	exon	66155616	66155695	.	-	.	gene_id "XLOC_009798"; transcript_id "Novel_000214"; exon_number "2"; gene_type "
At_chr1	Cufflinks	exon	66156163	66156266	.	-	.	gene_id "XLOC_009798"; transcript_id "Novel_000214"; exon_number "3"; gene_type "
At_chr1	Cufflinks	exon	66156449	66156706	.	-	.	gene_id "XLOC_009798"; transcript_id "Novel_000214"; exon_number "4"; gene_type "
At_chr1	Cufflinks	exon	66155292	66155301	.	-	.	gene_id "XLOC_009798"; transcript_id "Novel_000215"; exon_number "1"; gene_type "
At_chr1	Cufflinks	exon	66155482	66155541	.	-	.	gene_id "XLOC_009798"; transcript_id "Novel_000215"; exon_number "2"; gene_type "
At_chr1	Cufflinks	exon	66155616	66155695	.	-	.	gene_id "XLOC_009798"; transcript_id "Novel_000215"; exon_number "3"; gene_type "
At_chr1	Cufflinks	exon	66156163	66156266	.	-	.	gene_id "XLOC_009798"; transcript_id "Novel_000215"; exon_number "4"; gene_type "
At_chr1	Cufflinks	exon	66156449	66156719	.	-	.	gene_id "XLOC_009798"; transcript_id "Novel_000215"; exon_number "5"; gene_type "
At_chr1	Cufflinks	exon	80692997	80693699	.	-	.	gene_id "XLOC_010249"; transcript_id "Novel_000216"; exon_number "1"; gene_type "

At_chr1	Cufflinks	exon	80693811	80693904	.	-	.	gene_id "XLOC_010249"; transcript_id "Novel_000216"; exon_number "2"; gene_type "
At_chr1	Cufflinks	exon	80694010	80695210	.	-	.	gene_id "XLOC_010249"; transcript_id "Novel_000216"; exon_number "3"; gene_type "
At_chr1	Cufflinks	exon	80876890	80878576	.	-	.	gene_id "XLOC_010286"; transcript_id "Novel_000217"; exon_number "1"; gene_type "
At_chr1	Cufflinks	exon	80880347	80881357	.	-	.	gene_id "XLOC_010286"; transcript_id "Novel_000217"; exon_number "2"; gene_type "
At_chr1	Cufflinks	exon	86926333	86928695	.	-	.	gene_id "XLOC_010632"; transcript_id "Novel_000218"; exon_number "1"; gene_type "
At_chr1	Cufflinks	exon	86928782	86928810	.	-	.	gene_id "XLOC_010632"; transcript_id "Novel_000218"; exon_number "2"; gene_type "
At_chr1	Cufflinks	exon	86926632	86927321	.	-	.	gene_id "XLOC_010632"; transcript_id "Novel_000219"; exon_number "1"; gene_type "
At_chr1	Cufflinks	exon	86928396	86928695	.	-	.	gene_id "XLOC_010632"; transcript_id "Novel_000219"; exon_number "2"; gene_type "
At_chr1	Cufflinks	exon	86928782	86928810	.	-	.	gene_id "XLOC_010632"; transcript_id "Novel_000219"; exon_number "3"; gene_type "
At_chr1	Cufflinks	exon	87586805	87587018	.	-	.	gene_id "XLOC_010670"; transcript_id "Novel_000220"; exon_number "1"; gene_type "
At_chr1	Cufflinks	exon	87767598	87767667	.	-	.	gene_id "XLOC_010689"; transcript_id "Novel_000221"; exon_number "1"; gene_type "
At_chr1	Cufflinks	exon	87769158	87769300	.	-	.	gene_id "XLOC_010689"; transcript_id "Novel_000221"; exon_number "2"; gene_type "
At_chr1	Cufflinks	exon	92000764	92001307	.	-	.	gene_id "XLOC_010829"; transcript_id "Novel_000222"; exon_number "1"; gene_type "
At_chr1	Cufflinks	exon	92001413	92001493	.	-	.	gene_id "XLOC_010829"; transcript_id "Novel_000222"; exon_number "2"; gene_type "
At_chr1	Cufflinks	exon	92001574	92001991	.	-	.	gene_id "XLOC_010829"; transcript_id "Novel_000222"; exon_number "3"; gene_type "
At_chr1	Cufflinks	exon	92002196	92002296	.	-	.	gene_id "XLOC_010829"; transcript_id "Novel_000222"; exon_number "4"; gene_type "
At_chr1	Cufflinks	exon	92002554	92002631	.	-	.	gene_id "XLOC_010829"; transcript_id "Novel_000222"; exon_number "5"; gene_type "
At_chr1	Cufflinks	exon	92002780	92002875	.	-	.	gene_id "XLOC_010829"; transcript_id "Novel_000222"; exon_number "6"; gene_type "
At_chr1	Cufflinks	exon	92002970	92003069	.	-	.	gene_id "XLOC_010829"; transcript_id "Novel_000222"; exon_number "7"; gene_type "
At_chr1	Cufflinks	exon	92003170	92003219	.	-	.	gene_id "XLOC_010829"; transcript_id "Novel_000222"; exon_number "8"; gene_type "
At_chr1	Cufflinks	exon	92003300	92003401	.	-	.	gene_id "XLOC_010829"; transcript_id "Novel_000222"; exon_number "9"; gene_type "
At_chr1	Cufflinks	exon	92003486	92003563	.	-	.	gene_id "XLOC_010829"; transcript_id "Novel_000222"; exon_number "10"; gene_type
At_chr1	Cufflinks	exon	92003696	92003962	.	-	.	gene_id "XLOC_010829"; transcript_id "Novel_000222"; exon_number "11"; gene_type
At_chr1	Cufflinks	exon	92004072	92004171	.	-	.	gene_id "XLOC_010829"; transcript_id "Novel_000222"; exon_number "12"; gene_type
At_chr1	Cufflinks	exon	92004297	92004522	.	-	.	gene_id "XLOC_010829"; transcript_id "Novel_000222"; exon_number "13"; gene_type
At_chr1	Cufflinks	exon	92004622	92004732	.	-	.	gene_id "XLOC_010829"; transcript_id "Novel_000222"; exon_number "14"; gene_type
At_chr1	Cufflinks	exon	92004824	92004931	.	-	.	gene_id "XLOC_010829"; transcript_id "Novel_000222"; exon_number "15"; gene_type
At_chr1	Cufflinks	exon	92156108	92157877	.	-	.	gene_id "XLOC_010856"; transcript_id "Novel_000223"; exon_number "1"; gene_type "
At_chr1	Cufflinks	exon	92158022	92160608	.	-	.	gene_id "XLOC_010856"; transcript_id "Novel_000223"; exon_number "2"; gene_type "
At_chr1	Cufflinks	exon	96175510	96175687	.	-	.	gene_id "XLOC_011010"; transcript_id "Novel_000224"; exon_number "1"; gene_type "
At_chr1	Cufflinks	exon	96175777	96176003	.	-	.	gene_id "XLOC_011010"; transcript_id "Novel_000224"; exon_number "2"; gene_type "
At_chr1	Cufflinks	exon	1.01E+08	1.01E+08	.	-	.	gene_id "XLOC_011175"; transcript_id "Novel_000225"; exon_number "1"; gene_type "
At_chr1	Cufflinks	exon	1.01E+08	1.01E+08	.	-	.	gene_id "XLOC_011175"; transcript_id "Novel_000225"; exon_number "2"; gene_type "
At_chr1	Cufflinks	exon	1.04E+08	1.04E+08	.	-	.	gene_id "XLOC_011263"; transcript_id "Novel_000226"; exon_number "1"; gene_type "
At_chr1	Cufflinks	exon	1.05E+08	1.05E+08	.	-	.	gene_id "XLOC_011342"; transcript_id "Novel_000227"; exon_number "1"; gene_type "

At_chr1	Cufflinks	exon	1.05E+08	1.05E+08	.	-	.	gene_id "XLOC_011342"; transcript_id "Novel_000227"; exon_number "2"; gene_type "
At_chr1	Cufflinks	exon	1.06E+08	1.06E+08	.	-	.	gene_id "XLOC_011395"; transcript_id "Novel_000228"; exon_number "1"; gene_type "
At_chr1	Cufflinks	exon	1.06E+08	1.06E+08	.	-	.	gene_id "XLOC_011395"; transcript_id "Novel_000228"; exon_number "2"; gene_type "
At_chr1	Cufflinks	exon	1.06E+08	1.06E+08	.	-	.	gene_id "XLOC_011462"; transcript_id "Novel_000229"; exon_number "1"; gene_type "
At_chr1	Cufflinks	exon	1.06E+08	1.06E+08	.	-	.	gene_id "XLOC_011462"; transcript_id "Novel_000229"; exon_number "2"; gene_type "
At_chr1	Cufflinks	exon	1.06E+08	1.06E+08	.	-	.	gene_id "XLOC_011462"; transcript_id "Novel_000229"; exon_number "3"; gene_type "
At_chr1	Cufflinks	exon	1.06E+08	1.06E+08	.	-	.	gene_id "XLOC_011462"; transcript_id "Novel_000230"; exon_number "1"; gene_type "
At_chr1	Cufflinks	exon	1.06E+08	1.06E+08	.	-	.	gene_id "XLOC_011462"; transcript_id "Novel_000230"; exon_number "2"; gene_type "
At_chr10	Cufflinks	exon	2838080	2838767	.	+	.	gene_id "XLOC_011903"; transcript_id "Novel_000231"; exon_number "1"; gene_type "
At_chr10	Cufflinks	exon	2838891	2839394	.	+	.	gene_id "XLOC_011903"; transcript_id "Novel_000231"; exon_number "2"; gene_type "
At_chr10	Cufflinks	exon	2839445	2840576	.	+	.	gene_id "XLOC_011903"; transcript_id "Novel_000231"; exon_number "3"; gene_type "
At_chr10	Cufflinks	exon	5376182	5376553	.	+	.	gene_id "XLOC_012064"; transcript_id "Novel_000232"; exon_number "1"; gene_type "
At_chr10	Cufflinks	exon	5376636	5376759	.	+	.	gene_id "XLOC_012064"; transcript_id "Novel_000232"; exon_number "2"; gene_type "
At_chr10	Cufflinks	exon	5379043	5379386	.	+	.	gene_id "XLOC_012064"; transcript_id "Novel_000232"; exon_number "3"; gene_type "
At_chr10	Cufflinks	exon	5376182	5376553	.	+	.	gene_id "XLOC_012064"; transcript_id "Novel_000233"; exon_number "1"; gene_type "
At_chr10	Cufflinks	exon	5376636	5376759	.	+	.	gene_id "XLOC_012064"; transcript_id "Novel_000233"; exon_number "2"; gene_type "
At_chr10	Cufflinks	exon	5377199	5377368	.	+	.	gene_id "XLOC_012064"; transcript_id "Novel_000233"; exon_number "3"; gene_type "
At_chr10	Cufflinks	exon	5379043	5379386	.	+	.	gene_id "XLOC_012064"; transcript_id "Novel_000233"; exon_number "4"; gene_type "
At_chr10	Cufflinks	exon	5376206	5377368	.	+	.	gene_id "XLOC_012064"; transcript_id "Novel_000234"; exon_number "1"; gene_type "
At_chr10	Cufflinks	exon	5379043	5379373	.	+	.	gene_id "XLOC_012064"; transcript_id "Novel_000234"; exon_number "2"; gene_type "
At_chr10	Cufflinks	exon	9191354	9191519	.	+	.	gene_id "XLOC_012190"; transcript_id "Novel_000235"; exon_number "1"; gene_type "
At_chr10	Cufflinks	exon	9191634	9192675	.	+	.	gene_id "XLOC_012190"; transcript_id "Novel_000235"; exon_number "2"; gene_type "
At_chr10	Cufflinks	exon	10231587	10231817	.	+	.	gene_id "XLOC_012210"; transcript_id "Novel_000236"; exon_number "1"; gene_type "
At_chr10	Cufflinks	exon	20887068	20887419	.	+	.	gene_id "XLOC_012608"; transcript_id "Novel_000237"; exon_number "1"; gene_type "
At_chr10	Cufflinks	exon	20887744	20887811	.	+	.	gene_id "XLOC_012608"; transcript_id "Novel_000237"; exon_number "2"; gene_type "
At_chr10	Cufflinks	exon	20887904	20889311	.	+	.	gene_id "XLOC_012608"; transcript_id "Novel_000237"; exon_number "3"; gene_type "
At_chr10	Cufflinks	exon	21765902	21766125	.	+	.	gene_id "XLOC_012671"; transcript_id "Novel_000238"; exon_number "1"; gene_type "
At_chr10	Cufflinks	exon	22731588	22731802	.	+	.	gene_id "XLOC_012706"; transcript_id "Novel_000239"; exon_number "1"; gene_type "
At_chr10	Cufflinks	exon	22732654	22732717	.	+	.	gene_id "XLOC_012706"; transcript_id "Novel_000239"; exon_number "2"; gene_type "
At_chr10	Cufflinks	exon	22733192	22733293	.	+	.	gene_id "XLOC_012706"; transcript_id "Novel_000239"; exon_number "3"; gene_type "
At_chr10	Cufflinks	exon	22734113	22734191	.	+	.	gene_id "XLOC_012706"; transcript_id "Novel_000239"; exon_number "4"; gene_type "
At_chr10	Cufflinks	exon	22734638	22734737	.	+	.	gene_id "XLOC_012706"; transcript_id "Novel_000239"; exon_number "5"; gene_type "
At_chr10	Cufflinks	exon	22735936	22736810	.	+	.	gene_id "XLOC_012706"; transcript_id "Novel_000239"; exon_number "6"; gene_type "
At_chr10	Cufflinks	exon	22731599	22731802	.	+	.	gene_id "XLOC_012706"; transcript_id "Novel_000240"; exon_number "1"; gene_type "
At_chr10	Cufflinks	exon	22732654	22732764	.	+	.	gene_id "XLOC_012706"; transcript_id "Novel_000240"; exon_number "2"; gene_type "

At_chr10	Cufflinks	exon	22733192	22733293	.	+	.	gene_id "XLOC_012706"; transcript_id "Novel_000240"; exon_number "3"; gene_type "
At_chr10	Cufflinks	exon	22734113	22734191	.	+	.	gene_id "XLOC_012706"; transcript_id "Novel_000240"; exon_number "4"; gene_type "
At_chr10	Cufflinks	exon	22734638	22734737	.	+	.	gene_id "XLOC_012706"; transcript_id "Novel_000240"; exon_number "5"; gene_type "
At_chr10	Cufflinks	exon	22735936	22736711	.	+	.	gene_id "XLOC_012706"; transcript_id "Novel_000240"; exon_number "6"; gene_type "
At_chr10	Cufflinks	exon	22732831	22733293	.	+	.	gene_id "XLOC_012706"; transcript_id "Novel_000241"; exon_number "1"; gene_type "
At_chr10	Cufflinks	exon	22734113	22734191	.	+	.	gene_id "XLOC_012706"; transcript_id "Novel_000241"; exon_number "2"; gene_type "
At_chr10	Cufflinks	exon	22734638	22734737	.	+	.	gene_id "XLOC_012706"; transcript_id "Novel_000241"; exon_number "3"; gene_type "
At_chr10	Cufflinks	exon	22735936	22736564	.	+	.	gene_id "XLOC_012706"; transcript_id "Novel_000241"; exon_number "4"; gene_type "
At_chr10	Cufflinks	exon	24646306	24646514	.	+	.	gene_id "XLOC_012788"; transcript_id "Novel_000242"; exon_number "1"; gene_type "
At_chr10	Cufflinks	exon	24647376	24648246	.	+	.	gene_id "XLOC_012788"; transcript_id "Novel_000242"; exon_number "2"; gene_type "
At_chr10	Cufflinks	exon	24646306	24646518	.	+	.	gene_id "XLOC_012788"; transcript_id "Novel_000243"; exon_number "1"; gene_type "
At_chr10	Cufflinks	exon	24647376	24648246	.	+	.	gene_id "XLOC_012788"; transcript_id "Novel_000243"; exon_number "2"; gene_type "
At_chr10	Cufflinks	exon	24809371	24809703	.	+	.	gene_id "XLOC_012797"; transcript_id "Novel_000244"; exon_number "1"; gene_type "
At_chr10	Cufflinks	exon	24810166	24810272	.	+	.	gene_id "XLOC_012797"; transcript_id "Novel_000244"; exon_number "2"; gene_type "
At_chr10	Cufflinks	exon	24810748	24810896	.	+	.	gene_id "XLOC_012797"; transcript_id "Novel_000244"; exon_number "3"; gene_type "
At_chr10	Cufflinks	exon	24811567	24811881	.	+	.	gene_id "XLOC_012797"; transcript_id "Novel_000244"; exon_number "4"; gene_type "
At_chr10	Cufflinks	exon	24811986	24812039	.	+	.	gene_id "XLOC_012797"; transcript_id "Novel_000244"; exon_number "5"; gene_type "
At_chr10	Cufflinks	exon	24812220	24813233	.	+	.	gene_id "XLOC_012797"; transcript_id "Novel_000244"; exon_number "6"; gene_type "
At_chr10	Cufflinks	exon	24809371	24809703	.	+	.	gene_id "XLOC_012797"; transcript_id "Novel_000245"; exon_number "1"; gene_type "
At_chr10	Cufflinks	exon	24810166	24810272	.	+	.	gene_id "XLOC_012797"; transcript_id "Novel_000245"; exon_number "2"; gene_type "
At_chr10	Cufflinks	exon	24810748	24810896	.	+	.	gene_id "XLOC_012797"; transcript_id "Novel_000245"; exon_number "3"; gene_type "
At_chr10	Cufflinks	exon	24811567	24811724	.	+	.	gene_id "XLOC_012797"; transcript_id "Novel_000245"; exon_number "4"; gene_type "
At_chr10	Cufflinks	exon	24811816	24811881	.	+	.	gene_id "XLOC_012797"; transcript_id "Novel_000245"; exon_number "5"; gene_type "
At_chr10	Cufflinks	exon	24811986	24812039	.	+	.	gene_id "XLOC_012797"; transcript_id "Novel_000245"; exon_number "6"; gene_type "
At_chr10	Cufflinks	exon	24812220	24812402	.	+	.	gene_id "XLOC_012797"; transcript_id "Novel_000245"; exon_number "7"; gene_type "
At_chr10	Cufflinks	exon	24813137	24813233	.	+	.	gene_id "XLOC_012797"; transcript_id "Novel_000245"; exon_number "8"; gene_type "
At_chr10	Cufflinks	exon	24809655	24810272	.	+	.	gene_id "XLOC_012797"; transcript_id "Novel_000246"; exon_number "1"; gene_type "
At_chr10	Cufflinks	exon	24810748	24810896	.	+	.	gene_id "XLOC_012797"; transcript_id "Novel_000246"; exon_number "2"; gene_type "
At_chr10	Cufflinks	exon	24811567	24811724	.	+	.	gene_id "XLOC_012797"; transcript_id "Novel_000246"; exon_number "3"; gene_type "
At_chr10	Cufflinks	exon	24811816	24811881	.	+	.	gene_id "XLOC_012797"; transcript_id "Novel_000246"; exon_number "4"; gene_type "
At_chr10	Cufflinks	exon	24811986	24813233	.	+	.	gene_id "XLOC_012797"; transcript_id "Novel_000246"; exon_number "5"; gene_type "
At_chr10	Cufflinks	exon	24809657	24809703	.	+	.	gene_id "XLOC_012797"; transcript_id "Novel_000247"; exon_number "1"; gene_type "
At_chr10	Cufflinks	exon	24810166	24810272	.	+	.	gene_id "XLOC_012797"; transcript_id "Novel_000247"; exon_number "2"; gene_type "
At_chr10	Cufflinks	exon	24810748	24810896	.	+	.	gene_id "XLOC_012797"; transcript_id "Novel_000247"; exon_number "3"; gene_type "
At_chr10	Cufflinks	exon	24811567	24813207	.	+	.	gene_id "XLOC_012797"; transcript_id "Novel_000247"; exon_number "4"; gene_type "

At_chr10	Cufflinks	exon	24817339	24817888	.	+	.	gene_id "XLOC_012799"; transcript_id "Novel_000248"; exon_number "1"; gene_type "
At_chr10	Cufflinks	exon	24818099	24818604	.	+	.	gene_id "XLOC_012799"; transcript_id "Novel_000248"; exon_number "2"; gene_type "
At_chr10	Cufflinks	exon	24818689	24818906	.	+	.	gene_id "XLOC_012799"; transcript_id "Novel_000248"; exon_number "3"; gene_type "
At_chr10	Cufflinks	exon	24817339	24817888	.	+	.	gene_id "XLOC_012799"; transcript_id "Novel_000249"; exon_number "1"; gene_type "
At_chr10	Cufflinks	exon	24818099	24818250	.	+	.	gene_id "XLOC_012799"; transcript_id "Novel_000249"; exon_number "2"; gene_type "
At_chr10	Cufflinks	exon	24818329	24818604	.	+	.	gene_id "XLOC_012799"; transcript_id "Novel_000249"; exon_number "3"; gene_type "
At_chr10	Cufflinks	exon	24818689	24818906	.	+	.	gene_id "XLOC_012799"; transcript_id "Novel_000249"; exon_number "4"; gene_type "
At_chr10	Cufflinks	exon	29256450	29256607	.	+	.	gene_id "XLOC_013063"; transcript_id "Novel_000250"; exon_number "1"; gene_type "
At_chr10	Cufflinks	exon	29256884	29257033	.	+	.	gene_id "XLOC_013063"; transcript_id "Novel_000250"; exon_number "2"; gene_type "
At_chr10	Cufflinks	exon	29257118	29257235	.	+	.	gene_id "XLOC_013063"; transcript_id "Novel_000250"; exon_number "3"; gene_type "
At_chr10	Cufflinks	exon	29257318	29257677	.	+	.	gene_id "XLOC_013063"; transcript_id "Novel_000250"; exon_number "4"; gene_type "
At_chr10	Cufflinks	exon	29257846	29271418	.	+	.	gene_id "XLOC_013063"; transcript_id "Novel_000250"; exon_number "5"; gene_type "
At_chr10	Cufflinks	exon	29256450	29256607	.	+	.	gene_id "XLOC_013063"; transcript_id "Novel_000251"; exon_number "1"; gene_type "
At_chr10	Cufflinks	exon	29256884	29257235	.	+	.	gene_id "XLOC_013063"; transcript_id "Novel_000251"; exon_number "2"; gene_type "
At_chr10	Cufflinks	exon	29257318	29257677	.	+	.	gene_id "XLOC_013063"; transcript_id "Novel_000251"; exon_number "3"; gene_type "
At_chr10	Cufflinks	exon	29257846	29257893	.	+	.	gene_id "XLOC_013063"; transcript_id "Novel_000251"; exon_number "4"; gene_type "
At_chr10	Cufflinks	exon	29262958	29271418	.	+	.	gene_id "XLOC_013063"; transcript_id "Novel_000251"; exon_number "5"; gene_type "
At_chr10	Cufflinks	exon	29256450	29256607	.	+	.	gene_id "XLOC_013063"; transcript_id "Novel_000252"; exon_number "1"; gene_type "
At_chr10	Cufflinks	exon	29256884	29257235	.	+	.	gene_id "XLOC_013063"; transcript_id "Novel_000252"; exon_number "2"; gene_type "
At_chr10	Cufflinks	exon	29257318	29257677	.	+	.	gene_id "XLOC_013063"; transcript_id "Novel_000252"; exon_number "3"; gene_type "
At_chr10	Cufflinks	exon	29257846	29271418	.	+	.	gene_id "XLOC_013063"; transcript_id "Novel_000252"; exon_number "4"; gene_type "
At_chr10	Cufflinks	exon	31435889	31436311	.	+	.	gene_id "XLOC_013182"; transcript_id "Novel_000253"; exon_number "1"; gene_type "
At_chr10	Cufflinks	exon	31436600	31436732	.	+	.	gene_id "XLOC_013182"; transcript_id "Novel_000253"; exon_number "2"; gene_type "
At_chr10	Cufflinks	exon	31436842	31436913	.	+	.	gene_id "XLOC_013182"; transcript_id "Novel_000253"; exon_number "3"; gene_type "
At_chr10	Cufflinks	exon	31437017	31437082	.	+	.	gene_id "XLOC_013182"; transcript_id "Novel_000253"; exon_number "4"; gene_type "
At_chr10	Cufflinks	exon	31437172	31437243	.	+	.	gene_id "XLOC_013182"; transcript_id "Novel_000253"; exon_number "5"; gene_type "
At_chr10	Cufflinks	exon	31437345	31437855	.	+	.	gene_id "XLOC_013182"; transcript_id "Novel_000253"; exon_number "6"; gene_type "
At_chr10	Cufflinks	exon	31437967	31438032	.	+	.	gene_id "XLOC_013182"; transcript_id "Novel_000253"; exon_number "7"; gene_type "
At_chr10	Cufflinks	exon	31438111	31438176	.	+	.	gene_id "XLOC_013182"; transcript_id "Novel_000253"; exon_number "8"; gene_type "
At_chr10	Cufflinks	exon	31438264	31438544	.	+	.	gene_id "XLOC_013182"; transcript_id "Novel_000253"; exon_number "9"; gene_type "
At_chr10	Cufflinks	exon	31439066	31439319	.	+	.	gene_id "XLOC_013182"; transcript_id "Novel_000253"; exon_number "10"; gene_type
At_chr10	Cufflinks	exon	31439450	31439721	.	+	.	gene_id "XLOC_013182"; transcript_id "Novel_000253"; exon_number "11"; gene_type
At_chr10	Cufflinks	exon	31439820	31439955	.	+	.	gene_id "XLOC_013182"; transcript_id "Novel_000253"; exon_number "12"; gene_type
At_chr10	Cufflinks	exon	31440079	31440218	.	+	.	gene_id "XLOC_013182"; transcript_id "Novel_000253"; exon_number "13"; gene_type
At_chr10	Cufflinks	exon	31440336	31440492	.	+	.	gene_id "XLOC_013182"; transcript_id "Novel_000253"; exon_number "14"; gene_type

At_chr10	Cufflinks	exon	31440601	31440969	.	+	.	gene_id "XLOC_013182"; transcript_id "Novel_000253"; exon_number "15"; gene_type
At_chr10	Cufflinks	exon	31435970	31436311	.	+	.	gene_id "XLOC_013182"; transcript_id "Novel_000254"; exon_number "1"; gene_type "
At_chr10	Cufflinks	exon	31436600	31436732	.	+	.	gene_id "XLOC_013182"; transcript_id "Novel_000254"; exon_number "2"; gene_type "
At_chr10	Cufflinks	exon	31436842	31436913	.	+	.	gene_id "XLOC_013182"; transcript_id "Novel_000254"; exon_number "3"; gene_type "
At_chr10	Cufflinks	exon	31437017	31437082	.	+	.	gene_id "XLOC_013182"; transcript_id "Novel_000254"; exon_number "4"; gene_type "
At_chr10	Cufflinks	exon	31437172	31437243	.	+	.	gene_id "XLOC_013182"; transcript_id "Novel_000254"; exon_number "5"; gene_type "
At_chr10	Cufflinks	exon	31437345	31437416	.	+	.	gene_id "XLOC_013182"; transcript_id "Novel_000254"; exon_number "6"; gene_type "
At_chr10	Cufflinks	exon	31437784	31437855	.	+	.	gene_id "XLOC_013182"; transcript_id "Novel_000254"; exon_number "7"; gene_type "
At_chr10	Cufflinks	exon	31437967	31438032	.	+	.	gene_id "XLOC_013182"; transcript_id "Novel_000254"; exon_number "8"; gene_type "
At_chr10	Cufflinks	exon	31438111	31438176	.	+	.	gene_id "XLOC_013182"; transcript_id "Novel_000254"; exon_number "9"; gene_type "
At_chr10	Cufflinks	exon	31438264	31438544	.	+	.	gene_id "XLOC_013182"; transcript_id "Novel_000254"; exon_number "10"; gene_type
At_chr10	Cufflinks	exon	31439066	31439319	.	+	.	gene_id "XLOC_013182"; transcript_id "Novel_000254"; exon_number "11"; gene_type
At_chr10	Cufflinks	exon	31439450	31439721	.	+	.	gene_id "XLOC_013182"; transcript_id "Novel_000254"; exon_number "12"; gene_type
At_chr10	Cufflinks	exon	31439820	31439955	.	+	.	gene_id "XLOC_013182"; transcript_id "Novel_000254"; exon_number "13"; gene_type
At_chr10	Cufflinks	exon	31440079	31440218	.	+	.	gene_id "XLOC_013182"; transcript_id "Novel_000254"; exon_number "14"; gene_type
At_chr10	Cufflinks	exon	31440336	31440492	.	+	.	gene_id "XLOC_013182"; transcript_id "Novel_000254"; exon_number "15"; gene_type
At_chr10	Cufflinks	exon	31440601	31441132	.	+	.	gene_id "XLOC_013182"; transcript_id "Novel_000254"; exon_number "16"; gene_type
At_chr10	Cufflinks	exon	40122565	40122882	.	+	.	gene_id "XLOC_013569"; transcript_id "Novel_000255"; exon_number "1"; gene_type "
At_chr10	Cufflinks	exon	40316019	40316246	.	+	.	gene_id "XLOC_013580"; transcript_id "Novel_000256"; exon_number "1"; gene_type "
At_chr10	Cufflinks	exon	40316528	40317692	.	+	.	gene_id "XLOC_013580"; transcript_id "Novel_000256"; exon_number "2"; gene_type "
At_chr10	Cufflinks	exon	40318132	40318197	.	+	.	gene_id "XLOC_013580"; transcript_id "Novel_000256"; exon_number "3"; gene_type "
At_chr10	Cufflinks	exon	40319190	40319698	.	+	.	gene_id "XLOC_013580"; transcript_id "Novel_000256"; exon_number "4"; gene_type "
At_chr10	Cufflinks	exon	40338755	40339410	.	+	.	gene_id "XLOC_013588"; transcript_id "Novel_000257"; exon_number "1"; gene_type "
At_chr10	Cufflinks	exon	40339606	40339717	.	+	.	gene_id "XLOC_013588"; transcript_id "Novel_000257"; exon_number "2"; gene_type "
At_chr10	Cufflinks	exon	40342503	40342853	.	+	.	gene_id "XLOC_013588"; transcript_id "Novel_000257"; exon_number "3"; gene_type "
At_chr10	Cufflinks	exon	40342910	40343064	.	+	.	gene_id "XLOC_013588"; transcript_id "Novel_000258"; exon_number "1"; gene_type "
At_chr10	Cufflinks	exon	40343165	40343259	.	+	.	gene_id "XLOC_013588"; transcript_id "Novel_000258"; exon_number "2"; gene_type "
At_chr10	Cufflinks	exon	40343349	40343706	.	+	.	gene_id "XLOC_013588"; transcript_id "Novel_000258"; exon_number "3"; gene_type "
At_chr10	Cufflinks	exon	40343805	40344018	.	+	.	gene_id "XLOC_013588"; transcript_id "Novel_000258"; exon_number "4"; gene_type "
At_chr10	Cufflinks	exon	40345987	40346677	.	+	.	gene_id "XLOC_013588"; transcript_id "Novel_000258"; exon_number "5"; gene_type "
At_chr10	Cufflinks	exon	40342920	40343706	.	+	.	gene_id "XLOC_013588"; transcript_id "Novel_000259"; exon_number "1"; gene_type "
At_chr10	Cufflinks	exon	40343805	40344018	.	+	.	gene_id "XLOC_013588"; transcript_id "Novel_000259"; exon_number "2"; gene_type "
At_chr10	Cufflinks	exon	40345987	40346844	.	+	.	gene_id "XLOC_013588"; transcript_id "Novel_000259"; exon_number "3"; gene_type "
At_chr10	Cufflinks	exon	40342949	40343064	.	+	.	gene_id "XLOC_013588"; transcript_id "Novel_000260"; exon_number "1"; gene_type "
At_chr10	Cufflinks	exon	40343165	40343706	.	+	.	gene_id "XLOC_013588"; transcript_id "Novel_000260"; exon_number "2"; gene_type "

At_chr10	Cufflinks	exon	40343805	40344018	.	+	.	gene_id "XLOC_013588"; transcript_id "Novel_000260"; exon_number "3"; gene_type "
At_chr10	Cufflinks	exon	40345987	40346672	.	+	.	gene_id "XLOC_013588"; transcript_id "Novel_000260"; exon_number "4"; gene_type "
At_chr10	Cufflinks	exon	40958729	40958818	.	+	.	gene_id "XLOC_013620"; transcript_id "Novel_000261"; exon_number "1"; gene_type "
At_chr10	Cufflinks	exon	40958943	40959123	.	+	.	gene_id "XLOC_013620"; transcript_id "Novel_000261"; exon_number "2"; gene_type "
At_chr10	Cufflinks	exon	40959219	40960647	.	+	.	gene_id "XLOC_013620"; transcript_id "Novel_000261"; exon_number "3"; gene_type "
At_chr10	Cufflinks	exon	45095878	45095994	.	+	.	gene_id "XLOC_013761"; transcript_id "Novel_000262"; exon_number "1"; gene_type "
At_chr10	Cufflinks	exon	45096068	45096134	.	+	.	gene_id "XLOC_013761"; transcript_id "Novel_000262"; exon_number "2"; gene_type "
At_chr10	Cufflinks	exon	45096296	45096455	.	+	.	gene_id "XLOC_013761"; transcript_id "Novel_000262"; exon_number "3"; gene_type "
At_chr10	Cufflinks	exon	45095885	45095994	.	+	.	gene_id "XLOC_013761"; transcript_id "Novel_000263"; exon_number "1"; gene_type "
At_chr10	Cufflinks	exon	45096068	45096191	.	+	.	gene_id "XLOC_013761"; transcript_id "Novel_000263"; exon_number "2"; gene_type "
At_chr10	Cufflinks	exon	46925883	46926146	.	+	.	gene_id "XLOC_013827"; transcript_id "Novel_000264"; exon_number "1"; gene_type "
At_chr10	Cufflinks	exon	55349762	55352450	.	+	.	gene_id "XLOC_014163"; transcript_id "Novel_000265"; exon_number "1"; gene_type "
At_chr10	Cufflinks	exon	55352548	55354210	.	+	.	gene_id "XLOC_014163"; transcript_id "Novel_000265"; exon_number "2"; gene_type "
At_chr10	Cufflinks	exon	60394634	60394659	.	+	.	gene_id "XLOC_014297"; transcript_id "Novel_000266"; exon_number "1"; gene_type "
At_chr10	Cufflinks	exon	60394832	60395016	.	+	.	gene_id "XLOC_014297"; transcript_id "Novel_000266"; exon_number "2"; gene_type "
At_chr10	Cufflinks	exon	63739794	63740049	.	+	.	gene_id "XLOC_014477"; transcript_id "Novel_000267"; exon_number "1"; gene_type "
At_chr10	Cufflinks	exon	63740168	63740746	.	+	.	gene_id "XLOC_014477"; transcript_id "Novel_000267"; exon_number "2"; gene_type "
At_chr10	Cufflinks	exon	67449336	67449755	.	+	.	gene_id "XLOC_014797"; transcript_id "Novel_000268"; exon_number "1"; gene_type "
At_chr10	Cufflinks	exon	67449840	67450230	.	+	.	gene_id "XLOC_014797"; transcript_id "Novel_000268"; exon_number "2"; gene_type "
At_chr10	Cufflinks	exon	68506920	68506952	.	+	.	gene_id "XLOC_014891"; transcript_id "Novel_000269"; exon_number "1"; gene_type "
At_chr10	Cufflinks	exon	68508861	68508964	.	+	.	gene_id "XLOC_014891"; transcript_id "Novel_000269"; exon_number "2"; gene_type "
At_chr10	Cufflinks	exon	68509256	68509813	.	+	.	gene_id "XLOC_014891"; transcript_id "Novel_000269"; exon_number "3"; gene_type "
At_chr10	Cufflinks	exon	68506957	68507428	.	+	.	gene_id "XLOC_014891"; transcript_id "Novel_000270"; exon_number "1"; gene_type "
At_chr10	Cufflinks	exon	68508861	68508964	.	+	.	gene_id "XLOC_014891"; transcript_id "Novel_000270"; exon_number "2"; gene_type "
At_chr10	Cufflinks	exon	68509256	68509813	.	+	.	gene_id "XLOC_014891"; transcript_id "Novel_000270"; exon_number "3"; gene_type "
At_chr10	Cufflinks	exon	68508487	68508964	.	+	.	gene_id "XLOC_014891"; transcript_id "Novel_000271"; exon_number "1"; gene_type "
At_chr10	Cufflinks	exon	68509374	68509749	.	+	.	gene_id "XLOC_014891"; transcript_id "Novel_000271"; exon_number "2"; gene_type "
At_chr10	Cufflinks	exon	68899878	68899994	.	+	.	gene_id "XLOC_014916"; transcript_id "Novel_000272"; exon_number "1"; gene_type "
At_chr10	Cufflinks	exon	68900867	68901094	.	+	.	gene_id "XLOC_014916"; transcript_id "Novel_000272"; exon_number "2"; gene_type "
At_chr10	Cufflinks	exon	68901588	68902737	.	+	.	gene_id "XLOC_014916"; transcript_id "Novel_000272"; exon_number "3"; gene_type "
At_chr10	Cufflinks	exon	68899979	68900140	.	+	.	gene_id "XLOC_014916"; transcript_id "Novel_000273"; exon_number "1"; gene_type "
At_chr10	Cufflinks	exon	68900867	68901094	.	+	.	gene_id "XLOC_014916"; transcript_id "Novel_000273"; exon_number "2"; gene_type "
At_chr10	Cufflinks	exon	68901588	68902170	.	+	.	gene_id "XLOC_014916"; transcript_id "Novel_000273"; exon_number "3"; gene_type "
At_chr10	Cufflinks	exon	68902806	68902826	.	+	.	gene_id "XLOC_014916"; transcript_id "Novel_000273"; exon_number "4"; gene_type "
At_chr10	Cufflinks	exon	68904132	68905156	.	+	.	gene_id "XLOC_014916"; transcript_id "Novel_000274"; exon_number "1"; gene_type "

At_chr10	Cufflinks	exon	68905598	68905999	.	+	.	gene_id "XLOC_014916"; transcript_id "Novel_000274"; exon_number "2"; gene_type "
At_chr10	Cufflinks	exon	68906241	68906747	.	+	.	gene_id "XLOC_014916"; transcript_id "Novel_000274"; exon_number "3"; gene_type "
At_chr10	Cufflinks	exon	68905214	68905999	.	+	.	gene_id "XLOC_014916"; transcript_id "Novel_000275"; exon_number "1"; gene_type "
At_chr10	Cufflinks	exon	68906241	68907300	.	+	.	gene_id "XLOC_014916"; transcript_id "Novel_000275"; exon_number "2"; gene_type "
At_chr10	Cufflinks	exon	69265882	69266042	.	+	.	gene_id "XLOC_014946"; transcript_id "Novel_000276"; exon_number "1"; gene_type "
At_chr10	Cufflinks	exon	69267494	69269729	.	+	.	gene_id "XLOC_014946"; transcript_id "Novel_000276"; exon_number "2"; gene_type "
At_chr10	Cufflinks	exon	70081177	70081354	.	+	.	gene_id "XLOC_015016"; transcript_id "Novel_000277"; exon_number "1"; gene_type "
At_chr10	Cufflinks	exon	70081522	70081924	.	+	.	gene_id "XLOC_015016"; transcript_id "Novel_000277"; exon_number "2"; gene_type "
At_chr10	Cufflinks	exon	70082426	70082508	.	+	.	gene_id "XLOC_015016"; transcript_id "Novel_000277"; exon_number "3"; gene_type "
At_chr10	Cufflinks	exon	70082747	70082785	.	+	.	gene_id "XLOC_015016"; transcript_id "Novel_000277"; exon_number "4"; gene_type "
At_chr10	Cufflinks	exon	70083037	70083107	.	+	.	gene_id "XLOC_015016"; transcript_id "Novel_000277"; exon_number "5"; gene_type "
At_chr10	Cufflinks	exon	70083181	70083295	.	+	.	gene_id "XLOC_015016"; transcript_id "Novel_000277"; exon_number "6"; gene_type "
At_chr10	Cufflinks	exon	70083540	70083670	.	+	.	gene_id "XLOC_015016"; transcript_id "Novel_000277"; exon_number "7"; gene_type "
At_chr10	Cufflinks	exon	70083748	70083948	.	+	.	gene_id "XLOC_015016"; transcript_id "Novel_000277"; exon_number "8"; gene_type "
At_chr10	Cufflinks	exon	73009577	73010055	.	+	.	gene_id "XLOC_015168"; transcript_id "Novel_000278"; exon_number "1"; gene_type "
At_chr10	Cufflinks	exon	73010159	73010215	.	+	.	gene_id "XLOC_015168"; transcript_id "Novel_000278"; exon_number "2"; gene_type "
At_chr10	Cufflinks	exon	73010475	73010979	.	+	.	gene_id "XLOC_015168"; transcript_id "Novel_000278"; exon_number "3"; gene_type "
At_chr10	Cufflinks	exon	73009627	73010055	.	+	.	gene_id "XLOC_015168"; transcript_id "Novel_000279"; exon_number "1"; gene_type "
At_chr10	Cufflinks	exon	73010159	73010215	.	+	.	gene_id "XLOC_015168"; transcript_id "Novel_000279"; exon_number "2"; gene_type "
At_chr10	Cufflinks	exon	73010475	73010566	.	+	.	gene_id "XLOC_015168"; transcript_id "Novel_000279"; exon_number "3"; gene_type "
At_chr10	Cufflinks	exon	73010641	73011136	.	+	.	gene_id "XLOC_015168"; transcript_id "Novel_000279"; exon_number "4"; gene_type "
At_chr10	Cufflinks	exon	74124330	74124948	.	+	.	gene_id "XLOC_015202"; transcript_id "Novel_000280"; exon_number "1"; gene_type "
At_chr10	Cufflinks	exon	74125066	74125607	.	+	.	gene_id "XLOC_015202"; transcript_id "Novel_000280"; exon_number "2"; gene_type "
At_chr10	Cufflinks	exon	74131533	74133350	.	+	.	gene_id "XLOC_015202"; transcript_id "Novel_000280"; exon_number "3"; gene_type "
At_chr10	Cufflinks	exon	74483491	74484148	.	+	.	gene_id "XLOC_015247"; transcript_id "Novel_000281"; exon_number "1"; gene_type "
At_chr10	Cufflinks	exon	74484267	74484444	.	+	.	gene_id "XLOC_015247"; transcript_id "Novel_000281"; exon_number "2"; gene_type "
At_chr10	Cufflinks	exon	74484582	74485156	.	+	.	gene_id "XLOC_015247"; transcript_id "Novel_000281"; exon_number "3"; gene_type "
At_chr10	Cufflinks	exon	74485559	74485991	.	+	.	gene_id "XLOC_015247"; transcript_id "Novel_000281"; exon_number "4"; gene_type "
At_chr10	Cufflinks	exon	75622371	75622528	.	+	.	gene_id "XLOC_015342"; transcript_id "Novel_000282"; exon_number "1"; gene_type "
At_chr10	Cufflinks	exon	75623656	75623691	.	+	.	gene_id "XLOC_015342"; transcript_id "Novel_000282"; exon_number "2"; gene_type "
At_chr10	Cufflinks	exon	75623798	75623883	.	+	.	gene_id "XLOC_015342"; transcript_id "Novel_000282"; exon_number "3"; gene_type "
At_chr10	Cufflinks	exon	75624016	75624156	.	+	.	gene_id "XLOC_015342"; transcript_id "Novel_000282"; exon_number "4"; gene_type "
At_chr10	Cufflinks	exon	75624290	75624630	.	+	.	gene_id "XLOC_015342"; transcript_id "Novel_000282"; exon_number "5"; gene_type "
At_chr10	Cufflinks	exon	76437530	76437616	.	+	.	gene_id "XLOC_015408"; transcript_id "Novel_000283"; exon_number "1"; gene_type "
At_chr10	Cufflinks	exon	76438424	76439583	.	+	.	gene_id "XLOC_015408"; transcript_id "Novel_000283"; exon_number "2"; gene_type "

At_chr10	Cufflinks	exon	76565263	76566045	.	+	.	gene_id "XLOC_015416"; transcript_id "Novel_000284"; exon_number "1"; gene_type "
At_chr10	Cufflinks	exon	76567227	76567635	.	+	.	gene_id "XLOC_015416"; transcript_id "Novel_000284"; exon_number "2"; gene_type "
At_chr10	Cufflinks	exon	76565328	76565401	.	+	.	gene_id "XLOC_015416"; transcript_id "Novel_000285"; exon_number "1"; gene_type "
At_chr10	Cufflinks	exon	76565714	76566045	.	+	.	gene_id "XLOC_015416"; transcript_id "Novel_000285"; exon_number "2"; gene_type "
At_chr10	Cufflinks	exon	76567227	76567359	.	+	.	gene_id "XLOC_015416"; transcript_id "Novel_000285"; exon_number "3"; gene_type "
At_chr10	Cufflinks	exon	76568260	76568676	.	+	.	gene_id "XLOC_015416"; transcript_id "Novel_000285"; exon_number "4"; gene_type "
At_chr10	Cufflinks	exon	76565373	76565401	.	+	.	gene_id "XLOC_015416"; transcript_id "Novel_000286"; exon_number "1"; gene_type "
At_chr10	Cufflinks	exon	76565714	76566045	.	+	.	gene_id "XLOC_015416"; transcript_id "Novel_000286"; exon_number "2"; gene_type "
At_chr10	Cufflinks	exon	76567227	76567593	.	+	.	gene_id "XLOC_015416"; transcript_id "Novel_000286"; exon_number "3"; gene_type "
At_chr10	Cufflinks	exon	76616348	76616877	.	+	.	gene_id "XLOC_015427"; transcript_id "Novel_000287"; exon_number "1"; gene_type "
At_chr10	Cufflinks	exon	76616952	76617247	.	+	.	gene_id "XLOC_015427"; transcript_id "Novel_000287"; exon_number "2"; gene_type "
At_chr10	Cufflinks	exon	76617341	76617481	.	+	.	gene_id "XLOC_015427"; transcript_id "Novel_000287"; exon_number "3"; gene_type "
At_chr10	Cufflinks	exon	76617571	76618090	.	+	.	gene_id "XLOC_015427"; transcript_id "Novel_000287"; exon_number "4"; gene_type "
At_chr10	Cufflinks	exon	76655393	76655591	.	+	.	gene_id "XLOC_015430"; transcript_id "Novel_000288"; exon_number "1"; gene_type "
At_chr10	Cufflinks	exon	76655889	76656127	.	+	.	gene_id "XLOC_015430"; transcript_id "Novel_000288"; exon_number "2"; gene_type "
At_chr10	Cufflinks	exon	76656406	76656563	.	+	.	gene_id "XLOC_015430"; transcript_id "Novel_000288"; exon_number "3"; gene_type "
At_chr10	Cufflinks	exon	76656786	76656892	.	+	.	gene_id "XLOC_015430"; transcript_id "Novel_000288"; exon_number "4"; gene_type "
At_chr10	Cufflinks	exon	76657000	76657100	.	+	.	gene_id "XLOC_015430"; transcript_id "Novel_000288"; exon_number "5"; gene_type "
At_chr10	Cufflinks	exon	76657181	76657257	.	+	.	gene_id "XLOC_015430"; transcript_id "Novel_000288"; exon_number "6"; gene_type "
At_chr10	Cufflinks	exon	76657449	76657568	.	+	.	gene_id "XLOC_015430"; transcript_id "Novel_000288"; exon_number "7"; gene_type "
At_chr10	Cufflinks	exon	76657657	76657887	.	+	.	gene_id "XLOC_015430"; transcript_id "Novel_000288"; exon_number "8"; gene_type "
At_chr10	Cufflinks	exon	76658023	76658139	.	+	.	gene_id "XLOC_015430"; transcript_id "Novel_000288"; exon_number "9"; gene_type "
At_chr10	Cufflinks	exon	76658234	76658308	.	+	.	gene_id "XLOC_015430"; transcript_id "Novel_000288"; exon_number "10"; gene_type
At_chr10	Cufflinks	exon	76658393	76658470	.	+	.	gene_id "XLOC_015430"; transcript_id "Novel_000288"; exon_number "11"; gene_type
At_chr10	Cufflinks	exon	76658542	76658601	.	+	.	gene_id "XLOC_015430"; transcript_id "Novel_000288"; exon_number "12"; gene_type
At_chr10	Cufflinks	exon	76658686	76658748	.	+	.	gene_id "XLOC_015430"; transcript_id "Novel_000288"; exon_number "13"; gene_type
At_chr10	Cufflinks	exon	76658843	76658987	.	+	.	gene_id "XLOC_015430"; transcript_id "Novel_000288"; exon_number "14"; gene_type
At_chr10	Cufflinks	exon	76659087	76659225	.	+	.	gene_id "XLOC_015430"; transcript_id "Novel_000288"; exon_number "15"; gene_type
At_chr10	Cufflinks	exon	76659371	76659448	.	+	.	gene_id "XLOC_015430"; transcript_id "Novel_000288"; exon_number "16"; gene_type
At_chr10	Cufflinks	exon	76659533	76659692	.	+	.	gene_id "XLOC_015430"; transcript_id "Novel_000288"; exon_number "17"; gene_type
At_chr10	Cufflinks	exon	76659770	76659868	.	+	.	gene_id "XLOC_015430"; transcript_id "Novel_000288"; exon_number "18"; gene_type
At_chr10	Cufflinks	exon	76659979	76660083	.	+	.	gene_id "XLOC_015430"; transcript_id "Novel_000288"; exon_number "19"; gene_type
At_chr10	Cufflinks	exon	76660201	76660728	.	+	.	gene_id "XLOC_015430"; transcript_id "Novel_000288"; exon_number "20"; gene_type
At_chr10	Cufflinks	exon	76871575	76871736	.	+	.	gene_id "XLOC_015462"; transcript_id "Novel_000289"; exon_number "1"; gene_type "
At_chr10	Cufflinks	exon	76871832	76871955	.	+	.	gene_id "XLOC_015462"; transcript_id "Novel_000289"; exon_number "2"; gene_type "

At_chr10	Cufflinks	exon	76872045	76872110	.	+	.	gene_id "XLOC_015462"; transcript_id "Novel_000289"; exon_number "3"; gene_type "
At_chr10	Cufflinks	exon	76871613	76872219	.	+	.	gene_id "XLOC_015462"; transcript_id "Novel_000290"; exon_number "1"; gene_type "
At_chr10	Cufflinks	exon	76872331	76872396	.	+	.	gene_id "XLOC_015462"; transcript_id "Novel_000290"; exon_number "2"; gene_type "
At_chr10	Cufflinks	exon	76872978	76873026	.	+	.	gene_id "XLOC_015462"; transcript_id "Novel_000290"; exon_number "3"; gene_type "
At_chr10	Cufflinks	exon	76873133	76873218	.	+	.	gene_id "XLOC_015462"; transcript_id "Novel_000290"; exon_number "4"; gene_type "
At_chr10	Cufflinks	exon	76873321	76873399	.	+	.	gene_id "XLOC_015462"; transcript_id "Novel_000290"; exon_number "5"; gene_type "
At_chr10	Cufflinks	exon	76873525	76873627	.	+	.	gene_id "XLOC_015462"; transcript_id "Novel_000290"; exon_number "6"; gene_type "
At_chr10	Cufflinks	exon	76873744	76873802	.	+	.	gene_id "XLOC_015462"; transcript_id "Novel_000290"; exon_number "7"; gene_type "
At_chr10	Cufflinks	exon	76873906	76874586	.	+	.	gene_id "XLOC_015462"; transcript_id "Novel_000290"; exon_number "8"; gene_type "
At_chr10	Cufflinks	exon	76872253	76872396	.	+	.	gene_id "XLOC_015462"; transcript_id "Novel_000291"; exon_number "1"; gene_type "
At_chr10	Cufflinks	exon	76872978	76873026	.	+	.	gene_id "XLOC_015462"; transcript_id "Novel_000291"; exon_number "2"; gene_type "
At_chr10	Cufflinks	exon	76873133	76873218	.	+	.	gene_id "XLOC_015462"; transcript_id "Novel_000291"; exon_number "3"; gene_type "
At_chr10	Cufflinks	exon	76873321	76873399	.	+	.	gene_id "XLOC_015462"; transcript_id "Novel_000291"; exon_number "4"; gene_type "
At_chr10	Cufflinks	exon	76873525	76873627	.	+	.	gene_id "XLOC_015462"; transcript_id "Novel_000291"; exon_number "5"; gene_type "
At_chr10	Cufflinks	exon	76873744	76873802	.	+	.	gene_id "XLOC_015462"; transcript_id "Novel_000291"; exon_number "6"; gene_type "
At_chr10	Cufflinks	exon	76873906	76874465	.	+	.	gene_id "XLOC_015462"; transcript_id "Novel_000291"; exon_number "7"; gene_type "
At_chr10	Cufflinks	exon	76978812	76978907	.	+	.	gene_id "XLOC_015484"; transcript_id "Novel_000292"; exon_number "1"; gene_type "
At_chr10	Cufflinks	exon	76979023	76979225	.	+	.	gene_id "XLOC_015484"; transcript_id "Novel_000292"; exon_number "2"; gene_type "
At_chr10	Cufflinks	exon	76979341	76979395	.	+	.	gene_id "XLOC_015484"; transcript_id "Novel_000292"; exon_number "3"; gene_type "
At_chr10	Cufflinks	exon	76979472	76979569	.	+	.	gene_id "XLOC_015484"; transcript_id "Novel_000292"; exon_number "4"; gene_type "
At_chr10	Cufflinks	exon	76979655	76981119	.	+	.	gene_id "XLOC_015484"; transcript_id "Novel_000292"; exon_number "5"; gene_type "
At_chr10	Cufflinks	exon	76981224	76981343	.	+	.	gene_id "XLOC_015484"; transcript_id "Novel_000292"; exon_number "6"; gene_type "
At_chr10	Cufflinks	exon	76981528	76981794	.	+	.	gene_id "XLOC_015484"; transcript_id "Novel_000292"; exon_number "7"; gene_type "
At_chr10	Cufflinks	exon	76982476	76982661	.	+	.	gene_id "XLOC_015484"; transcript_id "Novel_000292"; exon_number "8"; gene_type "
At_chr10	Cufflinks	exon	76982851	76982931	.	+	.	gene_id "XLOC_015484"; transcript_id "Novel_000292"; exon_number "9"; gene_type "
At_chr10	Cufflinks	exon	76983178	76983235	.	+	.	gene_id "XLOC_015484"; transcript_id "Novel_000292"; exon_number "10"; gene_type
At_chr10	Cufflinks	exon	76983313	76983827	.	+	.	gene_id "XLOC_015484"; transcript_id "Novel_000292"; exon_number "11"; gene_type
At_chr10	Cufflinks	exon	76983946	76983999	.	+	.	gene_id "XLOC_015484"; transcript_id "Novel_000292"; exon_number "12"; gene_type
At_chr10	Cufflinks	exon	76984536	76984593	.	+	.	gene_id "XLOC_015484"; transcript_id "Novel_000292"; exon_number "13"; gene_type
At_chr10	Cufflinks	exon	76984706	76984896	.	+	.	gene_id "XLOC_015484"; transcript_id "Novel_000292"; exon_number "14"; gene_type
At_chr10	Cufflinks	exon	76984986	76985039	.	+	.	gene_id "XLOC_015484"; transcript_id "Novel_000292"; exon_number "15"; gene_type
At_chr10	Cufflinks	exon	76985130	76985149	.	+	.	gene_id "XLOC_015484"; transcript_id "Novel_000292"; exon_number "16"; gene_type
At_chr10	Cufflinks	exon	76978846	76978907	.	+	.	gene_id "XLOC_015484"; transcript_id "Novel_000293"; exon_number "1"; gene_type "
At_chr10	Cufflinks	exon	76979023	76979225	.	+	.	gene_id "XLOC_015484"; transcript_id "Novel_000293"; exon_number "2"; gene_type "
At_chr10	Cufflinks	exon	76979341	76979395	.	+	.	gene_id "XLOC_015484"; transcript_id "Novel_000293"; exon_number "3"; gene_type "

At_chr10	Cufflinks	exon	76979472	76979569	.	+	.	gene_id "XLOC_015484"; transcript_id "Novel_000293"; exon_number "4"; gene_type "
At_chr10	Cufflinks	exon	76979655	76981119	.	+	.	gene_id "XLOC_015484"; transcript_id "Novel_000293"; exon_number "5"; gene_type "
At_chr10	Cufflinks	exon	76981224	76981343	.	+	.	gene_id "XLOC_015484"; transcript_id "Novel_000293"; exon_number "6"; gene_type "
At_chr10	Cufflinks	exon	76981528	76982726	.	+	.	gene_id "XLOC_015484"; transcript_id "Novel_000293"; exon_number "7"; gene_type "
At_chr10	Cufflinks	exon	77053647	77053704	.	+	.	gene_id "XLOC_015497"; transcript_id "Novel_000294"; exon_number "1"; gene_type "
At_chr10	Cufflinks	exon	77054351	77054431	.	+	.	gene_id "XLOC_015497"; transcript_id "Novel_000294"; exon_number "2"; gene_type "
At_chr10	Cufflinks	exon	77054580	77054677	.	+	.	gene_id "XLOC_015497"; transcript_id "Novel_000294"; exon_number "3"; gene_type "
At_chr10	Cufflinks	exon	77054766	77055611	.	+	.	gene_id "XLOC_015497"; transcript_id "Novel_000294"; exon_number "4"; gene_type "
At_chr10	Cufflinks	exon	84285754	84287128	.	+	.	gene_id "XLOC_015813"; transcript_id "Novel_000295"; exon_number "1"; gene_type "
At_chr10	Cufflinks	exon	84287709	84289817	.	+	.	gene_id "XLOC_015813"; transcript_id "Novel_000295"; exon_number "2"; gene_type "
At_chr10	Cufflinks	exon	84285892	84287128	.	+	.	gene_id "XLOC_015813"; transcript_id "Novel_000296"; exon_number "1"; gene_type "
At_chr10	Cufflinks	exon	84287664	84289851	.	+	.	gene_id "XLOC_015813"; transcript_id "Novel_000296"; exon_number "2"; gene_type "
At_chr10	Cufflinks	exon	85359118	85359411	.	+	.	gene_id "XLOC_015849"; transcript_id "Novel_000297"; exon_number "1"; gene_type "
At_chr10	Cufflinks	exon	85359496	85360215	.	+	.	gene_id "XLOC_015849"; transcript_id "Novel_000297"; exon_number "2"; gene_type "
At_chr10	Cufflinks	exon	85359171	85359411	.	+	.	gene_id "XLOC_015849"; transcript_id "Novel_000298"; exon_number "1"; gene_type "
At_chr10	Cufflinks	exon	85359496	85359690	.	+	.	gene_id "XLOC_015849"; transcript_id "Novel_000298"; exon_number "2"; gene_type "
At_chr10	Cufflinks	exon	85360502	85365057	.	+	.	gene_id "XLOC_015849"; transcript_id "Novel_000298"; exon_number "3"; gene_type "
At_chr10	Cufflinks	exon	85359242	85359690	.	+	.	gene_id "XLOC_015849"; transcript_id "Novel_000299"; exon_number "1"; gene_type "
At_chr10	Cufflinks	exon	85360502	85361156	.	+	.	gene_id "XLOC_015849"; transcript_id "Novel_000299"; exon_number "2"; gene_type "
At_chr10	Cufflinks	exon	91458713	91459177	.	+	.	gene_id "XLOC_016041"; transcript_id "Novel_000300"; exon_number "1"; gene_type "
At_chr10	Cufflinks	exon	91459336	91459562	.	+	.	gene_id "XLOC_016041"; transcript_id "Novel_000300"; exon_number "2"; gene_type "
At_chr10	Cufflinks	exon	92100045	92100095	.	+	.	gene_id "XLOC_016052"; transcript_id "Novel_000301"; exon_number "1"; gene_type "
At_chr10	Cufflinks	exon	92100277	92100341	.	+	.	gene_id "XLOC_016052"; transcript_id "Novel_000301"; exon_number "2"; gene_type "
At_chr10	Cufflinks	exon	92100472	92100700	.	+	.	gene_id "XLOC_016052"; transcript_id "Novel_000301"; exon_number "3"; gene_type "
At_chr10	Cufflinks	exon	92100050	92100095	.	+	.	gene_id "XLOC_016052"; transcript_id "Novel_000302"; exon_number "1"; gene_type "
At_chr10	Cufflinks	exon	92100277	92100341	.	+	.	gene_id "XLOC_016052"; transcript_id "Novel_000302"; exon_number "2"; gene_type "
At_chr10	Cufflinks	exon	92100472	92100539	.	+	.	gene_id "XLOC_016052"; transcript_id "Novel_000302"; exon_number "3"; gene_type "
At_chr10	Cufflinks	exon	92106046	92106201	.	+	.	gene_id "XLOC_016052"; transcript_id "Novel_000302"; exon_number "4"; gene_type "
At_chr10	Cufflinks	exon	92673239	92673594	.	+	.	gene_id "XLOC_016075"; transcript_id "Novel_000303"; exon_number "1"; gene_type "
At_chr10	Cufflinks	exon	92673719	92674195	.	+	.	gene_id "XLOC_016075"; transcript_id "Novel_000303"; exon_number "2"; gene_type "
At_chr10	Cufflinks	exon	3117053	3117292	.	-	.	gene_id "XLOC_016316"; transcript_id "Novel_000304"; exon_number "1"; gene_type "
At_chr10	Cufflinks	exon	4151386	4152738	.	-	.	gene_id "XLOC_016375"; transcript_id "Novel_000305"; exon_number "1"; gene_type "
At_chr10	Cufflinks	exon	4154060	4154444	.	-	.	gene_id "XLOC_016375"; transcript_id "Novel_000305"; exon_number "2"; gene_type "
At_chr10	Cufflinks	exon	4151390	4152738	.	-	.	gene_id "XLOC_016375"; transcript_id "Novel_000306"; exon_number "1"; gene_type "
At_chr10	Cufflinks	exon	4154060	4154188	.	-	.	gene_id "XLOC_016375"; transcript_id "Novel_000306"; exon_number "2"; gene_type "

At_chr10	Cufflinks	exon	4154301	4154444	.	-	.	gene_id "XLOC_016375"; transcript_id "Novel_000306"; exon_number "3"; gene_type "
At_chr10	Cufflinks	exon	11677336	11678833	.	-	.	gene_id "XLOC_016640"; transcript_id "Novel_000307"; exon_number "1"; gene_type "
At_chr10	Cufflinks	exon	11679010	11679565	.	-	.	gene_id "XLOC_016640"; transcript_id "Novel_000307"; exon_number "2"; gene_type "
At_chr10	Cufflinks	exon	11679779	11679892	.	-	.	gene_id "XLOC_016640"; transcript_id "Novel_000307"; exon_number "3"; gene_type "
At_chr10	Cufflinks	exon	11679978	11680200	.	-	.	gene_id "XLOC_016640"; transcript_id "Novel_000307"; exon_number "4"; gene_type "
At_chr10	Cufflinks	exon	11680753	11680841	.	-	.	gene_id "XLOC_016640"; transcript_id "Novel_000307"; exon_number "5"; gene_type "
At_chr10	Cufflinks	exon	11681000	11681110	.	-	.	gene_id "XLOC_016640"; transcript_id "Novel_000307"; exon_number "6"; gene_type "
At_chr10	Cufflinks	exon	11681252	11681334	.	-	.	gene_id "XLOC_016640"; transcript_id "Novel_000307"; exon_number "7"; gene_type "
At_chr10	Cufflinks	exon	11677617	11679565	.	-	.	gene_id "XLOC_016640"; transcript_id "Novel_000308"; exon_number "1"; gene_type "
At_chr10	Cufflinks	exon	11679779	11679889	.	-	.	gene_id "XLOC_016640"; transcript_id "Novel_000308"; exon_number "2"; gene_type "
At_chr10	Cufflinks	exon	11679978	11680200	.	-	.	gene_id "XLOC_016640"; transcript_id "Novel_000308"; exon_number "3"; gene_type "
At_chr10	Cufflinks	exon	11680753	11680841	.	-	.	gene_id "XLOC_016640"; transcript_id "Novel_000308"; exon_number "4"; gene_type "
At_chr10	Cufflinks	exon	11681000	11681110	.	-	.	gene_id "XLOC_016640"; transcript_id "Novel_000308"; exon_number "5"; gene_type "
At_chr10	Cufflinks	exon	11681252	11681389	.	-	.	gene_id "XLOC_016640"; transcript_id "Novel_000308"; exon_number "6"; gene_type "
At_chr10	Cufflinks	exon	19199878	19202300	.	-	.	gene_id "XLOC_016891"; transcript_id "Novel_000309"; exon_number "1"; gene_type "
At_chr10	Cufflinks	exon	19203838	19204197	.	-	.	gene_id "XLOC_016891"; transcript_id "Novel_000309"; exon_number "2"; gene_type "
At_chr10	Cufflinks	exon	23699147	23699210	.	-	.	gene_id "XLOC_017133"; transcript_id "Novel_000310"; exon_number "1"; gene_type "
At_chr10	Cufflinks	exon	23699325	23699535	.	-	.	gene_id "XLOC_017133"; transcript_id "Novel_000310"; exon_number "2"; gene_type "
At_chr10	Cufflinks	exon	23891692	23893358	.	-	.	gene_id "XLOC_017157"; transcript_id "Novel_000311"; exon_number "1"; gene_type "
At_chr10	Cufflinks	exon	23893452	23893490	.	-	.	gene_id "XLOC_017157"; transcript_id "Novel_000311"; exon_number "2"; gene_type "
At_chr10	Cufflinks	exon	23894272	23894507	.	-	.	gene_id "XLOC_017157"; transcript_id "Novel_000311"; exon_number "3"; gene_type "
At_chr10	Cufflinks	exon	23895589	23896658	.	-	.	gene_id "XLOC_017158"; transcript_id "Novel_000312"; exon_number "1"; gene_type "
At_chr10	Cufflinks	exon	23896729	23896827	.	-	.	gene_id "XLOC_017158"; transcript_id "Novel_000312"; exon_number "2"; gene_type "
At_chr10	Cufflinks	exon	23896914	23897075	.	-	.	gene_id "XLOC_017158"; transcript_id "Novel_000312"; exon_number "3"; gene_type "
At_chr10	Cufflinks	exon	23897335	23897415	.	-	.	gene_id "XLOC_017158"; transcript_id "Novel_000312"; exon_number "4"; gene_type "
At_chr10	Cufflinks	exon	23906996	23907073	.	-	.	gene_id "XLOC_017158"; transcript_id "Novel_000312"; exon_number "5"; gene_type "
At_chr10	Cufflinks	exon	23907191	23907255	.	-	.	gene_id "XLOC_017158"; transcript_id "Novel_000312"; exon_number "6"; gene_type "
At_chr10	Cufflinks	exon	23907359	23907440	.	-	.	gene_id "XLOC_017158"; transcript_id "Novel_000312"; exon_number "7"; gene_type "
At_chr10	Cufflinks	exon	23907978	23908056	.	-	.	gene_id "XLOC_017158"; transcript_id "Novel_000312"; exon_number "8"; gene_type "
At_chr10	Cufflinks	exon	25079003	25079153	.	-	.	gene_id "XLOC_017207"; transcript_id "Novel_000313"; exon_number "1"; gene_type "
At_chr10	Cufflinks	exon	25079287	25079413	.	-	.	gene_id "XLOC_017207"; transcript_id "Novel_000313"; exon_number "2"; gene_type "
At_chr10	Cufflinks	exon	25079520	25079564	.	-	.	gene_id "XLOC_017207"; transcript_id "Novel_000313"; exon_number "3"; gene_type "
At_chr10	Cufflinks	exon	25097968	25098689	.	-	.	gene_id "XLOC_017211"; transcript_id "Novel_000314"; exon_number "1"; gene_type "
At_chr10	Cufflinks	exon	25098810	25098898	.	-	.	gene_id "XLOC_017211"; transcript_id "Novel_000314"; exon_number "2"; gene_type "
At_chr10	Cufflinks	exon	25099013	25099072	.	-	.	gene_id "XLOC_017211"; transcript_id "Novel_000314"; exon_number "3"; gene_type "

At_chr10	Cufflinks	exon	25098067	25098689	.	-	.	gene_id "XLOC_017211"; transcript_id "Novel_000315"; exon_number "1"; gene_type "
At_chr10	Cufflinks	exon	25098810	25098938	.	-	.	gene_id "XLOC_017211"; transcript_id "Novel_000315"; exon_number "2"; gene_type "
At_chr10	Cufflinks	exon	26479272	26479491	.	-	.	gene_id "XLOC_017296"; transcript_id "Novel_000316"; exon_number "1"; gene_type "
At_chr10	Cufflinks	exon	27852019	27852347	.	-	.	gene_id "XLOC_017409"; transcript_id "Novel_000317"; exon_number "1"; gene_type "
At_chr10	Cufflinks	exon	27852770	27853022	.	-	.	gene_id "XLOC_017409"; transcript_id "Novel_000317"; exon_number "2"; gene_type "
At_chr10	Cufflinks	exon	27853113	27853355	.	-	.	gene_id "XLOC_017409"; transcript_id "Novel_000317"; exon_number "3"; gene_type "
At_chr10	Cufflinks	exon	27853462	27853589	.	-	.	gene_id "XLOC_017409"; transcript_id "Novel_000317"; exon_number "4"; gene_type "
At_chr10	Cufflinks	exon	27853683	27853992	.	-	.	gene_id "XLOC_017409"; transcript_id "Novel_000317"; exon_number "5"; gene_type "
At_chr10	Cufflinks	exon	31445928	31445998	.	-	.	gene_id "XLOC_017625"; transcript_id "Novel_000318"; exon_number "1"; gene_type "
At_chr10	Cufflinks	exon	31446103	31446442	.	-	.	gene_id "XLOC_017625"; transcript_id "Novel_000318"; exon_number "2"; gene_type "
At_chr10	Cufflinks	exon	31959147	31959783	.	-	.	gene_id "XLOC_017646"; transcript_id "Novel_000319"; exon_number "1"; gene_type "
At_chr10	Cufflinks	exon	31960224	31960287	.	-	.	gene_id "XLOC_017646"; transcript_id "Novel_000319"; exon_number "2"; gene_type "
At_chr10	Cufflinks	exon	31960453	31960773	.	-	.	gene_id "XLOC_017646"; transcript_id "Novel_000319"; exon_number "3"; gene_type "
At_chr10	Cufflinks	exon	31963595	31963839	.	-	.	gene_id "XLOC_017646"; transcript_id "Novel_000319"; exon_number "4"; gene_type "
At_chr10	Cufflinks	exon	31963941	31964020	.	-	.	gene_id "XLOC_017646"; transcript_id "Novel_000319"; exon_number "5"; gene_type "
At_chr10	Cufflinks	exon	31964289	31964459	.	-	.	gene_id "XLOC_017646"; transcript_id "Novel_000319"; exon_number "6"; gene_type "
At_chr10	Cufflinks	exon	31964595	31965378	.	-	.	gene_id "XLOC_017646"; transcript_id "Novel_000319"; exon_number "7"; gene_type "
At_chr10	Cufflinks	exon	31965687	31965831	.	-	.	gene_id "XLOC_017646"; transcript_id "Novel_000319"; exon_number "8"; gene_type "
At_chr10	Cufflinks	exon	31966475	31968683	.	-	.	gene_id "XLOC_017646"; transcript_id "Novel_000319"; exon_number "9"; gene_type "
At_chr10	Cufflinks	exon	31959429	31959783	.	-	.	gene_id "XLOC_017646"; transcript_id "Novel_000320"; exon_number "1"; gene_type "
At_chr10	Cufflinks	exon	31960224	31960287	.	-	.	gene_id "XLOC_017646"; transcript_id "Novel_000320"; exon_number "2"; gene_type "
At_chr10	Cufflinks	exon	31960453	31960773	.	-	.	gene_id "XLOC_017646"; transcript_id "Novel_000320"; exon_number "3"; gene_type "
At_chr10	Cufflinks	exon	31963595	31963839	.	-	.	gene_id "XLOC_017646"; transcript_id "Novel_000320"; exon_number "4"; gene_type "
At_chr10	Cufflinks	exon	31963941	31964020	.	-	.	gene_id "XLOC_017646"; transcript_id "Novel_000320"; exon_number "5"; gene_type "
At_chr10	Cufflinks	exon	31964289	31964459	.	-	.	gene_id "XLOC_017646"; transcript_id "Novel_000320"; exon_number "6"; gene_type "
At_chr10	Cufflinks	exon	31964595	31965378	.	-	.	gene_id "XLOC_017646"; transcript_id "Novel_000320"; exon_number "7"; gene_type "
At_chr10	Cufflinks	exon	31965687	31965831	.	-	.	gene_id "XLOC_017646"; transcript_id "Novel_000320"; exon_number "8"; gene_type "
At_chr10	Cufflinks	exon	31966475	31968114	.	-	.	gene_id "XLOC_017646"; transcript_id "Novel_000320"; exon_number "9"; gene_type "
At_chr10	Cufflinks	exon	31968438	31968674	.	-	.	gene_id "XLOC_017646"; transcript_id "Novel_000320"; exon_number "10"; gene_type
At_chr10	Cufflinks	exon	36664550	36664595	.	-	.	gene_id "XLOC_017949"; transcript_id "Novel_000321"; exon_number "1"; gene_type "
At_chr10	Cufflinks	exon	36669182	36669375	.	-	.	gene_id "XLOC_017949"; transcript_id "Novel_000321"; exon_number "2"; gene_type "
At_chr10	Cufflinks	exon	47395571	47396303	.	-	.	gene_id "XLOC_018393"; transcript_id "Novel_000322"; exon_number "1"; gene_type "
At_chr10	Cufflinks	exon	47396413	47397079	.	-	.	gene_id "XLOC_018393"; transcript_id "Novel_000322"; exon_number "2"; gene_type "
At_chr10	Cufflinks	exon	47397222	47397254	.	-	.	gene_id "XLOC_018393"; transcript_id "Novel_000322"; exon_number "3"; gene_type "
At_chr10	Cufflinks	exon	53231718	53231898	.	-	.	gene_id "XLOC_018632"; transcript_id "Novel_000323"; exon_number "1"; gene_type "

At_chr10	Cufflinks	exon	53231996	53232064	.	-	.	gene_id "XLOC_018632"; transcript_id "Novel_000323"; exon_number "2"; gene_type "
At_chr10	Cufflinks	exon	53232201	53232299	.	-	.	gene_id "XLOC_018632"; transcript_id "Novel_000323"; exon_number "3"; gene_type "
At_chr10	Cufflinks	exon	53456483	53457707	.	-	.	gene_id "XLOC_018642"; transcript_id "Novel_000324"; exon_number "1"; gene_type "
At_chr10	Cufflinks	exon	53457868	53458017	.	-	.	gene_id "XLOC_018642"; transcript_id "Novel_000324"; exon_number "2"; gene_type "
At_chr10	Cufflinks	exon	53458178	53458275	.	-	.	gene_id "XLOC_018642"; transcript_id "Novel_000324"; exon_number "3"; gene_type "
At_chr10	Cufflinks	exon	53458379	53458519	.	-	.	gene_id "XLOC_018642"; transcript_id "Novel_000324"; exon_number "4"; gene_type "
At_chr10	Cufflinks	exon	53458972	53459094	.	-	.	gene_id "XLOC_018642"; transcript_id "Novel_000324"; exon_number "5"; gene_type "
At_chr10	Cufflinks	exon	66193422	66196057	.	-	.	gene_id "XLOC_019380"; transcript_id "Novel_000325"; exon_number "1"; gene_type "
At_chr10	Cufflinks	exon	66196210	66196324	.	-	.	gene_id "XLOC_019380"; transcript_id "Novel_000325"; exon_number "2"; gene_type "
At_chr10	Cufflinks	exon	66196474	66196711	.	-	.	gene_id "XLOC_019380"; transcript_id "Novel_000325"; exon_number "3"; gene_type "
At_chr10	Cufflinks	exon	66193425	66196057	.	-	.	gene_id "XLOC_019380"; transcript_id "Novel_000326"; exon_number "1"; gene_type "
At_chr10	Cufflinks	exon	66196210	66196284	.	-	.	gene_id "XLOC_019380"; transcript_id "Novel_000326"; exon_number "2"; gene_type "
At_chr10	Cufflinks	exon	66196474	66196720	.	-	.	gene_id "XLOC_019380"; transcript_id "Novel_000326"; exon_number "3"; gene_type "
At_chr10	Cufflinks	exon	68268583	68268861	.	-	.	gene_id "XLOC_019592"; transcript_id "Novel_000327"; exon_number "1"; gene_type "
At_chr10	Cufflinks	exon	68268936	68268971	.	-	.	gene_id "XLOC_019592"; transcript_id "Novel_000327"; exon_number "2"; gene_type "
At_chr10	Cufflinks	exon	68955570	68955897	.	-	.	gene_id "XLOC_019645"; transcript_id "Novel_000328"; exon_number "1"; gene_type "
At_chr10	Cufflinks	exon	68956358	68957120	.	-	.	gene_id "XLOC_019645"; transcript_id "Novel_000328"; exon_number "2"; gene_type "
At_chr10	Cufflinks	exon	68957943	68958211	.	-	.	gene_id "XLOC_019645"; transcript_id "Novel_000328"; exon_number "3"; gene_type "
At_chr10	Cufflinks	exon	68958283	68958513	.	-	.	gene_id "XLOC_019645"; transcript_id "Novel_000328"; exon_number "4"; gene_type "
At_chr10	Cufflinks	exon	68958618	68959358	.	-	.	gene_id "XLOC_019645"; transcript_id "Novel_000328"; exon_number "5"; gene_type "
At_chr10	Cufflinks	exon	68959665	68959837	.	-	.	gene_id "XLOC_019645"; transcript_id "Novel_000328"; exon_number "6"; gene_type "
At_chr10	Cufflinks	exon	68957094	68957120	.	-	.	gene_id "XLOC_019645"; transcript_id "Novel_000329"; exon_number "1"; gene_type "
At_chr10	Cufflinks	exon	68957943	68958211	.	-	.	gene_id "XLOC_019645"; transcript_id "Novel_000329"; exon_number "2"; gene_type "
At_chr10	Cufflinks	exon	68958283	68958513	.	-	.	gene_id "XLOC_019645"; transcript_id "Novel_000329"; exon_number "3"; gene_type "
At_chr10	Cufflinks	exon	68958618	68959643	.	-	.	gene_id "XLOC_019645"; transcript_id "Novel_000329"; exon_number "4"; gene_type "
At_chr10	Cufflinks	exon	68957176	68958211	.	-	.	gene_id "XLOC_019645"; transcript_id "Novel_000330"; exon_number "1"; gene_type "
At_chr10	Cufflinks	exon	68958283	68958513	.	-	.	gene_id "XLOC_019645"; transcript_id "Novel_000330"; exon_number "2"; gene_type "
At_chr10	Cufflinks	exon	68958618	68959358	.	-	.	gene_id "XLOC_019645"; transcript_id "Novel_000330"; exon_number "3"; gene_type "
At_chr10	Cufflinks	exon	68959665	68959786	.	-	.	gene_id "XLOC_019645"; transcript_id "Novel_000330"; exon_number "4"; gene_type "
At_chr10	Cufflinks	exon	69273380	69276854	.	-	.	gene_id "XLOC_019672"; transcript_id "Novel_000331"; exon_number "1"; gene_type "
At_chr10	Cufflinks	exon	69277199	69277255	.	-	.	gene_id "XLOC_019672"; transcript_id "Novel_000331"; exon_number "2"; gene_type "
At_chr10	Cufflinks	exon	69273558	69275754	.	-	.	gene_id "XLOC_019672"; transcript_id "Novel_000332"; exon_number "1"; gene_type "
At_chr10	Cufflinks	exon	69277199	69277364	.	-	.	gene_id "XLOC_019672"; transcript_id "Novel_000332"; exon_number "2"; gene_type "
At_chr10	Cufflinks	exon	70313290	70313564	.	-	.	gene_id "XLOC_019749"; transcript_id "Novel_000333"; exon_number "1"; gene_type "
At_chr10	Cufflinks	exon	76992539	76992972	.	-	.	gene_id "XLOC_020205"; transcript_id "Novel_000334"; exon_number "1"; gene_type "

At_chr10	Cufflinks	exon	76993056	76993188	.	-	.	gene_id "XLOC_020205"; transcript_id "Novel_000334"; exon_number "2"; gene_type "
At_chr10	Cufflinks	exon	76993264	76993307	.	-	.	gene_id "XLOC_020205"; transcript_id "Novel_000334"; exon_number "3"; gene_type "
At_chr10	Cufflinks	exon	77039688	77040022	.	-	.	gene_id "XLOC_020212"; transcript_id "Novel_000335"; exon_number "1"; gene_type "
At_chr10	Cufflinks	exon	77040094	77040255	.	-	.	gene_id "XLOC_020212"; transcript_id "Novel_000335"; exon_number "2"; gene_type "
At_chr10	Cufflinks	exon	77040979	77041385	.	-	.	gene_id "XLOC_020212"; transcript_id "Novel_000335"; exon_number "3"; gene_type "
At_chr10	Cufflinks	exon	78569472	78570054	.	-	.	gene_id "XLOC_020276"; transcript_id "Novel_000336"; exon_number "1"; gene_type "
At_chr10	Cufflinks	exon	78570205	78570343	.	-	.	gene_id "XLOC_020276"; transcript_id "Novel_000336"; exon_number "2"; gene_type "
At_chr10	Cufflinks	exon	78570438	78570651	.	-	.	gene_id "XLOC_020276"; transcript_id "Novel_000336"; exon_number "3"; gene_type "
At_chr10	Cufflinks	exon	90261275	90261474	.	-	.	gene_id "XLOC_020725"; transcript_id "Novel_000337"; exon_number "1"; gene_type "
At_chr11	Cufflinks	exon	1828000	1828085	.	+	.	gene_id "XLOC_021188"; transcript_id "Novel_000338"; exon_number "1"; gene_type "
At_chr11	Cufflinks	exon	1828285	1828503	.	+	.	gene_id "XLOC_021188"; transcript_id "Novel_000338"; exon_number "2"; gene_type "
At_chr11	Cufflinks	exon	1924990	1925095	.	+	.	gene_id "XLOC_021196"; transcript_id "Novel_000339"; exon_number "1"; gene_type "
At_chr11	Cufflinks	exon	1925192	1926488	.	+	.	gene_id "XLOC_021196"; transcript_id "Novel_000339"; exon_number "2"; gene_type "
At_chr11	Cufflinks	exon	3002502	3003013	.	+	.	gene_id "XLOC_021305"; transcript_id "Novel_000340"; exon_number "1"; gene_type "
At_chr11	Cufflinks	exon	3003106	3003192	.	+	.	gene_id "XLOC_021305"; transcript_id "Novel_000340"; exon_number "2"; gene_type "
At_chr11	Cufflinks	exon	3003287	3003389	.	+	.	gene_id "XLOC_021305"; transcript_id "Novel_000340"; exon_number "3"; gene_type "
At_chr11	Cufflinks	exon	3003959	3004025	.	+	.	gene_id "XLOC_021305"; transcript_id "Novel_000340"; exon_number "4"; gene_type "
At_chr11	Cufflinks	exon	3004113	3004354	.	+	.	gene_id "XLOC_021305"; transcript_id "Novel_000340"; exon_number "5"; gene_type "
At_chr11	Cufflinks	exon	3003607	3004025	.	+	.	gene_id "XLOC_021305"; transcript_id "Novel_000341"; exon_number "1"; gene_type "
At_chr11	Cufflinks	exon	3004113	3004332	.	+	.	gene_id "XLOC_021305"; transcript_id "Novel_000341"; exon_number "2"; gene_type "
At_chr11	Cufflinks	exon	3073011	3073136	.	+	.	gene_id "XLOC_021309"; transcript_id "Novel_000342"; exon_number "1"; gene_type "
At_chr11	Cufflinks	exon	3073230	3073357	.	+	.	gene_id "XLOC_021309"; transcript_id "Novel_000342"; exon_number "2"; gene_type "
At_chr11	Cufflinks	exon	3177498	3179174	.	+	.	gene_id "XLOC_021330"; transcript_id "Novel_000343"; exon_number "1"; gene_type "
At_chr11	Cufflinks	exon	3179324	3179363	.	+	.	gene_id "XLOC_021330"; transcript_id "Novel_000343"; exon_number "2"; gene_type "
At_chr11	Cufflinks	exon	3534818	3535207	.	+	.	gene_id "XLOC_021372"; transcript_id "Novel_000344"; exon_number "1"; gene_type "
At_chr11	Cufflinks	exon	3535480	3535996	.	+	.	gene_id "XLOC_021372"; transcript_id "Novel_000344"; exon_number "2"; gene_type "
At_chr11	Cufflinks	exon	6860251	6860383	.	+	.	gene_id "XLOC_021577"; transcript_id "Novel_000345"; exon_number "1"; gene_type "
At_chr11	Cufflinks	exon	6860512	6860575	.	+	.	gene_id "XLOC_021577"; transcript_id "Novel_000345"; exon_number "2"; gene_type "
At_chr11	Cufflinks	exon	6860687	6860909	.	+	.	gene_id "XLOC_021577"; transcript_id "Novel_000345"; exon_number "3"; gene_type "
At_chr11	Cufflinks	exon	7818983	7819073	.	+	.	gene_id "XLOC_021731"; transcript_id "Novel_000346"; exon_number "1"; gene_type "
At_chr11	Cufflinks	exon	7819174	7819291	.	+	.	gene_id "XLOC_021731"; transcript_id "Novel_000346"; exon_number "2"; gene_type "
At_chr11	Cufflinks	exon	7820767	7821279	.	+	.	gene_id "XLOC_021731"; transcript_id "Novel_000346"; exon_number "3"; gene_type "
At_chr11	Cufflinks	exon	10943903	10944090	.	+	.	gene_id "XLOC_021964"; transcript_id "Novel_000347"; exon_number "1"; gene_type "
At_chr11	Cufflinks	exon	10960852	10960876	.	+	.	gene_id "XLOC_021964"; transcript_id "Novel_000347"; exon_number "2"; gene_type "
At_chr11	Cufflinks	exon	10943905	10944142	.	+	.	gene_id "XLOC_021964"; transcript_id "Novel_000348"; exon_number "1"; gene_type "

At_chr11	Cufflinks	exon	10944229	10944810	.	+	.	gene_id "XLOC_021964"; transcript_id "Novel_000348"; exon_number "2"; gene_type "
At_chr11	Cufflinks	exon	10995175	10995644	.	+	.	gene_id "XLOC_021968"; transcript_id "Novel_000349"; exon_number "1"; gene_type "
At_chr11	Cufflinks	exon	10995729	10996274	.	+	.	gene_id "XLOC_021968"; transcript_id "Novel_000349"; exon_number "2"; gene_type "
At_chr11	Cufflinks	exon	12584180	12585355	.	+	.	gene_id "XLOC_022083"; transcript_id "Novel_000350"; exon_number "1"; gene_type "
At_chr11	Cufflinks	exon	12585526	12588595	.	+	.	gene_id "XLOC_022083"; transcript_id "Novel_000350"; exon_number "2"; gene_type "
At_chr11	Cufflinks	exon	12887008	12887212	.	+	.	gene_id "XLOC_022098"; transcript_id "Novel_000351"; exon_number "1"; gene_type "
At_chr11	Cufflinks	exon	12888359	12889297	.	+	.	gene_id "XLOC_022098"; transcript_id "Novel_000351"; exon_number "2"; gene_type "
At_chr11	Cufflinks	exon	12889413	12889470	.	+	.	gene_id "XLOC_022098"; transcript_id "Novel_000351"; exon_number "3"; gene_type "
At_chr11	Cufflinks	exon	12889600	12889715	.	+	.	gene_id "XLOC_022098"; transcript_id "Novel_000351"; exon_number "4"; gene_type "
At_chr11	Cufflinks	exon	12890844	12890980	.	+	.	gene_id "XLOC_022098"; transcript_id "Novel_000351"; exon_number "5"; gene_type "
At_chr11	Cufflinks	exon	12891107	12893136	.	+	.	gene_id "XLOC_022098"; transcript_id "Novel_000351"; exon_number "6"; gene_type "
At_chr11	Cufflinks	exon	15006480	15006830	.	+	.	gene_id "XLOC_022222"; transcript_id "Novel_000352"; exon_number "1"; gene_type "
At_chr11	Cufflinks	exon	15006939	15007557	.	+	.	gene_id "XLOC_022222"; transcript_id "Novel_000352"; exon_number "2"; gene_type "
At_chr11	Cufflinks	exon	15109300	15109638	.	+	.	gene_id "XLOC_022231"; transcript_id "Novel_000353"; exon_number "1"; gene_type "
At_chr11	Cufflinks	exon	15109993	15110169	.	+	.	gene_id "XLOC_022231"; transcript_id "Novel_000353"; exon_number "2"; gene_type "
At_chr11	Cufflinks	exon	20957914	20958082	.	+	.	gene_id "XLOC_022512"; transcript_id "Novel_000354"; exon_number "1"; gene_type "
At_chr11	Cufflinks	exon	20958522	20958624	.	+	.	gene_id "XLOC_022512"; transcript_id "Novel_000354"; exon_number "2"; gene_type "
At_chr11	Cufflinks	exon	20959101	20959973	.	+	.	gene_id "XLOC_022512"; transcript_id "Novel_000354"; exon_number "3"; gene_type "
At_chr11	Cufflinks	exon	20960289	20960389	.	+	.	gene_id "XLOC_022512"; transcript_id "Novel_000354"; exon_number "4"; gene_type "
At_chr11	Cufflinks	exon	20960486	20960600	.	+	.	gene_id "XLOC_022512"; transcript_id "Novel_000354"; exon_number "5"; gene_type "
At_chr11	Cufflinks	exon	20960821	20960898	.	+	.	gene_id "XLOC_022512"; transcript_id "Novel_000354"; exon_number "6"; gene_type "
At_chr11	Cufflinks	exon	20961059	20961151	.	+	.	gene_id "XLOC_022512"; transcript_id "Novel_000354"; exon_number "7"; gene_type "
At_chr11	Cufflinks	exon	20961360	20961396	.	+	.	gene_id "XLOC_022512"; transcript_id "Novel_000354"; exon_number "8"; gene_type "
At_chr11	Cufflinks	exon	20958008	20958082	.	+	.	gene_id "XLOC_022512"; transcript_id "Novel_000355"; exon_number "1"; gene_type "
At_chr11	Cufflinks	exon	20958519	20958624	.	+	.	gene_id "XLOC_022512"; transcript_id "Novel_000355"; exon_number "2"; gene_type "
At_chr11	Cufflinks	exon	20959101	20959973	.	+	.	gene_id "XLOC_022512"; transcript_id "Novel_000355"; exon_number "3"; gene_type "
At_chr11	Cufflinks	exon	20960289	20960389	.	+	.	gene_id "XLOC_022512"; transcript_id "Novel_000355"; exon_number "4"; gene_type "
At_chr11	Cufflinks	exon	20960486	20960600	.	+	.	gene_id "XLOC_022512"; transcript_id "Novel_000355"; exon_number "5"; gene_type "
At_chr11	Cufflinks	exon	20960821	20960898	.	+	.	gene_id "XLOC_022512"; transcript_id "Novel_000355"; exon_number "6"; gene_type "
At_chr11	Cufflinks	exon	20961059	20961151	.	+	.	gene_id "XLOC_022512"; transcript_id "Novel_000355"; exon_number "7"; gene_type "
At_chr11	Cufflinks	exon	20961355	20961684	.	+	.	gene_id "XLOC_022512"; transcript_id "Novel_000355"; exon_number "8"; gene_type "
At_chr11	Cufflinks	exon	20961533	20962117	.	+	.	gene_id "XLOC_022512"; transcript_id "Novel_000356"; exon_number "1"; gene_type "
At_chr11	Cufflinks	exon	20962671	20962733	.	+	.	gene_id "XLOC_022512"; transcript_id "Novel_000356"; exon_number "2"; gene_type "
At_chr11	Cufflinks	exon	20962951	20962981	.	+	.	gene_id "XLOC_022512"; transcript_id "Novel_000356"; exon_number "3"; gene_type "
At_chr11	Cufflinks	exon	20963205	20963251	.	+	.	gene_id "XLOC_022512"; transcript_id "Novel_000356"; exon_number "4"; gene_type "

At_chr11	Cufflinks	exon	20963858	20963916	.	+	.	gene_id "XLOC_022512"; transcript_id "Novel_000356"; exon_number "5"; gene_type "
At_chr11	Cufflinks	exon	20964035	20964625	.	+	.	gene_id "XLOC_022512"; transcript_id "Novel_000356"; exon_number "6"; gene_type "
At_chr11	Cufflinks	exon	21077238	21077357	.	+	.	gene_id "XLOC_022526"; transcript_id "Novel_000357"; exon_number "1"; gene_type "
At_chr11	Cufflinks	exon	21078320	21078643	.	+	.	gene_id "XLOC_022526"; transcript_id "Novel_000357"; exon_number "2"; gene_type "
At_chr11	Cufflinks	exon	21738116	21738174	.	+	.	gene_id "XLOC_022573"; transcript_id "Novel_000358"; exon_number "1"; gene_type "
At_chr11	Cufflinks	exon	21738753	21738830	.	+	.	gene_id "XLOC_022573"; transcript_id "Novel_000358"; exon_number "2"; gene_type "
At_chr11	Cufflinks	exon	21738922	21739065	.	+	.	gene_id "XLOC_022573"; transcript_id "Novel_000358"; exon_number "3"; gene_type "
At_chr11	Cufflinks	exon	21739578	21739634	.	+	.	gene_id "XLOC_022573"; transcript_id "Novel_000358"; exon_number "4"; gene_type "
At_chr11	Cufflinks	exon	21739800	21740669	.	+	.	gene_id "XLOC_022573"; transcript_id "Novel_000358"; exon_number "5"; gene_type "
At_chr11	Cufflinks	exon	21738116	21738174	.	+	.	gene_id "XLOC_022573"; transcript_id "Novel_000359"; exon_number "1"; gene_type "
At_chr11	Cufflinks	exon	21738753	21738830	.	+	.	gene_id "XLOC_022573"; transcript_id "Novel_000359"; exon_number "2"; gene_type "
At_chr11	Cufflinks	exon	21738922	21739102	.	+	.	gene_id "XLOC_022573"; transcript_id "Novel_000359"; exon_number "3"; gene_type "
At_chr11	Cufflinks	exon	21739578	21740669	.	+	.	gene_id "XLOC_022573"; transcript_id "Novel_000359"; exon_number "4"; gene_type "
At_chr11	Cufflinks	exon	21738120	21738174	.	+	.	gene_id "XLOC_022573"; transcript_id "Novel_000360"; exon_number "1"; gene_type "
At_chr11	Cufflinks	exon	21738753	21738830	.	+	.	gene_id "XLOC_022573"; transcript_id "Novel_000360"; exon_number "2"; gene_type "
At_chr11	Cufflinks	exon	21738922	21739102	.	+	.	gene_id "XLOC_022573"; transcript_id "Novel_000360"; exon_number "3"; gene_type "
At_chr11	Cufflinks	exon	21739578	21739675	.	+	.	gene_id "XLOC_022573"; transcript_id "Novel_000360"; exon_number "4"; gene_type "
At_chr11	Cufflinks	exon	21739800	21740898	.	+	.	gene_id "XLOC_022573"; transcript_id "Novel_000360"; exon_number "5"; gene_type "
At_chr11	Cufflinks	exon	21996441	21996889	.	+	.	gene_id "XLOC_022589"; transcript_id "Novel_000361"; exon_number "1"; gene_type "
At_chr11	Cufflinks	exon	21996984	21997586	.	+	.	gene_id "XLOC_022589"; transcript_id "Novel_000361"; exon_number "2"; gene_type "
At_chr11	Cufflinks	exon	22165503	22165781	.	+	.	gene_id "XLOC_022597"; transcript_id "Novel_000362"; exon_number "1"; gene_type "
At_chr11	Cufflinks	exon	22165871	22167376	.	+	.	gene_id "XLOC_022597"; transcript_id "Novel_000362"; exon_number "2"; gene_type "
At_chr11	Cufflinks	exon	22165513	22165781	.	+	.	gene_id "XLOC_022597"; transcript_id "Novel_000363"; exon_number "1"; gene_type "
At_chr11	Cufflinks	exon	22165871	22165989	.	+	.	gene_id "XLOC_022597"; transcript_id "Novel_000363"; exon_number "2"; gene_type "
At_chr11	Cufflinks	exon	22167055	22167117	.	+	.	gene_id "XLOC_022597"; transcript_id "Novel_000363"; exon_number "3"; gene_type "
At_chr11	Cufflinks	exon	22167305	22167350	.	+	.	gene_id "XLOC_022597"; transcript_id "Novel_000363"; exon_number "4"; gene_type "
At_chr11	Cufflinks	exon	22168341	22168559	.	+	.	gene_id "XLOC_022597"; transcript_id "Novel_000363"; exon_number "5"; gene_type "
At_chr11	Cufflinks	exon	22169945	22170702	.	+	.	gene_id "XLOC_022597"; transcript_id "Novel_000363"; exon_number "6"; gene_type "
At_chr11	Cufflinks	exon	22165524	22165781	.	+	.	gene_id "XLOC_022597"; transcript_id "Novel_000364"; exon_number "1"; gene_type "
At_chr11	Cufflinks	exon	22165871	22165989	.	+	.	gene_id "XLOC_022597"; transcript_id "Novel_000364"; exon_number "2"; gene_type "
At_chr11	Cufflinks	exon	22167055	22167117	.	+	.	gene_id "XLOC_022597"; transcript_id "Novel_000364"; exon_number "3"; gene_type "
At_chr11	Cufflinks	exon	22167305	22167350	.	+	.	gene_id "XLOC_022597"; transcript_id "Novel_000364"; exon_number "4"; gene_type "
At_chr11	Cufflinks	exon	22168341	22168559	.	+	.	gene_id "XLOC_022597"; transcript_id "Novel_000364"; exon_number "5"; gene_type "
At_chr11	Cufflinks	exon	22169945	22170060	.	+	.	gene_id "XLOC_022597"; transcript_id "Novel_000364"; exon_number "6"; gene_type "
At_chr11	Cufflinks	exon	22170792	22170960	.	+	.	gene_id "XLOC_022597"; transcript_id "Novel_000364"; exon_number "7"; gene_type "

At_chr11	Cufflinks	exon	22171216	22171291	.	+	.	gene_id "XLOC_022597"; transcript_id "Novel_000364"; exon_number "8"; gene_type "
At_chr11	Cufflinks	exon	22165527	22165781	.	+	.	gene_id "XLOC_022597"; transcript_id "Novel_000365"; exon_number "1"; gene_type "
At_chr11	Cufflinks	exon	22165871	22165989	.	+	.	gene_id "XLOC_022597"; transcript_id "Novel_000365"; exon_number "2"; gene_type "
At_chr11	Cufflinks	exon	22167055	22168559	.	+	.	gene_id "XLOC_022597"; transcript_id "Novel_000365"; exon_number "3"; gene_type "
At_chr11	Cufflinks	exon	22169945	22170060	.	+	.	gene_id "XLOC_022597"; transcript_id "Novel_000365"; exon_number "4"; gene_type "
At_chr11	Cufflinks	exon	22170792	22170960	.	+	.	gene_id "XLOC_022597"; transcript_id "Novel_000365"; exon_number "5"; gene_type "
At_chr11	Cufflinks	exon	22171216	22171313	.	+	.	gene_id "XLOC_022597"; transcript_id "Novel_000365"; exon_number "6"; gene_type "
At_chr11	Cufflinks	exon	22171453	22173994	.	+	.	gene_id "XLOC_022601"; transcript_id "Novel_000366"; exon_number "1"; gene_type "
At_chr11	Cufflinks	exon	22174803	22175001	.	+	.	gene_id "XLOC_022601"; transcript_id "Novel_000366"; exon_number "2"; gene_type "
At_chr11	Cufflinks	exon	22178081	22178159	.	+	.	gene_id "XLOC_022601"; transcript_id "Novel_000367"; exon_number "1"; gene_type "
At_chr11	Cufflinks	exon	22179145	22179330	.	+	.	gene_id "XLOC_022601"; transcript_id "Novel_000367"; exon_number "2"; gene_type "
At_chr11	Cufflinks	exon	22179551	22180315	.	+	.	gene_id "XLOC_022602"; transcript_id "Novel_000368"; exon_number "1"; gene_type "
At_chr11	Cufflinks	exon	22180457	22180573	.	+	.	gene_id "XLOC_022602"; transcript_id "Novel_000368"; exon_number "2"; gene_type "
At_chr11	Cufflinks	exon	22180816	22180872	.	+	.	gene_id "XLOC_022602"; transcript_id "Novel_000368"; exon_number "3"; gene_type "
At_chr11	Cufflinks	exon	22180957	22181747	.	+	.	gene_id "XLOC_022602"; transcript_id "Novel_000368"; exon_number "4"; gene_type "
At_chr11	Cufflinks	exon	22218446	22218973	.	+	.	gene_id "XLOC_022609"; transcript_id "Novel_000369"; exon_number "1"; gene_type "
At_chr11	Cufflinks	exon	22219046	22219595	.	+	.	gene_id "XLOC_022609"; transcript_id "Novel_000369"; exon_number "2"; gene_type "
At_chr11	Cufflinks	exon	22219713	22220296	.	+	.	gene_id "XLOC_022609"; transcript_id "Novel_000369"; exon_number "3"; gene_type "
At_chr11	Cufflinks	exon	22220384	22220533	.	+	.	gene_id "XLOC_022609"; transcript_id "Novel_000369"; exon_number "4"; gene_type "
At_chr11	Cufflinks	exon	22220650	22222152	.	+	.	gene_id "XLOC_022609"; transcript_id "Novel_000369"; exon_number "5"; gene_type "
At_chr11	Cufflinks	exon	22218616	22219447	.	+	.	gene_id "XLOC_022609"; transcript_id "Novel_000370"; exon_number "1"; gene_type "
At_chr11	Cufflinks	exon	22219530	22219595	.	+	.	gene_id "XLOC_022609"; transcript_id "Novel_000370"; exon_number "2"; gene_type "
At_chr11	Cufflinks	exon	22219713	22219846	.	+	.	gene_id "XLOC_022609"; transcript_id "Novel_000370"; exon_number "3"; gene_type "
At_chr11	Cufflinks	exon	22219441	22219595	.	+	.	gene_id "XLOC_022609"; transcript_id "Novel_000371"; exon_number "1"; gene_type "
At_chr11	Cufflinks	exon	22219713	22219868	.	+	.	gene_id "XLOC_022609"; transcript_id "Novel_000371"; exon_number "2"; gene_type "
At_chr11	Cufflinks	exon	22219993	22220067	.	+	.	gene_id "XLOC_022609"; transcript_id "Novel_000371"; exon_number "3"; gene_type "
At_chr11	Cufflinks	exon	22220141	22220296	.	+	.	gene_id "XLOC_022609"; transcript_id "Novel_000371"; exon_number "4"; gene_type "
At_chr11	Cufflinks	exon	22220384	22220533	.	+	.	gene_id "XLOC_022609"; transcript_id "Novel_000371"; exon_number "5"; gene_type "
At_chr11	Cufflinks	exon	22220650	22222211	.	+	.	gene_id "XLOC_022609"; transcript_id "Novel_000371"; exon_number "6"; gene_type "
At_chr11	Cufflinks	exon	23174421	23174658	.	+	.	gene_id "XLOC_022639"; transcript_id "Novel_000372"; exon_number "1"; gene_type "
At_chr11	Cufflinks	exon	23175329	23175524	.	+	.	gene_id "XLOC_022639"; transcript_id "Novel_000372"; exon_number "2"; gene_type "
At_chr11	Cufflinks	exon	23176144	23176252	.	+	.	gene_id "XLOC_022639"; transcript_id "Novel_000372"; exon_number "3"; gene_type "
At_chr11	Cufflinks	exon	23178284	23178654	.	+	.	gene_id "XLOC_022639"; transcript_id "Novel_000372"; exon_number "4"; gene_type "
At_chr11	Cufflinks	exon	24011137	24011291	.	+	.	gene_id "XLOC_022673"; transcript_id "Novel_000373"; exon_number "1"; gene_type "
At_chr11	Cufflinks	exon	24012379	24013049	.	+	.	gene_id "XLOC_022673"; transcript_id "Novel_000373"; exon_number "2"; gene_type "

At_chr11	Cufflinks	exon	24013198	24013455	.	+	.	gene_id "XLOC_022673"; transcript_id "Novel_000373"; exon_number "3"; gene_type "
At_chr11	Cufflinks	exon	24013743	24013865	.	+	.	gene_id "XLOC_022673"; transcript_id "Novel_000373"; exon_number "4"; gene_type "
At_chr11	Cufflinks	exon	24014029	24015037	.	+	.	gene_id "XLOC_022673"; transcript_id "Novel_000373"; exon_number "5"; gene_type "
At_chr11	Cufflinks	exon	27835539	27836371	.	+	.	gene_id "XLOC_022885"; transcript_id "Novel_000374"; exon_number "1"; gene_type "
At_chr11	Cufflinks	exon	27840112	27840186	.	+	.	gene_id "XLOC_022885"; transcript_id "Novel_000374"; exon_number "2"; gene_type "
At_chr11	Cufflinks	exon	27840291	27840395	.	+	.	gene_id "XLOC_022885"; transcript_id "Novel_000374"; exon_number "3"; gene_type "
At_chr11	Cufflinks	exon	27841015	27841100	.	+	.	gene_id "XLOC_022885"; transcript_id "Novel_000374"; exon_number "4"; gene_type "
At_chr11	Cufflinks	exon	27841970	27842090	.	+	.	gene_id "XLOC_022885"; transcript_id "Novel_000374"; exon_number "5"; gene_type "
At_chr11	Cufflinks	exon	27842782	27842861	.	+	.	gene_id "XLOC_022885"; transcript_id "Novel_000374"; exon_number "6"; gene_type "
At_chr11	Cufflinks	exon	27843093	27843155	.	+	.	gene_id "XLOC_022885"; transcript_id "Novel_000374"; exon_number "7"; gene_type "
At_chr11	Cufflinks	exon	27843273	27843394	.	+	.	gene_id "XLOC_022885"; transcript_id "Novel_000374"; exon_number "8"; gene_type "
At_chr11	Cufflinks	exon	27843506	27843591	.	+	.	gene_id "XLOC_022885"; transcript_id "Novel_000374"; exon_number "9"; gene_type "
At_chr11	Cufflinks	exon	27843916	27844040	.	+	.	gene_id "XLOC_022885"; transcript_id "Novel_000374"; exon_number "10"; gene_type
At_chr11	Cufflinks	exon	27844286	27844417	.	+	.	gene_id "XLOC_022885"; transcript_id "Novel_000374"; exon_number "11"; gene_type
At_chr11	Cufflinks	exon	27845178	27845437	.	+	.	gene_id "XLOC_022885"; transcript_id "Novel_000374"; exon_number "12"; gene_type
At_chr11	Cufflinks	exon	27835558	27836371	.	+	.	gene_id "XLOC_022885"; transcript_id "Novel_000375"; exon_number "1"; gene_type "
At_chr11	Cufflinks	exon	27840112	27840186	.	+	.	gene_id "XLOC_022885"; transcript_id "Novel_000375"; exon_number "2"; gene_type "
At_chr11	Cufflinks	exon	27840291	27840395	.	+	.	gene_id "XLOC_022885"; transcript_id "Novel_000375"; exon_number "3"; gene_type "
At_chr11	Cufflinks	exon	27841015	27841100	.	+	.	gene_id "XLOC_022885"; transcript_id "Novel_000375"; exon_number "4"; gene_type "
At_chr11	Cufflinks	exon	27841970	27842090	.	+	.	gene_id "XLOC_022885"; transcript_id "Novel_000375"; exon_number "5"; gene_type "
At_chr11	Cufflinks	exon	27842782	27842861	.	+	.	gene_id "XLOC_022885"; transcript_id "Novel_000375"; exon_number "6"; gene_type "
At_chr11	Cufflinks	exon	27843093	27843155	.	+	.	gene_id "XLOC_022885"; transcript_id "Novel_000375"; exon_number "7"; gene_type "
At_chr11	Cufflinks	exon	27843266	27843394	.	+	.	gene_id "XLOC_022885"; transcript_id "Novel_000375"; exon_number "8"; gene_type "
At_chr11	Cufflinks	exon	27843506	27843591	.	+	.	gene_id "XLOC_022885"; transcript_id "Novel_000375"; exon_number "9"; gene_type "
At_chr11	Cufflinks	exon	27843916	27844040	.	+	.	gene_id "XLOC_022885"; transcript_id "Novel_000375"; exon_number "10"; gene_type
At_chr11	Cufflinks	exon	27844286	27844417	.	+	.	gene_id "XLOC_022885"; transcript_id "Novel_000375"; exon_number "11"; gene_type
At_chr11	Cufflinks	exon	27845178	27845602	.	+	.	gene_id "XLOC_022885"; transcript_id "Novel_000375"; exon_number "12"; gene_type
At_chr11	Cufflinks	exon	27835558	27836371	.	+	.	gene_id "XLOC_022885"; transcript_id "Novel_000376"; exon_number "1"; gene_type "
At_chr11	Cufflinks	exon	27840112	27840395	.	+	.	gene_id "XLOC_022885"; transcript_id "Novel_000376"; exon_number "2"; gene_type "
At_chr11	Cufflinks	exon	27841015	27841100	.	+	.	gene_id "XLOC_022885"; transcript_id "Novel_000376"; exon_number "3"; gene_type "
At_chr11	Cufflinks	exon	27841970	27842090	.	+	.	gene_id "XLOC_022885"; transcript_id "Novel_000376"; exon_number "4"; gene_type "
At_chr11	Cufflinks	exon	27842782	27842861	.	+	.	gene_id "XLOC_022885"; transcript_id "Novel_000376"; exon_number "5"; gene_type "
At_chr11	Cufflinks	exon	27843093	27843155	.	+	.	gene_id "XLOC_022885"; transcript_id "Novel_000376"; exon_number "6"; gene_type "
At_chr11	Cufflinks	exon	27843273	27843394	.	+	.	gene_id "XLOC_022885"; transcript_id "Novel_000376"; exon_number "7"; gene_type "
At_chr11	Cufflinks	exon	27843506	27843591	.	+	.	gene_id "XLOC_022885"; transcript_id "Novel_000376"; exon_number "8"; gene_type "

At_chr11	Cufflinks	exon	27843916	27844040	.	+	.	gene_id "XLOC_022885"; transcript_id "Novel_000376"; exon_number "9"; gene_type "
At_chr11	Cufflinks	exon	27844286	27844417	.	+	.	gene_id "XLOC_022885"; transcript_id "Novel_000376"; exon_number "10"; gene_type
At_chr11	Cufflinks	exon	27845178	27845602	.	+	.	gene_id "XLOC_022885"; transcript_id "Novel_000376"; exon_number "11"; gene_type
At_chr11	Cufflinks	exon	27842911	27843155	.	+	.	gene_id "XLOC_022885"; transcript_id "Novel_000377"; exon_number "1"; gene_type "
At_chr11	Cufflinks	exon	27843266	27843394	.	+	.	gene_id "XLOC_022885"; transcript_id "Novel_000377"; exon_number "2"; gene_type "
At_chr11	Cufflinks	exon	27843506	27843591	.	+	.	gene_id "XLOC_022885"; transcript_id "Novel_000377"; exon_number "3"; gene_type "
At_chr11	Cufflinks	exon	27843916	27844040	.	+	.	gene_id "XLOC_022885"; transcript_id "Novel_000377"; exon_number "4"; gene_type "
At_chr11	Cufflinks	exon	27844286	27844417	.	+	.	gene_id "XLOC_022885"; transcript_id "Novel_000377"; exon_number "5"; gene_type "
At_chr11	Cufflinks	exon	27845178	27845684	.	+	.	gene_id "XLOC_022885"; transcript_id "Novel_000377"; exon_number "6"; gene_type "
At_chr11	Cufflinks	exon	29660526	29660851	.	+	.	gene_id "XLOC_022979"; transcript_id "Novel_000378"; exon_number "1"; gene_type "
At_chr11	Cufflinks	exon	29661245	29661581	.	+	.	gene_id "XLOC_022979"; transcript_id "Novel_000378"; exon_number "2"; gene_type "
At_chr11	Cufflinks	exon	31199488	31199767	.	+	.	gene_id "XLOC_023066"; transcript_id "Novel_000379"; exon_number "1"; gene_type "
At_chr11	Cufflinks	exon	31200288	31200747	.	+	.	gene_id "XLOC_023066"; transcript_id "Novel_000379"; exon_number "2"; gene_type "
At_chr11	Cufflinks	exon	31200840	31203070	.	+	.	gene_id "XLOC_023066"; transcript_id "Novel_000379"; exon_number "3"; gene_type "
At_chr11	Cufflinks	exon	31199488	31199767	.	+	.	gene_id "XLOC_023066"; transcript_id "Novel_000380"; exon_number "1"; gene_type "
At_chr11	Cufflinks	exon	31200288	31203070	.	+	.	gene_id "XLOC_023066"; transcript_id "Novel_000380"; exon_number "2"; gene_type "
At_chr11	Cufflinks	exon	40143475	40146159	.	+	.	gene_id "XLOC_023521"; transcript_id "Novel_000381"; exon_number "1"; gene_type "
At_chr11	Cufflinks	exon	40161940	40162129	.	+	.	gene_id "XLOC_023522"; transcript_id "Novel_000382"; exon_number "1"; gene_type "
At_chr11	Cufflinks	exon	40162242	40162361	.	+	.	gene_id "XLOC_023522"; transcript_id "Novel_000382"; exon_number "2"; gene_type "
At_chr11	Cufflinks	exon	46267507	46268055	.	+	.	gene_id "XLOC_023686"; transcript_id "Novel_000383"; exon_number "1"; gene_type "
At_chr11	Cufflinks	exon	46268138	46268215	.	+	.	gene_id "XLOC_023686"; transcript_id "Novel_000383"; exon_number "2"; gene_type "
At_chr11	Cufflinks	exon	46268594	46268701	.	+	.	gene_id "XLOC_023686"; transcript_id "Novel_000383"; exon_number "3"; gene_type "
At_chr11	Cufflinks	exon	46268942	46269004	.	+	.	gene_id "XLOC_023686"; transcript_id "Novel_000383"; exon_number "4"; gene_type "
At_chr11	Cufflinks	exon	46269114	46269275	.	+	.	gene_id "XLOC_023686"; transcript_id "Novel_000383"; exon_number "5"; gene_type "
At_chr11	Cufflinks	exon	46269921	46270022	.	+	.	gene_id "XLOC_023686"; transcript_id "Novel_000383"; exon_number "6"; gene_type "
At_chr11	Cufflinks	exon	46270128	46270170	.	+	.	gene_id "XLOC_023686"; transcript_id "Novel_000383"; exon_number "7"; gene_type "
At_chr11	Cufflinks	exon	46270266	46270583	.	+	.	gene_id "XLOC_023686"; transcript_id "Novel_000383"; exon_number "8"; gene_type "
At_chr11	Cufflinks	exon	46270679	46270843	.	+	.	gene_id "XLOC_023686"; transcript_id "Novel_000383"; exon_number "9"; gene_type "
At_chr11	Cufflinks	exon	46271339	46273239	.	+	.	gene_id "XLOC_023686"; transcript_id "Novel_000383"; exon_number "10"; gene_type
At_chr11	Cufflinks	exon	51187931	51188224	.	+	.	gene_id "XLOC_023861"; transcript_id "Novel_000384"; exon_number "1"; gene_type "
At_chr11	Cufflinks	exon	57122164	57122491	.	+	.	gene_id "XLOC_024226"; transcript_id "Novel_000385"; exon_number "1"; gene_type "
At_chr11	Cufflinks	exon	57122666	57122762	.	+	.	gene_id "XLOC_024226"; transcript_id "Novel_000385"; exon_number "2"; gene_type "
At_chr11	Cufflinks	exon	57123367	57125288	.	+	.	gene_id "XLOC_024226"; transcript_id "Novel_000385"; exon_number "3"; gene_type "
At_chr11	Cufflinks	exon	57122286	57122491	.	+	.	gene_id "XLOC_024226"; transcript_id "Novel_000386"; exon_number "1"; gene_type "
At_chr11	Cufflinks	exon	57122666	57125305	.	+	.	gene_id "XLOC_024226"; transcript_id "Novel_000386"; exon_number "2"; gene_type "

At_chr11	Cufflinks	exon	58053660	58053772	.	+	.	gene_id "XLOC_024320"; transcript_id "Novel_000387"; exon_number "1"; gene_type "
At_chr11	Cufflinks	exon	58053856	58054495	.	+	.	gene_id "XLOC_024320"; transcript_id "Novel_000387"; exon_number "2"; gene_type "
At_chr11	Cufflinks	exon	60569214	60569361	.	+	.	gene_id "XLOC_024424"; transcript_id "Novel_000388"; exon_number "1"; gene_type "
At_chr11	Cufflinks	exon	60569641	60570070	.	+	.	gene_id "XLOC_024424"; transcript_id "Novel_000388"; exon_number "2"; gene_type "
At_chr11	Cufflinks	exon	63301188	63302091	.	+	.	gene_id "XLOC_024657"; transcript_id "Novel_000389"; exon_number "1"; gene_type "
At_chr11	Cufflinks	exon	71626823	71627216	.	+	.	gene_id "XLOC_025229"; transcript_id "Novel_000390"; exon_number "1"; gene_type "
At_chr11	Cufflinks	exon	71627346	71627525	.	+	.	gene_id "XLOC_025229"; transcript_id "Novel_000390"; exon_number "2"; gene_type "
At_chr11	Cufflinks	exon	71627915	71628092	.	+	.	gene_id "XLOC_025229"; transcript_id "Novel_000390"; exon_number "3"; gene_type "
At_chr11	Cufflinks	exon	86443930	86444305	.	+	.	gene_id "XLOC_025661"; transcript_id "Novel_000391"; exon_number "1"; gene_type "
At_chr11	Cufflinks	exon	86445193	86445477	.	+	.	gene_id "XLOC_025661"; transcript_id "Novel_000391"; exon_number "2"; gene_type "
At_chr11	Cufflinks	exon	86445632	86445708	.	+	.	gene_id "XLOC_025661"; transcript_id "Novel_000391"; exon_number "3"; gene_type "
At_chr11	Cufflinks	exon	86445789	86445805	.	+	.	gene_id "XLOC_025661"; transcript_id "Novel_000391"; exon_number "4"; gene_type "
At_chr11	Cufflinks	exon	86443962	86444305	.	+	.	gene_id "XLOC_025661"; transcript_id "Novel_000392"; exon_number "1"; gene_type "
At_chr11	Cufflinks	exon	86445193	86445576	.	+	.	gene_id "XLOC_025661"; transcript_id "Novel_000392"; exon_number "2"; gene_type "
At_chr11	Cufflinks	exon	91285823	91286207	.	+	.	gene_id "XLOC_025893"; transcript_id "Novel_000393"; exon_number "1"; gene_type "
At_chr11	Cufflinks	exon	91288257	91290049	.	+	.	gene_id "XLOC_025893"; transcript_id "Novel_000393"; exon_number "2"; gene_type "
At_chr11	Cufflinks	exon	94561008	94561867	.	+	.	gene_id "XLOC_026055"; transcript_id "Novel_000394"; exon_number "1"; gene_type "
At_chr11	Cufflinks	exon	94562340	94563205	.	+	.	gene_id "XLOC_026055"; transcript_id "Novel_000394"; exon_number "2"; gene_type "
At_chr11	Cufflinks	exon	94561008	94561653	.	+	.	gene_id "XLOC_026055"; transcript_id "Novel_000395"; exon_number "1"; gene_type "
At_chr11	Cufflinks	exon	94561745	94561867	.	+	.	gene_id "XLOC_026055"; transcript_id "Novel_000395"; exon_number "2"; gene_type "
At_chr11	Cufflinks	exon	94562340	94563205	.	+	.	gene_id "XLOC_026055"; transcript_id "Novel_000395"; exon_number "3"; gene_type "
At_chr11	Cufflinks	exon	95146912	95147354	.	+	.	gene_id "XLOC_026082"; transcript_id "Novel_000396"; exon_number "1"; gene_type "
At_chr11	Cufflinks	exon	95147434	95147473	.	+	.	gene_id "XLOC_026082"; transcript_id "Novel_000396"; exon_number "2"; gene_type "
At_chr11	Cufflinks	exon	95147582	95148681	.	+	.	gene_id "XLOC_026082"; transcript_id "Novel_000396"; exon_number "3"; gene_type "
At_chr11	Cufflinks	exon	95146940	95147473	.	+	.	gene_id "XLOC_026082"; transcript_id "Novel_000397"; exon_number "1"; gene_type "
At_chr11	Cufflinks	exon	95147582	95148490	.	+	.	gene_id "XLOC_026082"; transcript_id "Novel_000397"; exon_number "2"; gene_type "
At_chr11	Cufflinks	exon	96048007	96049291	.	+	.	gene_id "XLOC_026128"; transcript_id "Novel_000398"; exon_number "1"; gene_type "
At_chr11	Cufflinks	exon	96049398	96049552	.	+	.	gene_id "XLOC_026128"; transcript_id "Novel_000398"; exon_number "2"; gene_type "
At_chr11	Cufflinks	exon	96048202	96048489	.	+	.	gene_id "XLOC_026128"; transcript_id "Novel_000399"; exon_number "1"; gene_type "
At_chr11	Cufflinks	exon	96048646	96048706	.	+	.	gene_id "XLOC_026128"; transcript_id "Novel_000399"; exon_number "2"; gene_type "
At_chr11	Cufflinks	exon	96048788	96048903	.	+	.	gene_id "XLOC_026128"; transcript_id "Novel_000399"; exon_number "3"; gene_type "
At_chr11	Cufflinks	exon	96048995	96049291	.	+	.	gene_id "XLOC_026128"; transcript_id "Novel_000399"; exon_number "4"; gene_type "
At_chr11	Cufflinks	exon	96049398	96049566	.	+	.	gene_id "XLOC_026128"; transcript_id "Novel_000399"; exon_number "5"; gene_type "
At_chr11	Cufflinks	exon	99155028	99155329	.	+	.	gene_id "XLOC_026227"; transcript_id "Novel_000400"; exon_number "1"; gene_type "
At_chr11	Cufflinks	exon	99155607	99155697	.	+	.	gene_id "XLOC_026227"; transcript_id "Novel_000400"; exon_number "2"; gene_type "

At_chr11	Cufflinks	exon	99156234	99156332	.	+	.	gene_id "XLOC_026227"; transcript_id "Novel_000400"; exon_number "3"; gene_type "
At_chr11	Cufflinks	exon	99156456	99156567	.	+	.	gene_id "XLOC_026227"; transcript_id "Novel_000400"; exon_number "4"; gene_type "
At_chr11	Cufflinks	exon	99156701	99156856	.	+	.	gene_id "XLOC_026227"; transcript_id "Novel_000400"; exon_number "5"; gene_type "
At_chr11	Cufflinks	exon	99157616	99157720	.	+	.	gene_id "XLOC_026227"; transcript_id "Novel_000400"; exon_number "6"; gene_type "
At_chr11	Cufflinks	exon	99157943	99158026	.	+	.	gene_id "XLOC_026227"; transcript_id "Novel_000400"; exon_number "7"; gene_type "
At_chr11	Cufflinks	exon	99158114	99158229	.	+	.	gene_id "XLOC_026227"; transcript_id "Novel_000400"; exon_number "8"; gene_type "
At_chr11	Cufflinks	exon	99158343	99158641	.	+	.	gene_id "XLOC_026227"; transcript_id "Novel_000400"; exon_number "9"; gene_type "
At_chr11	Cufflinks	exon	99158771	99158827	.	+	.	gene_id "XLOC_026227"; transcript_id "Novel_000400"; exon_number "10"; gene_type
At_chr11	Cufflinks	exon	99158926	99158992	.	+	.	gene_id "XLOC_026227"; transcript_id "Novel_000400"; exon_number "11"; gene_type
At_chr11	Cufflinks	exon	99159076	99159129	.	+	.	gene_id "XLOC_026227"; transcript_id "Novel_000400"; exon_number "12"; gene_type
At_chr11	Cufflinks	exon	99159227	99159301	.	+	.	gene_id "XLOC_026227"; transcript_id "Novel_000400"; exon_number "13"; gene_type
At_chr11	Cufflinks	exon	99159381	99159416	.	+	.	gene_id "XLOC_026227"; transcript_id "Novel_000400"; exon_number "14"; gene_type
At_chr11	Cufflinks	exon	99159492	99160607	.	+	.	gene_id "XLOC_026227"; transcript_id "Novel_000400"; exon_number "15"; gene_type
At_chr11	Cufflinks	exon	99155374	99155697	.	+	.	gene_id "XLOC_026227"; transcript_id "Novel_000401"; exon_number "1"; gene_type "
At_chr11	Cufflinks	exon	99156234	99156332	.	+	.	gene_id "XLOC_026227"; transcript_id "Novel_000401"; exon_number "2"; gene_type "
At_chr11	Cufflinks	exon	99156456	99156567	.	+	.	gene_id "XLOC_026227"; transcript_id "Novel_000401"; exon_number "3"; gene_type "
At_chr11	Cufflinks	exon	99156701	99156856	.	+	.	gene_id "XLOC_026227"; transcript_id "Novel_000401"; exon_number "4"; gene_type "
At_chr11	Cufflinks	exon	99157616	99157720	.	+	.	gene_id "XLOC_026227"; transcript_id "Novel_000401"; exon_number "5"; gene_type "
At_chr11	Cufflinks	exon	99157943	99158026	.	+	.	gene_id "XLOC_026227"; transcript_id "Novel_000401"; exon_number "6"; gene_type "
At_chr11	Cufflinks	exon	99158114	99158152	.	+	.	gene_id "XLOC_026227"; transcript_id "Novel_000401"; exon_number "7"; gene_type "
At_chr11	Cufflinks	exon	278713	279941	.	-	.	gene_id "XLOC_026386"; transcript_id "Novel_000402"; exon_number "1"; gene_type "
At_chr11	Cufflinks	exon	280202	280368	.	-	.	gene_id "XLOC_026386"; transcript_id "Novel_000402"; exon_number "2"; gene_type "
At_chr11	Cufflinks	exon	1061895	1064942	.	-	.	gene_id "XLOC_026490"; transcript_id "Novel_000403"; exon_number "1"; gene_type "
At_chr11	Cufflinks	exon	1065259	1065352	.	-	.	gene_id "XLOC_026490"; transcript_id "Novel_000403"; exon_number "2"; gene_type "
At_chr11	Cufflinks	exon	1431179	1431654	.	-	.	gene_id "XLOC_026532"; transcript_id "Novel_000404"; exon_number "1"; gene_type "
At_chr11	Cufflinks	exon	1433585	1433705	.	-	.	gene_id "XLOC_026532"; transcript_id "Novel_000404"; exon_number "2"; gene_type "
At_chr11	Cufflinks	exon	1431179	1431654	.	-	.	gene_id "XLOC_026532"; transcript_id "Novel_000405"; exon_number "1"; gene_type "
At_chr11	Cufflinks	exon	1433585	1433621	.	-	.	gene_id "XLOC_026532"; transcript_id "Novel_000405"; exon_number "2"; gene_type "
At_chr11	Cufflinks	exon	1434252	1434316	.	-	.	gene_id "XLOC_026532"; transcript_id "Novel_000405"; exon_number "3"; gene_type "
At_chr11	Cufflinks	exon	1434485	1435038	.	-	.	gene_id "XLOC_026532"; transcript_id "Novel_000405"; exon_number "4"; gene_type "
At_chr11	Cufflinks	exon	1431180	1431654	.	-	.	gene_id "XLOC_026532"; transcript_id "Novel_000406"; exon_number "1"; gene_type "
At_chr11	Cufflinks	exon	1433585	1433621	.	-	.	gene_id "XLOC_026532"; transcript_id "Novel_000406"; exon_number "2"; gene_type "
At_chr11	Cufflinks	exon	1433753	1434270	.	-	.	gene_id "XLOC_026532"; transcript_id "Novel_000406"; exon_number "3"; gene_type "
At_chr11	Cufflinks	exon	3547816	3548764	.	-	.	gene_id "XLOC_026720"; transcript_id "Novel_000407"; exon_number "1"; gene_type "
At_chr11	Cufflinks	exon	5796640	5797103	.	-	.	gene_id "XLOC_026901"; transcript_id "Novel_000408"; exon_number "1"; gene_type "

At_chr11	Cufflinks	exon	5797192	5798476	.	-	.	gene_id "XLOC_026901"; transcript_id "Novel_000408"; exon_number "2"; gene_type "
At_chr11	Cufflinks	exon	5798571	5798703	.	-	.	gene_id "XLOC_026901"; transcript_id "Novel_000408"; exon_number "3"; gene_type "
At_chr11	Cufflinks	exon	7817272	7817458	.	-	.	gene_id "XLOC_027126"; transcript_id "Novel_000409"; exon_number "1"; gene_type "
At_chr11	Cufflinks	exon	7817547	7817629	.	-	.	gene_id "XLOC_027126"; transcript_id "Novel_000409"; exon_number "2"; gene_type "
At_chr11	Cufflinks	exon	7817743	7817831	.	-	.	gene_id "XLOC_027126"; transcript_id "Novel_000409"; exon_number "3"; gene_type "
At_chr11	Cufflinks	exon	7818118	7818157	.	-	.	gene_id "XLOC_027126"; transcript_id "Novel_000409"; exon_number "4"; gene_type "
At_chr11	Cufflinks	exon	7818631	7818892	.	-	.	gene_id "XLOC_027126"; transcript_id "Novel_000409"; exon_number "5"; gene_type "
At_chr11	Cufflinks	exon	8395441	8396026	.	-	.	gene_id "XLOC_027182"; transcript_id "Novel_000410"; exon_number "1"; gene_type "
At_chr11	Cufflinks	exon	8398536	8398624	.	-	.	gene_id "XLOC_027182"; transcript_id "Novel_000410"; exon_number "2"; gene_type "
At_chr11	Cufflinks	exon	8395687	8396026	.	-	.	gene_id "XLOC_027182"; transcript_id "Novel_000411"; exon_number "1"; gene_type "
At_chr11	Cufflinks	exon	8397948	8398152	.	-	.	gene_id "XLOC_027182"; transcript_id "Novel_000411"; exon_number "2"; gene_type "
At_chr11	Cufflinks	exon	11269907	11270763	.	-	.	gene_id "XLOC_027335"; transcript_id "Novel_000412"; exon_number "1"; gene_type "
At_chr11	Cufflinks	exon	11270895	11271331	.	-	.	gene_id "XLOC_027335"; transcript_id "Novel_000412"; exon_number "2"; gene_type "
At_chr11	Cufflinks	exon	11294630	11295059	.	-	.	gene_id "XLOC_027338"; transcript_id "Novel_000413"; exon_number "1"; gene_type "
At_chr11	Cufflinks	exon	11295905	11296036	.	-	.	gene_id "XLOC_027338"; transcript_id "Novel_000413"; exon_number "2"; gene_type "
At_chr11	Cufflinks	exon	11296128	11296468	.	-	.	gene_id "XLOC_027338"; transcript_id "Novel_000413"; exon_number "3"; gene_type "
At_chr11	Cufflinks	exon	11373154	11373386	.	-	.	gene_id "XLOC_027343"; transcript_id "Novel_000414"; exon_number "1"; gene_type "
At_chr11	Cufflinks	exon	11373882	11374779	.	-	.	gene_id "XLOC_027343"; transcript_id "Novel_000414"; exon_number "2"; gene_type "
At_chr11	Cufflinks	exon	11378414	11379285	.	-	.	gene_id "XLOC_027343"; transcript_id "Novel_000414"; exon_number "3"; gene_type "
At_chr11	Cufflinks	exon	11587642	11587706	.	-	.	gene_id "XLOC_027363"; transcript_id "Novel_000415"; exon_number "1"; gene_type "
At_chr11	Cufflinks	exon	11587807	11588072	.	-	.	gene_id "XLOC_027363"; transcript_id "Novel_000415"; exon_number "2"; gene_type "
At_chr11	Cufflinks	exon	12143403	12144539	.	-	.	gene_id "XLOC_027404"; transcript_id "Novel_000416"; exon_number "1"; gene_type "
At_chr11	Cufflinks	exon	12144631	12145074	.	-	.	gene_id "XLOC_027404"; transcript_id "Novel_000416"; exon_number "2"; gene_type "
At_chr11	Cufflinks	exon	12145448	12146416	.	-	.	gene_id "XLOC_027404"; transcript_id "Novel_000416"; exon_number "3"; gene_type "
At_chr11	Cufflinks	exon	12146861	12146926	.	-	.	gene_id "XLOC_027404"; transcript_id "Novel_000416"; exon_number "4"; gene_type "
At_chr11	Cufflinks	exon	12147245	12147995	.	-	.	gene_id "XLOC_027404"; transcript_id "Novel_000416"; exon_number "5"; gene_type "
At_chr11	Cufflinks	exon	12143429	12144539	.	-	.	gene_id "XLOC_027404"; transcript_id "Novel_000417"; exon_number "1"; gene_type "
At_chr11	Cufflinks	exon	12144631	12144722	.	-	.	gene_id "XLOC_027404"; transcript_id "Novel_000417"; exon_number "2"; gene_type "
At_chr11	Cufflinks	exon	12144801	12146416	.	-	.	gene_id "XLOC_027404"; transcript_id "Novel_000417"; exon_number "3"; gene_type "
At_chr11	Cufflinks	exon	12146861	12146926	.	-	.	gene_id "XLOC_027404"; transcript_id "Novel_000417"; exon_number "4"; gene_type "
At_chr11	Cufflinks	exon	12147467	12147976	.	-	.	gene_id "XLOC_027404"; transcript_id "Novel_000417"; exon_number "5"; gene_type "
At_chr11	Cufflinks	exon	12143448	12144539	.	-	.	gene_id "XLOC_027404"; transcript_id "Novel_000418"; exon_number "1"; gene_type "
At_chr11	Cufflinks	exon	12144631	12144722	.	-	.	gene_id "XLOC_027404"; transcript_id "Novel_000418"; exon_number "2"; gene_type "
At_chr11	Cufflinks	exon	12144801	12145074	.	-	.	gene_id "XLOC_027404"; transcript_id "Novel_000418"; exon_number "3"; gene_type "
At_chr11	Cufflinks	exon	12145448	12146416	.	-	.	gene_id "XLOC_027404"; transcript_id "Novel_000418"; exon_number "4"; gene_type "

At_chr11	Cufflinks	exon	12146861	12146926	.	-	.	gene_id "XLOC_027404"; transcript_id "Novel_000418"; exon_number "5"; gene_type "
At_chr11	Cufflinks	exon	12147467	12147973	.	-	.	gene_id "XLOC_027404"; transcript_id "Novel_000418"; exon_number "6"; gene_type "
At_chr11	Cufflinks	exon	12319533	12319661	.	-	.	gene_id "XLOC_027418"; transcript_id "Novel_000419"; exon_number "1"; gene_type "
At_chr11	Cufflinks	exon	12329739	12329815	.	-	.	gene_id "XLOC_027418"; transcript_id "Novel_000419"; exon_number "2"; gene_type "
At_chr11	Cufflinks	exon	12330135	12331312	.	-	.	gene_id "XLOC_027418"; transcript_id "Novel_000419"; exon_number "3"; gene_type "
At_chr11	Cufflinks	exon	12327096	12329815	.	-	.	gene_id "XLOC_027418"; transcript_id "Novel_000420"; exon_number "1"; gene_type "
At_chr11	Cufflinks	exon	12330135	12331300	.	-	.	gene_id "XLOC_027418"; transcript_id "Novel_000420"; exon_number "2"; gene_type "
At_chr11	Cufflinks	exon	13479812	13480779	.	-	.	gene_id "XLOC_027524"; transcript_id "Novel_000421"; exon_number "1"; gene_type "
At_chr11	Cufflinks	exon	13481458	13482138	.	-	.	gene_id "XLOC_027524"; transcript_id "Novel_000421"; exon_number "2"; gene_type "
At_chr11	Cufflinks	exon	13479812	13480015	.	-	.	gene_id "XLOC_027524"; transcript_id "Novel_000422"; exon_number "1"; gene_type "
At_chr11	Cufflinks	exon	13480685	13480779	.	-	.	gene_id "XLOC_027524"; transcript_id "Novel_000422"; exon_number "2"; gene_type "
At_chr11	Cufflinks	exon	13481458	13482208	.	-	.	gene_id "XLOC_027524"; transcript_id "Novel_000422"; exon_number "3"; gene_type "
At_chr11	Cufflinks	exon	14873388	14873598	.	-	.	gene_id "XLOC_027600"; transcript_id "Novel_000423"; exon_number "1"; gene_type "
At_chr11	Cufflinks	exon	16212038	16212613	.	-	.	gene_id "XLOC_027658"; transcript_id "Novel_000424"; exon_number "1"; gene_type "
At_chr11	Cufflinks	exon	16213239	16213319	.	-	.	gene_id "XLOC_027658"; transcript_id "Novel_000424"; exon_number "2"; gene_type "
At_chr11	Cufflinks	exon	16214064	16214442	.	-	.	gene_id "XLOC_027658"; transcript_id "Novel_000424"; exon_number "3"; gene_type "
At_chr11	Cufflinks	exon	20922212	20922656	.	-	.	gene_id "XLOC_027931"; transcript_id "Novel_000425"; exon_number "1"; gene_type "
At_chr11	Cufflinks	exon	20922991	20923176	.	-	.	gene_id "XLOC_027931"; transcript_id "Novel_000425"; exon_number "2"; gene_type "
At_chr11	Cufflinks	exon	21257708	21259140	.	-	.	gene_id "XLOC_027991"; transcript_id "Novel_000426"; exon_number "1"; gene_type "
At_chr11	Cufflinks	exon	21259380	21259427	.	-	.	gene_id "XLOC_027991"; transcript_id "Novel_000426"; exon_number "2"; gene_type "
At_chr11	Cufflinks	exon	21259826	21259891	.	-	.	gene_id "XLOC_027991"; transcript_id "Novel_000426"; exon_number "3"; gene_type "
At_chr11	Cufflinks	exon	21260159	21260224	.	-	.	gene_id "XLOC_027991"; transcript_id "Novel_000426"; exon_number "4"; gene_type "
At_chr11	Cufflinks	exon	21260710	21260833	.	-	.	gene_id "XLOC_027991"; transcript_id "Novel_000426"; exon_number "5"; gene_type "
At_chr11	Cufflinks	exon	21262131	21262585	.	-	.	gene_id "XLOC_027991"; transcript_id "Novel_000426"; exon_number "6"; gene_type "
At_chr11	Cufflinks	exon	21257708	21259140	.	-	.	gene_id "XLOC_027991"; transcript_id "Novel_000427"; exon_number "1"; gene_type "
At_chr11	Cufflinks	exon	21259380	21259427	.	-	.	gene_id "XLOC_027991"; transcript_id "Novel_000427"; exon_number "2"; gene_type "
At_chr11	Cufflinks	exon	21259826	21259891	.	-	.	gene_id "XLOC_027991"; transcript_id "Novel_000427"; exon_number "3"; gene_type "
At_chr11	Cufflinks	exon	21260159	21260224	.	-	.	gene_id "XLOC_027991"; transcript_id "Novel_000427"; exon_number "4"; gene_type "
At_chr11	Cufflinks	exon	21260710	21260836	.	-	.	gene_id "XLOC_027991"; transcript_id "Novel_000427"; exon_number "5"; gene_type "
At_chr11	Cufflinks	exon	21262131	21262585	.	-	.	gene_id "XLOC_027991"; transcript_id "Novel_000427"; exon_number "6"; gene_type "
At_chr11	Cufflinks	exon	21257708	21259140	.	-	.	gene_id "XLOC_027991"; transcript_id "Novel_000428"; exon_number "1"; gene_type "
At_chr11	Cufflinks	exon	21259359	21259427	.	-	.	gene_id "XLOC_027991"; transcript_id "Novel_000428"; exon_number "2"; gene_type "
At_chr11	Cufflinks	exon	21259826	21259891	.	-	.	gene_id "XLOC_027991"; transcript_id "Novel_000428"; exon_number "3"; gene_type "
At_chr11	Cufflinks	exon	21260159	21260224	.	-	.	gene_id "XLOC_027991"; transcript_id "Novel_000428"; exon_number "4"; gene_type "
At_chr11	Cufflinks	exon	21260710	21260833	.	-	.	gene_id "XLOC_027991"; transcript_id "Novel_000428"; exon_number "5"; gene_type "

At_chr11	Cufflinks	exon	21262131	21262585	.	-	.	gene_id "XLOC_027991"; transcript_id "Novel_000428"; exon_number "6"; gene_type "
At_chr11	Cufflinks	exon	21257868	21260833	.	-	.	gene_id "XLOC_027991"; transcript_id "Novel_000429"; exon_number "1"; gene_type "
At_chr11	Cufflinks	exon	21262131	21262615	.	-	.	gene_id "XLOC_027991"; transcript_id "Novel_000429"; exon_number "2"; gene_type "
At_chr11	Cufflinks	exon	21258656	21259140	.	-	.	gene_id "XLOC_027991"; transcript_id "Novel_000430"; exon_number "1"; gene_type "
At_chr11	Cufflinks	exon	21259380	21259427	.	-	.	gene_id "XLOC_027991"; transcript_id "Novel_000430"; exon_number "2"; gene_type "
At_chr11	Cufflinks	exon	21259826	21259978	.	-	.	gene_id "XLOC_027991"; transcript_id "Novel_000430"; exon_number "3"; gene_type "
At_chr11	Cufflinks	exon	21260159	21260224	.	-	.	gene_id "XLOC_027991"; transcript_id "Novel_000430"; exon_number "4"; gene_type "
At_chr11	Cufflinks	exon	21260710	21260833	.	-	.	gene_id "XLOC_027991"; transcript_id "Novel_000430"; exon_number "5"; gene_type "
At_chr11	Cufflinks	exon	21262131	21262653	.	-	.	gene_id "XLOC_027991"; transcript_id "Novel_000430"; exon_number "6"; gene_type "
At_chr11	Cufflinks	exon	21708899	21708927	.	-	.	gene_id "XLOC_028010"; transcript_id "Novel_000431"; exon_number "1"; gene_type "
At_chr11	Cufflinks	exon	21709808	21710203	.	-	.	gene_id "XLOC_028010"; transcript_id "Novel_000431"; exon_number "2"; gene_type "
At_chr11	Cufflinks	exon	22017372	22017731	.	-	.	gene_id "XLOC_028024"; transcript_id "Novel_000432"; exon_number "1"; gene_type "
At_chr11	Cufflinks	exon	22017852	22017944	.	-	.	gene_id "XLOC_028024"; transcript_id "Novel_000432"; exon_number "2"; gene_type "
At_chr11	Cufflinks	exon	22018397	22018480	.	-	.	gene_id "XLOC_028024"; transcript_id "Novel_000432"; exon_number "3"; gene_type "
At_chr11	Cufflinks	exon	22018632	22018824	.	-	.	gene_id "XLOC_028024"; transcript_id "Novel_000432"; exon_number "4"; gene_type "
At_chr11	Cufflinks	exon	22019804	22019990	.	-	.	gene_id "XLOC_028024"; transcript_id "Novel_000432"; exon_number "5"; gene_type "
At_chr11	Cufflinks	exon	22201770	22202270	.	-	.	gene_id "XLOC_028039"; transcript_id "Novel_000433"; exon_number "1"; gene_type "
At_chr11	Cufflinks	exon	27733795	27733953	.	-	.	gene_id "XLOC_028370"; transcript_id "Novel_000434"; exon_number "1"; gene_type "
At_chr11	Cufflinks	exon	27734692	27734925	.	-	.	gene_id "XLOC_028370"; transcript_id "Novel_000434"; exon_number "2"; gene_type "
At_chr11	Cufflinks	exon	35004914	35005018	.	-	.	gene_id "XLOC_028772"; transcript_id "Novel_000435"; exon_number "1"; gene_type "
At_chr11	Cufflinks	exon	35005425	35005512	.	-	.	gene_id "XLOC_028772"; transcript_id "Novel_000435"; exon_number "2"; gene_type "
At_chr11	Cufflinks	exon	35006478	35006517	.	-	.	gene_id "XLOC_028772"; transcript_id "Novel_000435"; exon_number "3"; gene_type "
At_chr11	Cufflinks	exon	39776314	39776613	.	-	.	gene_id "XLOC_028951"; transcript_id "Novel_000436"; exon_number "1"; gene_type "
At_chr11	Cufflinks	exon	40164652	40164813	.	-	.	gene_id "XLOC_028962"; transcript_id "Novel_000437"; exon_number "1"; gene_type "
At_chr11	Cufflinks	exon	40166395	40166424	.	-	.	gene_id "XLOC_028962"; transcript_id "Novel_000437"; exon_number "2"; gene_type "
At_chr11	Cufflinks	exon	40166506	40166577	.	-	.	gene_id "XLOC_028962"; transcript_id "Novel_000437"; exon_number "3"; gene_type "
At_chr11	Cufflinks	exon	40167026	40168325	.	-	.	gene_id "XLOC_028962"; transcript_id "Novel_000437"; exon_number "4"; gene_type "
At_chr11	Cufflinks	exon	46122083	46124268	.	-	.	gene_id "XLOC_029109"; transcript_id "Novel_000438"; exon_number "1"; gene_type "
At_chr11	Cufflinks	exon	46124557	46124809	.	-	.	gene_id "XLOC_029109"; transcript_id "Novel_000438"; exon_number "2"; gene_type "
At_chr11	Cufflinks	exon	54314840	54315928	.	-	.	gene_id "XLOC_029381"; transcript_id "Novel_000439"; exon_number "1"; gene_type "
At_chr11	Cufflinks	exon	54316028	54318386	.	-	.	gene_id "XLOC_029381"; transcript_id "Novel_000439"; exon_number "2"; gene_type "
At_chr11	Cufflinks	exon	54314848	54315928	.	-	.	gene_id "XLOC_029381"; transcript_id "Novel_000440"; exon_number "1"; gene_type "
At_chr11	Cufflinks	exon	54316028	54317317	.	-	.	gene_id "XLOC_029381"; transcript_id "Novel_000440"; exon_number "2"; gene_type "
At_chr11	Cufflinks	exon	54317401	54318386	.	-	.	gene_id "XLOC_029381"; transcript_id "Novel_000440"; exon_number "3"; gene_type "
At_chr11	Cufflinks	exon	55341817	55342409	.	-	.	gene_id "XLOC_029454"; transcript_id "Novel_000441"; exon_number "1"; gene_type "

At_chr11 Cufflinks exon 55342516 55342835 . - . gene_id "XLOC_029454"; transcript_id "Novel_000441"; exon_number "2"; gene_type "
At_chr11 Cufflinks exon 56965361 56968474 . - . gene_id "XLOC_029635"; transcript_id "Novel_000442"; exon_number "1"; gene_type "
At_chr11 Cufflinks exon 56968681 56970433 . - . gene_id "XLOC_029635"; transcript_id "Novel_000442"; exon_number "2"; gene_type "
At_chr11 Cufflinks exon 57663659 57666178 . - . gene_id "XLOC_029705"; transcript_id "Novel_000443"; exon_number "1"; gene_type "
At_chr11 Cufflinks exon 57666419 57666706 . - . gene_id "XLOC_029705"; transcript_id "Novel_000443"; exon_number "2"; gene_type "
At_chr11 Cufflinks exon 58405397 58406179 . - . gene_id "XLOC_029779"; transcript_id "Novel_000444"; exon_number "1"; gene_type "
At_chr11 Cufflinks exon 58406269 58406605 . - . gene_id "XLOC_029779"; transcript_id "Novel_000444"; exon_number "2"; gene_type "
At_chr11 Cufflinks exon 58406710 58406837 . - . gene_id "XLOC_029779"; transcript_id "Novel_000444"; exon_number "3"; gene_type "
At_chr11 Cufflinks exon 58407107 58407550 . - . gene_id "XLOC_029779"; transcript_id "Novel_000444"; exon_number "4"; gene_type "
At_chr11 Cufflinks exon 58407623 58407713 . - . gene_id "XLOC_029779"; transcript_id "Novel_000444"; exon_number "5"; gene_type "
At_chr11 Cufflinks exon 58407797 58407906 . - . gene_id "XLOC_029779"; transcript_id "Novel_000444"; exon_number "6"; gene_type "
At_chr11 Cufflinks exon 58408177 58408286 . - . gene_id "XLOC_029779"; transcript_id "Novel_000444"; exon_number "7"; gene_type "
At_chr11 Cufflinks exon 58408405 58408507 . - . gene_id "XLOC_029779"; transcript_id "Novel_000444"; exon_number "8"; gene_type "
At_chr11 Cufflinks exon 58408902 58408946 . - . gene_id "XLOC_029779"; transcript_id "Novel_000444"; exon_number "9"; gene_type "
At_chr11 Cufflinks exon 58409062 58409186 . - . gene_id "XLOC_029779"; transcript_id "Novel_000444"; exon_number "10"; gene_type
At_chr11 Cufflinks exon 58409296 58409366 . - . gene_id "XLOC_029779"; transcript_id "Novel_000444"; exon_number "11"; gene_type
At_chr11 Cufflinks exon 58409468 58409632 . - . gene_id "XLOC_029779"; transcript_id "Novel_000444"; exon_number "12"; gene_type
At_chr11 Cufflinks exon 60787165 60787465 . - . gene_id "XLOC_029957"; transcript_id "Novel_000445"; exon_number "1"; gene_type "
At_chr11 Cufflinks exon 60964844 60964966 . - . gene_id "XLOC_029965"; transcript_id "Novel_000446"; exon_number "1"; gene_type "
At_chr11 Cufflinks exon 60965089 60965181 . - . gene_id "XLOC_029965"; transcript_id "Novel_000446"; exon_number "2"; gene_type "
At_chr11 Cufflinks exon 60965754 60966081 . - . gene_id "XLOC_029965"; transcript_id "Novel_000446"; exon_number "3"; gene_type "
At_chr11 Cufflinks exon 60966900 60967198 . - . gene_id "XLOC_029965"; transcript_id "Novel_000446"; exon_number "4"; gene_type "
At_chr11 Cufflinks exon 60968197 60968349 . - . gene_id "XLOC_029965"; transcript_id "Novel_000446"; exon_number "5"; gene_type "
At_chr11 Cufflinks exon 60968953 60969084 . - . gene_id "XLOC_029965"; transcript_id "Novel_000446"; exon_number "6"; gene_type "
At_chr11 Cufflinks exon 60964883 60964966 . - . gene_id "XLOC_029965"; transcript_id "Novel_000447"; exon_number "1"; gene_type "
At_chr11 Cufflinks exon 60965089 60965181 . - . gene_id "XLOC_029965"; transcript_id "Novel_000447"; exon_number "2"; gene_type "
At_chr11 Cufflinks exon 60965754 60966081 . - . gene_id "XLOC_029965"; transcript_id "Novel_000447"; exon_number "3"; gene_type "
At_chr11 Cufflinks exon 60967082 60967198 . - . gene_id "XLOC_029965"; transcript_id "Novel_000447"; exon_number "4"; gene_type "
At_chr11 Cufflinks exon 60968197 60968349 . - . gene_id "XLOC_029965"; transcript_id "Novel_000447"; exon_number "5"; gene_type "
At_chr11 Cufflinks exon 60968953 60969147 . - . gene_id "XLOC_029965"; transcript_id "Novel_000447"; exon_number "6"; gene_type "
At_chr11 Cufflinks exon 62865863 62866140 . - . gene_id "XLOC_030207"; transcript_id "Novel_000448"; exon_number "1"; gene_type "
At_chr11 Cufflinks exon 62913447 62913813 . - . gene_id "XLOC_030218"; transcript_id "Novel_000449"; exon_number "1"; gene_type "
At_chr11 Cufflinks exon 62914612 62915342 . - . gene_id "XLOC_030218"; transcript_id "Novel_000449"; exon_number "2"; gene_type "
At_chr11 Cufflinks exon 67254690 67256489 . - . gene_id "XLOC_030514"; transcript_id "Novel_000450"; exon_number "1"; gene_type "
At_chr11 Cufflinks exon 67256706 67256843 . - . gene_id "XLOC_030514"; transcript_id "Novel_000450"; exon_number "2"; gene_type "

At_chr11	Cufflinks	exon	67256937	67257092	.	-	.	gene_id "XLOC_030514"; transcript_id "Novel_000450"; exon_number "3"; gene_type "
At_chr11	Cufflinks	exon	67254690	67256489	.	-	.	gene_id "XLOC_030514"; transcript_id "Novel_000451"; exon_number "1"; gene_type "
At_chr11	Cufflinks	exon	67256937	67257092	.	-	.	gene_id "XLOC_030514"; transcript_id "Novel_000451"; exon_number "2"; gene_type "
At_chr11	Cufflinks	exon	67254690	67256489	.	-	.	gene_id "XLOC_030514"; transcript_id "Novel_000452"; exon_number "1"; gene_type "
At_chr11	Cufflinks	exon	67256706	67257092	.	-	.	gene_id "XLOC_030514"; transcript_id "Novel_000452"; exon_number "2"; gene_type "
At_chr11	Cufflinks	exon	67797232	67797390	.	-	.	gene_id "XLOC_030563"; transcript_id "Novel_000453"; exon_number "1"; gene_type "
At_chr11	Cufflinks	exon	67797779	67797845	.	-	.	gene_id "XLOC_030563"; transcript_id "Novel_000453"; exon_number "2"; gene_type "
At_chr11	Cufflinks	exon	67797938	67798047	.	-	.	gene_id "XLOC_030563"; transcript_id "Novel_000453"; exon_number "3"; gene_type "
At_chr11	Cufflinks	exon	67798522	67798622	.	-	.	gene_id "XLOC_030563"; transcript_id "Novel_000453"; exon_number "4"; gene_type "
At_chr11	Cufflinks	exon	67798869	67798955	.	-	.	gene_id "XLOC_030563"; transcript_id "Novel_000453"; exon_number "5"; gene_type "
At_chr11	Cufflinks	exon	68552445	68554166	.	-	.	gene_id "XLOC_030612"; transcript_id "Novel_000454"; exon_number "1"; gene_type "
At_chr11	Cufflinks	exon	68555076	68555924	.	-	.	gene_id "XLOC_030612"; transcript_id "Novel_000454"; exon_number "2"; gene_type "
At_chr11	Cufflinks	exon	69676621	69676841	.	-	.	gene_id "XLOC_030680"; transcript_id "Novel_000455"; exon_number "1"; gene_type "
At_chr11	Cufflinks	exon	76678986	76679210	.	-	.	gene_id "XLOC_030907"; transcript_id "Novel_000456"; exon_number "1"; gene_type "
At_chr11	Cufflinks	exon	81227524	81229382	.	-	.	gene_id "XLOC_031017"; transcript_id "Novel_000457"; exon_number "1"; gene_type "
At_chr11	Cufflinks	exon	84182834	84184422	.	-	.	gene_id "XLOC_031102"; transcript_id "Novel_000458"; exon_number "1"; gene_type "
At_chr11	Cufflinks	exon	84185571	84185637	.	-	.	gene_id "XLOC_031102"; transcript_id "Novel_000458"; exon_number "2"; gene_type "
At_chr11	Cufflinks	exon	84186569	84186646	.	-	.	gene_id "XLOC_031102"; transcript_id "Novel_000458"; exon_number "3"; gene_type "
At_chr11	Cufflinks	exon	84186957	84187202	.	-	.	gene_id "XLOC_031102"; transcript_id "Novel_000458"; exon_number "4"; gene_type "
At_chr11	Cufflinks	exon	85120794	85123804	.	-	.	gene_id "XLOC_031145"; transcript_id "Novel_000459"; exon_number "1"; gene_type "
At_chr11	Cufflinks	exon	85124351	85124644	.	-	.	gene_id "XLOC_031145"; transcript_id "Novel_000459"; exon_number "2"; gene_type "
At_chr11	Cufflinks	exon	89503551	89505292	.	-	.	gene_id "XLOC_031264"; transcript_id "Novel_000460"; exon_number "1"; gene_type "
At_chr11	Cufflinks	exon	89505400	89505635	.	-	.	gene_id "XLOC_031264"; transcript_id "Novel_000460"; exon_number "2"; gene_type "
At_chr11	Cufflinks	exon	89503588	89504220	.	-	.	gene_id "XLOC_031264"; transcript_id "Novel_000461"; exon_number "1"; gene_type "
At_chr11	Cufflinks	exon	89504681	89504707	.	-	.	gene_id "XLOC_031264"; transcript_id "Novel_000461"; exon_number "2"; gene_type "
At_chr11	Cufflinks	exon	89505218	89505292	.	-	.	gene_id "XLOC_031264"; transcript_id "Novel_000461"; exon_number "3"; gene_type "
At_chr11	Cufflinks	exon	89505400	89505656	.	-	.	gene_id "XLOC_031264"; transcript_id "Novel_000461"; exon_number "4"; gene_type "
At_chr11	Cufflinks	exon	89503685	89504027	.	-	.	gene_id "XLOC_031264"; transcript_id "Novel_000462"; exon_number "1"; gene_type "
At_chr11	Cufflinks	exon	89504106	89504220	.	-	.	gene_id "XLOC_031264"; transcript_id "Novel_000462"; exon_number "2"; gene_type "
At_chr11	Cufflinks	exon	89504681	89504707	.	-	.	gene_id "XLOC_031264"; transcript_id "Novel_000462"; exon_number "3"; gene_type "
At_chr11	Cufflinks	exon	89505218	89505292	.	-	.	gene_id "XLOC_031264"; transcript_id "Novel_000462"; exon_number "4"; gene_type "
At_chr11	Cufflinks	exon	89505400	89505641	.	-	.	gene_id "XLOC_031264"; transcript_id "Novel_000462"; exon_number "5"; gene_type "
At_chr11	Cufflinks	exon	90005662	90005904	.	-	.	gene_id "XLOC_031282"; transcript_id "Novel_000463"; exon_number "1"; gene_type "
At_chr11	Cufflinks	exon	90006408	90006858	.	-	.	gene_id "XLOC_031282"; transcript_id "Novel_000463"; exon_number "2"; gene_type "
At_chr11	Cufflinks	exon	90007846	90008020	.	-	.	gene_id "XLOC_031282"; transcript_id "Novel_000463"; exon_number "3"; gene_type "

At_chr11	Cufflinks	exon	90005662	90006858	.	-	.	gene_id "XLOC_031282"; transcript_id "Novel_000464"; exon_number "1"; gene_type "
At_chr11	Cufflinks	exon	90007846	90008020	.	-	.	gene_id "XLOC_031282"; transcript_id "Novel_000464"; exon_number "2"; gene_type "
At_chr11	Cufflinks	exon	90005723	90005904	.	-	.	gene_id "XLOC_031282"; transcript_id "Novel_000465"; exon_number "1"; gene_type "
At_chr11	Cufflinks	exon	90006408	90007558	.	-	.	gene_id "XLOC_031282"; transcript_id "Novel_000465"; exon_number "2"; gene_type "
At_chr11	Cufflinks	exon	93918064	93918337	.	-	.	gene_id "XLOC_031476"; transcript_id "Novel_000466"; exon_number "1"; gene_type "
At_chr11	Cufflinks	exon	95663685	95664361	.	-	.	gene_id "XLOC_031558"; transcript_id "Novel_000467"; exon_number "1"; gene_type "
At_chr11	Cufflinks	exon	95664826	95664891	.	-	.	gene_id "XLOC_031558"; transcript_id "Novel_000467"; exon_number "2"; gene_type "
At_chr11	Cufflinks	exon	95665008	95665077	.	-	.	gene_id "XLOC_031558"; transcript_id "Novel_000467"; exon_number "3"; gene_type "
At_chr11	Cufflinks	exon	95665172	95666041	.	-	.	gene_id "XLOC_031558"; transcript_id "Novel_000467"; exon_number "4"; gene_type "
At_chr11	Cufflinks	exon	95663785	95664361	.	-	.	gene_id "XLOC_031558"; transcript_id "Novel_000468"; exon_number "1"; gene_type "
At_chr11	Cufflinks	exon	95665008	95665077	.	-	.	gene_id "XLOC_031558"; transcript_id "Novel_000468"; exon_number "2"; gene_type "
At_chr11	Cufflinks	exon	95665172	95666084	.	-	.	gene_id "XLOC_031558"; transcript_id "Novel_000468"; exon_number "3"; gene_type "
At_chr11	Cufflinks	exon	96761873	96762453	.	-	.	gene_id "XLOC_031599"; transcript_id "Novel_000469"; exon_number "1"; gene_type "
At_chr11	Cufflinks	exon	96763402	96763488	.	-	.	gene_id "XLOC_031599"; transcript_id "Novel_000469"; exon_number "2"; gene_type "
At_chr11	Cufflinks	exon	96763599	96764154	.	-	.	gene_id "XLOC_031599"; transcript_id "Novel_000469"; exon_number "3"; gene_type "
At_chr11	Cufflinks	exon	96761873	96762453	.	-	.	gene_id "XLOC_031599"; transcript_id "Novel_000470"; exon_number "1"; gene_type "
At_chr11	Cufflinks	exon	96763402	96763488	.	-	.	gene_id "XLOC_031599"; transcript_id "Novel_000470"; exon_number "2"; gene_type "
At_chr11	Cufflinks	exon	96763599	96763704	.	-	.	gene_id "XLOC_031599"; transcript_id "Novel_000470"; exon_number "3"; gene_type "
At_chr11	Cufflinks	exon	96763785	96763973	.	-	.	gene_id "XLOC_031599"; transcript_id "Novel_000470"; exon_number "4"; gene_type "
At_chr11	Cufflinks	exon	96764217	96764443	.	-	.	gene_id "XLOC_031599"; transcript_id "Novel_000470"; exon_number "5"; gene_type "
At_chr11	Cufflinks	exon	96762141	96762453	.	-	.	gene_id "XLOC_031599"; transcript_id "Novel_000471"; exon_number "1"; gene_type "
At_chr11	Cufflinks	exon	96763402	96763488	.	-	.	gene_id "XLOC_031599"; transcript_id "Novel_000471"; exon_number "2"; gene_type "
At_chr11	Cufflinks	exon	96763599	96763704	.	-	.	gene_id "XLOC_031599"; transcript_id "Novel_000471"; exon_number "3"; gene_type "
At_chr11	Cufflinks	exon	96763785	96764459	.	-	.	gene_id "XLOC_031599"; transcript_id "Novel_000471"; exon_number "4"; gene_type "
At_chr11	Cufflinks	exon	98433974	98436208	.	-	.	gene_id "XLOC_031661"; transcript_id "Novel_000472"; exon_number "1"; gene_type "
At_chr11	Cufflinks	exon	98442109	98442385	.	-	.	gene_id "XLOC_031661"; transcript_id "Novel_000472"; exon_number "2"; gene_type "
At_chr11	Cufflinks	exon	98442490	98442937	.	-	.	gene_id "XLOC_031661"; transcript_id "Novel_000472"; exon_number "3"; gene_type "
At_chr11	Cufflinks	exon	99207630	99208428	.	-	.	gene_id "XLOC_031707"; transcript_id "Novel_000473"; exon_number "1"; gene_type "
At_chr11	Cufflinks	exon	99208804	99209004	.	-	.	gene_id "XLOC_031707"; transcript_id "Novel_000473"; exon_number "2"; gene_type "
At_chr11	Cufflinks	exon	99209164	99209184	.	-	.	gene_id "XLOC_031708"; transcript_id "Novel_000474"; exon_number "1"; gene_type "
At_chr11	Cufflinks	exon	99209864	99210059	.	-	.	gene_id "XLOC_031708"; transcript_id "Novel_000474"; exon_number "2"; gene_type "
At_chr11	Cufflinks	exon	99210230	99210362	.	-	.	gene_id "XLOC_031708"; transcript_id "Novel_000474"; exon_number "3"; gene_type "
At_chr11	Cufflinks	exon	99210517	99210979	.	-	.	gene_id "XLOC_031708"; transcript_id "Novel_000474"; exon_number "4"; gene_type "
At_chr11	Cufflinks	exon	99209166	99209184	.	-	.	gene_id "XLOC_031708"; transcript_id "Novel_000475"; exon_number "1"; gene_type "
At_chr11	Cufflinks	exon	99209864	99210059	.	-	.	gene_id "XLOC_031708"; transcript_id "Novel_000475"; exon_number "2"; gene_type "

At_chr11	Cufflinks	exon	99210230	99210382	.	-	.	gene_id "XLOC_031708"; transcript_id "Novel_000475"; exon_number "3"; gene_type "
At_chr11	Cufflinks	exon	99212084	99212396	.	-	.	gene_id "XLOC_031708"; transcript_id "Novel_000476"; exon_number "1"; gene_type "
At_chr11	Cufflinks	exon	99212684	99212728	.	-	.	gene_id "XLOC_031708"; transcript_id "Novel_000476"; exon_number "2"; gene_type "
At_chr11	Cufflinks	exon	99213066	99213180	.	-	.	gene_id "XLOC_031708"; transcript_id "Novel_000476"; exon_number "3"; gene_type "
At_chr11	Cufflinks	exon	99213276	99213382	.	-	.	gene_id "XLOC_031708"; transcript_id "Novel_000476"; exon_number "4"; gene_type "
At_chr11	Cufflinks	exon	99213817	99213911	.	-	.	gene_id "XLOC_031708"; transcript_id "Novel_000476"; exon_number "5"; gene_type "
At_chr11	Cufflinks	exon	99214056	99214170	.	-	.	gene_id "XLOC_031708"; transcript_id "Novel_000476"; exon_number "6"; gene_type "
At_chr11	Cufflinks	exon	99214574	99214625	.	-	.	gene_id "XLOC_031708"; transcript_id "Novel_000476"; exon_number "7"; gene_type "
At_chr11	Cufflinks	exon	99214832	99214893	.	-	.	gene_id "XLOC_031708"; transcript_id "Novel_000476"; exon_number "8"; gene_type "
At_chr11	Cufflinks	exon	99214976	99215262	.	-	.	gene_id "XLOC_031708"; transcript_id "Novel_000476"; exon_number "9"; gene_type "
At_chr11	Cufflinks	exon	99213019	99213382	.	-	.	gene_id "XLOC_031708"; transcript_id "Novel_000477"; exon_number "1"; gene_type "
At_chr11	Cufflinks	exon	99213817	99213911	.	-	.	gene_id "XLOC_031708"; transcript_id "Novel_000477"; exon_number "2"; gene_type "
At_chr11	Cufflinks	exon	99214056	99214170	.	-	.	gene_id "XLOC_031708"; transcript_id "Novel_000477"; exon_number "3"; gene_type "
At_chr11	Cufflinks	exon	99214574	99214625	.	-	.	gene_id "XLOC_031708"; transcript_id "Novel_000477"; exon_number "4"; gene_type "
At_chr11	Cufflinks	exon	99214832	99214893	.	-	.	gene_id "XLOC_031708"; transcript_id "Novel_000477"; exon_number "5"; gene_type "
At_chr11	Cufflinks	exon	99214976	99215164	.	-	.	gene_id "XLOC_031708"; transcript_id "Novel_000477"; exon_number "6"; gene_type "
At_chr12	Cufflinks	exon	4200943	4201335	.	+	.	gene_id "XLOC_032068"; transcript_id "Novel_000478"; exon_number "1"; gene_type "
At_chr12	Cufflinks	exon	4201638	4202279	.	+	.	gene_id "XLOC_032068"; transcript_id "Novel_000478"; exon_number "2"; gene_type "
At_chr12	Cufflinks	exon	4202474	4202978	.	+	.	gene_id "XLOC_032068"; transcript_id "Novel_000478"; exon_number "3"; gene_type "
At_chr12	Cufflinks	exon	4200943	4202279	.	+	.	gene_id "XLOC_032068"; transcript_id "Novel_000479"; exon_number "1"; gene_type "
At_chr12	Cufflinks	exon	4202474	4202978	.	+	.	gene_id "XLOC_032068"; transcript_id "Novel_000479"; exon_number "2"; gene_type "
At_chr12	Cufflinks	exon	5138425	5138519	.	+	.	gene_id "XLOC_032091"; transcript_id "Novel_000480"; exon_number "1"; gene_type "
At_chr12	Cufflinks	exon	5138618	5138793	.	+	.	gene_id "XLOC_032091"; transcript_id "Novel_000480"; exon_number "2"; gene_type "
At_chr12	Cufflinks	exon	9514925	9515694	.	+	.	gene_id "XLOC_032238"; transcript_id "Novel_000481"; exon_number "1"; gene_type "
At_chr12	Cufflinks	exon	9516147	9516383	.	+	.	gene_id "XLOC_032238"; transcript_id "Novel_000481"; exon_number "2"; gene_type "
At_chr12	Cufflinks	exon	9516597	9518272	.	+	.	gene_id "XLOC_032238"; transcript_id "Novel_000481"; exon_number "3"; gene_type "
At_chr12	Cufflinks	exon	9601400	9601729	.	+	.	gene_id "XLOC_032253"; transcript_id "Novel_000482"; exon_number "1"; gene_type "
At_chr12	Cufflinks	exon	15575440	15575616	.	+	.	gene_id "XLOC_032428"; transcript_id "Novel_000483"; exon_number "1"; gene_type "
At_chr12	Cufflinks	exon	15576091	15576176	.	+	.	gene_id "XLOC_032428"; transcript_id "Novel_000483"; exon_number "2"; gene_type "
At_chr12	Cufflinks	exon	15576874	15579563	.	+	.	gene_id "XLOC_032428"; transcript_id "Novel_000483"; exon_number "3"; gene_type "
At_chr12	Cufflinks	exon	36236556	36236785	.	+	.	gene_id "XLOC_033107"; transcript_id "Novel_000484"; exon_number "1"; gene_type "
At_chr12	Cufflinks	exon	37032229	37032933	.	+	.	gene_id "XLOC_033129"; transcript_id "Novel_000485"; exon_number "1"; gene_type "
At_chr12	Cufflinks	exon	37033396	37033522	.	+	.	gene_id "XLOC_033129"; transcript_id "Novel_000485"; exon_number "2"; gene_type "
At_chr12	Cufflinks	exon	37033907	37034105	.	+	.	gene_id "XLOC_033129"; transcript_id "Novel_000485"; exon_number "3"; gene_type "
At_chr12	Cufflinks	exon	37035217	37035327	.	+	.	gene_id "XLOC_033129"; transcript_id "Novel_000485"; exon_number "4"; gene_type "

At_chr12	Cufflinks	exon	37036994	37037027	.	+	.	gene_id "XLOC_033129"; transcript_id "Novel_000485"; exon_number "5"; gene_type "
At_chr12	Cufflinks	exon	37037752	37038011	.	+	.	gene_id "XLOC_033129"; transcript_id "Novel_000485"; exon_number "6"; gene_type "
At_chr12	Cufflinks	exon	37032261	37032933	.	+	.	gene_id "XLOC_033129"; transcript_id "Novel_000486"; exon_number "1"; gene_type "
At_chr12	Cufflinks	exon	37033396	37033522	.	+	.	gene_id "XLOC_033129"; transcript_id "Novel_000486"; exon_number "2"; gene_type "
At_chr12	Cufflinks	exon	37033907	37035429	.	+	.	gene_id "XLOC_033129"; transcript_id "Novel_000486"; exon_number "3"; gene_type "
At_chr12	Cufflinks	exon	37032276	37032648	.	+	.	gene_id "XLOC_033129"; transcript_id "Novel_000487"; exon_number "1"; gene_type "
At_chr12	Cufflinks	exon	37032733	37032933	.	+	.	gene_id "XLOC_033129"; transcript_id "Novel_000487"; exon_number "2"; gene_type "
At_chr12	Cufflinks	exon	37033396	37033522	.	+	.	gene_id "XLOC_033129"; transcript_id "Novel_000487"; exon_number "3"; gene_type "
At_chr12	Cufflinks	exon	37033907	37034105	.	+	.	gene_id "XLOC_033129"; transcript_id "Novel_000487"; exon_number "4"; gene_type "
At_chr12	Cufflinks	exon	37035217	37035327	.	+	.	gene_id "XLOC_033129"; transcript_id "Novel_000487"; exon_number "5"; gene_type "
At_chr12	Cufflinks	exon	37036994	37037027	.	+	.	gene_id "XLOC_033129"; transcript_id "Novel_000487"; exon_number "6"; gene_type "
At_chr12	Cufflinks	exon	37037752	37038012	.	+	.	gene_id "XLOC_033129"; transcript_id "Novel_000487"; exon_number "7"; gene_type "
At_chr12	Cufflinks	exon	41073528	41073791	.	+	.	gene_id "XLOC_033225"; transcript_id "Novel_000488"; exon_number "1"; gene_type "
At_chr12	Cufflinks	exon	43873422	43874434	.	+	.	gene_id "XLOC_033450"; transcript_id "Novel_000489"; exon_number "1"; gene_type "
At_chr12	Cufflinks	exon	43874751	43878186	.	+	.	gene_id "XLOC_033450"; transcript_id "Novel_000489"; exon_number "2"; gene_type "
At_chr12	Cufflinks	exon	45395913	45396142	.	+	.	gene_id "XLOC_033613"; transcript_id "Novel_000490"; exon_number "1"; gene_type "
At_chr12	Cufflinks	exon	45396262	45396578	.	+	.	gene_id "XLOC_033613"; transcript_id "Novel_000490"; exon_number "2"; gene_type "
At_chr12	Cufflinks	exon	45395913	45396142	.	+	.	gene_id "XLOC_033613"; transcript_id "Novel_000491"; exon_number "1"; gene_type "
At_chr12	Cufflinks	exon	45396262	45396339	.	+	.	gene_id "XLOC_033613"; transcript_id "Novel_000491"; exon_number "2"; gene_type "
At_chr12	Cufflinks	exon	45397268	45397359	.	+	.	gene_id "XLOC_033613"; transcript_id "Novel_000491"; exon_number "3"; gene_type "
At_chr12	Cufflinks	exon	45397438	45397471	.	+	.	gene_id "XLOC_033613"; transcript_id "Novel_000491"; exon_number "4"; gene_type "
At_chr12	Cufflinks	exon	45397569	45397918	.	+	.	gene_id "XLOC_033613"; transcript_id "Novel_000491"; exon_number "5"; gene_type "
At_chr12	Cufflinks	exon	45395914	45396142	.	+	.	gene_id "XLOC_033613"; transcript_id "Novel_000492"; exon_number "1"; gene_type "
At_chr12	Cufflinks	exon	45396262	45396339	.	+	.	gene_id "XLOC_033613"; transcript_id "Novel_000492"; exon_number "2"; gene_type "
At_chr12	Cufflinks	exon	45397268	45397904	.	+	.	gene_id "XLOC_033613"; transcript_id "Novel_000492"; exon_number "3"; gene_type "
At_chr12	Cufflinks	exon	46635861	46637300	.	+	.	gene_id "XLOC_033670"; transcript_id "Novel_000493"; exon_number "1"; gene_type "
At_chr12	Cufflinks	exon	46637471	46638055	.	+	.	gene_id "XLOC_033670"; transcript_id "Novel_000493"; exon_number "2"; gene_type "
At_chr12	Cufflinks	exon	50636868	50637178	.	+	.	gene_id "XLOC_033844"; transcript_id "Novel_000494"; exon_number "1"; gene_type "
At_chr12	Cufflinks	exon	50638173	50638273	.	+	.	gene_id "XLOC_033844"; transcript_id "Novel_000494"; exon_number "2"; gene_type "
At_chr12	Cufflinks	exon	50638382	50638654	.	+	.	gene_id "XLOC_033844"; transcript_id "Novel_000494"; exon_number "3"; gene_type "
At_chr12	Cufflinks	exon	51631166	51631280	.	+	.	gene_id "XLOC_034026"; transcript_id "Novel_000495"; exon_number "1"; gene_type "
At_chr12	Cufflinks	exon	51631466	51631531	.	+	.	gene_id "XLOC_034026"; transcript_id "Novel_000495"; exon_number "2"; gene_type "
At_chr12	Cufflinks	exon	51633269	51633611	.	+	.	gene_id "XLOC_034026"; transcript_id "Novel_000495"; exon_number "3"; gene_type "
At_chr12	Cufflinks	exon	51680083	51680121	.	+	.	gene_id "XLOC_034034"; transcript_id "Novel_000496"; exon_number "1"; gene_type "
At_chr12	Cufflinks	exon	51680527	51680580	.	+	.	gene_id "XLOC_034034"; transcript_id "Novel_000496"; exon_number "2"; gene_type "

At_chr12	Cufflinks	exon	51680759	51680836	.	+	.	gene_id "XLOC_034034"; transcript_id "Novel_000496"; exon_number "3"; gene_type "
At_chr12	Cufflinks	exon	51680956	51680980	.	+	.	gene_id "XLOC_034034"; transcript_id "Novel_000496"; exon_number "4"; gene_type "
At_chr12	Cufflinks	exon	51681075	51681107	.	+	.	gene_id "XLOC_034034"; transcript_id "Novel_000496"; exon_number "5"; gene_type "
At_chr12	Cufflinks	exon	51681508	51681629	.	+	.	gene_id "XLOC_034034"; transcript_id "Novel_000496"; exon_number "6"; gene_type "
At_chr12	Cufflinks	exon	51681762	51682677	.	+	.	gene_id "XLOC_034034"; transcript_id "Novel_000496"; exon_number "7"; gene_type "
At_chr12	Cufflinks	exon	51682767	51683341	.	+	.	gene_id "XLOC_034034"; transcript_id "Novel_000496"; exon_number "8"; gene_type "
At_chr12	Cufflinks	exon	53964161	53964587	.	+	.	gene_id "XLOC_034310"; transcript_id "Novel_000497"; exon_number "1"; gene_type "
At_chr12	Cufflinks	exon	53964669	53964740	.	+	.	gene_id "XLOC_034310"; transcript_id "Novel_000497"; exon_number "2"; gene_type "
At_chr12	Cufflinks	exon	53964810	53964883	.	+	.	gene_id "XLOC_034310"; transcript_id "Novel_000497"; exon_number "3"; gene_type "
At_chr12	Cufflinks	exon	53964964	53965307	.	+	.	gene_id "XLOC_034310"; transcript_id "Novel_000497"; exon_number "4"; gene_type "
At_chr12	Cufflinks	exon	53964161	53964587	.	+	.	gene_id "XLOC_034310"; transcript_id "Novel_000498"; exon_number "1"; gene_type "
At_chr12	Cufflinks	exon	53964669	53964883	.	+	.	gene_id "XLOC_034310"; transcript_id "Novel_000498"; exon_number "2"; gene_type "
At_chr12	Cufflinks	exon	53964964	53965307	.	+	.	gene_id "XLOC_034310"; transcript_id "Novel_000498"; exon_number "3"; gene_type "
At_chr12	Cufflinks	exon	54353069	54353376	.	+	.	gene_id "XLOC_034364"; transcript_id "Novel_000499"; exon_number "1"; gene_type "
At_chr12	Cufflinks	exon	54354927	54355633	.	+	.	gene_id "XLOC_034364"; transcript_id "Novel_000499"; exon_number "2"; gene_type "
At_chr12	Cufflinks	exon	54355746	54355840	.	+	.	gene_id "XLOC_034364"; transcript_id "Novel_000499"; exon_number "3"; gene_type "
At_chr12	Cufflinks	exon	54356033	54356104	.	+	.	gene_id "XLOC_034364"; transcript_id "Novel_000499"; exon_number "4"; gene_type "
At_chr12	Cufflinks	exon	54356210	54356371	.	+	.	gene_id "XLOC_034364"; transcript_id "Novel_000499"; exon_number "5"; gene_type "
At_chr12	Cufflinks	exon	54356452	54356520	.	+	.	gene_id "XLOC_034364"; transcript_id "Novel_000499"; exon_number "6"; gene_type "
At_chr12	Cufflinks	exon	56852671	56853400	.	+	.	gene_id "XLOC_034483"; transcript_id "Novel_000500"; exon_number "1"; gene_type "
At_chr12	Cufflinks	exon	56853518	56853580	.	+	.	gene_id "XLOC_034483"; transcript_id "Novel_000500"; exon_number "2"; gene_type "
At_chr12	Cufflinks	exon	56862941	56863110	.	+	.	gene_id "XLOC_034483"; transcript_id "Novel_000500"; exon_number "3"; gene_type "
At_chr12	Cufflinks	exon	56863207	56863831	.	+	.	gene_id "XLOC_034483"; transcript_id "Novel_000500"; exon_number "4"; gene_type "
At_chr12	Cufflinks	exon	56852671	56853400	.	+	.	gene_id "XLOC_034483"; transcript_id "Novel_000501"; exon_number "1"; gene_type "
At_chr12	Cufflinks	exon	56853518	56853580	.	+	.	gene_id "XLOC_034483"; transcript_id "Novel_000501"; exon_number "2"; gene_type "
At_chr12	Cufflinks	exon	56862931	56863110	.	+	.	gene_id "XLOC_034483"; transcript_id "Novel_000501"; exon_number "3"; gene_type "
At_chr12	Cufflinks	exon	56863207	56863831	.	+	.	gene_id "XLOC_034483"; transcript_id "Novel_000501"; exon_number "4"; gene_type "
At_chr12	Cufflinks	exon	68239634	68241266	.	+	.	gene_id "XLOC_034934"; transcript_id "Novel_000502"; exon_number "1"; gene_type "
At_chr12	Cufflinks	exon	68241656	68241898	.	+	.	gene_id "XLOC_034934"; transcript_id "Novel_000502"; exon_number "2"; gene_type "
At_chr12	Cufflinks	exon	70120878	70121508	.	+	.	gene_id "XLOC_034999"; transcript_id "Novel_000503"; exon_number "1"; gene_type "
At_chr12	Cufflinks	exon	70122114	70122164	.	+	.	gene_id "XLOC_034999"; transcript_id "Novel_000503"; exon_number "2"; gene_type "
At_chr12	Cufflinks	exon	70122849	70123134	.	+	.	gene_id "XLOC_034999"; transcript_id "Novel_000503"; exon_number "3"; gene_type "
At_chr12	Cufflinks	exon	70120979	70122029	.	+	.	gene_id "XLOC_034999"; transcript_id "Novel_000504"; exon_number "1"; gene_type "
At_chr12	Cufflinks	exon	70122114	70122164	.	+	.	gene_id "XLOC_034999"; transcript_id "Novel_000504"; exon_number "2"; gene_type "
At_chr12	Cufflinks	exon	70122849	70123105	.	+	.	gene_id "XLOC_034999"; transcript_id "Novel_000504"; exon_number "3"; gene_type "

At_chr12	Cufflinks	exon	71878569	71878607	.	+	.	gene_id "XLOC_035052"; transcript_id "Novel_000505"; exon_number "1"; gene_type "
At_chr12	Cufflinks	exon	71879162	71879377	.	+	.	gene_id "XLOC_035052"; transcript_id "Novel_000505"; exon_number "2"; gene_type "
At_chr12	Cufflinks	exon	71883356	71883397	.	+	.	gene_id "XLOC_035052"; transcript_id "Novel_000506"; exon_number "1"; gene_type "
At_chr12	Cufflinks	exon	71883981	71884079	.	+	.	gene_id "XLOC_035052"; transcript_id "Novel_000506"; exon_number "2"; gene_type "
At_chr12	Cufflinks	exon	71885017	71885110	.	+	.	gene_id "XLOC_035052"; transcript_id "Novel_000506"; exon_number "3"; gene_type "
At_chr12	Cufflinks	exon	80697646	80697939	.	+	.	gene_id "XLOC_035364"; transcript_id "Novel_000507"; exon_number "1"; gene_type "
At_chr12	Cufflinks	exon	80698191	80699722	.	+	.	gene_id "XLOC_035364"; transcript_id "Novel_000507"; exon_number "2"; gene_type "
At_chr12	Cufflinks	exon	81717079	81717217	.	+	.	gene_id "XLOC_035412"; transcript_id "Novel_000508"; exon_number "1"; gene_type "
At_chr12	Cufflinks	exon	81717297	81717398	.	+	.	gene_id "XLOC_035412"; transcript_id "Novel_000508"; exon_number "2"; gene_type "
At_chr12	Cufflinks	exon	81717962	81718069	.	+	.	gene_id "XLOC_035412"; transcript_id "Novel_000508"; exon_number "3"; gene_type "
At_chr12	Cufflinks	exon	81718189	81718679	.	+	.	gene_id "XLOC_035412"; transcript_id "Novel_000508"; exon_number "4"; gene_type "
At_chr12	Cufflinks	exon	89660679	89661076	.	+	.	gene_id "XLOC_035718"; transcript_id "Novel_000509"; exon_number "1"; gene_type "
At_chr12	Cufflinks	exon	89661313	89661449	.	+	.	gene_id "XLOC_035718"; transcript_id "Novel_000509"; exon_number "2"; gene_type "
At_chr12	Cufflinks	exon	89661551	89661712	.	+	.	gene_id "XLOC_035718"; transcript_id "Novel_000509"; exon_number "3"; gene_type "
At_chr12	Cufflinks	exon	89661855	89662129	.	+	.	gene_id "XLOC_035718"; transcript_id "Novel_000509"; exon_number "4"; gene_type "
At_chr12	Cufflinks	exon	89662218	89662355	.	+	.	gene_id "XLOC_035718"; transcript_id "Novel_000509"; exon_number "5"; gene_type "
At_chr12	Cufflinks	exon	89662534	89662725	.	+	.	gene_id "XLOC_035718"; transcript_id "Novel_000509"; exon_number "6"; gene_type "
At_chr12	Cufflinks	exon	89662805	89662903	.	+	.	gene_id "XLOC_035718"; transcript_id "Novel_000509"; exon_number "7"; gene_type "
At_chr12	Cufflinks	exon	89663070	89663557	.	+	.	gene_id "XLOC_035718"; transcript_id "Novel_000509"; exon_number "8"; gene_type "
At_chr12	Cufflinks	exon	89662099	89662355	.	+	.	gene_id "XLOC_035718"; transcript_id "Novel_000510"; exon_number "1"; gene_type "
At_chr12	Cufflinks	exon	89662534	89662725	.	+	.	gene_id "XLOC_035718"; transcript_id "Novel_000510"; exon_number "2"; gene_type "
At_chr12	Cufflinks	exon	89662805	89662903	.	+	.	gene_id "XLOC_035718"; transcript_id "Novel_000510"; exon_number "3"; gene_type "
At_chr12	Cufflinks	exon	89663070	89663517	.	+	.	gene_id "XLOC_035718"; transcript_id "Novel_000510"; exon_number "4"; gene_type "
At_chr12	Cufflinks	exon	90130517	90130977	.	+	.	gene_id "XLOC_035731"; transcript_id "Novel_000511"; exon_number "1"; gene_type "
At_chr12	Cufflinks	exon	90131242	90131530	.	+	.	gene_id "XLOC_035731"; transcript_id "Novel_000511"; exon_number "2"; gene_type "
At_chr12	Cufflinks	exon	90131633	90131732	.	+	.	gene_id "XLOC_035731"; transcript_id "Novel_000511"; exon_number "3"; gene_type "
At_chr12	Cufflinks	exon	90131937	90132203	.	+	.	gene_id "XLOC_035731"; transcript_id "Novel_000511"; exon_number "4"; gene_type "
At_chr12	Cufflinks	exon	90132321	90132398	.	+	.	gene_id "XLOC_035731"; transcript_id "Novel_000511"; exon_number "5"; gene_type "
At_chr12	Cufflinks	exon	90132529	90132630	.	+	.	gene_id "XLOC_035731"; transcript_id "Novel_000511"; exon_number "6"; gene_type "
At_chr12	Cufflinks	exon	90132729	90132778	.	+	.	gene_id "XLOC_035731"; transcript_id "Novel_000511"; exon_number "7"; gene_type "
At_chr12	Cufflinks	exon	90132867	90132966	.	+	.	gene_id "XLOC_035731"; transcript_id "Novel_000511"; exon_number "8"; gene_type "
At_chr12	Cufflinks	exon	90133071	90133136	.	+	.	gene_id "XLOC_035731"; transcript_id "Novel_000511"; exon_number "9"; gene_type "
At_chr12	Cufflinks	exon	90133218	90133331	.	+	.	gene_id "XLOC_035731"; transcript_id "Novel_000511"; exon_number "10"; gene_type
At_chr12	Cufflinks	exon	90133531	90133666	.	+	.	gene_id "XLOC_035731"; transcript_id "Novel_000511"; exon_number "11"; gene_type
At_chr12	Cufflinks	exon	90134121	90134512	.	+	.	gene_id "XLOC_035731"; transcript_id "Novel_000511"; exon_number "12"; gene_type

At_chr12	Cufflinks	exon	90134783	90134857	.	+	.	gene_id "XLOC_035731"; transcript_id "Novel_000511"; exon_number "13"; gene_type
At_chr12	Cufflinks	exon	90134971	90135218	.	+	.	gene_id "XLOC_035731"; transcript_id "Novel_000511"; exon_number "14"; gene_type
At_chr12	Cufflinks	exon	90130715	90131530	.	+	.	gene_id "XLOC_035731"; transcript_id "Novel_000512"; exon_number "1"; gene_type "
At_chr12	Cufflinks	exon	90131633	90131732	.	+	.	gene_id "XLOC_035731"; transcript_id "Novel_000512"; exon_number "2"; gene_type "
At_chr12	Cufflinks	exon	90131937	90132203	.	+	.	gene_id "XLOC_035731"; transcript_id "Novel_000512"; exon_number "3"; gene_type "
At_chr12	Cufflinks	exon	90132321	90132398	.	+	.	gene_id "XLOC_035731"; transcript_id "Novel_000512"; exon_number "4"; gene_type "
At_chr12	Cufflinks	exon	90132529	90132630	.	+	.	gene_id "XLOC_035731"; transcript_id "Novel_000512"; exon_number "5"; gene_type "
At_chr12	Cufflinks	exon	90132729	90132778	.	+	.	gene_id "XLOC_035731"; transcript_id "Novel_000512"; exon_number "6"; gene_type "
At_chr12	Cufflinks	exon	90132867	90132966	.	+	.	gene_id "XLOC_035731"; transcript_id "Novel_000512"; exon_number "7"; gene_type "
At_chr12	Cufflinks	exon	90133071	90133136	.	+	.	gene_id "XLOC_035731"; transcript_id "Novel_000512"; exon_number "8"; gene_type "
At_chr12	Cufflinks	exon	90133218	90133331	.	+	.	gene_id "XLOC_035731"; transcript_id "Novel_000512"; exon_number "9"; gene_type "
At_chr12	Cufflinks	exon	90133531	90133666	.	+	.	gene_id "XLOC_035731"; transcript_id "Novel_000512"; exon_number "10"; gene_type
At_chr12	Cufflinks	exon	90134121	90134512	.	+	.	gene_id "XLOC_035731"; transcript_id "Novel_000512"; exon_number "11"; gene_type
At_chr12	Cufflinks	exon	90134783	90134857	.	+	.	gene_id "XLOC_035731"; transcript_id "Novel_000512"; exon_number "12"; gene_type
At_chr12	Cufflinks	exon	90134971	90135346	.	+	.	gene_id "XLOC_035731"; transcript_id "Novel_000512"; exon_number "13"; gene_type
At_chr12	Cufflinks	exon	90131926	90132203	.	+	.	gene_id "XLOC_035731"; transcript_id "Novel_000513"; exon_number "1"; gene_type "
At_chr12	Cufflinks	exon	90132321	90132398	.	+	.	gene_id "XLOC_035731"; transcript_id "Novel_000513"; exon_number "2"; gene_type "
At_chr12	Cufflinks	exon	90132529	90132630	.	+	.	gene_id "XLOC_035731"; transcript_id "Novel_000513"; exon_number "3"; gene_type "
At_chr12	Cufflinks	exon	90132729	90132778	.	+	.	gene_id "XLOC_035731"; transcript_id "Novel_000513"; exon_number "4"; gene_type "
At_chr12	Cufflinks	exon	90132880	90132966	.	+	.	gene_id "XLOC_035731"; transcript_id "Novel_000513"; exon_number "5"; gene_type "
At_chr12	Cufflinks	exon	90133071	90133136	.	+	.	gene_id "XLOC_035731"; transcript_id "Novel_000513"; exon_number "6"; gene_type "
At_chr12	Cufflinks	exon	90133218	90133331	.	+	.	gene_id "XLOC_035731"; transcript_id "Novel_000513"; exon_number "7"; gene_type "
At_chr12	Cufflinks	exon	90133531	90133666	.	+	.	gene_id "XLOC_035731"; transcript_id "Novel_000513"; exon_number "8"; gene_type "
At_chr12	Cufflinks	exon	90134121	90134512	.	+	.	gene_id "XLOC_035731"; transcript_id "Novel_000513"; exon_number "9"; gene_type "
At_chr12	Cufflinks	exon	90134783	90134857	.	+	.	gene_id "XLOC_035731"; transcript_id "Novel_000513"; exon_number "10"; gene_type
At_chr12	Cufflinks	exon	90134971	90135252	.	+	.	gene_id "XLOC_035731"; transcript_id "Novel_000513"; exon_number "11"; gene_type
At_chr12	Cufflinks	exon	93942152	93942231	.	+	.	gene_id "XLOC_035820"; transcript_id "Novel_000514"; exon_number "1"; gene_type "
At_chr12	Cufflinks	exon	93944574	93944637	.	+	.	gene_id "XLOC_035820"; transcript_id "Novel_000514"; exon_number "2"; gene_type "
At_chr12	Cufflinks	exon	93944933	93944997	.	+	.	gene_id "XLOC_035820"; transcript_id "Novel_000514"; exon_number "3"; gene_type "
At_chr12	Cufflinks	exon	93945468	93945567	.	+	.	gene_id "XLOC_035820"; transcript_id "Novel_000514"; exon_number "4"; gene_type "
At_chr12	Cufflinks	exon	93945668	93945856	.	+	.	gene_id "XLOC_035820"; transcript_id "Novel_000514"; exon_number "5"; gene_type "
At_chr12	Cufflinks	exon	95041859	95042386	.	+	.	gene_id "XLOC_035931"; transcript_id "Novel_000515"; exon_number "1"; gene_type "
At_chr12	Cufflinks	exon	95042460	95045709	.	+	.	gene_id "XLOC_035931"; transcript_id "Novel_000515"; exon_number "2"; gene_type "
At_chr12	Cufflinks	exon	96374357	96374526	.	+	.	gene_id "XLOC_035999"; transcript_id "Novel_000516"; exon_number "1"; gene_type "
At_chr12	Cufflinks	exon	96374830	96375269	.	+	.	gene_id "XLOC_035999"; transcript_id "Novel_000516"; exon_number "2"; gene_type "

At_chr12	Cufflinks	exon	96374357	96374526	.	+	.	gene_id "XLOC_035999"; transcript_id "Novel_000517"; exon_number "1"; gene_type "
At_chr12	Cufflinks	exon	96374845	96375269	.	+	.	gene_id "XLOC_035999"; transcript_id "Novel_000517"; exon_number "2"; gene_type "
At_chr12	Cufflinks	exon	98721096	98721928	.	+	.	gene_id "XLOC_036089"; transcript_id "Novel_000518"; exon_number "1"; gene_type "
At_chr12	Cufflinks	exon	99946201	99947205	.	+	.	gene_id "XLOC_036149"; transcript_id "Novel_000519"; exon_number "1"; gene_type "
At_chr12	Cufflinks	exon	99947318	99947357	.	+	.	gene_id "XLOC_036149"; transcript_id "Novel_000519"; exon_number "2"; gene_type "
At_chr12	Cufflinks	exon	99948228	99948817	.	+	.	gene_id "XLOC_036149"; transcript_id "Novel_000519"; exon_number "3"; gene_type "
At_chr12	Cufflinks	exon	7175969	7176123	.	-	.	gene_id "XLOC_036425"; transcript_id "Novel_000520"; exon_number "1"; gene_type "
At_chr12	Cufflinks	exon	7176208	7176284	.	-	.	gene_id "XLOC_036425"; transcript_id "Novel_000520"; exon_number "2"; gene_type "
At_chr12	Cufflinks	exon	15389527	15389922	.	-	.	gene_id "XLOC_036713"; transcript_id "Novel_000521"; exon_number "1"; gene_type "
At_chr12	Cufflinks	exon	15390183	15390326	.	-	.	gene_id "XLOC_036713"; transcript_id "Novel_000521"; exon_number "2"; gene_type "
At_chr12	Cufflinks	exon	15390439	15390509	.	-	.	gene_id "XLOC_036713"; transcript_id "Novel_000521"; exon_number "3"; gene_type "
At_chr12	Cufflinks	exon	15391853	15391952	.	-	.	gene_id "XLOC_036713"; transcript_id "Novel_000521"; exon_number "4"; gene_type "
At_chr12	Cufflinks	exon	15392551	15392581	.	-	.	gene_id "XLOC_036713"; transcript_id "Novel_000521"; exon_number "5"; gene_type "
At_chr12	Cufflinks	exon	15392742	15392839	.	-	.	gene_id "XLOC_036713"; transcript_id "Novel_000521"; exon_number "6"; gene_type "
At_chr12	Cufflinks	exon	15393522	15393969	.	-	.	gene_id "XLOC_036713"; transcript_id "Novel_000521"; exon_number "7"; gene_type "
At_chr12	Cufflinks	exon	15390369	15392581	.	-	.	gene_id "XLOC_036713"; transcript_id "Novel_000522"; exon_number "1"; gene_type "
At_chr12	Cufflinks	exon	15392742	15392839	.	-	.	gene_id "XLOC_036713"; transcript_id "Novel_000522"; exon_number "2"; gene_type "
At_chr12	Cufflinks	exon	15393522	15393969	.	-	.	gene_id "XLOC_036713"; transcript_id "Novel_000522"; exon_number "3"; gene_type "
At_chr12	Cufflinks	exon	16827168	16827407	.	-	.	gene_id "XLOC_036760"; transcript_id "Novel_000523"; exon_number "1"; gene_type "
At_chr12	Cufflinks	exon	29489861	29490190	.	-	.	gene_id "XLOC_037140"; transcript_id "Novel_000524"; exon_number "1"; gene_type "
At_chr12	Cufflinks	exon	29491180	29491346	.	-	.	gene_id "XLOC_037140"; transcript_id "Novel_000524"; exon_number "2"; gene_type "
At_chr12	Cufflinks	exon	29491901	29492267	.	-	.	gene_id "XLOC_037140"; transcript_id "Novel_000524"; exon_number "3"; gene_type "
At_chr12	Cufflinks	exon	29489861	29490190	.	-	.	gene_id "XLOC_037140"; transcript_id "Novel_000525"; exon_number "1"; gene_type "
At_chr12	Cufflinks	exon	29491180	29492267	.	-	.	gene_id "XLOC_037140"; transcript_id "Novel_000525"; exon_number "2"; gene_type "
At_chr12	Cufflinks	exon	42219479	42220079	.	-	.	gene_id "XLOC_037573"; transcript_id "Novel_000526"; exon_number "1"; gene_type "
At_chr12	Cufflinks	exon	42220412	42220573	.	-	.	gene_id "XLOC_037573"; transcript_id "Novel_000526"; exon_number "2"; gene_type "
At_chr12	Cufflinks	exon	42220784	42221087	.	-	.	gene_id "XLOC_037573"; transcript_id "Novel_000526"; exon_number "3"; gene_type "
At_chr12	Cufflinks	exon	42244663	42245395	.	-	.	gene_id "XLOC_037581"; transcript_id "Novel_000527"; exon_number "1"; gene_type "
At_chr12	Cufflinks	exon	42246100	42246262	.	-	.	gene_id "XLOC_037581"; transcript_id "Novel_000527"; exon_number "2"; gene_type "
At_chr12	Cufflinks	exon	43834327	43834397	.	-	.	gene_id "XLOC_037765"; transcript_id "Novel_000528"; exon_number "1"; gene_type "
At_chr12	Cufflinks	exon	43834479	43834607	.	-	.	gene_id "XLOC_037765"; transcript_id "Novel_000528"; exon_number "2"; gene_type "
At_chr12	Cufflinks	exon	44258179	44258605	.	-	.	gene_id "XLOC_037809"; transcript_id "Novel_000529"; exon_number "1"; gene_type "
At_chr12	Cufflinks	exon	44260294	44260524	.	-	.	gene_id "XLOC_037809"; transcript_id "Novel_000529"; exon_number "2"; gene_type "
At_chr12	Cufflinks	exon	44465282	44466003	.	-	.	gene_id "XLOC_037840"; transcript_id "Novel_000530"; exon_number "1"; gene_type "
At_chr12	Cufflinks	exon	44466115	44466213	.	-	.	gene_id "XLOC_037840"; transcript_id "Novel_000530"; exon_number "2"; gene_type "

At_chr12	Cufflinks	exon	45552836	45554526	.	-	.	gene_id "XLOC_037981"; transcript_id "Novel_000531"; exon_number "1"; gene_type "
At_chr12	Cufflinks	exon	45555515	45555684	.	-	.	gene_id "XLOC_037981"; transcript_id "Novel_000531"; exon_number "2"; gene_type "
At_chr12	Cufflinks	exon	45552844	45553434	.	-	.	gene_id "XLOC_037981"; transcript_id "Novel_000532"; exon_number "1"; gene_type "
At_chr12	Cufflinks	exon	45553565	45554526	.	-	.	gene_id "XLOC_037981"; transcript_id "Novel_000532"; exon_number "2"; gene_type "
At_chr12	Cufflinks	exon	45555515	45555692	.	-	.	gene_id "XLOC_037981"; transcript_id "Novel_000532"; exon_number "3"; gene_type "
At_chr12	Cufflinks	exon	45552967	45553434	.	-	.	gene_id "XLOC_037981"; transcript_id "Novel_000533"; exon_number "1"; gene_type "
At_chr12	Cufflinks	exon	45553536	45554526	.	-	.	gene_id "XLOC_037981"; transcript_id "Novel_000533"; exon_number "2"; gene_type "
At_chr12	Cufflinks	exon	45555515	45555711	.	-	.	gene_id "XLOC_037981"; transcript_id "Novel_000533"; exon_number "3"; gene_type "
At_chr12	Cufflinks	exon	52801013	52801387	.	-	.	gene_id "XLOC_038459"; transcript_id "Novel_000534"; exon_number "1"; gene_type "
At_chr12	Cufflinks	exon	52801492	52801570	.	-	.	gene_id "XLOC_038459"; transcript_id "Novel_000534"; exon_number "2"; gene_type "
At_chr12	Cufflinks	exon	52801749	52801791	.	-	.	gene_id "XLOC_038459"; transcript_id "Novel_000534"; exon_number "3"; gene_type "
At_chr12	Cufflinks	exon	52802102	52802317	.	-	.	gene_id "XLOC_038459"; transcript_id "Novel_000534"; exon_number "4"; gene_type "
At_chr12	Cufflinks	exon	52802654	52802746	.	-	.	gene_id "XLOC_038459"; transcript_id "Novel_000534"; exon_number "5"; gene_type "
At_chr12	Cufflinks	exon	52802934	52802973	.	-	.	gene_id "XLOC_038459"; transcript_id "Novel_000534"; exon_number "6"; gene_type "
At_chr12	Cufflinks	exon	52803075	52803131	.	-	.	gene_id "XLOC_038459"; transcript_id "Novel_000534"; exon_number "7"; gene_type "
At_chr12	Cufflinks	exon	52804058	52804126	.	-	.	gene_id "XLOC_038459"; transcript_id "Novel_000534"; exon_number "8"; gene_type "
At_chr12	Cufflinks	exon	52804216	52804702	.	-	.	gene_id "XLOC_038459"; transcript_id "Novel_000534"; exon_number "9"; gene_type "
At_chr12	Cufflinks	exon	52802880	52803131	.	-	.	gene_id "XLOC_038459"; transcript_id "Novel_000535"; exon_number "1"; gene_type "
At_chr12	Cufflinks	exon	52804058	52804126	.	-	.	gene_id "XLOC_038459"; transcript_id "Novel_000535"; exon_number "2"; gene_type "
At_chr12	Cufflinks	exon	52804216	52804575	.	-	.	gene_id "XLOC_038459"; transcript_id "Novel_000535"; exon_number "3"; gene_type "
At_chr12	Cufflinks	exon	53857231	53857513	.	-	.	gene_id "XLOC_038555"; transcript_id "Novel_000536"; exon_number "1"; gene_type "
At_chr12	Cufflinks	exon	54081694	54083371	.	-	.	gene_id "XLOC_038586"; transcript_id "Novel_000537"; exon_number "1"; gene_type "
At_chr12	Cufflinks	exon	54083601	54083673	.	-	.	gene_id "XLOC_038586"; transcript_id "Novel_000537"; exon_number "2"; gene_type "
At_chr12	Cufflinks	exon	54083982	54084077	.	-	.	gene_id "XLOC_038586"; transcript_id "Novel_000537"; exon_number "3"; gene_type "
At_chr12	Cufflinks	exon	54084289	54084352	.	-	.	gene_id "XLOC_038586"; transcript_id "Novel_000537"; exon_number "4"; gene_type "
At_chr12	Cufflinks	exon	54084665	54084716	.	-	.	gene_id "XLOC_038586"; transcript_id "Novel_000537"; exon_number "5"; gene_type "
At_chr12	Cufflinks	exon	54085190	54085287	.	-	.	gene_id "XLOC_038586"; transcript_id "Novel_000537"; exon_number "6"; gene_type "
At_chr12	Cufflinks	exon	54085387	54085445	.	-	.	gene_id "XLOC_038586"; transcript_id "Novel_000537"; exon_number "7"; gene_type "
At_chr12	Cufflinks	exon	54085707	54085973	.	-	.	gene_id "XLOC_038586"; transcript_id "Novel_000537"; exon_number "8"; gene_type "
At_chr12	Cufflinks	exon	65288598	65288706	.	-	.	gene_id "XLOC_039001"; transcript_id "Novel_000538"; exon_number "1"; gene_type "
At_chr12	Cufflinks	exon	65288918	65289126	.	-	.	gene_id "XLOC_039001"; transcript_id "Novel_000538"; exon_number "2"; gene_type "
At_chr12	Cufflinks	exon	81578751	81579171	.	-	.	gene_id "XLOC_039521"; transcript_id "Novel_000539"; exon_number "1"; gene_type "
At_chr12	Cufflinks	exon	81579246	81579585	.	-	.	gene_id "XLOC_039521"; transcript_id "Novel_000539"; exon_number "2"; gene_type "
At_chr12	Cufflinks	exon	81582445	81582821	.	-	.	gene_id "XLOC_039522"; transcript_id "Novel_000540"; exon_number "1"; gene_type "
At_chr12	Cufflinks	exon	81583482	81584174	.	-	.	gene_id "XLOC_039522"; transcript_id "Novel_000540"; exon_number "2"; gene_type "

At_chr12	Cufflinks	exon	82132948	82133404	.	-	.	gene_id "XLOC_039551"; transcript_id "Novel_000541"; exon_number "1"; gene_type "
At_chr12	Cufflinks	exon	82133519	82134750	.	-	.	gene_id "XLOC_039551"; transcript_id "Novel_000541"; exon_number "2"; gene_type "
At_chr12	Cufflinks	exon	90208924	90209154	.	-	.	gene_id "XLOC_039878"; transcript_id "Novel_000542"; exon_number "1"; gene_type "
At_chr12	Cufflinks	exon	91111457	91114890	.	-	.	gene_id "XLOC_039900"; transcript_id "Novel_000543"; exon_number "1"; gene_type "
At_chr12	Cufflinks	exon	91115301	91116557	.	-	.	gene_id "XLOC_039900"; transcript_id "Novel_000543"; exon_number "2"; gene_type "
At_chr12	Cufflinks	exon	91111458	91114303	.	-	.	gene_id "XLOC_039900"; transcript_id "Novel_000544"; exon_number "1"; gene_type "
At_chr12	Cufflinks	exon	91114834	91114890	.	-	.	gene_id "XLOC_039900"; transcript_id "Novel_000544"; exon_number "2"; gene_type "
At_chr12	Cufflinks	exon	91116940	91117290	.	-	.	gene_id "XLOC_039900"; transcript_id "Novel_000544"; exon_number "3"; gene_type "
At_chr12	Cufflinks	exon	91117372	91117468	.	-	.	gene_id "XLOC_039900"; transcript_id "Novel_000544"; exon_number "4"; gene_type "
At_chr12	Cufflinks	exon	91111483	91114890	.	-	.	gene_id "XLOC_039900"; transcript_id "Novel_000545"; exon_number "1"; gene_type "
At_chr12	Cufflinks	exon	91117194	91117290	.	-	.	gene_id "XLOC_039900"; transcript_id "Novel_000545"; exon_number "2"; gene_type "
At_chr12	Cufflinks	exon	91117372	91117444	.	-	.	gene_id "XLOC_039900"; transcript_id "Novel_000545"; exon_number "3"; gene_type "
At_chr12	Cufflinks	exon	94200408	94200498	.	-	.	gene_id "XLOC_040034"; transcript_id "Novel_000546"; exon_number "1"; gene_type "
At_chr12	Cufflinks	exon	94200621	94201164	.	-	.	gene_id "XLOC_040034"; transcript_id "Novel_000546"; exon_number "2"; gene_type "
At_chr12	Cufflinks	exon	94200408	94200498	.	-	.	gene_id "XLOC_040034"; transcript_id "Novel_000547"; exon_number "1"; gene_type "
At_chr12	Cufflinks	exon	94200621	94200713	.	-	.	gene_id "XLOC_040034"; transcript_id "Novel_000547"; exon_number "2"; gene_type "
At_chr12	Cufflinks	exon	94201286	94201460	.	-	.	gene_id "XLOC_040034"; transcript_id "Novel_000547"; exon_number "3"; gene_type "
At_chr12	Cufflinks	exon	94200413	94200498	.	-	.	gene_id "XLOC_040034"; transcript_id "Novel_000548"; exon_number "1"; gene_type "
At_chr12	Cufflinks	exon	94200621	94200713	.	-	.	gene_id "XLOC_040034"; transcript_id "Novel_000548"; exon_number "2"; gene_type "
At_chr12	Cufflinks	exon	94201286	94201360	.	-	.	gene_id "XLOC_040034"; transcript_id "Novel_000548"; exon_number "3"; gene_type "
At_chr12	Cufflinks	exon	94201450	94202496	.	-	.	gene_id "XLOC_040034"; transcript_id "Novel_000548"; exon_number "4"; gene_type "
At_chr12	Cufflinks	exon	94202547	94202728	.	-	.	gene_id "XLOC_040034"; transcript_id "Novel_000549"; exon_number "1"; gene_type "
At_chr12	Cufflinks	exon	94203725	94203877	.	-	.	gene_id "XLOC_040034"; transcript_id "Novel_000549"; exon_number "2"; gene_type "
At_chr12	Cufflinks	exon	94204478	94204740	.	-	.	gene_id "XLOC_040034"; transcript_id "Novel_000549"; exon_number "3"; gene_type "
At_chr13	Cufflinks	exon	11179021	11179289	.	+	.	gene_id "XLOC_040854"; transcript_id "Novel_000550"; exon_number "1"; gene_type "
At_chr13	Cufflinks	exon	11180220	11180268	.	+	.	gene_id "XLOC_040854"; transcript_id "Novel_000550"; exon_number "2"; gene_type "
At_chr13	Cufflinks	exon	17126813	17127314	.	+	.	gene_id "XLOC_041282"; transcript_id "Novel_000551"; exon_number "1"; gene_type "
At_chr13	Cufflinks	exon	17127680	17127829	.	+	.	gene_id "XLOC_041282"; transcript_id "Novel_000551"; exon_number "2"; gene_type "
At_chr13	Cufflinks	exon	17127906	17128029	.	+	.	gene_id "XLOC_041282"; transcript_id "Novel_000551"; exon_number "3"; gene_type "
At_chr13	Cufflinks	exon	17128145	17128245	.	+	.	gene_id "XLOC_041282"; transcript_id "Novel_000551"; exon_number "4"; gene_type "
At_chr13	Cufflinks	exon	17130839	17130931	.	+	.	gene_id "XLOC_041282"; transcript_id "Novel_000551"; exon_number "5"; gene_type "
At_chr13	Cufflinks	exon	17131022	17131488	.	+	.	gene_id "XLOC_041282"; transcript_id "Novel_000551"; exon_number "6"; gene_type "
At_chr13	Cufflinks	exon	19285671	19285853	.	+	.	gene_id "XLOC_041455"; transcript_id "Novel_000552"; exon_number "1"; gene_type "
At_chr13	Cufflinks	exon	19286878	19286962	.	+	.	gene_id "XLOC_041455"; transcript_id "Novel_000552"; exon_number "2"; gene_type "
At_chr13	Cufflinks	exon	19287302	19288639	.	+	.	gene_id "XLOC_041455"; transcript_id "Novel_000552"; exon_number "3"; gene_type "

At_chr13	Cufflinks	exon	19285676	19285853	.	+	.	gene_id "XLOC_041455"; transcript_id "Novel_000553"; exon_number "1"; gene_type "
At_chr13	Cufflinks	exon	19286878	19287045	.	+	.	gene_id "XLOC_041455"; transcript_id "Novel_000553"; exon_number "2"; gene_type "
At_chr13	Cufflinks	exon	19631660	19632004	.	+	.	gene_id "XLOC_041471"; transcript_id "Novel_000554"; exon_number "1"; gene_type "
At_chr13	Cufflinks	exon	19632103	19632170	.	+	.	gene_id "XLOC_041471"; transcript_id "Novel_000554"; exon_number "2"; gene_type "
At_chr13	Cufflinks	exon	19632254	19632305	.	+	.	gene_id "XLOC_041471"; transcript_id "Novel_000554"; exon_number "3"; gene_type "
At_chr13	Cufflinks	exon	19632752	19635960	.	+	.	gene_id "XLOC_041471"; transcript_id "Novel_000554"; exon_number "4"; gene_type "
At_chr13	Cufflinks	exon	22932537	22932787	.	+	.	gene_id "XLOC_041776"; transcript_id "Novel_000555"; exon_number "1"; gene_type "
At_chr13	Cufflinks	exon	22933524	22933745	.	+	.	gene_id "XLOC_041776"; transcript_id "Novel_000555"; exon_number "2"; gene_type "
At_chr13	Cufflinks	exon	22933926	22934000	.	+	.	gene_id "XLOC_041776"; transcript_id "Novel_000555"; exon_number "3"; gene_type "
At_chr13	Cufflinks	exon	22934115	22934689	.	+	.	gene_id "XLOC_041776"; transcript_id "Novel_000555"; exon_number "4"; gene_type "
At_chr13	Cufflinks	exon	22942783	22942815	.	+	.	gene_id "XLOC_041776"; transcript_id "Novel_000556"; exon_number "1"; gene_type "
At_chr13	Cufflinks	exon	22943006	22943098	.	+	.	gene_id "XLOC_041776"; transcript_id "Novel_000556"; exon_number "2"; gene_type "
At_chr13	Cufflinks	exon	22943433	22943487	.	+	.	gene_id "XLOC_041776"; transcript_id "Novel_000556"; exon_number "3"; gene_type "
At_chr13	Cufflinks	exon	22944049	22944125	.	+	.	gene_id "XLOC_041776"; transcript_id "Novel_000556"; exon_number "4"; gene_type "
At_chr13	Cufflinks	exon	22944212	22944283	.	+	.	gene_id "XLOC_041776"; transcript_id "Novel_000556"; exon_number "5"; gene_type "
At_chr13	Cufflinks	exon	22943031	22943098	.	+	.	gene_id "XLOC_041776"; transcript_id "Novel_000557"; exon_number "1"; gene_type "
At_chr13	Cufflinks	exon	22943433	22943487	.	+	.	gene_id "XLOC_041776"; transcript_id "Novel_000557"; exon_number "2"; gene_type "
At_chr13	Cufflinks	exon	22944049	22944102	.	+	.	gene_id "XLOC_041776"; transcript_id "Novel_000557"; exon_number "3"; gene_type "
At_chr13	Cufflinks	exon	22944212	22944249	.	+	.	gene_id "XLOC_041776"; transcript_id "Novel_000557"; exon_number "4"; gene_type "
At_chr13	Cufflinks	exon	22944994	22945053	.	+	.	gene_id "XLOC_041784"; transcript_id "Novel_000558"; exon_number "1"; gene_type "
At_chr13	Cufflinks	exon	22945200	22945277	.	+	.	gene_id "XLOC_041784"; transcript_id "Novel_000558"; exon_number "2"; gene_type "
At_chr13	Cufflinks	exon	22945403	22945630	.	+	.	gene_id "XLOC_041784"; transcript_id "Novel_000558"; exon_number "3"; gene_type "
At_chr13	Cufflinks	exon	22950659	22950864	.	+	.	gene_id "XLOC_041784"; transcript_id "Novel_000559"; exon_number "1"; gene_type "
At_chr13	Cufflinks	exon	22951102	22951253	.	+	.	gene_id "XLOC_041784"; transcript_id "Novel_000559"; exon_number "2"; gene_type "
At_chr13	Cufflinks	exon	22951363	22951857	.	+	.	gene_id "XLOC_041784"; transcript_id "Novel_000559"; exon_number "3"; gene_type "
At_chr13	Cufflinks	exon	22951039	22951253	.	+	.	gene_id "XLOC_041784"; transcript_id "Novel_000560"; exon_number "1"; gene_type "
At_chr13	Cufflinks	exon	22951363	22951494	.	+	.	gene_id "XLOC_041784"; transcript_id "Novel_000560"; exon_number "2"; gene_type "
At_chr13	Cufflinks	exon	23025476	23025639	.	+	.	gene_id "XLOC_041797"; transcript_id "Novel_000561"; exon_number "1"; gene_type "
At_chr13	Cufflinks	exon	23026035	23026402	.	+	.	gene_id "XLOC_041797"; transcript_id "Novel_000561"; exon_number "2"; gene_type "
At_chr13	Cufflinks	exon	23027377	23027500	.	+	.	gene_id "XLOC_041797"; transcript_id "Novel_000561"; exon_number "3"; gene_type "
At_chr13	Cufflinks	exon	23028095	23029381	.	+	.	gene_id "XLOC_041797"; transcript_id "Novel_000561"; exon_number "4"; gene_type "
At_chr13	Cufflinks	exon	23025479	23025639	.	+	.	gene_id "XLOC_041797"; transcript_id "Novel_000562"; exon_number "1"; gene_type "
At_chr13	Cufflinks	exon	23026035	23027216	.	+	.	gene_id "XLOC_041797"; transcript_id "Novel_000562"; exon_number "2"; gene_type "
At_chr13	Cufflinks	exon	23025513	23025639	.	+	.	gene_id "XLOC_041797"; transcript_id "Novel_000563"; exon_number "1"; gene_type "
At_chr13	Cufflinks	exon	23026035	23026402	.	+	.	gene_id "XLOC_041797"; transcript_id "Novel_000563"; exon_number "2"; gene_type "

At_chr13	Cufflinks	exon	23027377	23027500	.	+	.	gene_id "XLOC_041797"; transcript_id "Novel_000563"; exon_number "3"; gene_type "
At_chr13	Cufflinks	exon	23028017	23028220	.	+	.	gene_id "XLOC_041797"; transcript_id "Novel_000563"; exon_number "4"; gene_type "
At_chr13	Cufflinks	exon	23029417	23029678	.	+	.	gene_id "XLOC_041797"; transcript_id "Novel_000563"; exon_number "5"; gene_type "
At_chr13	Cufflinks	exon	23027317	23027500	.	+	.	gene_id "XLOC_041797"; transcript_id "Novel_000564"; exon_number "1"; gene_type "
At_chr13	Cufflinks	exon	23028095	23028220	.	+	.	gene_id "XLOC_041797"; transcript_id "Novel_000564"; exon_number "2"; gene_type "
At_chr13	Cufflinks	exon	23029417	23029588	.	+	.	gene_id "XLOC_041797"; transcript_id "Novel_000564"; exon_number "3"; gene_type "
At_chr13	Cufflinks	exon	25595196	25595474	.	+	.	gene_id "XLOC_042162"; transcript_id "Novel_000565"; exon_number "1"; gene_type "
At_chr13	Cufflinks	exon	25595580	25599060	.	+	.	gene_id "XLOC_042162"; transcript_id "Novel_000565"; exon_number "2"; gene_type "
At_chr13	Cufflinks	exon	26053474	26053948	.	+	.	gene_id "XLOC_042221"; transcript_id "Novel_000566"; exon_number "1"; gene_type "
At_chr13	Cufflinks	exon	26054034	26054142	.	+	.	gene_id "XLOC_042221"; transcript_id "Novel_000566"; exon_number "2"; gene_type "
At_chr13	Cufflinks	exon	26054500	26054596	.	+	.	gene_id "XLOC_042221"; transcript_id "Novel_000566"; exon_number "3"; gene_type "
At_chr13	Cufflinks	exon	26225312	26225379	.	+	.	gene_id "XLOC_042237"; transcript_id "Novel_000567"; exon_number "1"; gene_type "
At_chr13	Cufflinks	exon	26225955	26226300	.	+	.	gene_id "XLOC_042237"; transcript_id "Novel_000567"; exon_number "2"; gene_type "
At_chr13	Cufflinks	exon	26226393	26228680	.	+	.	gene_id "XLOC_042237"; transcript_id "Novel_000567"; exon_number "3"; gene_type "
At_chr13	Cufflinks	exon	26225313	26225379	.	+	.	gene_id "XLOC_042237"; transcript_id "Novel_000568"; exon_number "1"; gene_type "
At_chr13	Cufflinks	exon	26225955	26226304	.	+	.	gene_id "XLOC_042237"; transcript_id "Novel_000568"; exon_number "2"; gene_type "
At_chr13	Cufflinks	exon	26226393	26229169	.	+	.	gene_id "XLOC_042237"; transcript_id "Novel_000568"; exon_number "3"; gene_type "
At_chr13	Cufflinks	exon	26225524	26225822	.	+	.	gene_id "XLOC_042237"; transcript_id "Novel_000569"; exon_number "1"; gene_type "
At_chr13	Cufflinks	exon	26225955	26226304	.	+	.	gene_id "XLOC_042237"; transcript_id "Novel_000569"; exon_number "2"; gene_type "
At_chr13	Cufflinks	exon	26226393	26228869	.	+	.	gene_id "XLOC_042237"; transcript_id "Novel_000569"; exon_number "3"; gene_type "
At_chr13	Cufflinks	exon	26540799	26541505	.	+	.	gene_id "XLOC_042256"; transcript_id "Novel_000570"; exon_number "1"; gene_type "
At_chr13	Cufflinks	exon	26541693	26541918	.	+	.	gene_id "XLOC_042256"; transcript_id "Novel_000570"; exon_number "2"; gene_type "
At_chr13	Cufflinks	exon	26565340	26565447	.	+	.	gene_id "XLOC_042257"; transcript_id "Novel_000571"; exon_number "1"; gene_type "
At_chr13	Cufflinks	exon	26566259	26566398	.	+	.	gene_id "XLOC_042257"; transcript_id "Novel_000571"; exon_number "2"; gene_type "
At_chr13	Cufflinks	exon	26566537	26566652	.	+	.	gene_id "XLOC_042257"; transcript_id "Novel_000571"; exon_number "3"; gene_type "
At_chr13	Cufflinks	exon	26566911	26569457	.	+	.	gene_id "XLOC_042257"; transcript_id "Novel_000571"; exon_number "4"; gene_type "
At_chr13	Cufflinks	exon	26565370	26565447	.	+	.	gene_id "XLOC_042257"; transcript_id "Novel_000572"; exon_number "1"; gene_type "
At_chr13	Cufflinks	exon	26566259	26566444	.	+	.	gene_id "XLOC_042257"; transcript_id "Novel_000572"; exon_number "2"; gene_type "
At_chr13	Cufflinks	exon	26566537	26566652	.	+	.	gene_id "XLOC_042257"; transcript_id "Novel_000572"; exon_number "3"; gene_type "
At_chr13	Cufflinks	exon	26566911	26566995	.	+	.	gene_id "XLOC_042257"; transcript_id "Novel_000572"; exon_number "4"; gene_type "
At_chr13	Cufflinks	exon	26569033	26569759	.	+	.	gene_id "XLOC_042257"; transcript_id "Novel_000572"; exon_number "5"; gene_type "
At_chr13	Cufflinks	exon	26565385	26565447	.	+	.	gene_id "XLOC_042257"; transcript_id "Novel_000573"; exon_number "1"; gene_type "
At_chr13	Cufflinks	exon	26565991	26566652	.	+	.	gene_id "XLOC_042257"; transcript_id "Novel_000573"; exon_number "2"; gene_type "
At_chr13	Cufflinks	exon	26566911	26569759	.	+	.	gene_id "XLOC_042257"; transcript_id "Novel_000573"; exon_number "3"; gene_type "
At_chr13	Cufflinks	exon	27454238	27454628	.	+	.	gene_id "XLOC_042295"; transcript_id "Novel_000574"; exon_number "1"; gene_type "

At_chr13	Cufflinks	exon	27455367	27455467	.	+	.	gene_id "XLOC_042295"; transcript_id "Novel_000574"; exon_number "2"; gene_type "
At_chr13	Cufflinks	exon	27456427	27457008	.	+	.	gene_id "XLOC_042295"; transcript_id "Novel_000574"; exon_number "3"; gene_type "
At_chr13	Cufflinks	exon	31763483	31763503	.	+	.	gene_id "XLOC_042469"; transcript_id "Novel_000575"; exon_number "1"; gene_type "
At_chr13	Cufflinks	exon	31763767	31763826	.	+	.	gene_id "XLOC_042469"; transcript_id "Novel_000575"; exon_number "2"; gene_type "
At_chr13	Cufflinks	exon	31764719	31764823	.	+	.	gene_id "XLOC_042469"; transcript_id "Novel_000575"; exon_number "3"; gene_type "
At_chr13	Cufflinks	exon	31765200	31765229	.	+	.	gene_id "XLOC_042469"; transcript_id "Novel_000575"; exon_number "4"; gene_type "
At_chr13	Cufflinks	exon	31765820	31765896	.	+	.	gene_id "XLOC_042469"; transcript_id "Novel_000575"; exon_number "5"; gene_type "
At_chr13	Cufflinks	exon	31766356	31766574	.	+	.	gene_id "XLOC_042469"; transcript_id "Novel_000575"; exon_number "6"; gene_type "
At_chr13	Cufflinks	exon	33283656	33283869	.	+	.	gene_id "XLOC_042540"; transcript_id "Novel_000576"; exon_number "1"; gene_type "
At_chr13	Cufflinks	exon	33306507	33306637	.	+	.	gene_id "XLOC_042542"; transcript_id "Novel_000577"; exon_number "1"; gene_type "
At_chr13	Cufflinks	exon	33309019	33309155	.	+	.	gene_id "XLOC_042542"; transcript_id "Novel_000577"; exon_number "2"; gene_type "
At_chr13	Cufflinks	exon	33309645	33310227	.	+	.	gene_id "XLOC_042542"; transcript_id "Novel_000577"; exon_number "3"; gene_type "
At_chr13	Cufflinks	exon	33310955	33311052	.	+	.	gene_id "XLOC_042542"; transcript_id "Novel_000577"; exon_number "4"; gene_type "
At_chr13	Cufflinks	exon	33311187	33311418	.	+	.	gene_id "XLOC_042542"; transcript_id "Novel_000577"; exon_number "5"; gene_type "
At_chr13	Cufflinks	exon	43528632	43528711	.	+	.	gene_id "XLOC_042943"; transcript_id "Novel_000578"; exon_number "1"; gene_type "
At_chr13	Cufflinks	exon	43528842	43528937	.	+	.	gene_id "XLOC_042943"; transcript_id "Novel_000578"; exon_number "2"; gene_type "
At_chr13	Cufflinks	exon	43530626	43532233	.	+	.	gene_id "XLOC_042943"; transcript_id "Novel_000578"; exon_number "3"; gene_type "
At_chr13	Cufflinks	exon	43532324	43533483	.	+	.	gene_id "XLOC_042943"; transcript_id "Novel_000578"; exon_number "4"; gene_type "
At_chr13	Cufflinks	exon	43528642	43528711	.	+	.	gene_id "XLOC_042943"; transcript_id "Novel_000579"; exon_number "1"; gene_type "
At_chr13	Cufflinks	exon	43528842	43528937	.	+	.	gene_id "XLOC_042943"; transcript_id "Novel_000579"; exon_number "2"; gene_type "
At_chr13	Cufflinks	exon	43530626	43531714	.	+	.	gene_id "XLOC_042943"; transcript_id "Novel_000579"; exon_number "3"; gene_type "
At_chr13	Cufflinks	exon	43531790	43533502	.	+	.	gene_id "XLOC_042943"; transcript_id "Novel_000579"; exon_number "4"; gene_type "
At_chr13	Cufflinks	exon	45086654	45087314	.	+	.	gene_id "XLOC_043022"; transcript_id "Novel_000580"; exon_number "1"; gene_type "
At_chr13	Cufflinks	exon	45087405	45088304	.	+	.	gene_id "XLOC_043022"; transcript_id "Novel_000580"; exon_number "2"; gene_type "
At_chr13	Cufflinks	exon	46462612	46462699	.	+	.	gene_id "XLOC_043060"; transcript_id "Novel_000581"; exon_number "1"; gene_type "
At_chr13	Cufflinks	exon	46463105	46463194	.	+	.	gene_id "XLOC_043060"; transcript_id "Novel_000581"; exon_number "2"; gene_type "
At_chr13	Cufflinks	exon	46463540	46463620	.	+	.	gene_id "XLOC_043060"; transcript_id "Novel_000581"; exon_number "3"; gene_type "
At_chr13	Cufflinks	exon	46464140	46464198	.	+	.	gene_id "XLOC_043060"; transcript_id "Novel_000581"; exon_number "4"; gene_type "
At_chr13	Cufflinks	exon	46464274	46464514	.	+	.	gene_id "XLOC_043060"; transcript_id "Novel_000581"; exon_number "5"; gene_type "
At_chr13	Cufflinks	exon	46465058	46465764	.	+	.	gene_id "XLOC_043060"; transcript_id "Novel_000581"; exon_number "6"; gene_type "
At_chr13	Cufflinks	exon	47340695	47340725	.	+	.	gene_id "XLOC_043082"; transcript_id "Novel_000582"; exon_number "1"; gene_type "
At_chr13	Cufflinks	exon	47340798	47340868	.	+	.	gene_id "XLOC_043082"; transcript_id "Novel_000582"; exon_number "2"; gene_type "
At_chr13	Cufflinks	exon	47340950	47341011	.	+	.	gene_id "XLOC_043082"; transcript_id "Novel_000582"; exon_number "3"; gene_type "
At_chr13	Cufflinks	exon	47342160	47342911	.	+	.	gene_id "XLOC_043082"; transcript_id "Novel_000582"; exon_number "4"; gene_type "
At_chr13	Cufflinks	exon	55557070	55557271	.	+	.	gene_id "XLOC_043355"; transcript_id "Novel_000583"; exon_number "1"; gene_type "

At_chr13	Cufflinks	exon	55557450	55560666	.	+	.	gene_id "XLOC_043355"; transcript_id "Novel_000583"; exon_number "2"; gene_type "
At_chr13	Cufflinks	exon	66888632	66888805	.	+	.	gene_id "XLOC_043712"; transcript_id "Novel_000584"; exon_number "1"; gene_type "
At_chr13	Cufflinks	exon	66888893	66889246	.	+	.	gene_id "XLOC_043712"; transcript_id "Novel_000584"; exon_number "2"; gene_type "
At_chr13	Cufflinks	exon	76116168	76116493	.	+	.	gene_id "XLOC_044040"; transcript_id "Novel_000585"; exon_number "1"; gene_type "
At_chr13	Cufflinks	exon	76116567	76116718	.	+	.	gene_id "XLOC_044040"; transcript_id "Novel_000585"; exon_number "2"; gene_type "
At_chr13	Cufflinks	exon	76116802	76116956	.	+	.	gene_id "XLOC_044040"; transcript_id "Novel_000585"; exon_number "3"; gene_type "
At_chr13	Cufflinks	exon	76117057	76117139	.	+	.	gene_id "XLOC_044040"; transcript_id "Novel_000585"; exon_number "4"; gene_type "
At_chr13	Cufflinks	exon	76117270	76117314	.	+	.	gene_id "XLOC_044040"; transcript_id "Novel_000585"; exon_number "5"; gene_type "
At_chr13	Cufflinks	exon	76117423	76117572	.	+	.	gene_id "XLOC_044040"; transcript_id "Novel_000585"; exon_number "6"; gene_type "
At_chr13	Cufflinks	exon	76117657	76117935	.	+	.	gene_id "XLOC_044040"; transcript_id "Novel_000585"; exon_number "7"; gene_type "
At_chr13	Cufflinks	exon	76118017	76118150	.	+	.	gene_id "XLOC_044040"; transcript_id "Novel_000585"; exon_number "8"; gene_type "
At_chr13	Cufflinks	exon	76118282	76118437	.	+	.	gene_id "XLOC_044040"; transcript_id "Novel_000585"; exon_number "9"; gene_type "
At_chr13	Cufflinks	exon	76118824	76118903	.	+	.	gene_id "XLOC_044040"; transcript_id "Novel_000585"; exon_number "10"; gene_type
At_chr13	Cufflinks	exon	76123474	76123839	.	+	.	gene_id "XLOC_044040"; transcript_id "Novel_000585"; exon_number "11"; gene_type
At_chr13	Cufflinks	exon	77376662	77379288	.	+	.	gene_id "XLOC_044138"; transcript_id "Novel_000586"; exon_number "1"; gene_type "
At_chr13	Cufflinks	exon	77379921	77380448	.	+	.	gene_id "XLOC_044138"; transcript_id "Novel_000586"; exon_number "2"; gene_type "
At_chr13	Cufflinks	exon	79894870	79895086	.	+	.	gene_id "XLOC_044337"; transcript_id "Novel_000587"; exon_number "1"; gene_type "
At_chr13	Cufflinks	exon	79896271	79898504	.	+	.	gene_id "XLOC_044337"; transcript_id "Novel_000587"; exon_number "2"; gene_type "
At_chr13	Cufflinks	exon	80441941	80442426	.	+	.	gene_id "XLOC_044365"; transcript_id "Novel_000588"; exon_number "1"; gene_type "
At_chr13	Cufflinks	exon	80442515	80442567	.	+	.	gene_id "XLOC_044365"; transcript_id "Novel_000588"; exon_number "2"; gene_type "
At_chr13	Cufflinks	exon	80442937	80443211	.	+	.	gene_id "XLOC_044365"; transcript_id "Novel_000588"; exon_number "3"; gene_type "
At_chr13	Cufflinks	exon	80527213	80527766	.	+	.	gene_id "XLOC_044389"; transcript_id "Novel_000589"; exon_number "1"; gene_type "
At_chr13	Cufflinks	exon	83522383	83522652	.	+	.	gene_id "XLOC_044497"; transcript_id "Novel_000590"; exon_number "1"; gene_type "
At_chr13	Cufflinks	exon	90198628	90198978	.	+	.	gene_id "XLOC_044777"; transcript_id "Novel_000591"; exon_number "1"; gene_type "
At_chr13	Cufflinks	exon	90199076	90199170	.	+	.	gene_id "XLOC_044777"; transcript_id "Novel_000591"; exon_number "2"; gene_type "
At_chr13	Cufflinks	exon	90199594	90200018	.	+	.	gene_id "XLOC_044777"; transcript_id "Novel_000591"; exon_number "3"; gene_type "
At_chr13	Cufflinks	exon	90200494	90200851	.	+	.	gene_id "XLOC_044777"; transcript_id "Novel_000591"; exon_number "4"; gene_type "
At_chr13	Cufflinks	exon	90201406	90201519	.	+	.	gene_id "XLOC_044777"; transcript_id "Novel_000591"; exon_number "5"; gene_type "
At_chr13	Cufflinks	exon	90201694	90202275	.	+	.	gene_id "XLOC_044777"; transcript_id "Novel_000591"; exon_number "6"; gene_type "
At_chr13	Cufflinks	exon	90198628	90199170	.	+	.	gene_id "XLOC_044777"; transcript_id "Novel_000592"; exon_number "1"; gene_type "
At_chr13	Cufflinks	exon	90199594	90200018	.	+	.	gene_id "XLOC_044777"; transcript_id "Novel_000592"; exon_number "2"; gene_type "
At_chr13	Cufflinks	exon	90200494	90200851	.	+	.	gene_id "XLOC_044777"; transcript_id "Novel_000592"; exon_number "3"; gene_type "
At_chr13	Cufflinks	exon	90201406	90201519	.	+	.	gene_id "XLOC_044777"; transcript_id "Novel_000592"; exon_number "4"; gene_type "
At_chr13	Cufflinks	exon	90201694	90202275	.	+	.	gene_id "XLOC_044777"; transcript_id "Novel_000592"; exon_number "5"; gene_type "
At_chr13	Cufflinks	exon	90199287	90200018	.	+	.	gene_id "XLOC_044777"; transcript_id "Novel_000593"; exon_number "1"; gene_type "

At_chr13	Cufflinks	exon	90200494	90200851	.	+	.	gene_id "XLOC_044777"; transcript_id "Novel_000593"; exon_number "2"; gene_type "
At_chr13	Cufflinks	exon	90201406	90201519	.	+	.	gene_id "XLOC_044777"; transcript_id "Novel_000593"; exon_number "3"; gene_type "
At_chr13	Cufflinks	exon	90201694	90202275	.	+	.	gene_id "XLOC_044777"; transcript_id "Novel_000593"; exon_number "4"; gene_type "
At_chr13	Cufflinks	exon	90218060	90218274	.	+	.	gene_id "XLOC_044779"; transcript_id "Novel_000594"; exon_number "1"; gene_type "
At_chr13	Cufflinks	exon	90218514	90218540	.	+	.	gene_id "XLOC_044779"; transcript_id "Novel_000594"; exon_number "2"; gene_type "
At_chr13	Cufflinks	exon	90218671	90218716	.	+	.	gene_id "XLOC_044779"; transcript_id "Novel_000594"; exon_number "3"; gene_type "
At_chr13	Cufflinks	exon	90219188	90219590	.	+	.	gene_id "XLOC_044779"; transcript_id "Novel_000595"; exon_number "1"; gene_type "
At_chr13	Cufflinks	exon	90219697	90219777	.	+	.	gene_id "XLOC_044779"; transcript_id "Novel_000595"; exon_number "2"; gene_type "
At_chr13	Cufflinks	exon	90219887	90220032	.	+	.	gene_id "XLOC_044779"; transcript_id "Novel_000595"; exon_number "3"; gene_type "
At_chr13	Cufflinks	exon	90220427	90220801	.	+	.	gene_id "XLOC_044779"; transcript_id "Novel_000595"; exon_number "4"; gene_type "
At_chr13	Cufflinks	exon	90219446	90219777	.	+	.	gene_id "XLOC_044779"; transcript_id "Novel_000596"; exon_number "1"; gene_type "
At_chr13	Cufflinks	exon	90219887	90220032	.	+	.	gene_id "XLOC_044779"; transcript_id "Novel_000596"; exon_number "2"; gene_type "
At_chr13	Cufflinks	exon	90220427	90221096	.	+	.	gene_id "XLOC_044779"; transcript_id "Novel_000596"; exon_number "3"; gene_type "
At_chr13	Cufflinks	exon	90257960	90258351	.	+	.	gene_id "XLOC_044783"; transcript_id "Novel_000597"; exon_number "1"; gene_type "
At_chr13	Cufflinks	exon	90261088	90261714	.	+	.	gene_id "XLOC_044783"; transcript_id "Novel_000597"; exon_number "2"; gene_type "
At_chr13	Cufflinks	exon	90262286	90262462	.	+	.	gene_id "XLOC_044783"; transcript_id "Novel_000598"; exon_number "1"; gene_type "
At_chr13	Cufflinks	exon	90262559	90262616	.	+	.	gene_id "XLOC_044783"; transcript_id "Novel_000598"; exon_number "2"; gene_type "
At_chr13	Cufflinks	exon	90262902	90263219	.	+	.	gene_id "XLOC_044783"; transcript_id "Novel_000598"; exon_number "3"; gene_type "
At_chr13	Cufflinks	exon	90263530	90263731	.	+	.	gene_id "XLOC_044783"; transcript_id "Novel_000598"; exon_number "4"; gene_type "
At_chr13	Cufflinks	exon	90263811	90264411	.	+	.	gene_id "XLOC_044783"; transcript_id "Novel_000598"; exon_number "5"; gene_type "
At_chr13	Cufflinks	exon	90264510	90264585	.	+	.	gene_id "XLOC_044783"; transcript_id "Novel_000598"; exon_number "6"; gene_type "
At_chr13	Cufflinks	exon	90264783	90265962	.	+	.	gene_id "XLOC_044783"; transcript_id "Novel_000598"; exon_number "7"; gene_type "
At_chr13	Cufflinks	exon	90262396	90262462	.	+	.	gene_id "XLOC_044783"; transcript_id "Novel_000599"; exon_number "1"; gene_type "
At_chr13	Cufflinks	exon	90262559	90262616	.	+	.	gene_id "XLOC_044783"; transcript_id "Novel_000599"; exon_number "2"; gene_type "
At_chr13	Cufflinks	exon	90262902	90263222	.	+	.	gene_id "XLOC_044783"; transcript_id "Novel_000599"; exon_number "3"; gene_type "
At_chr13	Cufflinks	exon	90263284	90263731	.	+	.	gene_id "XLOC_044783"; transcript_id "Novel_000600"; exon_number "1"; gene_type "
At_chr13	Cufflinks	exon	90263811	90264131	.	+	.	gene_id "XLOC_044783"; transcript_id "Novel_000600"; exon_number "2"; gene_type "
At_chr13	Cufflinks	exon	90264220	90264411	.	+	.	gene_id "XLOC_044783"; transcript_id "Novel_000600"; exon_number "3"; gene_type "
At_chr13	Cufflinks	exon	90264510	90264585	.	+	.	gene_id "XLOC_044783"; transcript_id "Novel_000600"; exon_number "4"; gene_type "
At_chr13	Cufflinks	exon	90264783	90265349	.	+	.	gene_id "XLOC_044783"; transcript_id "Novel_000600"; exon_number "5"; gene_type "
At_chr13	Cufflinks	exon	90266449	90266909	.	+	.	gene_id "XLOC_044783"; transcript_id "Novel_000600"; exon_number "6"; gene_type "
At_chr13	Cufflinks	exon	90354637	90355035	.	+	.	gene_id "XLOC_044801"; transcript_id "Novel_000601"; exon_number "1"; gene_type "
At_chr13	Cufflinks	exon	90355134	90355231	.	+	.	gene_id "XLOC_044801"; transcript_id "Novel_000601"; exon_number "2"; gene_type "
At_chr13	Cufflinks	exon	90355335	90355380	.	+	.	gene_id "XLOC_044801"; transcript_id "Novel_000601"; exon_number "3"; gene_type "
At_chr13	Cufflinks	exon	90355799	90355948	.	+	.	gene_id "XLOC_044801"; transcript_id "Novel_000601"; exon_number "4"; gene_type "

At_chr13	Cufflinks	exon	90356076	90358027	.	+	.	gene_id "XLOC_044801"; transcript_id "Novel_000601"; exon_number "5"; gene_type "
At_chr13	Cufflinks	exon	90362854	90363069	.	+	.	gene_id "XLOC_044805"; transcript_id "Novel_000602"; exon_number "1"; gene_type "
At_chr13	Cufflinks	exon	90364420	90365164	.	+	.	gene_id "XLOC_044805"; transcript_id "Novel_000602"; exon_number "2"; gene_type "
At_chr13	Cufflinks	exon	90365820	90365895	.	+	.	gene_id "XLOC_044805"; transcript_id "Novel_000602"; exon_number "3"; gene_type "
At_chr13	Cufflinks	exon	90366021	90366147	.	+	.	gene_id "XLOC_044805"; transcript_id "Novel_000602"; exon_number "4"; gene_type "
At_chr13	Cufflinks	exon	90366891	90368214	.	+	.	gene_id "XLOC_044805"; transcript_id "Novel_000602"; exon_number "5"; gene_type "
At_chr13	Cufflinks	exon	90362915	90363069	.	+	.	gene_id "XLOC_044805"; transcript_id "Novel_000603"; exon_number "1"; gene_type "
At_chr13	Cufflinks	exon	90364420	90365895	.	+	.	gene_id "XLOC_044805"; transcript_id "Novel_000603"; exon_number "2"; gene_type "
At_chr13	Cufflinks	exon	90366021	90366147	.	+	.	gene_id "XLOC_044805"; transcript_id "Novel_000603"; exon_number "3"; gene_type "
At_chr13	Cufflinks	exon	90366891	90368185	.	+	.	gene_id "XLOC_044805"; transcript_id "Novel_000603"; exon_number "4"; gene_type "
At_chr13	Cufflinks	exon	90890056	90890112	.	+	.	gene_id "XLOC_044883"; transcript_id "Novel_000604"; exon_number "1"; gene_type "
At_chr13	Cufflinks	exon	90890353	90890411	.	+	.	gene_id "XLOC_044883"; transcript_id "Novel_000604"; exon_number "2"; gene_type "
At_chr13	Cufflinks	exon	90891325	90893414	.	+	.	gene_id "XLOC_044883"; transcript_id "Novel_000604"; exon_number "3"; gene_type "
At_chr13	Cufflinks	exon	90890058	90890112	.	+	.	gene_id "XLOC_044883"; transcript_id "Novel_000605"; exon_number "1"; gene_type "
At_chr13	Cufflinks	exon	90890353	90890411	.	+	.	gene_id "XLOC_044883"; transcript_id "Novel_000605"; exon_number "2"; gene_type "
At_chr13	Cufflinks	exon	90891282	90893460	.	+	.	gene_id "XLOC_044883"; transcript_id "Novel_000605"; exon_number "3"; gene_type "
At_chr13	Cufflinks	exon	90890083	90890112	.	+	.	gene_id "XLOC_044883"; transcript_id "Novel_000606"; exon_number "1"; gene_type "
At_chr13	Cufflinks	exon	90890336	90890411	.	+	.	gene_id "XLOC_044883"; transcript_id "Novel_000606"; exon_number "2"; gene_type "
At_chr13	Cufflinks	exon	90891282	90893287	.	+	.	gene_id "XLOC_044883"; transcript_id "Novel_000606"; exon_number "3"; gene_type "
At_chr13	Cufflinks	exon	91471618	91471862	.	+	.	gene_id "XLOC_044974"; transcript_id "Novel_000607"; exon_number "1"; gene_type "
At_chr13	Cufflinks	exon	7827183	7828189	.	-	.	gene_id "XLOC_045328"; transcript_id "Novel_000608"; exon_number "1"; gene_type "
At_chr13	Cufflinks	exon	7829056	7829160	.	-	.	gene_id "XLOC_045328"; transcript_id "Novel_000608"; exon_number "2"; gene_type "
At_chr13	Cufflinks	exon	7829349	7829411	.	-	.	gene_id "XLOC_045328"; transcript_id "Novel_000608"; exon_number "3"; gene_type "
At_chr13	Cufflinks	exon	7829686	7829753	.	-	.	gene_id "XLOC_045328"; transcript_id "Novel_000608"; exon_number "4"; gene_type "
At_chr13	Cufflinks	exon	7830063	7830141	.	-	.	gene_id "XLOC_045328"; transcript_id "Novel_000608"; exon_number "5"; gene_type "
At_chr13	Cufflinks	exon	7830254	7830628	.	-	.	gene_id "XLOC_045328"; transcript_id "Novel_000608"; exon_number "6"; gene_type "
At_chr13	Cufflinks	exon	7828121	7828189	.	-	.	gene_id "XLOC_045328"; transcript_id "Novel_000609"; exon_number "1"; gene_type "
At_chr13	Cufflinks	exon	7828433	7828505	.	-	.	gene_id "XLOC_045328"; transcript_id "Novel_000609"; exon_number "2"; gene_type "
At_chr13	Cufflinks	exon	7829056	7829160	.	-	.	gene_id "XLOC_045328"; transcript_id "Novel_000609"; exon_number "3"; gene_type "
At_chr13	Cufflinks	exon	7829349	7830141	.	-	.	gene_id "XLOC_045328"; transcript_id "Novel_000609"; exon_number "4"; gene_type "
At_chr13	Cufflinks	exon	7830254	7830927	.	-	.	gene_id "XLOC_045328"; transcript_id "Novel_000609"; exon_number "5"; gene_type "
At_chr13	Cufflinks	exon	9421074	9426332	.	-	.	gene_id "XLOC_045408"; transcript_id "Novel_000610"; exon_number "1"; gene_type "
At_chr13	Cufflinks	exon	9426430	9430955	.	-	.	gene_id "XLOC_045408"; transcript_id "Novel_000610"; exon_number "2"; gene_type "
At_chr13	Cufflinks	exon	9421116	9430698	.	-	.	gene_id "XLOC_045408"; transcript_id "Novel_000611"; exon_number "1"; gene_type "
At_chr13	Cufflinks	exon	9430801	9430964	.	-	.	gene_id "XLOC_045408"; transcript_id "Novel_000611"; exon_number "2"; gene_type "

At_chr13	Cufflinks	exon	11291618	11291733	.	-	.	gene_id "XLOC_045457"; transcript_id "Novel_000612"; exon_number "1"; gene_type "
At_chr13	Cufflinks	exon	11291881	11292030	.	-	.	gene_id "XLOC_045457"; transcript_id "Novel_000612"; exon_number "2"; gene_type "
At_chr13	Cufflinks	exon	13183346	13183799	.	-	.	gene_id "XLOC_045544"; transcript_id "Novel_000613"; exon_number "1"; gene_type "
At_chr13	Cufflinks	exon	13184510	13184693	.	-	.	gene_id "XLOC_045544"; transcript_id "Novel_000613"; exon_number "2"; gene_type "
At_chr13	Cufflinks	exon	13184780	13184870	.	-	.	gene_id "XLOC_045544"; transcript_id "Novel_000613"; exon_number "3"; gene_type "
At_chr13	Cufflinks	exon	13184983	13185117	.	-	.	gene_id "XLOC_045544"; transcript_id "Novel_000613"; exon_number "4"; gene_type "
At_chr13	Cufflinks	exon	13659703	13659892	.	-	.	gene_id "XLOC_045615"; transcript_id "Novel_000614"; exon_number "1"; gene_type "
At_chr13	Cufflinks	exon	13659993	13660048	.	-	.	gene_id "XLOC_045615"; transcript_id "Novel_000614"; exon_number "2"; gene_type "
At_chr13	Cufflinks	exon	15030139	15031518	.	-	.	gene_id "XLOC_045697"; transcript_id "Novel_000615"; exon_number "1"; gene_type "
At_chr13	Cufflinks	exon	15032082	15032144	.	-	.	gene_id "XLOC_045697"; transcript_id "Novel_000615"; exon_number "2"; gene_type "
At_chr13	Cufflinks	exon	15396718	15398369	.	-	.	gene_id "XLOC_045734"; transcript_id "Novel_000616"; exon_number "1"; gene_type "
At_chr13	Cufflinks	exon	15398669	15398971	.	-	.	gene_id "XLOC_045734"; transcript_id "Novel_000616"; exon_number "2"; gene_type "
At_chr13	Cufflinks	exon	16490927	16491429	.	-	.	gene_id "XLOC_045836"; transcript_id "Novel_000617"; exon_number "1"; gene_type "
At_chr13	Cufflinks	exon	16491742	16491807	.	-	.	gene_id "XLOC_045836"; transcript_id "Novel_000617"; exon_number "2"; gene_type "
At_chr13	Cufflinks	exon	16492483	16492568	.	-	.	gene_id "XLOC_045836"; transcript_id "Novel_000617"; exon_number "3"; gene_type "
At_chr13	Cufflinks	exon	16493100	16493134	.	-	.	gene_id "XLOC_045836"; transcript_id "Novel_000617"; exon_number "4"; gene_type "
At_chr13	Cufflinks	exon	16493236	16493319	.	-	.	gene_id "XLOC_045836"; transcript_id "Novel_000617"; exon_number "5"; gene_type "
At_chr13	Cufflinks	exon	16493409	16493477	.	-	.	gene_id "XLOC_045836"; transcript_id "Novel_000617"; exon_number "6"; gene_type "
At_chr13	Cufflinks	exon	16494061	16494098	.	-	.	gene_id "XLOC_045836"; transcript_id "Novel_000617"; exon_number "7"; gene_type "
At_chr13	Cufflinks	exon	16494592	16494708	.	-	.	gene_id "XLOC_045836"; transcript_id "Novel_000617"; exon_number "8"; gene_type "
At_chr13	Cufflinks	exon	17130140	17132456	.	-	.	gene_id "XLOC_045884"; transcript_id "Novel_000618"; exon_number "1"; gene_type "
At_chr13	Cufflinks	exon	17132579	17132851	.	-	.	gene_id "XLOC_045884"; transcript_id "Novel_000618"; exon_number "2"; gene_type "
At_chr13	Cufflinks	exon	19086127	19087411	.	-	.	gene_id "XLOC_046056"; transcript_id "Novel_000619"; exon_number "1"; gene_type "
At_chr13	Cufflinks	exon	19088429	19088494	.	-	.	gene_id "XLOC_046056"; transcript_id "Novel_000619"; exon_number "2"; gene_type "
At_chr13	Cufflinks	exon	19088617	19089069	.	-	.	gene_id "XLOC_046056"; transcript_id "Novel_000619"; exon_number "3"; gene_type "
At_chr13	Cufflinks	exon	20677822	20678280	.	-	.	gene_id "XLOC_046174"; transcript_id "Novel_000620"; exon_number "1"; gene_type "
At_chr13	Cufflinks	exon	20678402	20680608	.	-	.	gene_id "XLOC_046174"; transcript_id "Novel_000620"; exon_number "2"; gene_type "
At_chr13	Cufflinks	exon	21408451	21408652	.	-	.	gene_id "XLOC_046235"; transcript_id "Novel_000621"; exon_number "1"; gene_type "
At_chr13	Cufflinks	exon	22857986	22859332	.	-	.	gene_id "XLOC_046388"; transcript_id "Novel_000622"; exon_number "1"; gene_type "
At_chr13	Cufflinks	exon	22873420	22873525	.	-	.	gene_id "XLOC_046394"; transcript_id "Novel_000623"; exon_number "1"; gene_type "
At_chr13	Cufflinks	exon	22873806	22873863	.	-	.	gene_id "XLOC_046394"; transcript_id "Novel_000623"; exon_number "2"; gene_type "
At_chr13	Cufflinks	exon	22873967	22874076	.	-	.	gene_id "XLOC_046394"; transcript_id "Novel_000623"; exon_number "3"; gene_type "
At_chr13	Cufflinks	exon	22874166	22874457	.	-	.	gene_id "XLOC_046394"; transcript_id "Novel_000623"; exon_number "4"; gene_type "
At_chr13	Cufflinks	exon	22904368	22904757	.	-	.	gene_id "XLOC_046394"; transcript_id "Novel_000624"; exon_number "1"; gene_type "
At_chr13	Cufflinks	exon	22904979	22905059	.	-	.	gene_id "XLOC_046394"; transcript_id "Novel_000624"; exon_number "2"; gene_type "

At_chr13	Cufflinks	exon	22905155	22905709	.	-	.	gene_id "XLOC_046394"; transcript_id "Novel_000624"; exon_number "3"; gene_type "
At_chr13	Cufflinks	exon	22905811	22905862	.	-	.	gene_id "XLOC_046394"; transcript_id "Novel_000624"; exon_number "4"; gene_type "
At_chr13	Cufflinks	exon	22905957	22906435	.	-	.	gene_id "XLOC_046394"; transcript_id "Novel_000624"; exon_number "5"; gene_type "
At_chr13	Cufflinks	exon	22906784	22906841	.	-	.	gene_id "XLOC_046394"; transcript_id "Novel_000624"; exon_number "6"; gene_type "
At_chr13	Cufflinks	exon	22906955	22907063	.	-	.	gene_id "XLOC_046394"; transcript_id "Novel_000624"; exon_number "7"; gene_type "
At_chr13	Cufflinks	exon	22907156	22907592	.	-	.	gene_id "XLOC_046394"; transcript_id "Novel_000624"; exon_number "8"; gene_type "
At_chr13	Cufflinks	exon	23022094	23022803	.	-	.	gene_id "XLOC_046427"; transcript_id "Novel_000625"; exon_number "1"; gene_type "
At_chr13	Cufflinks	exon	23022883	23023168	.	-	.	gene_id "XLOC_046427"; transcript_id "Novel_000625"; exon_number "2"; gene_type "
At_chr13	Cufflinks	exon	23038084	23039496	.	-	.	gene_id "XLOC_046428"; transcript_id "Novel_000626"; exon_number "1"; gene_type "
At_chr13	Cufflinks	exon	23039631	23039721	.	-	.	gene_id "XLOC_046428"; transcript_id "Novel_000626"; exon_number "2"; gene_type "
At_chr13	Cufflinks	exon	23717851	23718363	.	-	.	gene_id "XLOC_046498"; transcript_id "Novel_000627"; exon_number "1"; gene_type "
At_chr13	Cufflinks	exon	23718558	23718713	.	-	.	gene_id "XLOC_046498"; transcript_id "Novel_000627"; exon_number "2"; gene_type "
At_chr13	Cufflinks	exon	23820794	23820905	.	-	.	gene_id "XLOC_046507"; transcript_id "Novel_000628"; exon_number "1"; gene_type "
At_chr13	Cufflinks	exon	23821240	23821480	.	-	.	gene_id "XLOC_046507"; transcript_id "Novel_000628"; exon_number "2"; gene_type "
At_chr13	Cufflinks	exon	25794035	25795589	.	-	.	gene_id "XLOC_046838"; transcript_id "Novel_000629"; exon_number "1"; gene_type "
At_chr13	Cufflinks	exon	25795812	25795925	.	-	.	gene_id "XLOC_046838"; transcript_id "Novel_000629"; exon_number "2"; gene_type "
At_chr13	Cufflinks	exon	25796030	25796264	.	-	.	gene_id "XLOC_046838"; transcript_id "Novel_000629"; exon_number "3"; gene_type "
At_chr13	Cufflinks	exon	25796846	25796934	.	-	.	gene_id "XLOC_046838"; transcript_id "Novel_000629"; exon_number "4"; gene_type "
At_chr13	Cufflinks	exon	25797037	25797168	.	-	.	gene_id "XLOC_046838"; transcript_id "Novel_000629"; exon_number "5"; gene_type "
At_chr13	Cufflinks	exon	25797386	25797454	.	-	.	gene_id "XLOC_046838"; transcript_id "Novel_000629"; exon_number "6"; gene_type "
At_chr13	Cufflinks	exon	25794036	25795589	.	-	.	gene_id "XLOC_046838"; transcript_id "Novel_000630"; exon_number "1"; gene_type "
At_chr13	Cufflinks	exon	25795812	25795925	.	-	.	gene_id "XLOC_046838"; transcript_id "Novel_000630"; exon_number "2"; gene_type "
At_chr13	Cufflinks	exon	25796030	25796264	.	-	.	gene_id "XLOC_046838"; transcript_id "Novel_000630"; exon_number "3"; gene_type "
At_chr13	Cufflinks	exon	25796846	25797168	.	-	.	gene_id "XLOC_046838"; transcript_id "Novel_000630"; exon_number "4"; gene_type "
At_chr13	Cufflinks	exon	25797386	25797444	.	-	.	gene_id "XLOC_046838"; transcript_id "Novel_000630"; exon_number "5"; gene_type "
At_chr13	Cufflinks	exon	26426813	26426873	.	-	.	gene_id "XLOC_046898"; transcript_id "Novel_000631"; exon_number "1"; gene_type "
At_chr13	Cufflinks	exon	26426961	26427035	.	-	.	gene_id "XLOC_046898"; transcript_id "Novel_000631"; exon_number "2"; gene_type "
At_chr13	Cufflinks	exon	26427130	26427576	.	-	.	gene_id "XLOC_046898"; transcript_id "Novel_000631"; exon_number "3"; gene_type "
At_chr13	Cufflinks	exon	26427482	26427861	.	-	.	gene_id "XLOC_046898"; transcript_id "Novel_000632"; exon_number "1"; gene_type "
At_chr13	Cufflinks	exon	26428508	26428667	.	-	.	gene_id "XLOC_046898"; transcript_id "Novel_000632"; exon_number "2"; gene_type "
At_chr13	Cufflinks	exon	26518021	26518680	.	-	.	gene_id "XLOC_046930"; transcript_id "Novel_000633"; exon_number "1"; gene_type "
At_chr13	Cufflinks	exon	26518778	26518878	.	-	.	gene_id "XLOC_046930"; transcript_id "Novel_000633"; exon_number "2"; gene_type "
At_chr13	Cufflinks	exon	26519020	26519119	.	-	.	gene_id "XLOC_046930"; transcript_id "Novel_000633"; exon_number "3"; gene_type "
At_chr13	Cufflinks	exon	26519406	26519493	.	-	.	gene_id "XLOC_046930"; transcript_id "Novel_000633"; exon_number "4"; gene_type "
At_chr13	Cufflinks	exon	26519573	26519614	.	-	.	gene_id "XLOC_046930"; transcript_id "Novel_000633"; exon_number "5"; gene_type "

At_chr13	Cufflinks	exon	26519724	26519830	.	-	.	gene_id "XLOC_046930"; transcript_id "Novel_000633"; exon_number "6"; gene_type "
At_chr13	Cufflinks	exon	26520191	26520357	.	-	.	gene_id "XLOC_046930"; transcript_id "Novel_000633"; exon_number "7"; gene_type "
At_chr13	Cufflinks	exon	26520751	26520793	.	-	.	gene_id "XLOC_046930"; transcript_id "Novel_000633"; exon_number "8"; gene_type "
At_chr13	Cufflinks	exon	26518021	26518680	.	-	.	gene_id "XLOC_046930"; transcript_id "Novel_000634"; exon_number "1"; gene_type "
At_chr13	Cufflinks	exon	26518778	26518878	.	-	.	gene_id "XLOC_046930"; transcript_id "Novel_000634"; exon_number "2"; gene_type "
At_chr13	Cufflinks	exon	26519020	26519119	.	-	.	gene_id "XLOC_046930"; transcript_id "Novel_000634"; exon_number "3"; gene_type "
At_chr13	Cufflinks	exon	26519406	26519614	.	-	.	gene_id "XLOC_046930"; transcript_id "Novel_000634"; exon_number "4"; gene_type "
At_chr13	Cufflinks	exon	26519724	26519830	.	-	.	gene_id "XLOC_046930"; transcript_id "Novel_000634"; exon_number "5"; gene_type "
At_chr13	Cufflinks	exon	26520191	26520357	.	-	.	gene_id "XLOC_046930"; transcript_id "Novel_000634"; exon_number "6"; gene_type "
At_chr13	Cufflinks	exon	26520751	26520820	.	-	.	gene_id "XLOC_046930"; transcript_id "Novel_000634"; exon_number "7"; gene_type "
At_chr13	Cufflinks	exon	26992461	26992681	.	-	.	gene_id "XLOC_046967"; transcript_id "Novel_000635"; exon_number "1"; gene_type "
At_chr13	Cufflinks	exon	27201397	27202419	.	-	.	gene_id "XLOC_046975"; transcript_id "Novel_000636"; exon_number "1"; gene_type "
At_chr13	Cufflinks	exon	27202551	27202593	.	-	.	gene_id "XLOC_046975"; transcript_id "Novel_000636"; exon_number "2"; gene_type "
At_chr13	Cufflinks	exon	27202934	27202988	.	-	.	gene_id "XLOC_046975"; transcript_id "Novel_000636"; exon_number "3"; gene_type "
At_chr13	Cufflinks	exon	27203117	27203174	.	-	.	gene_id "XLOC_046975"; transcript_id "Novel_000636"; exon_number "4"; gene_type "
At_chr13	Cufflinks	exon	27203370	27203446	.	-	.	gene_id "XLOC_046975"; transcript_id "Novel_000636"; exon_number "5"; gene_type "
At_chr13	Cufflinks	exon	27203538	27203838	.	-	.	gene_id "XLOC_046975"; transcript_id "Novel_000636"; exon_number "6"; gene_type "
At_chr13	Cufflinks	exon	27331128	27331722	.	-	.	gene_id "XLOC_046996"; transcript_id "Novel_000637"; exon_number "1"; gene_type "
At_chr13	Cufflinks	exon	27331847	27331906	.	-	.	gene_id "XLOC_046996"; transcript_id "Novel_000637"; exon_number "2"; gene_type "
At_chr13	Cufflinks	exon	27332169	27332301	.	-	.	gene_id "XLOC_046996"; transcript_id "Novel_000637"; exon_number "3"; gene_type "
At_chr13	Cufflinks	exon	27332404	27332531	.	-	.	gene_id "XLOC_046996"; transcript_id "Novel_000637"; exon_number "4"; gene_type "
At_chr13	Cufflinks	exon	27332646	27332751	.	-	.	gene_id "XLOC_046996"; transcript_id "Novel_000637"; exon_number "5"; gene_type "
At_chr13	Cufflinks	exon	27332911	27332990	.	-	.	gene_id "XLOC_046996"; transcript_id "Novel_000637"; exon_number "6"; gene_type "
At_chr13	Cufflinks	exon	27333112	27333209	.	-	.	gene_id "XLOC_046996"; transcript_id "Novel_000637"; exon_number "7"; gene_type "
At_chr13	Cufflinks	exon	27333327	27333440	.	-	.	gene_id "XLOC_046996"; transcript_id "Novel_000637"; exon_number "8"; gene_type "
At_chr13	Cufflinks	exon	27333524	27333664	.	-	.	gene_id "XLOC_046996"; transcript_id "Novel_000637"; exon_number "9"; gene_type "
At_chr13	Cufflinks	exon	27333825	27333909	.	-	.	gene_id "XLOC_046996"; transcript_id "Novel_000637"; exon_number "10"; gene_type
At_chr13	Cufflinks	exon	27335177	27335612	.	-	.	gene_id "XLOC_046996"; transcript_id "Novel_000637"; exon_number "11"; gene_type
At_chr13	Cufflinks	exon	27331128	27331722	.	-	.	gene_id "XLOC_046996"; transcript_id "Novel_000638"; exon_number "1"; gene_type "
At_chr13	Cufflinks	exon	27331847	27331906	.	-	.	gene_id "XLOC_046996"; transcript_id "Novel_000638"; exon_number "2"; gene_type "
At_chr13	Cufflinks	exon	27332169	27332301	.	-	.	gene_id "XLOC_046996"; transcript_id "Novel_000638"; exon_number "3"; gene_type "
At_chr13	Cufflinks	exon	27332404	27332531	.	-	.	gene_id "XLOC_046996"; transcript_id "Novel_000638"; exon_number "4"; gene_type "
At_chr13	Cufflinks	exon	27332646	27332751	.	-	.	gene_id "XLOC_046996"; transcript_id "Novel_000638"; exon_number "5"; gene_type "
At_chr13	Cufflinks	exon	27332911	27332990	.	-	.	gene_id "XLOC_046996"; transcript_id "Novel_000638"; exon_number "6"; gene_type "
At_chr13	Cufflinks	exon	27333112	27333209	.	-	.	gene_id "XLOC_046996"; transcript_id "Novel_000638"; exon_number "7"; gene_type "

At_chr13	Cufflinks	exon	27333327	27333440	.	-	.	gene_id "XLOC_046996"; transcript_id "Novel_000638"; exon_number "8"; gene_type "
At_chr13	Cufflinks	exon	27333524	27333664	.	-	.	gene_id "XLOC_046996"; transcript_id "Novel_000638"; exon_number "9"; gene_type "
At_chr13	Cufflinks	exon	27333825	27333909	.	-	.	gene_id "XLOC_046996"; transcript_id "Novel_000638"; exon_number "10"; gene_type
At_chr13	Cufflinks	exon	27335177	27335385	.	-	.	gene_id "XLOC_046996"; transcript_id "Novel_000638"; exon_number "11"; gene_type
At_chr13	Cufflinks	exon	27335508	27335619	.	-	.	gene_id "XLOC_046996"; transcript_id "Novel_000638"; exon_number "12"; gene_type
At_chr13	Cufflinks	exon	27457247	27457865	.	-	.	gene_id "XLOC_047003"; transcript_id "Novel_000639"; exon_number "1"; gene_type "
At_chr13	Cufflinks	exon	27458322	27458654	.	-	.	gene_id "XLOC_047003"; transcript_id "Novel_000639"; exon_number "2"; gene_type "
At_chr13	Cufflinks	exon	27610571	27610620	.	-	.	gene_id "XLOC_047021"; transcript_id "Novel_000640"; exon_number "1"; gene_type "
At_chr13	Cufflinks	exon	27611138	27611231	.	-	.	gene_id "XLOC_047021"; transcript_id "Novel_000640"; exon_number "2"; gene_type "
At_chr13	Cufflinks	exon	27611479	27611567	.	-	.	gene_id "XLOC_047021"; transcript_id "Novel_000640"; exon_number "3"; gene_type "
At_chr13	Cufflinks	exon	27611669	27612024	.	-	.	gene_id "XLOC_047021"; transcript_id "Novel_000640"; exon_number "4"; gene_type "
At_chr13	Cufflinks	exon	27610590	27610620	.	-	.	gene_id "XLOC_047021"; transcript_id "Novel_000641"; exon_number "1"; gene_type "
At_chr13	Cufflinks	exon	27611138	27611231	.	-	.	gene_id "XLOC_047021"; transcript_id "Novel_000641"; exon_number "2"; gene_type "
At_chr13	Cufflinks	exon	27611479	27612019	.	-	.	gene_id "XLOC_047021"; transcript_id "Novel_000641"; exon_number "3"; gene_type "
At_chr13	Cufflinks	exon	27657096	27657359	.	-	.	gene_id "XLOC_047023"; transcript_id "Novel_000642"; exon_number "1"; gene_type "
At_chr13	Cufflinks	exon	27658044	27658094	.	-	.	gene_id "XLOC_047023"; transcript_id "Novel_000642"; exon_number "2"; gene_type "
At_chr13	Cufflinks	exon	27658179	27658245	.	-	.	gene_id "XLOC_047023"; transcript_id "Novel_000642"; exon_number "3"; gene_type "
At_chr13	Cufflinks	exon	27658664	27659187	.	-	.	gene_id "XLOC_047023"; transcript_id "Novel_000642"; exon_number "4"; gene_type "
At_chr13	Cufflinks	exon	27657096	27657359	.	-	.	gene_id "XLOC_047023"; transcript_id "Novel_000643"; exon_number "1"; gene_type "
At_chr13	Cufflinks	exon	27658044	27658094	.	-	.	gene_id "XLOC_047023"; transcript_id "Novel_000643"; exon_number "2"; gene_type "
At_chr13	Cufflinks	exon	27658179	27658245	.	-	.	gene_id "XLOC_047023"; transcript_id "Novel_000643"; exon_number "3"; gene_type "
At_chr13	Cufflinks	exon	27658664	27658705	.	-	.	gene_id "XLOC_047023"; transcript_id "Novel_000643"; exon_number "4"; gene_type "
At_chr13	Cufflinks	exon	27659518	27659713	.	-	.	gene_id "XLOC_047023"; transcript_id "Novel_000643"; exon_number "5"; gene_type "
At_chr13	Cufflinks	exon	27660062	27660114	.	-	.	gene_id "XLOC_047023"; transcript_id "Novel_000643"; exon_number "6"; gene_type "
At_chr13	Cufflinks	exon	27661165	27661314	.	-	.	gene_id "XLOC_047023"; transcript_id "Novel_000643"; exon_number "7"; gene_type "
At_chr13	Cufflinks	exon	27661803	27662041	.	-	.	gene_id "XLOC_047023"; transcript_id "Novel_000643"; exon_number "8"; gene_type "
At_chr13	Cufflinks	exon	27662121	27662276	.	-	.	gene_id "XLOC_047023"; transcript_id "Novel_000643"; exon_number "9"; gene_type "
At_chr13	Cufflinks	exon	27659371	27659713	.	-	.	gene_id "XLOC_047023"; transcript_id "Novel_000644"; exon_number "1"; gene_type "
At_chr13	Cufflinks	exon	27660062	27660114	.	-	.	gene_id "XLOC_047023"; transcript_id "Novel_000644"; exon_number "2"; gene_type "
At_chr13	Cufflinks	exon	27661165	27661314	.	-	.	gene_id "XLOC_047023"; transcript_id "Novel_000644"; exon_number "3"; gene_type "
At_chr13	Cufflinks	exon	27661803	27662181	.	-	.	gene_id "XLOC_047023"; transcript_id "Novel_000644"; exon_number "4"; gene_type "
At_chr13	Cufflinks	exon	27659385	27659713	.	-	.	gene_id "XLOC_047023"; transcript_id "Novel_000645"; exon_number "1"; gene_type "
At_chr13	Cufflinks	exon	27660062	27661390	.	-	.	gene_id "XLOC_047023"; transcript_id "Novel_000645"; exon_number "2"; gene_type "
At_chr13	Cufflinks	exon	27659433	27659713	.	-	.	gene_id "XLOC_047023"; transcript_id "Novel_000646"; exon_number "1"; gene_type "
At_chr13	Cufflinks	exon	27660062	27660114	.	-	.	gene_id "XLOC_047023"; transcript_id "Novel_000646"; exon_number "2"; gene_type "

At_chr13	Cufflinks	exon	27661165	27661314	.	-	.	gene_id "XLOC_047023"; transcript_id "Novel_000646"; exon_number "3"; gene_type "
At_chr13	Cufflinks	exon	27661803	27662041	.	-	.	gene_id "XLOC_047023"; transcript_id "Novel_000646"; exon_number "4"; gene_type "
At_chr13	Cufflinks	exon	27662121	27662212	.	-	.	gene_id "XLOC_047023"; transcript_id "Novel_000646"; exon_number "5"; gene_type "
At_chr13	Cufflinks	exon	27661043	27661314	.	-	.	gene_id "XLOC_047023"; transcript_id "Novel_000647"; exon_number "1"; gene_type "
At_chr13	Cufflinks	exon	27661803	27662041	.	-	.	gene_id "XLOC_047023"; transcript_id "Novel_000647"; exon_number "2"; gene_type "
At_chr13	Cufflinks	exon	27662121	27662264	.	-	.	gene_id "XLOC_047023"; transcript_id "Novel_000647"; exon_number "3"; gene_type "
At_chr13	Cufflinks	exon	27696354	27697225	.	-	.	gene_id "XLOC_047024"; transcript_id "Novel_000648"; exon_number "1"; gene_type "
At_chr13	Cufflinks	exon	27700673	27700754	.	-	.	gene_id "XLOC_047024"; transcript_id "Novel_000648"; exon_number "2"; gene_type "
At_chr13	Cufflinks	exon	27700840	27700937	.	-	.	gene_id "XLOC_047024"; transcript_id "Novel_000648"; exon_number "3"; gene_type "
At_chr13	Cufflinks	exon	27701045	27701125	.	-	.	gene_id "XLOC_047024"; transcript_id "Novel_000648"; exon_number "4"; gene_type "
At_chr13	Cufflinks	exon	27701230	27701321	.	-	.	gene_id "XLOC_047024"; transcript_id "Novel_000648"; exon_number "5"; gene_type "
At_chr13	Cufflinks	exon	27702531	27702581	.	-	.	gene_id "XLOC_047024"; transcript_id "Novel_000648"; exon_number "6"; gene_type "
At_chr13	Cufflinks	exon	27702808	27702877	.	-	.	gene_id "XLOC_047024"; transcript_id "Novel_000648"; exon_number "7"; gene_type "
At_chr13	Cufflinks	exon	27696591	27697225	.	-	.	gene_id "XLOC_047024"; transcript_id "Novel_000649"; exon_number "1"; gene_type "
At_chr13	Cufflinks	exon	27700673	27700754	.	-	.	gene_id "XLOC_047024"; transcript_id "Novel_000649"; exon_number "2"; gene_type "
At_chr13	Cufflinks	exon	27700840	27700937	.	-	.	gene_id "XLOC_047024"; transcript_id "Novel_000649"; exon_number "3"; gene_type "
At_chr13	Cufflinks	exon	27701045	27701125	.	-	.	gene_id "XLOC_047024"; transcript_id "Novel_000649"; exon_number "4"; gene_type "
At_chr13	Cufflinks	exon	27701230	27701321	.	-	.	gene_id "XLOC_047024"; transcript_id "Novel_000649"; exon_number "5"; gene_type "
At_chr13	Cufflinks	exon	27702531	27702587	.	-	.	gene_id "XLOC_047024"; transcript_id "Novel_000649"; exon_number "6"; gene_type "
At_chr13	Cufflinks	exon	27702808	27702822	.	-	.	gene_id "XLOC_047024"; transcript_id "Novel_000649"; exon_number "7"; gene_type "
At_chr13	Cufflinks	exon	31576780	31578573	.	-	.	gene_id "XLOC_047149"; transcript_id "Novel_000650"; exon_number "1"; gene_type "
At_chr13	Cufflinks	exon	31578660	31580475	.	-	.	gene_id "XLOC_047149"; transcript_id "Novel_000650"; exon_number "2"; gene_type "
At_chr13	Cufflinks	exon	31577136	31577604	.	-	.	gene_id "XLOC_047149"; transcript_id "Novel_000651"; exon_number "1"; gene_type "
At_chr13	Cufflinks	exon	31578119	31578175	.	-	.	gene_id "XLOC_047149"; transcript_id "Novel_000651"; exon_number "2"; gene_type "
At_chr13	Cufflinks	exon	31578660	31580491	.	-	.	gene_id "XLOC_047149"; transcript_id "Novel_000651"; exon_number "3"; gene_type "
At_chr13	Cufflinks	exon	31577161	31577604	.	-	.	gene_id "XLOC_047149"; transcript_id "Novel_000652"; exon_number "1"; gene_type "
At_chr13	Cufflinks	exon	31578119	31578573	.	-	.	gene_id "XLOC_047149"; transcript_id "Novel_000652"; exon_number "2"; gene_type "
At_chr13	Cufflinks	exon	31578660	31580503	.	-	.	gene_id "XLOC_047149"; transcript_id "Novel_000652"; exon_number "3"; gene_type "
At_chr13	Cufflinks	exon	32953141	32953332	.	-	.	gene_id "XLOC_047205"; transcript_id "Novel_000653"; exon_number "1"; gene_type "
At_chr13	Cufflinks	exon	32953731	32955102	.	-	.	gene_id "XLOC_047205"; transcript_id "Novel_000653"; exon_number "2"; gene_type "
At_chr13	Cufflinks	exon	32956263	32956544	.	-	.	gene_id "XLOC_047205"; transcript_id "Novel_000653"; exon_number "3"; gene_type "
At_chr13	Cufflinks	exon	45601294	45601496	.	-	.	gene_id "XLOC_047787"; transcript_id "Novel_000654"; exon_number "1"; gene_type "
At_chr13	Cufflinks	exon	47760157	47760555	.	-	.	gene_id "XLOC_047864"; transcript_id "Novel_000655"; exon_number "1"; gene_type "
At_chr13	Cufflinks	exon	47760677	47760771	.	-	.	gene_id "XLOC_047864"; transcript_id "Novel_000655"; exon_number "2"; gene_type "
At_chr13	Cufflinks	exon	55359332	55359372	.	-	.	gene_id "XLOC_048089"; transcript_id "Novel_000656"; exon_number "1"; gene_type "

At_chr13	Cufflinks	exon	55359467	55360184	.	-	.	gene_id "XLOC_048089"; transcript_id "Novel_000656"; exon_number "2"; gene_type "
At_chr13	Cufflinks	exon	55524187	55524625	.	-	.	gene_id "XLOC_048110"; transcript_id "Novel_000657"; exon_number "1"; gene_type "
At_chr13	Cufflinks	exon	55524974	55525226	.	-	.	gene_id "XLOC_048110"; transcript_id "Novel_000657"; exon_number "2"; gene_type "
At_chr13	Cufflinks	exon	55525302	55525544	.	-	.	gene_id "XLOC_048110"; transcript_id "Novel_000657"; exon_number "3"; gene_type "
At_chr13	Cufflinks	exon	55525645	55525775	.	-	.	gene_id "XLOC_048110"; transcript_id "Novel_000657"; exon_number "4"; gene_type "
At_chr13	Cufflinks	exon	55525851	55526029	.	-	.	gene_id "XLOC_048110"; transcript_id "Novel_000657"; exon_number "5"; gene_type "
At_chr13	Cufflinks	exon	76573951	76575120	.	-	.	gene_id "XLOC_048805"; transcript_id "Novel_000658"; exon_number "1"; gene_type "
At_chr13	Cufflinks	exon	76575241	76575336	.	-	.	gene_id "XLOC_048805"; transcript_id "Novel_000658"; exon_number "2"; gene_type "
At_chr13	Cufflinks	exon	76575431	76575466	.	-	.	gene_id "XLOC_048805"; transcript_id "Novel_000658"; exon_number "3"; gene_type "
At_chr13	Cufflinks	exon	76576602	76576811	.	-	.	gene_id "XLOC_048805"; transcript_id "Novel_000658"; exon_number "4"; gene_type "
At_chr13	Cufflinks	exon	78104636	78105135	.	-	.	gene_id "XLOC_048941"; transcript_id "Novel_000659"; exon_number "1"; gene_type "
At_chr13	Cufflinks	exon	78105208	78105441	.	-	.	gene_id "XLOC_048941"; transcript_id "Novel_000659"; exon_number "2"; gene_type "
At_chr13	Cufflinks	exon	78133266	78133384	.	-	.	gene_id "XLOC_048952"; transcript_id "Novel_000660"; exon_number "1"; gene_type "
At_chr13	Cufflinks	exon	78133456	78133743	.	-	.	gene_id "XLOC_048952"; transcript_id "Novel_000660"; exon_number "2"; gene_type "
At_chr13	Cufflinks	exon	84457359	84457570	.	-	.	gene_id "XLOC_049283"; transcript_id "Novel_000661"; exon_number "1"; gene_type "
At_chr13	Cufflinks	exon	85959284	85959869	.	-	.	gene_id "XLOC_049399"; transcript_id "Novel_000662"; exon_number "1"; gene_type "
At_chr13	Cufflinks	exon	85963597	85963662	.	-	.	gene_id "XLOC_049399"; transcript_id "Novel_000662"; exon_number "2"; gene_type "
At_chr13	Cufflinks	exon	85963753	85964216	.	-	.	gene_id "XLOC_049399"; transcript_id "Novel_000662"; exon_number "3"; gene_type "
At_chr13	Cufflinks	exon	90224435	90224818	.	-	.	gene_id "XLOC_049552"; transcript_id "Novel_000663"; exon_number "1"; gene_type "
At_chr13	Cufflinks	exon	90225607	90225766	.	-	.	gene_id "XLOC_049552"; transcript_id "Novel_000663"; exon_number "2"; gene_type "
At_chr13	Cufflinks	exon	90225849	90225970	.	-	.	gene_id "XLOC_049552"; transcript_id "Novel_000663"; exon_number "3"; gene_type "
At_chr13	Cufflinks	exon	90226393	90226514	.	-	.	gene_id "XLOC_049552"; transcript_id "Novel_000663"; exon_number "4"; gene_type "
At_chr13	Cufflinks	exon	90226611	90226731	.	-	.	gene_id "XLOC_049552"; transcript_id "Novel_000663"; exon_number "5"; gene_type "
At_chr13	Cufflinks	exon	90226833	90226932	.	-	.	gene_id "XLOC_049552"; transcript_id "Novel_000663"; exon_number "6"; gene_type "
At_chr13	Cufflinks	exon	90227537	90227772	.	-	.	gene_id "XLOC_049552"; transcript_id "Novel_000663"; exon_number "7"; gene_type "
At_chr13	Cufflinks	exon	90225433	90225766	.	-	.	gene_id "XLOC_049552"; transcript_id "Novel_000664"; exon_number "1"; gene_type "
At_chr13	Cufflinks	exon	90225849	90225970	.	-	.	gene_id "XLOC_049552"; transcript_id "Novel_000664"; exon_number "2"; gene_type "
At_chr13	Cufflinks	exon	90226393	90226514	.	-	.	gene_id "XLOC_049552"; transcript_id "Novel_000664"; exon_number "3"; gene_type "
At_chr13	Cufflinks	exon	90226611	90226731	.	-	.	gene_id "XLOC_049552"; transcript_id "Novel_000664"; exon_number "4"; gene_type "
At_chr13	Cufflinks	exon	90226833	90226932	.	-	.	gene_id "XLOC_049552"; transcript_id "Novel_000664"; exon_number "5"; gene_type "
At_chr13	Cufflinks	exon	90227537	90227740	.	-	.	gene_id "XLOC_049552"; transcript_id "Novel_000664"; exon_number "6"; gene_type "
At_chr13	Cufflinks	exon	90311641	90311784	.	-	.	gene_id "XLOC_049566"; transcript_id "Novel_000665"; exon_number "1"; gene_type "
At_chr13	Cufflinks	exon	90312060	90312187	.	-	.	gene_id "XLOC_049566"; transcript_id "Novel_000665"; exon_number "2"; gene_type "
At_chr13	Cufflinks	exon	91079724	91080110	.	-	.	gene_id "XLOC_049680"; transcript_id "Novel_000666"; exon_number "1"; gene_type "
At_chr13	Cufflinks	exon	91080476	91080679	.	-	.	gene_id "XLOC_049680"; transcript_id "Novel_000666"; exon_number "2"; gene_type "

At_chr13	Cufflinks	exon	91080772	91080945	.	-	.	gene_id "XLOC_049680"; transcript_id "Novel_000666"; exon_number "3"; gene_type "
At_chr13	Cufflinks	exon	91081084	91081406	.	-	.	gene_id "XLOC_049680"; transcript_id "Novel_000666"; exon_number "4"; gene_type "
At_chr13	Cufflinks	exon	91081480	91082298	.	-	.	gene_id "XLOC_049680"; transcript_id "Novel_000666"; exon_number "5"; gene_type "
At_chr13	Cufflinks	exon	91082417	91082535	.	-	.	gene_id "XLOC_049680"; transcript_id "Novel_000666"; exon_number "6"; gene_type "
At_chr13	Cufflinks	exon	91083792	91084467	.	-	.	gene_id "XLOC_049680"; transcript_id "Novel_000666"; exon_number "7"; gene_type "
At_chr13	Cufflinks	exon	91079727	91080110	.	-	.	gene_id "XLOC_049680"; transcript_id "Novel_000667"; exon_number "1"; gene_type "
At_chr13	Cufflinks	exon	91080476	91080679	.	-	.	gene_id "XLOC_049680"; transcript_id "Novel_000667"; exon_number "2"; gene_type "
At_chr13	Cufflinks	exon	91080772	91080945	.	-	.	gene_id "XLOC_049680"; transcript_id "Novel_000667"; exon_number "3"; gene_type "
At_chr13	Cufflinks	exon	91081084	91081218	.	-	.	gene_id "XLOC_049680"; transcript_id "Novel_000667"; exon_number "4"; gene_type "
At_chr13	Cufflinks	exon	91081317	91081406	.	-	.	gene_id "XLOC_049680"; transcript_id "Novel_000667"; exon_number "5"; gene_type "
At_chr13	Cufflinks	exon	91081480	91081557	.	-	.	gene_id "XLOC_049680"; transcript_id "Novel_000667"; exon_number "6"; gene_type "
At_chr13	Cufflinks	exon	91081642	91082298	.	-	.	gene_id "XLOC_049680"; transcript_id "Novel_000667"; exon_number "7"; gene_type "
At_chr13	Cufflinks	exon	91082417	91082535	.	-	.	gene_id "XLOC_049680"; transcript_id "Novel_000667"; exon_number "8"; gene_type "
At_chr13	Cufflinks	exon	91083792	91084592	.	-	.	gene_id "XLOC_049680"; transcript_id "Novel_000667"; exon_number "9"; gene_type "
At_chr2	Cufflinks	exon	3237512	3238126	.	+	.	gene_id "XLOC_049962"; transcript_id "Novel_000668"; exon_number "1"; gene_type "
At_chr2	Cufflinks	exon	3238383	3238480	.	+	.	gene_id "XLOC_049962"; transcript_id "Novel_000668"; exon_number "2"; gene_type "
At_chr2	Cufflinks	exon	3238568	3238598	.	+	.	gene_id "XLOC_049962"; transcript_id "Novel_000668"; exon_number "3"; gene_type "
At_chr2	Cufflinks	exon	3238681	3238780	.	+	.	gene_id "XLOC_049962"; transcript_id "Novel_000668"; exon_number "4"; gene_type "
At_chr2	Cufflinks	exon	3238855	3238925	.	+	.	gene_id "XLOC_049962"; transcript_id "Novel_000668"; exon_number "5"; gene_type "
At_chr2	Cufflinks	exon	3239045	3239188	.	+	.	gene_id "XLOC_049962"; transcript_id "Novel_000668"; exon_number "6"; gene_type "
At_chr2	Cufflinks	exon	3239281	3239850	.	+	.	gene_id "XLOC_049962"; transcript_id "Novel_000668"; exon_number "7"; gene_type "
At_chr2	Cufflinks	exon	3238169	3238598	.	+	.	gene_id "XLOC_049962"; transcript_id "Novel_000669"; exon_number "1"; gene_type "
At_chr2	Cufflinks	exon	3238681	3238780	.	+	.	gene_id "XLOC_049962"; transcript_id "Novel_000669"; exon_number "2"; gene_type "
At_chr2	Cufflinks	exon	3238855	3238925	.	+	.	gene_id "XLOC_049962"; transcript_id "Novel_000669"; exon_number "3"; gene_type "
At_chr2	Cufflinks	exon	3239045	3239188	.	+	.	gene_id "XLOC_049962"; transcript_id "Novel_000669"; exon_number "4"; gene_type "
At_chr2	Cufflinks	exon	3239281	3239908	.	+	.	gene_id "XLOC_049962"; transcript_id "Novel_000669"; exon_number "5"; gene_type "
At_chr2	Cufflinks	exon	5137019	5137571	.	+	.	gene_id "XLOC_050094"; transcript_id "Novel_000670"; exon_number "1"; gene_type "
At_chr2	Cufflinks	exon	5137669	5138790	.	+	.	gene_id "XLOC_050094"; transcript_id "Novel_000670"; exon_number "2"; gene_type "
At_chr2	Cufflinks	exon	6887052	6888057	.	+	.	gene_id "XLOC_050259"; transcript_id "Novel_000671"; exon_number "1"; gene_type "
At_chr2	Cufflinks	exon	6888621	6888665	.	+	.	gene_id "XLOC_050259"; transcript_id "Novel_000671"; exon_number "2"; gene_type "
At_chr2	Cufflinks	exon	6888817	6888851	.	+	.	gene_id "XLOC_050259"; transcript_id "Novel_000671"; exon_number "3"; gene_type "
At_chr2	Cufflinks	exon	9503723	9504025	.	+	.	gene_id "XLOC_050540"; transcript_id "Novel_000672"; exon_number "1"; gene_type "
At_chr2	Cufflinks	exon	10994646	10994686	.	+	.	gene_id "XLOC_050666"; transcript_id "Novel_000673"; exon_number "1"; gene_type "
At_chr2	Cufflinks	exon	10995281	10996946	.	+	.	gene_id "XLOC_050666"; transcript_id "Novel_000673"; exon_number "2"; gene_type "
At_chr2	Cufflinks	exon	11028197	11028364	.	+	.	gene_id "XLOC_050671"; transcript_id "Novel_000674"; exon_number "1"; gene_type "

At_chr2	Cufflinks	exon	11029010	11031157	.	+	.	gene_id "XLOC_050671"; transcript_id "Novel_000674"; exon_number "2"; gene_type "
At_chr2	Cufflinks	exon	12495641	12496190	.	+	.	gene_id "XLOC_050825"; transcript_id "Novel_000675"; exon_number "1"; gene_type "
At_chr2	Cufflinks	exon	12496285	12496728	.	+	.	gene_id "XLOC_050825"; transcript_id "Novel_000675"; exon_number "2"; gene_type "
At_chr2	Cufflinks	exon	12496262	12496734	.	+	.	gene_id "XLOC_050825"; transcript_id "Novel_000676"; exon_number "1"; gene_type "
At_chr2	Cufflinks	exon	12496814	12497123	.	+	.	gene_id "XLOC_050825"; transcript_id "Novel_000676"; exon_number "2"; gene_type "
At_chr2	Cufflinks	exon	12497238	12497741	.	+	.	gene_id "XLOC_050825"; transcript_id "Novel_000676"; exon_number "3"; gene_type "
At_chr2	Cufflinks	exon	13031242	13031839	.	+	.	gene_id "XLOC_050898"; transcript_id "Novel_000677"; exon_number "1"; gene_type "
At_chr2	Cufflinks	exon	13032651	13032697	.	+	.	gene_id "XLOC_050898"; transcript_id "Novel_000677"; exon_number "2"; gene_type "
At_chr2	Cufflinks	exon	13032781	13034829	.	+	.	gene_id "XLOC_050898"; transcript_id "Novel_000677"; exon_number "3"; gene_type "
At_chr2	Cufflinks	exon	13031260	13031839	.	+	.	gene_id "XLOC_050898"; transcript_id "Novel_000678"; exon_number "1"; gene_type "
At_chr2	Cufflinks	exon	13032651	13034482	.	+	.	gene_id "XLOC_050898"; transcript_id "Novel_000678"; exon_number "2"; gene_type "
At_chr2	Cufflinks	exon	14088237	14088465	.	+	.	gene_id "XLOC_050974"; transcript_id "Novel_000679"; exon_number "1"; gene_type "
At_chr2	Cufflinks	exon	16350246	16350508	.	+	.	gene_id "XLOC_051117"; transcript_id "Novel_000680"; exon_number "1"; gene_type "
At_chr2	Cufflinks	exon	16350590	16350630	.	+	.	gene_id "XLOC_051117"; transcript_id "Novel_000680"; exon_number "2"; gene_type "
At_chr2	Cufflinks	exon	29716885	29717109	.	+	.	gene_id "XLOC_051751"; transcript_id "Novel_000681"; exon_number "1"; gene_type "
At_chr2	Cufflinks	exon	29717248	29717330	.	+	.	gene_id "XLOC_051751"; transcript_id "Novel_000681"; exon_number "2"; gene_type "
At_chr2	Cufflinks	exon	29717410	29717670	.	+	.	gene_id "XLOC_051751"; transcript_id "Novel_000681"; exon_number "3"; gene_type "
At_chr2	Cufflinks	exon	29717781	29718215	.	+	.	gene_id "XLOC_051751"; transcript_id "Novel_000681"; exon_number "4"; gene_type "
At_chr2	Cufflinks	exon	31802701	31802812	.	+	.	gene_id "XLOC_051873"; transcript_id "Novel_000682"; exon_number "1"; gene_type "
At_chr2	Cufflinks	exon	31802890	31802978	.	+	.	gene_id "XLOC_051873"; transcript_id "Novel_000682"; exon_number "2"; gene_type "
At_chr2	Cufflinks	exon	37068541	37068746	.	+	.	gene_id "XLOC_052140"; transcript_id "Novel_000683"; exon_number "1"; gene_type "
At_chr2	Cufflinks	exon	37070144	37072101	.	+	.	gene_id "XLOC_052140"; transcript_id "Novel_000683"; exon_number "2"; gene_type "
At_chr2	Cufflinks	exon	39090575	39090599	.	+	.	gene_id "XLOC_052197"; transcript_id "Novel_000684"; exon_number "1"; gene_type "
At_chr2	Cufflinks	exon	39090770	39090861	.	+	.	gene_id "XLOC_052197"; transcript_id "Novel_000684"; exon_number "2"; gene_type "
At_chr2	Cufflinks	exon	39091367	39091622	.	+	.	gene_id "XLOC_052197"; transcript_id "Novel_000684"; exon_number "3"; gene_type "
At_chr2	Cufflinks	exon	42023352	42024216	.	+	.	gene_id "XLOC_052308"; transcript_id "Novel_000685"; exon_number "1"; gene_type "
At_chr2	Cufflinks	exon	42024276	42026384	.	+	.	gene_id "XLOC_052308"; transcript_id "Novel_000685"; exon_number "2"; gene_type "
At_chr2	Cufflinks	exon	49252746	49253118	.	+	.	gene_id "XLOC_052637"; transcript_id "Novel_000686"; exon_number "1"; gene_type "
At_chr2	Cufflinks	exon	49253823	49254240	.	+	.	gene_id "XLOC_052637"; transcript_id "Novel_000686"; exon_number "2"; gene_type "
At_chr2	Cufflinks	exon	58761009	58761086	.	+	.	gene_id "XLOC_053117"; transcript_id "Novel_000687"; exon_number "1"; gene_type "
At_chr2	Cufflinks	exon	58761192	58761425	.	+	.	gene_id "XLOC_053117"; transcript_id "Novel_000687"; exon_number "2"; gene_type "
At_chr2	Cufflinks	exon	58761791	58762779	.	+	.	gene_id "XLOC_053117"; transcript_id "Novel_000687"; exon_number "3"; gene_type "
At_chr2	Cufflinks	exon	60688907	60689686	.	+	.	gene_id "XLOC_053204"; transcript_id "Novel_000688"; exon_number "1"; gene_type "
At_chr2	Cufflinks	exon	60689791	60690261	.	+	.	gene_id "XLOC_053204"; transcript_id "Novel_000688"; exon_number "2"; gene_type "
At_chr2	Cufflinks	exon	60937597	60937821	.	+	.	gene_id "XLOC_053232"; transcript_id "Novel_000689"; exon_number "1"; gene_type "

At_chr2	Cufflinks	exon	1700697	1700765	.	-	.	gene_id "XLOC_053326"; transcript_id "Novel_000690"; exon_number "1"; gene_type "
At_chr2	Cufflinks	exon	1700894	1700956	.	-	.	gene_id "XLOC_053326"; transcript_id "Novel_000690"; exon_number "2"; gene_type "
At_chr2	Cufflinks	exon	1701063	1701133	.	-	.	gene_id "XLOC_053326"; transcript_id "Novel_000690"; exon_number "3"; gene_type "
At_chr2	Cufflinks	exon	1701222	1701330	.	-	.	gene_id "XLOC_053326"; transcript_id "Novel_000690"; exon_number "4"; gene_type "
At_chr2	Cufflinks	exon	1702037	1702114	.	-	.	gene_id "XLOC_053326"; transcript_id "Novel_000690"; exon_number "5"; gene_type "
At_chr2	Cufflinks	exon	1703807	1704125	.	-	.	gene_id "XLOC_053326"; transcript_id "Novel_000690"; exon_number "6"; gene_type "
At_chr2	Cufflinks	exon	5406066	5406748	.	-	.	gene_id "XLOC_053546"; transcript_id "Novel_000691"; exon_number "1"; gene_type "
At_chr2	Cufflinks	exon	5406824	5406985	.	-	.	gene_id "XLOC_053546"; transcript_id "Novel_000691"; exon_number "2"; gene_type "
At_chr2	Cufflinks	exon	5407068	5407252	.	-	.	gene_id "XLOC_053546"; transcript_id "Novel_000691"; exon_number "3"; gene_type "
At_chr2	Cufflinks	exon	5636604	5637269	.	-	.	gene_id "XLOC_053580"; transcript_id "Novel_000692"; exon_number "1"; gene_type "
At_chr2	Cufflinks	exon	5637370	5637935	.	-	.	gene_id "XLOC_053580"; transcript_id "Novel_000692"; exon_number "2"; gene_type "
At_chr2	Cufflinks	exon	5951429	5951660	.	-	.	gene_id "XLOC_053619"; transcript_id "Novel_000693"; exon_number "1"; gene_type "
At_chr2	Cufflinks	exon	7473243	7474324	.	-	.	gene_id "XLOC_053819"; transcript_id "Novel_000694"; exon_number "1"; gene_type "
At_chr2	Cufflinks	exon	7475335	7475369	.	-	.	gene_id "XLOC_053819"; transcript_id "Novel_000694"; exon_number "2"; gene_type "
At_chr2	Cufflinks	exon	7475461	7475544	.	-	.	gene_id "XLOC_053819"; transcript_id "Novel_000694"; exon_number "3"; gene_type "
At_chr2	Cufflinks	exon	7475665	7475727	.	-	.	gene_id "XLOC_053819"; transcript_id "Novel_000694"; exon_number "4"; gene_type "
At_chr2	Cufflinks	exon	7476713	7476750	.	-	.	gene_id "XLOC_053819"; transcript_id "Novel_000694"; exon_number "5"; gene_type "
At_chr2	Cufflinks	exon	7477170	7477364	.	-	.	gene_id "XLOC_053819"; transcript_id "Novel_000694"; exon_number "6"; gene_type "
At_chr2	Cufflinks	exon	7473271	7473310	.	-	.	gene_id "XLOC_053819"; transcript_id "Novel_000695"; exon_number "1"; gene_type "
At_chr2	Cufflinks	exon	7474239	7474324	.	-	.	gene_id "XLOC_053819"; transcript_id "Novel_000695"; exon_number "2"; gene_type "
At_chr2	Cufflinks	exon	7475335	7475369	.	-	.	gene_id "XLOC_053819"; transcript_id "Novel_000695"; exon_number "3"; gene_type "
At_chr2	Cufflinks	exon	7475461	7475544	.	-	.	gene_id "XLOC_053819"; transcript_id "Novel_000695"; exon_number "4"; gene_type "
At_chr2	Cufflinks	exon	7475665	7475721	.	-	.	gene_id "XLOC_053819"; transcript_id "Novel_000695"; exon_number "5"; gene_type "
At_chr2	Cufflinks	exon	7476713	7476750	.	-	.	gene_id "XLOC_053819"; transcript_id "Novel_000695"; exon_number "6"; gene_type "
At_chr2	Cufflinks	exon	7477170	7477274	.	-	.	gene_id "XLOC_053819"; transcript_id "Novel_000695"; exon_number "7"; gene_type "
At_chr2	Cufflinks	exon	8088651	8089467	.	-	.	gene_id "XLOC_053892"; transcript_id "Novel_000696"; exon_number "1"; gene_type "
At_chr2	Cufflinks	exon	8089573	8089660	.	-	.	gene_id "XLOC_053892"; transcript_id "Novel_000696"; exon_number "2"; gene_type "
At_chr2	Cufflinks	exon	8139074	8139442	.	-	.	gene_id "XLOC_053897"; transcript_id "Novel_000697"; exon_number "1"; gene_type "
At_chr2	Cufflinks	exon	11459459	11459835	.	-	.	gene_id "XLOC_054161"; transcript_id "Novel_000698"; exon_number "1"; gene_type "
At_chr2	Cufflinks	exon	11460427	11460810	.	-	.	gene_id "XLOC_054161"; transcript_id "Novel_000698"; exon_number "2"; gene_type "
At_chr2	Cufflinks	exon	11466242	11466460	.	-	.	gene_id "XLOC_054164"; transcript_id "Novel_000699"; exon_number "1"; gene_type "
At_chr2	Cufflinks	exon	11530459	11530741	.	-	.	gene_id "XLOC_054171"; transcript_id "Novel_000700"; exon_number "1"; gene_type "
At_chr2	Cufflinks	exon	11530818	11530876	.	-	.	gene_id "XLOC_054171"; transcript_id "Novel_000700"; exon_number "2"; gene_type "
At_chr2	Cufflinks	exon	17134809	17135827	.	-	.	gene_id "XLOC_054534"; transcript_id "Novel_000701"; exon_number "1"; gene_type "
At_chr2	Cufflinks	exon	17136150	17136209	.	-	.	gene_id "XLOC_054534"; transcript_id "Novel_000701"; exon_number "2"; gene_type "

At_chr2	Cufflinks	exon	17136314	17136463	.	-	.	gene_id "XLOC_054534"; transcript_id "Novel_000701"; exon_number "3"; gene_type "
At_chr2	Cufflinks	exon	17136554	17136640	.	-	.	gene_id "XLOC_054534"; transcript_id "Novel_000701"; exon_number "4"; gene_type "
At_chr2	Cufflinks	exon	17136713	17136783	.	-	.	gene_id "XLOC_054534"; transcript_id "Novel_000701"; exon_number "5"; gene_type "
At_chr2	Cufflinks	exon	17136862	17136946	.	-	.	gene_id "XLOC_054534"; transcript_id "Novel_000701"; exon_number "6"; gene_type "
At_chr2	Cufflinks	exon	17134809	17135827	.	-	.	gene_id "XLOC_054534"; transcript_id "Novel_000702"; exon_number "1"; gene_type "
At_chr2	Cufflinks	exon	17136150	17136209	.	-	.	gene_id "XLOC_054534"; transcript_id "Novel_000702"; exon_number "2"; gene_type "
At_chr2	Cufflinks	exon	17136314	17136463	.	-	.	gene_id "XLOC_054534"; transcript_id "Novel_000702"; exon_number "3"; gene_type "
At_chr2	Cufflinks	exon	17136554	17136783	.	-	.	gene_id "XLOC_054534"; transcript_id "Novel_000702"; exon_number "4"; gene_type "
At_chr2	Cufflinks	exon	17136862	17136955	.	-	.	gene_id "XLOC_054534"; transcript_id "Novel_000702"; exon_number "5"; gene_type "
At_chr2	Cufflinks	exon	17765029	17765204	.	-	.	gene_id "XLOC_054566"; transcript_id "Novel_000703"; exon_number "1"; gene_type "
At_chr2	Cufflinks	exon	17765304	17765359	.	-	.	gene_id "XLOC_054566"; transcript_id "Novel_000703"; exon_number "2"; gene_type "
At_chr2	Cufflinks	exon	17765520	17766292	.	-	.	gene_id "XLOC_054566"; transcript_id "Novel_000704"; exon_number "1"; gene_type "
At_chr2	Cufflinks	exon	17767058	17767594	.	-	.	gene_id "XLOC_054566"; transcript_id "Novel_000704"; exon_number "2"; gene_type "
At_chr2	Cufflinks	exon	19991431	19992043	.	-	.	gene_id "XLOC_054726"; transcript_id "Novel_000705"; exon_number "1"; gene_type "
At_chr2	Cufflinks	exon	19993857	19993985	.	-	.	gene_id "XLOC_054726"; transcript_id "Novel_000705"; exon_number "2"; gene_type "
At_chr2	Cufflinks	exon	19994102	19994149	.	-	.	gene_id "XLOC_054726"; transcript_id "Novel_000705"; exon_number "3"; gene_type "
At_chr2	Cufflinks	exon	19994314	19994511	.	-	.	gene_id "XLOC_054726"; transcript_id "Novel_000705"; exon_number "4"; gene_type "
At_chr2	Cufflinks	exon	22394525	22396910	.	-	.	gene_id "XLOC_054806"; transcript_id "Novel_000706"; exon_number "1"; gene_type "
At_chr2	Cufflinks	exon	22398128	22398302	.	-	.	gene_id "XLOC_054806"; transcript_id "Novel_000706"; exon_number "2"; gene_type "
At_chr2	Cufflinks	exon	22955227	22955492	.	-	.	gene_id "XLOC_054831"; transcript_id "Novel_000707"; exon_number "1"; gene_type "
At_chr2	Cufflinks	exon	22956555	22956697	.	-	.	gene_id "XLOC_054831"; transcript_id "Novel_000707"; exon_number "2"; gene_type "
At_chr2	Cufflinks	exon	22956835	22956847	.	-	.	gene_id "XLOC_054831"; transcript_id "Novel_000707"; exon_number "3"; gene_type "
At_chr2	Cufflinks	exon	22955286	22955492	.	-	.	gene_id "XLOC_054831"; transcript_id "Novel_000708"; exon_number "1"; gene_type "
At_chr2	Cufflinks	exon	22956555	22956847	.	-	.	gene_id "XLOC_054831"; transcript_id "Novel_000708"; exon_number "2"; gene_type "
At_chr2	Cufflinks	exon	22956952	22957049	.	-	.	gene_id "XLOC_054833"; transcript_id "Novel_000709"; exon_number "1"; gene_type "
At_chr2	Cufflinks	exon	22957617	22957710	.	-	.	gene_id "XLOC_054833"; transcript_id "Novel_000709"; exon_number "2"; gene_type "
At_chr2	Cufflinks	exon	22957985	22958218	.	-	.	gene_id "XLOC_054833"; transcript_id "Novel_000709"; exon_number "3"; gene_type "
At_chr2	Cufflinks	exon	22958317	22958640	.	-	.	gene_id "XLOC_054833"; transcript_id "Novel_000709"; exon_number "4"; gene_type "
At_chr2	Cufflinks	exon	22957193	22957710	.	-	.	gene_id "XLOC_054833"; transcript_id "Novel_000710"; exon_number "1"; gene_type "
At_chr2	Cufflinks	exon	22957985	22958218	.	-	.	gene_id "XLOC_054833"; transcript_id "Novel_000710"; exon_number "2"; gene_type "
At_chr2	Cufflinks	exon	22958317	22958640	.	-	.	gene_id "XLOC_054833"; transcript_id "Novel_000710"; exon_number "3"; gene_type "
At_chr2	Cufflinks	exon	22957719	22958218	.	-	.	gene_id "XLOC_054833"; transcript_id "Novel_000711"; exon_number "1"; gene_type "
At_chr2	Cufflinks	exon	22958317	22958640	.	-	.	gene_id "XLOC_054833"; transcript_id "Novel_000711"; exon_number "2"; gene_type "
At_chr2	Cufflinks	exon	23230164	23230825	.	-	.	gene_id "XLOC_054857"; transcript_id "Novel_000712"; exon_number "1"; gene_type "
At_chr2	Cufflinks	exon	23230898	23231018	.	-	.	gene_id "XLOC_054857"; transcript_id "Novel_000712"; exon_number "2"; gene_type "

At_chr2	Cufflinks	exon	23232288	23232353	.	-	.	gene_id "XLOC_054857"; transcript_id "Novel_000712"; exon_number "3"; gene_type "
At_chr2	Cufflinks	exon	23232529	23232684	.	-	.	gene_id "XLOC_054857"; transcript_id "Novel_000712"; exon_number "4"; gene_type "
At_chr2	Cufflinks	exon	23233876	23234029	.	-	.	gene_id "XLOC_054857"; transcript_id "Novel_000712"; exon_number "5"; gene_type "
At_chr2	Cufflinks	exon	23230164	23230825	.	-	.	gene_id "XLOC_054857"; transcript_id "Novel_000713"; exon_number "1"; gene_type "
At_chr2	Cufflinks	exon	23230898	23231018	.	-	.	gene_id "XLOC_054857"; transcript_id "Novel_000713"; exon_number "2"; gene_type "
At_chr2	Cufflinks	exon	23232284	23232353	.	-	.	gene_id "XLOC_054857"; transcript_id "Novel_000713"; exon_number "3"; gene_type "
At_chr2	Cufflinks	exon	23232529	23232684	.	-	.	gene_id "XLOC_054857"; transcript_id "Novel_000713"; exon_number "4"; gene_type "
At_chr2	Cufflinks	exon	23233876	23234029	.	-	.	gene_id "XLOC_054857"; transcript_id "Novel_000713"; exon_number "5"; gene_type "
At_chr2	Cufflinks	exon	28119366	28120495	.	-	.	gene_id "XLOC_055062"; transcript_id "Novel_000714"; exon_number "1"; gene_type "
At_chr2	Cufflinks	exon	28120653	28120871	.	-	.	gene_id "XLOC_055062"; transcript_id "Novel_000714"; exon_number "2"; gene_type "
At_chr2	Cufflinks	exon	28121486	28121515	.	-	.	gene_id "XLOC_055062"; transcript_id "Novel_000714"; exon_number "3"; gene_type "
At_chr2	Cufflinks	exon	28121582	28121683	.	-	.	gene_id "XLOC_055062"; transcript_id "Novel_000714"; exon_number "4"; gene_type "
At_chr2	Cufflinks	exon	28121977	28122057	.	-	.	gene_id "XLOC_055062"; transcript_id "Novel_000714"; exon_number "5"; gene_type "
At_chr2	Cufflinks	exon	28122649	28122750	.	-	.	gene_id "XLOC_055062"; transcript_id "Novel_000714"; exon_number "6"; gene_type "
At_chr2	Cufflinks	exon	28122847	28123258	.	-	.	gene_id "XLOC_055062"; transcript_id "Novel_000714"; exon_number "7"; gene_type "
At_chr2	Cufflinks	exon	28119366	28120495	.	-	.	gene_id "XLOC_055062"; transcript_id "Novel_000715"; exon_number "1"; gene_type "
At_chr2	Cufflinks	exon	28120653	28120871	.	-	.	gene_id "XLOC_055062"; transcript_id "Novel_000715"; exon_number "2"; gene_type "
At_chr2	Cufflinks	exon	28121486	28121500	.	-	.	gene_id "XLOC_055062"; transcript_id "Novel_000715"; exon_number "3"; gene_type "
At_chr2	Cufflinks	exon	28121567	28121683	.	-	.	gene_id "XLOC_055062"; transcript_id "Novel_000715"; exon_number "4"; gene_type "
At_chr2	Cufflinks	exon	28121977	28122057	.	-	.	gene_id "XLOC_055062"; transcript_id "Novel_000715"; exon_number "5"; gene_type "
At_chr2	Cufflinks	exon	28122649	28122750	.	-	.	gene_id "XLOC_055062"; transcript_id "Novel_000715"; exon_number "6"; gene_type "
At_chr2	Cufflinks	exon	28122847	28123258	.	-	.	gene_id "XLOC_055062"; transcript_id "Novel_000715"; exon_number "7"; gene_type "
At_chr2	Cufflinks	exon	28121287	28121500	.	-	.	gene_id "XLOC_055062"; transcript_id "Novel_000716"; exon_number "1"; gene_type "
At_chr2	Cufflinks	exon	28121567	28121683	.	-	.	gene_id "XLOC_055062"; transcript_id "Novel_000716"; exon_number "2"; gene_type "
At_chr2	Cufflinks	exon	28121977	28122057	.	-	.	gene_id "XLOC_055062"; transcript_id "Novel_000716"; exon_number "3"; gene_type "
At_chr2	Cufflinks	exon	28122649	28122750	.	-	.	gene_id "XLOC_055062"; transcript_id "Novel_000716"; exon_number "4"; gene_type "
At_chr2	Cufflinks	exon	28122847	28123296	.	-	.	gene_id "XLOC_055062"; transcript_id "Novel_000716"; exon_number "5"; gene_type "
At_chr2	Cufflinks	exon	39550619	39552048	.	-	.	gene_id "XLOC_055545"; transcript_id "Novel_000717"; exon_number "1"; gene_type "
At_chr2	Cufflinks	exon	39552713	39552820	.	-	.	gene_id "XLOC_055545"; transcript_id "Novel_000717"; exon_number "2"; gene_type "
At_chr2	Cufflinks	exon	47183281	47183768	.	-	.	gene_id "XLOC_055904"; transcript_id "Novel_000718"; exon_number "1"; gene_type "
At_chr2	Cufflinks	exon	47184165	47185478	.	-	.	gene_id "XLOC_055904"; transcript_id "Novel_000718"; exon_number "2"; gene_type "
At_chr2	Cufflinks	exon	56048536	56048930	.	-	.	gene_id "XLOC_056304"; transcript_id "Novel_000719"; exon_number "1"; gene_type "
At_chr2	Cufflinks	exon	56049100	56049219	.	-	.	gene_id "XLOC_056304"; transcript_id "Novel_000719"; exon_number "2"; gene_type "
At_chr2	Cufflinks	exon	58609962	58610019	.	-	.	gene_id "XLOC_056475"; transcript_id "Novel_000720"; exon_number "1"; gene_type "
At_chr2	Cufflinks	exon	58610134	58610362	.	-	.	gene_id "XLOC_056475"; transcript_id "Novel_000720"; exon_number "2"; gene_type "

At_chr2	Cufflinks	exon	59790033	59790409	.	-	.	gene_id "XLOC_056547"; transcript_id "Novel_000721"; exon_number "1"; gene_type "
At_chr2	Cufflinks	exon	59790865	59790963	.	-	.	gene_id "XLOC_056547"; transcript_id "Novel_000721"; exon_number "2"; gene_type "
At_chr2	Cufflinks	exon	59791060	59791116	.	-	.	gene_id "XLOC_056547"; transcript_id "Novel_000721"; exon_number "3"; gene_type "
At_chr2	Cufflinks	exon	59791207	59791321	.	-	.	gene_id "XLOC_056547"; transcript_id "Novel_000721"; exon_number "4"; gene_type "
At_chr2	Cufflinks	exon	59791402	59791467	.	-	.	gene_id "XLOC_056547"; transcript_id "Novel_000721"; exon_number "5"; gene_type "
At_chr2	Cufflinks	exon	59791557	59791601	.	-	.	gene_id "XLOC_056547"; transcript_id "Novel_000721"; exon_number "6"; gene_type "
At_chr2	Cufflinks	exon	59791740	59791779	.	-	.	gene_id "XLOC_056547"; transcript_id "Novel_000721"; exon_number "7"; gene_type "
At_chr2	Cufflinks	exon	59792017	59792066	.	-	.	gene_id "XLOC_056547"; transcript_id "Novel_000721"; exon_number "8"; gene_type "
At_chr2	Cufflinks	exon	59792156	59792240	.	-	.	gene_id "XLOC_056547"; transcript_id "Novel_000721"; exon_number "9"; gene_type "
At_chr2	Cufflinks	exon	59792367	59792461	.	-	.	gene_id "XLOC_056547"; transcript_id "Novel_000721"; exon_number "10"; gene_type
At_chr2	Cufflinks	exon	59792629	59792729	.	-	.	gene_id "XLOC_056547"; transcript_id "Novel_000721"; exon_number "11"; gene_type
At_chr3	Cufflinks	exon	9745586	9745904	.	+	.	gene_id "XLOC_057086"; transcript_id "Novel_000722"; exon_number "1"; gene_type "
At_chr3	Cufflinks	exon	9745992	9746065	.	+	.	gene_id "XLOC_057086"; transcript_id "Novel_000722"; exon_number "2"; gene_type "
At_chr3	Cufflinks	exon	9746222	9746354	.	+	.	gene_id "XLOC_057086"; transcript_id "Novel_000722"; exon_number "3"; gene_type "
At_chr3	Cufflinks	exon	9746761	9747026	.	+	.	gene_id "XLOC_057086"; transcript_id "Novel_000722"; exon_number "4"; gene_type "
At_chr3	Cufflinks	exon	11836932	11838265	.	+	.	gene_id "XLOC_057166"; transcript_id "Novel_000723"; exon_number "1"; gene_type "
At_chr3	Cufflinks	exon	11838377	11838493	.	+	.	gene_id "XLOC_057166"; transcript_id "Novel_000723"; exon_number "2"; gene_type "
At_chr3	Cufflinks	exon	11839895	11841387	.	+	.	gene_id "XLOC_057166"; transcript_id "Novel_000723"; exon_number "3"; gene_type "
At_chr3	Cufflinks	exon	19257346	19257577	.	+	.	gene_id "XLOC_057454"; transcript_id "Novel_000724"; exon_number "1"; gene_type "
At_chr3	Cufflinks	exon	19957320	19957344	.	+	.	gene_id "XLOC_057577"; transcript_id "Novel_000725"; exon_number "1"; gene_type "
At_chr3	Cufflinks	exon	19957494	19957575	.	+	.	gene_id "XLOC_057577"; transcript_id "Novel_000725"; exon_number "2"; gene_type "
At_chr3	Cufflinks	exon	19957704	19958040	.	+	.	gene_id "XLOC_057577"; transcript_id "Novel_000725"; exon_number "3"; gene_type "
At_chr3	Cufflinks	exon	20038576	20038793	.	+	.	gene_id "XLOC_057593"; transcript_id "Novel_000726"; exon_number "1"; gene_type "
At_chr3	Cufflinks	exon	20040236	20042009	.	+	.	gene_id "XLOC_057593"; transcript_id "Novel_000726"; exon_number "2"; gene_type "
At_chr3	Cufflinks	exon	27093697	27094228	.	+	.	gene_id "XLOC_057845"; transcript_id "Novel_000727"; exon_number "1"; gene_type "
At_chr3	Cufflinks	exon	27094760	27094842	.	+	.	gene_id "XLOC_057845"; transcript_id "Novel_000727"; exon_number "2"; gene_type "
At_chr3	Cufflinks	exon	27094967	27095062	.	+	.	gene_id "XLOC_057845"; transcript_id "Novel_000727"; exon_number "3"; gene_type "
At_chr3	Cufflinks	exon	27095810	27095978	.	+	.	gene_id "XLOC_057845"; transcript_id "Novel_000727"; exon_number "4"; gene_type "
At_chr3	Cufflinks	exon	27096059	27096201	.	+	.	gene_id "XLOC_057845"; transcript_id "Novel_000727"; exon_number "5"; gene_type "
At_chr3	Cufflinks	exon	27096273	27096359	.	+	.	gene_id "XLOC_057845"; transcript_id "Novel_000727"; exon_number "6"; gene_type "
At_chr3	Cufflinks	exon	27096575	27096760	.	+	.	gene_id "XLOC_057845"; transcript_id "Novel_000727"; exon_number "7"; gene_type "
At_chr3	Cufflinks	exon	27098107	27099003	.	+	.	gene_id "XLOC_057845"; transcript_id "Novel_000727"; exon_number "8"; gene_type "
At_chr3	Cufflinks	exon	27261786	27262052	.	+	.	gene_id "XLOC_057855"; transcript_id "Novel_000728"; exon_number "1"; gene_type "
At_chr3	Cufflinks	exon	27262177	27262255	.	+	.	gene_id "XLOC_057855"; transcript_id "Novel_000728"; exon_number "2"; gene_type "
At_chr3	Cufflinks	exon	27263127	27264125	.	+	.	gene_id "XLOC_057855"; transcript_id "Novel_000728"; exon_number "3"; gene_type "

At_chr3	Cufflinks	exon	27261786	27262052	.	+	.	gene_id "XLOC_057855"; transcript_id "Novel_000729"; exon_number "1"; gene_type "
At_chr3	Cufflinks	exon	27262177	27262255	.	+	.	gene_id "XLOC_057855"; transcript_id "Novel_000729"; exon_number "2"; gene_type "
At_chr3	Cufflinks	exon	27263127	27263195	.	+	.	gene_id "XLOC_057855"; transcript_id "Novel_000729"; exon_number "3"; gene_type "
At_chr3	Cufflinks	exon	27263311	27263476	.	+	.	gene_id "XLOC_057855"; transcript_id "Novel_000729"; exon_number "4"; gene_type "
At_chr3	Cufflinks	exon	27263578	27263671	.	+	.	gene_id "XLOC_057855"; transcript_id "Novel_000729"; exon_number "5"; gene_type "
At_chr3	Cufflinks	exon	27279206	27279602	.	+	.	gene_id "XLOC_057855"; transcript_id "Novel_000729"; exon_number "6"; gene_type "
At_chr3	Cufflinks	exon	27295678	27295955	.	+	.	gene_id "XLOC_057864"; transcript_id "Novel_000730"; exon_number "1"; gene_type "
At_chr3	Cufflinks	exon	27310760	27311133	.	+	.	gene_id "XLOC_057868"; transcript_id "Novel_000731"; exon_number "1"; gene_type "
At_chr3	Cufflinks	exon	27312380	27312814	.	+	.	gene_id "XLOC_057869"; transcript_id "Novel_000732"; exon_number "1"; gene_type "
At_chr3	Cufflinks	exon	30334782	30334961	.	+	.	gene_id "XLOC_058010"; transcript_id "Novel_000733"; exon_number "1"; gene_type "
At_chr3	Cufflinks	exon	30335779	30336187	.	+	.	gene_id "XLOC_058010"; transcript_id "Novel_000733"; exon_number "2"; gene_type "
At_chr3	Cufflinks	exon	30336891	30337508	.	+	.	gene_id "XLOC_058010"; transcript_id "Novel_000733"; exon_number "3"; gene_type "
At_chr3	Cufflinks	exon	30334898	30335000	.	+	.	gene_id "XLOC_058010"; transcript_id "Novel_000734"; exon_number "1"; gene_type "
At_chr3	Cufflinks	exon	30335057	30337620	.	+	.	gene_id "XLOC_058010"; transcript_id "Novel_000734"; exon_number "2"; gene_type "
At_chr3	Cufflinks	exon	30335037	30336187	.	+	.	gene_id "XLOC_058010"; transcript_id "Novel_000735"; exon_number "1"; gene_type "
At_chr3	Cufflinks	exon	30336891	30337508	.	+	.	gene_id "XLOC_058010"; transcript_id "Novel_000735"; exon_number "2"; gene_type "
At_chr3	Cufflinks	exon	36817812	36818084	.	+	.	gene_id "XLOC_058276"; transcript_id "Novel_000736"; exon_number "1"; gene_type "
At_chr3	Cufflinks	exon	36819250	36819508	.	+	.	gene_id "XLOC_058276"; transcript_id "Novel_000736"; exon_number "2"; gene_type "
At_chr3	Cufflinks	exon	36819707	36819818	.	+	.	gene_id "XLOC_058276"; transcript_id "Novel_000736"; exon_number "3"; gene_type "
At_chr3	Cufflinks	exon	36820123	36820168	.	+	.	gene_id "XLOC_058276"; transcript_id "Novel_000736"; exon_number "4"; gene_type "
At_chr3	Cufflinks	exon	36820250	36820364	.	+	.	gene_id "XLOC_058276"; transcript_id "Novel_000736"; exon_number "5"; gene_type "
At_chr3	Cufflinks	exon	36820623	36820690	.	+	.	gene_id "XLOC_058276"; transcript_id "Novel_000736"; exon_number "6"; gene_type "
At_chr3	Cufflinks	exon	36820843	36821680	.	+	.	gene_id "XLOC_058276"; transcript_id "Novel_000736"; exon_number "7"; gene_type "
At_chr3	Cufflinks	exon	36821765	36822302	.	+	.	gene_id "XLOC_058276"; transcript_id "Novel_000736"; exon_number "8"; gene_type "
At_chr3	Cufflinks	exon	36817812	36818084	.	+	.	gene_id "XLOC_058276"; transcript_id "Novel_000737"; exon_number "1"; gene_type "
At_chr3	Cufflinks	exon	36819250	36819508	.	+	.	gene_id "XLOC_058276"; transcript_id "Novel_000737"; exon_number "2"; gene_type "
At_chr3	Cufflinks	exon	36819707	36819818	.	+	.	gene_id "XLOC_058276"; transcript_id "Novel_000737"; exon_number "3"; gene_type "
At_chr3	Cufflinks	exon	36820033	36820168	.	+	.	gene_id "XLOC_058276"; transcript_id "Novel_000737"; exon_number "4"; gene_type "
At_chr3	Cufflinks	exon	36820250	36820364	.	+	.	gene_id "XLOC_058276"; transcript_id "Novel_000737"; exon_number "5"; gene_type "
At_chr3	Cufflinks	exon	36820623	36820690	.	+	.	gene_id "XLOC_058276"; transcript_id "Novel_000737"; exon_number "6"; gene_type "
At_chr3	Cufflinks	exon	36820843	36821680	.	+	.	gene_id "XLOC_058276"; transcript_id "Novel_000737"; exon_number "7"; gene_type "
At_chr3	Cufflinks	exon	36821765	36822302	.	+	.	gene_id "XLOC_058276"; transcript_id "Novel_000737"; exon_number "8"; gene_type "
At_chr3	Cufflinks	exon	36817812	36818084	.	+	.	gene_id "XLOC_058276"; transcript_id "Novel_000738"; exon_number "1"; gene_type "
At_chr3	Cufflinks	exon	36819250	36819508	.	+	.	gene_id "XLOC_058276"; transcript_id "Novel_000738"; exon_number "2"; gene_type "
At_chr3	Cufflinks	exon	36819707	36820168	.	+	.	gene_id "XLOC_058276"; transcript_id "Novel_000738"; exon_number "3"; gene_type "

At_chr3	Cufflinks	exon	36820250	36820690	.	+	.	gene_id "XLOC_058276"; transcript_id "Novel_000738"; exon_number "4"; gene_type "
At_chr3	Cufflinks	exon	36820843	36821680	.	+	.	gene_id "XLOC_058276"; transcript_id "Novel_000738"; exon_number "5"; gene_type "
At_chr3	Cufflinks	exon	36821765	36822302	.	+	.	gene_id "XLOC_058276"; transcript_id "Novel_000738"; exon_number "6"; gene_type "
At_chr3	Cufflinks	exon	36865590	36865920	.	+	.	gene_id "XLOC_058283"; transcript_id "Novel_000739"; exon_number "1"; gene_type "
At_chr3	Cufflinks	exon	36866587	36866910	.	+	.	gene_id "XLOC_058283"; transcript_id "Novel_000739"; exon_number "2"; gene_type "
At_chr3	Cufflinks	exon	38072341	38072616	.	+	.	gene_id "XLOC_058354"; transcript_id "Novel_000740"; exon_number "1"; gene_type "
At_chr3	Cufflinks	exon	38073613	38074957	.	+	.	gene_id "XLOC_058354"; transcript_id "Novel_000740"; exon_number "2"; gene_type "
At_chr3	Cufflinks	exon	41084988	41086386	.	+	.	gene_id "XLOC_058633"; transcript_id "Novel_000741"; exon_number "1"; gene_type "
At_chr3	Cufflinks	exon	41086519	41087194	.	+	.	gene_id "XLOC_058633"; transcript_id "Novel_000741"; exon_number "2"; gene_type "
At_chr3	Cufflinks	exon	41084988	41086386	.	+	.	gene_id "XLOC_058633"; transcript_id "Novel_000742"; exon_number "1"; gene_type "
At_chr3	Cufflinks	exon	41086519	41086622	.	+	.	gene_id "XLOC_058633"; transcript_id "Novel_000742"; exon_number "2"; gene_type "
At_chr3	Cufflinks	exon	41086905	41087194	.	+	.	gene_id "XLOC_058633"; transcript_id "Novel_000742"; exon_number "3"; gene_type "
At_chr3	Cufflinks	exon	41416973	41417227	.	+	.	gene_id "XLOC_058662"; transcript_id "Novel_000743"; exon_number "1"; gene_type "
At_chr3	Cufflinks	exon	41418081	41418150	.	+	.	gene_id "XLOC_058662"; transcript_id "Novel_000743"; exon_number "2"; gene_type "
At_chr3	Cufflinks	exon	41429570	41430575	.	+	.	gene_id "XLOC_058662"; transcript_id "Novel_000743"; exon_number "3"; gene_type "
At_chr3	Cufflinks	exon	41906849	41907009	.	+	.	gene_id "XLOC_058729"; transcript_id "Novel_000744"; exon_number "1"; gene_type "
At_chr3	Cufflinks	exon	41907122	41907326	.	+	.	gene_id "XLOC_058729"; transcript_id "Novel_000744"; exon_number "2"; gene_type "
At_chr3	Cufflinks	exon	41907915	41907987	.	+	.	gene_id "XLOC_058729"; transcript_id "Novel_000744"; exon_number "3"; gene_type "
At_chr3	Cufflinks	exon	41908310	41908403	.	+	.	gene_id "XLOC_058729"; transcript_id "Novel_000744"; exon_number "4"; gene_type "
At_chr3	Cufflinks	exon	41909004	41909714	.	+	.	gene_id "XLOC_058729"; transcript_id "Novel_000744"; exon_number "5"; gene_type "
At_chr3	Cufflinks	exon	41906849	41909104	.	+	.	gene_id "XLOC_058729"; transcript_id "Novel_000745"; exon_number "1"; gene_type "
At_chr3	Cufflinks	exon	41909582	41909729	.	+	.	gene_id "XLOC_058729"; transcript_id "Novel_000745"; exon_number "2"; gene_type "
At_chr3	Cufflinks	exon	44627319	44627476	.	+	.	gene_id "XLOC_058973"; transcript_id "Novel_000746"; exon_number "1"; gene_type "
At_chr3	Cufflinks	exon	44627580	44627666	.	+	.	gene_id "XLOC_058973"; transcript_id "Novel_000746"; exon_number "2"; gene_type "
At_chr3	Cufflinks	exon	44627767	44627869	.	+	.	gene_id "XLOC_058973"; transcript_id "Novel_000746"; exon_number "3"; gene_type "
At_chr3	Cufflinks	exon	44629957	44630023	.	+	.	gene_id "XLOC_058973"; transcript_id "Novel_000746"; exon_number "4"; gene_type "
At_chr3	Cufflinks	exon	44630103	44630551	.	+	.	gene_id "XLOC_058973"; transcript_id "Novel_000746"; exon_number "5"; gene_type "
At_chr3	Cufflinks	exon	46608694	46608953	.	+	.	gene_id "XLOC_059141"; transcript_id "Novel_000747"; exon_number "1"; gene_type "
At_chr3	Cufflinks	exon	47181707	47182101	.	+	.	gene_id "XLOC_059173"; transcript_id "Novel_000748"; exon_number "1"; gene_type "
At_chr3	Cufflinks	exon	47182631	47182750	.	+	.	gene_id "XLOC_059173"; transcript_id "Novel_000748"; exon_number "2"; gene_type "
At_chr3	Cufflinks	exon	47183960	47184750	.	+	.	gene_id "XLOC_059173"; transcript_id "Novel_000748"; exon_number "3"; gene_type "
At_chr3	Cufflinks	exon	47181740	47182750	.	+	.	gene_id "XLOC_059173"; transcript_id "Novel_000749"; exon_number "1"; gene_type "
At_chr3	Cufflinks	exon	47183960	47184048	.	+	.	gene_id "XLOC_059173"; transcript_id "Novel_000749"; exon_number "2"; gene_type "
At_chr3	Cufflinks	exon	47184198	47184546	.	+	.	gene_id "XLOC_059173"; transcript_id "Novel_000749"; exon_number "3"; gene_type "
At_chr3	Cufflinks	exon	47181740	47182101	.	+	.	gene_id "XLOC_059173"; transcript_id "Novel_000750"; exon_number "1"; gene_type "

At_chr3	Cufflinks	exon	47182679	47182750	.	+	.	gene_id "XLOC_059173"; transcript_id "Novel_000750"; exon_number "2"; gene_type "
At_chr3	Cufflinks	exon	47183960	47184048	.	+	.	gene_id "XLOC_059173"; transcript_id "Novel_000750"; exon_number "3"; gene_type "
At_chr3	Cufflinks	exon	47184198	47184546	.	+	.	gene_id "XLOC_059173"; transcript_id "Novel_000750"; exon_number "4"; gene_type "
At_chr3	Cufflinks	exon	47779057	47779389	.	+	.	gene_id "XLOC_059201"; transcript_id "Novel_000751"; exon_number "1"; gene_type "
At_chr3	Cufflinks	exon	47780567	47780949	.	+	.	gene_id "XLOC_059201"; transcript_id "Novel_000751"; exon_number "2"; gene_type "
At_chr3	Cufflinks	exon	47786344	47787548	.	+	.	gene_id "XLOC_059201"; transcript_id "Novel_000751"; exon_number "3"; gene_type "
At_chr3	Cufflinks	exon	47780387	47780949	.	+	.	gene_id "XLOC_059201"; transcript_id "Novel_000752"; exon_number "1"; gene_type "
At_chr3	Cufflinks	exon	47786344	47787548	.	+	.	gene_id "XLOC_059201"; transcript_id "Novel_000752"; exon_number "2"; gene_type "
At_chr3	Cufflinks	exon	69172912	69172940	.	+	.	gene_id "XLOC_059854"; transcript_id "Novel_000753"; exon_number "1"; gene_type "
At_chr3	Cufflinks	exon	69173051	69173587	.	+	.	gene_id "XLOC_059854"; transcript_id "Novel_000753"; exon_number "2"; gene_type "
At_chr3	Cufflinks	exon	69766502	69766723	.	+	.	gene_id "XLOC_059891"; transcript_id "Novel_000754"; exon_number "1"; gene_type "
At_chr3	Cufflinks	exon	69767881	69767991	.	+	.	gene_id "XLOC_059891"; transcript_id "Novel_000754"; exon_number "2"; gene_type "
At_chr3	Cufflinks	exon	70751204	70751469	.	+	.	gene_id "XLOC_059936"; transcript_id "Novel_000755"; exon_number "1"; gene_type "
At_chr3	Cufflinks	exon	72436760	72436993	.	+	.	gene_id "XLOC_059978"; transcript_id "Novel_000756"; exon_number "1"; gene_type "
At_chr3	Cufflinks	exon	72437244	72437406	.	+	.	gene_id "XLOC_059978"; transcript_id "Novel_000756"; exon_number "2"; gene_type "
At_chr3	Cufflinks	exon	72437542	72438059	.	+	.	gene_id "XLOC_059978"; transcript_id "Novel_000756"; exon_number "3"; gene_type "
At_chr3	Cufflinks	exon	72444481	72445161	.	+	.	gene_id "XLOC_059978"; transcript_id "Novel_000756"; exon_number "4"; gene_type "
At_chr3	Cufflinks	exon	72436761	72436989	.	+	.	gene_id "XLOC_059978"; transcript_id "Novel_000757"; exon_number "1"; gene_type "
At_chr3	Cufflinks	exon	72437244	72437406	.	+	.	gene_id "XLOC_059978"; transcript_id "Novel_000757"; exon_number "2"; gene_type "
At_chr3	Cufflinks	exon	72437542	72438059	.	+	.	gene_id "XLOC_059978"; transcript_id "Novel_000757"; exon_number "3"; gene_type "
At_chr3	Cufflinks	exon	72444481	72445172	.	+	.	gene_id "XLOC_059978"; transcript_id "Novel_000757"; exon_number "4"; gene_type "
At_chr3	Cufflinks	exon	72884845	72885176	.	+	.	gene_id "XLOC_059995"; transcript_id "Novel_000758"; exon_number "1"; gene_type "
At_chr3	Cufflinks	exon	72885305	72887431	.	+	.	gene_id "XLOC_059995"; transcript_id "Novel_000758"; exon_number "2"; gene_type "
At_chr3	Cufflinks	exon	72888049	72888125	.	+	.	gene_id "XLOC_059995"; transcript_id "Novel_000758"; exon_number "3"; gene_type "
At_chr3	Cufflinks	exon	72888227	72890818	.	+	.	gene_id "XLOC_059995"; transcript_id "Novel_000758"; exon_number "4"; gene_type "
At_chr3	Cufflinks	exon	72892266	72892349	.	+	.	gene_id "XLOC_059995"; transcript_id "Novel_000758"; exon_number "5"; gene_type "
At_chr3	Cufflinks	exon	72893495	72893616	.	+	.	gene_id "XLOC_059995"; transcript_id "Novel_000758"; exon_number "6"; gene_type "
At_chr3	Cufflinks	exon	72893719	72894362	.	+	.	gene_id "XLOC_059995"; transcript_id "Novel_000758"; exon_number "7"; gene_type "
At_chr3	Cufflinks	exon	72884865	72885176	.	+	.	gene_id "XLOC_059995"; transcript_id "Novel_000759"; exon_number "1"; gene_type "
At_chr3	Cufflinks	exon	72885305	72885380	.	+	.	gene_id "XLOC_059995"; transcript_id "Novel_000759"; exon_number "2"; gene_type "
At_chr3	Cufflinks	exon	72886605	72887247	.	+	.	gene_id "XLOC_059995"; transcript_id "Novel_000759"; exon_number "3"; gene_type "
At_chr3	Cufflinks	exon	72884899	72885176	.	+	.	gene_id "XLOC_059995"; transcript_id "Novel_000760"; exon_number "1"; gene_type "
At_chr3	Cufflinks	exon	72885305	72885438	.	+	.	gene_id "XLOC_059995"; transcript_id "Novel_000760"; exon_number "2"; gene_type "
At_chr3	Cufflinks	exon	72886605	72887431	.	+	.	gene_id "XLOC_059995"; transcript_id "Novel_000760"; exon_number "3"; gene_type "
At_chr3	Cufflinks	exon	72888049	72888125	.	+	.	gene_id "XLOC_059995"; transcript_id "Novel_000760"; exon_number "4"; gene_type "

At_chr3	Cufflinks	exon	72888227	72892786	.	+	.	gene_id "XLOC_059995"; transcript_id "Novel_000760"; exon_number "5"; gene_type "
At_chr3	Cufflinks	exon	81357536	81357672	.	+	.	gene_id "XLOC_060242"; transcript_id "Novel_000761"; exon_number "1"; gene_type "
At_chr3	Cufflinks	exon	81357932	81358006	.	+	.	gene_id "XLOC_060242"; transcript_id "Novel_000761"; exon_number "2"; gene_type "
At_chr3	Cufflinks	exon	81358782	81358815	.	+	.	gene_id "XLOC_060242"; transcript_id "Novel_000761"; exon_number "3"; gene_type "
At_chr3	Cufflinks	exon	81358966	81359521	.	+	.	gene_id "XLOC_060242"; transcript_id "Novel_000761"; exon_number "4"; gene_type "
At_chr3	Cufflinks	exon	85388372	85388873	.	+	.	gene_id "XLOC_060351"; transcript_id "Novel_000762"; exon_number "1"; gene_type "
At_chr3	Cufflinks	exon	85388985	85389071	.	+	.	gene_id "XLOC_060351"; transcript_id "Novel_000762"; exon_number "2"; gene_type "
At_chr3	Cufflinks	exon	85389170	85391533	.	+	.	gene_id "XLOC_060351"; transcript_id "Novel_000762"; exon_number "3"; gene_type "
At_chr3	Cufflinks	exon	85391617	85391673	.	+	.	gene_id "XLOC_060351"; transcript_id "Novel_000762"; exon_number "4"; gene_type "
At_chr3	Cufflinks	exon	85391825	85392696	.	+	.	gene_id "XLOC_060351"; transcript_id "Novel_000762"; exon_number "5"; gene_type "
At_chr3	Cufflinks	exon	85710360	85710852	.	+	.	gene_id "XLOC_060369"; transcript_id "Novel_000763"; exon_number "1"; gene_type "
At_chr3	Cufflinks	exon	85712355	85712462	.	+	.	gene_id "XLOC_060369"; transcript_id "Novel_000763"; exon_number "2"; gene_type "
At_chr3	Cufflinks	exon	85712550	85712661	.	+	.	gene_id "XLOC_060369"; transcript_id "Novel_000763"; exon_number "3"; gene_type "
At_chr3	Cufflinks	exon	85712759	85712986	.	+	.	gene_id "XLOC_060369"; transcript_id "Novel_000763"; exon_number "4"; gene_type "
At_chr3	Cufflinks	exon	85713110	85713471	.	+	.	gene_id "XLOC_060369"; transcript_id "Novel_000763"; exon_number "5"; gene_type "
At_chr3	Cufflinks	exon	85710387	85712462	.	+	.	gene_id "XLOC_060369"; transcript_id "Novel_000764"; exon_number "1"; gene_type "
At_chr3	Cufflinks	exon	85712550	85712661	.	+	.	gene_id "XLOC_060369"; transcript_id "Novel_000764"; exon_number "2"; gene_type "
At_chr3	Cufflinks	exon	85712759	85712986	.	+	.	gene_id "XLOC_060369"; transcript_id "Novel_000764"; exon_number "3"; gene_type "
At_chr3	Cufflinks	exon	85713110	85713460	.	+	.	gene_id "XLOC_060369"; transcript_id "Novel_000764"; exon_number "4"; gene_type "
At_chr3	Cufflinks	exon	85710387	85712661	.	+	.	gene_id "XLOC_060369"; transcript_id "Novel_000765"; exon_number "1"; gene_type "
At_chr3	Cufflinks	exon	85712759	85712986	.	+	.	gene_id "XLOC_060369"; transcript_id "Novel_000765"; exon_number "2"; gene_type "
At_chr3	Cufflinks	exon	85713110	85713460	.	+	.	gene_id "XLOC_060369"; transcript_id "Novel_000765"; exon_number "3"; gene_type "
At_chr3	Cufflinks	exon	85929297	85929815	.	+	.	gene_id "XLOC_060395"; transcript_id "Novel_000766"; exon_number "1"; gene_type "
At_chr3	Cufflinks	exon	85930506	85930769	.	+	.	gene_id "XLOC_060395"; transcript_id "Novel_000766"; exon_number "2"; gene_type "
At_chr3	Cufflinks	exon	85930853	85930940	.	+	.	gene_id "XLOC_060395"; transcript_id "Novel_000766"; exon_number "3"; gene_type "
At_chr3	Cufflinks	exon	85931199	85931435	.	+	.	gene_id "XLOC_060395"; transcript_id "Novel_000766"; exon_number "4"; gene_type "
At_chr3	Cufflinks	exon	85931511	85931570	.	+	.	gene_id "XLOC_060395"; transcript_id "Novel_000766"; exon_number "5"; gene_type "
At_chr3	Cufflinks	exon	85931657	85931764	.	+	.	gene_id "XLOC_060395"; transcript_id "Novel_000766"; exon_number "6"; gene_type "
At_chr3	Cufflinks	exon	85931880	85932106	.	+	.	gene_id "XLOC_060395"; transcript_id "Novel_000766"; exon_number "7"; gene_type "
At_chr3	Cufflinks	exon	85932226	85932496	.	+	.	gene_id "XLOC_060395"; transcript_id "Novel_000766"; exon_number "8"; gene_type "
At_chr3	Cufflinks	exon	85929360	85929815	.	+	.	gene_id "XLOC_060395"; transcript_id "Novel_000767"; exon_number "1"; gene_type "
At_chr3	Cufflinks	exon	85930506	85930769	.	+	.	gene_id "XLOC_060395"; transcript_id "Novel_000767"; exon_number "2"; gene_type "
At_chr3	Cufflinks	exon	85930853	85931570	.	+	.	gene_id "XLOC_060395"; transcript_id "Novel_000767"; exon_number "3"; gene_type "
At_chr3	Cufflinks	exon	85931657	85931764	.	+	.	gene_id "XLOC_060395"; transcript_id "Novel_000767"; exon_number "4"; gene_type "
At_chr3	Cufflinks	exon	85931880	85932106	.	+	.	gene_id "XLOC_060395"; transcript_id "Novel_000767"; exon_number "5"; gene_type "

At_chr3	Cufflinks	exon	85932226	85932657	.	+	.	gene_id "XLOC_060395"; transcript_id "Novel_000767"; exon_number "6"; gene_type "
At_chr3	Cufflinks	exon	85929360	85929815	.	+	.	gene_id "XLOC_060395"; transcript_id "Novel_000768"; exon_number "1"; gene_type "
At_chr3	Cufflinks	exon	85930506	85930769	.	+	.	gene_id "XLOC_060395"; transcript_id "Novel_000768"; exon_number "2"; gene_type "
At_chr3	Cufflinks	exon	85930853	85930940	.	+	.	gene_id "XLOC_060395"; transcript_id "Novel_000768"; exon_number "3"; gene_type "
At_chr3	Cufflinks	exon	85931045	85931107	.	+	.	gene_id "XLOC_060395"; transcript_id "Novel_000768"; exon_number "4"; gene_type "
At_chr3	Cufflinks	exon	85931199	85931435	.	+	.	gene_id "XLOC_060395"; transcript_id "Novel_000768"; exon_number "5"; gene_type "
At_chr3	Cufflinks	exon	85931511	85931570	.	+	.	gene_id "XLOC_060395"; transcript_id "Novel_000768"; exon_number "6"; gene_type "
At_chr3	Cufflinks	exon	85931657	85931764	.	+	.	gene_id "XLOC_060395"; transcript_id "Novel_000768"; exon_number "7"; gene_type "
At_chr3	Cufflinks	exon	85931880	85932106	.	+	.	gene_id "XLOC_060395"; transcript_id "Novel_000768"; exon_number "8"; gene_type "
At_chr3	Cufflinks	exon	85932226	85932657	.	+	.	gene_id "XLOC_060395"; transcript_id "Novel_000768"; exon_number "9"; gene_type "
At_chr3	Cufflinks	exon	85931436	85931764	.	+	.	gene_id "XLOC_060395"; transcript_id "Novel_000769"; exon_number "1"; gene_type "
At_chr3	Cufflinks	exon	85931880	85932106	.	+	.	gene_id "XLOC_060395"; transcript_id "Novel_000769"; exon_number "2"; gene_type "
At_chr3	Cufflinks	exon	85932226	85932628	.	+	.	gene_id "XLOC_060395"; transcript_id "Novel_000769"; exon_number "3"; gene_type "
At_chr3	Cufflinks	exon	85957115	85958025	.	+	.	gene_id "XLOC_060401"; transcript_id "Novel_000770"; exon_number "1"; gene_type "
At_chr3	Cufflinks	exon	85958113	85959705	.	+	.	gene_id "XLOC_060401"; transcript_id "Novel_000770"; exon_number "2"; gene_type "
At_chr3	Cufflinks	exon	93143937	93144367	.	+	.	gene_id "XLOC_060667"; transcript_id "Novel_000771"; exon_number "1"; gene_type "
At_chr3	Cufflinks	exon	93144524	93144574	.	+	.	gene_id "XLOC_060667"; transcript_id "Novel_000771"; exon_number "2"; gene_type "
At_chr3	Cufflinks	exon	93144999	93145138	.	+	.	gene_id "XLOC_060667"; transcript_id "Novel_000771"; exon_number "3"; gene_type "
At_chr3	Cufflinks	exon	93145274	93145481	.	+	.	gene_id "XLOC_060667"; transcript_id "Novel_000771"; exon_number "4"; gene_type "
At_chr3	Cufflinks	exon	93145581	93145614	.	+	.	gene_id "XLOC_060667"; transcript_id "Novel_000772"; exon_number "1"; gene_type "
At_chr3	Cufflinks	exon	93145799	93146724	.	+	.	gene_id "XLOC_060667"; transcript_id "Novel_000772"; exon_number "2"; gene_type "
At_chr3	Cufflinks	exon	93319902	93320159	.	+	.	gene_id "XLOC_060704"; transcript_id "Novel_000773"; exon_number "1"; gene_type "
At_chr3	Cufflinks	exon	93454564	93454720	.	+	.	gene_id "XLOC_060728"; transcript_id "Novel_000774"; exon_number "1"; gene_type "
At_chr3	Cufflinks	exon	93454797	93454897	.	+	.	gene_id "XLOC_060728"; transcript_id "Novel_000774"; exon_number "2"; gene_type "
At_chr3	Cufflinks	exon	9071249	9071446	.	-	.	gene_id "XLOC_061288"; transcript_id "Novel_000775"; exon_number "1"; gene_type "
At_chr3	Cufflinks	exon	9072138	9072269	.	-	.	gene_id "XLOC_061288"; transcript_id "Novel_000775"; exon_number "2"; gene_type "
At_chr3	Cufflinks	exon	9073485	9073542	.	-	.	gene_id "XLOC_061288"; transcript_id "Novel_000775"; exon_number "3"; gene_type "
At_chr3	Cufflinks	exon	9073642	9073681	.	-	.	gene_id "XLOC_061288"; transcript_id "Novel_000775"; exon_number "4"; gene_type "
At_chr3	Cufflinks	exon	9073851	9074002	.	-	.	gene_id "XLOC_061288"; transcript_id "Novel_000775"; exon_number "5"; gene_type "
At_chr3	Cufflinks	exon	9074082	9074135	.	-	.	gene_id "XLOC_061288"; transcript_id "Novel_000775"; exon_number "6"; gene_type "
At_chr3	Cufflinks	exon	9074254	9074334	.	-	.	gene_id "XLOC_061288"; transcript_id "Novel_000775"; exon_number "7"; gene_type "
At_chr3	Cufflinks	exon	9074431	9074631	.	-	.	gene_id "XLOC_061288"; transcript_id "Novel_000775"; exon_number "8"; gene_type "
At_chr3	Cufflinks	exon	9418158	9418930	.	-	.	gene_id "XLOC_061303"; transcript_id "Novel_000776"; exon_number "1"; gene_type "
At_chr3	Cufflinks	exon	9419485	9419861	.	-	.	gene_id "XLOC_061303"; transcript_id "Novel_000776"; exon_number "2"; gene_type "
At_chr3	Cufflinks	exon	9419968	9420544	.	-	.	gene_id "XLOC_061303"; transcript_id "Novel_000776"; exon_number "3"; gene_type "

At_chr3	Cufflinks	exon	20090498	20090944	.	-	.	gene_id "XLOC_061845"; transcript_id "Novel_000777"; exon_number "1"; gene_type "
At_chr3	Cufflinks	exon	20091044	20091816	.	-	.	gene_id "XLOC_061845"; transcript_id "Novel_000777"; exon_number "2"; gene_type "
At_chr3	Cufflinks	exon	20090516	20090944	.	-	.	gene_id "XLOC_061845"; transcript_id "Novel_000778"; exon_number "1"; gene_type "
At_chr3	Cufflinks	exon	20091044	20091771	.	-	.	gene_id "XLOC_061845"; transcript_id "Novel_000778"; exon_number "2"; gene_type "
At_chr3	Cufflinks	exon	20091931	20091994	.	-	.	gene_id "XLOC_061845"; transcript_id "Novel_000778"; exon_number "3"; gene_type "
At_chr3	Cufflinks	exon	20092092	20092138	.	-	.	gene_id "XLOC_061845"; transcript_id "Novel_000778"; exon_number "4"; gene_type "
At_chr3	Cufflinks	exon	20092435	20092592	.	-	.	gene_id "XLOC_061845"; transcript_id "Novel_000778"; exon_number "5"; gene_type "
At_chr3	Cufflinks	exon	20092677	20093145	.	-	.	gene_id "XLOC_061845"; transcript_id "Novel_000778"; exon_number "6"; gene_type "
At_chr3	Cufflinks	exon	20278587	20278856	.	-	.	gene_id "XLOC_061865"; transcript_id "Novel_000779"; exon_number "1"; gene_type "
At_chr3	Cufflinks	exon	27733952	27734654	.	-	.	gene_id "XLOC_062119"; transcript_id "Novel_000780"; exon_number "1"; gene_type "
At_chr3	Cufflinks	exon	27735816	27736186	.	-	.	gene_id "XLOC_062119"; transcript_id "Novel_000780"; exon_number "2"; gene_type "
At_chr3	Cufflinks	exon	33594876	33599673	.	-	.	gene_id "XLOC_062371"; transcript_id "Novel_000781"; exon_number "1"; gene_type "
At_chr3	Cufflinks	exon	33612580	33612697	.	-	.	gene_id "XLOC_062371"; transcript_id "Novel_000781"; exon_number "2"; gene_type "
At_chr3	Cufflinks	exon	33613572	33613711	.	-	.	gene_id "XLOC_062371"; transcript_id "Novel_000781"; exon_number "3"; gene_type "
At_chr3	Cufflinks	exon	33613801	33614135	.	-	.	gene_id "XLOC_062371"; transcript_id "Novel_000781"; exon_number "4"; gene_type "
At_chr3	Cufflinks	exon	35784138	35785021	.	-	.	gene_id "XLOC_062456"; transcript_id "Novel_000782"; exon_number "1"; gene_type "
At_chr3	Cufflinks	exon	35785274	35785483	.	-	.	gene_id "XLOC_062456"; transcript_id "Novel_000782"; exon_number "2"; gene_type "
At_chr3	Cufflinks	exon	36521318	36521981	.	-	.	gene_id "XLOC_062501"; transcript_id "Novel_000783"; exon_number "1"; gene_type "
At_chr3	Cufflinks	exon	36522072	36522266	.	-	.	gene_id "XLOC_062501"; transcript_id "Novel_000783"; exon_number "2"; gene_type "
At_chr3	Cufflinks	exon	36522357	36522626	.	-	.	gene_id "XLOC_062501"; transcript_id "Novel_000783"; exon_number "3"; gene_type "
At_chr3	Cufflinks	exon	36522757	36522931	.	-	.	gene_id "XLOC_062501"; transcript_id "Novel_000783"; exon_number "4"; gene_type "
At_chr3	Cufflinks	exon	36523039	36523510	.	-	.	gene_id "XLOC_062501"; transcript_id "Novel_000783"; exon_number "5"; gene_type "
At_chr3	Cufflinks	exon	36523622	36525623	.	-	.	gene_id "XLOC_062501"; transcript_id "Novel_000783"; exon_number "6"; gene_type "
At_chr3	Cufflinks	exon	36521328	36521981	.	-	.	gene_id "XLOC_062501"; transcript_id "Novel_000784"; exon_number "1"; gene_type "
At_chr3	Cufflinks	exon	36522072	36522266	.	-	.	gene_id "XLOC_062501"; transcript_id "Novel_000784"; exon_number "2"; gene_type "
At_chr3	Cufflinks	exon	36522357	36522626	.	-	.	gene_id "XLOC_062501"; transcript_id "Novel_000784"; exon_number "3"; gene_type "
At_chr3	Cufflinks	exon	36522757	36522931	.	-	.	gene_id "XLOC_062501"; transcript_id "Novel_000784"; exon_number "4"; gene_type "
At_chr3	Cufflinks	exon	36523039	36523510	.	-	.	gene_id "XLOC_062501"; transcript_id "Novel_000784"; exon_number "5"; gene_type "
At_chr3	Cufflinks	exon	36523622	36524959	.	-	.	gene_id "XLOC_062501"; transcript_id "Novel_000784"; exon_number "6"; gene_type "
At_chr3	Cufflinks	exon	36525561	36525793	.	-	.	gene_id "XLOC_062501"; transcript_id "Novel_000784"; exon_number "7"; gene_type "
At_chr3	Cufflinks	exon	36521343	36521981	.	-	.	gene_id "XLOC_062501"; transcript_id "Novel_000785"; exon_number "1"; gene_type "
At_chr3	Cufflinks	exon	36522072	36522266	.	-	.	gene_id "XLOC_062501"; transcript_id "Novel_000785"; exon_number "2"; gene_type "
At_chr3	Cufflinks	exon	36522357	36522626	.	-	.	gene_id "XLOC_062501"; transcript_id "Novel_000785"; exon_number "3"; gene_type "
At_chr3	Cufflinks	exon	36522757	36522931	.	-	.	gene_id "XLOC_062501"; transcript_id "Novel_000785"; exon_number "4"; gene_type "
At_chr3	Cufflinks	exon	36523039	36523149	.	-	.	gene_id "XLOC_062501"; transcript_id "Novel_000785"; exon_number "5"; gene_type "

At_chr3	Cufflinks	exon	36523240	36523510	.	-	.	gene_id "XLOC_062501"; transcript_id "Novel_000785"; exon_number "6"; gene_type "
At_chr3	Cufflinks	exon	36523622	36523771	.	-	.	gene_id "XLOC_062501"; transcript_id "Novel_000785"; exon_number "7"; gene_type "
At_chr3	Cufflinks	exon	36523861	36523889	.	-	.	gene_id "XLOC_062501"; transcript_id "Novel_000785"; exon_number "8"; gene_type "
At_chr3	Cufflinks	exon	36709828	36710240	.	-	.	gene_id "XLOC_062520"; transcript_id "Novel_000786"; exon_number "1"; gene_type "
At_chr3	Cufflinks	exon	36710700	36711427	.	-	.	gene_id "XLOC_062520"; transcript_id "Novel_000786"; exon_number "2"; gene_type "
At_chr3	Cufflinks	exon	36711968	36712317	.	-	.	gene_id "XLOC_062520"; transcript_id "Novel_000786"; exon_number "3"; gene_type "
At_chr3	Cufflinks	exon	36709835	36710240	.	-	.	gene_id "XLOC_062520"; transcript_id "Novel_000787"; exon_number "1"; gene_type "
At_chr3	Cufflinks	exon	36710700	36710846	.	-	.	gene_id "XLOC_062520"; transcript_id "Novel_000787"; exon_number "2"; gene_type "
At_chr3	Cufflinks	exon	36710979	36712307	.	-	.	gene_id "XLOC_062520"; transcript_id "Novel_000787"; exon_number "3"; gene_type "
At_chr3	Cufflinks	exon	36709857	36710240	.	-	.	gene_id "XLOC_062520"; transcript_id "Novel_000788"; exon_number "1"; gene_type "
At_chr3	Cufflinks	exon	36710700	36710846	.	-	.	gene_id "XLOC_062520"; transcript_id "Novel_000788"; exon_number "2"; gene_type "
At_chr3	Cufflinks	exon	36710979	36711427	.	-	.	gene_id "XLOC_062520"; transcript_id "Novel_000788"; exon_number "3"; gene_type "
At_chr3	Cufflinks	exon	36711968	36712292	.	-	.	gene_id "XLOC_062520"; transcript_id "Novel_000788"; exon_number "4"; gene_type "
At_chr3	Cufflinks	exon	36710475	36711427	.	-	.	gene_id "XLOC_062520"; transcript_id "Novel_000789"; exon_number "1"; gene_type "
At_chr3	Cufflinks	exon	36711968	36712315	.	-	.	gene_id "XLOC_062520"; transcript_id "Novel_000789"; exon_number "2"; gene_type "
At_chr3	Cufflinks	exon	37084778	37085035	.	-	.	gene_id "XLOC_062557"; transcript_id "Novel_000790"; exon_number "1"; gene_type "
At_chr3	Cufflinks	exon	37085132	37085254	.	-	.	gene_id "XLOC_062557"; transcript_id "Novel_000790"; exon_number "2"; gene_type "
At_chr3	Cufflinks	exon	37085557	37085834	.	-	.	gene_id "XLOC_062557"; transcript_id "Novel_000790"; exon_number "3"; gene_type "
At_chr3	Cufflinks	exon	37093521	37093986	.	-	.	gene_id "XLOC_062558"; transcript_id "Novel_000791"; exon_number "1"; gene_type "
At_chr3	Cufflinks	exon	37094499	37094618	.	-	.	gene_id "XLOC_062558"; transcript_id "Novel_000791"; exon_number "2"; gene_type "
At_chr3	Cufflinks	exon	37095296	37095374	.	-	.	gene_id "XLOC_062558"; transcript_id "Novel_000791"; exon_number "3"; gene_type "
At_chr3	Cufflinks	exon	37095579	37095736	.	-	.	gene_id "XLOC_062558"; transcript_id "Novel_000791"; exon_number "4"; gene_type "
At_chr3	Cufflinks	exon	37095821	37095910	.	-	.	gene_id "XLOC_062558"; transcript_id "Novel_000791"; exon_number "5"; gene_type "
At_chr3	Cufflinks	exon	37096200	37096545	.	-	.	gene_id "XLOC_062558"; transcript_id "Novel_000791"; exon_number "6"; gene_type "
At_chr3	Cufflinks	exon	37093604	37093986	.	-	.	gene_id "XLOC_062558"; transcript_id "Novel_000792"; exon_number "1"; gene_type "
At_chr3	Cufflinks	exon	37094499	37094615	.	-	.	gene_id "XLOC_062558"; transcript_id "Novel_000792"; exon_number "2"; gene_type "
At_chr3	Cufflinks	exon	37095296	37095374	.	-	.	gene_id "XLOC_062558"; transcript_id "Novel_000792"; exon_number "3"; gene_type "
At_chr3	Cufflinks	exon	37095579	37095736	.	-	.	gene_id "XLOC_062558"; transcript_id "Novel_000792"; exon_number "4"; gene_type "
At_chr3	Cufflinks	exon	37095821	37095910	.	-	.	gene_id "XLOC_062558"; transcript_id "Novel_000792"; exon_number "5"; gene_type "
At_chr3	Cufflinks	exon	37096200	37096545	.	-	.	gene_id "XLOC_062558"; transcript_id "Novel_000792"; exon_number "6"; gene_type "
At_chr3	Cufflinks	exon	39783587	39785767	.	-	.	gene_id "XLOC_062707"; transcript_id "Novel_000793"; exon_number "1"; gene_type "
At_chr3	Cufflinks	exon	39785972	39786665	.	-	.	gene_id "XLOC_062707"; transcript_id "Novel_000793"; exon_number "2"; gene_type "
At_chr3	Cufflinks	exon	39811942	39812427	.	-	.	gene_id "XLOC_062714"; transcript_id "Novel_000794"; exon_number "1"; gene_type "
At_chr3	Cufflinks	exon	39812505	39812646	.	-	.	gene_id "XLOC_062714"; transcript_id "Novel_000794"; exon_number "2"; gene_type "
At_chr3	Cufflinks	exon	39813478	39815103	.	-	.	gene_id "XLOC_062714"; transcript_id "Novel_000794"; exon_number "3"; gene_type "

At_chr3	Cufflinks	exon	40541554	40542806	.	-	.	gene_id "XLOC_062787"; transcript_id "Novel_000795"; exon_number "1"; gene_type "
At_chr3	Cufflinks	exon	40546115	40546194	.	-	.	gene_id "XLOC_062787"; transcript_id "Novel_000795"; exon_number "2"; gene_type "
At_chr3	Cufflinks	exon	40547044	40547110	.	-	.	gene_id "XLOC_062787"; transcript_id "Novel_000795"; exon_number "3"; gene_type "
At_chr3	Cufflinks	exon	42428528	42428732	.	-	.	gene_id "XLOC_062957"; transcript_id "Novel_000796"; exon_number "1"; gene_type "
At_chr3	Cufflinks	exon	42429138	42429311	.	-	.	gene_id "XLOC_062957"; transcript_id "Novel_000796"; exon_number "2"; gene_type "
At_chr3	Cufflinks	exon	42429396	42429470	.	-	.	gene_id "XLOC_062957"; transcript_id "Novel_000796"; exon_number "3"; gene_type "
At_chr3	Cufflinks	exon	42429597	42429677	.	-	.	gene_id "XLOC_062957"; transcript_id "Novel_000796"; exon_number "4"; gene_type "
At_chr3	Cufflinks	exon	42429770	42429844	.	-	.	gene_id "XLOC_062957"; transcript_id "Novel_000796"; exon_number "5"; gene_type "
At_chr3	Cufflinks	exon	42429922	42429996	.	-	.	gene_id "XLOC_062957"; transcript_id "Novel_000796"; exon_number "6"; gene_type "
At_chr3	Cufflinks	exon	42430260	42430469	.	-	.	gene_id "XLOC_062957"; transcript_id "Novel_000796"; exon_number "7"; gene_type "
At_chr3	Cufflinks	exon	42431364	42431835	.	-	.	gene_id "XLOC_062957"; transcript_id "Novel_000796"; exon_number "8"; gene_type "
At_chr3	Cufflinks	exon	43077934	43078139	.	-	.	gene_id "XLOC_063000"; transcript_id "Novel_000797"; exon_number "1"; gene_type "
At_chr3	Cufflinks	exon	44026495	44027171	.	-	.	gene_id "XLOC_063104"; transcript_id "Novel_000798"; exon_number "1"; gene_type "
At_chr3	Cufflinks	exon	44027518	44027692	.	-	.	gene_id "XLOC_063104"; transcript_id "Novel_000798"; exon_number "2"; gene_type "
At_chr3	Cufflinks	exon	44027815	44028983	.	-	.	gene_id "XLOC_063104"; transcript_id "Novel_000798"; exon_number "3"; gene_type "
At_chr3	Cufflinks	exon	44029061	44029522	.	-	.	gene_id "XLOC_063104"; transcript_id "Novel_000798"; exon_number "4"; gene_type "
At_chr3	Cufflinks	exon	44026727	44027171	.	-	.	gene_id "XLOC_063104"; transcript_id "Novel_000799"; exon_number "1"; gene_type "
At_chr3	Cufflinks	exon	44027518	44029517	.	-	.	gene_id "XLOC_063104"; transcript_id "Novel_000799"; exon_number "2"; gene_type "
At_chr3	Cufflinks	exon	49091550	49091921	.	-	.	gene_id "XLOC_063364"; transcript_id "Novel_000800"; exon_number "1"; gene_type "
At_chr3	Cufflinks	exon	49092018	49092789	.	-	.	gene_id "XLOC_063364"; transcript_id "Novel_000800"; exon_number "2"; gene_type "
At_chr3	Cufflinks	exon	49091618	49091924	.	-	.	gene_id "XLOC_063364"; transcript_id "Novel_000801"; exon_number "1"; gene_type "
At_chr3	Cufflinks	exon	49092018	49092792	.	-	.	gene_id "XLOC_063364"; transcript_id "Novel_000801"; exon_number "2"; gene_type "
At_chr3	Cufflinks	exon	49091636	49092420	.	-	.	gene_id "XLOC_063364"; transcript_id "Novel_000802"; exon_number "1"; gene_type "
At_chr3	Cufflinks	exon	49092699	49092813	.	-	.	gene_id "XLOC_063364"; transcript_id "Novel_000802"; exon_number "2"; gene_type "
At_chr3	Cufflinks	exon	52074516	52075490	.	-	.	gene_id "XLOC_063490"; transcript_id "Novel_000803"; exon_number "1"; gene_type "
At_chr3	Cufflinks	exon	52075575	52076010	.	-	.	gene_id "XLOC_063490"; transcript_id "Novel_000803"; exon_number "2"; gene_type "
At_chr3	Cufflinks	exon	67131264	67138513	.	-	.	gene_id "XLOC_063967"; transcript_id "Novel_000804"; exon_number "1"; gene_type "
At_chr3	Cufflinks	exon	67588738	67588785	.	-	.	gene_id "XLOC_063975"; transcript_id "Novel_000805"; exon_number "1"; gene_type "
At_chr3	Cufflinks	exon	67588885	67589013	.	-	.	gene_id "XLOC_063975"; transcript_id "Novel_000805"; exon_number "2"; gene_type "
At_chr3	Cufflinks	exon	67590180	67590219	.	-	.	gene_id "XLOC_063975"; transcript_id "Novel_000805"; exon_number "3"; gene_type "
At_chr3	Cufflinks	exon	85669103	85669784	.	-	.	gene_id "XLOC_064577"; transcript_id "Novel_000806"; exon_number "1"; gene_type "
At_chr3	Cufflinks	exon	85669989	85670086	.	-	.	gene_id "XLOC_064577"; transcript_id "Novel_000806"; exon_number "2"; gene_type "
At_chr3	Cufflinks	exon	85670437	85670577	.	-	.	gene_id "XLOC_064577"; transcript_id "Novel_000806"; exon_number "3"; gene_type "
At_chr3	Cufflinks	exon	85670654	85670771	.	-	.	gene_id "XLOC_064577"; transcript_id "Novel_000806"; exon_number "4"; gene_type "
At_chr3	Cufflinks	exon	85670850	85670856	.	-	.	gene_id "XLOC_064577"; transcript_id "Novel_000806"; exon_number "5"; gene_type "

At_chr3	Cufflinks	exon	85896426	85897279	.	-	.	gene_id "XLOC_064601"; transcript_id "Novel_000807"; exon_number "1"; gene_type "
At_chr3	Cufflinks	exon	85897491	85897934	.	-	.	gene_id "XLOC_064601"; transcript_id "Novel_000807"; exon_number "2"; gene_type "
At_chr3	Cufflinks	exon	93303791	93303896	.	-	.	gene_id "XLOC_064856"; transcript_id "Novel_000808"; exon_number "1"; gene_type "
At_chr3	Cufflinks	exon	93304106	93304263	.	-	.	gene_id "XLOC_064856"; transcript_id "Novel_000808"; exon_number "2"; gene_type "
At_chr3	Cufflinks	exon	93468494	93469212	.	-	.	gene_id "XLOC_064875"; transcript_id "Novel_000809"; exon_number "1"; gene_type "
At_chr3	Cufflinks	exon	93469305	93469347	.	-	.	gene_id "XLOC_064875"; transcript_id "Novel_000809"; exon_number "2"; gene_type "
At_chr3	Cufflinks	exon	93469452	93469776	.	-	.	gene_id "XLOC_064875"; transcript_id "Novel_000809"; exon_number "3"; gene_type "
At_chr3	Cufflinks	exon	96504481	96506307	.	-	.	gene_id "XLOC_064960"; transcript_id "Novel_000810"; exon_number "1"; gene_type "
At_chr3	Cufflinks	exon	96506717	96506972	.	-	.	gene_id "XLOC_064960"; transcript_id "Novel_000810"; exon_number "2"; gene_type "
At_chr3	Cufflinks	exon	96504551	96506307	.	-	.	gene_id "XLOC_064960"; transcript_id "Novel_000811"; exon_number "1"; gene_type "
At_chr3	Cufflinks	exon	96506772	96506995	.	-	.	gene_id "XLOC_064960"; transcript_id "Novel_000811"; exon_number "2"; gene_type "
At_chr4	Cufflinks	exon	812376	812419	.	+	.	gene_id "XLOC_065134"; transcript_id "Novel_000812"; exon_number "1"; gene_type "
At_chr4	Cufflinks	exon	812535	812642	.	+	.	gene_id "XLOC_065134"; transcript_id "Novel_000812"; exon_number "2"; gene_type "
At_chr4	Cufflinks	exon	813453	813706	.	+	.	gene_id "XLOC_065134"; transcript_id "Novel_000812"; exon_number "3"; gene_type "
At_chr4	Cufflinks	exon	813798	814009	.	+	.	gene_id "XLOC_065134"; transcript_id "Novel_000812"; exon_number "4"; gene_type "
At_chr4	Cufflinks	exon	814177	814441	.	+	.	gene_id "XLOC_065134"; transcript_id "Novel_000812"; exon_number "5"; gene_type "
At_chr4	Cufflinks	exon	814724	815307	.	+	.	gene_id "XLOC_065134"; transcript_id "Novel_000812"; exon_number "6"; gene_type "
At_chr4	Cufflinks	exon	812396	812642	.	+	.	gene_id "XLOC_065134"; transcript_id "Novel_000813"; exon_number "1"; gene_type "
At_chr4	Cufflinks	exon	813453	814009	.	+	.	gene_id "XLOC_065134"; transcript_id "Novel_000813"; exon_number "2"; gene_type "
At_chr4	Cufflinks	exon	814177	814441	.	+	.	gene_id "XLOC_065134"; transcript_id "Novel_000813"; exon_number "3"; gene_type "
At_chr4	Cufflinks	exon	814724	815259	.	+	.	gene_id "XLOC_065134"; transcript_id "Novel_000813"; exon_number "4"; gene_type "
At_chr4	Cufflinks	exon	870521	870646	.	+	.	gene_id "XLOC_065144"; transcript_id "Novel_000814"; exon_number "1"; gene_type "
At_chr4	Cufflinks	exon	870725	875145	.	+	.	gene_id "XLOC_065144"; transcript_id "Novel_000814"; exon_number "2"; gene_type "
At_chr4	Cufflinks	exon	4029538	4029934	.	+	.	gene_id "XLOC_065291"; transcript_id "Novel_000815"; exon_number "1"; gene_type "
At_chr4	Cufflinks	exon	4030036	4030064	.	+	.	gene_id "XLOC_065291"; transcript_id "Novel_000815"; exon_number "2"; gene_type "
At_chr4	Cufflinks	exon	4030131	4030157	.	+	.	gene_id "XLOC_065291"; transcript_id "Novel_000815"; exon_number "3"; gene_type "
At_chr4	Cufflinks	exon	5344207	5344318	.	+	.	gene_id "XLOC_065435"; transcript_id "Novel_000816"; exon_number "1"; gene_type "
At_chr4	Cufflinks	exon	5345715	5346020	.	+	.	gene_id "XLOC_065435"; transcript_id "Novel_000816"; exon_number "2"; gene_type "
At_chr4	Cufflinks	exon	8353115	8353874	.	+	.	gene_id "XLOC_065534"; transcript_id "Novel_000817"; exon_number "1"; gene_type "
At_chr4	Cufflinks	exon	8353962	8354070	.	+	.	gene_id "XLOC_065534"; transcript_id "Novel_000817"; exon_number "2"; gene_type "
At_chr4	Cufflinks	exon	8355092	8355239	.	+	.	gene_id "XLOC_065534"; transcript_id "Novel_000817"; exon_number "3"; gene_type "
At_chr4	Cufflinks	exon	8355794	8356208	.	+	.	gene_id "XLOC_065534"; transcript_id "Novel_000817"; exon_number "4"; gene_type "
At_chr4	Cufflinks	exon	11567482	11568010	.	+	.	gene_id "XLOC_065578"; transcript_id "Novel_000818"; exon_number "1"; gene_type "
At_chr4	Cufflinks	exon	11568138	11568324	.	+	.	gene_id "XLOC_065578"; transcript_id "Novel_000818"; exon_number "2"; gene_type "
At_chr4	Cufflinks	exon	11573829	11574016	.	+	.	gene_id "XLOC_065578"; transcript_id "Novel_000818"; exon_number "3"; gene_type "

At_chr4	Cufflinks	exon	11574196	11574248	.	+	.	gene_id "XLOC_065578"; transcript_id "Novel_000818"; exon_number "4"; gene_type "
At_chr4	Cufflinks	exon	11574354	11574388	.	+	.	gene_id "XLOC_065578"; transcript_id "Novel_000818"; exon_number "5"; gene_type "
At_chr4	Cufflinks	exon	11575640	11576168	.	+	.	gene_id "XLOC_065578"; transcript_id "Novel_000818"; exon_number "6"; gene_type "
At_chr4	Cufflinks	exon	16996598	16996973	.	+	.	gene_id "XLOC_065716"; transcript_id "Novel_000819"; exon_number "1"; gene_type "
At_chr4	Cufflinks	exon	16997453	16997520	.	+	.	gene_id "XLOC_065716"; transcript_id "Novel_000819"; exon_number "2"; gene_type "
At_chr4	Cufflinks	exon	16998216	16998371	.	+	.	gene_id "XLOC_065716"; transcript_id "Novel_000819"; exon_number "3"; gene_type "
At_chr4	Cufflinks	exon	16998648	16998968	.	+	.	gene_id "XLOC_065716"; transcript_id "Novel_000819"; exon_number "4"; gene_type "
At_chr4	Cufflinks	exon	23318131	23319061	.	+	.	gene_id "XLOC_065991"; transcript_id "Novel_000820"; exon_number "1"; gene_type "
At_chr4	Cufflinks	exon	23319147	23320301	.	+	.	gene_id "XLOC_065991"; transcript_id "Novel_000820"; exon_number "2"; gene_type "
At_chr4	Cufflinks	exon	23327215	23327306	.	+	.	gene_id "XLOC_065992"; transcript_id "Novel_000821"; exon_number "1"; gene_type "
At_chr4	Cufflinks	exon	23327476	23327580	.	+	.	gene_id "XLOC_065992"; transcript_id "Novel_000821"; exon_number "2"; gene_type "
At_chr4	Cufflinks	exon	23327888	23327932	.	+	.	gene_id "XLOC_065992"; transcript_id "Novel_000821"; exon_number "3"; gene_type "
At_chr4	Cufflinks	exon	23328130	23328235	.	+	.	gene_id "XLOC_065992"; transcript_id "Novel_000822"; exon_number "1"; gene_type "
At_chr4	Cufflinks	exon	23328366	23328551	.	+	.	gene_id "XLOC_065992"; transcript_id "Novel_000822"; exon_number "2"; gene_type "
At_chr4	Cufflinks	exon	23328651	23328942	.	+	.	gene_id "XLOC_065992"; transcript_id "Novel_000822"; exon_number "3"; gene_type "
At_chr4	Cufflinks	exon	23329172	23329316	.	+	.	gene_id "XLOC_065992"; transcript_id "Novel_000822"; exon_number "4"; gene_type "
At_chr4	Cufflinks	exon	23329407	23329549	.	+	.	gene_id "XLOC_065992"; transcript_id "Novel_000822"; exon_number "5"; gene_type "
At_chr4	Cufflinks	exon	23329782	23330044	.	+	.	gene_id "XLOC_065992"; transcript_id "Novel_000822"; exon_number "6"; gene_type "
At_chr4	Cufflinks	exon	23330321	23330419	.	+	.	gene_id "XLOC_065992"; transcript_id "Novel_000822"; exon_number "7"; gene_type "
At_chr4	Cufflinks	exon	23328205	23328235	.	+	.	gene_id "XLOC_065992"; transcript_id "Novel_000823"; exon_number "1"; gene_type "
At_chr4	Cufflinks	exon	23328366	23328551	.	+	.	gene_id "XLOC_065992"; transcript_id "Novel_000823"; exon_number "2"; gene_type "
At_chr4	Cufflinks	exon	23328651	23328723	.	+	.	gene_id "XLOC_065992"; transcript_id "Novel_000823"; exon_number "3"; gene_type "
At_chr4	Cufflinks	exon	23328857	23328942	.	+	.	gene_id "XLOC_065992"; transcript_id "Novel_000823"; exon_number "4"; gene_type "
At_chr4	Cufflinks	exon	23329172	23329316	.	+	.	gene_id "XLOC_065992"; transcript_id "Novel_000823"; exon_number "5"; gene_type "
At_chr4	Cufflinks	exon	23329407	23329549	.	+	.	gene_id "XLOC_065992"; transcript_id "Novel_000823"; exon_number "6"; gene_type "
At_chr4	Cufflinks	exon	23329782	23330044	.	+	.	gene_id "XLOC_065992"; transcript_id "Novel_000823"; exon_number "7"; gene_type "
At_chr4	Cufflinks	exon	23330321	23330555	.	+	.	gene_id "XLOC_065992"; transcript_id "Novel_000823"; exon_number "8"; gene_type "
At_chr4	Cufflinks	exon	23384420	23384820	.	+	.	gene_id "XLOC_066012"; transcript_id "Novel_000824"; exon_number "1"; gene_type "
At_chr4	Cufflinks	exon	23385222	23385263	.	+	.	gene_id "XLOC_066012"; transcript_id "Novel_000824"; exon_number "2"; gene_type "
At_chr4	Cufflinks	exon	23385029	23385614	.	+	.	gene_id "XLOC_066012"; transcript_id "Novel_000825"; exon_number "1"; gene_type "
At_chr4	Cufflinks	exon	23385745	23385849	.	+	.	gene_id "XLOC_066012"; transcript_id "Novel_000825"; exon_number "2"; gene_type "
At_chr4	Cufflinks	exon	23386059	23386113	.	+	.	gene_id "XLOC_066012"; transcript_id "Novel_000825"; exon_number "3"; gene_type "
At_chr4	Cufflinks	exon	23386556	23386666	.	+	.	gene_id "XLOC_066012"; transcript_id "Novel_000825"; exon_number "4"; gene_type "
At_chr4	Cufflinks	exon	23386750	23386800	.	+	.	gene_id "XLOC_066012"; transcript_id "Novel_000825"; exon_number "5"; gene_type "
At_chr4	Cufflinks	exon	23387270	23387445	.	+	.	gene_id "XLOC_066012"; transcript_id "Novel_000825"; exon_number "6"; gene_type "

At_chr4	Cufflinks	exon	23387538	23388621	.	+	.	gene_id "XLOC_066012"; transcript_id "Novel_000825"; exon_number "7"; gene_type "
At_chr4	Cufflinks	exon	23385029	23385614	.	+	.	gene_id "XLOC_066012"; transcript_id "Novel_000826"; exon_number "1"; gene_type "
At_chr4	Cufflinks	exon	23385745	23385849	.	+	.	gene_id "XLOC_066012"; transcript_id "Novel_000826"; exon_number "2"; gene_type "
At_chr4	Cufflinks	exon	23386059	23386113	.	+	.	gene_id "XLOC_066012"; transcript_id "Novel_000826"; exon_number "3"; gene_type "
At_chr4	Cufflinks	exon	23386556	23386666	.	+	.	gene_id "XLOC_066012"; transcript_id "Novel_000826"; exon_number "4"; gene_type "
At_chr4	Cufflinks	exon	23386750	23386800	.	+	.	gene_id "XLOC_066012"; transcript_id "Novel_000826"; exon_number "5"; gene_type "
At_chr4	Cufflinks	exon	23387270	23387445	.	+	.	gene_id "XLOC_066012"; transcript_id "Novel_000826"; exon_number "6"; gene_type "
At_chr4	Cufflinks	exon	23387538	23387645	.	+	.	gene_id "XLOC_066012"; transcript_id "Novel_000826"; exon_number "7"; gene_type "
At_chr4	Cufflinks	exon	23388807	23388880	.	+	.	gene_id "XLOC_066012"; transcript_id "Novel_000826"; exon_number "8"; gene_type "
At_chr4	Cufflinks	exon	23389270	23389445	.	+	.	gene_id "XLOC_066012"; transcript_id "Novel_000826"; exon_number "9"; gene_type "
At_chr4	Cufflinks	exon	23389606	23389701	.	+	.	gene_id "XLOC_066012"; transcript_id "Novel_000826"; exon_number "10"; gene_type
At_chr4	Cufflinks	exon	23390016	23390096	.	+	.	gene_id "XLOC_066012"; transcript_id "Novel_000826"; exon_number "11"; gene_type
At_chr4	Cufflinks	exon	23390238	23390291	.	+	.	gene_id "XLOC_066012"; transcript_id "Novel_000826"; exon_number "12"; gene_type
At_chr4	Cufflinks	exon	23390385	23390519	.	+	.	gene_id "XLOC_066012"; transcript_id "Novel_000826"; exon_number "13"; gene_type
At_chr4	Cufflinks	exon	23390592	23391559	.	+	.	gene_id "XLOC_066012"; transcript_id "Novel_000826"; exon_number "14"; gene_type
At_chr4	Cufflinks	exon	23385055	23385614	.	+	.	gene_id "XLOC_066012"; transcript_id "Novel_000827"; exon_number "1"; gene_type "
At_chr4	Cufflinks	exon	23385745	23385849	.	+	.	gene_id "XLOC_066012"; transcript_id "Novel_000827"; exon_number "2"; gene_type "
At_chr4	Cufflinks	exon	23386059	23386113	.	+	.	gene_id "XLOC_066012"; transcript_id "Novel_000827"; exon_number "3"; gene_type "
At_chr4	Cufflinks	exon	23386556	23386666	.	+	.	gene_id "XLOC_066012"; transcript_id "Novel_000827"; exon_number "4"; gene_type "
At_chr4	Cufflinks	exon	23386750	23386800	.	+	.	gene_id "XLOC_066012"; transcript_id "Novel_000827"; exon_number "5"; gene_type "
At_chr4	Cufflinks	exon	23387270	23387445	.	+	.	gene_id "XLOC_066012"; transcript_id "Novel_000827"; exon_number "6"; gene_type "
At_chr4	Cufflinks	exon	23387538	23387645	.	+	.	gene_id "XLOC_066012"; transcript_id "Novel_000827"; exon_number "7"; gene_type "
At_chr4	Cufflinks	exon	23388807	23388880	.	+	.	gene_id "XLOC_066012"; transcript_id "Novel_000827"; exon_number "8"; gene_type "
At_chr4	Cufflinks	exon	23389270	23389445	.	+	.	gene_id "XLOC_066012"; transcript_id "Novel_000827"; exon_number "9"; gene_type "
At_chr4	Cufflinks	exon	23389606	23389974	.	+	.	gene_id "XLOC_066012"; transcript_id "Novel_000827"; exon_number "10"; gene_type
At_chr4	Cufflinks	exon	23390285	23390519	.	+	.	gene_id "XLOC_066012"; transcript_id "Novel_000828"; exon_number "1"; gene_type "
At_chr4	Cufflinks	exon	23390592	23391305	.	+	.	gene_id "XLOC_066012"; transcript_id "Novel_000828"; exon_number "2"; gene_type "
At_chr4	Cufflinks	exon	23397253	23397438	.	+	.	gene_id "XLOC_066013"; transcript_id "Novel_000829"; exon_number "1"; gene_type "
At_chr4	Cufflinks	exon	23397517	23397781	.	+	.	gene_id "XLOC_066013"; transcript_id "Novel_000829"; exon_number "2"; gene_type "
At_chr4	Cufflinks	exon	23397913	23398528	.	+	.	gene_id "XLOC_066013"; transcript_id "Novel_000829"; exon_number "3"; gene_type "
At_chr4	Cufflinks	exon	23397318	23397438	.	+	.	gene_id "XLOC_066013"; transcript_id "Novel_000830"; exon_number "1"; gene_type "
At_chr4	Cufflinks	exon	23397517	23398502	.	+	.	gene_id "XLOC_066013"; transcript_id "Novel_000830"; exon_number "2"; gene_type "
At_chr4	Cufflinks	exon	23407859	23408207	.	+	.	gene_id "XLOC_066016"; transcript_id "Novel_000831"; exon_number "1"; gene_type "
At_chr4	Cufflinks	exon	23408410	23408524	.	+	.	gene_id "XLOC_066016"; transcript_id "Novel_000831"; exon_number "2"; gene_type "
At_chr4	Cufflinks	exon	23408717	23408791	.	+	.	gene_id "XLOC_066016"; transcript_id "Novel_000831"; exon_number "3"; gene_type "

At_chr4	Cufflinks	exon	23408904	23408975	.	+	.	gene_id "XLOC_066016"; transcript_id "Novel_000831"; exon_number "4"; gene_type "
At_chr4	Cufflinks	exon	23409091	23409228	.	+	.	gene_id "XLOC_066016"; transcript_id "Novel_000831"; exon_number "5"; gene_type "
At_chr4	Cufflinks	exon	23409506	23409539	.	+	.	gene_id "XLOC_066016"; transcript_id "Novel_000831"; exon_number "6"; gene_type "
At_chr4	Cufflinks	exon	23410098	23410207	.	+	.	gene_id "XLOC_066016"; transcript_id "Novel_000831"; exon_number "7"; gene_type "
At_chr4	Cufflinks	exon	23410356	23410442	.	+	.	gene_id "XLOC_066016"; transcript_id "Novel_000831"; exon_number "8"; gene_type "
At_chr4	Cufflinks	exon	23410581	23410664	.	+	.	gene_id "XLOC_066016"; transcript_id "Novel_000831"; exon_number "9"; gene_type "
At_chr4	Cufflinks	exon	23410755	23410820	.	+	.	gene_id "XLOC_066016"; transcript_id "Novel_000831"; exon_number "10"; gene_type
At_chr4	Cufflinks	exon	23410973	23411907	.	+	.	gene_id "XLOC_066016"; transcript_id "Novel_000831"; exon_number "11"; gene_type
At_chr4	Cufflinks	exon	23407866	23408207	.	+	.	gene_id "XLOC_066016"; transcript_id "Novel_000832"; exon_number "1"; gene_type "
At_chr4	Cufflinks	exon	23408410	23408524	.	+	.	gene_id "XLOC_066016"; transcript_id "Novel_000832"; exon_number "2"; gene_type "
At_chr4	Cufflinks	exon	23408717	23408791	.	+	.	gene_id "XLOC_066016"; transcript_id "Novel_000832"; exon_number "3"; gene_type "
At_chr4	Cufflinks	exon	23408904	23408975	.	+	.	gene_id "XLOC_066016"; transcript_id "Novel_000832"; exon_number "4"; gene_type "
At_chr4	Cufflinks	exon	23409091	23409228	.	+	.	gene_id "XLOC_066016"; transcript_id "Novel_000832"; exon_number "5"; gene_type "
At_chr4	Cufflinks	exon	23409506	23409539	.	+	.	gene_id "XLOC_066016"; transcript_id "Novel_000832"; exon_number "6"; gene_type "
At_chr4	Cufflinks	exon	23410098	23410207	.	+	.	gene_id "XLOC_066016"; transcript_id "Novel_000832"; exon_number "7"; gene_type "
At_chr4	Cufflinks	exon	23410356	23410442	.	+	.	gene_id "XLOC_066016"; transcript_id "Novel_000832"; exon_number "8"; gene_type "
At_chr4	Cufflinks	exon	23410581	23410664	.	+	.	gene_id "XLOC_066016"; transcript_id "Novel_000832"; exon_number "9"; gene_type "
At_chr4	Cufflinks	exon	23410755	23410820	.	+	.	gene_id "XLOC_066016"; transcript_id "Novel_000832"; exon_number "10"; gene_type
At_chr4	Cufflinks	exon	23410973	23411099	.	+	.	gene_id "XLOC_066016"; transcript_id "Novel_000832"; exon_number "11"; gene_type
At_chr4	Cufflinks	exon	23411215	23411717	.	+	.	gene_id "XLOC_066016"; transcript_id "Novel_000832"; exon_number "12"; gene_type
At_chr4	Cufflinks	exon	23445543	23446019	.	+	.	gene_id "XLOC_066027"; transcript_id "Novel_000833"; exon_number "1"; gene_type "
At_chr4	Cufflinks	exon	23447058	23447365	.	+	.	gene_id "XLOC_066027"; transcript_id "Novel_000833"; exon_number "2"; gene_type "
At_chr4	Cufflinks	exon	23447457	23447589	.	+	.	gene_id "XLOC_066027"; transcript_id "Novel_000833"; exon_number "3"; gene_type "
At_chr4	Cufflinks	exon	23447739	23448212	.	+	.	gene_id "XLOC_066027"; transcript_id "Novel_000833"; exon_number "4"; gene_type "
At_chr4	Cufflinks	exon	36590065	36590102	.	+	.	gene_id "XLOC_066439"; transcript_id "Novel_000834"; exon_number "1"; gene_type "
At_chr4	Cufflinks	exon	36590238	36590304	.	+	.	gene_id "XLOC_066439"; transcript_id "Novel_000834"; exon_number "2"; gene_type "
At_chr4	Cufflinks	exon	36590980	36591135	.	+	.	gene_id "XLOC_066439"; transcript_id "Novel_000834"; exon_number "3"; gene_type "
At_chr4	Cufflinks	exon	37752484	37752703	.	+	.	gene_id "XLOC_066610"; transcript_id "Novel_000835"; exon_number "1"; gene_type "
At_chr4	Cufflinks	exon	37753162	37753459	.	+	.	gene_id "XLOC_066610"; transcript_id "Novel_000835"; exon_number "2"; gene_type "
At_chr4	Cufflinks	exon	37754272	37754400	.	+	.	gene_id "XLOC_066610"; transcript_id "Novel_000835"; exon_number "3"; gene_type "
At_chr4	Cufflinks	exon	37755392	37755484	.	+	.	gene_id "XLOC_066610"; transcript_id "Novel_000835"; exon_number "4"; gene_type "
At_chr4	Cufflinks	exon	37755596	37755736	.	+	.	gene_id "XLOC_066610"; transcript_id "Novel_000835"; exon_number "5"; gene_type "
At_chr4	Cufflinks	exon	37756007	37756075	.	+	.	gene_id "XLOC_066610"; transcript_id "Novel_000835"; exon_number "6"; gene_type "
At_chr4	Cufflinks	exon	37756385	37756468	.	+	.	gene_id "XLOC_066610"; transcript_id "Novel_000835"; exon_number "7"; gene_type "
At_chr4	Cufflinks	exon	37756720	37756900	.	+	.	gene_id "XLOC_066610"; transcript_id "Novel_000835"; exon_number "8"; gene_type "

At_chr4	Cufflinks	exon	37757749	37757783	.	+	.	gene_id "XLOC_066610"; transcript_id "Novel_000835"; exon_number "9"; gene_type "
At_chr4	Cufflinks	exon	37752538	37752703	.	+	.	gene_id "XLOC_066610"; transcript_id "Novel_000836"; exon_number "1"; gene_type "
At_chr4	Cufflinks	exon	37753168	37753459	.	+	.	gene_id "XLOC_066610"; transcript_id "Novel_000836"; exon_number "2"; gene_type "
At_chr4	Cufflinks	exon	37754272	37754400	.	+	.	gene_id "XLOC_066610"; transcript_id "Novel_000836"; exon_number "3"; gene_type "
At_chr4	Cufflinks	exon	37755392	37755484	.	+	.	gene_id "XLOC_066610"; transcript_id "Novel_000836"; exon_number "4"; gene_type "
At_chr4	Cufflinks	exon	37755596	37755736	.	+	.	gene_id "XLOC_066610"; transcript_id "Novel_000836"; exon_number "5"; gene_type "
At_chr4	Cufflinks	exon	37756007	37756075	.	+	.	gene_id "XLOC_066610"; transcript_id "Novel_000836"; exon_number "6"; gene_type "
At_chr4	Cufflinks	exon	37756385	37756468	.	+	.	gene_id "XLOC_066610"; transcript_id "Novel_000836"; exon_number "7"; gene_type "
At_chr4	Cufflinks	exon	37756720	37757252	.	+	.	gene_id "XLOC_066610"; transcript_id "Novel_000836"; exon_number "8"; gene_type "
At_chr4	Cufflinks	exon	37758261	37758651	.	+	.	gene_id "XLOC_066610"; transcript_id "Novel_000837"; exon_number "1"; gene_type "
At_chr4	Cufflinks	exon	37758958	37759107	.	+	.	gene_id "XLOC_066610"; transcript_id "Novel_000837"; exon_number "2"; gene_type "
At_chr4	Cufflinks	exon	37759229	37760020	.	+	.	gene_id "XLOC_066610"; transcript_id "Novel_000837"; exon_number "3"; gene_type "
At_chr4	Cufflinks	exon	37758676	37759107	.	+	.	gene_id "XLOC_066610"; transcript_id "Novel_000838"; exon_number "1"; gene_type "
At_chr4	Cufflinks	exon	37759229	37760032	.	+	.	gene_id "XLOC_066610"; transcript_id "Novel_000838"; exon_number "2"; gene_type "
At_chr4	Cufflinks	exon	37782391	37782581	.	+	.	gene_id "XLOC_066615"; transcript_id "Novel_000839"; exon_number "1"; gene_type "
At_chr4	Cufflinks	exon	37782667	37782723	.	+	.	gene_id "XLOC_066615"; transcript_id "Novel_000839"; exon_number "2"; gene_type "
At_chr4	Cufflinks	exon	37782834	37782919	.	+	.	gene_id "XLOC_066615"; transcript_id "Novel_000839"; exon_number "3"; gene_type "
At_chr4	Cufflinks	exon	37782988	37783187	.	+	.	gene_id "XLOC_066615"; transcript_id "Novel_000839"; exon_number "4"; gene_type "
At_chr4	Cufflinks	exon	45929518	45929584	.	+	.	gene_id "XLOC_066938"; transcript_id "Novel_000840"; exon_number "1"; gene_type "
At_chr4	Cufflinks	exon	45930559	45930922	.	+	.	gene_id "XLOC_066938"; transcript_id "Novel_000840"; exon_number "2"; gene_type "
At_chr4	Cufflinks	exon	45931146	45933519	.	+	.	gene_id "XLOC_066938"; transcript_id "Novel_000840"; exon_number "3"; gene_type "
At_chr4	Cufflinks	exon	50040054	50040408	.	+	.	gene_id "XLOC_067048"; transcript_id "Novel_000841"; exon_number "1"; gene_type "
At_chr4	Cufflinks	exon	50041141	50041413	.	+	.	gene_id "XLOC_067048"; transcript_id "Novel_000841"; exon_number "2"; gene_type "
At_chr4	Cufflinks	exon	50046022	50046113	.	+	.	gene_id "XLOC_067048"; transcript_id "Novel_000841"; exon_number "3"; gene_type "
At_chr4	Cufflinks	exon	50046309	50046535	.	+	.	gene_id "XLOC_067048"; transcript_id "Novel_000841"; exon_number "4"; gene_type "
At_chr4	Cufflinks	exon	50046796	50046839	.	+	.	gene_id "XLOC_067048"; transcript_id "Novel_000841"; exon_number "5"; gene_type "
At_chr4	Cufflinks	exon	50040066	50040408	.	+	.	gene_id "XLOC_067048"; transcript_id "Novel_000842"; exon_number "1"; gene_type "
At_chr4	Cufflinks	exon	50041141	50041276	.	+	.	gene_id "XLOC_067048"; transcript_id "Novel_000842"; exon_number "2"; gene_type "
At_chr4	Cufflinks	exon	50041360	50041923	.	+	.	gene_id "XLOC_067048"; transcript_id "Novel_000842"; exon_number "3"; gene_type "
At_chr4	Cufflinks	exon	50040066	50040408	.	+	.	gene_id "XLOC_067048"; transcript_id "Novel_000843"; exon_number "1"; gene_type "
At_chr4	Cufflinks	exon	50041141	50041276	.	+	.	gene_id "XLOC_067048"; transcript_id "Novel_000843"; exon_number "2"; gene_type "
At_chr4	Cufflinks	exon	50041360	50041413	.	+	.	gene_id "XLOC_067048"; transcript_id "Novel_000843"; exon_number "3"; gene_type "
At_chr4	Cufflinks	exon	50046022	50046113	.	+	.	gene_id "XLOC_067048"; transcript_id "Novel_000843"; exon_number "4"; gene_type "
At_chr4	Cufflinks	exon	50046309	50046535	.	+	.	gene_id "XLOC_067048"; transcript_id "Novel_000843"; exon_number "5"; gene_type "
At_chr4	Cufflinks	exon	50046796	50046842	.	+	.	gene_id "XLOC_067048"; transcript_id "Novel_000843"; exon_number "6"; gene_type "

At_chr4	Cufflinks	exon	50040699	50041276	.	+	.	gene_id "XLOC_067048"; transcript_id "Novel_000844"; exon_number "1"; gene_type "
At_chr4	Cufflinks	exon	50041360	50041888	.	+	.	gene_id "XLOC_067048"; transcript_id "Novel_000844"; exon_number "2"; gene_type "
At_chr4	Cufflinks	exon	50046003	50046113	.	+	.	gene_id "XLOC_067048"; transcript_id "Novel_000845"; exon_number "1"; gene_type "
At_chr4	Cufflinks	exon	50046309	50046535	.	+	.	gene_id "XLOC_067048"; transcript_id "Novel_000845"; exon_number "2"; gene_type "
At_chr4	Cufflinks	exon	50046796	50046842	.	+	.	gene_id "XLOC_067048"; transcript_id "Novel_000845"; exon_number "3"; gene_type "
At_chr4	Cufflinks	exon	51133572	51134283	.	+	.	gene_id "XLOC_067086"; transcript_id "Novel_000846"; exon_number "1"; gene_type "
At_chr4	Cufflinks	exon	60805838	60806253	.	+	.	gene_id "XLOC_067298"; transcript_id "Novel_000847"; exon_number "1"; gene_type "
At_chr4	Cufflinks	exon	60806591	60806691	.	+	.	gene_id "XLOC_067298"; transcript_id "Novel_000847"; exon_number "2"; gene_type "
At_chr4	Cufflinks	exon	60806968	60807051	.	+	.	gene_id "XLOC_067298"; transcript_id "Novel_000847"; exon_number "3"; gene_type "
At_chr4	Cufflinks	exon	60807498	60807698	.	+	.	gene_id "XLOC_067298"; transcript_id "Novel_000848"; exon_number "1"; gene_type "
At_chr4	Cufflinks	exon	60808137	60808226	.	+	.	gene_id "XLOC_067298"; transcript_id "Novel_000848"; exon_number "2"; gene_type "
At_chr4	Cufflinks	exon	65427180	65427566	.	+	.	gene_id "XLOC_067470"; transcript_id "Novel_000849"; exon_number "1"; gene_type "
At_chr4	Cufflinks	exon	65427936	65428292	.	+	.	gene_id "XLOC_067470"; transcript_id "Novel_000849"; exon_number "2"; gene_type "
At_chr4	Cufflinks	exon	66615484	66615704	.	+	.	gene_id "XLOC_067534"; transcript_id "Novel_000850"; exon_number "1"; gene_type "
At_chr4	Cufflinks	exon	78237759	78238040	.	+	.	gene_id "XLOC_067937"; transcript_id "Novel_000851"; exon_number "1"; gene_type "
At_chr4	Cufflinks	exon	78238368	78239773	.	+	.	gene_id "XLOC_067937"; transcript_id "Novel_000851"; exon_number "2"; gene_type "
At_chr4	Cufflinks	exon	81895019	81895408	.	+	.	gene_id "XLOC_068048"; transcript_id "Novel_000852"; exon_number "1"; gene_type "
At_chr4	Cufflinks	exon	81895504	81895714	.	+	.	gene_id "XLOC_068048"; transcript_id "Novel_000852"; exon_number "2"; gene_type "
At_chr4	Cufflinks	exon	81895845	81896014	.	+	.	gene_id "XLOC_068048"; transcript_id "Novel_000852"; exon_number "3"; gene_type "
At_chr4	Cufflinks	exon	81896119	81897208	.	+	.	gene_id "XLOC_068048"; transcript_id "Novel_000852"; exon_number "4"; gene_type "
At_chr4	Cufflinks	exon	81895029	81896014	.	+	.	gene_id "XLOC_068048"; transcript_id "Novel_000853"; exon_number "1"; gene_type "
At_chr4	Cufflinks	exon	81896119	81896447	.	+	.	gene_id "XLOC_068048"; transcript_id "Novel_000853"; exon_number "2"; gene_type "
At_chr4	Cufflinks	exon	83661564	83661725	.	+	.	gene_id "XLOC_068102"; transcript_id "Novel_000854"; exon_number "1"; gene_type "
At_chr4	Cufflinks	exon	83662500	83662857	.	+	.	gene_id "XLOC_068102"; transcript_id "Novel_000854"; exon_number "2"; gene_type "
At_chr4	Cufflinks	exon	84012743	84013100	.	+	.	gene_id "XLOC_068145"; transcript_id "Novel_000855"; exon_number "1"; gene_type "
At_chr4	Cufflinks	exon	84013427	84013588	.	+	.	gene_id "XLOC_068145"; transcript_id "Novel_000855"; exon_number "2"; gene_type "
At_chr4	Cufflinks	exon	84015115	84015149	.	+	.	gene_id "XLOC_068145"; transcript_id "Novel_000856"; exon_number "1"; gene_type "
At_chr4	Cufflinks	exon	84015602	84015652	.	+	.	gene_id "XLOC_068145"; transcript_id "Novel_000856"; exon_number "2"; gene_type "
At_chr4	Cufflinks	exon	84015745	84015807	.	+	.	gene_id "XLOC_068145"; transcript_id "Novel_000856"; exon_number "3"; gene_type "
At_chr4	Cufflinks	exon	84015911	84015957	.	+	.	gene_id "XLOC_068145"; transcript_id "Novel_000856"; exon_number "4"; gene_type "
At_chr4	Cufflinks	exon	84016306	84016375	.	+	.	gene_id "XLOC_068145"; transcript_id "Novel_000856"; exon_number "5"; gene_type "
At_chr4	Cufflinks	exon	84016464	84016631	.	+	.	gene_id "XLOC_068145"; transcript_id "Novel_000856"; exon_number "6"; gene_type "
At_chr4	Cufflinks	exon	84345061	84345210	.	+	.	gene_id "XLOC_068167"; transcript_id "Novel_000857"; exon_number "1"; gene_type "
At_chr4	Cufflinks	exon	84345586	84345772	.	+	.	gene_id "XLOC_068167"; transcript_id "Novel_000857"; exon_number "2"; gene_type "
At_chr4	Cufflinks	exon	84346363	84346516	.	+	.	gene_id "XLOC_068167"; transcript_id "Novel_000857"; exon_number "3"; gene_type "

At_chr4	Cufflinks	exon	84346655	84346692	.	+	.	gene_id "XLOC_068167"; transcript_id "Novel_000857"; exon_number "4"; gene_type "
At_chr4	Cufflinks	exon	84346787	84346856	.	+	.	gene_id "XLOC_068167"; transcript_id "Novel_000857"; exon_number "5"; gene_type "
At_chr4	Cufflinks	exon	84346936	84347311	.	+	.	gene_id "XLOC_068167"; transcript_id "Novel_000857"; exon_number "6"; gene_type "
At_chr4	Cufflinks	exon	84346360	84346516	.	+	.	gene_id "XLOC_068167"; transcript_id "Novel_000858"; exon_number "1"; gene_type "
At_chr4	Cufflinks	exon	84346655	84346692	.	+	.	gene_id "XLOC_068167"; transcript_id "Novel_000858"; exon_number "2"; gene_type "
At_chr4	Cufflinks	exon	84346787	84346856	.	+	.	gene_id "XLOC_068167"; transcript_id "Novel_000858"; exon_number "3"; gene_type "
At_chr4	Cufflinks	exon	84346936	84347256	.	+	.	gene_id "XLOC_068167"; transcript_id "Novel_000858"; exon_number "4"; gene_type "
At_chr4	Cufflinks	exon	84647858	84648188	.	+	.	gene_id "XLOC_068203"; transcript_id "Novel_000859"; exon_number "1"; gene_type "
At_chr4	Cufflinks	exon	84648308	84648510	.	+	.	gene_id "XLOC_068203"; transcript_id "Novel_000859"; exon_number "2"; gene_type "
At_chr4	Cufflinks	exon	84712248	84712299	.	+	.	gene_id "XLOC_068222"; transcript_id "Novel_000860"; exon_number "1"; gene_type "
At_chr4	Cufflinks	exon	84712919	84713008	.	+	.	gene_id "XLOC_068222"; transcript_id "Novel_000860"; exon_number "2"; gene_type "
At_chr4	Cufflinks	exon	84713602	84713661	.	+	.	gene_id "XLOC_068222"; transcript_id "Novel_000860"; exon_number "3"; gene_type "
At_chr4	Cufflinks	exon	84713745	84713838	.	+	.	gene_id "XLOC_068222"; transcript_id "Novel_000860"; exon_number "4"; gene_type "
At_chr4	Cufflinks	exon	84722444	84722498	.	+	.	gene_id "XLOC_068223"; transcript_id "Novel_000861"; exon_number "1"; gene_type "
At_chr4	Cufflinks	exon	84722631	84722684	.	+	.	gene_id "XLOC_068223"; transcript_id "Novel_000861"; exon_number "2"; gene_type "
At_chr4	Cufflinks	exon	84722747	84722806	.	+	.	gene_id "XLOC_068223"; transcript_id "Novel_000861"; exon_number "3"; gene_type "
At_chr4	Cufflinks	exon	84722886	84722927	.	+	.	gene_id "XLOC_068223"; transcript_id "Novel_000861"; exon_number "4"; gene_type "
At_chr4	Cufflinks	exon	84746282	84746901	.	+	.	gene_id "XLOC_068229"; transcript_id "Novel_000862"; exon_number "1"; gene_type "
At_chr4	Cufflinks	exon	84748035	84748145	.	+	.	gene_id "XLOC_068229"; transcript_id "Novel_000862"; exon_number "2"; gene_type "
At_chr4	Cufflinks	exon	84748480	84748827	.	+	.	gene_id "XLOC_068229"; transcript_id "Novel_000862"; exon_number "3"; gene_type "
At_chr4	Cufflinks	exon	84749043	84749362	.	+	.	gene_id "XLOC_068229"; transcript_id "Novel_000862"; exon_number "4"; gene_type "
At_chr4	Cufflinks	exon	84749467	84749558	.	+	.	gene_id "XLOC_068229"; transcript_id "Novel_000862"; exon_number "5"; gene_type "
At_chr4	Cufflinks	exon	84750327	84750687	.	+	.	gene_id "XLOC_068229"; transcript_id "Novel_000862"; exon_number "6"; gene_type "
At_chr4	Cufflinks	exon	84751148	84751517	.	+	.	gene_id "XLOC_068229"; transcript_id "Novel_000862"; exon_number "7"; gene_type "
At_chr4	Cufflinks	exon	84746365	84746901	.	+	.	gene_id "XLOC_068229"; transcript_id "Novel_000863"; exon_number "1"; gene_type "
At_chr4	Cufflinks	exon	84748035	84748145	.	+	.	gene_id "XLOC_068229"; transcript_id "Novel_000863"; exon_number "2"; gene_type "
At_chr4	Cufflinks	exon	84748480	84748827	.	+	.	gene_id "XLOC_068229"; transcript_id "Novel_000863"; exon_number "3"; gene_type "
At_chr4	Cufflinks	exon	84749043	84749362	.	+	.	gene_id "XLOC_068229"; transcript_id "Novel_000863"; exon_number "4"; gene_type "
At_chr4	Cufflinks	exon	84749467	84749558	.	+	.	gene_id "XLOC_068229"; transcript_id "Novel_000863"; exon_number "5"; gene_type "
At_chr4	Cufflinks	exon	84750327	84750687	.	+	.	gene_id "XLOC_068229"; transcript_id "Novel_000863"; exon_number "6"; gene_type "
At_chr4	Cufflinks	exon	84751148	84751355	.	+	.	gene_id "XLOC_068229"; transcript_id "Novel_000863"; exon_number "7"; gene_type "
At_chr4	Cufflinks	exon	84752271	84752440	.	+	.	gene_id "XLOC_068229"; transcript_id "Novel_000863"; exon_number "8"; gene_type "
At_chr4	Cufflinks	exon	85281577	85281867	.	+	.	gene_id "XLOC_068282"; transcript_id "Novel_000864"; exon_number "1"; gene_type "
At_chr4	Cufflinks	exon	85281996	85282116	.	+	.	gene_id "XLOC_068282"; transcript_id "Novel_000864"; exon_number "2"; gene_type "
At_chr4	Cufflinks	exon	85281577	85281867	.	+	.	gene_id "XLOC_068282"; transcript_id "Novel_000865"; exon_number "1"; gene_type "

At_chr4	Cufflinks	exon	85281996	85282114	.	+	.	gene_id "XLOC_068282"; transcript_id "Novel_000865"; exon_number "2"; gene_type "
At_chr4	Cufflinks	exon	85282879	85283311	.	+	.	gene_id "XLOC_068282"; transcript_id "Novel_000865"; exon_number "3"; gene_type "
At_chr4	Cufflinks	exon	85283444	85283704	.	+	.	gene_id "XLOC_068282"; transcript_id "Novel_000865"; exon_number "4"; gene_type "
At_chr4	Cufflinks	exon	85284257	85284379	.	+	.	gene_id "XLOC_068282"; transcript_id "Novel_000865"; exon_number "5"; gene_type "
At_chr4	Cufflinks	exon	85284561	85284659	.	+	.	gene_id "XLOC_068282"; transcript_id "Novel_000865"; exon_number "6"; gene_type "
At_chr4	Cufflinks	exon	85284750	85285216	.	+	.	gene_id "XLOC_068282"; transcript_id "Novel_000865"; exon_number "7"; gene_type "
At_chr4	Cufflinks	exon	85282228	85283311	.	+	.	gene_id "XLOC_068282"; transcript_id "Novel_000866"; exon_number "1"; gene_type "
At_chr4	Cufflinks	exon	85283444	85283704	.	+	.	gene_id "XLOC_068282"; transcript_id "Novel_000866"; exon_number "2"; gene_type "
At_chr4	Cufflinks	exon	85284257	85284379	.	+	.	gene_id "XLOC_068282"; transcript_id "Novel_000866"; exon_number "3"; gene_type "
At_chr4	Cufflinks	exon	85284561	85285170	.	+	.	gene_id "XLOC_068282"; transcript_id "Novel_000866"; exon_number "4"; gene_type "
At_chr4	Cufflinks	exon	85364677	85364751	.	+	.	gene_id "XLOC_068296"; transcript_id "Novel_000867"; exon_number "1"; gene_type "
At_chr4	Cufflinks	exon	85364854	85364978	.	+	.	gene_id "XLOC_068296"; transcript_id "Novel_000867"; exon_number "2"; gene_type "
At_chr4	Cufflinks	exon	85365075	85365173	.	+	.	gene_id "XLOC_068296"; transcript_id "Novel_000867"; exon_number "3"; gene_type "
At_chr4	Cufflinks	exon	85365304	85366079	.	+	.	gene_id "XLOC_068296"; transcript_id "Novel_000867"; exon_number "4"; gene_type "
At_chr4	Cufflinks	exon	85364901	85365173	.	+	.	gene_id "XLOC_068296"; transcript_id "Novel_000868"; exon_number "1"; gene_type "
At_chr4	Cufflinks	exon	85365304	85365639	.	+	.	gene_id "XLOC_068296"; transcript_id "Novel_000868"; exon_number "2"; gene_type "
At_chr4	Cufflinks	exon	85687026	85687260	.	+	.	gene_id "XLOC_068369"; transcript_id "Novel_000869"; exon_number "1"; gene_type "
At_chr4	Cufflinks	exon	86301201	86301310	.	+	.	gene_id "XLOC_068434"; transcript_id "Novel_000870"; exon_number "1"; gene_type "
At_chr4	Cufflinks	exon	86302568	86302634	.	+	.	gene_id "XLOC_068434"; transcript_id "Novel_000870"; exon_number "2"; gene_type "
At_chr4	Cufflinks	exon	86302958	86303031	.	+	.	gene_id "XLOC_068434"; transcript_id "Novel_000870"; exon_number "3"; gene_type "
At_chr4	Cufflinks	exon	86303131	86303215	.	+	.	gene_id "XLOC_068434"; transcript_id "Novel_000870"; exon_number "4"; gene_type "
At_chr4	Cufflinks	exon	86303304	86303359	.	+	.	gene_id "XLOC_068434"; transcript_id "Novel_000870"; exon_number "5"; gene_type "
At_chr4	Cufflinks	exon	86303825	86303879	.	+	.	gene_id "XLOC_068434"; transcript_id "Novel_000870"; exon_number "6"; gene_type "
At_chr4	Cufflinks	exon	86304191	86304238	.	+	.	gene_id "XLOC_068434"; transcript_id "Novel_000870"; exon_number "7"; gene_type "
At_chr4	Cufflinks	exon	86304403	86304486	.	+	.	gene_id "XLOC_068434"; transcript_id "Novel_000870"; exon_number "8"; gene_type "
At_chr4	Cufflinks	exon	86304650	86304766	.	+	.	gene_id "XLOC_068434"; transcript_id "Novel_000870"; exon_number "9"; gene_type "
At_chr4	Cufflinks	exon	86304851	86304934	.	+	.	gene_id "XLOC_068434"; transcript_id "Novel_000870"; exon_number "10"; gene_type
At_chr4	Cufflinks	exon	86305018	86305143	.	+	.	gene_id "XLOC_068434"; transcript_id "Novel_000870"; exon_number "11"; gene_type
At_chr4	Cufflinks	exon	86305260	86305357	.	+	.	gene_id "XLOC_068434"; transcript_id "Novel_000870"; exon_number "12"; gene_type
At_chr4	Cufflinks	exon	86305527	86305880	.	+	.	gene_id "XLOC_068434"; transcript_id "Novel_000870"; exon_number "13"; gene_type
At_chr4	Cufflinks	exon	4238327	4238894	.	-	.	gene_id "XLOC_069070"; transcript_id "Novel_000871"; exon_number "1"; gene_type "
At_chr4	Cufflinks	exon	4240092	4240172	.	-	.	gene_id "XLOC_069070"; transcript_id "Novel_000871"; exon_number "2"; gene_type "
At_chr4	Cufflinks	exon	4241056	4241129	.	-	.	gene_id "XLOC_069070"; transcript_id "Novel_000871"; exon_number "3"; gene_type "
At_chr4	Cufflinks	exon	4395749	4397687	.	-	.	gene_id "XLOC_069082"; transcript_id "Novel_000872"; exon_number "1"; gene_type "
At_chr4	Cufflinks	exon	4398612	4398724	.	-	.	gene_id "XLOC_069082"; transcript_id "Novel_000872"; exon_number "2"; gene_type "

At_chr4	Cufflinks	exon	4395993	4397606	.	-	.	gene_id "XLOC_069082"; transcript_id "Novel_000873"; exon_number "1"; gene_type "
At_chr4	Cufflinks	exon	4403222	4403302	.	-	.	gene_id "XLOC_069082"; transcript_id "Novel_000873"; exon_number "2"; gene_type "
At_chr4	Cufflinks	exon	4404251	4404438	.	-	.	gene_id "XLOC_069082"; transcript_id "Novel_000873"; exon_number "3"; gene_type "
At_chr4	Cufflinks	exon	4396005	4397334	.	-	.	gene_id "XLOC_069082"; transcript_id "Novel_000874"; exon_number "1"; gene_type "
At_chr4	Cufflinks	exon	4397563	4397563	.	-	.	gene_id "XLOC_069082"; transcript_id "Novel_000874"; exon_number "2"; gene_type "
At_chr4	Cufflinks	exon	4396009	4397606	.	-	.	gene_id "XLOC_069082"; transcript_id "Novel_000875"; exon_number "1"; gene_type "
At_chr4	Cufflinks	exon	4403222	4404202	.	-	.	gene_id "XLOC_069082"; transcript_id "Novel_000875"; exon_number "2"; gene_type "
At_chr4	Cufflinks	exon	4401622	4403302	.	-	.	gene_id "XLOC_069082"; transcript_id "Novel_000876"; exon_number "1"; gene_type "
At_chr4	Cufflinks	exon	4404251	4404490	.	-	.	gene_id "XLOC_069082"; transcript_id "Novel_000876"; exon_number "2"; gene_type "
At_chr4	Cufflinks	exon	4408186	4409730	.	-	.	gene_id "XLOC_069084"; transcript_id "Novel_000877"; exon_number "1"; gene_type "
At_chr4	Cufflinks	exon	4410386	4410496	.	-	.	gene_id "XLOC_069084"; transcript_id "Novel_000877"; exon_number "2"; gene_type "
At_chr4	Cufflinks	exon	5009117	5009499	.	-	.	gene_id "XLOC_069175"; transcript_id "Novel_000878"; exon_number "1"; gene_type "
At_chr4	Cufflinks	exon	5195207	5195585	.	-	.	gene_id "XLOC_069190"; transcript_id "Novel_000879"; exon_number "1"; gene_type "
At_chr4	Cufflinks	exon	5195688	5195756	.	-	.	gene_id "XLOC_069190"; transcript_id "Novel_000879"; exon_number "2"; gene_type "
At_chr4	Cufflinks	exon	5195863	5195976	.	-	.	gene_id "XLOC_069190"; transcript_id "Novel_000879"; exon_number "3"; gene_type "
At_chr4	Cufflinks	exon	5196075	5196190	.	-	.	gene_id "XLOC_069190"; transcript_id "Novel_000879"; exon_number "4"; gene_type "
At_chr4	Cufflinks	exon	6061817	6062694	.	-	.	gene_id "XLOC_069234"; transcript_id "Novel_000880"; exon_number "1"; gene_type "
At_chr4	Cufflinks	exon	6063167	6063274	.	-	.	gene_id "XLOC_069234"; transcript_id "Novel_000880"; exon_number "2"; gene_type "
At_chr4	Cufflinks	exon	6063776	6065181	.	-	.	gene_id "XLOC_069234"; transcript_id "Novel_000880"; exon_number "3"; gene_type "
At_chr4	Cufflinks	exon	14954214	14955295	.	-	.	gene_id "XLOC_069446"; transcript_id "Novel_000881"; exon_number "1"; gene_type "
At_chr4	Cufflinks	exon	14955486	14956295	.	-	.	gene_id "XLOC_069446"; transcript_id "Novel_000881"; exon_number "2"; gene_type "
At_chr4	Cufflinks	exon	14956367	14957121	.	-	.	gene_id "XLOC_069446"; transcript_id "Novel_000881"; exon_number "3"; gene_type "
At_chr4	Cufflinks	exon	14957791	14957886	.	-	.	gene_id "XLOC_069446"; transcript_id "Novel_000881"; exon_number "4"; gene_type "
At_chr4	Cufflinks	exon	14954219	14956295	.	-	.	gene_id "XLOC_069446"; transcript_id "Novel_000882"; exon_number "1"; gene_type "
At_chr4	Cufflinks	exon	14956367	14957701	.	-	.	gene_id "XLOC_069446"; transcript_id "Novel_000882"; exon_number "2"; gene_type "
At_chr4	Cufflinks	exon	14954757	14955295	.	-	.	gene_id "XLOC_069446"; transcript_id "Novel_000883"; exon_number "1"; gene_type "
At_chr4	Cufflinks	exon	14955486	14955653	.	-	.	gene_id "XLOC_069446"; transcript_id "Novel_000883"; exon_number "2"; gene_type "
At_chr4	Cufflinks	exon	14955918	14956295	.	-	.	gene_id "XLOC_069446"; transcript_id "Novel_000883"; exon_number "3"; gene_type "
At_chr4	Cufflinks	exon	14956367	14956646	.	-	.	gene_id "XLOC_069446"; transcript_id "Novel_000883"; exon_number "4"; gene_type "
At_chr4	Cufflinks	exon	14957006	14957121	.	-	.	gene_id "XLOC_069446"; transcript_id "Novel_000883"; exon_number "5"; gene_type "
At_chr4	Cufflinks	exon	14957791	14957826	.	-	.	gene_id "XLOC_069446"; transcript_id "Novel_000883"; exon_number "6"; gene_type "
At_chr4	Cufflinks	exon	15778617	15786449	.	-	.	gene_id "XLOC_069485"; transcript_id "Novel_000884"; exon_number "1"; gene_type "
At_chr4	Cufflinks	exon	15786644	15787697	.	-	.	gene_id "XLOC_069485"; transcript_id "Novel_000884"; exon_number "2"; gene_type "
At_chr4	Cufflinks	exon	23414641	23415688	.	-	.	gene_id "XLOC_069758"; transcript_id "Novel_000885"; exon_number "1"; gene_type "
At_chr4	Cufflinks	exon	23486115	23486237	.	-	.	gene_id "XLOC_069767"; transcript_id "Novel_000886"; exon_number "1"; gene_type "

At_chr4	Cufflinks	exon	23487252	23487330	.	-	.	gene_id "XLOC_069767"; transcript_id "Novel_000886"; exon_number "2"; gene_type "
At_chr4	Cufflinks	exon	23487477	23487511	.	-	.	gene_id "XLOC_069767"; transcript_id "Novel_000886"; exon_number "3"; gene_type "
At_chr4	Cufflinks	exon	32959888	32960042	.	-	.	gene_id "XLOC_070023"; transcript_id "Novel_000887"; exon_number "1"; gene_type "
At_chr4	Cufflinks	exon	32960728	32961874	.	-	.	gene_id "XLOC_070023"; transcript_id "Novel_000887"; exon_number "2"; gene_type "
At_chr4	Cufflinks	exon	32962104	32962512	.	-	.	gene_id "XLOC_070023"; transcript_id "Novel_000888"; exon_number "1"; gene_type "
At_chr4	Cufflinks	exon	32963750	32963915	.	-	.	gene_id "XLOC_070023"; transcript_id "Novel_000888"; exon_number "2"; gene_type "
At_chr4	Cufflinks	exon	32965176	32965477	.	-	.	gene_id "XLOC_070023"; transcript_id "Novel_000888"; exon_number "3"; gene_type "
At_chr4	Cufflinks	exon	34540724	34541416	.	-	.	gene_id "XLOC_070067"; transcript_id "Novel_000889"; exon_number "1"; gene_type "
At_chr4	Cufflinks	exon	34548592	34548650	.	-	.	gene_id "XLOC_070067"; transcript_id "Novel_000889"; exon_number "2"; gene_type "
At_chr4	Cufflinks	exon	34549355	34549658	.	-	.	gene_id "XLOC_070067"; transcript_id "Novel_000889"; exon_number "3"; gene_type "
At_chr4	Cufflinks	exon	34637474	34637675	.	-	.	gene_id "XLOC_070070"; transcript_id "Novel_000890"; exon_number "1"; gene_type "
At_chr4	Cufflinks	exon	34739645	34739994	.	-	.	gene_id "XLOC_070082"; transcript_id "Novel_000891"; exon_number "1"; gene_type "
At_chr4	Cufflinks	exon	34740268	34741967	.	-	.	gene_id "XLOC_070082"; transcript_id "Novel_000891"; exon_number "2"; gene_type "
At_chr4	Cufflinks	exon	35761605	35761875	.	-	.	gene_id "XLOC_070115"; transcript_id "Novel_000892"; exon_number "1"; gene_type "
At_chr4	Cufflinks	exon	35762449	35762631	.	-	.	gene_id "XLOC_070115"; transcript_id "Novel_000892"; exon_number "2"; gene_type "
At_chr4	Cufflinks	exon	35762735	35762860	.	-	.	gene_id "XLOC_070115"; transcript_id "Novel_000892"; exon_number "3"; gene_type "
At_chr4	Cufflinks	exon	35762963	35763052	.	-	.	gene_id "XLOC_070115"; transcript_id "Novel_000892"; exon_number "4"; gene_type "
At_chr4	Cufflinks	exon	35763145	35763236	.	-	.	gene_id "XLOC_070115"; transcript_id "Novel_000892"; exon_number "5"; gene_type "
At_chr4	Cufflinks	exon	36738860	36739812	.	-	.	gene_id "XLOC_070221"; transcript_id "Novel_000893"; exon_number "1"; gene_type "
At_chr4	Cufflinks	exon	36740392	36740960	.	-	.	gene_id "XLOC_070221"; transcript_id "Novel_000893"; exon_number "2"; gene_type "
At_chr4	Cufflinks	exon	36738948	36739812	.	-	.	gene_id "XLOC_070221"; transcript_id "Novel_000894"; exon_number "1"; gene_type "
At_chr4	Cufflinks	exon	36740392	36740521	.	-	.	gene_id "XLOC_070221"; transcript_id "Novel_000894"; exon_number "2"; gene_type "
At_chr4	Cufflinks	exon	36740629	36741242	.	-	.	gene_id "XLOC_070221"; transcript_id "Novel_000894"; exon_number "3"; gene_type "
At_chr4	Cufflinks	exon	37307721	37307925	.	-	.	gene_id "XLOC_070313"; transcript_id "Novel_000895"; exon_number "1"; gene_type "
At_chr4	Cufflinks	exon	37308618	37308666	.	-	.	gene_id "XLOC_070313"; transcript_id "Novel_000895"; exon_number "2"; gene_type "
At_chr4	Cufflinks	exon	37310222	37310600	.	-	.	gene_id "XLOC_070313"; transcript_id "Novel_000895"; exon_number "3"; gene_type "
At_chr4	Cufflinks	exon	37459095	37459503	.	-	.	gene_id "XLOC_070342"; transcript_id "Novel_000896"; exon_number "1"; gene_type "
At_chr4	Cufflinks	exon	37459851	37459902	.	-	.	gene_id "XLOC_070342"; transcript_id "Novel_000896"; exon_number "2"; gene_type "
At_chr4	Cufflinks	exon	37460193	37460266	.	-	.	gene_id "XLOC_070342"; transcript_id "Novel_000896"; exon_number "3"; gene_type "
At_chr4	Cufflinks	exon	37460612	37460661	.	-	.	gene_id "XLOC_070342"; transcript_id "Novel_000896"; exon_number "4"; gene_type "
At_chr4	Cufflinks	exon	37460754	37460835	.	-	.	gene_id "XLOC_070342"; transcript_id "Novel_000896"; exon_number "5"; gene_type "
At_chr4	Cufflinks	exon	37461179	37461221	.	-	.	gene_id "XLOC_070342"; transcript_id "Novel_000896"; exon_number "6"; gene_type "
At_chr4	Cufflinks	exon	37461297	37461599	.	-	.	gene_id "XLOC_070342"; transcript_id "Novel_000896"; exon_number "7"; gene_type "
At_chr4	Cufflinks	exon	37459543	37459769	.	-	.	gene_id "XLOC_070342"; transcript_id "Novel_000897"; exon_number "1"; gene_type "
At_chr4	Cufflinks	exon	37459851	37459902	.	-	.	gene_id "XLOC_070342"; transcript_id "Novel_000897"; exon_number "2"; gene_type "

At_chr4	Cufflinks	exon	37460193	37460266	.	-	.	gene_id "XLOC_070342"; transcript_id "Novel_000897"; exon_number "3"; gene_type "
At_chr4	Cufflinks	exon	37460612	37460661	.	-	.	gene_id "XLOC_070342"; transcript_id "Novel_000897"; exon_number "4"; gene_type "
At_chr4	Cufflinks	exon	37460754	37460835	.	-	.	gene_id "XLOC_070342"; transcript_id "Novel_000897"; exon_number "5"; gene_type "
At_chr4	Cufflinks	exon	37461179	37461221	.	-	.	gene_id "XLOC_070342"; transcript_id "Novel_000897"; exon_number "6"; gene_type "
At_chr4	Cufflinks	exon	37461297	37461603	.	-	.	gene_id "XLOC_070342"; transcript_id "Novel_000897"; exon_number "7"; gene_type "
At_chr4	Cufflinks	exon	37698984	37700154	.	-	.	gene_id "XLOC_070375"; transcript_id "Novel_000898"; exon_number "1"; gene_type "
At_chr4	Cufflinks	exon	37762382	37762732	.	-	.	gene_id "XLOC_070389"; transcript_id "Novel_000899"; exon_number "1"; gene_type "
At_chr4	Cufflinks	exon	37763554	37763786	.	-	.	gene_id "XLOC_070389"; transcript_id "Novel_000899"; exon_number "2"; gene_type "
At_chr4	Cufflinks	exon	37764707	37765201	.	-	.	gene_id "XLOC_070389"; transcript_id "Novel_000899"; exon_number "3"; gene_type "
At_chr4	Cufflinks	exon	37763537	37763786	.	-	.	gene_id "XLOC_070389"; transcript_id "Novel_000900"; exon_number "1"; gene_type "
At_chr4	Cufflinks	exon	37764707	37765197	.	-	.	gene_id "XLOC_070389"; transcript_id "Novel_000900"; exon_number "2"; gene_type "
At_chr4	Cufflinks	exon	37898983	37899293	.	-	.	gene_id "XLOC_070426"; transcript_id "Novel_000901"; exon_number "1"; gene_type "
At_chr4	Cufflinks	exon	37899563	37899599	.	-	.	gene_id "XLOC_070426"; transcript_id "Novel_000901"; exon_number "2"; gene_type "
At_chr4	Cufflinks	exon	37900950	37901252	.	-	.	gene_id "XLOC_070426"; transcript_id "Novel_000901"; exon_number "3"; gene_type "
At_chr4	Cufflinks	exon	38154665	38155996	.	-	.	gene_id "XLOC_070487"; transcript_id "Novel_000902"; exon_number "1"; gene_type "
At_chr4	Cufflinks	exon	38156490	38156597	.	-	.	gene_id "XLOC_070487"; transcript_id "Novel_000902"; exon_number "2"; gene_type "
At_chr4	Cufflinks	exon	38332481	38335042	.	-	.	gene_id "XLOC_070522"; transcript_id "Novel_000903"; exon_number "1"; gene_type "
At_chr4	Cufflinks	exon	38336015	38336090	.	-	.	gene_id "XLOC_070522"; transcript_id "Novel_000903"; exon_number "2"; gene_type "
At_chr4	Cufflinks	exon	38336165	38336271	.	-	.	gene_id "XLOC_070522"; transcript_id "Novel_000903"; exon_number "3"; gene_type "
At_chr4	Cufflinks	exon	38332481	38335042	.	-	.	gene_id "XLOC_070522"; transcript_id "Novel_000904"; exon_number "1"; gene_type "
At_chr4	Cufflinks	exon	38336015	38336305	.	-	.	gene_id "XLOC_070522"; transcript_id "Novel_000904"; exon_number "2"; gene_type "
At_chr4	Cufflinks	exon	38338054	38339780	.	-	.	gene_id "XLOC_070522"; transcript_id "Novel_000905"; exon_number "1"; gene_type "
At_chr4	Cufflinks	exon	38341565	38341851	.	-	.	gene_id "XLOC_070522"; transcript_id "Novel_000905"; exon_number "2"; gene_type "
At_chr4	Cufflinks	exon	45137749	45137973	.	-	.	gene_id "XLOC_070736"; transcript_id "Novel_000906"; exon_number "1"; gene_type "
At_chr4	Cufflinks	exon	45138084	45138235	.	-	.	gene_id "XLOC_070736"; transcript_id "Novel_000906"; exon_number "2"; gene_type "
At_chr4	Cufflinks	exon	45139788	45139980	.	-	.	gene_id "XLOC_070736"; transcript_id "Novel_000906"; exon_number "3"; gene_type "
At_chr4	Cufflinks	exon	46482089	46482740	.	-	.	gene_id "XLOC_070773"; transcript_id "Novel_000907"; exon_number "1"; gene_type "
At_chr4	Cufflinks	exon	46482813	46482933	.	-	.	gene_id "XLOC_070773"; transcript_id "Novel_000907"; exon_number "2"; gene_type "
At_chr4	Cufflinks	exon	46485123	46485238	.	-	.	gene_id "XLOC_070773"; transcript_id "Novel_000907"; exon_number "3"; gene_type "
At_chr4	Cufflinks	exon	46482089	46482933	.	-	.	gene_id "XLOC_070773"; transcript_id "Novel_000908"; exon_number "1"; gene_type "
At_chr4	Cufflinks	exon	46485123	46485238	.	-	.	gene_id "XLOC_070773"; transcript_id "Novel_000908"; exon_number "2"; gene_type "
At_chr4	Cufflinks	exon	46859391	46859806	.	-	.	gene_id "XLOC_070780"; transcript_id "Novel_000909"; exon_number "1"; gene_type "
At_chr4	Cufflinks	exon	46859873	46860448	.	-	.	gene_id "XLOC_070780"; transcript_id "Novel_000909"; exon_number "2"; gene_type "
At_chr4	Cufflinks	exon	46860623	46864012	.	-	.	gene_id "XLOC_070780"; transcript_id "Novel_000909"; exon_number "3"; gene_type "
At_chr4	Cufflinks	exon	60814252	60814576	.	-	.	gene_id "XLOC_071142"; transcript_id "Novel_000910"; exon_number "1"; gene_type "

At_chr4	Cufflinks	exon	60814748	60814899	.	-	.	gene_id "XLOC_071142"; transcript_id "Novel_000910"; exon_number "2"; gene_type "
At_chr4	Cufflinks	exon	60815296	60815555	.	-	.	gene_id "XLOC_071142"; transcript_id "Novel_000910"; exon_number "3"; gene_type "
At_chr4	Cufflinks	exon	60816276	60816315	.	-	.	gene_id "XLOC_071142"; transcript_id "Novel_000910"; exon_number "4"; gene_type "
At_chr4	Cufflinks	exon	60816479	60816582	.	-	.	gene_id "XLOC_071142"; transcript_id "Novel_000910"; exon_number "5"; gene_type "
At_chr4	Cufflinks	exon	60817214	60817415	.	-	.	gene_id "XLOC_071142"; transcript_id "Novel_000910"; exon_number "6"; gene_type "
At_chr4	Cufflinks	exon	60817678	60817816	.	-	.	gene_id "XLOC_071142"; transcript_id "Novel_000910"; exon_number "7"; gene_type "
At_chr4	Cufflinks	exon	60818085	60818153	.	-	.	gene_id "XLOC_071142"; transcript_id "Novel_000910"; exon_number "8"; gene_type "
At_chr4	Cufflinks	exon	60818256	60818354	.	-	.	gene_id "XLOC_071142"; transcript_id "Novel_000910"; exon_number "9"; gene_type "
At_chr4	Cufflinks	exon	60818439	60818610	.	-	.	gene_id "XLOC_071142"; transcript_id "Novel_000910"; exon_number "10"; gene_type
At_chr4	Cufflinks	exon	60819003	60819045	.	-	.	gene_id "XLOC_071142"; transcript_id "Novel_000910"; exon_number "11"; gene_type
At_chr4	Cufflinks	exon	60819685	60819751	.	-	.	gene_id "XLOC_071142"; transcript_id "Novel_000910"; exon_number "12"; gene_type
At_chr4	Cufflinks	exon	60820295	60820385	.	-	.	gene_id "XLOC_071142"; transcript_id "Novel_000910"; exon_number "13"; gene_type
At_chr4	Cufflinks	exon	60820479	60820571	.	-	.	gene_id "XLOC_071142"; transcript_id "Novel_000910"; exon_number "14"; gene_type
At_chr4	Cufflinks	exon	60820685	60820997	.	-	.	gene_id "XLOC_071142"; transcript_id "Novel_000910"; exon_number "15"; gene_type
At_chr4	Cufflinks	exon	60821122	60821377	.	-	.	gene_id "XLOC_071142"; transcript_id "Novel_000910"; exon_number "16"; gene_type
At_chr4	Cufflinks	exon	60815913	60816315	.	-	.	gene_id "XLOC_071142"; transcript_id "Novel_000911"; exon_number "1"; gene_type "
At_chr4	Cufflinks	exon	60816479	60816582	.	-	.	gene_id "XLOC_071142"; transcript_id "Novel_000911"; exon_number "2"; gene_type "
At_chr4	Cufflinks	exon	60817214	60817415	.	-	.	gene_id "XLOC_071142"; transcript_id "Novel_000911"; exon_number "3"; gene_type "
At_chr4	Cufflinks	exon	60817678	60817816	.	-	.	gene_id "XLOC_071142"; transcript_id "Novel_000911"; exon_number "4"; gene_type "
At_chr4	Cufflinks	exon	60818085	60818153	.	-	.	gene_id "XLOC_071142"; transcript_id "Novel_000911"; exon_number "5"; gene_type "
At_chr4	Cufflinks	exon	60818256	60818354	.	-	.	gene_id "XLOC_071142"; transcript_id "Novel_000911"; exon_number "6"; gene_type "
At_chr4	Cufflinks	exon	60818439	60818610	.	-	.	gene_id "XLOC_071142"; transcript_id "Novel_000911"; exon_number "7"; gene_type "
At_chr4	Cufflinks	exon	60819003	60819045	.	-	.	gene_id "XLOC_071142"; transcript_id "Novel_000911"; exon_number "8"; gene_type "
At_chr4	Cufflinks	exon	60819685	60819751	.	-	.	gene_id "XLOC_071142"; transcript_id "Novel_000911"; exon_number "9"; gene_type "
At_chr4	Cufflinks	exon	60820295	60820385	.	-	.	gene_id "XLOC_071142"; transcript_id "Novel_000911"; exon_number "10"; gene_type
At_chr4	Cufflinks	exon	60820479	60820571	.	-	.	gene_id "XLOC_071142"; transcript_id "Novel_000911"; exon_number "11"; gene_type
At_chr4	Cufflinks	exon	60820685	60820997	.	-	.	gene_id "XLOC_071142"; transcript_id "Novel_000911"; exon_number "12"; gene_type
At_chr4	Cufflinks	exon	60821122	60821337	.	-	.	gene_id "XLOC_071142"; transcript_id "Novel_000911"; exon_number "13"; gene_type
At_chr4	Cufflinks	exon	60818326	60819045	.	-	.	gene_id "XLOC_071142"; transcript_id "Novel_000912"; exon_number "1"; gene_type "
At_chr4	Cufflinks	exon	60819685	60819751	.	-	.	gene_id "XLOC_071142"; transcript_id "Novel_000912"; exon_number "2"; gene_type "
At_chr4	Cufflinks	exon	60820295	60820385	.	-	.	gene_id "XLOC_071142"; transcript_id "Novel_000912"; exon_number "3"; gene_type "
At_chr4	Cufflinks	exon	60820479	60820571	.	-	.	gene_id "XLOC_071142"; transcript_id "Novel_000912"; exon_number "4"; gene_type "
At_chr4	Cufflinks	exon	60820685	60820997	.	-	.	gene_id "XLOC_071142"; transcript_id "Novel_000912"; exon_number "5"; gene_type "
At_chr4	Cufflinks	exon	60821122	60821331	.	-	.	gene_id "XLOC_071142"; transcript_id "Novel_000912"; exon_number "6"; gene_type "
At_chr4	Cufflinks	exon	63261789	63262324	.	-	.	gene_id "XLOC_071230"; transcript_id "Novel_000913"; exon_number "1"; gene_type "

At_chr4	Cufflinks	exon	63262419	63262524	.	-	.	gene_id "XLOC_071230"; transcript_id "Novel_000913"; exon_number "2"; gene_type "
At_chr4	Cufflinks	exon	63262658	63262903	.	-	.	gene_id "XLOC_071230"; transcript_id "Novel_000913"; exon_number "3"; gene_type "
At_chr4	Cufflinks	exon	66091258	66093311	.	-	.	gene_id "XLOC_071324"; transcript_id "Novel_000914"; exon_number "1"; gene_type "
At_chr4	Cufflinks	exon	66093447	66093492	.	-	.	gene_id "XLOC_071324"; transcript_id "Novel_000914"; exon_number "2"; gene_type "
At_chr4	Cufflinks	exon	66094106	66094687	.	-	.	gene_id "XLOC_071324"; transcript_id "Novel_000914"; exon_number "3"; gene_type "
At_chr4	Cufflinks	exon	66094811	66094936	.	-	.	gene_id "XLOC_071324"; transcript_id "Novel_000914"; exon_number "4"; gene_type "
At_chr4	Cufflinks	exon	66093605	66094687	.	-	.	gene_id "XLOC_071324"; transcript_id "Novel_000915"; exon_number "1"; gene_type "
At_chr4	Cufflinks	exon	66094811	66094918	.	-	.	gene_id "XLOC_071324"; transcript_id "Novel_000915"; exon_number "2"; gene_type "
At_chr4	Cufflinks	exon	69047154	69047177	.	-	.	gene_id "XLOC_071428"; transcript_id "Novel_000916"; exon_number "1"; gene_type "
At_chr4	Cufflinks	exon	69047573	69047637	.	-	.	gene_id "XLOC_071428"; transcript_id "Novel_000916"; exon_number "2"; gene_type "
At_chr4	Cufflinks	exon	69048179	69048333	.	-	.	gene_id "XLOC_071428"; transcript_id "Novel_000916"; exon_number "3"; gene_type "
At_chr4	Cufflinks	exon	69048361	69048452	.	-	.	gene_id "XLOC_071428"; transcript_id "Novel_000917"; exon_number "1"; gene_type "
At_chr4	Cufflinks	exon	69048552	69048662	.	-	.	gene_id "XLOC_071428"; transcript_id "Novel_000917"; exon_number "2"; gene_type "
At_chr4	Cufflinks	exon	82326656	82327397	.	-	.	gene_id "XLOC_071933"; transcript_id "Novel_000918"; exon_number "1"; gene_type "
At_chr4	Cufflinks	exon	82327545	82327972	.	-	.	gene_id "XLOC_071933"; transcript_id "Novel_000918"; exon_number "2"; gene_type "
At_chr4	Cufflinks	exon	82875226	82876900	.	-	.	gene_id "XLOC_071962"; transcript_id "Novel_000919"; exon_number "1"; gene_type "
At_chr4	Cufflinks	exon	83739414	83739875	.	-	.	gene_id "XLOC_072006"; transcript_id "Novel_000920"; exon_number "1"; gene_type "
At_chr4	Cufflinks	exon	83739962	83740264	.	-	.	gene_id "XLOC_072006"; transcript_id "Novel_000920"; exon_number "2"; gene_type "
At_chr4	Cufflinks	exon	84141680	84142499	.	-	.	gene_id "XLOC_072037"; transcript_id "Novel_000921"; exon_number "1"; gene_type "
At_chr4	Cufflinks	exon	84143001	84144833	.	-	.	gene_id "XLOC_072037"; transcript_id "Novel_000921"; exon_number "2"; gene_type "
At_chr4	Cufflinks	exon	84141697	84142499	.	-	.	gene_id "XLOC_072037"; transcript_id "Novel_000922"; exon_number "1"; gene_type "
At_chr4	Cufflinks	exon	84143001	84143452	.	-	.	gene_id "XLOC_072037"; transcript_id "Novel_000922"; exon_number "2"; gene_type "
At_chr4	Cufflinks	exon	84144104	84144197	.	-	.	gene_id "XLOC_072037"; transcript_id "Novel_000922"; exon_number "3"; gene_type "
At_chr4	Cufflinks	exon	84144273	84144618	.	-	.	gene_id "XLOC_072037"; transcript_id "Novel_000922"; exon_number "4"; gene_type "
At_chr4	Cufflinks	exon	84144774	84144850	.	-	.	gene_id "XLOC_072037"; transcript_id "Novel_000922"; exon_number "5"; gene_type "
At_chr4	Cufflinks	exon	84265585	84265751	.	-	.	gene_id "XLOC_072048"; transcript_id "Novel_000923"; exon_number "1"; gene_type "
At_chr4	Cufflinks	exon	84265844	84265877	.	-	.	gene_id "XLOC_072048"; transcript_id "Novel_000923"; exon_number "2"; gene_type "
At_chr4	Cufflinks	exon	84385054	84385557	.	-	.	gene_id "XLOC_072063"; transcript_id "Novel_000924"; exon_number "1"; gene_type "
At_chr4	Cufflinks	exon	84386790	84387015	.	-	.	gene_id "XLOC_072063"; transcript_id "Novel_000924"; exon_number "2"; gene_type "
At_chr4	Cufflinks	exon	84387096	84387452	.	-	.	gene_id "XLOC_072063"; transcript_id "Novel_000924"; exon_number "3"; gene_type "
At_chr4	Cufflinks	exon	84435554	84435929	.	-	.	gene_id "XLOC_072067"; transcript_id "Novel_000925"; exon_number "1"; gene_type "
At_chr4	Cufflinks	exon	84437234	84437357	.	-	.	gene_id "XLOC_072067"; transcript_id "Novel_000925"; exon_number "2"; gene_type "
At_chr4	Cufflinks	exon	84437451	84437604	.	-	.	gene_id "XLOC_072067"; transcript_id "Novel_000925"; exon_number "3"; gene_type "
At_chr4	Cufflinks	exon	84438365	84438742	.	-	.	gene_id "XLOC_072067"; transcript_id "Novel_000925"; exon_number "4"; gene_type "
At_chr4	Cufflinks	exon	84439129	84439289	.	-	.	gene_id "XLOC_072067"; transcript_id "Novel_000925"; exon_number "5"; gene_type "

At_chr4	Cufflinks	exon	84439846	84439865	.	-	.	gene_id "XLOC_072067"; transcript_id "Novel_000925"; exon_number "6"; gene_type "
At_chr4	Cufflinks	exon	84441027	84441985	.	-	.	gene_id "XLOC_072067"; transcript_id "Novel_000926"; exon_number "1"; gene_type "
At_chr4	Cufflinks	exon	84442083	84442519	.	-	.	gene_id "XLOC_072067"; transcript_id "Novel_000926"; exon_number "2"; gene_type "
At_chr4	Cufflinks	exon	84442727	84443495	.	-	.	gene_id "XLOC_072067"; transcript_id "Novel_000926"; exon_number "3"; gene_type "
At_chr4	Cufflinks	exon	84443604	84444009	.	-	.	gene_id "XLOC_072069"; transcript_id "Novel_000927"; exon_number "1"; gene_type "
At_chr4	Cufflinks	exon	84444143	84444493	.	-	.	gene_id "XLOC_072069"; transcript_id "Novel_000927"; exon_number "2"; gene_type "
At_chr4	Cufflinks	exon	84444629	84444759	.	-	.	gene_id "XLOC_072069"; transcript_id "Novel_000927"; exon_number "3"; gene_type "
At_chr4	Cufflinks	exon	84444872	84444944	.	-	.	gene_id "XLOC_072069"; transcript_id "Novel_000927"; exon_number "4"; gene_type "
At_chr4	Cufflinks	exon	84446902	84447486	.	-	.	gene_id "XLOC_072069"; transcript_id "Novel_000927"; exon_number "5"; gene_type "
At_chr4	Cufflinks	exon	84443614	84444009	.	-	.	gene_id "XLOC_072069"; transcript_id "Novel_000928"; exon_number "1"; gene_type "
At_chr4	Cufflinks	exon	84444143	84444759	.	-	.	gene_id "XLOC_072069"; transcript_id "Novel_000928"; exon_number "2"; gene_type "
At_chr4	Cufflinks	exon	84444872	84444944	.	-	.	gene_id "XLOC_072069"; transcript_id "Novel_000928"; exon_number "3"; gene_type "
At_chr4	Cufflinks	exon	84446902	84447509	.	-	.	gene_id "XLOC_072069"; transcript_id "Novel_000928"; exon_number "4"; gene_type "
At_chr4	Cufflinks	exon	84451635	84454072	.	-	.	gene_id "XLOC_072072"; transcript_id "Novel_000929"; exon_number "1"; gene_type "
At_chr4	Cufflinks	exon	84454170	84454608	.	-	.	gene_id "XLOC_072072"; transcript_id "Novel_000929"; exon_number "2"; gene_type "
At_chr4	Cufflinks	exon	84494485	84496617	.	-	.	gene_id "XLOC_072076"; transcript_id "Novel_000930"; exon_number "1"; gene_type "
At_chr4	Cufflinks	exon	84498501	84499335	.	-	.	gene_id "XLOC_072076"; transcript_id "Novel_000930"; exon_number "2"; gene_type "
At_chr4	Cufflinks	exon	86104914	86104967	.	-	.	gene_id "XLOC_072239"; transcript_id "Novel_000931"; exon_number "1"; gene_type "
At_chr4	Cufflinks	exon	86105070	86105181	.	-	.	gene_id "XLOC_072239"; transcript_id "Novel_000931"; exon_number "2"; gene_type "
At_chr4	Cufflinks	exon	86105268	86105519	.	-	.	gene_id "XLOC_072239"; transcript_id "Novel_000931"; exon_number "3"; gene_type "
At_chr4	Cufflinks	exon	86105621	86105722	.	-	.	gene_id "XLOC_072239"; transcript_id "Novel_000931"; exon_number "4"; gene_type "
At_chr4	Cufflinks	exon	86105802	86105919	.	-	.	gene_id "XLOC_072239"; transcript_id "Novel_000931"; exon_number "5"; gene_type "
At_chr4	Cufflinks	exon	86106065	86106203	.	-	.	gene_id "XLOC_072239"; transcript_id "Novel_000931"; exon_number "6"; gene_type "
At_chr4	Cufflinks	exon	86106440	86106627	.	-	.	gene_id "XLOC_072239"; transcript_id "Novel_000931"; exon_number "7"; gene_type "
At_chr4	Cufflinks	exon	86106711	86106821	.	-	.	gene_id "XLOC_072239"; transcript_id "Novel_000931"; exon_number "8"; gene_type "
At_chr4	Cufflinks	exon	86106962	86107024	.	-	.	gene_id "XLOC_072239"; transcript_id "Novel_000931"; exon_number "9"; gene_type "
At_chr4	Cufflinks	exon	86107556	86107655	.	-	.	gene_id "XLOC_072239"; transcript_id "Novel_000931"; exon_number "10"; gene_type
At_chr4	Cufflinks	exon	86107899	86108437	.	-	.	gene_id "XLOC_072239"; transcript_id "Novel_000931"; exon_number "11"; gene_type
At_chr4	Cufflinks	exon	86108577	86108887	.	-	.	gene_id "XLOC_072239"; transcript_id "Novel_000931"; exon_number "12"; gene_type
At_chr4	Cufflinks	exon	87611618	87611960	.	-	.	gene_id "XLOC_072364"; transcript_id "Novel_000932"; exon_number "1"; gene_type "
At_chr4	Cufflinks	exon	87612970	87613049	.	-	.	gene_id "XLOC_072364"; transcript_id "Novel_000932"; exon_number "2"; gene_type "
At_chr4	Cufflinks	exon	87613135	87615214	.	-	.	gene_id "XLOC_072364"; transcript_id "Novel_000932"; exon_number "3"; gene_type "
At_chr4	Cufflinks	exon	88392653	88393042	.	-	.	gene_id "XLOC_072414"; transcript_id "Novel_000933"; exon_number "1"; gene_type "
At_chr4	Cufflinks	exon	89374380	89374631	.	-	.	gene_id "XLOC_072527"; transcript_id "Novel_000934"; exon_number "1"; gene_type "
At_chr4	Cufflinks	exon	89535176	89536347	.	-	.	gene_id "XLOC_072533"; transcript_id "Novel_000935"; exon_number "1"; gene_type "

At_chr4	Cufflinks	exon	89537032	89537332	.	-	.	gene_id "XLOC_072533"; transcript_id "Novel_000935"; exon_number "2"; gene_type "
At_chr5	Cufflinks	exon	879697	879833	.	+	.	gene_id "XLOC_072863"; transcript_id "Novel_000936"; exon_number "1"; gene_type "
At_chr5	Cufflinks	exon	880750	882421	.	+	.	gene_id "XLOC_072863"; transcript_id "Novel_000936"; exon_number "2"; gene_type "
At_chr5	Cufflinks	exon	882536	883120	.	+	.	gene_id "XLOC_072863"; transcript_id "Novel_000936"; exon_number "3"; gene_type "
At_chr5	Cufflinks	exon	1733497	1733982	.	+	.	gene_id "XLOC_073044"; transcript_id "Novel_000937"; exon_number "1"; gene_type "
At_chr5	Cufflinks	exon	1734094	1734363	.	+	.	gene_id "XLOC_073044"; transcript_id "Novel_000937"; exon_number "2"; gene_type "
At_chr5	Cufflinks	exon	1735354	1736233	.	+	.	gene_id "XLOC_073044"; transcript_id "Novel_000937"; exon_number "3"; gene_type "
At_chr5	Cufflinks	exon	1740867	1741340	.	+	.	gene_id "XLOC_073045"; transcript_id "Novel_000938"; exon_number "1"; gene_type "
At_chr5	Cufflinks	exon	1741421	1741690	.	+	.	gene_id "XLOC_073045"; transcript_id "Novel_000938"; exon_number "2"; gene_type "
At_chr5	Cufflinks	exon	1741967	1743583	.	+	.	gene_id "XLOC_073045"; transcript_id "Novel_000938"; exon_number "3"; gene_type "
At_chr5	Cufflinks	exon	1740867	1741340	.	+	.	gene_id "XLOC_073045"; transcript_id "Novel_000939"; exon_number "1"; gene_type "
At_chr5	Cufflinks	exon	1741421	1741690	.	+	.	gene_id "XLOC_073045"; transcript_id "Novel_000939"; exon_number "2"; gene_type "
At_chr5	Cufflinks	exon	1741873	1743583	.	+	.	gene_id "XLOC_073045"; transcript_id "Novel_000939"; exon_number "3"; gene_type "
At_chr5	Cufflinks	exon	1740873	1741340	.	+	.	gene_id "XLOC_073045"; transcript_id "Novel_000940"; exon_number "1"; gene_type "
At_chr5	Cufflinks	exon	1741421	1742972	.	+	.	gene_id "XLOC_073045"; transcript_id "Novel_000940"; exon_number "2"; gene_type "
At_chr5	Cufflinks	exon	1862485	1863001	.	+	.	gene_id "XLOC_073074"; transcript_id "Novel_000941"; exon_number "1"; gene_type "
At_chr5	Cufflinks	exon	1863114	1863412	.	+	.	gene_id "XLOC_073074"; transcript_id "Novel_000941"; exon_number "2"; gene_type "
At_chr5	Cufflinks	exon	1864320	1864460	.	+	.	gene_id "XLOC_073074"; transcript_id "Novel_000941"; exon_number "3"; gene_type "
At_chr5	Cufflinks	exon	1864555	1864854	.	+	.	gene_id "XLOC_073074"; transcript_id "Novel_000941"; exon_number "4"; gene_type "
At_chr5	Cufflinks	exon	2317999	2318574	.	+	.	gene_id "XLOC_073144"; transcript_id "Novel_000942"; exon_number "1"; gene_type "
At_chr5	Cufflinks	exon	2318676	2319054	.	+	.	gene_id "XLOC_073144"; transcript_id "Novel_000942"; exon_number "2"; gene_type "
At_chr5	Cufflinks	exon	2320176	2320237	.	+	.	gene_id "XLOC_073144"; transcript_id "Novel_000942"; exon_number "3"; gene_type "
At_chr5	Cufflinks	exon	2320342	2320385	.	+	.	gene_id "XLOC_073144"; transcript_id "Novel_000942"; exon_number "4"; gene_type "
At_chr5	Cufflinks	exon	2320575	2320677	.	+	.	gene_id "XLOC_073144"; transcript_id "Novel_000942"; exon_number "5"; gene_type "
At_chr5	Cufflinks	exon	2320901	2320902	.	+	.	gene_id "XLOC_073144"; transcript_id "Novel_000942"; exon_number "6"; gene_type "
At_chr5	Cufflinks	exon	3715613	3715805	.	+	.	gene_id "XLOC_073368"; transcript_id "Novel_000943"; exon_number "1"; gene_type "
At_chr5	Cufflinks	exon	3716101	3718796	.	+	.	gene_id "XLOC_073368"; transcript_id "Novel_000943"; exon_number "2"; gene_type "
At_chr5	Cufflinks	exon	4240968	4241119	.	+	.	gene_id "XLOC_073418"; transcript_id "Novel_000944"; exon_number "1"; gene_type "
At_chr5	Cufflinks	exon	4241202	4241263	.	+	.	gene_id "XLOC_073418"; transcript_id "Novel_000944"; exon_number "2"; gene_type "
At_chr5	Cufflinks	exon	4241510	4241570	.	+	.	gene_id "XLOC_073418"; transcript_id "Novel_000944"; exon_number "3"; gene_type "
At_chr5	Cufflinks	exon	4241722	4241801	.	+	.	gene_id "XLOC_073418"; transcript_id "Novel_000944"; exon_number "4"; gene_type "
At_chr5	Cufflinks	exon	4242089	4242159	.	+	.	gene_id "XLOC_073418"; transcript_id "Novel_000944"; exon_number "5"; gene_type "
At_chr5	Cufflinks	exon	4242436	4242525	.	+	.	gene_id "XLOC_073418"; transcript_id "Novel_000944"; exon_number "6"; gene_type "
At_chr5	Cufflinks	exon	4242639	4242749	.	+	.	gene_id "XLOC_073418"; transcript_id "Novel_000944"; exon_number "7"; gene_type "
At_chr5	Cufflinks	exon	4242821	4243098	.	+	.	gene_id "XLOC_073418"; transcript_id "Novel_000944"; exon_number "8"; gene_type "

At_chr5	Cufflinks	exon	4278318	4278500	.	+	.	gene_id "XLOC_073425"; transcript_id "Novel_000945"; exon_number "1"; gene_type "
At_chr5	Cufflinks	exon	4278792	4278877	.	+	.	gene_id "XLOC_073425"; transcript_id "Novel_000945"; exon_number "2"; gene_type "
At_chr5	Cufflinks	exon	4282267	4282585	.	+	.	gene_id "XLOC_073427"; transcript_id "Novel_000946"; exon_number "1"; gene_type "
At_chr5	Cufflinks	exon	4283018	4283195	.	+	.	gene_id "XLOC_073427"; transcript_id "Novel_000946"; exon_number "2"; gene_type "
At_chr5	Cufflinks	exon	4283308	4283370	.	+	.	gene_id "XLOC_073427"; transcript_id "Novel_000946"; exon_number "3"; gene_type "
At_chr5	Cufflinks	exon	4283505	4283610	.	+	.	gene_id "XLOC_073427"; transcript_id "Novel_000946"; exon_number "4"; gene_type "
At_chr5	Cufflinks	exon	4283784	4283888	.	+	.	gene_id "XLOC_073427"; transcript_id "Novel_000946"; exon_number "5"; gene_type "
At_chr5	Cufflinks	exon	4283976	4284052	.	+	.	gene_id "XLOC_073427"; transcript_id "Novel_000946"; exon_number "6"; gene_type "
At_chr5	Cufflinks	exon	4284148	4285270	.	+	.	gene_id "XLOC_073427"; transcript_id "Novel_000946"; exon_number "7"; gene_type "
At_chr5	Cufflinks	exon	4282270	4282585	.	+	.	gene_id "XLOC_073427"; transcript_id "Novel_000947"; exon_number "1"; gene_type "
At_chr5	Cufflinks	exon	4283018	4283195	.	+	.	gene_id "XLOC_073427"; transcript_id "Novel_000947"; exon_number "2"; gene_type "
At_chr5	Cufflinks	exon	4283308	4283370	.	+	.	gene_id "XLOC_073427"; transcript_id "Novel_000947"; exon_number "3"; gene_type "
At_chr5	Cufflinks	exon	4283505	4283610	.	+	.	gene_id "XLOC_073427"; transcript_id "Novel_000947"; exon_number "4"; gene_type "
At_chr5	Cufflinks	exon	4283784	4283888	.	+	.	gene_id "XLOC_073427"; transcript_id "Novel_000947"; exon_number "5"; gene_type "
At_chr5	Cufflinks	exon	4283976	4284052	.	+	.	gene_id "XLOC_073427"; transcript_id "Novel_000947"; exon_number "6"; gene_type "
At_chr5	Cufflinks	exon	4284148	4284262	.	+	.	gene_id "XLOC_073427"; transcript_id "Novel_000947"; exon_number "7"; gene_type "
At_chr5	Cufflinks	exon	4284850	4284922	.	+	.	gene_id "XLOC_073427"; transcript_id "Novel_000947"; exon_number "8"; gene_type "
At_chr5	Cufflinks	exon	4285287	4285624	.	+	.	gene_id "XLOC_073427"; transcript_id "Novel_000947"; exon_number "9"; gene_type "
At_chr5	Cufflinks	exon	4305375	4305592	.	+	.	gene_id "XLOC_073429"; transcript_id "Novel_000948"; exon_number "1"; gene_type "
At_chr5	Cufflinks	exon	4305692	4306551	.	+	.	gene_id "XLOC_073429"; transcript_id "Novel_000948"; exon_number "2"; gene_type "
At_chr5	Cufflinks	exon	7314253	7314340	.	+	.	gene_id "XLOC_073719"; transcript_id "Novel_000949"; exon_number "1"; gene_type "
At_chr5	Cufflinks	exon	7314422	7314476	.	+	.	gene_id "XLOC_073719"; transcript_id "Novel_000949"; exon_number "2"; gene_type "
At_chr5	Cufflinks	exon	7314814	7314866	.	+	.	gene_id "XLOC_073719"; transcript_id "Novel_000949"; exon_number "3"; gene_type "
At_chr5	Cufflinks	exon	7315162	7315200	.	+	.	gene_id "XLOC_073719"; transcript_id "Novel_000949"; exon_number "4"; gene_type "
At_chr5	Cufflinks	exon	7315330	7315399	.	+	.	gene_id "XLOC_073719"; transcript_id "Novel_000949"; exon_number "5"; gene_type "
At_chr5	Cufflinks	exon	7315663	7315805	.	+	.	gene_id "XLOC_073719"; transcript_id "Novel_000949"; exon_number "6"; gene_type "
At_chr5	Cufflinks	exon	7782788	7783693	.	+	.	gene_id "XLOC_073753"; transcript_id "Novel_000950"; exon_number "1"; gene_type "
At_chr5	Cufflinks	exon	7783807	7784013	.	+	.	gene_id "XLOC_073753"; transcript_id "Novel_000950"; exon_number "2"; gene_type "
At_chr5	Cufflinks	exon	8411342	8411686	.	+	.	gene_id "XLOC_073825"; transcript_id "Novel_000951"; exon_number "1"; gene_type "
At_chr5	Cufflinks	exon	8411780	8411828	.	+	.	gene_id "XLOC_073825"; transcript_id "Novel_000951"; exon_number "2"; gene_type "
At_chr5	Cufflinks	exon	8412007	8412292	.	+	.	gene_id "XLOC_073825"; transcript_id "Novel_000951"; exon_number "3"; gene_type "
At_chr5	Cufflinks	exon	8413226	8413542	.	+	.	gene_id "XLOC_073825"; transcript_id "Novel_000951"; exon_number "4"; gene_type "
At_chr5	Cufflinks	exon	8413998	8414106	.	+	.	gene_id "XLOC_073825"; transcript_id "Novel_000951"; exon_number "5"; gene_type "
At_chr5	Cufflinks	exon	8414205	8415118	.	+	.	gene_id "XLOC_073825"; transcript_id "Novel_000951"; exon_number "6"; gene_type "
At_chr5	Cufflinks	exon	8411342	8411686	.	+	.	gene_id "XLOC_073825"; transcript_id "Novel_000952"; exon_number "1"; gene_type "

At_chr5	Cufflinks	exon	8411780	8411828	.	+	.	gene_id "XLOC_073825"; transcript_id "Novel_000952"; exon_number "2"; gene_type "
At_chr5	Cufflinks	exon	8412007	8412292	.	+	.	gene_id "XLOC_073825"; transcript_id "Novel_000952"; exon_number "3"; gene_type "
At_chr5	Cufflinks	exon	8413226	8413542	.	+	.	gene_id "XLOC_073825"; transcript_id "Novel_000952"; exon_number "4"; gene_type "
At_chr5	Cufflinks	exon	8413998	8415118	.	+	.	gene_id "XLOC_073825"; transcript_id "Novel_000952"; exon_number "5"; gene_type "
At_chr5	Cufflinks	exon	10007636	10007917	.	+	.	gene_id "XLOC_073954"; transcript_id "Novel_000953"; exon_number "1"; gene_type "
At_chr5	Cufflinks	exon	10008622	10008744	.	+	.	gene_id "XLOC_073954"; transcript_id "Novel_000953"; exon_number "2"; gene_type "
At_chr5	Cufflinks	exon	10008849	10008899	.	+	.	gene_id "XLOC_073954"; transcript_id "Novel_000953"; exon_number "3"; gene_type "
At_chr5	Cufflinks	exon	10009070	10009141	.	+	.	gene_id "XLOC_073954"; transcript_id "Novel_000953"; exon_number "4"; gene_type "
At_chr5	Cufflinks	exon	10009291	10009401	.	+	.	gene_id "XLOC_073954"; transcript_id "Novel_000953"; exon_number "5"; gene_type "
At_chr5	Cufflinks	exon	10009493	10009554	.	+	.	gene_id "XLOC_073954"; transcript_id "Novel_000953"; exon_number "6"; gene_type "
At_chr5	Cufflinks	exon	10010124	10010184	.	+	.	gene_id "XLOC_073954"; transcript_id "Novel_000953"; exon_number "7"; gene_type "
At_chr5	Cufflinks	exon	10010271	10010327	.	+	.	gene_id "XLOC_073954"; transcript_id "Novel_000953"; exon_number "8"; gene_type "
At_chr5	Cufflinks	exon	10010435	10010479	.	+	.	gene_id "XLOC_073954"; transcript_id "Novel_000953"; exon_number "9"; gene_type "
At_chr5	Cufflinks	exon	10011642	10012231	.	+	.	gene_id "XLOC_073954"; transcript_id "Novel_000953"; exon_number "10"; gene_type
At_chr5	Cufflinks	exon	12294281	12294485	.	+	.	gene_id "XLOC_074102"; transcript_id "Novel_000954"; exon_number "1"; gene_type "
At_chr5	Cufflinks	exon	12298657	12299253	.	+	.	gene_id "XLOC_074102"; transcript_id "Novel_000954"; exon_number "2"; gene_type "
At_chr5	Cufflinks	exon	12968787	12969065	.	+	.	gene_id "XLOC_074153"; transcript_id "Novel_000955"; exon_number "1"; gene_type "
At_chr5	Cufflinks	exon	12969238	12969886	.	+	.	gene_id "XLOC_074153"; transcript_id "Novel_000955"; exon_number "2"; gene_type "
At_chr5	Cufflinks	exon	13670952	13671262	.	+	.	gene_id "XLOC_074245"; transcript_id "Novel_000956"; exon_number "1"; gene_type "
At_chr5	Cufflinks	exon	13672700	13674705	.	+	.	gene_id "XLOC_074245"; transcript_id "Novel_000956"; exon_number "2"; gene_type "
At_chr5	Cufflinks	exon	16271844	16272102	.	+	.	gene_id "XLOC_074338"; transcript_id "Novel_000957"; exon_number "1"; gene_type "
At_chr5	Cufflinks	exon	16272184	16272255	.	+	.	gene_id "XLOC_074338"; transcript_id "Novel_000957"; exon_number "2"; gene_type "
At_chr5	Cufflinks	exon	16272332	16272544	.	+	.	gene_id "XLOC_074338"; transcript_id "Novel_000957"; exon_number "3"; gene_type "
At_chr5	Cufflinks	exon	16275816	16276127	.	+	.	gene_id "XLOC_074338"; transcript_id "Novel_000957"; exon_number "4"; gene_type "
At_chr5	Cufflinks	exon	16590891	16591279	.	+	.	gene_id "XLOC_074373"; transcript_id "Novel_000958"; exon_number "1"; gene_type "
At_chr5	Cufflinks	exon	16591964	16592071	.	+	.	gene_id "XLOC_074373"; transcript_id "Novel_000958"; exon_number "2"; gene_type "
At_chr5	Cufflinks	exon	16592837	16592991	.	+	.	gene_id "XLOC_074373"; transcript_id "Novel_000958"; exon_number "3"; gene_type "
At_chr5	Cufflinks	exon	16593098	16595136	.	+	.	gene_id "XLOC_074373"; transcript_id "Novel_000958"; exon_number "4"; gene_type "
At_chr5	Cufflinks	exon	16595240	16595398	.	+	.	gene_id "XLOC_074373"; transcript_id "Novel_000958"; exon_number "5"; gene_type "
At_chr5	Cufflinks	exon	16595534	16595600	.	+	.	gene_id "XLOC_074373"; transcript_id "Novel_000958"; exon_number "6"; gene_type "
At_chr5	Cufflinks	exon	16596196	16596509	.	+	.	gene_id "XLOC_074373"; transcript_id "Novel_000958"; exon_number "7"; gene_type "
At_chr5	Cufflinks	exon	16590909	16591279	.	+	.	gene_id "XLOC_074373"; transcript_id "Novel_000959"; exon_number "1"; gene_type "
At_chr5	Cufflinks	exon	16591964	16592071	.	+	.	gene_id "XLOC_074373"; transcript_id "Novel_000959"; exon_number "2"; gene_type "
At_chr5	Cufflinks	exon	16592837	16592991	.	+	.	gene_id "XLOC_074373"; transcript_id "Novel_000959"; exon_number "3"; gene_type "
At_chr5	Cufflinks	exon	16593364	16593416	.	+	.	gene_id "XLOC_074373"; transcript_id "Novel_000959"; exon_number "4"; gene_type "

At_chr5	Cufflinks	exon	16593891	16594982	.	+	.	gene_id "XLOC_074373"; transcript_id "Novel_000959"; exon_number "5"; gene_type "
At_chr5	Cufflinks	exon	16595071	16595136	.	+	.	gene_id "XLOC_074373"; transcript_id "Novel_000959"; exon_number "6"; gene_type "
At_chr5	Cufflinks	exon	16595240	16595398	.	+	.	gene_id "XLOC_074373"; transcript_id "Novel_000959"; exon_number "7"; gene_type "
At_chr5	Cufflinks	exon	16595534	16595600	.	+	.	gene_id "XLOC_074373"; transcript_id "Novel_000959"; exon_number "8"; gene_type "
At_chr5	Cufflinks	exon	16596318	16596520	.	+	.	gene_id "XLOC_074373"; transcript_id "Novel_000959"; exon_number "9"; gene_type "
At_chr5	Cufflinks	exon	16590909	16591279	.	+	.	gene_id "XLOC_074373"; transcript_id "Novel_000960"; exon_number "1"; gene_type "
At_chr5	Cufflinks	exon	16591964	16592071	.	+	.	gene_id "XLOC_074373"; transcript_id "Novel_000960"; exon_number "2"; gene_type "
At_chr5	Cufflinks	exon	16592837	16592991	.	+	.	gene_id "XLOC_074373"; transcript_id "Novel_000960"; exon_number "3"; gene_type "
At_chr5	Cufflinks	exon	16593364	16593416	.	+	.	gene_id "XLOC_074373"; transcript_id "Novel_000960"; exon_number "4"; gene_type "
At_chr5	Cufflinks	exon	16593891	16594982	.	+	.	gene_id "XLOC_074373"; transcript_id "Novel_000960"; exon_number "5"; gene_type "
At_chr5	Cufflinks	exon	16595071	16595136	.	+	.	gene_id "XLOC_074373"; transcript_id "Novel_000960"; exon_number "6"; gene_type "
At_chr5	Cufflinks	exon	16595240	16595398	.	+	.	gene_id "XLOC_074373"; transcript_id "Novel_000960"; exon_number "7"; gene_type "
At_chr5	Cufflinks	exon	16595534	16595600	.	+	.	gene_id "XLOC_074373"; transcript_id "Novel_000960"; exon_number "8"; gene_type "
At_chr5	Cufflinks	exon	16596196	16596520	.	+	.	gene_id "XLOC_074373"; transcript_id "Novel_000960"; exon_number "9"; gene_type "
At_chr5	Cufflinks	exon	16590913	16591279	.	+	.	gene_id "XLOC_074373"; transcript_id "Novel_000961"; exon_number "1"; gene_type "
At_chr5	Cufflinks	exon	16591964	16592071	.	+	.	gene_id "XLOC_074373"; transcript_id "Novel_000961"; exon_number "2"; gene_type "
At_chr5	Cufflinks	exon	16592837	16592991	.	+	.	gene_id "XLOC_074373"; transcript_id "Novel_000961"; exon_number "3"; gene_type "
At_chr5	Cufflinks	exon	16593098	16594982	.	+	.	gene_id "XLOC_074373"; transcript_id "Novel_000961"; exon_number "4"; gene_type "
At_chr5	Cufflinks	exon	16595071	16595136	.	+	.	gene_id "XLOC_074373"; transcript_id "Novel_000961"; exon_number "5"; gene_type "
At_chr5	Cufflinks	exon	16595240	16595398	.	+	.	gene_id "XLOC_074373"; transcript_id "Novel_000961"; exon_number "6"; gene_type "
At_chr5	Cufflinks	exon	16595534	16595600	.	+	.	gene_id "XLOC_074373"; transcript_id "Novel_000961"; exon_number "7"; gene_type "
At_chr5	Cufflinks	exon	16596196	16596510	.	+	.	gene_id "XLOC_074373"; transcript_id "Novel_000961"; exon_number "8"; gene_type "
At_chr5	Cufflinks	exon	24375694	24376171	.	+	.	gene_id "XLOC_074687"; transcript_id "Novel_000962"; exon_number "1"; gene_type "
At_chr5	Cufflinks	exon	24376561	24377469	.	+	.	gene_id "XLOC_074687"; transcript_id "Novel_000962"; exon_number "2"; gene_type "
At_chr5	Cufflinks	exon	24375694	24376171	.	+	.	gene_id "XLOC_074687"; transcript_id "Novel_000963"; exon_number "1"; gene_type "
At_chr5	Cufflinks	exon	24376561	24376758	.	+	.	gene_id "XLOC_074687"; transcript_id "Novel_000963"; exon_number "2"; gene_type "
At_chr5	Cufflinks	exon	24376913	24377511	.	+	.	gene_id "XLOC_074687"; transcript_id "Novel_000963"; exon_number "3"; gene_type "
At_chr5	Cufflinks	exon	24375704	24376771	.	+	.	gene_id "XLOC_074687"; transcript_id "Novel_000964"; exon_number "1"; gene_type "
At_chr5	Cufflinks	exon	24376913	24377448	.	+	.	gene_id "XLOC_074687"; transcript_id "Novel_000964"; exon_number "2"; gene_type "
At_chr5	Cufflinks	exon	25153298	25155276	.	+	.	gene_id "XLOC_074738"; transcript_id "Novel_000965"; exon_number "1"; gene_type "
At_chr5	Cufflinks	exon	27245663	27245877	.	+	.	gene_id "XLOC_074808"; transcript_id "Novel_000966"; exon_number "1"; gene_type "
At_chr5	Cufflinks	exon	38172111	38172336	.	+	.	gene_id "XLOC_075253"; transcript_id "Novel_000967"; exon_number "1"; gene_type "
At_chr5	Cufflinks	exon	38172553	38174033	.	+	.	gene_id "XLOC_075253"; transcript_id "Novel_000967"; exon_number "2"; gene_type "
At_chr5	Cufflinks	exon	38490440	38490611	.	+	.	gene_id "XLOC_075275"; transcript_id "Novel_000968"; exon_number "1"; gene_type "
At_chr5	Cufflinks	exon	38491834	38491887	.	+	.	gene_id "XLOC_075275"; transcript_id "Novel_000968"; exon_number "2"; gene_type "

At_chr5	Cufflinks	exon	38493309	38493400	.	+	.	gene_id "XLOC_075275"; transcript_id "Novel_000968"; exon_number "3"; gene_type "
At_chr5	Cufflinks	exon	38493526	38494440	.	+	.	gene_id "XLOC_075275"; transcript_id "Novel_000968"; exon_number "4"; gene_type "
At_chr5	Cufflinks	exon	38494521	38494969	.	+	.	gene_id "XLOC_075275"; transcript_id "Novel_000968"; exon_number "5"; gene_type "
At_chr5	Cufflinks	exon	38492905	38493400	.	+	.	gene_id "XLOC_075275"; transcript_id "Novel_000969"; exon_number "1"; gene_type "
At_chr5	Cufflinks	exon	38493526	38494440	.	+	.	gene_id "XLOC_075275"; transcript_id "Novel_000969"; exon_number "2"; gene_type "
At_chr5	Cufflinks	exon	38494521	38494968	.	+	.	gene_id "XLOC_075275"; transcript_id "Novel_000969"; exon_number "3"; gene_type "
At_chr5	Cufflinks	exon	38492969	38494440	.	+	.	gene_id "XLOC_075275"; transcript_id "Novel_000970"; exon_number "1"; gene_type "
At_chr5	Cufflinks	exon	38494521	38494963	.	+	.	gene_id "XLOC_075275"; transcript_id "Novel_000970"; exon_number "2"; gene_type "
At_chr5	Cufflinks	exon	38496716	38497076	.	+	.	gene_id "XLOC_075282"; transcript_id "Novel_000971"; exon_number "1"; gene_type "
At_chr5	Cufflinks	exon	38497247	38497457	.	+	.	gene_id "XLOC_075282"; transcript_id "Novel_000971"; exon_number "2"; gene_type "
At_chr5	Cufflinks	exon	38498067	38498332	.	+	.	gene_id "XLOC_075283"; transcript_id "Novel_000972"; exon_number "1"; gene_type "
At_chr5	Cufflinks	exon	38498436	38498675	.	+	.	gene_id "XLOC_075283"; transcript_id "Novel_000972"; exon_number "2"; gene_type "
At_chr5	Cufflinks	exon	38498805	38499271	.	+	.	gene_id "XLOC_075283"; transcript_id "Novel_000972"; exon_number "3"; gene_type "
At_chr5	Cufflinks	exon	38498366	38498675	.	+	.	gene_id "XLOC_075283"; transcript_id "Novel_000973"; exon_number "1"; gene_type "
At_chr5	Cufflinks	exon	38498805	38499256	.	+	.	gene_id "XLOC_075283"; transcript_id "Novel_000973"; exon_number "2"; gene_type "
At_chr5	Cufflinks	exon	38554881	38555066	.	+	.	gene_id "XLOC_075285"; transcript_id "Novel_000974"; exon_number "1"; gene_type "
At_chr5	Cufflinks	exon	38555179	38555211	.	+	.	gene_id "XLOC_075285"; transcript_id "Novel_000974"; exon_number "2"; gene_type "
At_chr5	Cufflinks	exon	38555320	38555420	.	+	.	gene_id "XLOC_075285"; transcript_id "Novel_000974"; exon_number "3"; gene_type "
At_chr5	Cufflinks	exon	38555505	38555620	.	+	.	gene_id "XLOC_075285"; transcript_id "Novel_000974"; exon_number "4"; gene_type "
At_chr5	Cufflinks	exon	38555713	38555812	.	+	.	gene_id "XLOC_075285"; transcript_id "Novel_000974"; exon_number "5"; gene_type "
At_chr5	Cufflinks	exon	38555894	38556101	.	+	.	gene_id "XLOC_075285"; transcript_id "Novel_000974"; exon_number "6"; gene_type "
At_chr5	Cufflinks	exon	38556188	38556291	.	+	.	gene_id "XLOC_075285"; transcript_id "Novel_000974"; exon_number "7"; gene_type "
At_chr5	Cufflinks	exon	38556381	38556523	.	+	.	gene_id "XLOC_075285"; transcript_id "Novel_000974"; exon_number "8"; gene_type "
At_chr5	Cufflinks	exon	38556618	38556701	.	+	.	gene_id "XLOC_075285"; transcript_id "Novel_000974"; exon_number "9"; gene_type "
At_chr5	Cufflinks	exon	38556795	38556884	.	+	.	gene_id "XLOC_075285"; transcript_id "Novel_000974"; exon_number "10"; gene_type
At_chr5	Cufflinks	exon	38556977	38557212	.	+	.	gene_id "XLOC_075285"; transcript_id "Novel_000974"; exon_number "11"; gene_type
At_chr5	Cufflinks	exon	39380908	39380931	.	+	.	gene_id "XLOC_075334"; transcript_id "Novel_000975"; exon_number "1"; gene_type "
At_chr5	Cufflinks	exon	39383121	39383177	.	+	.	gene_id "XLOC_075334"; transcript_id "Novel_000975"; exon_number "2"; gene_type "
At_chr5	Cufflinks	exon	39383280	39383465	.	+	.	gene_id "XLOC_075334"; transcript_id "Novel_000975"; exon_number "3"; gene_type "
At_chr5	Cufflinks	exon	43237272	43237384	.	+	.	gene_id "XLOC_075590"; transcript_id "Novel_000976"; exon_number "1"; gene_type "
At_chr5	Cufflinks	exon	43237464	43242004	.	+	.	gene_id "XLOC_075590"; transcript_id "Novel_000976"; exon_number "2"; gene_type "
At_chr5	Cufflinks	exon	43974300	43974455	.	+	.	gene_id "XLOC_075673"; transcript_id "Novel_000977"; exon_number "1"; gene_type "
At_chr5	Cufflinks	exon	43974539	43974899	.	+	.	gene_id "XLOC_075673"; transcript_id "Novel_000977"; exon_number "2"; gene_type "
At_chr5	Cufflinks	exon	43978974	43978997	.	+	.	gene_id "XLOC_075673"; transcript_id "Novel_000977"; exon_number "3"; gene_type "
At_chr5	Cufflinks	exon	43979082	43979125	.	+	.	gene_id "XLOC_075673"; transcript_id "Novel_000978"; exon_number "1"; gene_type "

At_chr5	Cufflinks	exon	43979399	43980473	.	+	.	gene_id "XLOC_075673"; transcript_id "Novel_000978"; exon_number "2"; gene_type "
At_chr5	Cufflinks	exon	44165402	44165583	.	+	.	gene_id "XLOC_075679"; transcript_id "Novel_000979"; exon_number "1"; gene_type "
At_chr5	Cufflinks	exon	44165660	44165725	.	+	.	gene_id "XLOC_075679"; transcript_id "Novel_000979"; exon_number "2"; gene_type "
At_chr5	Cufflinks	exon	46252876	46253165	.	+	.	gene_id "XLOC_075768"; transcript_id "Novel_000980"; exon_number "1"; gene_type "
At_chr5	Cufflinks	exon	46253315	46253345	.	+	.	gene_id "XLOC_075768"; transcript_id "Novel_000980"; exon_number "2"; gene_type "
At_chr5	Cufflinks	exon	47856586	47856669	.	+	.	gene_id "XLOC_075953"; transcript_id "Novel_000981"; exon_number "1"; gene_type "
At_chr5	Cufflinks	exon	47856867	47856937	.	+	.	gene_id "XLOC_075953"; transcript_id "Novel_000981"; exon_number "2"; gene_type "
At_chr5	Cufflinks	exon	47857243	47857329	.	+	.	gene_id "XLOC_075953"; transcript_id "Novel_000981"; exon_number "3"; gene_type "
At_chr5	Cufflinks	exon	47857907	47858583	.	+	.	gene_id "XLOC_075953"; transcript_id "Novel_000981"; exon_number "4"; gene_type "
At_chr5	Cufflinks	exon	50722114	50722624	.	+	.	gene_id "XLOC_076328"; transcript_id "Novel_000982"; exon_number "1"; gene_type "
At_chr5	Cufflinks	exon	50722719	50724362	.	+	.	gene_id "XLOC_076328"; transcript_id "Novel_000982"; exon_number "2"; gene_type "
At_chr5	Cufflinks	exon	50722114	50722624	.	+	.	gene_id "XLOC_076328"; transcript_id "Novel_000983"; exon_number "1"; gene_type "
At_chr5	Cufflinks	exon	50722719	50723109	.	+	.	gene_id "XLOC_076328"; transcript_id "Novel_000983"; exon_number "2"; gene_type "
At_chr5	Cufflinks	exon	50723199	50724382	.	+	.	gene_id "XLOC_076328"; transcript_id "Novel_000983"; exon_number "3"; gene_type "
At_chr5	Cufflinks	exon	50722114	50722624	.	+	.	gene_id "XLOC_076328"; transcript_id "Novel_000984"; exon_number "1"; gene_type "
At_chr5	Cufflinks	exon	50722719	50723109	.	+	.	gene_id "XLOC_076328"; transcript_id "Novel_000984"; exon_number "2"; gene_type "
At_chr5	Cufflinks	exon	50723199	50723239	.	+	.	gene_id "XLOC_076328"; transcript_id "Novel_000984"; exon_number "3"; gene_type "
At_chr5	Cufflinks	exon	50724437	50724478	.	+	.	gene_id "XLOC_076328"; transcript_id "Novel_000984"; exon_number "4"; gene_type "
At_chr5	Cufflinks	exon	50724549	50724670	.	+	.	gene_id "XLOC_076328"; transcript_id "Novel_000984"; exon_number "5"; gene_type "
At_chr5	Cufflinks	exon	50724747	50724855	.	+	.	gene_id "XLOC_076328"; transcript_id "Novel_000984"; exon_number "6"; gene_type "
At_chr5	Cufflinks	exon	50725100	50725429	.	+	.	gene_id "XLOC_076328"; transcript_id "Novel_000984"; exon_number "7"; gene_type "
At_chr5	Cufflinks	exon	50722124	50722624	.	+	.	gene_id "XLOC_076328"; transcript_id "Novel_000985"; exon_number "1"; gene_type "
At_chr5	Cufflinks	exon	50722719	50723109	.	+	.	gene_id "XLOC_076328"; transcript_id "Novel_000985"; exon_number "2"; gene_type "
At_chr5	Cufflinks	exon	50723199	50723239	.	+	.	gene_id "XLOC_076328"; transcript_id "Novel_000985"; exon_number "3"; gene_type "
At_chr5	Cufflinks	exon	50724437	50724670	.	+	.	gene_id "XLOC_076328"; transcript_id "Novel_000985"; exon_number "4"; gene_type "
At_chr5	Cufflinks	exon	50724747	50724855	.	+	.	gene_id "XLOC_076328"; transcript_id "Novel_000985"; exon_number "5"; gene_type "
At_chr5	Cufflinks	exon	50725100	50725631	.	+	.	gene_id "XLOC_076328"; transcript_id "Novel_000985"; exon_number "6"; gene_type "
At_chr5	Cufflinks	exon	50722140	50722624	.	+	.	gene_id "XLOC_076328"; transcript_id "Novel_000986"; exon_number "1"; gene_type "
At_chr5	Cufflinks	exon	50722719	50723109	.	+	.	gene_id "XLOC_076328"; transcript_id "Novel_000986"; exon_number "2"; gene_type "
At_chr5	Cufflinks	exon	50723199	50723239	.	+	.	gene_id "XLOC_076328"; transcript_id "Novel_000986"; exon_number "3"; gene_type "
At_chr5	Cufflinks	exon	50724437	50724478	.	+	.	gene_id "XLOC_076328"; transcript_id "Novel_000986"; exon_number "4"; gene_type "
At_chr5	Cufflinks	exon	50724549	50724855	.	+	.	gene_id "XLOC_076328"; transcript_id "Novel_000986"; exon_number "5"; gene_type "
At_chr5	Cufflinks	exon	50725100	50725626	.	+	.	gene_id "XLOC_076328"; transcript_id "Novel_000986"; exon_number "6"; gene_type "
At_chr5	Cufflinks	exon	51489978	51490210	.	+	.	gene_id "XLOC_076408"; transcript_id "Novel_000987"; exon_number "1"; gene_type "
At_chr5	Cufflinks	exon	51490379	51490547	.	+	.	gene_id "XLOC_076408"; transcript_id "Novel_000987"; exon_number "2"; gene_type "

At_chr5	Cufflinks	exon	51491284	51491372	.	+	.	gene_id "XLOC_076408"; transcript_id "Novel_000987"; exon_number "3"; gene_type "
At_chr5	Cufflinks	exon	51492583	51493178	.	+	.	gene_id "XLOC_076408"; transcript_id "Novel_000987"; exon_number "4"; gene_type "
At_chr5	Cufflinks	exon	51561964	51562112	.	+	.	gene_id "XLOC_076433"; transcript_id "Novel_000988"; exon_number "1"; gene_type "
At_chr5	Cufflinks	exon	51562212	51562321	.	+	.	gene_id "XLOC_076433"; transcript_id "Novel_000988"; exon_number "2"; gene_type "
At_chr5	Cufflinks	exon	51562503	51562993	.	+	.	gene_id "XLOC_076433"; transcript_id "Novel_000988"; exon_number "3"; gene_type "
At_chr5	Cufflinks	exon	51610295	51610554	.	+	.	gene_id "XLOC_076448"; transcript_id "Novel_000989"; exon_number "1"; gene_type "
At_chr5	Cufflinks	exon	51611477	51611570	.	+	.	gene_id "XLOC_076448"; transcript_id "Novel_000989"; exon_number "2"; gene_type "
At_chr5	Cufflinks	exon	51612254	51612323	.	+	.	gene_id "XLOC_076448"; transcript_id "Novel_000989"; exon_number "3"; gene_type "
At_chr5	Cufflinks	exon	51612448	51612822	.	+	.	gene_id "XLOC_076448"; transcript_id "Novel_000989"; exon_number "4"; gene_type "
At_chr5	Cufflinks	exon	51644337	51644411	.	+	.	gene_id "XLOC_076455"; transcript_id "Novel_000990"; exon_number "1"; gene_type "
At_chr5	Cufflinks	exon	51644597	51644736	.	+	.	gene_id "XLOC_076455"; transcript_id "Novel_000990"; exon_number "2"; gene_type "
At_chr5	Cufflinks	exon	51645662	51645787	.	+	.	gene_id "XLOC_076455"; transcript_id "Novel_000990"; exon_number "3"; gene_type "
At_chr5	Cufflinks	exon	51645915	51647671	.	+	.	gene_id "XLOC_076455"; transcript_id "Novel_000990"; exon_number "4"; gene_type "
At_chr5	Cufflinks	exon	51673044	51673265	.	+	.	gene_id "XLOC_076461"; transcript_id "Novel_000991"; exon_number "1"; gene_type "
At_chr5	Cufflinks	exon	51673383	51673404	.	+	.	gene_id "XLOC_076461"; transcript_id "Novel_000991"; exon_number "2"; gene_type "
At_chr5	Cufflinks	exon	51673503	51673608	.	+	.	gene_id "XLOC_076461"; transcript_id "Novel_000991"; exon_number "3"; gene_type "
At_chr5	Cufflinks	exon	51675122	51675229	.	+	.	gene_id "XLOC_076461"; transcript_id "Novel_000991"; exon_number "4"; gene_type "
At_chr5	Cufflinks	exon	51675641	51676104	.	+	.	gene_id "XLOC_076461"; transcript_id "Novel_000991"; exon_number "5"; gene_type "
At_chr5	Cufflinks	exon	51673045	51673404	.	+	.	gene_id "XLOC_076461"; transcript_id "Novel_000992"; exon_number "1"; gene_type "
At_chr5	Cufflinks	exon	51673503	51673658	.	+	.	gene_id "XLOC_076461"; transcript_id "Novel_000992"; exon_number "2"; gene_type "
At_chr5	Cufflinks	exon	1246572	1247677	.	-	.	gene_id "XLOC_076858"; transcript_id "Novel_000993"; exon_number "1"; gene_type "
At_chr5	Cufflinks	exon	1248340	1248411	.	-	.	gene_id "XLOC_076858"; transcript_id "Novel_000993"; exon_number "2"; gene_type "
At_chr5	Cufflinks	exon	1248500	1248642	.	-	.	gene_id "XLOC_076858"; transcript_id "Novel_000993"; exon_number "3"; gene_type "
At_chr5	Cufflinks	exon	1246589	1246638	.	-	.	gene_id "XLOC_076858"; transcript_id "Novel_000994"; exon_number "1"; gene_type "
At_chr5	Cufflinks	exon	1247573	1247677	.	-	.	gene_id "XLOC_076858"; transcript_id "Novel_000994"; exon_number "2"; gene_type "
At_chr5	Cufflinks	exon	1248353	1248411	.	-	.	gene_id "XLOC_076858"; transcript_id "Novel_000994"; exon_number "3"; gene_type "
At_chr5	Cufflinks	exon	1248500	1248714	.	-	.	gene_id "XLOC_076858"; transcript_id "Novel_000994"; exon_number "4"; gene_type "
At_chr5	Cufflinks	exon	1246589	1246638	.	-	.	gene_id "XLOC_076858"; transcript_id "Novel_000995"; exon_number "1"; gene_type "
At_chr5	Cufflinks	exon	1247573	1247677	.	-	.	gene_id "XLOC_076858"; transcript_id "Novel_000995"; exon_number "2"; gene_type "
At_chr5	Cufflinks	exon	1248340	1248411	.	-	.	gene_id "XLOC_076858"; transcript_id "Novel_000995"; exon_number "3"; gene_type "
At_chr5	Cufflinks	exon	1248500	1248714	.	-	.	gene_id "XLOC_076858"; transcript_id "Novel_000995"; exon_number "4"; gene_type "
At_chr5	Cufflinks	exon	1754121	1755148	.	-	.	gene_id "XLOC_076938"; transcript_id "Novel_000996"; exon_number "1"; gene_type "
At_chr5	Cufflinks	exon	1755969	1756238	.	-	.	gene_id "XLOC_076938"; transcript_id "Novel_000996"; exon_number "2"; gene_type "
At_chr5	Cufflinks	exon	1756321	1756847	.	-	.	gene_id "XLOC_076938"; transcript_id "Novel_000996"; exon_number "3"; gene_type "
At_chr5	Cufflinks	exon	2133774	2134486	.	-	.	gene_id "XLOC_076994"; transcript_id "Novel_000997"; exon_number "1"; gene_type "

At_chr5	Cufflinks	exon	2134575	2134758	.	-	.	gene_id "XLOC_076994"; transcript_id "Novel_000997"; exon_number "2"; gene_type "
At_chr5	Cufflinks	exon	2135138	2135470	.	-	.	gene_id "XLOC_076994"; transcript_id "Novel_000997"; exon_number "3"; gene_type "
At_chr5	Cufflinks	exon	2135863	2136000	.	-	.	gene_id "XLOC_076994"; transcript_id "Novel_000997"; exon_number "4"; gene_type "
At_chr5	Cufflinks	exon	2136755	2136884	.	-	.	gene_id "XLOC_076994"; transcript_id "Novel_000997"; exon_number "5"; gene_type "
At_chr5	Cufflinks	exon	2137648	2137873	.	-	.	gene_id "XLOC_076994"; transcript_id "Novel_000997"; exon_number "6"; gene_type "
At_chr5	Cufflinks	exon	2137947	2138096	.	-	.	gene_id "XLOC_076994"; transcript_id "Novel_000997"; exon_number "7"; gene_type "
At_chr5	Cufflinks	exon	2133859	2134486	.	-	.	gene_id "XLOC_076994"; transcript_id "Novel_000998"; exon_number "1"; gene_type "
At_chr5	Cufflinks	exon	2134575	2134758	.	-	.	gene_id "XLOC_076994"; transcript_id "Novel_000998"; exon_number "2"; gene_type "
At_chr5	Cufflinks	exon	2135138	2135470	.	-	.	gene_id "XLOC_076994"; transcript_id "Novel_000998"; exon_number "3"; gene_type "
At_chr5	Cufflinks	exon	2135863	2136000	.	-	.	gene_id "XLOC_076994"; transcript_id "Novel_000998"; exon_number "4"; gene_type "
At_chr5	Cufflinks	exon	2136755	2136884	.	-	.	gene_id "XLOC_076994"; transcript_id "Novel_000998"; exon_number "5"; gene_type "
At_chr5	Cufflinks	exon	2137648	2137868	.	-	.	gene_id "XLOC_076994"; transcript_id "Novel_000998"; exon_number "6"; gene_type "
At_chr5	Cufflinks	exon	2137947	2138089	.	-	.	gene_id "XLOC_076994"; transcript_id "Novel_000998"; exon_number "7"; gene_type "
At_chr5	Cufflinks	exon	2133932	2134486	.	-	.	gene_id "XLOC_076994"; transcript_id "Novel_000999"; exon_number "1"; gene_type "
At_chr5	Cufflinks	exon	2134575	2134758	.	-	.	gene_id "XLOC_076994"; transcript_id "Novel_000999"; exon_number "2"; gene_type "
At_chr5	Cufflinks	exon	2135138	2136000	.	-	.	gene_id "XLOC_076994"; transcript_id "Novel_000999"; exon_number "3"; gene_type "
At_chr5	Cufflinks	exon	2136755	2136884	.	-	.	gene_id "XLOC_076994"; transcript_id "Novel_000999"; exon_number "4"; gene_type "
At_chr5	Cufflinks	exon	2137648	2137865	.	-	.	gene_id "XLOC_076994"; transcript_id "Novel_000999"; exon_number "5"; gene_type "
At_chr5	Cufflinks	exon	2137947	2138025	.	-	.	gene_id "XLOC_076994"; transcript_id "Novel_000999"; exon_number "6"; gene_type "
At_chr5	Cufflinks	exon	2134021	2134486	.	-	.	gene_id "XLOC_076994"; transcript_id "Novel_001000"; exon_number "1"; gene_type "
At_chr5	Cufflinks	exon	2134575	2134758	.	-	.	gene_id "XLOC_076994"; transcript_id "Novel_001000"; exon_number "2"; gene_type "
At_chr5	Cufflinks	exon	2135138	2135470	.	-	.	gene_id "XLOC_076994"; transcript_id "Novel_001000"; exon_number "3"; gene_type "
At_chr5	Cufflinks	exon	2135863	2136000	.	-	.	gene_id "XLOC_076994"; transcript_id "Novel_001000"; exon_number "4"; gene_type "
At_chr5	Cufflinks	exon	2136755	2136884	.	-	.	gene_id "XLOC_076994"; transcript_id "Novel_001000"; exon_number "5"; gene_type "
At_chr5	Cufflinks	exon	2137648	2137865	.	-	.	gene_id "XLOC_076994"; transcript_id "Novel_001000"; exon_number "6"; gene_type "
At_chr5	Cufflinks	exon	2137947	2138090	.	-	.	gene_id "XLOC_076994"; transcript_id "Novel_001000"; exon_number "7"; gene_type "
At_chr5	Cufflinks	exon	2356898	2357366	.	-	.	gene_id "XLOC_077027"; transcript_id "Novel_001001"; exon_number "1"; gene_type "
At_chr5	Cufflinks	exon	2357459	2357593	.	-	.	gene_id "XLOC_077027"; transcript_id "Novel_001001"; exon_number "2"; gene_type "
At_chr5	Cufflinks	exon	2358351	2358491	.	-	.	gene_id "XLOC_077027"; transcript_id "Novel_001001"; exon_number "3"; gene_type "
At_chr5	Cufflinks	exon	2358592	2358978	.	-	.	gene_id "XLOC_077027"; transcript_id "Novel_001001"; exon_number "4"; gene_type "
At_chr5	Cufflinks	exon	2356981	2358491	.	-	.	gene_id "XLOC_077027"; transcript_id "Novel_001002"; exon_number "1"; gene_type "
At_chr5	Cufflinks	exon	2358592	2359051	.	-	.	gene_id "XLOC_077027"; transcript_id "Novel_001002"; exon_number "2"; gene_type "
At_chr5	Cufflinks	exon	2444892	2446967	.	-	.	gene_id "XLOC_077038"; transcript_id "Novel_001003"; exon_number "1"; gene_type "
At_chr5	Cufflinks	exon	2447306	2447408	.	-	.	gene_id "XLOC_077038"; transcript_id "Novel_001003"; exon_number "2"; gene_type "
At_chr5	Cufflinks	exon	3646722	3647053	.	-	.	gene_id "XLOC_077169"; transcript_id "Novel_001004"; exon_number "1"; gene_type "

At_chr5	Cufflinks	exon	3648166	3648309	.	-	.	gene_id "XLOC_077169"; transcript_id "Novel_001004"; exon_number "2"; gene_type "
At_chr5	Cufflinks	exon	3648714	3648784	.	-	.	gene_id "XLOC_077169"; transcript_id "Novel_001004"; exon_number "3"; gene_type "
At_chr5	Cufflinks	exon	3649184	3649287	.	-	.	gene_id "XLOC_077169"; transcript_id "Novel_001004"; exon_number "4"; gene_type "
At_chr5	Cufflinks	exon	3650121	3650337	.	-	.	gene_id "XLOC_077169"; transcript_id "Novel_001004"; exon_number "5"; gene_type "
At_chr5	Cufflinks	exon	3650634	3650972	.	-	.	gene_id "XLOC_077169"; transcript_id "Novel_001004"; exon_number "6"; gene_type "
At_chr5	Cufflinks	exon	3646728	3647053	.	-	.	gene_id "XLOC_077169"; transcript_id "Novel_001005"; exon_number "1"; gene_type "
At_chr5	Cufflinks	exon	3648166	3648309	.	-	.	gene_id "XLOC_077169"; transcript_id "Novel_001005"; exon_number "2"; gene_type "
At_chr5	Cufflinks	exon	3648714	3648784	.	-	.	gene_id "XLOC_077169"; transcript_id "Novel_001005"; exon_number "3"; gene_type "
At_chr5	Cufflinks	exon	3649188	3649287	.	-	.	gene_id "XLOC_077169"; transcript_id "Novel_001005"; exon_number "4"; gene_type "
At_chr5	Cufflinks	exon	3650121	3650151	.	-	.	gene_id "XLOC_077169"; transcript_id "Novel_001005"; exon_number "5"; gene_type "
At_chr5	Cufflinks	exon	3650240	3650337	.	-	.	gene_id "XLOC_077169"; transcript_id "Novel_001005"; exon_number "6"; gene_type "
At_chr5	Cufflinks	exon	3650634	3650991	.	-	.	gene_id "XLOC_077169"; transcript_id "Novel_001005"; exon_number "7"; gene_type "
At_chr5	Cufflinks	exon	3646731	3647053	.	-	.	gene_id "XLOC_077169"; transcript_id "Novel_001006"; exon_number "1"; gene_type "
At_chr5	Cufflinks	exon	3648166	3648309	.	-	.	gene_id "XLOC_077169"; transcript_id "Novel_001006"; exon_number "2"; gene_type "
At_chr5	Cufflinks	exon	3648714	3648784	.	-	.	gene_id "XLOC_077169"; transcript_id "Novel_001006"; exon_number "3"; gene_type "
At_chr5	Cufflinks	exon	3649184	3649287	.	-	.	gene_id "XLOC_077169"; transcript_id "Novel_001006"; exon_number "4"; gene_type "
At_chr5	Cufflinks	exon	3650121	3650151	.	-	.	gene_id "XLOC_077169"; transcript_id "Novel_001006"; exon_number "5"; gene_type "
At_chr5	Cufflinks	exon	3650240	3650337	.	-	.	gene_id "XLOC_077169"; transcript_id "Novel_001006"; exon_number "6"; gene_type "
At_chr5	Cufflinks	exon	3650634	3650945	.	-	.	gene_id "XLOC_077169"; transcript_id "Novel_001006"; exon_number "7"; gene_type "
At_chr5	Cufflinks	exon	3648960	3649287	.	-	.	gene_id "XLOC_077169"; transcript_id "Novel_001007"; exon_number "1"; gene_type "
At_chr5	Cufflinks	exon	3650121	3650151	.	-	.	gene_id "XLOC_077169"; transcript_id "Novel_001007"; exon_number "2"; gene_type "
At_chr5	Cufflinks	exon	3650240	3650337	.	-	.	gene_id "XLOC_077169"; transcript_id "Novel_001007"; exon_number "3"; gene_type "
At_chr5	Cufflinks	exon	3650634	3650983	.	-	.	gene_id "XLOC_077169"; transcript_id "Novel_001007"; exon_number "4"; gene_type "
At_chr5	Cufflinks	exon	3890273	3891254	.	-	.	gene_id "XLOC_077194"; transcript_id "Novel_001008"; exon_number "1"; gene_type "
At_chr5	Cufflinks	exon	3892841	3892948	.	-	.	gene_id "XLOC_077194"; transcript_id "Novel_001008"; exon_number "2"; gene_type "
At_chr5	Cufflinks	exon	4260325	4260591	.	-	.	gene_id "XLOC_077235"; transcript_id "Novel_001009"; exon_number "1"; gene_type "
At_chr5	Cufflinks	exon	4261904	4262176	.	-	.	gene_id "XLOC_077235"; transcript_id "Novel_001009"; exon_number "2"; gene_type "
At_chr5	Cufflinks	exon	5004490	5004554	.	-	.	gene_id "XLOC_077321"; transcript_id "Novel_001010"; exon_number "1"; gene_type "
At_chr5	Cufflinks	exon	5004632	5004784	.	-	.	gene_id "XLOC_077321"; transcript_id "Novel_001010"; exon_number "2"; gene_type "
At_chr5	Cufflinks	exon	9922483	9923313	.	-	.	gene_id "XLOC_077756"; transcript_id "Novel_001011"; exon_number "1"; gene_type "
At_chr5	Cufflinks	exon	9924484	9924528	.	-	.	gene_id "XLOC_077756"; transcript_id "Novel_001011"; exon_number "2"; gene_type "
At_chr5	Cufflinks	exon	9924636	9924692	.	-	.	gene_id "XLOC_077756"; transcript_id "Novel_001011"; exon_number "3"; gene_type "
At_chr5	Cufflinks	exon	9924779	9924839	.	-	.	gene_id "XLOC_077756"; transcript_id "Novel_001011"; exon_number "4"; gene_type "
At_chr5	Cufflinks	exon	9925406	9925467	.	-	.	gene_id "XLOC_077756"; transcript_id "Novel_001011"; exon_number "5"; gene_type "
At_chr5	Cufflinks	exon	9925556	9925666	.	-	.	gene_id "XLOC_077756"; transcript_id "Novel_001011"; exon_number "6"; gene_type "

At_chr5	Cufflinks	exon	9925821	9925892	.	-	.	gene_id "XLOC_077756"; transcript_id "Novel_001011"; exon_number "7"; gene_type "
At_chr5	Cufflinks	exon	9926063	9926113	.	-	.	gene_id "XLOC_077756"; transcript_id "Novel_001011"; exon_number "8"; gene_type "
At_chr5	Cufflinks	exon	9926219	9926341	.	-	.	gene_id "XLOC_077756"; transcript_id "Novel_001011"; exon_number "9"; gene_type "
At_chr5	Cufflinks	exon	9926983	9927239	.	-	.	gene_id "XLOC_077756"; transcript_id "Novel_001011"; exon_number "10"; gene_type
At_chr5	Cufflinks	exon	12261926	12262618	.	-	.	gene_id "XLOC_077901"; transcript_id "Novel_001012"; exon_number "1"; gene_type "
At_chr5	Cufflinks	exon	12263054	12263305	.	-	.	gene_id "XLOC_077901"; transcript_id "Novel_001012"; exon_number "2"; gene_type "
At_chr5	Cufflinks	exon	13515462	13515555	.	-	.	gene_id "XLOC_078001"; transcript_id "Novel_001013"; exon_number "1"; gene_type "
At_chr5	Cufflinks	exon	13516108	13516254	.	-	.	gene_id "XLOC_078001"; transcript_id "Novel_001013"; exon_number "2"; gene_type "
At_chr5	Cufflinks	exon	13517278	13517970	.	-	.	gene_id "XLOC_078001"; transcript_id "Novel_001013"; exon_number "3"; gene_type "
At_chr5	Cufflinks	exon	18969608	18969826	.	-	.	gene_id "XLOC_078238"; transcript_id "Novel_001014"; exon_number "1"; gene_type "
At_chr5	Cufflinks	exon	32489926	32490053	.	-	.	gene_id "XLOC_078713"; transcript_id "Novel_001015"; exon_number "1"; gene_type "
At_chr5	Cufflinks	exon	32490292	32490448	.	-	.	gene_id "XLOC_078713"; transcript_id "Novel_001015"; exon_number "2"; gene_type "
At_chr5	Cufflinks	exon	32491077	32491548	.	-	.	gene_id "XLOC_078713"; transcript_id "Novel_001015"; exon_number "3"; gene_type "
At_chr5	Cufflinks	exon	32489926	32490053	.	-	.	gene_id "XLOC_078713"; transcript_id "Novel_001016"; exon_number "1"; gene_type "
At_chr5	Cufflinks	exon	32490292	32490448	.	-	.	gene_id "XLOC_078713"; transcript_id "Novel_001016"; exon_number "2"; gene_type "
At_chr5	Cufflinks	exon	32491077	32491248	.	-	.	gene_id "XLOC_078713"; transcript_id "Novel_001016"; exon_number "3"; gene_type "
At_chr5	Cufflinks	exon	32492573	32493138	.	-	.	gene_id "XLOC_078713"; transcript_id "Novel_001016"; exon_number "4"; gene_type "
At_chr5	Cufflinks	exon	37341815	37342001	.	-	.	gene_id "XLOC_078906"; transcript_id "Novel_001017"; exon_number "1"; gene_type "
At_chr5	Cufflinks	exon	37342152	37343960	.	-	.	gene_id "XLOC_078906"; transcript_id "Novel_001017"; exon_number "2"; gene_type "
At_chr5	Cufflinks	exon	37345599	37345685	.	-	.	gene_id "XLOC_078907"; transcript_id "Novel_001018"; exon_number "1"; gene_type "
At_chr5	Cufflinks	exon	37346868	37347931	.	-	.	gene_id "XLOC_078907"; transcript_id "Novel_001018"; exon_number "2"; gene_type "
At_chr5	Cufflinks	exon	37731515	37732449	.	-	.	gene_id "XLOC_078919"; transcript_id "Novel_001019"; exon_number "1"; gene_type "
At_chr5	Cufflinks	exon	37733148	37733532	.	-	.	gene_id "XLOC_078919"; transcript_id "Novel_001019"; exon_number "2"; gene_type "
At_chr5	Cufflinks	exon	37733801	37734417	.	-	.	gene_id "XLOC_078919"; transcript_id "Novel_001019"; exon_number "3"; gene_type "
At_chr5	Cufflinks	exon	37731792	37732449	.	-	.	gene_id "XLOC_078919"; transcript_id "Novel_001020"; exon_number "1"; gene_type "
At_chr5	Cufflinks	exon	37733148	37734415	.	-	.	gene_id "XLOC_078919"; transcript_id "Novel_001020"; exon_number "2"; gene_type "
At_chr5	Cufflinks	exon	37731888	37733532	.	-	.	gene_id "XLOC_078919"; transcript_id "Novel_001021"; exon_number "1"; gene_type "
At_chr5	Cufflinks	exon	37733801	37734413	.	-	.	gene_id "XLOC_078919"; transcript_id "Novel_001021"; exon_number "2"; gene_type "
At_chr5	Cufflinks	exon	37734482	37735014	.	-	.	gene_id "XLOC_078920"; transcript_id "Novel_001022"; exon_number "1"; gene_type "
At_chr5	Cufflinks	exon	37735138	37735178	.	-	.	gene_id "XLOC_078920"; transcript_id "Novel_001022"; exon_number "2"; gene_type "
At_chr5	Cufflinks	exon	37736625	37736727	.	-	.	gene_id "XLOC_078920"; transcript_id "Novel_001022"; exon_number "3"; gene_type "
At_chr5	Cufflinks	exon	37734495	37734634	.	-	.	gene_id "XLOC_078920"; transcript_id "Novel_001023"; exon_number "1"; gene_type "
At_chr5	Cufflinks	exon	37734734	37735014	.	-	.	gene_id "XLOC_078920"; transcript_id "Novel_001023"; exon_number "2"; gene_type "
At_chr5	Cufflinks	exon	37735138	37736731	.	-	.	gene_id "XLOC_078920"; transcript_id "Novel_001023"; exon_number "3"; gene_type "
At_chr5	Cufflinks	exon	37734498	37734634	.	-	.	gene_id "XLOC_078920"; transcript_id "Novel_001024"; exon_number "1"; gene_type "

At_chr5	Cufflinks	exon	37734734	37736717	.	-	.	gene_id "XLOC_078920"; transcript_id "Novel_001024"; exon_number "2"; gene_type "
At_chr5	Cufflinks	exon	37785886	37786802	.	-	.	gene_id "XLOC_078925"; transcript_id "Novel_001025"; exon_number "1"; gene_type "
At_chr5	Cufflinks	exon	37797419	37797496	.	-	.	gene_id "XLOC_078925"; transcript_id "Novel_001025"; exon_number "2"; gene_type "
At_chr5	Cufflinks	exon	37797630	37798291	.	-	.	gene_id "XLOC_078925"; transcript_id "Novel_001025"; exon_number "3"; gene_type "
At_chr5	Cufflinks	exon	38040333	38040912	.	-	.	gene_id "XLOC_078946"; transcript_id "Novel_001026"; exon_number "1"; gene_type "
At_chr5	Cufflinks	exon	38040983	38041042	.	-	.	gene_id "XLOC_078946"; transcript_id "Novel_001026"; exon_number "2"; gene_type "
At_chr5	Cufflinks	exon	38041131	38041251	.	-	.	gene_id "XLOC_078946"; transcript_id "Novel_001026"; exon_number "3"; gene_type "
At_chr5	Cufflinks	exon	38041449	38041702	.	-	.	gene_id "XLOC_078946"; transcript_id "Novel_001026"; exon_number "4"; gene_type "
At_chr5	Cufflinks	exon	38106670	38108344	.	-	.	gene_id "XLOC_078951"; transcript_id "Novel_001027"; exon_number "1"; gene_type "
At_chr5	Cufflinks	exon	38108454	38108747	.	-	.	gene_id "XLOC_078951"; transcript_id "Novel_001027"; exon_number "2"; gene_type "
At_chr5	Cufflinks	exon	38108857	38108994	.	-	.	gene_id "XLOC_078951"; transcript_id "Novel_001027"; exon_number "3"; gene_type "
At_chr5	Cufflinks	exon	38109071	38109111	.	-	.	gene_id "XLOC_078951"; transcript_id "Novel_001027"; exon_number "4"; gene_type "
At_chr5	Cufflinks	exon	38107005	38108344	.	-	.	gene_id "XLOC_078951"; transcript_id "Novel_001028"; exon_number "1"; gene_type "
At_chr5	Cufflinks	exon	38108454	38108747	.	-	.	gene_id "XLOC_078951"; transcript_id "Novel_001028"; exon_number "2"; gene_type "
At_chr5	Cufflinks	exon	38108857	38108918	.	-	.	gene_id "XLOC_078951"; transcript_id "Novel_001028"; exon_number "3"; gene_type "
At_chr5	Cufflinks	exon	38109071	38109162	.	-	.	gene_id "XLOC_078951"; transcript_id "Novel_001028"; exon_number "4"; gene_type "
At_chr5	Cufflinks	exon	38107005	38108344	.	-	.	gene_id "XLOC_078951"; transcript_id "Novel_001029"; exon_number "1"; gene_type "
At_chr5	Cufflinks	exon	38108454	38108747	.	-	.	gene_id "XLOC_078951"; transcript_id "Novel_001029"; exon_number "2"; gene_type "
At_chr5	Cufflinks	exon	38108857	38108921	.	-	.	gene_id "XLOC_078951"; transcript_id "Novel_001029"; exon_number "3"; gene_type "
At_chr5	Cufflinks	exon	38109071	38109165	.	-	.	gene_id "XLOC_078951"; transcript_id "Novel_001029"; exon_number "4"; gene_type "
At_chr5	Cufflinks	exon	38107576	38108344	.	-	.	gene_id "XLOC_078951"; transcript_id "Novel_001030"; exon_number "1"; gene_type "
At_chr5	Cufflinks	exon	38108454	38108747	.	-	.	gene_id "XLOC_078951"; transcript_id "Novel_001030"; exon_number "2"; gene_type "
At_chr5	Cufflinks	exon	38108857	38108981	.	-	.	gene_id "XLOC_078951"; transcript_id "Novel_001030"; exon_number "3"; gene_type "
At_chr5	Cufflinks	exon	38109071	38109176	.	-	.	gene_id "XLOC_078951"; transcript_id "Novel_001030"; exon_number "4"; gene_type "
At_chr5	Cufflinks	exon	38203232	38203653	.	-	.	gene_id "XLOC_078956"; transcript_id "Novel_001031"; exon_number "1"; gene_type "
At_chr5	Cufflinks	exon	38203804	38204023	.	-	.	gene_id "XLOC_078956"; transcript_id "Novel_001031"; exon_number "2"; gene_type "
At_chr5	Cufflinks	exon	38358453	38359147	.	-	.	gene_id "XLOC_078977"; transcript_id "Novel_001032"; exon_number "1"; gene_type "
At_chr5	Cufflinks	exon	38360929	38361441	.	-	.	gene_id "XLOC_078977"; transcript_id "Novel_001032"; exon_number "2"; gene_type "
At_chr5	Cufflinks	exon	38361517	38362609	.	-	.	gene_id "XLOC_078977"; transcript_id "Novel_001033"; exon_number "1"; gene_type "
At_chr5	Cufflinks	exon	38362794	38363023	.	-	.	gene_id "XLOC_078977"; transcript_id "Novel_001033"; exon_number "2"; gene_type "
At_chr5	Cufflinks	exon	38474345	38475009	.	-	.	gene_id "XLOC_078988"; transcript_id "Novel_001034"; exon_number "1"; gene_type "
At_chr5	Cufflinks	exon	38475548	38475780	.	-	.	gene_id "XLOC_078988"; transcript_id "Novel_001034"; exon_number "2"; gene_type "
At_chr5	Cufflinks	exon	38476312	38476382	.	-	.	gene_id "XLOC_078988"; transcript_id "Novel_001034"; exon_number "3"; gene_type "
At_chr5	Cufflinks	exon	38477033	38477083	.	-	.	gene_id "XLOC_078988"; transcript_id "Novel_001034"; exon_number "4"; gene_type "
At_chr5	Cufflinks	exon	38478211	38478270	.	-	.	gene_id "XLOC_078988"; transcript_id "Novel_001034"; exon_number "5"; gene_type "

At_chr5	Cufflinks	exon	38478970	38479079	.	-	.	gene_id "XLOC_078988"; transcript_id "Novel_001034"; exon_number "6"; gene_type "
At_chr5	Cufflinks	exon	38479880	38480244	.	-	.	gene_id "XLOC_078988"; transcript_id "Novel_001034"; exon_number "7"; gene_type "
At_chr5	Cufflinks	exon	38475382	38476382	.	-	.	gene_id "XLOC_078988"; transcript_id "Novel_001035"; exon_number "1"; gene_type "
At_chr5	Cufflinks	exon	38477033	38477083	.	-	.	gene_id "XLOC_078988"; transcript_id "Novel_001035"; exon_number "2"; gene_type "
At_chr5	Cufflinks	exon	38478211	38478270	.	-	.	gene_id "XLOC_078988"; transcript_id "Novel_001035"; exon_number "3"; gene_type "
At_chr5	Cufflinks	exon	38478970	38479079	.	-	.	gene_id "XLOC_078988"; transcript_id "Novel_001035"; exon_number "4"; gene_type "
At_chr5	Cufflinks	exon	38479880	38480247	.	-	.	gene_id "XLOC_078988"; transcript_id "Novel_001035"; exon_number "5"; gene_type "
At_chr5	Cufflinks	exon	38477615	38478270	.	-	.	gene_id "XLOC_078988"; transcript_id "Novel_001036"; exon_number "1"; gene_type "
At_chr5	Cufflinks	exon	38478970	38479079	.	-	.	gene_id "XLOC_078988"; transcript_id "Novel_001036"; exon_number "2"; gene_type "
At_chr5	Cufflinks	exon	38479880	38480247	.	-	.	gene_id "XLOC_078988"; transcript_id "Novel_001036"; exon_number "3"; gene_type "
At_chr5	Cufflinks	exon	38501955	38502174	.	-	.	gene_id "XLOC_078993"; transcript_id "Novel_001037"; exon_number "1"; gene_type "
At_chr5	Cufflinks	exon	38502260	38502348	.	-	.	gene_id "XLOC_078993"; transcript_id "Novel_001037"; exon_number "2"; gene_type "
At_chr5	Cufflinks	exon	38502795	38502828	.	-	.	gene_id "XLOC_078993"; transcript_id "Novel_001037"; exon_number "3"; gene_type "
At_chr5	Cufflinks	exon	38691204	38692813	.	-	.	gene_id "XLOC_078999"; transcript_id "Novel_001038"; exon_number "1"; gene_type "
At_chr5	Cufflinks	exon	38693340	38693437	.	-	.	gene_id "XLOC_078999"; transcript_id "Novel_001038"; exon_number "2"; gene_type "
At_chr5	Cufflinks	exon	38693794	38693881	.	-	.	gene_id "XLOC_078999"; transcript_id "Novel_001038"; exon_number "3"; gene_type "
At_chr5	Cufflinks	exon	38693969	38694345	.	-	.	gene_id "XLOC_078999"; transcript_id "Novel_001038"; exon_number "4"; gene_type "
At_chr5	Cufflinks	exon	40803740	40804570	.	-	.	gene_id "XLOC_079134"; transcript_id "Novel_001039"; exon_number "1"; gene_type "
At_chr5	Cufflinks	exon	44547199	44548647	.	-	.	gene_id "XLOC_079365"; transcript_id "Novel_001040"; exon_number "1"; gene_type "
At_chr5	Cufflinks	exon	44550361	44550612	.	-	.	gene_id "XLOC_079365"; transcript_id "Novel_001040"; exon_number "2"; gene_type "
At_chr5	Cufflinks	exon	47085929	47087911	.	-	.	gene_id "XLOC_079561"; transcript_id "Novel_001041"; exon_number "1"; gene_type "
At_chr5	Cufflinks	exon	47088007	47088185	.	-	.	gene_id "XLOC_079561"; transcript_id "Novel_001041"; exon_number "2"; gene_type "
At_chr5	Cufflinks	exon	47096744	47096793	.	-	.	gene_id "XLOC_079561"; transcript_id "Novel_001041"; exon_number "3"; gene_type "
At_chr5	Cufflinks	exon	47099484	47099622	.	-	.	gene_id "XLOC_079561"; transcript_id "Novel_001041"; exon_number "4"; gene_type "
At_chr5	Cufflinks	exon	47099719	47099806	.	-	.	gene_id "XLOC_079561"; transcript_id "Novel_001041"; exon_number "5"; gene_type "
At_chr5	Cufflinks	exon	47101164	47101331	.	-	.	gene_id "XLOC_079561"; transcript_id "Novel_001041"; exon_number "6"; gene_type "
At_chr5	Cufflinks	exon	47101583	47101698	.	-	.	gene_id "XLOC_079561"; transcript_id "Novel_001041"; exon_number "7"; gene_type "
At_chr5	Cufflinks	exon	47096899	47099622	.	-	.	gene_id "XLOC_079561"; transcript_id "Novel_001042"; exon_number "1"; gene_type "
At_chr5	Cufflinks	exon	47099719	47099806	.	-	.	gene_id "XLOC_079561"; transcript_id "Novel_001042"; exon_number "2"; gene_type "
At_chr5	Cufflinks	exon	47101164	47101331	.	-	.	gene_id "XLOC_079561"; transcript_id "Novel_001042"; exon_number "3"; gene_type "
At_chr5	Cufflinks	exon	47101583	47101692	.	-	.	gene_id "XLOC_079561"; transcript_id "Novel_001042"; exon_number "4"; gene_type "
At_chr5	Cufflinks	exon	49200932	49201189	.	-	.	gene_id "XLOC_079749"; transcript_id "Novel_001043"; exon_number "1"; gene_type "
At_chr5	Cufflinks	exon	49201293	49201534	.	-	.	gene_id "XLOC_079749"; transcript_id "Novel_001043"; exon_number "2"; gene_type "
At_chr5	Cufflinks	exon	49200939	49201177	.	-	.	gene_id "XLOC_079749"; transcript_id "Novel_001044"; exon_number "1"; gene_type "
At_chr5	Cufflinks	exon	49201293	49201456	.	-	.	gene_id "XLOC_079749"; transcript_id "Novel_001044"; exon_number "2"; gene_type "

At_chr5	Cufflinks	exon	49534020	49536972	.	-	.	gene_id "XLOC_079792"; transcript_id "Novel_001045"; exon_number "1"; gene_type "
At_chr5	Cufflinks	exon	49537290	49537564	.	-	.	gene_id "XLOC_079792"; transcript_id "Novel_001045"; exon_number "2"; gene_type "
At_chr5	Cufflinks	exon	51501133	51501617	.	-	.	gene_id "XLOC_080035"; transcript_id "Novel_001046"; exon_number "1"; gene_type "
At_chr5	Cufflinks	exon	51501713	51501773	.	-	.	gene_id "XLOC_080035"; transcript_id "Novel_001046"; exon_number "2"; gene_type "
At_chr5	Cufflinks	exon	51549400	51549644	.	-	.	gene_id "XLOC_080047"; transcript_id "Novel_001047"; exon_number "1"; gene_type "
At_chr5	Cufflinks	exon	51549798	51550351	.	-	.	gene_id "XLOC_080047"; transcript_id "Novel_001047"; exon_number "2"; gene_type "
At_chr5	Cufflinks	exon	51646750	51647090	.	-	.	gene_id "XLOC_080062"; transcript_id "Novel_001048"; exon_number "1"; gene_type "
At_chr5	Cufflinks	exon	51648152	51648192	.	-	.	gene_id "XLOC_080062"; transcript_id "Novel_001048"; exon_number "2"; gene_type "
At_chr5	Cufflinks	exon	51648325	51648492	.	-	.	gene_id "XLOC_080062"; transcript_id "Novel_001048"; exon_number "3"; gene_type "
At_chr5	Cufflinks	exon	51646767	51648192	.	-	.	gene_id "XLOC_080062"; transcript_id "Novel_001049"; exon_number "1"; gene_type "
At_chr5	Cufflinks	exon	51648325	51648507	.	-	.	gene_id "XLOC_080062"; transcript_id "Novel_001049"; exon_number "2"; gene_type "
At_chr5	Cufflinks	exon	52802012	52802242	.	-	.	gene_id "XLOC_080167"; transcript_id "Novel_001050"; exon_number "1"; gene_type "
At_chr5	Cufflinks	exon	52810036	52810344	.	-	.	gene_id "XLOC_080169"; transcript_id "Novel_001051"; exon_number "1"; gene_type "
At_chr6	Cufflinks	exon	5658347	5659348	.	+	.	gene_id "XLOC_080931"; transcript_id "Novel_001052"; exon_number "1"; gene_type "
At_chr6	Cufflinks	exon	5659444	5659533	.	+	.	gene_id "XLOC_080931"; transcript_id "Novel_001052"; exon_number "2"; gene_type "
At_chr6	Cufflinks	exon	5659631	5659697	.	+	.	gene_id "XLOC_080931"; transcript_id "Novel_001052"; exon_number "3"; gene_type "
At_chr6	Cufflinks	exon	5659773	5659836	.	+	.	gene_id "XLOC_080931"; transcript_id "Novel_001052"; exon_number "4"; gene_type "
At_chr6	Cufflinks	exon	5659924	5660029	.	+	.	gene_id "XLOC_080931"; transcript_id "Novel_001052"; exon_number "5"; gene_type "
At_chr6	Cufflinks	exon	5664588	5664754	.	+	.	gene_id "XLOC_080931"; transcript_id "Novel_001052"; exon_number "6"; gene_type "
At_chr6	Cufflinks	exon	5664831	5664944	.	+	.	gene_id "XLOC_080931"; transcript_id "Novel_001052"; exon_number "7"; gene_type "
At_chr6	Cufflinks	exon	6801283	6801556	.	+	.	gene_id "XLOC_081037"; transcript_id "Novel_001053"; exon_number "1"; gene_type "
At_chr6	Cufflinks	exon	6801736	6801785	.	+	.	gene_id "XLOC_081037"; transcript_id "Novel_001053"; exon_number "2"; gene_type "
At_chr6	Cufflinks	exon	6802039	6802332	.	+	.	gene_id "XLOC_081037"; transcript_id "Novel_001053"; exon_number "3"; gene_type "
At_chr6	Cufflinks	exon	6804090	6804251	.	+	.	gene_id "XLOC_081037"; transcript_id "Novel_001053"; exon_number "4"; gene_type "
At_chr6	Cufflinks	exon	6805038	6806160	.	+	.	gene_id "XLOC_081037"; transcript_id "Novel_001053"; exon_number "5"; gene_type "
At_chr6	Cufflinks	exon	6801283	6802332	.	+	.	gene_id "XLOC_081037"; transcript_id "Novel_001054"; exon_number "1"; gene_type "
At_chr6	Cufflinks	exon	6804090	6804251	.	+	.	gene_id "XLOC_081037"; transcript_id "Novel_001054"; exon_number "2"; gene_type "
At_chr6	Cufflinks	exon	6805038	6806160	.	+	.	gene_id "XLOC_081037"; transcript_id "Novel_001054"; exon_number "3"; gene_type "
At_chr6	Cufflinks	exon	6801298	6801556	.	+	.	gene_id "XLOC_081037"; transcript_id "Novel_001055"; exon_number "1"; gene_type "
At_chr6	Cufflinks	exon	6802039	6802332	.	+	.	gene_id "XLOC_081037"; transcript_id "Novel_001055"; exon_number "2"; gene_type "
At_chr6	Cufflinks	exon	6804090	6804251	.	+	.	gene_id "XLOC_081037"; transcript_id "Novel_001055"; exon_number "3"; gene_type "
At_chr6	Cufflinks	exon	6805038	6806035	.	+	.	gene_id "XLOC_081037"; transcript_id "Novel_001055"; exon_number "4"; gene_type "
At_chr6	Cufflinks	exon	7156372	7156713	.	+	.	gene_id "XLOC_081065"; transcript_id "Novel_001056"; exon_number "1"; gene_type "
At_chr6	Cufflinks	exon	7158193	7158276	.	+	.	gene_id "XLOC_081065"; transcript_id "Novel_001056"; exon_number "2"; gene_type "
At_chr6	Cufflinks	exon	7158682	7158843	.	+	.	gene_id "XLOC_081065"; transcript_id "Novel_001056"; exon_number "3"; gene_type "

At_chr6	Cufflinks	exon	7158949	7159066	.	+	.	gene_id "XLOC_081065"; transcript_id "Novel_001056"; exon_number "4"; gene_type "
At_chr6	Cufflinks	exon	7159521	7160128	.	+	.	gene_id "XLOC_081065"; transcript_id "Novel_001056"; exon_number "5"; gene_type "
At_chr6	Cufflinks	exon	7307910	7308124	.	+	.	gene_id "XLOC_081083"; transcript_id "Novel_001057"; exon_number "1"; gene_type "
At_chr6	Cufflinks	exon	7311426	7313858	.	+	.	gene_id "XLOC_081083"; transcript_id "Novel_001057"; exon_number "2"; gene_type "
At_chr6	Cufflinks	exon	7310449	7312497	.	+	.	gene_id "XLOC_081083"; transcript_id "Novel_001058"; exon_number "1"; gene_type "
At_chr6	Cufflinks	exon	7312604	7313969	.	+	.	gene_id "XLOC_081083"; transcript_id "Novel_001058"; exon_number "2"; gene_type "
At_chr6	Cufflinks	exon	9271671	9271814	.	+	.	gene_id "XLOC_081212"; transcript_id "Novel_001059"; exon_number "1"; gene_type "
At_chr6	Cufflinks	exon	9272062	9272439	.	+	.	gene_id "XLOC_081212"; transcript_id "Novel_001059"; exon_number "2"; gene_type "
At_chr6	Cufflinks	exon	9751430	9752113	.	+	.	gene_id "XLOC_081244"; transcript_id "Novel_001060"; exon_number "1"; gene_type "
At_chr6	Cufflinks	exon	9752262	9752503	.	+	.	gene_id "XLOC_081244"; transcript_id "Novel_001060"; exon_number "2"; gene_type "
At_chr6	Cufflinks	exon	9752719	9752763	.	+	.	gene_id "XLOC_081244"; transcript_id "Novel_001060"; exon_number "3"; gene_type "
At_chr6	Cufflinks	exon	9753412	9753478	.	+	.	gene_id "XLOC_081244"; transcript_id "Novel_001060"; exon_number "4"; gene_type "
At_chr6	Cufflinks	exon	9753707	9753778	.	+	.	gene_id "XLOC_081244"; transcript_id "Novel_001060"; exon_number "5"; gene_type "
At_chr6	Cufflinks	exon	9753974	9754227	.	+	.	gene_id "XLOC_081244"; transcript_id "Novel_001060"; exon_number "6"; gene_type "
At_chr6	Cufflinks	exon	9754309	9754366	.	+	.	gene_id "XLOC_081244"; transcript_id "Novel_001060"; exon_number "7"; gene_type "
At_chr6	Cufflinks	exon	9754500	9754667	.	+	.	gene_id "XLOC_081244"; transcript_id "Novel_001060"; exon_number "8"; gene_type "
At_chr6	Cufflinks	exon	9754839	9754913	.	+	.	gene_id "XLOC_081244"; transcript_id "Novel_001060"; exon_number "9"; gene_type "
At_chr6	Cufflinks	exon	9755070	9755106	.	+	.	gene_id "XLOC_081244"; transcript_id "Novel_001060"; exon_number "10"; gene_type
At_chr6	Cufflinks	exon	9751837	9752113	.	+	.	gene_id "XLOC_081244"; transcript_id "Novel_001061"; exon_number "1"; gene_type "
At_chr6	Cufflinks	exon	9752262	9752503	.	+	.	gene_id "XLOC_081244"; transcript_id "Novel_001061"; exon_number "2"; gene_type "
At_chr6	Cufflinks	exon	9752719	9752763	.	+	.	gene_id "XLOC_081244"; transcript_id "Novel_001061"; exon_number "3"; gene_type "
At_chr6	Cufflinks	exon	9753412	9753478	.	+	.	gene_id "XLOC_081244"; transcript_id "Novel_001061"; exon_number "4"; gene_type "
At_chr6	Cufflinks	exon	9753707	9753778	.	+	.	gene_id "XLOC_081244"; transcript_id "Novel_001061"; exon_number "5"; gene_type "
At_chr6	Cufflinks	exon	9753974	9754227	.	+	.	gene_id "XLOC_081244"; transcript_id "Novel_001061"; exon_number "6"; gene_type "
At_chr6	Cufflinks	exon	9754309	9754366	.	+	.	gene_id "XLOC_081244"; transcript_id "Novel_001061"; exon_number "7"; gene_type "
At_chr6	Cufflinks	exon	9754500	9754667	.	+	.	gene_id "XLOC_081244"; transcript_id "Novel_001061"; exon_number "8"; gene_type "
At_chr6	Cufflinks	exon	9754839	9755839	.	+	.	gene_id "XLOC_081244"; transcript_id "Novel_001061"; exon_number "9"; gene_type "
At_chr6	Cufflinks	exon	11470505	11470676	.	+	.	gene_id "XLOC_081349"; transcript_id "Novel_001062"; exon_number "1"; gene_type "
At_chr6	Cufflinks	exon	11470863	11472428	.	+	.	gene_id "XLOC_081349"; transcript_id "Novel_001062"; exon_number "2"; gene_type "
At_chr6	Cufflinks	exon	11470513	11470676	.	+	.	gene_id "XLOC_081349"; transcript_id "Novel_001063"; exon_number "1"; gene_type "
At_chr6	Cufflinks	exon	11470840	11472428	.	+	.	gene_id "XLOC_081349"; transcript_id "Novel_001063"; exon_number "2"; gene_type "
At_chr6	Cufflinks	exon	12605761	12605970	.	+	.	gene_id "XLOC_081406"; transcript_id "Novel_001064"; exon_number "1"; gene_type "
At_chr6	Cufflinks	exon	12606041	12606889	.	+	.	gene_id "XLOC_081406"; transcript_id "Novel_001064"; exon_number "2"; gene_type "
At_chr6	Cufflinks	exon	12607022	12607046	.	+	.	gene_id "XLOC_081406"; transcript_id "Novel_001064"; exon_number "3"; gene_type "
At_chr6	Cufflinks	exon	16281952	16282636	.	+	.	gene_id "XLOC_081542"; transcript_id "Novel_001065"; exon_number "1"; gene_type "

At_chr6	Cufflinks	exon	16284075	16284299	.	+	.	gene_id "XLOC_081542"; transcript_id "Novel_001065"; exon_number "2"; gene_type "
At_chr6	Cufflinks	exon	16284410	16284977	.	+	.	gene_id "XLOC_081542"; transcript_id "Novel_001065"; exon_number "3"; gene_type "
At_chr6	Cufflinks	exon	16283568	16284299	.	+	.	gene_id "XLOC_081542"; transcript_id "Novel_001066"; exon_number "1"; gene_type "
At_chr6	Cufflinks	exon	16284410	16284751	.	+	.	gene_id "XLOC_081542"; transcript_id "Novel_001066"; exon_number "2"; gene_type "
At_chr6	Cufflinks	exon	16448051	16448997	.	+	.	gene_id "XLOC_081552"; transcript_id "Novel_001067"; exon_number "1"; gene_type "
At_chr6	Cufflinks	exon	16450820	16450983	.	+	.	gene_id "XLOC_081553"; transcript_id "Novel_001068"; exon_number "1"; gene_type "
At_chr6	Cufflinks	exon	16451571	16454488	.	+	.	gene_id "XLOC_081553"; transcript_id "Novel_001068"; exon_number "2"; gene_type "
At_chr6	Cufflinks	exon	21492446	21492502	.	+	.	gene_id "XLOC_081715"; transcript_id "Novel_001069"; exon_number "1"; gene_type "
At_chr6	Cufflinks	exon	21492677	21492976	.	+	.	gene_id "XLOC_081715"; transcript_id "Novel_001069"; exon_number "2"; gene_type "
At_chr6	Cufflinks	exon	21495127	21495172	.	+	.	gene_id "XLOC_081715"; transcript_id "Novel_001069"; exon_number "3"; gene_type "
At_chr6	Cufflinks	exon	21496526	21497358	.	+	.	gene_id "XLOC_081715"; transcript_id "Novel_001069"; exon_number "4"; gene_type "
At_chr6	Cufflinks	exon	30593149	30594162	.	+	.	gene_id "XLOC_082023"; transcript_id "Novel_001070"; exon_number "1"; gene_type "
At_chr6	Cufflinks	exon	32758190	32758871	.	+	.	gene_id "XLOC_082083"; transcript_id "Novel_001071"; exon_number "1"; gene_type "
At_chr6	Cufflinks	exon	32759344	32759424	.	+	.	gene_id "XLOC_082083"; transcript_id "Novel_001071"; exon_number "2"; gene_type "
At_chr6	Cufflinks	exon	32759938	32760288	.	+	.	gene_id "XLOC_082083"; transcript_id "Novel_001071"; exon_number "3"; gene_type "
At_chr6	Cufflinks	exon	33530365	33530493	.	+	.	gene_id "XLOC_082119"; transcript_id "Novel_001072"; exon_number "1"; gene_type "
At_chr6	Cufflinks	exon	33530831	33532089	.	+	.	gene_id "XLOC_082119"; transcript_id "Novel_001072"; exon_number "2"; gene_type "
At_chr6	Cufflinks	exon	35806281	35806751	.	+	.	gene_id "XLOC_082199"; transcript_id "Novel_001073"; exon_number "1"; gene_type "
At_chr6	Cufflinks	exon	35807254	35808989	.	+	.	gene_id "XLOC_082199"; transcript_id "Novel_001073"; exon_number "2"; gene_type "
At_chr6	Cufflinks	exon	35806567	35806751	.	+	.	gene_id "XLOC_082199"; transcript_id "Novel_001074"; exon_number "1"; gene_type "
At_chr6	Cufflinks	exon	35807254	35807409	.	+	.	gene_id "XLOC_082199"; transcript_id "Novel_001074"; exon_number "2"; gene_type "
At_chr6	Cufflinks	exon	35808578	35808992	.	+	.	gene_id "XLOC_082199"; transcript_id "Novel_001074"; exon_number "3"; gene_type "
At_chr6	Cufflinks	exon	35806569	35806751	.	+	.	gene_id "XLOC_082199"; transcript_id "Novel_001075"; exon_number "1"; gene_type "
At_chr6	Cufflinks	exon	35807254	35807409	.	+	.	gene_id "XLOC_082199"; transcript_id "Novel_001075"; exon_number "2"; gene_type "
At_chr6	Cufflinks	exon	35807612	35807692	.	+	.	gene_id "XLOC_082199"; transcript_id "Novel_001075"; exon_number "3"; gene_type "
At_chr6	Cufflinks	exon	35808578	35808989	.	+	.	gene_id "XLOC_082199"; transcript_id "Novel_001075"; exon_number "4"; gene_type "
At_chr6	Cufflinks	exon	37818414	37818569	.	+	.	gene_id "XLOC_082343"; transcript_id "Novel_001076"; exon_number "1"; gene_type "
At_chr6	Cufflinks	exon	37818837	37819898	.	+	.	gene_id "XLOC_082343"; transcript_id "Novel_001076"; exon_number "2"; gene_type "
At_chr6	Cufflinks	exon	37819994	37820164	.	+	.	gene_id "XLOC_082343"; transcript_id "Novel_001076"; exon_number "3"; gene_type "
At_chr6	Cufflinks	exon	37820485	37821119	.	+	.	gene_id "XLOC_082343"; transcript_id "Novel_001076"; exon_number "4"; gene_type "
At_chr6	Cufflinks	exon	37818468	37818581	.	+	.	gene_id "XLOC_082343"; transcript_id "Novel_001077"; exon_number "1"; gene_type "
At_chr6	Cufflinks	exon	37818837	37819672	.	+	.	gene_id "XLOC_082343"; transcript_id "Novel_001077"; exon_number "2"; gene_type "
At_chr6	Cufflinks	exon	37819764	37820937	.	+	.	gene_id "XLOC_082343"; transcript_id "Novel_001077"; exon_number "3"; gene_type "
At_chr6	Cufflinks	exon	40911344	40913033	.	+	.	gene_id "XLOC_082573"; transcript_id "Novel_001078"; exon_number "1"; gene_type "
At_chr6	Cufflinks	exon	40916833	40917653	.	+	.	gene_id "XLOC_082573"; transcript_id "Novel_001078"; exon_number "2"; gene_type "

At_chr6	Cufflinks	exon	42964448	42964553	.	+	.	gene_id "XLOC_082758"; transcript_id "Novel_001079"; exon_number "1"; gene_type "
At_chr6	Cufflinks	exon	42964668	42965857	.	+	.	gene_id "XLOC_082758"; transcript_id "Novel_001079"; exon_number "2"; gene_type "
At_chr6	Cufflinks	exon	43013385	43013567	.	+	.	gene_id "XLOC_082763"; transcript_id "Novel_001080"; exon_number "1"; gene_type "
At_chr6	Cufflinks	exon	43013693	43014397	.	+	.	gene_id "XLOC_082763"; transcript_id "Novel_001080"; exon_number "2"; gene_type "
At_chr6	Cufflinks	exon	44923654	44923958	.	+	.	gene_id "XLOC_082962"; transcript_id "Novel_001081"; exon_number "1"; gene_type "
At_chr6	Cufflinks	exon	44924038	44927275	.	+	.	gene_id "XLOC_082962"; transcript_id "Novel_001081"; exon_number "2"; gene_type "
At_chr6	Cufflinks	exon	44923661	44925735	.	+	.	gene_id "XLOC_082962"; transcript_id "Novel_001082"; exon_number "1"; gene_type "
At_chr6	Cufflinks	exon	44926766	44927985	.	+	.	gene_id "XLOC_082962"; transcript_id "Novel_001082"; exon_number "2"; gene_type "
At_chr6	Cufflinks	exon	46161318	46161391	.	+	.	gene_id "XLOC_083128"; transcript_id "Novel_001083"; exon_number "1"; gene_type "
At_chr6	Cufflinks	exon	46161479	46161606	.	+	.	gene_id "XLOC_083128"; transcript_id "Novel_001083"; exon_number "2"; gene_type "
At_chr6	Cufflinks	exon	47826168	47826785	.	+	.	gene_id "XLOC_083270"; transcript_id "Novel_001084"; exon_number "1"; gene_type "
At_chr6	Cufflinks	exon	47828312	47828868	.	+	.	gene_id "XLOC_083270"; transcript_id "Novel_001084"; exon_number "2"; gene_type "
At_chr6	Cufflinks	exon	47889834	47890300	.	+	.	gene_id "XLOC_083279"; transcript_id "Novel_001085"; exon_number "1"; gene_type "
At_chr6	Cufflinks	exon	47890903	47890993	.	+	.	gene_id "XLOC_083279"; transcript_id "Novel_001085"; exon_number "2"; gene_type "
At_chr6	Cufflinks	exon	47912462	47912568	.	+	.	gene_id "XLOC_083284"; transcript_id "Novel_001086"; exon_number "1"; gene_type "
At_chr6	Cufflinks	exon	47912780	47912950	.	+	.	gene_id "XLOC_083284"; transcript_id "Novel_001086"; exon_number "2"; gene_type "
At_chr6	Cufflinks	exon	50040606	50042617	.	+	.	gene_id "XLOC_083587"; transcript_id "Novel_001087"; exon_number "1"; gene_type "
At_chr6	Cufflinks	exon	50043021	50043710	.	+	.	gene_id "XLOC_083587"; transcript_id "Novel_001087"; exon_number "2"; gene_type "
At_chr6	Cufflinks	exon	52867672	52867900	.	+	.	gene_id "XLOC_083898"; transcript_id "Novel_001088"; exon_number "1"; gene_type "
At_chr6	Cufflinks	exon	52868154	52872551	.	+	.	gene_id "XLOC_083898"; transcript_id "Novel_001088"; exon_number "2"; gene_type "
At_chr6	Cufflinks	exon	52867673	52867900	.	+	.	gene_id "XLOC_083898"; transcript_id "Novel_001089"; exon_number "1"; gene_type "
At_chr6	Cufflinks	exon	52868154	52868313	.	+	.	gene_id "XLOC_083898"; transcript_id "Novel_001089"; exon_number "2"; gene_type "
At_chr6	Cufflinks	exon	52868476	52868527	.	+	.	gene_id "XLOC_083898"; transcript_id "Novel_001089"; exon_number "3"; gene_type "
At_chr6	Cufflinks	exon	52868610	52872509	.	+	.	gene_id "XLOC_083898"; transcript_id "Novel_001089"; exon_number "4"; gene_type "
At_chr6	Cufflinks	exon	53679969	53680232	.	+	.	gene_id "XLOC_084005"; transcript_id "Novel_001090"; exon_number "1"; gene_type "
At_chr6	Cufflinks	exon	53787784	53788114	.	+	.	gene_id "XLOC_084017"; transcript_id "Novel_001091"; exon_number "1"; gene_type "
At_chr6	Cufflinks	exon	53790512	53794023	.	+	.	gene_id "XLOC_084017"; transcript_id "Novel_001091"; exon_number "2"; gene_type "
At_chr6	Cufflinks	exon	54257573	54261297	.	+	.	gene_id "XLOC_084086"; transcript_id "Novel_001092"; exon_number "1"; gene_type "
At_chr6	Cufflinks	exon	54261416	54261535	.	+	.	gene_id "XLOC_084086"; transcript_id "Novel_001092"; exon_number "2"; gene_type "
At_chr6	Cufflinks	exon	54262258	54262405	.	+	.	gene_id "XLOC_084086"; transcript_id "Novel_001092"; exon_number "3"; gene_type "
At_chr6	Cufflinks	exon	54262648	54263057	.	+	.	gene_id "XLOC_084086"; transcript_id "Novel_001092"; exon_number "4"; gene_type "
At_chr6	Cufflinks	exon	54263159	54263391	.	+	.	gene_id "XLOC_084086"; transcript_id "Novel_001093"; exon_number "1"; gene_type "
At_chr6	Cufflinks	exon	54263463	54264585	.	+	.	gene_id "XLOC_084086"; transcript_id "Novel_001093"; exon_number "2"; gene_type "
At_chr6	Cufflinks	exon	54263164	54264387	.	+	.	gene_id "XLOC_084086"; transcript_id "Novel_001094"; exon_number "1"; gene_type "
At_chr6	Cufflinks	exon	54264474	54264611	.	+	.	gene_id "XLOC_084086"; transcript_id "Novel_001094"; exon_number "2"; gene_type "

At_chr6	Cufflinks	exon	54264810	54265717	.	+	.	gene_id "XLOC_084086"; transcript_id "Novel_001094"; exon_number "3"; gene_type "
At_chr6	Cufflinks	exon	54263178	54263391	.	+	.	gene_id "XLOC_084086"; transcript_id "Novel_001095"; exon_number "1"; gene_type "
At_chr6	Cufflinks	exon	54263463	54263875	.	+	.	gene_id "XLOC_084086"; transcript_id "Novel_001095"; exon_number "2"; gene_type "
At_chr6	Cufflinks	exon	54264289	54264387	.	+	.	gene_id "XLOC_084086"; transcript_id "Novel_001095"; exon_number "3"; gene_type "
At_chr6	Cufflinks	exon	54264474	54264611	.	+	.	gene_id "XLOC_084086"; transcript_id "Novel_001095"; exon_number "4"; gene_type "
At_chr6	Cufflinks	exon	54264810	54266007	.	+	.	gene_id "XLOC_084086"; transcript_id "Novel_001095"; exon_number "5"; gene_type "
At_chr6	Cufflinks	exon	54266840	54268086	.	+	.	gene_id "XLOC_084087"; transcript_id "Novel_001096"; exon_number "1"; gene_type "
At_chr6	Cufflinks	exon	54275222	54275804	.	+	.	gene_id "XLOC_084091"; transcript_id "Novel_001097"; exon_number "1"; gene_type "
At_chr6	Cufflinks	exon	54275904	54276523	.	+	.	gene_id "XLOC_084091"; transcript_id "Novel_001097"; exon_number "2"; gene_type "
At_chr6	Cufflinks	exon	54313464	54313590	.	+	.	gene_id "XLOC_084097"; transcript_id "Novel_001098"; exon_number "1"; gene_type "
At_chr6	Cufflinks	exon	54314883	54315676	.	+	.	gene_id "XLOC_084097"; transcript_id "Novel_001098"; exon_number "2"; gene_type "
At_chr6	Cufflinks	exon	54336456	54336520	.	+	.	gene_id "XLOC_084106"; transcript_id "Novel_001099"; exon_number "1"; gene_type "
At_chr6	Cufflinks	exon	54336772	54337391	.	+	.	gene_id "XLOC_084106"; transcript_id "Novel_001099"; exon_number "2"; gene_type "
At_chr6	Cufflinks	exon	54336456	54336520	.	+	.	gene_id "XLOC_084106"; transcript_id "Novel_001100"; exon_number "1"; gene_type "
At_chr6	Cufflinks	exon	54336772	54337306	.	+	.	gene_id "XLOC_084106"; transcript_id "Novel_001100"; exon_number "2"; gene_type "
At_chr6	Cufflinks	exon	54337476	54337664	.	+	.	gene_id "XLOC_084106"; transcript_id "Novel_001100"; exon_number "3"; gene_type "
At_chr6	Cufflinks	exon	54337742	54338118	.	+	.	gene_id "XLOC_084106"; transcript_id "Novel_001100"; exon_number "4"; gene_type "
At_chr6	Cufflinks	exon	54336607	54336610	.	+	.	gene_id "XLOC_084106"; transcript_id "Novel_001101"; exon_number "1"; gene_type "
At_chr6	Cufflinks	exon	54336772	54337306	.	+	.	gene_id "XLOC_084106"; transcript_id "Novel_001101"; exon_number "2"; gene_type "
At_chr6	Cufflinks	exon	54337476	54337664	.	+	.	gene_id "XLOC_084106"; transcript_id "Novel_001101"; exon_number "3"; gene_type "
At_chr6	Cufflinks	exon	54337742	54338059	.	+	.	gene_id "XLOC_084106"; transcript_id "Novel_001101"; exon_number "4"; gene_type "
At_chr6	Cufflinks	exon	54336648	54337306	.	+	.	gene_id "XLOC_084106"; transcript_id "Novel_001102"; exon_number "1"; gene_type "
At_chr6	Cufflinks	exon	54337476	54337664	.	+	.	gene_id "XLOC_084106"; transcript_id "Novel_001102"; exon_number "2"; gene_type "
At_chr6	Cufflinks	exon	54337742	54338118	.	+	.	gene_id "XLOC_084106"; transcript_id "Novel_001102"; exon_number "3"; gene_type "
At_chr6	Cufflinks	exon	54490664	54490993	.	+	.	gene_id "XLOC_084133"; transcript_id "Novel_001103"; exon_number "1"; gene_type "
At_chr6	Cufflinks	exon	54492325	54492542	.	+	.	gene_id "XLOC_084133"; transcript_id "Novel_001103"; exon_number "2"; gene_type "
At_chr6	Cufflinks	exon	54492619	54492798	.	+	.	gene_id "XLOC_084133"; transcript_id "Novel_001103"; exon_number "3"; gene_type "
At_chr6	Cufflinks	exon	54492314	54492542	.	+	.	gene_id "XLOC_084133"; transcript_id "Novel_001104"; exon_number "1"; gene_type "
At_chr6	Cufflinks	exon	54492619	54492803	.	+	.	gene_id "XLOC_084133"; transcript_id "Novel_001104"; exon_number "2"; gene_type "
At_chr6	Cufflinks	exon	54493108	54493163	.	+	.	gene_id "XLOC_084138"; transcript_id "Novel_001105"; exon_number "1"; gene_type "
At_chr6	Cufflinks	exon	54493252	54494328	.	+	.	gene_id "XLOC_084138"; transcript_id "Novel_001105"; exon_number "2"; gene_type "
At_chr6	Cufflinks	exon	54551467	54551949	.	+	.	gene_id "XLOC_084156"; transcript_id "Novel_001106"; exon_number "1"; gene_type "
At_chr6	Cufflinks	exon	54553229	54553888	.	+	.	gene_id "XLOC_084156"; transcript_id "Novel_001106"; exon_number "2"; gene_type "
At_chr6	Cufflinks	exon	54551475	54551949	.	+	.	gene_id "XLOC_084156"; transcript_id "Novel_001107"; exon_number "1"; gene_type "
At_chr6	Cufflinks	exon	54553229	54553361	.	+	.	gene_id "XLOC_084156"; transcript_id "Novel_001107"; exon_number "2"; gene_type "

At_chr6	Cufflinks	exon	54553891	54554913	.	+	.	gene_id "XLOC_084156"; transcript_id "Novel_001107"; exon_number "3"; gene_type "
At_chr6	Cufflinks	exon	54619344	54619525	.	+	.	gene_id "XLOC_084171"; transcript_id "Novel_001108"; exon_number "1"; gene_type "
At_chr6	Cufflinks	exon	54619643	54619729	.	+	.	gene_id "XLOC_084171"; transcript_id "Novel_001108"; exon_number "2"; gene_type "
At_chr6	Cufflinks	exon	54620336	54620359	.	+	.	gene_id "XLOC_084172"; transcript_id "Novel_001109"; exon_number "1"; gene_type "
At_chr6	Cufflinks	exon	54620456	54620671	.	+	.	gene_id "XLOC_084172"; transcript_id "Novel_001109"; exon_number "2"; gene_type "
At_chr6	Cufflinks	exon	54652626	54653096	.	+	.	gene_id "XLOC_084177"; transcript_id "Novel_001110"; exon_number "1"; gene_type "
At_chr6	Cufflinks	exon	54653176	54653475	.	+	.	gene_id "XLOC_084177"; transcript_id "Novel_001110"; exon_number "2"; gene_type "
At_chr6	Cufflinks	exon	54698972	54700767	.	+	.	gene_id "XLOC_084183"; transcript_id "Novel_001111"; exon_number "1"; gene_type "
At_chr6	Cufflinks	exon	54701096	54701455	.	+	.	gene_id "XLOC_084183"; transcript_id "Novel_001111"; exon_number "2"; gene_type "
At_chr6	Cufflinks	exon	54699017	54703735	.	+	.	gene_id "XLOC_084183"; transcript_id "Novel_001112"; exon_number "1"; gene_type "
At_chr6	Cufflinks	exon	54704080	54706213	.	+	.	gene_id "XLOC_084183"; transcript_id "Novel_001112"; exon_number "2"; gene_type "
At_chr6	Cufflinks	exon	54723945	54725432	.	+	.	gene_id "XLOC_084184"; transcript_id "Novel_001113"; exon_number "1"; gene_type "
At_chr6	Cufflinks	exon	54740250	54740898	.	+	.	gene_id "XLOC_084184"; transcript_id "Novel_001114"; exon_number "1"; gene_type "
At_chr6	Cufflinks	exon	54740979	54743269	.	+	.	gene_id "XLOC_084184"; transcript_id "Novel_001114"; exon_number "2"; gene_type "
At_chr6	Cufflinks	exon	54743951	54744097	.	+	.	gene_id "XLOC_084184"; transcript_id "Novel_001114"; exon_number "3"; gene_type "
At_chr6	Cufflinks	exon	54744348	54745442	.	+	.	gene_id "XLOC_084184"; transcript_id "Novel_001114"; exon_number "4"; gene_type "
At_chr6	Cufflinks	exon	54740308	54742681	.	+	.	gene_id "XLOC_084184"; transcript_id "Novel_001115"; exon_number "1"; gene_type "
At_chr6	Cufflinks	exon	54742976	54743283	.	+	.	gene_id "XLOC_084184"; transcript_id "Novel_001115"; exon_number "2"; gene_type "
At_chr6	Cufflinks	exon	54740308	54740898	.	+	.	gene_id "XLOC_084184"; transcript_id "Novel_001116"; exon_number "1"; gene_type "
At_chr6	Cufflinks	exon	54740979	54742681	.	+	.	gene_id "XLOC_084184"; transcript_id "Novel_001116"; exon_number "2"; gene_type "
At_chr6	Cufflinks	exon	54742976	54743269	.	+	.	gene_id "XLOC_084184"; transcript_id "Novel_001116"; exon_number "3"; gene_type "
At_chr6	Cufflinks	exon	54743951	54744097	.	+	.	gene_id "XLOC_084184"; transcript_id "Novel_001116"; exon_number "4"; gene_type "
At_chr6	Cufflinks	exon	54744348	54744492	.	+	.	gene_id "XLOC_084184"; transcript_id "Novel_001116"; exon_number "5"; gene_type "
At_chr6	Cufflinks	exon	54747706	54747918	.	+	.	gene_id "XLOC_084184"; transcript_id "Novel_001117"; exon_number "1"; gene_type "
At_chr6	Cufflinks	exon	54748199	54748252	.	+	.	gene_id "XLOC_084184"; transcript_id "Novel_001117"; exon_number "2"; gene_type "
At_chr6	Cufflinks	exon	54748623	54750720	.	+	.	gene_id "XLOC_084184"; transcript_id "Novel_001117"; exon_number "3"; gene_type "
At_chr6	Cufflinks	exon	54750796	54752339	.	+	.	gene_id "XLOC_084184"; transcript_id "Novel_001117"; exon_number "4"; gene_type "
At_chr6	Cufflinks	exon	54748207	54748252	.	+	.	gene_id "XLOC_084184"; transcript_id "Novel_001118"; exon_number "1"; gene_type "
At_chr6	Cufflinks	exon	54748623	54749893	.	+	.	gene_id "XLOC_084184"; transcript_id "Novel_001118"; exon_number "2"; gene_type "
At_chr6	Cufflinks	exon	54750646	54750940	.	+	.	gene_id "XLOC_084184"; transcript_id "Novel_001118"; exon_number "3"; gene_type "
At_chr6	Cufflinks	exon	54748595	54751567	.	+	.	gene_id "XLOC_084184"; transcript_id "Novel_001119"; exon_number "1"; gene_type "
At_chr6	Cufflinks	exon	54751876	54752201	.	+	.	gene_id "XLOC_084184"; transcript_id "Novel_001119"; exon_number "2"; gene_type "
At_chr6	Cufflinks	exon	54879863	54880528	.	+	.	gene_id "XLOC_084212"; transcript_id "Novel_001120"; exon_number "1"; gene_type "
At_chr6	Cufflinks	exon	54880616	54881856	.	+	.	gene_id "XLOC_084212"; transcript_id "Novel_001120"; exon_number "2"; gene_type "
At_chr6	Cufflinks	exon	54879901	54881695	.	+	.	gene_id "XLOC_084212"; transcript_id "Novel_001121"; exon_number "1"; gene_type "

At_chr6	Cufflinks	exon	54882007	54882907	.	+	.	gene_id "XLOC_084212"; transcript_id "Novel_001121"; exon_number "2"; gene_type "
At_chr6	Cufflinks	exon	54888952	54889266	.	+	.	gene_id "XLOC_084212"; transcript_id "Novel_001122"; exon_number "1"; gene_type "
At_chr6	Cufflinks	exon	54889423	54889630	.	+	.	gene_id "XLOC_084212"; transcript_id "Novel_001122"; exon_number "2"; gene_type "
At_chr6	Cufflinks	exon	54896618	54897129	.	+	.	gene_id "XLOC_084215"; transcript_id "Novel_001123"; exon_number "1"; gene_type "
At_chr6	Cufflinks	exon	54897228	54898081	.	+	.	gene_id "XLOC_084215"; transcript_id "Novel_001123"; exon_number "2"; gene_type "
At_chr6	Cufflinks	exon	1592338	1593664	.	-	.	gene_id "XLOC_084485"; transcript_id "Novel_001124"; exon_number "1"; gene_type "
At_chr6	Cufflinks	exon	1593757	1594026	.	-	.	gene_id "XLOC_084485"; transcript_id "Novel_001124"; exon_number "2"; gene_type "
At_chr6	Cufflinks	exon	1594129	1594403	.	-	.	gene_id "XLOC_084485"; transcript_id "Novel_001124"; exon_number "3"; gene_type "
At_chr6	Cufflinks	exon	1594951	1595164	.	-	.	gene_id "XLOC_084485"; transcript_id "Novel_001124"; exon_number "4"; gene_type "
At_chr6	Cufflinks	exon	2308864	2309542	.	-	.	gene_id "XLOC_084544"; transcript_id "Novel_001125"; exon_number "1"; gene_type "
At_chr6	Cufflinks	exon	2309738	2309842	.	-	.	gene_id "XLOC_084544"; transcript_id "Novel_001125"; exon_number "2"; gene_type "
At_chr6	Cufflinks	exon	2309929	2310179	.	-	.	gene_id "XLOC_084544"; transcript_id "Novel_001125"; exon_number "3"; gene_type "
At_chr6	Cufflinks	exon	2825566	2828417	.	-	.	gene_id "XLOC_084595"; transcript_id "Novel_001126"; exon_number "1"; gene_type "
At_chr6	Cufflinks	exon	2828505	2828854	.	-	.	gene_id "XLOC_084595"; transcript_id "Novel_001126"; exon_number "2"; gene_type "
At_chr6	Cufflinks	exon	2828981	2829401	.	-	.	gene_id "XLOC_084595"; transcript_id "Novel_001126"; exon_number "3"; gene_type "
At_chr6	Cufflinks	exon	2825788	2828417	.	-	.	gene_id "XLOC_084595"; transcript_id "Novel_001127"; exon_number "1"; gene_type "
At_chr6	Cufflinks	exon	2828505	2828854	.	-	.	gene_id "XLOC_084595"; transcript_id "Novel_001127"; exon_number "2"; gene_type "
At_chr6	Cufflinks	exon	2829421	2829472	.	-	.	gene_id "XLOC_084595"; transcript_id "Novel_001127"; exon_number "3"; gene_type "
At_chr6	Cufflinks	exon	2917598	2917966	.	-	.	gene_id "XLOC_084609"; transcript_id "Novel_001128"; exon_number "1"; gene_type "
At_chr6	Cufflinks	exon	2918318	2918426	.	-	.	gene_id "XLOC_084609"; transcript_id "Novel_001128"; exon_number "2"; gene_type "
At_chr6	Cufflinks	exon	2918512	2918940	.	-	.	gene_id "XLOC_084609"; transcript_id "Novel_001128"; exon_number "3"; gene_type "
At_chr6	Cufflinks	exon	3414110	3419687	.	-	.	gene_id "XLOC_084706"; transcript_id "Novel_001129"; exon_number "1"; gene_type "
At_chr6	Cufflinks	exon	3419846	3420373	.	-	.	gene_id "XLOC_084706"; transcript_id "Novel_001129"; exon_number "2"; gene_type "
At_chr6	Cufflinks	exon	3414110	3419687	.	-	.	gene_id "XLOC_084706"; transcript_id "Novel_001130"; exon_number "1"; gene_type "
At_chr6	Cufflinks	exon	3419846	3419957	.	-	.	gene_id "XLOC_084706"; transcript_id "Novel_001130"; exon_number "2"; gene_type "
At_chr6	Cufflinks	exon	3420063	3420159	.	-	.	gene_id "XLOC_084706"; transcript_id "Novel_001130"; exon_number "3"; gene_type "
At_chr6	Cufflinks	exon	3420277	3420374	.	-	.	gene_id "XLOC_084706"; transcript_id "Novel_001130"; exon_number "4"; gene_type "
At_chr6	Cufflinks	exon	11012758	11013100	.	-	.	gene_id "XLOC_085350"; transcript_id "Novel_001131"; exon_number "1"; gene_type "
At_chr6	Cufflinks	exon	11013211	11013316	.	-	.	gene_id "XLOC_085350"; transcript_id "Novel_001131"; exon_number "2"; gene_type "
At_chr6	Cufflinks	exon	11013470	11013744	.	-	.	gene_id "XLOC_085350"; transcript_id "Novel_001131"; exon_number "3"; gene_type "
At_chr6	Cufflinks	exon	11012758	11013316	.	-	.	gene_id "XLOC_085350"; transcript_id "Novel_001132"; exon_number "1"; gene_type "
At_chr6	Cufflinks	exon	11013470	11013744	.	-	.	gene_id "XLOC_085350"; transcript_id "Novel_001132"; exon_number "2"; gene_type "
At_chr6	Cufflinks	exon	16375695	16376184	.	-	.	gene_id "XLOC_085611"; transcript_id "Novel_001133"; exon_number "1"; gene_type "
At_chr6	Cufflinks	exon	16376304	16376589	.	-	.	gene_id "XLOC_085611"; transcript_id "Novel_001133"; exon_number "2"; gene_type "
At_chr6	Cufflinks	exon	16376108	16376214	.	-	.	gene_id "XLOC_085611"; transcript_id "Novel_001134"; exon_number "1"; gene_type "

At_chr6	Cufflinks	exon	16376304	16376596	.	-	.	gene_id "XLOC_085611"; transcript_id "Novel_001134"; exon_number "2"; gene_type "
At_chr6	Cufflinks	exon	17284357	17285195	.	-	.	gene_id "XLOC_085636"; transcript_id "Novel_001135"; exon_number "1"; gene_type "
At_chr6	Cufflinks	exon	17285285	17285554	.	-	.	gene_id "XLOC_085636"; transcript_id "Novel_001135"; exon_number "2"; gene_type "
At_chr6	Cufflinks	exon	17285969	17286662	.	-	.	gene_id "XLOC_085636"; transcript_id "Novel_001135"; exon_number "3"; gene_type "
At_chr6	Cufflinks	exon	22366828	22367267	.	-	.	gene_id "XLOC_085845"; transcript_id "Novel_001136"; exon_number "1"; gene_type "
At_chr6	Cufflinks	exon	22368443	22368832	.	-	.	gene_id "XLOC_085845"; transcript_id "Novel_001136"; exon_number "2"; gene_type "
At_chr6	Cufflinks	exon	22368969	22369131	.	-	.	gene_id "XLOC_085845"; transcript_id "Novel_001136"; exon_number "3"; gene_type "
At_chr6	Cufflinks	exon	26023462	26024005	.	-	.	gene_id "XLOC_085960"; transcript_id "Novel_001137"; exon_number "1"; gene_type "
At_chr6	Cufflinks	exon	26024101	26024275	.	-	.	gene_id "XLOC_085960"; transcript_id "Novel_001137"; exon_number "2"; gene_type "
At_chr6	Cufflinks	exon	26024568	26024664	.	-	.	gene_id "XLOC_085960"; transcript_id "Novel_001137"; exon_number "3"; gene_type "
At_chr6	Cufflinks	exon	26024945	26025008	.	-	.	gene_id "XLOC_085960"; transcript_id "Novel_001137"; exon_number "4"; gene_type "
At_chr6	Cufflinks	exon	26025617	26025783	.	-	.	gene_id "XLOC_085960"; transcript_id "Novel_001137"; exon_number "5"; gene_type "
At_chr6	Cufflinks	exon	26025976	26026047	.	-	.	gene_id "XLOC_085960"; transcript_id "Novel_001137"; exon_number "6"; gene_type "
At_chr6	Cufflinks	exon	26026243	26026316	.	-	.	gene_id "XLOC_085960"; transcript_id "Novel_001137"; exon_number "7"; gene_type "
At_chr6	Cufflinks	exon	26023614	26024005	.	-	.	gene_id "XLOC_085960"; transcript_id "Novel_001138"; exon_number "1"; gene_type "
At_chr6	Cufflinks	exon	26024101	26025783	.	-	.	gene_id "XLOC_085960"; transcript_id "Novel_001138"; exon_number "2"; gene_type "
At_chr6	Cufflinks	exon	26025976	26026047	.	-	.	gene_id "XLOC_085960"; transcript_id "Novel_001138"; exon_number "3"; gene_type "
At_chr6	Cufflinks	exon	26026243	26026354	.	-	.	gene_id "XLOC_085960"; transcript_id "Novel_001138"; exon_number "4"; gene_type "
At_chr6	Cufflinks	exon	28386300	28386778	.	-	.	gene_id "XLOC_086038"; transcript_id "Novel_001139"; exon_number "1"; gene_type "
At_chr6	Cufflinks	exon	28387456	28389579	.	-	.	gene_id "XLOC_086038"; transcript_id "Novel_001139"; exon_number "2"; gene_type "
At_chr6	Cufflinks	exon	35177522	35179734	.	-	.	gene_id "XLOC_086281"; transcript_id "Novel_001140"; exon_number "1"; gene_type "
At_chr6	Cufflinks	exon	35179800	35180109	.	-	.	gene_id "XLOC_086281"; transcript_id "Novel_001140"; exon_number "2"; gene_type "
At_chr6	Cufflinks	exon	35180293	35180335	.	-	.	gene_id "XLOC_086281"; transcript_id "Novel_001140"; exon_number "3"; gene_type "
At_chr6	Cufflinks	exon	35180549	35180651	.	-	.	gene_id "XLOC_086281"; transcript_id "Novel_001140"; exon_number "4"; gene_type "
At_chr6	Cufflinks	exon	35180766	35181121	.	-	.	gene_id "XLOC_086281"; transcript_id "Novel_001140"; exon_number "5"; gene_type "
At_chr6	Cufflinks	exon	35177676	35178430	.	-	.	gene_id "XLOC_086281"; transcript_id "Novel_001141"; exon_number "1"; gene_type "
At_chr6	Cufflinks	exon	35178885	35179092	.	-	.	gene_id "XLOC_086281"; transcript_id "Novel_001141"; exon_number "2"; gene_type "
At_chr6	Cufflinks	exon	35179579	35179699	.	-	.	gene_id "XLOC_086281"; transcript_id "Novel_001141"; exon_number "3"; gene_type "
At_chr6	Cufflinks	exon	35179800	35180109	.	-	.	gene_id "XLOC_086281"; transcript_id "Novel_001141"; exon_number "4"; gene_type "
At_chr6	Cufflinks	exon	35180293	35180335	.	-	.	gene_id "XLOC_086281"; transcript_id "Novel_001141"; exon_number "5"; gene_type "
At_chr6	Cufflinks	exon	35180549	35180651	.	-	.	gene_id "XLOC_086281"; transcript_id "Novel_001141"; exon_number "6"; gene_type "
At_chr6	Cufflinks	exon	35180766	35181138	.	-	.	gene_id "XLOC_086281"; transcript_id "Novel_001141"; exon_number "7"; gene_type "
At_chr6	Cufflinks	exon	37978963	37979265	.	-	.	gene_id "XLOC_086409"; transcript_id "Novel_001142"; exon_number "1"; gene_type "
At_chr6	Cufflinks	exon	37979340	37979603	.	-	.	gene_id "XLOC_086409"; transcript_id "Novel_001142"; exon_number "2"; gene_type "
At_chr6	Cufflinks	exon	37978964	37979252	.	-	.	gene_id "XLOC_086409"; transcript_id "Novel_001143"; exon_number "1"; gene_type "

At_chr6	Cufflinks	exon	37979340	37979465	.	-	.	gene_id "XLOC_086409"; transcript_id "Novel_001143"; exon_number "2"; gene_type "
At_chr6	Cufflinks	exon	38003756	38004321	.	-	.	gene_id "XLOC_086409"; transcript_id "Novel_001144"; exon_number "1"; gene_type "
At_chr6	Cufflinks	exon	38004396	38004521	.	-	.	gene_id "XLOC_086409"; transcript_id "Novel_001144"; exon_number "2"; gene_type "
At_chr6	Cufflinks	exon	38004612	38004706	.	-	.	gene_id "XLOC_086409"; transcript_id "Novel_001144"; exon_number "3"; gene_type "
At_chr6	Cufflinks	exon	38005252	38005337	.	-	.	gene_id "XLOC_086409"; transcript_id "Novel_001144"; exon_number "4"; gene_type "
At_chr6	Cufflinks	exon	38005414	38005563	.	-	.	gene_id "XLOC_086409"; transcript_id "Novel_001144"; exon_number "5"; gene_type "
At_chr6	Cufflinks	exon	38005643	38005741	.	-	.	gene_id "XLOC_086409"; transcript_id "Novel_001144"; exon_number "6"; gene_type "
At_chr6	Cufflinks	exon	38003756	38004521	.	-	.	gene_id "XLOC_086409"; transcript_id "Novel_001145"; exon_number "1"; gene_type "
At_chr6	Cufflinks	exon	38004612	38004706	.	-	.	gene_id "XLOC_086409"; transcript_id "Novel_001145"; exon_number "2"; gene_type "
At_chr6	Cufflinks	exon	38005252	38005337	.	-	.	gene_id "XLOC_086409"; transcript_id "Novel_001145"; exon_number "3"; gene_type "
At_chr6	Cufflinks	exon	38005414	38005563	.	-	.	gene_id "XLOC_086409"; transcript_id "Novel_001145"; exon_number "4"; gene_type "
At_chr6	Cufflinks	exon	38005643	38005741	.	-	.	gene_id "XLOC_086409"; transcript_id "Novel_001145"; exon_number "5"; gene_type "
At_chr6	Cufflinks	exon	38003931	38004321	.	-	.	gene_id "XLOC_086409"; transcript_id "Novel_001146"; exon_number "1"; gene_type "
At_chr6	Cufflinks	exon	38004396	38004521	.	-	.	gene_id "XLOC_086409"; transcript_id "Novel_001146"; exon_number "2"; gene_type "
At_chr6	Cufflinks	exon	38004612	38004706	.	-	.	gene_id "XLOC_086409"; transcript_id "Novel_001146"; exon_number "3"; gene_type "
At_chr6	Cufflinks	exon	38005252	38005337	.	-	.	gene_id "XLOC_086409"; transcript_id "Novel_001146"; exon_number "4"; gene_type "
At_chr6	Cufflinks	exon	38005414	38005563	.	-	.	gene_id "XLOC_086409"; transcript_id "Novel_001146"; exon_number "5"; gene_type "
At_chr6	Cufflinks	exon	38005680	38005738	.	-	.	gene_id "XLOC_086409"; transcript_id "Novel_001146"; exon_number "6"; gene_type "
At_chr6	Cufflinks	exon	37981056	37981096	.	-	.	gene_id "XLOC_086412"; transcript_id "Novel_001147"; exon_number "1"; gene_type "
At_chr6	Cufflinks	exon	37981172	37981324	.	-	.	gene_id "XLOC_086412"; transcript_id "Novel_001147"; exon_number "2"; gene_type "
At_chr6	Cufflinks	exon	37981422	37981528	.	-	.	gene_id "XLOC_086412"; transcript_id "Novel_001147"; exon_number "3"; gene_type "
At_chr6	Cufflinks	exon	38519606	38524470	.	-	.	gene_id "XLOC_086450"; transcript_id "Novel_001148"; exon_number "1"; gene_type "
At_chr6	Cufflinks	exon	38524948	38525074	.	-	.	gene_id "XLOC_086450"; transcript_id "Novel_001148"; exon_number "2"; gene_type "
At_chr6	Cufflinks	exon	38519702	38523609	.	-	.	gene_id "XLOC_086450"; transcript_id "Novel_001149"; exon_number "1"; gene_type "
At_chr6	Cufflinks	exon	38524416	38525136	.	-	.	gene_id "XLOC_086450"; transcript_id "Novel_001149"; exon_number "2"; gene_type "
At_chr6	Cufflinks	exon	38637930	38638566	.	-	.	gene_id "XLOC_086459"; transcript_id "Novel_001150"; exon_number "1"; gene_type "
At_chr6	Cufflinks	exon	38638834	38638951	.	-	.	gene_id "XLOC_086459"; transcript_id "Novel_001150"; exon_number "2"; gene_type "
At_chr6	Cufflinks	exon	39287981	39288026	.	-	.	gene_id "XLOC_086519"; transcript_id "Novel_001151"; exon_number "1"; gene_type "
At_chr6	Cufflinks	exon	39289815	39289877	.	-	.	gene_id "XLOC_086519"; transcript_id "Novel_001151"; exon_number "2"; gene_type "
At_chr6	Cufflinks	exon	39294092	39294139	.	-	.	gene_id "XLOC_086519"; transcript_id "Novel_001151"; exon_number "3"; gene_type "
At_chr6	Cufflinks	exon	39294202	39294264	.	-	.	gene_id "XLOC_086519"; transcript_id "Novel_001151"; exon_number "4"; gene_type "
At_chr6	Cufflinks	exon	39296007	39296050	.	-	.	gene_id "XLOC_086519"; transcript_id "Novel_001151"; exon_number "5"; gene_type "
At_chr6	Cufflinks	exon	39296145	39296306	.	-	.	gene_id "XLOC_086519"; transcript_id "Novel_001151"; exon_number "6"; gene_type "
At_chr6	Cufflinks	exon	42851012	42852305	.	-	.	gene_id "XLOC_086762"; transcript_id "Novel_001152"; exon_number "1"; gene_type "
At_chr6	Cufflinks	exon	42853533	42853740	.	-	.	gene_id "XLOC_086762"; transcript_id "Novel_001152"; exon_number "2"; gene_type "

At_chr6	Cufflinks	exon	43016683	43017590	.	-	.	gene_id "XLOC_086784"; transcript_id "Novel_001153"; exon_number "1"; gene_type "
At_chr6	Cufflinks	exon	43017771	43017835	.	-	.	gene_id "XLOC_086784"; transcript_id "Novel_001153"; exon_number "2"; gene_type "
At_chr6	Cufflinks	exon	43018123	43018202	.	-	.	gene_id "XLOC_086784"; transcript_id "Novel_001153"; exon_number "3"; gene_type "
At_chr6	Cufflinks	exon	43018497	43018566	.	-	.	gene_id "XLOC_086784"; transcript_id "Novel_001153"; exon_number "4"; gene_type "
At_chr6	Cufflinks	exon	43018855	43019075	.	-	.	gene_id "XLOC_086784"; transcript_id "Novel_001153"; exon_number "5"; gene_type "
At_chr6	Cufflinks	exon	43019334	43019432	.	-	.	gene_id "XLOC_086784"; transcript_id "Novel_001153"; exon_number "6"; gene_type "
At_chr6	Cufflinks	exon	43019849	43020478	.	-	.	gene_id "XLOC_086784"; transcript_id "Novel_001153"; exon_number "7"; gene_type "
At_chr6	Cufflinks	exon	43017081	43017590	.	-	.	gene_id "XLOC_086784"; transcript_id "Novel_001154"; exon_number "1"; gene_type "
At_chr6	Cufflinks	exon	43017771	43017835	.	-	.	gene_id "XLOC_086784"; transcript_id "Novel_001154"; exon_number "2"; gene_type "
At_chr6	Cufflinks	exon	43018123	43018462	.	-	.	gene_id "XLOC_086784"; transcript_id "Novel_001154"; exon_number "3"; gene_type "
At_chr6	Cufflinks	exon	43018295	43018566	.	-	.	gene_id "XLOC_086784"; transcript_id "Novel_001155"; exon_number "1"; gene_type "
At_chr6	Cufflinks	exon	43018855	43019075	.	-	.	gene_id "XLOC_086784"; transcript_id "Novel_001155"; exon_number "2"; gene_type "
At_chr6	Cufflinks	exon	43019334	43019432	.	-	.	gene_id "XLOC_086784"; transcript_id "Novel_001155"; exon_number "3"; gene_type "
At_chr6	Cufflinks	exon	43019849	43020470	.	-	.	gene_id "XLOC_086784"; transcript_id "Novel_001155"; exon_number "4"; gene_type "
At_chr6	Cufflinks	exon	43083161	43084852	.	-	.	gene_id "XLOC_086790"; transcript_id "Novel_001156"; exon_number "1"; gene_type "
At_chr6	Cufflinks	exon	43084934	43085048	.	-	.	gene_id "XLOC_086790"; transcript_id "Novel_001156"; exon_number "2"; gene_type "
At_chr6	Cufflinks	exon	43085932	43086039	.	-	.	gene_id "XLOC_086790"; transcript_id "Novel_001156"; exon_number "3"; gene_type "
At_chr6	Cufflinks	exon	43086187	43086279	.	-	.	gene_id "XLOC_086790"; transcript_id "Novel_001156"; exon_number "4"; gene_type "
At_chr6	Cufflinks	exon	43086629	43087085	.	-	.	gene_id "XLOC_086790"; transcript_id "Novel_001156"; exon_number "5"; gene_type "
At_chr6	Cufflinks	exon	43085441	43086279	.	-	.	gene_id "XLOC_086790"; transcript_id "Novel_001157"; exon_number "1"; gene_type "
At_chr6	Cufflinks	exon	43086629	43086998	.	-	.	gene_id "XLOC_086790"; transcript_id "Novel_001157"; exon_number "2"; gene_type "
At_chr6	Cufflinks	exon	43086024	43086039	.	-	.	gene_id "XLOC_086790"; transcript_id "Novel_001158"; exon_number "1"; gene_type "
At_chr6	Cufflinks	exon	43086185	43086279	.	-	.	gene_id "XLOC_086790"; transcript_id "Novel_001158"; exon_number "2"; gene_type "
At_chr6	Cufflinks	exon	43086629	43087031	.	-	.	gene_id "XLOC_086790"; transcript_id "Novel_001158"; exon_number "3"; gene_type "
At_chr6	Cufflinks	exon	43471553	43472290	.	-	.	gene_id "XLOC_086835"; transcript_id "Novel_001159"; exon_number "1"; gene_type "
At_chr6	Cufflinks	exon	43472370	43473072	.	-	.	gene_id "XLOC_086835"; transcript_id "Novel_001159"; exon_number "2"; gene_type "
At_chr6	Cufflinks	exon	43473208	43473371	.	-	.	gene_id "XLOC_086835"; transcript_id "Novel_001159"; exon_number "3"; gene_type "
At_chr6	Cufflinks	exon	43473482	43473547	.	-	.	gene_id "XLOC_086835"; transcript_id "Novel_001159"; exon_number "4"; gene_type "
At_chr6	Cufflinks	exon	43473636	43473796	.	-	.	gene_id "XLOC_086835"; transcript_id "Novel_001159"; exon_number "5"; gene_type "
At_chr6	Cufflinks	exon	43473891	43474104	.	-	.	gene_id "XLOC_086835"; transcript_id "Novel_001159"; exon_number "6"; gene_type "
At_chr6	Cufflinks	exon	43474971	43475517	.	-	.	gene_id "XLOC_086835"; transcript_id "Novel_001159"; exon_number "7"; gene_type "
At_chr6	Cufflinks	exon	43471558	43472290	.	-	.	gene_id "XLOC_086835"; transcript_id "Novel_001160"; exon_number "1"; gene_type "
At_chr6	Cufflinks	exon	43472370	43473072	.	-	.	gene_id "XLOC_086835"; transcript_id "Novel_001160"; exon_number "2"; gene_type "
At_chr6	Cufflinks	exon	43473208	43473371	.	-	.	gene_id "XLOC_086835"; transcript_id "Novel_001160"; exon_number "3"; gene_type "
At_chr6	Cufflinks	exon	43473482	43473547	.	-	.	gene_id "XLOC_086835"; transcript_id "Novel_001160"; exon_number "4"; gene_type "

At_chr6	Cufflinks	exon	43473636	43475532	.	-	.	gene_id "XLOC_086835"; transcript_id "Novel_001160"; exon_number "5"; gene_type "
At_chr6	Cufflinks	exon	43575752	43576139	.	-	.	gene_id "XLOC_086841"; transcript_id "Novel_001161"; exon_number "1"; gene_type "
At_chr6	Cufflinks	exon	43576242	43576517	.	-	.	gene_id "XLOC_086841"; transcript_id "Novel_001161"; exon_number "2"; gene_type "
At_chr6	Cufflinks	exon	43576597	43576613	.	-	.	gene_id "XLOC_086841"; transcript_id "Novel_001161"; exon_number "3"; gene_type "
At_chr6	Cufflinks	exon	43663736	43663799	.	-	.	gene_id "XLOC_086841"; transcript_id "Novel_001161"; exon_number "4"; gene_type "
At_chr6	Cufflinks	exon	43663910	43664041	.	-	.	gene_id "XLOC_086841"; transcript_id "Novel_001161"; exon_number "5"; gene_type "
At_chr6	Cufflinks	exon	43575752	43576139	.	-	.	gene_id "XLOC_086841"; transcript_id "Novel_001162"; exon_number "1"; gene_type "
At_chr6	Cufflinks	exon	43576242	43576517	.	-	.	gene_id "XLOC_086841"; transcript_id "Novel_001162"; exon_number "2"; gene_type "
At_chr6	Cufflinks	exon	43663719	43663799	.	-	.	gene_id "XLOC_086841"; transcript_id "Novel_001162"; exon_number "3"; gene_type "
At_chr6	Cufflinks	exon	43663910	43664041	.	-	.	gene_id "XLOC_086841"; transcript_id "Novel_001162"; exon_number "4"; gene_type "
At_chr6	Cufflinks	exon	43576620	43576921	.	-	.	gene_id "XLOC_086842"; transcript_id "Novel_001163"; exon_number "1"; gene_type "
At_chr6	Cufflinks	exon	43577019	43577240	.	-	.	gene_id "XLOC_086842"; transcript_id "Novel_001163"; exon_number "2"; gene_type "
At_chr6	Cufflinks	exon	43577332	43577412	.	-	.	gene_id "XLOC_086842"; transcript_id "Novel_001163"; exon_number "3"; gene_type "
At_chr6	Cufflinks	exon	43577497	43577658	.	-	.	gene_id "XLOC_086842"; transcript_id "Novel_001163"; exon_number "4"; gene_type "
At_chr6	Cufflinks	exon	43577769	43577915	.	-	.	gene_id "XLOC_086842"; transcript_id "Novel_001163"; exon_number "5"; gene_type "
At_chr6	Cufflinks	exon	43578508	43578644	.	-	.	gene_id "XLOC_086842"; transcript_id "Novel_001164"; exon_number "1"; gene_type "
At_chr6	Cufflinks	exon	43666740	43667070	.	-	.	gene_id "XLOC_086842"; transcript_id "Novel_001164"; exon_number "2"; gene_type "
At_chr6	Cufflinks	exon	43662666	43663261	.	-	.	gene_id "XLOC_086844"; transcript_id "Novel_001165"; exon_number "1"; gene_type "
At_chr6	Cufflinks	exon	43663363	43663470	.	-	.	gene_id "XLOC_086844"; transcript_id "Novel_001165"; exon_number "2"; gene_type "
At_chr6	Cufflinks	exon	43663553	43663613	.	-	.	gene_id "XLOC_086844"; transcript_id "Novel_001165"; exon_number "3"; gene_type "
At_chr6	Cufflinks	exon	43662704	43663261	.	-	.	gene_id "XLOC_086844"; transcript_id "Novel_001166"; exon_number "1"; gene_type "
At_chr6	Cufflinks	exon	43663363	43663610	.	-	.	gene_id "XLOC_086844"; transcript_id "Novel_001166"; exon_number "2"; gene_type "
At_chr6	Cufflinks	exon	43807019	43807141	.	-	.	gene_id "XLOC_086859"; transcript_id "Novel_001167"; exon_number "1"; gene_type "
At_chr6	Cufflinks	exon	43807771	43807937	.	-	.	gene_id "XLOC_086859"; transcript_id "Novel_001167"; exon_number "2"; gene_type "
At_chr6	Cufflinks	exon	43808203	43808255	.	-	.	gene_id "XLOC_086859"; transcript_id "Novel_001167"; exon_number "3"; gene_type "
At_chr6	Cufflinks	exon	43808337	43808385	.	-	.	gene_id "XLOC_086859"; transcript_id "Novel_001167"; exon_number "4"; gene_type "
At_chr6	Cufflinks	exon	43808708	43808788	.	-	.	gene_id "XLOC_086860"; transcript_id "Novel_001168"; exon_number "1"; gene_type "
At_chr6	Cufflinks	exon	43809133	43809244	.	-	.	gene_id "XLOC_086860"; transcript_id "Novel_001168"; exon_number "2"; gene_type "
At_chr6	Cufflinks	exon	43809338	43809616	.	-	.	gene_id "XLOC_086860"; transcript_id "Novel_001168"; exon_number "3"; gene_type "
At_chr6	Cufflinks	exon	43901806	43902269	.	-	.	gene_id "XLOC_086867"; transcript_id "Novel_001169"; exon_number "1"; gene_type "
At_chr6	Cufflinks	exon	43905542	43905630	.	-	.	gene_id "XLOC_086867"; transcript_id "Novel_001169"; exon_number "2"; gene_type "
At_chr6	Cufflinks	exon	43906945	43907010	.	-	.	gene_id "XLOC_086867"; transcript_id "Novel_001169"; exon_number "3"; gene_type "
At_chr6	Cufflinks	exon	43907100	43907292	.	-	.	gene_id "XLOC_086867"; transcript_id "Novel_001169"; exon_number "4"; gene_type "
At_chr6	Cufflinks	exon	44489957	44495084	.	-	.	gene_id "XLOC_086919"; transcript_id "Novel_001170"; exon_number "1"; gene_type "
At_chr6	Cufflinks	exon	44495182	44495552	.	-	.	gene_id "XLOC_086919"; transcript_id "Novel_001170"; exon_number "2"; gene_type "

At_chr6	Cufflinks	exon	44495626	44495743	.	-	.	gene_id "XLOC_086919"; transcript_id "Novel_001170"; exon_number "3"; gene_type "
At_chr6	Cufflinks	exon	44495847	44496050	.	-	.	gene_id "XLOC_086919"; transcript_id "Novel_001170"; exon_number "4"; gene_type "
At_chr6	Cufflinks	exon	44496240	44496488	.	-	.	gene_id "XLOC_086919"; transcript_id "Novel_001170"; exon_number "5"; gene_type "
At_chr6	Cufflinks	exon	44636342	44636545	.	-	.	gene_id "XLOC_086927"; transcript_id "Novel_001171"; exon_number "1"; gene_type "
At_chr6	Cufflinks	exon	44995004	44995612	.	-	.	gene_id "XLOC_086970"; transcript_id "Novel_001172"; exon_number "1"; gene_type "
At_chr6	Cufflinks	exon	44996031	44996124	.	-	.	gene_id "XLOC_086970"; transcript_id "Novel_001172"; exon_number "2"; gene_type "
At_chr6	Cufflinks	exon	44996644	44997522	.	-	.	gene_id "XLOC_086970"; transcript_id "Novel_001172"; exon_number "3"; gene_type "
At_chr6	Cufflinks	exon	44997602	44997752	.	-	.	gene_id "XLOC_086970"; transcript_id "Novel_001172"; exon_number "4"; gene_type "
At_chr6	Cufflinks	exon	44996544	44996716	.	-	.	gene_id "XLOC_086970"; transcript_id "Novel_001173"; exon_number "1"; gene_type "
At_chr6	Cufflinks	exon	44997305	44997522	.	-	.	gene_id "XLOC_086970"; transcript_id "Novel_001173"; exon_number "2"; gene_type "
At_chr6	Cufflinks	exon	44997602	44997753	.	-	.	gene_id "XLOC_086970"; transcript_id "Novel_001173"; exon_number "3"; gene_type "
At_chr6	Cufflinks	exon	45162325	45164627	.	-	.	gene_id "XLOC_086980"; transcript_id "Novel_001174"; exon_number "1"; gene_type "
At_chr6	Cufflinks	exon	45165306	45165455	.	-	.	gene_id "XLOC_086980"; transcript_id "Novel_001174"; exon_number "2"; gene_type "
At_chr6	Cufflinks	exon	47544694	47545921	.	-	.	gene_id "XLOC_087249"; transcript_id "Novel_001175"; exon_number "1"; gene_type "
At_chr6	Cufflinks	exon	47547299	47547405	.	-	.	gene_id "XLOC_087249"; transcript_id "Novel_001175"; exon_number "2"; gene_type "
At_chr6	Cufflinks	exon	47547487	47547647	.	-	.	gene_id "XLOC_087249"; transcript_id "Novel_001175"; exon_number "3"; gene_type "
At_chr6	Cufflinks	exon	47835488	47835803	.	-	.	gene_id "XLOC_087282"; transcript_id "Novel_001176"; exon_number "1"; gene_type "
At_chr6	Cufflinks	exon	47836224	47837601	.	-	.	gene_id "XLOC_087282"; transcript_id "Novel_001176"; exon_number "2"; gene_type "
At_chr6	Cufflinks	exon	47837823	47838234	.	-	.	gene_id "XLOC_087282"; transcript_id "Novel_001176"; exon_number "3"; gene_type "
At_chr6	Cufflinks	exon	47838490	47838638	.	-	.	gene_id "XLOC_087282"; transcript_id "Novel_001176"; exon_number "4"; gene_type "
At_chr6	Cufflinks	exon	47838738	47838768	.	-	.	gene_id "XLOC_087282"; transcript_id "Novel_001176"; exon_number "5"; gene_type "
At_chr6	Cufflinks	exon	47838894	47838975	.	-	.	gene_id "XLOC_087282"; transcript_id "Novel_001176"; exon_number "6"; gene_type "
At_chr6	Cufflinks	exon	48145230	48145235	.	-	.	gene_id "XLOC_087321"; transcript_id "Novel_001177"; exon_number "1"; gene_type "
At_chr6	Cufflinks	exon	48145336	48145406	.	-	.	gene_id "XLOC_087321"; transcript_id "Novel_001177"; exon_number "2"; gene_type "
At_chr6	Cufflinks	exon	48146291	48146553	.	-	.	gene_id "XLOC_087321"; transcript_id "Novel_001177"; exon_number "3"; gene_type "
At_chr6	Cufflinks	exon	48253903	48255031	.	-	.	gene_id "XLOC_087361"; transcript_id "Novel_001178"; exon_number "1"; gene_type "
At_chr6	Cufflinks	exon	48255394	48255985	.	-	.	gene_id "XLOC_087361"; transcript_id "Novel_001178"; exon_number "2"; gene_type "
At_chr6	Cufflinks	exon	48253903	48255031	.	-	.	gene_id "XLOC_087361"; transcript_id "Novel_001179"; exon_number "1"; gene_type "
At_chr6	Cufflinks	exon	48255174	48255239	.	-	.	gene_id "XLOC_087361"; transcript_id "Novel_001179"; exon_number "2"; gene_type "
At_chr6	Cufflinks	exon	48255394	48255985	.	-	.	gene_id "XLOC_087361"; transcript_id "Novel_001179"; exon_number "3"; gene_type "
At_chr6	Cufflinks	exon	48505606	48505702	.	-	.	gene_id "XLOC_087391"; transcript_id "Novel_001180"; exon_number "1"; gene_type "
At_chr6	Cufflinks	exon	48507001	48507161	.	-	.	gene_id "XLOC_087391"; transcript_id "Novel_001180"; exon_number "2"; gene_type "
At_chr6	Cufflinks	exon	48508554	48508732	.	-	.	gene_id "XLOC_087391"; transcript_id "Novel_001180"; exon_number "3"; gene_type "
At_chr6	Cufflinks	exon	48506176	48507161	.	-	.	gene_id "XLOC_087391"; transcript_id "Novel_001181"; exon_number "1"; gene_type "
At_chr6	Cufflinks	exon	48508554	48508841	.	-	.	gene_id "XLOC_087391"; transcript_id "Novel_001181"; exon_number "2"; gene_type "

At_chr6	Cufflinks	exon	48604831	48605186	.	-	.	gene_id "XLOC_087415"; transcript_id "Novel_001182"; exon_number "1"; gene_type "
At_chr6	Cufflinks	exon	48605317	48605457	.	-	.	gene_id "XLOC_087415"; transcript_id "Novel_001182"; exon_number "2"; gene_type "
At_chr6	Cufflinks	exon	48606392	48606457	.	-	.	gene_id "XLOC_087415"; transcript_id "Novel_001182"; exon_number "3"; gene_type "
At_chr6	Cufflinks	exon	48608999	48609218	.	-	.	gene_id "XLOC_087415"; transcript_id "Novel_001183"; exon_number "1"; gene_type "
At_chr6	Cufflinks	exon	48609326	48609529	.	-	.	gene_id "XLOC_087415"; transcript_id "Novel_001183"; exon_number "2"; gene_type "
At_chr6	Cufflinks	exon	48811448	48814185	.	-	.	gene_id "XLOC_087432"; transcript_id "Novel_001184"; exon_number "1"; gene_type "
At_chr6	Cufflinks	exon	48814278	48814769	.	-	.	gene_id "XLOC_087432"; transcript_id "Novel_001184"; exon_number "2"; gene_type "
At_chr6	Cufflinks	exon	49728333	49729197	.	-	.	gene_id "XLOC_087552"; transcript_id "Novel_001185"; exon_number "1"; gene_type "
At_chr6	Cufflinks	exon	49729655	49729736	.	-	.	gene_id "XLOC_087552"; transcript_id "Novel_001185"; exon_number "2"; gene_type "
At_chr6	Cufflinks	exon	49729809	49729856	.	-	.	gene_id "XLOC_087552"; transcript_id "Novel_001185"; exon_number "3"; gene_type "
At_chr6	Cufflinks	exon	49730147	49730289	.	-	.	gene_id "XLOC_087552"; transcript_id "Novel_001185"; exon_number "4"; gene_type "
At_chr6	Cufflinks	exon	49730489	49731462	.	-	.	gene_id "XLOC_087552"; transcript_id "Novel_001185"; exon_number "5"; gene_type "
At_chr6	Cufflinks	exon	49731585	49731627	.	-	.	gene_id "XLOC_087552"; transcript_id "Novel_001185"; exon_number "6"; gene_type "
At_chr6	Cufflinks	exon	49731961	49732069	.	-	.	gene_id "XLOC_087552"; transcript_id "Novel_001185"; exon_number "7"; gene_type "
At_chr6	Cufflinks	exon	49732146	49732289	.	-	.	gene_id "XLOC_087552"; transcript_id "Novel_001185"; exon_number "8"; gene_type "
At_chr6	Cufflinks	exon	49728334	49729197	.	-	.	gene_id "XLOC_087552"; transcript_id "Novel_001186"; exon_number "1"; gene_type "
At_chr6	Cufflinks	exon	49729655	49729736	.	-	.	gene_id "XLOC_087552"; transcript_id "Novel_001186"; exon_number "2"; gene_type "
At_chr6	Cufflinks	exon	49729809	49729856	.	-	.	gene_id "XLOC_087552"; transcript_id "Novel_001186"; exon_number "3"; gene_type "
At_chr6	Cufflinks	exon	49730147	49730289	.	-	.	gene_id "XLOC_087552"; transcript_id "Novel_001186"; exon_number "4"; gene_type "
At_chr6	Cufflinks	exon	49730489	49730577	.	-	.	gene_id "XLOC_087552"; transcript_id "Novel_001186"; exon_number "5"; gene_type "
At_chr6	Cufflinks	exon	49731397	49731462	.	-	.	gene_id "XLOC_087552"; transcript_id "Novel_001186"; exon_number "6"; gene_type "
At_chr6	Cufflinks	exon	49731585	49731627	.	-	.	gene_id "XLOC_087552"; transcript_id "Novel_001186"; exon_number "7"; gene_type "
At_chr6	Cufflinks	exon	49731961	49732069	.	-	.	gene_id "XLOC_087552"; transcript_id "Novel_001186"; exon_number "8"; gene_type "
At_chr6	Cufflinks	exon	49732146	49732307	.	-	.	gene_id "XLOC_087552"; transcript_id "Novel_001186"; exon_number "9"; gene_type "
At_chr6	Cufflinks	exon	49730012	49730289	.	-	.	gene_id "XLOC_087552"; transcript_id "Novel_001187"; exon_number "1"; gene_type "
At_chr6	Cufflinks	exon	49730489	49730577	.	-	.	gene_id "XLOC_087552"; transcript_id "Novel_001187"; exon_number "2"; gene_type "
At_chr6	Cufflinks	exon	49731103	49731462	.	-	.	gene_id "XLOC_087552"; transcript_id "Novel_001187"; exon_number "3"; gene_type "
At_chr6	Cufflinks	exon	49731585	49731627	.	-	.	gene_id "XLOC_087552"; transcript_id "Novel_001187"; exon_number "4"; gene_type "
At_chr6	Cufflinks	exon	49731961	49732069	.	-	.	gene_id "XLOC_087552"; transcript_id "Novel_001187"; exon_number "5"; gene_type "
At_chr6	Cufflinks	exon	49732146	49732318	.	-	.	gene_id "XLOC_087552"; transcript_id "Novel_001187"; exon_number "6"; gene_type "
At_chr6	Cufflinks	exon	52229535	52229736	.	-	.	gene_id "XLOC_087838"; transcript_id "Novel_001188"; exon_number "1"; gene_type "
At_chr6	Cufflinks	exon	52405909	52406627	.	-	.	gene_id "XLOC_087858"; transcript_id "Novel_001189"; exon_number "1"; gene_type "
At_chr6	Cufflinks	exon	52406704	52407070	.	-	.	gene_id "XLOC_087858"; transcript_id "Novel_001189"; exon_number "2"; gene_type "
At_chr6	Cufflinks	exon	52407805	52408091	.	-	.	gene_id "XLOC_087858"; transcript_id "Novel_001189"; exon_number "3"; gene_type "
At_chr6	Cufflinks	exon	53497909	53498257	.	-	.	gene_id "XLOC_087975"; transcript_id "Novel_001190"; exon_number "1"; gene_type "

At_chr6	Cufflinks	exon	53510903	53511028	.	-	.	gene_id "XLOC_087975"; transcript_id "Novel_001190"; exon_number "2"; gene_type "
At_chr6	Cufflinks	exon	54355473	54355810	.	-	.	gene_id "XLOC_088094"; transcript_id "Novel_001191"; exon_number "1"; gene_type "
At_chr6	Cufflinks	exon	54356648	54356712	.	-	.	gene_id "XLOC_088094"; transcript_id "Novel_001191"; exon_number "2"; gene_type "
At_chr6	Cufflinks	exon	54439485	54440018	.	-	.	gene_id "XLOC_088114"; transcript_id "Novel_001192"; exon_number "1"; gene_type "
At_chr6	Cufflinks	exon	54440109	54440382	.	-	.	gene_id "XLOC_088114"; transcript_id "Novel_001192"; exon_number "2"; gene_type "
At_chr6	Cufflinks	exon	54544674	54544878	.	-	.	gene_id "XLOC_088133"; transcript_id "Novel_001193"; exon_number "1"; gene_type "
At_chr6	Cufflinks	exon	54545005	54545074	.	-	.	gene_id "XLOC_088133"; transcript_id "Novel_001193"; exon_number "2"; gene_type "
At_chr6	Cufflinks	exon	55290219	55291919	.	-	.	gene_id "XLOC_088208"; transcript_id "Novel_001194"; exon_number "1"; gene_type "
At_chr6	Cufflinks	exon	55292437	55292584	.	-	.	gene_id "XLOC_088208"; transcript_id "Novel_001194"; exon_number "2"; gene_type "
At_chr6	Cufflinks	exon	55290478	55291334	.	-	.	gene_id "XLOC_088208"; transcript_id "Novel_001195"; exon_number "1"; gene_type "
At_chr6	Cufflinks	exon	55291460	55291919	.	-	.	gene_id "XLOC_088208"; transcript_id "Novel_001195"; exon_number "2"; gene_type "
At_chr6	Cufflinks	exon	55292437	55292561	.	-	.	gene_id "XLOC_088208"; transcript_id "Novel_001195"; exon_number "3"; gene_type "
At_chr6	Cufflinks	exon	55343952	55344518	.	-	.	gene_id "XLOC_088217"; transcript_id "Novel_001196"; exon_number "1"; gene_type "
At_chr6	Cufflinks	exon	55344600	55345907	.	-	.	gene_id "XLOC_088217"; transcript_id "Novel_001196"; exon_number "2"; gene_type "
At_chr6	Cufflinks	exon	55343960	55344518	.	-	.	gene_id "XLOC_088217"; transcript_id "Novel_001197"; exon_number "1"; gene_type "
At_chr6	Cufflinks	exon	55344600	55344954	.	-	.	gene_id "XLOC_088217"; transcript_id "Novel_001197"; exon_number "2"; gene_type "
At_chr6	Cufflinks	exon	55345390	55345921	.	-	.	gene_id "XLOC_088217"; transcript_id "Novel_001197"; exon_number "3"; gene_type "
At_chr6	Cufflinks	exon	55771170	55771233	.	-	.	gene_id "XLOC_088280"; transcript_id "Novel_001198"; exon_number "1"; gene_type "
At_chr6	Cufflinks	exon	55771321	55771612	.	-	.	gene_id "XLOC_088280"; transcript_id "Novel_001198"; exon_number "2"; gene_type "
At_chr7	Cufflinks	exon	1830891	1831148	.	+	.	gene_id "XLOC_088677"; transcript_id "Novel_001199"; exon_number "1"; gene_type "
At_chr7	Cufflinks	exon	1831242	1831292	.	+	.	gene_id "XLOC_088677"; transcript_id "Novel_001199"; exon_number "2"; gene_type "
At_chr7	Cufflinks	exon	1831869	1832473	.	+	.	gene_id "XLOC_088677"; transcript_id "Novel_001199"; exon_number "3"; gene_type "
At_chr7	Cufflinks	exon	1894891	1895667	.	+	.	gene_id "XLOC_088680"; transcript_id "Novel_001200"; exon_number "1"; gene_type "
At_chr7	Cufflinks	exon	1895944	1896004	.	+	.	gene_id "XLOC_088680"; transcript_id "Novel_001200"; exon_number "2"; gene_type "
At_chr7	Cufflinks	exon	1896117	1896215	.	+	.	gene_id "XLOC_088680"; transcript_id "Novel_001200"; exon_number "3"; gene_type "
At_chr7	Cufflinks	exon	1896672	1896773	.	+	.	gene_id "XLOC_088680"; transcript_id "Novel_001200"; exon_number "4"; gene_type "
At_chr7	Cufflinks	exon	1896863	1896904	.	+	.	gene_id "XLOC_088680"; transcript_id "Novel_001200"; exon_number "5"; gene_type "
At_chr7	Cufflinks	exon	1897356	1897462	.	+	.	gene_id "XLOC_088680"; transcript_id "Novel_001200"; exon_number "6"; gene_type "
At_chr7	Cufflinks	exon	1897707	1898193	.	+	.	gene_id "XLOC_088680"; transcript_id "Novel_001200"; exon_number "7"; gene_type "
At_chr7	Cufflinks	exon	1896939	1897462	.	+	.	gene_id "XLOC_088680"; transcript_id "Novel_001201"; exon_number "1"; gene_type "
At_chr7	Cufflinks	exon	1897707	1898047	.	+	.	gene_id "XLOC_088680"; transcript_id "Novel_001201"; exon_number "2"; gene_type "
At_chr7	Cufflinks	exon	2178119	2178458	.	+	.	gene_id "XLOC_088719"; transcript_id "Novel_001202"; exon_number "1"; gene_type "
At_chr7	Cufflinks	exon	2178741	2178888	.	+	.	gene_id "XLOC_088719"; transcript_id "Novel_001202"; exon_number "2"; gene_type "
At_chr7	Cufflinks	exon	2179655	2179722	.	+	.	gene_id "XLOC_088719"; transcript_id "Novel_001202"; exon_number "3"; gene_type "
At_chr7	Cufflinks	exon	2532879	2533016	.	+	.	gene_id "XLOC_088768"; transcript_id "Novel_001203"; exon_number "1"; gene_type "

At_chr7	Cufflinks	exon	2533959	2534247	.	+	.	gene_id "XLOC_088768"; transcript_id "Novel_001203"; exon_number "2"; gene_type "
At_chr7	Cufflinks	exon	3935417	3935787	.	+	.	gene_id "XLOC_088909"; transcript_id "Novel_001204"; exon_number "1"; gene_type "
At_chr7	Cufflinks	exon	3935897	3935965	.	+	.	gene_id "XLOC_088909"; transcript_id "Novel_001204"; exon_number "2"; gene_type "
At_chr7	Cufflinks	exon	3936066	3936129	.	+	.	gene_id "XLOC_088909"; transcript_id "Novel_001204"; exon_number "3"; gene_type "
At_chr7	Cufflinks	exon	3936258	3936337	.	+	.	gene_id "XLOC_088909"; transcript_id "Novel_001204"; exon_number "4"; gene_type "
At_chr7	Cufflinks	exon	3936491	3936574	.	+	.	gene_id "XLOC_088909"; transcript_id "Novel_001204"; exon_number "5"; gene_type "
At_chr7	Cufflinks	exon	3936768	3936852	.	+	.	gene_id "XLOC_088909"; transcript_id "Novel_001204"; exon_number "6"; gene_type "
At_chr7	Cufflinks	exon	3937008	3937093	.	+	.	gene_id "XLOC_088909"; transcript_id "Novel_001204"; exon_number "7"; gene_type "
At_chr7	Cufflinks	exon	3937193	3937314	.	+	.	gene_id "XLOC_088909"; transcript_id "Novel_001204"; exon_number "8"; gene_type "
At_chr7	Cufflinks	exon	3937460	3937520	.	+	.	gene_id "XLOC_088909"; transcript_id "Novel_001204"; exon_number "9"; gene_type "
At_chr7	Cufflinks	exon	3937916	3938014	.	+	.	gene_id "XLOC_088909"; transcript_id "Novel_001204"; exon_number "10"; gene_type
At_chr7	Cufflinks	exon	3938098	3938199	.	+	.	gene_id "XLOC_088909"; transcript_id "Novel_001204"; exon_number "11"; gene_type
At_chr7	Cufflinks	exon	3938283	3938324	.	+	.	gene_id "XLOC_088909"; transcript_id "Novel_001204"; exon_number "12"; gene_type
At_chr7	Cufflinks	exon	3938419	3939172	.	+	.	gene_id "XLOC_088909"; transcript_id "Novel_001204"; exon_number "13"; gene_type
At_chr7	Cufflinks	exon	3935420	3935616	.	+	.	gene_id "XLOC_088909"; transcript_id "Novel_001205"; exon_number "1"; gene_type "
At_chr7	Cufflinks	exon	3935707	3935787	.	+	.	gene_id "XLOC_088909"; transcript_id "Novel_001205"; exon_number "2"; gene_type "
At_chr7	Cufflinks	exon	3935897	3935965	.	+	.	gene_id "XLOC_088909"; transcript_id "Novel_001205"; exon_number "3"; gene_type "
At_chr7	Cufflinks	exon	3936066	3936129	.	+	.	gene_id "XLOC_088909"; transcript_id "Novel_001205"; exon_number "4"; gene_type "
At_chr7	Cufflinks	exon	3936258	3936337	.	+	.	gene_id "XLOC_088909"; transcript_id "Novel_001205"; exon_number "5"; gene_type "
At_chr7	Cufflinks	exon	3936491	3936574	.	+	.	gene_id "XLOC_088909"; transcript_id "Novel_001205"; exon_number "6"; gene_type "
At_chr7	Cufflinks	exon	3936768	3936852	.	+	.	gene_id "XLOC_088909"; transcript_id "Novel_001205"; exon_number "7"; gene_type "
At_chr7	Cufflinks	exon	3937008	3937093	.	+	.	gene_id "XLOC_088909"; transcript_id "Novel_001205"; exon_number "8"; gene_type "
At_chr7	Cufflinks	exon	3937193	3937314	.	+	.	gene_id "XLOC_088909"; transcript_id "Novel_001205"; exon_number "9"; gene_type "
At_chr7	Cufflinks	exon	3937460	3937520	.	+	.	gene_id "XLOC_088909"; transcript_id "Novel_001205"; exon_number "10"; gene_type
At_chr7	Cufflinks	exon	3937916	3938014	.	+	.	gene_id "XLOC_088909"; transcript_id "Novel_001205"; exon_number "11"; gene_type
At_chr7	Cufflinks	exon	3938098	3938199	.	+	.	gene_id "XLOC_088909"; transcript_id "Novel_001205"; exon_number "12"; gene_type
At_chr7	Cufflinks	exon	3938283	3938324	.	+	.	gene_id "XLOC_088909"; transcript_id "Novel_001205"; exon_number "13"; gene_type
At_chr7	Cufflinks	exon	3938419	3939303	.	+	.	gene_id "XLOC_088909"; transcript_id "Novel_001205"; exon_number "14"; gene_type
At_chr7	Cufflinks	exon	4395146	4395694	.	+	.	gene_id "XLOC_089022"; transcript_id "Novel_001206"; exon_number "1"; gene_type "
At_chr7	Cufflinks	exon	4396513	4396582	.	+	.	gene_id "XLOC_089022"; transcript_id "Novel_001206"; exon_number "2"; gene_type "
At_chr7	Cufflinks	exon	4396703	4396875	.	+	.	gene_id "XLOC_089022"; transcript_id "Novel_001206"; exon_number "3"; gene_type "
At_chr7	Cufflinks	exon	4397308	4397482	.	+	.	gene_id "XLOC_089022"; transcript_id "Novel_001206"; exon_number "4"; gene_type "
At_chr7	Cufflinks	exon	4397838	4398004	.	+	.	gene_id "XLOC_089022"; transcript_id "Novel_001206"; exon_number "5"; gene_type "
At_chr7	Cufflinks	exon	4398122	4398341	.	+	.	gene_id "XLOC_089022"; transcript_id "Novel_001206"; exon_number "6"; gene_type "
At_chr7	Cufflinks	exon	4398438	4398542	.	+	.	gene_id "XLOC_089022"; transcript_id "Novel_001206"; exon_number "7"; gene_type "

At_chr7	Cufflinks	exon	4398810	4398962	.	+	.	gene_id "XLOC_089022"; transcript_id "Novel_001206"; exon_number "8"; gene_type "
At_chr7	Cufflinks	exon	4399089	4399755	.	+	.	gene_id "XLOC_089022"; transcript_id "Novel_001206"; exon_number "9"; gene_type "
At_chr7	Cufflinks	exon	4451692	4452055	.	+	.	gene_id "XLOC_089032"; transcript_id "Novel_001207"; exon_number "1"; gene_type "
At_chr7	Cufflinks	exon	4452175	4452253	.	+	.	gene_id "XLOC_089032"; transcript_id "Novel_001207"; exon_number "2"; gene_type "
At_chr7	Cufflinks	exon	4452356	4452481	.	+	.	gene_id "XLOC_089032"; transcript_id "Novel_001207"; exon_number "3"; gene_type "
At_chr7	Cufflinks	exon	4594304	4595131	.	+	.	gene_id "XLOC_089060"; transcript_id "Novel_001208"; exon_number "1"; gene_type "
At_chr7	Cufflinks	exon	4595239	4595495	.	+	.	gene_id "XLOC_089060"; transcript_id "Novel_001208"; exon_number "2"; gene_type "
At_chr7	Cufflinks	exon	4595590	4595736	.	+	.	gene_id "XLOC_089060"; transcript_id "Novel_001208"; exon_number "3"; gene_type "
At_chr7	Cufflinks	exon	4596369	4596969	.	+	.	gene_id "XLOC_089060"; transcript_id "Novel_001208"; exon_number "4"; gene_type "
At_chr7	Cufflinks	exon	4620472	4621120	.	+	.	gene_id "XLOC_089065"; transcript_id "Novel_001209"; exon_number "1"; gene_type "
At_chr7	Cufflinks	exon	4656030	4656144	.	+	.	gene_id "XLOC_089076"; transcript_id "Novel_001210"; exon_number "1"; gene_type "
At_chr7	Cufflinks	exon	4656325	4656419	.	+	.	gene_id "XLOC_089076"; transcript_id "Novel_001210"; exon_number "2"; gene_type "
At_chr7	Cufflinks	exon	4656907	4657012	.	+	.	gene_id "XLOC_089076"; transcript_id "Novel_001210"; exon_number "3"; gene_type "
At_chr7	Cufflinks	exon	4657081	4657130	.	+	.	gene_id "XLOC_089076"; transcript_id "Novel_001210"; exon_number "4"; gene_type "
At_chr7	Cufflinks	exon	4657385	4658084	.	+	.	gene_id "XLOC_089076"; transcript_id "Novel_001210"; exon_number "5"; gene_type "
At_chr7	Cufflinks	exon	4658224	4658270	.	+	.	gene_id "XLOC_089076"; transcript_id "Novel_001211"; exon_number "1"; gene_type "
At_chr7	Cufflinks	exon	4658593	4659715	.	+	.	gene_id "XLOC_089076"; transcript_id "Novel_001211"; exon_number "2"; gene_type "
At_chr7	Cufflinks	exon	4659807	4660733	.	+	.	gene_id "XLOC_089076"; transcript_id "Novel_001211"; exon_number "3"; gene_type "
At_chr7	Cufflinks	exon	4658612	4658655	.	+	.	gene_id "XLOC_089076"; transcript_id "Novel_001212"; exon_number "1"; gene_type "
At_chr7	Cufflinks	exon	4659663	4659715	.	+	.	gene_id "XLOC_089076"; transcript_id "Novel_001212"; exon_number "2"; gene_type "
At_chr7	Cufflinks	exon	4659807	4660438	.	+	.	gene_id "XLOC_089076"; transcript_id "Novel_001212"; exon_number "3"; gene_type "
At_chr7	Cufflinks	exon	4786795	4787620	.	+	.	gene_id "XLOC_089100"; transcript_id "Novel_001213"; exon_number "1"; gene_type "
At_chr7	Cufflinks	exon	4788666	4788783	.	+	.	gene_id "XLOC_089100"; transcript_id "Novel_001213"; exon_number "2"; gene_type "
At_chr7	Cufflinks	exon	4788990	4789219	.	+	.	gene_id "XLOC_089100"; transcript_id "Novel_001213"; exon_number "3"; gene_type "
At_chr7	Cufflinks	exon	4789314	4789553	.	+	.	gene_id "XLOC_089100"; transcript_id "Novel_001213"; exon_number "4"; gene_type "
At_chr7	Cufflinks	exon	4790004	4790157	.	+	.	gene_id "XLOC_089100"; transcript_id "Novel_001213"; exon_number "5"; gene_type "
At_chr7	Cufflinks	exon	4790254	4790339	.	+	.	gene_id "XLOC_089100"; transcript_id "Novel_001213"; exon_number "6"; gene_type "
At_chr7	Cufflinks	exon	4790856	4790970	.	+	.	gene_id "XLOC_089100"; transcript_id "Novel_001213"; exon_number "7"; gene_type "
At_chr7	Cufflinks	exon	4791240	4791312	.	+	.	gene_id "XLOC_089100"; transcript_id "Novel_001213"; exon_number "8"; gene_type "
At_chr7	Cufflinks	exon	4791574	4791884	.	+	.	gene_id "XLOC_089100"; transcript_id "Novel_001213"; exon_number "9"; gene_type "
At_chr7	Cufflinks	exon	4787346	4787620	.	+	.	gene_id "XLOC_089100"; transcript_id "Novel_001214"; exon_number "1"; gene_type "
At_chr7	Cufflinks	exon	4788666	4788783	.	+	.	gene_id "XLOC_089100"; transcript_id "Novel_001214"; exon_number "2"; gene_type "
At_chr7	Cufflinks	exon	4788990	4789219	.	+	.	gene_id "XLOC_089100"; transcript_id "Novel_001214"; exon_number "3"; gene_type "
At_chr7	Cufflinks	exon	4789314	4789553	.	+	.	gene_id "XLOC_089100"; transcript_id "Novel_001214"; exon_number "4"; gene_type "
At_chr7	Cufflinks	exon	4790004	4790157	.	+	.	gene_id "XLOC_089100"; transcript_id "Novel_001214"; exon_number "5"; gene_type "

At_chr7	Cufflinks	exon	4790254	4790339	.	+	.	gene_id "XLOC_089100"; transcript_id "Novel_001214"; exon_number "6"; gene_type "
At_chr7	Cufflinks	exon	4790666	4790763	.	+	.	gene_id "XLOC_089100"; transcript_id "Novel_001214"; exon_number "7"; gene_type "
At_chr7	Cufflinks	exon	4790856	4790970	.	+	.	gene_id "XLOC_089100"; transcript_id "Novel_001214"; exon_number "8"; gene_type "
At_chr7	Cufflinks	exon	4791240	4791312	.	+	.	gene_id "XLOC_089100"; transcript_id "Novel_001214"; exon_number "9"; gene_type "
At_chr7	Cufflinks	exon	4791574	4792087	.	+	.	gene_id "XLOC_089100"; transcript_id "Novel_001214"; exon_number "10"; gene_type
At_chr7	Cufflinks	exon	6477017	6477073	.	+	.	gene_id "XLOC_089401"; transcript_id "Novel_001215"; exon_number "1"; gene_type "
At_chr7	Cufflinks	exon	6478107	6479042	.	+	.	gene_id "XLOC_089401"; transcript_id "Novel_001215"; exon_number "2"; gene_type "
At_chr7	Cufflinks	exon	6973307	6973367	.	+	.	gene_id "XLOC_089489"; transcript_id "Novel_001216"; exon_number "1"; gene_type "
At_chr7	Cufflinks	exon	6974017	6974597	.	+	.	gene_id "XLOC_089489"; transcript_id "Novel_001216"; exon_number "2"; gene_type "
At_chr7	Cufflinks	exon	6974781	6974881	.	+	.	gene_id "XLOC_089489"; transcript_id "Novel_001216"; exon_number "3"; gene_type "
At_chr7	Cufflinks	exon	6975270	6975353	.	+	.	gene_id "XLOC_089489"; transcript_id "Novel_001216"; exon_number "4"; gene_type "
At_chr7	Cufflinks	exon	6975453	6975563	.	+	.	gene_id "XLOC_089489"; transcript_id "Novel_001216"; exon_number "5"; gene_type "
At_chr7	Cufflinks	exon	6975681	6976048	.	+	.	gene_id "XLOC_089489"; transcript_id "Novel_001216"; exon_number "6"; gene_type "
At_chr7	Cufflinks	exon	7201472	7201677	.	+	.	gene_id "XLOC_089526"; transcript_id "Novel_001217"; exon_number "1"; gene_type "
At_chr7	Cufflinks	exon	7201769	7201856	.	+	.	gene_id "XLOC_089526"; transcript_id "Novel_001217"; exon_number "2"; gene_type "
At_chr7	Cufflinks	exon	7201484	7201677	.	+	.	gene_id "XLOC_089526"; transcript_id "Novel_001218"; exon_number "1"; gene_type "
At_chr7	Cufflinks	exon	7201769	7201834	.	+	.	gene_id "XLOC_089526"; transcript_id "Novel_001218"; exon_number "2"; gene_type "
At_chr7	Cufflinks	exon	7201979	7202006	.	+	.	gene_id "XLOC_089526"; transcript_id "Novel_001218"; exon_number "3"; gene_type "
At_chr7	Cufflinks	exon	8627630	8627928	.	+	.	gene_id "XLOC_089767"; transcript_id "Novel_001219"; exon_number "1"; gene_type "
At_chr7	Cufflinks	exon	8628443	8628663	.	+	.	gene_id "XLOC_089767"; transcript_id "Novel_001219"; exon_number "2"; gene_type "
At_chr7	Cufflinks	exon	8628759	8628927	.	+	.	gene_id "XLOC_089767"; transcript_id "Novel_001219"; exon_number "3"; gene_type "
At_chr7	Cufflinks	exon	8629013	8629102	.	+	.	gene_id "XLOC_089767"; transcript_id "Novel_001219"; exon_number "4"; gene_type "
At_chr7	Cufflinks	exon	8629194	8629495	.	+	.	gene_id "XLOC_089767"; transcript_id "Novel_001219"; exon_number "5"; gene_type "
At_chr7	Cufflinks	exon	8629593	8631465	.	+	.	gene_id "XLOC_089767"; transcript_id "Novel_001219"; exon_number "6"; gene_type "
At_chr7	Cufflinks	exon	8627634	8627928	.	+	.	gene_id "XLOC_089767"; transcript_id "Novel_001220"; exon_number "1"; gene_type "
At_chr7	Cufflinks	exon	8628443	8628663	.	+	.	gene_id "XLOC_089767"; transcript_id "Novel_001220"; exon_number "2"; gene_type "
At_chr7	Cufflinks	exon	8628759	8628927	.	+	.	gene_id "XLOC_089767"; transcript_id "Novel_001220"; exon_number "3"; gene_type "
At_chr7	Cufflinks	exon	8629013	8629102	.	+	.	gene_id "XLOC_089767"; transcript_id "Novel_001220"; exon_number "4"; gene_type "
At_chr7	Cufflinks	exon	8629194	8629495	.	+	.	gene_id "XLOC_089767"; transcript_id "Novel_001220"; exon_number "5"; gene_type "
At_chr7	Cufflinks	exon	8629593	8629863	.	+	.	gene_id "XLOC_089767"; transcript_id "Novel_001220"; exon_number "6"; gene_type "
At_chr7	Cufflinks	exon	8630603	8631268	.	+	.	gene_id "XLOC_089767"; transcript_id "Novel_001220"; exon_number "7"; gene_type "
At_chr7	Cufflinks	exon	9101848	9102394	.	+	.	gene_id "XLOC_089841"; transcript_id "Novel_001221"; exon_number "1"; gene_type "
At_chr7	Cufflinks	exon	9102933	9103058	.	+	.	gene_id "XLOC_089841"; transcript_id "Novel_001221"; exon_number "2"; gene_type "
At_chr7	Cufflinks	exon	9103149	9103299	.	+	.	gene_id "XLOC_089841"; transcript_id "Novel_001221"; exon_number "3"; gene_type "
At_chr7	Cufflinks	exon	9103411	9103867	.	+	.	gene_id "XLOC_089841"; transcript_id "Novel_001222"; exon_number "1"; gene_type "

At_chr7	Cufflinks	exon	9104320	9104517	.	+	.	gene_id "XLOC_089841"; transcript_id "Novel_001222"; exon_number "2"; gene_type "
At_chr7	Cufflinks	exon	9103455	9103486	.	+	.	gene_id "XLOC_089841"; transcript_id "Novel_001223"; exon_number "1"; gene_type "
At_chr7	Cufflinks	exon	9103588	9103867	.	+	.	gene_id "XLOC_089841"; transcript_id "Novel_001223"; exon_number "2"; gene_type "
At_chr7	Cufflinks	exon	9104320	9104535	.	+	.	gene_id "XLOC_089841"; transcript_id "Novel_001223"; exon_number "3"; gene_type "
At_chr7	Cufflinks	exon	9410794	9412752	.	+	.	gene_id "XLOC_089912"; transcript_id "Novel_001224"; exon_number "1"; gene_type "
At_chr7	Cufflinks	exon	9413274	9413812	.	+	.	gene_id "XLOC_089912"; transcript_id "Novel_001224"; exon_number "2"; gene_type "
At_chr7	Cufflinks	exon	9569919	9570939	.	+	.	gene_id "XLOC_089930"; transcript_id "Novel_001225"; exon_number "1"; gene_type "
At_chr7	Cufflinks	exon	9571088	9571168	.	+	.	gene_id "XLOC_089930"; transcript_id "Novel_001225"; exon_number "2"; gene_type "
At_chr7	Cufflinks	exon	9571339	9571445	.	+	.	gene_id "XLOC_089930"; transcript_id "Novel_001225"; exon_number "3"; gene_type "
At_chr7	Cufflinks	exon	9571895	9572279	.	+	.	gene_id "XLOC_089930"; transcript_id "Novel_001225"; exon_number "4"; gene_type "
At_chr7	Cufflinks	exon	11269554	11269855	.	+	.	gene_id "XLOC_090147"; transcript_id "Novel_001226"; exon_number "1"; gene_type "
At_chr7	Cufflinks	exon	11271159	11272246	.	+	.	gene_id "XLOC_090147"; transcript_id "Novel_001226"; exon_number "2"; gene_type "
At_chr7	Cufflinks	exon	12040284	12040956	.	+	.	gene_id "XLOC_090264"; transcript_id "Novel_001227"; exon_number "1"; gene_type "
At_chr7	Cufflinks	exon	12041550	12041634	.	+	.	gene_id "XLOC_090264"; transcript_id "Novel_001227"; exon_number "2"; gene_type "
At_chr7	Cufflinks	exon	12041919	12042091	.	+	.	gene_id "XLOC_090264"; transcript_id "Novel_001227"; exon_number "3"; gene_type "
At_chr7	Cufflinks	exon	12042211	12042286	.	+	.	gene_id "XLOC_090264"; transcript_id "Novel_001227"; exon_number "4"; gene_type "
At_chr7	Cufflinks	exon	12042391	12042453	.	+	.	gene_id "XLOC_090264"; transcript_id "Novel_001227"; exon_number "5"; gene_type "
At_chr7	Cufflinks	exon	12042586	12042841	.	+	.	gene_id "XLOC_090264"; transcript_id "Novel_001227"; exon_number "6"; gene_type "
At_chr7	Cufflinks	exon	12040319	12040956	.	+	.	gene_id "XLOC_090264"; transcript_id "Novel_001228"; exon_number "1"; gene_type "
At_chr7	Cufflinks	exon	12041550	12041634	.	+	.	gene_id "XLOC_090264"; transcript_id "Novel_001228"; exon_number "2"; gene_type "
At_chr7	Cufflinks	exon	12041919	12042091	.	+	.	gene_id "XLOC_090264"; transcript_id "Novel_001228"; exon_number "3"; gene_type "
At_chr7	Cufflinks	exon	12042211	12042310	.	+	.	gene_id "XLOC_090264"; transcript_id "Novel_001228"; exon_number "4"; gene_type "
At_chr7	Cufflinks	exon	12042391	12042453	.	+	.	gene_id "XLOC_090264"; transcript_id "Novel_001228"; exon_number "5"; gene_type "
At_chr7	Cufflinks	exon	12042586	12042586	.	+	.	gene_id "XLOC_090264"; transcript_id "Novel_001228"; exon_number "6"; gene_type "
At_chr7	Cufflinks	exon	12040335	12040956	.	+	.	gene_id "XLOC_090264"; transcript_id "Novel_001229"; exon_number "1"; gene_type "
At_chr7	Cufflinks	exon	12041550	12041634	.	+	.	gene_id "XLOC_090264"; transcript_id "Novel_001229"; exon_number "2"; gene_type "
At_chr7	Cufflinks	exon	12041919	12042091	.	+	.	gene_id "XLOC_090264"; transcript_id "Novel_001229"; exon_number "3"; gene_type "
At_chr7	Cufflinks	exon	12042211	12042725	.	+	.	gene_id "XLOC_090264"; transcript_id "Novel_001229"; exon_number "4"; gene_type "
At_chr7	Cufflinks	exon	12040397	12040956	.	+	.	gene_id "XLOC_090264"; transcript_id "Novel_001230"; exon_number "1"; gene_type "
At_chr7	Cufflinks	exon	12041550	12041634	.	+	.	gene_id "XLOC_090264"; transcript_id "Novel_001230"; exon_number "2"; gene_type "
At_chr7	Cufflinks	exon	12041919	12042091	.	+	.	gene_id "XLOC_090264"; transcript_id "Novel_001230"; exon_number "3"; gene_type "
At_chr7	Cufflinks	exon	12042211	12042286	.	+	.	gene_id "XLOC_090264"; transcript_id "Novel_001230"; exon_number "4"; gene_type "
At_chr7	Cufflinks	exon	12042391	12042703	.	+	.	gene_id "XLOC_090264"; transcript_id "Novel_001230"; exon_number "5"; gene_type "
At_chr7	Cufflinks	exon	14182751	14182866	.	+	.	gene_id "XLOC_090434"; transcript_id "Novel_001231"; exon_number "1"; gene_type "
At_chr7	Cufflinks	exon	14183114	14183177	.	+	.	gene_id "XLOC_090434"; transcript_id "Novel_001231"; exon_number "2"; gene_type "

At_chr7	Cufflinks	exon	14183651	14183715	.	+	.	gene_id "XLOC_090434"; transcript_id "Novel_001231"; exon_number "3"; gene_type "
At_chr7	Cufflinks	exon	14183949	14186343	.	+	.	gene_id "XLOC_090434"; transcript_id "Novel_001231"; exon_number "4"; gene_type "
At_chr7	Cufflinks	exon	14182775	14182866	.	+	.	gene_id "XLOC_090434"; transcript_id "Novel_001232"; exon_number "1"; gene_type "
At_chr7	Cufflinks	exon	14183117	14183177	.	+	.	gene_id "XLOC_090434"; transcript_id "Novel_001232"; exon_number "2"; gene_type "
At_chr7	Cufflinks	exon	14183651	14183715	.	+	.	gene_id "XLOC_090434"; transcript_id "Novel_001232"; exon_number "3"; gene_type "
At_chr7	Cufflinks	exon	14183949	14184041	.	+	.	gene_id "XLOC_090434"; transcript_id "Novel_001232"; exon_number "4"; gene_type "
At_chr7	Cufflinks	exon	14184665	14184791	.	+	.	gene_id "XLOC_090434"; transcript_id "Novel_001232"; exon_number "5"; gene_type "
At_chr7	Cufflinks	exon	14185232	14186783	.	+	.	gene_id "XLOC_090434"; transcript_id "Novel_001232"; exon_number "6"; gene_type "
At_chr7	Cufflinks	exon	16126024	16127026	.	+	.	gene_id "XLOC_090495"; transcript_id "Novel_001233"; exon_number "1"; gene_type "
At_chr7	Cufflinks	exon	16127347	16131471	.	+	.	gene_id "XLOC_090495"; transcript_id "Novel_001233"; exon_number "2"; gene_type "
At_chr7	Cufflinks	exon	16851500	16851794	.	+	.	gene_id "XLOC_090550"; transcript_id "Novel_001234"; exon_number "1"; gene_type "
At_chr7	Cufflinks	exon	16852336	16852490	.	+	.	gene_id "XLOC_090550"; transcript_id "Novel_001234"; exon_number "2"; gene_type "
At_chr7	Cufflinks	exon	16853371	16853883	.	+	.	gene_id "XLOC_090550"; transcript_id "Novel_001234"; exon_number "3"; gene_type "
At_chr7	Cufflinks	exon	28535667	28538455	.	+	.	gene_id "XLOC_090971"; transcript_id "Novel_001235"; exon_number "1"; gene_type "
At_chr7	Cufflinks	exon	29066333	29066436	.	+	.	gene_id "XLOC_091046"; transcript_id "Novel_001236"; exon_number "1"; gene_type "
At_chr7	Cufflinks	exon	29066529	29066610	.	+	.	gene_id "XLOC_091046"; transcript_id "Novel_001236"; exon_number "2"; gene_type "
At_chr7	Cufflinks	exon	29066766	29066900	.	+	.	gene_id "XLOC_091046"; transcript_id "Novel_001236"; exon_number "3"; gene_type "
At_chr7	Cufflinks	exon	29066360	29066436	.	+	.	gene_id "XLOC_091046"; transcript_id "Novel_001237"; exon_number "1"; gene_type "
At_chr7	Cufflinks	exon	29066529	29066610	.	+	.	gene_id "XLOC_091046"; transcript_id "Novel_001237"; exon_number "2"; gene_type "
At_chr7	Cufflinks	exon	29066766	29066885	.	+	.	gene_id "XLOC_091046"; transcript_id "Novel_001237"; exon_number "3"; gene_type "
At_chr7	Cufflinks	exon	29066961	29067039	.	+	.	gene_id "XLOC_091046"; transcript_id "Novel_001237"; exon_number "4"; gene_type "
At_chr7	Cufflinks	exon	29067708	29067794	.	+	.	gene_id "XLOC_091046"; transcript_id "Novel_001237"; exon_number "5"; gene_type "
At_chr7	Cufflinks	exon	29068948	29069010	.	+	.	gene_id "XLOC_091046"; transcript_id "Novel_001237"; exon_number "6"; gene_type "
At_chr7	Cufflinks	exon	29069410	29069546	.	+	.	gene_id "XLOC_091046"; transcript_id "Novel_001237"; exon_number "7"; gene_type "
At_chr7	Cufflinks	exon	29070630	29070806	.	+	.	gene_id "XLOC_091050"; transcript_id "Novel_001238"; exon_number "1"; gene_type "
At_chr7	Cufflinks	exon	29070897	29071550	.	+	.	gene_id "XLOC_091050"; transcript_id "Novel_001238"; exon_number "2"; gene_type "
At_chr7	Cufflinks	exon	29072079	29072152	.	+	.	gene_id "XLOC_091050"; transcript_id "Novel_001238"; exon_number "3"; gene_type "
At_chr7	Cufflinks	exon	29072247	29072338	.	+	.	gene_id "XLOC_091050"; transcript_id "Novel_001238"; exon_number "4"; gene_type "
At_chr7	Cufflinks	exon	29073375	29073444	.	+	.	gene_id "XLOC_091050"; transcript_id "Novel_001238"; exon_number "5"; gene_type "
At_chr7	Cufflinks	exon	29074733	29074823	.	+	.	gene_id "XLOC_091050"; transcript_id "Novel_001238"; exon_number "6"; gene_type "
At_chr7	Cufflinks	exon	29074917	29074967	.	+	.	gene_id "XLOC_091050"; transcript_id "Novel_001238"; exon_number "7"; gene_type "
At_chr7	Cufflinks	exon	29075544	29076796	.	+	.	gene_id "XLOC_091050"; transcript_id "Novel_001238"; exon_number "8"; gene_type "
At_chr7	Cufflinks	exon	29070679	29071550	.	+	.	gene_id "XLOC_091050"; transcript_id "Novel_001239"; exon_number "1"; gene_type "
At_chr7	Cufflinks	exon	29072079	29072152	.	+	.	gene_id "XLOC_091050"; transcript_id "Novel_001239"; exon_number "2"; gene_type "
At_chr7	Cufflinks	exon	29072247	29072338	.	+	.	gene_id "XLOC_091050"; transcript_id "Novel_001239"; exon_number "3"; gene_type "

At_chr7	Cufflinks	exon	29073375	29073444	.	+	.	gene_id "XLOC_091050"; transcript_id "Novel_001239"; exon_number "4"; gene_type "
At_chr7	Cufflinks	exon	29074733	29074823	.	+	.	gene_id "XLOC_091050"; transcript_id "Novel_001239"; exon_number "5"; gene_type "
At_chr7	Cufflinks	exon	29074917	29074967	.	+	.	gene_id "XLOC_091050"; transcript_id "Novel_001239"; exon_number "6"; gene_type "
At_chr7	Cufflinks	exon	29075544	29076543	.	+	.	gene_id "XLOC_091050"; transcript_id "Novel_001239"; exon_number "7"; gene_type "
At_chr7	Cufflinks	exon	29074083	29074967	.	+	.	gene_id "XLOC_091050"; transcript_id "Novel_001240"; exon_number "1"; gene_type "
At_chr7	Cufflinks	exon	29075544	29076386	.	+	.	gene_id "XLOC_091050"; transcript_id "Novel_001240"; exon_number "2"; gene_type "
At_chr7	Cufflinks	exon	31638901	31639543	.	+	.	gene_id "XLOC_091279"; transcript_id "Novel_001241"; exon_number "1"; gene_type "
At_chr7	Cufflinks	exon	31640248	31640692	.	+	.	gene_id "XLOC_091279"; transcript_id "Novel_001241"; exon_number "2"; gene_type "
At_chr7	Cufflinks	exon	31640773	31642447	.	+	.	gene_id "XLOC_091279"; transcript_id "Novel_001242"; exon_number "1"; gene_type "
At_chr7	Cufflinks	exon	31642559	31643596	.	+	.	gene_id "XLOC_091279"; transcript_id "Novel_001242"; exon_number "2"; gene_type "
At_chr7	Cufflinks	exon	1942533	1943878	.	-	.	gene_id "XLOC_091584"; transcript_id "Novel_001243"; exon_number "1"; gene_type "
At_chr7	Cufflinks	exon	1944143	1945002	.	-	.	gene_id "XLOC_091584"; transcript_id "Novel_001243"; exon_number "2"; gene_type "
At_chr7	Cufflinks	exon	2212629	2212933	.	-	.	gene_id "XLOC_091609"; transcript_id "Novel_001244"; exon_number "1"; gene_type "
At_chr7	Cufflinks	exon	2214798	2215046	.	-	.	gene_id "XLOC_091609"; transcript_id "Novel_001244"; exon_number "2"; gene_type "
At_chr7	Cufflinks	exon	2215147	2215179	.	-	.	gene_id "XLOC_091609"; transcript_id "Novel_001244"; exon_number "3"; gene_type "
At_chr7	Cufflinks	exon	2215651	2215835	.	-	.	gene_id "XLOC_091609"; transcript_id "Novel_001244"; exon_number "4"; gene_type "
At_chr7	Cufflinks	exon	2223605	2223834	.	-	.	gene_id "XLOC_091614"; transcript_id "Novel_001245"; exon_number "1"; gene_type "
At_chr7	Cufflinks	exon	2223935	2223983	.	-	.	gene_id "XLOC_091614"; transcript_id "Novel_001245"; exon_number "2"; gene_type "
At_chr7	Cufflinks	exon	2224164	2224365	.	-	.	gene_id "XLOC_091614"; transcript_id "Novel_001245"; exon_number "3"; gene_type "
At_chr7	Cufflinks	exon	4252482	4252686	.	-	.	gene_id "XLOC_091892"; transcript_id "Novel_001246"; exon_number "1"; gene_type "
At_chr7	Cufflinks	exon	4500396	4502277	.	-	.	gene_id "XLOC_091924"; transcript_id "Novel_001247"; exon_number "1"; gene_type "
At_chr7	Cufflinks	exon	4581122	4581289	.	-	.	gene_id "XLOC_091935"; transcript_id "Novel_001248"; exon_number "1"; gene_type "
At_chr7	Cufflinks	exon	4581389	4581458	.	-	.	gene_id "XLOC_091935"; transcript_id "Novel_001248"; exon_number "2"; gene_type "
At_chr7	Cufflinks	exon	4581545	4581638	.	-	.	gene_id "XLOC_091935"; transcript_id "Novel_001248"; exon_number "3"; gene_type "
At_chr7	Cufflinks	exon	4581952	4582052	.	-	.	gene_id "XLOC_091935"; transcript_id "Novel_001248"; exon_number "4"; gene_type "
At_chr7	Cufflinks	exon	4582248	4582316	.	-	.	gene_id "XLOC_091935"; transcript_id "Novel_001248"; exon_number "5"; gene_type "
At_chr7	Cufflinks	exon	4582398	4583146	.	-	.	gene_id "XLOC_091935"; transcript_id "Novel_001248"; exon_number "6"; gene_type "
At_chr7	Cufflinks	exon	4581122	4581289	.	-	.	gene_id "XLOC_091935"; transcript_id "Novel_001249"; exon_number "1"; gene_type "
At_chr7	Cufflinks	exon	4581389	4581458	.	-	.	gene_id "XLOC_091935"; transcript_id "Novel_001249"; exon_number "2"; gene_type "
At_chr7	Cufflinks	exon	4581545	4581638	.	-	.	gene_id "XLOC_091935"; transcript_id "Novel_001249"; exon_number "3"; gene_type "
At_chr7	Cufflinks	exon	4581952	4582052	.	-	.	gene_id "XLOC_091935"; transcript_id "Novel_001249"; exon_number "4"; gene_type "
At_chr7	Cufflinks	exon	4582248	4582316	.	-	.	gene_id "XLOC_091935"; transcript_id "Novel_001249"; exon_number "5"; gene_type "
At_chr7	Cufflinks	exon	4582398	4582612	.	-	.	gene_id "XLOC_091935"; transcript_id "Novel_001249"; exon_number "6"; gene_type "
At_chr7	Cufflinks	exon	4583174	4583222	.	-	.	gene_id "XLOC_091935"; transcript_id "Novel_001249"; exon_number "7"; gene_type "
At_chr7	Cufflinks	exon	4583435	4583566	.	-	.	gene_id "XLOC_091935"; transcript_id "Novel_001249"; exon_number "8"; gene_type "

At_chr7	Cufflinks	exon	4583902	4584499	.	-	.	gene_id "XLOC_091935"; transcript_id "Novel_001249"; exon_number "9"; gene_type "
At_chr7	Cufflinks	exon	4581125	4581638	.	-	.	gene_id "XLOC_091935"; transcript_id "Novel_001250"; exon_number "1"; gene_type "
At_chr7	Cufflinks	exon	4581952	4582052	.	-	.	gene_id "XLOC_091935"; transcript_id "Novel_001250"; exon_number "2"; gene_type "
At_chr7	Cufflinks	exon	4582248	4582316	.	-	.	gene_id "XLOC_091935"; transcript_id "Novel_001250"; exon_number "3"; gene_type "
At_chr7	Cufflinks	exon	4582398	4582612	.	-	.	gene_id "XLOC_091935"; transcript_id "Novel_001250"; exon_number "4"; gene_type "
At_chr7	Cufflinks	exon	4583174	4583222	.	-	.	gene_id "XLOC_091935"; transcript_id "Novel_001250"; exon_number "5"; gene_type "
At_chr7	Cufflinks	exon	4583435	4583566	.	-	.	gene_id "XLOC_091935"; transcript_id "Novel_001250"; exon_number "6"; gene_type "
At_chr7	Cufflinks	exon	4583902	4584479	.	-	.	gene_id "XLOC_091935"; transcript_id "Novel_001250"; exon_number "7"; gene_type "
At_chr7	Cufflinks	exon	4581131	4581289	.	-	.	gene_id "XLOC_091935"; transcript_id "Novel_001251"; exon_number "1"; gene_type "
At_chr7	Cufflinks	exon	4581389	4581458	.	-	.	gene_id "XLOC_091935"; transcript_id "Novel_001251"; exon_number "2"; gene_type "
At_chr7	Cufflinks	exon	4581545	4581638	.	-	.	gene_id "XLOC_091935"; transcript_id "Novel_001251"; exon_number "3"; gene_type "
At_chr7	Cufflinks	exon	4581952	4582052	.	-	.	gene_id "XLOC_091935"; transcript_id "Novel_001251"; exon_number "4"; gene_type "
At_chr7	Cufflinks	exon	4582248	4582316	.	-	.	gene_id "XLOC_091935"; transcript_id "Novel_001251"; exon_number "5"; gene_type "
At_chr7	Cufflinks	exon	4582398	4582612	.	-	.	gene_id "XLOC_091935"; transcript_id "Novel_001251"; exon_number "6"; gene_type "
At_chr7	Cufflinks	exon	4583174	4583222	.	-	.	gene_id "XLOC_091935"; transcript_id "Novel_001251"; exon_number "7"; gene_type "
At_chr7	Cufflinks	exon	4583435	4584417	.	-	.	gene_id "XLOC_091935"; transcript_id "Novel_001251"; exon_number "8"; gene_type "
At_chr7	Cufflinks	exon	4584540	4584940	.	-	.	gene_id "XLOC_091936"; transcript_id "Novel_001252"; exon_number "1"; gene_type "
At_chr7	Cufflinks	exon	4586038	4586121	.	-	.	gene_id "XLOC_091936"; transcript_id "Novel_001252"; exon_number "2"; gene_type "
At_chr7	Cufflinks	exon	4608888	4609090	.	-	.	gene_id "XLOC_091938"; transcript_id "Novel_001253"; exon_number "1"; gene_type "
At_chr7	Cufflinks	exon	6022458	6023485	.	-	.	gene_id "XLOC_092193"; transcript_id "Novel_001254"; exon_number "1"; gene_type "
At_chr7	Cufflinks	exon	6023616	6023745	.	-	.	gene_id "XLOC_092193"; transcript_id "Novel_001254"; exon_number "2"; gene_type "
At_chr7	Cufflinks	exon	6024017	6024387	.	-	.	gene_id "XLOC_092193"; transcript_id "Novel_001254"; exon_number "3"; gene_type "
At_chr7	Cufflinks	exon	7580148	7580915	.	-	.	gene_id "XLOC_092473"; transcript_id "Novel_001255"; exon_number "1"; gene_type "
At_chr7	Cufflinks	exon	7581002	7581092	.	-	.	gene_id "XLOC_092473"; transcript_id "Novel_001255"; exon_number "2"; gene_type "
At_chr7	Cufflinks	exon	7581291	7583232	.	-	.	gene_id "XLOC_092473"; transcript_id "Novel_001255"; exon_number "3"; gene_type "
At_chr7	Cufflinks	exon	7585874	7587235	.	-	.	gene_id "XLOC_092473"; transcript_id "Novel_001256"; exon_number "1"; gene_type "
At_chr7	Cufflinks	exon	7587666	7587914	.	-	.	gene_id "XLOC_092473"; transcript_id "Novel_001256"; exon_number "2"; gene_type "
At_chr7	Cufflinks	exon	8324821	8325173	.	-	.	gene_id "XLOC_092566"; transcript_id "Novel_001257"; exon_number "1"; gene_type "
At_chr7	Cufflinks	exon	9357728	9358629	.	-	.	gene_id "XLOC_092758"; transcript_id "Novel_001258"; exon_number "1"; gene_type "
At_chr7	Cufflinks	exon	9358763	9358870	.	-	.	gene_id "XLOC_092758"; transcript_id "Novel_001258"; exon_number "2"; gene_type "
At_chr7	Cufflinks	exon	9360301	9360705	.	-	.	gene_id "XLOC_092758"; transcript_id "Novel_001259"; exon_number "1"; gene_type "
At_chr7	Cufflinks	exon	9361095	9361150	.	-	.	gene_id "XLOC_092758"; transcript_id "Novel_001259"; exon_number "2"; gene_type "
At_chr7	Cufflinks	exon	9361541	9361647	.	-	.	gene_id "XLOC_092758"; transcript_id "Novel_001259"; exon_number "3"; gene_type "
At_chr7	Cufflinks	exon	9361730	9362769	.	-	.	gene_id "XLOC_092758"; transcript_id "Novel_001259"; exon_number "4"; gene_type "
At_chr7	Cufflinks	exon	9550967	9551208	.	-	.	gene_id "XLOC_092798"; transcript_id "Novel_001260"; exon_number "1"; gene_type "

At_chr7	Cufflinks	exon	9551807	9551957	.	-	.	gene_id "XLOC_092798"; transcript_id "Novel_001260"; exon_number "2"; gene_type "
At_chr7	Cufflinks	exon	9552339	9552985	.	-	.	gene_id "XLOC_092798"; transcript_id "Novel_001260"; exon_number "3"; gene_type "
At_chr7	Cufflinks	exon	9553212	9553257	.	-	.	gene_id "XLOC_092798"; transcript_id "Novel_001260"; exon_number "4"; gene_type "
At_chr7	Cufflinks	exon	9553390	9553425	.	-	.	gene_id "XLOC_092798"; transcript_id "Novel_001260"; exon_number "5"; gene_type "
At_chr7	Cufflinks	exon	9764400	9766454	.	-	.	gene_id "XLOC_092842"; transcript_id "Novel_001261"; exon_number "1"; gene_type "
At_chr7	Cufflinks	exon	9782868	9783039	.	-	.	gene_id "XLOC_092842"; transcript_id "Novel_001261"; exon_number "2"; gene_type "
At_chr7	Cufflinks	exon	9783313	9783547	.	-	.	gene_id "XLOC_092842"; transcript_id "Novel_001261"; exon_number "3"; gene_type "
At_chr7	Cufflinks	exon	9850256	9851335	.	-	.	gene_id "XLOC_092859"; transcript_id "Novel_001262"; exon_number "1"; gene_type "
At_chr7	Cufflinks	exon	9851607	9851822	.	-	.	gene_id "XLOC_092859"; transcript_id "Novel_001262"; exon_number "2"; gene_type "
At_chr7	Cufflinks	exon	9896750	9897827	.	-	.	gene_id "XLOC_092864"; transcript_id "Novel_001263"; exon_number "1"; gene_type "
At_chr7	Cufflinks	exon	9898338	9898411	.	-	.	gene_id "XLOC_092864"; transcript_id "Novel_001263"; exon_number "2"; gene_type "
At_chr7	Cufflinks	exon	9898503	9898730	.	-	.	gene_id "XLOC_092864"; transcript_id "Novel_001263"; exon_number "3"; gene_type "
At_chr7	Cufflinks	exon	9901035	9901549	.	-	.	gene_id "XLOC_092865"; transcript_id "Novel_001264"; exon_number "1"; gene_type "
At_chr7	Cufflinks	exon	9901691	9902558	.	-	.	gene_id "XLOC_092865"; transcript_id "Novel_001264"; exon_number "2"; gene_type "
At_chr7	Cufflinks	exon	10081551	10082079	.	-	.	gene_id "XLOC_092902"; transcript_id "Novel_001265"; exon_number "1"; gene_type "
At_chr7	Cufflinks	exon	10083518	10083665	.	-	.	gene_id "XLOC_092902"; transcript_id "Novel_001265"; exon_number "2"; gene_type "
At_chr7	Cufflinks	exon	10083828	10084011	.	-	.	gene_id "XLOC_092902"; transcript_id "Novel_001265"; exon_number "3"; gene_type "
At_chr7	Cufflinks	exon	11343635	11343965	.	-	.	gene_id "XLOC_093100"; transcript_id "Novel_001266"; exon_number "1"; gene_type "
At_chr7	Cufflinks	exon	11344150	11344310	.	-	.	gene_id "XLOC_093100"; transcript_id "Novel_001266"; exon_number "2"; gene_type "
At_chr7	Cufflinks	exon	11344412	11344540	.	-	.	gene_id "XLOC_093100"; transcript_id "Novel_001266"; exon_number "3"; gene_type "
At_chr7	Cufflinks	exon	11344636	11344729	.	-	.	gene_id "XLOC_093100"; transcript_id "Novel_001266"; exon_number "4"; gene_type "
At_chr7	Cufflinks	exon	11344958	11345031	.	-	.	gene_id "XLOC_093100"; transcript_id "Novel_001266"; exon_number "5"; gene_type "
At_chr7	Cufflinks	exon	11345129	11345264	.	-	.	gene_id "XLOC_093100"; transcript_id "Novel_001266"; exon_number "6"; gene_type "
At_chr7	Cufflinks	exon	11345349	11345508	.	-	.	gene_id "XLOC_093100"; transcript_id "Novel_001266"; exon_number "7"; gene_type "
At_chr7	Cufflinks	exon	14001373	14001575	.	-	.	gene_id "XLOC_093368"; transcript_id "Novel_001267"; exon_number "1"; gene_type "
At_chr7	Cufflinks	exon	14001836	14001873	.	-	.	gene_id "XLOC_093368"; transcript_id "Novel_001267"; exon_number "2"; gene_type "
At_chr7	Cufflinks	exon	16983056	16983476	.	-	.	gene_id "XLOC_093492"; transcript_id "Novel_001268"; exon_number "1"; gene_type "
At_chr7	Cufflinks	exon	16984363	16984783	.	-	.	gene_id "XLOC_093492"; transcript_id "Novel_001268"; exon_number "2"; gene_type "
At_chr7	Cufflinks	exon	28003436	28003767	.	-	.	gene_id "XLOC_093847"; transcript_id "Novel_001269"; exon_number "1"; gene_type "
At_chr7	Cufflinks	exon	28003947	28004003	.	-	.	gene_id "XLOC_093847"; transcript_id "Novel_001269"; exon_number "2"; gene_type "
At_chr7	Cufflinks	exon	28004079	28004222	.	-	.	gene_id "XLOC_093847"; transcript_id "Novel_001269"; exon_number "3"; gene_type "
At_chr7	Cufflinks	exon	28004815	28004931	.	-	.	gene_id "XLOC_093847"; transcript_id "Novel_001269"; exon_number "4"; gene_type "
At_chr7	Cufflinks	exon	28005016	28005216	.	-	.	gene_id "XLOC_093847"; transcript_id "Novel_001269"; exon_number "5"; gene_type "
At_chr7	Cufflinks	exon	28005314	28005516	.	-	.	gene_id "XLOC_093847"; transcript_id "Novel_001269"; exon_number "6"; gene_type "
At_chr7	Cufflinks	exon	28006105	28006212	.	-	.	gene_id "XLOC_093847"; transcript_id "Novel_001269"; exon_number "7"; gene_type "

At_chr7	Cufflinks	exon	28006307	28006381	.	-	.	gene_id "XLOC_093847"; transcript_id "Novel_001269"; exon_number "8"; gene_type "
At_chr7	Cufflinks	exon	28006459	28006918	.	-	.	gene_id "XLOC_093847"; transcript_id "Novel_001269"; exon_number "9"; gene_type "
At_chr7	Cufflinks	exon	28007020	28007081	.	-	.	gene_id "XLOC_093847"; transcript_id "Novel_001269"; exon_number "10"; gene_type
At_chr7	Cufflinks	exon	28007413	28007551	.	-	.	gene_id "XLOC_093847"; transcript_id "Novel_001269"; exon_number "11"; gene_type
At_chr7	Cufflinks	exon	28005130	28006212	.	-	.	gene_id "XLOC_093847"; transcript_id "Novel_001270"; exon_number "1"; gene_type "
At_chr7	Cufflinks	exon	28006307	28006381	.	-	.	gene_id "XLOC_093847"; transcript_id "Novel_001270"; exon_number "2"; gene_type "
At_chr7	Cufflinks	exon	28006459	28006918	.	-	.	gene_id "XLOC_093847"; transcript_id "Novel_001270"; exon_number "3"; gene_type "
At_chr7	Cufflinks	exon	28007020	28007081	.	-	.	gene_id "XLOC_093847"; transcript_id "Novel_001270"; exon_number "4"; gene_type "
At_chr7	Cufflinks	exon	28007413	28007553	.	-	.	gene_id "XLOC_093847"; transcript_id "Novel_001270"; exon_number "5"; gene_type "
At_chr7	Cufflinks	exon	28488517	28491003	.	-	.	gene_id "XLOC_093879"; transcript_id "Novel_001271"; exon_number "1"; gene_type "
At_chr7	Cufflinks	exon	28491104	28492805	.	-	.	gene_id "XLOC_093879"; transcript_id "Novel_001271"; exon_number "2"; gene_type "
At_chr7	Cufflinks	exon	28574602	28575075	.	-	.	gene_id "XLOC_093884"; transcript_id "Novel_001272"; exon_number "1"; gene_type "
At_chr7	Cufflinks	exon	28575331	28575470	.	-	.	gene_id "XLOC_093884"; transcript_id "Novel_001272"; exon_number "2"; gene_type "
At_chr7	Cufflinks	exon	28576940	28577021	.	-	.	gene_id "XLOC_093884"; transcript_id "Novel_001272"; exon_number "3"; gene_type "
At_chr7	Cufflinks	exon	28577098	28577197	.	-	.	gene_id "XLOC_093884"; transcript_id "Novel_001272"; exon_number "4"; gene_type "
At_chr7	Cufflinks	exon	29084219	29085393	.	-	.	gene_id "XLOC_093925"; transcript_id "Novel_001273"; exon_number "1"; gene_type "
At_chr7	Cufflinks	exon	29085482	29085985	.	-	.	gene_id "XLOC_093925"; transcript_id "Novel_001273"; exon_number "2"; gene_type "
At_chr7	Cufflinks	exon	29661615	29661706	.	-	.	gene_id "XLOC_093969"; transcript_id "Novel_001274"; exon_number "1"; gene_type "
At_chr7	Cufflinks	exon	29661830	29661905	.	-	.	gene_id "XLOC_093969"; transcript_id "Novel_001274"; exon_number "2"; gene_type "
At_chr7	Cufflinks	exon	29663139	29663211	.	-	.	gene_id "XLOC_093969"; transcript_id "Novel_001274"; exon_number "3"; gene_type "
At_chr7	Cufflinks	exon	29663617	29663674	.	-	.	gene_id "XLOC_093969"; transcript_id "Novel_001274"; exon_number "4"; gene_type "
At_chr7	Cufflinks	exon	29663813	29664016	.	-	.	gene_id "XLOC_093969"; transcript_id "Novel_001274"; exon_number "5"; gene_type "
At_chr8	Cufflinks	exon	3194585	3194790	.	+	.	gene_id "XLOC_094507"; transcript_id "Novel_001275"; exon_number "1"; gene_type "
At_chr8	Cufflinks	exon	3196195	3196273	.	+	.	gene_id "XLOC_094507"; transcript_id "Novel_001275"; exon_number "2"; gene_type "
At_chr8	Cufflinks	exon	3196704	3196803	.	+	.	gene_id "XLOC_094507"; transcript_id "Novel_001275"; exon_number "3"; gene_type "
At_chr8	Cufflinks	exon	3197107	3201456	.	+	.	gene_id "XLOC_094507"; transcript_id "Novel_001275"; exon_number "4"; gene_type "
At_chr8	Cufflinks	exon	12843221	12843343	.	+	.	gene_id "XLOC_094869"; transcript_id "Novel_001276"; exon_number "1"; gene_type "
At_chr8	Cufflinks	exon	12843786	12844699	.	+	.	gene_id "XLOC_094869"; transcript_id "Novel_001276"; exon_number "2"; gene_type "
At_chr8	Cufflinks	exon	12869755	12870089	.	+	.	gene_id "XLOC_094875"; transcript_id "Novel_001277"; exon_number "1"; gene_type "
At_chr8	Cufflinks	exon	12870983	12871668	.	+	.	gene_id "XLOC_094875"; transcript_id "Novel_001277"; exon_number "2"; gene_type "
At_chr8	Cufflinks	exon	13108922	13109164	.	+	.	gene_id "XLOC_094896"; transcript_id "Novel_001278"; exon_number "1"; gene_type "
At_chr8	Cufflinks	exon	13109253	13109390	.	+	.	gene_id "XLOC_094896"; transcript_id "Novel_001278"; exon_number "2"; gene_type "
At_chr8	Cufflinks	exon	13116126	13117210	.	+	.	gene_id "XLOC_094896"; transcript_id "Novel_001278"; exon_number "3"; gene_type "
At_chr8	Cufflinks	exon	24783638	24783960	.	+	.	gene_id "XLOC_095334"; transcript_id "Novel_001279"; exon_number "1"; gene_type "
At_chr8	Cufflinks	exon	24784404	24784457	.	+	.	gene_id "XLOC_095334"; transcript_id "Novel_001279"; exon_number "2"; gene_type "

At_chr8	Cufflinks	exon	24784636	24784659	.	+	.	gene_id "XLOC_095334"; transcript_id "Novel_001279"; exon_number "3"; gene_type "
At_chr8	Cufflinks	exon	24784724	24784747	.	+	.	gene_id "XLOC_095334"; transcript_id "Novel_001279"; exon_number "4"; gene_type "
At_chr8	Cufflinks	exon	30343101	30343348	.	+	.	gene_id "XLOC_095484"; transcript_id "Novel_001280"; exon_number "1"; gene_type "
At_chr8	Cufflinks	exon	30343453	30343500	.	+	.	gene_id "XLOC_095484"; transcript_id "Novel_001280"; exon_number "2"; gene_type "
At_chr8	Cufflinks	exon	31658968	31659239	.	+	.	gene_id "XLOC_095521"; transcript_id "Novel_001281"; exon_number "1"; gene_type "
At_chr8	Cufflinks	exon	33582240	33582461	.	+	.	gene_id "XLOC_095590"; transcript_id "Novel_001282"; exon_number "1"; gene_type "
At_chr8	Cufflinks	exon	33582629	33582690	.	+	.	gene_id "XLOC_095590"; transcript_id "Novel_001282"; exon_number "2"; gene_type "
At_chr8	Cufflinks	exon	33584911	33586468	.	+	.	gene_id "XLOC_095590"; transcript_id "Novel_001282"; exon_number "3"; gene_type "
At_chr8	Cufflinks	exon	33582260	33582461	.	+	.	gene_id "XLOC_095590"; transcript_id "Novel_001283"; exon_number "1"; gene_type "
At_chr8	Cufflinks	exon	33582629	33582690	.	+	.	gene_id "XLOC_095590"; transcript_id "Novel_001283"; exon_number "2"; gene_type "
At_chr8	Cufflinks	exon	33584911	33584970	.	+	.	gene_id "XLOC_095590"; transcript_id "Novel_001283"; exon_number "3"; gene_type "
At_chr8	Cufflinks	exon	33585248	33586475	.	+	.	gene_id "XLOC_095590"; transcript_id "Novel_001283"; exon_number "4"; gene_type "
At_chr8	Cufflinks	exon	33582527	33582690	.	+	.	gene_id "XLOC_095590"; transcript_id "Novel_001284"; exon_number "1"; gene_type "
At_chr8	Cufflinks	exon	33584911	33584970	.	+	.	gene_id "XLOC_095590"; transcript_id "Novel_001284"; exon_number "2"; gene_type "
At_chr8	Cufflinks	exon	33585248	33586425	.	+	.	gene_id "XLOC_095590"; transcript_id "Novel_001284"; exon_number "3"; gene_type "
At_chr8	Cufflinks	exon	38049687	38049907	.	+	.	gene_id "XLOC_095833"; transcript_id "Novel_001285"; exon_number "1"; gene_type "
At_chr8	Cufflinks	exon	38050438	38050557	.	+	.	gene_id "XLOC_095833"; transcript_id "Novel_001285"; exon_number "2"; gene_type "
At_chr8	Cufflinks	exon	38051796	38051861	.	+	.	gene_id "XLOC_095833"; transcript_id "Novel_001285"; exon_number "3"; gene_type "
At_chr8	Cufflinks	exon	38052011	38052555	.	+	.	gene_id "XLOC_095833"; transcript_id "Novel_001285"; exon_number "4"; gene_type "
At_chr8	Cufflinks	exon	40137487	40140013	.	+	.	gene_id "XLOC_096045"; transcript_id "Novel_001286"; exon_number "1"; gene_type "
At_chr8	Cufflinks	exon	40140532	40141131	.	+	.	gene_id "XLOC_096045"; transcript_id "Novel_001286"; exon_number "2"; gene_type "
At_chr8	Cufflinks	exon	40137631	40137656	.	+	.	gene_id "XLOC_096045"; transcript_id "Novel_001287"; exon_number "1"; gene_type "
At_chr8	Cufflinks	exon	40137797	40137972	.	+	.	gene_id "XLOC_096045"; transcript_id "Novel_001287"; exon_number "2"; gene_type "
At_chr8	Cufflinks	exon	40139817	40140013	.	+	.	gene_id "XLOC_096045"; transcript_id "Novel_001287"; exon_number "3"; gene_type "
At_chr8	Cufflinks	exon	40140532	40141242	.	+	.	gene_id "XLOC_096045"; transcript_id "Novel_001287"; exon_number "4"; gene_type "
At_chr8	Cufflinks	exon	40137686	40137972	.	+	.	gene_id "XLOC_096045"; transcript_id "Novel_001288"; exon_number "1"; gene_type "
At_chr8	Cufflinks	exon	40139817	40140013	.	+	.	gene_id "XLOC_096045"; transcript_id "Novel_001288"; exon_number "2"; gene_type "
At_chr8	Cufflinks	exon	40140532	40141229	.	+	.	gene_id "XLOC_096045"; transcript_id "Novel_001288"; exon_number "3"; gene_type "
At_chr8	Cufflinks	exon	41141033	41141349	.	+	.	gene_id "XLOC_096122"; transcript_id "Novel_001289"; exon_number "1"; gene_type "
At_chr8	Cufflinks	exon	41142755	41143729	.	+	.	gene_id "XLOC_096122"; transcript_id "Novel_001289"; exon_number "2"; gene_type "
At_chr8	Cufflinks	exon	43152441	43152665	.	+	.	gene_id "XLOC_096393"; transcript_id "Novel_001290"; exon_number "1"; gene_type "
At_chr8	Cufflinks	exon	43152853	43153343	.	+	.	gene_id "XLOC_096393"; transcript_id "Novel_001290"; exon_number "2"; gene_type "
At_chr8	Cufflinks	exon	43153426	43153787	.	+	.	gene_id "XLOC_096393"; transcript_id "Novel_001290"; exon_number "3"; gene_type "
At_chr8	Cufflinks	exon	43153885	43157116	.	+	.	gene_id "XLOC_096393"; transcript_id "Novel_001290"; exon_number "4"; gene_type "
At_chr8	Cufflinks	exon	43960103	43960973	.	+	.	gene_id "XLOC_096535"; transcript_id "Novel_001291"; exon_number "1"; gene_type "

At_chr8	Cufflinks	exon	43961065	43961662	.	+	.	gene_id "XLOC_096535"; transcript_id "Novel_001291"; exon_number "2"; gene_type "
At_chr8	Cufflinks	exon	43990644	43990879	.	+	.	gene_id "XLOC_096548"; transcript_id "Novel_001292"; exon_number "1"; gene_type "
At_chr8	Cufflinks	exon	43991775	43993172	.	+	.	gene_id "XLOC_096548"; transcript_id "Novel_001292"; exon_number "2"; gene_type "
At_chr8	Cufflinks	exon	44005861	44005995	.	+	.	gene_id "XLOC_096556"; transcript_id "Novel_001293"; exon_number "1"; gene_type "
At_chr8	Cufflinks	exon	44006310	44007854	.	+	.	gene_id "XLOC_096556"; transcript_id "Novel_001293"; exon_number "2"; gene_type "
At_chr8	Cufflinks	exon	44023609	44024319	.	+	.	gene_id "XLOC_096562"; transcript_id "Novel_001294"; exon_number "1"; gene_type "
At_chr8	Cufflinks	exon	44024405	44025169	.	+	.	gene_id "XLOC_096562"; transcript_id "Novel_001294"; exon_number "2"; gene_type "
At_chr8	Cufflinks	exon	45885008	45885181	.	+	.	gene_id "XLOC_096822"; transcript_id "Novel_001295"; exon_number "1"; gene_type "
At_chr8	Cufflinks	exon	45885299	45885501	.	+	.	gene_id "XLOC_096822"; transcript_id "Novel_001295"; exon_number "2"; gene_type "
At_chr8	Cufflinks	exon	45885600	45885698	.	+	.	gene_id "XLOC_096822"; transcript_id "Novel_001295"; exon_number "3"; gene_type "
At_chr8	Cufflinks	exon	45885780	45886051	.	+	.	gene_id "XLOC_096822"; transcript_id "Novel_001295"; exon_number "4"; gene_type "
At_chr8	Cufflinks	exon	45886163	45886256	.	+	.	gene_id "XLOC_096822"; transcript_id "Novel_001295"; exon_number "5"; gene_type "
At_chr8	Cufflinks	exon	45886443	45886540	.	+	.	gene_id "XLOC_096822"; transcript_id "Novel_001295"; exon_number "6"; gene_type "
At_chr8	Cufflinks	exon	45886711	45886805	.	+	.	gene_id "XLOC_096822"; transcript_id "Novel_001295"; exon_number "7"; gene_type "
At_chr8	Cufflinks	exon	45886931	45887044	.	+	.	gene_id "XLOC_096822"; transcript_id "Novel_001295"; exon_number "8"; gene_type "
At_chr8	Cufflinks	exon	45887192	45887266	.	+	.	gene_id "XLOC_096822"; transcript_id "Novel_001295"; exon_number "9"; gene_type "
At_chr8	Cufflinks	exon	45887435	45888500	.	+	.	gene_id "XLOC_096822"; transcript_id "Novel_001295"; exon_number "10"; gene_type
At_chr8	Cufflinks	exon	45886000	45886051	.	+	.	gene_id "XLOC_096822"; transcript_id "Novel_001296"; exon_number "1"; gene_type "
At_chr8	Cufflinks	exon	45886163	45886256	.	+	.	gene_id "XLOC_096822"; transcript_id "Novel_001296"; exon_number "2"; gene_type "
At_chr8	Cufflinks	exon	45886443	45886540	.	+	.	gene_id "XLOC_096822"; transcript_id "Novel_001296"; exon_number "3"; gene_type "
At_chr8	Cufflinks	exon	45886704	45886805	.	+	.	gene_id "XLOC_096822"; transcript_id "Novel_001296"; exon_number "4"; gene_type "
At_chr8	Cufflinks	exon	45886931	45887044	.	+	.	gene_id "XLOC_096822"; transcript_id "Novel_001296"; exon_number "5"; gene_type "
At_chr8	Cufflinks	exon	45887192	45888490	.	+	.	gene_id "XLOC_096822"; transcript_id "Novel_001296"; exon_number "6"; gene_type "
At_chr8	Cufflinks	exon	45952372	45953070	.	+	.	gene_id "XLOC_096828"; transcript_id "Novel_001297"; exon_number "1"; gene_type "
At_chr8	Cufflinks	exon	46424453	46424620	.	+	.	gene_id "XLOC_096851"; transcript_id "Novel_001298"; exon_number "1"; gene_type "
At_chr8	Cufflinks	exon	46424709	46424784	.	+	.	gene_id "XLOC_096851"; transcript_id "Novel_001298"; exon_number "2"; gene_type "
At_chr8	Cufflinks	exon	46430178	46430187	.	+	.	gene_id "XLOC_096851"; transcript_id "Novel_001298"; exon_number "3"; gene_type "
At_chr8	Cufflinks	exon	46479197	46479414	.	+	.	gene_id "XLOC_096866"; transcript_id "Novel_001299"; exon_number "1"; gene_type "
At_chr8	Cufflinks	exon	46479967	46480016	.	+	.	gene_id "XLOC_096866"; transcript_id "Novel_001299"; exon_number "2"; gene_type "
At_chr8	Cufflinks	exon	46482210	46482462	.	+	.	gene_id "XLOC_096867"; transcript_id "Novel_001300"; exon_number "1"; gene_type "
At_chr8	Cufflinks	exon	46483079	46483919	.	+	.	gene_id "XLOC_096867"; transcript_id "Novel_001300"; exon_number "2"; gene_type "
At_chr8	Cufflinks	exon	46511760	46511861	.	+	.	gene_id "XLOC_096869"; transcript_id "Novel_001301"; exon_number "1"; gene_type "
At_chr8	Cufflinks	exon	46512031	46512149	.	+	.	gene_id "XLOC_096869"; transcript_id "Novel_001301"; exon_number "2"; gene_type "
At_chr8	Cufflinks	exon	46512357	46512536	.	+	.	gene_id "XLOC_096869"; transcript_id "Novel_001301"; exon_number "3"; gene_type "
At_chr8	Cufflinks	exon	46513065	46513136	.	+	.	gene_id "XLOC_096869"; transcript_id "Novel_001301"; exon_number "4"; gene_type "

At_chr8	Cufflinks	exon	46513220	46513419	.	+	.	gene_id "XLOC_096869"; transcript_id "Novel_001301"; exon_number "5"; gene_type "
At_chr8	Cufflinks	exon	46513675	46514090	.	+	.	gene_id "XLOC_096869"; transcript_id "Novel_001301"; exon_number "6"; gene_type "
At_chr8	Cufflinks	exon	46514290	46514474	.	+	.	gene_id "XLOC_096869"; transcript_id "Novel_001301"; exon_number "7"; gene_type "
At_chr8	Cufflinks	exon	46514635	46515698	.	+	.	gene_id "XLOC_096869"; transcript_id "Novel_001301"; exon_number "8"; gene_type "
At_chr8	Cufflinks	exon	46879300	46881609	.	+	.	gene_id "XLOC_096882"; transcript_id "Novel_001302"; exon_number "1"; gene_type "
At_chr8	Cufflinks	exon	46881867	46882331	.	+	.	gene_id "XLOC_096882"; transcript_id "Novel_001302"; exon_number "2"; gene_type "
At_chr8	Cufflinks	exon	46879326	46880612	.	+	.	gene_id "XLOC_096882"; transcript_id "Novel_001303"; exon_number "1"; gene_type "
At_chr8	Cufflinks	exon	46880742	46880821	.	+	.	gene_id "XLOC_096882"; transcript_id "Novel_001303"; exon_number "2"; gene_type "
At_chr8	Cufflinks	exon	46880905	46880997	.	+	.	gene_id "XLOC_096882"; transcript_id "Novel_001303"; exon_number "3"; gene_type "
At_chr8	Cufflinks	exon	46881129	46881206	.	+	.	gene_id "XLOC_096882"; transcript_id "Novel_001303"; exon_number "4"; gene_type "
At_chr8	Cufflinks	exon	46881478	46881609	.	+	.	gene_id "XLOC_096882"; transcript_id "Novel_001303"; exon_number "5"; gene_type "
At_chr8	Cufflinks	exon	46881867	46882603	.	+	.	gene_id "XLOC_096882"; transcript_id "Novel_001303"; exon_number "6"; gene_type "
At_chr8	Cufflinks	exon	46879347	46880612	.	+	.	gene_id "XLOC_096882"; transcript_id "Novel_001304"; exon_number "1"; gene_type "
At_chr8	Cufflinks	exon	46880742	46880821	.	+	.	gene_id "XLOC_096882"; transcript_id "Novel_001304"; exon_number "2"; gene_type "
At_chr8	Cufflinks	exon	46881129	46881206	.	+	.	gene_id "XLOC_096882"; transcript_id "Novel_001304"; exon_number "3"; gene_type "
At_chr8	Cufflinks	exon	46881478	46881609	.	+	.	gene_id "XLOC_096882"; transcript_id "Novel_001304"; exon_number "4"; gene_type "
At_chr8	Cufflinks	exon	46881867	46882475	.	+	.	gene_id "XLOC_096882"; transcript_id "Novel_001304"; exon_number "5"; gene_type "
At_chr8	Cufflinks	exon	46879350	46880136	.	+	.	gene_id "XLOC_096882"; transcript_id "Novel_001305"; exon_number "1"; gene_type "
At_chr8	Cufflinks	exon	46880216	46880293	.	+	.	gene_id "XLOC_096882"; transcript_id "Novel_001305"; exon_number "2"; gene_type "
At_chr8	Cufflinks	exon	46880536	46880612	.	+	.	gene_id "XLOC_096882"; transcript_id "Novel_001305"; exon_number "3"; gene_type "
At_chr8	Cufflinks	exon	46880742	46880821	.	+	.	gene_id "XLOC_096882"; transcript_id "Novel_001305"; exon_number "4"; gene_type "
At_chr8	Cufflinks	exon	46880905	46880997	.	+	.	gene_id "XLOC_096882"; transcript_id "Novel_001305"; exon_number "5"; gene_type "
At_chr8	Cufflinks	exon	46881129	46881206	.	+	.	gene_id "XLOC_096882"; transcript_id "Novel_001305"; exon_number "6"; gene_type "
At_chr8	Cufflinks	exon	46881478	46881609	.	+	.	gene_id "XLOC_096882"; transcript_id "Novel_001305"; exon_number "7"; gene_type "
At_chr8	Cufflinks	exon	46881867	46882422	.	+	.	gene_id "XLOC_096882"; transcript_id "Novel_001305"; exon_number "8"; gene_type "
At_chr8	Cufflinks	exon	47026075	47026097	.	+	.	gene_id "XLOC_096887"; transcript_id "Novel_001306"; exon_number "1"; gene_type "
At_chr8	Cufflinks	exon	47026189	47027405	.	+	.	gene_id "XLOC_096887"; transcript_id "Novel_001306"; exon_number "2"; gene_type "
At_chr8	Cufflinks	exon	47203291	47203598	.	+	.	gene_id "XLOC_096894"; transcript_id "Novel_001307"; exon_number "1"; gene_type "
At_chr8	Cufflinks	exon	47206112	47206173	.	+	.	gene_id "XLOC_096894"; transcript_id "Novel_001307"; exon_number "2"; gene_type "
At_chr8	Cufflinks	exon	47207844	47211272	.	+	.	gene_id "XLOC_096894"; transcript_id "Novel_001307"; exon_number "3"; gene_type "
At_chr8	Cufflinks	exon	47784249	47784712	.	+	.	gene_id "XLOC_096929"; transcript_id "Novel_001308"; exon_number "1"; gene_type "
At_chr8	Cufflinks	exon	47785487	47785568	.	+	.	gene_id "XLOC_096929"; transcript_id "Novel_001308"; exon_number "2"; gene_type "
At_chr8	Cufflinks	exon	47786553	47787277	.	+	.	gene_id "XLOC_096929"; transcript_id "Novel_001308"; exon_number "3"; gene_type "
At_chr8	Cufflinks	exon	47784259	47784712	.	+	.	gene_id "XLOC_096929"; transcript_id "Novel_001309"; exon_number "1"; gene_type "
At_chr8	Cufflinks	exon	47785487	47785568	.	+	.	gene_id "XLOC_096929"; transcript_id "Novel_001309"; exon_number "2"; gene_type "

At_chr8	Cufflinks	exon	47786330	47787266	.	+	.	gene_id "XLOC_096929"; transcript_id "Novel_001309"; exon_number "3"; gene_type "
At_chr8	Cufflinks	exon	49833688	49833831	.	+	.	gene_id "XLOC_097017"; transcript_id "Novel_001310"; exon_number "1"; gene_type "
At_chr8	Cufflinks	exon	49834260	49835180	.	+	.	gene_id "XLOC_097017"; transcript_id "Novel_001310"; exon_number "2"; gene_type "
At_chr8	Cufflinks	exon	49874847	49875239	.	+	.	gene_id "XLOC_097020"; transcript_id "Novel_001311"; exon_number "1"; gene_type "
At_chr8	Cufflinks	exon	49875510	49875624	.	+	.	gene_id "XLOC_097020"; transcript_id "Novel_001311"; exon_number "2"; gene_type "
At_chr8	Cufflinks	exon	49876335	49876808	.	+	.	gene_id "XLOC_097020"; transcript_id "Novel_001311"; exon_number "3"; gene_type "
At_chr8	Cufflinks	exon	49879181	49879685	.	+	.	gene_id "XLOC_097020"; transcript_id "Novel_001311"; exon_number "4"; gene_type "
At_chr8	Cufflinks	exon	49879855	49879895	.	+	.	gene_id "XLOC_097020"; transcript_id "Novel_001311"; exon_number "5"; gene_type "
At_chr8	Cufflinks	exon	49880069	49880453	.	+	.	gene_id "XLOC_097020"; transcript_id "Novel_001311"; exon_number "6"; gene_type "
At_chr8	Cufflinks	exon	49875149	49875239	.	+	.	gene_id "XLOC_097020"; transcript_id "Novel_001312"; exon_number "1"; gene_type "
At_chr8	Cufflinks	exon	49877584	49877698	.	+	.	gene_id "XLOC_097020"; transcript_id "Novel_001312"; exon_number "2"; gene_type "
At_chr8	Cufflinks	exon	49878404	49878877	.	+	.	gene_id "XLOC_097020"; transcript_id "Novel_001312"; exon_number "3"; gene_type "
At_chr8	Cufflinks	exon	49879181	49879685	.	+	.	gene_id "XLOC_097020"; transcript_id "Novel_001312"; exon_number "4"; gene_type "
At_chr8	Cufflinks	exon	49879855	49879895	.	+	.	gene_id "XLOC_097020"; transcript_id "Novel_001312"; exon_number "5"; gene_type "
At_chr8	Cufflinks	exon	49880069	49880446	.	+	.	gene_id "XLOC_097020"; transcript_id "Novel_001312"; exon_number "6"; gene_type "
At_chr8	Cufflinks	exon	52064535	52064806	.	+	.	gene_id "XLOC_097081"; transcript_id "Novel_001313"; exon_number "1"; gene_type "
At_chr8	Cufflinks	exon	52065650	52065760	.	+	.	gene_id "XLOC_097081"; transcript_id "Novel_001313"; exon_number "2"; gene_type "
At_chr8	Cufflinks	exon	52067107	52067925	.	+	.	gene_id "XLOC_097081"; transcript_id "Novel_001313"; exon_number "3"; gene_type "
At_chr8	Cufflinks	exon	53527603	53527819	.	+	.	gene_id "XLOC_097111"; transcript_id "Novel_001314"; exon_number "1"; gene_type "
At_chr8	Cufflinks	exon	53527895	53527995	.	+	.	gene_id "XLOC_097111"; transcript_id "Novel_001314"; exon_number "2"; gene_type "
At_chr8	Cufflinks	exon	53528097	53528214	.	+	.	gene_id "XLOC_097111"; transcript_id "Novel_001314"; exon_number "3"; gene_type "
At_chr8	Cufflinks	exon	53528657	53528709	.	+	.	gene_id "XLOC_097111"; transcript_id "Novel_001314"; exon_number "4"; gene_type "
At_chr8	Cufflinks	exon	53528992	53529015	.	+	.	gene_id "XLOC_097111"; transcript_id "Novel_001314"; exon_number "5"; gene_type "
At_chr8	Cufflinks	exon	53529118	53529462	.	+	.	gene_id "XLOC_097111"; transcript_id "Novel_001314"; exon_number "6"; gene_type "
At_chr8	Cufflinks	exon	53529569	53529887	.	+	.	gene_id "XLOC_097111"; transcript_id "Novel_001314"; exon_number "7"; gene_type "
At_chr8	Cufflinks	exon	53531140	53531267	.	+	.	gene_id "XLOC_097111"; transcript_id "Novel_001314"; exon_number "8"; gene_type "
At_chr8	Cufflinks	exon	53531854	53532295	.	+	.	gene_id "XLOC_097111"; transcript_id "Novel_001314"; exon_number "9"; gene_type "
At_chr8	Cufflinks	exon	53527610	53527995	.	+	.	gene_id "XLOC_097111"; transcript_id "Novel_001315"; exon_number "1"; gene_type "
At_chr8	Cufflinks	exon	53528097	53528709	.	+	.	gene_id "XLOC_097111"; transcript_id "Novel_001315"; exon_number "2"; gene_type "
At_chr8	Cufflinks	exon	53528992	53529903	.	+	.	gene_id "XLOC_097111"; transcript_id "Novel_001315"; exon_number "3"; gene_type "
At_chr8	Cufflinks	exon	53527614	53527819	.	+	.	gene_id "XLOC_097111"; transcript_id "Novel_001316"; exon_number "1"; gene_type "
At_chr8	Cufflinks	exon	53527895	53527995	.	+	.	gene_id "XLOC_097111"; transcript_id "Novel_001316"; exon_number "2"; gene_type "
At_chr8	Cufflinks	exon	53528097	53528214	.	+	.	gene_id "XLOC_097111"; transcript_id "Novel_001316"; exon_number "3"; gene_type "
At_chr8	Cufflinks	exon	53528657	53528709	.	+	.	gene_id "XLOC_097111"; transcript_id "Novel_001316"; exon_number "4"; gene_type "
At_chr8	Cufflinks	exon	53528992	53529015	.	+	.	gene_id "XLOC_097111"; transcript_id "Novel_001316"; exon_number "5"; gene_type "

At_chr8	Cufflinks	exon	53529118	53529462	.	+	.	gene_id "XLOC_097111"; transcript_id "Novel_001316"; exon_number "6"; gene_type "
At_chr8	Cufflinks	exon	53529569	53529887	.	+	.	gene_id "XLOC_097111"; transcript_id "Novel_001316"; exon_number "7"; gene_type "
At_chr8	Cufflinks	exon	53531140	53532440	.	+	.	gene_id "XLOC_097111"; transcript_id "Novel_001316"; exon_number "8"; gene_type "
At_chr8	Cufflinks	exon	57109509	57110831	.	+	.	gene_id "XLOC_097230"; transcript_id "Novel_001317"; exon_number "1"; gene_type "
At_chr8	Cufflinks	exon	57114446	57114814	.	+	.	gene_id "XLOC_097230"; transcript_id "Novel_001317"; exon_number "2"; gene_type "
At_chr8	Cufflinks	exon	57109509	57109826	.	+	.	gene_id "XLOC_097230"; transcript_id "Novel_001318"; exon_number "1"; gene_type "
At_chr8	Cufflinks	exon	57110116	57110831	.	+	.	gene_id "XLOC_097230"; transcript_id "Novel_001318"; exon_number "2"; gene_type "
At_chr8	Cufflinks	exon	57114446	57114814	.	+	.	gene_id "XLOC_097230"; transcript_id "Novel_001318"; exon_number "3"; gene_type "
At_chr8	Cufflinks	exon	57109551	57109826	.	+	.	gene_id "XLOC_097230"; transcript_id "Novel_001319"; exon_number "1"; gene_type "
At_chr8	Cufflinks	exon	57110586	57110831	.	+	.	gene_id "XLOC_097230"; transcript_id "Novel_001319"; exon_number "2"; gene_type "
At_chr8	Cufflinks	exon	57114446	57114983	.	+	.	gene_id "XLOC_097230"; transcript_id "Novel_001319"; exon_number "3"; gene_type "
At_chr8	Cufflinks	exon	60179539	60180153	.	+	.	gene_id "XLOC_097417"; transcript_id "Novel_001320"; exon_number "1"; gene_type "
At_chr8	Cufflinks	exon	60180499	60181095	.	+	.	gene_id "XLOC_097417"; transcript_id "Novel_001320"; exon_number "2"; gene_type "
At_chr8	Cufflinks	exon	60181411	60182131	.	+	.	gene_id "XLOC_097418"; transcript_id "Novel_001321"; exon_number "1"; gene_type "
At_chr8	Cufflinks	exon	60182986	60183747	.	+	.	gene_id "XLOC_097418"; transcript_id "Novel_001321"; exon_number "2"; gene_type "
At_chr8	Cufflinks	exon	68337352	68337425	.	+	.	gene_id "XLOC_097682"; transcript_id "Novel_001322"; exon_number "1"; gene_type "
At_chr8	Cufflinks	exon	68337733	68337842	.	+	.	gene_id "XLOC_097682"; transcript_id "Novel_001322"; exon_number "2"; gene_type "
At_chr8	Cufflinks	exon	68338856	68338921	.	+	.	gene_id "XLOC_097682"; transcript_id "Novel_001322"; exon_number "3"; gene_type "
At_chr8	Cufflinks	exon	68339071	68339512	.	+	.	gene_id "XLOC_097682"; transcript_id "Novel_001322"; exon_number "4"; gene_type "
At_chr8	Cufflinks	exon	68337362	68337425	.	+	.	gene_id "XLOC_097682"; transcript_id "Novel_001323"; exon_number "1"; gene_type "
At_chr8	Cufflinks	exon	68337584	68337651	.	+	.	gene_id "XLOC_097682"; transcript_id "Novel_001323"; exon_number "2"; gene_type "
At_chr8	Cufflinks	exon	68337733	68337842	.	+	.	gene_id "XLOC_097682"; transcript_id "Novel_001323"; exon_number "3"; gene_type "
At_chr8	Cufflinks	exon	68338856	68338921	.	+	.	gene_id "XLOC_097682"; transcript_id "Novel_001323"; exon_number "4"; gene_type "
At_chr8	Cufflinks	exon	68339071	68339512	.	+	.	gene_id "XLOC_097682"; transcript_id "Novel_001323"; exon_number "5"; gene_type "
At_chr8	Cufflinks	exon	68337473	68337842	.	+	.	gene_id "XLOC_097682"; transcript_id "Novel_001324"; exon_number "1"; gene_type "
At_chr8	Cufflinks	exon	68338856	68338921	.	+	.	gene_id "XLOC_097682"; transcript_id "Novel_001324"; exon_number "2"; gene_type "
At_chr8	Cufflinks	exon	68339071	68339513	.	+	.	gene_id "XLOC_097682"; transcript_id "Novel_001324"; exon_number "3"; gene_type "
At_chr8	Cufflinks	exon	68337473	68337651	.	+	.	gene_id "XLOC_097682"; transcript_id "Novel_001325"; exon_number "1"; gene_type "
At_chr8	Cufflinks	exon	68337733	68337842	.	+	.	gene_id "XLOC_097682"; transcript_id "Novel_001325"; exon_number "2"; gene_type "
At_chr8	Cufflinks	exon	68338856	68338921	.	+	.	gene_id "XLOC_097682"; transcript_id "Novel_001325"; exon_number "3"; gene_type "
At_chr8	Cufflinks	exon	68339071	68339513	.	+	.	gene_id "XLOC_097682"; transcript_id "Novel_001325"; exon_number "4"; gene_type "
At_chr8	Cufflinks	exon	72214895	72215093	.	+	.	gene_id "XLOC_097837"; transcript_id "Novel_001326"; exon_number "1"; gene_type "
At_chr8	Cufflinks	exon	72215181	72215255	.	+	.	gene_id "XLOC_097837"; transcript_id "Novel_001326"; exon_number "2"; gene_type "
At_chr8	Cufflinks	exon	72215406	72215743	.	+	.	gene_id "XLOC_097837"; transcript_id "Novel_001326"; exon_number "3"; gene_type "
At_chr8	Cufflinks	exon	73093622	73093998	.	+	.	gene_id "XLOC_097880"; transcript_id "Novel_001327"; exon_number "1"; gene_type "

At_chr8	Cufflinks	exon	73095066	73095504	.	+	.	gene_id "XLOC_097880"; transcript_id "Novel_001327"; exon_number "2"; gene_type "
At_chr8	Cufflinks	exon	74871298	74871547	.	+	.	gene_id "XLOC_097999"; transcript_id "Novel_001328"; exon_number "1"; gene_type "
At_chr8	Cufflinks	exon	74872160	74872223	.	+	.	gene_id "XLOC_097999"; transcript_id "Novel_001328"; exon_number "2"; gene_type "
At_chr8	Cufflinks	exon	74873053	74873149	.	+	.	gene_id "XLOC_097999"; transcript_id "Novel_001328"; exon_number "3"; gene_type "
At_chr8	Cufflinks	exon	74873800	74874177	.	+	.	gene_id "XLOC_097999"; transcript_id "Novel_001328"; exon_number "4"; gene_type "
At_chr8	Cufflinks	exon	78469579	78469760	.	+	.	gene_id "XLOC_098392"; transcript_id "Novel_001329"; exon_number "1"; gene_type "
At_chr8	Cufflinks	exon	78470178	78470284	.	+	.	gene_id "XLOC_098392"; transcript_id "Novel_001329"; exon_number "2"; gene_type "
At_chr8	Cufflinks	exon	297191	299326	.	-	.	gene_id "XLOC_098448"; transcript_id "Novel_001330"; exon_number "1"; gene_type "
At_chr8	Cufflinks	exon	299433	299749	.	-	.	gene_id "XLOC_098448"; transcript_id "Novel_001330"; exon_number "2"; gene_type "
At_chr8	Cufflinks	exon	299888	299963	.	-	.	gene_id "XLOC_098448"; transcript_id "Novel_001330"; exon_number "3"; gene_type "
At_chr8	Cufflinks	exon	300156	300239	.	-	.	gene_id "XLOC_098448"; transcript_id "Novel_001330"; exon_number "4"; gene_type "
At_chr8	Cufflinks	exon	300334	301020	.	-	.	gene_id "XLOC_098448"; transcript_id "Novel_001330"; exon_number "5"; gene_type "
At_chr8	Cufflinks	exon	301110	302496	.	-	.	gene_id "XLOC_098448"; transcript_id "Novel_001330"; exon_number "6"; gene_type "
At_chr8	Cufflinks	exon	302625	302686	.	-	.	gene_id "XLOC_098448"; transcript_id "Novel_001330"; exon_number "7"; gene_type "
At_chr8	Cufflinks	exon	303669	303761	.	-	.	gene_id "XLOC_098448"; transcript_id "Novel_001330"; exon_number "8"; gene_type "
At_chr8	Cufflinks	exon	304000	304249	.	-	.	gene_id "XLOC_098448"; transcript_id "Novel_001330"; exon_number "9"; gene_type "
At_chr8	Cufflinks	exon	297274	297956	.	-	.	gene_id "XLOC_098448"; transcript_id "Novel_001331"; exon_number "1"; gene_type "
At_chr8	Cufflinks	exon	298047	299326	.	-	.	gene_id "XLOC_098448"; transcript_id "Novel_001331"; exon_number "2"; gene_type "
At_chr8	Cufflinks	exon	299433	299749	.	-	.	gene_id "XLOC_098448"; transcript_id "Novel_001331"; exon_number "3"; gene_type "
At_chr8	Cufflinks	exon	299888	299963	.	-	.	gene_id "XLOC_098448"; transcript_id "Novel_001331"; exon_number "4"; gene_type "
At_chr8	Cufflinks	exon	300156	300239	.	-	.	gene_id "XLOC_098448"; transcript_id "Novel_001331"; exon_number "5"; gene_type "
At_chr8	Cufflinks	exon	300334	301020	.	-	.	gene_id "XLOC_098448"; transcript_id "Novel_001331"; exon_number "6"; gene_type "
At_chr8	Cufflinks	exon	301110	302496	.	-	.	gene_id "XLOC_098448"; transcript_id "Novel_001331"; exon_number "7"; gene_type "
At_chr8	Cufflinks	exon	302625	302686	.	-	.	gene_id "XLOC_098448"; transcript_id "Novel_001331"; exon_number "8"; gene_type "
At_chr8	Cufflinks	exon	303669	303761	.	-	.	gene_id "XLOC_098448"; transcript_id "Novel_001331"; exon_number "9"; gene_type "
At_chr8	Cufflinks	exon	304000	304256	.	-	.	gene_id "XLOC_098448"; transcript_id "Novel_001331"; exon_number "10"; gene_type
At_chr8	Cufflinks	exon	297312	297956	.	-	.	gene_id "XLOC_098448"; transcript_id "Novel_001332"; exon_number "1"; gene_type "
At_chr8	Cufflinks	exon	298047	299326	.	-	.	gene_id "XLOC_098448"; transcript_id "Novel_001332"; exon_number "2"; gene_type "
At_chr8	Cufflinks	exon	299433	299749	.	-	.	gene_id "XLOC_098448"; transcript_id "Novel_001332"; exon_number "3"; gene_type "
At_chr8	Cufflinks	exon	299888	299963	.	-	.	gene_id "XLOC_098448"; transcript_id "Novel_001332"; exon_number "4"; gene_type "
At_chr8	Cufflinks	exon	300156	300239	.	-	.	gene_id "XLOC_098448"; transcript_id "Novel_001332"; exon_number "5"; gene_type "
At_chr8	Cufflinks	exon	300334	301020	.	-	.	gene_id "XLOC_098448"; transcript_id "Novel_001332"; exon_number "6"; gene_type "
At_chr8	Cufflinks	exon	301110	302496	.	-	.	gene_id "XLOC_098448"; transcript_id "Novel_001332"; exon_number "7"; gene_type "
At_chr8	Cufflinks	exon	302625	302686	.	-	.	gene_id "XLOC_098448"; transcript_id "Novel_001332"; exon_number "8"; gene_type "
At_chr8	Cufflinks	exon	303669	303923	.	-	.	gene_id "XLOC_098448"; transcript_id "Novel_001332"; exon_number "9"; gene_type "

At_chr8	Cufflinks	exon	2419029	2419993	.	-	.	gene_id "XLOC_098583"; transcript_id "Novel_001333"; exon_number "1"; gene_type "
At_chr8	Cufflinks	exon	2420353	2420861	.	-	.	gene_id "XLOC_098583"; transcript_id "Novel_001333"; exon_number "2"; gene_type "
At_chr8	Cufflinks	exon	2421417	2421518	.	-	.	gene_id "XLOC_098583"; transcript_id "Novel_001333"; exon_number "3"; gene_type "
At_chr8	Cufflinks	exon	2551599	2551717	.	-	.	gene_id "XLOC_098589"; transcript_id "Novel_001334"; exon_number "1"; gene_type "
At_chr8	Cufflinks	exon	2552442	2552562	.	-	.	gene_id "XLOC_098589"; transcript_id "Novel_001334"; exon_number "2"; gene_type "
At_chr8	Cufflinks	exon	2552634	2552711	.	-	.	gene_id "XLOC_098589"; transcript_id "Novel_001334"; exon_number "3"; gene_type "
At_chr8	Cufflinks	exon	2553013	2553099	.	-	.	gene_id "XLOC_098589"; transcript_id "Novel_001334"; exon_number "4"; gene_type "
At_chr8	Cufflinks	exon	2551600	2551717	.	-	.	gene_id "XLOC_098589"; transcript_id "Novel_001335"; exon_number "1"; gene_type "
At_chr8	Cufflinks	exon	2552442	2552562	.	-	.	gene_id "XLOC_098589"; transcript_id "Novel_001335"; exon_number "2"; gene_type "
At_chr8	Cufflinks	exon	2552634	2552974	.	-	.	gene_id "XLOC_098589"; transcript_id "Novel_001335"; exon_number "3"; gene_type "
At_chr8	Cufflinks	exon	2551864	2552562	.	-	.	gene_id "XLOC_098589"; transcript_id "Novel_001336"; exon_number "1"; gene_type "
At_chr8	Cufflinks	exon	2552634	2552711	.	-	.	gene_id "XLOC_098589"; transcript_id "Novel_001336"; exon_number "2"; gene_type "
At_chr8	Cufflinks	exon	2552838	2552959	.	-	.	gene_id "XLOC_098589"; transcript_id "Novel_001336"; exon_number "3"; gene_type "
At_chr8	Cufflinks	exon	3975763	3977541	.	-	.	gene_id "XLOC_098695"; transcript_id "Novel_001337"; exon_number "1"; gene_type "
At_chr8	Cufflinks	exon	3977627	3977868	.	-	.	gene_id "XLOC_098695"; transcript_id "Novel_001337"; exon_number "2"; gene_type "
At_chr8	Cufflinks	exon	4424564	4425168	.	-	.	gene_id "XLOC_098709"; transcript_id "Novel_001338"; exon_number "1"; gene_type "
At_chr8	Cufflinks	exon	4425371	4426091	.	-	.	gene_id "XLOC_098709"; transcript_id "Novel_001338"; exon_number "2"; gene_type "
At_chr8	Cufflinks	exon	4426229	4426341	.	-	.	gene_id "XLOC_098709"; transcript_id "Novel_001338"; exon_number "3"; gene_type "
At_chr8	Cufflinks	exon	4426427	4426931	.	-	.	gene_id "XLOC_098709"; transcript_id "Novel_001338"; exon_number "4"; gene_type "
At_chr8	Cufflinks	exon	4424580	4425168	.	-	.	gene_id "XLOC_098709"; transcript_id "Novel_001339"; exon_number "1"; gene_type "
At_chr8	Cufflinks	exon	4425371	4426091	.	-	.	gene_id "XLOC_098709"; transcript_id "Novel_001339"; exon_number "2"; gene_type "
At_chr8	Cufflinks	exon	4426229	4426910	.	-	.	gene_id "XLOC_098709"; transcript_id "Novel_001339"; exon_number "3"; gene_type "
At_chr8	Cufflinks	exon	13294120	13296200	.	-	.	gene_id "XLOC_099065"; transcript_id "Novel_001340"; exon_number "1"; gene_type "
At_chr8	Cufflinks	exon	13296289	13296387	.	-	.	gene_id "XLOC_099065"; transcript_id "Novel_001340"; exon_number "2"; gene_type "
At_chr8	Cufflinks	exon	13296468	13296603	.	-	.	gene_id "XLOC_099065"; transcript_id "Novel_001340"; exon_number "3"; gene_type "
At_chr8	Cufflinks	exon	13296676	13296822	.	-	.	gene_id "XLOC_099065"; transcript_id "Novel_001340"; exon_number "4"; gene_type "
At_chr8	Cufflinks	exon	13296917	13297054	.	-	.	gene_id "XLOC_099065"; transcript_id "Novel_001340"; exon_number "5"; gene_type "
At_chr8	Cufflinks	exon	13295861	13296200	.	-	.	gene_id "XLOC_099065"; transcript_id "Novel_001341"; exon_number "1"; gene_type "
At_chr8	Cufflinks	exon	13296289	13296387	.	-	.	gene_id "XLOC_099065"; transcript_id "Novel_001341"; exon_number "2"; gene_type "
At_chr8	Cufflinks	exon	13296468	13296822	.	-	.	gene_id "XLOC_099065"; transcript_id "Novel_001341"; exon_number "3"; gene_type "
At_chr8	Cufflinks	exon	13296917	13297078	.	-	.	gene_id "XLOC_099065"; transcript_id "Novel_001341"; exon_number "4"; gene_type "
At_chr8	Cufflinks	exon	15617614	15618015	.	-	.	gene_id "XLOC_099131"; transcript_id "Novel_001342"; exon_number "1"; gene_type "
At_chr8	Cufflinks	exon	15619361	15619427	.	-	.	gene_id "XLOC_099131"; transcript_id "Novel_001342"; exon_number "2"; gene_type "
At_chr8	Cufflinks	exon	15619832	15620089	.	-	.	gene_id "XLOC_099131"; transcript_id "Novel_001342"; exon_number "3"; gene_type "
At_chr8	Cufflinks	exon	15617614	15618018	.	-	.	gene_id "XLOC_099131"; transcript_id "Novel_001343"; exon_number "1"; gene_type "

At_chr8	Cufflinks	exon	15619361	15619427	.	-	.	gene_id "XLOC_099131"; transcript_id "Novel_001343"; exon_number "2"; gene_type "
At_chr8	Cufflinks	exon	15619832	15620089	.	-	.	gene_id "XLOC_099131"; transcript_id "Novel_001343"; exon_number "3"; gene_type "
At_chr8	Cufflinks	exon	15776160	15776502	.	-	.	gene_id "XLOC_099146"; transcript_id "Novel_001344"; exon_number "1"; gene_type "
At_chr8	Cufflinks	exon	15777033	15777579	.	-	.	gene_id "XLOC_099146"; transcript_id "Novel_001344"; exon_number "2"; gene_type "
At_chr8	Cufflinks	exon	22981816	22982070	.	-	.	gene_id "XLOC_099409"; transcript_id "Novel_001345"; exon_number "1"; gene_type "
At_chr8	Cufflinks	exon	22982382	22982542	.	-	.	gene_id "XLOC_099409"; transcript_id "Novel_001345"; exon_number "2"; gene_type "
At_chr8	Cufflinks	exon	24855488	24856585	.	-	.	gene_id "XLOC_099468"; transcript_id "Novel_001346"; exon_number "1"; gene_type "
At_chr8	Cufflinks	exon	24856677	24856705	.	-	.	gene_id "XLOC_099468"; transcript_id "Novel_001346"; exon_number "2"; gene_type "
At_chr8	Cufflinks	exon	24856803	24856904	.	-	.	gene_id "XLOC_099468"; transcript_id "Novel_001346"; exon_number "3"; gene_type "
At_chr8	Cufflinks	exon	24857064	24857112	.	-	.	gene_id "XLOC_099468"; transcript_id "Novel_001346"; exon_number "4"; gene_type "
At_chr8	Cufflinks	exon	31660484	31660690	.	-	.	gene_id "XLOC_099665"; transcript_id "Novel_001347"; exon_number "1"; gene_type "
At_chr8	Cufflinks	exon	33852951	33853159	.	-	.	gene_id "XLOC_099766"; transcript_id "Novel_001348"; exon_number "1"; gene_type "
At_chr8	Cufflinks	exon	34767081	34768009	.	-	.	gene_id "XLOC_099814"; transcript_id "Novel_001349"; exon_number "1"; gene_type "
At_chr8	Cufflinks	exon	34768265	34768448	.	-	.	gene_id "XLOC_099814"; transcript_id "Novel_001349"; exon_number "2"; gene_type "
At_chr8	Cufflinks	exon	35305791	35308400	.	-	.	gene_id "XLOC_099853"; transcript_id "Novel_001350"; exon_number "1"; gene_type "
At_chr8	Cufflinks	exon	35308926	35309090	.	-	.	gene_id "XLOC_099853"; transcript_id "Novel_001350"; exon_number "2"; gene_type "
At_chr8	Cufflinks	exon	38087793	38088274	.	-	.	gene_id "XLOC_099982"; transcript_id "Novel_001351"; exon_number "1"; gene_type "
At_chr8	Cufflinks	exon	38089139	38089267	.	-	.	gene_id "XLOC_099982"; transcript_id "Novel_001351"; exon_number "2"; gene_type "
At_chr8	Cufflinks	exon	38089698	38090021	.	-	.	gene_id "XLOC_099982"; transcript_id "Novel_001351"; exon_number "3"; gene_type "
At_chr8	Cufflinks	exon	38087962	38088274	.	-	.	gene_id "XLOC_099982"; transcript_id "Novel_001352"; exon_number "1"; gene_type "
At_chr8	Cufflinks	exon	38089139	38090052	.	-	.	gene_id "XLOC_099982"; transcript_id "Novel_001352"; exon_number "2"; gene_type "
At_chr8	Cufflinks	exon	44797135	44798183	.	-	.	gene_id "XLOC_100810"; transcript_id "Novel_001353"; exon_number "1"; gene_type "
At_chr8	Cufflinks	exon	44798496	44798776	.	-	.	gene_id "XLOC_100810"; transcript_id "Novel_001353"; exon_number "2"; gene_type "
At_chr8	Cufflinks	exon	46514556	46515457	.	-	.	gene_id "XLOC_100929"; transcript_id "Novel_001354"; exon_number "1"; gene_type "
At_chr8	Cufflinks	exon	46515554	46515648	.	-	.	gene_id "XLOC_100929"; transcript_id "Novel_001354"; exon_number "2"; gene_type "
At_chr8	Cufflinks	exon	46515906	46516059	.	-	.	gene_id "XLOC_100929"; transcript_id "Novel_001354"; exon_number "3"; gene_type "
At_chr8	Cufflinks	exon	46516166	46516294	.	-	.	gene_id "XLOC_100929"; transcript_id "Novel_001354"; exon_number "4"; gene_type "
At_chr8	Cufflinks	exon	46516674	46516764	.	-	.	gene_id "XLOC_100929"; transcript_id "Novel_001354"; exon_number "5"; gene_type "
At_chr8	Cufflinks	exon	46517018	46517256	.	-	.	gene_id "XLOC_100929"; transcript_id "Novel_001354"; exon_number "6"; gene_type "
At_chr8	Cufflinks	exon	46517393	46517550	.	-	.	gene_id "XLOC_100929"; transcript_id "Novel_001354"; exon_number "7"; gene_type "
At_chr8	Cufflinks	exon	46517639	46517686	.	-	.	gene_id "XLOC_100929"; transcript_id "Novel_001354"; exon_number "8"; gene_type "
At_chr8	Cufflinks	exon	46517930	46517984	.	-	.	gene_id "XLOC_100929"; transcript_id "Novel_001354"; exon_number "9"; gene_type "
At_chr8	Cufflinks	exon	46518080	46518148	.	-	.	gene_id "XLOC_100929"; transcript_id "Novel_001354"; exon_number "10"; gene_type
At_chr8	Cufflinks	exon	46518428	46518534	.	-	.	gene_id "XLOC_100929"; transcript_id "Novel_001354"; exon_number "11"; gene_type
At_chr8	Cufflinks	exon	46518752	46518818	.	-	.	gene_id "XLOC_100929"; transcript_id "Novel_001354"; exon_number "12"; gene_type

At_chr8	Cufflinks	exon	46518913	46519265	.	-	.	gene_id "XLOC_100929"; transcript_id "Novel_001354"; exon_number "13"; gene_type
At_chr8	Cufflinks	exon	46514733	46515457	.	-	.	gene_id "XLOC_100929"; transcript_id "Novel_001355"; exon_number "1"; gene_type "
At_chr8	Cufflinks	exon	46515554	46515648	.	-	.	gene_id "XLOC_100929"; transcript_id "Novel_001355"; exon_number "2"; gene_type "
At_chr8	Cufflinks	exon	46515906	46516059	.	-	.	gene_id "XLOC_100929"; transcript_id "Novel_001355"; exon_number "3"; gene_type "
At_chr8	Cufflinks	exon	46516166	46516294	.	-	.	gene_id "XLOC_100929"; transcript_id "Novel_001355"; exon_number "4"; gene_type "
At_chr8	Cufflinks	exon	46517018	46517686	.	-	.	gene_id "XLOC_100929"; transcript_id "Novel_001355"; exon_number "5"; gene_type "
At_chr8	Cufflinks	exon	46517930	46517984	.	-	.	gene_id "XLOC_100929"; transcript_id "Novel_001355"; exon_number "6"; gene_type "
At_chr8	Cufflinks	exon	46518080	46518148	.	-	.	gene_id "XLOC_100929"; transcript_id "Novel_001355"; exon_number "7"; gene_type "
At_chr8	Cufflinks	exon	46518428	46518534	.	-	.	gene_id "XLOC_100929"; transcript_id "Novel_001355"; exon_number "8"; gene_type "
At_chr8	Cufflinks	exon	46518752	46518818	.	-	.	gene_id "XLOC_100929"; transcript_id "Novel_001355"; exon_number "9"; gene_type "
At_chr8	Cufflinks	exon	46518913	46519227	.	-	.	gene_id "XLOC_100929"; transcript_id "Novel_001355"; exon_number "10"; gene_type
At_chr8	Cufflinks	exon	46514871	46515457	.	-	.	gene_id "XLOC_100929"; transcript_id "Novel_001356"; exon_number "1"; gene_type "
At_chr8	Cufflinks	exon	46515554	46515648	.	-	.	gene_id "XLOC_100929"; transcript_id "Novel_001356"; exon_number "2"; gene_type "
At_chr8	Cufflinks	exon	46515906	46516059	.	-	.	gene_id "XLOC_100929"; transcript_id "Novel_001356"; exon_number "3"; gene_type "
At_chr8	Cufflinks	exon	46516166	46516294	.	-	.	gene_id "XLOC_100929"; transcript_id "Novel_001356"; exon_number "4"; gene_type "
At_chr8	Cufflinks	exon	46516674	46516764	.	-	.	gene_id "XLOC_100929"; transcript_id "Novel_001356"; exon_number "5"; gene_type "
At_chr8	Cufflinks	exon	46517018	46517550	.	-	.	gene_id "XLOC_100929"; transcript_id "Novel_001356"; exon_number "6"; gene_type "
At_chr8	Cufflinks	exon	46517639	46517686	.	-	.	gene_id "XLOC_100929"; transcript_id "Novel_001356"; exon_number "7"; gene_type "
At_chr8	Cufflinks	exon	46517930	46517984	.	-	.	gene_id "XLOC_100929"; transcript_id "Novel_001356"; exon_number "8"; gene_type "
At_chr8	Cufflinks	exon	46518080	46518148	.	-	.	gene_id "XLOC_100929"; transcript_id "Novel_001356"; exon_number "9"; gene_type "
At_chr8	Cufflinks	exon	46518428	46518534	.	-	.	gene_id "XLOC_100929"; transcript_id "Novel_001356"; exon_number "10"; gene_type
At_chr8	Cufflinks	exon	46518752	46518818	.	-	.	gene_id "XLOC_100929"; transcript_id "Novel_001356"; exon_number "11"; gene_type
At_chr8	Cufflinks	exon	46518913	46519228	.	-	.	gene_id "XLOC_100929"; transcript_id "Novel_001356"; exon_number "12"; gene_type
At_chr8	Cufflinks	exon	46515003	46515457	.	-	.	gene_id "XLOC_100929"; transcript_id "Novel_001357"; exon_number "1"; gene_type "
At_chr8	Cufflinks	exon	46515554	46515648	.	-	.	gene_id "XLOC_100929"; transcript_id "Novel_001357"; exon_number "2"; gene_type "
At_chr8	Cufflinks	exon	46515906	46516059	.	-	.	gene_id "XLOC_100929"; transcript_id "Novel_001357"; exon_number "3"; gene_type "
At_chr8	Cufflinks	exon	46516166	46516294	.	-	.	gene_id "XLOC_100929"; transcript_id "Novel_001357"; exon_number "4"; gene_type "
At_chr8	Cufflinks	exon	46516674	46516764	.	-	.	gene_id "XLOC_100929"; transcript_id "Novel_001357"; exon_number "5"; gene_type "
At_chr8	Cufflinks	exon	46517018	46517686	.	-	.	gene_id "XLOC_100929"; transcript_id "Novel_001357"; exon_number "6"; gene_type "
At_chr8	Cufflinks	exon	46517930	46517984	.	-	.	gene_id "XLOC_100929"; transcript_id "Novel_001357"; exon_number "7"; gene_type "
At_chr8	Cufflinks	exon	46518080	46518148	.	-	.	gene_id "XLOC_100929"; transcript_id "Novel_001357"; exon_number "8"; gene_type "
At_chr8	Cufflinks	exon	46518428	46518534	.	-	.	gene_id "XLOC_100929"; transcript_id "Novel_001357"; exon_number "9"; gene_type "
At_chr8	Cufflinks	exon	46518752	46518818	.	-	.	gene_id "XLOC_100929"; transcript_id "Novel_001357"; exon_number "10"; gene_type
At_chr8	Cufflinks	exon	46518913	46519264	.	-	.	gene_id "XLOC_100929"; transcript_id "Novel_001357"; exon_number "11"; gene_type
At_chr8	Cufflinks	exon	47767737	47767832	.	-	.	gene_id "XLOC_101032"; transcript_id "Novel_001358"; exon_number "1"; gene_type "

At_chr8	Cufflinks	exon	47768494	47768650	.	-	.	gene_id "XLOC_101032"; transcript_id "Novel_001358"; exon_number "2"; gene_type "
At_chr8	Cufflinks	exon	48102312	48102400	.	-	.	gene_id "XLOC_101043"; transcript_id "Novel_001359"; exon_number "1"; gene_type "
At_chr8	Cufflinks	exon	48102507	48102575	.	-	.	gene_id "XLOC_101043"; transcript_id "Novel_001359"; exon_number "2"; gene_type "
At_chr8	Cufflinks	exon	48102653	48102768	.	-	.	gene_id "XLOC_101043"; transcript_id "Novel_001359"; exon_number "3"; gene_type "
At_chr8	Cufflinks	exon	48103838	48103881	.	-	.	gene_id "XLOC_101044"; transcript_id "Novel_001360"; exon_number "1"; gene_type "
At_chr8	Cufflinks	exon	48103970	48104041	.	-	.	gene_id "XLOC_101044"; transcript_id "Novel_001360"; exon_number "2"; gene_type "
At_chr8	Cufflinks	exon	48104148	48104298	.	-	.	gene_id "XLOC_101044"; transcript_id "Novel_001360"; exon_number "3"; gene_type "
At_chr8	Cufflinks	exon	48104365	48104759	.	-	.	gene_id "XLOC_101044"; transcript_id "Novel_001360"; exon_number "4"; gene_type "
At_chr8	Cufflinks	exon	50067924	50068435	.	-	.	gene_id "XLOC_101153"; transcript_id "Novel_001361"; exon_number "1"; gene_type "
At_chr8	Cufflinks	exon	50068549	50068704	.	-	.	gene_id "XLOC_101153"; transcript_id "Novel_001361"; exon_number "2"; gene_type "
At_chr8	Cufflinks	exon	50068818	50068889	.	-	.	gene_id "XLOC_101153"; transcript_id "Novel_001361"; exon_number "3"; gene_type "
At_chr8	Cufflinks	exon	50069677	50069749	.	-	.	gene_id "XLOC_101153"; transcript_id "Novel_001361"; exon_number "4"; gene_type "
At_chr8	Cufflinks	exon	50070274	50070348	.	-	.	gene_id "XLOC_101153"; transcript_id "Novel_001361"; exon_number "5"; gene_type "
At_chr8	Cufflinks	exon	50070541	50070800	.	-	.	gene_id "XLOC_101153"; transcript_id "Novel_001361"; exon_number "6"; gene_type "
At_chr8	Cufflinks	exon	50068013	50068289	.	-	.	gene_id "XLOC_101153"; transcript_id "Novel_001362"; exon_number "1"; gene_type "
At_chr8	Cufflinks	exon	50068357	50068435	.	-	.	gene_id "XLOC_101153"; transcript_id "Novel_001362"; exon_number "2"; gene_type "
At_chr8	Cufflinks	exon	50068549	50068685	.	-	.	gene_id "XLOC_101153"; transcript_id "Novel_001362"; exon_number "3"; gene_type "
At_chr8	Cufflinks	exon	50068818	50068889	.	-	.	gene_id "XLOC_101153"; transcript_id "Novel_001362"; exon_number "4"; gene_type "
At_chr8	Cufflinks	exon	50069677	50069749	.	-	.	gene_id "XLOC_101153"; transcript_id "Novel_001362"; exon_number "5"; gene_type "
At_chr8	Cufflinks	exon	50070274	50070348	.	-	.	gene_id "XLOC_101153"; transcript_id "Novel_001362"; exon_number "6"; gene_type "
At_chr8	Cufflinks	exon	50070541	50070764	.	-	.	gene_id "XLOC_101153"; transcript_id "Novel_001362"; exon_number "7"; gene_type "
At_chr8	Cufflinks	exon	50068113	50068276	.	-	.	gene_id "XLOC_101153"; transcript_id "Novel_001363"; exon_number "1"; gene_type "
At_chr8	Cufflinks	exon	50068357	50068435	.	-	.	gene_id "XLOC_101153"; transcript_id "Novel_001363"; exon_number "2"; gene_type "
At_chr8	Cufflinks	exon	50068549	50069125	.	-	.	gene_id "XLOC_101153"; transcript_id "Novel_001363"; exon_number "3"; gene_type "
At_chr8	Cufflinks	exon	52932804	52933282	.	-	.	gene_id "XLOC_101233"; transcript_id "Novel_001364"; exon_number "1"; gene_type "
At_chr8	Cufflinks	exon	52933408	52933544	.	-	.	gene_id "XLOC_101233"; transcript_id "Novel_001364"; exon_number "2"; gene_type "
At_chr8	Cufflinks	exon	52934668	52934745	.	-	.	gene_id "XLOC_101233"; transcript_id "Novel_001364"; exon_number "3"; gene_type "
At_chr8	Cufflinks	exon	52932930	52933544	.	-	.	gene_id "XLOC_101233"; transcript_id "Novel_001365"; exon_number "1"; gene_type "
At_chr8	Cufflinks	exon	52934668	52934761	.	-	.	gene_id "XLOC_101233"; transcript_id "Novel_001365"; exon_number "2"; gene_type "
At_chr8	Cufflinks	exon	57461165	57461866	.	-	.	gene_id "XLOC_101384"; transcript_id "Novel_001366"; exon_number "1"; gene_type "
At_chr8	Cufflinks	exon	57462681	57462789	.	-	.	gene_id "XLOC_101384"; transcript_id "Novel_001366"; exon_number "2"; gene_type "
At_chr8	Cufflinks	exon	57536563	57538167	.	-	.	gene_id "XLOC_101397"; transcript_id "Novel_001367"; exon_number "1"; gene_type "
At_chr8	Cufflinks	exon	64513994	64514380	.	-	.	gene_id "XLOC_101583"; transcript_id "Novel_001368"; exon_number "1"; gene_type "
At_chr8	Cufflinks	exon	64514496	64514571	.	-	.	gene_id "XLOC_101583"; transcript_id "Novel_001368"; exon_number "2"; gene_type "
At_chr8	Cufflinks	exon	64514670	64514801	.	-	.	gene_id "XLOC_101583"; transcript_id "Novel_001368"; exon_number "3"; gene_type "

At_chr8	Cufflinks	exon	64515578	64515687	.	-	.	gene_id "XLOC_101583"; transcript_id "Novel_001368"; exon_number "4"; gene_type "
At_chr8	Cufflinks	exon	64515774	64515988	.	-	.	gene_id "XLOC_101583"; transcript_id "Novel_001368"; exon_number "5"; gene_type "
At_chr8	Cufflinks	exon	64516100	64516140	.	-	.	gene_id "XLOC_101583"; transcript_id "Novel_001368"; exon_number "6"; gene_type "
At_chr8	Cufflinks	exon	66033350	66033362	.	-	.	gene_id "XLOC_101635"; transcript_id "Novel_001369"; exon_number "1"; gene_type "
At_chr8	Cufflinks	exon	66034398	66035697	.	-	.	gene_id "XLOC_101635"; transcript_id "Novel_001369"; exon_number "2"; gene_type "
At_chr8	Cufflinks	exon	66036681	66037040	.	-	.	gene_id "XLOC_101635"; transcript_id "Novel_001369"; exon_number "3"; gene_type "
At_chr8	Cufflinks	exon	66037336	66037569	.	-	.	gene_id "XLOC_101635"; transcript_id "Novel_001369"; exon_number "4"; gene_type "
At_chr8	Cufflinks	exon	66037712	66037907	.	-	.	gene_id "XLOC_101635"; transcript_id "Novel_001369"; exon_number "5"; gene_type "
At_chr8	Cufflinks	exon	67200669	67200879	.	-	.	gene_id "XLOC_101667"; transcript_id "Novel_001370"; exon_number "1"; gene_type "
At_chr8	Cufflinks	exon	70819569	70822606	.	-	.	gene_id "XLOC_101837"; transcript_id "Novel_001371"; exon_number "1"; gene_type "
At_chr8	Cufflinks	exon	71512973	71513191	.	-	.	gene_id "XLOC_101859"; transcript_id "Novel_001372"; exon_number "1"; gene_type "
At_chr8	Cufflinks	exon	71513287	71513337	.	-	.	gene_id "XLOC_101859"; transcript_id "Novel_001372"; exon_number "2"; gene_type "
At_chr8	Cufflinks	exon	71656406	71657428	.	-	.	gene_id "XLOC_101874"; transcript_id "Novel_001373"; exon_number "1"; gene_type "
At_chr8	Cufflinks	exon	73544721	73545320	.	-	.	gene_id "XLOC_102053"; transcript_id "Novel_001374"; exon_number "1"; gene_type "
At_chr8	Cufflinks	exon	73545401	73545464	.	-	.	gene_id "XLOC_102053"; transcript_id "Novel_001374"; exon_number "2"; gene_type "
At_chr8	Cufflinks	exon	73545622	73545695	.	-	.	gene_id "XLOC_102053"; transcript_id "Novel_001374"; exon_number "3"; gene_type "
At_chr8	Cufflinks	exon	73546410	73546525	.	-	.	gene_id "XLOC_102053"; transcript_id "Novel_001374"; exon_number "4"; gene_type "
At_chr8	Cufflinks	exon	73546699	73546964	.	-	.	gene_id "XLOC_102053"; transcript_id "Novel_001374"; exon_number "5"; gene_type "
At_chr8	Cufflinks	exon	73548145	73548424	.	-	.	gene_id "XLOC_102053"; transcript_id "Novel_001374"; exon_number "6"; gene_type "
At_chr8	Cufflinks	exon	73545053	73545320	.	-	.	gene_id "XLOC_102053"; transcript_id "Novel_001375"; exon_number "1"; gene_type "
At_chr8	Cufflinks	exon	73545401	73545464	.	-	.	gene_id "XLOC_102053"; transcript_id "Novel_001375"; exon_number "2"; gene_type "
At_chr8	Cufflinks	exon	73545622	73545695	.	-	.	gene_id "XLOC_102053"; transcript_id "Novel_001375"; exon_number "3"; gene_type "
At_chr8	Cufflinks	exon	73546410	73546525	.	-	.	gene_id "XLOC_102053"; transcript_id "Novel_001375"; exon_number "4"; gene_type "
At_chr8	Cufflinks	exon	73546699	73547149	.	-	.	gene_id "XLOC_102053"; transcript_id "Novel_001375"; exon_number "5"; gene_type "
At_chr8	Cufflinks	exon	74776568	74776903	.	-	.	gene_id "XLOC_102147"; transcript_id "Novel_001376"; exon_number "1"; gene_type "
At_chr8	Cufflinks	exon	74777000	74777152	.	-	.	gene_id "XLOC_102147"; transcript_id "Novel_001376"; exon_number "2"; gene_type "
At_chr8	Cufflinks	exon	74777881	74777948	.	-	.	gene_id "XLOC_102147"; transcript_id "Novel_001376"; exon_number "3"; gene_type "
At_chr8	Cufflinks	exon	74778055	74778212	.	-	.	gene_id "XLOC_102147"; transcript_id "Novel_001376"; exon_number "4"; gene_type "
At_chr8	Cufflinks	exon	76826282	76827145	.	-	.	gene_id "XLOC_102311"; transcript_id "Novel_001377"; exon_number "1"; gene_type "
At_chr8	Cufflinks	exon	76827255	76827802	.	-	.	gene_id "XLOC_102311"; transcript_id "Novel_001377"; exon_number "2"; gene_type "
At_chr8	Cufflinks	exon	76826292	76827145	.	-	.	gene_id "XLOC_102311"; transcript_id "Novel_001378"; exon_number "1"; gene_type "
At_chr8	Cufflinks	exon	76827255	76827384	.	-	.	gene_id "XLOC_102311"; transcript_id "Novel_001378"; exon_number "2"; gene_type "
At_chr8	Cufflinks	exon	76827502	76827746	.	-	.	gene_id "XLOC_102311"; transcript_id "Novel_001378"; exon_number "3"; gene_type "
At_chr8	Cufflinks	exon	76826294	76827145	.	-	.	gene_id "XLOC_102311"; transcript_id "Novel_001379"; exon_number "1"; gene_type "
At_chr8	Cufflinks	exon	76827262	76827691	.	-	.	gene_id "XLOC_102311"; transcript_id "Novel_001379"; exon_number "2"; gene_type "

At_chr8	Cufflinks	exon	76895963	76897543	.	-	.	gene_id "XLOC_102313"; transcript_id "Novel_001380"; exon_number "1"; gene_type "
At_chr8	Cufflinks	exon	76898459	76898904	.	-	.	gene_id "XLOC_102313"; transcript_id "Novel_001380"; exon_number "2"; gene_type "
At_chr8	Cufflinks	exon	77720895	77722888	.	-	.	gene_id "XLOC_102381"; transcript_id "Novel_001381"; exon_number "1"; gene_type "
At_chr8	Cufflinks	exon	77723576	77723657	.	-	.	gene_id "XLOC_102381"; transcript_id "Novel_001381"; exon_number "2"; gene_type "
At_chr8	Cufflinks	exon	78401401	78404503	.	-	.	gene_id "XLOC_102450"; transcript_id "Novel_001382"; exon_number "1"; gene_type "
At_chr8	Cufflinks	exon	78404824	78405919	.	-	.	gene_id "XLOC_102450"; transcript_id "Novel_001382"; exon_number "2"; gene_type "
At_chr9	Cufflinks	exon	769007	769369	.	+	.	gene_id "XLOC_102575"; transcript_id "Novel_001383"; exon_number "1"; gene_type "
At_chr9	Cufflinks	exon	771367	771503	.	+	.	gene_id "XLOC_102575"; transcript_id "Novel_001383"; exon_number "2"; gene_type "
At_chr9	Cufflinks	exon	771619	771721	.	+	.	gene_id "XLOC_102575"; transcript_id "Novel_001383"; exon_number "3"; gene_type "
At_chr9	Cufflinks	exon	771840	771934	.	+	.	gene_id "XLOC_102575"; transcript_id "Novel_001383"; exon_number "4"; gene_type "
At_chr9	Cufflinks	exon	772655	772731	.	+	.	gene_id "XLOC_102575"; transcript_id "Novel_001383"; exon_number "5"; gene_type "
At_chr9	Cufflinks	exon	774168	775069	.	+	.	gene_id "XLOC_102575"; transcript_id "Novel_001383"; exon_number "6"; gene_type "
At_chr9	Cufflinks	exon	771324	771503	.	+	.	gene_id "XLOC_102575"; transcript_id "Novel_001384"; exon_number "1"; gene_type "
At_chr9	Cufflinks	exon	771619	771721	.	+	.	gene_id "XLOC_102575"; transcript_id "Novel_001384"; exon_number "2"; gene_type "
At_chr9	Cufflinks	exon	771840	771934	.	+	.	gene_id "XLOC_102575"; transcript_id "Novel_001384"; exon_number "3"; gene_type "
At_chr9	Cufflinks	exon	772655	772731	.	+	.	gene_id "XLOC_102575"; transcript_id "Novel_001384"; exon_number "4"; gene_type "
At_chr9	Cufflinks	exon	774168	775069	.	+	.	gene_id "XLOC_102575"; transcript_id "Novel_001384"; exon_number "5"; gene_type "
At_chr9	Cufflinks	exon	3073886	3074030	.	+	.	gene_id "XLOC_102742"; transcript_id "Novel_001385"; exon_number "1"; gene_type "
At_chr9	Cufflinks	exon	3074132	3074247	.	+	.	gene_id "XLOC_102742"; transcript_id "Novel_001385"; exon_number "2"; gene_type "
At_chr9	Cufflinks	exon	3075266	3076286	.	+	.	gene_id "XLOC_102742"; transcript_id "Novel_001385"; exon_number "3"; gene_type "
At_chr9	Cufflinks	exon	7993261	7993337	.	+	.	gene_id "XLOC_102945"; transcript_id "Novel_001386"; exon_number "1"; gene_type "
At_chr9	Cufflinks	exon	7993945	7994192	.	+	.	gene_id "XLOC_102945"; transcript_id "Novel_001386"; exon_number "2"; gene_type "
At_chr9	Cufflinks	exon	7994876	7995575	.	+	.	gene_id "XLOC_102945"; transcript_id "Novel_001386"; exon_number "3"; gene_type "
At_chr9	Cufflinks	exon	7993270	7993337	.	+	.	gene_id "XLOC_102945"; transcript_id "Novel_001387"; exon_number "1"; gene_type "
At_chr9	Cufflinks	exon	7993945	7994246	.	+	.	gene_id "XLOC_102945"; transcript_id "Novel_001387"; exon_number "2"; gene_type "
At_chr9	Cufflinks	exon	8146574	8147093	.	+	.	gene_id "XLOC_102957"; transcript_id "Novel_001388"; exon_number "1"; gene_type "
At_chr9	Cufflinks	exon	8147762	8149100	.	+	.	gene_id "XLOC_102957"; transcript_id "Novel_001388"; exon_number "2"; gene_type "
At_chr9	Cufflinks	exon	8146574	8147093	.	+	.	gene_id "XLOC_102957"; transcript_id "Novel_001389"; exon_number "1"; gene_type "
At_chr9	Cufflinks	exon	8147833	8149100	.	+	.	gene_id "XLOC_102957"; transcript_id "Novel_001389"; exon_number "2"; gene_type "
At_chr9	Cufflinks	exon	8146574	8147093	.	+	.	gene_id "XLOC_102957"; transcript_id "Novel_001390"; exon_number "1"; gene_type "
At_chr9	Cufflinks	exon	8147824	8149100	.	+	.	gene_id "XLOC_102957"; transcript_id "Novel_001390"; exon_number "2"; gene_type "
At_chr9	Cufflinks	exon	8330098	8330745	.	+	.	gene_id "XLOC_102966"; transcript_id "Novel_001391"; exon_number "1"; gene_type "
At_chr9	Cufflinks	exon	8330816	8331795	.	+	.	gene_id "XLOC_102966"; transcript_id "Novel_001391"; exon_number "2"; gene_type "
At_chr9	Cufflinks	exon	8330098	8330745	.	+	.	gene_id "XLOC_102966"; transcript_id "Novel_001392"; exon_number "1"; gene_type "
At_chr9	Cufflinks	exon	8330816	8330921	.	+	.	gene_id "XLOC_102966"; transcript_id "Novel_001392"; exon_number "2"; gene_type "

At_chr9	Cufflinks	exon	8331004	8331795	.	+	.	gene_id "XLOC_102966"; transcript_id "Novel_001392"; exon_number "3"; gene_type "
At_chr9	Cufflinks	exon	8394327	8397266	.	+	.	gene_id "XLOC_102970"; transcript_id "Novel_001393"; exon_number "1"; gene_type "
At_chr9	Cufflinks	exon	10231322	10231762	.	+	.	gene_id "XLOC_103018"; transcript_id "Novel_001394"; exon_number "1"; gene_type "
At_chr9	Cufflinks	exon	10242897	10243127	.	+	.	gene_id "XLOC_103020"; transcript_id "Novel_001395"; exon_number "1"; gene_type "
At_chr9	Cufflinks	exon	11909417	11909875	.	+	.	gene_id "XLOC_103076"; transcript_id "Novel_001396"; exon_number "1"; gene_type "
At_chr9	Cufflinks	exon	11910518	11914222	.	+	.	gene_id "XLOC_103076"; transcript_id "Novel_001396"; exon_number "2"; gene_type "
At_chr9	Cufflinks	exon	19649332	19649503	.	+	.	gene_id "XLOC_103332"; transcript_id "Novel_001397"; exon_number "1"; gene_type "
At_chr9	Cufflinks	exon	19650506	19650664	.	+	.	gene_id "XLOC_103332"; transcript_id "Novel_001397"; exon_number "2"; gene_type "
At_chr9	Cufflinks	exon	19651135	19651188	.	+	.	gene_id "XLOC_103332"; transcript_id "Novel_001397"; exon_number "3"; gene_type "
At_chr9	Cufflinks	exon	19651274	19651354	.	+	.	gene_id "XLOC_103332"; transcript_id "Novel_001397"; exon_number "4"; gene_type "
At_chr9	Cufflinks	exon	19656373	19656510	.	+	.	gene_id "XLOC_103332"; transcript_id "Novel_001397"; exon_number "5"; gene_type "
At_chr9	Cufflinks	exon	19657604	19657763	.	+	.	gene_id "XLOC_103332"; transcript_id "Novel_001397"; exon_number "6"; gene_type "
At_chr9	Cufflinks	exon	19659750	19659829	.	+	.	gene_id "XLOC_103332"; transcript_id "Novel_001397"; exon_number "7"; gene_type "
At_chr9	Cufflinks	exon	19659961	19660106	.	+	.	gene_id "XLOC_103332"; transcript_id "Novel_001397"; exon_number "8"; gene_type "
At_chr9	Cufflinks	exon	19660244	19660301	.	+	.	gene_id "XLOC_103332"; transcript_id "Novel_001397"; exon_number "9"; gene_type "
At_chr9	Cufflinks	exon	19660384	19660782	.	+	.	gene_id "XLOC_103332"; transcript_id "Novel_001397"; exon_number "10"; gene_type
At_chr9	Cufflinks	exon	22095568	22099019	.	+	.	gene_id "XLOC_103429"; transcript_id "Novel_001398"; exon_number "1"; gene_type "
At_chr9	Cufflinks	exon	22608985	22609224	.	+	.	gene_id "XLOC_103520"; transcript_id "Novel_001399"; exon_number "1"; gene_type "
At_chr9	Cufflinks	exon	22609475	22609507	.	+	.	gene_id "XLOC_103520"; transcript_id "Novel_001399"; exon_number "2"; gene_type "
At_chr9	Cufflinks	exon	22610134	22610244	.	+	.	gene_id "XLOC_103520"; transcript_id "Novel_001399"; exon_number "3"; gene_type "
At_chr9	Cufflinks	exon	22610330	22610425	.	+	.	gene_id "XLOC_103520"; transcript_id "Novel_001399"; exon_number "4"; gene_type "
At_chr9	Cufflinks	exon	22610820	22610894	.	+	.	gene_id "XLOC_103520"; transcript_id "Novel_001399"; exon_number "5"; gene_type "
At_chr9	Cufflinks	exon	22610982	22611298	.	+	.	gene_id "XLOC_103520"; transcript_id "Novel_001399"; exon_number "6"; gene_type "
At_chr9	Cufflinks	exon	22608985	22609224	.	+	.	gene_id "XLOC_103520"; transcript_id "Novel_001400"; exon_number "1"; gene_type "
At_chr9	Cufflinks	exon	22609475	22609507	.	+	.	gene_id "XLOC_103520"; transcript_id "Novel_001400"; exon_number "2"; gene_type "
At_chr9	Cufflinks	exon	22610128	22610244	.	+	.	gene_id "XLOC_103520"; transcript_id "Novel_001400"; exon_number "3"; gene_type "
At_chr9	Cufflinks	exon	22610330	22610425	.	+	.	gene_id "XLOC_103520"; transcript_id "Novel_001400"; exon_number "4"; gene_type "
At_chr9	Cufflinks	exon	22610820	22610894	.	+	.	gene_id "XLOC_103520"; transcript_id "Novel_001400"; exon_number "5"; gene_type "
At_chr9	Cufflinks	exon	22610982	22611298	.	+	.	gene_id "XLOC_103520"; transcript_id "Novel_001400"; exon_number "6"; gene_type "
At_chr9	Cufflinks	exon	23010546	23010923	.	+	.	gene_id "XLOC_103548"; transcript_id "Novel_001401"; exon_number "1"; gene_type "
At_chr9	Cufflinks	exon	23011039	23011082	.	+	.	gene_id "XLOC_103548"; transcript_id "Novel_001401"; exon_number "2"; gene_type "
At_chr9	Cufflinks	exon	23011246	23011313	.	+	.	gene_id "XLOC_103548"; transcript_id "Novel_001401"; exon_number "3"; gene_type "
At_chr9	Cufflinks	exon	23012497	23013436	.	+	.	gene_id "XLOC_103548"; transcript_id "Novel_001401"; exon_number "4"; gene_type "
At_chr9	Cufflinks	exon	23494828	23495134	.	+	.	gene_id "XLOC_103570"; transcript_id "Novel_001402"; exon_number "1"; gene_type "
At_chr9	Cufflinks	exon	23495211	23495297	.	+	.	gene_id "XLOC_103570"; transcript_id "Novel_001402"; exon_number "2"; gene_type "

At_chr9	Cufflinks	exon	23495465	23495516	.	+	.	gene_id "XLOC_103570"; transcript_id "Novel_001402"; exon_number "3"; gene_type "
At_chr9	Cufflinks	exon	23497535	23499743	.	+	.	gene_id "XLOC_103570"; transcript_id "Novel_001402"; exon_number "4"; gene_type "
At_chr9	Cufflinks	exon	28235896	28236105	.	+	.	gene_id "XLOC_103735"; transcript_id "Novel_001403"; exon_number "1"; gene_type "
At_chr9	Cufflinks	exon	28682950	28683220	.	+	.	gene_id "XLOC_103750"; transcript_id "Novel_001404"; exon_number "1"; gene_type "
At_chr9	Cufflinks	exon	28683758	28686193	.	+	.	gene_id "XLOC_103750"; transcript_id "Novel_001404"; exon_number "2"; gene_type "
At_chr9	Cufflinks	exon	28682956	28683220	.	+	.	gene_id "XLOC_103750"; transcript_id "Novel_001405"; exon_number "1"; gene_type "
At_chr9	Cufflinks	exon	28683736	28686018	.	+	.	gene_id "XLOC_103750"; transcript_id "Novel_001405"; exon_number "2"; gene_type "
At_chr9	Cufflinks	exon	32493422	32493639	.	+	.	gene_id "XLOC_104050"; transcript_id "Novel_001406"; exon_number "1"; gene_type "
At_chr9	Cufflinks	exon	32811555	32812043	.	+	.	gene_id "XLOC_104111"; transcript_id "Novel_001407"; exon_number "1"; gene_type "
At_chr9	Cufflinks	exon	32812123	32812496	.	+	.	gene_id "XLOC_104111"; transcript_id "Novel_001407"; exon_number "2"; gene_type "
At_chr9	Cufflinks	exon	32811559	32812037	.	+	.	gene_id "XLOC_104111"; transcript_id "Novel_001408"; exon_number "1"; gene_type "
At_chr9	Cufflinks	exon	32812123	32812472	.	+	.	gene_id "XLOC_104111"; transcript_id "Novel_001408"; exon_number "2"; gene_type "
At_chr9	Cufflinks	exon	32850918	32851256	.	+	.	gene_id "XLOC_104118"; transcript_id "Novel_001409"; exon_number "1"; gene_type "
At_chr9	Cufflinks	exon	32851348	32851436	.	+	.	gene_id "XLOC_104118"; transcript_id "Novel_001409"; exon_number "2"; gene_type "
At_chr9	Cufflinks	exon	32853078	32853148	.	+	.	gene_id "XLOC_104118"; transcript_id "Novel_001409"; exon_number "3"; gene_type "
At_chr9	Cufflinks	exon	32853248	32853295	.	+	.	gene_id "XLOC_104118"; transcript_id "Novel_001409"; exon_number "4"; gene_type "
At_chr9	Cufflinks	exon	33443504	33443770	.	+	.	gene_id "XLOC_104198"; transcript_id "Novel_001410"; exon_number "1"; gene_type "
At_chr9	Cufflinks	exon	33443863	33443901	.	+	.	gene_id "XLOC_104198"; transcript_id "Novel_001410"; exon_number "2"; gene_type "
At_chr9	Cufflinks	exon	33444726	33444817	.	+	.	gene_id "XLOC_104198"; transcript_id "Novel_001410"; exon_number "3"; gene_type "
At_chr9	Cufflinks	exon	33445871	33446120	.	+	.	gene_id "XLOC_104198"; transcript_id "Novel_001410"; exon_number "4"; gene_type "
At_chr9	Cufflinks	exon	33446597	33447298	.	+	.	gene_id "XLOC_104198"; transcript_id "Novel_001410"; exon_number "5"; gene_type "
At_chr9	Cufflinks	exon	33447462	33447570	.	+	.	gene_id "XLOC_104198"; transcript_id "Novel_001410"; exon_number "6"; gene_type "
At_chr9	Cufflinks	exon	33449630	33449722	.	+	.	gene_id "XLOC_104198"; transcript_id "Novel_001410"; exon_number "7"; gene_type "
At_chr9	Cufflinks	exon	33451242	33451402	.	+	.	gene_id "XLOC_104198"; transcript_id "Novel_001410"; exon_number "8"; gene_type "
At_chr9	Cufflinks	exon	33452025	33453092	.	+	.	gene_id "XLOC_104198"; transcript_id "Novel_001410"; exon_number "9"; gene_type "
At_chr9	Cufflinks	exon	33443524	33443770	.	+	.	gene_id "XLOC_104198"; transcript_id "Novel_001411"; exon_number "1"; gene_type "
At_chr9	Cufflinks	exon	33443863	33444644	.	+	.	gene_id "XLOC_104198"; transcript_id "Novel_001411"; exon_number "2"; gene_type "
At_chr9	Cufflinks	exon	37136874	37137953	.	+	.	gene_id "XLOC_104567"; transcript_id "Novel_001412"; exon_number "1"; gene_type "
At_chr9	Cufflinks	exon	37138385	37138657	.	+	.	gene_id "XLOC_104567"; transcript_id "Novel_001412"; exon_number "2"; gene_type "
At_chr9	Cufflinks	exon	37139085	37139347	.	+	.	gene_id "XLOC_104567"; transcript_id "Novel_001412"; exon_number "3"; gene_type "
At_chr9	Cufflinks	exon	37140825	37141417	.	+	.	gene_id "XLOC_104567"; transcript_id "Novel_001412"; exon_number "4"; gene_type "
At_chr9	Cufflinks	exon	37136874	37137698	.	+	.	gene_id "XLOC_104567"; transcript_id "Novel_001413"; exon_number "1"; gene_type "
At_chr9	Cufflinks	exon	37137826	37137953	.	+	.	gene_id "XLOC_104567"; transcript_id "Novel_001413"; exon_number "2"; gene_type "
At_chr9	Cufflinks	exon	37138385	37138657	.	+	.	gene_id "XLOC_104567"; transcript_id "Novel_001413"; exon_number "3"; gene_type "
At_chr9	Cufflinks	exon	37139085	37139347	.	+	.	gene_id "XLOC_104567"; transcript_id "Novel_001413"; exon_number "4"; gene_type "

At_chr9	Cufflinks	exon	37140825	37141417	.	+	.	gene_id "XLOC_104567"; transcript_id "Novel_001413"; exon_number "5"; gene_type "
At_chr9	Cufflinks	exon	37234260	37234361	.	+	.	gene_id "XLOC_104577"; transcript_id "Novel_001414"; exon_number "1"; gene_type "
At_chr9	Cufflinks	exon	37234587	37234692	.	+	.	gene_id "XLOC_104577"; transcript_id "Novel_001414"; exon_number "2"; gene_type "
At_chr9	Cufflinks	exon	37299744	37299827	.	+	.	gene_id "XLOC_104591"; transcript_id "Novel_001415"; exon_number "1"; gene_type "
At_chr9	Cufflinks	exon	37300996	37301182	.	+	.	gene_id "XLOC_104591"; transcript_id "Novel_001415"; exon_number "2"; gene_type "
At_chr9	Cufflinks	exon	37301276	37301441	.	+	.	gene_id "XLOC_104591"; transcript_id "Novel_001415"; exon_number "3"; gene_type "
At_chr9	Cufflinks	exon	37301549	37301586	.	+	.	gene_id "XLOC_104591"; transcript_id "Novel_001415"; exon_number "4"; gene_type "
At_chr9	Cufflinks	exon	37301674	37301725	.	+	.	gene_id "XLOC_104591"; transcript_id "Novel_001415"; exon_number "5"; gene_type "
At_chr9	Cufflinks	exon	37301850	37302159	.	+	.	gene_id "XLOC_104591"; transcript_id "Novel_001415"; exon_number "6"; gene_type "
At_chr9	Cufflinks	exon	40707305	40707330	.	+	.	gene_id "XLOC_105070"; transcript_id "Novel_001416"; exon_number "1"; gene_type "
At_chr9	Cufflinks	exon	40707427	40707604	.	+	.	gene_id "XLOC_105070"; transcript_id "Novel_001416"; exon_number "2"; gene_type "
At_chr9	Cufflinks	exon	41983414	41983473	.	+	.	gene_id "XLOC_105273"; transcript_id "Novel_001417"; exon_number "1"; gene_type "
At_chr9	Cufflinks	exon	41983606	41984785	.	+	.	gene_id "XLOC_105273"; transcript_id "Novel_001417"; exon_number "2"; gene_type "
At_chr9	Cufflinks	exon	42726943	42729307	.	+	.	gene_id "XLOC_105389"; transcript_id "Novel_001418"; exon_number "1"; gene_type "
At_chr9	Cufflinks	exon	42729602	42730334	.	+	.	gene_id "XLOC_105389"; transcript_id "Novel_001418"; exon_number "2"; gene_type "
At_chr9	Cufflinks	exon	42726960	42729181	.	+	.	gene_id "XLOC_105389"; transcript_id "Novel_001419"; exon_number "1"; gene_type "
At_chr9	Cufflinks	exon	42729278	42729307	.	+	.	gene_id "XLOC_105389"; transcript_id "Novel_001419"; exon_number "2"; gene_type "
At_chr9	Cufflinks	exon	42729602	42729795	.	+	.	gene_id "XLOC_105389"; transcript_id "Novel_001419"; exon_number "3"; gene_type "
At_chr9	Cufflinks	exon	43713785	43714356	.	+	.	gene_id "XLOC_105579"; transcript_id "Novel_001420"; exon_number "1"; gene_type "
At_chr9	Cufflinks	exon	43715413	43715682	.	+	.	gene_id "XLOC_105579"; transcript_id "Novel_001420"; exon_number "2"; gene_type "
At_chr9	Cufflinks	exon	43715776	43716621	.	+	.	gene_id "XLOC_105579"; transcript_id "Novel_001420"; exon_number "3"; gene_type "
At_chr9	Cufflinks	exon	43972636	43973447	.	+	.	gene_id "XLOC_105607"; transcript_id "Novel_001421"; exon_number "1"; gene_type "
At_chr9	Cufflinks	exon	43973799	43973861	.	+	.	gene_id "XLOC_105607"; transcript_id "Novel_001421"; exon_number "2"; gene_type "
At_chr9	Cufflinks	exon	43973962	43974278	.	+	.	gene_id "XLOC_105607"; transcript_id "Novel_001421"; exon_number "3"; gene_type "
At_chr9	Cufflinks	exon	43974447	43974573	.	+	.	gene_id "XLOC_105607"; transcript_id "Novel_001421"; exon_number "4"; gene_type "
At_chr9	Cufflinks	exon	43972948	43973447	.	+	.	gene_id "XLOC_105607"; transcript_id "Novel_001422"; exon_number "1"; gene_type "
At_chr9	Cufflinks	exon	43973799	43973861	.	+	.	gene_id "XLOC_105607"; transcript_id "Novel_001422"; exon_number "2"; gene_type "
At_chr9	Cufflinks	exon	43973962	43974039	.	+	.	gene_id "XLOC_105607"; transcript_id "Novel_001422"; exon_number "3"; gene_type "
At_chr9	Cufflinks	exon	43974116	43974278	.	+	.	gene_id "XLOC_105607"; transcript_id "Novel_001422"; exon_number "4"; gene_type "
At_chr9	Cufflinks	exon	43974447	43974907	.	+	.	gene_id "XLOC_105607"; transcript_id "Novel_001422"; exon_number "5"; gene_type "
At_chr9	Cufflinks	exon	44489424	44489873	.	+	.	gene_id "XLOC_105709"; transcript_id "Novel_001423"; exon_number "1"; gene_type "
At_chr9	Cufflinks	exon	44489966	44490142	.	+	.	gene_id "XLOC_105709"; transcript_id "Novel_001423"; exon_number "2"; gene_type "
At_chr9	Cufflinks	exon	44490252	44490476	.	+	.	gene_id "XLOC_105709"; transcript_id "Novel_001423"; exon_number "3"; gene_type "
At_chr9	Cufflinks	exon	44490588	44490716	.	+	.	gene_id "XLOC_105709"; transcript_id "Novel_001423"; exon_number "4"; gene_type "
At_chr9	Cufflinks	exon	44490933	44491487	.	+	.	gene_id "XLOC_105709"; transcript_id "Novel_001423"; exon_number "5"; gene_type "

At_chr9	Cufflinks	exon	45262486	45262720	.	+	.	gene_id "XLOC_105865"; transcript_id "Novel_001424"; exon_number "1"; gene_type "
At_chr9	Cufflinks	exon	45265078	45265312	.	+	.	gene_id "XLOC_105866"; transcript_id "Novel_001425"; exon_number "1"; gene_type "
At_chr9	Cufflinks	exon	45399473	45399903	.	+	.	gene_id "XLOC_105895"; transcript_id "Novel_001426"; exon_number "1"; gene_type "
At_chr9	Cufflinks	exon	45400703	45400989	.	+	.	gene_id "XLOC_105895"; transcript_id "Novel_001426"; exon_number "2"; gene_type "
At_chr9	Cufflinks	exon	45399654	45399681	.	+	.	gene_id "XLOC_105895"; transcript_id "Novel_001427"; exon_number "1"; gene_type "
At_chr9	Cufflinks	exon	45399757	45399903	.	+	.	gene_id "XLOC_105895"; transcript_id "Novel_001427"; exon_number "2"; gene_type "
At_chr9	Cufflinks	exon	45400703	45401034	.	+	.	gene_id "XLOC_105895"; transcript_id "Novel_001427"; exon_number "3"; gene_type "
At_chr9	Cufflinks	exon	45402419	45402705	.	+	.	gene_id "XLOC_105897"; transcript_id "Novel_001428"; exon_number "1"; gene_type "
At_chr9	Cufflinks	exon	45403693	45403890	.	+	.	gene_id "XLOC_105897"; transcript_id "Novel_001428"; exon_number "2"; gene_type "
At_chr9	Cufflinks	exon	45404038	45404186	.	+	.	gene_id "XLOC_105897"; transcript_id "Novel_001428"; exon_number "3"; gene_type "
At_chr9	Cufflinks	exon	45404302	45405223	.	+	.	gene_id "XLOC_105897"; transcript_id "Novel_001428"; exon_number "4"; gene_type "
At_chr9	Cufflinks	exon	45402481	45402705	.	+	.	gene_id "XLOC_105897"; transcript_id "Novel_001429"; exon_number "1"; gene_type "
At_chr9	Cufflinks	exon	45403693	45404186	.	+	.	gene_id "XLOC_105897"; transcript_id "Novel_001429"; exon_number "2"; gene_type "
At_chr9	Cufflinks	exon	45404302	45404997	.	+	.	gene_id "XLOC_105897"; transcript_id "Novel_001429"; exon_number "3"; gene_type "
At_chr9	Cufflinks	exon	47247139	47247536	.	+	.	gene_id "XLOC_106198"; transcript_id "Novel_001430"; exon_number "1"; gene_type "
At_chr9	Cufflinks	exon	48066313	48066480	.	+	.	gene_id "XLOC_106290"; transcript_id "Novel_001431"; exon_number "1"; gene_type "
At_chr9	Cufflinks	exon	48066567	48066634	.	+	.	gene_id "XLOC_106290"; transcript_id "Novel_001431"; exon_number "2"; gene_type "
At_chr9	Cufflinks	exon	48067095	48067205	.	+	.	gene_id "XLOC_106290"; transcript_id "Novel_001431"; exon_number "3"; gene_type "
At_chr9	Cufflinks	exon	48067606	48067692	.	+	.	gene_id "XLOC_106290"; transcript_id "Novel_001431"; exon_number "4"; gene_type "
At_chr9	Cufflinks	exon	48067836	48068044	.	+	.	gene_id "XLOC_106290"; transcript_id "Novel_001431"; exon_number "5"; gene_type "
At_chr9	Cufflinks	exon	48068132	48068664	.	+	.	gene_id "XLOC_106290"; transcript_id "Novel_001431"; exon_number "6"; gene_type "
At_chr9	Cufflinks	exon	49168173	49168641	.	+	.	gene_id "XLOC_106454"; transcript_id "Novel_001432"; exon_number "1"; gene_type "
At_chr9	Cufflinks	exon	49169339	49169410	.	+	.	gene_id "XLOC_106454"; transcript_id "Novel_001432"; exon_number "2"; gene_type "
At_chr9	Cufflinks	exon	49169692	49169787	.	+	.	gene_id "XLOC_106454"; transcript_id "Novel_001432"; exon_number "3"; gene_type "
At_chr9	Cufflinks	exon	49169942	49170166	.	+	.	gene_id "XLOC_106454"; transcript_id "Novel_001432"; exon_number "4"; gene_type "
At_chr9	Cufflinks	exon	49170732	49170829	.	+	.	gene_id "XLOC_106454"; transcript_id "Novel_001432"; exon_number "5"; gene_type "
At_chr9	Cufflinks	exon	49171084	49171171	.	+	.	gene_id "XLOC_106454"; transcript_id "Novel_001432"; exon_number "6"; gene_type "
At_chr9	Cufflinks	exon	49171378	49171716	.	+	.	gene_id "XLOC_106454"; transcript_id "Novel_001432"; exon_number "7"; gene_type "
At_chr9	Cufflinks	exon	49171811	49172053	.	+	.	gene_id "XLOC_106454"; transcript_id "Novel_001432"; exon_number "8"; gene_type "
At_chr9	Cufflinks	exon	49172156	49173764	.	+	.	gene_id "XLOC_106454"; transcript_id "Novel_001432"; exon_number "9"; gene_type "
At_chr9	Cufflinks	exon	49168184	49168641	.	+	.	gene_id "XLOC_106454"; transcript_id "Novel_001433"; exon_number "1"; gene_type "
At_chr9	Cufflinks	exon	49169339	49169410	.	+	.	gene_id "XLOC_106454"; transcript_id "Novel_001433"; exon_number "2"; gene_type "
At_chr9	Cufflinks	exon	49169692	49169787	.	+	.	gene_id "XLOC_106454"; transcript_id "Novel_001433"; exon_number "3"; gene_type "
At_chr9	Cufflinks	exon	49169954	49170166	.	+	.	gene_id "XLOC_106454"; transcript_id "Novel_001433"; exon_number "4"; gene_type "
At_chr9	Cufflinks	exon	49170732	49170829	.	+	.	gene_id "XLOC_106454"; transcript_id "Novel_001433"; exon_number "5"; gene_type "

At_chr9	Cufflinks	exon	49171084	49171171	.	+	.	gene_id "XLOC_106454"; transcript_id "Novel_001433"; exon_number "6"; gene_type "
At_chr9	Cufflinks	exon	49171378	49171716	.	+	.	gene_id "XLOC_106454"; transcript_id "Novel_001433"; exon_number "7"; gene_type "
At_chr9	Cufflinks	exon	49171811	49172053	.	+	.	gene_id "XLOC_106454"; transcript_id "Novel_001433"; exon_number "8"; gene_type "
At_chr9	Cufflinks	exon	49172156	49173685	.	+	.	gene_id "XLOC_106454"; transcript_id "Novel_001433"; exon_number "9"; gene_type "
At_chr9	Cufflinks	exon	49168187	49168641	.	+	.	gene_id "XLOC_106454"; transcript_id "Novel_001434"; exon_number "1"; gene_type "
At_chr9	Cufflinks	exon	49169339	49169410	.	+	.	gene_id "XLOC_106454"; transcript_id "Novel_001434"; exon_number "2"; gene_type "
At_chr9	Cufflinks	exon	49169692	49169787	.	+	.	gene_id "XLOC_106454"; transcript_id "Novel_001434"; exon_number "3"; gene_type "
At_chr9	Cufflinks	exon	49169942	49170166	.	+	.	gene_id "XLOC_106454"; transcript_id "Novel_001434"; exon_number "4"; gene_type "
At_chr9	Cufflinks	exon	49170732	49170829	.	+	.	gene_id "XLOC_106454"; transcript_id "Novel_001434"; exon_number "5"; gene_type "
At_chr9	Cufflinks	exon	49171084	49171171	.	+	.	gene_id "XLOC_106454"; transcript_id "Novel_001434"; exon_number "6"; gene_type "
At_chr9	Cufflinks	exon	49171378	49172053	.	+	.	gene_id "XLOC_106454"; transcript_id "Novel_001434"; exon_number "7"; gene_type "
At_chr9	Cufflinks	exon	49172156	49173511	.	+	.	gene_id "XLOC_106454"; transcript_id "Novel_001434"; exon_number "8"; gene_type "
At_chr9	Cufflinks	exon	49175266	49176376	.	+	.	gene_id "XLOC_106456"; transcript_id "Novel_001435"; exon_number "1"; gene_type "
At_chr9	Cufflinks	exon	49176483	49176777	.	+	.	gene_id "XLOC_106456"; transcript_id "Novel_001435"; exon_number "2"; gene_type "
At_chr9	Cufflinks	exon	49176919	49177228	.	+	.	gene_id "XLOC_106456"; transcript_id "Novel_001435"; exon_number "3"; gene_type "
At_chr9	Cufflinks	exon	49177721	49177878	.	+	.	gene_id "XLOC_106456"; transcript_id "Novel_001435"; exon_number "4"; gene_type "
At_chr9	Cufflinks	exon	49178023	49178695	.	+	.	gene_id "XLOC_106456"; transcript_id "Novel_001435"; exon_number "5"; gene_type "
At_chr9	Cufflinks	exon	49175266	49176376	.	+	.	gene_id "XLOC_106456"; transcript_id "Novel_001436"; exon_number "1"; gene_type "
At_chr9	Cufflinks	exon	49176483	49176777	.	+	.	gene_id "XLOC_106456"; transcript_id "Novel_001436"; exon_number "2"; gene_type "
At_chr9	Cufflinks	exon	49176919	49177228	.	+	.	gene_id "XLOC_106456"; transcript_id "Novel_001436"; exon_number "3"; gene_type "
At_chr9	Cufflinks	exon	49177721	49178695	.	+	.	gene_id "XLOC_106456"; transcript_id "Novel_001436"; exon_number "4"; gene_type "
At_chr9	Cufflinks	exon	49175268	49176376	.	+	.	gene_id "XLOC_106456"; transcript_id "Novel_001437"; exon_number "1"; gene_type "
At_chr9	Cufflinks	exon	49176483	49177228	.	+	.	gene_id "XLOC_106456"; transcript_id "Novel_001437"; exon_number "2"; gene_type "
At_chr9	Cufflinks	exon	49177721	49178013	.	+	.	gene_id "XLOC_106456"; transcript_id "Novel_001437"; exon_number "3"; gene_type "
At_chr9	Cufflinks	exon	49179068	49180397	.	+	.	gene_id "XLOC_106458"; transcript_id "Novel_001438"; exon_number "1"; gene_type "
At_chr9	Cufflinks	exon	49182075	49182236	.	+	.	gene_id "XLOC_106458"; transcript_id "Novel_001438"; exon_number "2"; gene_type "
At_chr9	Cufflinks	exon	49182318	49183057	.	+	.	gene_id "XLOC_106458"; transcript_id "Novel_001438"; exon_number "3"; gene_type "
At_chr9	Cufflinks	exon	49273764	49274024	.	+	.	gene_id "XLOC_106467"; transcript_id "Novel_001439"; exon_number "1"; gene_type "
At_chr9	Cufflinks	exon	49274723	49275704	.	+	.	gene_id "XLOC_106467"; transcript_id "Novel_001439"; exon_number "2"; gene_type "
At_chr9	Cufflinks	exon	49302563	49303509	.	+	.	gene_id "XLOC_106477"; transcript_id "Novel_001440"; exon_number "1"; gene_type "
At_chr9	Cufflinks	exon	49422902	49423518	.	+	.	gene_id "XLOC_106495"; transcript_id "Novel_001441"; exon_number "1"; gene_type "
At_chr9	Cufflinks	exon	49426254	49426925	.	+	.	gene_id "XLOC_106495"; transcript_id "Novel_001441"; exon_number "2"; gene_type "
At_chr9	Cufflinks	exon	50636800	50637606	.	+	.	gene_id "XLOC_106661"; transcript_id "Novel_001442"; exon_number "1"; gene_type "
At_chr9	Cufflinks	exon	50837086	50837824	.	+	.	gene_id "XLOC_106697"; transcript_id "Novel_001443"; exon_number "1"; gene_type "
At_chr9	Cufflinks	exon	50838527	50838639	.	+	.	gene_id "XLOC_106697"; transcript_id "Novel_001443"; exon_number "2"; gene_type "

At_chr9	Cufflinks	exon	50838732	50838827	.	+	.	gene_id "XLOC_106697"; transcript_id "Novel_001443"; exon_number "3"; gene_type "
At_chr9	Cufflinks	exon	50838955	50839011	.	+	.	gene_id "XLOC_106697"; transcript_id "Novel_001443"; exon_number "4"; gene_type "
At_chr9	Cufflinks	exon	50839140	50839295	.	+	.	gene_id "XLOC_106697"; transcript_id "Novel_001443"; exon_number "5"; gene_type "
At_chr9	Cufflinks	exon	50839405	50839489	.	+	.	gene_id "XLOC_106697"; transcript_id "Novel_001443"; exon_number "6"; gene_type "
At_chr9	Cufflinks	exon	50839684	50839774	.	+	.	gene_id "XLOC_106697"; transcript_id "Novel_001443"; exon_number "7"; gene_type "
At_chr9	Cufflinks	exon	50839895	50840059	.	+	.	gene_id "XLOC_106697"; transcript_id "Novel_001443"; exon_number "8"; gene_type "
At_chr9	Cufflinks	exon	50840181	50840477	.	+	.	gene_id "XLOC_106697"; transcript_id "Novel_001443"; exon_number "9"; gene_type "
At_chr9	Cufflinks	exon	51759311	51759548	.	+	.	gene_id "XLOC_106830"; transcript_id "Novel_001444"; exon_number "1"; gene_type "
At_chr9	Cufflinks	exon	51760233	51760291	.	+	.	gene_id "XLOC_106830"; transcript_id "Novel_001444"; exon_number "2"; gene_type "
At_chr9	Cufflinks	exon	51760372	51761312	.	+	.	gene_id "XLOC_106830"; transcript_id "Novel_001444"; exon_number "3"; gene_type "
At_chr9	Cufflinks	exon	52557367	52557933	.	+	.	gene_id "XLOC_106965"; transcript_id "Novel_001445"; exon_number "1"; gene_type "
At_chr9	Cufflinks	exon	52558543	52558640	.	+	.	gene_id "XLOC_106965"; transcript_id "Novel_001445"; exon_number "2"; gene_type "
At_chr9	Cufflinks	exon	52558768	52558798	.	+	.	gene_id "XLOC_106965"; transcript_id "Novel_001445"; exon_number "3"; gene_type "
At_chr9	Cufflinks	exon	52559515	52559665	.	+	.	gene_id "XLOC_106965"; transcript_id "Novel_001445"; exon_number "4"; gene_type "
At_chr9	Cufflinks	exon	52557547	52557933	.	+	.	gene_id "XLOC_106965"; transcript_id "Novel_001446"; exon_number "1"; gene_type "
At_chr9	Cufflinks	exon	52558543	52558640	.	+	.	gene_id "XLOC_106965"; transcript_id "Novel_001446"; exon_number "2"; gene_type "
At_chr9	Cufflinks	exon	52558768	52558798	.	+	.	gene_id "XLOC_106965"; transcript_id "Novel_001446"; exon_number "3"; gene_type "
At_chr9	Cufflinks	exon	52559515	52559614	.	+	.	gene_id "XLOC_106965"; transcript_id "Novel_001446"; exon_number "4"; gene_type "
At_chr9	Cufflinks	exon	52560588	52560658	.	+	.	gene_id "XLOC_106965"; transcript_id "Novel_001446"; exon_number "5"; gene_type "
At_chr9	Cufflinks	exon	52560746	52560889	.	+	.	gene_id "XLOC_106965"; transcript_id "Novel_001446"; exon_number "6"; gene_type "
At_chr9	Cufflinks	exon	52561014	52561667	.	+	.	gene_id "XLOC_106965"; transcript_id "Novel_001446"; exon_number "7"; gene_type "
At_chr9	Cufflinks	exon	52557577	52557933	.	+	.	gene_id "XLOC_106965"; transcript_id "Novel_001447"; exon_number "1"; gene_type "
At_chr9	Cufflinks	exon	52558543	52558640	.	+	.	gene_id "XLOC_106965"; transcript_id "Novel_001447"; exon_number "2"; gene_type "
At_chr9	Cufflinks	exon	52558768	52558798	.	+	.	gene_id "XLOC_106965"; transcript_id "Novel_001447"; exon_number "3"; gene_type "
At_chr9	Cufflinks	exon	52559515	52559614	.	+	.	gene_id "XLOC_106965"; transcript_id "Novel_001447"; exon_number "4"; gene_type "
At_chr9	Cufflinks	exon	52560588	52560658	.	+	.	gene_id "XLOC_106965"; transcript_id "Novel_001447"; exon_number "5"; gene_type "
At_chr9	Cufflinks	exon	52560746	52561788	.	+	.	gene_id "XLOC_106965"; transcript_id "Novel_001447"; exon_number "6"; gene_type "
At_chr9	Cufflinks	exon	52559754	52560889	.	+	.	gene_id "XLOC_106965"; transcript_id "Novel_001448"; exon_number "1"; gene_type "
At_chr9	Cufflinks	exon	52561014	52561472	.	+	.	gene_id "XLOC_106965"; transcript_id "Novel_001448"; exon_number "2"; gene_type "
At_chr9	Cufflinks	exon	54443351	54443825	.	+	.	gene_id "XLOC_107337"; transcript_id "Novel_001449"; exon_number "1"; gene_type "
At_chr9	Cufflinks	exon	54443940	54444004	.	+	.	gene_id "XLOC_107337"; transcript_id "Novel_001449"; exon_number "2"; gene_type "
At_chr9	Cufflinks	exon	54444100	54444160	.	+	.	gene_id "XLOC_107337"; transcript_id "Novel_001449"; exon_number "3"; gene_type "
At_chr9	Cufflinks	exon	54444366	54444512	.	+	.	gene_id "XLOC_107337"; transcript_id "Novel_001449"; exon_number "4"; gene_type "
At_chr9	Cufflinks	exon	54444650	54444876	.	+	.	gene_id "XLOC_107337"; transcript_id "Novel_001449"; exon_number "5"; gene_type "
At_chr9	Cufflinks	exon	55456847	55457229	.	+	.	gene_id "XLOC_107524"; transcript_id "Novel_001450"; exon_number "1"; gene_type "

At_chr9	Cufflinks	exon	55457643	55457768	.	+	.	gene_id "XLOC_107524"; transcript_id "Novel_001450"; exon_number "2"; gene_type "
At_chr9	Cufflinks	exon	55457929	55457967	.	+	.	gene_id "XLOC_107524"; transcript_id "Novel_001450"; exon_number "3"; gene_type "
At_chr9	Cufflinks	exon	55458461	55459849	.	+	.	gene_id "XLOC_107524"; transcript_id "Novel_001450"; exon_number "4"; gene_type "
At_chr9	Cufflinks	exon	55522544	55522675	.	+	.	gene_id "XLOC_107531"; transcript_id "Novel_001451"; exon_number "1"; gene_type "
At_chr9	Cufflinks	exon	55524351	55524821	.	+	.	gene_id "XLOC_107531"; transcript_id "Novel_001451"; exon_number "2"; gene_type "
At_chr9	Cufflinks	exon	55524904	55525020	.	+	.	gene_id "XLOC_107531"; transcript_id "Novel_001451"; exon_number "3"; gene_type "
At_chr9	Cufflinks	exon	55525251	55525293	.	+	.	gene_id "XLOC_107531"; transcript_id "Novel_001451"; exon_number "4"; gene_type "
At_chr9	Cufflinks	exon	55902594	55902668	.	+	.	gene_id "XLOC_107581"; transcript_id "Novel_001452"; exon_number "1"; gene_type "
At_chr9	Cufflinks	exon	55902775	55902825	.	+	.	gene_id "XLOC_107581"; transcript_id "Novel_001452"; exon_number "2"; gene_type "
At_chr9	Cufflinks	exon	55902928	55903631	.	+	.	gene_id "XLOC_107581"; transcript_id "Novel_001452"; exon_number "3"; gene_type "
At_chr9	Cufflinks	exon	55983167	55983192	.	+	.	gene_id "XLOC_107603"; transcript_id "Novel_001453"; exon_number "1"; gene_type "
At_chr9	Cufflinks	exon	55983752	55983881	.	+	.	gene_id "XLOC_107603"; transcript_id "Novel_001453"; exon_number "2"; gene_type "
At_chr9	Cufflinks	exon	55984016	55984296	.	+	.	gene_id "XLOC_107603"; transcript_id "Novel_001453"; exon_number "3"; gene_type "
At_chr9	Cufflinks	exon	56232199	56232505	.	+	.	gene_id "XLOC_107640"; transcript_id "Novel_001454"; exon_number "1"; gene_type "
At_chr9	Cufflinks	exon	56232583	56233108	.	+	.	gene_id "XLOC_107640"; transcript_id "Novel_001454"; exon_number "2"; gene_type "
At_chr9	Cufflinks	exon	56338598	56338892	.	+	.	gene_id "XLOC_107657"; transcript_id "Novel_001455"; exon_number "1"; gene_type "
At_chr9	Cufflinks	exon	56339829	56339950	.	+	.	gene_id "XLOC_107657"; transcript_id "Novel_001455"; exon_number "2"; gene_type "
At_chr9	Cufflinks	exon	56349706	56350106	.	+	.	gene_id "XLOC_107659"; transcript_id "Novel_001456"; exon_number "1"; gene_type "
At_chr9	Cufflinks	exon	56352183	56352277	.	+	.	gene_id "XLOC_107659"; transcript_id "Novel_001456"; exon_number "2"; gene_type "
At_chr9	Cufflinks	exon	56349785	56350106	.	+	.	gene_id "XLOC_107659"; transcript_id "Novel_001457"; exon_number "1"; gene_type "
At_chr9	Cufflinks	exon	56351454	56351563	.	+	.	gene_id "XLOC_107659"; transcript_id "Novel_001457"; exon_number "2"; gene_type "
At_chr9	Cufflinks	exon	56430817	56431826	.	+	.	gene_id "XLOC_107668"; transcript_id "Novel_001458"; exon_number "1"; gene_type "
At_chr9	Cufflinks	exon	56432028	56432117	.	+	.	gene_id "XLOC_107668"; transcript_id "Novel_001458"; exon_number "2"; gene_type "
At_chr9	Cufflinks	exon	56432255	56432364	.	+	.	gene_id "XLOC_107668"; transcript_id "Novel_001458"; exon_number "3"; gene_type "
At_chr9	Cufflinks	exon	56432509	56433597	.	+	.	gene_id "XLOC_107668"; transcript_id "Novel_001458"; exon_number "4"; gene_type "
At_chr9	Cufflinks	exon	56433939	56433988	.	+	.	gene_id "XLOC_107668"; transcript_id "Novel_001458"; exon_number "5"; gene_type "
At_chr9	Cufflinks	exon	56434325	56434778	.	+	.	gene_id "XLOC_107668"; transcript_id "Novel_001458"; exon_number "6"; gene_type "
At_chr9	Cufflinks	exon	56430817	56432117	.	+	.	gene_id "XLOC_107668"; transcript_id "Novel_001459"; exon_number "1"; gene_type "
At_chr9	Cufflinks	exon	56432255	56432364	.	+	.	gene_id "XLOC_107668"; transcript_id "Novel_001459"; exon_number "2"; gene_type "
At_chr9	Cufflinks	exon	56432509	56432688	.	+	.	gene_id "XLOC_107668"; transcript_id "Novel_001459"; exon_number "3"; gene_type "
At_chr9	Cufflinks	exon	56433463	56433597	.	+	.	gene_id "XLOC_107668"; transcript_id "Novel_001459"; exon_number "4"; gene_type "
At_chr9	Cufflinks	exon	56433939	56433988	.	+	.	gene_id "XLOC_107668"; transcript_id "Novel_001459"; exon_number "5"; gene_type "
At_chr9	Cufflinks	exon	56434325	56434778	.	+	.	gene_id "XLOC_107668"; transcript_id "Novel_001459"; exon_number "6"; gene_type "
At_chr9	Cufflinks	exon	56430817	56431826	.	+	.	gene_id "XLOC_107668"; transcript_id "Novel_001460"; exon_number "1"; gene_type "
At_chr9	Cufflinks	exon	56432028	56432117	.	+	.	gene_id "XLOC_107668"; transcript_id "Novel_001460"; exon_number "2"; gene_type "

At_chr9	Cufflinks	exon	56432255	56432364	.	+	.	gene_id "XLOC_107668"; transcript_id "Novel_001460"; exon_number "3"; gene_type "
At_chr9	Cufflinks	exon	56432509	56432688	.	+	.	gene_id "XLOC_107668"; transcript_id "Novel_001460"; exon_number "4"; gene_type "
At_chr9	Cufflinks	exon	56433463	56433597	.	+	.	gene_id "XLOC_107668"; transcript_id "Novel_001460"; exon_number "5"; gene_type "
At_chr9	Cufflinks	exon	56433939	56433988	.	+	.	gene_id "XLOC_107668"; transcript_id "Novel_001460"; exon_number "6"; gene_type "
At_chr9	Cufflinks	exon	56434325	56434782	.	+	.	gene_id "XLOC_107668"; transcript_id "Novel_001460"; exon_number "7"; gene_type "
At_chr9	Cufflinks	exon	56583259	56583488	.	+	.	gene_id "XLOC_107680"; transcript_id "Novel_001461"; exon_number "1"; gene_type "
At_chr9	Cufflinks	exon	56584035	56585000	.	+	.	gene_id "XLOC_107680"; transcript_id "Novel_001461"; exon_number "2"; gene_type "
At_chr9	Cufflinks	exon	56583259	56583555	.	+	.	gene_id "XLOC_107680"; transcript_id "Novel_001462"; exon_number "1"; gene_type "
At_chr9	Cufflinks	exon	56584035	56585000	.	+	.	gene_id "XLOC_107680"; transcript_id "Novel_001462"; exon_number "2"; gene_type "
At_chr9	Cufflinks	exon	56585164	56585534	.	+	.	gene_id "XLOC_107681"; transcript_id "Novel_001463"; exon_number "1"; gene_type "
At_chr9	Cufflinks	exon	56585626	56585789	.	+	.	gene_id "XLOC_107681"; transcript_id "Novel_001463"; exon_number "2"; gene_type "
At_chr9	Cufflinks	exon	56587898	56588854	.	+	.	gene_id "XLOC_107681"; transcript_id "Novel_001463"; exon_number "3"; gene_type "
At_chr9	Cufflinks	exon	56719098	56720439	.	+	.	gene_id "XLOC_107681"; transcript_id "Novel_001464"; exon_number "1"; gene_type "
At_chr9	Cufflinks	exon	56720531	56723545	.	+	.	gene_id "XLOC_107681"; transcript_id "Novel_001464"; exon_number "2"; gene_type "
At_chr9	Cufflinks	exon	56825052	56827724	.	+	.	gene_id "XLOC_107725"; transcript_id "Novel_001465"; exon_number "1"; gene_type "
At_chr9	Cufflinks	exon	56846702	56849390	.	+	.	gene_id "XLOC_107726"; transcript_id "Novel_001466"; exon_number "1"; gene_type "
At_chr9	Cufflinks	exon	58309364	58309468	.	+	.	gene_id "XLOC_107936"; transcript_id "Novel_001467"; exon_number "1"; gene_type "
At_chr9	Cufflinks	exon	58309604	58310903	.	+	.	gene_id "XLOC_107936"; transcript_id "Novel_001467"; exon_number "2"; gene_type "
At_chr9	Cufflinks	exon	58311010	58311090	.	+	.	gene_id "XLOC_107936"; transcript_id "Novel_001467"; exon_number "3"; gene_type "
At_chr9	Cufflinks	exon	58311200	58311234	.	+	.	gene_id "XLOC_107936"; transcript_id "Novel_001467"; exon_number "4"; gene_type "
At_chr9	Cufflinks	exon	58309450	58309468	.	+	.	gene_id "XLOC_107936"; transcript_id "Novel_001468"; exon_number "1"; gene_type "
At_chr9	Cufflinks	exon	58309604	58313309	.	+	.	gene_id "XLOC_107936"; transcript_id "Novel_001468"; exon_number "2"; gene_type "
At_chr9	Cufflinks	exon	59015952	59016132	.	+	.	gene_id "XLOC_108040"; transcript_id "Novel_001469"; exon_number "1"; gene_type "
At_chr9	Cufflinks	exon	59016445	59017450	.	+	.	gene_id "XLOC_108040"; transcript_id "Novel_001469"; exon_number "2"; gene_type "
At_chr9	Cufflinks	exon	59015953	59016040	.	+	.	gene_id "XLOC_108040"; transcript_id "Novel_001470"; exon_number "1"; gene_type "
At_chr9	Cufflinks	exon	59016445	59017133	.	+	.	gene_id "XLOC_108040"; transcript_id "Novel_001470"; exon_number "2"; gene_type "
At_chr9	Cufflinks	exon	59689287	59689670	.	+	.	gene_id "XLOC_108146"; transcript_id "Novel_001471"; exon_number "1"; gene_type "
At_chr9	Cufflinks	exon	59689814	59689966	.	+	.	gene_id "XLOC_108146"; transcript_id "Novel_001471"; exon_number "2"; gene_type "
At_chr9	Cufflinks	exon	59690230	59690375	.	+	.	gene_id "XLOC_108146"; transcript_id "Novel_001471"; exon_number "3"; gene_type "
At_chr9	Cufflinks	exon	59691121	59691204	.	+	.	gene_id "XLOC_108146"; transcript_id "Novel_001471"; exon_number "4"; gene_type "
At_chr9	Cufflinks	exon	59694252	59694393	.	+	.	gene_id "XLOC_108146"; transcript_id "Novel_001471"; exon_number "5"; gene_type "
At_chr9	Cufflinks	exon	59694594	59694894	.	+	.	gene_id "XLOC_108146"; transcript_id "Novel_001471"; exon_number "6"; gene_type "
At_chr9	Cufflinks	exon	59689287	59689670	.	+	.	gene_id "XLOC_108146"; transcript_id "Novel_001472"; exon_number "1"; gene_type "
At_chr9	Cufflinks	exon	59689814	59690375	.	+	.	gene_id "XLOC_108146"; transcript_id "Novel_001472"; exon_number "2"; gene_type "
At_chr9	Cufflinks	exon	59691121	59691204	.	+	.	gene_id "XLOC_108146"; transcript_id "Novel_001472"; exon_number "3"; gene_type "

At_chr9	Cufflinks	exon	59694252	59694393	.	+	.	gene_id "XLOC_108146"; transcript_id "Novel_001472"; exon_number "4"; gene_type "
At_chr9	Cufflinks	exon	59694594	59694894	.	+	.	gene_id "XLOC_108146"; transcript_id "Novel_001472"; exon_number "5"; gene_type "
At_chr9	Cufflinks	exon	59809564	59809745	.	+	.	gene_id "XLOC_108157"; transcript_id "Novel_001473"; exon_number "1"; gene_type "
At_chr9	Cufflinks	exon	59810095	59810235	.	+	.	gene_id "XLOC_108157"; transcript_id "Novel_001473"; exon_number "2"; gene_type "
At_chr9	Cufflinks	exon	59810379	59810918	.	+	.	gene_id "XLOC_108157"; transcript_id "Novel_001473"; exon_number "3"; gene_type "
At_chr9	Cufflinks	exon	59985740	59986218	.	+	.	gene_id "XLOC_108173"; transcript_id "Novel_001474"; exon_number "1"; gene_type "
At_chr9	Cufflinks	exon	59986349	59986581	.	+	.	gene_id "XLOC_108173"; transcript_id "Novel_001474"; exon_number "2"; gene_type "
At_chr9	Cufflinks	exon	59986665	59986715	.	+	.	gene_id "XLOC_108173"; transcript_id "Novel_001474"; exon_number "3"; gene_type "
At_chr9	Cufflinks	exon	59986881	59987516	.	+	.	gene_id "XLOC_108173"; transcript_id "Novel_001474"; exon_number "4"; gene_type "
At_chr9	Cufflinks	exon	59988574	59988854	.	+	.	gene_id "XLOC_108174"; transcript_id "Novel_001475"; exon_number "1"; gene_type "
At_chr9	Cufflinks	exon	59989367	59989457	.	+	.	gene_id "XLOC_108174"; transcript_id "Novel_001475"; exon_number "2"; gene_type "
At_chr9	Cufflinks	exon	59989651	59989960	.	+	.	gene_id "XLOC_108174"; transcript_id "Novel_001475"; exon_number "3"; gene_type "
At_chr9	Cufflinks	exon	59990099	59990175	.	+	.	gene_id "XLOC_108174"; transcript_id "Novel_001475"; exon_number "4"; gene_type "
At_chr9	Cufflinks	exon	59991195	59994705	.	+	.	gene_id "XLOC_108174"; transcript_id "Novel_001476"; exon_number "1"; gene_type "
At_chr9	Cufflinks	exon	59994787	59995788	.	+	.	gene_id "XLOC_108174"; transcript_id "Novel_001476"; exon_number "2"; gene_type "
At_chr9	Cufflinks	exon	59996095	59996115	.	+	.	gene_id "XLOC_108174"; transcript_id "Novel_001476"; exon_number "3"; gene_type "
At_chr9	Cufflinks	exon	61548162	61548500	.	+	.	gene_id "XLOC_108297"; transcript_id "Novel_001477"; exon_number "1"; gene_type "
At_chr9	Cufflinks	exon	61549833	61550051	.	+	.	gene_id "XLOC_108297"; transcript_id "Novel_001477"; exon_number "2"; gene_type "
At_chr9	Cufflinks	exon	62041604	62041708	.	+	.	gene_id "XLOC_108350"; transcript_id "Novel_001478"; exon_number "1"; gene_type "
At_chr9	Cufflinks	exon	62043881	62046248	.	+	.	gene_id "XLOC_108350"; transcript_id "Novel_001478"; exon_number "2"; gene_type "
At_chr9	Cufflinks	exon	62042567	62042909	.	+	.	gene_id "XLOC_108350"; transcript_id "Novel_001479"; exon_number "1"; gene_type "
At_chr9	Cufflinks	exon	62043881	62046163	.	+	.	gene_id "XLOC_108350"; transcript_id "Novel_001479"; exon_number "2"; gene_type "
At_chr9	Cufflinks	exon	62134219	62134414	.	+	.	gene_id "XLOC_108370"; transcript_id "Novel_001480"; exon_number "1"; gene_type "
At_chr9	Cufflinks	exon	62134532	62134569	.	+	.	gene_id "XLOC_108370"; transcript_id "Novel_001480"; exon_number "2"; gene_type "
At_chr9	Cufflinks	exon	62135148	62135245	.	+	.	gene_id "XLOC_108370"; transcript_id "Novel_001480"; exon_number "3"; gene_type "
At_chr9	Cufflinks	exon	62135422	62137608	.	+	.	gene_id "XLOC_108370"; transcript_id "Novel_001480"; exon_number "4"; gene_type "
At_chr9	Cufflinks	exon	62134219	62134414	.	+	.	gene_id "XLOC_108370"; transcript_id "Novel_001481"; exon_number "1"; gene_type "
At_chr9	Cufflinks	exon	62134532	62134569	.	+	.	gene_id "XLOC_108370"; transcript_id "Novel_001481"; exon_number "2"; gene_type "
At_chr9	Cufflinks	exon	62135148	62135245	.	+	.	gene_id "XLOC_108370"; transcript_id "Novel_001481"; exon_number "3"; gene_type "
At_chr9	Cufflinks	exon	62135422	62135477	.	+	.	gene_id "XLOC_108370"; transcript_id "Novel_001481"; exon_number "4"; gene_type "
At_chr9	Cufflinks	exon	62136318	62137681	.	+	.	gene_id "XLOC_108370"; transcript_id "Novel_001481"; exon_number "5"; gene_type "
At_chr9	Cufflinks	exon	62669491	62669727	.	+	.	gene_id "XLOC_108440"; transcript_id "Novel_001482"; exon_number "1"; gene_type "
At_chr9	Cufflinks	exon	62670860	62671119	.	+	.	gene_id "XLOC_108440"; transcript_id "Novel_001482"; exon_number "2"; gene_type "
At_chr9	Cufflinks	exon	62671564	62672253	.	+	.	gene_id "XLOC_108440"; transcript_id "Novel_001482"; exon_number "3"; gene_type "
At_chr9	Cufflinks	exon	62674713	62674745	.	+	.	gene_id "XLOC_108443"; transcript_id "Novel_001483"; exon_number "1"; gene_type "

At_chr9	Cufflinks	exon	62674826	62674930	.	+	.	gene_id "XLOC_108443"; transcript_id "Novel_001483"; exon_number "2"; gene_type "
At_chr9	Cufflinks	exon	62676136	62676395	.	+	.	gene_id "XLOC_108443"; transcript_id "Novel_001483"; exon_number "3"; gene_type "
At_chr9	Cufflinks	exon	62676847	62677299	.	+	.	gene_id "XLOC_108443"; transcript_id "Novel_001483"; exon_number "4"; gene_type "
At_chr9	Cufflinks	exon	62674735	62674930	.	+	.	gene_id "XLOC_108443"; transcript_id "Novel_001484"; exon_number "1"; gene_type "
At_chr9	Cufflinks	exon	62676136	62676395	.	+	.	gene_id "XLOC_108443"; transcript_id "Novel_001484"; exon_number "2"; gene_type "
At_chr9	Cufflinks	exon	62676847	62677303	.	+	.	gene_id "XLOC_108443"; transcript_id "Novel_001484"; exon_number "3"; gene_type "
At_chr9	Cufflinks	exon	64325722	64325878	.	+	.	gene_id "XLOC_108628"; transcript_id "Novel_001485"; exon_number "1"; gene_type "
At_chr9	Cufflinks	exon	64326164	64327035	.	+	.	gene_id "XLOC_108628"; transcript_id "Novel_001485"; exon_number "2"; gene_type "
At_chr9	Cufflinks	exon	67054955	67055161	.	+	.	gene_id "XLOC_108839"; transcript_id "Novel_001486"; exon_number "1"; gene_type "
At_chr9	Cufflinks	exon	67225642	67225719	.	+	.	gene_id "XLOC_108859"; transcript_id "Novel_001487"; exon_number "1"; gene_type "
At_chr9	Cufflinks	exon	67225804	67225858	.	+	.	gene_id "XLOC_108859"; transcript_id "Novel_001487"; exon_number "2"; gene_type "
At_chr9	Cufflinks	exon	67225937	67225985	.	+	.	gene_id "XLOC_108859"; transcript_id "Novel_001487"; exon_number "3"; gene_type "
At_chr9	Cufflinks	exon	67226125	67226589	.	+	.	gene_id "XLOC_108859"; transcript_id "Novel_001487"; exon_number "4"; gene_type "
At_chr9	Cufflinks	exon	72069682	72069952	.	+	.	gene_id "XLOC_109099"; transcript_id "Novel_001488"; exon_number "1"; gene_type "
At_chr9	Cufflinks	exon	72071303	72072008	.	+	.	gene_id "XLOC_109099"; transcript_id "Novel_001488"; exon_number "2"; gene_type "
At_chr9	Cufflinks	exon	80414411	80414574	.	+	.	gene_id "XLOC_109471"; transcript_id "Novel_001489"; exon_number "1"; gene_type "
At_chr9	Cufflinks	exon	80417928	80420671	.	+	.	gene_id "XLOC_109471"; transcript_id "Novel_001489"; exon_number "2"; gene_type "
At_chr9	Cufflinks	exon	80420464	80421013	.	+	.	gene_id "XLOC_109471"; transcript_id "Novel_001490"; exon_number "1"; gene_type "
At_chr9	Cufflinks	exon	80423573	80425235	.	+	.	gene_id "XLOC_109471"; transcript_id "Novel_001490"; exon_number "2"; gene_type "
At_chr9	Cufflinks	exon	80444066	80445377	.	+	.	gene_id "XLOC_109476"; transcript_id "Novel_001491"; exon_number "1"; gene_type "
At_chr9	Cufflinks	exon	80446328	80448129	.	+	.	gene_id "XLOC_109476"; transcript_id "Novel_001491"; exon_number "2"; gene_type "
At_chr9	Cufflinks	exon	83061234	83061443	.	+	.	gene_id "XLOC_109601"; transcript_id "Novel_001492"; exon_number "1"; gene_type "
At_chr9	Cufflinks	exon	83999270	83999409	.	+	.	gene_id "XLOC_109704"; transcript_id "Novel_001493"; exon_number "1"; gene_type "
At_chr9	Cufflinks	exon	84000301	84000453	.	+	.	gene_id "XLOC_109704"; transcript_id "Novel_001493"; exon_number "2"; gene_type "
At_chr9	Cufflinks	exon	84001190	84001678	.	+	.	gene_id "XLOC_109704"; transcript_id "Novel_001493"; exon_number "3"; gene_type "
At_chr9	Cufflinks	exon	84449676	84449951	.	+	.	gene_id "XLOC_109792"; transcript_id "Novel_001494"; exon_number "1"; gene_type "
At_chr9	Cufflinks	exon	84450777	84450919	.	+	.	gene_id "XLOC_109792"; transcript_id "Novel_001494"; exon_number "2"; gene_type "
At_chr9	Cufflinks	exon	84451009	84451128	.	+	.	gene_id "XLOC_109792"; transcript_id "Novel_001494"; exon_number "3"; gene_type "
At_chr9	Cufflinks	exon	84451219	84451747	.	+	.	gene_id "XLOC_109792"; transcript_id "Novel_001494"; exon_number "4"; gene_type "
At_chr9	Cufflinks	exon	85196482	85196772	.	+	.	gene_id "XLOC_109953"; transcript_id "Novel_001495"; exon_number "1"; gene_type "
At_chr9	Cufflinks	exon	85459446	85459651	.	+	.	gene_id "XLOC_109982"; transcript_id "Novel_001496"; exon_number "1"; gene_type "
At_chr9	Cufflinks	exon	86694280	86694639	.	+	.	gene_id "XLOC_110060"; transcript_id "Novel_001497"; exon_number "1"; gene_type "
At_chr9	Cufflinks	exon	86695241	86695350	.	+	.	gene_id "XLOC_110060"; transcript_id "Novel_001497"; exon_number "2"; gene_type "
At_chr9	Cufflinks	exon	86695411	86696554	.	+	.	gene_id "XLOC_110060"; transcript_id "Novel_001497"; exon_number "3"; gene_type "
At_chr9	Cufflinks	exon	86694280	86695350	.	+	.	gene_id "XLOC_110060"; transcript_id "Novel_001498"; exon_number "1"; gene_type "

At_chr9	Cufflinks	exon	86695411	86696554	.	+	.	gene_id "XLOC_110060"; transcript_id "Novel_001498"; exon_number "2"; gene_type "
At_chr9	Cufflinks	exon	86694280	86695350	.	+	.	gene_id "XLOC_110060"; transcript_id "Novel_001499"; exon_number "1"; gene_type "
At_chr9	Cufflinks	exon	86695411	86695548	.	+	.	gene_id "XLOC_110060"; transcript_id "Novel_001499"; exon_number "2"; gene_type "
At_chr9	Cufflinks	exon	86695663	86696554	.	+	.	gene_id "XLOC_110060"; transcript_id "Novel_001499"; exon_number "3"; gene_type "
At_chr9	Cufflinks	exon	86694290	86694639	.	+	.	gene_id "XLOC_110060"; transcript_id "Novel_001500"; exon_number "1"; gene_type "
At_chr9	Cufflinks	exon	86695241	86695350	.	+	.	gene_id "XLOC_110060"; transcript_id "Novel_001500"; exon_number "2"; gene_type "
At_chr9	Cufflinks	exon	86695411	86695548	.	+	.	gene_id "XLOC_110060"; transcript_id "Novel_001500"; exon_number "3"; gene_type "
At_chr9	Cufflinks	exon	86695663	86696555	.	+	.	gene_id "XLOC_110060"; transcript_id "Novel_001500"; exon_number "4"; gene_type "
At_chr9	Cufflinks	exon	89184241	89184558	.	+	.	gene_id "XLOC_110143"; transcript_id "Novel_001501"; exon_number "1"; gene_type "
At_chr9	Cufflinks	exon	89184704	89184800	.	+	.	gene_id "XLOC_110143"; transcript_id "Novel_001501"; exon_number "2"; gene_type "
At_chr9	Cufflinks	exon	89184909	89185027	.	+	.	gene_id "XLOC_110143"; transcript_id "Novel_001501"; exon_number "3"; gene_type "
At_chr9	Cufflinks	exon	89185581	89185748	.	+	.	gene_id "XLOC_110143"; transcript_id "Novel_001501"; exon_number "4"; gene_type "
At_chr9	Cufflinks	exon	89186359	89186487	.	+	.	gene_id "XLOC_110143"; transcript_id "Novel_001501"; exon_number "5"; gene_type "
At_chr9	Cufflinks	exon	89187013	89187073	.	+	.	gene_id "XLOC_110143"; transcript_id "Novel_001501"; exon_number "6"; gene_type "
At_chr9	Cufflinks	exon	89187576	89187679	.	+	.	gene_id "XLOC_110143"; transcript_id "Novel_001501"; exon_number "7"; gene_type "
At_chr9	Cufflinks	exon	89187825	89187908	.	+	.	gene_id "XLOC_110143"; transcript_id "Novel_001501"; exon_number "8"; gene_type "
At_chr9	Cufflinks	exon	89188168	89188274	.	+	.	gene_id "XLOC_110143"; transcript_id "Novel_001501"; exon_number "9"; gene_type "
At_chr9	Cufflinks	exon	89188875	89189251	.	+	.	gene_id "XLOC_110143"; transcript_id "Novel_001501"; exon_number "10"; gene_type
At_chr9	Cufflinks	exon	89189471	89189514	.	+	.	gene_id "XLOC_110143"; transcript_id "Novel_001502"; exon_number "1"; gene_type "
At_chr9	Cufflinks	exon	89189740	89189805	.	+	.	gene_id "XLOC_110143"; transcript_id "Novel_001502"; exon_number "2"; gene_type "
At_chr9	Cufflinks	exon	89190222	89190294	.	+	.	gene_id "XLOC_110143"; transcript_id "Novel_001502"; exon_number "3"; gene_type "
At_chr9	Cufflinks	exon	89190392	89190478	.	+	.	gene_id "XLOC_110143"; transcript_id "Novel_001502"; exon_number "4"; gene_type "
At_chr9	Cufflinks	exon	89190788	89190842	.	+	.	gene_id "XLOC_110143"; transcript_id "Novel_001502"; exon_number "5"; gene_type "
At_chr9	Cufflinks	exon	89190934	89191370	.	+	.	gene_id "XLOC_110143"; transcript_id "Novel_001502"; exon_number "6"; gene_type "
At_chr9	Cufflinks	exon	89347791	89347948	.	+	.	gene_id "XLOC_110157"; transcript_id "Novel_001503"; exon_number "1"; gene_type "
At_chr9	Cufflinks	exon	89348114	89348235	.	+	.	gene_id "XLOC_110157"; transcript_id "Novel_001503"; exon_number "2"; gene_type "
At_chr9	Cufflinks	exon	89348368	89348408	.	+	.	gene_id "XLOC_110157"; transcript_id "Novel_001503"; exon_number "3"; gene_type "
At_chr9	Cufflinks	exon	89348496	89348594	.	+	.	gene_id "XLOC_110157"; transcript_id "Novel_001503"; exon_number "4"; gene_type "
At_chr9	Cufflinks	exon	89349286	89349382	.	+	.	gene_id "XLOC_110157"; transcript_id "Novel_001503"; exon_number "5"; gene_type "
At_chr9	Cufflinks	exon	89983864	89984208	.	+	.	gene_id "XLOC_110198"; transcript_id "Novel_001504"; exon_number "1"; gene_type "
At_chr9	Cufflinks	exon	89985173	89986414	.	+	.	gene_id "XLOC_110198"; transcript_id "Novel_001504"; exon_number "2"; gene_type "
At_chr9	Cufflinks	exon	1612032	1613414	.	-	.	gene_id "XLOC_110439"; transcript_id "Novel_001505"; exon_number "1"; gene_type "
At_chr9	Cufflinks	exon	1614853	1614977	.	-	.	gene_id "XLOC_110439"; transcript_id "Novel_001505"; exon_number "2"; gene_type "
At_chr9	Cufflinks	exon	1612553	1613388	.	-	.	gene_id "XLOC_110439"; transcript_id "Novel_001506"; exon_number "1"; gene_type "
At_chr9	Cufflinks	exon	1614853	1614912	.	-	.	gene_id "XLOC_110439"; transcript_id "Novel_001506"; exon_number "2"; gene_type "

At_chr9	Cufflinks	exon	8511090	8511374	.	-	.	gene_id "XLOC_110806"; transcript_id "Novel_001507"; exon_number "1"; gene_type "
At_chr9	Cufflinks	exon	8511511	8511572	.	-	.	gene_id "XLOC_110806"; transcript_id "Novel_001507"; exon_number "2"; gene_type "
At_chr9	Cufflinks	exon	8511678	8511753	.	-	.	gene_id "XLOC_110806"; transcript_id "Novel_001507"; exon_number "3"; gene_type "
At_chr9	Cufflinks	exon	8511873	8512045	.	-	.	gene_id "XLOC_110806"; transcript_id "Novel_001507"; exon_number "4"; gene_type "
At_chr9	Cufflinks	exon	8512327	8512411	.	-	.	gene_id "XLOC_110806"; transcript_id "Novel_001507"; exon_number "5"; gene_type "
At_chr9	Cufflinks	exon	8512513	8512829	.	-	.	gene_id "XLOC_110806"; transcript_id "Novel_001507"; exon_number "6"; gene_type "
At_chr9	Cufflinks	exon	8511158	8511374	.	-	.	gene_id "XLOC_110806"; transcript_id "Novel_001508"; exon_number "1"; gene_type "
At_chr9	Cufflinks	exon	8511511	8511572	.	-	.	gene_id "XLOC_110806"; transcript_id "Novel_001508"; exon_number "2"; gene_type "
At_chr9	Cufflinks	exon	8511678	8511753	.	-	.	gene_id "XLOC_110806"; transcript_id "Novel_001508"; exon_number "3"; gene_type "
At_chr9	Cufflinks	exon	8511873	8512045	.	-	.	gene_id "XLOC_110806"; transcript_id "Novel_001508"; exon_number "4"; gene_type "
At_chr9	Cufflinks	exon	8512327	8512411	.	-	.	gene_id "XLOC_110806"; transcript_id "Novel_001508"; exon_number "5"; gene_type "
At_chr9	Cufflinks	exon	8513007	8513696	.	-	.	gene_id "XLOC_110806"; transcript_id "Novel_001508"; exon_number "6"; gene_type "
At_chr9	Cufflinks	exon	18282879	18283595	.	-	.	gene_id "XLOC_111218"; transcript_id "Novel_001509"; exon_number "1"; gene_type "
At_chr9	Cufflinks	exon	18284222	18284268	.	-	.	gene_id "XLOC_111218"; transcript_id "Novel_001509"; exon_number "2"; gene_type "
At_chr9	Cufflinks	exon	18284342	18284489	.	-	.	gene_id "XLOC_111218"; transcript_id "Novel_001509"; exon_number "3"; gene_type "
At_chr9	Cufflinks	exon	18282879	18283595	.	-	.	gene_id "XLOC_111218"; transcript_id "Novel_001510"; exon_number "1"; gene_type "
At_chr9	Cufflinks	exon	18284222	18284544	.	-	.	gene_id "XLOC_111218"; transcript_id "Novel_001510"; exon_number "2"; gene_type "
At_chr9	Cufflinks	exon	28936800	28937315	.	-	.	gene_id "XLOC_111738"; transcript_id "Novel_001511"; exon_number "1"; gene_type "
At_chr9	Cufflinks	exon	28937397	28937825	.	-	.	gene_id "XLOC_111738"; transcript_id "Novel_001511"; exon_number "2"; gene_type "
At_chr9	Cufflinks	exon	28937926	28938154	.	-	.	gene_id "XLOC_111738"; transcript_id "Novel_001511"; exon_number "3"; gene_type "
At_chr9	Cufflinks	exon	28938255	28938513	.	-	.	gene_id "XLOC_111738"; transcript_id "Novel_001511"; exon_number "4"; gene_type "
At_chr9	Cufflinks	exon	28938601	28938721	.	-	.	gene_id "XLOC_111738"; transcript_id "Novel_001511"; exon_number "5"; gene_type "
At_chr9	Cufflinks	exon	28938802	28938931	.	-	.	gene_id "XLOC_111738"; transcript_id "Novel_001511"; exon_number "6"; gene_type "
At_chr9	Cufflinks	exon	28939023	28939094	.	-	.	gene_id "XLOC_111738"; transcript_id "Novel_001511"; exon_number "7"; gene_type "
At_chr9	Cufflinks	exon	28939177	28939225	.	-	.	gene_id "XLOC_111738"; transcript_id "Novel_001511"; exon_number "8"; gene_type "
At_chr9	Cufflinks	exon	28939312	28939633	.	-	.	gene_id "XLOC_111738"; transcript_id "Novel_001511"; exon_number "9"; gene_type "
At_chr9	Cufflinks	exon	28939710	28940327	.	-	.	gene_id "XLOC_111738"; transcript_id "Novel_001511"; exon_number "10"; gene_type
At_chr9	Cufflinks	exon	28936800	28937315	.	-	.	gene_id "XLOC_111738"; transcript_id "Novel_001512"; exon_number "1"; gene_type "
At_chr9	Cufflinks	exon	28937397	28937825	.	-	.	gene_id "XLOC_111738"; transcript_id "Novel_001512"; exon_number "2"; gene_type "
At_chr9	Cufflinks	exon	28937926	28937970	.	-	.	gene_id "XLOC_111738"; transcript_id "Novel_001512"; exon_number "3"; gene_type "
At_chr9	Cufflinks	exon	28938050	28938154	.	-	.	gene_id "XLOC_111738"; transcript_id "Novel_001512"; exon_number "4"; gene_type "
At_chr9	Cufflinks	exon	28938255	28938513	.	-	.	gene_id "XLOC_111738"; transcript_id "Novel_001512"; exon_number "5"; gene_type "
At_chr9	Cufflinks	exon	28938601	28938721	.	-	.	gene_id "XLOC_111738"; transcript_id "Novel_001512"; exon_number "6"; gene_type "
At_chr9	Cufflinks	exon	28938802	28938931	.	-	.	gene_id "XLOC_111738"; transcript_id "Novel_001512"; exon_number "7"; gene_type "
At_chr9	Cufflinks	exon	28939023	28939094	.	-	.	gene_id "XLOC_111738"; transcript_id "Novel_001512"; exon_number "8"; gene_type "

At_chr9	Cufflinks	exon	28939177	28939225	.	-	.	gene_id "XLOC_111738"; transcript_id "Novel_001512"; exon_number "9"; gene_type "
At_chr9	Cufflinks	exon	28939312	28939633	.	-	.	gene_id "XLOC_111738"; transcript_id "Novel_001512"; exon_number "10"; gene_type
At_chr9	Cufflinks	exon	28939710	28940327	.	-	.	gene_id "XLOC_111738"; transcript_id "Novel_001512"; exon_number "11"; gene_type
At_chr9	Cufflinks	exon	28936950	28937315	.	-	.	gene_id "XLOC_111738"; transcript_id "Novel_001513"; exon_number "1"; gene_type "
At_chr9	Cufflinks	exon	28937397	28937825	.	-	.	gene_id "XLOC_111738"; transcript_id "Novel_001513"; exon_number "2"; gene_type "
At_chr9	Cufflinks	exon	28937926	28937970	.	-	.	gene_id "XLOC_111738"; transcript_id "Novel_001513"; exon_number "3"; gene_type "
At_chr9	Cufflinks	exon	28938050	28938154	.	-	.	gene_id "XLOC_111738"; transcript_id "Novel_001513"; exon_number "4"; gene_type "
At_chr9	Cufflinks	exon	28938255	28938513	.	-	.	gene_id "XLOC_111738"; transcript_id "Novel_001513"; exon_number "5"; gene_type "
At_chr9	Cufflinks	exon	28938601	28938721	.	-	.	gene_id "XLOC_111738"; transcript_id "Novel_001513"; exon_number "6"; gene_type "
At_chr9	Cufflinks	exon	28938802	28938931	.	-	.	gene_id "XLOC_111738"; transcript_id "Novel_001513"; exon_number "7"; gene_type "
At_chr9	Cufflinks	exon	28939023	28939094	.	-	.	gene_id "XLOC_111738"; transcript_id "Novel_001513"; exon_number "8"; gene_type "
At_chr9	Cufflinks	exon	28939177	28939225	.	-	.	gene_id "XLOC_111738"; transcript_id "Novel_001513"; exon_number "9"; gene_type "
At_chr9	Cufflinks	exon	28939312	28940333	.	-	.	gene_id "XLOC_111738"; transcript_id "Novel_001513"; exon_number "10"; gene_type
At_chr9	Cufflinks	exon	28971914	28972262	.	-	.	gene_id "XLOC_111742"; transcript_id "Novel_001514"; exon_number "1"; gene_type "
At_chr9	Cufflinks	exon	28972389	28972805	.	-	.	gene_id "XLOC_111742"; transcript_id "Novel_001514"; exon_number "2"; gene_type "
At_chr9	Cufflinks	exon	29388171	29388537	.	-	.	gene_id "XLOC_111760"; transcript_id "Novel_001515"; exon_number "1"; gene_type "
At_chr9	Cufflinks	exon	29388945	29389007	.	-	.	gene_id "XLOC_111760"; transcript_id "Novel_001515"; exon_number "2"; gene_type "
At_chr9	Cufflinks	exon	29389211	29389254	.	-	.	gene_id "XLOC_111760"; transcript_id "Novel_001515"; exon_number "3"; gene_type "
At_chr9	Cufflinks	exon	29389469	29389614	.	-	.	gene_id "XLOC_111760"; transcript_id "Novel_001515"; exon_number "4"; gene_type "
At_chr9	Cufflinks	exon	29389858	29389877	.	-	.	gene_id "XLOC_111760"; transcript_id "Novel_001515"; exon_number "5"; gene_type "
At_chr9	Cufflinks	exon	29390358	29390493	.	-	.	gene_id "XLOC_111760"; transcript_id "Novel_001515"; exon_number "6"; gene_type "
At_chr9	Cufflinks	exon	29390835	29391054	.	-	.	gene_id "XLOC_111760"; transcript_id "Novel_001515"; exon_number "7"; gene_type "
At_chr9	Cufflinks	exon	29391475	29391655	.	-	.	gene_id "XLOC_111760"; transcript_id "Novel_001515"; exon_number "8"; gene_type "
At_chr9	Cufflinks	exon	29392150	29392173	.	-	.	gene_id "XLOC_111760"; transcript_id "Novel_001515"; exon_number "9"; gene_type "
At_chr9	Cufflinks	exon	29392333	29392929	.	-	.	gene_id "XLOC_111760"; transcript_id "Novel_001515"; exon_number "10"; gene_type
At_chr9	Cufflinks	exon	29519393	29519861	.	-	.	gene_id "XLOC_111767"; transcript_id "Novel_001516"; exon_number "1"; gene_type "
At_chr9	Cufflinks	exon	29520491	29520547	.	-	.	gene_id "XLOC_111767"; transcript_id "Novel_001516"; exon_number "2"; gene_type "
At_chr9	Cufflinks	exon	29521237	29521312	.	-	.	gene_id "XLOC_111767"; transcript_id "Novel_001516"; exon_number "3"; gene_type "
At_chr9	Cufflinks	exon	29523875	29523940	.	-	.	gene_id "XLOC_111767"; transcript_id "Novel_001517"; exon_number "1"; gene_type "
At_chr9	Cufflinks	exon	29524542	29524777	.	-	.	gene_id "XLOC_111767"; transcript_id "Novel_001517"; exon_number "2"; gene_type "
At_chr9	Cufflinks	exon	29524858	29525240	.	-	.	gene_id "XLOC_111767"; transcript_id "Novel_001517"; exon_number "3"; gene_type "
At_chr9	Cufflinks	exon	29526220	29526253	.	-	.	gene_id "XLOC_111767"; transcript_id "Novel_001517"; exon_number "4"; gene_type "
At_chr9	Cufflinks	exon	29524689	29524777	.	-	.	gene_id "XLOC_111767"; transcript_id "Novel_001518"; exon_number "1"; gene_type "
At_chr9	Cufflinks	exon	29524858	29525522	.	-	.	gene_id "XLOC_111767"; transcript_id "Novel_001518"; exon_number "2"; gene_type "
At_chr9	Cufflinks	exon	31866197	31866757	.	-	.	gene_id "XLOC_111933"; transcript_id "Novel_001519"; exon_number "1"; gene_type "

At_chr9	Cufflinks	exon	31866993	31869417	.	-	.	gene_id "XLOC_111933"; transcript_id "Novel_001519"; exon_number "2"; gene_type "
At_chr9	Cufflinks	exon	32027028	32027260	.	-	.	gene_id "XLOC_111943"; transcript_id "Novel_001520"; exon_number "1"; gene_type "
At_chr9	Cufflinks	exon	32629015	32629106	.	-	.	gene_id "XLOC_111997"; transcript_id "Novel_001521"; exon_number "1"; gene_type "
At_chr9	Cufflinks	exon	32630662	32630732	.	-	.	gene_id "XLOC_111997"; transcript_id "Novel_001521"; exon_number "2"; gene_type "
At_chr9	Cufflinks	exon	32631451	32631530	.	-	.	gene_id "XLOC_111997"; transcript_id "Novel_001521"; exon_number "3"; gene_type "
At_chr9	Cufflinks	exon	32631769	32631943	.	-	.	gene_id "XLOC_111997"; transcript_id "Novel_001521"; exon_number "4"; gene_type "
At_chr9	Cufflinks	exon	32632161	32632451	.	-	.	gene_id "XLOC_111997"; transcript_id "Novel_001521"; exon_number "5"; gene_type "
At_chr9	Cufflinks	exon	32629019	32629112	.	-	.	gene_id "XLOC_111997"; transcript_id "Novel_001522"; exon_number "1"; gene_type "
At_chr9	Cufflinks	exon	32630662	32630732	.	-	.	gene_id "XLOC_111997"; transcript_id "Novel_001522"; exon_number "2"; gene_type "
At_chr9	Cufflinks	exon	32631451	32631530	.	-	.	gene_id "XLOC_111997"; transcript_id "Novel_001522"; exon_number "3"; gene_type "
At_chr9	Cufflinks	exon	32631769	32631943	.	-	.	gene_id "XLOC_111997"; transcript_id "Novel_001522"; exon_number "4"; gene_type "
At_chr9	Cufflinks	exon	32632161	32632456	.	-	.	gene_id "XLOC_111997"; transcript_id "Novel_001522"; exon_number "5"; gene_type "
At_chr9	Cufflinks	exon	32629019	32630732	.	-	.	gene_id "XLOC_111997"; transcript_id "Novel_001523"; exon_number "1"; gene_type "
At_chr9	Cufflinks	exon	32631451	32631530	.	-	.	gene_id "XLOC_111997"; transcript_id "Novel_001523"; exon_number "2"; gene_type "
At_chr9	Cufflinks	exon	32631769	32631943	.	-	.	gene_id "XLOC_111997"; transcript_id "Novel_001523"; exon_number "3"; gene_type "
At_chr9	Cufflinks	exon	32632161	32632456	.	-	.	gene_id "XLOC_111997"; transcript_id "Novel_001523"; exon_number "4"; gene_type "
At_chr9	Cufflinks	exon	32631106	32631530	.	-	.	gene_id "XLOC_111997"; transcript_id "Novel_001524"; exon_number "1"; gene_type "
At_chr9	Cufflinks	exon	32631769	32631943	.	-	.	gene_id "XLOC_111997"; transcript_id "Novel_001524"; exon_number "2"; gene_type "
At_chr9	Cufflinks	exon	32632161	32632449	.	-	.	gene_id "XLOC_111997"; transcript_id "Novel_001524"; exon_number "3"; gene_type "
At_chr9	Cufflinks	exon	33201837	33202391	.	-	.	gene_id "XLOC_112056"; transcript_id "Novel_001525"; exon_number "1"; gene_type "
At_chr9	Cufflinks	exon	33203271	33204487	.	-	.	gene_id "XLOC_112056"; transcript_id "Novel_001525"; exon_number "2"; gene_type "
At_chr9	Cufflinks	exon	33537125	33537425	.	-	.	gene_id "XLOC_112087"; transcript_id "Novel_001526"; exon_number "1"; gene_type "
At_chr9	Cufflinks	exon	33537781	33537838	.	-	.	gene_id "XLOC_112087"; transcript_id "Novel_001526"; exon_number "2"; gene_type "
At_chr9	Cufflinks	exon	33537186	33537425	.	-	.	gene_id "XLOC_112087"; transcript_id "Novel_001527"; exon_number "1"; gene_type "
At_chr9	Cufflinks	exon	33537692	33537809	.	-	.	gene_id "XLOC_112087"; transcript_id "Novel_001527"; exon_number "2"; gene_type "
At_chr9	Cufflinks	exon	35421478	35422147	.	-	.	gene_id "XLOC_112301"; transcript_id "Novel_001528"; exon_number "1"; gene_type "
At_chr9	Cufflinks	exon	35423257	35423758	.	-	.	gene_id "XLOC_112301"; transcript_id "Novel_001528"; exon_number "2"; gene_type "
At_chr9	Cufflinks	exon	36528429	36528531	.	-	.	gene_id "XLOC_112423"; transcript_id "Novel_001529"; exon_number "1"; gene_type "
At_chr9	Cufflinks	exon	36528601	36528862	.	-	.	gene_id "XLOC_112423"; transcript_id "Novel_001529"; exon_number "2"; gene_type "
At_chr9	Cufflinks	exon	37141943	37143283	.	-	.	gene_id "XLOC_112482"; transcript_id "Novel_001530"; exon_number "1"; gene_type "
At_chr9	Cufflinks	exon	37143371	37143797	.	-	.	gene_id "XLOC_112482"; transcript_id "Novel_001530"; exon_number "2"; gene_type "
At_chr9	Cufflinks	exon	37144101	37144143	.	-	.	gene_id "XLOC_112482"; transcript_id "Novel_001530"; exon_number "3"; gene_type "
At_chr9	Cufflinks	exon	37144711	37144855	.	-	.	gene_id "XLOC_112482"; transcript_id "Novel_001530"; exon_number "4"; gene_type "
At_chr9	Cufflinks	exon	37144946	37145054	.	-	.	gene_id "XLOC_112482"; transcript_id "Novel_001530"; exon_number "5"; gene_type "
At_chr9	Cufflinks	exon	37145146	37145282	.	-	.	gene_id "XLOC_112482"; transcript_id "Novel_001530"; exon_number "6"; gene_type "

At_chr9	Cufflinks	exon	37145369	37145465	.	-	.	gene_id "XLOC_112482"; transcript_id "Novel_001530"; exon_number "7"; gene_type "
At_chr9	Cufflinks	exon	37146263	37146330	.	-	.	gene_id "XLOC_112482"; transcript_id "Novel_001530"; exon_number "8"; gene_type "
At_chr9	Cufflinks	exon	37146422	37146662	.	-	.	gene_id "XLOC_112482"; transcript_id "Novel_001530"; exon_number "9"; gene_type "
At_chr9	Cufflinks	exon	37830593	37831243	.	-	.	gene_id "XLOC_112534"; transcript_id "Novel_001531"; exon_number "1"; gene_type "
At_chr9	Cufflinks	exon	37831974	37832904	.	-	.	gene_id "XLOC_112534"; transcript_id "Novel_001531"; exon_number "2"; gene_type "
At_chr9	Cufflinks	exon	38429324	38429777	.	-	.	gene_id "XLOC_112609"; transcript_id "Novel_001532"; exon_number "1"; gene_type "
At_chr9	Cufflinks	exon	38429895	38430107	.	-	.	gene_id "XLOC_112609"; transcript_id "Novel_001532"; exon_number "2"; gene_type "
At_chr9	Cufflinks	exon	38430242	38430702	.	-	.	gene_id "XLOC_112609"; transcript_id "Novel_001532"; exon_number "3"; gene_type "
At_chr9	Cufflinks	exon	38431297	38431510	.	-	.	gene_id "XLOC_112609"; transcript_id "Novel_001532"; exon_number "4"; gene_type "
At_chr9	Cufflinks	exon	41650537	41651328	.	-	.	gene_id "XLOC_113172"; transcript_id "Novel_001533"; exon_number "1"; gene_type "
At_chr9	Cufflinks	exon	41652394	41652480	.	-	.	gene_id "XLOC_113172"; transcript_id "Novel_001533"; exon_number "2"; gene_type "
At_chr9	Cufflinks	exon	41978324	41978536	.	-	.	gene_id "XLOC_113228"; transcript_id "Novel_001534"; exon_number "1"; gene_type "
At_chr9	Cufflinks	exon	43826342	43827118	.	-	.	gene_id "XLOC_113561"; transcript_id "Novel_001535"; exon_number "1"; gene_type "
At_chr9	Cufflinks	exon	43827215	43827358	.	-	.	gene_id "XLOC_113561"; transcript_id "Novel_001535"; exon_number "2"; gene_type "
At_chr9	Cufflinks	exon	43827490	43827560	.	-	.	gene_id "XLOC_113561"; transcript_id "Novel_001535"; exon_number "3"; gene_type "
At_chr9	Cufflinks	exon	43827688	43827787	.	-	.	gene_id "XLOC_113561"; transcript_id "Novel_001535"; exon_number "4"; gene_type "
At_chr9	Cufflinks	exon	43828030	43828060	.	-	.	gene_id "XLOC_113561"; transcript_id "Novel_001535"; exon_number "5"; gene_type "
At_chr9	Cufflinks	exon	43828159	43828256	.	-	.	gene_id "XLOC_113561"; transcript_id "Novel_001535"; exon_number "6"; gene_type "
At_chr9	Cufflinks	exon	43828764	43829113	.	-	.	gene_id "XLOC_113561"; transcript_id "Novel_001535"; exon_number "7"; gene_type "
At_chr9	Cufflinks	exon	45117283	45118072	.	-	.	gene_id "XLOC_113809"; transcript_id "Novel_001536"; exon_number "1"; gene_type "
At_chr9	Cufflinks	exon	46105010	46107717	.	-	.	gene_id "XLOC_113999"; transcript_id "Novel_001537"; exon_number "1"; gene_type "
At_chr9	Cufflinks	exon	46108090	46108206	.	-	.	gene_id "XLOC_113999"; transcript_id "Novel_001537"; exon_number "2"; gene_type "
At_chr9	Cufflinks	exon	46105010	46105790	.	-	.	gene_id "XLOC_113999"; transcript_id "Novel_001538"; exon_number "1"; gene_type "
At_chr9	Cufflinks	exon	46105888	46107717	.	-	.	gene_id "XLOC_113999"; transcript_id "Novel_001538"; exon_number "2"; gene_type "
At_chr9	Cufflinks	exon	46108090	46108206	.	-	.	gene_id "XLOC_113999"; transcript_id "Novel_001538"; exon_number "3"; gene_type "
At_chr9	Cufflinks	exon	46542145	46545789	.	-	.	gene_id "XLOC_114074"; transcript_id "Novel_001539"; exon_number "1"; gene_type "
At_chr9	Cufflinks	exon	46546808	46546918	.	-	.	gene_id "XLOC_114074"; transcript_id "Novel_001539"; exon_number "2"; gene_type "
At_chr9	Cufflinks	exon	46547982	46548115	.	-	.	gene_id "XLOC_114074"; transcript_id "Novel_001539"; exon_number "3"; gene_type "
At_chr9	Cufflinks	exon	46548293	46548691	.	-	.	gene_id "XLOC_114074"; transcript_id "Novel_001539"; exon_number "4"; gene_type "
At_chr9	Cufflinks	exon	46616677	46619356	.	-	.	gene_id "XLOC_114091"; transcript_id "Novel_001540"; exon_number "1"; gene_type "
At_chr9	Cufflinks	exon	46619571	46619697	.	-	.	gene_id "XLOC_114091"; transcript_id "Novel_001540"; exon_number "2"; gene_type "
At_chr9	Cufflinks	exon	46616677	46619356	.	-	.	gene_id "XLOC_114091"; transcript_id "Novel_001541"; exon_number "1"; gene_type "
At_chr9	Cufflinks	exon	46619567	46619699	.	-	.	gene_id "XLOC_114091"; transcript_id "Novel_001541"; exon_number "2"; gene_type "
At_chr9	Cufflinks	exon	47301077	47310932	.	-	.	gene_id "XLOC_114231"; transcript_id "Novel_001542"; exon_number "1"; gene_type "
At_chr9	Cufflinks	exon	49141663	49142573	.	-	.	gene_id "XLOC_114472"; transcript_id "Novel_001543"; exon_number "1"; gene_type "

At_chr9	Cufflinks	exon	49142689	49143212	.	-	.	gene_id "XLOC_114472"; transcript_id "Novel_001543"; exon_number "2"; gene_type "
At_chr9	Cufflinks	exon	49144124	49144580	.	-	.	gene_id "XLOC_114472"; transcript_id "Novel_001544"; exon_number "1"; gene_type "
At_chr9	Cufflinks	exon	49144682	49144819	.	-	.	gene_id "XLOC_114472"; transcript_id "Novel_001544"; exon_number "2"; gene_type "
At_chr9	Cufflinks	exon	49145195	49145338	.	-	.	gene_id "XLOC_114472"; transcript_id "Novel_001544"; exon_number "3"; gene_type "
At_chr9	Cufflinks	exon	49186783	49187006	.	-	.	gene_id "XLOC_114472"; transcript_id "Novel_001545"; exon_number "1"; gene_type "
At_chr9	Cufflinks	exon	49189097	49189432	.	-	.	gene_id "XLOC_114472"; transcript_id "Novel_001545"; exon_number "2"; gene_type "
At_chr9	Cufflinks	exon	49191200	49191365	.	-	.	gene_id "XLOC_114472"; transcript_id "Novel_001546"; exon_number "1"; gene_type "
At_chr9	Cufflinks	exon	49191458	49191670	.	-	.	gene_id "XLOC_114472"; transcript_id "Novel_001546"; exon_number "2"; gene_type "
At_chr9	Cufflinks	exon	49191936	49192061	.	-	.	gene_id "XLOC_114472"; transcript_id "Novel_001546"; exon_number "3"; gene_type "
At_chr9	Cufflinks	exon	49192163	49192300	.	-	.	gene_id "XLOC_114472"; transcript_id "Novel_001546"; exon_number "4"; gene_type "
At_chr9	Cufflinks	exon	49192659	49192712	.	-	.	gene_id "XLOC_114472"; transcript_id "Novel_001546"; exon_number "5"; gene_type "
At_chr9	Cufflinks	exon	49145393	49145540	.	-	.	gene_id "XLOC_114473"; transcript_id "Novel_001547"; exon_number "1"; gene_type "
At_chr9	Cufflinks	exon	49145678	49145772	.	-	.	gene_id "XLOC_114473"; transcript_id "Novel_001547"; exon_number "2"; gene_type "
At_chr9	Cufflinks	exon	49146207	49146437	.	-	.	gene_id "XLOC_114473"; transcript_id "Novel_001548"; exon_number "1"; gene_type "
At_chr9	Cufflinks	exon	49146532	49146646	.	-	.	gene_id "XLOC_114473"; transcript_id "Novel_001548"; exon_number "2"; gene_type "
At_chr9	Cufflinks	exon	49147070	49147251	.	-	.	gene_id "XLOC_114473"; transcript_id "Novel_001548"; exon_number "3"; gene_type "
At_chr9	Cufflinks	exon	49147339	49147534	.	-	.	gene_id "XLOC_114473"; transcript_id "Novel_001548"; exon_number "4"; gene_type "
At_chr9	Cufflinks	exon	49147932	49148020	.	-	.	gene_id "XLOC_114473"; transcript_id "Novel_001548"; exon_number "5"; gene_type "
At_chr9	Cufflinks	exon	49192767	49193045	.	-	.	gene_id "XLOC_114473"; transcript_id "Novel_001549"; exon_number "1"; gene_type "
At_chr9	Cufflinks	exon	49193182	49193448	.	-	.	gene_id "XLOC_114473"; transcript_id "Novel_001549"; exon_number "2"; gene_type "
At_chr9	Cufflinks	exon	49193752	49193938	.	-	.	gene_id "XLOC_114473"; transcript_id "Novel_001549"; exon_number "3"; gene_type "
At_chr9	Cufflinks	exon	49194040	49194154	.	-	.	gene_id "XLOC_114473"; transcript_id "Novel_001549"; exon_number "4"; gene_type "
At_chr9	Cufflinks	exon	49194609	49194790	.	-	.	gene_id "XLOC_114473"; transcript_id "Novel_001549"; exon_number "5"; gene_type "
At_chr9	Cufflinks	exon	49194894	49195089	.	-	.	gene_id "XLOC_114473"; transcript_id "Novel_001549"; exon_number "6"; gene_type "
At_chr9	Cufflinks	exon	49195570	49195908	.	-	.	gene_id "XLOC_114473"; transcript_id "Novel_001549"; exon_number "7"; gene_type "
At_chr9	Cufflinks	exon	49193053	49193448	.	-	.	gene_id "XLOC_114473"; transcript_id "Novel_001550"; exon_number "1"; gene_type "
At_chr9	Cufflinks	exon	49193752	49193938	.	-	.	gene_id "XLOC_114473"; transcript_id "Novel_001550"; exon_number "2"; gene_type "
At_chr9	Cufflinks	exon	49194040	49194154	.	-	.	gene_id "XLOC_114473"; transcript_id "Novel_001550"; exon_number "3"; gene_type "
At_chr9	Cufflinks	exon	49194609	49194790	.	-	.	gene_id "XLOC_114473"; transcript_id "Novel_001550"; exon_number "4"; gene_type "
At_chr9	Cufflinks	exon	49194894	49195089	.	-	.	gene_id "XLOC_114473"; transcript_id "Novel_001550"; exon_number "5"; gene_type "
At_chr9	Cufflinks	exon	49195570	49195840	.	-	.	gene_id "XLOC_114473"; transcript_id "Novel_001550"; exon_number "6"; gene_type "
At_chr9	Cufflinks	exon	49193694	49193938	.	-	.	gene_id "XLOC_114473"; transcript_id "Novel_001551"; exon_number "1"; gene_type "
At_chr9	Cufflinks	exon	49194040	49194154	.	-	.	gene_id "XLOC_114473"; transcript_id "Novel_001551"; exon_number "2"; gene_type "
At_chr9	Cufflinks	exon	49194609	49194790	.	-	.	gene_id "XLOC_114473"; transcript_id "Novel_001551"; exon_number "3"; gene_type "
At_chr9	Cufflinks	exon	49194894	49195089	.	-	.	gene_id "XLOC_114473"; transcript_id "Novel_001551"; exon_number "4"; gene_type "

At_chr9	Cufflinks	exon	49195570	49195908	.	-	.	gene_id "XLOC_114473"; transcript_id "Novel_001551"; exon_number "5"; gene_type "
At_chr9	Cufflinks	exon	49182926	49183254	.	-	.	gene_id "XLOC_114480"; transcript_id "Novel_001552"; exon_number "1"; gene_type "
At_chr9	Cufflinks	exon	49183550	49183614	.	-	.	gene_id "XLOC_114480"; transcript_id "Novel_001552"; exon_number "2"; gene_type "
At_chr9	Cufflinks	exon	49183723	49183785	.	-	.	gene_id "XLOC_114480"; transcript_id "Novel_001552"; exon_number "3"; gene_type "
At_chr9	Cufflinks	exon	49184117	49184165	.	-	.	gene_id "XLOC_114480"; transcript_id "Novel_001552"; exon_number "4"; gene_type "
At_chr9	Cufflinks	exon	49184467	49184750	.	-	.	gene_id "XLOC_114480"; transcript_id "Novel_001552"; exon_number "5"; gene_type "
At_chr9	Cufflinks	exon	49323302	49324039	.	-	.	gene_id "XLOC_114498"; transcript_id "Novel_001553"; exon_number "1"; gene_type "
At_chr9	Cufflinks	exon	49324121	49324568	.	-	.	gene_id "XLOC_114498"; transcript_id "Novel_001553"; exon_number "2"; gene_type "
At_chr9	Cufflinks	exon	50704186	50704885	.	-	.	gene_id "XLOC_114665"; transcript_id "Novel_001554"; exon_number "1"; gene_type "
At_chr9	Cufflinks	exon	50704992	50705084	.	-	.	gene_id "XLOC_114665"; transcript_id "Novel_001554"; exon_number "2"; gene_type "
At_chr9	Cufflinks	exon	50705199	50705958	.	-	.	gene_id "XLOC_114665"; transcript_id "Novel_001554"; exon_number "3"; gene_type "
At_chr9	Cufflinks	exon	50706313	50706427	.	-	.	gene_id "XLOC_114665"; transcript_id "Novel_001554"; exon_number "4"; gene_type "
At_chr9	Cufflinks	exon	50704930	50705084	.	-	.	gene_id "XLOC_114665"; transcript_id "Novel_001555"; exon_number "1"; gene_type "
At_chr9	Cufflinks	exon	50705199	50705958	.	-	.	gene_id "XLOC_114665"; transcript_id "Novel_001555"; exon_number "2"; gene_type "
At_chr9	Cufflinks	exon	50706313	50706332	.	-	.	gene_id "XLOC_114665"; transcript_id "Novel_001555"; exon_number "3"; gene_type "
At_chr9	Cufflinks	exon	52672485	52673507	.	-	.	gene_id "XLOC_114993"; transcript_id "Novel_001556"; exon_number "1"; gene_type "
At_chr9	Cufflinks	exon	52673629	52673715	.	-	.	gene_id "XLOC_114993"; transcript_id "Novel_001556"; exon_number "2"; gene_type "
At_chr9	Cufflinks	exon	52673819	52673872	.	-	.	gene_id "XLOC_114993"; transcript_id "Novel_001556"; exon_number "3"; gene_type "
At_chr9	Cufflinks	exon	52674122	52674184	.	-	.	gene_id "XLOC_114993"; transcript_id "Novel_001556"; exon_number "4"; gene_type "
At_chr9	Cufflinks	exon	52674273	52674320	.	-	.	gene_id "XLOC_114993"; transcript_id "Novel_001556"; exon_number "5"; gene_type "
At_chr9	Cufflinks	exon	52674454	52675051	.	-	.	gene_id "XLOC_114993"; transcript_id "Novel_001556"; exon_number "6"; gene_type "
At_chr9	Cufflinks	exon	52672485	52673507	.	-	.	gene_id "XLOC_114993"; transcript_id "Novel_001557"; exon_number "1"; gene_type "
At_chr9	Cufflinks	exon	52673629	52673715	.	-	.	gene_id "XLOC_114993"; transcript_id "Novel_001557"; exon_number "2"; gene_type "
At_chr9	Cufflinks	exon	52673819	52673872	.	-	.	gene_id "XLOC_114993"; transcript_id "Novel_001557"; exon_number "3"; gene_type "
At_chr9	Cufflinks	exon	52674122	52674184	.	-	.	gene_id "XLOC_114993"; transcript_id "Novel_001557"; exon_number "4"; gene_type "
At_chr9	Cufflinks	exon	52674273	52674320	.	-	.	gene_id "XLOC_114993"; transcript_id "Novel_001557"; exon_number "5"; gene_type "
At_chr9	Cufflinks	exon	52674454	52674633	.	-	.	gene_id "XLOC_114993"; transcript_id "Novel_001557"; exon_number "6"; gene_type "
At_chr9	Cufflinks	exon	52675167	52675209	.	-	.	gene_id "XLOC_114993"; transcript_id "Novel_001557"; exon_number "7"; gene_type "
At_chr9	Cufflinks	exon	53135722	53136101	.	-	.	gene_id "XLOC_115076"; transcript_id "Novel_001558"; exon_number "1"; gene_type "
At_chr9	Cufflinks	exon	53136213	53136442	.	-	.	gene_id "XLOC_115076"; transcript_id "Novel_001558"; exon_number "2"; gene_type "
At_chr9	Cufflinks	exon	53136714	53136811	.	-	.	gene_id "XLOC_115076"; transcript_id "Novel_001558"; exon_number "3"; gene_type "
At_chr9	Cufflinks	exon	53137003	53138101	.	-	.	gene_id "XLOC_115076"; transcript_id "Novel_001558"; exon_number "4"; gene_type "
At_chr9	Cufflinks	exon	53138690	53138769	.	-	.	gene_id "XLOC_115076"; transcript_id "Novel_001558"; exon_number "5"; gene_type "
At_chr9	Cufflinks	exon	53139062	53139239	.	-	.	gene_id "XLOC_115076"; transcript_id "Novel_001558"; exon_number "6"; gene_type "
At_chr9	Cufflinks	exon	53135728	53136101	.	-	.	gene_id "XLOC_115076"; transcript_id "Novel_001559"; exon_number "1"; gene_type "

At_chr9	Cufflinks	exon	53136213	53136305	.	-	.	gene_id "XLOC_115076"; transcript_id "Novel_001559"; exon_number "2"; gene_type "
At_chr9	Cufflinks	exon	53136380	53136442	.	-	.	gene_id "XLOC_115076"; transcript_id "Novel_001559"; exon_number "3"; gene_type "
At_chr9	Cufflinks	exon	53136714	53136811	.	-	.	gene_id "XLOC_115076"; transcript_id "Novel_001559"; exon_number "4"; gene_type "
At_chr9	Cufflinks	exon	53137003	53137791	.	-	.	gene_id "XLOC_115076"; transcript_id "Novel_001559"; exon_number "5"; gene_type "
At_chr9	Cufflinks	exon	53137172	53137945	.	-	.	gene_id "XLOC_115076"; transcript_id "Novel_001560"; exon_number "1"; gene_type "
At_chr9	Cufflinks	exon	53138029	53138101	.	-	.	gene_id "XLOC_115076"; transcript_id "Novel_001560"; exon_number "2"; gene_type "
At_chr9	Cufflinks	exon	53138690	53138769	.	-	.	gene_id "XLOC_115076"; transcript_id "Novel_001560"; exon_number "3"; gene_type "
At_chr9	Cufflinks	exon	53139062	53139239	.	-	.	gene_id "XLOC_115076"; transcript_id "Novel_001560"; exon_number "4"; gene_type "
At_chr9	Cufflinks	exon	53898682	53898996	.	-	.	gene_id "XLOC_115223"; transcript_id "Novel_001561"; exon_number "1"; gene_type "
At_chr9	Cufflinks	exon	54422865	54423055	.	-	.	gene_id "XLOC_115330"; transcript_id "Novel_001562"; exon_number "1"; gene_type "
At_chr9	Cufflinks	exon	54423322	54423658	.	-	.	gene_id "XLOC_115330"; transcript_id "Novel_001562"; exon_number "2"; gene_type "
At_chr9	Cufflinks	exon	56042877	56043740	.	-	.	gene_id "XLOC_115635"; transcript_id "Novel_001563"; exon_number "1"; gene_type "
At_chr9	Cufflinks	exon	56043834	56044152	.	-	.	gene_id "XLOC_115635"; transcript_id "Novel_001563"; exon_number "2"; gene_type "
At_chr9	Cufflinks	exon	56073728	56075173	.	-	.	gene_id "XLOC_115643"; transcript_id "Novel_001564"; exon_number "1"; gene_type "
At_chr9	Cufflinks	exon	56075280	56075866	.	-	.	gene_id "XLOC_115643"; transcript_id "Novel_001564"; exon_number "2"; gene_type "
At_chr9	Cufflinks	exon	56147115	56147258	.	-	.	gene_id "XLOC_115654"; transcript_id "Novel_001565"; exon_number "1"; gene_type "
At_chr9	Cufflinks	exon	56147667	56147833	.	-	.	gene_id "XLOC_115654"; transcript_id "Novel_001565"; exon_number "2"; gene_type "
At_chr9	Cufflinks	exon	56251965	56252251	.	-	.	gene_id "XLOC_115674"; transcript_id "Novel_001566"; exon_number "1"; gene_type "
At_chr9	Cufflinks	exon	56252328	56252404	.	-	.	gene_id "XLOC_115674"; transcript_id "Novel_001566"; exon_number "2"; gene_type "
At_chr9	Cufflinks	exon	56252604	56252761	.	-	.	gene_id "XLOC_115674"; transcript_id "Novel_001566"; exon_number "3"; gene_type "
At_chr9	Cufflinks	exon	56253151	56253236	.	-	.	gene_id "XLOC_115674"; transcript_id "Novel_001566"; exon_number "4"; gene_type "
At_chr9	Cufflinks	exon	56253327	56254057	.	-	.	gene_id "XLOC_115674"; transcript_id "Novel_001566"; exon_number "5"; gene_type "
At_chr9	Cufflinks	exon	56252190	56252404	.	-	.	gene_id "XLOC_115674"; transcript_id "Novel_001567"; exon_number "1"; gene_type "
At_chr9	Cufflinks	exon	56252604	56252761	.	-	.	gene_id "XLOC_115674"; transcript_id "Novel_001567"; exon_number "2"; gene_type "
At_chr9	Cufflinks	exon	56253151	56253236	.	-	.	gene_id "XLOC_115674"; transcript_id "Novel_001567"; exon_number "3"; gene_type "
At_chr9	Cufflinks	exon	56253327	56254050	.	-	.	gene_id "XLOC_115674"; transcript_id "Novel_001567"; exon_number "4"; gene_type "
At_chr9	Cufflinks	exon	56294362	56294530	.	-	.	gene_id "XLOC_115679"; transcript_id "Novel_001568"; exon_number "1"; gene_type "
At_chr9	Cufflinks	exon	56296352	56296509	.	-	.	gene_id "XLOC_115679"; transcript_id "Novel_001568"; exon_number "2"; gene_type "
At_chr9	Cufflinks	exon	56294408	56294530	.	-	.	gene_id "XLOC_115679"; transcript_id "Novel_001569"; exon_number "1"; gene_type "
At_chr9	Cufflinks	exon	56296352	56296459	.	-	.	gene_id "XLOC_115679"; transcript_id "Novel_001569"; exon_number "2"; gene_type "
At_chr9	Cufflinks	exon	56296701	56296727	.	-	.	gene_id "XLOC_115679"; transcript_id "Novel_001569"; exon_number "3"; gene_type "
At_chr9	Cufflinks	exon	56294409	56294530	.	-	.	gene_id "XLOC_115679"; transcript_id "Novel_001570"; exon_number "1"; gene_type "
At_chr9	Cufflinks	exon	56296352	56296459	.	-	.	gene_id "XLOC_115679"; transcript_id "Novel_001570"; exon_number "2"; gene_type "
At_chr9	Cufflinks	exon	56296654	56296701	.	-	.	gene_id "XLOC_115679"; transcript_id "Novel_001570"; exon_number "3"; gene_type "
At_chr9	Cufflinks	exon	56362496	56362548	.	-	.	gene_id "XLOC_115686"; transcript_id "Novel_001571"; exon_number "1"; gene_type "

At_chr9	Cufflinks	exon	56371464	56371922	.	-	.	gene_id "XLOC_115686"; transcript_id "Novel_001571"; exon_number "2"; gene_type "
At_chr9	Cufflinks	exon	56363098	56363205	.	-	.	gene_id "XLOC_115686"; transcript_id "Novel_001572"; exon_number "1"; gene_type "
At_chr9	Cufflinks	exon	56383911	56384027	.	-	.	gene_id "XLOC_115686"; transcript_id "Novel_001572"; exon_number "2"; gene_type "
At_chr9	Cufflinks	exon	56363433	56363486	.	-	.	gene_id "XLOC_115686"; transcript_id "Novel_001573"; exon_number "1"; gene_type "
At_chr9	Cufflinks	exon	56374743	56374844	.	-	.	gene_id "XLOC_115686"; transcript_id "Novel_001573"; exon_number "2"; gene_type "
At_chr9	Cufflinks	exon	56374936	56375334	.	-	.	gene_id "XLOC_115686"; transcript_id "Novel_001573"; exon_number "3"; gene_type "
At_chr9	Cufflinks	exon	56372052	56372269	.	-	.	gene_id "XLOC_115686"; transcript_id "Novel_001574"; exon_number "1"; gene_type "
At_chr9	Cufflinks	exon	56374743	56374844	.	-	.	gene_id "XLOC_115686"; transcript_id "Novel_001574"; exon_number "2"; gene_type "
At_chr9	Cufflinks	exon	56374936	56375336	.	-	.	gene_id "XLOC_115686"; transcript_id "Novel_001574"; exon_number "3"; gene_type "
At_chr9	Cufflinks	exon	56374423	56374844	.	-	.	gene_id "XLOC_115686"; transcript_id "Novel_001575"; exon_number "1"; gene_type "
At_chr9	Cufflinks	exon	56374936	56375336	.	-	.	gene_id "XLOC_115686"; transcript_id "Novel_001575"; exon_number "2"; gene_type "
At_chr9	Cufflinks	exon	56438230	56439090	.	-	.	gene_id "XLOC_115695"; transcript_id "Novel_001576"; exon_number "1"; gene_type "
At_chr9	Cufflinks	exon	56439352	56439653	.	-	.	gene_id "XLOC_115695"; transcript_id "Novel_001576"; exon_number "2"; gene_type "
At_chr9	Cufflinks	exon	56439772	56440121	.	-	.	gene_id "XLOC_115695"; transcript_id "Novel_001576"; exon_number "3"; gene_type "
At_chr9	Cufflinks	exon	57028184	57028572	.	-	.	gene_id "XLOC_115763"; transcript_id "Novel_001577"; exon_number "1"; gene_type "
At_chr9	Cufflinks	exon	57028892	57028954	.	-	.	gene_id "XLOC_115763"; transcript_id "Novel_001577"; exon_number "2"; gene_type "
At_chr9	Cufflinks	exon	57029033	57029214	.	-	.	gene_id "XLOC_115763"; transcript_id "Novel_001577"; exon_number "3"; gene_type "
At_chr9	Cufflinks	exon	58607252	58607735	.	-	.	gene_id "XLOC_115988"; transcript_id "Novel_001578"; exon_number "1"; gene_type "
At_chr9	Cufflinks	exon	59847432	59847662	.	-	.	gene_id "XLOC_116098"; transcript_id "Novel_001579"; exon_number "1"; gene_type "
At_chr9	Cufflinks	exon	59847790	59847919	.	-	.	gene_id "XLOC_116098"; transcript_id "Novel_001579"; exon_number "2"; gene_type "
At_chr9	Cufflinks	exon	59848018	59848188	.	-	.	gene_id "XLOC_116098"; transcript_id "Novel_001579"; exon_number "3"; gene_type "
At_chr9	Cufflinks	exon	59848284	59848406	.	-	.	gene_id "XLOC_116098"; transcript_id "Novel_001579"; exon_number "4"; gene_type "
At_chr9	Cufflinks	exon	59848590	59848694	.	-	.	gene_id "XLOC_116098"; transcript_id "Novel_001579"; exon_number "5"; gene_type "
At_chr9	Cufflinks	exon	59848787	59849531	.	-	.	gene_id "XLOC_116098"; transcript_id "Novel_001579"; exon_number "6"; gene_type "
At_chr9	Cufflinks	exon	59850104	59850404	.	-	.	gene_id "XLOC_116098"; transcript_id "Novel_001579"; exon_number "7"; gene_type "
At_chr9	Cufflinks	exon	59847508	59847662	.	-	.	gene_id "XLOC_116098"; transcript_id "Novel_001580"; exon_number "1"; gene_type "
At_chr9	Cufflinks	exon	59847790	59847919	.	-	.	gene_id "XLOC_116098"; transcript_id "Novel_001580"; exon_number "2"; gene_type "
At_chr9	Cufflinks	exon	59848018	59848188	.	-	.	gene_id "XLOC_116098"; transcript_id "Novel_001580"; exon_number "3"; gene_type "
At_chr9	Cufflinks	exon	59848284	59848406	.	-	.	gene_id "XLOC_116098"; transcript_id "Novel_001580"; exon_number "4"; gene_type "
At_chr9	Cufflinks	exon	59848590	59848694	.	-	.	gene_id "XLOC_116098"; transcript_id "Novel_001580"; exon_number "5"; gene_type "
At_chr9	Cufflinks	exon	59848787	59849817	.	-	.	gene_id "XLOC_116098"; transcript_id "Novel_001580"; exon_number "6"; gene_type "
At_chr9	Cufflinks	exon	59961464	59962358	.	-	.	gene_id "XLOC_116140"; transcript_id "Novel_001581"; exon_number "1"; gene_type "
At_chr9	Cufflinks	exon	59962575	59963076	.	-	.	gene_id "XLOC_116140"; transcript_id "Novel_001581"; exon_number "2"; gene_type "
At_chr9	Cufflinks	exon	59963150	59963232	.	-	.	gene_id "XLOC_116140"; transcript_id "Novel_001581"; exon_number "3"; gene_type "
At_chr9	Cufflinks	exon	59963562	59963729	.	-	.	gene_id "XLOC_116140"; transcript_id "Novel_001581"; exon_number "4"; gene_type "

At_chr9	Cufflinks	exon	59963823	59964292	.	-	.	gene_id "XLOC_116140"; transcript_id "Novel_001581"; exon_number "5"; gene_type "
At_chr9	Cufflinks	exon	59964421	59964461	.	-	.	gene_id "XLOC_116140"; transcript_id "Novel_001581"; exon_number "6"; gene_type "
At_chr9	Cufflinks	exon	59964541	59964632	.	-	.	gene_id "XLOC_116140"; transcript_id "Novel_001581"; exon_number "7"; gene_type "
At_chr9	Cufflinks	exon	59965095	59965197	.	-	.	gene_id "XLOC_116140"; transcript_id "Novel_001581"; exon_number "8"; gene_type "
At_chr9	Cufflinks	exon	59961464	59963076	.	-	.	gene_id "XLOC_116140"; transcript_id "Novel_001582"; exon_number "1"; gene_type "
At_chr9	Cufflinks	exon	59963150	59963232	.	-	.	gene_id "XLOC_116140"; transcript_id "Novel_001582"; exon_number "2"; gene_type "
At_chr9	Cufflinks	exon	59963562	59963729	.	-	.	gene_id "XLOC_116140"; transcript_id "Novel_001582"; exon_number "3"; gene_type "
At_chr9	Cufflinks	exon	59963823	59964045	.	-	.	gene_id "XLOC_116140"; transcript_id "Novel_001582"; exon_number "4"; gene_type "
At_chr9	Cufflinks	exon	59964128	59964461	.	-	.	gene_id "XLOC_116140"; transcript_id "Novel_001582"; exon_number "5"; gene_type "
At_chr9	Cufflinks	exon	59964541	59964632	.	-	.	gene_id "XLOC_116140"; transcript_id "Novel_001582"; exon_number "6"; gene_type "
At_chr9	Cufflinks	exon	59965095	59965197	.	-	.	gene_id "XLOC_116140"; transcript_id "Novel_001582"; exon_number "7"; gene_type "
At_chr9	Cufflinks	exon	59961551	59962358	.	-	.	gene_id "XLOC_116140"; transcript_id "Novel_001583"; exon_number "1"; gene_type "
At_chr9	Cufflinks	exon	59962575	59962789	.	-	.	gene_id "XLOC_116140"; transcript_id "Novel_001583"; exon_number "2"; gene_type "
At_chr9	Cufflinks	exon	59963014	59963076	.	-	.	gene_id "XLOC_116140"; transcript_id "Novel_001583"; exon_number "3"; gene_type "
At_chr9	Cufflinks	exon	59963150	59963232	.	-	.	gene_id "XLOC_116140"; transcript_id "Novel_001583"; exon_number "4"; gene_type "
At_chr9	Cufflinks	exon	59963562	59963729	.	-	.	gene_id "XLOC_116140"; transcript_id "Novel_001583"; exon_number "5"; gene_type "
At_chr9	Cufflinks	exon	59963823	59964461	.	-	.	gene_id "XLOC_116140"; transcript_id "Novel_001583"; exon_number "6"; gene_type "
At_chr9	Cufflinks	exon	59964541	59964632	.	-	.	gene_id "XLOC_116140"; transcript_id "Novel_001583"; exon_number "7"; gene_type "
At_chr9	Cufflinks	exon	59965095	59965197	.	-	.	gene_id "XLOC_116140"; transcript_id "Novel_001583"; exon_number "8"; gene_type "
At_chr9	Cufflinks	exon	59961797	59962358	.	-	.	gene_id "XLOC_116140"; transcript_id "Novel_001584"; exon_number "1"; gene_type "
At_chr9	Cufflinks	exon	59962575	59963076	.	-	.	gene_id "XLOC_116140"; transcript_id "Novel_001584"; exon_number "2"; gene_type "
At_chr9	Cufflinks	exon	59963150	59963232	.	-	.	gene_id "XLOC_116140"; transcript_id "Novel_001584"; exon_number "3"; gene_type "
At_chr9	Cufflinks	exon	59963562	59963729	.	-	.	gene_id "XLOC_116140"; transcript_id "Novel_001584"; exon_number "4"; gene_type "
At_chr9	Cufflinks	exon	59963823	59964045	.	-	.	gene_id "XLOC_116140"; transcript_id "Novel_001584"; exon_number "5"; gene_type "
At_chr9	Cufflinks	exon	59964128	59964461	.	-	.	gene_id "XLOC_116140"; transcript_id "Novel_001584"; exon_number "6"; gene_type "
At_chr9	Cufflinks	exon	59964541	59964632	.	-	.	gene_id "XLOC_116140"; transcript_id "Novel_001584"; exon_number "7"; gene_type "
At_chr9	Cufflinks	exon	59965095	59965171	.	-	.	gene_id "XLOC_116140"; transcript_id "Novel_001584"; exon_number "8"; gene_type "
At_chr9	Cufflinks	exon	59961809	59962358	.	-	.	gene_id "XLOC_116140"; transcript_id "Novel_001585"; exon_number "1"; gene_type "
At_chr9	Cufflinks	exon	59962575	59962789	.	-	.	gene_id "XLOC_116140"; transcript_id "Novel_001585"; exon_number "2"; gene_type "
At_chr9	Cufflinks	exon	59963014	59963076	.	-	.	gene_id "XLOC_116140"; transcript_id "Novel_001585"; exon_number "3"; gene_type "
At_chr9	Cufflinks	exon	59963150	59963232	.	-	.	gene_id "XLOC_116140"; transcript_id "Novel_001585"; exon_number "4"; gene_type "
At_chr9	Cufflinks	exon	59963562	59963729	.	-	.	gene_id "XLOC_116140"; transcript_id "Novel_001585"; exon_number "5"; gene_type "
At_chr9	Cufflinks	exon	59963823	59964045	.	-	.	gene_id "XLOC_116140"; transcript_id "Novel_001585"; exon_number "6"; gene_type "
At_chr9	Cufflinks	exon	59964128	59964461	.	-	.	gene_id "XLOC_116140"; transcript_id "Novel_001585"; exon_number "7"; gene_type "
At_chr9	Cufflinks	exon	59964541	59964632	.	-	.	gene_id "XLOC_116140"; transcript_id "Novel_001585"; exon_number "8"; gene_type "

At_chr9	Cufflinks	exon	59965095	59965276	.	-	.	gene_id "XLOC_116140"; transcript_id "Novel_001585"; exon_number "9"; gene_type "
At_chr9	Cufflinks	exon	59970059	59970172	.	-	.	gene_id "XLOC_116140"; transcript_id "Novel_001585"; exon_number "10"; gene_type
At_chr9	Cufflinks	exon	59970502	59970586	.	-	.	gene_id "XLOC_116140"; transcript_id "Novel_001585"; exon_number "11"; gene_type
At_chr9	Cufflinks	exon	59970682	59970886	.	-	.	gene_id "XLOC_116140"; transcript_id "Novel_001585"; exon_number "12"; gene_type
At_chr9	Cufflinks	exon	59971130	59971394	.	-	.	gene_id "XLOC_116140"; transcript_id "Novel_001585"; exon_number "13"; gene_type
At_chr9	Cufflinks	exon	61524319	61526705	.	-	.	gene_id "XLOC_116228"; transcript_id "Novel_001586"; exon_number "1"; gene_type "
At_chr9	Cufflinks	exon	61527678	61527779	.	-	.	gene_id "XLOC_116228"; transcript_id "Novel_001586"; exon_number "2"; gene_type "
At_chr9	Cufflinks	exon	61528898	61529013	.	-	.	gene_id "XLOC_116228"; transcript_id "Novel_001586"; exon_number "3"; gene_type "
At_chr9	Cufflinks	exon	61524418	61526705	.	-	.	gene_id "XLOC_116228"; transcript_id "Novel_001587"; exon_number "1"; gene_type "
At_chr9	Cufflinks	exon	61527678	61527811	.	-	.	gene_id "XLOC_116228"; transcript_id "Novel_001587"; exon_number "2"; gene_type "
At_chr9	Cufflinks	exon	61528898	61529110	.	-	.	gene_id "XLOC_116228"; transcript_id "Novel_001587"; exon_number "3"; gene_type "
At_chr9	Cufflinks	exon	61524428	61527779	.	-	.	gene_id "XLOC_116228"; transcript_id "Novel_001588"; exon_number "1"; gene_type "
At_chr9	Cufflinks	exon	61528898	61528967	.	-	.	gene_id "XLOC_116228"; transcript_id "Novel_001588"; exon_number "2"; gene_type "
At_chr9	Cufflinks	exon	61524480	61526705	.	-	.	gene_id "XLOC_116228"; transcript_id "Novel_001589"; exon_number "1"; gene_type "
At_chr9	Cufflinks	exon	61527678	61528970	.	-	.	gene_id "XLOC_116228"; transcript_id "Novel_001589"; exon_number "2"; gene_type "
At_chr9	Cufflinks	exon	64354283	64354564	.	-	.	gene_id "XLOC_116575"; transcript_id "Novel_001590"; exon_number "1"; gene_type "
At_chr9	Cufflinks	exon	64354655	64354701	.	-	.	gene_id "XLOC_116575"; transcript_id "Novel_001590"; exon_number "2"; gene_type "
At_chr9	Cufflinks	exon	64354807	64354852	.	-	.	gene_id "XLOC_116575"; transcript_id "Novel_001590"; exon_number "3"; gene_type "
At_chr9	Cufflinks	exon	64354969	64355011	.	-	.	gene_id "XLOC_116575"; transcript_id "Novel_001590"; exon_number "4"; gene_type "
At_chr9	Cufflinks	exon	64355471	64355551	.	-	.	gene_id "XLOC_116575"; transcript_id "Novel_001590"; exon_number "5"; gene_type "
At_chr9	Cufflinks	exon	66323722	66324708	.	-	.	gene_id "XLOC_116768"; transcript_id "Novel_001591"; exon_number "1"; gene_type "
At_chr9	Cufflinks	exon	66324807	66324891	.	-	.	gene_id "XLOC_116768"; transcript_id "Novel_001591"; exon_number "2"; gene_type "
At_chr9	Cufflinks	exon	66334546	66335966	.	-	.	gene_id "XLOC_116769"; transcript_id "Novel_001592"; exon_number "1"; gene_type "
At_chr9	Cufflinks	exon	66337925	66338329	.	-	.	gene_id "XLOC_116769"; transcript_id "Novel_001592"; exon_number "2"; gene_type "
At_chr9	Cufflinks	exon	71514252	71514340	.	-	.	gene_id "XLOC_117053"; transcript_id "Novel_001593"; exon_number "1"; gene_type "
At_chr9	Cufflinks	exon	71514432	71514656	.	-	.	gene_id "XLOC_117053"; transcript_id "Novel_001593"; exon_number "2"; gene_type "
At_chr9	Cufflinks	exon	72935198	72936354	.	-	.	gene_id "XLOC_117146"; transcript_id "Novel_001594"; exon_number "1"; gene_type "
At_chr9	Cufflinks	exon	77317262	77317429	.	-	.	gene_id "XLOC_117291"; transcript_id "Novel_001595"; exon_number "1"; gene_type "
At_chr9	Cufflinks	exon	77317548	77317613	.	-	.	gene_id "XLOC_117291"; transcript_id "Novel_001595"; exon_number "2"; gene_type "
At_chr9	Cufflinks	exon	81541383	81541469	.	-	.	gene_id "XLOC_117485"; transcript_id "Novel_001596"; exon_number "1"; gene_type "
At_chr9	Cufflinks	exon	81541585	81541701	.	-	.	gene_id "XLOC_117485"; transcript_id "Novel_001596"; exon_number "2"; gene_type "
At_chr9	Cufflinks	exon	81541777	81541870	.	-	.	gene_id "XLOC_117485"; transcript_id "Novel_001596"; exon_number "3"; gene_type "
At_chr9	Cufflinks	exon	84950489	84950553	.	-	.	gene_id "XLOC_117847"; transcript_id "Novel_001597"; exon_number "1"; gene_type "
At_chr9	Cufflinks	exon	84951436	84951528	.	-	.	gene_id "XLOC_117847"; transcript_id "Novel_001597"; exon_number "2"; gene_type "
At_chr9	Cufflinks	exon	84951622	84951695	.	-	.	gene_id "XLOC_117847"; transcript_id "Novel_001597"; exon_number "3"; gene_type "

At_chr9	Cufflinks	exon	84952550	84952720	.	-	.	gene_id "XLOC_117847"; transcript_id "Novel_001597"; exon_number "4"; gene_type "
At_chr9	Cufflinks	exon	88072553	88072876	.	-	.	gene_id "XLOC_118034"; transcript_id "Novel_001598"; exon_number "1"; gene_type "
At_chr9	Cufflinks	exon	88072963	88073355	.	-	.	gene_id "XLOC_118034"; transcript_id "Novel_001598"; exon_number "2"; gene_type "
At_chr9	Cufflinks	exon	88073444	88073542	.	-	.	gene_id "XLOC_118034"; transcript_id "Novel_001598"; exon_number "3"; gene_type "
At_chr9	Cufflinks	exon	88074420	88074488	.	-	.	gene_id "XLOC_118034"; transcript_id "Novel_001598"; exon_number "4"; gene_type "
At_chr9	Cufflinks	exon	88074599	88075046	.	-	.	gene_id "XLOC_118034"; transcript_id "Novel_001598"; exon_number "5"; gene_type "
At_chr9	Cufflinks	exon	89918546	89918888	.	-	.	gene_id "XLOC_118095"; transcript_id "Novel_001599"; exon_number "1"; gene_type "
At_chr9	Cufflinks	exon	89919046	89921174	.	-	.	gene_id "XLOC_118095"; transcript_id "Novel_001599"; exon_number "2"; gene_type "
At_chr9	Cufflinks	exon	90295287	90296127	.	-	.	gene_id "XLOC_118140"; transcript_id "Novel_001600"; exon_number "1"; gene_type "
Dt_chr1	Cufflinks	exon	4674310	4674611	.	+	.	gene_id "XLOC_118632"; transcript_id "Novel_001601"; exon_number "1"; gene_type "
Dt_chr1	Cufflinks	exon	4675528	4676186	.	+	.	gene_id "XLOC_118632"; transcript_id "Novel_001601"; exon_number "2"; gene_type "
Dt_chr1	Cufflinks	exon	4676440	4676912	.	+	.	gene_id "XLOC_118632"; transcript_id "Novel_001601"; exon_number "3"; gene_type "
Dt_chr1	Cufflinks	exon	4674352	4674611	.	+	.	gene_id "XLOC_118632"; transcript_id "Novel_001602"; exon_number "1"; gene_type "
Dt_chr1	Cufflinks	exon	4675528	4676911	.	+	.	gene_id "XLOC_118632"; transcript_id "Novel_001602"; exon_number "2"; gene_type "
Dt_chr1	Cufflinks	exon	15283653	15283992	.	+	.	gene_id "XLOC_118918"; transcript_id "Novel_001603"; exon_number "1"; gene_type "
Dt_chr1	Cufflinks	exon	15284665	15284795	.	+	.	gene_id "XLOC_118918"; transcript_id "Novel_001603"; exon_number "2"; gene_type "
Dt_chr1	Cufflinks	exon	15285956	15286221	.	+	.	gene_id "XLOC_118918"; transcript_id "Novel_001603"; exon_number "3"; gene_type "
Dt_chr1	Cufflinks	exon	20233103	20233307	.	+	.	gene_id "XLOC_119045"; transcript_id "Novel_001604"; exon_number "1"; gene_type "
Dt_chr1	Cufflinks	exon	20234002	20234143	.	+	.	gene_id "XLOC_119045"; transcript_id "Novel_001604"; exon_number "2"; gene_type "
Dt_chr1	Cufflinks	exon	22272077	22272208	.	+	.	gene_id "XLOC_119126"; transcript_id "Novel_001605"; exon_number "1"; gene_type "
Dt_chr1	Cufflinks	exon	22272856	22272983	.	+	.	gene_id "XLOC_119126"; transcript_id "Novel_001605"; exon_number "2"; gene_type "
Dt_chr1	Cufflinks	exon	22279184	22279258	.	+	.	gene_id "XLOC_119126"; transcript_id "Novel_001606"; exon_number "1"; gene_type "
Dt_chr1	Cufflinks	exon	22279479	22279605	.	+	.	gene_id "XLOC_119126"; transcript_id "Novel_001606"; exon_number "2"; gene_type "
Dt_chr1	Cufflinks	exon	22279930	22280362	.	+	.	gene_id "XLOC_119126"; transcript_id "Novel_001606"; exon_number "3"; gene_type "
Dt_chr1	Cufflinks	exon	27316875	27316938	.	+	.	gene_id "XLOC_119452"; transcript_id "Novel_001607"; exon_number "1"; gene_type "
Dt_chr1	Cufflinks	exon	27317093	27317172	.	+	.	gene_id "XLOC_119452"; transcript_id "Novel_001607"; exon_number "2"; gene_type "
Dt_chr1	Cufflinks	exon	27317349	27317567	.	+	.	gene_id "XLOC_119452"; transcript_id "Novel_001607"; exon_number "3"; gene_type "
Dt_chr1	Cufflinks	exon	27317670	27318826	.	+	.	gene_id "XLOC_119452"; transcript_id "Novel_001607"; exon_number "4"; gene_type "
Dt_chr1	Cufflinks	exon	27721044	27721158	.	+	.	gene_id "XLOC_119492"; transcript_id "Novel_001608"; exon_number "1"; gene_type "
Dt_chr1	Cufflinks	exon	27721436	27722745	.	+	.	gene_id "XLOC_119492"; transcript_id "Novel_001608"; exon_number "2"; gene_type "
Dt_chr1	Cufflinks	exon	29552959	29553192	.	+	.	gene_id "XLOC_119616"; transcript_id "Novel_001609"; exon_number "1"; gene_type "
Dt_chr1	Cufflinks	exon	29553272	29554574	.	+	.	gene_id "XLOC_119616"; transcript_id "Novel_001609"; exon_number "2"; gene_type "
Dt_chr1	Cufflinks	exon	29555091	29555637	.	+	.	gene_id "XLOC_119616"; transcript_id "Novel_001609"; exon_number "3"; gene_type "
Dt_chr1	Cufflinks	exon	29552959	29553192	.	+	.	gene_id "XLOC_119616"; transcript_id "Novel_001610"; exon_number "1"; gene_type "
Dt_chr1	Cufflinks	exon	29553272	29553385	.	+	.	gene_id "XLOC_119616"; transcript_id "Novel_001610"; exon_number "2"; gene_type "

Dt_chr1	Cufflinks	exon	29553559	29555984	.	+	.	gene_id "XLOC_119616"; transcript_id "Novel_001610"; exon_number "3"; gene_type "
Dt_chr1	Cufflinks	exon	29622696	29622874	.	+	.	gene_id "XLOC_119621"; transcript_id "Novel_001611"; exon_number "1"; gene_type "
Dt_chr1	Cufflinks	exon	29622952	29623121	.	+	.	gene_id "XLOC_119621"; transcript_id "Novel_001611"; exon_number "2"; gene_type "
Dt_chr1	Cufflinks	exon	29623217	29623383	.	+	.	gene_id "XLOC_119621"; transcript_id "Novel_001611"; exon_number "3"; gene_type "
Dt_chr1	Cufflinks	exon	30499525	30499773	.	+	.	gene_id "XLOC_119673"; transcript_id "Novel_001612"; exon_number "1"; gene_type "
Dt_chr1	Cufflinks	exon	33620300	33620820	.	+	.	gene_id "XLOC_119968"; transcript_id "Novel_001613"; exon_number "1"; gene_type "
Dt_chr1	Cufflinks	exon	34757398	34757723	.	+	.	gene_id "XLOC_120068"; transcript_id "Novel_001614"; exon_number "1"; gene_type "
Dt_chr1	Cufflinks	exon	39792256	39792738	.	+	.	gene_id "XLOC_120754"; transcript_id "Novel_001615"; exon_number "1"; gene_type "
Dt_chr1	Cufflinks	exon	39793085	39793114	.	+	.	gene_id "XLOC_120754"; transcript_id "Novel_001615"; exon_number "2"; gene_type "
Dt_chr1	Cufflinks	exon	40627990	40628312	.	+	.	gene_id "XLOC_120891"; transcript_id "Novel_001616"; exon_number "1"; gene_type "
Dt_chr1	Cufflinks	exon	40628433	40628504	.	+	.	gene_id "XLOC_120891"; transcript_id "Novel_001616"; exon_number "2"; gene_type "
Dt_chr1	Cufflinks	exon	40629112	40629192	.	+	.	gene_id "XLOC_120891"; transcript_id "Novel_001616"; exon_number "3"; gene_type "
Dt_chr1	Cufflinks	exon	40631074	40631472	.	+	.	gene_id "XLOC_120891"; transcript_id "Novel_001616"; exon_number "4"; gene_type "
Dt_chr1	Cufflinks	exon	40628104	40628312	.	+	.	gene_id "XLOC_120891"; transcript_id "Novel_001617"; exon_number "1"; gene_type "
Dt_chr1	Cufflinks	exon	40628433	40628504	.	+	.	gene_id "XLOC_120891"; transcript_id "Novel_001617"; exon_number "2"; gene_type "
Dt_chr1	Cufflinks	exon	40629112	40629192	.	+	.	gene_id "XLOC_120891"; transcript_id "Novel_001617"; exon_number "3"; gene_type "
Dt_chr1	Cufflinks	exon	40630251	40630264	.	+	.	gene_id "XLOC_120891"; transcript_id "Novel_001617"; exon_number "4"; gene_type "
Dt_chr1	Cufflinks	exon	40735067	40735312	.	+	.	gene_id "XLOC_120910"; transcript_id "Novel_001618"; exon_number "1"; gene_type "
Dt_chr1	Cufflinks	exon	40899031	40899176	.	+	.	gene_id "XLOC_120953"; transcript_id "Novel_001619"; exon_number "1"; gene_type "
Dt_chr1	Cufflinks	exon	40901496	40901583	.	+	.	gene_id "XLOC_120953"; transcript_id "Novel_001619"; exon_number "2"; gene_type "
Dt_chr1	Cufflinks	exon	40975102	40975411	.	+	.	gene_id "XLOC_120961"; transcript_id "Novel_001620"; exon_number "1"; gene_type "
Dt_chr1	Cufflinks	exon	40975504	40975717	.	+	.	gene_id "XLOC_120961"; transcript_id "Novel_001620"; exon_number "2"; gene_type "
Dt_chr1	Cufflinks	exon	40975874	40976198	.	+	.	gene_id "XLOC_120961"; transcript_id "Novel_001620"; exon_number "3"; gene_type "
Dt_chr1	Cufflinks	exon	40976469	40976794	.	+	.	gene_id "XLOC_120961"; transcript_id "Novel_001621"; exon_number "1"; gene_type "
Dt_chr1	Cufflinks	exon	40977337	40977392	.	+	.	gene_id "XLOC_120961"; transcript_id "Novel_001621"; exon_number "2"; gene_type "
Dt_chr1	Cufflinks	exon	40977500	40977589	.	+	.	gene_id "XLOC_120961"; transcript_id "Novel_001621"; exon_number "3"; gene_type "
Dt_chr1	Cufflinks	exon	40977671	40977776	.	+	.	gene_id "XLOC_120961"; transcript_id "Novel_001621"; exon_number "4"; gene_type "
Dt_chr1	Cufflinks	exon	40978019	40978104	.	+	.	gene_id "XLOC_120962"; transcript_id "Novel_001622"; exon_number "1"; gene_type "
Dt_chr1	Cufflinks	exon	40978200	40978328	.	+	.	gene_id "XLOC_120962"; transcript_id "Novel_001622"; exon_number "2"; gene_type "
Dt_chr1	Cufflinks	exon	40978406	40978480	.	+	.	gene_id "XLOC_120962"; transcript_id "Novel_001622"; exon_number "3"; gene_type "
Dt_chr1	Cufflinks	exon	40978994	40979162	.	+	.	gene_id "XLOC_120962"; transcript_id "Novel_001622"; exon_number "4"; gene_type "
Dt_chr1	Cufflinks	exon	40979683	40979820	.	+	.	gene_id "XLOC_120962"; transcript_id "Novel_001622"; exon_number "5"; gene_type "
Dt_chr1	Cufflinks	exon	41079018	41079181	.	+	.	gene_id "XLOC_120977"; transcript_id "Novel_001623"; exon_number "1"; gene_type "
Dt_chr1	Cufflinks	exon	41079695	41080218	.	+	.	gene_id "XLOC_120977"; transcript_id "Novel_001623"; exon_number "2"; gene_type "
Dt_chr1	Cufflinks	exon	41382150	41382191	.	+	.	gene_id "XLOC_121028"; transcript_id "Novel_001624"; exon_number "1"; gene_type "

Dt_chr1	Cufflinks	exon	41382548	41383665	.	+	.	gene_id "XLOC_121028"; transcript_id "Novel_001624"; exon_number "2"; gene_type "
Dt_chr1	Cufflinks	exon	41554102	41554176	.	+	.	gene_id "XLOC_121067"; transcript_id "Novel_001625"; exon_number "1"; gene_type "
Dt_chr1	Cufflinks	exon	41554264	41554656	.	+	.	gene_id "XLOC_121067"; transcript_id "Novel_001625"; exon_number "2"; gene_type "
Dt_chr1	Cufflinks	exon	41554751	41554853	.	+	.	gene_id "XLOC_121067"; transcript_id "Novel_001625"; exon_number "3"; gene_type "
Dt_chr1	Cufflinks	exon	41555305	41555425	.	+	.	gene_id "XLOC_121067"; transcript_id "Novel_001625"; exon_number "4"; gene_type "
Dt_chr1	Cufflinks	exon	41555518	41555784	.	+	.	gene_id "XLOC_121067"; transcript_id "Novel_001625"; exon_number "5"; gene_type "
Dt_chr1	Cufflinks	exon	41555918	41556251	.	+	.	gene_id "XLOC_121067"; transcript_id "Novel_001625"; exon_number "6"; gene_type "
Dt_chr1	Cufflinks	exon	41556838	41556925	.	+	.	gene_id "XLOC_121067"; transcript_id "Novel_001626"; exon_number "1"; gene_type "
Dt_chr1	Cufflinks	exon	41557050	41557263	.	+	.	gene_id "XLOC_121067"; transcript_id "Novel_001626"; exon_number "2"; gene_type "
Dt_chr1	Cufflinks	exon	41557357	41557497	.	+	.	gene_id "XLOC_121067"; transcript_id "Novel_001626"; exon_number "3"; gene_type "
Dt_chr1	Cufflinks	exon	41556974	41557263	.	+	.	gene_id "XLOC_121067"; transcript_id "Novel_001627"; exon_number "1"; gene_type "
Dt_chr1	Cufflinks	exon	41557357	41557454	.	+	.	gene_id "XLOC_121067"; transcript_id "Novel_001627"; exon_number "2"; gene_type "
Dt_chr1	Cufflinks	exon	41786959	41787440	.	+	.	gene_id "XLOC_121111"; transcript_id "Novel_001628"; exon_number "1"; gene_type "
Dt_chr1	Cufflinks	exon	41788427	41788546	.	+	.	gene_id "XLOC_121111"; transcript_id "Novel_001628"; exon_number "2"; gene_type "
Dt_chr1	Cufflinks	exon	41789318	41789438	.	+	.	gene_id "XLOC_121111"; transcript_id "Novel_001628"; exon_number "3"; gene_type "
Dt_chr1	Cufflinks	exon	41789524	41789676	.	+	.	gene_id "XLOC_121111"; transcript_id "Novel_001628"; exon_number "4"; gene_type "
Dt_chr1	Cufflinks	exon	42154441	42155166	.	+	.	gene_id "XLOC_121158"; transcript_id "Novel_001629"; exon_number "1"; gene_type "
Dt_chr1	Cufflinks	exon	42155258	42156106	.	+	.	gene_id "XLOC_121158"; transcript_id "Novel_001629"; exon_number "2"; gene_type "
Dt_chr1	Cufflinks	exon	42521028	42521328	.	+	.	gene_id "XLOC_121216"; transcript_id "Novel_001630"; exon_number "1"; gene_type "
Dt_chr1	Cufflinks	exon	42521423	42521480	.	+	.	gene_id "XLOC_121216"; transcript_id "Novel_001630"; exon_number "2"; gene_type "
Dt_chr1	Cufflinks	exon	42607810	42608155	.	+	.	gene_id "XLOC_121233"; transcript_id "Novel_001631"; exon_number "1"; gene_type "
Dt_chr1	Cufflinks	exon	42608868	42608926	.	+	.	gene_id "XLOC_121233"; transcript_id "Novel_001631"; exon_number "2"; gene_type "
Dt_chr1	Cufflinks	exon	42609010	42609677	.	+	.	gene_id "XLOC_121233"; transcript_id "Novel_001631"; exon_number "3"; gene_type "
Dt_chr1	Cufflinks	exon	42609750	42610053	.	+	.	gene_id "XLOC_121233"; transcript_id "Novel_001631"; exon_number "4"; gene_type "
Dt_chr1	Cufflinks	exon	42607886	42608155	.	+	.	gene_id "XLOC_121233"; transcript_id "Novel_001632"; exon_number "1"; gene_type "
Dt_chr1	Cufflinks	exon	42608868	42609443	.	+	.	gene_id "XLOC_121233"; transcript_id "Novel_001632"; exon_number "2"; gene_type "
Dt_chr1	Cufflinks	exon	42609750	42609821	.	+	.	gene_id "XLOC_121233"; transcript_id "Novel_001632"; exon_number "3"; gene_type "
Dt_chr1	Cufflinks	exon	42607888	42608155	.	+	.	gene_id "XLOC_121233"; transcript_id "Novel_001633"; exon_number "1"; gene_type "
Dt_chr1	Cufflinks	exon	42609010	42609526	.	+	.	gene_id "XLOC_121233"; transcript_id "Novel_001633"; exon_number "2"; gene_type "
Dt_chr1	Cufflinks	exon	42683837	42684563	.	+	.	gene_id "XLOC_121240"; transcript_id "Novel_001634"; exon_number "1"; gene_type "
Dt_chr1	Cufflinks	exon	42845093	42845347	.	+	.	gene_id "XLOC_121277"; transcript_id "Novel_001635"; exon_number "1"; gene_type "
Dt_chr1	Cufflinks	exon	42845686	42847932	.	+	.	gene_id "XLOC_121277"; transcript_id "Novel_001635"; exon_number "2"; gene_type "
Dt_chr1	Cufflinks	exon	42845180	42845347	.	+	.	gene_id "XLOC_121277"; transcript_id "Novel_001636"; exon_number "1"; gene_type "
Dt_chr1	Cufflinks	exon	42845686	42846708	.	+	.	gene_id "XLOC_121277"; transcript_id "Novel_001636"; exon_number "2"; gene_type "
Dt_chr1	Cufflinks	exon	42846806	42846831	.	+	.	gene_id "XLOC_121277"; transcript_id "Novel_001636"; exon_number "3"; gene_type "

Dt_chr1	Cufflinks	exon	42847277	42847348	.	+	.	gene_id "XLOC_121277"; transcript_id "Novel_001636"; exon_number "4"; gene_type "
Dt_chr1	Cufflinks	exon	42847602	42847658	.	+	.	gene_id "XLOC_121277"; transcript_id "Novel_001636"; exon_number "5"; gene_type "
Dt_chr1	Cufflinks	exon	42847756	42848210	.	+	.	gene_id "XLOC_121277"; transcript_id "Novel_001636"; exon_number "6"; gene_type "
Dt_chr1	Cufflinks	exon	42996406	42997227	.	+	.	gene_id "XLOC_121303"; transcript_id "Novel_001637"; exon_number "1"; gene_type "
Dt_chr1	Cufflinks	exon	42997314	42997820	.	+	.	gene_id "XLOC_121303"; transcript_id "Novel_001637"; exon_number "2"; gene_type "
Dt_chr1	Cufflinks	exon	43031208	43031605	.	+	.	gene_id "XLOC_121309"; transcript_id "Novel_001638"; exon_number "1"; gene_type "
Dt_chr1	Cufflinks	exon	43031727	43031986	.	+	.	gene_id "XLOC_121309"; transcript_id "Novel_001638"; exon_number "2"; gene_type "
Dt_chr1	Cufflinks	exon	43156211	43157238	.	+	.	gene_id "XLOC_121347"; transcript_id "Novel_001639"; exon_number "1"; gene_type "
Dt_chr1	Cufflinks	exon	43157664	43157675	.	+	.	gene_id "XLOC_121347"; transcript_id "Novel_001639"; exon_number "2"; gene_type "
Dt_chr1	Cufflinks	exon	43162475	43162558	.	+	.	gene_id "XLOC_121349"; transcript_id "Novel_001640"; exon_number "1"; gene_type "
Dt_chr1	Cufflinks	exon	43162647	43162782	.	+	.	gene_id "XLOC_121349"; transcript_id "Novel_001640"; exon_number "2"; gene_type "
Dt_chr1	Cufflinks	exon	43163152	43163253	.	+	.	gene_id "XLOC_121349"; transcript_id "Novel_001640"; exon_number "3"; gene_type "
Dt_chr1	Cufflinks	exon	43163341	43164466	.	+	.	gene_id "XLOC_121349"; transcript_id "Novel_001640"; exon_number "4"; gene_type "
Dt_chr1	Cufflinks	exon	43209430	43209504	.	+	.	gene_id "XLOC_121360"; transcript_id "Novel_001641"; exon_number "1"; gene_type "
Dt_chr1	Cufflinks	exon	43209822	43209950	.	+	.	gene_id "XLOC_121360"; transcript_id "Novel_001641"; exon_number "2"; gene_type "
Dt_chr1	Cufflinks	exon	43210129	43211246	.	+	.	gene_id "XLOC_121360"; transcript_id "Novel_001641"; exon_number "3"; gene_type "
Dt_chr1	Cufflinks	exon	43211654	43211896	.	+	.	gene_id "XLOC_121361"; transcript_id "Novel_001642"; exon_number "1"; gene_type "
Dt_chr1	Cufflinks	exon	43212240	43212526	.	+	.	gene_id "XLOC_121361"; transcript_id "Novel_001642"; exon_number "2"; gene_type "
Dt_chr1	Cufflinks	exon	43212675	43212784	.	+	.	gene_id "XLOC_121361"; transcript_id "Novel_001642"; exon_number "3"; gene_type "
Dt_chr1	Cufflinks	exon	43212926	43213087	.	+	.	gene_id "XLOC_121362"; transcript_id "Novel_001643"; exon_number "1"; gene_type "
Dt_chr1	Cufflinks	exon	43213172	43213629	.	+	.	gene_id "XLOC_121362"; transcript_id "Novel_001643"; exon_number "2"; gene_type "
Dt_chr1	Cufflinks	exon	43251437	43253460	.	+	.	gene_id "XLOC_121366"; transcript_id "Novel_001644"; exon_number "1"; gene_type "
Dt_chr1	Cufflinks	exon	43254100	43254587	.	+	.	gene_id "XLOC_121366"; transcript_id "Novel_001644"; exon_number "2"; gene_type "
Dt_chr1	Cufflinks	exon	43251440	43253108	.	+	.	gene_id "XLOC_121366"; transcript_id "Novel_001645"; exon_number "1"; gene_type "
Dt_chr1	Cufflinks	exon	43253303	43253460	.	+	.	gene_id "XLOC_121366"; transcript_id "Novel_001645"; exon_number "2"; gene_type "
Dt_chr1	Cufflinks	exon	43254100	43254181	.	+	.	gene_id "XLOC_121366"; transcript_id "Novel_001645"; exon_number "3"; gene_type "
Dt_chr1	Cufflinks	exon	43254763	43255510	.	+	.	gene_id "XLOC_121366"; transcript_id "Novel_001645"; exon_number "4"; gene_type "
Dt_chr1	Cufflinks	exon	43258891	43258956	.	+	.	gene_id "XLOC_121368"; transcript_id "Novel_001646"; exon_number "1"; gene_type "
Dt_chr1	Cufflinks	exon	43259155	43259233	.	+	.	gene_id "XLOC_121368"; transcript_id "Novel_001646"; exon_number "2"; gene_type "
Dt_chr1	Cufflinks	exon	43259386	43259893	.	+	.	gene_id "XLOC_121368"; transcript_id "Novel_001646"; exon_number "3"; gene_type "
Dt_chr1	Cufflinks	exon	43261758	43261887	.	+	.	gene_id "XLOC_121368"; transcript_id "Novel_001646"; exon_number "4"; gene_type "
Dt_chr1	Cufflinks	exon	43262139	43262723	.	+	.	gene_id "XLOC_121368"; transcript_id "Novel_001646"; exon_number "5"; gene_type "
Dt_chr1	Cufflinks	exon	43258891	43258956	.	+	.	gene_id "XLOC_121368"; transcript_id "Novel_001647"; exon_number "1"; gene_type "
Dt_chr1	Cufflinks	exon	43259155	43259233	.	+	.	gene_id "XLOC_121368"; transcript_id "Novel_001647"; exon_number "2"; gene_type "
Dt_chr1	Cufflinks	exon	43259386	43259505	.	+	.	gene_id "XLOC_121368"; transcript_id "Novel_001647"; exon_number "3"; gene_type "

Dt_chr1	Cufflinks	exon	43259765	43259893	.	+	.	gene_id "XLOC_121368"; transcript_id "Novel_001647"; exon_number "4"; gene_type "
Dt_chr1	Cufflinks	exon	43261758	43261887	.	+	.	gene_id "XLOC_121368"; transcript_id "Novel_001647"; exon_number "5"; gene_type "
Dt_chr1	Cufflinks	exon	43262139	43262723	.	+	.	gene_id "XLOC_121368"; transcript_id "Novel_001647"; exon_number "6"; gene_type "
Dt_chr1	Cufflinks	exon	43259903	43261887	.	+	.	gene_id "XLOC_121368"; transcript_id "Novel_001648"; exon_number "1"; gene_type "
Dt_chr1	Cufflinks	exon	43262139	43262489	.	+	.	gene_id "XLOC_121368"; transcript_id "Novel_001648"; exon_number "2"; gene_type "
Dt_chr1	Cufflinks	exon	43292138	43292369	.	+	.	gene_id "XLOC_121373"; transcript_id "Novel_001649"; exon_number "1"; gene_type "
Dt_chr1	Cufflinks	exon	43293414	43294371	.	+	.	gene_id "XLOC_121373"; transcript_id "Novel_001649"; exon_number "2"; gene_type "
Dt_chr1	Cufflinks	exon	43336968	43338345	.	+	.	gene_id "XLOC_121390"; transcript_id "Novel_001650"; exon_number "1"; gene_type "
Dt_chr1	Cufflinks	exon	43338476	43340838	.	+	.	gene_id "XLOC_121390"; transcript_id "Novel_001650"; exon_number "2"; gene_type "
Dt_chr1	Cufflinks	exon	43336985	43337274	.	+	.	gene_id "XLOC_121390"; transcript_id "Novel_001651"; exon_number "1"; gene_type "
Dt_chr1	Cufflinks	exon	43337593	43337722	.	+	.	gene_id "XLOC_121390"; transcript_id "Novel_001651"; exon_number "2"; gene_type "
Dt_chr1	Cufflinks	exon	43337821	43338345	.	+	.	gene_id "XLOC_121390"; transcript_id "Novel_001651"; exon_number "3"; gene_type "
Dt_chr1	Cufflinks	exon	43338476	43340364	.	+	.	gene_id "XLOC_121390"; transcript_id "Novel_001651"; exon_number "4"; gene_type "
Dt_chr1	Cufflinks	exon	43337056	43337274	.	+	.	gene_id "XLOC_121390"; transcript_id "Novel_001652"; exon_number "1"; gene_type "
Dt_chr1	Cufflinks	exon	43337593	43338345	.	+	.	gene_id "XLOC_121390"; transcript_id "Novel_001652"; exon_number "2"; gene_type "
Dt_chr1	Cufflinks	exon	43338476	43340677	.	+	.	gene_id "XLOC_121390"; transcript_id "Novel_001652"; exon_number "3"; gene_type "
Dt_chr1	Cufflinks	exon	43354876	43356623	.	+	.	gene_id "XLOC_121398"; transcript_id "Novel_001653"; exon_number "1"; gene_type "
Dt_chr1	Cufflinks	exon	43356915	43357070	.	+	.	gene_id "XLOC_121398"; transcript_id "Novel_001653"; exon_number "2"; gene_type "
Dt_chr1	Cufflinks	exon	43357579	43357830	.	+	.	gene_id "XLOC_121398"; transcript_id "Novel_001653"; exon_number "3"; gene_type "
Dt_chr1	Cufflinks	exon	43357909	43358509	.	+	.	gene_id "XLOC_121398"; transcript_id "Novel_001653"; exon_number "4"; gene_type "
Dt_chr1	Cufflinks	exon	43354876	43355234	.	+	.	gene_id "XLOC_121398"; transcript_id "Novel_001654"; exon_number "1"; gene_type "
Dt_chr1	Cufflinks	exon	43356915	43357070	.	+	.	gene_id "XLOC_121398"; transcript_id "Novel_001654"; exon_number "2"; gene_type "
Dt_chr1	Cufflinks	exon	43357579	43357830	.	+	.	gene_id "XLOC_121398"; transcript_id "Novel_001654"; exon_number "3"; gene_type "
Dt_chr1	Cufflinks	exon	43357909	43358509	.	+	.	gene_id "XLOC_121398"; transcript_id "Novel_001654"; exon_number "4"; gene_type "
Dt_chr1	Cufflinks	exon	43354876	43356005	.	+	.	gene_id "XLOC_121398"; transcript_id "Novel_001655"; exon_number "1"; gene_type "
Dt_chr1	Cufflinks	exon	43356556	43356623	.	+	.	gene_id "XLOC_121398"; transcript_id "Novel_001655"; exon_number "2"; gene_type "
Dt_chr1	Cufflinks	exon	43356915	43357070	.	+	.	gene_id "XLOC_121398"; transcript_id "Novel_001655"; exon_number "3"; gene_type "
Dt_chr1	Cufflinks	exon	43357579	43357830	.	+	.	gene_id "XLOC_121398"; transcript_id "Novel_001655"; exon_number "4"; gene_type "
Dt_chr1	Cufflinks	exon	43357909	43358509	.	+	.	gene_id "XLOC_121398"; transcript_id "Novel_001655"; exon_number "5"; gene_type "
Dt_chr1	Cufflinks	exon	43397431	43397807	.	+	.	gene_id "XLOC_121400"; transcript_id "Novel_001656"; exon_number "1"; gene_type "
Dt_chr1	Cufflinks	exon	43397913	43398062	.	+	.	gene_id "XLOC_121400"; transcript_id "Novel_001656"; exon_number "2"; gene_type "
Dt_chr1	Cufflinks	exon	43401968	43402039	.	+	.	gene_id "XLOC_121400"; transcript_id "Novel_001657"; exon_number "1"; gene_type "
Dt_chr1	Cufflinks	exon	43402128	43402464	.	+	.	gene_id "XLOC_121400"; transcript_id "Novel_001657"; exon_number "2"; gene_type "
Dt_chr1	Cufflinks	exon	43402566	43402655	.	+	.	gene_id "XLOC_121401"; transcript_id "Novel_001658"; exon_number "1"; gene_type "
Dt_chr1	Cufflinks	exon	43402737	43402836	.	+	.	gene_id "XLOC_121401"; transcript_id "Novel_001658"; exon_number "2"; gene_type "

Dt_chr1	Cufflinks	exon	43403390	43403539	.	+	.	gene_id "XLOC_121401"; transcript_id "Novel_001658"; exon_number "3"; gene_type "
Dt_chr1	Cufflinks	exon	43403965	43404515	.	+	.	gene_id "XLOC_121401"; transcript_id "Novel_001658"; exon_number "4"; gene_type "
Dt_chr1	Cufflinks	exon	43568403	43568650	.	+	.	gene_id "XLOC_121425"; transcript_id "Novel_001659"; exon_number "1"; gene_type "
Dt_chr1	Cufflinks	exon	43568749	43569734	.	+	.	gene_id "XLOC_121425"; transcript_id "Novel_001659"; exon_number "2"; gene_type "
Dt_chr1	Cufflinks	exon	43568403	43568650	.	+	.	gene_id "XLOC_121425"; transcript_id "Novel_001660"; exon_number "1"; gene_type "
Dt_chr1	Cufflinks	exon	43568749	43568789	.	+	.	gene_id "XLOC_121425"; transcript_id "Novel_001660"; exon_number "2"; gene_type "
Dt_chr1	Cufflinks	exon	43569111	43569166	.	+	.	gene_id "XLOC_121425"; transcript_id "Novel_001660"; exon_number "3"; gene_type "
Dt_chr1	Cufflinks	exon	43570149	43570200	.	+	.	gene_id "XLOC_121425"; transcript_id "Novel_001660"; exon_number "4"; gene_type "
Dt_chr1	Cufflinks	exon	43570287	43570372	.	+	.	gene_id "XLOC_121425"; transcript_id "Novel_001660"; exon_number "5"; gene_type "
Dt_chr1	Cufflinks	exon	43570505	43571892	.	+	.	gene_id "XLOC_121425"; transcript_id "Novel_001660"; exon_number "6"; gene_type "
Dt_chr1	Cufflinks	exon	43568442	43568650	.	+	.	gene_id "XLOC_121425"; transcript_id "Novel_001661"; exon_number "1"; gene_type "
Dt_chr1	Cufflinks	exon	43568749	43568789	.	+	.	gene_id "XLOC_121425"; transcript_id "Novel_001661"; exon_number "2"; gene_type "
Dt_chr1	Cufflinks	exon	43569111	43569166	.	+	.	gene_id "XLOC_121425"; transcript_id "Novel_001661"; exon_number "3"; gene_type "
Dt_chr1	Cufflinks	exon	43570149	43570200	.	+	.	gene_id "XLOC_121425"; transcript_id "Novel_001661"; exon_number "4"; gene_type "
Dt_chr1	Cufflinks	exon	43570287	43570372	.	+	.	gene_id "XLOC_121425"; transcript_id "Novel_001661"; exon_number "5"; gene_type "
Dt_chr1	Cufflinks	exon	43570505	43570645	.	+	.	gene_id "XLOC_121425"; transcript_id "Novel_001661"; exon_number "6"; gene_type "
Dt_chr1	Cufflinks	exon	43571355	43572213	.	+	.	gene_id "XLOC_121425"; transcript_id "Novel_001661"; exon_number "7"; gene_type "
Dt_chr1	Cufflinks	exon	43721031	43721215	.	+	.	gene_id "XLOC_121441"; transcript_id "Novel_001662"; exon_number "1"; gene_type "
Dt_chr1	Cufflinks	exon	43721828	43722459	.	+	.	gene_id "XLOC_121441"; transcript_id "Novel_001662"; exon_number "2"; gene_type "
Dt_chr1	Cufflinks	exon	43721031	43721215	.	+	.	gene_id "XLOC_121441"; transcript_id "Novel_001663"; exon_number "1"; gene_type "
Dt_chr1	Cufflinks	exon	43721828	43721927	.	+	.	gene_id "XLOC_121441"; transcript_id "Novel_001663"; exon_number "2"; gene_type "
Dt_chr1	Cufflinks	exon	43722227	43722324	.	+	.	gene_id "XLOC_121441"; transcript_id "Novel_001663"; exon_number "3"; gene_type "
Dt_chr1	Cufflinks	exon	43722411	43722459	.	+	.	gene_id "XLOC_121441"; transcript_id "Novel_001663"; exon_number "4"; gene_type "
Dt_chr1	Cufflinks	exon	43721036	43721927	.	+	.	gene_id "XLOC_121441"; transcript_id "Novel_001664"; exon_number "1"; gene_type "
Dt_chr1	Cufflinks	exon	43722227	43722324	.	+	.	gene_id "XLOC_121441"; transcript_id "Novel_001664"; exon_number "2"; gene_type "
Dt_chr1	Cufflinks	exon	43722422	43722459	.	+	.	gene_id "XLOC_121441"; transcript_id "Novel_001664"; exon_number "3"; gene_type "
Dt_chr1	Cufflinks	exon	43721066	43721215	.	+	.	gene_id "XLOC_121441"; transcript_id "Novel_001665"; exon_number "1"; gene_type "
Dt_chr1	Cufflinks	exon	43721828	43721927	.	+	.	gene_id "XLOC_121441"; transcript_id "Novel_001665"; exon_number "2"; gene_type "
Dt_chr1	Cufflinks	exon	43722227	43722324	.	+	.	gene_id "XLOC_121441"; transcript_id "Novel_001665"; exon_number "3"; gene_type "
Dt_chr1	Cufflinks	exon	43722422	43722456	.	+	.	gene_id "XLOC_121441"; transcript_id "Novel_001665"; exon_number "4"; gene_type "
Dt_chr1	Cufflinks	exon	43827902	43828087	.	+	.	gene_id "XLOC_121459"; transcript_id "Novel_001666"; exon_number "1"; gene_type "
Dt_chr1	Cufflinks	exon	43828540	43828600	.	+	.	gene_id "XLOC_121459"; transcript_id "Novel_001666"; exon_number "2"; gene_type "
Dt_chr1	Cufflinks	exon	43828700	43828963	.	+	.	gene_id "XLOC_121459"; transcript_id "Novel_001666"; exon_number "3"; gene_type "
Dt_chr1	Cufflinks	exon	43829187	43829423	.	+	.	gene_id "XLOC_121459"; transcript_id "Novel_001666"; exon_number "4"; gene_type "
Dt_chr1	Cufflinks	exon	43829512	43829633	.	+	.	gene_id "XLOC_121459"; transcript_id "Novel_001666"; exon_number "5"; gene_type "

Dt_chr1	Cufflinks	exon	43829721	43830197	.	+	.	gene_id "XLOC_121459"; transcript_id "Novel_001666"; exon_number "6"; gene_type "
Dt_chr1	Cufflinks	exon	43830548	43831173	.	+	.	gene_id "XLOC_121459"; transcript_id "Novel_001666"; exon_number "7"; gene_type "
Dt_chr1	Cufflinks	exon	43827918	43828087	.	+	.	gene_id "XLOC_121459"; transcript_id "Novel_001667"; exon_number "1"; gene_type "
Dt_chr1	Cufflinks	exon	43828540	43828600	.	+	.	gene_id "XLOC_121459"; transcript_id "Novel_001667"; exon_number "2"; gene_type "
Dt_chr1	Cufflinks	exon	43828700	43829202	.	+	.	gene_id "XLOC_121459"; transcript_id "Novel_001667"; exon_number "3"; gene_type "
Dt_chr1	Cufflinks	exon	43827918	43828087	.	+	.	gene_id "XLOC_121459"; transcript_id "Novel_001668"; exon_number "1"; gene_type "
Dt_chr1	Cufflinks	exon	43828540	43828600	.	+	.	gene_id "XLOC_121459"; transcript_id "Novel_001668"; exon_number "2"; gene_type "
Dt_chr1	Cufflinks	exon	43828700	43828963	.	+	.	gene_id "XLOC_121459"; transcript_id "Novel_001668"; exon_number "3"; gene_type "
Dt_chr1	Cufflinks	exon	43829187	43829633	.	+	.	gene_id "XLOC_121459"; transcript_id "Novel_001668"; exon_number "4"; gene_type "
Dt_chr1	Cufflinks	exon	43829721	43830197	.	+	.	gene_id "XLOC_121459"; transcript_id "Novel_001668"; exon_number "5"; gene_type "
Dt_chr1	Cufflinks	exon	43830548	43832101	.	+	.	gene_id "XLOC_121459"; transcript_id "Novel_001668"; exon_number "6"; gene_type "
Dt_chr1	Cufflinks	exon	44538917	44539242	.	+	.	gene_id "XLOC_121539"; transcript_id "Novel_001669"; exon_number "1"; gene_type "
Dt_chr1	Cufflinks	exon	44540054	44540168	.	+	.	gene_id "XLOC_121539"; transcript_id "Novel_001669"; exon_number "2"; gene_type "
Dt_chr1	Cufflinks	exon	44540676	44540851	.	+	.	gene_id "XLOC_121539"; transcript_id "Novel_001669"; exon_number "3"; gene_type "
Dt_chr1	Cufflinks	exon	44541489	44541584	.	+	.	gene_id "XLOC_121539"; transcript_id "Novel_001669"; exon_number "4"; gene_type "
Dt_chr1	Cufflinks	exon	44541668	44541825	.	+	.	gene_id "XLOC_121539"; transcript_id "Novel_001669"; exon_number "5"; gene_type "
Dt_chr1	Cufflinks	exon	50908659	50909217	.	+	.	gene_id "XLOC_122229"; transcript_id "Novel_001670"; exon_number "1"; gene_type "
Dt_chr1	Cufflinks	exon	50911447	50911562	.	+	.	gene_id "XLOC_122229"; transcript_id "Novel_001670"; exon_number "2"; gene_type "
Dt_chr1	Cufflinks	exon	50911660	50912213	.	+	.	gene_id "XLOC_122229"; transcript_id "Novel_001670"; exon_number "3"; gene_type "
Dt_chr1	Cufflinks	exon	53568732	53570826	.	+	.	gene_id "XLOC_122490"; transcript_id "Novel_001671"; exon_number "1"; gene_type "
Dt_chr1	Cufflinks	exon	53570967	53571314	.	+	.	gene_id "XLOC_122490"; transcript_id "Novel_001671"; exon_number "2"; gene_type "
Dt_chr1	Cufflinks	exon	53568893	53569407	.	+	.	gene_id "XLOC_122490"; transcript_id "Novel_001672"; exon_number "1"; gene_type "
Dt_chr1	Cufflinks	exon	53569834	53569918	.	+	.	gene_id "XLOC_122490"; transcript_id "Novel_001672"; exon_number "2"; gene_type "
Dt_chr1	Cufflinks	exon	53570785	53570826	.	+	.	gene_id "XLOC_122490"; transcript_id "Novel_001672"; exon_number "3"; gene_type "
Dt_chr1	Cufflinks	exon	53570967	53571549	.	+	.	gene_id "XLOC_122490"; transcript_id "Novel_001672"; exon_number "4"; gene_type "
Dt_chr1	Cufflinks	exon	53837728	53838007	.	+	.	gene_id "XLOC_122507"; transcript_id "Novel_001673"; exon_number "1"; gene_type "
Dt_chr1	Cufflinks	exon	53950956	53951340	.	+	.	gene_id "XLOC_122523"; transcript_id "Novel_001674"; exon_number "1"; gene_type "
Dt_chr1	Cufflinks	exon	53952684	53954046	.	+	.	gene_id "XLOC_122523"; transcript_id "Novel_001674"; exon_number "2"; gene_type "
Dt_chr1	Cufflinks	exon	53950956	53951340	.	+	.	gene_id "XLOC_122523"; transcript_id "Novel_001675"; exon_number "1"; gene_type "
Dt_chr1	Cufflinks	exon	53952684	53952770	.	+	.	gene_id "XLOC_122523"; transcript_id "Novel_001675"; exon_number "2"; gene_type "
Dt_chr1	Cufflinks	exon	53953114	53953206	.	+	.	gene_id "XLOC_122523"; transcript_id "Novel_001675"; exon_number "3"; gene_type "
Dt_chr1	Cufflinks	exon	53953718	53953772	.	+	.	gene_id "XLOC_122523"; transcript_id "Novel_001675"; exon_number "4"; gene_type "
Dt_chr1	Cufflinks	exon	53954032	53954046	.	+	.	gene_id "XLOC_122523"; transcript_id "Novel_001675"; exon_number "5"; gene_type "
Dt_chr1	Cufflinks	exon	53950964	53951353	.	+	.	gene_id "XLOC_122523"; transcript_id "Novel_001676"; exon_number "1"; gene_type "
Dt_chr1	Cufflinks	exon	53952684	53952770	.	+	.	gene_id "XLOC_122523"; transcript_id "Novel_001676"; exon_number "2"; gene_type "

Dt_chr1	Cufflinks	exon	53953114	53953206	.	+	.	gene_id "XLOC_122523"; transcript_id "Novel_001676"; exon_number "3"; gene_type "
Dt_chr1	Cufflinks	exon	53953718	53953772	.	+	.	gene_id "XLOC_122523"; transcript_id "Novel_001676"; exon_number "4"; gene_type "
Dt_chr1	Cufflinks	exon	53954032	53954059	.	+	.	gene_id "XLOC_122523"; transcript_id "Novel_001676"; exon_number "5"; gene_type "
Dt_chr1	Cufflinks	exon	54736403	54736424	.	+	.	gene_id "XLOC_122630"; transcript_id "Novel_001677"; exon_number "1"; gene_type "
Dt_chr1	Cufflinks	exon	54736510	54736752	.	+	.	gene_id "XLOC_122630"; transcript_id "Novel_001677"; exon_number "2"; gene_type "
Dt_chr1	Cufflinks	exon	55027878	55029144	.	+	.	gene_id "XLOC_122666"; transcript_id "Novel_001678"; exon_number "1"; gene_type "
Dt_chr1	Cufflinks	exon	55030191	55031187	.	+	.	gene_id "XLOC_122666"; transcript_id "Novel_001678"; exon_number "2"; gene_type "
Dt_chr1	Cufflinks	exon	57763474	57763738	.	+	.	gene_id "XLOC_122909"; transcript_id "Novel_001679"; exon_number "1"; gene_type "
Dt_chr1	Cufflinks	exon	57764014	57764208	.	+	.	gene_id "XLOC_122909"; transcript_id "Novel_001679"; exon_number "2"; gene_type "
Dt_chr1	Cufflinks	exon	57763474	57763623	.	+	.	gene_id "XLOC_122909"; transcript_id "Novel_001680"; exon_number "1"; gene_type "
Dt_chr1	Cufflinks	exon	57764014	57764208	.	+	.	gene_id "XLOC_122909"; transcript_id "Novel_001680"; exon_number "2"; gene_type "
Dt_chr1	Cufflinks	exon	60370272	60370307	.	+	.	gene_id "XLOC_123105"; transcript_id "Novel_001681"; exon_number "1"; gene_type "
Dt_chr1	Cufflinks	exon	60370619	60371108	.	+	.	gene_id "XLOC_123105"; transcript_id "Novel_001681"; exon_number "2"; gene_type "
Dt_chr1	Cufflinks	exon	61683491	61683611	.	+	.	gene_id "XLOC_123173"; transcript_id "Novel_001682"; exon_number "1"; gene_type "
Dt_chr1	Cufflinks	exon	61684196	61684545	.	+	.	gene_id "XLOC_123173"; transcript_id "Novel_001682"; exon_number "2"; gene_type "
Dt_chr1	Cufflinks	exon	64083744	64083999	.	+	.	gene_id "XLOC_123326"; transcript_id "Novel_001683"; exon_number "1"; gene_type "
Dt_chr1	Cufflinks	exon	64084891	64085549	.	+	.	gene_id "XLOC_123326"; transcript_id "Novel_001683"; exon_number "2"; gene_type "
Dt_chr1	Cufflinks	exon	64085802	64086637	.	+	.	gene_id "XLOC_123326"; transcript_id "Novel_001683"; exon_number "3"; gene_type "
Dt_chr1	Cufflinks	exon	64083744	64083999	.	+	.	gene_id "XLOC_123326"; transcript_id "Novel_001684"; exon_number "1"; gene_type "
Dt_chr1	Cufflinks	exon	64084891	64086637	.	+	.	gene_id "XLOC_123326"; transcript_id "Novel_001684"; exon_number "2"; gene_type "
Dt_chr1	Cufflinks	exon	64084261	64085549	.	+	.	gene_id "XLOC_123326"; transcript_id "Novel_001685"; exon_number "1"; gene_type "
Dt_chr1	Cufflinks	exon	64085802	64086154	.	+	.	gene_id "XLOC_123326"; transcript_id "Novel_001685"; exon_number "2"; gene_type "
Dt_chr1	Cufflinks	exon	71612664	71613054	.	+	.	gene_id "XLOC_123718"; transcript_id "Novel_001686"; exon_number "1"; gene_type "
Dt_chr1	Cufflinks	exon	71613416	71613692	.	+	.	gene_id "XLOC_123718"; transcript_id "Novel_001686"; exon_number "2"; gene_type "
Dt_chr1	Cufflinks	exon	72369079	72369379	.	+	.	gene_id "XLOC_123747"; transcript_id "Novel_001687"; exon_number "1"; gene_type "
Dt_chr1	Cufflinks	exon	72369687	72369747	.	+	.	gene_id "XLOC_123747"; transcript_id "Novel_001687"; exon_number "2"; gene_type "
Dt_chr1	Cufflinks	exon	75302705	75302888	.	+	.	gene_id "XLOC_123898"; transcript_id "Novel_001688"; exon_number "1"; gene_type "
Dt_chr1	Cufflinks	exon	75303343	75303400	.	+	.	gene_id "XLOC_123898"; transcript_id "Novel_001688"; exon_number "2"; gene_type "
Dt_chr1	Cufflinks	exon	75306495	75308370	.	+	.	gene_id "XLOC_123898"; transcript_id "Novel_001688"; exon_number "3"; gene_type "
Dt_chr1	Cufflinks	exon	75302778	75302888	.	+	.	gene_id "XLOC_123898"; transcript_id "Novel_001689"; exon_number "1"; gene_type "
Dt_chr1	Cufflinks	exon	75306495	75308343	.	+	.	gene_id "XLOC_123898"; transcript_id "Novel_001689"; exon_number "2"; gene_type "
Dt_chr1	Cufflinks	exon	76184594	76184859	.	+	.	gene_id "XLOC_123963"; transcript_id "Novel_001690"; exon_number "1"; gene_type "
Dt_chr1	Cufflinks	exon	76185148	76185261	.	+	.	gene_id "XLOC_123963"; transcript_id "Novel_001690"; exon_number "2"; gene_type "
Dt_chr1	Cufflinks	exon	76185791	76185886	.	+	.	gene_id "XLOC_123963"; transcript_id "Novel_001690"; exon_number "3"; gene_type "
Dt_chr1	Cufflinks	exon	76186111	76186153	.	+	.	gene_id "XLOC_123963"; transcript_id "Novel_001690"; exon_number "4"; gene_type "

Dt_chr1	Cufflinks	exon	76186239	76188974	.	+	.	gene_id "XLOC_123963"; transcript_id "Novel_001690"; exon_number "5"; gene_type "
Dt_chr1	Cufflinks	exon	76184622	76184859	.	+	.	gene_id "XLOC_123963"; transcript_id "Novel_001691"; exon_number "1"; gene_type "
Dt_chr1	Cufflinks	exon	76185148	76185261	.	+	.	gene_id "XLOC_123963"; transcript_id "Novel_001691"; exon_number "2"; gene_type "
Dt_chr1	Cufflinks	exon	76185791	76185886	.	+	.	gene_id "XLOC_123963"; transcript_id "Novel_001691"; exon_number "3"; gene_type "
Dt_chr1	Cufflinks	exon	76186111	76186153	.	+	.	gene_id "XLOC_123963"; transcript_id "Novel_001691"; exon_number "4"; gene_type "
Dt_chr1	Cufflinks	exon	76186239	76186550	.	+	.	gene_id "XLOC_123963"; transcript_id "Novel_001691"; exon_number "5"; gene_type "
Dt_chr1	Cufflinks	exon	76188206	76188304	.	+	.	gene_id "XLOC_123963"; transcript_id "Novel_001691"; exon_number "6"; gene_type "
Dt_chr1	Cufflinks	exon	76188426	76188973	.	+	.	gene_id "XLOC_123963"; transcript_id "Novel_001691"; exon_number "7"; gene_type "
Dt_chr1	Cufflinks	exon	76184626	76184859	.	+	.	gene_id "XLOC_123963"; transcript_id "Novel_001692"; exon_number "1"; gene_type "
Dt_chr1	Cufflinks	exon	76185148	76185261	.	+	.	gene_id "XLOC_123963"; transcript_id "Novel_001692"; exon_number "2"; gene_type "
Dt_chr1	Cufflinks	exon	76185791	76185886	.	+	.	gene_id "XLOC_123963"; transcript_id "Novel_001692"; exon_number "3"; gene_type "
Dt_chr1	Cufflinks	exon	76186111	76186153	.	+	.	gene_id "XLOC_123963"; transcript_id "Novel_001692"; exon_number "4"; gene_type "
Dt_chr1	Cufflinks	exon	76186239	76186394	.	+	.	gene_id "XLOC_123963"; transcript_id "Novel_001692"; exon_number "5"; gene_type "
Dt_chr1	Cufflinks	exon	76186470	76186550	.	+	.	gene_id "XLOC_123963"; transcript_id "Novel_001692"; exon_number "6"; gene_type "
Dt_chr1	Cufflinks	exon	76188206	76188304	.	+	.	gene_id "XLOC_123963"; transcript_id "Novel_001692"; exon_number "7"; gene_type "
Dt_chr1	Cufflinks	exon	76188426	76188976	.	+	.	gene_id "XLOC_123963"; transcript_id "Novel_001692"; exon_number "8"; gene_type "
Dt_chr1	Cufflinks	exon	76422371	76422594	.	+	.	gene_id "XLOC_123979"; transcript_id "Novel_001693"; exon_number "1"; gene_type "
Dt_chr1	Cufflinks	exon	76621827	76622060	.	+	.	gene_id "XLOC_124005"; transcript_id "Novel_001694"; exon_number "1"; gene_type "
Dt_chr1	Cufflinks	exon	76625702	76625968	.	+	.	gene_id "XLOC_124005"; transcript_id "Novel_001694"; exon_number "2"; gene_type "
Dt_chr1	Cufflinks	exon	76626063	76626431	.	+	.	gene_id "XLOC_124005"; transcript_id "Novel_001694"; exon_number "3"; gene_type "
Dt_chr1	Cufflinks	exon	80025150	80025351	.	+	.	gene_id "XLOC_124263"; transcript_id "Novel_001695"; exon_number "1"; gene_type "
Dt_chr1	Cufflinks	exon	80027151	80027962	.	+	.	gene_id "XLOC_124263"; transcript_id "Novel_001695"; exon_number "2"; gene_type "
Dt_chr1	Cufflinks	exon	85466680	85471562	.	+	.	gene_id "XLOC_124725"; transcript_id "Novel_001696"; exon_number "1"; gene_type "
Dt_chr1	Cufflinks	exon	85471690	85472128	.	+	.	gene_id "XLOC_124725"; transcript_id "Novel_001696"; exon_number "2"; gene_type "
Dt_chr1	Cufflinks	exon	85472228	85472542	.	+	.	gene_id "XLOC_124725"; transcript_id "Novel_001696"; exon_number "3"; gene_type "
Dt_chr1	Cufflinks	exon	85466688	85467576	.	+	.	gene_id "XLOC_124725"; transcript_id "Novel_001697"; exon_number "1"; gene_type "
Dt_chr1	Cufflinks	exon	85470857	85471562	.	+	.	gene_id "XLOC_124725"; transcript_id "Novel_001697"; exon_number "2"; gene_type "
Dt_chr1	Cufflinks	exon	85471690	85472128	.	+	.	gene_id "XLOC_124725"; transcript_id "Novel_001697"; exon_number "3"; gene_type "
Dt_chr1	Cufflinks	exon	85472228	85472459	.	+	.	gene_id "XLOC_124725"; transcript_id "Novel_001697"; exon_number "4"; gene_type "
Dt_chr1	Cufflinks	exon	90325519	90326843	.	+	.	gene_id "XLOC_124932"; transcript_id "Novel_001698"; exon_number "1"; gene_type "
Dt_chr1	Cufflinks	exon	90338102	90338166	.	+	.	gene_id "XLOC_124932"; transcript_id "Novel_001698"; exon_number "2"; gene_type "
Dt_chr1	Cufflinks	exon	90338561	90338640	.	+	.	gene_id "XLOC_124932"; transcript_id "Novel_001698"; exon_number "3"; gene_type "
Dt_chr1	Cufflinks	exon	90339551	90339648	.	+	.	gene_id "XLOC_124932"; transcript_id "Novel_001698"; exon_number "4"; gene_type "
Dt_chr1	Cufflinks	exon	90339781	90339999	.	+	.	gene_id "XLOC_124932"; transcript_id "Novel_001698"; exon_number "5"; gene_type "
Dt_chr1	Cufflinks	exon	1999653	2000955	.	-	.	gene_id "XLOC_125315"; transcript_id "Novel_001699"; exon_number "1"; gene_type "

Dt_chr1	Cufflinks	exon	2002665	2002778	.	-	.	gene_id "XLOC_125315"; transcript_id "Novel_001699"; exon_number "2"; gene_type "
Dt_chr1	Cufflinks	exon	2002861	2003151	.	-	.	gene_id "XLOC_125315"; transcript_id "Novel_001699"; exon_number "3"; gene_type "
Dt_chr1	Cufflinks	exon	2004138	2004445	.	-	.	gene_id "XLOC_125315"; transcript_id "Novel_001699"; exon_number "4"; gene_type "
Dt_chr1	Cufflinks	exon	2004758	2005643	.	-	.	gene_id "XLOC_125315"; transcript_id "Novel_001699"; exon_number "5"; gene_type "
Dt_chr1	Cufflinks	exon	2000112	2002778	.	-	.	gene_id "XLOC_125315"; transcript_id "Novel_001700"; exon_number "1"; gene_type "
Dt_chr1	Cufflinks	exon	2002861	2005675	.	-	.	gene_id "XLOC_125315"; transcript_id "Novel_001700"; exon_number "2"; gene_type "
Dt_chr1	Cufflinks	exon	4667806	4668244	.	-	.	gene_id "XLOC_125466"; transcript_id "Novel_001701"; exon_number "1"; gene_type "
Dt_chr1	Cufflinks	exon	4669677	4670131	.	-	.	gene_id "XLOC_125466"; transcript_id "Novel_001701"; exon_number "2"; gene_type "
Dt_chr1	Cufflinks	exon	4670271	4670507	.	-	.	gene_id "XLOC_125466"; transcript_id "Novel_001701"; exon_number "3"; gene_type "
Dt_chr1	Cufflinks	exon	4667815	4670131	.	-	.	gene_id "XLOC_125466"; transcript_id "Novel_001702"; exon_number "1"; gene_type "
Dt_chr1	Cufflinks	exon	4670271	4670499	.	-	.	gene_id "XLOC_125466"; transcript_id "Novel_001702"; exon_number "2"; gene_type "
Dt_chr1	Cufflinks	exon	5163338	5163856	.	-	.	gene_id "XLOC_125498"; transcript_id "Novel_001703"; exon_number "1"; gene_type "
Dt_chr1	Cufflinks	exon	5164004	5164274	.	-	.	gene_id "XLOC_125498"; transcript_id "Novel_001703"; exon_number "2"; gene_type "
Dt_chr1	Cufflinks	exon	24524685	24527708	.	-	.	gene_id "XLOC_126159"; transcript_id "Novel_001704"; exon_number "1"; gene_type "
Dt_chr1	Cufflinks	exon	24527851	24528211	.	-	.	gene_id "XLOC_126159"; transcript_id "Novel_001704"; exon_number "2"; gene_type "
Dt_chr1	Cufflinks	exon	24528294	24529535	.	-	.	gene_id "XLOC_126159"; transcript_id "Novel_001704"; exon_number "3"; gene_type "
Dt_chr1	Cufflinks	exon	24524959	24528211	.	-	.	gene_id "XLOC_126159"; transcript_id "Novel_001705"; exon_number "1"; gene_type "
Dt_chr1	Cufflinks	exon	24528294	24528375	.	-	.	gene_id "XLOC_126159"; transcript_id "Novel_001705"; exon_number "2"; gene_type "
Dt_chr1	Cufflinks	exon	24528474	24529295	.	-	.	gene_id "XLOC_126159"; transcript_id "Novel_001705"; exon_number "3"; gene_type "
Dt_chr1	Cufflinks	exon	24524971	24527708	.	-	.	gene_id "XLOC_126159"; transcript_id "Novel_001706"; exon_number "1"; gene_type "
Dt_chr1	Cufflinks	exon	24527851	24528211	.	-	.	gene_id "XLOC_126159"; transcript_id "Novel_001706"; exon_number "2"; gene_type "
Dt_chr1	Cufflinks	exon	24528294	24528375	.	-	.	gene_id "XLOC_126159"; transcript_id "Novel_001706"; exon_number "3"; gene_type "
Dt_chr1	Cufflinks	exon	24528474	24529236	.	-	.	gene_id "XLOC_126159"; transcript_id "Novel_001706"; exon_number "4"; gene_type "
Dt_chr1	Cufflinks	exon	27313942	27315161	.	-	.	gene_id "XLOC_126338"; transcript_id "Novel_001707"; exon_number "1"; gene_type "
Dt_chr1	Cufflinks	exon	27315258	27315294	.	-	.	gene_id "XLOC_126338"; transcript_id "Novel_001707"; exon_number "2"; gene_type "
Dt_chr1	Cufflinks	exon	27315458	27315575	.	-	.	gene_id "XLOC_126338"; transcript_id "Novel_001707"; exon_number "3"; gene_type "
Dt_chr1	Cufflinks	exon	27315906	27316035	.	-	.	gene_id "XLOC_126338"; transcript_id "Novel_001707"; exon_number "4"; gene_type "
Dt_chr1	Cufflinks	exon	27316132	27316357	.	-	.	gene_id "XLOC_126338"; transcript_id "Novel_001707"; exon_number "5"; gene_type "
Dt_chr1	Cufflinks	exon	27976260	27976666	.	-	.	gene_id "XLOC_126402"; transcript_id "Novel_001708"; exon_number "1"; gene_type "
Dt_chr1	Cufflinks	exon	27977903	27978067	.	-	.	gene_id "XLOC_126402"; transcript_id "Novel_001708"; exon_number "2"; gene_type "
Dt_chr1	Cufflinks	exon	33770113	33771507	.	-	.	gene_id "XLOC_126904"; transcript_id "Novel_001709"; exon_number "1"; gene_type "
Dt_chr1	Cufflinks	exon	33771797	33771915	.	-	.	gene_id "XLOC_126904"; transcript_id "Novel_001709"; exon_number "2"; gene_type "
Dt_chr1	Cufflinks	exon	36012196	36012290	.	-	.	gene_id "XLOC_127067"; transcript_id "Novel_001710"; exon_number "1"; gene_type "
Dt_chr1	Cufflinks	exon	36012366	36012422	.	-	.	gene_id "XLOC_127067"; transcript_id "Novel_001710"; exon_number "2"; gene_type "
Dt_chr1	Cufflinks	exon	36012551	36012613	.	-	.	gene_id "XLOC_127067"; transcript_id "Novel_001710"; exon_number "3"; gene_type "

Dt_chr1	Cufflinks	exon	36012720	36012790	.	-	.	gene_id "XLOC_127067"; transcript_id "Novel_001710"; exon_number "4"; gene_type "
Dt_chr1	Cufflinks	exon	36012878	36012986	.	-	.	gene_id "XLOC_127067"; transcript_id "Novel_001710"; exon_number "5"; gene_type "
Dt_chr1	Cufflinks	exon	36013694	36013889	.	-	.	gene_id "XLOC_127067"; transcript_id "Novel_001710"; exon_number "6"; gene_type "
Dt_chr1	Cufflinks	exon	36015462	36015609	.	-	.	gene_id "XLOC_127067"; transcript_id "Novel_001710"; exon_number "7"; gene_type "
Dt_chr1	Cufflinks	exon	37678781	37679796	.	-	.	gene_id "XLOC_127275"; transcript_id "Novel_001711"; exon_number "1"; gene_type "
Dt_chr1	Cufflinks	exon	37679882	37684284	.	-	.	gene_id "XLOC_127275"; transcript_id "Novel_001711"; exon_number "2"; gene_type "
Dt_chr1	Cufflinks	exon	40348346	40349751	.	-	.	gene_id "XLOC_127732"; transcript_id "Novel_001712"; exon_number "1"; gene_type "
Dt_chr1	Cufflinks	exon	40350037	40350341	.	-	.	gene_id "XLOC_127732"; transcript_id "Novel_001712"; exon_number "2"; gene_type "
Dt_chr1	Cufflinks	exon	40693811	40693949	.	-	.	gene_id "XLOC_127770"; transcript_id "Novel_001713"; exon_number "1"; gene_type "
Dt_chr1	Cufflinks	exon	40694849	40694959	.	-	.	gene_id "XLOC_127770"; transcript_id "Novel_001713"; exon_number "2"; gene_type "
Dt_chr1	Cufflinks	exon	40695864	40695969	.	-	.	gene_id "XLOC_127770"; transcript_id "Novel_001713"; exon_number "3"; gene_type "
Dt_chr1	Cufflinks	exon	40697507	40697877	.	-	.	gene_id "XLOC_127770"; transcript_id "Novel_001713"; exon_number "4"; gene_type "
Dt_chr1	Cufflinks	exon	40698088	40698281	.	-	.	gene_id "XLOC_127770"; transcript_id "Novel_001713"; exon_number "5"; gene_type "
Dt_chr1	Cufflinks	exon	40693812	40693949	.	-	.	gene_id "XLOC_127770"; transcript_id "Novel_001714"; exon_number "1"; gene_type "
Dt_chr1	Cufflinks	exon	40694849	40694959	.	-	.	gene_id "XLOC_127770"; transcript_id "Novel_001714"; exon_number "2"; gene_type "
Dt_chr1	Cufflinks	exon	40695864	40695969	.	-	.	gene_id "XLOC_127770"; transcript_id "Novel_001714"; exon_number "3"; gene_type "
Dt_chr1	Cufflinks	exon	40697507	40697965	.	-	.	gene_id "XLOC_127770"; transcript_id "Novel_001714"; exon_number "4"; gene_type "
Dt_chr1	Cufflinks	exon	40698088	40698324	.	-	.	gene_id "XLOC_127770"; transcript_id "Novel_001714"; exon_number "5"; gene_type "
Dt_chr1	Cufflinks	exon	40693812	40693949	.	-	.	gene_id "XLOC_127770"; transcript_id "Novel_001715"; exon_number "1"; gene_type "
Dt_chr1	Cufflinks	exon	40694849	40694959	.	-	.	gene_id "XLOC_127770"; transcript_id "Novel_001715"; exon_number "2"; gene_type "
Dt_chr1	Cufflinks	exon	40695864	40695969	.	-	.	gene_id "XLOC_127770"; transcript_id "Novel_001715"; exon_number "3"; gene_type "
Dt_chr1	Cufflinks	exon	40697507	40697543	.	-	.	gene_id "XLOC_127770"; transcript_id "Novel_001715"; exon_number "4"; gene_type "
Dt_chr1	Cufflinks	exon	40697619	40697877	.	-	.	gene_id "XLOC_127770"; transcript_id "Novel_001715"; exon_number "5"; gene_type "
Dt_chr1	Cufflinks	exon	40698088	40698324	.	-	.	gene_id "XLOC_127770"; transcript_id "Novel_001715"; exon_number "6"; gene_type "
Dt_chr1	Cufflinks	exon	40697159	40697965	.	-	.	gene_id "XLOC_127770"; transcript_id "Novel_001716"; exon_number "1"; gene_type "
Dt_chr1	Cufflinks	exon	40698088	40698237	.	-	.	gene_id "XLOC_127770"; transcript_id "Novel_001716"; exon_number "2"; gene_type "
Dt_chr1	Cufflinks	exon	40714118	40714521	.	-	.	gene_id "XLOC_127781"; transcript_id "Novel_001717"; exon_number "1"; gene_type "
Dt_chr1	Cufflinks	exon	40714607	40714675	.	-	.	gene_id "XLOC_127781"; transcript_id "Novel_001717"; exon_number "2"; gene_type "
Dt_chr1	Cufflinks	exon	40715780	40715906	.	-	.	gene_id "XLOC_127781"; transcript_id "Novel_001717"; exon_number "3"; gene_type "
Dt_chr1	Cufflinks	exon	40716011	40716118	.	-	.	gene_id "XLOC_127781"; transcript_id "Novel_001717"; exon_number "4"; gene_type "
Dt_chr1	Cufflinks	exon	40716205	40716348	.	-	.	gene_id "XLOC_127781"; transcript_id "Novel_001717"; exon_number "5"; gene_type "
Dt_chr1	Cufflinks	exon	40714166	40714521	.	-	.	gene_id "XLOC_127781"; transcript_id "Novel_001718"; exon_number "1"; gene_type "
Dt_chr1	Cufflinks	exon	40714607	40715769	.	-	.	gene_id "XLOC_127781"; transcript_id "Novel_001718"; exon_number "2"; gene_type "
Dt_chr1	Cufflinks	exon	40818530	40818791	.	-	.	gene_id "XLOC_127799"; transcript_id "Novel_001719"; exon_number "1"; gene_type "
Dt_chr1	Cufflinks	exon	40818881	40819073	.	-	.	gene_id "XLOC_127799"; transcript_id "Novel_001719"; exon_number "2"; gene_type "

Dt_chr1	Cufflinks	exon	40821843	40822330	.	-	.	gene_id "XLOC_127799"; transcript_id "Novel_001719"; exon_number "3"; gene_type "
Dt_chr1	Cufflinks	exon	40851844	40852846	.	-	.	gene_id "XLOC_127804"; transcript_id "Novel_001720"; exon_number "1"; gene_type "
Dt_chr1	Cufflinks	exon	40852945	40853399	.	-	.	gene_id "XLOC_127804"; transcript_id "Novel_001720"; exon_number "2"; gene_type "
Dt_chr1	Cufflinks	exon	40853501	40853700	.	-	.	gene_id "XLOC_127805"; transcript_id "Novel_001721"; exon_number "1"; gene_type "
Dt_chr1	Cufflinks	exon	40853791	40853883	.	-	.	gene_id "XLOC_127805"; transcript_id "Novel_001721"; exon_number "2"; gene_type "
Dt_chr1	Cufflinks	exon	40853983	40854267	.	-	.	gene_id "XLOC_127805"; transcript_id "Novel_001721"; exon_number "3"; gene_type "
Dt_chr1	Cufflinks	exon	40853735	40853883	.	-	.	gene_id "XLOC_127805"; transcript_id "Novel_001722"; exon_number "1"; gene_type "
Dt_chr1	Cufflinks	exon	40853983	40854233	.	-	.	gene_id "XLOC_127805"; transcript_id "Novel_001722"; exon_number "2"; gene_type "
Dt_chr1	Cufflinks	exon	40936504	40937444	.	-	.	gene_id "XLOC_127823"; transcript_id "Novel_001723"; exon_number "1"; gene_type "
Dt_chr1	Cufflinks	exon	41014132	41014711	.	-	.	gene_id "XLOC_127835"; transcript_id "Novel_001724"; exon_number "1"; gene_type "
Dt_chr1	Cufflinks	exon	41015167	41015376	.	-	.	gene_id "XLOC_127835"; transcript_id "Novel_001724"; exon_number "2"; gene_type "
Dt_chr1	Cufflinks	exon	41015460	41016094	.	-	.	gene_id "XLOC_127835"; transcript_id "Novel_001724"; exon_number "3"; gene_type "
Dt_chr1	Cufflinks	exon	41014615	41014711	.	-	.	gene_id "XLOC_127835"; transcript_id "Novel_001725"; exon_number "1"; gene_type "
Dt_chr1	Cufflinks	exon	41015167	41016137	.	-	.	gene_id "XLOC_127835"; transcript_id "Novel_001725"; exon_number "2"; gene_type "
Dt_chr1	Cufflinks	exon	41034381	41035071	.	-	.	gene_id "XLOC_127838"; transcript_id "Novel_001726"; exon_number "1"; gene_type "
Dt_chr1	Cufflinks	exon	41035910	41036232	.	-	.	gene_id "XLOC_127838"; transcript_id "Novel_001726"; exon_number "2"; gene_type "
Dt_chr1	Cufflinks	exon	41037537	41037742	.	-	.	gene_id "XLOC_127838"; transcript_id "Novel_001726"; exon_number "3"; gene_type "
Dt_chr1	Cufflinks	exon	41137540	41137642	.	-	.	gene_id "XLOC_127862"; transcript_id "Novel_001727"; exon_number "1"; gene_type "
Dt_chr1	Cufflinks	exon	41137750	41137885	.	-	.	gene_id "XLOC_127862"; transcript_id "Novel_001727"; exon_number "2"; gene_type "
Dt_chr1	Cufflinks	exon	41383903	41384706	.	-	.	gene_id "XLOC_127906"; transcript_id "Novel_001728"; exon_number "1"; gene_type "
Dt_chr1	Cufflinks	exon	41384795	41384926	.	-	.	gene_id "XLOC_127906"; transcript_id "Novel_001728"; exon_number "2"; gene_type "
Dt_chr1	Cufflinks	exon	41385508	41385529	.	-	.	gene_id "XLOC_127906"; transcript_id "Novel_001728"; exon_number "3"; gene_type "
Dt_chr1	Cufflinks	exon	41391584	41391819	.	-	.	gene_id "XLOC_127908"; transcript_id "Novel_001729"; exon_number "1"; gene_type "
Dt_chr1	Cufflinks	exon	41391959	41392057	.	-	.	gene_id "XLOC_127908"; transcript_id "Novel_001729"; exon_number "2"; gene_type "
Dt_chr1	Cufflinks	exon	41392506	41392603	.	-	.	gene_id "XLOC_127908"; transcript_id "Novel_001729"; exon_number "3"; gene_type "
Dt_chr1	Cufflinks	exon	41420402	41420437	.	-	.	gene_id "XLOC_127916"; transcript_id "Novel_001730"; exon_number "1"; gene_type "
Dt_chr1	Cufflinks	exon	41420823	41421560	.	-	.	gene_id "XLOC_127916"; transcript_id "Novel_001730"; exon_number "2"; gene_type "
Dt_chr1	Cufflinks	exon	41421665	41421877	.	-	.	gene_id "XLOC_127916"; transcript_id "Novel_001730"; exon_number "3"; gene_type "
Dt_chr1	Cufflinks	exon	41420405	41420437	.	-	.	gene_id "XLOC_127916"; transcript_id "Novel_001731"; exon_number "1"; gene_type "
Dt_chr1	Cufflinks	exon	41420823	41421428	.	-	.	gene_id "XLOC_127916"; transcript_id "Novel_001731"; exon_number "2"; gene_type "
Dt_chr1	Cufflinks	exon	41421493	41421560	.	-	.	gene_id "XLOC_127916"; transcript_id "Novel_001731"; exon_number "3"; gene_type "
Dt_chr1	Cufflinks	exon	41421665	41421940	.	-	.	gene_id "XLOC_127916"; transcript_id "Novel_001731"; exon_number "4"; gene_type "
Dt_chr1	Cufflinks	exon	41420634	41421560	.	-	.	gene_id "XLOC_127916"; transcript_id "Novel_001732"; exon_number "1"; gene_type "
Dt_chr1	Cufflinks	exon	41421665	41421940	.	-	.	gene_id "XLOC_127916"; transcript_id "Novel_001732"; exon_number "2"; gene_type "
Dt_chr1	Cufflinks	exon	41420634	41421419	.	-	.	gene_id "XLOC_127916"; transcript_id "Novel_001733"; exon_number "1"; gene_type "

Dt_chr1	Cufflinks	exon	41421493	41421560	.	-	.	gene_id "XLOC_127916"; transcript_id "Novel_001733"; exon_number "2"; gene_type "
Dt_chr1	Cufflinks	exon	41421665	41421940	.	-	.	gene_id "XLOC_127916"; transcript_id "Novel_001733"; exon_number "3"; gene_type "
Dt_chr1	Cufflinks	exon	41461336	41462507	.	-	.	gene_id "XLOC_127920"; transcript_id "Novel_001734"; exon_number "1"; gene_type "
Dt_chr1	Cufflinks	exon	41462601	41462826	.	-	.	gene_id "XLOC_127920"; transcript_id "Novel_001734"; exon_number "2"; gene_type "
Dt_chr1	Cufflinks	exon	41464367	41466121	.	-	.	gene_id "XLOC_127920"; transcript_id "Novel_001734"; exon_number "3"; gene_type "
Dt_chr1	Cufflinks	exon	41466371	41468903	.	-	.	gene_id "XLOC_127920"; transcript_id "Novel_001734"; exon_number "4"; gene_type "
Dt_chr1	Cufflinks	exon	41469053	41469274	.	-	.	gene_id "XLOC_127920"; transcript_id "Novel_001734"; exon_number "5"; gene_type "
Dt_chr1	Cufflinks	exon	41461386	41462503	.	-	.	gene_id "XLOC_127920"; transcript_id "Novel_001735"; exon_number "1"; gene_type "
Dt_chr1	Cufflinks	exon	41462601	41462826	.	-	.	gene_id "XLOC_127920"; transcript_id "Novel_001735"; exon_number "2"; gene_type "
Dt_chr1	Cufflinks	exon	41464367	41466121	.	-	.	gene_id "XLOC_127920"; transcript_id "Novel_001735"; exon_number "3"; gene_type "
Dt_chr1	Cufflinks	exon	41466371	41467444	.	-	.	gene_id "XLOC_127920"; transcript_id "Novel_001735"; exon_number "4"; gene_type "
Dt_chr1	Cufflinks	exon	41807568	41810412	.	-	.	gene_id "XLOC_127968"; transcript_id "Novel_001736"; exon_number "1"; gene_type "
Dt_chr1	Cufflinks	exon	41810570	41810951	.	-	.	gene_id "XLOC_127968"; transcript_id "Novel_001736"; exon_number "2"; gene_type "
Dt_chr1	Cufflinks	exon	41807875	41810429	.	-	.	gene_id "XLOC_127968"; transcript_id "Novel_001737"; exon_number "1"; gene_type "
Dt_chr1	Cufflinks	exon	41810570	41810963	.	-	.	gene_id "XLOC_127968"; transcript_id "Novel_001737"; exon_number "2"; gene_type "
Dt_chr1	Cufflinks	exon	41966430	41967200	.	-	.	gene_id "XLOC_127990"; transcript_id "Novel_001738"; exon_number "1"; gene_type "
Dt_chr1	Cufflinks	exon	41967281	41967621	.	-	.	gene_id "XLOC_127990"; transcript_id "Novel_001738"; exon_number "2"; gene_type "
Dt_chr1	Cufflinks	exon	41967702	41968249	.	-	.	gene_id "XLOC_127990"; transcript_id "Novel_001738"; exon_number "3"; gene_type "
Dt_chr1	Cufflinks	exon	41968359	41968576	.	-	.	gene_id "XLOC_127990"; transcript_id "Novel_001738"; exon_number "4"; gene_type "
Dt_chr1	Cufflinks	exon	41969688	41969790	.	-	.	gene_id "XLOC_127990"; transcript_id "Novel_001738"; exon_number "5"; gene_type "
Dt_chr1	Cufflinks	exon	41969896	41970175	.	-	.	gene_id "XLOC_127990"; transcript_id "Novel_001738"; exon_number "6"; gene_type "
Dt_chr1	Cufflinks	exon	41966476	41967200	.	-	.	gene_id "XLOC_127990"; transcript_id "Novel_001739"; exon_number "1"; gene_type "
Dt_chr1	Cufflinks	exon	41967281	41967621	.	-	.	gene_id "XLOC_127990"; transcript_id "Novel_001739"; exon_number "2"; gene_type "
Dt_chr1	Cufflinks	exon	41967702	41968576	.	-	.	gene_id "XLOC_127990"; transcript_id "Novel_001739"; exon_number "3"; gene_type "
Dt_chr1	Cufflinks	exon	41969688	41969790	.	-	.	gene_id "XLOC_127990"; transcript_id "Novel_001739"; exon_number "4"; gene_type "
Dt_chr1	Cufflinks	exon	41969896	41970106	.	-	.	gene_id "XLOC_127990"; transcript_id "Novel_001739"; exon_number "5"; gene_type "
Dt_chr1	Cufflinks	exon	41966534	41967200	.	-	.	gene_id "XLOC_127990"; transcript_id "Novel_001740"; exon_number "1"; gene_type "
Dt_chr1	Cufflinks	exon	41967281	41967621	.	-	.	gene_id "XLOC_127990"; transcript_id "Novel_001740"; exon_number "2"; gene_type "
Dt_chr1	Cufflinks	exon	41967702	41968249	.	-	.	gene_id "XLOC_127990"; transcript_id "Novel_001740"; exon_number "3"; gene_type "
Dt_chr1	Cufflinks	exon	41968359	41968576	.	-	.	gene_id "XLOC_127990"; transcript_id "Novel_001740"; exon_number "4"; gene_type "
Dt_chr1	Cufflinks	exon	41969688	41969995	.	-	.	gene_id "XLOC_127990"; transcript_id "Novel_001740"; exon_number "5"; gene_type "
Dt_chr1	Cufflinks	exon	42116823	42117725	.	-	.	gene_id "XLOC_128025"; transcript_id "Novel_001741"; exon_number "1"; gene_type "
Dt_chr1	Cufflinks	exon	42117823	42118229	.	-	.	gene_id "XLOC_128025"; transcript_id "Novel_001741"; exon_number "2"; gene_type "
Dt_chr1	Cufflinks	exon	42118322	42118634	.	-	.	gene_id "XLOC_128025"; transcript_id "Novel_001741"; exon_number "3"; gene_type "
Dt_chr1	Cufflinks	exon	42118722	42120185	.	-	.	gene_id "XLOC_128025"; transcript_id "Novel_001741"; exon_number "4"; gene_type "

Dt_chr1	Cufflinks	exon	42116823	42117725	.	-	.	gene_id "XLOC_128025"; transcript_id "Novel_001742"; exon_number "1"; gene_type "
Dt_chr1	Cufflinks	exon	42117823	42118229	.	-	.	gene_id "XLOC_128025"; transcript_id "Novel_001742"; exon_number "2"; gene_type "
Dt_chr1	Cufflinks	exon	42118322	42118634	.	-	.	gene_id "XLOC_128025"; transcript_id "Novel_001742"; exon_number "3"; gene_type "
Dt_chr1	Cufflinks	exon	42118722	42120061	.	-	.	gene_id "XLOC_128025"; transcript_id "Novel_001742"; exon_number "4"; gene_type "
Dt_chr1	Cufflinks	exon	42122140	42122595	.	-	.	gene_id "XLOC_128025"; transcript_id "Novel_001742"; exon_number "5"; gene_type "
Dt_chr1	Cufflinks	exon	42117020	42117725	.	-	.	gene_id "XLOC_128025"; transcript_id "Novel_001743"; exon_number "1"; gene_type "
Dt_chr1	Cufflinks	exon	42117823	42120061	.	-	.	gene_id "XLOC_128025"; transcript_id "Novel_001743"; exon_number "2"; gene_type "
Dt_chr1	Cufflinks	exon	42122140	42122826	.	-	.	gene_id "XLOC_128025"; transcript_id "Novel_001743"; exon_number "3"; gene_type "
Dt_chr1	Cufflinks	exon	42117021	42117725	.	-	.	gene_id "XLOC_128025"; transcript_id "Novel_001744"; exon_number "1"; gene_type "
Dt_chr1	Cufflinks	exon	42117823	42118229	.	-	.	gene_id "XLOC_128025"; transcript_id "Novel_001744"; exon_number "2"; gene_type "
Dt_chr1	Cufflinks	exon	42118322	42120061	.	-	.	gene_id "XLOC_128025"; transcript_id "Novel_001744"; exon_number "3"; gene_type "
Dt_chr1	Cufflinks	exon	42122140	42122647	.	-	.	gene_id "XLOC_128025"; transcript_id "Novel_001744"; exon_number "4"; gene_type "
Dt_chr1	Cufflinks	exon	42170532	42171136	.	-	.	gene_id "XLOC_128038"; transcript_id "Novel_001745"; exon_number "1"; gene_type "
Dt_chr1	Cufflinks	exon	42171213	42171378	.	-	.	gene_id "XLOC_128038"; transcript_id "Novel_001745"; exon_number "2"; gene_type "
Dt_chr1	Cufflinks	exon	42171478	42171657	.	-	.	gene_id "XLOC_128038"; transcript_id "Novel_001745"; exon_number "3"; gene_type "
Dt_chr1	Cufflinks	exon	42171793	42172127	.	-	.	gene_id "XLOC_128038"; transcript_id "Novel_001745"; exon_number "4"; gene_type "
Dt_chr1	Cufflinks	exon	42175311	42175779	.	-	.	gene_id "XLOC_128039"; transcript_id "Novel_001746"; exon_number "1"; gene_type "
Dt_chr1	Cufflinks	exon	42176489	42176563	.	-	.	gene_id "XLOC_128039"; transcript_id "Novel_001746"; exon_number "2"; gene_type "
Dt_chr1	Cufflinks	exon	42176641	42176703	.	-	.	gene_id "XLOC_128039"; transcript_id "Novel_001746"; exon_number "3"; gene_type "
Dt_chr1	Cufflinks	exon	42176952	42177176	.	-	.	gene_id "XLOC_128039"; transcript_id "Novel_001746"; exon_number "4"; gene_type "
Dt_chr1	Cufflinks	exon	42177479	42177620	.	-	.	gene_id "XLOC_128039"; transcript_id "Novel_001746"; exon_number "5"; gene_type "
Dt_chr1	Cufflinks	exon	42177702	42177863	.	-	.	gene_id "XLOC_128039"; transcript_id "Novel_001746"; exon_number "6"; gene_type "
Dt_chr1	Cufflinks	exon	42178071	42178243	.	-	.	gene_id "XLOC_128039"; transcript_id "Novel_001746"; exon_number "7"; gene_type "
Dt_chr1	Cufflinks	exon	42178485	42179174	.	-	.	gene_id "XLOC_128039"; transcript_id "Novel_001746"; exon_number "8"; gene_type "
Dt_chr1	Cufflinks	exon	42225506	42226118	.	-	.	gene_id "XLOC_128048"; transcript_id "Novel_001747"; exon_number "1"; gene_type "
Dt_chr1	Cufflinks	exon	42226197	42226759	.	-	.	gene_id "XLOC_128048"; transcript_id "Novel_001747"; exon_number "2"; gene_type "
Dt_chr1	Cufflinks	exon	42293146	42293795	.	-	.	gene_id "XLOC_128061"; transcript_id "Novel_001748"; exon_number "1"; gene_type "
Dt_chr1	Cufflinks	exon	42294203	42294474	.	-	.	gene_id "XLOC_128061"; transcript_id "Novel_001748"; exon_number "2"; gene_type "
Dt_chr1	Cufflinks	exon	42294561	42295514	.	-	.	gene_id "XLOC_128061"; transcript_id "Novel_001748"; exon_number "3"; gene_type "
Dt_chr1	Cufflinks	exon	42295959	42296262	.	-	.	gene_id "XLOC_128061"; transcript_id "Novel_001748"; exon_number "4"; gene_type "
Dt_chr1	Cufflinks	exon	42296720	42297603	.	-	.	gene_id "XLOC_128061"; transcript_id "Novel_001748"; exon_number "5"; gene_type "
Dt_chr1	Cufflinks	exon	42297727	42297939	.	-	.	gene_id "XLOC_128061"; transcript_id "Novel_001748"; exon_number "6"; gene_type "
Dt_chr1	Cufflinks	exon	42298148	42298644	.	-	.	gene_id "XLOC_128061"; transcript_id "Novel_001748"; exon_number "7"; gene_type "
Dt_chr1	Cufflinks	exon	42298877	42299217	.	-	.	gene_id "XLOC_128061"; transcript_id "Novel_001748"; exon_number "8"; gene_type "
Dt_chr1	Cufflinks	exon	42302952	42303471	.	-	.	gene_id "XLOC_128064"; transcript_id "Novel_001749"; exon_number "1"; gene_type "

Dt_chr1	Cufflinks	exon	42303691	42303807	.	-	.	gene_id "XLOC_128064"; transcript_id "Novel_001749"; exon_number "2"; gene_type "
Dt_chr1	Cufflinks	exon	42304171	42304443	.	-	.	gene_id "XLOC_128064"; transcript_id "Novel_001749"; exon_number "3"; gene_type "
Dt_chr1	Cufflinks	exon	42304587	42304670	.	-	.	gene_id "XLOC_128064"; transcript_id "Novel_001749"; exon_number "4"; gene_type "
Dt_chr1	Cufflinks	exon	42323669	42324358	.	-	.	gene_id "XLOC_128066"; transcript_id "Novel_001750"; exon_number "1"; gene_type "
Dt_chr1	Cufflinks	exon	42324437	42324553	.	-	.	gene_id "XLOC_128066"; transcript_id "Novel_001750"; exon_number "2"; gene_type "
Dt_chr1	Cufflinks	exon	42324644	42324967	.	-	.	gene_id "XLOC_128066"; transcript_id "Novel_001750"; exon_number "3"; gene_type "
Dt_chr1	Cufflinks	exon	42325062	42325121	.	-	.	gene_id "XLOC_128066"; transcript_id "Novel_001750"; exon_number "4"; gene_type "
Dt_chr1	Cufflinks	exon	42325215	42325501	.	-	.	gene_id "XLOC_128066"; transcript_id "Novel_001750"; exon_number "5"; gene_type "
Dt_chr1	Cufflinks	exon	42397952	42399031	.	-	.	gene_id "XLOC_128081"; transcript_id "Novel_001751"; exon_number "1"; gene_type "
Dt_chr1	Cufflinks	exon	42399111	42399182	.	-	.	gene_id "XLOC_128081"; transcript_id "Novel_001751"; exon_number "2"; gene_type "
Dt_chr1	Cufflinks	exon	42399502	42399545	.	-	.	gene_id "XLOC_128081"; transcript_id "Novel_001751"; exon_number "3"; gene_type "
Dt_chr1	Cufflinks	exon	42399626	42399712	.	-	.	gene_id "XLOC_128081"; transcript_id "Novel_001751"; exon_number "4"; gene_type "
Dt_chr1	Cufflinks	exon	42399838	42399889	.	-	.	gene_id "XLOC_128081"; transcript_id "Novel_001751"; exon_number "5"; gene_type "
Dt_chr1	Cufflinks	exon	42400800	42400873	.	-	.	gene_id "XLOC_128081"; transcript_id "Novel_001751"; exon_number "6"; gene_type "
Dt_chr1	Cufflinks	exon	42401154	42401261	.	-	.	gene_id "XLOC_128081"; transcript_id "Novel_001751"; exon_number "7"; gene_type "
Dt_chr1	Cufflinks	exon	42401404	42402219	.	-	.	gene_id "XLOC_128081"; transcript_id "Novel_001751"; exon_number "8"; gene_type "
Dt_chr1	Cufflinks	exon	42402375	42402422	.	-	.	gene_id "XLOC_128081"; transcript_id "Novel_001751"; exon_number "9"; gene_type "
Dt_chr1	Cufflinks	exon	42402772	42402886	.	-	.	gene_id "XLOC_128081"; transcript_id "Novel_001751"; exon_number "10"; gene_type
Dt_chr1	Cufflinks	exon	42403201	42403382	.	-	.	gene_id "XLOC_128081"; transcript_id "Novel_001751"; exon_number "11"; gene_type
Dt_chr1	Cufflinks	exon	42403486	42403689	.	-	.	gene_id "XLOC_128081"; transcript_id "Novel_001751"; exon_number "12"; gene_type
Dt_chr1	Cufflinks	exon	42404826	42405191	.	-	.	gene_id "XLOC_128081"; transcript_id "Novel_001751"; exon_number "13"; gene_type
Dt_chr1	Cufflinks	exon	42398106	42399031	.	-	.	gene_id "XLOC_128081"; transcript_id "Novel_001752"; exon_number "1"; gene_type "
Dt_chr1	Cufflinks	exon	42399111	42399182	.	-	.	gene_id "XLOC_128081"; transcript_id "Novel_001752"; exon_number "2"; gene_type "
Dt_chr1	Cufflinks	exon	42399502	42399545	.	-	.	gene_id "XLOC_128081"; transcript_id "Novel_001752"; exon_number "3"; gene_type "
Dt_chr1	Cufflinks	exon	42399626	42399712	.	-	.	gene_id "XLOC_128081"; transcript_id "Novel_001752"; exon_number "4"; gene_type "
Dt_chr1	Cufflinks	exon	42399838	42399889	.	-	.	gene_id "XLOC_128081"; transcript_id "Novel_001752"; exon_number "5"; gene_type "
Dt_chr1	Cufflinks	exon	42400800	42400873	.	-	.	gene_id "XLOC_128081"; transcript_id "Novel_001752"; exon_number "6"; gene_type "
Dt_chr1	Cufflinks	exon	42401154	42401261	.	-	.	gene_id "XLOC_128081"; transcript_id "Novel_001752"; exon_number "7"; gene_type "
Dt_chr1	Cufflinks	exon	42401404	42402219	.	-	.	gene_id "XLOC_128081"; transcript_id "Novel_001752"; exon_number "8"; gene_type "
Dt_chr1	Cufflinks	exon	42402375	42402422	.	-	.	gene_id "XLOC_128081"; transcript_id "Novel_001752"; exon_number "9"; gene_type "
Dt_chr1	Cufflinks	exon	42402772	42402886	.	-	.	gene_id "XLOC_128081"; transcript_id "Novel_001752"; exon_number "10"; gene_type
Dt_chr1	Cufflinks	exon	42403201	42403382	.	-	.	gene_id "XLOC_128081"; transcript_id "Novel_001752"; exon_number "11"; gene_type
Dt_chr1	Cufflinks	exon	42403486	42405188	.	-	.	gene_id "XLOC_128081"; transcript_id "Novel_001752"; exon_number "12"; gene_type
Dt_chr1	Cufflinks	exon	42611255	42611658	.	-	.	gene_id "XLOC_128115"; transcript_id "Novel_001753"; exon_number "1"; gene_type "
Dt_chr1	Cufflinks	exon	42612064	42612172	.	-	.	gene_id "XLOC_128115"; transcript_id "Novel_001753"; exon_number "2"; gene_type "

Dt_chr1	Cufflinks	exon	42612265	42612381	.	-	.	gene_id "XLOC_128115"; transcript_id "Novel_001753"; exon_number "3"; gene_type "
Dt_chr1	Cufflinks	exon	42612479	42612542	.	-	.	gene_id "XLOC_128115"; transcript_id "Novel_001753"; exon_number "4"; gene_type "
Dt_chr1	Cufflinks	exon	42612829	42612937	.	-	.	gene_id "XLOC_128115"; transcript_id "Novel_001753"; exon_number "5"; gene_type "
Dt_chr1	Cufflinks	exon	42613433	42613479	.	-	.	gene_id "XLOC_128115"; transcript_id "Novel_001753"; exon_number "6"; gene_type "
Dt_chr1	Cufflinks	exon	42613739	42613857	.	-	.	gene_id "XLOC_128115"; transcript_id "Novel_001753"; exon_number "7"; gene_type "
Dt_chr1	Cufflinks	exon	42611360	42611658	.	-	.	gene_id "XLOC_128115"; transcript_id "Novel_001754"; exon_number "1"; gene_type "
Dt_chr1	Cufflinks	exon	42612064	42612381	.	-	.	gene_id "XLOC_128115"; transcript_id "Novel_001754"; exon_number "2"; gene_type "
Dt_chr1	Cufflinks	exon	42612479	42612542	.	-	.	gene_id "XLOC_128115"; transcript_id "Novel_001754"; exon_number "3"; gene_type "
Dt_chr1	Cufflinks	exon	42612829	42612937	.	-	.	gene_id "XLOC_128115"; transcript_id "Novel_001754"; exon_number "4"; gene_type "
Dt_chr1	Cufflinks	exon	42613433	42613479	.	-	.	gene_id "XLOC_128115"; transcript_id "Novel_001754"; exon_number "5"; gene_type "
Dt_chr1	Cufflinks	exon	42613739	42613864	.	-	.	gene_id "XLOC_128115"; transcript_id "Novel_001754"; exon_number "6"; gene_type "
Dt_chr1	Cufflinks	exon	42788755	42789752	.	-	.	gene_id "XLOC_128154"; transcript_id "Novel_001755"; exon_number "1"; gene_type "
Dt_chr1	Cufflinks	exon	42789847	42789915	.	-	.	gene_id "XLOC_128154"; transcript_id "Novel_001755"; exon_number "2"; gene_type "
Dt_chr1	Cufflinks	exon	42790870	42790978	.	-	.	gene_id "XLOC_128154"; transcript_id "Novel_001755"; exon_number "3"; gene_type "
Dt_chr1	Cufflinks	exon	42928206	42928610	.	-	.	gene_id "XLOC_128189"; transcript_id "Novel_001756"; exon_number "1"; gene_type "
Dt_chr1	Cufflinks	exon	42928811	42928919	.	-	.	gene_id "XLOC_128189"; transcript_id "Novel_001756"; exon_number "2"; gene_type "
Dt_chr1	Cufflinks	exon	42929003	42929119	.	-	.	gene_id "XLOC_128189"; transcript_id "Novel_001756"; exon_number "3"; gene_type "
Dt_chr1	Cufflinks	exon	42929207	42930052	.	-	.	gene_id "XLOC_128189"; transcript_id "Novel_001756"; exon_number "4"; gene_type "
Dt_chr1	Cufflinks	exon	42930127	42930246	.	-	.	gene_id "XLOC_128189"; transcript_id "Novel_001756"; exon_number "5"; gene_type "
Dt_chr1	Cufflinks	exon	42930361	42930770	.	-	.	gene_id "XLOC_128189"; transcript_id "Novel_001756"; exon_number "6"; gene_type "
Dt_chr1	Cufflinks	exon	42931034	42931266	.	-	.	gene_id "XLOC_128189"; transcript_id "Novel_001756"; exon_number "7"; gene_type "
Dt_chr1	Cufflinks	exon	42928669	42928919	.	-	.	gene_id "XLOC_128189"; transcript_id "Novel_001757"; exon_number "1"; gene_type "
Dt_chr1	Cufflinks	exon	42929003	42929119	.	-	.	gene_id "XLOC_128189"; transcript_id "Novel_001757"; exon_number "2"; gene_type "
Dt_chr1	Cufflinks	exon	42929207	42930052	.	-	.	gene_id "XLOC_128189"; transcript_id "Novel_001757"; exon_number "3"; gene_type "
Dt_chr1	Cufflinks	exon	42930127	42930246	.	-	.	gene_id "XLOC_128189"; transcript_id "Novel_001757"; exon_number "4"; gene_type "
Dt_chr1	Cufflinks	exon	42930361	42931233	.	-	.	gene_id "XLOC_128189"; transcript_id "Novel_001757"; exon_number "5"; gene_type "
Dt_chr1	Cufflinks	exon	43220620	43220882	.	-	.	gene_id "XLOC_128238"; transcript_id "Novel_001758"; exon_number "1"; gene_type "
Dt_chr1	Cufflinks	exon	43220985	43221105	.	-	.	gene_id "XLOC_128238"; transcript_id "Novel_001758"; exon_number "2"; gene_type "
Dt_chr1	Cufflinks	exon	43221605	43221932	.	-	.	gene_id "XLOC_128238"; transcript_id "Novel_001758"; exon_number "3"; gene_type "
Dt_chr1	Cufflinks	exon	43223527	43224197	.	-	.	gene_id "XLOC_128238"; transcript_id "Novel_001759"; exon_number "1"; gene_type "
Dt_chr1	Cufflinks	exon	43224273	43224334	.	-	.	gene_id "XLOC_128238"; transcript_id "Novel_001759"; exon_number "2"; gene_type "
Dt_chr1	Cufflinks	exon	43224786	43224857	.	-	.	gene_id "XLOC_128238"; transcript_id "Novel_001759"; exon_number "3"; gene_type "
Dt_chr1	Cufflinks	exon	43225653	43225808	.	-	.	gene_id "XLOC_128238"; transcript_id "Novel_001759"; exon_number "4"; gene_type "
Dt_chr1	Cufflinks	exon	43380604	43380753	.	-	.	gene_id "XLOC_128253"; transcript_id "Novel_001760"; exon_number "1"; gene_type "
Dt_chr1	Cufflinks	exon	43380834	43381366	.	-	.	gene_id "XLOC_128253"; transcript_id "Novel_001760"; exon_number "2"; gene_type "

Dt_chr1	Cufflinks	exon	43381468	43381641	.	-	.	gene_id "XLOC_128253"; transcript_id "Novel_001760"; exon_number "3"; gene_type "
Dt_chr1	Cufflinks	exon	43506044	43507078	.	-	.	gene_id "XLOC_128265"; transcript_id "Novel_001761"; exon_number "1"; gene_type "
Dt_chr1	Cufflinks	exon	43507186	43507771	.	-	.	gene_id "XLOC_128265"; transcript_id "Novel_001761"; exon_number "2"; gene_type "
Dt_chr1	Cufflinks	exon	43507864	43507926	.	-	.	gene_id "XLOC_128265"; transcript_id "Novel_001761"; exon_number "3"; gene_type "
Dt_chr1	Cufflinks	exon	43508008	43508210	.	-	.	gene_id "XLOC_128265"; transcript_id "Novel_001761"; exon_number "4"; gene_type "
Dt_chr1	Cufflinks	exon	43507953	43508121	.	-	.	gene_id "XLOC_128265"; transcript_id "Novel_001762"; exon_number "1"; gene_type "
Dt_chr1	Cufflinks	exon	43508852	43509013	.	-	.	gene_id "XLOC_128265"; transcript_id "Novel_001762"; exon_number "2"; gene_type "
Dt_chr1	Cufflinks	exon	43509109	43509171	.	-	.	gene_id "XLOC_128265"; transcript_id "Novel_001762"; exon_number "3"; gene_type "
Dt_chr1	Cufflinks	exon	43509614	43509720	.	-	.	gene_id "XLOC_128265"; transcript_id "Novel_001762"; exon_number "4"; gene_type "
Dt_chr1	Cufflinks	exon	43510071	43510567	.	-	.	gene_id "XLOC_128265"; transcript_id "Novel_001762"; exon_number "5"; gene_type "
Dt_chr1	Cufflinks	exon	43508150	43509013	.	-	.	gene_id "XLOC_128265"; transcript_id "Novel_001763"; exon_number "1"; gene_type "
Dt_chr1	Cufflinks	exon	43509109	43509171	.	-	.	gene_id "XLOC_128265"; transcript_id "Novel_001763"; exon_number "2"; gene_type "
Dt_chr1	Cufflinks	exon	43509614	43509720	.	-	.	gene_id "XLOC_128265"; transcript_id "Novel_001763"; exon_number "3"; gene_type "
Dt_chr1	Cufflinks	exon	43510071	43510148	.	-	.	gene_id "XLOC_128265"; transcript_id "Novel_001763"; exon_number "4"; gene_type "
Dt_chr1	Cufflinks	exon	43510230	43510621	.	-	.	gene_id "XLOC_128265"; transcript_id "Novel_001763"; exon_number "5"; gene_type "
Dt_chr1	Cufflinks	exon	43512192	43512834	.	-	.	gene_id "XLOC_128270"; transcript_id "Novel_001764"; exon_number "1"; gene_type "
Dt_chr1	Cufflinks	exon	43513021	43513064	.	-	.	gene_id "XLOC_128270"; transcript_id "Novel_001764"; exon_number "2"; gene_type "
Dt_chr1	Cufflinks	exon	43544904	43545246	.	-	.	gene_id "XLOC_128282"; transcript_id "Novel_001765"; exon_number "1"; gene_type "
Dt_chr1	Cufflinks	exon	43545343	43545415	.	-	.	gene_id "XLOC_128282"; transcript_id "Novel_001765"; exon_number "2"; gene_type "
Dt_chr1	Cufflinks	exon	43545838	43545953	.	-	.	gene_id "XLOC_128282"; transcript_id "Novel_001765"; exon_number "3"; gene_type "
Dt_chr1	Cufflinks	exon	43641344	43641860	.	-	.	gene_id "XLOC_128295"; transcript_id "Novel_001766"; exon_number "1"; gene_type "
Dt_chr1	Cufflinks	exon	43642351	43642578	.	-	.	gene_id "XLOC_128295"; transcript_id "Novel_001766"; exon_number "2"; gene_type "
Dt_chr1	Cufflinks	exon	43650047	43651284	.	-	.	gene_id "XLOC_128296"; transcript_id "Novel_001767"; exon_number "1"; gene_type "
Dt_chr1	Cufflinks	exon	43651496	43652238	.	-	.	gene_id "XLOC_128296"; transcript_id "Novel_001767"; exon_number "2"; gene_type "
Dt_chr1	Cufflinks	exon	43665339	43667114	.	-	.	gene_id "XLOC_128300"; transcript_id "Novel_001768"; exon_number "1"; gene_type "
Dt_chr1	Cufflinks	exon	43670194	43670584	.	-	.	gene_id "XLOC_128300"; transcript_id "Novel_001768"; exon_number "2"; gene_type "
Dt_chr1	Cufflinks	exon	43671513	43671743	.	-	.	gene_id "XLOC_128300"; transcript_id "Novel_001768"; exon_number "3"; gene_type "
Dt_chr1	Cufflinks	exon	43671849	43671941	.	-	.	gene_id "XLOC_128300"; transcript_id "Novel_001768"; exon_number "4"; gene_type "
Dt_chr1	Cufflinks	exon	43672056	43672272	.	-	.	gene_id "XLOC_128300"; transcript_id "Novel_001768"; exon_number "5"; gene_type "
Dt_chr1	Cufflinks	exon	43665352	43665730	.	-	.	gene_id "XLOC_128300"; transcript_id "Novel_001769"; exon_number "1"; gene_type "
Dt_chr1	Cufflinks	exon	43665806	43665883	.	-	.	gene_id "XLOC_128300"; transcript_id "Novel_001769"; exon_number "2"; gene_type "
Dt_chr1	Cufflinks	exon	43666745	43667114	.	-	.	gene_id "XLOC_128300"; transcript_id "Novel_001769"; exon_number "3"; gene_type "
Dt_chr1	Cufflinks	exon	43667275	43668251	.	-	.	gene_id "XLOC_128300"; transcript_id "Novel_001769"; exon_number "4"; gene_type "
Dt_chr1	Cufflinks	exon	43665352	43665883	.	-	.	gene_id "XLOC_128300"; transcript_id "Novel_001770"; exon_number "1"; gene_type "
Dt_chr1	Cufflinks	exon	43666745	43667114	.	-	.	gene_id "XLOC_128300"; transcript_id "Novel_001770"; exon_number "2"; gene_type "

Dt_chr1	Cufflinks	exon	43667275	43668251	.	-	.	gene_id "XLOC_128300";	transcript_id "Novel_001770";	exon_number "3";	gene_type "
Dt_chr1	Cufflinks	exon	43665352	43667114	.	-	.	gene_id "XLOC_128300";	transcript_id "Novel_001771";	exon_number "1";	gene_type "
Dt_chr1	Cufflinks	exon	43667275	43668266	.	-	.	gene_id "XLOC_128300";	transcript_id "Novel_001771";	exon_number "2";	gene_type "
Dt_chr1	Cufflinks	exon	43665352	43665730	.	-	.	gene_id "XLOC_128300";	transcript_id "Novel_001772";	exon_number "1";	gene_type "
Dt_chr1	Cufflinks	exon	43665806	43665883	.	-	.	gene_id "XLOC_128300";	transcript_id "Novel_001772";	exon_number "2";	gene_type "
Dt_chr1	Cufflinks	exon	43666745	43667114	.	-	.	gene_id "XLOC_128300";	transcript_id "Novel_001772";	exon_number "3";	gene_type "
Dt_chr1	Cufflinks	exon	43670194	43670584	.	-	.	gene_id "XLOC_128300";	transcript_id "Novel_001772";	exon_number "4";	gene_type "
Dt_chr1	Cufflinks	exon	43671513	43671743	.	-	.	gene_id "XLOC_128300";	transcript_id "Novel_001772";	exon_number "5";	gene_type "
Dt_chr1	Cufflinks	exon	43671849	43671941	.	-	.	gene_id "XLOC_128300";	transcript_id "Novel_001772";	exon_number "6";	gene_type "
Dt_chr1	Cufflinks	exon	43672056	43672263	.	-	.	gene_id "XLOC_128300";	transcript_id "Novel_001772";	exon_number "7";	gene_type "
Dt_chr1	Cufflinks	exon	43681970	43682397	.	-	.	gene_id "XLOC_128301";	transcript_id "Novel_001773";	exon_number "1";	gene_type "
Dt_chr1	Cufflinks	exon	43682484	43682690	.	-	.	gene_id "XLOC_128301";	transcript_id "Novel_001773";	exon_number "2";	gene_type "
Dt_chr1	Cufflinks	exon	43682828	43683037	.	-	.	gene_id "XLOC_128301";	transcript_id "Novel_001773";	exon_number "3";	gene_type "
Dt_chr1	Cufflinks	exon	43683320	43683368	.	-	.	gene_id "XLOC_128301";	transcript_id "Novel_001773";	exon_number "4";	gene_type "
Dt_chr1	Cufflinks	exon	43683644	43683843	.	-	.	gene_id "XLOC_128301";	transcript_id "Novel_001773";	exon_number "5";	gene_type "
Dt_chr1	Cufflinks	exon	43683943	43684028	.	-	.	gene_id "XLOC_128301";	transcript_id "Novel_001773";	exon_number "6";	gene_type "
Dt_chr1	Cufflinks	exon	43684159	43684315	.	-	.	gene_id "XLOC_128301";	transcript_id "Novel_001773";	exon_number "7";	gene_type "
Dt_chr1	Cufflinks	exon	43684396	43684560	.	-	.	gene_id "XLOC_128301";	transcript_id "Novel_001773";	exon_number "8";	gene_type "
Dt_chr1	Cufflinks	exon	43684697	43685128	.	-	.	gene_id "XLOC_128301";	transcript_id "Novel_001773";	exon_number "9";	gene_type "
Dt_chr1	Cufflinks	exon	43681974	43682397	.	-	.	gene_id "XLOC_128301";	transcript_id "Novel_001774";	exon_number "1";	gene_type "
Dt_chr1	Cufflinks	exon	43682484	43682690	.	-	.	gene_id "XLOC_128301";	transcript_id "Novel_001774";	exon_number "2";	gene_type "
Dt_chr1	Cufflinks	exon	43682828	43683037	.	-	.	gene_id "XLOC_128301";	transcript_id "Novel_001774";	exon_number "3";	gene_type "
Dt_chr1	Cufflinks	exon	43683320	43683625	.	-	.	gene_id "XLOC_128301";	transcript_id "Novel_001774";	exon_number "4";	gene_type "
Dt_chr1	Cufflinks	exon	43681975	43682397	.	-	.	gene_id "XLOC_128301";	transcript_id "Novel_001775";	exon_number "1";	gene_type "
Dt_chr1	Cufflinks	exon	43682484	43682690	.	-	.	gene_id "XLOC_128301";	transcript_id "Novel_001775";	exon_number "2";	gene_type "
Dt_chr1	Cufflinks	exon	43682828	43683037	.	-	.	gene_id "XLOC_128301";	transcript_id "Novel_001775";	exon_number "3";	gene_type "
Dt_chr1	Cufflinks	exon	43683320	43683368	.	-	.	gene_id "XLOC_128301";	transcript_id "Novel_001775";	exon_number "4";	gene_type "
Dt_chr1	Cufflinks	exon	43683644	43683843	.	-	.	gene_id "XLOC_128301";	transcript_id "Novel_001775";	exon_number "5";	gene_type "
Dt_chr1	Cufflinks	exon	43683943	43684028	.	-	.	gene_id "XLOC_128301";	transcript_id "Novel_001775";	exon_number "6";	gene_type "
Dt_chr1	Cufflinks	exon	43684159	43684560	.	-	.	gene_id "XLOC_128301";	transcript_id "Novel_001775";	exon_number "7";	gene_type "
Dt_chr1	Cufflinks	exon	43684697	43685035	.	-	.	gene_id "XLOC_128301";	transcript_id "Novel_001775";	exon_number "8";	gene_type "
Dt_chr1	Cufflinks	exon	43758050	43759330	.	-	.	gene_id "XLOC_128314";	transcript_id "Novel_001776";	exon_number "1";	gene_type "
Dt_chr1	Cufflinks	exon	43759661	43759764	.	-	.	gene_id "XLOC_128314";	transcript_id "Novel_001776";	exon_number "2";	gene_type "
Dt_chr1	Cufflinks	exon	43760003	43760545	.	-	.	gene_id "XLOC_128314";	transcript_id "Novel_001776";	exon_number "3";	gene_type "
Dt_chr1	Cufflinks	exon	43870478	43870948	.	-	.	gene_id "XLOC_128338";	transcript_id "Novel_001777";	exon_number "1";	gene_type "

Dt_chr1	Cufflinks	exon	43871082	43871159	.	-	.	gene_id "XLOC_128338"; transcript_id "Novel_001777"; exon_number "2"; gene_type "
Dt_chr1	Cufflinks	exon	43871273	43871494	.	-	.	gene_id "XLOC_128338"; transcript_id "Novel_001777"; exon_number "3"; gene_type "
Dt_chr1	Cufflinks	exon	43871501	43871983	.	-	.	gene_id "XLOC_128338"; transcript_id "Novel_001778"; exon_number "1"; gene_type "
Dt_chr1	Cufflinks	exon	43872270	43872367	.	-	.	gene_id "XLOC_128338"; transcript_id "Novel_001778"; exon_number "2"; gene_type "
Dt_chr1	Cufflinks	exon	43872447	43872568	.	-	.	gene_id "XLOC_128338"; transcript_id "Novel_001778"; exon_number "3"; gene_type "
Dt_chr1	Cufflinks	exon	43872646	43872922	.	-	.	gene_id "XLOC_128338"; transcript_id "Novel_001778"; exon_number "4"; gene_type "
Dt_chr1	Cufflinks	exon	43873100	43873282	.	-	.	gene_id "XLOC_128338"; transcript_id "Novel_001778"; exon_number "5"; gene_type "
Dt_chr1	Cufflinks	exon	43873619	43873732	.	-	.	gene_id "XLOC_128338"; transcript_id "Novel_001778"; exon_number "6"; gene_type "
Dt_chr1	Cufflinks	exon	43873838	43873949	.	-	.	gene_id "XLOC_128338"; transcript_id "Novel_001778"; exon_number "7"; gene_type "
Dt_chr1	Cufflinks	exon	43874034	43874143	.	-	.	gene_id "XLOC_128338"; transcript_id "Novel_001778"; exon_number "8"; gene_type "
Dt_chr1	Cufflinks	exon	43874232	43874385	.	-	.	gene_id "XLOC_128338"; transcript_id "Novel_001778"; exon_number "9"; gene_type "
Dt_chr1	Cufflinks	exon	43874536	43874765	.	-	.	gene_id "XLOC_128338"; transcript_id "Novel_001778"; exon_number "10"; gene_type
Dt_chr1	Cufflinks	exon	43874492	43874959	.	-	.	gene_id "XLOC_128338"; transcript_id "Novel_001779"; exon_number "1"; gene_type "
Dt_chr1	Cufflinks	exon	43875056	43875158	.	-	.	gene_id "XLOC_128338"; transcript_id "Novel_001779"; exon_number "2"; gene_type "
Dt_chr1	Cufflinks	exon	43875245	43875390	.	-	.	gene_id "XLOC_128338"; transcript_id "Novel_001779"; exon_number "3"; gene_type "
Dt_chr1	Cufflinks	exon	43876560	43876813	.	-	.	gene_id "XLOC_128338"; transcript_id "Novel_001779"; exon_number "4"; gene_type "
Dt_chr1	Cufflinks	exon	43886367	43887017	.	-	.	gene_id "XLOC_128341"; transcript_id "Novel_001780"; exon_number "1"; gene_type "
Dt_chr1	Cufflinks	exon	43887099	43887240	.	-	.	gene_id "XLOC_128341"; transcript_id "Novel_001780"; exon_number "2"; gene_type "
Dt_chr1	Cufflinks	exon	43887627	43888063	.	-	.	gene_id "XLOC_128341"; transcript_id "Novel_001780"; exon_number "3"; gene_type "
Dt_chr1	Cufflinks	exon	43963717	43963857	.	-	.	gene_id "XLOC_128344"; transcript_id "Novel_001781"; exon_number "1"; gene_type "
Dt_chr1	Cufflinks	exon	43966250	43966867	.	-	.	gene_id "XLOC_128344"; transcript_id "Novel_001781"; exon_number "2"; gene_type "
Dt_chr1	Cufflinks	exon	43986520	43987262	.	-	.	gene_id "XLOC_128345"; transcript_id "Novel_001782"; exon_number "1"; gene_type "
Dt_chr1	Cufflinks	exon	43987348	43987547	.	-	.	gene_id "XLOC_128345"; transcript_id "Novel_001782"; exon_number "2"; gene_type "
Dt_chr1	Cufflinks	exon	43987643	43987844	.	-	.	gene_id "XLOC_128345"; transcript_id "Novel_001782"; exon_number "3"; gene_type "
Dt_chr1	Cufflinks	exon	43988434	43989914	.	-	.	gene_id "XLOC_128345"; transcript_id "Novel_001782"; exon_number "4"; gene_type "
Dt_chr1	Cufflinks	exon	44984381	44984644	.	-	.	gene_id "XLOC_128456"; transcript_id "Novel_001783"; exon_number "1"; gene_type "
Dt_chr1	Cufflinks	exon	44986328	44986459	.	-	.	gene_id "XLOC_128456"; transcript_id "Novel_001783"; exon_number "2"; gene_type "
Dt_chr1	Cufflinks	exon	45266285	45266898	.	-	.	gene_id "XLOC_128492"; transcript_id "Novel_001784"; exon_number "1"; gene_type "
Dt_chr1	Cufflinks	exon	45267697	45268690	.	-	.	gene_id "XLOC_128492"; transcript_id "Novel_001784"; exon_number "2"; gene_type "
Dt_chr1	Cufflinks	exon	46207336	46207968	.	-	.	gene_id "XLOC_128633"; transcript_id "Novel_001785"; exon_number "1"; gene_type "
Dt_chr1	Cufflinks	exon	46208224	46208330	.	-	.	gene_id "XLOC_128633"; transcript_id "Novel_001785"; exon_number "2"; gene_type "
Dt_chr1	Cufflinks	exon	46208563	46208620	.	-	.	gene_id "XLOC_128633"; transcript_id "Novel_001785"; exon_number "3"; gene_type "
Dt_chr1	Cufflinks	exon	46209529	46209630	.	-	.	gene_id "XLOC_128633"; transcript_id "Novel_001785"; exon_number "4"; gene_type "
Dt_chr1	Cufflinks	exon	46952121	46952406	.	-	.	gene_id "XLOC_128711"; transcript_id "Novel_001786"; exon_number "1"; gene_type "
Dt_chr1	Cufflinks	exon	46953167	46953301	.	-	.	gene_id "XLOC_128711"; transcript_id "Novel_001786"; exon_number "2"; gene_type "

Dt_chr1	Cufflinks	exon	47530184	47530732	.	-	.	gene_id "XLOC_128772"; transcript_id "Novel_001787"; exon_number "1"; gene_type "
Dt_chr1	Cufflinks	exon	47531337	47531469	.	-	.	gene_id "XLOC_128772"; transcript_id "Novel_001787"; exon_number "2"; gene_type "
Dt_chr1	Cufflinks	exon	47532647	47532696	.	-	.	gene_id "XLOC_128772"; transcript_id "Novel_001787"; exon_number "3"; gene_type "
Dt_chr1	Cufflinks	exon	47530221	47530732	.	-	.	gene_id "XLOC_128772"; transcript_id "Novel_001788"; exon_number "1"; gene_type "
Dt_chr1	Cufflinks	exon	47531337	47532638	.	-	.	gene_id "XLOC_128772"; transcript_id "Novel_001788"; exon_number "2"; gene_type "
Dt_chr1	Cufflinks	exon	47612933	47615299	.	-	.	gene_id "XLOC_128787"; transcript_id "Novel_001789"; exon_number "1"; gene_type "
Dt_chr1	Cufflinks	exon	47616351	47616395	.	-	.	gene_id "XLOC_128787"; transcript_id "Novel_001789"; exon_number "2"; gene_type "
Dt_chr1	Cufflinks	exon	47617390	47617720	.	-	.	gene_id "XLOC_128787"; transcript_id "Novel_001789"; exon_number "3"; gene_type "
Dt_chr1	Cufflinks	exon	47612933	47615299	.	-	.	gene_id "XLOC_128787"; transcript_id "Novel_001790"; exon_number "1"; gene_type "
Dt_chr1	Cufflinks	exon	47616355	47616395	.	-	.	gene_id "XLOC_128787"; transcript_id "Novel_001790"; exon_number "2"; gene_type "
Dt_chr1	Cufflinks	exon	47617390	47617720	.	-	.	gene_id "XLOC_128787"; transcript_id "Novel_001790"; exon_number "3"; gene_type "
Dt_chr1	Cufflinks	exon	51995883	51999585	.	-	.	gene_id "XLOC_129256"; transcript_id "Novel_001791"; exon_number "1"; gene_type "
Dt_chr1	Cufflinks	exon	51999711	51999812	.	-	.	gene_id "XLOC_129256"; transcript_id "Novel_001791"; exon_number "2"; gene_type "
Dt_chr1	Cufflinks	exon	52000299	52000433	.	-	.	gene_id "XLOC_129256"; transcript_id "Novel_001791"; exon_number "3"; gene_type "
Dt_chr1	Cufflinks	exon	52000597	52000655	.	-	.	gene_id "XLOC_129256"; transcript_id "Novel_001791"; exon_number "4"; gene_type "
Dt_chr1	Cufflinks	exon	51996020	51998925	.	-	.	gene_id "XLOC_129256"; transcript_id "Novel_001792"; exon_number "1"; gene_type "
Dt_chr1	Cufflinks	exon	51999012	51999071	.	-	.	gene_id "XLOC_129256"; transcript_id "Novel_001792"; exon_number "2"; gene_type "
Dt_chr1	Cufflinks	exon	51999538	51999585	.	-	.	gene_id "XLOC_129256"; transcript_id "Novel_001792"; exon_number "3"; gene_type "
Dt_chr1	Cufflinks	exon	51999711	51999812	.	-	.	gene_id "XLOC_129256"; transcript_id "Novel_001792"; exon_number "4"; gene_type "
Dt_chr1	Cufflinks	exon	52000299	52000446	.	-	.	gene_id "XLOC_129256"; transcript_id "Novel_001792"; exon_number "5"; gene_type "
Dt_chr1	Cufflinks	exon	52000597	52000651	.	-	.	gene_id "XLOC_129256"; transcript_id "Novel_001792"; exon_number "6"; gene_type "
Dt_chr1	Cufflinks	exon	51996020	51998336	.	-	.	gene_id "XLOC_129256"; transcript_id "Novel_001793"; exon_number "1"; gene_type "
Dt_chr1	Cufflinks	exon	51999012	51999071	.	-	.	gene_id "XLOC_129256"; transcript_id "Novel_001793"; exon_number "2"; gene_type "
Dt_chr1	Cufflinks	exon	51999538	51999585	.	-	.	gene_id "XLOC_129256"; transcript_id "Novel_001793"; exon_number "3"; gene_type "
Dt_chr1	Cufflinks	exon	51999711	51999812	.	-	.	gene_id "XLOC_129256"; transcript_id "Novel_001793"; exon_number "4"; gene_type "
Dt_chr1	Cufflinks	exon	52000299	52000651	.	-	.	gene_id "XLOC_129256"; transcript_id "Novel_001793"; exon_number "5"; gene_type "
Dt_chr1	Cufflinks	exon	51996055	51998333	.	-	.	gene_id "XLOC_129256"; transcript_id "Novel_001794"; exon_number "1"; gene_type "
Dt_chr1	Cufflinks	exon	51998614	51999585	.	-	.	gene_id "XLOC_129256"; transcript_id "Novel_001794"; exon_number "2"; gene_type "
Dt_chr1	Cufflinks	exon	51999711	51999812	.	-	.	gene_id "XLOC_129256"; transcript_id "Novel_001794"; exon_number "3"; gene_type "
Dt_chr1	Cufflinks	exon	52000299	52000446	.	-	.	gene_id "XLOC_129256"; transcript_id "Novel_001794"; exon_number "4"; gene_type "
Dt_chr1	Cufflinks	exon	52000597	52000691	.	-	.	gene_id "XLOC_129256"; transcript_id "Novel_001794"; exon_number "5"; gene_type "
Dt_chr1	Cufflinks	exon	51996056	51999585	.	-	.	gene_id "XLOC_129256"; transcript_id "Novel_001795"; exon_number "1"; gene_type "
Dt_chr1	Cufflinks	exon	51999711	51999812	.	-	.	gene_id "XLOC_129256"; transcript_id "Novel_001795"; exon_number "2"; gene_type "
Dt_chr1	Cufflinks	exon	52000299	52000446	.	-	.	gene_id "XLOC_129256"; transcript_id "Novel_001795"; exon_number "3"; gene_type "
Dt_chr1	Cufflinks	exon	52000597	52000701	.	-	.	gene_id "XLOC_129256"; transcript_id "Novel_001795"; exon_number "4"; gene_type "

Dt_chr1	Cufflinks	exon	52209743	52210743	.	-	.	gene_id "XLOC_129277"; transcript_id "Novel_001796"; exon_number "1"; gene_type "
Dt_chr1	Cufflinks	exon	52210963	52211039	.	-	.	gene_id "XLOC_129277"; transcript_id "Novel_001796"; exon_number "2"; gene_type "
Dt_chr1	Cufflinks	exon	52211147	52211783	.	-	.	gene_id "XLOC_129277"; transcript_id "Novel_001796"; exon_number "3"; gene_type "
Dt_chr1	Cufflinks	exon	52211878	52212833	.	-	.	gene_id "XLOC_129277"; transcript_id "Novel_001796"; exon_number "4"; gene_type "
Dt_chr1	Cufflinks	exon	52209743	52210743	.	-	.	gene_id "XLOC_129277"; transcript_id "Novel_001797"; exon_number "1"; gene_type "
Dt_chr1	Cufflinks	exon	52210963	52211039	.	-	.	gene_id "XLOC_129277"; transcript_id "Novel_001797"; exon_number "2"; gene_type "
Dt_chr1	Cufflinks	exon	52211147	52211368	.	-	.	gene_id "XLOC_129277"; transcript_id "Novel_001797"; exon_number "3"; gene_type "
Dt_chr1	Cufflinks	exon	52211756	52211783	.	-	.	gene_id "XLOC_129277"; transcript_id "Novel_001797"; exon_number "4"; gene_type "
Dt_chr1	Cufflinks	exon	52211878	52212833	.	-	.	gene_id "XLOC_129277"; transcript_id "Novel_001797"; exon_number "5"; gene_type "
Dt_chr1	Cufflinks	exon	52209986	52210743	.	-	.	gene_id "XLOC_129277"; transcript_id "Novel_001798"; exon_number "1"; gene_type "
Dt_chr1	Cufflinks	exon	52210963	52211039	.	-	.	gene_id "XLOC_129277"; transcript_id "Novel_001798"; exon_number "2"; gene_type "
Dt_chr1	Cufflinks	exon	52211147	52211368	.	-	.	gene_id "XLOC_129277"; transcript_id "Novel_001798"; exon_number "3"; gene_type "
Dt_chr1	Cufflinks	exon	52211756	52211783	.	-	.	gene_id "XLOC_129277"; transcript_id "Novel_001798"; exon_number "4"; gene_type "
Dt_chr1	Cufflinks	exon	52211878	52211929	.	-	.	gene_id "XLOC_129277"; transcript_id "Novel_001798"; exon_number "5"; gene_type "
Dt_chr1	Cufflinks	exon	52212246	52212305	.	-	.	gene_id "XLOC_129277"; transcript_id "Novel_001798"; exon_number "6"; gene_type "
Dt_chr1	Cufflinks	exon	52212484	52212913	.	-	.	gene_id "XLOC_129277"; transcript_id "Novel_001798"; exon_number "7"; gene_type "
Dt_chr1	Cufflinks	exon	52332103	52332521	.	-	.	gene_id "XLOC_129286"; transcript_id "Novel_001799"; exon_number "1"; gene_type "
Dt_chr1	Cufflinks	exon	52332601	52332680	.	-	.	gene_id "XLOC_129286"; transcript_id "Novel_001799"; exon_number "2"; gene_type "
Dt_chr1	Cufflinks	exon	52332836	52332917	.	-	.	gene_id "XLOC_129286"; transcript_id "Novel_001799"; exon_number "3"; gene_type "
Dt_chr1	Cufflinks	exon	52333087	52333151	.	-	.	gene_id "XLOC_129286"; transcript_id "Novel_001799"; exon_number "4"; gene_type "
Dt_chr1	Cufflinks	exon	52333257	52333904	.	-	.	gene_id "XLOC_129286"; transcript_id "Novel_001799"; exon_number "5"; gene_type "
Dt_chr1	Cufflinks	exon	52332103	52332521	.	-	.	gene_id "XLOC_129286"; transcript_id "Novel_001800"; exon_number "1"; gene_type "
Dt_chr1	Cufflinks	exon	52332601	52332680	.	-	.	gene_id "XLOC_129286"; transcript_id "Novel_001800"; exon_number "2"; gene_type "
Dt_chr1	Cufflinks	exon	52332836	52332917	.	-	.	gene_id "XLOC_129286"; transcript_id "Novel_001800"; exon_number "3"; gene_type "
Dt_chr1	Cufflinks	exon	52333087	52333151	.	-	.	gene_id "XLOC_129286"; transcript_id "Novel_001800"; exon_number "4"; gene_type "
Dt_chr1	Cufflinks	exon	52333257	52333846	.	-	.	gene_id "XLOC_129286"; transcript_id "Novel_001800"; exon_number "5"; gene_type "
Dt_chr1	Cufflinks	exon	52334370	52334454	.	-	.	gene_id "XLOC_129286"; transcript_id "Novel_001800"; exon_number "6"; gene_type "
Dt_chr1	Cufflinks	exon	52334563	52334660	.	-	.	gene_id "XLOC_129286"; transcript_id "Novel_001800"; exon_number "7"; gene_type "
Dt_chr1	Cufflinks	exon	52334760	52334823	.	-	.	gene_id "XLOC_129286"; transcript_id "Novel_001800"; exon_number "8"; gene_type "
Dt_chr1	Cufflinks	exon	52334993	52335119	.	-	.	gene_id "XLOC_129286"; transcript_id "Novel_001800"; exon_number "9"; gene_type "
Dt_chr1	Cufflinks	exon	52335266	52335348	.	-	.	gene_id "XLOC_129286"; transcript_id "Novel_001800"; exon_number "10"; gene_type
Dt_chr1	Cufflinks	exon	52335495	52335592	.	-	.	gene_id "XLOC_129286"; transcript_id "Novel_001800"; exon_number "11"; gene_type
Dt_chr1	Cufflinks	exon	52335893	52336153	.	-	.	gene_id "XLOC_129286"; transcript_id "Novel_001800"; exon_number "12"; gene_type
Dt_chr1	Cufflinks	exon	52332159	52332521	.	-	.	gene_id "XLOC_129286"; transcript_id "Novel_001801"; exon_number "1"; gene_type "
Dt_chr1	Cufflinks	exon	52332601	52332680	.	-	.	gene_id "XLOC_129286"; transcript_id "Novel_001801"; exon_number "2"; gene_type "

Dt_chr1	Cufflinks	exon	52332836	52332917	.	-	.	gene_id "XLOC_129286"; transcript_id "Novel_001801"; exon_number "3"; gene_type "
Dt_chr1	Cufflinks	exon	52333087	52333151	.	-	.	gene_id "XLOC_129286"; transcript_id "Novel_001801"; exon_number "4"; gene_type "
Dt_chr1	Cufflinks	exon	52333257	52333358	.	-	.	gene_id "XLOC_129286"; transcript_id "Novel_001801"; exon_number "5"; gene_type "
Dt_chr1	Cufflinks	exon	52333723	52333846	.	-	.	gene_id "XLOC_129286"; transcript_id "Novel_001801"; exon_number "6"; gene_type "
Dt_chr1	Cufflinks	exon	52334370	52334454	.	-	.	gene_id "XLOC_129286"; transcript_id "Novel_001801"; exon_number "7"; gene_type "
Dt_chr1	Cufflinks	exon	52334563	52334660	.	-	.	gene_id "XLOC_129286"; transcript_id "Novel_001801"; exon_number "8"; gene_type "
Dt_chr1	Cufflinks	exon	52334760	52334823	.	-	.	gene_id "XLOC_129286"; transcript_id "Novel_001801"; exon_number "9"; gene_type "
Dt_chr1	Cufflinks	exon	52334993	52335119	.	-	.	gene_id "XLOC_129286"; transcript_id "Novel_001801"; exon_number "10"; gene_type
Dt_chr1	Cufflinks	exon	52335266	52335348	.	-	.	gene_id "XLOC_129286"; transcript_id "Novel_001801"; exon_number "11"; gene_type
Dt_chr1	Cufflinks	exon	52335495	52335592	.	-	.	gene_id "XLOC_129286"; transcript_id "Novel_001801"; exon_number "12"; gene_type
Dt_chr1	Cufflinks	exon	52335893	52336154	.	-	.	gene_id "XLOC_129286"; transcript_id "Novel_001801"; exon_number "13"; gene_type
Dt_chr1	Cufflinks	exon	52332166	52332521	.	-	.	gene_id "XLOC_129286"; transcript_id "Novel_001802"; exon_number "1"; gene_type "
Dt_chr1	Cufflinks	exon	52332601	52332680	.	-	.	gene_id "XLOC_129286"; transcript_id "Novel_001802"; exon_number "2"; gene_type "
Dt_chr1	Cufflinks	exon	52332836	52332917	.	-	.	gene_id "XLOC_129286"; transcript_id "Novel_001802"; exon_number "3"; gene_type "
Dt_chr1	Cufflinks	exon	52333087	52333151	.	-	.	gene_id "XLOC_129286"; transcript_id "Novel_001802"; exon_number "4"; gene_type "
Dt_chr1	Cufflinks	exon	52333257	52333358	.	-	.	gene_id "XLOC_129286"; transcript_id "Novel_001802"; exon_number "5"; gene_type "
Dt_chr1	Cufflinks	exon	52333723	52333846	.	-	.	gene_id "XLOC_129286"; transcript_id "Novel_001802"; exon_number "6"; gene_type "
Dt_chr1	Cufflinks	exon	52334370	52334454	.	-	.	gene_id "XLOC_129286"; transcript_id "Novel_001802"; exon_number "7"; gene_type "
Dt_chr1	Cufflinks	exon	52334563	52334660	.	-	.	gene_id "XLOC_129286"; transcript_id "Novel_001802"; exon_number "8"; gene_type "
Dt_chr1	Cufflinks	exon	52334760	52334823	.	-	.	gene_id "XLOC_129286"; transcript_id "Novel_001802"; exon_number "9"; gene_type "
Dt_chr1	Cufflinks	exon	52334993	52335119	.	-	.	gene_id "XLOC_129286"; transcript_id "Novel_001802"; exon_number "10"; gene_type
Dt_chr1	Cufflinks	exon	52335255	52335348	.	-	.	gene_id "XLOC_129286"; transcript_id "Novel_001802"; exon_number "11"; gene_type
Dt_chr1	Cufflinks	exon	52335495	52335592	.	-	.	gene_id "XLOC_129286"; transcript_id "Novel_001802"; exon_number "12"; gene_type
Dt_chr1	Cufflinks	exon	52335893	52336154	.	-	.	gene_id "XLOC_129286"; transcript_id "Novel_001802"; exon_number "13"; gene_type
Dt_chr1	Cufflinks	exon	52332175	52332521	.	-	.	gene_id "XLOC_129286"; transcript_id "Novel_001803"; exon_number "1"; gene_type "
Dt_chr1	Cufflinks	exon	52332601	52332680	.	-	.	gene_id "XLOC_129286"; transcript_id "Novel_001803"; exon_number "2"; gene_type "
Dt_chr1	Cufflinks	exon	52332836	52332912	.	-	.	gene_id "XLOC_129286"; transcript_id "Novel_001803"; exon_number "3"; gene_type "
Dt_chr1	Cufflinks	exon	52333087	52333151	.	-	.	gene_id "XLOC_129286"; transcript_id "Novel_001803"; exon_number "4"; gene_type "
Dt_chr1	Cufflinks	exon	52333257	52333358	.	-	.	gene_id "XLOC_129286"; transcript_id "Novel_001803"; exon_number "5"; gene_type "
Dt_chr1	Cufflinks	exon	52333723	52333846	.	-	.	gene_id "XLOC_129286"; transcript_id "Novel_001803"; exon_number "6"; gene_type "
Dt_chr1	Cufflinks	exon	52334370	52334454	.	-	.	gene_id "XLOC_129286"; transcript_id "Novel_001803"; exon_number "7"; gene_type "
Dt_chr1	Cufflinks	exon	52334563	52334660	.	-	.	gene_id "XLOC_129286"; transcript_id "Novel_001803"; exon_number "8"; gene_type "
Dt_chr1	Cufflinks	exon	52334760	52334823	.	-	.	gene_id "XLOC_129286"; transcript_id "Novel_001803"; exon_number "9"; gene_type "
Dt_chr1	Cufflinks	exon	52334993	52335119	.	-	.	gene_id "XLOC_129286"; transcript_id "Novel_001803"; exon_number "10"; gene_type
Dt_chr1	Cufflinks	exon	52335255	52335348	.	-	.	gene_id "XLOC_129286"; transcript_id "Novel_001803"; exon_number "11"; gene_type

Dt_chr1	Cufflinks	exon	52335495	52335592	.	-	.	gene_id "XLOC_129286"; transcript_id "Novel_001803"; exon_number "12"; gene_type
Dt_chr1	Cufflinks	exon	52335893	52336147	.	-	.	gene_id "XLOC_129286"; transcript_id "Novel_001803"; exon_number "13"; gene_type
Dt_chr1	Cufflinks	exon	52332184	52332521	.	-	.	gene_id "XLOC_129286"; transcript_id "Novel_001804"; exon_number "1"; gene_type "
Dt_chr1	Cufflinks	exon	52332601	52332680	.	-	.	gene_id "XLOC_129286"; transcript_id "Novel_001804"; exon_number "2"; gene_type "
Dt_chr1	Cufflinks	exon	52332836	52332912	.	-	.	gene_id "XLOC_129286"; transcript_id "Novel_001804"; exon_number "3"; gene_type "
Dt_chr1	Cufflinks	exon	52333087	52333151	.	-	.	gene_id "XLOC_129286"; transcript_id "Novel_001804"; exon_number "4"; gene_type "
Dt_chr1	Cufflinks	exon	52333257	52333846	.	-	.	gene_id "XLOC_129286"; transcript_id "Novel_001804"; exon_number "5"; gene_type "
Dt_chr1	Cufflinks	exon	52334370	52334454	.	-	.	gene_id "XLOC_129286"; transcript_id "Novel_001804"; exon_number "6"; gene_type "
Dt_chr1	Cufflinks	exon	52334563	52334660	.	-	.	gene_id "XLOC_129286"; transcript_id "Novel_001804"; exon_number "7"; gene_type "
Dt_chr1	Cufflinks	exon	52334760	52334823	.	-	.	gene_id "XLOC_129286"; transcript_id "Novel_001804"; exon_number "8"; gene_type "
Dt_chr1	Cufflinks	exon	52334993	52335119	.	-	.	gene_id "XLOC_129286"; transcript_id "Novel_001804"; exon_number "9"; gene_type "
Dt_chr1	Cufflinks	exon	52335255	52335348	.	-	.	gene_id "XLOC_129286"; transcript_id "Novel_001804"; exon_number "10"; gene_type
Dt_chr1	Cufflinks	exon	52335495	52335592	.	-	.	gene_id "XLOC_129286"; transcript_id "Novel_001804"; exon_number "11"; gene_type
Dt_chr1	Cufflinks	exon	52335893	52336143	.	-	.	gene_id "XLOC_129286"; transcript_id "Novel_001804"; exon_number "12"; gene_type
Dt_chr1	Cufflinks	exon	52626442	52627220	.	-	.	gene_id "XLOC_129319"; transcript_id "Novel_001805"; exon_number "1"; gene_type "
Dt_chr1	Cufflinks	exon	52633228	52633339	.	-	.	gene_id "XLOC_129319"; transcript_id "Novel_001805"; exon_number "2"; gene_type "
Dt_chr1	Cufflinks	exon	52633488	52633572	.	-	.	gene_id "XLOC_129319"; transcript_id "Novel_001805"; exon_number "3"; gene_type "
Dt_chr1	Cufflinks	exon	52634772	52634941	.	-	.	gene_id "XLOC_129319"; transcript_id "Novel_001805"; exon_number "4"; gene_type "
Dt_chr1	Cufflinks	exon	52632077	52633572	.	-	.	gene_id "XLOC_129319"; transcript_id "Novel_001806"; exon_number "1"; gene_type "
Dt_chr1	Cufflinks	exon	52634772	52634941	.	-	.	gene_id "XLOC_129319"; transcript_id "Novel_001806"; exon_number "2"; gene_type "
Dt_chr1	Cufflinks	exon	54260432	54261329	.	-	.	gene_id "XLOC_129464"; transcript_id "Novel_001807"; exon_number "1"; gene_type "
Dt_chr1	Cufflinks	exon	54263127	54263241	.	-	.	gene_id "XLOC_129464"; transcript_id "Novel_001807"; exon_number "2"; gene_type "
Dt_chr1	Cufflinks	exon	54803193	54803414	.	-	.	gene_id "XLOC_129495"; transcript_id "Novel_001808"; exon_number "1"; gene_type "
Dt_chr1	Cufflinks	exon	54803525	54804582	.	-	.	gene_id "XLOC_129495"; transcript_id "Novel_001808"; exon_number "2"; gene_type "
Dt_chr1	Cufflinks	exon	55419110	55419644	.	-	.	gene_id "XLOC_129556"; transcript_id "Novel_001809"; exon_number "1"; gene_type "
Dt_chr1	Cufflinks	exon	55419746	55419964	.	-	.	gene_id "XLOC_129556"; transcript_id "Novel_001809"; exon_number "2"; gene_type "
Dt_chr1	Cufflinks	exon	55420142	55420221	.	-	.	gene_id "XLOC_129556"; transcript_id "Novel_001809"; exon_number "3"; gene_type "
Dt_chr1	Cufflinks	exon	55420373	55420436	.	-	.	gene_id "XLOC_129556"; transcript_id "Novel_001809"; exon_number "4"; gene_type "
Dt_chr1	Cufflinks	exon	55634272	55634917	.	-	.	gene_id "XLOC_129573"; transcript_id "Novel_001810"; exon_number "1"; gene_type "
Dt_chr1	Cufflinks	exon	55635489	55635546	.	-	.	gene_id "XLOC_129573"; transcript_id "Novel_001810"; exon_number "2"; gene_type "
Dt_chr1	Cufflinks	exon	55636891	55636936	.	-	.	gene_id "XLOC_129573"; transcript_id "Novel_001810"; exon_number "3"; gene_type "
Dt_chr1	Cufflinks	exon	58460452	58460889	.	-	.	gene_id "XLOC_129882"; transcript_id "Novel_001811"; exon_number "1"; gene_type "
Dt_chr1	Cufflinks	exon	58460971	58461214	.	-	.	gene_id "XLOC_129882"; transcript_id "Novel_001811"; exon_number "2"; gene_type "
Dt_chr1	Cufflinks	exon	58461535	58461588	.	-	.	gene_id "XLOC_129882"; transcript_id "Novel_001811"; exon_number "3"; gene_type "
Dt_chr1	Cufflinks	exon	58461675	58461921	.	-	.	gene_id "XLOC_129882"; transcript_id "Novel_001811"; exon_number "4"; gene_type "

Dt_chr1	Cufflinks	exon	58465173	58465378	.	-	.	gene_id "XLOC_129882"; transcript_id "Novel_001811"; exon_number "5"; gene_type "
Dt_chr1	Cufflinks	exon	58460453	58460889	.	-	.	gene_id "XLOC_129882"; transcript_id "Novel_001812"; exon_number "1"; gene_type "
Dt_chr1	Cufflinks	exon	58460971	58461214	.	-	.	gene_id "XLOC_129882"; transcript_id "Novel_001812"; exon_number "2"; gene_type "
Dt_chr1	Cufflinks	exon	58461535	58461921	.	-	.	gene_id "XLOC_129882"; transcript_id "Novel_001812"; exon_number "3"; gene_type "
Dt_chr1	Cufflinks	exon	58465173	58465336	.	-	.	gene_id "XLOC_129882"; transcript_id "Novel_001812"; exon_number "4"; gene_type "
Dt_chr1	Cufflinks	exon	58460453	58460889	.	-	.	gene_id "XLOC_129882"; transcript_id "Novel_001813"; exon_number "1"; gene_type "
Dt_chr1	Cufflinks	exon	58460971	58461147	.	-	.	gene_id "XLOC_129882"; transcript_id "Novel_001813"; exon_number "2"; gene_type "
Dt_chr1	Cufflinks	exon	58461535	58461588	.	-	.	gene_id "XLOC_129882"; transcript_id "Novel_001813"; exon_number "3"; gene_type "
Dt_chr1	Cufflinks	exon	58461675	58461921	.	-	.	gene_id "XLOC_129882"; transcript_id "Novel_001813"; exon_number "4"; gene_type "
Dt_chr1	Cufflinks	exon	58465173	58465510	.	-	.	gene_id "XLOC_129882"; transcript_id "Novel_001813"; exon_number "5"; gene_type "
Dt_chr1	Cufflinks	exon	58460456	58460889	.	-	.	gene_id "XLOC_129882"; transcript_id "Novel_001814"; exon_number "1"; gene_type "
Dt_chr1	Cufflinks	exon	58460971	58461147	.	-	.	gene_id "XLOC_129882"; transcript_id "Novel_001814"; exon_number "2"; gene_type "
Dt_chr1	Cufflinks	exon	58461535	58461921	.	-	.	gene_id "XLOC_129882"; transcript_id "Novel_001814"; exon_number "3"; gene_type "
Dt_chr1	Cufflinks	exon	58465173	58465323	.	-	.	gene_id "XLOC_129882"; transcript_id "Novel_001814"; exon_number "4"; gene_type "
Dt_chr1	Cufflinks	exon	61523628	61524375	.	-	.	gene_id "XLOC_130074"; transcript_id "Novel_001815"; exon_number "1"; gene_type "
Dt_chr1	Cufflinks	exon	61534090	61534167	.	-	.	gene_id "XLOC_130074"; transcript_id "Novel_001815"; exon_number "2"; gene_type "
Dt_chr1	Cufflinks	exon	61534350	61534406	.	-	.	gene_id "XLOC_130074"; transcript_id "Novel_001815"; exon_number "3"; gene_type "
Dt_chr1	Cufflinks	exon	61535295	61535545	.	-	.	gene_id "XLOC_130074"; transcript_id "Novel_001815"; exon_number "4"; gene_type "
Dt_chr1	Cufflinks	exon	62728395	62728796	.	-	.	gene_id "XLOC_130139"; transcript_id "Novel_001816"; exon_number "1"; gene_type "
Dt_chr1	Cufflinks	exon	62729341	62729457	.	-	.	gene_id "XLOC_130139"; transcript_id "Novel_001816"; exon_number "2"; gene_type "
Dt_chr1	Cufflinks	exon	62729943	62730074	.	-	.	gene_id "XLOC_130139"; transcript_id "Novel_001816"; exon_number "3"; gene_type "
Dt_chr1	Cufflinks	exon	62730987	62731323	.	-	.	gene_id "XLOC_130139"; transcript_id "Novel_001816"; exon_number "4"; gene_type "
Dt_chr1	Cufflinks	exon	62819453	62821108	.	-	.	gene_id "XLOC_130151"; transcript_id "Novel_001817"; exon_number "1"; gene_type "
Dt_chr1	Cufflinks	exon	62821626	62822230	.	-	.	gene_id "XLOC_130151"; transcript_id "Novel_001817"; exon_number "2"; gene_type "
Dt_chr1	Cufflinks	exon	64074790	64075310	.	-	.	gene_id "XLOC_130221"; transcript_id "Novel_001818"; exon_number "1"; gene_type "
Dt_chr1	Cufflinks	exon	64076780	64077234	.	-	.	gene_id "XLOC_130221"; transcript_id "Novel_001818"; exon_number "2"; gene_type "
Dt_chr1	Cufflinks	exon	64077379	64077547	.	-	.	gene_id "XLOC_130221"; transcript_id "Novel_001818"; exon_number "3"; gene_type "
Dt_chr1	Cufflinks	exon	64076604	64077234	.	-	.	gene_id "XLOC_130221"; transcript_id "Novel_001819"; exon_number "1"; gene_type "
Dt_chr1	Cufflinks	exon	64077379	64077554	.	-	.	gene_id "XLOC_130221"; transcript_id "Novel_001819"; exon_number "2"; gene_type "
Dt_chr1	Cufflinks	exon	65258545	65258654	.	-	.	gene_id "XLOC_130284"; transcript_id "Novel_001820"; exon_number "1"; gene_type "
Dt_chr1	Cufflinks	exon	65259348	65259588	.	-	.	gene_id "XLOC_130284"; transcript_id "Novel_001820"; exon_number "2"; gene_type "
Dt_chr1	Cufflinks	exon	73423763	73426240	.	-	.	gene_id "XLOC_130728"; transcript_id "Novel_001821"; exon_number "1"; gene_type "
Dt_chr1	Cufflinks	exon	73426334	73427504	.	-	.	gene_id "XLOC_130728"; transcript_id "Novel_001821"; exon_number "2"; gene_type "
Dt_chr1	Cufflinks	exon	74594589	74595414	.	-	.	gene_id "XLOC_130792"; transcript_id "Novel_001822"; exon_number "1"; gene_type "
Dt_chr1	Cufflinks	exon	74596821	74596869	.	-	.	gene_id "XLOC_130792"; transcript_id "Novel_001822"; exon_number "2"; gene_type "

Dt_chr1	Cufflinks	exon	74596985	74597038	.	-	.	gene_id "XLOC_130792"; transcript_id "Novel_001822"; exon_number "3"; gene_type "
Dt_chr1	Cufflinks	exon	74597189	74597248	.	-	.	gene_id "XLOC_130792"; transcript_id "Novel_001822"; exon_number "4"; gene_type "
Dt_chr1	Cufflinks	exon	74597404	74597462	.	-	.	gene_id "XLOC_130792"; transcript_id "Novel_001822"; exon_number "5"; gene_type "
Dt_chr1	Cufflinks	exon	74598253	74599093	.	-	.	gene_id "XLOC_130792"; transcript_id "Novel_001822"; exon_number "6"; gene_type "
Dt_chr1	Cufflinks	exon	74599484	74599546	.	-	.	gene_id "XLOC_130792"; transcript_id "Novel_001822"; exon_number "7"; gene_type "
Dt_chr1	Cufflinks	exon	74599671	74600436	.	-	.	gene_id "XLOC_130792"; transcript_id "Novel_001822"; exon_number "8"; gene_type "
Dt_chr1	Cufflinks	exon	74594589	74595414	.	-	.	gene_id "XLOC_130792"; transcript_id "Novel_001823"; exon_number "1"; gene_type "
Dt_chr1	Cufflinks	exon	74596821	74596869	.	-	.	gene_id "XLOC_130792"; transcript_id "Novel_001823"; exon_number "2"; gene_type "
Dt_chr1	Cufflinks	exon	74596985	74597038	.	-	.	gene_id "XLOC_130792"; transcript_id "Novel_001823"; exon_number "3"; gene_type "
Dt_chr1	Cufflinks	exon	74597189	74599546	.	-	.	gene_id "XLOC_130792"; transcript_id "Novel_001823"; exon_number "4"; gene_type "
Dt_chr1	Cufflinks	exon	74599671	74600436	.	-	.	gene_id "XLOC_130792"; transcript_id "Novel_001823"; exon_number "5"; gene_type "
Dt_chr1	Cufflinks	exon	74594986	74595414	.	-	.	gene_id "XLOC_130792"; transcript_id "Novel_001824"; exon_number "1"; gene_type "
Dt_chr1	Cufflinks	exon	74596821	74596869	.	-	.	gene_id "XLOC_130792"; transcript_id "Novel_001824"; exon_number "2"; gene_type "
Dt_chr1	Cufflinks	exon	74596985	74597038	.	-	.	gene_id "XLOC_130792"; transcript_id "Novel_001824"; exon_number "3"; gene_type "
Dt_chr1	Cufflinks	exon	74597189	74597248	.	-	.	gene_id "XLOC_130792"; transcript_id "Novel_001824"; exon_number "4"; gene_type "
Dt_chr1	Cufflinks	exon	74597404	74597462	.	-	.	gene_id "XLOC_130792"; transcript_id "Novel_001824"; exon_number "5"; gene_type "
Dt_chr1	Cufflinks	exon	74598253	74598384	.	-	.	gene_id "XLOC_130792"; transcript_id "Novel_001824"; exon_number "6"; gene_type "
Dt_chr1	Cufflinks	exon	74599024	74599093	.	-	.	gene_id "XLOC_130792"; transcript_id "Novel_001824"; exon_number "7"; gene_type "
Dt_chr1	Cufflinks	exon	74599484	74599546	.	-	.	gene_id "XLOC_130792"; transcript_id "Novel_001824"; exon_number "8"; gene_type "
Dt_chr1	Cufflinks	exon	74599671	74600446	.	-	.	gene_id "XLOC_130792"; transcript_id "Novel_001824"; exon_number "9"; gene_type "
Dt_chr1	Cufflinks	exon	74595116	74595414	.	-	.	gene_id "XLOC_130792"; transcript_id "Novel_001825"; exon_number "1"; gene_type "
Dt_chr1	Cufflinks	exon	74596821	74596869	.	-	.	gene_id "XLOC_130792"; transcript_id "Novel_001825"; exon_number "2"; gene_type "
Dt_chr1	Cufflinks	exon	74596985	74597038	.	-	.	gene_id "XLOC_130792"; transcript_id "Novel_001825"; exon_number "3"; gene_type "
Dt_chr1	Cufflinks	exon	74597189	74597248	.	-	.	gene_id "XLOC_130792"; transcript_id "Novel_001825"; exon_number "4"; gene_type "
Dt_chr1	Cufflinks	exon	74597404	74599545	.	-	.	gene_id "XLOC_130792"; transcript_id "Novel_001825"; exon_number "5"; gene_type "
Dt_chr1	Cufflinks	exon	74914197	74916362	.	-	.	gene_id "XLOC_130816"; transcript_id "Novel_001826"; exon_number "1"; gene_type "
Dt_chr1	Cufflinks	exon	74916456	74918062	.	-	.	gene_id "XLOC_130816"; transcript_id "Novel_001826"; exon_number "2"; gene_type "
Dt_chr1	Cufflinks	exon	75523888	75524616	.	-	.	gene_id "XLOC_130881"; transcript_id "Novel_001827"; exon_number "1"; gene_type "
Dt_chr1	Cufflinks	exon	75525419	75527031	.	-	.	gene_id "XLOC_130881"; transcript_id "Novel_001827"; exon_number "2"; gene_type "
Dt_chr1	Cufflinks	exon	75523935	75524452	.	-	.	gene_id "XLOC_130881"; transcript_id "Novel_001828"; exon_number "1"; gene_type "
Dt_chr1	Cufflinks	exon	75524545	75524616	.	-	.	gene_id "XLOC_130881"; transcript_id "Novel_001828"; exon_number "2"; gene_type "
Dt_chr1	Cufflinks	exon	75525419	75527030	.	-	.	gene_id "XLOC_130881"; transcript_id "Novel_001828"; exon_number "3"; gene_type "
Dt_chr1	Cufflinks	exon	75524064	75524452	.	-	.	gene_id "XLOC_130881"; transcript_id "Novel_001829"; exon_number "1"; gene_type "
Dt_chr1	Cufflinks	exon	75524545	75524616	.	-	.	gene_id "XLOC_130881"; transcript_id "Novel_001829"; exon_number "2"; gene_type "
Dt_chr1	Cufflinks	exon	75525419	75525621	.	-	.	gene_id "XLOC_130881"; transcript_id "Novel_001829"; exon_number "3"; gene_type "

Dt_chr1	Cufflinks	exon	75525781	75527030	.	-	.	gene_id "XLOC_130881"; transcript_id "Novel_001829"; exon_number "4"; gene_type "
Dt_chr1	Cufflinks	exon	77594948	77595965	.	-	.	gene_id "XLOC_131073"; transcript_id "Novel_001830"; exon_number "1"; gene_type "
Dt_chr1	Cufflinks	exon	77597037	77597084	.	-	.	gene_id "XLOC_131073"; transcript_id "Novel_001830"; exon_number "2"; gene_type "
Dt_chr1	Cufflinks	exon	77597182	77597224	.	-	.	gene_id "XLOC_131073"; transcript_id "Novel_001830"; exon_number "3"; gene_type "
Dt_chr1	Cufflinks	exon	77597327	77597390	.	-	.	gene_id "XLOC_131073"; transcript_id "Novel_001830"; exon_number "4"; gene_type "
Dt_chr1	Cufflinks	exon	77597524	77597576	.	-	.	gene_id "XLOC_131073"; transcript_id "Novel_001830"; exon_number "5"; gene_type "
Dt_chr1	Cufflinks	exon	77597650	77598023	.	-	.	gene_id "XLOC_131073"; transcript_id "Novel_001830"; exon_number "6"; gene_type "
Dt_chr1	Cufflinks	exon	77597337	77597390	.	-	.	gene_id "XLOC_131073"; transcript_id "Novel_001831"; exon_number "1"; gene_type "
Dt_chr1	Cufflinks	exon	77597524	77597576	.	-	.	gene_id "XLOC_131073"; transcript_id "Novel_001831"; exon_number "2"; gene_type "
Dt_chr1	Cufflinks	exon	77597650	77597771	.	-	.	gene_id "XLOC_131073"; transcript_id "Novel_001831"; exon_number "3"; gene_type "
Dt_chr1	Cufflinks	exon	77597855	77598035	.	-	.	gene_id "XLOC_131073"; transcript_id "Novel_001831"; exon_number "4"; gene_type "
Dt_chr1	Cufflinks	exon	79944876	79945481	.	-	.	gene_id "XLOC_131186"; transcript_id "Novel_001832"; exon_number "1"; gene_type "
Dt_chr1	Cufflinks	exon	79945745	79945903	.	-	.	gene_id "XLOC_131186"; transcript_id "Novel_001832"; exon_number "2"; gene_type "
Dt_chr1	Cufflinks	exon	79945975	79946134	.	-	.	gene_id "XLOC_131186"; transcript_id "Novel_001832"; exon_number "3"; gene_type "
Dt_chr1	Cufflinks	exon	79946221	79946785	.	-	.	gene_id "XLOC_131186"; transcript_id "Novel_001832"; exon_number "4"; gene_type "
Dt_chr1	Cufflinks	exon	79947520	79947710	.	-	.	gene_id "XLOC_131186"; transcript_id "Novel_001832"; exon_number "5"; gene_type "
Dt_chr1	Cufflinks	exon	81106990	81107528	.	-	.	gene_id "XLOC_131286"; transcript_id "Novel_001833"; exon_number "1"; gene_type "
Dt_chr1	Cufflinks	exon	81107664	81108241	.	-	.	gene_id "XLOC_131286"; transcript_id "Novel_001833"; exon_number "2"; gene_type "
Dt_chr1	Cufflinks	exon	85805843	85806167	.	-	.	gene_id "XLOC_131702"; transcript_id "Novel_001834"; exon_number "1"; gene_type "
Dt_chr1	Cufflinks	exon	85807265	85807434	.	-	.	gene_id "XLOC_131702"; transcript_id "Novel_001834"; exon_number "2"; gene_type "
Dt_chr1	Cufflinks	exon	85808163	85808500	.	-	.	gene_id "XLOC_131702"; transcript_id "Novel_001834"; exon_number "3"; gene_type "
Dt_chr1	Cufflinks	exon	91105675	91107356	.	-	.	gene_id "XLOC_131945"; transcript_id "Novel_001835"; exon_number "1"; gene_type "
Dt_chr1	Cufflinks	exon	91109887	91109934	.	-	.	gene_id "XLOC_131945"; transcript_id "Novel_001835"; exon_number "2"; gene_type "
Dt_chr1	Cufflinks	exon	91110071	91110443	.	-	.	gene_id "XLOC_131945"; transcript_id "Novel_001835"; exon_number "3"; gene_type "
Dt_chr1	Cufflinks	exon	94218919	94218998	.	-	.	gene_id "XLOC_132139"; transcript_id "Novel_001836"; exon_number "1"; gene_type "
Dt_chr1	Cufflinks	exon	94236270	94236515	.	-	.	gene_id "XLOC_132139"; transcript_id "Novel_001836"; exon_number "2"; gene_type "
Dt_chr10	Cufflinks	exon	736046	736091	.	+	.	gene_id "XLOC_132370"; transcript_id "Novel_001837"; exon_number "1"; gene_type "
Dt_chr10	Cufflinks	exon	736173	736278	.	+	.	gene_id "XLOC_132370"; transcript_id "Novel_001837"; exon_number "2"; gene_type "
Dt_chr10	Cufflinks	exon	736887	737600	.	+	.	gene_id "XLOC_132370"; transcript_id "Novel_001837"; exon_number "3"; gene_type "
Dt_chr10	Cufflinks	exon	1351547	1351930	.	+	.	gene_id "XLOC_132492"; transcript_id "Novel_001838"; exon_number "1"; gene_type "
Dt_chr10	Cufflinks	exon	1352201	1353474	.	+	.	gene_id "XLOC_132492"; transcript_id "Novel_001838"; exon_number "2"; gene_type "
Dt_chr10	Cufflinks	exon	1353610	1357356	.	+	.	gene_id "XLOC_132492"; transcript_id "Novel_001838"; exon_number "3"; gene_type "
Dt_chr10	Cufflinks	exon	1389905	1390004	.	+	.	gene_id "XLOC_132495"; transcript_id "Novel_001839"; exon_number "1"; gene_type "
Dt_chr10	Cufflinks	exon	1390114	1390318	.	+	.	gene_id "XLOC_132495"; transcript_id "Novel_001839"; exon_number "2"; gene_type "
Dt_chr10	Cufflinks	exon	6692369	6692534	.	+	.	gene_id "XLOC_132895"; transcript_id "Novel_001840"; exon_number "1"; gene_type "

Dt_chr10	Cufflinks	exon	6693550	6693597	.	+	.	gene_id "XLOC_132895"; transcript_id "Novel_001840"; exon_number "2"; gene_type "
Dt_chr10	Cufflinks	exon	6694857	6695797	.	+	.	gene_id "XLOC_132895"; transcript_id "Novel_001840"; exon_number "3"; gene_type "
Dt_chr10	Cufflinks	exon	6776687	6776976	.	+	.	gene_id "XLOC_132904"; transcript_id "Novel_001841"; exon_number "1"; gene_type "
Dt_chr10	Cufflinks	exon	6777074	6778186	.	+	.	gene_id "XLOC_132904"; transcript_id "Novel_001841"; exon_number "2"; gene_type "
Dt_chr10	Cufflinks	exon	6776687	6776976	.	+	.	gene_id "XLOC_132904"; transcript_id "Novel_001842"; exon_number "1"; gene_type "
Dt_chr10	Cufflinks	exon	6777082	6778186	.	+	.	gene_id "XLOC_132904"; transcript_id "Novel_001842"; exon_number "2"; gene_type "
Dt_chr10	Cufflinks	exon	7731227	7731505	.	+	.	gene_id "XLOC_132981"; transcript_id "Novel_001843"; exon_number "1"; gene_type "
Dt_chr10	Cufflinks	exon	7731582	7731991	.	+	.	gene_id "XLOC_132981"; transcript_id "Novel_001843"; exon_number "2"; gene_type "
Dt_chr10	Cufflinks	exon	7732080	7732214	.	+	.	gene_id "XLOC_132981"; transcript_id "Novel_001843"; exon_number "3"; gene_type "
Dt_chr10	Cufflinks	exon	7733028	7733210	.	+	.	gene_id "XLOC_132981"; transcript_id "Novel_001843"; exon_number "4"; gene_type "
Dt_chr10	Cufflinks	exon	7733982	7734143	.	+	.	gene_id "XLOC_132981"; transcript_id "Novel_001843"; exon_number "5"; gene_type "
Dt_chr10	Cufflinks	exon	7734436	7735049	.	+	.	gene_id "XLOC_132981"; transcript_id "Novel_001843"; exon_number "6"; gene_type "
Dt_chr10	Cufflinks	exon	8555018	8555143	.	+	.	gene_id "XLOC_133082"; transcript_id "Novel_001844"; exon_number "1"; gene_type "
Dt_chr10	Cufflinks	exon	8555289	8555538	.	+	.	gene_id "XLOC_133082"; transcript_id "Novel_001844"; exon_number "2"; gene_type "
Dt_chr10	Cufflinks	exon	8555766	8555894	.	+	.	gene_id "XLOC_133082"; transcript_id "Novel_001844"; exon_number "3"; gene_type "
Dt_chr10	Cufflinks	exon	8555984	8556045	.	+	.	gene_id "XLOC_133082"; transcript_id "Novel_001844"; exon_number "4"; gene_type "
Dt_chr10	Cufflinks	exon	8556296	8556470	.	+	.	gene_id "XLOC_133082"; transcript_id "Novel_001844"; exon_number "5"; gene_type "
Dt_chr10	Cufflinks	exon	8813259	8813356	.	+	.	gene_id "XLOC_133116"; transcript_id "Novel_001845"; exon_number "1"; gene_type "
Dt_chr10	Cufflinks	exon	8813656	8813709	.	+	.	gene_id "XLOC_133116"; transcript_id "Novel_001845"; exon_number "2"; gene_type "
Dt_chr10	Cufflinks	exon	8814382	8816892	.	+	.	gene_id "XLOC_133116"; transcript_id "Novel_001845"; exon_number "3"; gene_type "
Dt_chr10	Cufflinks	exon	8888461	8893752	.	+	.	gene_id "XLOC_133139"; transcript_id "Novel_001846"; exon_number "1"; gene_type "
Dt_chr10	Cufflinks	exon	8893926	8894482	.	+	.	gene_id "XLOC_133139"; transcript_id "Novel_001846"; exon_number "2"; gene_type "
Dt_chr10	Cufflinks	exon	8978725	8979193	.	+	.	gene_id "XLOC_133161"; transcript_id "Novel_001847"; exon_number "1"; gene_type "
Dt_chr10	Cufflinks	exon	8979814	8980163	.	+	.	gene_id "XLOC_133161"; transcript_id "Novel_001847"; exon_number "2"; gene_type "
Dt_chr10	Cufflinks	exon	9168814	9168973	.	+	.	gene_id "XLOC_133190"; transcript_id "Novel_001848"; exon_number "1"; gene_type "
Dt_chr10	Cufflinks	exon	9169086	9169165	.	+	.	gene_id "XLOC_133190"; transcript_id "Novel_001848"; exon_number "2"; gene_type "
Dt_chr10	Cufflinks	exon	9903866	9904273	.	+	.	gene_id "XLOC_133262"; transcript_id "Novel_001849"; exon_number "1"; gene_type "
Dt_chr10	Cufflinks	exon	9904366	9905077	.	+	.	gene_id "XLOC_133262"; transcript_id "Novel_001849"; exon_number "2"; gene_type "
Dt_chr10	Cufflinks	exon	9905794	9905834	.	+	.	gene_id "XLOC_133262"; transcript_id "Novel_001849"; exon_number "3"; gene_type "
Dt_chr10	Cufflinks	exon	9907251	9907527	.	+	.	gene_id "XLOC_133262"; transcript_id "Novel_001849"; exon_number "4"; gene_type "
Dt_chr10	Cufflinks	exon	9903920	9904273	.	+	.	gene_id "XLOC_133262"; transcript_id "Novel_001850"; exon_number "1"; gene_type "
Dt_chr10	Cufflinks	exon	9904366	9904647	.	+	.	gene_id "XLOC_133262"; transcript_id "Novel_001850"; exon_number "2"; gene_type "
Dt_chr10	Cufflinks	exon	9904749	9904840	.	+	.	gene_id "XLOC_133262"; transcript_id "Novel_001850"; exon_number "3"; gene_type "
Dt_chr10	Cufflinks	exon	9905034	9905077	.	+	.	gene_id "XLOC_133262"; transcript_id "Novel_001850"; exon_number "4"; gene_type "
Dt_chr10	Cufflinks	exon	9905794	9905834	.	+	.	gene_id "XLOC_133262"; transcript_id "Novel_001850"; exon_number "5"; gene_type "

Dt_chr10	Cufflinks	exon	9907251	9907688	.	+	.	gene_id "XLOC_133262"; transcript_id "Novel_001850"; exon_number "6"; gene_type "
Dt_chr10	Cufflinks	exon	9903920	9904138	.	+	.	gene_id "XLOC_133262"; transcript_id "Novel_001851"; exon_number "1"; gene_type "
Dt_chr10	Cufflinks	exon	9904217	9904273	.	+	.	gene_id "XLOC_133262"; transcript_id "Novel_001851"; exon_number "2"; gene_type "
Dt_chr10	Cufflinks	exon	9904366	9905077	.	+	.	gene_id "XLOC_133262"; transcript_id "Novel_001851"; exon_number "3"; gene_type "
Dt_chr10	Cufflinks	exon	9905794	9905834	.	+	.	gene_id "XLOC_133262"; transcript_id "Novel_001851"; exon_number "4"; gene_type "
Dt_chr10	Cufflinks	exon	9907251	9907688	.	+	.	gene_id "XLOC_133262"; transcript_id "Novel_001851"; exon_number "5"; gene_type "
Dt_chr10	Cufflinks	exon	9903922	9904138	.	+	.	gene_id "XLOC_133262"; transcript_id "Novel_001852"; exon_number "1"; gene_type "
Dt_chr10	Cufflinks	exon	9904217	9904273	.	+	.	gene_id "XLOC_133262"; transcript_id "Novel_001852"; exon_number "2"; gene_type "
Dt_chr10	Cufflinks	exon	9904366	9904647	.	+	.	gene_id "XLOC_133262"; transcript_id "Novel_001852"; exon_number "3"; gene_type "
Dt_chr10	Cufflinks	exon	9904749	9904840	.	+	.	gene_id "XLOC_133262"; transcript_id "Novel_001852"; exon_number "4"; gene_type "
Dt_chr10	Cufflinks	exon	9905034	9905077	.	+	.	gene_id "XLOC_133262"; transcript_id "Novel_001852"; exon_number "5"; gene_type "
Dt_chr10	Cufflinks	exon	9905794	9905834	.	+	.	gene_id "XLOC_133262"; transcript_id "Novel_001852"; exon_number "6"; gene_type "
Dt_chr10	Cufflinks	exon	9907251	9908297	.	+	.	gene_id "XLOC_133262"; transcript_id "Novel_001852"; exon_number "7"; gene_type "
Dt_chr10	Cufflinks	exon	9903935	9904647	.	+	.	gene_id "XLOC_133262"; transcript_id "Novel_001853"; exon_number "1"; gene_type "
Dt_chr10	Cufflinks	exon	9904749	9904840	.	+	.	gene_id "XLOC_133262"; transcript_id "Novel_001853"; exon_number "2"; gene_type "
Dt_chr10	Cufflinks	exon	9905034	9905077	.	+	.	gene_id "XLOC_133262"; transcript_id "Novel_001853"; exon_number "3"; gene_type "
Dt_chr10	Cufflinks	exon	9905794	9905834	.	+	.	gene_id "XLOC_133262"; transcript_id "Novel_001853"; exon_number "4"; gene_type "
Dt_chr10	Cufflinks	exon	9907251	9907563	.	+	.	gene_id "XLOC_133262"; transcript_id "Novel_001853"; exon_number "5"; gene_type "
Dt_chr10	Cufflinks	exon	9903942	9904138	.	+	.	gene_id "XLOC_133262"; transcript_id "Novel_001854"; exon_number "1"; gene_type "
Dt_chr10	Cufflinks	exon	9904217	9904273	.	+	.	gene_id "XLOC_133262"; transcript_id "Novel_001854"; exon_number "2"; gene_type "
Dt_chr10	Cufflinks	exon	9904366	9905308	.	+	.	gene_id "XLOC_133262"; transcript_id "Novel_001854"; exon_number "3"; gene_type "
Dt_chr10	Cufflinks	exon	9903943	9904138	.	+	.	gene_id "XLOC_133262"; transcript_id "Novel_001855"; exon_number "1"; gene_type "
Dt_chr10	Cufflinks	exon	9904217	9904273	.	+	.	gene_id "XLOC_133262"; transcript_id "Novel_001855"; exon_number "2"; gene_type "
Dt_chr10	Cufflinks	exon	9904366	9904677	.	+	.	gene_id "XLOC_133262"; transcript_id "Novel_001855"; exon_number "3"; gene_type "
Dt_chr10	Cufflinks	exon	9904749	9904840	.	+	.	gene_id "XLOC_133262"; transcript_id "Novel_001855"; exon_number "4"; gene_type "
Dt_chr10	Cufflinks	exon	9905034	9905077	.	+	.	gene_id "XLOC_133262"; transcript_id "Novel_001855"; exon_number "5"; gene_type "
Dt_chr10	Cufflinks	exon	9905794	9905834	.	+	.	gene_id "XLOC_133262"; transcript_id "Novel_001855"; exon_number "6"; gene_type "
Dt_chr10	Cufflinks	exon	9907251	9907692	.	+	.	gene_id "XLOC_133262"; transcript_id "Novel_001855"; exon_number "7"; gene_type "
Dt_chr10	Cufflinks	exon	12460209	12460884	.	+	.	gene_id "XLOC_133550"; transcript_id "Novel_001856"; exon_number "1"; gene_type "
Dt_chr10	Cufflinks	exon	12461418	12461640	.	+	.	gene_id "XLOC_133550"; transcript_id "Novel_001856"; exon_number "2"; gene_type "
Dt_chr10	Cufflinks	exon	12461756	12461835	.	+	.	gene_id "XLOC_133550"; transcript_id "Novel_001856"; exon_number "3"; gene_type "
Dt_chr10	Cufflinks	exon	12462920	12463080	.	+	.	gene_id "XLOC_133550"; transcript_id "Novel_001856"; exon_number "4"; gene_type "
Dt_chr10	Cufflinks	exon	12463162	12463715	.	+	.	gene_id "XLOC_133550"; transcript_id "Novel_001856"; exon_number "5"; gene_type "
Dt_chr10	Cufflinks	exon	12460238	12460884	.	+	.	gene_id "XLOC_133550"; transcript_id "Novel_001857"; exon_number "1"; gene_type "
Dt_chr10	Cufflinks	exon	12461418	12461640	.	+	.	gene_id "XLOC_133550"; transcript_id "Novel_001857"; exon_number "2"; gene_type "

Dt_chr10	Cufflinks	exon	12461756	12461835	.	+	.	gene_id "XLOC_133550"; transcript_id "Novel_001857"; exon_number "3"; gene_type "
Dt_chr10	Cufflinks	exon	12462926	12463080	.	+	.	gene_id "XLOC_133550"; transcript_id "Novel_001857"; exon_number "4"; gene_type "
Dt_chr10	Cufflinks	exon	12463162	12463736	.	+	.	gene_id "XLOC_133550"; transcript_id "Novel_001857"; exon_number "5"; gene_type "
Dt_chr10	Cufflinks	exon	12460239	12460874	.	+	.	gene_id "XLOC_133550"; transcript_id "Novel_001858"; exon_number "1"; gene_type "
Dt_chr10	Cufflinks	exon	12461418	12461525	.	+	.	gene_id "XLOC_133550"; transcript_id "Novel_001858"; exon_number "2"; gene_type "
Dt_chr10	Cufflinks	exon	12462097	12463080	.	+	.	gene_id "XLOC_133550"; transcript_id "Novel_001859"; exon_number "1"; gene_type "
Dt_chr10	Cufflinks	exon	12463162	12463228	.	+	.	gene_id "XLOC_133550"; transcript_id "Novel_001859"; exon_number "2"; gene_type "
Dt_chr10	Cufflinks	exon	14640927	14641152	.	+	.	gene_id "XLOC_133701"; transcript_id "Novel_001860"; exon_number "1"; gene_type "
Dt_chr10	Cufflinks	exon	15897325	15897738	.	+	.	gene_id "XLOC_133767"; transcript_id "Novel_001861"; exon_number "1"; gene_type "
Dt_chr10	Cufflinks	exon	15897846	15897943	.	+	.	gene_id "XLOC_133767"; transcript_id "Novel_001861"; exon_number "2"; gene_type "
Dt_chr10	Cufflinks	exon	15898031	15898084	.	+	.	gene_id "XLOC_133767"; transcript_id "Novel_001861"; exon_number "3"; gene_type "
Dt_chr10	Cufflinks	exon	15898707	15898795	.	+	.	gene_id "XLOC_133767"; transcript_id "Novel_001861"; exon_number "4"; gene_type "
Dt_chr10	Cufflinks	exon	15898896	15899372	.	+	.	gene_id "XLOC_133767"; transcript_id "Novel_001861"; exon_number "5"; gene_type "
Dt_chr10	Cufflinks	exon	15897341	15897738	.	+	.	gene_id "XLOC_133767"; transcript_id "Novel_001862"; exon_number "1"; gene_type "
Dt_chr10	Cufflinks	exon	15897846	15897943	.	+	.	gene_id "XLOC_133767"; transcript_id "Novel_001862"; exon_number "2"; gene_type "
Dt_chr10	Cufflinks	exon	15898031	15898084	.	+	.	gene_id "XLOC_133767"; transcript_id "Novel_001862"; exon_number "3"; gene_type "
Dt_chr10	Cufflinks	exon	15898707	15898795	.	+	.	gene_id "XLOC_133767"; transcript_id "Novel_001862"; exon_number "4"; gene_type "
Dt_chr10	Cufflinks	exon	15898896	15899026	.	+	.	gene_id "XLOC_133767"; transcript_id "Novel_001862"; exon_number "5"; gene_type "
Dt_chr10	Cufflinks	exon	15899685	15899711	.	+	.	gene_id "XLOC_133767"; transcript_id "Novel_001862"; exon_number "6"; gene_type "
Dt_chr10	Cufflinks	exon	15899845	15899937	.	+	.	gene_id "XLOC_133767"; transcript_id "Novel_001862"; exon_number "7"; gene_type "
Dt_chr10	Cufflinks	exon	15900124	15900370	.	+	.	gene_id "XLOC_133767"; transcript_id "Novel_001862"; exon_number "8"; gene_type "
Dt_chr10	Cufflinks	exon	15898022	15898084	.	+	.	gene_id "XLOC_133767"; transcript_id "Novel_001863"; exon_number "1"; gene_type "
Dt_chr10	Cufflinks	exon	15898707	15898795	.	+	.	gene_id "XLOC_133767"; transcript_id "Novel_001863"; exon_number "2"; gene_type "
Dt_chr10	Cufflinks	exon	15898896	15899141	.	+	.	gene_id "XLOC_133767"; transcript_id "Novel_001863"; exon_number "3"; gene_type "
Dt_chr10	Cufflinks	exon	18485285	18488416	.	+	.	gene_id "XLOC_133968"; transcript_id "Novel_001864"; exon_number "1"; gene_type "
Dt_chr10	Cufflinks	exon	18488533	18488560	.	+	.	gene_id "XLOC_133968"; transcript_id "Novel_001864"; exon_number "2"; gene_type "
Dt_chr10	Cufflinks	exon	19118826	19119626	.	+	.	gene_id "XLOC_134024"; transcript_id "Novel_001865"; exon_number "1"; gene_type "
Dt_chr10	Cufflinks	exon	19119743	19119920	.	+	.	gene_id "XLOC_134024"; transcript_id "Novel_001865"; exon_number "2"; gene_type "
Dt_chr10	Cufflinks	exon	19120054	19120628	.	+	.	gene_id "XLOC_134024"; transcript_id "Novel_001865"; exon_number "3"; gene_type "
Dt_chr10	Cufflinks	exon	19121033	19121356	.	+	.	gene_id "XLOC_134024"; transcript_id "Novel_001865"; exon_number "4"; gene_type "
Dt_chr10	Cufflinks	exon	19121918	19122049	.	+	.	gene_id "XLOC_134024"; transcript_id "Novel_001865"; exon_number "5"; gene_type "
Dt_chr10	Cufflinks	exon	19123055	19123600	.	+	.	gene_id "XLOC_134024"; transcript_id "Novel_001865"; exon_number "6"; gene_type "
Dt_chr10	Cufflinks	exon	19118854	19119048	.	+	.	gene_id "XLOC_134024"; transcript_id "Novel_001866"; exon_number "1"; gene_type "
Dt_chr10	Cufflinks	exon	19119387	19119626	.	+	.	gene_id "XLOC_134024"; transcript_id "Novel_001866"; exon_number "2"; gene_type "
Dt_chr10	Cufflinks	exon	19119743	19119920	.	+	.	gene_id "XLOC_134024"; transcript_id "Novel_001866"; exon_number "3"; gene_type "

Dt_chr10	Cufflinks	exon	19120054	19120628	.	+	.	gene_id "XLOC_134024"; transcript_id "Novel_001866"; exon_number "4"; gene_type "
Dt_chr10	Cufflinks	exon	19121033	19121354	.	+	.	gene_id "XLOC_134024"; transcript_id "Novel_001866"; exon_number "5"; gene_type "
Dt_chr10	Cufflinks	exon	19188184	19188651	.	+	.	gene_id "XLOC_134034"; transcript_id "Novel_001867"; exon_number "1"; gene_type "
Dt_chr10	Cufflinks	exon	19188750	19188815	.	+	.	gene_id "XLOC_134034"; transcript_id "Novel_001867"; exon_number "2"; gene_type "
Dt_chr10	Cufflinks	exon	19616923	19617182	.	+	.	gene_id "XLOC_134084"; transcript_id "Novel_001868"; exon_number "1"; gene_type "
Dt_chr10	Cufflinks	exon	19620004	19620086	.	+	.	gene_id "XLOC_134084"; transcript_id "Novel_001868"; exon_number "2"; gene_type "
Dt_chr10	Cufflinks	exon	19620785	19622405	.	+	.	gene_id "XLOC_134084"; transcript_id "Novel_001868"; exon_number "3"; gene_type "
Dt_chr10	Cufflinks	exon	19961094	19961434	.	+	.	gene_id "XLOC_134136"; transcript_id "Novel_001869"; exon_number "1"; gene_type "
Dt_chr10	Cufflinks	exon	19961882	19962019	.	+	.	gene_id "XLOC_134136"; transcript_id "Novel_001869"; exon_number "2"; gene_type "
Dt_chr10	Cufflinks	exon	19962107	19962211	.	+	.	gene_id "XLOC_134136"; transcript_id "Novel_001869"; exon_number "3"; gene_type "
Dt_chr10	Cufflinks	exon	20675398	20675621	.	+	.	gene_id "XLOC_134212"; transcript_id "Novel_001870"; exon_number "1"; gene_type "
Dt_chr10	Cufflinks	exon	21705446	21705738	.	+	.	gene_id "XLOC_134275"; transcript_id "Novel_001871"; exon_number "1"; gene_type "
Dt_chr10	Cufflinks	exon	21705880	21706230	.	+	.	gene_id "XLOC_134275"; transcript_id "Novel_001871"; exon_number "2"; gene_type "
Dt_chr10	Cufflinks	exon	22172172	22172634	.	+	.	gene_id "XLOC_134327"; transcript_id "Novel_001872"; exon_number "1"; gene_type "
Dt_chr10	Cufflinks	exon	22173029	22174285	.	+	.	gene_id "XLOC_134327"; transcript_id "Novel_001872"; exon_number "2"; gene_type "
Dt_chr10	Cufflinks	exon	22756094	22756529	.	+	.	gene_id "XLOC_134398"; transcript_id "Novel_001873"; exon_number "1"; gene_type "
Dt_chr10	Cufflinks	exon	22758696	22759128	.	+	.	gene_id "XLOC_134398"; transcript_id "Novel_001873"; exon_number "2"; gene_type "
Dt_chr10	Cufflinks	exon	22756107	22756384	.	+	.	gene_id "XLOC_134398"; transcript_id "Novel_001874"; exon_number "1"; gene_type "
Dt_chr10	Cufflinks	exon	22756477	22756529	.	+	.	gene_id "XLOC_134398"; transcript_id "Novel_001874"; exon_number "2"; gene_type "
Dt_chr10	Cufflinks	exon	22758696	22759105	.	+	.	gene_id "XLOC_134398"; transcript_id "Novel_001874"; exon_number "3"; gene_type "
Dt_chr10	Cufflinks	exon	23302643	23302892	.	+	.	gene_id "XLOC_134454"; transcript_id "Novel_001875"; exon_number "1"; gene_type "
Dt_chr10	Cufflinks	exon	23304301	23306843	.	+	.	gene_id "XLOC_134454"; transcript_id "Novel_001875"; exon_number "2"; gene_type "
Dt_chr10	Cufflinks	exon	25745129	25745366	.	+	.	gene_id "XLOC_134739"; transcript_id "Novel_001876"; exon_number "1"; gene_type "
Dt_chr10	Cufflinks	exon	25745986	25746233	.	+	.	gene_id "XLOC_134739"; transcript_id "Novel_001876"; exon_number "2"; gene_type "
Dt_chr10	Cufflinks	exon	26841524	26841692	.	+	.	gene_id "XLOC_134854"; transcript_id "Novel_001877"; exon_number "1"; gene_type "
Dt_chr10	Cufflinks	exon	26843100	26843147	.	+	.	gene_id "XLOC_134854"; transcript_id "Novel_001877"; exon_number "2"; gene_type "
Dt_chr10	Cufflinks	exon	26844585	26847528	.	+	.	gene_id "XLOC_134854"; transcript_id "Novel_001877"; exon_number "3"; gene_type "
Dt_chr10	Cufflinks	exon	28563247	28563418	.	+	.	gene_id "XLOC_135009"; transcript_id "Novel_001878"; exon_number "1"; gene_type "
Dt_chr10	Cufflinks	exon	28563792	28564311	.	+	.	gene_id "XLOC_135009"; transcript_id "Novel_001878"; exon_number "2"; gene_type "
Dt_chr10	Cufflinks	exon	28564499	28564855	.	+	.	gene_id "XLOC_135009"; transcript_id "Novel_001878"; exon_number "3"; gene_type "
Dt_chr10	Cufflinks	exon	28564952	28565059	.	+	.	gene_id "XLOC_135009"; transcript_id "Novel_001878"; exon_number "4"; gene_type "
Dt_chr10	Cufflinks	exon	28565145	28565285	.	+	.	gene_id "XLOC_135009"; transcript_id "Novel_001878"; exon_number "5"; gene_type "
Dt_chr10	Cufflinks	exon	28565390	28566092	.	+	.	gene_id "XLOC_135009"; transcript_id "Novel_001878"; exon_number "6"; gene_type "
Dt_chr10	Cufflinks	exon	28563254	28563418	.	+	.	gene_id "XLOC_135009"; transcript_id "Novel_001879"; exon_number "1"; gene_type "
Dt_chr10	Cufflinks	exon	28563792	28563957	.	+	.	gene_id "XLOC_135009"; transcript_id "Novel_001879"; exon_number "2"; gene_type "

Dt_chr10	Cufflinks	exon	28564032	28564156	.	+	.	gene_id "XLOC_135009"; transcript_id "Novel_001879"; exon_number "3"; gene_type "
Dt_chr10	Cufflinks	exon	28564247	28564311	.	+	.	gene_id "XLOC_135009"; transcript_id "Novel_001879"; exon_number "4"; gene_type "
Dt_chr10	Cufflinks	exon	28564499	28564660	.	+	.	gene_id "XLOC_135009"; transcript_id "Novel_001879"; exon_number "5"; gene_type "
Dt_chr10	Cufflinks	exon	28564808	28564855	.	+	.	gene_id "XLOC_135009"; transcript_id "Novel_001879"; exon_number "6"; gene_type "
Dt_chr10	Cufflinks	exon	28564952	28565059	.	+	.	gene_id "XLOC_135009"; transcript_id "Novel_001879"; exon_number "7"; gene_type "
Dt_chr10	Cufflinks	exon	28565145	28565285	.	+	.	gene_id "XLOC_135009"; transcript_id "Novel_001879"; exon_number "8"; gene_type "
Dt_chr10	Cufflinks	exon	28565390	28565536	.	+	.	gene_id "XLOC_135009"; transcript_id "Novel_001879"; exon_number "9"; gene_type "
Dt_chr10	Cufflinks	exon	28563254	28563418	.	+	.	gene_id "XLOC_135009"; transcript_id "Novel_001880"; exon_number "1"; gene_type "
Dt_chr10	Cufflinks	exon	28563792	28563957	.	+	.	gene_id "XLOC_135009"; transcript_id "Novel_001880"; exon_number "2"; gene_type "
Dt_chr10	Cufflinks	exon	28564032	28564156	.	+	.	gene_id "XLOC_135009"; transcript_id "Novel_001880"; exon_number "3"; gene_type "
Dt_chr10	Cufflinks	exon	28564247	28564311	.	+	.	gene_id "XLOC_135009"; transcript_id "Novel_001880"; exon_number "4"; gene_type "
Dt_chr10	Cufflinks	exon	28564499	28564711	.	+	.	gene_id "XLOC_135009"; transcript_id "Novel_001880"; exon_number "5"; gene_type "
Dt_chr10	Cufflinks	exon	28564808	28564855	.	+	.	gene_id "XLOC_135009"; transcript_id "Novel_001880"; exon_number "6"; gene_type "
Dt_chr10	Cufflinks	exon	28564952	28565059	.	+	.	gene_id "XLOC_135009"; transcript_id "Novel_001880"; exon_number "7"; gene_type "
Dt_chr10	Cufflinks	exon	28565145	28565285	.	+	.	gene_id "XLOC_135009"; transcript_id "Novel_001880"; exon_number "8"; gene_type "
Dt_chr10	Cufflinks	exon	28565390	28565536	.	+	.	gene_id "XLOC_135009"; transcript_id "Novel_001880"; exon_number "9"; gene_type "
Dt_chr10	Cufflinks	exon	28614095	28614304	.	+	.	gene_id "XLOC_135022"; transcript_id "Novel_001881"; exon_number "1"; gene_type "
Dt_chr10	Cufflinks	exon	28614406	28614482	.	+	.	gene_id "XLOC_135022"; transcript_id "Novel_001881"; exon_number "2"; gene_type "
Dt_chr10	Cufflinks	exon	28614600	28614691	.	+	.	gene_id "XLOC_135022"; transcript_id "Novel_001881"; exon_number "3"; gene_type "
Dt_chr10	Cufflinks	exon	30036325	30036577	.	+	.	gene_id "XLOC_135183"; transcript_id "Novel_001882"; exon_number "1"; gene_type "
Dt_chr10	Cufflinks	exon	30036658	30037098	.	+	.	gene_id "XLOC_135183"; transcript_id "Novel_001882"; exon_number "2"; gene_type "
Dt_chr10	Cufflinks	exon	30155867	30155963	.	+	.	gene_id "XLOC_135201"; transcript_id "Novel_001883"; exon_number "1"; gene_type "
Dt_chr10	Cufflinks	exon	30156057	30156209	.	+	.	gene_id "XLOC_135201"; transcript_id "Novel_001883"; exon_number "2"; gene_type "
Dt_chr10	Cufflinks	exon	30156326	30156979	.	+	.	gene_id "XLOC_135201"; transcript_id "Novel_001883"; exon_number "3"; gene_type "
Dt_chr10	Cufflinks	exon	30249366	30249580	.	+	.	gene_id "XLOC_135211"; transcript_id "Novel_001884"; exon_number "1"; gene_type "
Dt_chr10	Cufflinks	exon	30249666	30250004	.	+	.	gene_id "XLOC_135211"; transcript_id "Novel_001884"; exon_number "2"; gene_type "
Dt_chr10	Cufflinks	exon	30477405	30477797	.	+	.	gene_id "XLOC_135224"; transcript_id "Novel_001885"; exon_number "1"; gene_type "
Dt_chr10	Cufflinks	exon	30477908	30477971	.	+	.	gene_id "XLOC_135224"; transcript_id "Novel_001885"; exon_number "2"; gene_type "
Dt_chr10	Cufflinks	exon	30478428	30479263	.	+	.	gene_id "XLOC_135224"; transcript_id "Novel_001885"; exon_number "3"; gene_type "
Dt_chr10	Cufflinks	exon	30477405	30477609	.	+	.	gene_id "XLOC_135224"; transcript_id "Novel_001886"; exon_number "1"; gene_type "
Dt_chr10	Cufflinks	exon	30477694	30477797	.	+	.	gene_id "XLOC_135224"; transcript_id "Novel_001886"; exon_number "2"; gene_type "
Dt_chr10	Cufflinks	exon	30477908	30479390	.	+	.	gene_id "XLOC_135224"; transcript_id "Novel_001886"; exon_number "3"; gene_type "
Dt_chr10	Cufflinks	exon	30477408	30477609	.	+	.	gene_id "XLOC_135224"; transcript_id "Novel_001887"; exon_number "1"; gene_type "
Dt_chr10	Cufflinks	exon	30477694	30477797	.	+	.	gene_id "XLOC_135224"; transcript_id "Novel_001887"; exon_number "2"; gene_type "
Dt_chr10	Cufflinks	exon	30477908	30477971	.	+	.	gene_id "XLOC_135224"; transcript_id "Novel_001887"; exon_number "3"; gene_type "

Dt_chr10	Cufflinks	exon	30478428	30478523	.	+	.	gene_id "XLOC_135224"; transcript_id "Novel_001887"; exon_number "4"; gene_type "
Dt_chr10	Cufflinks	exon	30478598	30479289	.	+	.	gene_id "XLOC_135224"; transcript_id "Novel_001887"; exon_number "5"; gene_type "
Dt_chr10	Cufflinks	exon	34796281	34796891	.	+	.	gene_id "XLOC_135440"; transcript_id "Novel_001888"; exon_number "1"; gene_type "
Dt_chr10	Cufflinks	exon	34796999	34799443	.	+	.	gene_id "XLOC_135440"; transcript_id "Novel_001888"; exon_number "2"; gene_type "
Dt_chr10	Cufflinks	exon	34800253	34800404	.	+	.	gene_id "XLOC_135440"; transcript_id "Novel_001888"; exon_number "3"; gene_type "
Dt_chr10	Cufflinks	exon	34800566	34800870	.	+	.	gene_id "XLOC_135440"; transcript_id "Novel_001888"; exon_number "4"; gene_type "
Dt_chr10	Cufflinks	exon	35752140	35752479	.	+	.	gene_id "XLOC_135493"; transcript_id "Novel_001889"; exon_number "1"; gene_type "
Dt_chr10	Cufflinks	exon	35753958	35756579	.	+	.	gene_id "XLOC_135493"; transcript_id "Novel_001889"; exon_number "2"; gene_type "
Dt_chr10	Cufflinks	exon	40459780	40459888	.	+	.	gene_id "XLOC_135669"; transcript_id "Novel_001890"; exon_number "1"; gene_type "
Dt_chr10	Cufflinks	exon	40460186	40460337	.	+	.	gene_id "XLOC_135669"; transcript_id "Novel_001890"; exon_number "2"; gene_type "
Dt_chr10	Cufflinks	exon	40460649	40460752	.	+	.	gene_id "XLOC_135669"; transcript_id "Novel_001890"; exon_number "3"; gene_type "
Dt_chr10	Cufflinks	exon	40461152	40461241	.	+	.	gene_id "XLOC_135669"; transcript_id "Novel_001890"; exon_number "4"; gene_type "
Dt_chr10	Cufflinks	exon	40461430	40461501	.	+	.	gene_id "XLOC_135669"; transcript_id "Novel_001890"; exon_number "5"; gene_type "
Dt_chr10	Cufflinks	exon	40461714	40461814	.	+	.	gene_id "XLOC_135669"; transcript_id "Novel_001890"; exon_number "6"; gene_type "
Dt_chr10	Cufflinks	exon	40461923	40462699	.	+	.	gene_id "XLOC_135669"; transcript_id "Novel_001890"; exon_number "7"; gene_type "
Dt_chr10	Cufflinks	exon	41370251	41370306	.	+	.	gene_id "XLOC_135702"; transcript_id "Novel_001891"; exon_number "1"; gene_type "
Dt_chr10	Cufflinks	exon	41370630	41370782	.	+	.	gene_id "XLOC_135702"; transcript_id "Novel_001891"; exon_number "2"; gene_type "
Dt_chr10	Cufflinks	exon	41370868	41370987	.	+	.	gene_id "XLOC_135702"; transcript_id "Novel_001891"; exon_number "3"; gene_type "
Dt_chr10	Cufflinks	exon	49485615	49486187	.	+	.	gene_id "XLOC_136280"; transcript_id "Novel_001892"; exon_number "1"; gene_type "
Dt_chr10	Cufflinks	exon	3940425	3941365	.	-	.	gene_id "XLOC_137102"; transcript_id "Novel_001893"; exon_number "1"; gene_type "
Dt_chr10	Cufflinks	exon	3941909	3943504	.	-	.	gene_id "XLOC_137102"; transcript_id "Novel_001893"; exon_number "2"; gene_type "
Dt_chr10	Cufflinks	exon	7431043	7434013	.	-	.	gene_id "XLOC_137318"; transcript_id "Novel_001894"; exon_number "1"; gene_type "
Dt_chr10	Cufflinks	exon	7436213	7436414	.	-	.	gene_id "XLOC_137318"; transcript_id "Novel_001894"; exon_number "2"; gene_type "
Dt_chr10	Cufflinks	exon	7431540	7431770	.	-	.	gene_id "XLOC_137318"; transcript_id "Novel_001895"; exon_number "1"; gene_type "
Dt_chr10	Cufflinks	exon	7431892	7431947	.	-	.	gene_id "XLOC_137318"; transcript_id "Novel_001895"; exon_number "2"; gene_type "
Dt_chr10	Cufflinks	exon	7432036	7434013	.	-	.	gene_id "XLOC_137318"; transcript_id "Novel_001895"; exon_number "3"; gene_type "
Dt_chr10	Cufflinks	exon	7436213	7436374	.	-	.	gene_id "XLOC_137318"; transcript_id "Novel_001895"; exon_number "4"; gene_type "
Dt_chr10	Cufflinks	exon	8881714	8881932	.	-	.	gene_id "XLOC_137463"; transcript_id "Novel_001896"; exon_number "1"; gene_type "
Dt_chr10	Cufflinks	exon	9131937	9133771	.	-	.	gene_id "XLOC_137517"; transcript_id "Novel_001897"; exon_number "1"; gene_type "
Dt_chr10	Cufflinks	exon	9134044	9134323	.	-	.	gene_id "XLOC_137517"; transcript_id "Novel_001897"; exon_number "2"; gene_type "
Dt_chr10	Cufflinks	exon	10829797	10830666	.	-	.	gene_id "XLOC_137699"; transcript_id "Novel_001898"; exon_number "1"; gene_type "
Dt_chr10	Cufflinks	exon	10830841	10830905	.	-	.	gene_id "XLOC_137699"; transcript_id "Novel_001898"; exon_number "2"; gene_type "
Dt_chr10	Cufflinks	exon	10831197	10831466	.	-	.	gene_id "XLOC_137699"; transcript_id "Novel_001898"; exon_number "3"; gene_type "
Dt_chr10	Cufflinks	exon	12303497	12303995	.	-	.	gene_id "XLOC_137829"; transcript_id "Novel_001899"; exon_number "1"; gene_type "
Dt_chr10	Cufflinks	exon	12304079	12304194	.	-	.	gene_id "XLOC_137829"; transcript_id "Novel_001899"; exon_number "2"; gene_type "

Dt_chr10	Cufflinks	exon	12306573	12306923	.	-	.	gene_id "XLOC_137829"; transcript_id "Novel_001899"; exon_number "3"; gene_type "
Dt_chr10	Cufflinks	exon	13516880	13517441	.	-	.	gene_id "XLOC_137901"; transcript_id "Novel_001900"; exon_number "1"; gene_type "
Dt_chr10	Cufflinks	exon	13517565	13517673	.	-	.	gene_id "XLOC_137901"; transcript_id "Novel_001900"; exon_number "2"; gene_type "
Dt_chr10	Cufflinks	exon	13517752	13517873	.	-	.	gene_id "XLOC_137901"; transcript_id "Novel_001900"; exon_number "3"; gene_type "
Dt_chr10	Cufflinks	exon	13517950	13517991	.	-	.	gene_id "XLOC_137901"; transcript_id "Novel_001900"; exon_number "4"; gene_type "
Dt_chr10	Cufflinks	exon	13519433	13519479	.	-	.	gene_id "XLOC_137901"; transcript_id "Novel_001900"; exon_number "5"; gene_type "
Dt_chr10	Cufflinks	exon	13519563	13519902	.	-	.	gene_id "XLOC_137901"; transcript_id "Novel_001900"; exon_number "6"; gene_type "
Dt_chr10	Cufflinks	exon	13519990	13520368	.	-	.	gene_id "XLOC_137901"; transcript_id "Novel_001900"; exon_number "7"; gene_type "
Dt_chr10	Cufflinks	exon	16456436	16456809	.	-	.	gene_id "XLOC_138150"; transcript_id "Novel_001901"; exon_number "1"; gene_type "
Dt_chr10	Cufflinks	exon	16456888	16459458	.	-	.	gene_id "XLOC_138150"; transcript_id "Novel_001901"; exon_number "2"; gene_type "
Dt_chr10	Cufflinks	exon	17546412	17546781	.	-	.	gene_id "XLOC_138252"; transcript_id "Novel_001902"; exon_number "1"; gene_type "
Dt_chr10	Cufflinks	exon	17549134	17549373	.	-	.	gene_id "XLOC_138252"; transcript_id "Novel_001902"; exon_number "2"; gene_type "
Dt_chr10	Cufflinks	exon	18950609	18951077	.	-	.	gene_id "XLOC_138363"; transcript_id "Novel_001903"; exon_number "1"; gene_type "
Dt_chr10	Cufflinks	exon	18951343	18952416	.	-	.	gene_id "XLOC_138363"; transcript_id "Novel_001903"; exon_number "2"; gene_type "
Dt_chr10	Cufflinks	exon	18950611	18951077	.	-	.	gene_id "XLOC_138363"; transcript_id "Novel_001904"; exon_number "1"; gene_type "
Dt_chr10	Cufflinks	exon	18951343	18951443	.	-	.	gene_id "XLOC_138363"; transcript_id "Novel_001904"; exon_number "2"; gene_type "
Dt_chr10	Cufflinks	exon	18952376	18952525	.	-	.	gene_id "XLOC_138363"; transcript_id "Novel_001904"; exon_number "3"; gene_type "
Dt_chr10	Cufflinks	exon	18952947	18953010	.	-	.	gene_id "XLOC_138363"; transcript_id "Novel_001904"; exon_number "4"; gene_type "
Dt_chr10	Cufflinks	exon	18953108	18953148	.	-	.	gene_id "XLOC_138363"; transcript_id "Novel_001904"; exon_number "5"; gene_type "
Dt_chr10	Cufflinks	exon	18953237	18953699	.	-	.	gene_id "XLOC_138363"; transcript_id "Novel_001904"; exon_number "6"; gene_type "
Dt_chr10	Cufflinks	exon	19875637	19876002	.	-	.	gene_id "XLOC_138440"; transcript_id "Novel_001905"; exon_number "1"; gene_type "
Dt_chr10	Cufflinks	exon	19876089	19876166	.	-	.	gene_id "XLOC_138440"; transcript_id "Novel_001905"; exon_number "2"; gene_type "
Dt_chr10	Cufflinks	exon	19876260	19876439	.	-	.	gene_id "XLOC_138440"; transcript_id "Novel_001905"; exon_number "3"; gene_type "
Dt_chr10	Cufflinks	exon	19876520	19876609	.	-	.	gene_id "XLOC_138440"; transcript_id "Novel_001905"; exon_number "4"; gene_type "
Dt_chr10	Cufflinks	exon	19876897	19877014	.	-	.	gene_id "XLOC_138440"; transcript_id "Novel_001905"; exon_number "5"; gene_type "
Dt_chr10	Cufflinks	exon	19877117	19877173	.	-	.	gene_id "XLOC_138440"; transcript_id "Novel_001905"; exon_number "6"; gene_type "
Dt_chr10	Cufflinks	exon	19877526	19877595	.	-	.	gene_id "XLOC_138440"; transcript_id "Novel_001905"; exon_number "7"; gene_type "
Dt_chr10	Cufflinks	exon	19877711	19877951	.	-	.	gene_id "XLOC_138440"; transcript_id "Novel_001905"; exon_number "8"; gene_type "
Dt_chr10	Cufflinks	exon	21881129	21881334	.	-	.	gene_id "XLOC_138631"; transcript_id "Novel_001906"; exon_number "1"; gene_type "
Dt_chr10	Cufflinks	exon	21881480	21881772	.	-	.	gene_id "XLOC_138631"; transcript_id "Novel_001906"; exon_number "2"; gene_type "
Dt_chr10	Cufflinks	exon	21882139	21882479	.	-	.	gene_id "XLOC_138631"; transcript_id "Novel_001906"; exon_number "3"; gene_type "
Dt_chr10	Cufflinks	exon	22619903	22620488	.	-	.	gene_id "XLOC_138698"; transcript_id "Novel_001907"; exon_number "1"; gene_type "
Dt_chr10	Cufflinks	exon	22620686	22620766	.	-	.	gene_id "XLOC_138698"; transcript_id "Novel_001907"; exon_number "2"; gene_type "
Dt_chr10	Cufflinks	exon	22620893	22620956	.	-	.	gene_id "XLOC_138698"; transcript_id "Novel_001907"; exon_number "3"; gene_type "
Dt_chr10	Cufflinks	exon	22621043	22621106	.	-	.	gene_id "XLOC_138698"; transcript_id "Novel_001907"; exon_number "4"; gene_type "

Dt_chr10	Cufflinks	exon	22621341	22621395	.	-	.	gene_id "XLOC_138698"; transcript_id "Novel_001907"; exon_number "5"; gene_type "
Dt_chr10	Cufflinks	exon	22621840	22621893	.	-	.	gene_id "XLOC_138698"; transcript_id "Novel_001907"; exon_number "6"; gene_type "
Dt_chr10	Cufflinks	exon	22621888	22621933	.	-	.	gene_id "XLOC_138698"; transcript_id "Novel_001908"; exon_number "1"; gene_type "
Dt_chr10	Cufflinks	exon	22622543	22622592	.	-	.	gene_id "XLOC_138698"; transcript_id "Novel_001908"; exon_number "2"; gene_type "
Dt_chr10	Cufflinks	exon	22622676	22622770	.	-	.	gene_id "XLOC_138698"; transcript_id "Novel_001908"; exon_number "3"; gene_type "
Dt_chr10	Cufflinks	exon	22622866	22622940	.	-	.	gene_id "XLOC_138698"; transcript_id "Novel_001908"; exon_number "4"; gene_type "
Dt_chr10	Cufflinks	exon	22623151	22623206	.	-	.	gene_id "XLOC_138698"; transcript_id "Novel_001908"; exon_number "5"; gene_type "
Dt_chr10	Cufflinks	exon	22623341	22623702	.	-	.	gene_id "XLOC_138698"; transcript_id "Novel_001908"; exon_number "6"; gene_type "
Dt_chr10	Cufflinks	exon	22935796	22936043	.	-	.	gene_id "XLOC_138725"; transcript_id "Novel_001909"; exon_number "1"; gene_type "
Dt_chr10	Cufflinks	exon	22936796	22936864	.	-	.	gene_id "XLOC_138725"; transcript_id "Novel_001909"; exon_number "2"; gene_type "
Dt_chr10	Cufflinks	exon	22936957	22937032	.	-	.	gene_id "XLOC_138725"; transcript_id "Novel_001909"; exon_number "3"; gene_type "
Dt_chr10	Cufflinks	exon	22937127	22937175	.	-	.	gene_id "XLOC_138725"; transcript_id "Novel_001909"; exon_number "4"; gene_type "
Dt_chr10	Cufflinks	exon	22937277	22937854	.	-	.	gene_id "XLOC_138725"; transcript_id "Novel_001909"; exon_number "5"; gene_type "
Dt_chr10	Cufflinks	exon	22937948	22937982	.	-	.	gene_id "XLOC_138725"; transcript_id "Novel_001910"; exon_number "1"; gene_type "
Dt_chr10	Cufflinks	exon	22938255	22939259	.	-	.	gene_id "XLOC_138725"; transcript_id "Novel_001910"; exon_number "2"; gene_type "
Dt_chr10	Cufflinks	exon	23137427	23140739	.	-	.	gene_id "XLOC_138748"; transcript_id "Novel_001911"; exon_number "1"; gene_type "
Dt_chr10	Cufflinks	exon	23141731	23142073	.	-	.	gene_id "XLOC_138748"; transcript_id "Novel_001911"; exon_number "2"; gene_type "
Dt_chr10	Cufflinks	exon	23349113	23351317	.	-	.	gene_id "XLOC_138770"; transcript_id "Novel_001912"; exon_number "1"; gene_type "
Dt_chr10	Cufflinks	exon	23352725	23352989	.	-	.	gene_id "XLOC_138770"; transcript_id "Novel_001912"; exon_number "2"; gene_type "
Dt_chr10	Cufflinks	exon	25284053	25285350	.	-	.	gene_id "XLOC_139004"; transcript_id "Novel_001913"; exon_number "1"; gene_type "
Dt_chr10	Cufflinks	exon	25285506	25285819	.	-	.	gene_id "XLOC_139004"; transcript_id "Novel_001913"; exon_number "2"; gene_type "
Dt_chr10	Cufflinks	exon	25621417	25621949	.	-	.	gene_id "XLOC_139025"; transcript_id "Novel_001914"; exon_number "1"; gene_type "
Dt_chr10	Cufflinks	exon	25622233	25622317	.	-	.	gene_id "XLOC_139025"; transcript_id "Novel_001914"; exon_number "2"; gene_type "
Dt_chr10	Cufflinks	exon	25630755	25631871	.	-	.	gene_id "XLOC_139028"; transcript_id "Novel_001915"; exon_number "1"; gene_type "
Dt_chr10	Cufflinks	exon	25632915	25632962	.	-	.	gene_id "XLOC_139028"; transcript_id "Novel_001915"; exon_number "2"; gene_type "
Dt_chr10	Cufflinks	exon	25633092	25633166	.	-	.	gene_id "XLOC_139028"; transcript_id "Novel_001915"; exon_number "3"; gene_type "
Dt_chr10	Cufflinks	exon	25633445	25633682	.	-	.	gene_id "XLOC_139028"; transcript_id "Novel_001915"; exon_number "4"; gene_type "
Dt_chr10	Cufflinks	exon	25631935	25632962	.	-	.	gene_id "XLOC_139028"; transcript_id "Novel_001916"; exon_number "1"; gene_type "
Dt_chr10	Cufflinks	exon	25633092	25633166	.	-	.	gene_id "XLOC_139028"; transcript_id "Novel_001916"; exon_number "2"; gene_type "
Dt_chr10	Cufflinks	exon	25633445	25633682	.	-	.	gene_id "XLOC_139028"; transcript_id "Novel_001916"; exon_number "3"; gene_type "
Dt_chr10	Cufflinks	exon	26502093	26502322	.	-	.	gene_id "XLOC_139094"; transcript_id "Novel_001917"; exon_number "1"; gene_type "
Dt_chr10	Cufflinks	exon	27079701	27080252	.	-	.	gene_id "XLOC_139138"; transcript_id "Novel_001918"; exon_number "1"; gene_type "
Dt_chr10	Cufflinks	exon	27080474	27080535	.	-	.	gene_id "XLOC_139138"; transcript_id "Novel_001918"; exon_number "2"; gene_type "
Dt_chr10	Cufflinks	exon	27080624	27080732	.	-	.	gene_id "XLOC_139138"; transcript_id "Novel_001918"; exon_number "3"; gene_type "
Dt_chr10	Cufflinks	exon	27081046	27081103	.	-	.	gene_id "XLOC_139138"; transcript_id "Novel_001918"; exon_number "4"; gene_type "

Dt_chr10	Cufflinks	exon	27081372	27081449	.	-	.	gene_id "XLOC_139138"; transcript_id "Novel_001918"; exon_number "5"; gene_type "
Dt_chr10	Cufflinks	exon	27082399	27082448	.	-	.	gene_id "XLOC_139138"; transcript_id "Novel_001918"; exon_number "6"; gene_type "
Dt_chr10	Cufflinks	exon	27082638	27082718	.	-	.	gene_id "XLOC_139138"; transcript_id "Novel_001918"; exon_number "7"; gene_type "
Dt_chr10	Cufflinks	exon	27082909	27083107	.	-	.	gene_id "XLOC_139138"; transcript_id "Novel_001918"; exon_number "8"; gene_type "
Dt_chr10	Cufflinks	exon	27080719	27081103	.	-	.	gene_id "XLOC_139138"; transcript_id "Novel_001919"; exon_number "1"; gene_type "
Dt_chr10	Cufflinks	exon	27081372	27081449	.	-	.	gene_id "XLOC_139138"; transcript_id "Novel_001919"; exon_number "2"; gene_type "
Dt_chr10	Cufflinks	exon	27082399	27082448	.	-	.	gene_id "XLOC_139138"; transcript_id "Novel_001919"; exon_number "3"; gene_type "
Dt_chr10	Cufflinks	exon	27082638	27082718	.	-	.	gene_id "XLOC_139138"; transcript_id "Novel_001919"; exon_number "4"; gene_type "
Dt_chr10	Cufflinks	exon	27082909	27083106	.	-	.	gene_id "XLOC_139138"; transcript_id "Novel_001919"; exon_number "5"; gene_type "
Dt_chr10	Cufflinks	exon	27081107	27081449	.	-	.	gene_id "XLOC_139138"; transcript_id "Novel_001920"; exon_number "1"; gene_type "
Dt_chr10	Cufflinks	exon	27082399	27082448	.	-	.	gene_id "XLOC_139138"; transcript_id "Novel_001920"; exon_number "2"; gene_type "
Dt_chr10	Cufflinks	exon	27082638	27082718	.	-	.	gene_id "XLOC_139138"; transcript_id "Novel_001920"; exon_number "3"; gene_type "
Dt_chr10	Cufflinks	exon	27082909	27083107	.	-	.	gene_id "XLOC_139138"; transcript_id "Novel_001920"; exon_number "4"; gene_type "
Dt_chr10	Cufflinks	exon	27392895	27393863	.	-	.	gene_id "XLOC_139173"; transcript_id "Novel_001921"; exon_number "1"; gene_type "
Dt_chr10	Cufflinks	exon	27393974	27394912	.	-	.	gene_id "XLOC_139173"; transcript_id "Novel_001921"; exon_number "2"; gene_type "
Dt_chr10	Cufflinks	exon	27568246	27568576	.	-	.	gene_id "XLOC_139187"; transcript_id "Novel_001922"; exon_number "1"; gene_type "
Dt_chr10	Cufflinks	exon	27568670	27568742	.	-	.	gene_id "XLOC_139187"; transcript_id "Novel_001922"; exon_number "2"; gene_type "
Dt_chr10	Cufflinks	exon	27569407	27569478	.	-	.	gene_id "XLOC_139187"; transcript_id "Novel_001922"; exon_number "3"; gene_type "
Dt_chr10	Cufflinks	exon	27569604	27569672	.	-	.	gene_id "XLOC_139187"; transcript_id "Novel_001922"; exon_number "4"; gene_type "
Dt_chr10	Cufflinks	exon	27569769	27569899	.	-	.	gene_id "XLOC_139187"; transcript_id "Novel_001922"; exon_number "5"; gene_type "
Dt_chr10	Cufflinks	exon	27570283	27570386	.	-	.	gene_id "XLOC_139187"; transcript_id "Novel_001922"; exon_number "6"; gene_type "
Dt_chr10	Cufflinks	exon	27577125	27577339	.	-	.	gene_id "XLOC_139191"; transcript_id "Novel_001923"; exon_number "1"; gene_type "
Dt_chr10	Cufflinks	exon	27577487	27577533	.	-	.	gene_id "XLOC_139191"; transcript_id "Novel_001923"; exon_number "2"; gene_type "
Dt_chr10	Cufflinks	exon	27577917	27578535	.	-	.	gene_id "XLOC_139191"; transcript_id "Novel_001923"; exon_number "3"; gene_type "
Dt_chr10	Cufflinks	exon	27577125	27577339	.	-	.	gene_id "XLOC_139191"; transcript_id "Novel_001924"; exon_number "1"; gene_type "
Dt_chr10	Cufflinks	exon	27577487	27577533	.	-	.	gene_id "XLOC_139191"; transcript_id "Novel_001924"; exon_number "2"; gene_type "
Dt_chr10	Cufflinks	exon	27577917	27577973	.	-	.	gene_id "XLOC_139191"; transcript_id "Novel_001924"; exon_number "3"; gene_type "
Dt_chr10	Cufflinks	exon	27578863	27578918	.	-	.	gene_id "XLOC_139191"; transcript_id "Novel_001924"; exon_number "4"; gene_type "
Dt_chr10	Cufflinks	exon	27577174	27577339	.	-	.	gene_id "XLOC_139191"; transcript_id "Novel_001925"; exon_number "1"; gene_type "
Dt_chr10	Cufflinks	exon	27577487	27577759	.	-	.	gene_id "XLOC_139191"; transcript_id "Novel_001925"; exon_number "2"; gene_type "
Dt_chr10	Cufflinks	exon	27585128	27585480	.	-	.	gene_id "XLOC_139193"; transcript_id "Novel_001926"; exon_number "1"; gene_type "
Dt_chr10	Cufflinks	exon	27585580	27586056	.	-	.	gene_id "XLOC_139193"; transcript_id "Novel_001926"; exon_number "2"; gene_type "
Dt_chr10	Cufflinks	exon	27586108	27586966	.	-	.	gene_id "XLOC_139193"; transcript_id "Novel_001927"; exon_number "1"; gene_type "
Dt_chr10	Cufflinks	exon	27587069	27587116	.	-	.	gene_id "XLOC_139193"; transcript_id "Novel_001927"; exon_number "2"; gene_type "
Dt_chr10	Cufflinks	exon	27587204	27587302	.	-	.	gene_id "XLOC_139193"; transcript_id "Novel_001927"; exon_number "3"; gene_type "

Dt_chr10	Cufflinks	exon	27587414	27587571	.	-	.	gene_id "XLOC_139193"; transcript_id "Novel_001927"; exon_number "4"; gene_type "
Dt_chr10	Cufflinks	exon	27747791	27748332	.	-	.	gene_id "XLOC_139201"; transcript_id "Novel_001928"; exon_number "1"; gene_type "
Dt_chr10	Cufflinks	exon	27748479	27748628	.	-	.	gene_id "XLOC_139201"; transcript_id "Novel_001928"; exon_number "2"; gene_type "
Dt_chr10	Cufflinks	exon	27748784	27748883	.	-	.	gene_id "XLOC_139201"; transcript_id "Novel_001928"; exon_number "3"; gene_type "
Dt_chr10	Cufflinks	exon	27749008	27749330	.	-	.	gene_id "XLOC_139201"; transcript_id "Novel_001928"; exon_number "4"; gene_type "
Dt_chr10	Cufflinks	exon	27749405	27749539	.	-	.	gene_id "XLOC_139201"; transcript_id "Novel_001928"; exon_number "5"; gene_type "
Dt_chr10	Cufflinks	exon	27749650	27749725	.	-	.	gene_id "XLOC_139201"; transcript_id "Novel_001928"; exon_number "6"; gene_type "
Dt_chr10	Cufflinks	exon	27750220	27750393	.	-	.	gene_id "XLOC_139201"; transcript_id "Novel_001929"; exon_number "1"; gene_type "
Dt_chr10	Cufflinks	exon	27750485	27750614	.	-	.	gene_id "XLOC_139201"; transcript_id "Novel_001929"; exon_number "2"; gene_type "
Dt_chr10	Cufflinks	exon	27752274	27752992	.	-	.	gene_id "XLOC_139201"; transcript_id "Novel_001930"; exon_number "1"; gene_type "
Dt_chr10	Cufflinks	exon	27753101	27753365	.	-	.	gene_id "XLOC_139201"; transcript_id "Novel_001930"; exon_number "2"; gene_type "
Dt_chr10	Cufflinks	exon	28672486	28672830	.	-	.	gene_id "XLOC_139271"; transcript_id "Novel_001931"; exon_number "1"; gene_type "
Dt_chr10	Cufflinks	exon	28673166	28673839	.	-	.	gene_id "XLOC_139271"; transcript_id "Novel_001931"; exon_number "2"; gene_type "
Dt_chr10	Cufflinks	exon	28674119	28674297	.	-	.	gene_id "XLOC_139271"; transcript_id "Novel_001931"; exon_number "3"; gene_type "
Dt_chr10	Cufflinks	exon	28674393	28674439	.	-	.	gene_id "XLOC_139271"; transcript_id "Novel_001931"; exon_number "4"; gene_type "
Dt_chr10	Cufflinks	exon	28674537	28674918	.	-	.	gene_id "XLOC_139271"; transcript_id "Novel_001931"; exon_number "5"; gene_type "
Dt_chr10	Cufflinks	exon	29281164	29283550	.	-	.	gene_id "XLOC_139291"; transcript_id "Novel_001932"; exon_number "1"; gene_type "
Dt_chr10	Cufflinks	exon	29283912	29284246	.	-	.	gene_id "XLOC_139291"; transcript_id "Novel_001932"; exon_number "2"; gene_type "
Dt_chr10	Cufflinks	exon	29484036	29484120	.	-	.	gene_id "XLOC_139317"; transcript_id "Novel_001933"; exon_number "1"; gene_type "
Dt_chr10	Cufflinks	exon	29485594	29486536	.	-	.	gene_id "XLOC_139317"; transcript_id "Novel_001933"; exon_number "2"; gene_type "
Dt_chr10	Cufflinks	exon	29486671	29486828	.	-	.	gene_id "XLOC_139319"; transcript_id "Novel_001934"; exon_number "1"; gene_type "
Dt_chr10	Cufflinks	exon	29486920	29487065	.	-	.	gene_id "XLOC_139319"; transcript_id "Novel_001934"; exon_number "2"; gene_type "
Dt_chr10	Cufflinks	exon	29951122	29951458	.	-	.	gene_id "XLOC_139357"; transcript_id "Novel_001935"; exon_number "1"; gene_type "
Dt_chr10	Cufflinks	exon	29952176	29952298	.	-	.	gene_id "XLOC_139357"; transcript_id "Novel_001935"; exon_number "2"; gene_type "
Dt_chr10	Cufflinks	exon	29953636	29953701	.	-	.	gene_id "XLOC_139357"; transcript_id "Novel_001935"; exon_number "3"; gene_type "
Dt_chr10	Cufflinks	exon	29954489	29954558	.	-	.	gene_id "XLOC_139357"; transcript_id "Novel_001935"; exon_number "4"; gene_type "
Dt_chr10	Cufflinks	exon	29954636	29954780	.	-	.	gene_id "XLOC_139357"; transcript_id "Novel_001935"; exon_number "5"; gene_type "
Dt_chr10	Cufflinks	exon	29954857	29954931	.	-	.	gene_id "XLOC_139357"; transcript_id "Novel_001935"; exon_number "6"; gene_type "
Dt_chr10	Cufflinks	exon	29955011	29955113	.	-	.	gene_id "XLOC_139357"; transcript_id "Novel_001935"; exon_number "7"; gene_type "
Dt_chr10	Cufflinks	exon	29955199	29955255	.	-	.	gene_id "XLOC_139357"; transcript_id "Novel_001935"; exon_number "8"; gene_type "
Dt_chr10	Cufflinks	exon	29955358	29956526	.	-	.	gene_id "XLOC_139357"; transcript_id "Novel_001935"; exon_number "9"; gene_type "
Dt_chr10	Cufflinks	exon	30084553	30085457	.	-	.	gene_id "XLOC_139392"; transcript_id "Novel_001936"; exon_number "1"; gene_type "
Dt_chr10	Cufflinks	exon	30085640	30085781	.	-	.	gene_id "XLOC_139392"; transcript_id "Novel_001936"; exon_number "2"; gene_type "
Dt_chr10	Cufflinks	exon	30085858	30085941	.	-	.	gene_id "XLOC_139392"; transcript_id "Novel_001936"; exon_number "3"; gene_type "
Dt_chr10	Cufflinks	exon	30086061	30086118	.	-	.	gene_id "XLOC_139392"; transcript_id "Novel_001936"; exon_number "4"; gene_type "

Dt_chr10	Cufflinks	exon	30087124	30088024	.	-	.	gene_id "XLOC_139392"; transcript_id "Novel_001936"; exon_number "5"; gene_type "
Dt_chr10	Cufflinks	exon	30084554	30085457	.	-	.	gene_id "XLOC_139392"; transcript_id "Novel_001937"; exon_number "1"; gene_type "
Dt_chr10	Cufflinks	exon	30085640	30085781	.	-	.	gene_id "XLOC_139392"; transcript_id "Novel_001937"; exon_number "2"; gene_type "
Dt_chr10	Cufflinks	exon	30085858	30085941	.	-	.	gene_id "XLOC_139392"; transcript_id "Novel_001937"; exon_number "3"; gene_type "
Dt_chr10	Cufflinks	exon	30086061	30086118	.	-	.	gene_id "XLOC_139392"; transcript_id "Novel_001937"; exon_number "4"; gene_type "
Dt_chr10	Cufflinks	exon	30087124	30087925	.	-	.	gene_id "XLOC_139392"; transcript_id "Novel_001937"; exon_number "5"; gene_type "
Dt_chr10	Cufflinks	exon	30088160	30088465	.	-	.	gene_id "XLOC_139392"; transcript_id "Novel_001937"; exon_number "6"; gene_type "
Dt_chr10	Cufflinks	exon	30086590	30087925	.	-	.	gene_id "XLOC_139392"; transcript_id "Novel_001938"; exon_number "1"; gene_type "
Dt_chr10	Cufflinks	exon	30088160	30088322	.	-	.	gene_id "XLOC_139392"; transcript_id "Novel_001938"; exon_number "2"; gene_type "
Dt_chr10	Cufflinks	exon	30184891	30185184	.	-	.	gene_id "XLOC_139397"; transcript_id "Novel_001939"; exon_number "1"; gene_type "
Dt_chr10	Cufflinks	exon	30185659	30185742	.	-	.	gene_id "XLOC_139397"; transcript_id "Novel_001939"; exon_number "2"; gene_type "
Dt_chr10	Cufflinks	exon	30185835	30185879	.	-	.	gene_id "XLOC_139397"; transcript_id "Novel_001939"; exon_number "3"; gene_type "
Dt_chr10	Cufflinks	exon	30675406	30676606	.	-	.	gene_id "XLOC_139424"; transcript_id "Novel_001940"; exon_number "1"; gene_type "
Dt_chr10	Cufflinks	exon	30677696	30677806	.	-	.	gene_id "XLOC_139424"; transcript_id "Novel_001940"; exon_number "2"; gene_type "
Dt_chr10	Cufflinks	exon	30677886	30677957	.	-	.	gene_id "XLOC_139424"; transcript_id "Novel_001940"; exon_number "3"; gene_type "
Dt_chr10	Cufflinks	exon	30678047	30678146	.	-	.	gene_id "XLOC_139424"; transcript_id "Novel_001940"; exon_number "4"; gene_type "
Dt_chr10	Cufflinks	exon	30678295	30678363	.	-	.	gene_id "XLOC_139424"; transcript_id "Novel_001940"; exon_number "5"; gene_type "
Dt_chr10	Cufflinks	exon	30678571	30678611	.	-	.	gene_id "XLOC_139424"; transcript_id "Novel_001940"; exon_number "6"; gene_type "
Dt_chr10	Cufflinks	exon	30678725	30679224	.	-	.	gene_id "XLOC_139424"; transcript_id "Novel_001940"; exon_number "7"; gene_type "
Dt_chr10	Cufflinks	exon	30675406	30676606	.	-	.	gene_id "XLOC_139424"; transcript_id "Novel_001941"; exon_number "1"; gene_type "
Dt_chr10	Cufflinks	exon	30677696	30677806	.	-	.	gene_id "XLOC_139424"; transcript_id "Novel_001941"; exon_number "2"; gene_type "
Dt_chr10	Cufflinks	exon	30677886	30677957	.	-	.	gene_id "XLOC_139424"; transcript_id "Novel_001941"; exon_number "3"; gene_type "
Dt_chr10	Cufflinks	exon	30678047	30678146	.	-	.	gene_id "XLOC_139424"; transcript_id "Novel_001941"; exon_number "4"; gene_type "
Dt_chr10	Cufflinks	exon	30678295	30678363	.	-	.	gene_id "XLOC_139424"; transcript_id "Novel_001941"; exon_number "5"; gene_type "
Dt_chr10	Cufflinks	exon	30678571	30678611	.	-	.	gene_id "XLOC_139424"; transcript_id "Novel_001941"; exon_number "6"; gene_type "
Dt_chr10	Cufflinks	exon	30678725	30678970	.	-	.	gene_id "XLOC_139424"; transcript_id "Novel_001941"; exon_number "7"; gene_type "
Dt_chr10	Cufflinks	exon	30679234	30679476	.	-	.	gene_id "XLOC_139424"; transcript_id "Novel_001941"; exon_number "8"; gene_type "
Dt_chr10	Cufflinks	exon	30680396	30680558	.	-	.	gene_id "XLOC_139424"; transcript_id "Novel_001941"; exon_number "9"; gene_type "
Dt_chr10	Cufflinks	exon	30694444	30695313	.	-	.	gene_id "XLOC_139427"; transcript_id "Novel_001942"; exon_number "1"; gene_type "
Dt_chr10	Cufflinks	exon	30695547	30695613	.	-	.	gene_id "XLOC_139427"; transcript_id "Novel_001942"; exon_number "2"; gene_type "
Dt_chr10	Cufflinks	exon	30695947	30696092	.	-	.	gene_id "XLOC_139427"; transcript_id "Novel_001942"; exon_number "3"; gene_type "
Dt_chr10	Cufflinks	exon	30696260	30696343	.	-	.	gene_id "XLOC_139427"; transcript_id "Novel_001942"; exon_number "4"; gene_type "
Dt_chr10	Cufflinks	exon	30696624	30696720	.	-	.	gene_id "XLOC_139427"; transcript_id "Novel_001942"; exon_number "5"; gene_type "
Dt_chr10	Cufflinks	exon	30696820	30697024	.	-	.	gene_id "XLOC_139427"; transcript_id "Novel_001942"; exon_number "6"; gene_type "
Dt_chr10	Cufflinks	exon	30697155	30697551	.	-	.	gene_id "XLOC_139427"; transcript_id "Novel_001942"; exon_number "7"; gene_type "

Dt_chr10	Cufflinks	exon	30697642	30697881	.	-	.	gene_id "XLOC_139427"; transcript_id "Novel_001942"; exon_number "8"; gene_type "
Dt_chr10	Cufflinks	exon	30697975	30698177	.	-	.	gene_id "XLOC_139427"; transcript_id "Novel_001942"; exon_number "9"; gene_type "
Dt_chr10	Cufflinks	exon	30694444	30695313	.	-	.	gene_id "XLOC_139427"; transcript_id "Novel_001943"; exon_number "1"; gene_type "
Dt_chr10	Cufflinks	exon	30695547	30695613	.	-	.	gene_id "XLOC_139427"; transcript_id "Novel_001943"; exon_number "2"; gene_type "
Dt_chr10	Cufflinks	exon	30695947	30696092	.	-	.	gene_id "XLOC_139427"; transcript_id "Novel_001943"; exon_number "3"; gene_type "
Dt_chr10	Cufflinks	exon	30696260	30696343	.	-	.	gene_id "XLOC_139427"; transcript_id "Novel_001943"; exon_number "4"; gene_type "
Dt_chr10	Cufflinks	exon	30696624	30696720	.	-	.	gene_id "XLOC_139427"; transcript_id "Novel_001943"; exon_number "5"; gene_type "
Dt_chr10	Cufflinks	exon	30696820	30696862	.	-	.	gene_id "XLOC_139427"; transcript_id "Novel_001943"; exon_number "6"; gene_type "
Dt_chr10	Cufflinks	exon	30696954	30697024	.	-	.	gene_id "XLOC_139427"; transcript_id "Novel_001943"; exon_number "7"; gene_type "
Dt_chr10	Cufflinks	exon	30697155	30698215	.	-	.	gene_id "XLOC_139427"; transcript_id "Novel_001943"; exon_number "8"; gene_type "
Dt_chr10	Cufflinks	exon	30694595	30695313	.	-	.	gene_id "XLOC_139427"; transcript_id "Novel_001944"; exon_number "1"; gene_type "
Dt_chr10	Cufflinks	exon	30695547	30695613	.	-	.	gene_id "XLOC_139427"; transcript_id "Novel_001944"; exon_number "2"; gene_type "
Dt_chr10	Cufflinks	exon	30695947	30696092	.	-	.	gene_id "XLOC_139427"; transcript_id "Novel_001944"; exon_number "3"; gene_type "
Dt_chr10	Cufflinks	exon	30696260	30696720	.	-	.	gene_id "XLOC_139427"; transcript_id "Novel_001944"; exon_number "4"; gene_type "
Dt_chr10	Cufflinks	exon	30696820	30697024	.	-	.	gene_id "XLOC_139427"; transcript_id "Novel_001944"; exon_number "5"; gene_type "
Dt_chr10	Cufflinks	exon	30697155	30697881	.	-	.	gene_id "XLOC_139427"; transcript_id "Novel_001944"; exon_number "6"; gene_type "
Dt_chr10	Cufflinks	exon	30697975	30698177	.	-	.	gene_id "XLOC_139427"; transcript_id "Novel_001944"; exon_number "7"; gene_type "
Dt_chr10	Cufflinks	exon	30697104	30697551	.	-	.	gene_id "XLOC_139427"; transcript_id "Novel_001945"; exon_number "1"; gene_type "
Dt_chr10	Cufflinks	exon	30697642	30698196	.	-	.	gene_id "XLOC_139427"; transcript_id "Novel_001945"; exon_number "2"; gene_type "
Dt_chr10	Cufflinks	exon	32584948	32586232	.	-	.	gene_id "XLOC_139549"; transcript_id "Novel_001946"; exon_number "1"; gene_type "
Dt_chr10	Cufflinks	exon	32586327	32586560	.	-	.	gene_id "XLOC_139549"; transcript_id "Novel_001946"; exon_number "2"; gene_type "
Dt_chr10	Cufflinks	exon	32587372	32589054	.	-	.	gene_id "XLOC_139549"; transcript_id "Novel_001946"; exon_number "3"; gene_type "
Dt_chr10	Cufflinks	exon	32584958	32586560	.	-	.	gene_id "XLOC_139549"; transcript_id "Novel_001947"; exon_number "1"; gene_type "
Dt_chr10	Cufflinks	exon	32587372	32589036	.	-	.	gene_id "XLOC_139549"; transcript_id "Novel_001947"; exon_number "2"; gene_type "
Dt_chr10	Cufflinks	exon	32584970	32586560	.	-	.	gene_id "XLOC_139549"; transcript_id "Novel_001948"; exon_number "1"; gene_type "
Dt_chr10	Cufflinks	exon	32587372	32587542	.	-	.	gene_id "XLOC_139549"; transcript_id "Novel_001948"; exon_number "2"; gene_type "
Dt_chr10	Cufflinks	exon	32588420	32589060	.	-	.	gene_id "XLOC_139549"; transcript_id "Novel_001948"; exon_number "3"; gene_type "
Dt_chr10	Cufflinks	exon	43156625	43157354	.	-	.	gene_id "XLOC_140000"; transcript_id "Novel_001949"; exon_number "1"; gene_type "
Dt_chr10	Cufflinks	exon	43157723	43157843	.	-	.	gene_id "XLOC_140000"; transcript_id "Novel_001949"; exon_number "2"; gene_type "
Dt_chr10	Cufflinks	exon	43157965	43158067	.	-	.	gene_id "XLOC_140000"; transcript_id "Novel_001949"; exon_number "3"; gene_type "
Dt_chr10	Cufflinks	exon	43158306	43158493	.	-	.	gene_id "XLOC_140000"; transcript_id "Novel_001949"; exon_number "4"; gene_type "
Dt_chr10	Cufflinks	exon	43157059	43157843	.	-	.	gene_id "XLOC_140000"; transcript_id "Novel_001950"; exon_number "1"; gene_type "
Dt_chr10	Cufflinks	exon	43157965	43158067	.	-	.	gene_id "XLOC_140000"; transcript_id "Novel_001950"; exon_number "2"; gene_type "
Dt_chr10	Cufflinks	exon	43158306	43158493	.	-	.	gene_id "XLOC_140000"; transcript_id "Novel_001950"; exon_number "3"; gene_type "
Dt_chr10	Cufflinks	exon	44072032	44073455	.	-	.	gene_id "XLOC_140034"; transcript_id "Novel_001951"; exon_number "1"; gene_type "

Dt_chr10	Cufflinks	exon	44075254	44075363	.	-	.	gene_id "XLOC_140034"; transcript_id "Novel_001951"; exon_number "2"; gene_type "
Dt_chr10	Cufflinks	exon	44075470	44075828	.	-	.	gene_id "XLOC_140034"; transcript_id "Novel_001951"; exon_number "3"; gene_type "
Dt_chr10	Cufflinks	exon	44076091	44076262	.	-	.	gene_id "XLOC_140034"; transcript_id "Novel_001951"; exon_number "4"; gene_type "
Dt_chr10	Cufflinks	exon	44076539	44076758	.	-	.	gene_id "XLOC_140034"; transcript_id "Novel_001951"; exon_number "5"; gene_type "
Dt_chr10	Cufflinks	exon	44072266	44073455	.	-	.	gene_id "XLOC_140034"; transcript_id "Novel_001952"; exon_number "1"; gene_type "
Dt_chr10	Cufflinks	exon	44075254	44075363	.	-	.	gene_id "XLOC_140034"; transcript_id "Novel_001952"; exon_number "2"; gene_type "
Dt_chr10	Cufflinks	exon	44075470	44076262	.	-	.	gene_id "XLOC_140034"; transcript_id "Novel_001952"; exon_number "3"; gene_type "
Dt_chr10	Cufflinks	exon	44076539	44076719	.	-	.	gene_id "XLOC_140034"; transcript_id "Novel_001952"; exon_number "4"; gene_type "
Dt_chr10	Cufflinks	exon	44072282	44073455	.	-	.	gene_id "XLOC_140034"; transcript_id "Novel_001953"; exon_number "1"; gene_type "
Dt_chr10	Cufflinks	exon	44075244	44075363	.	-	.	gene_id "XLOC_140034"; transcript_id "Novel_001953"; exon_number "2"; gene_type "
Dt_chr10	Cufflinks	exon	44075470	44075828	.	-	.	gene_id "XLOC_140034"; transcript_id "Novel_001953"; exon_number "3"; gene_type "
Dt_chr10	Cufflinks	exon	44076091	44076262	.	-	.	gene_id "XLOC_140034"; transcript_id "Novel_001953"; exon_number "4"; gene_type "
Dt_chr10	Cufflinks	exon	44076539	44076758	.	-	.	gene_id "XLOC_140034"; transcript_id "Novel_001953"; exon_number "5"; gene_type "
Dt_chr10	Cufflinks	exon	45360960	45362063	.	-	.	gene_id "XLOC_140144"; transcript_id "Novel_001954"; exon_number "1"; gene_type "
Dt_chr10	Cufflinks	exon	45362144	45365075	.	-	.	gene_id "XLOC_140144"; transcript_id "Novel_001954"; exon_number "2"; gene_type "
Dt_chr10	Cufflinks	exon	45362513	45363462	.	-	.	gene_id "XLOC_140144"; transcript_id "Novel_001955"; exon_number "1"; gene_type "
Dt_chr10	Cufflinks	exon	45364458	45365006	.	-	.	gene_id "XLOC_140144"; transcript_id "Novel_001955"; exon_number "2"; gene_type "
Dt_chr10	Cufflinks	exon	47013559	47014000	.	-	.	gene_id "XLOC_140329"; transcript_id "Novel_001956"; exon_number "1"; gene_type "
Dt_chr10	Cufflinks	exon	47014964	47015025	.	-	.	gene_id "XLOC_140329"; transcript_id "Novel_001956"; exon_number "2"; gene_type "
Dt_chr10	Cufflinks	exon	47017980	47018075	.	-	.	gene_id "XLOC_140329"; transcript_id "Novel_001956"; exon_number "3"; gene_type "
Dt_chr10	Cufflinks	exon	47013566	47014000	.	-	.	gene_id "XLOC_140329"; transcript_id "Novel_001957"; exon_number "1"; gene_type "
Dt_chr10	Cufflinks	exon	47014964	47015025	.	-	.	gene_id "XLOC_140329"; transcript_id "Novel_001957"; exon_number "2"; gene_type "
Dt_chr10	Cufflinks	exon	47015751	47015795	.	-	.	gene_id "XLOC_140329"; transcript_id "Novel_001957"; exon_number "3"; gene_type "
Dt_chr10	Cufflinks	exon	47017980	47018025	.	-	.	gene_id "XLOC_140329"; transcript_id "Novel_001957"; exon_number "4"; gene_type "
Dt_chr10	Cufflinks	exon	48618680	48618925	.	-	.	gene_id "XLOC_140411"; transcript_id "Novel_001958"; exon_number "1"; gene_type "
Dt_chr10	Cufflinks	exon	56066298	56066776	.	-	.	gene_id "XLOC_140817"; transcript_id "Novel_001959"; exon_number "1"; gene_type "
Dt_chr10	Cufflinks	exon	56067029	56067086	.	-	.	gene_id "XLOC_140817"; transcript_id "Novel_001959"; exon_number "2"; gene_type "
Dt_chr10	Cufflinks	exon	56068109	56068162	.	-	.	gene_id "XLOC_140817"; transcript_id "Novel_001959"; exon_number "3"; gene_type "
Dt_chr10	Cufflinks	exon	56069269	56069472	.	-	.	gene_id "XLOC_140817"; transcript_id "Novel_001959"; exon_number "4"; gene_type "
Dt_chr10	Cufflinks	exon	56070560	56070613	.	-	.	gene_id "XLOC_140817"; transcript_id "Novel_001959"; exon_number "5"; gene_type "
Dt_chr10	Cufflinks	exon	56070933	56071211	.	-	.	gene_id "XLOC_140817"; transcript_id "Novel_001959"; exon_number "6"; gene_type "
Dt_chr10	Cufflinks	exon	56071777	56071841	.	-	.	gene_id "XLOC_140817"; transcript_id "Novel_001959"; exon_number "7"; gene_type "
Dt_chr10	Cufflinks	exon	56071936	56072043	.	-	.	gene_id "XLOC_140817"; transcript_id "Novel_001959"; exon_number "8"; gene_type "
Dt_chr10	Cufflinks	exon	56072740	56072923	.	-	.	gene_id "XLOC_140817"; transcript_id "Novel_001959"; exon_number "9"; gene_type "
Dt_chr10	Cufflinks	exon	56073003	56073194	.	-	.	gene_id "XLOC_140817"; transcript_id "Novel_001959"; exon_number "10"; gene_type

Dt_chr10	Cufflinks	exon	56066368	56066776	.	-	.	gene_id "XLOC_140817"; transcript_id "Novel_001960"; exon_number "1"; gene_type "
Dt_chr10	Cufflinks	exon	56067029	56067086	.	-	.	gene_id "XLOC_140817"; transcript_id "Novel_001960"; exon_number "2"; gene_type "
Dt_chr10	Cufflinks	exon	56068109	56068162	.	-	.	gene_id "XLOC_140817"; transcript_id "Novel_001960"; exon_number "3"; gene_type "
Dt_chr10	Cufflinks	exon	56069269	56069472	.	-	.	gene_id "XLOC_140817"; transcript_id "Novel_001960"; exon_number "4"; gene_type "
Dt_chr10	Cufflinks	exon	56070560	56070613	.	-	.	gene_id "XLOC_140817"; transcript_id "Novel_001960"; exon_number "5"; gene_type "
Dt_chr10	Cufflinks	exon	56070933	56071211	.	-	.	gene_id "XLOC_140817"; transcript_id "Novel_001960"; exon_number "6"; gene_type "
Dt_chr10	Cufflinks	exon	56071777	56071841	.	-	.	gene_id "XLOC_140817"; transcript_id "Novel_001960"; exon_number "7"; gene_type "
Dt_chr10	Cufflinks	exon	56071936	56072043	.	-	.	gene_id "XLOC_140817"; transcript_id "Novel_001960"; exon_number "8"; gene_type "
Dt_chr10	Cufflinks	exon	56072740	56072923	.	-	.	gene_id "XLOC_140817"; transcript_id "Novel_001960"; exon_number "9"; gene_type "
Dt_chr10	Cufflinks	exon	56073003	56073127	.	-	.	gene_id "XLOC_140817"; transcript_id "Novel_001960"; exon_number "10"; gene_type
Dt_chr10	Cufflinks	exon	56073282	56073406	.	-	.	gene_id "XLOC_140817"; transcript_id "Novel_001960"; exon_number "11"; gene_type
Dt_chr10	Cufflinks	exon	56073504	56073969	.	-	.	gene_id "XLOC_140817"; transcript_id "Novel_001960"; exon_number "12"; gene_type
Dt_chr10	Cufflinks	exon	56068838	56070613	.	-	.	gene_id "XLOC_140817"; transcript_id "Novel_001961"; exon_number "1"; gene_type "
Dt_chr10	Cufflinks	exon	56070933	56071211	.	-	.	gene_id "XLOC_140817"; transcript_id "Novel_001961"; exon_number "2"; gene_type "
Dt_chr10	Cufflinks	exon	56071777	56071841	.	-	.	gene_id "XLOC_140817"; transcript_id "Novel_001961"; exon_number "3"; gene_type "
Dt_chr10	Cufflinks	exon	56071936	56072043	.	-	.	gene_id "XLOC_140817"; transcript_id "Novel_001961"; exon_number "4"; gene_type "
Dt_chr10	Cufflinks	exon	56072512	56072579	.	-	.	gene_id "XLOC_140817"; transcript_id "Novel_001961"; exon_number "5"; gene_type "
Dt_chr10	Cufflinks	exon	56073366	56073406	.	-	.	gene_id "XLOC_140817"; transcript_id "Novel_001962"; exon_number "1"; gene_type "
Dt_chr10	Cufflinks	exon	56073504	56073926	.	-	.	gene_id "XLOC_140817"; transcript_id "Novel_001962"; exon_number "2"; gene_type "
Dt_chr10	Cufflinks	exon	56074453	56074708	.	-	.	gene_id "XLOC_140817"; transcript_id "Novel_001962"; exon_number "3"; gene_type "
Dt_chr11	Cufflinks	exon	507093	507219	.	+	.	gene_id "XLOC_141048"; transcript_id "Novel_001963"; exon_number "1"; gene_type "
Dt_chr11	Cufflinks	exon	507877	507968	.	+	.	gene_id "XLOC_141048"; transcript_id "Novel_001963"; exon_number "2"; gene_type "
Dt_chr11	Cufflinks	exon	508366	508577	.	+	.	gene_id "XLOC_141048"; transcript_id "Novel_001963"; exon_number "3"; gene_type "
Dt_chr11	Cufflinks	exon	1023279	1023580	.	+	.	gene_id "XLOC_141129"; transcript_id "Novel_001964"; exon_number "1"; gene_type "
Dt_chr11	Cufflinks	exon	1023773	1023849	.	+	.	gene_id "XLOC_141129"; transcript_id "Novel_001964"; exon_number "2"; gene_type "
Dt_chr11	Cufflinks	exon	1023968	1025238	.	+	.	gene_id "XLOC_141129"; transcript_id "Novel_001964"; exon_number "3"; gene_type "
Dt_chr11	Cufflinks	exon	1374141	1374442	.	+	.	gene_id "XLOC_141188"; transcript_id "Novel_001965"; exon_number "1"; gene_type "
Dt_chr11	Cufflinks	exon	1374822	1374962	.	+	.	gene_id "XLOC_141188"; transcript_id "Novel_001965"; exon_number "2"; gene_type "
Dt_chr11	Cufflinks	exon	1375077	1375140	.	+	.	gene_id "XLOC_141188"; transcript_id "Novel_001965"; exon_number "3"; gene_type "
Dt_chr11	Cufflinks	exon	1375731	1375833	.	+	.	gene_id "XLOC_141188"; transcript_id "Novel_001965"; exon_number "4"; gene_type "
Dt_chr11	Cufflinks	exon	1376116	1376437	.	+	.	gene_id "XLOC_141188"; transcript_id "Novel_001965"; exon_number "5"; gene_type "
Dt_chr11	Cufflinks	exon	1376543	1377024	.	+	.	gene_id "XLOC_141188"; transcript_id "Novel_001965"; exon_number "6"; gene_type "
Dt_chr11	Cufflinks	exon	1374141	1374442	.	+	.	gene_id "XLOC_141188"; transcript_id "Novel_001966"; exon_number "1"; gene_type "
Dt_chr11	Cufflinks	exon	1374822	1374962	.	+	.	gene_id "XLOC_141188"; transcript_id "Novel_001966"; exon_number "2"; gene_type "
Dt_chr11	Cufflinks	exon	1375077	1375140	.	+	.	gene_id "XLOC_141188"; transcript_id "Novel_001966"; exon_number "3"; gene_type "

Dt_chr11	Cufflinks	exon	1375731	1377024	.	+	.	gene_id "XLOC_141188"; transcript_id "Novel_001966"; exon_number "4"; gene_type "
Dt_chr11	Cufflinks	exon	1605275	1605374	.	+	.	gene_id "XLOC_141224"; transcript_id "Novel_001967"; exon_number "1"; gene_type "
Dt_chr11	Cufflinks	exon	1607038	1609206	.	+	.	gene_id "XLOC_141224"; transcript_id "Novel_001967"; exon_number "2"; gene_type "
Dt_chr11	Cufflinks	exon	3832417	3832533	.	+	.	gene_id "XLOC_141515"; transcript_id "Novel_001968"; exon_number "1"; gene_type "
Dt_chr11	Cufflinks	exon	3832636	3832801	.	+	.	gene_id "XLOC_141515"; transcript_id "Novel_001968"; exon_number "2"; gene_type "
Dt_chr11	Cufflinks	exon	5073217	5073289	.	+	.	gene_id "XLOC_141686"; transcript_id "Novel_001969"; exon_number "1"; gene_type "
Dt_chr11	Cufflinks	exon	5074450	5074636	.	+	.	gene_id "XLOC_141686"; transcript_id "Novel_001969"; exon_number "2"; gene_type "
Dt_chr11	Cufflinks	exon	5074730	5074895	.	+	.	gene_id "XLOC_141686"; transcript_id "Novel_001969"; exon_number "3"; gene_type "
Dt_chr11	Cufflinks	exon	5075004	5075041	.	+	.	gene_id "XLOC_141686"; transcript_id "Novel_001969"; exon_number "4"; gene_type "
Dt_chr11	Cufflinks	exon	5075133	5075184	.	+	.	gene_id "XLOC_141686"; transcript_id "Novel_001969"; exon_number "5"; gene_type "
Dt_chr11	Cufflinks	exon	5075299	5075887	.	+	.	gene_id "XLOC_141686"; transcript_id "Novel_001969"; exon_number "6"; gene_type "
Dt_chr11	Cufflinks	exon	6469987	6470144	.	+	.	gene_id "XLOC_141845"; transcript_id "Novel_001970"; exon_number "1"; gene_type "
Dt_chr11	Cufflinks	exon	6470384	6473208	.	+	.	gene_id "XLOC_141845"; transcript_id "Novel_001970"; exon_number "2"; gene_type "
Dt_chr11	Cufflinks	exon	8613111	8613732	.	+	.	gene_id "XLOC_142106"; transcript_id "Novel_001971"; exon_number "1"; gene_type "
Dt_chr11	Cufflinks	exon	8615070	8615121	.	+	.	gene_id "XLOC_142106"; transcript_id "Novel_001971"; exon_number "2"; gene_type "
Dt_chr11	Cufflinks	exon	8615615	8615695	.	+	.	gene_id "XLOC_142106"; transcript_id "Novel_001971"; exon_number "3"; gene_type "
Dt_chr11	Cufflinks	exon	8616095	8616213	.	+	.	gene_id "XLOC_142106"; transcript_id "Novel_001971"; exon_number "4"; gene_type "
Dt_chr11	Cufflinks	exon	8616519	8616738	.	+	.	gene_id "XLOC_142106"; transcript_id "Novel_001971"; exon_number "5"; gene_type "
Dt_chr11	Cufflinks	exon	8616848	8616966	.	+	.	gene_id "XLOC_142106"; transcript_id "Novel_001971"; exon_number "6"; gene_type "
Dt_chr11	Cufflinks	exon	8617088	8617963	.	+	.	gene_id "XLOC_142106"; transcript_id "Novel_001971"; exon_number "7"; gene_type "
Dt_chr11	Cufflinks	exon	11382356	11382437	.	+	.	gene_id "XLOC_142378"; transcript_id "Novel_001972"; exon_number "1"; gene_type "
Dt_chr11	Cufflinks	exon	11382535	11383041	.	+	.	gene_id "XLOC_142378"; transcript_id "Novel_001972"; exon_number "2"; gene_type "
Dt_chr11	Cufflinks	exon	12106025	12106373	.	+	.	gene_id "XLOC_142441"; transcript_id "Novel_001973"; exon_number "1"; gene_type "
Dt_chr11	Cufflinks	exon	12106787	12106941	.	+	.	gene_id "XLOC_142441"; transcript_id "Novel_001973"; exon_number "2"; gene_type "
Dt_chr11	Cufflinks	exon	12110137	12110780	.	+	.	gene_id "XLOC_142441"; transcript_id "Novel_001973"; exon_number "3"; gene_type "
Dt_chr11	Cufflinks	exon	12106025	12106373	.	+	.	gene_id "XLOC_142441"; transcript_id "Novel_001974"; exon_number "1"; gene_type "
Dt_chr11	Cufflinks	exon	12106455	12106537	.	+	.	gene_id "XLOC_142441"; transcript_id "Novel_001974"; exon_number "2"; gene_type "
Dt_chr11	Cufflinks	exon	12106787	12106941	.	+	.	gene_id "XLOC_142441"; transcript_id "Novel_001974"; exon_number "3"; gene_type "
Dt_chr11	Cufflinks	exon	12110137	12110780	.	+	.	gene_id "XLOC_142441"; transcript_id "Novel_001974"; exon_number "4"; gene_type "
Dt_chr11	Cufflinks	exon	16822018	16822243	.	+	.	gene_id "XLOC_142738"; transcript_id "Novel_001975"; exon_number "1"; gene_type "
Dt_chr11	Cufflinks	exon	16824779	16826129	.	+	.	gene_id "XLOC_142738"; transcript_id "Novel_001975"; exon_number "2"; gene_type "
Dt_chr11	Cufflinks	exon	19603284	19604047	.	+	.	gene_id "XLOC_142955"; transcript_id "Novel_001976"; exon_number "1"; gene_type "
Dt_chr11	Cufflinks	exon	19604212	19604389	.	+	.	gene_id "XLOC_142955"; transcript_id "Novel_001976"; exon_number "2"; gene_type "
Dt_chr11	Cufflinks	exon	19605722	19606191	.	+	.	gene_id "XLOC_142955"; transcript_id "Novel_001976"; exon_number "3"; gene_type "
Dt_chr11	Cufflinks	exon	19606632	19606771	.	+	.	gene_id "XLOC_142955"; transcript_id "Novel_001976"; exon_number "4"; gene_type "

Dt_chr11	Cufflinks	exon	19603284	19604047	.	+	.	gene_id "XLOC_142955"; transcript_id "Novel_001977"; exon_number "1"; gene_type "
Dt_chr11	Cufflinks	exon	19604212	19604389	.	+	.	gene_id "XLOC_142955"; transcript_id "Novel_001977"; exon_number "2"; gene_type "
Dt_chr11	Cufflinks	exon	19605722	19606191	.	+	.	gene_id "XLOC_142955"; transcript_id "Novel_001977"; exon_number "3"; gene_type "
Dt_chr11	Cufflinks	exon	19606632	19606760	.	+	.	gene_id "XLOC_142955"; transcript_id "Novel_001977"; exon_number "4"; gene_type "
Dt_chr11	Cufflinks	exon	19607170	19607809	.	+	.	gene_id "XLOC_142955"; transcript_id "Novel_001977"; exon_number "5"; gene_type "
Dt_chr11	Cufflinks	exon	19653547	19654886	.	+	.	gene_id "XLOC_142972"; transcript_id "Novel_001978"; exon_number "1"; gene_type "
Dt_chr11	Cufflinks	exon	19655561	19655702	.	+	.	gene_id "XLOC_142972"; transcript_id "Novel_001978"; exon_number "2"; gene_type "
Dt_chr11	Cufflinks	exon	19655799	19655845	.	+	.	gene_id "XLOC_142972"; transcript_id "Novel_001978"; exon_number "3"; gene_type "
Dt_chr11	Cufflinks	exon	19655969	19656894	.	+	.	gene_id "XLOC_142972"; transcript_id "Novel_001978"; exon_number "4"; gene_type "
Dt_chr11	Cufflinks	exon	19654792	19654886	.	+	.	gene_id "XLOC_142972"; transcript_id "Novel_001979"; exon_number "1"; gene_type "
Dt_chr11	Cufflinks	exon	19655561	19656444	.	+	.	gene_id "XLOC_142972"; transcript_id "Novel_001979"; exon_number "2"; gene_type "
Dt_chr11	Cufflinks	exon	19654935	19655702	.	+	.	gene_id "XLOC_142972"; transcript_id "Novel_001980"; exon_number "1"; gene_type "
Dt_chr11	Cufflinks	exon	19655799	19655827	.	+	.	gene_id "XLOC_142972"; transcript_id "Novel_001980"; exon_number "2"; gene_type "
Dt_chr11	Cufflinks	exon	26468722	26468922	.	+	.	gene_id "XLOC_143594"; transcript_id "Novel_001981"; exon_number "1"; gene_type "
Dt_chr11	Cufflinks	exon	26469085	26469368	.	+	.	gene_id "XLOC_143594"; transcript_id "Novel_001981"; exon_number "2"; gene_type "
Dt_chr11	Cufflinks	exon	26470404	26472534	.	+	.	gene_id "XLOC_143594"; transcript_id "Novel_001981"; exon_number "3"; gene_type "
Dt_chr11	Cufflinks	exon	26468742	26469368	.	+	.	gene_id "XLOC_143594"; transcript_id "Novel_001982"; exon_number "1"; gene_type "
Dt_chr11	Cufflinks	exon	26470404	26472531	.	+	.	gene_id "XLOC_143594"; transcript_id "Novel_001982"; exon_number "2"; gene_type "
Dt_chr11	Cufflinks	exon	28185663	28185830	.	+	.	gene_id "XLOC_143693"; transcript_id "Novel_001983"; exon_number "1"; gene_type "
Dt_chr11	Cufflinks	exon	28186596	28186867	.	+	.	gene_id "XLOC_143693"; transcript_id "Novel_001983"; exon_number "2"; gene_type "
Dt_chr11	Cufflinks	exon	30289178	30289782	.	+	.	gene_id "XLOC_143846"; transcript_id "Novel_001984"; exon_number "1"; gene_type "
Dt_chr11	Cufflinks	exon	30291209	30293468	.	+	.	gene_id "XLOC_143846"; transcript_id "Novel_001984"; exon_number "2"; gene_type "
Dt_chr11	Cufflinks	exon	30289325	30289782	.	+	.	gene_id "XLOC_143846"; transcript_id "Novel_001985"; exon_number "1"; gene_type "
Dt_chr11	Cufflinks	exon	30291209	30291256	.	+	.	gene_id "XLOC_143846"; transcript_id "Novel_001985"; exon_number "2"; gene_type "
Dt_chr11	Cufflinks	exon	30291427	30293536	.	+	.	gene_id "XLOC_143846"; transcript_id "Novel_001985"; exon_number "3"; gene_type "
Dt_chr11	Cufflinks	exon	31515761	31516157	.	+	.	gene_id "XLOC_143903"; transcript_id "Novel_001986"; exon_number "1"; gene_type "
Dt_chr11	Cufflinks	exon	31516233	31516554	.	+	.	gene_id "XLOC_143903"; transcript_id "Novel_001986"; exon_number "2"; gene_type "
Dt_chr11	Cufflinks	exon	31517772	31518371	.	+	.	gene_id "XLOC_143903"; transcript_id "Novel_001987"; exon_number "1"; gene_type "
Dt_chr11	Cufflinks	exon	31518458	31518498	.	+	.	gene_id "XLOC_143903"; transcript_id "Novel_001987"; exon_number "2"; gene_type "
Dt_chr11	Cufflinks	exon	31518611	31518712	.	+	.	gene_id "XLOC_143903"; transcript_id "Novel_001988"; exon_number "1"; gene_type "
Dt_chr11	Cufflinks	exon	31518826	31518937	.	+	.	gene_id "XLOC_143903"; transcript_id "Novel_001988"; exon_number "2"; gene_type "
Dt_chr11	Cufflinks	exon	31518619	31519412	.	+	.	gene_id "XLOC_143903"; transcript_id "Novel_001989"; exon_number "1"; gene_type "
Dt_chr11	Cufflinks	exon	31520004	31520593	.	+	.	gene_id "XLOC_143903"; transcript_id "Novel_001989"; exon_number "2"; gene_type "
Dt_chr11	Cufflinks	exon	31555839	31555949	.	+	.	gene_id "XLOC_143905"; transcript_id "Novel_001990"; exon_number "1"; gene_type "
Dt_chr11	Cufflinks	exon	31556171	31556329	.	+	.	gene_id "XLOC_143905"; transcript_id "Novel_001990"; exon_number "2"; gene_type "

Dt_chr11	Cufflinks	exon	31556426	31556574	.	+	.	gene_id "XLOC_143905"; transcript_id "Novel_001990"; exon_number "3"; gene_type "
Dt_chr11	Cufflinks	exon	32161273	32161530	.	+	.	gene_id "XLOC_143940"; transcript_id "Novel_001991"; exon_number "1"; gene_type "
Dt_chr11	Cufflinks	exon	32163025	32163084	.	+	.	gene_id "XLOC_143940"; transcript_id "Novel_001991"; exon_number "2"; gene_type "
Dt_chr11	Cufflinks	exon	32163402	32163451	.	+	.	gene_id "XLOC_143940"; transcript_id "Novel_001991"; exon_number "3"; gene_type "
Dt_chr11	Cufflinks	exon	32163547	32163574	.	+	.	gene_id "XLOC_143940"; transcript_id "Novel_001991"; exon_number "4"; gene_type "
Dt_chr11	Cufflinks	exon	32163962	32164192	.	+	.	gene_id "XLOC_143940"; transcript_id "Novel_001991"; exon_number "5"; gene_type "
Dt_chr11	Cufflinks	exon	32164300	32164376	.	+	.	gene_id "XLOC_143940"; transcript_id "Novel_001991"; exon_number "6"; gene_type "
Dt_chr11	Cufflinks	exon	32164591	32164740	.	+	.	gene_id "XLOC_143940"; transcript_id "Novel_001991"; exon_number "7"; gene_type "
Dt_chr11	Cufflinks	exon	32164853	32165256	.	+	.	gene_id "XLOC_143940"; transcript_id "Novel_001991"; exon_number "8"; gene_type "
Dt_chr11	Cufflinks	exon	32162143	32164192	.	+	.	gene_id "XLOC_143940"; transcript_id "Novel_001992"; exon_number "1"; gene_type "
Dt_chr11	Cufflinks	exon	32164300	32164376	.	+	.	gene_id "XLOC_143940"; transcript_id "Novel_001992"; exon_number "2"; gene_type "
Dt_chr11	Cufflinks	exon	32164591	32164740	.	+	.	gene_id "XLOC_143940"; transcript_id "Novel_001992"; exon_number "3"; gene_type "
Dt_chr11	Cufflinks	exon	32164853	32165249	.	+	.	gene_id "XLOC_143940"; transcript_id "Novel_001992"; exon_number "4"; gene_type "
Dt_chr11	Cufflinks	exon	35825769	35825927	.	+	.	gene_id "XLOC_144120"; transcript_id "Novel_001993"; exon_number "1"; gene_type "
Dt_chr11	Cufflinks	exon	35826146	35828573	.	+	.	gene_id "XLOC_144120"; transcript_id "Novel_001993"; exon_number "2"; gene_type "
Dt_chr11	Cufflinks	exon	35825826	35825927	.	+	.	gene_id "XLOC_144120"; transcript_id "Novel_001994"; exon_number "1"; gene_type "
Dt_chr11	Cufflinks	exon	35826112	35828628	.	+	.	gene_id "XLOC_144120"; transcript_id "Novel_001994"; exon_number "2"; gene_type "
Dt_chr11	Cufflinks	exon	35825944	35826275	.	+	.	gene_id "XLOC_144120"; transcript_id "Novel_001995"; exon_number "1"; gene_type "
Dt_chr11	Cufflinks	exon	35827376	35828628	.	+	.	gene_id "XLOC_144120"; transcript_id "Novel_001995"; exon_number "2"; gene_type "
Dt_chr11	Cufflinks	exon	39281204	39281518	.	+	.	gene_id "XLOC_144309"; transcript_id "Novel_001996"; exon_number "1"; gene_type "
Dt_chr11	Cufflinks	exon	39281631	39281707	.	+	.	gene_id "XLOC_144309"; transcript_id "Novel_001996"; exon_number "2"; gene_type "
Dt_chr11	Cufflinks	exon	39282547	39283626	.	+	.	gene_id "XLOC_144309"; transcript_id "Novel_001996"; exon_number "3"; gene_type "
Dt_chr11	Cufflinks	exon	39281548	39281707	.	+	.	gene_id "XLOC_144309"; transcript_id "Novel_001997"; exon_number "1"; gene_type "
Dt_chr11	Cufflinks	exon	39282547	39283626	.	+	.	gene_id "XLOC_144309"; transcript_id "Novel_001997"; exon_number "2"; gene_type "
Dt_chr11	Cufflinks	exon	39283942	39284500	.	+	.	gene_id "XLOC_144309"; transcript_id "Novel_001998"; exon_number "1"; gene_type "
Dt_chr11	Cufflinks	exon	39284652	39284909	.	+	.	gene_id "XLOC_144309"; transcript_id "Novel_001998"; exon_number "2"; gene_type "
Dt_chr11	Cufflinks	exon	42734367	42734765	.	+	.	gene_id "XLOC_144432"; transcript_id "Novel_001999"; exon_number "1"; gene_type "
Dt_chr11	Cufflinks	exon	42734878	42734914	.	+	.	gene_id "XLOC_144432"; transcript_id "Novel_001999"; exon_number "2"; gene_type "
Dt_chr11	Cufflinks	exon	42735015	42735136	.	+	.	gene_id "XLOC_144432"; transcript_id "Novel_001999"; exon_number "3"; gene_type "
Dt_chr11	Cufflinks	exon	42735251	42735974	.	+	.	gene_id "XLOC_144432"; transcript_id "Novel_001999"; exon_number "4"; gene_type "
Dt_chr11	Cufflinks	exon	42734367	42734765	.	+	.	gene_id "XLOC_144432"; transcript_id "Novel_002000"; exon_number "1"; gene_type "
Dt_chr11	Cufflinks	exon	42734878	42734933	.	+	.	gene_id "XLOC_144432"; transcript_id "Novel_002000"; exon_number "2"; gene_type "
Dt_chr11	Cufflinks	exon	42735015	42735136	.	+	.	gene_id "XLOC_144432"; transcript_id "Novel_002000"; exon_number "3"; gene_type "
Dt_chr11	Cufflinks	exon	42735251	42735974	.	+	.	gene_id "XLOC_144432"; transcript_id "Novel_002000"; exon_number "4"; gene_type "
Dt_chr11	Cufflinks	exon	42734412	42734765	.	+	.	gene_id "XLOC_144432"; transcript_id "Novel_002001"; exon_number "1"; gene_type "

Dt_chr11	Cufflinks	exon	42734878	42734933	.	+	.	gene_id "XLOC_144432"; transcript_id "Novel_002001"; exon_number "2"; gene_type "
Dt_chr11	Cufflinks	exon	42735015	42735895	.	+	.	gene_id "XLOC_144432"; transcript_id "Novel_002001"; exon_number "3"; gene_type "
Dt_chr11	Cufflinks	exon	43167137	43167643	.	+	.	gene_id "XLOC_144453"; transcript_id "Novel_002002"; exon_number "1"; gene_type "
Dt_chr11	Cufflinks	exon	43167791	43167904	.	+	.	gene_id "XLOC_144453"; transcript_id "Novel_002002"; exon_number "2"; gene_type "
Dt_chr11	Cufflinks	exon	43169461	43169500	.	+	.	gene_id "XLOC_144453"; transcript_id "Novel_002003"; exon_number "1"; gene_type "
Dt_chr11	Cufflinks	exon	43169621	43169698	.	+	.	gene_id "XLOC_144453"; transcript_id "Novel_002003"; exon_number "2"; gene_type "
Dt_chr11	Cufflinks	exon	43170152	43170268	.	+	.	gene_id "XLOC_144453"; transcript_id "Novel_002003"; exon_number "3"; gene_type "
Dt_chr11	Cufflinks	exon	43170521	43170916	.	+	.	gene_id "XLOC_144453"; transcript_id "Novel_002003"; exon_number "4"; gene_type "
Dt_chr11	Cufflinks	exon	43169668	43169698	.	+	.	gene_id "XLOC_144453"; transcript_id "Novel_002004"; exon_number "1"; gene_type "
Dt_chr11	Cufflinks	exon	43170104	43170268	.	+	.	gene_id "XLOC_144453"; transcript_id "Novel_002004"; exon_number "2"; gene_type "
Dt_chr11	Cufflinks	exon	43170521	43170923	.	+	.	gene_id "XLOC_144453"; transcript_id "Novel_002004"; exon_number "3"; gene_type "
Dt_chr11	Cufflinks	exon	43369111	43369223	.	+	.	gene_id "XLOC_144458"; transcript_id "Novel_002005"; exon_number "1"; gene_type "
Dt_chr11	Cufflinks	exon	43369300	43369648	.	+	.	gene_id "XLOC_144458"; transcript_id "Novel_002005"; exon_number "2"; gene_type "
Dt_chr11	Cufflinks	exon	43370887	43370943	.	+	.	gene_id "XLOC_144458"; transcript_id "Novel_002005"; exon_number "3"; gene_type "
Dt_chr11	Cufflinks	exon	43371198	43372729	.	+	.	gene_id "XLOC_144458"; transcript_id "Novel_002005"; exon_number "4"; gene_type "
Dt_chr11	Cufflinks	exon	43372850	43372978	.	+	.	gene_id "XLOC_144458"; transcript_id "Novel_002005"; exon_number "5"; gene_type "
Dt_chr11	Cufflinks	exon	43373134	43373313	.	+	.	gene_id "XLOC_144458"; transcript_id "Novel_002005"; exon_number "6"; gene_type "
Dt_chr11	Cufflinks	exon	43373578	43373705	.	+	.	gene_id "XLOC_144458"; transcript_id "Novel_002005"; exon_number "7"; gene_type "
Dt_chr11	Cufflinks	exon	43374965	43374996	.	+	.	gene_id "XLOC_144458"; transcript_id "Novel_002006"; exon_number "1"; gene_type "
Dt_chr11	Cufflinks	exon	43375217	43375346	.	+	.	gene_id "XLOC_144458"; transcript_id "Novel_002006"; exon_number "2"; gene_type "
Dt_chr11	Cufflinks	exon	43375443	43375571	.	+	.	gene_id "XLOC_144458"; transcript_id "Novel_002006"; exon_number "3"; gene_type "
Dt_chr11	Cufflinks	exon	43375664	43375801	.	+	.	gene_id "XLOC_144458"; transcript_id "Novel_002006"; exon_number "4"; gene_type "
Dt_chr11	Cufflinks	exon	43375905	43375982	.	+	.	gene_id "XLOC_144458"; transcript_id "Novel_002006"; exon_number "5"; gene_type "
Dt_chr11	Cufflinks	exon	43376093	43376184	.	+	.	gene_id "XLOC_144458"; transcript_id "Novel_002006"; exon_number "6"; gene_type "
Dt_chr11	Cufflinks	exon	43376441	43376827	.	+	.	gene_id "XLOC_144458"; transcript_id "Novel_002006"; exon_number "7"; gene_type "
Dt_chr11	Cufflinks	exon	44535942	44536148	.	+	.	gene_id "XLOC_144542"; transcript_id "Novel_002007"; exon_number "1"; gene_type "
Dt_chr11	Cufflinks	exon	44536446	44537520	.	+	.	gene_id "XLOC_144542"; transcript_id "Novel_002007"; exon_number "2"; gene_type "
Dt_chr11	Cufflinks	exon	45572035	45573501	.	+	.	gene_id "XLOC_144613"; transcript_id "Novel_002008"; exon_number "1"; gene_type "
Dt_chr11	Cufflinks	exon	45573711	45574413	.	+	.	gene_id "XLOC_144613"; transcript_id "Novel_002008"; exon_number "2"; gene_type "
Dt_chr11	Cufflinks	exon	46997714	46997832	.	+	.	gene_id "XLOC_144730"; transcript_id "Novel_002009"; exon_number "1"; gene_type "
Dt_chr11	Cufflinks	exon	46997926	46998019	.	+	.	gene_id "XLOC_144730"; transcript_id "Novel_002009"; exon_number "2"; gene_type "
Dt_chr11	Cufflinks	exon	46999381	46999514	.	+	.	gene_id "XLOC_144730"; transcript_id "Novel_002009"; exon_number "3"; gene_type "
Dt_chr11	Cufflinks	exon	46999609	47000270	.	+	.	gene_id "XLOC_144730"; transcript_id "Novel_002009"; exon_number "4"; gene_type "
Dt_chr11	Cufflinks	exon	48010591	48010640	.	+	.	gene_id "XLOC_144864"; transcript_id "Novel_002010"; exon_number "1"; gene_type "
Dt_chr11	Cufflinks	exon	48010724	48011372	.	+	.	gene_id "XLOC_144864"; transcript_id "Novel_002010"; exon_number "2"; gene_type "

Dt_chr11	Cufflinks	exon	51042633	51043043	.	+	.	gene_id "XLOC_145108"; transcript_id "Novel_002011"; exon_number "1"; gene_type "
Dt_chr11	Cufflinks	exon	51043154	51043291	.	+	.	gene_id "XLOC_145108"; transcript_id "Novel_002011"; exon_number "2"; gene_type "
Dt_chr11	Cufflinks	exon	51043367	51044382	.	+	.	gene_id "XLOC_145108"; transcript_id "Novel_002011"; exon_number "3"; gene_type "
Dt_chr11	Cufflinks	exon	51042641	51043043	.	+	.	gene_id "XLOC_145108"; transcript_id "Novel_002012"; exon_number "1"; gene_type "
Dt_chr11	Cufflinks	exon	51043154	51043291	.	+	.	gene_id "XLOC_145108"; transcript_id "Novel_002012"; exon_number "2"; gene_type "
Dt_chr11	Cufflinks	exon	51043367	51043424	.	+	.	gene_id "XLOC_145108"; transcript_id "Novel_002012"; exon_number "3"; gene_type "
Dt_chr11	Cufflinks	exon	51043570	51043832	.	+	.	gene_id "XLOC_145108"; transcript_id "Novel_002012"; exon_number "4"; gene_type "
Dt_chr11	Cufflinks	exon	51043914	51044780	.	+	.	gene_id "XLOC_145108"; transcript_id "Novel_002012"; exon_number "5"; gene_type "
Dt_chr11	Cufflinks	exon	54643871	54643989	.	+	.	gene_id "XLOC_145357"; transcript_id "Novel_002013"; exon_number "1"; gene_type "
Dt_chr11	Cufflinks	exon	54644369	54644795	.	+	.	gene_id "XLOC_145357"; transcript_id "Novel_002013"; exon_number "2"; gene_type "
Dt_chr11	Cufflinks	exon	54644893	54646466	.	+	.	gene_id "XLOC_145357"; transcript_id "Novel_002013"; exon_number "3"; gene_type "
Dt_chr11	Cufflinks	exon	54646677	54646777	.	+	.	gene_id "XLOC_145357"; transcript_id "Novel_002013"; exon_number "4"; gene_type "
Dt_chr11	Cufflinks	exon	54646940	54647473	.	+	.	gene_id "XLOC_145357"; transcript_id "Novel_002013"; exon_number "5"; gene_type "
Dt_chr11	Cufflinks	exon	54647596	54647645	.	+	.	gene_id "XLOC_145357"; transcript_id "Novel_002013"; exon_number "6"; gene_type "
Dt_chr11	Cufflinks	exon	54647767	54647848	.	+	.	gene_id "XLOC_145357"; transcript_id "Novel_002013"; exon_number "7"; gene_type "
Dt_chr11	Cufflinks	exon	54648020	54648114	.	+	.	gene_id "XLOC_145357"; transcript_id "Novel_002013"; exon_number "8"; gene_type "
Dt_chr11	Cufflinks	exon	54648557	54649440	.	+	.	gene_id "XLOC_145357"; transcript_id "Novel_002013"; exon_number "9"; gene_type "
Dt_chr11	Cufflinks	exon	54748398	54748535	.	+	.	gene_id "XLOC_145366"; transcript_id "Novel_002014"; exon_number "1"; gene_type "
Dt_chr11	Cufflinks	exon	54748659	54749240	.	+	.	gene_id "XLOC_145366"; transcript_id "Novel_002014"; exon_number "2"; gene_type "
Dt_chr11	Cufflinks	exon	54749854	54749933	.	+	.	gene_id "XLOC_145366"; transcript_id "Novel_002014"; exon_number "3"; gene_type "
Dt_chr11	Cufflinks	exon	54750035	54750341	.	+	.	gene_id "XLOC_145366"; transcript_id "Novel_002014"; exon_number "4"; gene_type "
Dt_chr11	Cufflinks	exon	56362463	56362754	.	+	.	gene_id "XLOC_145439"; transcript_id "Novel_002015"; exon_number "1"; gene_type "
Dt_chr11	Cufflinks	exon	56363362	56364620	.	+	.	gene_id "XLOC_145439"; transcript_id "Novel_002015"; exon_number "2"; gene_type "
Dt_chr11	Cufflinks	exon	56362491	56363126	.	+	.	gene_id "XLOC_145439"; transcript_id "Novel_002016"; exon_number "1"; gene_type "
Dt_chr11	Cufflinks	exon	56363362	56363498	.	+	.	gene_id "XLOC_145439"; transcript_id "Novel_002016"; exon_number "2"; gene_type "
Dt_chr11	Cufflinks	exon	56363789	56365174	.	+	.	gene_id "XLOC_145439"; transcript_id "Novel_002016"; exon_number "3"; gene_type "
Dt_chr11	Cufflinks	exon	56638740	56638852	.	+	.	gene_id "XLOC_145462"; transcript_id "Novel_002017"; exon_number "1"; gene_type "
Dt_chr11	Cufflinks	exon	56638953	56639089	.	+	.	gene_id "XLOC_145462"; transcript_id "Novel_002017"; exon_number "2"; gene_type "
Dt_chr11	Cufflinks	exon	58139709	58139794	.	+	.	gene_id "XLOC_145631"; transcript_id "Novel_002018"; exon_number "1"; gene_type "
Dt_chr11	Cufflinks	exon	58139959	58140022	.	+	.	gene_id "XLOC_145631"; transcript_id "Novel_002018"; exon_number "2"; gene_type "
Dt_chr11	Cufflinks	exon	58140115	58140370	.	+	.	gene_id "XLOC_145631"; transcript_id "Novel_002018"; exon_number "3"; gene_type "
Dt_chr11	Cufflinks	exon	60553991	60554075	.	+	.	gene_id "XLOC_145838"; transcript_id "Novel_002019"; exon_number "1"; gene_type "
Dt_chr11	Cufflinks	exon	60554471	60554510	.	+	.	gene_id "XLOC_145838"; transcript_id "Novel_002019"; exon_number "2"; gene_type "
Dt_chr11	Cufflinks	exon	60554620	60554774	.	+	.	gene_id "XLOC_145838"; transcript_id "Novel_002019"; exon_number "3"; gene_type "
Dt_chr11	Cufflinks	exon	60554856	60554932	.	+	.	gene_id "XLOC_145838"; transcript_id "Novel_002019"; exon_number "4"; gene_type "

Dt_chr11	Cufflinks	exon	60556127	60556392	.	+	.	gene_id "XLOC_145839"; transcript_id "Novel_002020"; exon_number "1"; gene_type "
Dt_chr11	Cufflinks	exon	60556547	60556647	.	+	.	gene_id "XLOC_145839"; transcript_id "Novel_002020"; exon_number "2"; gene_type "
Dt_chr11	Cufflinks	exon	60556773	60556877	.	+	.	gene_id "XLOC_145839"; transcript_id "Novel_002020"; exon_number "3"; gene_type "
Dt_chr11	Cufflinks	exon	60557028	60557360	.	+	.	gene_id "XLOC_145839"; transcript_id "Novel_002020"; exon_number "4"; gene_type "
Dt_chr11	Cufflinks	exon	60556367	60556647	.	+	.	gene_id "XLOC_145839"; transcript_id "Novel_002021"; exon_number "1"; gene_type "
Dt_chr11	Cufflinks	exon	60556773	60556877	.	+	.	gene_id "XLOC_145839"; transcript_id "Novel_002021"; exon_number "2"; gene_type "
Dt_chr11	Cufflinks	exon	60557028	60557360	.	+	.	gene_id "XLOC_145839"; transcript_id "Novel_002021"; exon_number "3"; gene_type "
Dt_chr11	Cufflinks	exon	61000006	61001089	.	+	.	gene_id "XLOC_145906"; transcript_id "Novel_002022"; exon_number "1"; gene_type "
Dt_chr11	Cufflinks	exon	1195813	1195954	.	-	.	gene_id "XLOC_146115"; transcript_id "Novel_002023"; exon_number "1"; gene_type "
Dt_chr11	Cufflinks	exon	1196095	1196178	.	-	.	gene_id "XLOC_146115"; transcript_id "Novel_002023"; exon_number "2"; gene_type "
Dt_chr11	Cufflinks	exon	1197358	1197407	.	-	.	gene_id "XLOC_146115"; transcript_id "Novel_002023"; exon_number "3"; gene_type "
Dt_chr11	Cufflinks	exon	1199532	1199616	.	-	.	gene_id "XLOC_146115"; transcript_id "Novel_002023"; exon_number "4"; gene_type "
Dt_chr11	Cufflinks	exon	1195927	1195954	.	-	.	gene_id "XLOC_146115"; transcript_id "Novel_002024"; exon_number "1"; gene_type "
Dt_chr11	Cufflinks	exon	1196095	1196178	.	-	.	gene_id "XLOC_146115"; transcript_id "Novel_002024"; exon_number "2"; gene_type "
Dt_chr11	Cufflinks	exon	1197358	1197647	.	-	.	gene_id "XLOC_146115"; transcript_id "Novel_002024"; exon_number "3"; gene_type "
Dt_chr11	Cufflinks	exon	3065122	3065957	.	-	.	gene_id "XLOC_146337"; transcript_id "Novel_002025"; exon_number "1"; gene_type "
Dt_chr11	Cufflinks	exon	3067501	3067860	.	-	.	gene_id "XLOC_146337"; transcript_id "Novel_002025"; exon_number "2"; gene_type "
Dt_chr11	Cufflinks	exon	3385095	3386269	.	-	.	gene_id "XLOC_146392"; transcript_id "Novel_002026"; exon_number "1"; gene_type "
Dt_chr11	Cufflinks	exon	3386417	3386683	.	-	.	gene_id "XLOC_146392"; transcript_id "Novel_002026"; exon_number "2"; gene_type "
Dt_chr11	Cufflinks	exon	3385276	3385735	.	-	.	gene_id "XLOC_146392"; transcript_id "Novel_002027"; exon_number "1"; gene_type "
Dt_chr11	Cufflinks	exon	3386047	3386689	.	-	.	gene_id "XLOC_146392"; transcript_id "Novel_002027"; exon_number "2"; gene_type "
Dt_chr11	Cufflinks	exon	5624526	5625086	.	-	.	gene_id "XLOC_146715"; transcript_id "Novel_002028"; exon_number "1"; gene_type "
Dt_chr11	Cufflinks	exon	5625456	5627647	.	-	.	gene_id "XLOC_146715"; transcript_id "Novel_002028"; exon_number "2"; gene_type "
Dt_chr11	Cufflinks	exon	5629056	5629143	.	-	.	gene_id "XLOC_146715"; transcript_id "Novel_002028"; exon_number "3"; gene_type "
Dt_chr11	Cufflinks	exon	6152061	6152527	.	-	.	gene_id "XLOC_146806"; transcript_id "Novel_002029"; exon_number "1"; gene_type "
Dt_chr11	Cufflinks	exon	6153269	6154121	.	-	.	gene_id "XLOC_146806"; transcript_id "Novel_002029"; exon_number "2"; gene_type "
Dt_chr11	Cufflinks	exon	7365505	7368300	.	-	.	gene_id "XLOC_146952"; transcript_id "Novel_002030"; exon_number "1"; gene_type "
Dt_chr11	Cufflinks	exon	7368393	7368449	.	-	.	gene_id "XLOC_146952"; transcript_id "Novel_002030"; exon_number "2"; gene_type "
Dt_chr11	Cufflinks	exon	7368529	7368741	.	-	.	gene_id "XLOC_146952"; transcript_id "Novel_002030"; exon_number "3"; gene_type "
Dt_chr11	Cufflinks	exon	7365505	7368449	.	-	.	gene_id "XLOC_146952"; transcript_id "Novel_002031"; exon_number "1"; gene_type "
Dt_chr11	Cufflinks	exon	7368529	7368741	.	-	.	gene_id "XLOC_146952"; transcript_id "Novel_002031"; exon_number "2"; gene_type "
Dt_chr11	Cufflinks	exon	7365545	7367621	.	-	.	gene_id "XLOC_146952"; transcript_id "Novel_002032"; exon_number "1"; gene_type "
Dt_chr11	Cufflinks	exon	7367815	7367907	.	-	.	gene_id "XLOC_146952"; transcript_id "Novel_002032"; exon_number "2"; gene_type "
Dt_chr11	Cufflinks	exon	7368009	7368740	.	-	.	gene_id "XLOC_146952"; transcript_id "Novel_002032"; exon_number "3"; gene_type "
Dt_chr11	Cufflinks	exon	7365545	7367621	.	-	.	gene_id "XLOC_146952"; transcript_id "Novel_002033"; exon_number "1"; gene_type "

Dt_chr11	Cufflinks	exon	7367815	7368300	.	-	.	gene_id "XLOC_146952"; transcript_id "Novel_002033"; exon_number "2"; gene_type "
Dt_chr11	Cufflinks	exon	7368393	7368449	.	-	.	gene_id "XLOC_146952"; transcript_id "Novel_002033"; exon_number "3"; gene_type "
Dt_chr11	Cufflinks	exon	7368529	7368740	.	-	.	gene_id "XLOC_146952"; transcript_id "Novel_002033"; exon_number "4"; gene_type "
Dt_chr11	Cufflinks	exon	7366717	7366841	.	-	.	gene_id "XLOC_146952"; transcript_id "Novel_002034"; exon_number "1"; gene_type "
Dt_chr11	Cufflinks	exon	7367578	7367621	.	-	.	gene_id "XLOC_146952"; transcript_id "Novel_002034"; exon_number "2"; gene_type "
Dt_chr11	Cufflinks	exon	7367815	7367907	.	-	.	gene_id "XLOC_146952"; transcript_id "Novel_002034"; exon_number "3"; gene_type "
Dt_chr11	Cufflinks	exon	7367979	7368741	.	-	.	gene_id "XLOC_146952"; transcript_id "Novel_002034"; exon_number "4"; gene_type "
Dt_chr11	Cufflinks	exon	7476798	7477230	.	-	.	gene_id "XLOC_146960"; transcript_id "Novel_002035"; exon_number "1"; gene_type "
Dt_chr11	Cufflinks	exon	7477510	7477658	.	-	.	gene_id "XLOC_146960"; transcript_id "Novel_002035"; exon_number "2"; gene_type "
Dt_chr11	Cufflinks	exon	7491813	7492525	.	-	.	gene_id "XLOC_146961"; transcript_id "Novel_002036"; exon_number "1"; gene_type "
Dt_chr11	Cufflinks	exon	7492598	7492632	.	-	.	gene_id "XLOC_146961"; transcript_id "Novel_002036"; exon_number "2"; gene_type "
Dt_chr11	Cufflinks	exon	7492946	7493048	.	-	.	gene_id "XLOC_146961"; transcript_id "Novel_002036"; exon_number "3"; gene_type "
Dt_chr11	Cufflinks	exon	7493289	7493367	.	-	.	gene_id "XLOC_146961"; transcript_id "Novel_002036"; exon_number "4"; gene_type "
Dt_chr11	Cufflinks	exon	7958722	7960642	.	-	.	gene_id "XLOC_147002"; transcript_id "Novel_002037"; exon_number "1"; gene_type "
Dt_chr11	Cufflinks	exon	7960714	7960882	.	-	.	gene_id "XLOC_147002"; transcript_id "Novel_002037"; exon_number "2"; gene_type "
Dt_chr11	Cufflinks	exon	10786221	10789410	.	-	.	gene_id "XLOC_147254"; transcript_id "Novel_002038"; exon_number "1"; gene_type "
Dt_chr11	Cufflinks	exon	10789651	10791749	.	-	.	gene_id "XLOC_147254"; transcript_id "Novel_002038"; exon_number "2"; gene_type "
Dt_chr11	Cufflinks	exon	10786364	10790241	.	-	.	gene_id "XLOC_147254"; transcript_id "Novel_002039"; exon_number "1"; gene_type "
Dt_chr11	Cufflinks	exon	10790321	10791688	.	-	.	gene_id "XLOC_147254"; transcript_id "Novel_002039"; exon_number "2"; gene_type "
Dt_chr11	Cufflinks	exon	10786656	10789761	.	-	.	gene_id "XLOC_147254"; transcript_id "Novel_002040"; exon_number "1"; gene_type "
Dt_chr11	Cufflinks	exon	10789967	10791689	.	-	.	gene_id "XLOC_147254"; transcript_id "Novel_002040"; exon_number "2"; gene_type "
Dt_chr11	Cufflinks	exon	12498572	12498777	.	-	.	gene_id "XLOC_147418"; transcript_id "Novel_002041"; exon_number "1"; gene_type "
Dt_chr11	Cufflinks	exon	12499084	12500182	.	-	.	gene_id "XLOC_147418"; transcript_id "Novel_002041"; exon_number "2"; gene_type "
Dt_chr11	Cufflinks	exon	13198687	13200128	.	-	.	gene_id "XLOC_147482"; transcript_id "Novel_002042"; exon_number "1"; gene_type "
Dt_chr11	Cufflinks	exon	13200219	13200416	.	-	.	gene_id "XLOC_147482"; transcript_id "Novel_002042"; exon_number "2"; gene_type "
Dt_chr11	Cufflinks	exon	13200778	13201010	.	-	.	gene_id "XLOC_147482"; transcript_id "Novel_002042"; exon_number "3"; gene_type "
Dt_chr11	Cufflinks	exon	13198991	13199149	.	-	.	gene_id "XLOC_147482"; transcript_id "Novel_002043"; exon_number "1"; gene_type "
Dt_chr11	Cufflinks	exon	13199996	13200128	.	-	.	gene_id "XLOC_147482"; transcript_id "Novel_002043"; exon_number "2"; gene_type "
Dt_chr11	Cufflinks	exon	13200219	13200416	.	-	.	gene_id "XLOC_147482"; transcript_id "Novel_002043"; exon_number "3"; gene_type "
Dt_chr11	Cufflinks	exon	13200778	13201053	.	-	.	gene_id "XLOC_147482"; transcript_id "Novel_002043"; exon_number "4"; gene_type "
Dt_chr11	Cufflinks	exon	13199237	13200128	.	-	.	gene_id "XLOC_147482"; transcript_id "Novel_002044"; exon_number "1"; gene_type "
Dt_chr11	Cufflinks	exon	13200778	13201076	.	-	.	gene_id "XLOC_147482"; transcript_id "Novel_002044"; exon_number "2"; gene_type "
Dt_chr11	Cufflinks	exon	14737306	14737468	.	-	.	gene_id "XLOC_147575"; transcript_id "Novel_002045"; exon_number "1"; gene_type "
Dt_chr11	Cufflinks	exon	14738026	14738868	.	-	.	gene_id "XLOC_147575"; transcript_id "Novel_002045"; exon_number "2"; gene_type "
Dt_chr11	Cufflinks	exon	14740626	14740763	.	-	.	gene_id "XLOC_147575"; transcript_id "Novel_002045"; exon_number "3"; gene_type "

Dt_chr11	Cufflinks	exon	14740835	14741104	.	-	.	gene_id "XLOC_147575"; transcript_id "Novel_002045"; exon_number "4"; gene_type "
Dt_chr11	Cufflinks	exon	19763252	19763597	.	-	.	gene_id "XLOC_148009"; transcript_id "Novel_002046"; exon_number "1"; gene_type "
Dt_chr11	Cufflinks	exon	19764275	19764326	.	-	.	gene_id "XLOC_148009"; transcript_id "Novel_002046"; exon_number "2"; gene_type "
Dt_chr11	Cufflinks	exon	19764495	19764568	.	-	.	gene_id "XLOC_148009"; transcript_id "Novel_002046"; exon_number "3"; gene_type "
Dt_chr11	Cufflinks	exon	19764844	19765185	.	-	.	gene_id "XLOC_148009"; transcript_id "Novel_002046"; exon_number "4"; gene_type "
Dt_chr11	Cufflinks	exon	21663674	21670217	.	-	.	gene_id "XLOC_148136"; transcript_id "Novel_002047"; exon_number "1"; gene_type "
Dt_chr11	Cufflinks	exon	21671801	21671854	.	-	.	gene_id "XLOC_148136"; transcript_id "Novel_002047"; exon_number "2"; gene_type "
Dt_chr11	Cufflinks	exon	21673685	21673922	.	-	.	gene_id "XLOC_148136"; transcript_id "Novel_002047"; exon_number "3"; gene_type "
Dt_chr11	Cufflinks	exon	21863787	21864433	.	-	.	gene_id "XLOC_148159"; transcript_id "Novel_002048"; exon_number "1"; gene_type "
Dt_chr11	Cufflinks	exon	21864940	21865229	.	-	.	gene_id "XLOC_148159"; transcript_id "Novel_002048"; exon_number "2"; gene_type "
Dt_chr11	Cufflinks	exon	23472808	23474048	.	-	.	gene_id "XLOC_148309"; transcript_id "Novel_002049"; exon_number "1"; gene_type "
Dt_chr11	Cufflinks	exon	23475292	23476054	.	-	.	gene_id "XLOC_148309"; transcript_id "Novel_002049"; exon_number "2"; gene_type "
Dt_chr11	Cufflinks	exon	26757211	26757891	.	-	.	gene_id "XLOC_148521"; transcript_id "Novel_002050"; exon_number "1"; gene_type "
Dt_chr11	Cufflinks	exon	26757998	26758410	.	-	.	gene_id "XLOC_148521"; transcript_id "Novel_002050"; exon_number "2"; gene_type "
Dt_chr11	Cufflinks	exon	26758640	26758880	.	-	.	gene_id "XLOC_148521"; transcript_id "Novel_002050"; exon_number "3"; gene_type "
Dt_chr11	Cufflinks	exon	26758982	26759248	.	-	.	gene_id "XLOC_148521"; transcript_id "Novel_002050"; exon_number "4"; gene_type "
Dt_chr11	Cufflinks	exon	26757324	26757891	.	-	.	gene_id "XLOC_148521"; transcript_id "Novel_002051"; exon_number "1"; gene_type "
Dt_chr11	Cufflinks	exon	26757998	26759248	.	-	.	gene_id "XLOC_148521"; transcript_id "Novel_002051"; exon_number "2"; gene_type "
Dt_chr11	Cufflinks	exon	26757324	26757891	.	-	.	gene_id "XLOC_148521"; transcript_id "Novel_002052"; exon_number "1"; gene_type "
Dt_chr11	Cufflinks	exon	26757998	26758880	.	-	.	gene_id "XLOC_148521"; transcript_id "Novel_002052"; exon_number "2"; gene_type "
Dt_chr11	Cufflinks	exon	26758982	26759264	.	-	.	gene_id "XLOC_148521"; transcript_id "Novel_002052"; exon_number "3"; gene_type "
Dt_chr11	Cufflinks	exon	28054413	28054703	.	-	.	gene_id "XLOC_148579"; transcript_id "Novel_002053"; exon_number "1"; gene_type "
Dt_chr11	Cufflinks	exon	28054794	28055246	.	-	.	gene_id "XLOC_148579"; transcript_id "Novel_002053"; exon_number "2"; gene_type "
Dt_chr11	Cufflinks	exon	28055334	28055993	.	-	.	gene_id "XLOC_148579"; transcript_id "Novel_002053"; exon_number "3"; gene_type "
Dt_chr11	Cufflinks	exon	28056103	28056209	.	-	.	gene_id "XLOC_148579"; transcript_id "Novel_002053"; exon_number "4"; gene_type "
Dt_chr11	Cufflinks	exon	28056331	28056625	.	-	.	gene_id "XLOC_148579"; transcript_id "Novel_002053"; exon_number "5"; gene_type "
Dt_chr11	Cufflinks	exon	28054413	28054703	.	-	.	gene_id "XLOC_148579"; transcript_id "Novel_002054"; exon_number "1"; gene_type "
Dt_chr11	Cufflinks	exon	28054794	28055246	.	-	.	gene_id "XLOC_148579"; transcript_id "Novel_002054"; exon_number "2"; gene_type "
Dt_chr11	Cufflinks	exon	28055334	28056209	.	-	.	gene_id "XLOC_148579"; transcript_id "Novel_002054"; exon_number "3"; gene_type "
Dt_chr11	Cufflinks	exon	28056331	28056625	.	-	.	gene_id "XLOC_148579"; transcript_id "Novel_002054"; exon_number "4"; gene_type "
Dt_chr11	Cufflinks	exon	30312040	30312061	.	-	.	gene_id "XLOC_148675"; transcript_id "Novel_002055"; exon_number "1"; gene_type "
Dt_chr11	Cufflinks	exon	30312156	30312271	.	-	.	gene_id "XLOC_148675"; transcript_id "Novel_002055"; exon_number "2"; gene_type "
Dt_chr11	Cufflinks	exon	30313177	30313274	.	-	.	gene_id "XLOC_148675"; transcript_id "Novel_002055"; exon_number "3"; gene_type "
Dt_chr11	Cufflinks	exon	30312042	30312061	.	-	.	gene_id "XLOC_148675"; transcript_id "Novel_002056"; exon_number "1"; gene_type "
Dt_chr11	Cufflinks	exon	30312156	30312271	.	-	.	gene_id "XLOC_148675"; transcript_id "Novel_002056"; exon_number "2"; gene_type "

Dt_chr11	Cufflinks	exon	30313177	30313270	.	-	.	gene_id "XLOC_148675"; transcript_id "Novel_002056"; exon_number "3"; gene_type "
Dt_chr11	Cufflinks	exon	30313731	30314064	.	-	.	gene_id "XLOC_148675"; transcript_id "Novel_002056"; exon_number "4"; gene_type "
Dt_chr11	Cufflinks	exon	36222611	36223575	.	-	.	gene_id "XLOC_148993"; transcript_id "Novel_002057"; exon_number "1"; gene_type "
Dt_chr11	Cufflinks	exon	36223680	36223801	.	-	.	gene_id "XLOC_148993"; transcript_id "Novel_002057"; exon_number "2"; gene_type "
Dt_chr11	Cufflinks	exon	36224563	36224865	.	-	.	gene_id "XLOC_148993"; transcript_id "Novel_002057"; exon_number "3"; gene_type "
Dt_chr11	Cufflinks	exon	36225636	36225725	.	-	.	gene_id "XLOC_148993"; transcript_id "Novel_002057"; exon_number "4"; gene_type "
Dt_chr11	Cufflinks	exon	36222613	36223575	.	-	.	gene_id "XLOC_148993"; transcript_id "Novel_002058"; exon_number "1"; gene_type "
Dt_chr11	Cufflinks	exon	36223680	36223801	.	-	.	gene_id "XLOC_148993"; transcript_id "Novel_002058"; exon_number "2"; gene_type "
Dt_chr11	Cufflinks	exon	36224563	36225738	.	-	.	gene_id "XLOC_148993"; transcript_id "Novel_002058"; exon_number "3"; gene_type "
Dt_chr11	Cufflinks	exon	36222644	36223575	.	-	.	gene_id "XLOC_148993"; transcript_id "Novel_002059"; exon_number "1"; gene_type "
Dt_chr11	Cufflinks	exon	36223680	36225578	.	-	.	gene_id "XLOC_148993"; transcript_id "Novel_002059"; exon_number "2"; gene_type "
Dt_chr11	Cufflinks	exon	36222937	36223575	.	-	.	gene_id "XLOC_148993"; transcript_id "Novel_002060"; exon_number "1"; gene_type "
Dt_chr11	Cufflinks	exon	36223680	36224865	.	-	.	gene_id "XLOC_148993"; transcript_id "Novel_002060"; exon_number "2"; gene_type "
Dt_chr11	Cufflinks	exon	36225631	36225738	.	-	.	gene_id "XLOC_148993"; transcript_id "Novel_002060"; exon_number "3"; gene_type "
Dt_chr11	Cufflinks	exon	36224546	36224865	.	-	.	gene_id "XLOC_148993"; transcript_id "Novel_002061"; exon_number "1"; gene_type "
Dt_chr11	Cufflinks	exon	36225636	36225754	.	-	.	gene_id "XLOC_148993"; transcript_id "Novel_002061"; exon_number "2"; gene_type "
Dt_chr11	Cufflinks	exon	37583313	37583628	.	-	.	gene_id "XLOC_149058"; transcript_id "Novel_002062"; exon_number "1"; gene_type "
Dt_chr11	Cufflinks	exon	37583713	37583770	.	-	.	gene_id "XLOC_149058"; transcript_id "Novel_002062"; exon_number "2"; gene_type "
Dt_chr11	Cufflinks	exon	37585519	37585746	.	-	.	gene_id "XLOC_149058"; transcript_id "Novel_002062"; exon_number "3"; gene_type "
Dt_chr11	Cufflinks	exon	37583313	37583628	.	-	.	gene_id "XLOC_149058"; transcript_id "Novel_002063"; exon_number "1"; gene_type "
Dt_chr11	Cufflinks	exon	37583713	37583770	.	-	.	gene_id "XLOC_149058"; transcript_id "Novel_002063"; exon_number "2"; gene_type "
Dt_chr11	Cufflinks	exon	37584732	37584793	.	-	.	gene_id "XLOC_149058"; transcript_id "Novel_002063"; exon_number "3"; gene_type "
Dt_chr11	Cufflinks	exon	37585519	37585746	.	-	.	gene_id "XLOC_149058"; transcript_id "Novel_002063"; exon_number "4"; gene_type "
Dt_chr11	Cufflinks	exon	37583386	37583628	.	-	.	gene_id "XLOC_149058"; transcript_id "Novel_002064"; exon_number "1"; gene_type "
Dt_chr11	Cufflinks	exon	37583713	37584351	.	-	.	gene_id "XLOC_149058"; transcript_id "Novel_002064"; exon_number "2"; gene_type "
Dt_chr11	Cufflinks	exon	37584630	37585049	.	-	.	gene_id "XLOC_149058"; transcript_id "Novel_002065"; exon_number "1"; gene_type "
Dt_chr11	Cufflinks	exon	37585519	37585746	.	-	.	gene_id "XLOC_149058"; transcript_id "Novel_002065"; exon_number "2"; gene_type "
Dt_chr11	Cufflinks	exon	39236947	39238735	.	-	.	gene_id "XLOC_149114"; transcript_id "Novel_002066"; exon_number "1"; gene_type "
Dt_chr11	Cufflinks	exon	39254013	39254245	.	-	.	gene_id "XLOC_149114"; transcript_id "Novel_002066"; exon_number "2"; gene_type "
Dt_chr11	Cufflinks	exon	39254826	39255235	.	-	.	gene_id "XLOC_149114"; transcript_id "Novel_002066"; exon_number "3"; gene_type "
Dt_chr11	Cufflinks	exon	39251701	39251753	.	-	.	gene_id "XLOC_149114"; transcript_id "Novel_002067"; exon_number "1"; gene_type "
Dt_chr11	Cufflinks	exon	39252452	39255254	.	-	.	gene_id "XLOC_149114"; transcript_id "Novel_002067"; exon_number "2"; gene_type "
Dt_chr11	Cufflinks	exon	40393649	40394200	.	-	.	gene_id "XLOC_149157"; transcript_id "Novel_002068"; exon_number "1"; gene_type "
Dt_chr11	Cufflinks	exon	40394477	40395424	.	-	.	gene_id "XLOC_149157"; transcript_id "Novel_002068"; exon_number "2"; gene_type "
Dt_chr11	Cufflinks	exon	40488159	40489105	.	-	.	gene_id "XLOC_149168"; transcript_id "Novel_002069"; exon_number "1"; gene_type "

Dt_chr11	Cufflinks	exon	40489226	40489328	.	-	.	gene_id "XLOC_149168"; transcript_id "Novel_002069"; exon_number "2"; gene_type "
Dt_chr11	Cufflinks	exon	40489567	40489747	.	-	.	gene_id "XLOC_149168"; transcript_id "Novel_002069"; exon_number "3"; gene_type "
Dt_chr11	Cufflinks	exon	43279424	43279803	.	-	.	gene_id "XLOC_149269"; transcript_id "Novel_002070"; exon_number "1"; gene_type "
Dt_chr11	Cufflinks	exon	43279876	43279989	.	-	.	gene_id "XLOC_149269"; transcript_id "Novel_002070"; exon_number "2"; gene_type "
Dt_chr11	Cufflinks	exon	43280943	43281011	.	-	.	gene_id "XLOC_149269"; transcript_id "Novel_002070"; exon_number "3"; gene_type "
Dt_chr11	Cufflinks	exon	43282252	43282292	.	-	.	gene_id "XLOC_149269"; transcript_id "Novel_002070"; exon_number "4"; gene_type "
Dt_chr11	Cufflinks	exon	43282531	43282650	.	-	.	gene_id "XLOC_149269"; transcript_id "Novel_002070"; exon_number "5"; gene_type "
Dt_chr11	Cufflinks	exon	43283300	43283470	.	-	.	gene_id "XLOC_149269"; transcript_id "Novel_002070"; exon_number "6"; gene_type "
Dt_chr11	Cufflinks	exon	43283571	43283809	.	-	.	gene_id "XLOC_149269"; transcript_id "Novel_002070"; exon_number "7"; gene_type "
Dt_chr11	Cufflinks	exon	43279424	43279803	.	-	.	gene_id "XLOC_149269"; transcript_id "Novel_002071"; exon_number "1"; gene_type "
Dt_chr11	Cufflinks	exon	43279876	43279989	.	-	.	gene_id "XLOC_149269"; transcript_id "Novel_002071"; exon_number "2"; gene_type "
Dt_chr11	Cufflinks	exon	43280943	43281011	.	-	.	gene_id "XLOC_149269"; transcript_id "Novel_002071"; exon_number "3"; gene_type "
Dt_chr11	Cufflinks	exon	43282531	43282650	.	-	.	gene_id "XLOC_149269"; transcript_id "Novel_002071"; exon_number "4"; gene_type "
Dt_chr11	Cufflinks	exon	43283300	43283809	.	-	.	gene_id "XLOC_149269"; transcript_id "Novel_002071"; exon_number "5"; gene_type "
Dt_chr11	Cufflinks	exon	43279508	43279989	.	-	.	gene_id "XLOC_149269"; transcript_id "Novel_002072"; exon_number "1"; gene_type "
Dt_chr11	Cufflinks	exon	43280943	43281523	.	-	.	gene_id "XLOC_149269"; transcript_id "Novel_002072"; exon_number "2"; gene_type "
Dt_chr11	Cufflinks	exon	43282252	43282292	.	-	.	gene_id "XLOC_149269"; transcript_id "Novel_002072"; exon_number "3"; gene_type "
Dt_chr11	Cufflinks	exon	43282531	43282650	.	-	.	gene_id "XLOC_149269"; transcript_id "Novel_002072"; exon_number "4"; gene_type "
Dt_chr11	Cufflinks	exon	43283300	43283470	.	-	.	gene_id "XLOC_149269"; transcript_id "Novel_002072"; exon_number "5"; gene_type "
Dt_chr11	Cufflinks	exon	43283571	43283821	.	-	.	gene_id "XLOC_149269"; transcript_id "Novel_002072"; exon_number "6"; gene_type "
Dt_chr11	Cufflinks	exon	44405783	44406166	.	-	.	gene_id "XLOC_149320"; transcript_id "Novel_002073"; exon_number "1"; gene_type "
Dt_chr11	Cufflinks	exon	44406475	44407515	.	-	.	gene_id "XLOC_149320"; transcript_id "Novel_002073"; exon_number "2"; gene_type "
Dt_chr11	Cufflinks	exon	45358628	45359156	.	-	.	gene_id "XLOC_149398"; transcript_id "Novel_002074"; exon_number "1"; gene_type "
Dt_chr11	Cufflinks	exon	45359239	45359640	.	-	.	gene_id "XLOC_149398"; transcript_id "Novel_002074"; exon_number "2"; gene_type "
Dt_chr11	Cufflinks	exon	45457561	45458467	.	-	.	gene_id "XLOC_149407"; transcript_id "Novel_002075"; exon_number "1"; gene_type "
Dt_chr11	Cufflinks	exon	45458566	45458623	.	-	.	gene_id "XLOC_149407"; transcript_id "Novel_002075"; exon_number "2"; gene_type "
Dt_chr11	Cufflinks	exon	45460311	45460748	.	-	.	gene_id "XLOC_149407"; transcript_id "Novel_002075"; exon_number "3"; gene_type "
Dt_chr11	Cufflinks	exon	46817048	46817442	.	-	.	gene_id "XLOC_149544"; transcript_id "Novel_002076"; exon_number "1"; gene_type "
Dt_chr11	Cufflinks	exon	46819208	46819255	.	-	.	gene_id "XLOC_149544"; transcript_id "Novel_002076"; exon_number "2"; gene_type "
Dt_chr11	Cufflinks	exon	46819487	46819689	.	-	.	gene_id "XLOC_149544"; transcript_id "Novel_002076"; exon_number "3"; gene_type "
Dt_chr11	Cufflinks	exon	47283928	47284203	.	-	.	gene_id "XLOC_149586"; transcript_id "Novel_002077"; exon_number "1"; gene_type "
Dt_chr11	Cufflinks	exon	48064074	48067892	.	-	.	gene_id "XLOC_149694"; transcript_id "Novel_002078"; exon_number "1"; gene_type "
Dt_chr11	Cufflinks	exon	48068059	48068484	.	-	.	gene_id "XLOC_149694"; transcript_id "Novel_002078"; exon_number "2"; gene_type "
Dt_chr11	Cufflinks	exon	48616207	48616559	.	-	.	gene_id "XLOC_149810"; transcript_id "Novel_002079"; exon_number "1"; gene_type "
Dt_chr11	Cufflinks	exon	48618129	48618622	.	-	.	gene_id "XLOC_149810"; transcript_id "Novel_002079"; exon_number "2"; gene_type "

Dt_chr11	Cufflinks	exon	50000185	50000577	.	-	.	gene_id "XLOC_149950"; transcript_id "Novel_002080"; exon_number "1"; gene_type "
Dt_chr11	Cufflinks	exon	53549773	53550221	.	-	.	gene_id "XLOC_150206"; transcript_id "Novel_002081"; exon_number "1"; gene_type "
Dt_chr11	Cufflinks	exon	53550319	53550404	.	-	.	gene_id "XLOC_150206"; transcript_id "Novel_002081"; exon_number "2"; gene_type "
Dt_chr11	Cufflinks	exon	54311655	54312136	.	-	.	gene_id "XLOC_150235"; transcript_id "Novel_002082"; exon_number "1"; gene_type "
Dt_chr11	Cufflinks	exon	54312327	54312486	.	-	.	gene_id "XLOC_150235"; transcript_id "Novel_002082"; exon_number "2"; gene_type "
Dt_chr11	Cufflinks	exon	54311655	54312136	.	-	.	gene_id "XLOC_150235"; transcript_id "Novel_002083"; exon_number "1"; gene_type "
Dt_chr11	Cufflinks	exon	54312381	54312502	.	-	.	gene_id "XLOC_150235"; transcript_id "Novel_002083"; exon_number "2"; gene_type "
Dt_chr12	Cufflinks	exon	1259394	1259528	.	+	.	gene_id "XLOC_151131"; transcript_id "Novel_002084"; exon_number "1"; gene_type "
Dt_chr12	Cufflinks	exon	1259665	1259731	.	+	.	gene_id "XLOC_151131"; transcript_id "Novel_002084"; exon_number "2"; gene_type "
Dt_chr12	Cufflinks	exon	1260726	1262246	.	+	.	gene_id "XLOC_151131"; transcript_id "Novel_002084"; exon_number "3"; gene_type "
Dt_chr12	Cufflinks	exon	1259394	1259528	.	+	.	gene_id "XLOC_151131"; transcript_id "Novel_002085"; exon_number "1"; gene_type "
Dt_chr12	Cufflinks	exon	1259665	1259735	.	+	.	gene_id "XLOC_151131"; transcript_id "Novel_002085"; exon_number "2"; gene_type "
Dt_chr12	Cufflinks	exon	1260726	1262246	.	+	.	gene_id "XLOC_151131"; transcript_id "Novel_002085"; exon_number "3"; gene_type "
Dt_chr12	Cufflinks	exon	1396266	1396395	.	+	.	gene_id "XLOC_151149"; transcript_id "Novel_002086"; exon_number "1"; gene_type "
Dt_chr12	Cufflinks	exon	1396485	1396642	.	+	.	gene_id "XLOC_151149"; transcript_id "Novel_002086"; exon_number "2"; gene_type "
Dt_chr12	Cufflinks	exon	1397464	1397534	.	+	.	gene_id "XLOC_151149"; transcript_id "Novel_002086"; exon_number "3"; gene_type "
Dt_chr12	Cufflinks	exon	1396330	1396395	.	+	.	gene_id "XLOC_151149"; transcript_id "Novel_002087"; exon_number "1"; gene_type "
Dt_chr12	Cufflinks	exon	1396485	1396724	.	+	.	gene_id "XLOC_151149"; transcript_id "Novel_002087"; exon_number "2"; gene_type "
Dt_chr12	Cufflinks	exon	1632337	1632538	.	+	.	gene_id "XLOC_151207"; transcript_id "Novel_002088"; exon_number "1"; gene_type "
Dt_chr12	Cufflinks	exon	1632720	1632769	.	+	.	gene_id "XLOC_151207"; transcript_id "Novel_002088"; exon_number "2"; gene_type "
Dt_chr12	Cufflinks	exon	2088812	2089069	.	+	.	gene_id "XLOC_151262"; transcript_id "Novel_002089"; exon_number "1"; gene_type "
Dt_chr12	Cufflinks	exon	3192322	3192695	.	+	.	gene_id "XLOC_151387"; transcript_id "Novel_002090"; exon_number "1"; gene_type "
Dt_chr12	Cufflinks	exon	3192793	3193108	.	+	.	gene_id "XLOC_151387"; transcript_id "Novel_002090"; exon_number "2"; gene_type "
Dt_chr12	Cufflinks	exon	3193733	3193828	.	+	.	gene_id "XLOC_151387"; transcript_id "Novel_002090"; exon_number "3"; gene_type "
Dt_chr12	Cufflinks	exon	3194336	3194404	.	+	.	gene_id "XLOC_151387"; transcript_id "Novel_002090"; exon_number "4"; gene_type "
Dt_chr12	Cufflinks	exon	3195245	3195373	.	+	.	gene_id "XLOC_151387"; transcript_id "Novel_002090"; exon_number "5"; gene_type "
Dt_chr12	Cufflinks	exon	3195740	3195839	.	+	.	gene_id "XLOC_151387"; transcript_id "Novel_002090"; exon_number "6"; gene_type "
Dt_chr12	Cufflinks	exon	3196286	3197152	.	+	.	gene_id "XLOC_151387"; transcript_id "Novel_002090"; exon_number "7"; gene_type "
Dt_chr12	Cufflinks	exon	4286930	4287277	.	+	.	gene_id "XLOC_151546"; transcript_id "Novel_002091"; exon_number "1"; gene_type "
Dt_chr12	Cufflinks	exon	4287580	4287760	.	+	.	gene_id "XLOC_151546"; transcript_id "Novel_002091"; exon_number "2"; gene_type "
Dt_chr12	Cufflinks	exon	4289395	4289882	.	+	.	gene_id "XLOC_151546"; transcript_id "Novel_002091"; exon_number "3"; gene_type "
Dt_chr12	Cufflinks	exon	4287072	4287154	.	+	.	gene_id "XLOC_151546"; transcript_id "Novel_002092"; exon_number "1"; gene_type "
Dt_chr12	Cufflinks	exon	4287587	4287760	.	+	.	gene_id "XLOC_151546"; transcript_id "Novel_002092"; exon_number "2"; gene_type "
Dt_chr12	Cufflinks	exon	4289395	4289877	.	+	.	gene_id "XLOC_151546"; transcript_id "Novel_002092"; exon_number "3"; gene_type "
Dt_chr12	Cufflinks	exon	5346844	5347045	.	+	.	gene_id "XLOC_151728"; transcript_id "Novel_002093"; exon_number "1"; gene_type "

Dt_chr12	Cufflinks	exon	5347118	5347168	.	+	.	gene_id "XLOC_151728"; transcript_id "Novel_002093"; exon_number "2"; gene_type "
Dt_chr12	Cufflinks	exon	5347262	5347338	.	+	.	gene_id "XLOC_151728"; transcript_id "Novel_002093"; exon_number "3"; gene_type "
Dt_chr12	Cufflinks	exon	5347434	5347521	.	+	.	gene_id "XLOC_151728"; transcript_id "Novel_002093"; exon_number "4"; gene_type "
Dt_chr12	Cufflinks	exon	5347606	5347881	.	+	.	gene_id "XLOC_151728"; transcript_id "Novel_002093"; exon_number "5"; gene_type "
Dt_chr12	Cufflinks	exon	5347977	5348225	.	+	.	gene_id "XLOC_151728"; transcript_id "Novel_002093"; exon_number "6"; gene_type "
Dt_chr12	Cufflinks	exon	5348312	5348623	.	+	.	gene_id "XLOC_151728"; transcript_id "Novel_002093"; exon_number "7"; gene_type "
Dt_chr12	Cufflinks	exon	5347571	5347881	.	+	.	gene_id "XLOC_151728"; transcript_id "Novel_002094"; exon_number "1"; gene_type "
Dt_chr12	Cufflinks	exon	5347977	5348225	.	+	.	gene_id "XLOC_151728"; transcript_id "Novel_002094"; exon_number "2"; gene_type "
Dt_chr12	Cufflinks	exon	5348312	5348623	.	+	.	gene_id "XLOC_151728"; transcript_id "Novel_002094"; exon_number "3"; gene_type "
Dt_chr12	Cufflinks	exon	5643937	5644194	.	+	.	gene_id "XLOC_151793"; transcript_id "Novel_002095"; exon_number "1"; gene_type "
Dt_chr12	Cufflinks	exon	5645600	5645656	.	+	.	gene_id "XLOC_151793"; transcript_id "Novel_002095"; exon_number "2"; gene_type "
Dt_chr12	Cufflinks	exon	5646533	5649264	.	+	.	gene_id "XLOC_151793"; transcript_id "Novel_002095"; exon_number "3"; gene_type "
Dt_chr12	Cufflinks	exon	5900971	5901213	.	+	.	gene_id "XLOC_151824"; transcript_id "Novel_002096"; exon_number "1"; gene_type "
Dt_chr12	Cufflinks	exon	6033577	6033848	.	+	.	gene_id "XLOC_151844"; transcript_id "Novel_002097"; exon_number "1"; gene_type "
Dt_chr12	Cufflinks	exon	6034653	6036151	.	+	.	gene_id "XLOC_151844"; transcript_id "Novel_002097"; exon_number "2"; gene_type "
Dt_chr12	Cufflinks	exon	6036822	6037168	.	+	.	gene_id "XLOC_151844"; transcript_id "Novel_002097"; exon_number "3"; gene_type "
Dt_chr12	Cufflinks	exon	6047906	6047983	.	+	.	gene_id "XLOC_151849"; transcript_id "Novel_002098"; exon_number "1"; gene_type "
Dt_chr12	Cufflinks	exon	6048313	6048992	.	+	.	gene_id "XLOC_151849"; transcript_id "Novel_002098"; exon_number "2"; gene_type "
Dt_chr12	Cufflinks	exon	6049096	6049135	.	+	.	gene_id "XLOC_151849"; transcript_id "Novel_002098"; exon_number "3"; gene_type "
Dt_chr12	Cufflinks	exon	6049367	6049419	.	+	.	gene_id "XLOC_151849"; transcript_id "Novel_002098"; exon_number "4"; gene_type "
Dt_chr12	Cufflinks	exon	6049682	6049727	.	+	.	gene_id "XLOC_151849"; transcript_id "Novel_002098"; exon_number "5"; gene_type "
Dt_chr12	Cufflinks	exon	6049887	6049979	.	+	.	gene_id "XLOC_151849"; transcript_id "Novel_002098"; exon_number "6"; gene_type "
Dt_chr12	Cufflinks	exon	6050573	6050695	.	+	.	gene_id "XLOC_151849"; transcript_id "Novel_002098"; exon_number "7"; gene_type "
Dt_chr12	Cufflinks	exon	6050814	6050865	.	+	.	gene_id "XLOC_151849"; transcript_id "Novel_002098"; exon_number "8"; gene_type "
Dt_chr12	Cufflinks	exon	6050953	6051028	.	+	.	gene_id "XLOC_151849"; transcript_id "Novel_002098"; exon_number "9"; gene_type "
Dt_chr12	Cufflinks	exon	6051120	6051201	.	+	.	gene_id "XLOC_151849"; transcript_id "Novel_002098"; exon_number "10"; gene_type
Dt_chr12	Cufflinks	exon	6051292	6051342	.	+	.	gene_id "XLOC_151849"; transcript_id "Novel_002098"; exon_number "11"; gene_type
Dt_chr12	Cufflinks	exon	6051698	6051790	.	+	.	gene_id "XLOC_151849"; transcript_id "Novel_002098"; exon_number "12"; gene_type
Dt_chr12	Cufflinks	exon	6051930	6052504	.	+	.	gene_id "XLOC_151849"; transcript_id "Novel_002098"; exon_number "13"; gene_type
Dt_chr12	Cufflinks	exon	8500904	8501498	.	+	.	gene_id "XLOC_152102"; transcript_id "Novel_002099"; exon_number "1"; gene_type "
Dt_chr12	Cufflinks	exon	8502073	8502148	.	+	.	gene_id "XLOC_152102"; transcript_id "Novel_002099"; exon_number "2"; gene_type "
Dt_chr12	Cufflinks	exon	8502237	8503013	.	+	.	gene_id "XLOC_152102"; transcript_id "Novel_002099"; exon_number "3"; gene_type "
Dt_chr12	Cufflinks	exon	8503131	8503754	.	+	.	gene_id "XLOC_152102"; transcript_id "Novel_002099"; exon_number "4"; gene_type "
Dt_chr12	Cufflinks	exon	8504273	8504527	.	+	.	gene_id "XLOC_152102"; transcript_id "Novel_002099"; exon_number "5"; gene_type "
Dt_chr12	Cufflinks	exon	9279665	9279994	.	+	.	gene_id "XLOC_152169"; transcript_id "Novel_002100"; exon_number "1"; gene_type "

Dt_chr12	Cufflinks	exon	9280928	9282316	.	+	.	gene_id "XLOC_152169"; transcript_id "Novel_002100"; exon_number "2"; gene_type "
Dt_chr12	Cufflinks	exon	10921286	10921782	.	+	.	gene_id "XLOC_152356"; transcript_id "Novel_002101"; exon_number "1"; gene_type "
Dt_chr12	Cufflinks	exon	10922170	10922285	.	+	.	gene_id "XLOC_152356"; transcript_id "Novel_002101"; exon_number "2"; gene_type "
Dt_chr12	Cufflinks	exon	10922400	10922463	.	+	.	gene_id "XLOC_152356"; transcript_id "Novel_002101"; exon_number "3"; gene_type "
Dt_chr12	Cufflinks	exon	10923068	10923170	.	+	.	gene_id "XLOC_152356"; transcript_id "Novel_002101"; exon_number "4"; gene_type "
Dt_chr12	Cufflinks	exon	10923453	10923774	.	+	.	gene_id "XLOC_152356"; transcript_id "Novel_002101"; exon_number "5"; gene_type "
Dt_chr12	Cufflinks	exon	10923880	10924000	.	+	.	gene_id "XLOC_152356"; transcript_id "Novel_002101"; exon_number "6"; gene_type "
Dt_chr12	Cufflinks	exon	10921350	10921782	.	+	.	gene_id "XLOC_152356"; transcript_id "Novel_002102"; exon_number "1"; gene_type "
Dt_chr12	Cufflinks	exon	10922170	10922285	.	+	.	gene_id "XLOC_152356"; transcript_id "Novel_002102"; exon_number "2"; gene_type "
Dt_chr12	Cufflinks	exon	10922400	10922463	.	+	.	gene_id "XLOC_152356"; transcript_id "Novel_002102"; exon_number "3"; gene_type "
Dt_chr12	Cufflinks	exon	10923068	10923170	.	+	.	gene_id "XLOC_152356"; transcript_id "Novel_002102"; exon_number "4"; gene_type "
Dt_chr12	Cufflinks	exon	10923453	10924066	.	+	.	gene_id "XLOC_152356"; transcript_id "Novel_002102"; exon_number "5"; gene_type "
Dt_chr12	Cufflinks	exon	10921467	10921782	.	+	.	gene_id "XLOC_152356"; transcript_id "Novel_002103"; exon_number "1"; gene_type "
Dt_chr12	Cufflinks	exon	10922170	10922285	.	+	.	gene_id "XLOC_152356"; transcript_id "Novel_002103"; exon_number "2"; gene_type "
Dt_chr12	Cufflinks	exon	10922400	10922463	.	+	.	gene_id "XLOC_152356"; transcript_id "Novel_002103"; exon_number "3"; gene_type "
Dt_chr12	Cufflinks	exon	10923068	10923993	.	+	.	gene_id "XLOC_152356"; transcript_id "Novel_002103"; exon_number "4"; gene_type "
Dt_chr12	Cufflinks	exon	11175261	11175293	.	+	.	gene_id "XLOC_152389"; transcript_id "Novel_002104"; exon_number "1"; gene_type "
Dt_chr12	Cufflinks	exon	11175642	11175869	.	+	.	gene_id "XLOC_152389"; transcript_id "Novel_002104"; exon_number "2"; gene_type "
Dt_chr12	Cufflinks	exon	11202356	11202459	.	+	.	gene_id "XLOC_152392"; transcript_id "Novel_002105"; exon_number "1"; gene_type "
Dt_chr12	Cufflinks	exon	11202561	11203206	.	+	.	gene_id "XLOC_152392"; transcript_id "Novel_002105"; exon_number "2"; gene_type "
Dt_chr12	Cufflinks	exon	11369856	11370220	.	+	.	gene_id "XLOC_152415"; transcript_id "Novel_002106"; exon_number "1"; gene_type "
Dt_chr12	Cufflinks	exon	11371918	11371994	.	+	.	gene_id "XLOC_152415"; transcript_id "Novel_002106"; exon_number "2"; gene_type "
Dt_chr12	Cufflinks	exon	11372192	11372276	.	+	.	gene_id "XLOC_152415"; transcript_id "Novel_002106"; exon_number "3"; gene_type "
Dt_chr12	Cufflinks	exon	11372525	11373238	.	+	.	gene_id "XLOC_152415"; transcript_id "Novel_002106"; exon_number "4"; gene_type "
Dt_chr12	Cufflinks	exon	11369948	11370220	.	+	.	gene_id "XLOC_152415"; transcript_id "Novel_002107"; exon_number "1"; gene_type "
Dt_chr12	Cufflinks	exon	11370408	11370453	.	+	.	gene_id "XLOC_152415"; transcript_id "Novel_002107"; exon_number "2"; gene_type "
Dt_chr12	Cufflinks	exon	11371918	11371994	.	+	.	gene_id "XLOC_152415"; transcript_id "Novel_002107"; exon_number "3"; gene_type "
Dt_chr12	Cufflinks	exon	11372192	11372276	.	+	.	gene_id "XLOC_152415"; transcript_id "Novel_002107"; exon_number "4"; gene_type "
Dt_chr12	Cufflinks	exon	11372525	11373122	.	+	.	gene_id "XLOC_152415"; transcript_id "Novel_002107"; exon_number "5"; gene_type "
Dt_chr12	Cufflinks	exon	11371372	11371994	.	+	.	gene_id "XLOC_152415"; transcript_id "Novel_002108"; exon_number "1"; gene_type "
Dt_chr12	Cufflinks	exon	11372192	11372276	.	+	.	gene_id "XLOC_152415"; transcript_id "Novel_002108"; exon_number "2"; gene_type "
Dt_chr12	Cufflinks	exon	11372525	11373197	.	+	.	gene_id "XLOC_152415"; transcript_id "Novel_002108"; exon_number "3"; gene_type "
Dt_chr12	Cufflinks	exon	11476442	11476881	.	+	.	gene_id "XLOC_152432"; transcript_id "Novel_002109"; exon_number "1"; gene_type "
Dt_chr12	Cufflinks	exon	11476973	11477433	.	+	.	gene_id "XLOC_152432"; transcript_id "Novel_002109"; exon_number "2"; gene_type "
Dt_chr12	Cufflinks	exon	11478332	11478584	.	+	.	gene_id "XLOC_152432"; transcript_id "Novel_002109"; exon_number "3"; gene_type "

Dt_chr12	Cufflinks	exon	11479048	11479299	.	+	.	gene_id "XLOC_152432"; transcript_id "Novel_002109"; exon_number "4"; gene_type "
Dt_chr12	Cufflinks	exon	11476456	11476881	.	+	.	gene_id "XLOC_152432"; transcript_id "Novel_002110"; exon_number "1"; gene_type "
Dt_chr12	Cufflinks	exon	11476973	11477100	.	+	.	gene_id "XLOC_152432"; transcript_id "Novel_002110"; exon_number "2"; gene_type "
Dt_chr12	Cufflinks	exon	11477191	11477433	.	+	.	gene_id "XLOC_152432"; transcript_id "Novel_002110"; exon_number "3"; gene_type "
Dt_chr12	Cufflinks	exon	11478332	11478584	.	+	.	gene_id "XLOC_152432"; transcript_id "Novel_002110"; exon_number "4"; gene_type "
Dt_chr12	Cufflinks	exon	11479048	11479263	.	+	.	gene_id "XLOC_152432"; transcript_id "Novel_002110"; exon_number "5"; gene_type "
Dt_chr12	Cufflinks	exon	11515132	11515376	.	+	.	gene_id "XLOC_152435"; transcript_id "Novel_002111"; exon_number "1"; gene_type "
Dt_chr12	Cufflinks	exon	11515491	11515733	.	+	.	gene_id "XLOC_152435"; transcript_id "Novel_002111"; exon_number "2"; gene_type "
Dt_chr12	Cufflinks	exon	11515825	11516077	.	+	.	gene_id "XLOC_152435"; transcript_id "Novel_002111"; exon_number "3"; gene_type "
Dt_chr12	Cufflinks	exon	11516894	11517226	.	+	.	gene_id "XLOC_152435"; transcript_id "Novel_002111"; exon_number "4"; gene_type "
Dt_chr12	Cufflinks	exon	11688410	11688702	.	+	.	gene_id "XLOC_152463"; transcript_id "Novel_002112"; exon_number "1"; gene_type "
Dt_chr12	Cufflinks	exon	11688798	11689244	.	+	.	gene_id "XLOC_152463"; transcript_id "Novel_002112"; exon_number "2"; gene_type "
Dt_chr12	Cufflinks	exon	11688433	11691165	.	+	.	gene_id "XLOC_152463"; transcript_id "Novel_002113"; exon_number "1"; gene_type "
Dt_chr12	Cufflinks	exon	11691282	11691360	.	+	.	gene_id "XLOC_152463"; transcript_id "Novel_002113"; exon_number "2"; gene_type "
Dt_chr12	Cufflinks	exon	11692511	11692582	.	+	.	gene_id "XLOC_152463"; transcript_id "Novel_002113"; exon_number "3"; gene_type "
Dt_chr12	Cufflinks	exon	11692678	11693007	.	+	.	gene_id "XLOC_152463"; transcript_id "Novel_002113"; exon_number "4"; gene_type "
Dt_chr12	Cufflinks	exon	21301419	21301852	.	+	.	gene_id "XLOC_153057"; transcript_id "Novel_002114"; exon_number "1"; gene_type "
Dt_chr12	Cufflinks	exon	21301971	21302065	.	+	.	gene_id "XLOC_153057"; transcript_id "Novel_002114"; exon_number "2"; gene_type "
Dt_chr12	Cufflinks	exon	21302460	21302559	.	+	.	gene_id "XLOC_153057"; transcript_id "Novel_002114"; exon_number "3"; gene_type "
Dt_chr12	Cufflinks	exon	21302774	21302878	.	+	.	gene_id "XLOC_153057"; transcript_id "Novel_002114"; exon_number "4"; gene_type "
Dt_chr12	Cufflinks	exon	21303040	21306778	.	+	.	gene_id "XLOC_153057"; transcript_id "Novel_002114"; exon_number "5"; gene_type "
Dt_chr12	Cufflinks	exon	22794689	22794941	.	+	.	gene_id "XLOC_153154"; transcript_id "Novel_002115"; exon_number "1"; gene_type "
Dt_chr12	Cufflinks	exon	22795504	22795731	.	+	.	gene_id "XLOC_153154"; transcript_id "Novel_002115"; exon_number "2"; gene_type "
Dt_chr12	Cufflinks	exon	22795837	22796914	.	+	.	gene_id "XLOC_153154"; transcript_id "Novel_002115"; exon_number "3"; gene_type "
Dt_chr12	Cufflinks	exon	26247071	26249144	.	+	.	gene_id "XLOC_153309"; transcript_id "Novel_002116"; exon_number "1"; gene_type "
Dt_chr12	Cufflinks	exon	26249230	26249756	.	+	.	gene_id "XLOC_153309"; transcript_id "Novel_002116"; exon_number "2"; gene_type "
Dt_chr12	Cufflinks	exon	29293918	29294653	.	+	.	gene_id "XLOC_153535"; transcript_id "Novel_002117"; exon_number "1"; gene_type "
Dt_chr12	Cufflinks	exon	29294761	29295042	.	+	.	gene_id "XLOC_153535"; transcript_id "Novel_002117"; exon_number "2"; gene_type "
Dt_chr12	Cufflinks	exon	29459669	29459692	.	+	.	gene_id "XLOC_153555"; transcript_id "Novel_002118"; exon_number "1"; gene_type "
Dt_chr12	Cufflinks	exon	29460853	29462847	.	+	.	gene_id "XLOC_153555"; transcript_id "Novel_002118"; exon_number "2"; gene_type "
Dt_chr12	Cufflinks	exon	29459697	29460548	.	+	.	gene_id "XLOC_153555"; transcript_id "Novel_002119"; exon_number "1"; gene_type "
Dt_chr12	Cufflinks	exon	29460853	29462718	.	+	.	gene_id "XLOC_153555"; transcript_id "Novel_002119"; exon_number "2"; gene_type "
Dt_chr12	Cufflinks	exon	30308804	30308946	.	+	.	gene_id "XLOC_153654"; transcript_id "Novel_002120"; exon_number "1"; gene_type "
Dt_chr12	Cufflinks	exon	30309354	30309453	.	+	.	gene_id "XLOC_153654"; transcript_id "Novel_002120"; exon_number "2"; gene_type "
Dt_chr12	Cufflinks	exon	30531403	30533197	.	+	.	gene_id "XLOC_153664"; transcript_id "Novel_002121"; exon_number "1"; gene_type "

Dt_chr12	Cufflinks	exon	30533404	30535111	.	+	.	gene_id "XLOC_153664"; transcript_id "Novel_002121"; exon_number "2"; gene_type "
Dt_chr12	Cufflinks	exon	31310341	31310661	.	+	.	gene_id "XLOC_153750"; transcript_id "Novel_002122"; exon_number "1"; gene_type "
Dt_chr12	Cufflinks	exon	31311335	31311432	.	+	.	gene_id "XLOC_153750"; transcript_id "Novel_002122"; exon_number "2"; gene_type "
Dt_chr12	Cufflinks	exon	31311593	31311623	.	+	.	gene_id "XLOC_153750"; transcript_id "Novel_002122"; exon_number "3"; gene_type "
Dt_chr12	Cufflinks	exon	31312224	31312323	.	+	.	gene_id "XLOC_153750"; transcript_id "Novel_002122"; exon_number "4"; gene_type "
Dt_chr12	Cufflinks	exon	31313668	31313738	.	+	.	gene_id "XLOC_153750"; transcript_id "Novel_002122"; exon_number "5"; gene_type "
Dt_chr12	Cufflinks	exon	31313851	31313994	.	+	.	gene_id "XLOC_153750"; transcript_id "Novel_002122"; exon_number "6"; gene_type "
Dt_chr12	Cufflinks	exon	31314256	31314563	.	+	.	gene_id "XLOC_153750"; transcript_id "Novel_002122"; exon_number "7"; gene_type "
Dt_chr12	Cufflinks	exon	31312082	31312323	.	+	.	gene_id "XLOC_153750"; transcript_id "Novel_002123"; exon_number "1"; gene_type "
Dt_chr12	Cufflinks	exon	31313668	31313738	.	+	.	gene_id "XLOC_153750"; transcript_id "Novel_002123"; exon_number "2"; gene_type "
Dt_chr12	Cufflinks	exon	31313851	31313994	.	+	.	gene_id "XLOC_153750"; transcript_id "Novel_002123"; exon_number "3"; gene_type "
Dt_chr12	Cufflinks	exon	31314256	31314563	.	+	.	gene_id "XLOC_153750"; transcript_id "Novel_002123"; exon_number "4"; gene_type "
Dt_chr12	Cufflinks	exon	31745507	31747086	.	+	.	gene_id "XLOC_153781"; transcript_id "Novel_002124"; exon_number "1"; gene_type "
Dt_chr12	Cufflinks	exon	34208330	34208621	.	+	.	gene_id "XLOC_153918"; transcript_id "Novel_002125"; exon_number "1"; gene_type "
Dt_chr12	Cufflinks	exon	34208975	34209118	.	+	.	gene_id "XLOC_153918"; transcript_id "Novel_002125"; exon_number "2"; gene_type "
Dt_chr12	Cufflinks	exon	34209702	34209907	.	+	.	gene_id "XLOC_153918"; transcript_id "Novel_002125"; exon_number "3"; gene_type "
Dt_chr12	Cufflinks	exon	34210083	34210693	.	+	.	gene_id "XLOC_153918"; transcript_id "Novel_002125"; exon_number "4"; gene_type "
Dt_chr12	Cufflinks	exon	37086915	37087133	.	+	.	gene_id "XLOC_154065"; transcript_id "Novel_002126"; exon_number "1"; gene_type "
Dt_chr12	Cufflinks	exon	1963976	1964176	.	-	.	gene_id "XLOC_154417"; transcript_id "Novel_002127"; exon_number "1"; gene_type "
Dt_chr12	Cufflinks	exon	1985825	1986073	.	-	.	gene_id "XLOC_154418"; transcript_id "Novel_002128"; exon_number "1"; gene_type "
Dt_chr12	Cufflinks	exon	2836415	2836627	.	-	.	gene_id "XLOC_154491"; transcript_id "Novel_002129"; exon_number "1"; gene_type "
Dt_chr12	Cufflinks	exon	3254680	3256825	.	-	.	gene_id "XLOC_154587"; transcript_id "Novel_002130"; exon_number "1"; gene_type "
Dt_chr12	Cufflinks	exon	3258251	3258331	.	-	.	gene_id "XLOC_154587"; transcript_id "Novel_002130"; exon_number "2"; gene_type "
Dt_chr12	Cufflinks	exon	3258449	3258554	.	-	.	gene_id "XLOC_154587"; transcript_id "Novel_002130"; exon_number "3"; gene_type "
Dt_chr12	Cufflinks	exon	3258659	3258817	.	-	.	gene_id "XLOC_154587"; transcript_id "Novel_002130"; exon_number "4"; gene_type "
Dt_chr12	Cufflinks	exon	3254705	3256825	.	-	.	gene_id "XLOC_154587"; transcript_id "Novel_002131"; exon_number "1"; gene_type "
Dt_chr12	Cufflinks	exon	3258251	3258331	.	-	.	gene_id "XLOC_154587"; transcript_id "Novel_002131"; exon_number "2"; gene_type "
Dt_chr12	Cufflinks	exon	3258449	3258793	.	-	.	gene_id "XLOC_154587"; transcript_id "Novel_002131"; exon_number "3"; gene_type "
Dt_chr12	Cufflinks	exon	3258098	3258331	.	-	.	gene_id "XLOC_154587"; transcript_id "Novel_002132"; exon_number "1"; gene_type "
Dt_chr12	Cufflinks	exon	3258449	3258787	.	-	.	gene_id "XLOC_154587"; transcript_id "Novel_002132"; exon_number "2"; gene_type "
Dt_chr12	Cufflinks	exon	3258098	3258331	.	-	.	gene_id "XLOC_154587"; transcript_id "Novel_002133"; exon_number "1"; gene_type "
Dt_chr12	Cufflinks	exon	3258449	3258554	.	-	.	gene_id "XLOC_154587"; transcript_id "Novel_002133"; exon_number "2"; gene_type "
Dt_chr12	Cufflinks	exon	3258659	3258800	.	-	.	gene_id "XLOC_154587"; transcript_id "Novel_002133"; exon_number "3"; gene_type "
Dt_chr12	Cufflinks	exon	3360058	3360965	.	-	.	gene_id "XLOC_154601"; transcript_id "Novel_002134"; exon_number "1"; gene_type "
Dt_chr12	Cufflinks	exon	3361045	3361152	.	-	.	gene_id "XLOC_154601"; transcript_id "Novel_002134"; exon_number "2"; gene_type "

Dt_chr12	Cufflinks	exon	3361615	3361765	.	-	.	gene_id "XLOC_154601"; transcript_id "Novel_002134"; exon_number "3"; gene_type "
Dt_chr12	Cufflinks	exon	3360279	3360965	.	-	.	gene_id "XLOC_154601"; transcript_id "Novel_002135"; exon_number "1"; gene_type "
Dt_chr12	Cufflinks	exon	3361045	3361152	.	-	.	gene_id "XLOC_154601"; transcript_id "Novel_002135"; exon_number "2"; gene_type "
Dt_chr12	Cufflinks	exon	3361615	3361729	.	-	.	gene_id "XLOC_154601"; transcript_id "Novel_002135"; exon_number "3"; gene_type "
Dt_chr12	Cufflinks	exon	3362430	3362605	.	-	.	gene_id "XLOC_154601"; transcript_id "Novel_002135"; exon_number "4"; gene_type "
Dt_chr12	Cufflinks	exon	3764136	3765667	.	-	.	gene_id "XLOC_154657"; transcript_id "Novel_002136"; exon_number "1"; gene_type "
Dt_chr12	Cufflinks	exon	3766584	3766997	.	-	.	gene_id "XLOC_154657"; transcript_id "Novel_002136"; exon_number "2"; gene_type "
Dt_chr12	Cufflinks	exon	4141421	4142278	.	-	.	gene_id "XLOC_154695"; transcript_id "Novel_002137"; exon_number "1"; gene_type "
Dt_chr12	Cufflinks	exon	4144370	4144459	.	-	.	gene_id "XLOC_154695"; transcript_id "Novel_002137"; exon_number "2"; gene_type "
Dt_chr12	Cufflinks	exon	4144774	4144898	.	-	.	gene_id "XLOC_154695"; transcript_id "Novel_002137"; exon_number "3"; gene_type "
Dt_chr12	Cufflinks	exon	4523472	4524603	.	-	.	gene_id "XLOC_154783"; transcript_id "Novel_002138"; exon_number "1"; gene_type "
Dt_chr12	Cufflinks	exon	4525289	4525361	.	-	.	gene_id "XLOC_154783"; transcript_id "Novel_002138"; exon_number "2"; gene_type "
Dt_chr12	Cufflinks	exon	4525584	4525679	.	-	.	gene_id "XLOC_154783"; transcript_id "Novel_002138"; exon_number "3"; gene_type "
Dt_chr12	Cufflinks	exon	4525925	4525988	.	-	.	gene_id "XLOC_154783"; transcript_id "Novel_002138"; exon_number "4"; gene_type "
Dt_chr12	Cufflinks	exon	4526688	4526739	.	-	.	gene_id "XLOC_154783"; transcript_id "Novel_002138"; exon_number "5"; gene_type "
Dt_chr12	Cufflinks	exon	4527282	4527379	.	-	.	gene_id "XLOC_154783"; transcript_id "Novel_002138"; exon_number "6"; gene_type "
Dt_chr12	Cufflinks	exon	4527467	4527525	.	-	.	gene_id "XLOC_154783"; transcript_id "Novel_002138"; exon_number "7"; gene_type "
Dt_chr12	Cufflinks	exon	4527769	4528280	.	-	.	gene_id "XLOC_154783"; transcript_id "Novel_002138"; exon_number "8"; gene_type "
Dt_chr12	Cufflinks	exon	4681334	4682299	.	-	.	gene_id "XLOC_154804"; transcript_id "Novel_002139"; exon_number "1"; gene_type "
Dt_chr12	Cufflinks	exon	5280594	5280930	.	-	.	gene_id "XLOC_154876"; transcript_id "Novel_002140"; exon_number "1"; gene_type "
Dt_chr12	Cufflinks	exon	5281005	5281132	.	-	.	gene_id "XLOC_154876"; transcript_id "Novel_002140"; exon_number "2"; gene_type "
Dt_chr12	Cufflinks	exon	7024497	7024671	.	-	.	gene_id "XLOC_155094"; transcript_id "Novel_002141"; exon_number "1"; gene_type "
Dt_chr12	Cufflinks	exon	7024817	7024882	.	-	.	gene_id "XLOC_155094"; transcript_id "Novel_002141"; exon_number "2"; gene_type "
Dt_chr12	Cufflinks	exon	7025022	7025070	.	-	.	gene_id "XLOC_155094"; transcript_id "Novel_002141"; exon_number "3"; gene_type "
Dt_chr12	Cufflinks	exon	7025252	7025308	.	-	.	gene_id "XLOC_155094"; transcript_id "Novel_002141"; exon_number "4"; gene_type "
Dt_chr12	Cufflinks	exon	8328643	8328832	.	-	.	gene_id "XLOC_155214"; transcript_id "Novel_002142"; exon_number "1"; gene_type "
Dt_chr12	Cufflinks	exon	8329680	8329809	.	-	.	gene_id "XLOC_155214"; transcript_id "Novel_002142"; exon_number "2"; gene_type "
Dt_chr12	Cufflinks	exon	8331287	8331595	.	-	.	gene_id "XLOC_155214"; transcript_id "Novel_002142"; exon_number "3"; gene_type "
Dt_chr12	Cufflinks	exon	8329254	8329443	.	-	.	gene_id "XLOC_155214"; transcript_id "Novel_002143"; exon_number "1"; gene_type "
Dt_chr12	Cufflinks	exon	8329680	8329809	.	-	.	gene_id "XLOC_155214"; transcript_id "Novel_002143"; exon_number "2"; gene_type "
Dt_chr12	Cufflinks	exon	8331287	8331595	.	-	.	gene_id "XLOC_155214"; transcript_id "Novel_002143"; exon_number "3"; gene_type "
Dt_chr12	Cufflinks	exon	8543668	8544269	.	-	.	gene_id "XLOC_155243"; transcript_id "Novel_002144"; exon_number "1"; gene_type "
Dt_chr12	Cufflinks	exon	8544365	8544513	.	-	.	gene_id "XLOC_155243"; transcript_id "Novel_002144"; exon_number "2"; gene_type "
Dt_chr12	Cufflinks	exon	8544611	8544696	.	-	.	gene_id "XLOC_155243"; transcript_id "Novel_002144"; exon_number "3"; gene_type "
Dt_chr12	Cufflinks	exon	8544797	8545812	.	-	.	gene_id "XLOC_155243"; transcript_id "Novel_002144"; exon_number "4"; gene_type "

Dt_chr12	Cufflinks	exon	9286614	9286659	.	-	.	gene_id "XLOC_155316"; transcript_id "Novel_002145"; exon_number "1"; gene_type "
Dt_chr12	Cufflinks	exon	9287279	9287561	.	-	.	gene_id "XLOC_155316"; transcript_id "Novel_002145"; exon_number "2"; gene_type "
Dt_chr12	Cufflinks	exon	10178649	10179401	.	-	.	gene_id "XLOC_155397"; transcript_id "Novel_002146"; exon_number "1"; gene_type "
Dt_chr12	Cufflinks	exon	10179529	10179996	.	-	.	gene_id "XLOC_155397"; transcript_id "Novel_002146"; exon_number "2"; gene_type "
Dt_chr12	Cufflinks	exon	10656161	10657099	.	-	.	gene_id "XLOC_155471"; transcript_id "Novel_002147"; exon_number "1"; gene_type "
Dt_chr12	Cufflinks	exon	10657197	10657309	.	-	.	gene_id "XLOC_155471"; transcript_id "Novel_002147"; exon_number "2"; gene_type "
Dt_chr12	Cufflinks	exon	12862611	12862836	.	-	.	gene_id "XLOC_155732"; transcript_id "Novel_002148"; exon_number "1"; gene_type "
Dt_chr12	Cufflinks	exon	12863079	12863163	.	-	.	gene_id "XLOC_155732"; transcript_id "Novel_002148"; exon_number "2"; gene_type "
Dt_chr12	Cufflinks	exon	12863287	12863379	.	-	.	gene_id "XLOC_155732"; transcript_id "Novel_002148"; exon_number "3"; gene_type "
Dt_chr12	Cufflinks	exon	12863517	12863616	.	-	.	gene_id "XLOC_155732"; transcript_id "Novel_002148"; exon_number "4"; gene_type "
Dt_chr12	Cufflinks	exon	12865727	12866016	.	-	.	gene_id "XLOC_155732"; transcript_id "Novel_002148"; exon_number "5"; gene_type "
Dt_chr12	Cufflinks	exon	12862972	12863379	.	-	.	gene_id "XLOC_155732"; transcript_id "Novel_002149"; exon_number "1"; gene_type "
Dt_chr12	Cufflinks	exon	12863517	12863616	.	-	.	gene_id "XLOC_155732"; transcript_id "Novel_002149"; exon_number "2"; gene_type "
Dt_chr12	Cufflinks	exon	12865727	12865890	.	-	.	gene_id "XLOC_155732"; transcript_id "Novel_002149"; exon_number "3"; gene_type "
Dt_chr12	Cufflinks	exon	15458340	15458595	.	-	.	gene_id "XLOC_155885"; transcript_id "Novel_002150"; exon_number "1"; gene_type "
Dt_chr12	Cufflinks	exon	15459581	15459684	.	-	.	gene_id "XLOC_155885"; transcript_id "Novel_002150"; exon_number "2"; gene_type "
Dt_chr12	Cufflinks	exon	15459809	15460222	.	-	.	gene_id "XLOC_155885"; transcript_id "Novel_002150"; exon_number "3"; gene_type "
Dt_chr12	Cufflinks	exon	15471236	15474604	.	-	.	gene_id "XLOC_155892"; transcript_id "Novel_002151"; exon_number "1"; gene_type "
Dt_chr12	Cufflinks	exon	15477083	15477315	.	-	.	gene_id "XLOC_155892"; transcript_id "Novel_002151"; exon_number "2"; gene_type "
Dt_chr12	Cufflinks	exon	16980380	16982454	.	-	.	gene_id "XLOC_156005"; transcript_id "Novel_002152"; exon_number "1"; gene_type "
Dt_chr12	Cufflinks	exon	17407899	17410319	.	-	.	gene_id "XLOC_156040"; transcript_id "Novel_002153"; exon_number "1"; gene_type "
Dt_chr12	Cufflinks	exon	17411234	17411278	.	-	.	gene_id "XLOC_156040"; transcript_id "Novel_002153"; exon_number "2"; gene_type "
Dt_chr12	Cufflinks	exon	17412593	17412743	.	-	.	gene_id "XLOC_156040"; transcript_id "Novel_002153"; exon_number "3"; gene_type "
Dt_chr12	Cufflinks	exon	17953842	17954364	.	-	.	gene_id "XLOC_156087"; transcript_id "Novel_002154"; exon_number "1"; gene_type "
Dt_chr12	Cufflinks	exon	17954742	17954803	.	-	.	gene_id "XLOC_156087"; transcript_id "Novel_002154"; exon_number "2"; gene_type "
Dt_chr12	Cufflinks	exon	17955112	17955381	.	-	.	gene_id "XLOC_156087"; transcript_id "Novel_002154"; exon_number "3"; gene_type "
Dt_chr12	Cufflinks	exon	18726978	18727077	.	-	.	gene_id "XLOC_156114"; transcript_id "Novel_002155"; exon_number "1"; gene_type "
Dt_chr12	Cufflinks	exon	18727234	18727722	.	-	.	gene_id "XLOC_156114"; transcript_id "Novel_002155"; exon_number "2"; gene_type "
Dt_chr12	Cufflinks	exon	24213659	24213868	.	-	.	gene_id "XLOC_156403"; transcript_id "Novel_002156"; exon_number "1"; gene_type "
Dt_chr12	Cufflinks	exon	28942910	28944054	.	-	.	gene_id "XLOC_156710"; transcript_id "Novel_002157"; exon_number "1"; gene_type "
Dt_chr12	Cufflinks	exon	28944957	28945038	.	-	.	gene_id "XLOC_156710"; transcript_id "Novel_002157"; exon_number "2"; gene_type "
Dt_chr12	Cufflinks	exon	28945154	28945228	.	-	.	gene_id "XLOC_156710"; transcript_id "Novel_002157"; exon_number "3"; gene_type "
Dt_chr12	Cufflinks	exon	28945998	28946086	.	-	.	gene_id "XLOC_156710"; transcript_id "Novel_002157"; exon_number "4"; gene_type "
Dt_chr12	Cufflinks	exon	29080335	29081131	.	-	.	gene_id "XLOC_156733"; transcript_id "Novel_002158"; exon_number "1"; gene_type "
Dt_chr12	Cufflinks	exon	29081225	29081767	.	-	.	gene_id "XLOC_156733"; transcript_id "Novel_002158"; exon_number "2"; gene_type "

Dt_chr12	Cufflinks	exon	29139298	29139422	.	-	.	gene_id "XLOC_156748"; transcript_id "Novel_002159"; exon_number "1"; gene_type "
Dt_chr12	Cufflinks	exon	29139765	29139854	.	-	.	gene_id "XLOC_156748"; transcript_id "Novel_002159"; exon_number "2"; gene_type "
Dt_chr12	Cufflinks	exon	29140277	29140411	.	-	.	gene_id "XLOC_156748"; transcript_id "Novel_002159"; exon_number "3"; gene_type "
Dt_chr12	Cufflinks	exon	29140532	29140606	.	-	.	gene_id "XLOC_156748"; transcript_id "Novel_002159"; exon_number "4"; gene_type "
Dt_chr12	Cufflinks	exon	29141196	29141295	.	-	.	gene_id "XLOC_156748"; transcript_id "Novel_002159"; exon_number "5"; gene_type "
Dt_chr12	Cufflinks	exon	29141658	29141818	.	-	.	gene_id "XLOC_156748"; transcript_id "Novel_002159"; exon_number "6"; gene_type "
Dt_chr12	Cufflinks	exon	29141946	29142093	.	-	.	gene_id "XLOC_156748"; transcript_id "Novel_002159"; exon_number "7"; gene_type "
Dt_chr12	Cufflinks	exon	29142298	29142697	.	-	.	gene_id "XLOC_156748"; transcript_id "Novel_002159"; exon_number "8"; gene_type "
Dt_chr12	Cufflinks	exon	29143218	29143269	.	-	.	gene_id "XLOC_156748"; transcript_id "Novel_002159"; exon_number "9"; gene_type "
Dt_chr12	Cufflinks	exon	29139306	29139422	.	-	.	gene_id "XLOC_156748"; transcript_id "Novel_002160"; exon_number "1"; gene_type "
Dt_chr12	Cufflinks	exon	29139765	29139854	.	-	.	gene_id "XLOC_156748"; transcript_id "Novel_002160"; exon_number "2"; gene_type "
Dt_chr12	Cufflinks	exon	29140277	29140411	.	-	.	gene_id "XLOC_156748"; transcript_id "Novel_002160"; exon_number "3"; gene_type "
Dt_chr12	Cufflinks	exon	29140532	29140606	.	-	.	gene_id "XLOC_156748"; transcript_id "Novel_002160"; exon_number "4"; gene_type "
Dt_chr12	Cufflinks	exon	29141196	29141295	.	-	.	gene_id "XLOC_156748"; transcript_id "Novel_002160"; exon_number "5"; gene_type "
Dt_chr12	Cufflinks	exon	29141658	29142093	.	-	.	gene_id "XLOC_156748"; transcript_id "Novel_002160"; exon_number "6"; gene_type "
Dt_chr12	Cufflinks	exon	29142298	29142697	.	-	.	gene_id "XLOC_156748"; transcript_id "Novel_002160"; exon_number "7"; gene_type "
Dt_chr12	Cufflinks	exon	29143218	29143284	.	-	.	gene_id "XLOC_156748"; transcript_id "Novel_002160"; exon_number "8"; gene_type "
Dt_chr12	Cufflinks	exon	29143421	29143639	.	-	.	gene_id "XLOC_156748"; transcript_id "Novel_002160"; exon_number "9"; gene_type "
Dt_chr12	Cufflinks	exon	29143997	29144138	.	-	.	gene_id "XLOC_156748"; transcript_id "Novel_002160"; exon_number "10"; gene_type
Dt_chr12	Cufflinks	exon	29144300	29144361	.	-	.	gene_id "XLOC_156748"; transcript_id "Novel_002160"; exon_number "11"; gene_type
Dt_chr12	Cufflinks	exon	29144584	29144656	.	-	.	gene_id "XLOC_156748"; transcript_id "Novel_002160"; exon_number "12"; gene_type
Dt_chr12	Cufflinks	exon	29144881	29144990	.	-	.	gene_id "XLOC_156748"; transcript_id "Novel_002160"; exon_number "13"; gene_type
Dt_chr12	Cufflinks	exon	29145086	29145450	.	-	.	gene_id "XLOC_156748"; transcript_id "Novel_002160"; exon_number "14"; gene_type
Dt_chr12	Cufflinks	exon	29143360	29143639	.	-	.	gene_id "XLOC_156748"; transcript_id "Novel_002161"; exon_number "1"; gene_type "
Dt_chr12	Cufflinks	exon	29143997	29144138	.	-	.	gene_id "XLOC_156748"; transcript_id "Novel_002161"; exon_number "2"; gene_type "
Dt_chr12	Cufflinks	exon	29144300	29144361	.	-	.	gene_id "XLOC_156748"; transcript_id "Novel_002161"; exon_number "3"; gene_type "
Dt_chr12	Cufflinks	exon	29144584	29144656	.	-	.	gene_id "XLOC_156748"; transcript_id "Novel_002161"; exon_number "4"; gene_type "
Dt_chr12	Cufflinks	exon	29144881	29144990	.	-	.	gene_id "XLOC_156748"; transcript_id "Novel_002161"; exon_number "5"; gene_type "
Dt_chr12	Cufflinks	exon	29145086	29145487	.	-	.	gene_id "XLOC_156748"; transcript_id "Novel_002161"; exon_number "6"; gene_type "
Dt_chr12	Cufflinks	exon	29187643	29187823	.	-	.	gene_id "XLOC_156754"; transcript_id "Novel_002162"; exon_number "1"; gene_type "
Dt_chr12	Cufflinks	exon	29187921	29188016	.	-	.	gene_id "XLOC_156754"; transcript_id "Novel_002162"; exon_number "2"; gene_type "
Dt_chr12	Cufflinks	exon	29337805	29338400	.	-	.	gene_id "XLOC_156772"; transcript_id "Novel_002163"; exon_number "1"; gene_type "
Dt_chr12	Cufflinks	exon	29339281	29339388	.	-	.	gene_id "XLOC_156772"; transcript_id "Novel_002163"; exon_number "2"; gene_type "
Dt_chr12	Cufflinks	exon	29339556	29340658	.	-	.	gene_id "XLOC_156772"; transcript_id "Novel_002163"; exon_number "3"; gene_type "
Dt_chr12	Cufflinks	exon	29337818	29338400	.	-	.	gene_id "XLOC_156772"; transcript_id "Novel_002164"; exon_number "1"; gene_type "

Dt_chr12	Cufflinks	exon	29339281	29339388	.	-	.	gene_id "XLOC_156772"; transcript_id "Novel_002164"; exon_number "2"; gene_type "
Dt_chr12	Cufflinks	exon	29339556	29339841	.	-	.	gene_id "XLOC_156772"; transcript_id "Novel_002164"; exon_number "3"; gene_type "
Dt_chr12	Cufflinks	exon	29339923	29340825	.	-	.	gene_id "XLOC_156772"; transcript_id "Novel_002164"; exon_number "4"; gene_type "
Dt_chr12	Cufflinks	exon	31163554	31165955	.	-	.	gene_id "XLOC_156949"; transcript_id "Novel_002165"; exon_number "1"; gene_type "
Dt_chr12	Cufflinks	exon	31166219	31166450	.	-	.	gene_id "XLOC_156949"; transcript_id "Novel_002165"; exon_number "2"; gene_type "
Dt_chr12	Cufflinks	exon	31164232	31165150	.	-	.	gene_id "XLOC_156949"; transcript_id "Novel_002166"; exon_number "1"; gene_type "
Dt_chr12	Cufflinks	exon	31165870	31165955	.	-	.	gene_id "XLOC_156949"; transcript_id "Novel_002166"; exon_number "2"; gene_type "
Dt_chr12	Cufflinks	exon	31166219	31166394	.	-	.	gene_id "XLOC_156949"; transcript_id "Novel_002166"; exon_number "3"; gene_type "
Dt_chr12	Cufflinks	exon	31536708	31537817	.	-	.	gene_id "XLOC_156978"; transcript_id "Novel_002167"; exon_number "1"; gene_type "
Dt_chr12	Cufflinks	exon	31538642	31538734	.	-	.	gene_id "XLOC_156978"; transcript_id "Novel_002167"; exon_number "2"; gene_type "
Dt_chr12	Cufflinks	exon	31538822	31538927	.	-	.	gene_id "XLOC_156978"; transcript_id "Novel_002167"; exon_number "3"; gene_type "
Dt_chr12	Cufflinks	exon	31539100	31539187	.	-	.	gene_id "XLOC_156978"; transcript_id "Novel_002167"; exon_number "4"; gene_type "
Dt_chr12	Cufflinks	exon	31539286	31539382	.	-	.	gene_id "XLOC_156978"; transcript_id "Novel_002167"; exon_number "5"; gene_type "
Dt_chr12	Cufflinks	exon	31539949	31540032	.	-	.	gene_id "XLOC_156978"; transcript_id "Novel_002167"; exon_number "6"; gene_type "
Dt_chr12	Cufflinks	exon	31540175	31540262	.	-	.	gene_id "XLOC_156978"; transcript_id "Novel_002167"; exon_number "7"; gene_type "
Dt_chr12	Cufflinks	exon	31540922	31541015	.	-	.	gene_id "XLOC_156978"; transcript_id "Novel_002167"; exon_number "8"; gene_type "
Dt_chr12	Cufflinks	exon	31541104	31541212	.	-	.	gene_id "XLOC_156978"; transcript_id "Novel_002167"; exon_number "9"; gene_type "
Dt_chr12	Cufflinks	exon	31541300	31541885	.	-	.	gene_id "XLOC_156978"; transcript_id "Novel_002167"; exon_number "10"; gene_type
Dt_chr12	Cufflinks	exon	31536771	31537817	.	-	.	gene_id "XLOC_156978"; transcript_id "Novel_002168"; exon_number "1"; gene_type "
Dt_chr12	Cufflinks	exon	31538642	31538734	.	-	.	gene_id "XLOC_156978"; transcript_id "Novel_002168"; exon_number "2"; gene_type "
Dt_chr12	Cufflinks	exon	31538822	31538927	.	-	.	gene_id "XLOC_156978"; transcript_id "Novel_002168"; exon_number "3"; gene_type "
Dt_chr12	Cufflinks	exon	31539100	31539187	.	-	.	gene_id "XLOC_156978"; transcript_id "Novel_002168"; exon_number "4"; gene_type "
Dt_chr12	Cufflinks	exon	31539286	31539382	.	-	.	gene_id "XLOC_156978"; transcript_id "Novel_002168"; exon_number "5"; gene_type "
Dt_chr12	Cufflinks	exon	31539949	31540032	.	-	.	gene_id "XLOC_156978"; transcript_id "Novel_002168"; exon_number "6"; gene_type "
Dt_chr12	Cufflinks	exon	31540175	31540262	.	-	.	gene_id "XLOC_156978"; transcript_id "Novel_002168"; exon_number "7"; gene_type "
Dt_chr12	Cufflinks	exon	31540922	31541015	.	-	.	gene_id "XLOC_156978"; transcript_id "Novel_002168"; exon_number "8"; gene_type "
Dt_chr12	Cufflinks	exon	31541104	31541212	.	-	.	gene_id "XLOC_156978"; transcript_id "Novel_002168"; exon_number "9"; gene_type "
Dt_chr12	Cufflinks	exon	31541300	31541604	.	-	.	gene_id "XLOC_156978"; transcript_id "Novel_002168"; exon_number "10"; gene_type
Dt_chr12	Cufflinks	exon	31542060	31542550	.	-	.	gene_id "XLOC_156978"; transcript_id "Novel_002168"; exon_number "11"; gene_type
Dt_chr12	Cufflinks	exon	36915086	36918542	.	-	.	gene_id "XLOC_157259"; transcript_id "Novel_002169"; exon_number "1"; gene_type "
Dt_chr12	Cufflinks	exon	36924304	36924522	.	-	.	gene_id "XLOC_157259"; transcript_id "Novel_002169"; exon_number "2"; gene_type "
Dt_chr13	Cufflinks	exon	1487276	1487512	.	+	.	gene_id "XLOC_157503"; transcript_id "Novel_002170"; exon_number "1"; gene_type "
Dt_chr13	Cufflinks	exon	3606060	3606755	.	+	.	gene_id "XLOC_157584"; transcript_id "Novel_002171"; exon_number "1"; gene_type "
Dt_chr13	Cufflinks	exon	3607042	3607081	.	+	.	gene_id "XLOC_157584"; transcript_id "Novel_002171"; exon_number "2"; gene_type "
Dt_chr13	Cufflinks	exon	3607211	3607750	.	+	.	gene_id "XLOC_157584"; transcript_id "Novel_002171"; exon_number "3"; gene_type "

Dt_chr13	Cufflinks	exon	7092842	7093116	.	+	.	gene_id "XLOC_157722"; transcript_id "Novel_002172"; exon_number "1"; gene_type "
Dt_chr13	Cufflinks	exon	7093445	7096180	.	+	.	gene_id "XLOC_157722"; transcript_id "Novel_002172"; exon_number "2"; gene_type "
Dt_chr13	Cufflinks	exon	7092847	7093528	.	+	.	gene_id "XLOC_157722"; transcript_id "Novel_002173"; exon_number "1"; gene_type "
Dt_chr13	Cufflinks	exon	7094173	7096215	.	+	.	gene_id "XLOC_157722"; transcript_id "Novel_002173"; exon_number "2"; gene_type "
Dt_chr13	Cufflinks	exon	7327513	7328519	.	+	.	gene_id "XLOC_157728"; transcript_id "Novel_002174"; exon_number "1"; gene_type "
Dt_chr13	Cufflinks	exon	7328959	7331031	.	+	.	gene_id "XLOC_157728"; transcript_id "Novel_002174"; exon_number "2"; gene_type "
Dt_chr13	Cufflinks	exon	7327515	7328519	.	+	.	gene_id "XLOC_157728"; transcript_id "Novel_002175"; exon_number "1"; gene_type "
Dt_chr13	Cufflinks	exon	7328964	7331037	.	+	.	gene_id "XLOC_157728"; transcript_id "Novel_002175"; exon_number "2"; gene_type "
Dt_chr13	Cufflinks	exon	7327525	7327788	.	+	.	gene_id "XLOC_157728"; transcript_id "Novel_002176"; exon_number "1"; gene_type "
Dt_chr13	Cufflinks	exon	7328217	7331670	.	+	.	gene_id "XLOC_157728"; transcript_id "Novel_002176"; exon_number "2"; gene_type "
Dt_chr13	Cufflinks	exon	9797026	9798706	.	+	.	gene_id "XLOC_157993"; transcript_id "Novel_002177"; exon_number "1"; gene_type "
Dt_chr13	Cufflinks	exon	13197507	13197566	.	+	.	gene_id "XLOC_158221"; transcript_id "Novel_002178"; exon_number "1"; gene_type "
Dt_chr13	Cufflinks	exon	13197962	13198132	.	+	.	gene_id "XLOC_158221"; transcript_id "Novel_002178"; exon_number "2"; gene_type "
Dt_chr13	Cufflinks	exon	13198505	13198611	.	+	.	gene_id "XLOC_158221"; transcript_id "Novel_002178"; exon_number "3"; gene_type "
Dt_chr13	Cufflinks	exon	13198721	13198762	.	+	.	gene_id "XLOC_158221"; transcript_id "Novel_002178"; exon_number "4"; gene_type "
Dt_chr13	Cufflinks	exon	13198842	13198929	.	+	.	gene_id "XLOC_158221"; transcript_id "Novel_002178"; exon_number "5"; gene_type "
Dt_chr13	Cufflinks	exon	13199216	13199315	.	+	.	gene_id "XLOC_158221"; transcript_id "Novel_002178"; exon_number "6"; gene_type "
Dt_chr13	Cufflinks	exon	13199457	13199557	.	+	.	gene_id "XLOC_158221"; transcript_id "Novel_002178"; exon_number "7"; gene_type "
Dt_chr13	Cufflinks	exon	13199654	13199927	.	+	.	gene_id "XLOC_158221"; transcript_id "Novel_002178"; exon_number "8"; gene_type "
Dt_chr13	Cufflinks	exon	13197511	13197566	.	+	.	gene_id "XLOC_158221"; transcript_id "Novel_002179"; exon_number "1"; gene_type "
Dt_chr13	Cufflinks	exon	13197966	13198132	.	+	.	gene_id "XLOC_158221"; transcript_id "Novel_002179"; exon_number "2"; gene_type "
Dt_chr13	Cufflinks	exon	13198505	13198611	.	+	.	gene_id "XLOC_158221"; transcript_id "Novel_002179"; exon_number "3"; gene_type "
Dt_chr13	Cufflinks	exon	13198721	13198762	.	+	.	gene_id "XLOC_158221"; transcript_id "Novel_002179"; exon_number "4"; gene_type "
Dt_chr13	Cufflinks	exon	13198842	13198929	.	+	.	gene_id "XLOC_158221"; transcript_id "Novel_002179"; exon_number "5"; gene_type "
Dt_chr13	Cufflinks	exon	13199216	13199315	.	+	.	gene_id "XLOC_158221"; transcript_id "Novel_002179"; exon_number "6"; gene_type "
Dt_chr13	Cufflinks	exon	13199457	13199557	.	+	.	gene_id "XLOC_158221"; transcript_id "Novel_002179"; exon_number "7"; gene_type "
Dt_chr13	Cufflinks	exon	13199654	13200331	.	+	.	gene_id "XLOC_158221"; transcript_id "Novel_002179"; exon_number "8"; gene_type "
Dt_chr13	Cufflinks	exon	13197519	13198132	.	+	.	gene_id "XLOC_158221"; transcript_id "Novel_002180"; exon_number "1"; gene_type "
Dt_chr13	Cufflinks	exon	13198505	13198611	.	+	.	gene_id "XLOC_158221"; transcript_id "Novel_002180"; exon_number "2"; gene_type "
Dt_chr13	Cufflinks	exon	13198721	13198762	.	+	.	gene_id "XLOC_158221"; transcript_id "Novel_002180"; exon_number "3"; gene_type "
Dt_chr13	Cufflinks	exon	13198842	13198929	.	+	.	gene_id "XLOC_158221"; transcript_id "Novel_002180"; exon_number "4"; gene_type "
Dt_chr13	Cufflinks	exon	13199216	13199315	.	+	.	gene_id "XLOC_158221"; transcript_id "Novel_002180"; exon_number "5"; gene_type "
Dt_chr13	Cufflinks	exon	13199457	13199557	.	+	.	gene_id "XLOC_158221"; transcript_id "Novel_002180"; exon_number "6"; gene_type "
Dt_chr13	Cufflinks	exon	13199654	13199942	.	+	.	gene_id "XLOC_158221"; transcript_id "Novel_002180"; exon_number "7"; gene_type "
Dt_chr13	Cufflinks	exon	16655416	16655687	.	+	.	gene_id "XLOC_158440"; transcript_id "Novel_002181"; exon_number "1"; gene_type "

Dt_chr13	Cufflinks	exon	16655789	16655916	.	+	.	gene_id "XLOC_158440"; transcript_id "Novel_002181"; exon_number "2"; gene_type "
Dt_chr13	Cufflinks	exon	16656058	16656300	.	+	.	gene_id "XLOC_158440"; transcript_id "Novel_002181"; exon_number "3"; gene_type "
Dt_chr13	Cufflinks	exon	16656421	16656673	.	+	.	gene_id "XLOC_158440"; transcript_id "Novel_002181"; exon_number "4"; gene_type "
Dt_chr13	Cufflinks	exon	16659689	16660273	.	+	.	gene_id "XLOC_158440"; transcript_id "Novel_002181"; exon_number "5"; gene_type "
Dt_chr13	Cufflinks	exon	16720388	16720616	.	+	.	gene_id "XLOC_158453"; transcript_id "Novel_002182"; exon_number "1"; gene_type "
Dt_chr13	Cufflinks	exon	16720957	16721029	.	+	.	gene_id "XLOC_158453"; transcript_id "Novel_002182"; exon_number "2"; gene_type "
Dt_chr13	Cufflinks	exon	16721356	16721383	.	+	.	gene_id "XLOC_158453"; transcript_id "Novel_002182"; exon_number "3"; gene_type "
Dt_chr13	Cufflinks	exon	18158900	18159257	.	+	.	gene_id "XLOC_158596"; transcript_id "Novel_002183"; exon_number "1"; gene_type "
Dt_chr13	Cufflinks	exon	19707101	19707528	.	+	.	gene_id "XLOC_158784"; transcript_id "Novel_002184"; exon_number "1"; gene_type "
Dt_chr13	Cufflinks	exon	19709302	19709416	.	+	.	gene_id "XLOC_158784"; transcript_id "Novel_002184"; exon_number "2"; gene_type "
Dt_chr13	Cufflinks	exon	19709510	19709591	.	+	.	gene_id "XLOC_158784"; transcript_id "Novel_002184"; exon_number "3"; gene_type "
Dt_chr13	Cufflinks	exon	19709801	19709878	.	+	.	gene_id "XLOC_158784"; transcript_id "Novel_002184"; exon_number "4"; gene_type "
Dt_chr13	Cufflinks	exon	19710069	19710939	.	+	.	gene_id "XLOC_158784"; transcript_id "Novel_002184"; exon_number "5"; gene_type "
Dt_chr13	Cufflinks	exon	19707102	19707528	.	+	.	gene_id "XLOC_158784"; transcript_id "Novel_002185"; exon_number "1"; gene_type "
Dt_chr13	Cufflinks	exon	19709302	19709416	.	+	.	gene_id "XLOC_158784"; transcript_id "Novel_002185"; exon_number "2"; gene_type "
Dt_chr13	Cufflinks	exon	19709510	19709591	.	+	.	gene_id "XLOC_158784"; transcript_id "Novel_002185"; exon_number "3"; gene_type "
Dt_chr13	Cufflinks	exon	19709827	19709878	.	+	.	gene_id "XLOC_158784"; transcript_id "Novel_002185"; exon_number "4"; gene_type "
Dt_chr13	Cufflinks	exon	19710069	19711454	.	+	.	gene_id "XLOC_158784"; transcript_id "Novel_002185"; exon_number "5"; gene_type "
Dt_chr13	Cufflinks	exon	22038220	22038566	.	+	.	gene_id "XLOC_159043"; transcript_id "Novel_002186"; exon_number "1"; gene_type "
Dt_chr13	Cufflinks	exon	22040415	22040859	.	+	.	gene_id "XLOC_159043"; transcript_id "Novel_002186"; exon_number "2"; gene_type "
Dt_chr13	Cufflinks	exon	22854689	22855118	.	+	.	gene_id "XLOC_159188"; transcript_id "Novel_002187"; exon_number "1"; gene_type "
Dt_chr13	Cufflinks	exon	22855373	22855847	.	+	.	gene_id "XLOC_159188"; transcript_id "Novel_002187"; exon_number "2"; gene_type "
Dt_chr13	Cufflinks	exon	22856178	22856336	.	+	.	gene_id "XLOC_159188"; transcript_id "Novel_002187"; exon_number "3"; gene_type "
Dt_chr13	Cufflinks	exon	22854717	22855118	.	+	.	gene_id "XLOC_159188"; transcript_id "Novel_002188"; exon_number "1"; gene_type "
Dt_chr13	Cufflinks	exon	22855373	22855963	.	+	.	gene_id "XLOC_159188"; transcript_id "Novel_002188"; exon_number "2"; gene_type "
Dt_chr13	Cufflinks	exon	22854717	22855118	.	+	.	gene_id "XLOC_159188"; transcript_id "Novel_002189"; exon_number "1"; gene_type "
Dt_chr13	Cufflinks	exon	22855373	22855847	.	+	.	gene_id "XLOC_159188"; transcript_id "Novel_002189"; exon_number "2"; gene_type "
Dt_chr13	Cufflinks	exon	22857780	22857835	.	+	.	gene_id "XLOC_159188"; transcript_id "Novel_002189"; exon_number "3"; gene_type "
Dt_chr13	Cufflinks	exon	22857915	22858045	.	+	.	gene_id "XLOC_159188"; transcript_id "Novel_002189"; exon_number "4"; gene_type "
Dt_chr13	Cufflinks	exon	22858135	22858231	.	+	.	gene_id "XLOC_159188"; transcript_id "Novel_002189"; exon_number "5"; gene_type "
Dt_chr13	Cufflinks	exon	22858313	22858480	.	+	.	gene_id "XLOC_159188"; transcript_id "Novel_002189"; exon_number "6"; gene_type "
Dt_chr13	Cufflinks	exon	22859102	22859931	.	+	.	gene_id "XLOC_159188"; transcript_id "Novel_002189"; exon_number "7"; gene_type "
Dt_chr13	Cufflinks	exon	23258291	23258400	.	+	.	gene_id "XLOC_159280"; transcript_id "Novel_002190"; exon_number "1"; gene_type "
Dt_chr13	Cufflinks	exon	23258521	23258582	.	+	.	gene_id "XLOC_159280"; transcript_id "Novel_002190"; exon_number "2"; gene_type "
Dt_chr13	Cufflinks	exon	23259056	23259120	.	+	.	gene_id "XLOC_159280"; transcript_id "Novel_002190"; exon_number "3"; gene_type "

Dt_chr13	Cufflinks	exon	23259353	23261408	.	+	.	gene_id "XLOC_159280"; transcript_id "Novel_002190"; exon_number "4"; gene_type "
Dt_chr13	Cufflinks	exon	23258291	23258400	.	+	.	gene_id "XLOC_159280"; transcript_id "Novel_002191"; exon_number "1"; gene_type "
Dt_chr13	Cufflinks	exon	23258521	23258582	.	+	.	gene_id "XLOC_159280"; transcript_id "Novel_002191"; exon_number "2"; gene_type "
Dt_chr13	Cufflinks	exon	23259353	23259449	.	+	.	gene_id "XLOC_159280"; transcript_id "Novel_002191"; exon_number "3"; gene_type "
Dt_chr13	Cufflinks	exon	23260069	23261538	.	+	.	gene_id "XLOC_159280"; transcript_id "Novel_002191"; exon_number "4"; gene_type "
Dt_chr13	Cufflinks	exon	24385807	24391202	.	+	.	gene_id "XLOC_159389"; transcript_id "Novel_002192"; exon_number "1"; gene_type "
Dt_chr13	Cufflinks	exon	24401809	24401958	.	+	.	gene_id "XLOC_159389"; transcript_id "Novel_002192"; exon_number "2"; gene_type "
Dt_chr13	Cufflinks	exon	24385851	24391202	.	+	.	gene_id "XLOC_159389"; transcript_id "Novel_002193"; exon_number "1"; gene_type "
Dt_chr13	Cufflinks	exon	24391807	24391910	.	+	.	gene_id "XLOC_159389"; transcript_id "Novel_002193"; exon_number "2"; gene_type "
Dt_chr13	Cufflinks	exon	24392103	24392204	.	+	.	gene_id "XLOC_159389"; transcript_id "Novel_002193"; exon_number "3"; gene_type "
Dt_chr13	Cufflinks	exon	24581550	24581747	.	+	.	gene_id "XLOC_159435"; transcript_id "Novel_002194"; exon_number "1"; gene_type "
Dt_chr13	Cufflinks	exon	24582340	24583859	.	+	.	gene_id "XLOC_159435"; transcript_id "Novel_002194"; exon_number "2"; gene_type "
Dt_chr13	Cufflinks	exon	24635456	24635647	.	+	.	gene_id "XLOC_159440"; transcript_id "Novel_002195"; exon_number "1"; gene_type "
Dt_chr13	Cufflinks	exon	24635919	24636436	.	+	.	gene_id "XLOC_159440"; transcript_id "Novel_002195"; exon_number "2"; gene_type "
Dt_chr13	Cufflinks	exon	24962203	24963152	.	+	.	gene_id "XLOC_159452"; transcript_id "Novel_002196"; exon_number "1"; gene_type "
Dt_chr13	Cufflinks	exon	24963234	24963376	.	+	.	gene_id "XLOC_159452"; transcript_id "Novel_002196"; exon_number "2"; gene_type "
Dt_chr13	Cufflinks	exon	25938507	25939117	.	+	.	gene_id "XLOC_159496"; transcript_id "Novel_002197"; exon_number "1"; gene_type "
Dt_chr13	Cufflinks	exon	25939857	25939991	.	+	.	gene_id "XLOC_159496"; transcript_id "Novel_002197"; exon_number "2"; gene_type "
Dt_chr13	Cufflinks	exon	25940705	25940761	.	+	.	gene_id "XLOC_159496"; transcript_id "Novel_002197"; exon_number "3"; gene_type "
Dt_chr13	Cufflinks	exon	25940911	25941045	.	+	.	gene_id "XLOC_159496"; transcript_id "Novel_002197"; exon_number "4"; gene_type "
Dt_chr13	Cufflinks	exon	25941177	25941449	.	+	.	gene_id "XLOC_159496"; transcript_id "Novel_002197"; exon_number "5"; gene_type "
Dt_chr13	Cufflinks	exon	26349688	26349915	.	+	.	gene_id "XLOC_159513"; transcript_id "Novel_002198"; exon_number "1"; gene_type "
Dt_chr13	Cufflinks	exon	26762542	26762965	.	+	.	gene_id "XLOC_159533"; transcript_id "Novel_002199"; exon_number "1"; gene_type "
Dt_chr13	Cufflinks	exon	26763063	26763896	.	+	.	gene_id "XLOC_159533"; transcript_id "Novel_002199"; exon_number "2"; gene_type "
Dt_chr13	Cufflinks	exon	38507898	38508596	.	+	.	gene_id "XLOC_160201"; transcript_id "Novel_002200"; exon_number "1"; gene_type "
Dt_chr13	Cufflinks	exon	38508808	38508901	.	+	.	gene_id "XLOC_160201"; transcript_id "Novel_002200"; exon_number "2"; gene_type "
Dt_chr13	Cufflinks	exon	38509364	38509755	.	+	.	gene_id "XLOC_160201"; transcript_id "Novel_002200"; exon_number "3"; gene_type "
Dt_chr13	Cufflinks	exon	40132495	40132599	.	+	.	gene_id "XLOC_160292"; transcript_id "Novel_002201"; exon_number "1"; gene_type "
Dt_chr13	Cufflinks	exon	40134044	40134137	.	+	.	gene_id "XLOC_160292"; transcript_id "Novel_002201"; exon_number "2"; gene_type "
Dt_chr13	Cufflinks	exon	40134288	40134414	.	+	.	gene_id "XLOC_160292"; transcript_id "Novel_002201"; exon_number "3"; gene_type "
Dt_chr13	Cufflinks	exon	40134937	40135469	.	+	.	gene_id "XLOC_160292"; transcript_id "Novel_002201"; exon_number "4"; gene_type "
Dt_chr13	Cufflinks	exon	40135576	40136806	.	+	.	gene_id "XLOC_160292"; transcript_id "Novel_002201"; exon_number "5"; gene_type "
Dt_chr13	Cufflinks	exon	41669795	41671706	.	+	.	gene_id "XLOC_160358"; transcript_id "Novel_002202"; exon_number "1"; gene_type "
Dt_chr13	Cufflinks	exon	41671793	41673204	.	+	.	gene_id "XLOC_160358"; transcript_id "Novel_002202"; exon_number "2"; gene_type "
Dt_chr13	Cufflinks	exon	42469460	42469591	.	+	.	gene_id "XLOC_160452"; transcript_id "Novel_002203"; exon_number "1"; gene_type "

Dt_chr13	Cufflinks	exon	42469683	42469762	.	+	.	gene_id "XLOC_160452"; transcript_id "Novel_002203"; exon_number "2"; gene_type "
Dt_chr13	Cufflinks	exon	45957336	45957569	.	+	.	gene_id "XLOC_160716"; transcript_id "Novel_002204"; exon_number "1"; gene_type "
Dt_chr13	Cufflinks	exon	48447206	48447389	.	+	.	gene_id "XLOC_160862"; transcript_id "Novel_002205"; exon_number "1"; gene_type "
Dt_chr13	Cufflinks	exon	48447901	48448022	.	+	.	gene_id "XLOC_160862"; transcript_id "Novel_002205"; exon_number "2"; gene_type "
Dt_chr13	Cufflinks	exon	48448110	48448214	.	+	.	gene_id "XLOC_160862"; transcript_id "Novel_002205"; exon_number "3"; gene_type "
Dt_chr13	Cufflinks	exon	48448887	48449122	.	+	.	gene_id "XLOC_160862"; transcript_id "Novel_002205"; exon_number "4"; gene_type "
Dt_chr13	Cufflinks	exon	51629885	51629986	.	+	.	gene_id "XLOC_161178"; transcript_id "Novel_002206"; exon_number "1"; gene_type "
Dt_chr13	Cufflinks	exon	51630070	51630201	.	+	.	gene_id "XLOC_161178"; transcript_id "Novel_002206"; exon_number "2"; gene_type "
Dt_chr13	Cufflinks	exon	51630563	51630705	.	+	.	gene_id "XLOC_161178"; transcript_id "Novel_002206"; exon_number "3"; gene_type "
Dt_chr13	Cufflinks	exon	52280654	52284769	.	+	.	gene_id "XLOC_161253"; transcript_id "Novel_002207"; exon_number "1"; gene_type "
Dt_chr13	Cufflinks	exon	52285845	52286591	.	+	.	gene_id "XLOC_161253"; transcript_id "Novel_002207"; exon_number "2"; gene_type "
Dt_chr13	Cufflinks	exon	53234561	53234768	.	+	.	gene_id "XLOC_161345"; transcript_id "Novel_002208"; exon_number "1"; gene_type "
Dt_chr13	Cufflinks	exon	53235028	53235313	.	+	.	gene_id "XLOC_161345"; transcript_id "Novel_002208"; exon_number "2"; gene_type "
Dt_chr13	Cufflinks	exon	53235509	53235867	.	+	.	gene_id "XLOC_161345"; transcript_id "Novel_002208"; exon_number "3"; gene_type "
Dt_chr13	Cufflinks	exon	53235967	53236338	.	+	.	gene_id "XLOC_161345"; transcript_id "Novel_002208"; exon_number "4"; gene_type "
Dt_chr13	Cufflinks	exon	54175473	54175511	.	+	.	gene_id "XLOC_161463"; transcript_id "Novel_002209"; exon_number "1"; gene_type "
Dt_chr13	Cufflinks	exon	54176468	54176824	.	+	.	gene_id "XLOC_161463"; transcript_id "Novel_002209"; exon_number "2"; gene_type "
Dt_chr13	Cufflinks	exon	55179466	55179655	.	+	.	gene_id "XLOC_161614"; transcript_id "Novel_002210"; exon_number "1"; gene_type "
Dt_chr13	Cufflinks	exon	55179749	55179803	.	+	.	gene_id "XLOC_161614"; transcript_id "Novel_002210"; exon_number "2"; gene_type "
Dt_chr13	Cufflinks	exon	55180609	55180855	.	+	.	gene_id "XLOC_161614"; transcript_id "Novel_002210"; exon_number "3"; gene_type "
Dt_chr13	Cufflinks	exon	55181995	55182061	.	+	.	gene_id "XLOC_161616"; transcript_id "Novel_002211"; exon_number "1"; gene_type "
Dt_chr13	Cufflinks	exon	55182239	55182298	.	+	.	gene_id "XLOC_161616"; transcript_id "Novel_002211"; exon_number "2"; gene_type "
Dt_chr13	Cufflinks	exon	55182650	55182705	.	+	.	gene_id "XLOC_161616"; transcript_id "Novel_002211"; exon_number "3"; gene_type "
Dt_chr13	Cufflinks	exon	55182881	55182986	.	+	.	gene_id "XLOC_161616"; transcript_id "Novel_002211"; exon_number "4"; gene_type "
Dt_chr13	Cufflinks	exon	55183068	55183136	.	+	.	gene_id "XLOC_161616"; transcript_id "Novel_002211"; exon_number "5"; gene_type "
Dt_chr13	Cufflinks	exon	55183249	55183696	.	+	.	gene_id "XLOC_161616"; transcript_id "Novel_002211"; exon_number "6"; gene_type "
Dt_chr13	Cufflinks	exon	3361613	3363339	.	-	.	gene_id "XLOC_161893"; transcript_id "Novel_002212"; exon_number "1"; gene_type "
Dt_chr13	Cufflinks	exon	3363429	3363900	.	-	.	gene_id "XLOC_161893"; transcript_id "Novel_002212"; exon_number "2"; gene_type "
Dt_chr13	Cufflinks	exon	7079922	7080752	.	-	.	gene_id "XLOC_162035"; transcript_id "Novel_002213"; exon_number "1"; gene_type "
Dt_chr13	Cufflinks	exon	7080914	7081334	.	-	.	gene_id "XLOC_162035"; transcript_id "Novel_002213"; exon_number "2"; gene_type "
Dt_chr13	Cufflinks	exon	7995019	7995224	.	-	.	gene_id "XLOC_162133"; transcript_id "Novel_002214"; exon_number "1"; gene_type "
Dt_chr13	Cufflinks	exon	7995320	7995365	.	-	.	gene_id "XLOC_162133"; transcript_id "Novel_002214"; exon_number "2"; gene_type "
Dt_chr13	Cufflinks	exon	8352324	8352446	.	-	.	gene_id "XLOC_162168"; transcript_id "Novel_002215"; exon_number "1"; gene_type "
Dt_chr13	Cufflinks	exon	8352569	8352651	.	-	.	gene_id "XLOC_162168"; transcript_id "Novel_002215"; exon_number "2"; gene_type "
Dt_chr13	Cufflinks	exon	9577399	9577429	.	-	.	gene_id "XLOC_162292"; transcript_id "Novel_002216"; exon_number "1"; gene_type "

Dt_chr13	Cufflinks	exon	9577534	9577600	.	-	.	gene_id "XLOC_162292"; transcript_id "Novel_002216"; exon_number "2"; gene_type "
Dt_chr13	Cufflinks	exon	9577723	9577850	.	-	.	gene_id "XLOC_162292"; transcript_id "Novel_002216"; exon_number "3"; gene_type "
Dt_chr13	Cufflinks	exon	12777181	12777741	.	-	.	gene_id "XLOC_162489"; transcript_id "Novel_002217"; exon_number "1"; gene_type "
Dt_chr13	Cufflinks	exon	12777974	12779450	.	-	.	gene_id "XLOC_162489"; transcript_id "Novel_002217"; exon_number "2"; gene_type "
Dt_chr13	Cufflinks	exon	12779540	12779578	.	-	.	gene_id "XLOC_162489"; transcript_id "Novel_002217"; exon_number "3"; gene_type "
Dt_chr13	Cufflinks	exon	12779680	12779910	.	-	.	gene_id "XLOC_162489"; transcript_id "Novel_002217"; exon_number "4"; gene_type "
Dt_chr13	Cufflinks	exon	13150186	13150843	.	-	.	gene_id "XLOC_162531"; transcript_id "Novel_002218"; exon_number "1"; gene_type "
Dt_chr13	Cufflinks	exon	13154535	13154787	.	-	.	gene_id "XLOC_162531"; transcript_id "Novel_002218"; exon_number "2"; gene_type "
Dt_chr13	Cufflinks	exon	13551431	13551858	.	-	.	gene_id "XLOC_162575"; transcript_id "Novel_002219"; exon_number "1"; gene_type "
Dt_chr13	Cufflinks	exon	13551992	13552018	.	-	.	gene_id "XLOC_162575"; transcript_id "Novel_002219"; exon_number "2"; gene_type "
Dt_chr13	Cufflinks	exon	13552725	13552828	.	-	.	gene_id "XLOC_162575"; transcript_id "Novel_002219"; exon_number "3"; gene_type "
Dt_chr13	Cufflinks	exon	13553062	13554159	.	-	.	gene_id "XLOC_162575"; transcript_id "Novel_002219"; exon_number "4"; gene_type "
Dt_chr13	Cufflinks	exon	13951496	13952219	.	-	.	gene_id "XLOC_162596"; transcript_id "Novel_002220"; exon_number "1"; gene_type "
Dt_chr13	Cufflinks	exon	13952305	13952504	.	-	.	gene_id "XLOC_162596"; transcript_id "Novel_002220"; exon_number "2"; gene_type "
Dt_chr13	Cufflinks	exon	13952600	13952801	.	-	.	gene_id "XLOC_162596"; transcript_id "Novel_002220"; exon_number "3"; gene_type "
Dt_chr13	Cufflinks	exon	13953392	13955574	.	-	.	gene_id "XLOC_162596"; transcript_id "Novel_002220"; exon_number "4"; gene_type "
Dt_chr13	Cufflinks	exon	13989073	13989656	.	-	.	gene_id "XLOC_162612"; transcript_id "Novel_002221"; exon_number "1"; gene_type "
Dt_chr13	Cufflinks	exon	13990084	13990134	.	-	.	gene_id "XLOC_162612"; transcript_id "Novel_002221"; exon_number "2"; gene_type "
Dt_chr13	Cufflinks	exon	13991178	13991501	.	-	.	gene_id "XLOC_162612"; transcript_id "Novel_002221"; exon_number "3"; gene_type "
Dt_chr13	Cufflinks	exon	14250674	14252071	.	-	.	gene_id "XLOC_162645"; transcript_id "Novel_002222"; exon_number "1"; gene_type "
Dt_chr13	Cufflinks	exon	14252296	14252521	.	-	.	gene_id "XLOC_162645"; transcript_id "Novel_002222"; exon_number "2"; gene_type "
Dt_chr13	Cufflinks	exon	15111082	15112252	.	-	.	gene_id "XLOC_162718"; transcript_id "Novel_002223"; exon_number "1"; gene_type "
Dt_chr13	Cufflinks	exon	15112660	15113063	.	-	.	gene_id "XLOC_162718"; transcript_id "Novel_002223"; exon_number "2"; gene_type "
Dt_chr13	Cufflinks	exon	17038851	17039525	.	-	.	gene_id "XLOC_162869"; transcript_id "Novel_002224"; exon_number "1"; gene_type "
Dt_chr13	Cufflinks	exon	17040464	17040572	.	-	.	gene_id "XLOC_162869"; transcript_id "Novel_002224"; exon_number "2"; gene_type "
Dt_chr13	Cufflinks	exon	17041212	17041566	.	-	.	gene_id "XLOC_162869"; transcript_id "Novel_002224"; exon_number "3"; gene_type "
Dt_chr13	Cufflinks	exon	17039316	17040572	.	-	.	gene_id "XLOC_162869"; transcript_id "Novel_002225"; exon_number "1"; gene_type "
Dt_chr13	Cufflinks	exon	17041212	17041545	.	-	.	gene_id "XLOC_162869"; transcript_id "Novel_002225"; exon_number "2"; gene_type "
Dt_chr13	Cufflinks	exon	17897020	17898822	.	-	.	gene_id "XLOC_162942"; transcript_id "Novel_002226"; exon_number "1"; gene_type "
Dt_chr13	Cufflinks	exon	17898907	17901890	.	-	.	gene_id "XLOC_162942"; transcript_id "Novel_002226"; exon_number "2"; gene_type "
Dt_chr13	Cufflinks	exon	17897020	17897620	.	-	.	gene_id "XLOC_162942"; transcript_id "Novel_002227"; exon_number "1"; gene_type "
Dt_chr13	Cufflinks	exon	17898517	17898609	.	-	.	gene_id "XLOC_162942"; transcript_id "Novel_002227"; exon_number "2"; gene_type "
Dt_chr13	Cufflinks	exon	17898743	17901890	.	-	.	gene_id "XLOC_162942"; transcript_id "Novel_002227"; exon_number "3"; gene_type "
Dt_chr13	Cufflinks	exon	17897020	17897620	.	-	.	gene_id "XLOC_162942"; transcript_id "Novel_002228"; exon_number "1"; gene_type "
Dt_chr13	Cufflinks	exon	17898517	17898609	.	-	.	gene_id "XLOC_162942"; transcript_id "Novel_002228"; exon_number "2"; gene_type "

Dt_chr13	Cufflinks	exon	17898743	17898822	.	-	.	gene_id "XLOC_162942"; transcript_id "Novel_002228"; exon_number "3"; gene_type "
Dt_chr13	Cufflinks	exon	17898907	17901890	.	-	.	gene_id "XLOC_162942"; transcript_id "Novel_002228"; exon_number "4"; gene_type "
Dt_chr13	Cufflinks	exon	17897020	17897620	.	-	.	gene_id "XLOC_162942"; transcript_id "Novel_002229"; exon_number "1"; gene_type "
Dt_chr13	Cufflinks	exon	17898517	17901890	.	-	.	gene_id "XLOC_162942"; transcript_id "Novel_002229"; exon_number "2"; gene_type "
Dt_chr13	Cufflinks	exon	19466777	19469156	.	-	.	gene_id "XLOC_163122"; transcript_id "Novel_002230"; exon_number "1"; gene_type "
Dt_chr13	Cufflinks	exon	19471794	19472048	.	-	.	gene_id "XLOC_163122"; transcript_id "Novel_002230"; exon_number "2"; gene_type "
Dt_chr13	Cufflinks	exon	19731112	19731848	.	-	.	gene_id "XLOC_163162"; transcript_id "Novel_002231"; exon_number "1"; gene_type "
Dt_chr13	Cufflinks	exon	19733103	19733191	.	-	.	gene_id "XLOC_163162"; transcript_id "Novel_002231"; exon_number "2"; gene_type "
Dt_chr13	Cufflinks	exon	19733316	19733535	.	-	.	gene_id "XLOC_163162"; transcript_id "Novel_002231"; exon_number "3"; gene_type "
Dt_chr13	Cufflinks	exon	21088182	21088425	.	-	.	gene_id "XLOC_163266"; transcript_id "Novel_002232"; exon_number "1"; gene_type "
Dt_chr13	Cufflinks	exon	21088702	21088767	.	-	.	gene_id "XLOC_163266"; transcript_id "Novel_002232"; exon_number "2"; gene_type "
Dt_chr13	Cufflinks	exon	21089443	21089528	.	-	.	gene_id "XLOC_163266"; transcript_id "Novel_002232"; exon_number "3"; gene_type "
Dt_chr13	Cufflinks	exon	21090060	21090094	.	-	.	gene_id "XLOC_163266"; transcript_id "Novel_002232"; exon_number "4"; gene_type "
Dt_chr13	Cufflinks	exon	21090196	21090279	.	-	.	gene_id "XLOC_163266"; transcript_id "Novel_002232"; exon_number "5"; gene_type "
Dt_chr13	Cufflinks	exon	21090369	21090437	.	-	.	gene_id "XLOC_163266"; transcript_id "Novel_002232"; exon_number "6"; gene_type "
Dt_chr13	Cufflinks	exon	21091017	21091054	.	-	.	gene_id "XLOC_163266"; transcript_id "Novel_002232"; exon_number "7"; gene_type "
Dt_chr13	Cufflinks	exon	21091543	21091600	.	-	.	gene_id "XLOC_163266"; transcript_id "Novel_002232"; exon_number "8"; gene_type "
Dt_chr13	Cufflinks	exon	22050484	22050718	.	-	.	gene_id "XLOC_163422"; transcript_id "Novel_002233"; exon_number "1"; gene_type "
Dt_chr13	Cufflinks	exon	22051041	22051093	.	-	.	gene_id "XLOC_163422"; transcript_id "Novel_002233"; exon_number "2"; gene_type "
Dt_chr13	Cufflinks	exon	22052301	22052372	.	-	.	gene_id "XLOC_163422"; transcript_id "Novel_002233"; exon_number "3"; gene_type "
Dt_chr13	Cufflinks	exon	22052701	22052782	.	-	.	gene_id "XLOC_163422"; transcript_id "Novel_002233"; exon_number "4"; gene_type "
Dt_chr13	Cufflinks	exon	22052920	22053020	.	-	.	gene_id "XLOC_163422"; transcript_id "Novel_002233"; exon_number "5"; gene_type "
Dt_chr13	Cufflinks	exon	22053353	22053486	.	-	.	gene_id "XLOC_163422"; transcript_id "Novel_002233"; exon_number "6"; gene_type "
Dt_chr13	Cufflinks	exon	22571188	22572204	.	-	.	gene_id "XLOC_163499"; transcript_id "Novel_002234"; exon_number "1"; gene_type "
Dt_chr13	Cufflinks	exon	22572527	22572603	.	-	.	gene_id "XLOC_163499"; transcript_id "Novel_002234"; exon_number "2"; gene_type "
Dt_chr13	Cufflinks	exon	23231955	23232364	.	-	.	gene_id "XLOC_163585"; transcript_id "Novel_002235"; exon_number "1"; gene_type "
Dt_chr13	Cufflinks	exon	23410443	23410863	.	-	.	gene_id "XLOC_163607"; transcript_id "Novel_002236"; exon_number "1"; gene_type "
Dt_chr13	Cufflinks	exon	23411937	23412015	.	-	.	gene_id "XLOC_163607"; transcript_id "Novel_002236"; exon_number "2"; gene_type "
Dt_chr13	Cufflinks	exon	23412318	23412605	.	-	.	gene_id "XLOC_163607"; transcript_id "Novel_002236"; exon_number "3"; gene_type "
Dt_chr13	Cufflinks	exon	23410613	23412015	.	-	.	gene_id "XLOC_163607"; transcript_id "Novel_002237"; exon_number "1"; gene_type "
Dt_chr13	Cufflinks	exon	23412318	23412610	.	-	.	gene_id "XLOC_163607"; transcript_id "Novel_002237"; exon_number "2"; gene_type "
Dt_chr13	Cufflinks	exon	23736445	23738612	.	-	.	gene_id "XLOC_163638"; transcript_id "Novel_002238"; exon_number "1"; gene_type "
Dt_chr13	Cufflinks	exon	23749267	23749369	.	-	.	gene_id "XLOC_163638"; transcript_id "Novel_002238"; exon_number "2"; gene_type "
Dt_chr13	Cufflinks	exon	23750488	23750590	.	-	.	gene_id "XLOC_163638"; transcript_id "Novel_002238"; exon_number "3"; gene_type "
Dt_chr13	Cufflinks	exon	23750980	23751232	.	-	.	gene_id "XLOC_163638"; transcript_id "Novel_002238"; exon_number "4"; gene_type "

Dt_chr13	Cufflinks	exon	23753571	23753686	.	-	.	gene_id "XLOC_163638"; transcript_id "Novel_002238"; exon_number "5"; gene_type "
Dt_chr13	Cufflinks	exon	23753772	23754024	.	-	.	gene_id "XLOC_163638"; transcript_id "Novel_002238"; exon_number "6"; gene_type "
Dt_chr13	Cufflinks	exon	23754124	23754358	.	-	.	gene_id "XLOC_163638"; transcript_id "Novel_002238"; exon_number "7"; gene_type "
Dt_chr13	Cufflinks	exon	23754433	23754508	.	-	.	gene_id "XLOC_163638"; transcript_id "Novel_002238"; exon_number "8"; gene_type "
Dt_chr13	Cufflinks	exon	23754615	23754673	.	-	.	gene_id "XLOC_163638"; transcript_id "Novel_002238"; exon_number "9"; gene_type "
Dt_chr13	Cufflinks	exon	23754758	23754824	.	-	.	gene_id "XLOC_163638"; transcript_id "Novel_002238"; exon_number "10"; gene_type
Dt_chr13	Cufflinks	exon	23754909	23755225	.	-	.	gene_id "XLOC_163638"; transcript_id "Novel_002238"; exon_number "11"; gene_type
Dt_chr13	Cufflinks	exon	23737702	23738612	.	-	.	gene_id "XLOC_163638"; transcript_id "Novel_002239"; exon_number "1"; gene_type "
Dt_chr13	Cufflinks	exon	23749267	23749369	.	-	.	gene_id "XLOC_163638"; transcript_id "Novel_002239"; exon_number "2"; gene_type "
Dt_chr13	Cufflinks	exon	23750488	23750590	.	-	.	gene_id "XLOC_163638"; transcript_id "Novel_002239"; exon_number "3"; gene_type "
Dt_chr13	Cufflinks	exon	23750980	23751405	.	-	.	gene_id "XLOC_163638"; transcript_id "Novel_002239"; exon_number "4"; gene_type "
Dt_chr13	Cufflinks	exon	23748684	23749369	.	-	.	gene_id "XLOC_163638"; transcript_id "Novel_002240"; exon_number "1"; gene_type "
Dt_chr13	Cufflinks	exon	23750488	23750590	.	-	.	gene_id "XLOC_163638"; transcript_id "Novel_002240"; exon_number "2"; gene_type "
Dt_chr13	Cufflinks	exon	23750980	23752122	.	-	.	gene_id "XLOC_163638"; transcript_id "Novel_002240"; exon_number "3"; gene_type "
Dt_chr13	Cufflinks	exon	25059987	25060356	.	-	.	gene_id "XLOC_163752"; transcript_id "Novel_002241"; exon_number "1"; gene_type "
Dt_chr13	Cufflinks	exon	25060436	25061343	.	-	.	gene_id "XLOC_163752"; transcript_id "Novel_002241"; exon_number "2"; gene_type "
Dt_chr13	Cufflinks	exon	25061476	25061530	.	-	.	gene_id "XLOC_163752"; transcript_id "Novel_002241"; exon_number "3"; gene_type "
Dt_chr13	Cufflinks	exon	25930424	25930924	.	-	.	gene_id "XLOC_163795"; transcript_id "Novel_002242"; exon_number "1"; gene_type "
Dt_chr13	Cufflinks	exon	25933212	25933335	.	-	.	gene_id "XLOC_163795"; transcript_id "Novel_002242"; exon_number "2"; gene_type "
Dt_chr13	Cufflinks	exon	25933418	25933738	.	-	.	gene_id "XLOC_163795"; transcript_id "Novel_002242"; exon_number "3"; gene_type "
Dt_chr13	Cufflinks	exon	25930503	25930924	.	-	.	gene_id "XLOC_163795"; transcript_id "Novel_002243"; exon_number "1"; gene_type "
Dt_chr13	Cufflinks	exon	25932602	25932770	.	-	.	gene_id "XLOC_163795"; transcript_id "Novel_002243"; exon_number "2"; gene_type "
Dt_chr13	Cufflinks	exon	25933212	25933335	.	-	.	gene_id "XLOC_163795"; transcript_id "Novel_002243"; exon_number "3"; gene_type "
Dt_chr13	Cufflinks	exon	25933418	25933719	.	-	.	gene_id "XLOC_163795"; transcript_id "Novel_002243"; exon_number "4"; gene_type "
Dt_chr13	Cufflinks	exon	25930503	25930924	.	-	.	gene_id "XLOC_163795"; transcript_id "Novel_002244"; exon_number "1"; gene_type "
Dt_chr13	Cufflinks	exon	25933212	25933719	.	-	.	gene_id "XLOC_163795"; transcript_id "Novel_002244"; exon_number "2"; gene_type "
Dt_chr13	Cufflinks	exon	25930596	25930924	.	-	.	gene_id "XLOC_163795"; transcript_id "Novel_002245"; exon_number "1"; gene_type "
Dt_chr13	Cufflinks	exon	25932602	25932887	.	-	.	gene_id "XLOC_163795"; transcript_id "Novel_002245"; exon_number "2"; gene_type "
Dt_chr13	Cufflinks	exon	25930604	25933335	.	-	.	gene_id "XLOC_163795"; transcript_id "Novel_002246"; exon_number "1"; gene_type "
Dt_chr13	Cufflinks	exon	25933418	25933643	.	-	.	gene_id "XLOC_163795"; transcript_id "Novel_002246"; exon_number "2"; gene_type "
Dt_chr13	Cufflinks	exon	27165382	27165867	.	-	.	gene_id "XLOC_163860"; transcript_id "Novel_002247"; exon_number "1"; gene_type "
Dt_chr13	Cufflinks	exon	27182493	27182704	.	-	.	gene_id "XLOC_163860"; transcript_id "Novel_002247"; exon_number "2"; gene_type "
Dt_chr13	Cufflinks	exon	27879880	27880240	.	-	.	gene_id "XLOC_163921"; transcript_id "Novel_002248"; exon_number "1"; gene_type "
Dt_chr13	Cufflinks	exon	27880579	27880940	.	-	.	gene_id "XLOC_163921"; transcript_id "Novel_002248"; exon_number "2"; gene_type "
Dt_chr13	Cufflinks	exon	27879890	27880240	.	-	.	gene_id "XLOC_163921"; transcript_id "Novel_002249"; exon_number "1"; gene_type "

Dt_chr13	Cufflinks	exon	27880579	27880712	.	-	.	gene_id "XLOC_163921"; transcript_id "Novel_002249"; exon_number "2"; gene_type "
Dt_chr13	Cufflinks	exon	27880800	27880998	.	-	.	gene_id "XLOC_163921"; transcript_id "Novel_002249"; exon_number "3"; gene_type "
Dt_chr13	Cufflinks	exon	32848429	32848706	.	-	.	gene_id "XLOC_164223"; transcript_id "Novel_002250"; exon_number "1"; gene_type "
Dt_chr13	Cufflinks	exon	32849305	32858864	.	-	.	gene_id "XLOC_164223"; transcript_id "Novel_002250"; exon_number "2"; gene_type "
Dt_chr13	Cufflinks	exon	36373413	36374691	.	-	.	gene_id "XLOC_164399"; transcript_id "Novel_002251"; exon_number "1"; gene_type "
Dt_chr13	Cufflinks	exon	36374771	36375065	.	-	.	gene_id "XLOC_164399"; transcript_id "Novel_002251"; exon_number "2"; gene_type "
Dt_chr13	Cufflinks	exon	36918837	36919340	.	-	.	gene_id "XLOC_164423"; transcript_id "Novel_002252"; exon_number "1"; gene_type "
Dt_chr13	Cufflinks	exon	36923165	36925116	.	-	.	gene_id "XLOC_164423"; transcript_id "Novel_002252"; exon_number "2"; gene_type "
Dt_chr13	Cufflinks	exon	41571286	41571406	.	-	.	gene_id "XLOC_164686"; transcript_id "Novel_002253"; exon_number "1"; gene_type "
Dt_chr13	Cufflinks	exon	41571490	41571577	.	-	.	gene_id "XLOC_164686"; transcript_id "Novel_002253"; exon_number "2"; gene_type "
Dt_chr13	Cufflinks	exon	41572424	41572459	.	-	.	gene_id "XLOC_164686"; transcript_id "Novel_002253"; exon_number "3"; gene_type "
Dt_chr13	Cufflinks	exon	47455750	47456092	.	-	.	gene_id "XLOC_165108"; transcript_id "Novel_002254"; exon_number "1"; gene_type "
Dt_chr13	Cufflinks	exon	47456191	47456248	.	-	.	gene_id "XLOC_165108"; transcript_id "Novel_002254"; exon_number "2"; gene_type "
Dt_chr13	Cufflinks	exon	47457215	47457690	.	-	.	gene_id "XLOC_165108"; transcript_id "Novel_002254"; exon_number "3"; gene_type "
Dt_chr13	Cufflinks	exon	47455925	47456248	.	-	.	gene_id "XLOC_165108"; transcript_id "Novel_002255"; exon_number "1"; gene_type "
Dt_chr13	Cufflinks	exon	47457215	47457672	.	-	.	gene_id "XLOC_165108"; transcript_id "Novel_002255"; exon_number "2"; gene_type "
Dt_chr13	Cufflinks	exon	51677028	51677462	.	-	.	gene_id "XLOC_165514"; transcript_id "Novel_002256"; exon_number "1"; gene_type "
Dt_chr13	Cufflinks	exon	51677549	51678038	.	-	.	gene_id "XLOC_165514"; transcript_id "Novel_002256"; exon_number "2"; gene_type "
Dt_chr13	Cufflinks	exon	51678205	51678324	.	-	.	gene_id "XLOC_165514"; transcript_id "Novel_002256"; exon_number "3"; gene_type "
Dt_chr13	Cufflinks	exon	51678415	51678522	.	-	.	gene_id "XLOC_165514"; transcript_id "Novel_002256"; exon_number "4"; gene_type "
Dt_chr13	Cufflinks	exon	51679073	51679394	.	-	.	gene_id "XLOC_165514"; transcript_id "Novel_002256"; exon_number "5"; gene_type "
Dt_chr13	Cufflinks	exon	51680054	51680184	.	-	.	gene_id "XLOC_165514"; transcript_id "Novel_002256"; exon_number "6"; gene_type "
Dt_chr13	Cufflinks	exon	52271888	52274657	.	-	.	gene_id "XLOC_165557"; transcript_id "Novel_002257"; exon_number "1"; gene_type "
Dt_chr13	Cufflinks	exon	52274945	52275419	.	-	.	gene_id "XLOC_165557"; transcript_id "Novel_002257"; exon_number "2"; gene_type "
Dt_chr13	Cufflinks	exon	52780992	52781840	.	-	.	gene_id "XLOC_165612"; transcript_id "Novel_002258"; exon_number "1"; gene_type "
Dt_chr13	Cufflinks	exon	53605733	53606287	.	-	.	gene_id "XLOC_165716"; transcript_id "Novel_002259"; exon_number "1"; gene_type "
Dt_chr13	Cufflinks	exon	53606379	53606490	.	-	.	gene_id "XLOC_165716"; transcript_id "Novel_002259"; exon_number "2"; gene_type "
Dt_chr13	Cufflinks	exon	53606562	53607252	.	-	.	gene_id "XLOC_165716"; transcript_id "Novel_002259"; exon_number "3"; gene_type "
Dt_chr13	Cufflinks	exon	53607330	53607421	.	-	.	gene_id "XLOC_165716"; transcript_id "Novel_002259"; exon_number "4"; gene_type "
Dt_chr13	Cufflinks	exon	53607517	53607570	.	-	.	gene_id "XLOC_165716"; transcript_id "Novel_002259"; exon_number "5"; gene_type "
Dt_chr13	Cufflinks	exon	53608173	53608251	.	-	.	gene_id "XLOC_165716"; transcript_id "Novel_002259"; exon_number "6"; gene_type "
Dt_chr13	Cufflinks	exon	53608438	53608558	.	-	.	gene_id "XLOC_165716"; transcript_id "Novel_002259"; exon_number "7"; gene_type "
Dt_chr13	Cufflinks	exon	53605837	53606287	.	-	.	gene_id "XLOC_165716"; transcript_id "Novel_002260"; exon_number "1"; gene_type "
Dt_chr13	Cufflinks	exon	53606379	53606490	.	-	.	gene_id "XLOC_165716"; transcript_id "Novel_002260"; exon_number "2"; gene_type "
Dt_chr13	Cufflinks	exon	53606562	53606629	.	-	.	gene_id "XLOC_165716"; transcript_id "Novel_002260"; exon_number "3"; gene_type "

Dt_chr13	Cufflinks	exon	53607139	53607252	.	-	.	gene_id "XLOC_165716"; transcript_id "Novel_002260"; exon_number "4"; gene_type "
Dt_chr13	Cufflinks	exon	53607330	53607421	.	-	.	gene_id "XLOC_165716"; transcript_id "Novel_002260"; exon_number "5"; gene_type "
Dt_chr13	Cufflinks	exon	53607517	53607570	.	-	.	gene_id "XLOC_165716"; transcript_id "Novel_002260"; exon_number "6"; gene_type "
Dt_chr13	Cufflinks	exon	53608173	53608251	.	-	.	gene_id "XLOC_165716"; transcript_id "Novel_002260"; exon_number "7"; gene_type "
Dt_chr13	Cufflinks	exon	53608438	53608568	.	-	.	gene_id "XLOC_165716"; transcript_id "Novel_002260"; exon_number "8"; gene_type "
Dt_chr13	Cufflinks	exon	53605852	53606287	.	-	.	gene_id "XLOC_165716"; transcript_id "Novel_002261"; exon_number "1"; gene_type "
Dt_chr13	Cufflinks	exon	53606379	53606931	.	-	.	gene_id "XLOC_165716"; transcript_id "Novel_002261"; exon_number "2"; gene_type "
Dt_chr13	Cufflinks	exon	53606381	53607252	.	-	.	gene_id "XLOC_165716"; transcript_id "Novel_002262"; exon_number "1"; gene_type "
Dt_chr13	Cufflinks	exon	53607330	53607421	.	-	.	gene_id "XLOC_165716"; transcript_id "Novel_002262"; exon_number "2"; gene_type "
Dt_chr13	Cufflinks	exon	53607517	53607570	.	-	.	gene_id "XLOC_165716"; transcript_id "Novel_002262"; exon_number "3"; gene_type "
Dt_chr13	Cufflinks	exon	53608173	53608251	.	-	.	gene_id "XLOC_165716"; transcript_id "Novel_002262"; exon_number "4"; gene_type "
Dt_chr13	Cufflinks	exon	53608438	53608582	.	-	.	gene_id "XLOC_165716"; transcript_id "Novel_002262"; exon_number "5"; gene_type "
Dt_chr13	Cufflinks	exon	54156968	54157044	.	-	.	gene_id "XLOC_165786"; transcript_id "Novel_002263"; exon_number "1"; gene_type "
Dt_chr13	Cufflinks	exon	54157234	54157597	.	-	.	gene_id "XLOC_165786"; transcript_id "Novel_002263"; exon_number "2"; gene_type "
Dt_chr13	Cufflinks	exon	54471148	54471194	.	-	.	gene_id "XLOC_165836"; transcript_id "Novel_002264"; exon_number "1"; gene_type "
Dt_chr13	Cufflinks	exon	54472257	54472319	.	-	.	gene_id "XLOC_165836"; transcript_id "Novel_002264"; exon_number "2"; gene_type "
Dt_chr13	Cufflinks	exon	54473707	54473777	.	-	.	gene_id "XLOC_165836"; transcript_id "Novel_002264"; exon_number "3"; gene_type "
Dt_chr13	Cufflinks	exon	54475613	54475764	.	-	.	gene_id "XLOC_165836"; transcript_id "Novel_002264"; exon_number "4"; gene_type "
Dt_chr13	Cufflinks	exon	54653277	54654063	.	-	.	gene_id "XLOC_165874"; transcript_id "Novel_002265"; exon_number "1"; gene_type "
Dt_chr13	Cufflinks	exon	54654600	54654702	.	-	.	gene_id "XLOC_165874"; transcript_id "Novel_002265"; exon_number "2"; gene_type "
Dt_chr13	Cufflinks	exon	54654802	54655105	.	-	.	gene_id "XLOC_165874"; transcript_id "Novel_002265"; exon_number "3"; gene_type "
Dt_chr13	Cufflinks	exon	54653428	54653676	.	-	.	gene_id "XLOC_165874"; transcript_id "Novel_002266"; exon_number "1"; gene_type "
Dt_chr13	Cufflinks	exon	54653775	54654025	.	-	.	gene_id "XLOC_165874"; transcript_id "Novel_002266"; exon_number "2"; gene_type "
Dt_chr13	Cufflinks	exon	54654517	54654702	.	-	.	gene_id "XLOC_165874"; transcript_id "Novel_002267"; exon_number "1"; gene_type "
Dt_chr13	Cufflinks	exon	54654802	54655073	.	-	.	gene_id "XLOC_165874"; transcript_id "Novel_002267"; exon_number "2"; gene_type "
Dt_chr13	Cufflinks	exon	54660185	54660630	.	-	.	gene_id "XLOC_165875"; transcript_id "Novel_002268"; exon_number "1"; gene_type "
Dt_chr13	Cufflinks	exon	54660915	54661020	.	-	.	gene_id "XLOC_165875"; transcript_id "Novel_002268"; exon_number "2"; gene_type "
Dt_chr13	Cufflinks	exon	54661553	54661648	.	-	.	gene_id "XLOC_165875"; transcript_id "Novel_002268"; exon_number "3"; gene_type "
Dt_chr13	Cufflinks	exon	54661730	54661979	.	-	.	gene_id "XLOC_165875"; transcript_id "Novel_002268"; exon_number "4"; gene_type "
Dt_chr13	Cufflinks	exon	54660616	54660630	.	-	.	gene_id "XLOC_165875"; transcript_id "Novel_002269"; exon_number "1"; gene_type "
Dt_chr13	Cufflinks	exon	54660915	54661020	.	-	.	gene_id "XLOC_165875"; transcript_id "Novel_002269"; exon_number "2"; gene_type "
Dt_chr13	Cufflinks	exon	54661553	54663125	.	-	.	gene_id "XLOC_165875"; transcript_id "Novel_002269"; exon_number "3"; gene_type "
Dt_chr13	Cufflinks	exon	54663216	54664243	.	-	.	gene_id "XLOC_165875"; transcript_id "Novel_002269"; exon_number "4"; gene_type "
Dt_chr13	Cufflinks	exon	54712018	54712897	.	-	.	gene_id "XLOC_165890"; transcript_id "Novel_002270"; exon_number "1"; gene_type "
Dt_chr13	Cufflinks	exon	54713000	54713293	.	-	.	gene_id "XLOC_165890"; transcript_id "Novel_002270"; exon_number "2"; gene_type "

Dt_chr13	Cufflinks	exon	54713392	54713536	.	-	.	gene_id "XLOC_165890"; transcript_id "Novel_002270"; exon_number "3"; gene_type "
Dt_chr13	Cufflinks	exon	54714238	54714452	.	-	.	gene_id "XLOC_165890"; transcript_id "Novel_002270"; exon_number "4"; gene_type "
Dt_chr13	Cufflinks	exon	54800398	54800839	.	-	.	gene_id "XLOC_165907"; transcript_id "Novel_002271"; exon_number "1"; gene_type "
Dt_chr13	Cufflinks	exon	54801033	54801119	.	-	.	gene_id "XLOC_165907"; transcript_id "Novel_002271"; exon_number "2"; gene_type "
Dt_chr13	Cufflinks	exon	54801227	54801423	.	-	.	gene_id "XLOC_165907"; transcript_id "Novel_002271"; exon_number "3"; gene_type "
Dt_chr13	Cufflinks	exon	54801810	54801905	.	-	.	gene_id "XLOC_165907"; transcript_id "Novel_002271"; exon_number "4"; gene_type "
Dt_chr13	Cufflinks	exon	54802052	54802091	.	-	.	gene_id "XLOC_165907"; transcript_id "Novel_002271"; exon_number "5"; gene_type "
Dt_chr13	Cufflinks	exon	54802568	54802684	.	-	.	gene_id "XLOC_165907"; transcript_id "Novel_002271"; exon_number "6"; gene_type "
Dt_chr13	Cufflinks	exon	54803355	54803413	.	-	.	gene_id "XLOC_165907"; transcript_id "Novel_002271"; exon_number "7"; gene_type "
Dt_chr13	Cufflinks	exon	55643472	55643651	.	-	.	gene_id "XLOC_166085"; transcript_id "Novel_002272"; exon_number "1"; gene_type "
Dt_chr13	Cufflinks	exon	55643741	55643820	.	-	.	gene_id "XLOC_166085"; transcript_id "Novel_002272"; exon_number "2"; gene_type "
Dt_chr13	Cufflinks	exon	55644086	55644203	.	-	.	gene_id "XLOC_166086"; transcript_id "Novel_002273"; exon_number "1"; gene_type "
Dt_chr13	Cufflinks	exon	55644286	55644858	.	-	.	gene_id "XLOC_166086"; transcript_id "Novel_002273"; exon_number "2"; gene_type "
Dt_chr13	Cufflinks	exon	55727471	55729098	.	-	.	gene_id "XLOC_166102"; transcript_id "Novel_002274"; exon_number "1"; gene_type "
Dt_chr13	Cufflinks	exon	55729184	55729747	.	-	.	gene_id "XLOC_166102"; transcript_id "Novel_002274"; exon_number "2"; gene_type "
Dt_chr13	Cufflinks	exon	55729826	55729916	.	-	.	gene_id "XLOC_166102"; transcript_id "Novel_002274"; exon_number "3"; gene_type "
Dt_chr13	Cufflinks	exon	55730255	55730489	.	-	.	gene_id "XLOC_166102"; transcript_id "Novel_002274"; exon_number "4"; gene_type "
Dt_chr13	Cufflinks	exon	55727525	55729098	.	-	.	gene_id "XLOC_166102"; transcript_id "Novel_002275"; exon_number "1"; gene_type "
Dt_chr13	Cufflinks	exon	55729184	55729916	.	-	.	gene_id "XLOC_166102"; transcript_id "Novel_002275"; exon_number "2"; gene_type "
Dt_chr13	Cufflinks	exon	55730255	55730482	.	-	.	gene_id "XLOC_166102"; transcript_id "Novel_002275"; exon_number "3"; gene_type "
Dt_chr2	Cufflinks	exon	3034583	3034690	.	+	.	gene_id "XLOC_166345"; transcript_id "Novel_002276"; exon_number "1"; gene_type "
Dt_chr2	Cufflinks	exon	3035119	3035181	.	+	.	gene_id "XLOC_166345"; transcript_id "Novel_002276"; exon_number "2"; gene_type "
Dt_chr2	Cufflinks	exon	3035439	3035478	.	+	.	gene_id "XLOC_166345"; transcript_id "Novel_002276"; exon_number "3"; gene_type "
Dt_chr2	Cufflinks	exon	3035580	3035803	.	+	.	gene_id "XLOC_166345"; transcript_id "Novel_002276"; exon_number "4"; gene_type "
Dt_chr2	Cufflinks	exon	3036140	3036633	.	+	.	gene_id "XLOC_166345"; transcript_id "Novel_002276"; exon_number "5"; gene_type "
Dt_chr2	Cufflinks	exon	3036812	3037116	.	+	.	gene_id "XLOC_166345"; transcript_id "Novel_002276"; exon_number "6"; gene_type "
Dt_chr2	Cufflinks	exon	3037651	3037849	.	+	.	gene_id "XLOC_166345"; transcript_id "Novel_002276"; exon_number "7"; gene_type "
Dt_chr2	Cufflinks	exon	3037940	3038093	.	+	.	gene_id "XLOC_166345"; transcript_id "Novel_002276"; exon_number "8"; gene_type "
Dt_chr2	Cufflinks	exon	3038345	3038603	.	+	.	gene_id "XLOC_166345"; transcript_id "Novel_002276"; exon_number "9"; gene_type "
Dt_chr2	Cufflinks	exon	3038812	3039169	.	+	.	gene_id "XLOC_166345"; transcript_id "Novel_002276"; exon_number "10"; gene_type
Dt_chr2	Cufflinks	exon	5602092	5602629	.	+	.	gene_id "XLOC_166429"; transcript_id "Novel_002277"; exon_number "1"; gene_type "
Dt_chr2	Cufflinks	exon	5602775	5603297	.	+	.	gene_id "XLOC_166429"; transcript_id "Novel_002277"; exon_number "2"; gene_type "
Dt_chr2	Cufflinks	exon	7004802	7005206	.	+	.	gene_id "XLOC_166534"; transcript_id "Novel_002278"; exon_number "1"; gene_type "
Dt_chr2	Cufflinks	exon	7005303	7005392	.	+	.	gene_id "XLOC_166534"; transcript_id "Novel_002278"; exon_number "2"; gene_type "
Dt_chr2	Cufflinks	exon	7007336	7007929	.	+	.	gene_id "XLOC_166534"; transcript_id "Novel_002278"; exon_number "3"; gene_type "

Dt_chr2	Cufflinks	exon	7438802	7439150	.	+	.	gene_id "XLOC_166580"; transcript_id "Novel_002279"; exon_number "1"; gene_type "
Dt_chr2	Cufflinks	exon	7440317	7440502	.	+	.	gene_id "XLOC_166580"; transcript_id "Novel_002279"; exon_number "2"; gene_type "
Dt_chr2	Cufflinks	exon	7440590	7440627	.	+	.	gene_id "XLOC_166580"; transcript_id "Novel_002279"; exon_number "3"; gene_type "
Dt_chr2	Cufflinks	exon	7440711	7441225	.	+	.	gene_id "XLOC_166580"; transcript_id "Novel_002279"; exon_number "4"; gene_type "
Dt_chr2	Cufflinks	exon	7441310	7441457	.	+	.	gene_id "XLOC_166580"; transcript_id "Novel_002279"; exon_number "5"; gene_type "
Dt_chr2	Cufflinks	exon	7438802	7439150	.	+	.	gene_id "XLOC_166580"; transcript_id "Novel_002280"; exon_number "1"; gene_type "
Dt_chr2	Cufflinks	exon	7439304	7439408	.	+	.	gene_id "XLOC_166580"; transcript_id "Novel_002280"; exon_number "2"; gene_type "
Dt_chr2	Cufflinks	exon	7440422	7440627	.	+	.	gene_id "XLOC_166580"; transcript_id "Novel_002280"; exon_number "3"; gene_type "
Dt_chr2	Cufflinks	exon	7440711	7441225	.	+	.	gene_id "XLOC_166580"; transcript_id "Novel_002280"; exon_number "4"; gene_type "
Dt_chr2	Cufflinks	exon	7441310	7441457	.	+	.	gene_id "XLOC_166580"; transcript_id "Novel_002280"; exon_number "5"; gene_type "
Dt_chr2	Cufflinks	exon	7438809	7439150	.	+	.	gene_id "XLOC_166580"; transcript_id "Novel_002281"; exon_number "1"; gene_type "
Dt_chr2	Cufflinks	exon	7439304	7439340	.	+	.	gene_id "XLOC_166580"; transcript_id "Novel_002281"; exon_number "2"; gene_type "
Dt_chr2	Cufflinks	exon	7440354	7440502	.	+	.	gene_id "XLOC_166580"; transcript_id "Novel_002281"; exon_number "3"; gene_type "
Dt_chr2	Cufflinks	exon	7440590	7441225	.	+	.	gene_id "XLOC_166580"; transcript_id "Novel_002281"; exon_number "4"; gene_type "
Dt_chr2	Cufflinks	exon	7441310	7441465	.	+	.	gene_id "XLOC_166580"; transcript_id "Novel_002281"; exon_number "5"; gene_type "
Dt_chr2	Cufflinks	exon	7438809	7439150	.	+	.	gene_id "XLOC_166580"; transcript_id "Novel_002282"; exon_number "1"; gene_type "
Dt_chr2	Cufflinks	exon	7439304	7439408	.	+	.	gene_id "XLOC_166580"; transcript_id "Novel_002282"; exon_number "2"; gene_type "
Dt_chr2	Cufflinks	exon	7440422	7440502	.	+	.	gene_id "XLOC_166580"; transcript_id "Novel_002282"; exon_number "3"; gene_type "
Dt_chr2	Cufflinks	exon	7440590	7440627	.	+	.	gene_id "XLOC_166580"; transcript_id "Novel_002282"; exon_number "4"; gene_type "
Dt_chr2	Cufflinks	exon	7440711	7441225	.	+	.	gene_id "XLOC_166580"; transcript_id "Novel_002282"; exon_number "5"; gene_type "
Dt_chr2	Cufflinks	exon	7441310	7441465	.	+	.	gene_id "XLOC_166580"; transcript_id "Novel_002282"; exon_number "6"; gene_type "
Dt_chr2	Cufflinks	exon	7438889	7439150	.	+	.	gene_id "XLOC_166580"; transcript_id "Novel_002283"; exon_number "1"; gene_type "
Dt_chr2	Cufflinks	exon	7439304	7439340	.	+	.	gene_id "XLOC_166580"; transcript_id "Novel_002283"; exon_number "2"; gene_type "
Dt_chr2	Cufflinks	exon	7440354	7441225	.	+	.	gene_id "XLOC_166580"; transcript_id "Novel_002283"; exon_number "3"; gene_type "
Dt_chr2	Cufflinks	exon	7441310	7441457	.	+	.	gene_id "XLOC_166580"; transcript_id "Novel_002283"; exon_number "4"; gene_type "
Dt_chr2	Cufflinks	exon	7438901	7439150	.	+	.	gene_id "XLOC_166580"; transcript_id "Novel_002284"; exon_number "1"; gene_type "
Dt_chr2	Cufflinks	exon	7439304	7439408	.	+	.	gene_id "XLOC_166580"; transcript_id "Novel_002284"; exon_number "2"; gene_type "
Dt_chr2	Cufflinks	exon	7440422	7441225	.	+	.	gene_id "XLOC_166580"; transcript_id "Novel_002284"; exon_number "3"; gene_type "
Dt_chr2	Cufflinks	exon	7441310	7441457	.	+	.	gene_id "XLOC_166580"; transcript_id "Novel_002284"; exon_number "4"; gene_type "
Dt_chr2	Cufflinks	exon	7545756	7546139	.	+	.	gene_id "XLOC_166589"; transcript_id "Novel_002285"; exon_number "1"; gene_type "
Dt_chr2	Cufflinks	exon	7546504	7546607	.	+	.	gene_id "XLOC_166589"; transcript_id "Novel_002285"; exon_number "2"; gene_type "
Dt_chr2	Cufflinks	exon	7546787	7546900	.	+	.	gene_id "XLOC_166589"; transcript_id "Novel_002285"; exon_number "3"; gene_type "
Dt_chr2	Cufflinks	exon	7548046	7548129	.	+	.	gene_id "XLOC_166589"; transcript_id "Novel_002285"; exon_number "4"; gene_type "
Dt_chr2	Cufflinks	exon	7548324	7548767	.	+	.	gene_id "XLOC_166589"; transcript_id "Novel_002285"; exon_number "5"; gene_type "
Dt_chr2	Cufflinks	exon	10116015	10116572	.	+	.	gene_id "XLOC_166831"; transcript_id "Novel_002286"; exon_number "1"; gene_type "

Dt_chr2	Cufflinks	exon	10116662	10117486	.	+	.	gene_id "XLOC_166831"; transcript_id "Novel_002286"; exon_number "2"; gene_type "
Dt_chr2	Cufflinks	exon	10315802	10316191	.	+	.	gene_id "XLOC_166863"; transcript_id "Novel_002287"; exon_number "1"; gene_type "
Dt_chr2	Cufflinks	exon	10318468	10318663	.	+	.	gene_id "XLOC_166863"; transcript_id "Novel_002287"; exon_number "2"; gene_type "
Dt_chr2	Cufflinks	exon	10318738	10319293	.	+	.	gene_id "XLOC_166863"; transcript_id "Novel_002287"; exon_number "3"; gene_type "
Dt_chr2	Cufflinks	exon	10445723	10446520	.	+	.	gene_id "XLOC_166881"; transcript_id "Novel_002288"; exon_number "1"; gene_type "
Dt_chr2	Cufflinks	exon	10446642	10447789	.	+	.	gene_id "XLOC_166881"; transcript_id "Novel_002288"; exon_number "2"; gene_type "
Dt_chr2	Cufflinks	exon	10456563	10456686	.	+	.	gene_id "XLOC_166886"; transcript_id "Novel_002289"; exon_number "1"; gene_type "
Dt_chr2	Cufflinks	exon	10456787	10456888	.	+	.	gene_id "XLOC_166886"; transcript_id "Novel_002289"; exon_number "2"; gene_type "
Dt_chr2	Cufflinks	exon	10457167	10457237	.	+	.	gene_id "XLOC_166886"; transcript_id "Novel_002289"; exon_number "3"; gene_type "
Dt_chr2	Cufflinks	exon	10457315	10457377	.	+	.	gene_id "XLOC_166886"; transcript_id "Novel_002289"; exon_number "4"; gene_type "
Dt_chr2	Cufflinks	exon	10457471	10458136	.	+	.	gene_id "XLOC_166886"; transcript_id "Novel_002289"; exon_number "5"; gene_type "
Dt_chr2	Cufflinks	exon	10522883	10523258	.	+	.	gene_id "XLOC_166895"; transcript_id "Novel_002290"; exon_number "1"; gene_type "
Dt_chr2	Cufflinks	exon	10523733	10523796	.	+	.	gene_id "XLOC_166895"; transcript_id "Novel_002290"; exon_number "2"; gene_type "
Dt_chr2	Cufflinks	exon	10524049	10524078	.	+	.	gene_id "XLOC_166895"; transcript_id "Novel_002290"; exon_number "3"; gene_type "
Dt_chr2	Cufflinks	exon	10524322	10524429	.	+	.	gene_id "XLOC_166895"; transcript_id "Novel_002290"; exon_number "4"; gene_type "
Dt_chr2	Cufflinks	exon	10524529	10524791	.	+	.	gene_id "XLOC_166895"; transcript_id "Novel_002290"; exon_number "5"; gene_type "
Dt_chr2	Cufflinks	exon	10525520	10525782	.	+	.	gene_id "XLOC_166895"; transcript_id "Novel_002290"; exon_number "6"; gene_type "
Dt_chr2	Cufflinks	exon	10522889	10523258	.	+	.	gene_id "XLOC_166895"; transcript_id "Novel_002291"; exon_number "1"; gene_type "
Dt_chr2	Cufflinks	exon	10524049	10524078	.	+	.	gene_id "XLOC_166895"; transcript_id "Novel_002291"; exon_number "2"; gene_type "
Dt_chr2	Cufflinks	exon	10524322	10524429	.	+	.	gene_id "XLOC_166895"; transcript_id "Novel_002291"; exon_number "3"; gene_type "
Dt_chr2	Cufflinks	exon	10524529	10524791	.	+	.	gene_id "XLOC_166895"; transcript_id "Novel_002291"; exon_number "4"; gene_type "
Dt_chr2	Cufflinks	exon	10525520	10525792	.	+	.	gene_id "XLOC_166895"; transcript_id "Novel_002291"; exon_number "5"; gene_type "
Dt_chr2	Cufflinks	exon	10522889	10523258	.	+	.	gene_id "XLOC_166895"; transcript_id "Novel_002292"; exon_number "1"; gene_type "
Dt_chr2	Cufflinks	exon	10523733	10524078	.	+	.	gene_id "XLOC_166895"; transcript_id "Novel_002292"; exon_number "2"; gene_type "
Dt_chr2	Cufflinks	exon	10524322	10524429	.	+	.	gene_id "XLOC_166895"; transcript_id "Novel_002292"; exon_number "3"; gene_type "
Dt_chr2	Cufflinks	exon	10524529	10524791	.	+	.	gene_id "XLOC_166895"; transcript_id "Novel_002292"; exon_number "4"; gene_type "
Dt_chr2	Cufflinks	exon	10525520	10525792	.	+	.	gene_id "XLOC_166895"; transcript_id "Novel_002292"; exon_number "5"; gene_type "
Dt_chr2	Cufflinks	exon	10684727	10684933	.	+	.	gene_id "XLOC_166902"; transcript_id "Novel_002293"; exon_number "1"; gene_type "
Dt_chr2	Cufflinks	exon	10685020	10686547	.	+	.	gene_id "XLOC_166902"; transcript_id "Novel_002293"; exon_number "2"; gene_type "
Dt_chr2	Cufflinks	exon	10684729	10684933	.	+	.	gene_id "XLOC_166902"; transcript_id "Novel_002294"; exon_number "1"; gene_type "
Dt_chr2	Cufflinks	exon	10685020	10685367	.	+	.	gene_id "XLOC_166902"; transcript_id "Novel_002294"; exon_number "2"; gene_type "
Dt_chr2	Cufflinks	exon	10685442	10685516	.	+	.	gene_id "XLOC_166902"; transcript_id "Novel_002294"; exon_number "3"; gene_type "
Dt_chr2	Cufflinks	exon	10700452	10700556	.	+	.	gene_id "XLOC_166912"; transcript_id "Novel_002295"; exon_number "1"; gene_type "
Dt_chr2	Cufflinks	exon	10700652	10700779	.	+	.	gene_id "XLOC_166912"; transcript_id "Novel_002295"; exon_number "2"; gene_type "
Dt_chr2	Cufflinks	exon	10760776	10760856	.	+	.	gene_id "XLOC_166920"; transcript_id "Novel_002296"; exon_number "1"; gene_type "

Dt_chr2	Cufflinks	exon	10761030	10761289	.	+	.	gene_id "XLOC_166920"; transcript_id "Novel_002296"; exon_number "2"; gene_type "
Dt_chr2	Cufflinks	exon	10794174	10794616	.	+	.	gene_id "XLOC_166925"; transcript_id "Novel_002297"; exon_number "1"; gene_type "
Dt_chr2	Cufflinks	exon	10794692	10794907	.	+	.	gene_id "XLOC_166925"; transcript_id "Novel_002297"; exon_number "2"; gene_type "
Dt_chr2	Cufflinks	exon	10795058	10795372	.	+	.	gene_id "XLOC_166925"; transcript_id "Novel_002297"; exon_number "3"; gene_type "
Dt_chr2	Cufflinks	exon	10795499	10795588	.	+	.	gene_id "XLOC_166925"; transcript_id "Novel_002297"; exon_number "4"; gene_type "
Dt_chr2	Cufflinks	exon	10795675	10795758	.	+	.	gene_id "XLOC_166925"; transcript_id "Novel_002297"; exon_number "5"; gene_type "
Dt_chr2	Cufflinks	exon	10796042	10796077	.	+	.	gene_id "XLOC_166925"; transcript_id "Novel_002297"; exon_number "6"; gene_type "
Dt_chr2	Cufflinks	exon	10796177	10796266	.	+	.	gene_id "XLOC_166925"; transcript_id "Novel_002297"; exon_number "7"; gene_type "
Dt_chr2	Cufflinks	exon	10796356	10796450	.	+	.	gene_id "XLOC_166925"; transcript_id "Novel_002297"; exon_number "8"; gene_type "
Dt_chr2	Cufflinks	exon	10796543	10796556	.	+	.	gene_id "XLOC_166925"; transcript_id "Novel_002297"; exon_number "9"; gene_type "
Dt_chr2	Cufflinks	exon	10856912	10857054	.	+	.	gene_id "XLOC_166929"; transcript_id "Novel_002298"; exon_number "1"; gene_type "
Dt_chr2	Cufflinks	exon	10857212	10857673	.	+	.	gene_id "XLOC_166929"; transcript_id "Novel_002298"; exon_number "2"; gene_type "
Dt_chr2	Cufflinks	exon	11372562	11372924	.	+	.	gene_id "XLOC_166982"; transcript_id "Novel_002299"; exon_number "1"; gene_type "
Dt_chr2	Cufflinks	exon	11373039	11373118	.	+	.	gene_id "XLOC_166982"; transcript_id "Novel_002299"; exon_number "2"; gene_type "
Dt_chr2	Cufflinks	exon	11373479	11373884	.	+	.	gene_id "XLOC_166982"; transcript_id "Novel_002299"; exon_number "3"; gene_type "
Dt_chr2	Cufflinks	exon	11374049	11374472	.	+	.	gene_id "XLOC_166982"; transcript_id "Novel_002299"; exon_number "4"; gene_type "
Dt_chr2	Cufflinks	exon	11462312	11462520	.	+	.	gene_id "XLOC_166989"; transcript_id "Novel_002300"; exon_number "1"; gene_type "
Dt_chr2	Cufflinks	exon	11463077	11463153	.	+	.	gene_id "XLOC_166989"; transcript_id "Novel_002300"; exon_number "2"; gene_type "
Dt_chr2	Cufflinks	exon	11463463	11463562	.	+	.	gene_id "XLOC_166989"; transcript_id "Novel_002300"; exon_number "3"; gene_type "
Dt_chr2	Cufflinks	exon	11463816	11463837	.	+	.	gene_id "XLOC_166989"; transcript_id "Novel_002300"; exon_number "4"; gene_type "
Dt_chr2	Cufflinks	exon	11462363	11462520	.	+	.	gene_id "XLOC_166989"; transcript_id "Novel_002301"; exon_number "1"; gene_type "
Dt_chr2	Cufflinks	exon	11463077	11463153	.	+	.	gene_id "XLOC_166989"; transcript_id "Novel_002301"; exon_number "2"; gene_type "
Dt_chr2	Cufflinks	exon	11463463	11463562	.	+	.	gene_id "XLOC_166989"; transcript_id "Novel_002301"; exon_number "3"; gene_type "
Dt_chr2	Cufflinks	exon	11463699	11463834	.	+	.	gene_id "XLOC_166989"; transcript_id "Novel_002301"; exon_number "4"; gene_type "
Dt_chr2	Cufflinks	exon	11600689	11601471	.	+	.	gene_id "XLOC_167012"; transcript_id "Novel_002302"; exon_number "1"; gene_type "
Dt_chr2	Cufflinks	exon	11601544	11601772	.	+	.	gene_id "XLOC_167012"; transcript_id "Novel_002302"; exon_number "2"; gene_type "
Dt_chr2	Cufflinks	exon	11600697	11601719	.	+	.	gene_id "XLOC_167012"; transcript_id "Novel_002303"; exon_number "1"; gene_type "
Dt_chr2	Cufflinks	exon	11601811	11602901	.	+	.	gene_id "XLOC_167012"; transcript_id "Novel_002303"; exon_number "2"; gene_type "
Dt_chr2	Cufflinks	exon	11600701	11601163	.	+	.	gene_id "XLOC_167012"; transcript_id "Novel_002304"; exon_number "1"; gene_type "
Dt_chr2	Cufflinks	exon	11601234	11602472	.	+	.	gene_id "XLOC_167012"; transcript_id "Novel_002304"; exon_number "2"; gene_type "
Dt_chr2	Cufflinks	exon	11601828	11602169	.	+	.	gene_id "XLOC_167012"; transcript_id "Novel_002305"; exon_number "1"; gene_type "
Dt_chr2	Cufflinks	exon	11602251	11603046	.	+	.	gene_id "XLOC_167012"; transcript_id "Novel_002305"; exon_number "2"; gene_type "
Dt_chr2	Cufflinks	exon	11652657	11652931	.	+	.	gene_id "XLOC_167016"; transcript_id "Novel_002306"; exon_number "1"; gene_type "
Dt_chr2	Cufflinks	exon	11653041	11653097	.	+	.	gene_id "XLOC_167016"; transcript_id "Novel_002306"; exon_number "2"; gene_type "
Dt_chr2	Cufflinks	exon	11653185	11653239	.	+	.	gene_id "XLOC_167016"; transcript_id "Novel_002306"; exon_number "3"; gene_type "

Dt_chr2	Cufflinks	exon	11860063	11860126	.	+	.	gene_id "XLOC_167032"; transcript_id "Novel_002307"; exon_number "1"; gene_type "
Dt_chr2	Cufflinks	exon	11869587	11869992	.	+	.	gene_id "XLOC_167032"; transcript_id "Novel_002307"; exon_number "2"; gene_type "
Dt_chr2	Cufflinks	exon	11913446	11913560	.	+	.	gene_id "XLOC_167050"; transcript_id "Novel_002308"; exon_number "1"; gene_type "
Dt_chr2	Cufflinks	exon	11913645	11914248	.	+	.	gene_id "XLOC_167050"; transcript_id "Novel_002308"; exon_number "2"; gene_type "
Dt_chr2	Cufflinks	exon	11916948	11917163	.	+	.	gene_id "XLOC_167050"; transcript_id "Novel_002308"; exon_number "3"; gene_type "
Dt_chr2	Cufflinks	exon	11917274	11917748	.	+	.	gene_id "XLOC_167050"; transcript_id "Novel_002308"; exon_number "4"; gene_type "
Dt_chr2	Cufflinks	exon	11917934	11918380	.	+	.	gene_id "XLOC_167050"; transcript_id "Novel_002309"; exon_number "1"; gene_type "
Dt_chr2	Cufflinks	exon	11918470	11918706	.	+	.	gene_id "XLOC_167050"; transcript_id "Novel_002309"; exon_number "2"; gene_type "
Dt_chr2	Cufflinks	exon	11918783	11918869	.	+	.	gene_id "XLOC_167050"; transcript_id "Novel_002309"; exon_number "3"; gene_type "
Dt_chr2	Cufflinks	exon	11918952	11919637	.	+	.	gene_id "XLOC_167050"; transcript_id "Novel_002309"; exon_number "4"; gene_type "
Dt_chr2	Cufflinks	exon	12200286	12201155	.	+	.	gene_id "XLOC_167069"; transcript_id "Novel_002310"; exon_number "1"; gene_type "
Dt_chr2	Cufflinks	exon	12201257	12201791	.	+	.	gene_id "XLOC_167069"; transcript_id "Novel_002310"; exon_number "2"; gene_type "
Dt_chr2	Cufflinks	exon	12211413	12212260	.	+	.	gene_id "XLOC_167070"; transcript_id "Novel_002311"; exon_number "1"; gene_type "
Dt_chr2	Cufflinks	exon	12212360	12212915	.	+	.	gene_id "XLOC_167070"; transcript_id "Novel_002311"; exon_number "2"; gene_type "
Dt_chr2	Cufflinks	exon	12328473	12328625	.	+	.	gene_id "XLOC_167077"; transcript_id "Novel_002312"; exon_number "1"; gene_type "
Dt_chr2	Cufflinks	exon	12328726	12329393	.	+	.	gene_id "XLOC_167077"; transcript_id "Novel_002312"; exon_number "2"; gene_type "
Dt_chr2	Cufflinks	exon	12410850	12411302	.	+	.	gene_id "XLOC_167084"; transcript_id "Novel_002313"; exon_number "1"; gene_type "
Dt_chr2	Cufflinks	exon	12411402	12411444	.	+	.	gene_id "XLOC_167084"; transcript_id "Novel_002313"; exon_number "2"; gene_type "
Dt_chr2	Cufflinks	exon	12412136	12412251	.	+	.	gene_id "XLOC_167085"; transcript_id "Novel_002314"; exon_number "1"; gene_type "
Dt_chr2	Cufflinks	exon	12412390	12412492	.	+	.	gene_id "XLOC_167085"; transcript_id "Novel_002314"; exon_number "2"; gene_type "
Dt_chr2	Cufflinks	exon	12412592	12412937	.	+	.	gene_id "XLOC_167085"; transcript_id "Novel_002314"; exon_number "3"; gene_type "
Dt_chr2	Cufflinks	exon	12413024	12413359	.	+	.	gene_id "XLOC_167085"; transcript_id "Novel_002314"; exon_number "4"; gene_type "
Dt_chr2	Cufflinks	exon	12581063	12581520	.	+	.	gene_id "XLOC_167103"; transcript_id "Novel_002315"; exon_number "1"; gene_type "
Dt_chr2	Cufflinks	exon	12582225	12583383	.	+	.	gene_id "XLOC_167103"; transcript_id "Novel_002316"; exon_number "1"; gene_type "
Dt_chr2	Cufflinks	exon	12583568	12583687	.	+	.	gene_id "XLOC_167103"; transcript_id "Novel_002316"; exon_number "2"; gene_type "
Dt_chr2	Cufflinks	exon	12584052	12584106	.	+	.	gene_id "XLOC_167103"; transcript_id "Novel_002316"; exon_number "3"; gene_type "
Dt_chr2	Cufflinks	exon	12584465	12584575	.	+	.	gene_id "XLOC_167103"; transcript_id "Novel_002316"; exon_number "4"; gene_type "
Dt_chr2	Cufflinks	exon	12584923	12584973	.	+	.	gene_id "XLOC_167103"; transcript_id "Novel_002316"; exon_number "5"; gene_type "
Dt_chr2	Cufflinks	exon	12585763	12585938	.	+	.	gene_id "XLOC_167103"; transcript_id "Novel_002316"; exon_number "6"; gene_type "
Dt_chr2	Cufflinks	exon	12586017	12586124	.	+	.	gene_id "XLOC_167103"; transcript_id "Novel_002316"; exon_number "7"; gene_type "
Dt_chr2	Cufflinks	exon	12586585	12586658	.	+	.	gene_id "XLOC_167103"; transcript_id "Novel_002316"; exon_number "8"; gene_type "
Dt_chr2	Cufflinks	exon	12586822	12586997	.	+	.	gene_id "XLOC_167103"; transcript_id "Novel_002316"; exon_number "9"; gene_type "
Dt_chr2	Cufflinks	exon	12587166	12587274	.	+	.	gene_id "XLOC_167103"; transcript_id "Novel_002316"; exon_number "10"; gene_type
Dt_chr2	Cufflinks	exon	12587830	12587910	.	+	.	gene_id "XLOC_167103"; transcript_id "Novel_002316"; exon_number "11"; gene_type
Dt_chr2	Cufflinks	exon	12588004	12588057	.	+	.	gene_id "XLOC_167103"; transcript_id "Novel_002316"; exon_number "12"; gene_type

Dt_chr2	Cufflinks	exon	12588161	12588295	.	+	.	gene_id "XLOC_167103"; transcript_id "Novel_002316"; exon_number "13"; gene_type
Dt_chr2	Cufflinks	exon	12588481	12589090	.	+	.	gene_id "XLOC_167103"; transcript_id "Novel_002316"; exon_number "14"; gene_type
Dt_chr2	Cufflinks	exon	12585250	12585938	.	+	.	gene_id "XLOC_167103"; transcript_id "Novel_002317"; exon_number "1"; gene_type "
Dt_chr2	Cufflinks	exon	12586017	12586124	.	+	.	gene_id "XLOC_167103"; transcript_id "Novel_002317"; exon_number "2"; gene_type "
Dt_chr2	Cufflinks	exon	12586585	12586658	.	+	.	gene_id "XLOC_167103"; transcript_id "Novel_002317"; exon_number "3"; gene_type "
Dt_chr2	Cufflinks	exon	12586822	12587824	.	+	.	gene_id "XLOC_167103"; transcript_id "Novel_002317"; exon_number "4"; gene_type "
Dt_chr2	Cufflinks	exon	12850017	12850396	.	+	.	gene_id "XLOC_167126"; transcript_id "Novel_002318"; exon_number "1"; gene_type "
Dt_chr2	Cufflinks	exon	12851149	12851509	.	+	.	gene_id "XLOC_167126"; transcript_id "Novel_002318"; exon_number "2"; gene_type "
Dt_chr2	Cufflinks	exon	12852076	12852819	.	+	.	gene_id "XLOC_167126"; transcript_id "Novel_002318"; exon_number "3"; gene_type "
Dt_chr2	Cufflinks	exon	12850020	12850396	.	+	.	gene_id "XLOC_167126"; transcript_id "Novel_002319"; exon_number "1"; gene_type "
Dt_chr2	Cufflinks	exon	12851149	12851909	.	+	.	gene_id "XLOC_167126"; transcript_id "Novel_002319"; exon_number "2"; gene_type "
Dt_chr2	Cufflinks	exon	12917300	12917512	.	+	.	gene_id "XLOC_167135"; transcript_id "Novel_002320"; exon_number "1"; gene_type "
Dt_chr2	Cufflinks	exon	12917606	12917638	.	+	.	gene_id "XLOC_167135"; transcript_id "Novel_002320"; exon_number "2"; gene_type "
Dt_chr2	Cufflinks	exon	12986941	12987305	.	+	.	gene_id "XLOC_167151"; transcript_id "Novel_002321"; exon_number "1"; gene_type "
Dt_chr2	Cufflinks	exon	12988256	12989657	.	+	.	gene_id "XLOC_167151"; transcript_id "Novel_002321"; exon_number "2"; gene_type "
Dt_chr2	Cufflinks	exon	13191670	13191962	.	+	.	gene_id "XLOC_167163"; transcript_id "Novel_002322"; exon_number "1"; gene_type "
Dt_chr2	Cufflinks	exon	13192652	13193894	.	+	.	gene_id "XLOC_167163"; transcript_id "Novel_002322"; exon_number "2"; gene_type "
Dt_chr2	Cufflinks	exon	13648941	13649202	.	+	.	gene_id "XLOC_167189"; transcript_id "Novel_002323"; exon_number "1"; gene_type "
Dt_chr2	Cufflinks	exon	13649395	13649519	.	+	.	gene_id "XLOC_167189"; transcript_id "Novel_002323"; exon_number "2"; gene_type "
Dt_chr2	Cufflinks	exon	13651012	13651061	.	+	.	gene_id "XLOC_167189"; transcript_id "Novel_002323"; exon_number "3"; gene_type "
Dt_chr2	Cufflinks	exon	13651890	13651955	.	+	.	gene_id "XLOC_167189"; transcript_id "Novel_002323"; exon_number "4"; gene_type "
Dt_chr2	Cufflinks	exon	13652036	13652066	.	+	.	gene_id "XLOC_167189"; transcript_id "Novel_002323"; exon_number "5"; gene_type "
Dt_chr2	Cufflinks	exon	13648941	13649519	.	+	.	gene_id "XLOC_167189"; transcript_id "Novel_002324"; exon_number "1"; gene_type "
Dt_chr2	Cufflinks	exon	13651012	13651061	.	+	.	gene_id "XLOC_167189"; transcript_id "Novel_002324"; exon_number "2"; gene_type "
Dt_chr2	Cufflinks	exon	13651890	13651955	.	+	.	gene_id "XLOC_167189"; transcript_id "Novel_002324"; exon_number "3"; gene_type "
Dt_chr2	Cufflinks	exon	13652036	13652066	.	+	.	gene_id "XLOC_167189"; transcript_id "Novel_002324"; exon_number "4"; gene_type "
Dt_chr2	Cufflinks	exon	13656744	13657452	.	+	.	gene_id "XLOC_167197"; transcript_id "Novel_002325"; exon_number "1"; gene_type "
Dt_chr2	Cufflinks	exon	13657559	13657898	.	+	.	gene_id "XLOC_167197"; transcript_id "Novel_002325"; exon_number "2"; gene_type "
Dt_chr2	Cufflinks	exon	13686817	13687035	.	+	.	gene_id "XLOC_167206"; transcript_id "Novel_002326"; exon_number "1"; gene_type "
Dt_chr2	Cufflinks	exon	14056758	14057089	.	+	.	gene_id "XLOC_167236"; transcript_id "Novel_002327"; exon_number "1"; gene_type "
Dt_chr2	Cufflinks	exon	14057184	14057244	.	+	.	gene_id "XLOC_167236"; transcript_id "Novel_002327"; exon_number "2"; gene_type "
Dt_chr2	Cufflinks	exon	14479133	14479906	.	+	.	gene_id "XLOC_167276"; transcript_id "Novel_002328"; exon_number "1"; gene_type "
Dt_chr2	Cufflinks	exon	14480150	14480539	.	+	.	gene_id "XLOC_167276"; transcript_id "Novel_002328"; exon_number "2"; gene_type "
Dt_chr2	Cufflinks	exon	14994520	14994741	.	+	.	gene_id "XLOC_167310"; transcript_id "Novel_002329"; exon_number "1"; gene_type "
Dt_chr2	Cufflinks	exon	14996532	14998107	.	+	.	gene_id "XLOC_167310"; transcript_id "Novel_002329"; exon_number "2"; gene_type "

Dt_chr2	Cufflinks	exon	17277062	17277329	.	+	.	gene_id "XLOC_167491"; transcript_id "Novel_002330"; exon_number "1"; gene_type "
Dt_chr2	Cufflinks	exon	17277824	17278024	.	+	.	gene_id "XLOC_167491"; transcript_id "Novel_002330"; exon_number "2"; gene_type "
Dt_chr2	Cufflinks	exon	18353911	18353970	.	+	.	gene_id "XLOC_167573"; transcript_id "Novel_002331"; exon_number "1"; gene_type "
Dt_chr2	Cufflinks	exon	18354155	18354320	.	+	.	gene_id "XLOC_167573"; transcript_id "Novel_002331"; exon_number "2"; gene_type "
Dt_chr2	Cufflinks	exon	18718214	18718247	.	+	.	gene_id "XLOC_167584"; transcript_id "Novel_002332"; exon_number "1"; gene_type "
Dt_chr2	Cufflinks	exon	18718373	18718616	.	+	.	gene_id "XLOC_167584"; transcript_id "Novel_002332"; exon_number "2"; gene_type "
Dt_chr2	Cufflinks	exon	18718734	18719119	.	+	.	gene_id "XLOC_167584"; transcript_id "Novel_002332"; exon_number "3"; gene_type "
Dt_chr2	Cufflinks	exon	19765910	19766336	.	+	.	gene_id "XLOC_167646"; transcript_id "Novel_002333"; exon_number "1"; gene_type "
Dt_chr2	Cufflinks	exon	19767234	19767384	.	+	.	gene_id "XLOC_167646"; transcript_id "Novel_002333"; exon_number "2"; gene_type "
Dt_chr2	Cufflinks	exon	19767465	19767579	.	+	.	gene_id "XLOC_167646"; transcript_id "Novel_002333"; exon_number "3"; gene_type "
Dt_chr2	Cufflinks	exon	19768248	19768971	.	+	.	gene_id "XLOC_167646"; transcript_id "Novel_002333"; exon_number "4"; gene_type "
Dt_chr2	Cufflinks	exon	19769373	19770093	.	+	.	gene_id "XLOC_167646"; transcript_id "Novel_002333"; exon_number "5"; gene_type "
Dt_chr2	Cufflinks	exon	19765910	19765963	.	+	.	gene_id "XLOC_167646"; transcript_id "Novel_002334"; exon_number "1"; gene_type "
Dt_chr2	Cufflinks	exon	19766086	19766336	.	+	.	gene_id "XLOC_167646"; transcript_id "Novel_002334"; exon_number "2"; gene_type "
Dt_chr2	Cufflinks	exon	19767245	19767384	.	+	.	gene_id "XLOC_167646"; transcript_id "Novel_002334"; exon_number "3"; gene_type "
Dt_chr2	Cufflinks	exon	19767465	19767579	.	+	.	gene_id "XLOC_167646"; transcript_id "Novel_002334"; exon_number "4"; gene_type "
Dt_chr2	Cufflinks	exon	19768248	19768971	.	+	.	gene_id "XLOC_167646"; transcript_id "Novel_002334"; exon_number "5"; gene_type "
Dt_chr2	Cufflinks	exon	19769373	19769555	.	+	.	gene_id "XLOC_167646"; transcript_id "Novel_002334"; exon_number "6"; gene_type "
Dt_chr2	Cufflinks	exon	19771097	19771286	.	+	.	gene_id "XLOC_167646"; transcript_id "Novel_002334"; exon_number "7"; gene_type "
Dt_chr2	Cufflinks	exon	19765918	19766336	.	+	.	gene_id "XLOC_167646"; transcript_id "Novel_002335"; exon_number "1"; gene_type "
Dt_chr2	Cufflinks	exon	19767245	19767384	.	+	.	gene_id "XLOC_167646"; transcript_id "Novel_002335"; exon_number "2"; gene_type "
Dt_chr2	Cufflinks	exon	19767465	19767579	.	+	.	gene_id "XLOC_167646"; transcript_id "Novel_002335"; exon_number "3"; gene_type "
Dt_chr2	Cufflinks	exon	19768248	19768971	.	+	.	gene_id "XLOC_167646"; transcript_id "Novel_002335"; exon_number "4"; gene_type "
Dt_chr2	Cufflinks	exon	19769373	19769555	.	+	.	gene_id "XLOC_167646"; transcript_id "Novel_002335"; exon_number "5"; gene_type "
Dt_chr2	Cufflinks	exon	19769780	19770152	.	+	.	gene_id "XLOC_167646"; transcript_id "Novel_002335"; exon_number "6"; gene_type "
Dt_chr2	Cufflinks	exon	19765918	19766336	.	+	.	gene_id "XLOC_167646"; transcript_id "Novel_002336"; exon_number "1"; gene_type "
Dt_chr2	Cufflinks	exon	19767245	19767384	.	+	.	gene_id "XLOC_167646"; transcript_id "Novel_002336"; exon_number "2"; gene_type "
Dt_chr2	Cufflinks	exon	19767465	19767579	.	+	.	gene_id "XLOC_167646"; transcript_id "Novel_002336"; exon_number "3"; gene_type "
Dt_chr2	Cufflinks	exon	19768248	19768971	.	+	.	gene_id "XLOC_167646"; transcript_id "Novel_002336"; exon_number "4"; gene_type "
Dt_chr2	Cufflinks	exon	19769373	19769555	.	+	.	gene_id "XLOC_167646"; transcript_id "Novel_002336"; exon_number "5"; gene_type "
Dt_chr2	Cufflinks	exon	19771097	19771467	.	+	.	gene_id "XLOC_167646"; transcript_id "Novel_002336"; exon_number "6"; gene_type "
Dt_chr2	Cufflinks	exon	19765935	19766336	.	+	.	gene_id "XLOC_167646"; transcript_id "Novel_002337"; exon_number "1"; gene_type "
Dt_chr2	Cufflinks	exon	19767234	19767384	.	+	.	gene_id "XLOC_167646"; transcript_id "Novel_002337"; exon_number "2"; gene_type "
Dt_chr2	Cufflinks	exon	19767465	19767579	.	+	.	gene_id "XLOC_167646"; transcript_id "Novel_002337"; exon_number "3"; gene_type "
Dt_chr2	Cufflinks	exon	19768248	19768971	.	+	.	gene_id "XLOC_167646"; transcript_id "Novel_002337"; exon_number "4"; gene_type "

Dt_chr2	Cufflinks	exon	19769373	19769555	.	+	.	gene_id "XLOC_167646"; transcript_id "Novel_002337"; exon_number "5"; gene_type "
Dt_chr2	Cufflinks	exon	19769780	19770142	.	+	.	gene_id "XLOC_167646"; transcript_id "Novel_002337"; exon_number "6"; gene_type "
Dt_chr2	Cufflinks	exon	19765952	19766336	.	+	.	gene_id "XLOC_167646"; transcript_id "Novel_002338"; exon_number "1"; gene_type "
Dt_chr2	Cufflinks	exon	19767245	19767384	.	+	.	gene_id "XLOC_167646"; transcript_id "Novel_002338"; exon_number "2"; gene_type "
Dt_chr2	Cufflinks	exon	19767465	19767579	.	+	.	gene_id "XLOC_167646"; transcript_id "Novel_002338"; exon_number "3"; gene_type "
Dt_chr2	Cufflinks	exon	19768248	19768971	.	+	.	gene_id "XLOC_167646"; transcript_id "Novel_002338"; exon_number "4"; gene_type "
Dt_chr2	Cufflinks	exon	19769373	19770150	.	+	.	gene_id "XLOC_167646"; transcript_id "Novel_002338"; exon_number "5"; gene_type "
Dt_chr2	Cufflinks	exon	20271917	20272000	.	+	.	gene_id "XLOC_167684"; transcript_id "Novel_002339"; exon_number "1"; gene_type "
Dt_chr2	Cufflinks	exon	20272907	20273022	.	+	.	gene_id "XLOC_167684"; transcript_id "Novel_002339"; exon_number "2"; gene_type "
Dt_chr2	Cufflinks	exon	20273117	20273351	.	+	.	gene_id "XLOC_167684"; transcript_id "Novel_002339"; exon_number "3"; gene_type "
Dt_chr2	Cufflinks	exon	20948405	20948627	.	+	.	gene_id "XLOC_167753"; transcript_id "Novel_002340"; exon_number "1"; gene_type "
Dt_chr2	Cufflinks	exon	21149949	21150586	.	+	.	gene_id "XLOC_167797"; transcript_id "Novel_002341"; exon_number "1"; gene_type "
Dt_chr2	Cufflinks	exon	21151391	21152588	.	+	.	gene_id "XLOC_167797"; transcript_id "Novel_002341"; exon_number "2"; gene_type "
Dt_chr2	Cufflinks	exon	21152725	21153058	.	+	.	gene_id "XLOC_167797"; transcript_id "Novel_002341"; exon_number "3"; gene_type "
Dt_chr2	Cufflinks	exon	21150087	21150586	.	+	.	gene_id "XLOC_167797"; transcript_id "Novel_002342"; exon_number "1"; gene_type "
Dt_chr2	Cufflinks	exon	21151391	21151525	.	+	.	gene_id "XLOC_167797"; transcript_id "Novel_002342"; exon_number "2"; gene_type "
Dt_chr2	Cufflinks	exon	21152249	21152588	.	+	.	gene_id "XLOC_167797"; transcript_id "Novel_002342"; exon_number "3"; gene_type "
Dt_chr2	Cufflinks	exon	21152725	21153801	.	+	.	gene_id "XLOC_167797"; transcript_id "Novel_002342"; exon_number "4"; gene_type "
Dt_chr2	Cufflinks	exon	21150087	21150586	.	+	.	gene_id "XLOC_167797"; transcript_id "Novel_002343"; exon_number "1"; gene_type "
Dt_chr2	Cufflinks	exon	21151391	21152305	.	+	.	gene_id "XLOC_167797"; transcript_id "Novel_002343"; exon_number "2"; gene_type "
Dt_chr2	Cufflinks	exon	21152454	21152588	.	+	.	gene_id "XLOC_167797"; transcript_id "Novel_002343"; exon_number "3"; gene_type "
Dt_chr2	Cufflinks	exon	21152725	21153801	.	+	.	gene_id "XLOC_167797"; transcript_id "Novel_002343"; exon_number "4"; gene_type "
Dt_chr2	Cufflinks	exon	28157291	28157369	.	+	.	gene_id "XLOC_168078"; transcript_id "Novel_002344"; exon_number "1"; gene_type "
Dt_chr2	Cufflinks	exon	28158029	28159825	.	+	.	gene_id "XLOC_168078"; transcript_id "Novel_002344"; exon_number "2"; gene_type "
Dt_chr2	Cufflinks	exon	29718279	29718300	.	+	.	gene_id "XLOC_168168"; transcript_id "Novel_002345"; exon_number "1"; gene_type "
Dt_chr2	Cufflinks	exon	29718393	29720441	.	+	.	gene_id "XLOC_168168"; transcript_id "Novel_002345"; exon_number "2"; gene_type "
Dt_chr2	Cufflinks	exon	29968968	29969434	.	+	.	gene_id "XLOC_168202"; transcript_id "Novel_002346"; exon_number "1"; gene_type "
Dt_chr2	Cufflinks	exon	29970438	29970501	.	+	.	gene_id "XLOC_168202"; transcript_id "Novel_002346"; exon_number "2"; gene_type "
Dt_chr2	Cufflinks	exon	29970743	29970857	.	+	.	gene_id "XLOC_168202"; transcript_id "Novel_002346"; exon_number "3"; gene_type "
Dt_chr2	Cufflinks	exon	29970995	29971094	.	+	.	gene_id "XLOC_168202"; transcript_id "Novel_002346"; exon_number "4"; gene_type "
Dt_chr2	Cufflinks	exon	29971711	29971757	.	+	.	gene_id "XLOC_168202"; transcript_id "Novel_002346"; exon_number "5"; gene_type "
Dt_chr2	Cufflinks	exon	29973172	29973620	.	+	.	gene_id "XLOC_168202"; transcript_id "Novel_002346"; exon_number "6"; gene_type "
Dt_chr2	Cufflinks	exon	29970292	29971757	.	+	.	gene_id "XLOC_168202"; transcript_id "Novel_002347"; exon_number "1"; gene_type "
Dt_chr2	Cufflinks	exon	29973172	29973380	.	+	.	gene_id "XLOC_168202"; transcript_id "Novel_002347"; exon_number "2"; gene_type "
Dt_chr2	Cufflinks	exon	30819228	30819309	.	+	.	gene_id "XLOC_168266"; transcript_id "Novel_002348"; exon_number "1"; gene_type "

Dt_chr2	Cufflinks	exon	30819414	30819890	.	+	.	gene_id "XLOC_168266"; transcript_id "Novel_002348"; exon_number "2"; gene_type "
Dt_chr2	Cufflinks	exon	33181553	33182563	.	+	.	gene_id "XLOC_168406"; transcript_id "Novel_002349"; exon_number "1"; gene_type "
Dt_chr2	Cufflinks	exon	33183193	33187977	.	+	.	gene_id "XLOC_168406"; transcript_id "Novel_002349"; exon_number "2"; gene_type "
Dt_chr2	Cufflinks	exon	37730708	37731877	.	+	.	gene_id "XLOC_168694"; transcript_id "Novel_002350"; exon_number "1"; gene_type "
Dt_chr2	Cufflinks	exon	37732029	37732104	.	+	.	gene_id "XLOC_168694"; transcript_id "Novel_002350"; exon_number "2"; gene_type "
Dt_chr2	Cufflinks	exon	37733402	37733471	.	+	.	gene_id "XLOC_168694"; transcript_id "Novel_002350"; exon_number "3"; gene_type "
Dt_chr2	Cufflinks	exon	37733779	37733880	.	+	.	gene_id "XLOC_168694"; transcript_id "Novel_002350"; exon_number "4"; gene_type "
Dt_chr2	Cufflinks	exon	37736519	37737069	.	+	.	gene_id "XLOC_168694"; transcript_id "Novel_002350"; exon_number "5"; gene_type "
Dt_chr2	Cufflinks	exon	37730709	37731877	.	+	.	gene_id "XLOC_168694"; transcript_id "Novel_002351"; exon_number "1"; gene_type "
Dt_chr2	Cufflinks	exon	37732003	37732104	.	+	.	gene_id "XLOC_168694"; transcript_id "Novel_002351"; exon_number "2"; gene_type "
Dt_chr2	Cufflinks	exon	37733402	37733471	.	+	.	gene_id "XLOC_168694"; transcript_id "Novel_002351"; exon_number "3"; gene_type "
Dt_chr2	Cufflinks	exon	37733779	37733880	.	+	.	gene_id "XLOC_168694"; transcript_id "Novel_002351"; exon_number "4"; gene_type "
Dt_chr2	Cufflinks	exon	37736519	37737061	.	+	.	gene_id "XLOC_168694"; transcript_id "Novel_002351"; exon_number "5"; gene_type "
Dt_chr2	Cufflinks	exon	37730716	37732104	.	+	.	gene_id "XLOC_168694"; transcript_id "Novel_002352"; exon_number "1"; gene_type "
Dt_chr2	Cufflinks	exon	37733402	37733471	.	+	.	gene_id "XLOC_168694"; transcript_id "Novel_002352"; exon_number "2"; gene_type "
Dt_chr2	Cufflinks	exon	37733779	37733880	.	+	.	gene_id "XLOC_168694"; transcript_id "Novel_002352"; exon_number "3"; gene_type "
Dt_chr2	Cufflinks	exon	37736519	37737182	.	+	.	gene_id "XLOC_168694"; transcript_id "Novel_002352"; exon_number "4"; gene_type "
Dt_chr2	Cufflinks	exon	39408563	39408825	.	+	.	gene_id "XLOC_168841"; transcript_id "Novel_002353"; exon_number "1"; gene_type "
Dt_chr2	Cufflinks	exon	39408928	39409167	.	+	.	gene_id "XLOC_168841"; transcript_id "Novel_002353"; exon_number "2"; gene_type "
Dt_chr2	Cufflinks	exon	39419796	39420038	.	+	.	gene_id "XLOC_168850"; transcript_id "Novel_002354"; exon_number "1"; gene_type "
Dt_chr2	Cufflinks	exon	39420657	39420766	.	+	.	gene_id "XLOC_168850"; transcript_id "Novel_002354"; exon_number "2"; gene_type "
Dt_chr2	Cufflinks	exon	39420866	39420987	.	+	.	gene_id "XLOC_168850"; transcript_id "Novel_002354"; exon_number "3"; gene_type "
Dt_chr2	Cufflinks	exon	39421067	39421445	.	+	.	gene_id "XLOC_168850"; transcript_id "Novel_002354"; exon_number "4"; gene_type "
Dt_chr2	Cufflinks	exon	39419796	39420038	.	+	.	gene_id "XLOC_168850"; transcript_id "Novel_002355"; exon_number "1"; gene_type "
Dt_chr2	Cufflinks	exon	39420657	39420766	.	+	.	gene_id "XLOC_168850"; transcript_id "Novel_002355"; exon_number "2"; gene_type "
Dt_chr2	Cufflinks	exon	39420866	39421445	.	+	.	gene_id "XLOC_168850"; transcript_id "Novel_002355"; exon_number "3"; gene_type "
Dt_chr2	Cufflinks	exon	39420495	39420766	.	+	.	gene_id "XLOC_168850"; transcript_id "Novel_002356"; exon_number "1"; gene_type "
Dt_chr2	Cufflinks	exon	39420866	39420987	.	+	.	gene_id "XLOC_168850"; transcript_id "Novel_002356"; exon_number "2"; gene_type "
Dt_chr2	Cufflinks	exon	39421067	39421485	.	+	.	gene_id "XLOC_168850"; transcript_id "Novel_002356"; exon_number "3"; gene_type "
Dt_chr2	Cufflinks	exon	41134246	41134475	.	+	.	gene_id "XLOC_168977"; transcript_id "Novel_002357"; exon_number "1"; gene_type "
Dt_chr2	Cufflinks	exon	41134568	41134665	.	+	.	gene_id "XLOC_168977"; transcript_id "Novel_002357"; exon_number "2"; gene_type "
Dt_chr2	Cufflinks	exon	41134757	41134787	.	+	.	gene_id "XLOC_168977"; transcript_id "Novel_002357"; exon_number "3"; gene_type "
Dt_chr2	Cufflinks	exon	41134872	41134971	.	+	.	gene_id "XLOC_168977"; transcript_id "Novel_002357"; exon_number "4"; gene_type "
Dt_chr2	Cufflinks	exon	41135048	41135118	.	+	.	gene_id "XLOC_168977"; transcript_id "Novel_002357"; exon_number "5"; gene_type "
Dt_chr2	Cufflinks	exon	41135222	41135365	.	+	.	gene_id "XLOC_168977"; transcript_id "Novel_002357"; exon_number "6"; gene_type "

Dt_chr2	Cufflinks	exon	41135451	41136136	.	+	.	gene_id "XLOC_168977"; transcript_id "Novel_002357"; exon_number "7"; gene_type "
Dt_chr2	Cufflinks	exon	43170684	43170810	.	+	.	gene_id "XLOC_169160"; transcript_id "Novel_002358"; exon_number "1"; gene_type "
Dt_chr2	Cufflinks	exon	43171963	43172025	.	+	.	gene_id "XLOC_169160"; transcript_id "Novel_002358"; exon_number "2"; gene_type "
Dt_chr2	Cufflinks	exon	43172096	43172175	.	+	.	gene_id "XLOC_169160"; transcript_id "Novel_002358"; exon_number "3"; gene_type "
Dt_chr2	Cufflinks	exon	43172289	43172330	.	+	.	gene_id "XLOC_169160"; transcript_id "Novel_002358"; exon_number "4"; gene_type "
Dt_chr2	Cufflinks	exon	43172422	43173326	.	+	.	gene_id "XLOC_169160"; transcript_id "Novel_002358"; exon_number "5"; gene_type "
Dt_chr2	Cufflinks	exon	43171602	43172025	.	+	.	gene_id "XLOC_169160"; transcript_id "Novel_002359"; exon_number "1"; gene_type "
Dt_chr2	Cufflinks	exon	43172096	43172175	.	+	.	gene_id "XLOC_169160"; transcript_id "Novel_002359"; exon_number "2"; gene_type "
Dt_chr2	Cufflinks	exon	43172422	43172761	.	+	.	gene_id "XLOC_169160"; transcript_id "Novel_002359"; exon_number "3"; gene_type "
Dt_chr2	Cufflinks	exon	44218136	44220557	.	+	.	gene_id "XLOC_169262"; transcript_id "Novel_002360"; exon_number "1"; gene_type "
Dt_chr2	Cufflinks	exon	44227659	44229533	.	+	.	gene_id "XLOC_169264"; transcript_id "Novel_002361"; exon_number "1"; gene_type "
Dt_chr2	Cufflinks	exon	44258553	44258689	.	+	.	gene_id "XLOC_169272"; transcript_id "Novel_002362"; exon_number "1"; gene_type "
Dt_chr2	Cufflinks	exon	44258800	44258907	.	+	.	gene_id "XLOC_169272"; transcript_id "Novel_002362"; exon_number "2"; gene_type "
Dt_chr2	Cufflinks	exon	44259875	44259956	.	+	.	gene_id "XLOC_169272"; transcript_id "Novel_002362"; exon_number "3"; gene_type "
Dt_chr2	Cufflinks	exon	44260188	44260317	.	+	.	gene_id "XLOC_169272"; transcript_id "Novel_002362"; exon_number "4"; gene_type "
Dt_chr2	Cufflinks	exon	44260412	44260554	.	+	.	gene_id "XLOC_169272"; transcript_id "Novel_002362"; exon_number "5"; gene_type "
Dt_chr2	Cufflinks	exon	44260637	44260723	.	+	.	gene_id "XLOC_169272"; transcript_id "Novel_002362"; exon_number "6"; gene_type "
Dt_chr2	Cufflinks	exon	44261599	44261872	.	+	.	gene_id "XLOC_169272"; transcript_id "Novel_002362"; exon_number "7"; gene_type "
Dt_chr2	Cufflinks	exon	44258554	44258689	.	+	.	gene_id "XLOC_169272"; transcript_id "Novel_002363"; exon_number "1"; gene_type "
Dt_chr2	Cufflinks	exon	44258800	44258907	.	+	.	gene_id "XLOC_169272"; transcript_id "Novel_002363"; exon_number "2"; gene_type "
Dt_chr2	Cufflinks	exon	44259875	44259956	.	+	.	gene_id "XLOC_169272"; transcript_id "Novel_002363"; exon_number "3"; gene_type "
Dt_chr2	Cufflinks	exon	44260188	44260554	.	+	.	gene_id "XLOC_169272"; transcript_id "Novel_002363"; exon_number "4"; gene_type "
Dt_chr2	Cufflinks	exon	44260637	44260723	.	+	.	gene_id "XLOC_169272"; transcript_id "Novel_002363"; exon_number "5"; gene_type "
Dt_chr2	Cufflinks	exon	44261599	44261924	.	+	.	gene_id "XLOC_169272"; transcript_id "Novel_002363"; exon_number "6"; gene_type "
Dt_chr2	Cufflinks	exon	45829936	45830166	.	+	.	gene_id "XLOC_169490"; transcript_id "Novel_002364"; exon_number "1"; gene_type "
Dt_chr2	Cufflinks	exon	45830333	45830448	.	+	.	gene_id "XLOC_169490"; transcript_id "Novel_002364"; exon_number "2"; gene_type "
Dt_chr2	Cufflinks	exon	45830865	45831603	.	+	.	gene_id "XLOC_169490"; transcript_id "Novel_002364"; exon_number "3"; gene_type "
Dt_chr2	Cufflinks	exon	45829937	45830166	.	+	.	gene_id "XLOC_169490"; transcript_id "Novel_002365"; exon_number "1"; gene_type "
Dt_chr2	Cufflinks	exon	45830333	45831149	.	+	.	gene_id "XLOC_169490"; transcript_id "Novel_002365"; exon_number "2"; gene_type "
Dt_chr2	Cufflinks	exon	46047971	46049178	.	+	.	gene_id "XLOC_169507"; transcript_id "Novel_002366"; exon_number "1"; gene_type "
Dt_chr2	Cufflinks	exon	47055651	47056751	.	+	.	gene_id "XLOC_169613"; transcript_id "Novel_002367"; exon_number "1"; gene_type "
Dt_chr2	Cufflinks	exon	50133092	50133246	.	+	.	gene_id "XLOC_169895"; transcript_id "Novel_002368"; exon_number "1"; gene_type "
Dt_chr2	Cufflinks	exon	50133353	50133575	.	+	.	gene_id "XLOC_169895"; transcript_id "Novel_002368"; exon_number "2"; gene_type "
Dt_chr2	Cufflinks	exon	436636	436677	.	-	.	gene_id "XLOC_169920"; transcript_id "Novel_002369"; exon_number "1"; gene_type "
Dt_chr2	Cufflinks	exon	437641	437947	.	-	.	gene_id "XLOC_169920"; transcript_id "Novel_002369"; exon_number "2"; gene_type "

Dt_chr2	Cufflinks	exon	7707930	7708369	.	-	.	gene_id "XLOC_170271"; transcript_id "Novel_002370"; exon_number "1"; gene_type "
Dt_chr2	Cufflinks	exon	7708856	7709135	.	-	.	gene_id "XLOC_170271"; transcript_id "Novel_002370"; exon_number "2"; gene_type "
Dt_chr2	Cufflinks	exon	7710527	7710707	.	-	.	gene_id "XLOC_170271"; transcript_id "Novel_002370"; exon_number "3"; gene_type "
Dt_chr2	Cufflinks	exon	10702294	10702510	.	-	.	gene_id "XLOC_170598"; transcript_id "Novel_002371"; exon_number "1"; gene_type "
Dt_chr2	Cufflinks	exon	10702699	10702833	.	-	.	gene_id "XLOC_170598"; transcript_id "Novel_002371"; exon_number "2"; gene_type "
Dt_chr2	Cufflinks	exon	10703430	10703550	.	-	.	gene_id "XLOC_170598"; transcript_id "Novel_002371"; exon_number "3"; gene_type "
Dt_chr2	Cufflinks	exon	10703891	10704031	.	-	.	gene_id "XLOC_170598"; transcript_id "Novel_002371"; exon_number "4"; gene_type "
Dt_chr2	Cufflinks	exon	10704184	10704357	.	-	.	gene_id "XLOC_170598"; transcript_id "Novel_002371"; exon_number "5"; gene_type "
Dt_chr2	Cufflinks	exon	10704556	10705531	.	-	.	gene_id "XLOC_170598"; transcript_id "Novel_002371"; exon_number "6"; gene_type "
Dt_chr2	Cufflinks	exon	10702312	10702528	.	-	.	gene_id "XLOC_170598"; transcript_id "Novel_002372"; exon_number "1"; gene_type "
Dt_chr2	Cufflinks	exon	10702699	10702833	.	-	.	gene_id "XLOC_170598"; transcript_id "Novel_002372"; exon_number "2"; gene_type "
Dt_chr2	Cufflinks	exon	10703430	10703550	.	-	.	gene_id "XLOC_170598"; transcript_id "Novel_002372"; exon_number "3"; gene_type "
Dt_chr2	Cufflinks	exon	10703891	10704031	.	-	.	gene_id "XLOC_170598"; transcript_id "Novel_002372"; exon_number "4"; gene_type "
Dt_chr2	Cufflinks	exon	10704180	10704357	.	-	.	gene_id "XLOC_170598"; transcript_id "Novel_002372"; exon_number "5"; gene_type "
Dt_chr2	Cufflinks	exon	10704556	10705585	.	-	.	gene_id "XLOC_170598"; transcript_id "Novel_002372"; exon_number "6"; gene_type "
Dt_chr2	Cufflinks	exon	10705700	10706250	.	-	.	gene_id "XLOC_170598"; transcript_id "Novel_002373"; exon_number "1"; gene_type "
Dt_chr2	Cufflinks	exon	10706729	10706770	.	-	.	gene_id "XLOC_170598"; transcript_id "Novel_002373"; exon_number "2"; gene_type "
Dt_chr2	Cufflinks	exon	10706974	10708495	.	-	.	gene_id "XLOC_170598"; transcript_id "Novel_002373"; exon_number "3"; gene_type "
Dt_chr2	Cufflinks	exon	10767058	10767987	.	-	.	gene_id "XLOC_170605"; transcript_id "Novel_002374"; exon_number "1"; gene_type "
Dt_chr2	Cufflinks	exon	10768089	10770929	.	-	.	gene_id "XLOC_170605"; transcript_id "Novel_002374"; exon_number "2"; gene_type "
Dt_chr2	Cufflinks	exon	10900118	10900720	.	-	.	gene_id "XLOC_170631"; transcript_id "Novel_002375"; exon_number "1"; gene_type "
Dt_chr2	Cufflinks	exon	10900804	10900919	.	-	.	gene_id "XLOC_170631"; transcript_id "Novel_002375"; exon_number "2"; gene_type "
Dt_chr2	Cufflinks	exon	10904115	10904383	.	-	.	gene_id "XLOC_170631"; transcript_id "Novel_002375"; exon_number "3"; gene_type "
Dt_chr2	Cufflinks	exon	11049892	11050309	.	-	.	gene_id "XLOC_170676"; transcript_id "Novel_002376"; exon_number "1"; gene_type "
Dt_chr2	Cufflinks	exon	11050440	11050730	.	-	.	gene_id "XLOC_170676"; transcript_id "Novel_002376"; exon_number "2"; gene_type "
Dt_chr2	Cufflinks	exon	11051443	11052245	.	-	.	gene_id "XLOC_170678"; transcript_id "Novel_002377"; exon_number "1"; gene_type "
Dt_chr2	Cufflinks	exon	11052687	11052841	.	-	.	gene_id "XLOC_170678"; transcript_id "Novel_002377"; exon_number "2"; gene_type "
Dt_chr2	Cufflinks	exon	11053157	11053401	.	-	.	gene_id "XLOC_170678"; transcript_id "Novel_002377"; exon_number "3"; gene_type "
Dt_chr2	Cufflinks	exon	11054052	11054346	.	-	.	gene_id "XLOC_170678"; transcript_id "Novel_002377"; exon_number "4"; gene_type "
Dt_chr2	Cufflinks	exon	11054547	11055540	.	-	.	gene_id "XLOC_170678"; transcript_id "Novel_002377"; exon_number "5"; gene_type "
Dt_chr2	Cufflinks	exon	11056363	11056480	.	-	.	gene_id "XLOC_170678"; transcript_id "Novel_002377"; exon_number "6"; gene_type "
Dt_chr2	Cufflinks	exon	11051682	11052245	.	-	.	gene_id "XLOC_170678"; transcript_id "Novel_002378"; exon_number "1"; gene_type "
Dt_chr2	Cufflinks	exon	11052687	11053401	.	-	.	gene_id "XLOC_170678"; transcript_id "Novel_002378"; exon_number "2"; gene_type "
Dt_chr2	Cufflinks	exon	11054052	11054346	.	-	.	gene_id "XLOC_170678"; transcript_id "Novel_002378"; exon_number "3"; gene_type "
Dt_chr2	Cufflinks	exon	11054547	11055979	.	-	.	gene_id "XLOC_170678"; transcript_id "Novel_002378"; exon_number "4"; gene_type "

Dt_chr2	Cufflinks	exon	11051794	11052245	.	-	.	gene_id "XLOC_170678"; transcript_id "Novel_002379"; exon_number "1"; gene_type "
Dt_chr2	Cufflinks	exon	11052687	11052841	.	-	.	gene_id "XLOC_170678"; transcript_id "Novel_002379"; exon_number "2"; gene_type "
Dt_chr2	Cufflinks	exon	11053157	11053401	.	-	.	gene_id "XLOC_170678"; transcript_id "Novel_002379"; exon_number "3"; gene_type "
Dt_chr2	Cufflinks	exon	11054052	11054346	.	-	.	gene_id "XLOC_170678"; transcript_id "Novel_002379"; exon_number "4"; gene_type "
Dt_chr2	Cufflinks	exon	11054547	11056181	.	-	.	gene_id "XLOC_170678"; transcript_id "Novel_002379"; exon_number "5"; gene_type "
Dt_chr2	Cufflinks	exon	11344932	11349692	.	-	.	gene_id "XLOC_170719"; transcript_id "Novel_002380"; exon_number "1"; gene_type "
Dt_chr2	Cufflinks	exon	11350128	11350287	.	-	.	gene_id "XLOC_170719"; transcript_id "Novel_002380"; exon_number "2"; gene_type "
Dt_chr2	Cufflinks	exon	11414468	11414491	.	-	.	gene_id "XLOC_170726"; transcript_id "Novel_002381"; exon_number "1"; gene_type "
Dt_chr2	Cufflinks	exon	11414784	11414864	.	-	.	gene_id "XLOC_170726"; transcript_id "Novel_002381"; exon_number "2"; gene_type "
Dt_chr2	Cufflinks	exon	11415027	11415238	.	-	.	gene_id "XLOC_170726"; transcript_id "Novel_002381"; exon_number "3"; gene_type "
Dt_chr2	Cufflinks	exon	11517819	11518189	.	-	.	gene_id "XLOC_170736"; transcript_id "Novel_002382"; exon_number "1"; gene_type "
Dt_chr2	Cufflinks	exon	11518512	11518595	.	-	.	gene_id "XLOC_170736"; transcript_id "Novel_002382"; exon_number "2"; gene_type "
Dt_chr2	Cufflinks	exon	11518694	11519412	.	-	.	gene_id "XLOC_170736"; transcript_id "Novel_002382"; exon_number "3"; gene_type "
Dt_chr2	Cufflinks	exon	11520565	11520678	.	-	.	gene_id "XLOC_170736"; transcript_id "Novel_002382"; exon_number "4"; gene_type "
Dt_chr2	Cufflinks	exon	11573176	11574057	.	-	.	gene_id "XLOC_170744"; transcript_id "Novel_002383"; exon_number "1"; gene_type "
Dt_chr2	Cufflinks	exon	11575312	11575390	.	-	.	gene_id "XLOC_170744"; transcript_id "Novel_002383"; exon_number "2"; gene_type "
Dt_chr2	Cufflinks	exon	11575538	11575627	.	-	.	gene_id "XLOC_170744"; transcript_id "Novel_002383"; exon_number "3"; gene_type "
Dt_chr2	Cufflinks	exon	11575729	11575970	.	-	.	gene_id "XLOC_170744"; transcript_id "Novel_002383"; exon_number "4"; gene_type "
Dt_chr2	Cufflinks	exon	11577314	11577409	.	-	.	gene_id "XLOC_170744"; transcript_id "Novel_002383"; exon_number "5"; gene_type "
Dt_chr2	Cufflinks	exon	11578751	11578920	.	-	.	gene_id "XLOC_170744"; transcript_id "Novel_002383"; exon_number "6"; gene_type "
Dt_chr2	Cufflinks	exon	11574478	11575390	.	-	.	gene_id "XLOC_170744"; transcript_id "Novel_002384"; exon_number "1"; gene_type "
Dt_chr2	Cufflinks	exon	11575538	11575627	.	-	.	gene_id "XLOC_170744"; transcript_id "Novel_002384"; exon_number "2"; gene_type "
Dt_chr2	Cufflinks	exon	11575729	11575970	.	-	.	gene_id "XLOC_170744"; transcript_id "Novel_002384"; exon_number "3"; gene_type "
Dt_chr2	Cufflinks	exon	11577314	11577409	.	-	.	gene_id "XLOC_170744"; transcript_id "Novel_002384"; exon_number "4"; gene_type "
Dt_chr2	Cufflinks	exon	11578751	11578871	.	-	.	gene_id "XLOC_170744"; transcript_id "Novel_002384"; exon_number "5"; gene_type "
Dt_chr2	Cufflinks	exon	11889795	11890363	.	-	.	gene_id "XLOC_170782"; transcript_id "Novel_002385"; exon_number "1"; gene_type "
Dt_chr2	Cufflinks	exon	11890438	11890524	.	-	.	gene_id "XLOC_170782"; transcript_id "Novel_002385"; exon_number "2"; gene_type "
Dt_chr2	Cufflinks	exon	11890649	11890885	.	-	.	gene_id "XLOC_170782"; transcript_id "Novel_002385"; exon_number "3"; gene_type "
Dt_chr2	Cufflinks	exon	11890985	11891096	.	-	.	gene_id "XLOC_170782"; transcript_id "Novel_002385"; exon_number "4"; gene_type "
Dt_chr2	Cufflinks	exon	11891716	11892032	.	-	.	gene_id "XLOC_170782"; transcript_id "Novel_002385"; exon_number "5"; gene_type "
Dt_chr2	Cufflinks	exon	11892314	11892529	.	-	.	gene_id "XLOC_170782"; transcript_id "Novel_002385"; exon_number "6"; gene_type "
Dt_chr2	Cufflinks	exon	11893810	11894149	.	-	.	gene_id "XLOC_170782"; transcript_id "Novel_002385"; exon_number "7"; gene_type "
Dt_chr2	Cufflinks	exon	11889803	11890524	.	-	.	gene_id "XLOC_170782"; transcript_id "Novel_002386"; exon_number "1"; gene_type "
Dt_chr2	Cufflinks	exon	11890649	11890885	.	-	.	gene_id "XLOC_170782"; transcript_id "Novel_002386"; exon_number "2"; gene_type "
Dt_chr2	Cufflinks	exon	11890985	11891096	.	-	.	gene_id "XLOC_170782"; transcript_id "Novel_002386"; exon_number "3"; gene_type "

Dt_chr2	Cufflinks	exon	11891716	11892032	.	-	.	gene_id "XLOC_170782"; transcript_id "Novel_002386"; exon_number "4"; gene_type "
Dt_chr2	Cufflinks	exon	11892314	11892529	.	-	.	gene_id "XLOC_170782"; transcript_id "Novel_002386"; exon_number "5"; gene_type "
Dt_chr2	Cufflinks	exon	11893810	11893920	.	-	.	gene_id "XLOC_170782"; transcript_id "Novel_002386"; exon_number "6"; gene_type "
Dt_chr2	Cufflinks	exon	11889805	11890524	.	-	.	gene_id "XLOC_170782"; transcript_id "Novel_002387"; exon_number "1"; gene_type "
Dt_chr2	Cufflinks	exon	11890649	11890885	.	-	.	gene_id "XLOC_170782"; transcript_id "Novel_002387"; exon_number "2"; gene_type "
Dt_chr2	Cufflinks	exon	11890985	11891096	.	-	.	gene_id "XLOC_170782"; transcript_id "Novel_002387"; exon_number "3"; gene_type "
Dt_chr2	Cufflinks	exon	11891716	11892032	.	-	.	gene_id "XLOC_170782"; transcript_id "Novel_002387"; exon_number "4"; gene_type "
Dt_chr2	Cufflinks	exon	11892314	11892942	.	-	.	gene_id "XLOC_170782"; transcript_id "Novel_002387"; exon_number "5"; gene_type "
Dt_chr2	Cufflinks	exon	12666380	12667100	.	-	.	gene_id "XLOC_170822"; transcript_id "Novel_002388"; exon_number "1"; gene_type "
Dt_chr2	Cufflinks	exon	12668019	12668228	.	-	.	gene_id "XLOC_170822"; transcript_id "Novel_002388"; exon_number "2"; gene_type "
Dt_chr2	Cufflinks	exon	12666404	12666413	.	-	.	gene_id "XLOC_170822"; transcript_id "Novel_002389"; exon_number "1"; gene_type "
Dt_chr2	Cufflinks	exon	12666989	12668261	.	-	.	gene_id "XLOC_170822"; transcript_id "Novel_002389"; exon_number "2"; gene_type "
Dt_chr2	Cufflinks	exon	12770264	12770393	.	-	.	gene_id "XLOC_170835"; transcript_id "Novel_002390"; exon_number "1"; gene_type "
Dt_chr2	Cufflinks	exon	12770467	12770570	.	-	.	gene_id "XLOC_170835"; transcript_id "Novel_002390"; exon_number "2"; gene_type "
Dt_chr2	Cufflinks	exon	12770732	12770955	.	-	.	gene_id "XLOC_170836"; transcript_id "Novel_002391"; exon_number "1"; gene_type "
Dt_chr2	Cufflinks	exon	12771046	12771111	.	-	.	gene_id "XLOC_170836"; transcript_id "Novel_002391"; exon_number "2"; gene_type "
Dt_chr2	Cufflinks	exon	13152219	13152914	.	-	.	gene_id "XLOC_170868"; transcript_id "Novel_002392"; exon_number "1"; gene_type "
Dt_chr2	Cufflinks	exon	13153000	13153613	.	-	.	gene_id "XLOC_170868"; transcript_id "Novel_002392"; exon_number "2"; gene_type "
Dt_chr2	Cufflinks	exon	13939211	13939936	.	-	.	gene_id "XLOC_170939"; transcript_id "Novel_002393"; exon_number "1"; gene_type "
Dt_chr2	Cufflinks	exon	13940057	13940128	.	-	.	gene_id "XLOC_170939"; transcript_id "Novel_002393"; exon_number "2"; gene_type "
Dt_chr2	Cufflinks	exon	14478463	14479684	.	-	.	gene_id "XLOC_170970"; transcript_id "Novel_002394"; exon_number "1"; gene_type "
Dt_chr2	Cufflinks	exon	14479928	14480540	.	-	.	gene_id "XLOC_170970"; transcript_id "Novel_002394"; exon_number "2"; gene_type "
Dt_chr2	Cufflinks	exon	15097385	15097642	.	-	.	gene_id "XLOC_171002"; transcript_id "Novel_002395"; exon_number "1"; gene_type "
Dt_chr2	Cufflinks	exon	15098744	15102217	.	-	.	gene_id "XLOC_171002"; transcript_id "Novel_002396"; exon_number "1"; gene_type "
Dt_chr2	Cufflinks	exon	15103635	15103682	.	-	.	gene_id "XLOC_171002"; transcript_id "Novel_002396"; exon_number "2"; gene_type "
Dt_chr2	Cufflinks	exon	15104954	15105138	.	-	.	gene_id "XLOC_171002"; transcript_id "Novel_002396"; exon_number "3"; gene_type "
Dt_chr2	Cufflinks	exon	15944022	15944238	.	-	.	gene_id "XLOC_171061"; transcript_id "Novel_002397"; exon_number "1"; gene_type "
Dt_chr2	Cufflinks	exon	17782001	17782702	.	-	.	gene_id "XLOC_171197"; transcript_id "Novel_002398"; exon_number "1"; gene_type "
Dt_chr2	Cufflinks	exon	17783392	17783553	.	-	.	gene_id "XLOC_171197"; transcript_id "Novel_002398"; exon_number "2"; gene_type "
Dt_chr2	Cufflinks	exon	17783643	17783789	.	-	.	gene_id "XLOC_171197"; transcript_id "Novel_002398"; exon_number "3"; gene_type "
Dt_chr2	Cufflinks	exon	17783949	17784136	.	-	.	gene_id "XLOC_171197"; transcript_id "Novel_002398"; exon_number "4"; gene_type "
Dt_chr2	Cufflinks	exon	17782001	17782476	.	-	.	gene_id "XLOC_171197"; transcript_id "Novel_002399"; exon_number "1"; gene_type "
Dt_chr2	Cufflinks	exon	17782593	17782702	.	-	.	gene_id "XLOC_171197"; transcript_id "Novel_002399"; exon_number "2"; gene_type "
Dt_chr2	Cufflinks	exon	17783392	17783553	.	-	.	gene_id "XLOC_171197"; transcript_id "Novel_002399"; exon_number "3"; gene_type "
Dt_chr2	Cufflinks	exon	17783643	17783789	.	-	.	gene_id "XLOC_171197"; transcript_id "Novel_002399"; exon_number "4"; gene_type "

Dt_chr2	Cufflinks	exon	17783949	17784136	.	-	.	gene_id "XLOC_171197"; transcript_id "Novel_002399"; exon_number "5"; gene_type "
Dt_chr2	Cufflinks	exon	17783159	17783553	.	-	.	gene_id "XLOC_171197"; transcript_id "Novel_002400"; exon_number "1"; gene_type "
Dt_chr2	Cufflinks	exon	17783643	17783789	.	-	.	gene_id "XLOC_171197"; transcript_id "Novel_002400"; exon_number "2"; gene_type "
Dt_chr2	Cufflinks	exon	17783949	17784039	.	-	.	gene_id "XLOC_171197"; transcript_id "Novel_002400"; exon_number "3"; gene_type "
Dt_chr2	Cufflinks	exon	17920280	17920672	.	-	.	gene_id "XLOC_171221"; transcript_id "Novel_002401"; exon_number "1"; gene_type "
Dt_chr2	Cufflinks	exon	17920756	17921418	.	-	.	gene_id "XLOC_171221"; transcript_id "Novel_002401"; exon_number "2"; gene_type "
Dt_chr2	Cufflinks	exon	19136997	19137712	.	-	.	gene_id "XLOC_171265"; transcript_id "Novel_002402"; exon_number "1"; gene_type "
Dt_chr2	Cufflinks	exon	19138102	19138321	.	-	.	gene_id "XLOC_171265"; transcript_id "Novel_002402"; exon_number "2"; gene_type "
Dt_chr2	Cufflinks	exon	19137078	19138307	.	-	.	gene_id "XLOC_171265"; transcript_id "Novel_002403"; exon_number "1"; gene_type "
Dt_chr2	Cufflinks	exon	19138495	19138642	.	-	.	gene_id "XLOC_171265"; transcript_id "Novel_002403"; exon_number "2"; gene_type "
Dt_chr2	Cufflinks	exon	19138783	19139747	.	-	.	gene_id "XLOC_171265"; transcript_id "Novel_002403"; exon_number "3"; gene_type "
Dt_chr2	Cufflinks	exon	19137150	19137712	.	-	.	gene_id "XLOC_171265"; transcript_id "Novel_002404"; exon_number "1"; gene_type "
Dt_chr2	Cufflinks	exon	19138102	19138307	.	-	.	gene_id "XLOC_171265"; transcript_id "Novel_002404"; exon_number "2"; gene_type "
Dt_chr2	Cufflinks	exon	19138495	19138642	.	-	.	gene_id "XLOC_171265"; transcript_id "Novel_002404"; exon_number "3"; gene_type "
Dt_chr2	Cufflinks	exon	19138783	19139691	.	-	.	gene_id "XLOC_171265"; transcript_id "Novel_002404"; exon_number "4"; gene_type "
Dt_chr2	Cufflinks	exon	19137721	19138642	.	-	.	gene_id "XLOC_171265"; transcript_id "Novel_002405"; exon_number "1"; gene_type "
Dt_chr2	Cufflinks	exon	19138783	19139376	.	-	.	gene_id "XLOC_171265"; transcript_id "Novel_002405"; exon_number "2"; gene_type "
Dt_chr2	Cufflinks	exon	19162598	19162668	.	-	.	gene_id "XLOC_171269"; transcript_id "Novel_002406"; exon_number "1"; gene_type "
Dt_chr2	Cufflinks	exon	19162942	19163131	.	-	.	gene_id "XLOC_171269"; transcript_id "Novel_002406"; exon_number "2"; gene_type "
Dt_chr2	Cufflinks	exon	19506953	19507956	.	-	.	gene_id "XLOC_171292"; transcript_id "Novel_002407"; exon_number "1"; gene_type "
Dt_chr2	Cufflinks	exon	19508182	19509787	.	-	.	gene_id "XLOC_171292"; transcript_id "Novel_002407"; exon_number "2"; gene_type "
Dt_chr2	Cufflinks	exon	19509865	19510555	.	-	.	gene_id "XLOC_171292"; transcript_id "Novel_002407"; exon_number "3"; gene_type "
Dt_chr2	Cufflinks	exon	19506956	19507956	.	-	.	gene_id "XLOC_171292"; transcript_id "Novel_002408"; exon_number "1"; gene_type "
Dt_chr2	Cufflinks	exon	19508182	19509297	.	-	.	gene_id "XLOC_171292"; transcript_id "Novel_002408"; exon_number "2"; gene_type "
Dt_chr2	Cufflinks	exon	19509468	19509787	.	-	.	gene_id "XLOC_171292"; transcript_id "Novel_002408"; exon_number "3"; gene_type "
Dt_chr2	Cufflinks	exon	19509865	19510527	.	-	.	gene_id "XLOC_171292"; transcript_id "Novel_002408"; exon_number "4"; gene_type "
Dt_chr2	Cufflinks	exon	19741842	19742056	.	-	.	gene_id "XLOC_171310"; transcript_id "Novel_002409"; exon_number "1"; gene_type "
Dt_chr2	Cufflinks	exon	19742207	19742299	.	-	.	gene_id "XLOC_171310"; transcript_id "Novel_002409"; exon_number "2"; gene_type "
Dt_chr2	Cufflinks	exon	19742433	19743162	.	-	.	gene_id "XLOC_171310"; transcript_id "Novel_002409"; exon_number "3"; gene_type "
Dt_chr2	Cufflinks	exon	23330431	23331759	.	-	.	gene_id "XLOC_171564"; transcript_id "Novel_002410"; exon_number "1"; gene_type "
Dt_chr2	Cufflinks	exon	23342351	23342440	.	-	.	gene_id "XLOC_171564"; transcript_id "Novel_002410"; exon_number "2"; gene_type "
Dt_chr2	Cufflinks	exon	29409381	29409587	.	-	.	gene_id "XLOC_171824"; transcript_id "Novel_002411"; exon_number "1"; gene_type "
Dt_chr2	Cufflinks	exon	38779458	38779794	.	-	.	gene_id "XLOC_172357"; transcript_id "Novel_002412"; exon_number "1"; gene_type "
Dt_chr2	Cufflinks	exon	38779928	38780324	.	-	.	gene_id "XLOC_172357"; transcript_id "Novel_002412"; exon_number "2"; gene_type "
Dt_chr2	Cufflinks	exon	38801970	38803088	.	-	.	gene_id "XLOC_172359"; transcript_id "Novel_002413"; exon_number "1"; gene_type "

Dt_chr2	Cufflinks	exon	38805184	38805806	.	-	.	gene_id "XLOC_172359"; transcript_id "Novel_002413"; exon_number "2"; gene_type "
Dt_chr2	Cufflinks	exon	41444611	41446522	.	-	.	gene_id "XLOC_172580"; transcript_id "Novel_002414"; exon_number "1"; gene_type "
Dt_chr2	Cufflinks	exon	41449796	41449932	.	-	.	gene_id "XLOC_172580"; transcript_id "Novel_002414"; exon_number "2"; gene_type "
Dt_chr2	Cufflinks	exon	44191180	44191722	.	-	.	gene_id "XLOC_172876"; transcript_id "Novel_002415"; exon_number "1"; gene_type "
Dt_chr2	Cufflinks	exon	44192213	44192469	.	-	.	gene_id "XLOC_172876"; transcript_id "Novel_002415"; exon_number "2"; gene_type "
Dt_chr2	Cufflinks	exon	44246856	44247050	.	-	.	gene_id "XLOC_172887"; transcript_id "Novel_002416"; exon_number "1"; gene_type "
Dt_chr2	Cufflinks	exon	44247348	44247534	.	-	.	gene_id "XLOC_172887"; transcript_id "Novel_002416"; exon_number "2"; gene_type "
Dt_chr2	Cufflinks	exon	44247618	44247756	.	-	.	gene_id "XLOC_172887"; transcript_id "Novel_002416"; exon_number "3"; gene_type "
Dt_chr2	Cufflinks	exon	44248658	44248730	.	-	.	gene_id "XLOC_172887"; transcript_id "Novel_002416"; exon_number "4"; gene_type "
Dt_chr2	Cufflinks	exon	44248845	44249011	.	-	.	gene_id "XLOC_172887"; transcript_id "Novel_002416"; exon_number "5"; gene_type "
Dt_chr2	Cufflinks	exon	44859627	44859833	.	-	.	gene_id "XLOC_172983"; transcript_id "Novel_002417"; exon_number "1"; gene_type "
Dt_chr2	Cufflinks	exon	44861303	44861650	.	-	.	gene_id "XLOC_172983"; transcript_id "Novel_002417"; exon_number "2"; gene_type "
Dt_chr2	Cufflinks	exon	48952206	48955293	.	-	.	gene_id "XLOC_173360"; transcript_id "Novel_002418"; exon_number "1"; gene_type "
Dt_chr2	Cufflinks	exon	48958423	48958648	.	-	.	gene_id "XLOC_173360"; transcript_id "Novel_002418"; exon_number "2"; gene_type "
Dt_chr3	Cufflinks	exon	1202431	1202596	.	+	.	gene_id "XLOC_173609"; transcript_id "Novel_002419"; exon_number "1"; gene_type "
Dt_chr3	Cufflinks	exon	1202902	1203048	.	+	.	gene_id "XLOC_173609"; transcript_id "Novel_002419"; exon_number "2"; gene_type "
Dt_chr3	Cufflinks	exon	1203929	1204127	.	+	.	gene_id "XLOC_173609"; transcript_id "Novel_002419"; exon_number "3"; gene_type "
Dt_chr3	Cufflinks	exon	1887047	1888137	.	+	.	gene_id "XLOC_173641"; transcript_id "Novel_002420"; exon_number "1"; gene_type "
Dt_chr3	Cufflinks	exon	1888923	1889228	.	+	.	gene_id "XLOC_173641"; transcript_id "Novel_002420"; exon_number "2"; gene_type "
Dt_chr3	Cufflinks	exon	1887053	1888137	.	+	.	gene_id "XLOC_173641"; transcript_id "Novel_002421"; exon_number "1"; gene_type "
Dt_chr3	Cufflinks	exon	1888923	1889149	.	+	.	gene_id "XLOC_173641"; transcript_id "Novel_002421"; exon_number "2"; gene_type "
Dt_chr3	Cufflinks	exon	1889242	1889390	.	+	.	gene_id "XLOC_173641"; transcript_id "Novel_002421"; exon_number "3"; gene_type "
Dt_chr3	Cufflinks	exon	1889527	1890507	.	+	.	gene_id "XLOC_173641"; transcript_id "Novel_002421"; exon_number "4"; gene_type "
Dt_chr3	Cufflinks	exon	1887068	1888137	.	+	.	gene_id "XLOC_173641"; transcript_id "Novel_002422"; exon_number "1"; gene_type "
Dt_chr3	Cufflinks	exon	1888923	1889149	.	+	.	gene_id "XLOC_173641"; transcript_id "Novel_002422"; exon_number "2"; gene_type "
Dt_chr3	Cufflinks	exon	1889242	1889584	.	+	.	gene_id "XLOC_173641"; transcript_id "Novel_002422"; exon_number "3"; gene_type "
Dt_chr3	Cufflinks	exon	1889670	1889863	.	+	.	gene_id "XLOC_173641"; transcript_id "Novel_002423"; exon_number "1"; gene_type "
Dt_chr3	Cufflinks	exon	1891739	1891804	.	+	.	gene_id "XLOC_173641"; transcript_id "Novel_002423"; exon_number "2"; gene_type "
Dt_chr3	Cufflinks	exon	1891962	1892051	.	+	.	gene_id "XLOC_173641"; transcript_id "Novel_002423"; exon_number "3"; gene_type "
Dt_chr3	Cufflinks	exon	1892192	1892420	.	+	.	gene_id "XLOC_173641"; transcript_id "Novel_002423"; exon_number "4"; gene_type "
Dt_chr3	Cufflinks	exon	1892758	1893422	.	+	.	gene_id "XLOC_173641"; transcript_id "Novel_002423"; exon_number "5"; gene_type "
Dt_chr3	Cufflinks	exon	1890781	1891804	.	+	.	gene_id "XLOC_173641"; transcript_id "Novel_002424"; exon_number "1"; gene_type "
Dt_chr3	Cufflinks	exon	1891962	1892051	.	+	.	gene_id "XLOC_173641"; transcript_id "Novel_002424"; exon_number "2"; gene_type "
Dt_chr3	Cufflinks	exon	1892192	1892420	.	+	.	gene_id "XLOC_173641"; transcript_id "Novel_002424"; exon_number "3"; gene_type "
Dt_chr3	Cufflinks	exon	1892758	1893422	.	+	.	gene_id "XLOC_173641"; transcript_id "Novel_002424"; exon_number "4"; gene_type "

Dt_chr3	Cufflinks	exon	12497686	12497809	.	+	.	gene_id "XLOC_174249"; transcript_id "Novel_002425"; exon_number "1"; gene_type "
Dt_chr3	Cufflinks	exon	12498687	12498957	.	+	.	gene_id "XLOC_174249"; transcript_id "Novel_002425"; exon_number "2"; gene_type "
Dt_chr3	Cufflinks	exon	12499392	12499556	.	+	.	gene_id "XLOC_174249"; transcript_id "Novel_002425"; exon_number "3"; gene_type "
Dt_chr3	Cufflinks	exon	12499674	12500272	.	+	.	gene_id "XLOC_174249"; transcript_id "Novel_002425"; exon_number "4"; gene_type "
Dt_chr3	Cufflinks	exon	12499333	12499556	.	+	.	gene_id "XLOC_174249"; transcript_id "Novel_002426"; exon_number "1"; gene_type "
Dt_chr3	Cufflinks	exon	12499674	12500013	.	+	.	gene_id "XLOC_174249"; transcript_id "Novel_002426"; exon_number "2"; gene_type "
Dt_chr3	Cufflinks	exon	15632027	15632555	.	+	.	gene_id "XLOC_174565"; transcript_id "Novel_002427"; exon_number "1"; gene_type "
Dt_chr3	Cufflinks	exon	15633200	15633322	.	+	.	gene_id "XLOC_174565"; transcript_id "Novel_002427"; exon_number "2"; gene_type "
Dt_chr3	Cufflinks	exon	15633427	15633477	.	+	.	gene_id "XLOC_174565"; transcript_id "Novel_002427"; exon_number "3"; gene_type "
Dt_chr3	Cufflinks	exon	15633647	15633718	.	+	.	gene_id "XLOC_174565"; transcript_id "Novel_002427"; exon_number "4"; gene_type "
Dt_chr3	Cufflinks	exon	15633873	15633983	.	+	.	gene_id "XLOC_174565"; transcript_id "Novel_002427"; exon_number "5"; gene_type "
Dt_chr3	Cufflinks	exon	15634072	15634133	.	+	.	gene_id "XLOC_174565"; transcript_id "Novel_002427"; exon_number "6"; gene_type "
Dt_chr3	Cufflinks	exon	15634696	15634756	.	+	.	gene_id "XLOC_174565"; transcript_id "Novel_002427"; exon_number "7"; gene_type "
Dt_chr3	Cufflinks	exon	15634843	15634899	.	+	.	gene_id "XLOC_174565"; transcript_id "Novel_002427"; exon_number "8"; gene_type "
Dt_chr3	Cufflinks	exon	15635007	15635051	.	+	.	gene_id "XLOC_174565"; transcript_id "Novel_002427"; exon_number "9"; gene_type "
Dt_chr3	Cufflinks	exon	15636223	15636594	.	+	.	gene_id "XLOC_174565"; transcript_id "Novel_002427"; exon_number "10"; gene_type
Dt_chr3	Cufflinks	exon	15721586	15721804	.	+	.	gene_id "XLOC_174570"; transcript_id "Novel_002428"; exon_number "1"; gene_type "
Dt_chr3	Cufflinks	exon	15721937	15721963	.	+	.	gene_id "XLOC_174570"; transcript_id "Novel_002428"; exon_number "2"; gene_type "
Dt_chr3	Cufflinks	exon	15722076	15722876	.	+	.	gene_id "XLOC_174570"; transcript_id "Novel_002428"; exon_number "3"; gene_type "
Dt_chr3	Cufflinks	exon	15721599	15721804	.	+	.	gene_id "XLOC_174570"; transcript_id "Novel_002429"; exon_number "1"; gene_type "
Dt_chr3	Cufflinks	exon	15721937	15722512	.	+	.	gene_id "XLOC_174570"; transcript_id "Novel_002429"; exon_number "2"; gene_type "
Dt_chr3	Cufflinks	exon	15722581	15723408	.	+	.	gene_id "XLOC_174570"; transcript_id "Novel_002429"; exon_number "3"; gene_type "
Dt_chr3	Cufflinks	exon	16185569	16189110	.	+	.	gene_id "XLOC_174589"; transcript_id "Novel_002430"; exon_number "1"; gene_type "
Dt_chr3	Cufflinks	exon	16189208	16189597	.	+	.	gene_id "XLOC_174589"; transcript_id "Novel_002430"; exon_number "2"; gene_type "
Dt_chr3	Cufflinks	exon	20372968	20373654	.	+	.	gene_id "XLOC_174907"; transcript_id "Novel_002431"; exon_number "1"; gene_type "
Dt_chr3	Cufflinks	exon	20438900	20439564	.	+	.	gene_id "XLOC_174913"; transcript_id "Novel_002432"; exon_number "1"; gene_type "
Dt_chr3	Cufflinks	exon	20439649	20439767	.	+	.	gene_id "XLOC_174913"; transcript_id "Novel_002432"; exon_number "2"; gene_type "
Dt_chr3	Cufflinks	exon	20440065	20440391	.	+	.	gene_id "XLOC_174913"; transcript_id "Novel_002432"; exon_number "3"; gene_type "
Dt_chr3	Cufflinks	exon	20439056	20439362	.	+	.	gene_id "XLOC_174913"; transcript_id "Novel_002433"; exon_number "1"; gene_type "
Dt_chr3	Cufflinks	exon	20439455	20439564	.	+	.	gene_id "XLOC_174913"; transcript_id "Novel_002433"; exon_number "2"; gene_type "
Dt_chr3	Cufflinks	exon	20439649	20441369	.	+	.	gene_id "XLOC_174913"; transcript_id "Novel_002433"; exon_number "3"; gene_type "
Dt_chr3	Cufflinks	exon	20439057	20439564	.	+	.	gene_id "XLOC_174913"; transcript_id "Novel_002434"; exon_number "1"; gene_type "
Dt_chr3	Cufflinks	exon	20439649	20441046	.	+	.	gene_id "XLOC_174913"; transcript_id "Novel_002434"; exon_number "2"; gene_type "
Dt_chr3	Cufflinks	exon	20442702	20442948	.	+	.	gene_id "XLOC_174914"; transcript_id "Novel_002435"; exon_number "1"; gene_type "
Dt_chr3	Cufflinks	exon	20443051	20443279	.	+	.	gene_id "XLOC_174914"; transcript_id "Novel_002435"; exon_number "2"; gene_type "

Dt_chr3	Cufflinks	exon	20443611	20443920	.	+	.	gene_id "XLOC_174914"; transcript_id "Novel_002435"; exon_number "3"; gene_type "
Dt_chr3	Cufflinks	exon	20442720	20442948	.	+	.	gene_id "XLOC_174914"; transcript_id "Novel_002436"; exon_number "1"; gene_type "
Dt_chr3	Cufflinks	exon	20443051	20443160	.	+	.	gene_id "XLOC_174914"; transcript_id "Novel_002436"; exon_number "2"; gene_type "
Dt_chr3	Cufflinks	exon	20443225	20443279	.	+	.	gene_id "XLOC_174914"; transcript_id "Novel_002436"; exon_number "3"; gene_type "
Dt_chr3	Cufflinks	exon	20443611	20444301	.	+	.	gene_id "XLOC_174914"; transcript_id "Novel_002436"; exon_number "4"; gene_type "
Dt_chr3	Cufflinks	exon	22433552	22435217	.	+	.	gene_id "XLOC_175158"; transcript_id "Novel_002437"; exon_number "1"; gene_type "
Dt_chr3	Cufflinks	exon	22435341	22435596	.	+	.	gene_id "XLOC_175158"; transcript_id "Novel_002437"; exon_number "2"; gene_type "
Dt_chr3	Cufflinks	exon	22436583	22436790	.	+	.	gene_id "XLOC_175160"; transcript_id "Novel_002438"; exon_number "1"; gene_type "
Dt_chr3	Cufflinks	exon	22437828	22437900	.	+	.	gene_id "XLOC_175160"; transcript_id "Novel_002438"; exon_number "2"; gene_type "
Dt_chr3	Cufflinks	exon	22438098	22438303	.	+	.	gene_id "XLOC_175160"; transcript_id "Novel_002438"; exon_number "3"; gene_type "
Dt_chr3	Cufflinks	exon	22438383	22439578	.	+	.	gene_id "XLOC_175160"; transcript_id "Novel_002438"; exon_number "4"; gene_type "
Dt_chr3	Cufflinks	exon	22443815	22445358	.	+	.	gene_id "XLOC_175160"; transcript_id "Novel_002439"; exon_number "1"; gene_type "
Dt_chr3	Cufflinks	exon	22446357	22446436	.	+	.	gene_id "XLOC_175160"; transcript_id "Novel_002439"; exon_number "2"; gene_type "
Dt_chr3	Cufflinks	exon	22446526	22446580	.	+	.	gene_id "XLOC_175160"; transcript_id "Novel_002439"; exon_number "3"; gene_type "
Dt_chr3	Cufflinks	exon	22446822	22446919	.	+	.	gene_id "XLOC_175160"; transcript_id "Novel_002439"; exon_number "4"; gene_type "
Dt_chr3	Cufflinks	exon	22447432	22447500	.	+	.	gene_id "XLOC_175160"; transcript_id "Novel_002439"; exon_number "5"; gene_type "
Dt_chr3	Cufflinks	exon	22447654	22448045	.	+	.	gene_id "XLOC_175160"; transcript_id "Novel_002439"; exon_number "6"; gene_type "
Dt_chr3	Cufflinks	exon	22457640	22457927	.	+	.	gene_id "XLOC_175160"; transcript_id "Novel_002440"; exon_number "1"; gene_type "
Dt_chr3	Cufflinks	exon	22458443	22458667	.	+	.	gene_id "XLOC_175160"; transcript_id "Novel_002440"; exon_number "2"; gene_type "
Dt_chr3	Cufflinks	exon	22459667	22459732	.	+	.	gene_id "XLOC_175160"; transcript_id "Novel_002440"; exon_number "3"; gene_type "
Dt_chr3	Cufflinks	exon	22460952	22461239	.	+	.	gene_id "XLOC_175160"; transcript_id "Novel_002440"; exon_number "4"; gene_type "
Dt_chr3	Cufflinks	exon	22461375	22461524	.	+	.	gene_id "XLOC_175160"; transcript_id "Novel_002440"; exon_number "5"; gene_type "
Dt_chr3	Cufflinks	exon	22457665	22457927	.	+	.	gene_id "XLOC_175160"; transcript_id "Novel_002441"; exon_number "1"; gene_type "
Dt_chr3	Cufflinks	exon	22458443	22458708	.	+	.	gene_id "XLOC_175160"; transcript_id "Novel_002441"; exon_number "2"; gene_type "
Dt_chr3	Cufflinks	exon	22458046	22458667	.	+	.	gene_id "XLOC_175160"; transcript_id "Novel_002442"; exon_number "1"; gene_type "
Dt_chr3	Cufflinks	exon	22459667	22459732	.	+	.	gene_id "XLOC_175160"; transcript_id "Novel_002442"; exon_number "2"; gene_type "
Dt_chr3	Cufflinks	exon	22460952	22461239	.	+	.	gene_id "XLOC_175160"; transcript_id "Novel_002442"; exon_number "3"; gene_type "
Dt_chr3	Cufflinks	exon	22461375	22461447	.	+	.	gene_id "XLOC_175160"; transcript_id "Novel_002442"; exon_number "4"; gene_type "
Dt_chr3	Cufflinks	exon	22461582	22461776	.	+	.	gene_id "XLOC_175160"; transcript_id "Novel_002443"; exon_number "1"; gene_type "
Dt_chr3	Cufflinks	exon	22462123	22462569	.	+	.	gene_id "XLOC_175160"; transcript_id "Novel_002443"; exon_number "2"; gene_type "
Dt_chr3	Cufflinks	exon	22461614	22461776	.	+	.	gene_id "XLOC_175160"; transcript_id "Novel_002444"; exon_number "1"; gene_type "
Dt_chr3	Cufflinks	exon	22462123	22462301	.	+	.	gene_id "XLOC_175160"; transcript_id "Novel_002444"; exon_number "2"; gene_type "
Dt_chr3	Cufflinks	exon	22463513	22463562	.	+	.	gene_id "XLOC_175160"; transcript_id "Novel_002444"; exon_number "3"; gene_type "
Dt_chr3	Cufflinks	exon	22463994	22464818	.	+	.	gene_id "XLOC_175160"; transcript_id "Novel_002444"; exon_number "4"; gene_type "
Dt_chr3	Cufflinks	exon	22466899	22467544	.	+	.	gene_id "XLOC_175160"; transcript_id "Novel_002445"; exon_number "1"; gene_type "

Dt_chr3	Cufflinks	exon	22468070	22468333	.	+	.	gene_id "XLOC_175160"; transcript_id "Novel_002445"; exon_number "2"; gene_type "
Dt_chr3	Cufflinks	exon	22468497	22468690	.	+	.	gene_id "XLOC_175160"; transcript_id "Novel_002445"; exon_number "3"; gene_type "
Dt_chr3	Cufflinks	exon	22468813	22469002	.	+	.	gene_id "XLOC_175160"; transcript_id "Novel_002445"; exon_number "4"; gene_type "
Dt_chr3	Cufflinks	exon	22469444	22469603	.	+	.	gene_id "XLOC_175160"; transcript_id "Novel_002445"; exon_number "5"; gene_type "
Dt_chr3	Cufflinks	exon	22470323	22470439	.	+	.	gene_id "XLOC_175160"; transcript_id "Novel_002445"; exon_number "6"; gene_type "
Dt_chr3	Cufflinks	exon	22470622	22470753	.	+	.	gene_id "XLOC_175160"; transcript_id "Novel_002445"; exon_number "7"; gene_type "
Dt_chr3	Cufflinks	exon	22471096	22472807	.	+	.	gene_id "XLOC_175160"; transcript_id "Novel_002445"; exon_number "8"; gene_type "
Dt_chr3	Cufflinks	exon	22475594	22477760	.	+	.	gene_id "XLOC_175175"; transcript_id "Novel_002446"; exon_number "1"; gene_type "
Dt_chr3	Cufflinks	exon	22478573	22478738	.	+	.	gene_id "XLOC_175175"; transcript_id "Novel_002446"; exon_number "2"; gene_type "
Dt_chr3	Cufflinks	exon	22505323	22505337	.	+	.	gene_id "XLOC_175186"; transcript_id "Novel_002447"; exon_number "1"; gene_type "
Dt_chr3	Cufflinks	exon	22505425	22505712	.	+	.	gene_id "XLOC_175186"; transcript_id "Novel_002447"; exon_number "2"; gene_type "
Dt_chr3	Cufflinks	exon	22886290	22886861	.	+	.	gene_id "XLOC_175233"; transcript_id "Novel_002448"; exon_number "1"; gene_type "
Dt_chr3	Cufflinks	exon	22886967	22887515	.	+	.	gene_id "XLOC_175233"; transcript_id "Novel_002448"; exon_number "2"; gene_type "
Dt_chr3	Cufflinks	exon	23576106	23576485	.	+	.	gene_id "XLOC_175288"; transcript_id "Novel_002449"; exon_number "1"; gene_type "
Dt_chr3	Cufflinks	exon	23577225	23577585	.	+	.	gene_id "XLOC_175288"; transcript_id "Novel_002449"; exon_number "2"; gene_type "
Dt_chr3	Cufflinks	exon	24435099	24435329	.	+	.	gene_id "XLOC_175350"; transcript_id "Novel_002450"; exon_number "1"; gene_type "
Dt_chr3	Cufflinks	exon	24435725	24435798	.	+	.	gene_id "XLOC_175350"; transcript_id "Novel_002450"; exon_number "2"; gene_type "
Dt_chr3	Cufflinks	exon	24435901	24435977	.	+	.	gene_id "XLOC_175350"; transcript_id "Novel_002450"; exon_number "3"; gene_type "
Dt_chr3	Cufflinks	exon	24436073	24436132	.	+	.	gene_id "XLOC_175350"; transcript_id "Novel_002450"; exon_number "4"; gene_type "
Dt_chr3	Cufflinks	exon	24442310	24442340	.	+	.	gene_id "XLOC_175350"; transcript_id "Novel_002450"; exon_number "5"; gene_type "
Dt_chr3	Cufflinks	exon	24442432	24442600	.	+	.	gene_id "XLOC_175350"; transcript_id "Novel_002450"; exon_number "6"; gene_type "
Dt_chr3	Cufflinks	exon	24435245	24435329	.	+	.	gene_id "XLOC_175350"; transcript_id "Novel_002451"; exon_number "1"; gene_type "
Dt_chr3	Cufflinks	exon	24435725	24435798	.	+	.	gene_id "XLOC_175350"; transcript_id "Novel_002451"; exon_number "2"; gene_type "
Dt_chr3	Cufflinks	exon	24435901	24435977	.	+	.	gene_id "XLOC_175350"; transcript_id "Novel_002451"; exon_number "3"; gene_type "
Dt_chr3	Cufflinks	exon	24442250	24442340	.	+	.	gene_id "XLOC_175350"; transcript_id "Novel_002451"; exon_number "4"; gene_type "
Dt_chr3	Cufflinks	exon	24442432	24442742	.	+	.	gene_id "XLOC_175350"; transcript_id "Novel_002451"; exon_number "5"; gene_type "
Dt_chr3	Cufflinks	exon	24435308	24435329	.	+	.	gene_id "XLOC_175350"; transcript_id "Novel_002452"; exon_number "1"; gene_type "
Dt_chr3	Cufflinks	exon	24441905	24441978	.	+	.	gene_id "XLOC_175350"; transcript_id "Novel_002452"; exon_number "2"; gene_type "
Dt_chr3	Cufflinks	exon	24442077	24442153	.	+	.	gene_id "XLOC_175350"; transcript_id "Novel_002452"; exon_number "3"; gene_type "
Dt_chr3	Cufflinks	exon	24442250	24442340	.	+	.	gene_id "XLOC_175350"; transcript_id "Novel_002452"; exon_number "4"; gene_type "
Dt_chr3	Cufflinks	exon	24442432	24442742	.	+	.	gene_id "XLOC_175350"; transcript_id "Novel_002452"; exon_number "5"; gene_type "
Dt_chr3	Cufflinks	exon	24441480	24441515	.	+	.	gene_id "XLOC_175350"; transcript_id "Novel_002453"; exon_number "1"; gene_type "
Dt_chr3	Cufflinks	exon	24441905	24441978	.	+	.	gene_id "XLOC_175350"; transcript_id "Novel_002453"; exon_number "2"; gene_type "
Dt_chr3	Cufflinks	exon	24442077	24442153	.	+	.	gene_id "XLOC_175350"; transcript_id "Novel_002453"; exon_number "3"; gene_type "
Dt_chr3	Cufflinks	exon	24442250	24442340	.	+	.	gene_id "XLOC_175350"; transcript_id "Novel_002453"; exon_number "4"; gene_type "

Dt_chr3	Cufflinks	exon	24442432	24442610	.	+	.	gene_id "XLOC_175350"; transcript_id "Novel_002453"; exon_number "5"; gene_type "
Dt_chr3	Cufflinks	exon	27204789	27204972	.	+	.	gene_id "XLOC_175689"; transcript_id "Novel_002454"; exon_number "1"; gene_type "
Dt_chr3	Cufflinks	exon	27205059	27207370	.	+	.	gene_id "XLOC_175689"; transcript_id "Novel_002454"; exon_number "2"; gene_type "
Dt_chr3	Cufflinks	exon	27204799	27204972	.	+	.	gene_id "XLOC_175689"; transcript_id "Novel_002455"; exon_number "1"; gene_type "
Dt_chr3	Cufflinks	exon	27205059	27205152	.	+	.	gene_id "XLOC_175689"; transcript_id "Novel_002455"; exon_number "2"; gene_type "
Dt_chr3	Cufflinks	exon	27206147	27207377	.	+	.	gene_id "XLOC_175689"; transcript_id "Novel_002455"; exon_number "3"; gene_type "
Dt_chr3	Cufflinks	exon	27204809	27204972	.	+	.	gene_id "XLOC_175689"; transcript_id "Novel_002456"; exon_number "1"; gene_type "
Dt_chr3	Cufflinks	exon	27205059	27205152	.	+	.	gene_id "XLOC_175689"; transcript_id "Novel_002456"; exon_number "2"; gene_type "
Dt_chr3	Cufflinks	exon	27206218	27207366	.	+	.	gene_id "XLOC_175689"; transcript_id "Novel_002456"; exon_number "3"; gene_type "
Dt_chr3	Cufflinks	exon	27408387	27408627	.	+	.	gene_id "XLOC_175709"; transcript_id "Novel_002457"; exon_number "1"; gene_type "
Dt_chr3	Cufflinks	exon	27408890	27411172	.	+	.	gene_id "XLOC_175709"; transcript_id "Novel_002457"; exon_number "2"; gene_type "
Dt_chr3	Cufflinks	exon	27811180	27811273	.	+	.	gene_id "XLOC_175748"; transcript_id "Novel_002458"; exon_number "1"; gene_type "
Dt_chr3	Cufflinks	exon	27811781	27811916	.	+	.	gene_id "XLOC_175748"; transcript_id "Novel_002458"; exon_number "2"; gene_type "
Dt_chr3	Cufflinks	exon	27812521	27812600	.	+	.	gene_id "XLOC_175748"; transcript_id "Novel_002458"; exon_number "3"; gene_type "
Dt_chr3	Cufflinks	exon	27816532	27816984	.	+	.	gene_id "XLOC_175748"; transcript_id "Novel_002458"; exon_number "4"; gene_type "
Dt_chr3	Cufflinks	exon	28405638	28406245	.	+	.	gene_id "XLOC_175795"; transcript_id "Novel_002459"; exon_number "1"; gene_type "
Dt_chr3	Cufflinks	exon	28407145	28407783	.	+	.	gene_id "XLOC_175795"; transcript_id "Novel_002459"; exon_number "2"; gene_type "
Dt_chr3	Cufflinks	exon	2232221	2234275	.	-	.	gene_id "XLOC_176605"; transcript_id "Novel_002460"; exon_number "1"; gene_type "
Dt_chr3	Cufflinks	exon	2242440	2242550	.	-	.	gene_id "XLOC_176605"; transcript_id "Novel_002460"; exon_number "2"; gene_type "
Dt_chr3	Cufflinks	exon	2242666	2243015	.	-	.	gene_id "XLOC_176605"; transcript_id "Novel_002460"; exon_number "3"; gene_type "
Dt_chr3	Cufflinks	exon	2243274	2243556	.	-	.	gene_id "XLOC_176605"; transcript_id "Novel_002460"; exon_number "4"; gene_type "
Dt_chr3	Cufflinks	exon	2243655	2243967	.	-	.	gene_id "XLOC_176605"; transcript_id "Novel_002460"; exon_number "5"; gene_type "
Dt_chr3	Cufflinks	exon	8644841	8647808	.	-	.	gene_id "XLOC_176888"; transcript_id "Novel_002461"; exon_number "1"; gene_type "
Dt_chr3	Cufflinks	exon	8786487	8787278	.	-	.	gene_id "XLOC_176893"; transcript_id "Novel_002462"; exon_number "1"; gene_type "
Dt_chr3	Cufflinks	exon	8787366	8787436	.	-	.	gene_id "XLOC_176893"; transcript_id "Novel_002462"; exon_number "2"; gene_type "
Dt_chr3	Cufflinks	exon	10473252	10474422	.	-	.	gene_id "XLOC_177054"; transcript_id "Novel_002463"; exon_number "1"; gene_type "
Dt_chr3	Cufflinks	exon	10475781	10476350	.	-	.	gene_id "XLOC_177054"; transcript_id "Novel_002463"; exon_number "2"; gene_type "
Dt_chr3	Cufflinks	exon	10473286	10473689	.	-	.	gene_id "XLOC_177054"; transcript_id "Novel_002464"; exon_number "1"; gene_type "
Dt_chr3	Cufflinks	exon	10474297	10474422	.	-	.	gene_id "XLOC_177054"; transcript_id "Novel_002464"; exon_number "2"; gene_type "
Dt_chr3	Cufflinks	exon	10475781	10476248	.	-	.	gene_id "XLOC_177054"; transcript_id "Novel_002464"; exon_number "3"; gene_type "
Dt_chr3	Cufflinks	exon	11040812	11041343	.	-	.	gene_id "XLOC_177119"; transcript_id "Novel_002465"; exon_number "1"; gene_type "
Dt_chr3	Cufflinks	exon	11041651	11041746	.	-	.	gene_id "XLOC_177119"; transcript_id "Novel_002465"; exon_number "2"; gene_type "
Dt_chr3	Cufflinks	exon	11042503	11042577	.	-	.	gene_id "XLOC_177119"; transcript_id "Novel_002465"; exon_number "3"; gene_type "
Dt_chr3	Cufflinks	exon	11043095	11043565	.	-	.	gene_id "XLOC_177119"; transcript_id "Novel_002465"; exon_number "4"; gene_type "
Dt_chr3	Cufflinks	exon	12540835	12541680	.	-	.	gene_id "XLOC_177243"; transcript_id "Novel_002466"; exon_number "1"; gene_type "

Dt_chr3	Cufflinks	exon	12542995	12543533	.	-	.	gene_id "XLOC_177243"; transcript_id "Novel_002466"; exon_number "2"; gene_type "
Dt_chr3	Cufflinks	exon	15546787	15547372	.	-	.	gene_id "XLOC_177491"; transcript_id "Novel_002467"; exon_number "1"; gene_type "
Dt_chr3	Cufflinks	exon	15548528	15548572	.	-	.	gene_id "XLOC_177491"; transcript_id "Novel_002467"; exon_number "2"; gene_type "
Dt_chr3	Cufflinks	exon	15548680	15548736	.	-	.	gene_id "XLOC_177491"; transcript_id "Novel_002467"; exon_number "3"; gene_type "
Dt_chr3	Cufflinks	exon	15548823	15548883	.	-	.	gene_id "XLOC_177491"; transcript_id "Novel_002467"; exon_number "4"; gene_type "
Dt_chr3	Cufflinks	exon	15549452	15549513	.	-	.	gene_id "XLOC_177491"; transcript_id "Novel_002467"; exon_number "5"; gene_type "
Dt_chr3	Cufflinks	exon	15549605	15549715	.	-	.	gene_id "XLOC_177491"; transcript_id "Novel_002467"; exon_number "6"; gene_type "
Dt_chr3	Cufflinks	exon	15549865	15549936	.	-	.	gene_id "XLOC_177491"; transcript_id "Novel_002467"; exon_number "7"; gene_type "
Dt_chr3	Cufflinks	exon	15550107	15550157	.	-	.	gene_id "XLOC_177491"; transcript_id "Novel_002467"; exon_number "8"; gene_type "
Dt_chr3	Cufflinks	exon	15550262	15550384	.	-	.	gene_id "XLOC_177491"; transcript_id "Novel_002467"; exon_number "9"; gene_type "
Dt_chr3	Cufflinks	exon	15550959	15551225	.	-	.	gene_id "XLOC_177491"; transcript_id "Novel_002467"; exon_number "10"; gene_type
Dt_chr3	Cufflinks	exon	15867797	15867866	.	-	.	gene_id "XLOC_177512"; transcript_id "Novel_002468"; exon_number "1"; gene_type "
Dt_chr3	Cufflinks	exon	15868761	15869385	.	-	.	gene_id "XLOC_177512"; transcript_id "Novel_002468"; exon_number "2"; gene_type "
Dt_chr3	Cufflinks	exon	17184433	17184647	.	-	.	gene_id "XLOC_177615"; transcript_id "Novel_002469"; exon_number "1"; gene_type "
Dt_chr3	Cufflinks	exon	20425079	20425603	.	-	.	gene_id "XLOC_177894"; transcript_id "Novel_002470"; exon_number "1"; gene_type "
Dt_chr3	Cufflinks	exon	20426369	20426660	.	-	.	gene_id "XLOC_177894"; transcript_id "Novel_002470"; exon_number "2"; gene_type "
Dt_chr3	Cufflinks	exon	20426748	20426939	.	-	.	gene_id "XLOC_177894"; transcript_id "Novel_002470"; exon_number "3"; gene_type "
Dt_chr3	Cufflinks	exon	20425079	20425603	.	-	.	gene_id "XLOC_177894"; transcript_id "Novel_002471"; exon_number "1"; gene_type "
Dt_chr3	Cufflinks	exon	20426369	20426660	.	-	.	gene_id "XLOC_177894"; transcript_id "Novel_002471"; exon_number "2"; gene_type "
Dt_chr3	Cufflinks	exon	20426748	20426870	.	-	.	gene_id "XLOC_177894"; transcript_id "Novel_002471"; exon_number "3"; gene_type "
Dt_chr3	Cufflinks	exon	20426965	20427782	.	-	.	gene_id "XLOC_177894"; transcript_id "Novel_002471"; exon_number "4"; gene_type "
Dt_chr3	Cufflinks	exon	20665344	20666574	.	-	.	gene_id "XLOC_177926"; transcript_id "Novel_002472"; exon_number "1"; gene_type "
Dt_chr3	Cufflinks	exon	20666756	20666941	.	-	.	gene_id "XLOC_177926"; transcript_id "Novel_002472"; exon_number "2"; gene_type "
Dt_chr3	Cufflinks	exon	20668658	20668835	.	-	.	gene_id "XLOC_177926"; transcript_id "Novel_002472"; exon_number "3"; gene_type "
Dt_chr3	Cufflinks	exon	22176025	22176194	.	-	.	gene_id "XLOC_178089"; transcript_id "Novel_002473"; exon_number "1"; gene_type "
Dt_chr3	Cufflinks	exon	22176292	22176363	.	-	.	gene_id "XLOC_178089"; transcript_id "Novel_002473"; exon_number "2"; gene_type "
Dt_chr3	Cufflinks	exon	22176707	22176774	.	-	.	gene_id "XLOC_178089"; transcript_id "Novel_002473"; exon_number "3"; gene_type "
Dt_chr3	Cufflinks	exon	22177209	22177285	.	-	.	gene_id "XLOC_178089"; transcript_id "Novel_002473"; exon_number "4"; gene_type "
Dt_chr3	Cufflinks	exon	22178677	22180964	.	-	.	gene_id "XLOC_178089"; transcript_id "Novel_002473"; exon_number "5"; gene_type "
Dt_chr3	Cufflinks	exon	22176039	22176194	.	-	.	gene_id "XLOC_178089"; transcript_id "Novel_002474"; exon_number "1"; gene_type "
Dt_chr3	Cufflinks	exon	22176292	22176363	.	-	.	gene_id "XLOC_178089"; transcript_id "Novel_002474"; exon_number "2"; gene_type "
Dt_chr3	Cufflinks	exon	22176707	22176774	.	-	.	gene_id "XLOC_178089"; transcript_id "Novel_002474"; exon_number "3"; gene_type "
Dt_chr3	Cufflinks	exon	22177209	22177285	.	-	.	gene_id "XLOC_178089"; transcript_id "Novel_002474"; exon_number "4"; gene_type "
Dt_chr3	Cufflinks	exon	22178677	22179373	.	-	.	gene_id "XLOC_178089"; transcript_id "Novel_002474"; exon_number "5"; gene_type "
Dt_chr3	Cufflinks	exon	22179796	22180016	.	-	.	gene_id "XLOC_178089"; transcript_id "Novel_002474"; exon_number "6"; gene_type "

Dt_chr3	Cufflinks	exon	22180710	22180944	.	-	.	gene_id "XLOC_178089"; transcript_id "Novel_002474"; exon_number "7"; gene_type "
Dt_chr3	Cufflinks	exon	22404246	22404965	.	-	.	gene_id "XLOC_178125"; transcript_id "Novel_002475"; exon_number "1"; gene_type "
Dt_chr3	Cufflinks	exon	22405057	22405454	.	-	.	gene_id "XLOC_178125"; transcript_id "Novel_002475"; exon_number "2"; gene_type "
Dt_chr3	Cufflinks	exon	22407804	22408362	.	-	.	gene_id "XLOC_178125"; transcript_id "Novel_002475"; exon_number "3"; gene_type "
Dt_chr3	Cufflinks	exon	22422080	22422407	.	-	.	gene_id "XLOC_178128"; transcript_id "Novel_002476"; exon_number "1"; gene_type "
Dt_chr3	Cufflinks	exon	22422533	22422640	.	-	.	gene_id "XLOC_178128"; transcript_id "Novel_002476"; exon_number "2"; gene_type "
Dt_chr3	Cufflinks	exon	22422736	22422878	.	-	.	gene_id "XLOC_178128"; transcript_id "Novel_002476"; exon_number "3"; gene_type "
Dt_chr3	Cufflinks	exon	22423777	22423824	.	-	.	gene_id "XLOC_178128"; transcript_id "Novel_002476"; exon_number "4"; gene_type "
Dt_chr3	Cufflinks	exon	22423910	22424039	.	-	.	gene_id "XLOC_178128"; transcript_id "Novel_002476"; exon_number "5"; gene_type "
Dt_chr3	Cufflinks	exon	22425485	22425832	.	-	.	gene_id "XLOC_178128"; transcript_id "Novel_002476"; exon_number "6"; gene_type "
Dt_chr3	Cufflinks	exon	22425972	22427090	.	-	.	gene_id "XLOC_178128"; transcript_id "Novel_002476"; exon_number "7"; gene_type "
Dt_chr3	Cufflinks	exon	22427229	22427413	.	-	.	gene_id "XLOC_178128"; transcript_id "Novel_002476"; exon_number "8"; gene_type "
Dt_chr3	Cufflinks	exon	22427516	22427819	.	-	.	gene_id "XLOC_178128"; transcript_id "Novel_002476"; exon_number "9"; gene_type "
Dt_chr3	Cufflinks	exon	22422208	22422407	.	-	.	gene_id "XLOC_178128"; transcript_id "Novel_002477"; exon_number "1"; gene_type "
Dt_chr3	Cufflinks	exon	22422533	22422640	.	-	.	gene_id "XLOC_178128"; transcript_id "Novel_002477"; exon_number "2"; gene_type "
Dt_chr3	Cufflinks	exon	22422736	22423251	.	-	.	gene_id "XLOC_178128"; transcript_id "Novel_002477"; exon_number "3"; gene_type "
Dt_chr3	Cufflinks	exon	22771267	22773047	.	-	.	gene_id "XLOC_178160"; transcript_id "Novel_002478"; exon_number "1"; gene_type "
Dt_chr3	Cufflinks	exon	22773324	22773583	.	-	.	gene_id "XLOC_178160"; transcript_id "Novel_002478"; exon_number "2"; gene_type "
Dt_chr3	Cufflinks	exon	23800795	23801047	.	-	.	gene_id "XLOC_178305"; transcript_id "Novel_002479"; exon_number "1"; gene_type "
Dt_chr3	Cufflinks	exon	24205659	24205766	.	-	.	gene_id "XLOC_178360"; transcript_id "Novel_002480"; exon_number "1"; gene_type "
Dt_chr3	Cufflinks	exon	24205892	24206002	.	-	.	gene_id "XLOC_178360"; transcript_id "Novel_002480"; exon_number "2"; gene_type "
Dt_chr3	Cufflinks	exon	24206369	24206602	.	-	.	gene_id "XLOC_178360"; transcript_id "Novel_002480"; exon_number "3"; gene_type "
Dt_chr3	Cufflinks	exon	25710014	25710236	.	-	.	gene_id "XLOC_178516"; transcript_id "Novel_002481"; exon_number "1"; gene_type "
Dt_chr3	Cufflinks	exon	25710701	25710740	.	-	.	gene_id "XLOC_178516"; transcript_id "Novel_002481"; exon_number "2"; gene_type "
Dt_chr3	Cufflinks	exon	26258910	26259547	.	-	.	gene_id "XLOC_178575"; transcript_id "Novel_002482"; exon_number "1"; gene_type "
Dt_chr3	Cufflinks	exon	26259837	26260016	.	-	.	gene_id "XLOC_178575"; transcript_id "Novel_002482"; exon_number "2"; gene_type "
Dt_chr3	Cufflinks	exon	26260172	26260251	.	-	.	gene_id "XLOC_178575"; transcript_id "Novel_002482"; exon_number "3"; gene_type "
Dt_chr3	Cufflinks	exon	26260645	26260770	.	-	.	gene_id "XLOC_178575"; transcript_id "Novel_002482"; exon_number "4"; gene_type "
Dt_chr3	Cufflinks	exon	26260882	26261043	.	-	.	gene_id "XLOC_178575"; transcript_id "Novel_002482"; exon_number "5"; gene_type "
Dt_chr3	Cufflinks	exon	26261218	26261335	.	-	.	gene_id "XLOC_178575"; transcript_id "Novel_002482"; exon_number "6"; gene_type "
Dt_chr3	Cufflinks	exon	26261457	26261523	.	-	.	gene_id "XLOC_178575"; transcript_id "Novel_002482"; exon_number "7"; gene_type "
Dt_chr3	Cufflinks	exon	26258967	26259547	.	-	.	gene_id "XLOC_178575"; transcript_id "Novel_002483"; exon_number "1"; gene_type "
Dt_chr3	Cufflinks	exon	26259837	26260016	.	-	.	gene_id "XLOC_178575"; transcript_id "Novel_002483"; exon_number "2"; gene_type "
Dt_chr3	Cufflinks	exon	26260645	26260770	.	-	.	gene_id "XLOC_178575"; transcript_id "Novel_002483"; exon_number "3"; gene_type "
Dt_chr3	Cufflinks	exon	26260882	26261043	.	-	.	gene_id "XLOC_178575"; transcript_id "Novel_002483"; exon_number "4"; gene_type "

Dt_chr3	Cufflinks	exon	26261218	26261335	.	-	.	gene_id "XLOC_178575"; transcript_id "Novel_002483"; exon_number "5"; gene_type "
Dt_chr3	Cufflinks	exon	26261457	26261682	.	-	.	gene_id "XLOC_178575"; transcript_id "Novel_002483"; exon_number "6"; gene_type "
Dt_chr3	Cufflinks	exon	26262468	26262581	.	-	.	gene_id "XLOC_178575"; transcript_id "Novel_002484"; exon_number "1"; gene_type "
Dt_chr3	Cufflinks	exon	26262657	26262759	.	-	.	gene_id "XLOC_178575"; transcript_id "Novel_002484"; exon_number "2"; gene_type "
Dt_chr3	Cufflinks	exon	26262973	26263098	.	-	.	gene_id "XLOC_178575"; transcript_id "Novel_002484"; exon_number "3"; gene_type "
Dt_chr3	Cufflinks	exon	26263794	26263868	.	-	.	gene_id "XLOC_178575"; transcript_id "Novel_002484"; exon_number "4"; gene_type "
Dt_chr3	Cufflinks	exon	26264257	26264395	.	-	.	gene_id "XLOC_178575"; transcript_id "Novel_002484"; exon_number "5"; gene_type "
Dt_chr3	Cufflinks	exon	26262468	26262759	.	-	.	gene_id "XLOC_178575"; transcript_id "Novel_002485"; exon_number "1"; gene_type "
Dt_chr3	Cufflinks	exon	26262973	26263098	.	-	.	gene_id "XLOC_178575"; transcript_id "Novel_002485"; exon_number "2"; gene_type "
Dt_chr3	Cufflinks	exon	26263794	26263868	.	-	.	gene_id "XLOC_178575"; transcript_id "Novel_002485"; exon_number "3"; gene_type "
Dt_chr3	Cufflinks	exon	26264359	26264440	.	-	.	gene_id "XLOC_178575"; transcript_id "Novel_002485"; exon_number "4"; gene_type "
Dt_chr3	Cufflinks	exon	26262469	26262581	.	-	.	gene_id "XLOC_178575"; transcript_id "Novel_002486"; exon_number "1"; gene_type "
Dt_chr3	Cufflinks	exon	26262657	26262759	.	-	.	gene_id "XLOC_178575"; transcript_id "Novel_002486"; exon_number "2"; gene_type "
Dt_chr3	Cufflinks	exon	26262973	26263098	.	-	.	gene_id "XLOC_178575"; transcript_id "Novel_002486"; exon_number "3"; gene_type "
Dt_chr3	Cufflinks	exon	26263794	26263868	.	-	.	gene_id "XLOC_178575"; transcript_id "Novel_002486"; exon_number "4"; gene_type "
Dt_chr3	Cufflinks	exon	26264359	26264396	.	-	.	gene_id "XLOC_178575"; transcript_id "Novel_002486"; exon_number "5"; gene_type "
Dt_chr3	Cufflinks	exon	26380899	26381786	.	-	.	gene_id "XLOC_178584"; transcript_id "Novel_002487"; exon_number "1"; gene_type "
Dt_chr3	Cufflinks	exon	26381871	26382616	.	-	.	gene_id "XLOC_178584"; transcript_id "Novel_002487"; exon_number "2"; gene_type "
Dt_chr3	Cufflinks	exon	26382779	26382863	.	-	.	gene_id "XLOC_178584"; transcript_id "Novel_002487"; exon_number "3"; gene_type "
Dt_chr3	Cufflinks	exon	26381529	26382616	.	-	.	gene_id "XLOC_178584"; transcript_id "Novel_002488"; exon_number "1"; gene_type "
Dt_chr3	Cufflinks	exon	26382779	26383029	.	-	.	gene_id "XLOC_178584"; transcript_id "Novel_002488"; exon_number "2"; gene_type "
Dt_chr3	Cufflinks	exon	26672306	26674816	.	-	.	gene_id "XLOC_178593"; transcript_id "Novel_002489"; exon_number "1"; gene_type "
Dt_chr3	Cufflinks	exon	26674913	26675218	.	-	.	gene_id "XLOC_178593"; transcript_id "Novel_002489"; exon_number "2"; gene_type "
Dt_chr3	Cufflinks	exon	26672306	26674128	.	-	.	gene_id "XLOC_178593"; transcript_id "Novel_002490"; exon_number "1"; gene_type "
Dt_chr3	Cufflinks	exon	26674215	26675223	.	-	.	gene_id "XLOC_178593"; transcript_id "Novel_002490"; exon_number "2"; gene_type "
Dt_chr3	Cufflinks	exon	26674437	26674552	.	-	.	gene_id "XLOC_178593"; transcript_id "Novel_002491"; exon_number "1"; gene_type "
Dt_chr3	Cufflinks	exon	26674631	26675303	.	-	.	gene_id "XLOC_178593"; transcript_id "Novel_002491"; exon_number "2"; gene_type "
Dt_chr3	Cufflinks	exon	27459810	27459994	.	-	.	gene_id "XLOC_178643"; transcript_id "Novel_002492"; exon_number "1"; gene_type "
Dt_chr3	Cufflinks	exon	27461188	27461281	.	-	.	gene_id "XLOC_178643"; transcript_id "Novel_002492"; exon_number "2"; gene_type "
Dt_chr3	Cufflinks	exon	27461760	27461817	.	-	.	gene_id "XLOC_178643"; transcript_id "Novel_002492"; exon_number "3"; gene_type "
Dt_chr3	Cufflinks	exon	27461975	27462083	.	-	.	gene_id "XLOC_178643"; transcript_id "Novel_002492"; exon_number "4"; gene_type "
Dt_chr3	Cufflinks	exon	27462210	27462695	.	-	.	gene_id "XLOC_178643"; transcript_id "Novel_002492"; exon_number "5"; gene_type "
Dt_chr3	Cufflinks	exon	27459836	27459994	.	-	.	gene_id "XLOC_178643"; transcript_id "Novel_002493"; exon_number "1"; gene_type "
Dt_chr3	Cufflinks	exon	27461188	27461281	.	-	.	gene_id "XLOC_178643"; transcript_id "Novel_002493"; exon_number "2"; gene_type "
Dt_chr3	Cufflinks	exon	27461760	27461817	.	-	.	gene_id "XLOC_178643"; transcript_id "Novel_002493"; exon_number "3"; gene_type "

Dt_chr3	Cufflinks	exon	27461975	27463020	.	-	.	gene_id "XLOC_178643"; transcript_id "Novel_002493"; exon_number "4"; gene_type "
Dt_chr3	Cufflinks	exon	27524809	27525233	.	-	.	gene_id "XLOC_178646"; transcript_id "Novel_002494"; exon_number "1"; gene_type "
Dt_chr3	Cufflinks	exon	27526038	27526123	.	-	.	gene_id "XLOC_178646"; transcript_id "Novel_002494"; exon_number "2"; gene_type "
Dt_chr3	Cufflinks	exon	27527166	27527289	.	-	.	gene_id "XLOC_178646"; transcript_id "Novel_002494"; exon_number "3"; gene_type "
Dt_chr3	Cufflinks	exon	27527376	27527637	.	-	.	gene_id "XLOC_178646"; transcript_id "Novel_002494"; exon_number "4"; gene_type "
Dt_chr3	Cufflinks	exon	27524848	27525233	.	-	.	gene_id "XLOC_178646"; transcript_id "Novel_002495"; exon_number "1"; gene_type "
Dt_chr3	Cufflinks	exon	27526038	27526123	.	-	.	gene_id "XLOC_178646"; transcript_id "Novel_002495"; exon_number "2"; gene_type "
Dt_chr3	Cufflinks	exon	27527166	27527725	.	-	.	gene_id "XLOC_178646"; transcript_id "Novel_002495"; exon_number "3"; gene_type "
Dt_chr3	Cufflinks	exon	27524858	27525233	.	-	.	gene_id "XLOC_178646"; transcript_id "Novel_002496"; exon_number "1"; gene_type "
Dt_chr3	Cufflinks	exon	27526038	27527626	.	-	.	gene_id "XLOC_178646"; transcript_id "Novel_002496"; exon_number "2"; gene_type "
Dt_chr3	Cufflinks	exon	27524885	27525233	.	-	.	gene_id "XLOC_178646"; transcript_id "Novel_002497"; exon_number "1"; gene_type "
Dt_chr3	Cufflinks	exon	27526038	27526194	.	-	.	gene_id "XLOC_178646"; transcript_id "Novel_002497"; exon_number "2"; gene_type "
Dt_chr3	Cufflinks	exon	27527166	27527596	.	-	.	gene_id "XLOC_178646"; transcript_id "Novel_002497"; exon_number "3"; gene_type "
Dt_chr3	Cufflinks	exon	27652446	27653891	.	-	.	gene_id "XLOC_178682"; transcript_id "Novel_002498"; exon_number "1"; gene_type "
Dt_chr3	Cufflinks	exon	27654567	27654620	.	-	.	gene_id "XLOC_178682"; transcript_id "Novel_002498"; exon_number "2"; gene_type "
Dt_chr3	Cufflinks	exon	27654905	27655061	.	-	.	gene_id "XLOC_178682"; transcript_id "Novel_002498"; exon_number "3"; gene_type "
Dt_chr3	Cufflinks	exon	35692364	35692580	.	-	.	gene_id "XLOC_179086"; transcript_id "Novel_002499"; exon_number "1"; gene_type "
Dt_chr3	Cufflinks	exon	35834976	35836467	.	-	.	gene_id "XLOC_179094"; transcript_id "Novel_002500"; exon_number "1"; gene_type "
Dt_chr3	Cufflinks	exon	35836700	35836884	.	-	.	gene_id "XLOC_179094"; transcript_id "Novel_002500"; exon_number "2"; gene_type "
Dt_chr3	Cufflinks	exon	38108984	38109355	.	-	.	gene_id "XLOC_179189"; transcript_id "Novel_002501"; exon_number "1"; gene_type "
Dt_chr3	Cufflinks	exon	38109696	38109803	.	-	.	gene_id "XLOC_179189"; transcript_id "Novel_002501"; exon_number "2"; gene_type "
Dt_chr3	Cufflinks	exon	38110105	38110200	.	-	.	gene_id "XLOC_179189"; transcript_id "Novel_002501"; exon_number "3"; gene_type "
Dt_chr3	Cufflinks	exon	38110279	38110441	.	-	.	gene_id "XLOC_179189"; transcript_id "Novel_002501"; exon_number "4"; gene_type "
Dt_chr3	Cufflinks	exon	38110526	38110552	.	-	.	gene_id "XLOC_179189"; transcript_id "Novel_002501"; exon_number "5"; gene_type "
Dt_chr3	Cufflinks	exon	38110694	38110747	.	-	.	gene_id "XLOC_179189"; transcript_id "Novel_002501"; exon_number "6"; gene_type "
Dt_chr3	Cufflinks	exon	38111086	38111220	.	-	.	gene_id "XLOC_179189"; transcript_id "Novel_002501"; exon_number "7"; gene_type "
Dt_chr3	Cufflinks	exon	38111309	38111577	.	-	.	gene_id "XLOC_179189"; transcript_id "Novel_002501"; exon_number "8"; gene_type "
Dt_chr4	Cufflinks	exon	6198803	6199012	.	+	.	gene_id "XLOC_179721"; transcript_id "Novel_002502"; exon_number "1"; gene_type "
Dt_chr4	Cufflinks	exon	6210731	6211010	.	+	.	gene_id "XLOC_179722"; transcript_id "Novel_002503"; exon_number "1"; gene_type "
Dt_chr4	Cufflinks	exon	6285248	6285448	.	+	.	gene_id "XLOC_179739"; transcript_id "Novel_002504"; exon_number "1"; gene_type "
Dt_chr4	Cufflinks	exon	6286115	6286438	.	+	.	gene_id "XLOC_179739"; transcript_id "Novel_002504"; exon_number "2"; gene_type "
Dt_chr4	Cufflinks	exon	7733194	7736645	.	+	.	gene_id "XLOC_179824"; transcript_id "Novel_002505"; exon_number "1"; gene_type "
Dt_chr4	Cufflinks	exon	7737342	7737546	.	+	.	gene_id "XLOC_179824"; transcript_id "Novel_002505"; exon_number "2"; gene_type "
Dt_chr4	Cufflinks	exon	7767308	7767360	.	+	.	gene_id "XLOC_179835"; transcript_id "Novel_002506"; exon_number "1"; gene_type "
Dt_chr4	Cufflinks	exon	7767461	7767562	.	+	.	gene_id "XLOC_179835"; transcript_id "Novel_002506"; exon_number "2"; gene_type "

Dt_chr4	Cufflinks	exon	7767677	7767720	.	+	.	gene_id "XLOC_179835"; transcript_id "Novel_002506"; exon_number "3"; gene_type "
Dt_chr4	Cufflinks	exon	7770406	7770483	.	+	.	gene_id "XLOC_179835"; transcript_id "Novel_002506"; exon_number "4"; gene_type "
Dt_chr4	Cufflinks	exon	7770580	7770633	.	+	.	gene_id "XLOC_179835"; transcript_id "Novel_002506"; exon_number "5"; gene_type "
Dt_chr4	Cufflinks	exon	7770768	7770838	.	+	.	gene_id "XLOC_179835"; transcript_id "Novel_002506"; exon_number "6"; gene_type "
Dt_chr4	Cufflinks	exon	7771273	7771554	.	+	.	gene_id "XLOC_179835"; transcript_id "Novel_002506"; exon_number "7"; gene_type "
Dt_chr4	Cufflinks	exon	7767308	7767360	.	+	.	gene_id "XLOC_179835"; transcript_id "Novel_002507"; exon_number "1"; gene_type "
Dt_chr4	Cufflinks	exon	7767461	7767562	.	+	.	gene_id "XLOC_179835"; transcript_id "Novel_002507"; exon_number "2"; gene_type "
Dt_chr4	Cufflinks	exon	7767677	7767720	.	+	.	gene_id "XLOC_179835"; transcript_id "Novel_002507"; exon_number "3"; gene_type "
Dt_chr4	Cufflinks	exon	7770406	7770483	.	+	.	gene_id "XLOC_179835"; transcript_id "Novel_002507"; exon_number "4"; gene_type "
Dt_chr4	Cufflinks	exon	7770580	7770633	.	+	.	gene_id "XLOC_179835"; transcript_id "Novel_002507"; exon_number "5"; gene_type "
Dt_chr4	Cufflinks	exon	7770768	7771554	.	+	.	gene_id "XLOC_179835"; transcript_id "Novel_002507"; exon_number "6"; gene_type "
Dt_chr4	Cufflinks	exon	7767349	7767562	.	+	.	gene_id "XLOC_179835"; transcript_id "Novel_002508"; exon_number "1"; gene_type "
Dt_chr4	Cufflinks	exon	7767677	7767720	.	+	.	gene_id "XLOC_179835"; transcript_id "Novel_002508"; exon_number "2"; gene_type "
Dt_chr4	Cufflinks	exon	7770406	7770483	.	+	.	gene_id "XLOC_179835"; transcript_id "Novel_002508"; exon_number "3"; gene_type "
Dt_chr4	Cufflinks	exon	7770580	7770633	.	+	.	gene_id "XLOC_179835"; transcript_id "Novel_002508"; exon_number "4"; gene_type "
Dt_chr4	Cufflinks	exon	7770768	7770838	.	+	.	gene_id "XLOC_179835"; transcript_id "Novel_002508"; exon_number "5"; gene_type "
Dt_chr4	Cufflinks	exon	7771273	7771509	.	+	.	gene_id "XLOC_179835"; transcript_id "Novel_002508"; exon_number "6"; gene_type "
Dt_chr4	Cufflinks	exon	9821979	9822222	.	+	.	gene_id "XLOC_179924"; transcript_id "Novel_002509"; exon_number "1"; gene_type "
Dt_chr4	Cufflinks	exon	9822988	9823091	.	+	.	gene_id "XLOC_179924"; transcript_id "Novel_002509"; exon_number "2"; gene_type "
Dt_chr4	Cufflinks	exon	9823740	9823801	.	+	.	gene_id "XLOC_179924"; transcript_id "Novel_002509"; exon_number "3"; gene_type "
Dt_chr4	Cufflinks	exon	9821979	9822222	.	+	.	gene_id "XLOC_179924"; transcript_id "Novel_002510"; exon_number "1"; gene_type "
Dt_chr4	Cufflinks	exon	9823000	9823091	.	+	.	gene_id "XLOC_179924"; transcript_id "Novel_002510"; exon_number "2"; gene_type "
Dt_chr4	Cufflinks	exon	9823740	9823802	.	+	.	gene_id "XLOC_179924"; transcript_id "Novel_002510"; exon_number "3"; gene_type "
Dt_chr4	Cufflinks	exon	10054491	10056855	.	+	.	gene_id "XLOC_179939"; transcript_id "Novel_002511"; exon_number "1"; gene_type "
Dt_chr4	Cufflinks	exon	10056953	10058082	.	+	.	gene_id "XLOC_179939"; transcript_id "Novel_002511"; exon_number "2"; gene_type "
Dt_chr4	Cufflinks	exon	10054504	10055479	.	+	.	gene_id "XLOC_179939"; transcript_id "Novel_002512"; exon_number "1"; gene_type "
Dt_chr4	Cufflinks	exon	10055563	10056847	.	+	.	gene_id "XLOC_179939"; transcript_id "Novel_002512"; exon_number "2"; gene_type "
Dt_chr4	Cufflinks	exon	11295772	11296031	.	+	.	gene_id "XLOC_180024"; transcript_id "Novel_002513"; exon_number "1"; gene_type "
Dt_chr4	Cufflinks	exon	11296267	11296359	.	+	.	gene_id "XLOC_180024"; transcript_id "Novel_002513"; exon_number "2"; gene_type "
Dt_chr4	Cufflinks	exon	11297336	11297397	.	+	.	gene_id "XLOC_180024"; transcript_id "Novel_002513"; exon_number "3"; gene_type "
Dt_chr4	Cufflinks	exon	11297526	11298912	.	+	.	gene_id "XLOC_180024"; transcript_id "Novel_002513"; exon_number "4"; gene_type "
Dt_chr4	Cufflinks	exon	11299002	11299688	.	+	.	gene_id "XLOC_180024"; transcript_id "Novel_002513"; exon_number "5"; gene_type "
Dt_chr4	Cufflinks	exon	11299783	11299866	.	+	.	gene_id "XLOC_180024"; transcript_id "Novel_002513"; exon_number "6"; gene_type "
Dt_chr4	Cufflinks	exon	11300059	11300134	.	+	.	gene_id "XLOC_180024"; transcript_id "Novel_002513"; exon_number "7"; gene_type "
Dt_chr4	Cufflinks	exon	11300273	11300589	.	+	.	gene_id "XLOC_180024"; transcript_id "Novel_002513"; exon_number "8"; gene_type "

Dt_chr4	Cufflinks	exon	11300695	11302813	.	+	.	gene_id "XLOC_180024"; transcript_id "Novel_002513"; exon_number "9"; gene_type "
Dt_chr4	Cufflinks	exon	11295883	11296031	.	+	.	gene_id "XLOC_180024"; transcript_id "Novel_002514"; exon_number "1"; gene_type "
Dt_chr4	Cufflinks	exon	11296267	11296359	.	+	.	gene_id "XLOC_180024"; transcript_id "Novel_002514"; exon_number "2"; gene_type "
Dt_chr4	Cufflinks	exon	11297526	11298912	.	+	.	gene_id "XLOC_180024"; transcript_id "Novel_002514"; exon_number "3"; gene_type "
Dt_chr4	Cufflinks	exon	11299002	11299688	.	+	.	gene_id "XLOC_180024"; transcript_id "Novel_002514"; exon_number "4"; gene_type "
Dt_chr4	Cufflinks	exon	11299783	11299866	.	+	.	gene_id "XLOC_180024"; transcript_id "Novel_002514"; exon_number "5"; gene_type "
Dt_chr4	Cufflinks	exon	11300059	11300134	.	+	.	gene_id "XLOC_180024"; transcript_id "Novel_002514"; exon_number "6"; gene_type "
Dt_chr4	Cufflinks	exon	11300273	11300589	.	+	.	gene_id "XLOC_180024"; transcript_id "Novel_002514"; exon_number "7"; gene_type "
Dt_chr4	Cufflinks	exon	11300695	11301977	.	+	.	gene_id "XLOC_180024"; transcript_id "Novel_002514"; exon_number "8"; gene_type "
Dt_chr4	Cufflinks	exon	11302068	11302781	.	+	.	gene_id "XLOC_180024"; transcript_id "Novel_002514"; exon_number "9"; gene_type "
Dt_chr4	Cufflinks	exon	14372817	14372986	.	+	.	gene_id "XLOC_180170"; transcript_id "Novel_002515"; exon_number "1"; gene_type "
Dt_chr4	Cufflinks	exon	14373260	14373327	.	+	.	gene_id "XLOC_180170"; transcript_id "Novel_002515"; exon_number "2"; gene_type "
Dt_chr4	Cufflinks	exon	14374070	14375284	.	+	.	gene_id "XLOC_180170"; transcript_id "Novel_002515"; exon_number "3"; gene_type "
Dt_chr4	Cufflinks	exon	14372817	14372986	.	+	.	gene_id "XLOC_180170"; transcript_id "Novel_002516"; exon_number "1"; gene_type "
Dt_chr4	Cufflinks	exon	14373260	14373327	.	+	.	gene_id "XLOC_180170"; transcript_id "Novel_002516"; exon_number "2"; gene_type "
Dt_chr4	Cufflinks	exon	14374273	14375284	.	+	.	gene_id "XLOC_180170"; transcript_id "Novel_002516"; exon_number "3"; gene_type "
Dt_chr4	Cufflinks	exon	14373157	14373327	.	+	.	gene_id "XLOC_180170"; transcript_id "Novel_002517"; exon_number "1"; gene_type "
Dt_chr4	Cufflinks	exon	14374273	14375284	.	+	.	gene_id "XLOC_180170"; transcript_id "Novel_002517"; exon_number "2"; gene_type "
Dt_chr4	Cufflinks	exon	15060058	15060411	.	+	.	gene_id "XLOC_180239"; transcript_id "Novel_002518"; exon_number "1"; gene_type "
Dt_chr4	Cufflinks	exon	15060764	15061163	.	+	.	gene_id "XLOC_180239"; transcript_id "Novel_002518"; exon_number "2"; gene_type "
Dt_chr4	Cufflinks	exon	15061253	15063254	.	+	.	gene_id "XLOC_180239"; transcript_id "Novel_002518"; exon_number "3"; gene_type "
Dt_chr4	Cufflinks	exon	15060063	15060856	.	+	.	gene_id "XLOC_180239"; transcript_id "Novel_002519"; exon_number "1"; gene_type "
Dt_chr4	Cufflinks	exon	15061055	15061163	.	+	.	gene_id "XLOC_180239"; transcript_id "Novel_002519"; exon_number "2"; gene_type "
Dt_chr4	Cufflinks	exon	15061253	15062462	.	+	.	gene_id "XLOC_180239"; transcript_id "Novel_002519"; exon_number "3"; gene_type "
Dt_chr4	Cufflinks	exon	15060063	15060411	.	+	.	gene_id "XLOC_180239"; transcript_id "Novel_002520"; exon_number "1"; gene_type "
Dt_chr4	Cufflinks	exon	15061055	15061163	.	+	.	gene_id "XLOC_180239"; transcript_id "Novel_002520"; exon_number "2"; gene_type "
Dt_chr4	Cufflinks	exon	15061253	15062462	.	+	.	gene_id "XLOC_180239"; transcript_id "Novel_002520"; exon_number "3"; gene_type "
Dt_chr4	Cufflinks	exon	15340642	15340862	.	+	.	gene_id "XLOC_180279"; transcript_id "Novel_002521"; exon_number "1"; gene_type "
Dt_chr4	Cufflinks	exon	15503777	15504068	.	+	.	gene_id "XLOC_180295"; transcript_id "Novel_002522"; exon_number "1"; gene_type "
Dt_chr4	Cufflinks	exon	15505057	15505406	.	+	.	gene_id "XLOC_180295"; transcript_id "Novel_002522"; exon_number "2"; gene_type "
Dt_chr4	Cufflinks	exon	15505483	15507645	.	+	.	gene_id "XLOC_180295"; transcript_id "Novel_002522"; exon_number "3"; gene_type "
Dt_chr4	Cufflinks	exon	15503876	15504068	.	+	.	gene_id "XLOC_180295"; transcript_id "Novel_002523"; exon_number "1"; gene_type "
Dt_chr4	Cufflinks	exon	15505483	15508015	.	+	.	gene_id "XLOC_180295"; transcript_id "Novel_002523"; exon_number "2"; gene_type "
Dt_chr4	Cufflinks	exon	15503887	15504068	.	+	.	gene_id "XLOC_180295"; transcript_id "Novel_002524"; exon_number "1"; gene_type "
Dt_chr4	Cufflinks	exon	15505057	15508015	.	+	.	gene_id "XLOC_180295"; transcript_id "Novel_002524"; exon_number "2"; gene_type "

Dt_chr4	Cufflinks	exon	15503887	15504068	.	+	.	gene_id "XLOC_180295"; transcript_id "Novel_002525"; exon_number "1"; gene_type "
Dt_chr4	Cufflinks	exon	15505502	15508015	.	+	.	gene_id "XLOC_180295"; transcript_id "Novel_002525"; exon_number "2"; gene_type "
Dt_chr4	Cufflinks	exon	15504709	15505406	.	+	.	gene_id "XLOC_180295"; transcript_id "Novel_002526"; exon_number "1"; gene_type "
Dt_chr4	Cufflinks	exon	15505483	15507645	.	+	.	gene_id "XLOC_180295"; transcript_id "Novel_002526"; exon_number "2"; gene_type "
Dt_chr4	Cufflinks	exon	16522179	16525604	.	+	.	gene_id "XLOC_180362"; transcript_id "Novel_002527"; exon_number "1"; gene_type "
Dt_chr4	Cufflinks	exon	16525738	16525830	.	+	.	gene_id "XLOC_180362"; transcript_id "Novel_002527"; exon_number "2"; gene_type "
Dt_chr4	Cufflinks	exon	16525992	16526264	.	+	.	gene_id "XLOC_180362"; transcript_id "Novel_002527"; exon_number "3"; gene_type "
Dt_chr4	Cufflinks	exon	16522180	16522257	.	+	.	gene_id "XLOC_180362"; transcript_id "Novel_002528"; exon_number "1"; gene_type "
Dt_chr4	Cufflinks	exon	16523181	16523242	.	+	.	gene_id "XLOC_180362"; transcript_id "Novel_002528"; exon_number "2"; gene_type "
Dt_chr4	Cufflinks	exon	16523592	16523702	.	+	.	gene_id "XLOC_180362"; transcript_id "Novel_002528"; exon_number "3"; gene_type "
Dt_chr4	Cufflinks	exon	16523787	16524040	.	+	.	gene_id "XLOC_180362"; transcript_id "Novel_002528"; exon_number "4"; gene_type "
Dt_chr4	Cufflinks	exon	16525485	16525604	.	+	.	gene_id "XLOC_180362"; transcript_id "Novel_002528"; exon_number "5"; gene_type "
Dt_chr4	Cufflinks	exon	16525738	16525830	.	+	.	gene_id "XLOC_180362"; transcript_id "Novel_002528"; exon_number "6"; gene_type "
Dt_chr4	Cufflinks	exon	16525992	16526197	.	+	.	gene_id "XLOC_180362"; transcript_id "Novel_002528"; exon_number "7"; gene_type "
Dt_chr4	Cufflinks	exon	20177563	20178052	.	+	.	gene_id "XLOC_180544"; transcript_id "Novel_002529"; exon_number "1"; gene_type "
Dt_chr4	Cufflinks	exon	20178180	20178498	.	+	.	gene_id "XLOC_180544"; transcript_id "Novel_002529"; exon_number "2"; gene_type "
Dt_chr4	Cufflinks	exon	20178611	20178703	.	+	.	gene_id "XLOC_180544"; transcript_id "Novel_002529"; exon_number "3"; gene_type "
Dt_chr4	Cufflinks	exon	20178797	20178887	.	+	.	gene_id "XLOC_180544"; transcript_id "Novel_002529"; exon_number "4"; gene_type "
Dt_chr4	Cufflinks	exon	20179425	20179491	.	+	.	gene_id "XLOC_180544"; transcript_id "Novel_002529"; exon_number "5"; gene_type "
Dt_chr4	Cufflinks	exon	20180127	20180169	.	+	.	gene_id "XLOC_180544"; transcript_id "Novel_002529"; exon_number "6"; gene_type "
Dt_chr4	Cufflinks	exon	20180562	20180733	.	+	.	gene_id "XLOC_180544"; transcript_id "Novel_002529"; exon_number "7"; gene_type "
Dt_chr4	Cufflinks	exon	20180818	20180916	.	+	.	gene_id "XLOC_180544"; transcript_id "Novel_002529"; exon_number "8"; gene_type "
Dt_chr4	Cufflinks	exon	20181019	20181087	.	+	.	gene_id "XLOC_180544"; transcript_id "Novel_002529"; exon_number "9"; gene_type "
Dt_chr4	Cufflinks	exon	20181356	20181494	.	+	.	gene_id "XLOC_180544"; transcript_id "Novel_002529"; exon_number "10"; gene_type
Dt_chr4	Cufflinks	exon	20181744	20181945	.	+	.	gene_id "XLOC_180544"; transcript_id "Novel_002529"; exon_number "11"; gene_type
Dt_chr4	Cufflinks	exon	20182583	20182686	.	+	.	gene_id "XLOC_180544"; transcript_id "Novel_002529"; exon_number "12"; gene_type
Dt_chr4	Cufflinks	exon	20182846	20182885	.	+	.	gene_id "XLOC_180544"; transcript_id "Novel_002529"; exon_number "13"; gene_type
Dt_chr4	Cufflinks	exon	20183604	20184943	.	+	.	gene_id "XLOC_180544"; transcript_id "Novel_002529"; exon_number "14"; gene_type
Dt_chr4	Cufflinks	exon	20177610	20178052	.	+	.	gene_id "XLOC_180544"; transcript_id "Novel_002530"; exon_number "1"; gene_type "
Dt_chr4	Cufflinks	exon	20178180	20178498	.	+	.	gene_id "XLOC_180544"; transcript_id "Novel_002530"; exon_number "2"; gene_type "
Dt_chr4	Cufflinks	exon	20178611	20178703	.	+	.	gene_id "XLOC_180544"; transcript_id "Novel_002530"; exon_number "3"; gene_type "
Dt_chr4	Cufflinks	exon	20178797	20178887	.	+	.	gene_id "XLOC_180544"; transcript_id "Novel_002530"; exon_number "4"; gene_type "
Dt_chr4	Cufflinks	exon	20179425	20179491	.	+	.	gene_id "XLOC_180544"; transcript_id "Novel_002530"; exon_number "5"; gene_type "
Dt_chr4	Cufflinks	exon	20180127	20180169	.	+	.	gene_id "XLOC_180544"; transcript_id "Novel_002530"; exon_number "6"; gene_type "
Dt_chr4	Cufflinks	exon	20180562	20180733	.	+	.	gene_id "XLOC_180544"; transcript_id "Novel_002530"; exon_number "7"; gene_type "

Dt_chr4	Cufflinks	exon	20180818	20180916	.	+	.	gene_id "XLOC_180544"; transcript_id "Novel_002530"; exon_number "8"; gene_type "
Dt_chr4	Cufflinks	exon	20181019	20181087	.	+	.	gene_id "XLOC_180544"; transcript_id "Novel_002530"; exon_number "9"; gene_type "
Dt_chr4	Cufflinks	exon	20181356	20181494	.	+	.	gene_id "XLOC_180544"; transcript_id "Novel_002530"; exon_number "10"; gene_type
Dt_chr4	Cufflinks	exon	20181744	20181945	.	+	.	gene_id "XLOC_180544"; transcript_id "Novel_002530"; exon_number "11"; gene_type
Dt_chr4	Cufflinks	exon	20182583	20182686	.	+	.	gene_id "XLOC_180544"; transcript_id "Novel_002530"; exon_number "12"; gene_type
Dt_chr4	Cufflinks	exon	20182846	20182885	.	+	.	gene_id "XLOC_180544"; transcript_id "Novel_002530"; exon_number "13"; gene_type
Dt_chr4	Cufflinks	exon	20183604	20183821	.	+	.	gene_id "XLOC_180544"; transcript_id "Novel_002530"; exon_number "14"; gene_type
Dt_chr4	Cufflinks	exon	20184032	20184097	.	+	.	gene_id "XLOC_180544"; transcript_id "Novel_002530"; exon_number "15"; gene_type
Dt_chr4	Cufflinks	exon	20184219	20184979	.	+	.	gene_id "XLOC_180544"; transcript_id "Novel_002530"; exon_number "16"; gene_type
Dt_chr4	Cufflinks	exon	20177764	20178052	.	+	.	gene_id "XLOC_180544"; transcript_id "Novel_002531"; exon_number "1"; gene_type "
Dt_chr4	Cufflinks	exon	20178180	20178498	.	+	.	gene_id "XLOC_180544"; transcript_id "Novel_002531"; exon_number "2"; gene_type "
Dt_chr4	Cufflinks	exon	20178611	20178703	.	+	.	gene_id "XLOC_180544"; transcript_id "Novel_002531"; exon_number "3"; gene_type "
Dt_chr4	Cufflinks	exon	20178797	20178887	.	+	.	gene_id "XLOC_180544"; transcript_id "Novel_002531"; exon_number "4"; gene_type "
Dt_chr4	Cufflinks	exon	20179425	20179491	.	+	.	gene_id "XLOC_180544"; transcript_id "Novel_002531"; exon_number "5"; gene_type "
Dt_chr4	Cufflinks	exon	20180127	20180169	.	+	.	gene_id "XLOC_180544"; transcript_id "Novel_002531"; exon_number "6"; gene_type "
Dt_chr4	Cufflinks	exon	20180562	20180733	.	+	.	gene_id "XLOC_180544"; transcript_id "Novel_002531"; exon_number "7"; gene_type "
Dt_chr4	Cufflinks	exon	20180818	20180916	.	+	.	gene_id "XLOC_180544"; transcript_id "Novel_002531"; exon_number "8"; gene_type "
Dt_chr4	Cufflinks	exon	20181019	20181087	.	+	.	gene_id "XLOC_180544"; transcript_id "Novel_002531"; exon_number "9"; gene_type "
Dt_chr4	Cufflinks	exon	20181356	20181494	.	+	.	gene_id "XLOC_180544"; transcript_id "Novel_002531"; exon_number "10"; gene_type
Dt_chr4	Cufflinks	exon	20181744	20181945	.	+	.	gene_id "XLOC_180544"; transcript_id "Novel_002531"; exon_number "11"; gene_type
Dt_chr4	Cufflinks	exon	20182583	20182686	.	+	.	gene_id "XLOC_180544"; transcript_id "Novel_002531"; exon_number "12"; gene_type
Dt_chr4	Cufflinks	exon	20182846	20182885	.	+	.	gene_id "XLOC_180544"; transcript_id "Novel_002531"; exon_number "13"; gene_type
Dt_chr4	Cufflinks	exon	20183604	20184369	.	+	.	gene_id "XLOC_180544"; transcript_id "Novel_002531"; exon_number "14"; gene_type
Dt_chr4	Cufflinks	exon	20184540	20185464	.	+	.	gene_id "XLOC_180544"; transcript_id "Novel_002531"; exon_number "15"; gene_type
Dt_chr4	Cufflinks	exon	20177838	20178052	.	+	.	gene_id "XLOC_180544"; transcript_id "Novel_002532"; exon_number "1"; gene_type "
Dt_chr4	Cufflinks	exon	20178180	20178498	.	+	.	gene_id "XLOC_180544"; transcript_id "Novel_002532"; exon_number "2"; gene_type "
Dt_chr4	Cufflinks	exon	20178611	20178703	.	+	.	gene_id "XLOC_180544"; transcript_id "Novel_002532"; exon_number "3"; gene_type "
Dt_chr4	Cufflinks	exon	20178797	20178887	.	+	.	gene_id "XLOC_180544"; transcript_id "Novel_002532"; exon_number "4"; gene_type "
Dt_chr4	Cufflinks	exon	20179425	20179491	.	+	.	gene_id "XLOC_180544"; transcript_id "Novel_002532"; exon_number "5"; gene_type "
Dt_chr4	Cufflinks	exon	20180127	20180169	.	+	.	gene_id "XLOC_180544"; transcript_id "Novel_002532"; exon_number "6"; gene_type "
Dt_chr4	Cufflinks	exon	20180562	20180733	.	+	.	gene_id "XLOC_180544"; transcript_id "Novel_002532"; exon_number "7"; gene_type "
Dt_chr4	Cufflinks	exon	20180818	20180916	.	+	.	gene_id "XLOC_180544"; transcript_id "Novel_002532"; exon_number "8"; gene_type "
Dt_chr4	Cufflinks	exon	20181019	20181087	.	+	.	gene_id "XLOC_180544"; transcript_id "Novel_002532"; exon_number "9"; gene_type "
Dt_chr4	Cufflinks	exon	20181356	20181494	.	+	.	gene_id "XLOC_180544"; transcript_id "Novel_002532"; exon_number "10"; gene_type
Dt_chr4	Cufflinks	exon	20181744	20181945	.	+	.	gene_id "XLOC_180544"; transcript_id "Novel_002532"; exon_number "11"; gene_type

Dt_chr4	Cufflinks	exon	20182583	20182686	.	+	.	gene_id "XLOC_180544"; transcript_id "Novel_002532"; exon_number "12"; gene_type
Dt_chr4	Cufflinks	exon	20182846	20182885	.	+	.	gene_id "XLOC_180544"; transcript_id "Novel_002532"; exon_number "13"; gene_type
Dt_chr4	Cufflinks	exon	20183604	20183821	.	+	.	gene_id "XLOC_180544"; transcript_id "Novel_002532"; exon_number "14"; gene_type
Dt_chr4	Cufflinks	exon	20184032	20184097	.	+	.	gene_id "XLOC_180544"; transcript_id "Novel_002532"; exon_number "15"; gene_type
Dt_chr4	Cufflinks	exon	20184219	20184369	.	+	.	gene_id "XLOC_180544"; transcript_id "Novel_002532"; exon_number "16"; gene_type
Dt_chr4	Cufflinks	exon	20184540	20185542	.	+	.	gene_id "XLOC_180544"; transcript_id "Novel_002532"; exon_number "17"; gene_type
Dt_chr4	Cufflinks	exon	22208153	22208546	.	+	.	gene_id "XLOC_180682"; transcript_id "Novel_002533"; exon_number "1"; gene_type "
Dt_chr4	Cufflinks	exon	22208652	22209003	.	+	.	gene_id "XLOC_180682"; transcript_id "Novel_002533"; exon_number "2"; gene_type "
Dt_chr4	Cufflinks	exon	22208233	22208542	.	+	.	gene_id "XLOC_180682"; transcript_id "Novel_002534"; exon_number "1"; gene_type "
Dt_chr4	Cufflinks	exon	22208652	22209026	.	+	.	gene_id "XLOC_180682"; transcript_id "Novel_002534"; exon_number "2"; gene_type "
Dt_chr4	Cufflinks	exon	22208236	22208546	.	+	.	gene_id "XLOC_180682"; transcript_id "Novel_002535"; exon_number "1"; gene_type "
Dt_chr4	Cufflinks	exon	22208655	22209028	.	+	.	gene_id "XLOC_180682"; transcript_id "Novel_002535"; exon_number "2"; gene_type "
Dt_chr4	Cufflinks	exon	22704104	22704197	.	+	.	gene_id "XLOC_180740"; transcript_id "Novel_002536"; exon_number "1"; gene_type "
Dt_chr4	Cufflinks	exon	22704290	22704379	.	+	.	gene_id "XLOC_180740"; transcript_id "Novel_002536"; exon_number "2"; gene_type "
Dt_chr4	Cufflinks	exon	22704474	22704602	.	+	.	gene_id "XLOC_180740"; transcript_id "Novel_002536"; exon_number "3"; gene_type "
Dt_chr4	Cufflinks	exon	22704706	22704888	.	+	.	gene_id "XLOC_180740"; transcript_id "Novel_002536"; exon_number "4"; gene_type "
Dt_chr4	Cufflinks	exon	22705639	22706029	.	+	.	gene_id "XLOC_180740"; transcript_id "Novel_002536"; exon_number "5"; gene_type "
Dt_chr4	Cufflinks	exon	23282549	23282811	.	+	.	gene_id "XLOC_180791"; transcript_id "Novel_002537"; exon_number "1"; gene_type "
Dt_chr4	Cufflinks	exon	23396111	23396148	.	+	.	gene_id "XLOC_180799"; transcript_id "Novel_002538"; exon_number "1"; gene_type "
Dt_chr4	Cufflinks	exon	23396390	23396413	.	+	.	gene_id "XLOC_180799"; transcript_id "Novel_002538"; exon_number "2"; gene_type "
Dt_chr4	Cufflinks	exon	23396486	23397168	.	+	.	gene_id "XLOC_180799"; transcript_id "Novel_002538"; exon_number "3"; gene_type "
Dt_chr4	Cufflinks	exon	23397505	23397611	.	+	.	gene_id "XLOC_180799"; transcript_id "Novel_002538"; exon_number "4"; gene_type "
Dt_chr4	Cufflinks	exon	23397911	23397958	.	+	.	gene_id "XLOC_180799"; transcript_id "Novel_002538"; exon_number "5"; gene_type "
Dt_chr4	Cufflinks	exon	23398786	23398933	.	+	.	gene_id "XLOC_180799"; transcript_id "Novel_002538"; exon_number "6"; gene_type "
Dt_chr4	Cufflinks	exon	26678493	26679374	.	+	.	gene_id "XLOC_180952"; transcript_id "Novel_002539"; exon_number "1"; gene_type "
Dt_chr4	Cufflinks	exon	26679788	26679949	.	+	.	gene_id "XLOC_180952"; transcript_id "Novel_002539"; exon_number "2"; gene_type "
Dt_chr4	Cufflinks	exon	26680267	26680350	.	+	.	gene_id "XLOC_180952"; transcript_id "Novel_002539"; exon_number "3"; gene_type "
Dt_chr4	Cufflinks	exon	26719047	26719388	.	+	.	gene_id "XLOC_180952"; transcript_id "Novel_002539"; exon_number "4"; gene_type "
Dt_chr4	Cufflinks	exon	26716935	26717037	.	+	.	gene_id "XLOC_180952"; transcript_id "Novel_002540"; exon_number "1"; gene_type "
Dt_chr4	Cufflinks	exon	26717335	26717532	.	+	.	gene_id "XLOC_180952"; transcript_id "Novel_002540"; exon_number "2"; gene_type "
Dt_chr4	Cufflinks	exon	26717683	26717994	.	+	.	gene_id "XLOC_180952"; transcript_id "Novel_002540"; exon_number "3"; gene_type "
Dt_chr4	Cufflinks	exon	26718400	26718561	.	+	.	gene_id "XLOC_180952"; transcript_id "Novel_002540"; exon_number "4"; gene_type "
Dt_chr4	Cufflinks	exon	26718880	26718963	.	+	.	gene_id "XLOC_180952"; transcript_id "Novel_002540"; exon_number "5"; gene_type "
Dt_chr4	Cufflinks	exon	26719047	26719403	.	+	.	gene_id "XLOC_180952"; transcript_id "Novel_002540"; exon_number "6"; gene_type "
Dt_chr4	Cufflinks	exon	26716961	26717994	.	+	.	gene_id "XLOC_180952"; transcript_id "Novel_002541"; exon_number "1"; gene_type "

Dt_chr4	Cufflinks	exon	26718400	26718561	.	+	.	gene_id "XLOC_180952"; transcript_id "Novel_002541"; exon_number "2"; gene_type "
Dt_chr4	Cufflinks	exon	26718880	26719403	.	+	.	gene_id "XLOC_180952"; transcript_id "Novel_002541"; exon_number "3"; gene_type "
Dt_chr4	Cufflinks	exon	26834520	26835921	.	+	.	gene_id "XLOC_180960"; transcript_id "Novel_002542"; exon_number "1"; gene_type "
Dt_chr4	Cufflinks	exon	27190426	27190629	.	+	.	gene_id "XLOC_180987"; transcript_id "Novel_002543"; exon_number "1"; gene_type "
Dt_chr4	Cufflinks	exon	27313589	27314783	.	+	.	gene_id "XLOC_180998"; transcript_id "Novel_002544"; exon_number "1"; gene_type "
Dt_chr4	Cufflinks	exon	27316723	27317594	.	+	.	gene_id "XLOC_180998"; transcript_id "Novel_002544"; exon_number "2"; gene_type "
Dt_chr4	Cufflinks	exon	27328269	27330171	.	+	.	gene_id "XLOC_181002"; transcript_id "Novel_002545"; exon_number "1"; gene_type "
Dt_chr4	Cufflinks	exon	27506500	27506761	.	+	.	gene_id "XLOC_181036"; transcript_id "Novel_002546"; exon_number "1"; gene_type "
Dt_chr4	Cufflinks	exon	27506878	27508847	.	+	.	gene_id "XLOC_181036"; transcript_id "Novel_002546"; exon_number "2"; gene_type "
Dt_chr4	Cufflinks	exon	27508953	27509885	.	+	.	gene_id "XLOC_181036"; transcript_id "Novel_002546"; exon_number "3"; gene_type "
Dt_chr4	Cufflinks	exon	27738093	27738218	.	+	.	gene_id "XLOC_181056"; transcript_id "Novel_002547"; exon_number "1"; gene_type "
Dt_chr4	Cufflinks	exon	27738354	27738503	.	+	.	gene_id "XLOC_181056"; transcript_id "Novel_002547"; exon_number "2"; gene_type "
Dt_chr4	Cufflinks	exon	27738622	27738711	.	+	.	gene_id "XLOC_181056"; transcript_id "Novel_002547"; exon_number "3"; gene_type "
Dt_chr4	Cufflinks	exon	27739348	27739610	.	+	.	gene_id "XLOC_181056"; transcript_id "Novel_002547"; exon_number "4"; gene_type "
Dt_chr4	Cufflinks	exon	27848992	27849702	.	+	.	gene_id "XLOC_181067"; transcript_id "Novel_002548"; exon_number "1"; gene_type "
Dt_chr4	Cufflinks	exon	27849800	27849950	.	+	.	gene_id "XLOC_181067"; transcript_id "Novel_002548"; exon_number "2"; gene_type "
Dt_chr4	Cufflinks	exon	27849017	27849718	.	+	.	gene_id "XLOC_181067"; transcript_id "Novel_002549"; exon_number "1"; gene_type "
Dt_chr4	Cufflinks	exon	27849800	27849921	.	+	.	gene_id "XLOC_181067"; transcript_id "Novel_002549"; exon_number "2"; gene_type "
Dt_chr4	Cufflinks	exon	31429827	31429931	.	+	.	gene_id "XLOC_181467"; transcript_id "Novel_002550"; exon_number "1"; gene_type "
Dt_chr4	Cufflinks	exon	31431246	31431910	.	+	.	gene_id "XLOC_181467"; transcript_id "Novel_002550"; exon_number "2"; gene_type "
Dt_chr4	Cufflinks	exon	34994029	34994078	.	+	.	gene_id "XLOC_181675"; transcript_id "Novel_002551"; exon_number "1"; gene_type "
Dt_chr4	Cufflinks	exon	34994270	34994365	.	+	.	gene_id "XLOC_181675"; transcript_id "Novel_002551"; exon_number "2"; gene_type "
Dt_chr4	Cufflinks	exon	34994488	34994565	.	+	.	gene_id "XLOC_181675"; transcript_id "Novel_002551"; exon_number "3"; gene_type "
Dt_chr4	Cufflinks	exon	34994660	34994836	.	+	.	gene_id "XLOC_181675"; transcript_id "Novel_002551"; exon_number "4"; gene_type "
Dt_chr4	Cufflinks	exon	34995715	34995783	.	+	.	gene_id "XLOC_181675"; transcript_id "Novel_002551"; exon_number "5"; gene_type "
Dt_chr4	Cufflinks	exon	34995875	34996639	.	+	.	gene_id "XLOC_181675"; transcript_id "Novel_002551"; exon_number "6"; gene_type "
Dt_chr4	Cufflinks	exon	2270302	2270577	.	-	.	gene_id "XLOC_181842"; transcript_id "Novel_002552"; exon_number "1"; gene_type "
Dt_chr4	Cufflinks	exon	2271534	2271615	.	-	.	gene_id "XLOC_181842"; transcript_id "Novel_002552"; exon_number "2"; gene_type "
Dt_chr4	Cufflinks	exon	2271716	2271804	.	-	.	gene_id "XLOC_181842"; transcript_id "Novel_002552"; exon_number "3"; gene_type "
Dt_chr4	Cufflinks	exon	2273998	2274109	.	-	.	gene_id "XLOC_181842"; transcript_id "Novel_002552"; exon_number "4"; gene_type "
Dt_chr4	Cufflinks	exon	2270302	2270577	.	-	.	gene_id "XLOC_181842"; transcript_id "Novel_002553"; exon_number "1"; gene_type "
Dt_chr4	Cufflinks	exon	2271534	2271627	.	-	.	gene_id "XLOC_181842"; transcript_id "Novel_002553"; exon_number "2"; gene_type "
Dt_chr4	Cufflinks	exon	2271716	2271804	.	-	.	gene_id "XLOC_181842"; transcript_id "Novel_002553"; exon_number "3"; gene_type "
Dt_chr4	Cufflinks	exon	2273998	2274109	.	-	.	gene_id "XLOC_181842"; transcript_id "Novel_002553"; exon_number "4"; gene_type "
Dt_chr4	Cufflinks	exon	6144844	6145080	.	-	.	gene_id "XLOC_182078"; transcript_id "Novel_002554"; exon_number "1"; gene_type "

Dt_chr4	Cufflinks	exon	6260674	6260958	.	-	.	gene_id "XLOC_182090"; transcript_id "Novel_002555"; exon_number "1"; gene_type "
Dt_chr4	Cufflinks	exon	6538887	6540239	.	-	.	gene_id "XLOC_182113"; transcript_id "Novel_002556"; exon_number "1"; gene_type "
Dt_chr4	Cufflinks	exon	6554933	6555428	.	-	.	gene_id "XLOC_182116"; transcript_id "Novel_002557"; exon_number "1"; gene_type "
Dt_chr4	Cufflinks	exon	6555799	6555879	.	-	.	gene_id "XLOC_182116"; transcript_id "Novel_002557"; exon_number "2"; gene_type "
Dt_chr4	Cufflinks	exon	6556780	6557025	.	-	.	gene_id "XLOC_182116"; transcript_id "Novel_002557"; exon_number "3"; gene_type "
Dt_chr4	Cufflinks	exon	14805526	14805559	.	-	.	gene_id "XLOC_182523"; transcript_id "Novel_002558"; exon_number "1"; gene_type "
Dt_chr4	Cufflinks	exon	14807498	14807623	.	-	.	gene_id "XLOC_182523"; transcript_id "Novel_002558"; exon_number "2"; gene_type "
Dt_chr4	Cufflinks	exon	14808041	14808567	.	-	.	gene_id "XLOC_182523"; transcript_id "Novel_002558"; exon_number "3"; gene_type "
Dt_chr4	Cufflinks	exon	14806903	14807623	.	-	.	gene_id "XLOC_182523"; transcript_id "Novel_002559"; exon_number "1"; gene_type "
Dt_chr4	Cufflinks	exon	14808041	14808518	.	-	.	gene_id "XLOC_182523"; transcript_id "Novel_002559"; exon_number "2"; gene_type "
Dt_chr4	Cufflinks	exon	20809178	20812259	.	-	.	gene_id "XLOC_182848"; transcript_id "Novel_002560"; exon_number "1"; gene_type "
Dt_chr4	Cufflinks	exon	20819486	20819659	.	-	.	gene_id "XLOC_182848"; transcript_id "Novel_002560"; exon_number "2"; gene_type "
Dt_chr4	Cufflinks	exon	20819864	20819941	.	-	.	gene_id "XLOC_182848"; transcript_id "Novel_002560"; exon_number "3"; gene_type "
Dt_chr4	Cufflinks	exon	20821096	20821215	.	-	.	gene_id "XLOC_182848"; transcript_id "Novel_002560"; exon_number "4"; gene_type "
Dt_chr4	Cufflinks	exon	20821324	20821724	.	-	.	gene_id "XLOC_182848"; transcript_id "Novel_002560"; exon_number "5"; gene_type "
Dt_chr4	Cufflinks	exon	20822433	20822517	.	-	.	gene_id "XLOC_182848"; transcript_id "Novel_002560"; exon_number "6"; gene_type "
Dt_chr4	Cufflinks	exon	20817935	20819659	.	-	.	gene_id "XLOC_182848"; transcript_id "Novel_002561"; exon_number "1"; gene_type "
Dt_chr4	Cufflinks	exon	20819864	20819941	.	-	.	gene_id "XLOC_182848"; transcript_id "Novel_002561"; exon_number "2"; gene_type "
Dt_chr4	Cufflinks	exon	20821096	20821215	.	-	.	gene_id "XLOC_182848"; transcript_id "Novel_002561"; exon_number "3"; gene_type "
Dt_chr4	Cufflinks	exon	20821324	20822506	.	-	.	gene_id "XLOC_182848"; transcript_id "Novel_002561"; exon_number "4"; gene_type "
Dt_chr4	Cufflinks	exon	20878653	20878897	.	-	.	gene_id "XLOC_182859"; transcript_id "Novel_002562"; exon_number "1"; gene_type "
Dt_chr4	Cufflinks	exon	20878988	20879107	.	-	.	gene_id "XLOC_182859"; transcript_id "Novel_002562"; exon_number "2"; gene_type "
Dt_chr4	Cufflinks	exon	20880013	20880106	.	-	.	gene_id "XLOC_182859"; transcript_id "Novel_002562"; exon_number "3"; gene_type "
Dt_chr4	Cufflinks	exon	20880570	20881022	.	-	.	gene_id "XLOC_182859"; transcript_id "Novel_002562"; exon_number "4"; gene_type "
Dt_chr4	Cufflinks	exon	23190819	23193888	.	-	.	gene_id "XLOC_183065"; transcript_id "Novel_002563"; exon_number "1"; gene_type "
Dt_chr4	Cufflinks	exon	23193980	23194413	.	-	.	gene_id "XLOC_183065"; transcript_id "Novel_002563"; exon_number "2"; gene_type "
Dt_chr4	Cufflinks	exon	23190820	23191360	.	-	.	gene_id "XLOC_183065"; transcript_id "Novel_002564"; exon_number "1"; gene_type "
Dt_chr4	Cufflinks	exon	23193840	23193888	.	-	.	gene_id "XLOC_183065"; transcript_id "Novel_002564"; exon_number "2"; gene_type "
Dt_chr4	Cufflinks	exon	23193980	23194411	.	-	.	gene_id "XLOC_183065"; transcript_id "Novel_002564"; exon_number "3"; gene_type "
Dt_chr4	Cufflinks	exon	26359417	26359627	.	-	.	gene_id "XLOC_183251"; transcript_id "Novel_002565"; exon_number "1"; gene_type "
Dt_chr4	Cufflinks	exon	26464532	26466164	.	-	.	gene_id "XLOC_183258"; transcript_id "Novel_002566"; exon_number "1"; gene_type "
Dt_chr4	Cufflinks	exon	26466292	26467255	.	-	.	gene_id "XLOC_183258"; transcript_id "Novel_002566"; exon_number "2"; gene_type "
Dt_chr4	Cufflinks	exon	26468335	26468700	.	-	.	gene_id "XLOC_183258"; transcript_id "Novel_002567"; exon_number "1"; gene_type "
Dt_chr4	Cufflinks	exon	26468799	26468904	.	-	.	gene_id "XLOC_183258"; transcript_id "Novel_002567"; exon_number "2"; gene_type "
Dt_chr4	Cufflinks	exon	26469638	26469692	.	-	.	gene_id "XLOC_183258"; transcript_id "Novel_002567"; exon_number "3"; gene_type "

Dt_chr4	Cufflinks	exon	26470703	26471030	.	-	.	gene_id "XLOC_183258"; transcript_id "Novel_002567"; exon_number "4"; gene_type "
Dt_chr4	Cufflinks	exon	26468335	26468700	.	-	.	gene_id "XLOC_183258"; transcript_id "Novel_002568"; exon_number "1"; gene_type "
Dt_chr4	Cufflinks	exon	26468799	26468904	.	-	.	gene_id "XLOC_183258"; transcript_id "Novel_002568"; exon_number "2"; gene_type "
Dt_chr4	Cufflinks	exon	26470703	26471030	.	-	.	gene_id "XLOC_183258"; transcript_id "Novel_002568"; exon_number "3"; gene_type "
Dt_chr4	Cufflinks	exon	26468372	26468700	.	-	.	gene_id "XLOC_183258"; transcript_id "Novel_002569"; exon_number "1"; gene_type "
Dt_chr4	Cufflinks	exon	26468799	26468904	.	-	.	gene_id "XLOC_183258"; transcript_id "Novel_002569"; exon_number "2"; gene_type "
Dt_chr4	Cufflinks	exon	26469512	26469543	.	-	.	gene_id "XLOC_183258"; transcript_id "Novel_002569"; exon_number "3"; gene_type "
Dt_chr4	Cufflinks	exon	26469638	26469692	.	-	.	gene_id "XLOC_183258"; transcript_id "Novel_002569"; exon_number "4"; gene_type "
Dt_chr4	Cufflinks	exon	26470703	26470993	.	-	.	gene_id "XLOC_183258"; transcript_id "Novel_002569"; exon_number "5"; gene_type "
Dt_chr4	Cufflinks	exon	26468395	26468700	.	-	.	gene_id "XLOC_183258"; transcript_id "Novel_002570"; exon_number "1"; gene_type "
Dt_chr4	Cufflinks	exon	26468799	26469627	.	-	.	gene_id "XLOC_183258"; transcript_id "Novel_002570"; exon_number "2"; gene_type "
Dt_chr4	Cufflinks	exon	27312879	27313097	.	-	.	gene_id "XLOC_183323"; transcript_id "Novel_002571"; exon_number "1"; gene_type "
Dt_chr4	Cufflinks	exon	27313345	27313435	.	-	.	gene_id "XLOC_183323"; transcript_id "Novel_002571"; exon_number "2"; gene_type "
Dt_chr4	Cufflinks	exon	27313548	27314799	.	-	.	gene_id "XLOC_183323"; transcript_id "Novel_002571"; exon_number "3"; gene_type "
Dt_chr4	Cufflinks	exon	27313081	27313435	.	-	.	gene_id "XLOC_183323"; transcript_id "Novel_002572"; exon_number "1"; gene_type "
Dt_chr4	Cufflinks	exon	27313548	27314865	.	-	.	gene_id "XLOC_183323"; transcript_id "Novel_002572"; exon_number "2"; gene_type "
Dt_chr4	Cufflinks	exon	27549157	27549659	.	-	.	gene_id "XLOC_183358"; transcript_id "Novel_002573"; exon_number "1"; gene_type "
Dt_chr4	Cufflinks	exon	27550121	27550163	.	-	.	gene_id "XLOC_183358"; transcript_id "Novel_002573"; exon_number "2"; gene_type "
Dt_chr4	Cufflinks	exon	27550519	27550610	.	-	.	gene_id "XLOC_183359"; transcript_id "Novel_002574"; exon_number "1"; gene_type "
Dt_chr4	Cufflinks	exon	27550841	27550970	.	-	.	gene_id "XLOC_183359"; transcript_id "Novel_002574"; exon_number "2"; gene_type "
Dt_chr4	Cufflinks	exon	27551143	27552149	.	-	.	gene_id "XLOC_183359"; transcript_id "Novel_002574"; exon_number "3"; gene_type "
Dt_chr4	Cufflinks	exon	27550554	27550610	.	-	.	gene_id "XLOC_183359"; transcript_id "Novel_002575"; exon_number "1"; gene_type "
Dt_chr4	Cufflinks	exon	27550841	27550970	.	-	.	gene_id "XLOC_183359"; transcript_id "Novel_002575"; exon_number "2"; gene_type "
Dt_chr4	Cufflinks	exon	27551143	27551372	.	-	.	gene_id "XLOC_183359"; transcript_id "Novel_002575"; exon_number "3"; gene_type "
Dt_chr4	Cufflinks	exon	27551451	27552303	.	-	.	gene_id "XLOC_183359"; transcript_id "Novel_002575"; exon_number "4"; gene_type "
Dt_chr4	Cufflinks	exon	27550860	27550970	.	-	.	gene_id "XLOC_183359"; transcript_id "Novel_002576"; exon_number "1"; gene_type "
Dt_chr4	Cufflinks	exon	27551143	27551191	.	-	.	gene_id "XLOC_183359"; transcript_id "Novel_002576"; exon_number "2"; gene_type "
Dt_chr4	Cufflinks	exon	27551451	27551507	.	-	.	gene_id "XLOC_183359"; transcript_id "Novel_002576"; exon_number "3"; gene_type "
Dt_chr4	Cufflinks	exon	27552475	27552754	.	-	.	gene_id "XLOC_183359"; transcript_id "Novel_002576"; exon_number "4"; gene_type "
Dt_chr4	Cufflinks	exon	29887744	29887816	.	-	.	gene_id "XLOC_183579"; transcript_id "Novel_002577"; exon_number "1"; gene_type "
Dt_chr4	Cufflinks	exon	29887919	29888030	.	-	.	gene_id "XLOC_183579"; transcript_id "Novel_002577"; exon_number "2"; gene_type "
Dt_chr4	Cufflinks	exon	29888116	29888367	.	-	.	gene_id "XLOC_183579"; transcript_id "Novel_002577"; exon_number "3"; gene_type "
Dt_chr4	Cufflinks	exon	29888468	29888569	.	-	.	gene_id "XLOC_183579"; transcript_id "Novel_002577"; exon_number "4"; gene_type "
Dt_chr4	Cufflinks	exon	29888649	29888766	.	-	.	gene_id "XLOC_183579"; transcript_id "Novel_002577"; exon_number "5"; gene_type "
Dt_chr4	Cufflinks	exon	29888913	29889051	.	-	.	gene_id "XLOC_183579"; transcript_id "Novel_002577"; exon_number "6"; gene_type "

Dt_chr4	Cufflinks	exon	29889288	29889475	.	-	.	gene_id "XLOC_183579"; transcript_id "Novel_002577"; exon_number "7"; gene_type "
Dt_chr4	Cufflinks	exon	29889559	29889669	.	-	.	gene_id "XLOC_183579"; transcript_id "Novel_002577"; exon_number "8"; gene_type "
Dt_chr4	Cufflinks	exon	29889812	29889874	.	-	.	gene_id "XLOC_183579"; transcript_id "Novel_002577"; exon_number "9"; gene_type "
Dt_chr4	Cufflinks	exon	29890473	29890572	.	-	.	gene_id "XLOC_183579"; transcript_id "Novel_002577"; exon_number "10"; gene_type
Dt_chr4	Cufflinks	exon	29890816	29891351	.	-	.	gene_id "XLOC_183579"; transcript_id "Novel_002577"; exon_number "11"; gene_type
Dt_chr4	Cufflinks	exon	29891767	29892077	.	-	.	gene_id "XLOC_183579"; transcript_id "Novel_002577"; exon_number "12"; gene_type
Dt_chr4	Cufflinks	exon	29891553	29891625	.	-	.	gene_id "XLOC_183579"; transcript_id "Novel_002578"; exon_number "1"; gene_type "
Dt_chr4	Cufflinks	exon	29891767	29892070	.	-	.	gene_id "XLOC_183579"; transcript_id "Novel_002578"; exon_number "2"; gene_type "
Dt_chr4	Cufflinks	exon	30683722	30684191	.	-	.	gene_id "XLOC_183682"; transcript_id "Novel_002579"; exon_number "1"; gene_type "
Dt_chr4	Cufflinks	exon	30684577	30684763	.	-	.	gene_id "XLOC_183682"; transcript_id "Novel_002579"; exon_number "2"; gene_type "
Dt_chr4	Cufflinks	exon	30684880	30685006	.	-	.	gene_id "XLOC_183682"; transcript_id "Novel_002579"; exon_number "3"; gene_type "
Dt_chr4	Cufflinks	exon	30685423	30686243	.	-	.	gene_id "XLOC_183682"; transcript_id "Novel_002579"; exon_number "4"; gene_type "
Dt_chr4	Cufflinks	exon	34203182	34204926	.	-	.	gene_id "XLOC_183937"; transcript_id "Novel_002580"; exon_number "1"; gene_type "
Dt_chr4	Cufflinks	exon	34205951	34207669	.	-	.	gene_id "XLOC_183937"; transcript_id "Novel_002580"; exon_number "2"; gene_type "
Dt_chr5	Cufflinks	exon	864084	864135	.	+	.	gene_id "XLOC_184163"; transcript_id "Novel_002581"; exon_number "1"; gene_type "
Dt_chr5	Cufflinks	exon	864215	864278	.	+	.	gene_id "XLOC_184163"; transcript_id "Novel_002581"; exon_number "2"; gene_type "
Dt_chr5	Cufflinks	exon	864887	865170	.	+	.	gene_id "XLOC_184163"; transcript_id "Novel_002581"; exon_number "3"; gene_type "
Dt_chr5	Cufflinks	exon	1454553	1455235	.	+	.	gene_id "XLOC_184214"; transcript_id "Novel_002582"; exon_number "1"; gene_type "
Dt_chr5	Cufflinks	exon	1455637	1455930	.	+	.	gene_id "XLOC_184214"; transcript_id "Novel_002582"; exon_number "2"; gene_type "
Dt_chr5	Cufflinks	exon	1456327	1456381	.	+	.	gene_id "XLOC_184214"; transcript_id "Novel_002582"; exon_number "3"; gene_type "
Dt_chr5	Cufflinks	exon	1456641	1456713	.	+	.	gene_id "XLOC_184214"; transcript_id "Novel_002582"; exon_number "4"; gene_type "
Dt_chr5	Cufflinks	exon	1456958	1457293	.	+	.	gene_id "XLOC_184214"; transcript_id "Novel_002582"; exon_number "5"; gene_type "
Dt_chr5	Cufflinks	exon	6581481	6581708	.	+	.	gene_id "XLOC_184578"; transcript_id "Novel_002583"; exon_number "1"; gene_type "
Dt_chr5	Cufflinks	exon	6581787	6581867	.	+	.	gene_id "XLOC_184578"; transcript_id "Novel_002583"; exon_number "2"; gene_type "
Dt_chr5	Cufflinks	exon	6581950	6582853	.	+	.	gene_id "XLOC_184578"; transcript_id "Novel_002583"; exon_number "3"; gene_type "
Dt_chr5	Cufflinks	exon	6824656	6825023	.	+	.	gene_id "XLOC_184617"; transcript_id "Novel_002584"; exon_number "1"; gene_type "
Dt_chr5	Cufflinks	exon	7196614	7197266	.	+	.	gene_id "XLOC_184689"; transcript_id "Novel_002585"; exon_number "1"; gene_type "
Dt_chr5	Cufflinks	exon	10460543	10460810	.	+	.	gene_id "XLOC_185189"; transcript_id "Novel_002586"; exon_number "1"; gene_type "
Dt_chr5	Cufflinks	exon	10460894	10461061	.	+	.	gene_id "XLOC_185189"; transcript_id "Novel_002586"; exon_number "2"; gene_type "
Dt_chr5	Cufflinks	exon	10461143	10461441	.	+	.	gene_id "XLOC_185189"; transcript_id "Novel_002586"; exon_number "3"; gene_type "
Dt_chr5	Cufflinks	exon	10651642	10651848	.	+	.	gene_id "XLOC_185222"; transcript_id "Novel_002587"; exon_number "1"; gene_type "
Dt_chr5	Cufflinks	exon	10652053	10652086	.	+	.	gene_id "XLOC_185222"; transcript_id "Novel_002587"; exon_number "2"; gene_type "
Dt_chr5	Cufflinks	exon	10652204	10652265	.	+	.	gene_id "XLOC_185222"; transcript_id "Novel_002587"; exon_number "3"; gene_type "
Dt_chr5	Cufflinks	exon	10652976	10653028	.	+	.	gene_id "XLOC_185222"; transcript_id "Novel_002587"; exon_number "4"; gene_type "
Dt_chr5	Cufflinks	exon	10653415	10653722	.	+	.	gene_id "XLOC_185222"; transcript_id "Novel_002587"; exon_number "5"; gene_type "

Dt_chr5	Cufflinks	exon	11395419	11396369	.	+	.	gene_id "XLOC_185347"; transcript_id "Novel_002588"; exon_number "1"; gene_type "
Dt_chr5	Cufflinks	exon	11396538	11396616	.	+	.	gene_id "XLOC_185347"; transcript_id "Novel_002588"; exon_number "2"; gene_type "
Dt_chr5	Cufflinks	exon	11396774	11396942	.	+	.	gene_id "XLOC_185347"; transcript_id "Novel_002588"; exon_number "3"; gene_type "
Dt_chr5	Cufflinks	exon	11395609	11396369	.	+	.	gene_id "XLOC_185347"; transcript_id "Novel_002589"; exon_number "1"; gene_type "
Dt_chr5	Cufflinks	exon	11396538	11396616	.	+	.	gene_id "XLOC_185347"; transcript_id "Novel_002589"; exon_number "2"; gene_type "
Dt_chr5	Cufflinks	exon	11396774	11396833	.	+	.	gene_id "XLOC_185347"; transcript_id "Novel_002589"; exon_number "3"; gene_type "
Dt_chr5	Cufflinks	exon	11396930	11397051	.	+	.	gene_id "XLOC_185347"; transcript_id "Novel_002589"; exon_number "4"; gene_type "
Dt_chr5	Cufflinks	exon	11397148	11397219	.	+	.	gene_id "XLOC_185347"; transcript_id "Novel_002589"; exon_number "5"; gene_type "
Dt_chr5	Cufflinks	exon	11397318	11397967	.	+	.	gene_id "XLOC_185347"; transcript_id "Novel_002589"; exon_number "6"; gene_type "
Dt_chr5	Cufflinks	exon	11397041	11397219	.	+	.	gene_id "XLOC_185347"; transcript_id "Novel_002590"; exon_number "1"; gene_type "
Dt_chr5	Cufflinks	exon	11397318	11397457	.	+	.	gene_id "XLOC_185347"; transcript_id "Novel_002590"; exon_number "2"; gene_type "
Dt_chr5	Cufflinks	exon	11398013	11398417	.	+	.	gene_id "XLOC_185347"; transcript_id "Novel_002590"; exon_number "3"; gene_type "
Dt_chr5	Cufflinks	exon	12946632	12947293	.	+	.	gene_id "XLOC_185587"; transcript_id "Novel_002591"; exon_number "1"; gene_type "
Dt_chr5	Cufflinks	exon	12947386	12947456	.	+	.	gene_id "XLOC_185587"; transcript_id "Novel_002591"; exon_number "2"; gene_type "
Dt_chr5	Cufflinks	exon	12948284	12948365	.	+	.	gene_id "XLOC_185587"; transcript_id "Novel_002591"; exon_number "3"; gene_type "
Dt_chr5	Cufflinks	exon	12949785	12949846	.	+	.	gene_id "XLOC_185587"; transcript_id "Novel_002591"; exon_number "4"; gene_type "
Dt_chr5	Cufflinks	exon	12951008	12951872	.	+	.	gene_id "XLOC_185587"; transcript_id "Novel_002591"; exon_number "5"; gene_type "
Dt_chr5	Cufflinks	exon	12946769	12947090	.	+	.	gene_id "XLOC_185587"; transcript_id "Novel_002592"; exon_number "1"; gene_type "
Dt_chr5	Cufflinks	exon	12947163	12947293	.	+	.	gene_id "XLOC_185587"; transcript_id "Novel_002592"; exon_number "2"; gene_type "
Dt_chr5	Cufflinks	exon	12947386	12947456	.	+	.	gene_id "XLOC_185587"; transcript_id "Novel_002592"; exon_number "3"; gene_type "
Dt_chr5	Cufflinks	exon	12948284	12950340	.	+	.	gene_id "XLOC_185587"; transcript_id "Novel_002592"; exon_number "4"; gene_type "
Dt_chr5	Cufflinks	exon	15962609	15962871	.	+	.	gene_id "XLOC_185961"; transcript_id "Novel_002593"; exon_number "1"; gene_type "
Dt_chr5	Cufflinks	exon	15962949	15963136	.	+	.	gene_id "XLOC_185961"; transcript_id "Novel_002593"; exon_number "2"; gene_type "
Dt_chr5	Cufflinks	exon	15964317	15964476	.	+	.	gene_id "XLOC_185961"; transcript_id "Novel_002593"; exon_number "3"; gene_type "
Dt_chr5	Cufflinks	exon	15964552	15964713	.	+	.	gene_id "XLOC_185961"; transcript_id "Novel_002593"; exon_number "4"; gene_type "
Dt_chr5	Cufflinks	exon	15964811	15965116	.	+	.	gene_id "XLOC_185961"; transcript_id "Novel_002593"; exon_number "5"; gene_type "
Dt_chr5	Cufflinks	exon	15965187	15965812	.	+	.	gene_id "XLOC_185961"; transcript_id "Novel_002593"; exon_number "6"; gene_type "
Dt_chr5	Cufflinks	exon	15962731	15962871	.	+	.	gene_id "XLOC_185961"; transcript_id "Novel_002594"; exon_number "1"; gene_type "
Dt_chr5	Cufflinks	exon	15962949	15963597	.	+	.	gene_id "XLOC_185961"; transcript_id "Novel_002594"; exon_number "2"; gene_type "
Dt_chr5	Cufflinks	exon	16141340	16141922	.	+	.	gene_id "XLOC_185982"; transcript_id "Novel_002595"; exon_number "1"; gene_type "
Dt_chr5	Cufflinks	exon	16142137	16142299	.	+	.	gene_id "XLOC_185982"; transcript_id "Novel_002595"; exon_number "2"; gene_type "
Dt_chr5	Cufflinks	exon	16142420	16142566	.	+	.	gene_id "XLOC_185982"; transcript_id "Novel_002595"; exon_number "3"; gene_type "
Dt_chr5	Cufflinks	exon	16142686	16142748	.	+	.	gene_id "XLOC_185982"; transcript_id "Novel_002595"; exon_number "4"; gene_type "
Dt_chr5	Cufflinks	exon	16142830	16142906	.	+	.	gene_id "XLOC_185982"; transcript_id "Novel_002595"; exon_number "5"; gene_type "
Dt_chr5	Cufflinks	exon	16143165	16143270	.	+	.	gene_id "XLOC_185982"; transcript_id "Novel_002595"; exon_number "6"; gene_type "

Dt_chr5	Cufflinks	exon	16143865	16143967	.	+	.	gene_id "XLOC_185982"; transcript_id "Novel_002595"; exon_number "7"; gene_type "
Dt_chr5	Cufflinks	exon	16144051	16144202	.	+	.	gene_id "XLOC_185982"; transcript_id "Novel_002595"; exon_number "8"; gene_type "
Dt_chr5	Cufflinks	exon	16144314	16144379	.	+	.	gene_id "XLOC_185982"; transcript_id "Novel_002595"; exon_number "9"; gene_type "
Dt_chr5	Cufflinks	exon	16144455	16145299	.	+	.	gene_id "XLOC_185982"; transcript_id "Novel_002595"; exon_number "10"; gene_type
Dt_chr5	Cufflinks	exon	16611279	16612985	.	+	.	gene_id "XLOC_186066"; transcript_id "Novel_002596"; exon_number "1"; gene_type "
Dt_chr5	Cufflinks	exon	16613884	16615377	.	+	.	gene_id "XLOC_186066"; transcript_id "Novel_002596"; exon_number "2"; gene_type "
Dt_chr5	Cufflinks	exon	19131976	19132191	.	+	.	gene_id "XLOC_186468"; transcript_id "Novel_002597"; exon_number "1"; gene_type "
Dt_chr5	Cufflinks	exon	19132288	19132808	.	+	.	gene_id "XLOC_186468"; transcript_id "Novel_002597"; exon_number "2"; gene_type "
Dt_chr5	Cufflinks	exon	19715173	19718308	.	+	.	gene_id "XLOC_186505"; transcript_id "Novel_002598"; exon_number "1"; gene_type "
Dt_chr5	Cufflinks	exon	19718444	19720856	.	+	.	gene_id "XLOC_186505"; transcript_id "Novel_002598"; exon_number "2"; gene_type "
Dt_chr5	Cufflinks	exon	20195604	20200011	.	+	.	gene_id "XLOC_186587"; transcript_id "Novel_002599"; exon_number "1"; gene_type "
Dt_chr5	Cufflinks	exon	20200549	20200673	.	+	.	gene_id "XLOC_186587"; transcript_id "Novel_002599"; exon_number "2"; gene_type "
Dt_chr5	Cufflinks	exon	25166486	25168420	.	+	.	gene_id "XLOC_187076"; transcript_id "Novel_002600"; exon_number "1"; gene_type "
Dt_chr5	Cufflinks	exon	25168934	25170012	.	+	.	gene_id "XLOC_187076"; transcript_id "Novel_002600"; exon_number "2"; gene_type "
Dt_chr5	Cufflinks	exon	27265616	27265776	.	+	.	gene_id "XLOC_187264"; transcript_id "Novel_002601"; exon_number "1"; gene_type "
Dt_chr5	Cufflinks	exon	27266392	27266548	.	+	.	gene_id "XLOC_187264"; transcript_id "Novel_002601"; exon_number "2"; gene_type "
Dt_chr5	Cufflinks	exon	27266793	27266890	.	+	.	gene_id "XLOC_187264"; transcript_id "Novel_002601"; exon_number "3"; gene_type "
Dt_chr5	Cufflinks	exon	27702142	27702486	.	+	.	gene_id "XLOC_187291"; transcript_id "Novel_002602"; exon_number "1"; gene_type "
Dt_chr5	Cufflinks	exon	27703045	27703214	.	+	.	gene_id "XLOC_187291"; transcript_id "Novel_002602"; exon_number "2"; gene_type "
Dt_chr5	Cufflinks	exon	27703978	27704104	.	+	.	gene_id "XLOC_187291"; transcript_id "Novel_002602"; exon_number "3"; gene_type "
Dt_chr5	Cufflinks	exon	27705589	27705851	.	+	.	gene_id "XLOC_187291"; transcript_id "Novel_002602"; exon_number "4"; gene_type "
Dt_chr5	Cufflinks	exon	27706065	27707758	.	+	.	gene_id "XLOC_187291"; transcript_id "Novel_002602"; exon_number "5"; gene_type "
Dt_chr5	Cufflinks	exon	27702149	27702486	.	+	.	gene_id "XLOC_187291"; transcript_id "Novel_002603"; exon_number "1"; gene_type "
Dt_chr5	Cufflinks	exon	27703045	27703274	.	+	.	gene_id "XLOC_187291"; transcript_id "Novel_002603"; exon_number "2"; gene_type "
Dt_chr5	Cufflinks	exon	27704043	27704104	.	+	.	gene_id "XLOC_187291"; transcript_id "Novel_002604"; exon_number "1"; gene_type "
Dt_chr5	Cufflinks	exon	27705589	27707635	.	+	.	gene_id "XLOC_187291"; transcript_id "Novel_002604"; exon_number "2"; gene_type "
Dt_chr5	Cufflinks	exon	27709475	27709880	.	+	.	gene_id "XLOC_187291"; transcript_id "Novel_002605"; exon_number "1"; gene_type "
Dt_chr5	Cufflinks	exon	27710283	27710501	.	+	.	gene_id "XLOC_187291"; transcript_id "Novel_002605"; exon_number "2"; gene_type "
Dt_chr5	Cufflinks	exon	27710647	27710975	.	+	.	gene_id "XLOC_187291"; transcript_id "Novel_002605"; exon_number "3"; gene_type "
Dt_chr5	Cufflinks	exon	27711111	27711268	.	+	.	gene_id "XLOC_187291"; transcript_id "Novel_002605"; exon_number "4"; gene_type "
Dt_chr5	Cufflinks	exon	27709960	27710501	.	+	.	gene_id "XLOC_187291"; transcript_id "Novel_002606"; exon_number "1"; gene_type "
Dt_chr5	Cufflinks	exon	27710647	27710975	.	+	.	gene_id "XLOC_187291"; transcript_id "Novel_002606"; exon_number "2"; gene_type "
Dt_chr5	Cufflinks	exon	27711111	27711309	.	+	.	gene_id "XLOC_187291"; transcript_id "Novel_002606"; exon_number "3"; gene_type "
Dt_chr5	Cufflinks	exon	28158020	28158062	.	+	.	gene_id "XLOC_187320"; transcript_id "Novel_002607"; exon_number "1"; gene_type "
Dt_chr5	Cufflinks	exon	28158187	28161799	.	+	.	gene_id "XLOC_187320"; transcript_id "Novel_002607"; exon_number "2"; gene_type "

Dt_chr5	Cufflinks	exon	33976230	33976472	.	+	.	gene_id "XLOC_187665"; transcript_id "Novel_002608"; exon_number "1"; gene_type "
Dt_chr5	Cufflinks	exon	33976589	33976904	.	+	.	gene_id "XLOC_187665"; transcript_id "Novel_002608"; exon_number "2"; gene_type "
Dt_chr5	Cufflinks	exon	34686256	34686671	.	+	.	gene_id "XLOC_187733"; transcript_id "Novel_002609"; exon_number "1"; gene_type "
Dt_chr5	Cufflinks	exon	34686957	34687089	.	+	.	gene_id "XLOC_187733"; transcript_id "Novel_002609"; exon_number "2"; gene_type "
Dt_chr5	Cufflinks	exon	34687190	34687261	.	+	.	gene_id "XLOC_187733"; transcript_id "Novel_002609"; exon_number "3"; gene_type "
Dt_chr5	Cufflinks	exon	34687367	34687432	.	+	.	gene_id "XLOC_187733"; transcript_id "Novel_002609"; exon_number "4"; gene_type "
Dt_chr5	Cufflinks	exon	34687522	34687593	.	+	.	gene_id "XLOC_187733"; transcript_id "Novel_002609"; exon_number "5"; gene_type "
Dt_chr5	Cufflinks	exon	34687695	34687766	.	+	.	gene_id "XLOC_187733"; transcript_id "Novel_002609"; exon_number "6"; gene_type "
Dt_chr5	Cufflinks	exon	34688135	34688206	.	+	.	gene_id "XLOC_187733"; transcript_id "Novel_002609"; exon_number "7"; gene_type "
Dt_chr5	Cufflinks	exon	34688303	34688368	.	+	.	gene_id "XLOC_187733"; transcript_id "Novel_002609"; exon_number "8"; gene_type "
Dt_chr5	Cufflinks	exon	34688447	34688512	.	+	.	gene_id "XLOC_187733"; transcript_id "Novel_002609"; exon_number "9"; gene_type "
Dt_chr5	Cufflinks	exon	34688600	34688880	.	+	.	gene_id "XLOC_187733"; transcript_id "Novel_002609"; exon_number "10"; gene_type
Dt_chr5	Cufflinks	exon	34689356	34689692	.	+	.	gene_id "XLOC_187733"; transcript_id "Novel_002609"; exon_number "11"; gene_type
Dt_chr5	Cufflinks	exon	34686316	34686671	.	+	.	gene_id "XLOC_187733"; transcript_id "Novel_002610"; exon_number "1"; gene_type "
Dt_chr5	Cufflinks	exon	34686957	34687089	.	+	.	gene_id "XLOC_187733"; transcript_id "Novel_002610"; exon_number "2"; gene_type "
Dt_chr5	Cufflinks	exon	34687190	34687261	.	+	.	gene_id "XLOC_187733"; transcript_id "Novel_002610"; exon_number "3"; gene_type "
Dt_chr5	Cufflinks	exon	34687367	34687432	.	+	.	gene_id "XLOC_187733"; transcript_id "Novel_002610"; exon_number "4"; gene_type "
Dt_chr5	Cufflinks	exon	34687522	34687593	.	+	.	gene_id "XLOC_187733"; transcript_id "Novel_002610"; exon_number "5"; gene_type "
Dt_chr5	Cufflinks	exon	34687695	34687766	.	+	.	gene_id "XLOC_187733"; transcript_id "Novel_002610"; exon_number "6"; gene_type "
Dt_chr5	Cufflinks	exon	34688135	34688206	.	+	.	gene_id "XLOC_187733"; transcript_id "Novel_002610"; exon_number "7"; gene_type "
Dt_chr5	Cufflinks	exon	34688303	34688368	.	+	.	gene_id "XLOC_187733"; transcript_id "Novel_002610"; exon_number "8"; gene_type "
Dt_chr5	Cufflinks	exon	34688447	34688512	.	+	.	gene_id "XLOC_187733"; transcript_id "Novel_002610"; exon_number "9"; gene_type "
Dt_chr5	Cufflinks	exon	34688600	34688880	.	+	.	gene_id "XLOC_187733"; transcript_id "Novel_002610"; exon_number "10"; gene_type
Dt_chr5	Cufflinks	exon	34689356	34689609	.	+	.	gene_id "XLOC_187733"; transcript_id "Novel_002610"; exon_number "11"; gene_type
Dt_chr5	Cufflinks	exon	34689740	34690011	.	+	.	gene_id "XLOC_187733"; transcript_id "Novel_002610"; exon_number "12"; gene_type
Dt_chr5	Cufflinks	exon	34690113	34690248	.	+	.	gene_id "XLOC_187733"; transcript_id "Novel_002610"; exon_number "13"; gene_type
Dt_chr5	Cufflinks	exon	34690372	34690511	.	+	.	gene_id "XLOC_187733"; transcript_id "Novel_002610"; exon_number "14"; gene_type
Dt_chr5	Cufflinks	exon	34690628	34690784	.	+	.	gene_id "XLOC_187733"; transcript_id "Novel_002610"; exon_number "15"; gene_type
Dt_chr5	Cufflinks	exon	34690893	34691504	.	+	.	gene_id "XLOC_187733"; transcript_id "Novel_002610"; exon_number "16"; gene_type
Dt_chr5	Cufflinks	exon	35693922	35693959	.	+	.	gene_id "XLOC_187843"; transcript_id "Novel_002611"; exon_number "1"; gene_type "
Dt_chr5	Cufflinks	exon	35698905	35699335	.	+	.	gene_id "XLOC_187843"; transcript_id "Novel_002611"; exon_number "2"; gene_type "
Dt_chr5	Cufflinks	exon	40536500	40537320	.	+	.	gene_id "XLOC_188174"; transcript_id "Novel_002612"; exon_number "1"; gene_type "
Dt_chr5	Cufflinks	exon	40538193	40538700	.	+	.	gene_id "XLOC_188174"; transcript_id "Novel_002612"; exon_number "2"; gene_type "
Dt_chr5	Cufflinks	exon	41498892	41499093	.	+	.	gene_id "XLOC_188283"; transcript_id "Novel_002613"; exon_number "1"; gene_type "
Dt_chr5	Cufflinks	exon	41499452	41499535	.	+	.	gene_id "XLOC_188283"; transcript_id "Novel_002613"; exon_number "2"; gene_type "

Dt_chr5	Cufflinks	exon	41500368	41500433	.	+	.	gene_id "XLOC_188283"; transcript_id "Novel_002613"; exon_number "3"; gene_type "
Dt_chr5	Cufflinks	exon	41501679	41501812	.	+	.	gene_id "XLOC_188283"; transcript_id "Novel_002613"; exon_number "4"; gene_type "
Dt_chr5	Cufflinks	exon	41501952	41504672	.	+	.	gene_id "XLOC_188283"; transcript_id "Novel_002613"; exon_number "5"; gene_type "
Dt_chr5	Cufflinks	exon	41498930	41499093	.	+	.	gene_id "XLOC_188283"; transcript_id "Novel_002614"; exon_number "1"; gene_type "
Dt_chr5	Cufflinks	exon	41499452	41500433	.	+	.	gene_id "XLOC_188283"; transcript_id "Novel_002614"; exon_number "2"; gene_type "
Dt_chr5	Cufflinks	exon	41501679	41501812	.	+	.	gene_id "XLOC_188283"; transcript_id "Novel_002614"; exon_number "3"; gene_type "
Dt_chr5	Cufflinks	exon	41501952	41503543	.	+	.	gene_id "XLOC_188283"; transcript_id "Novel_002614"; exon_number "4"; gene_type "
Dt_chr5	Cufflinks	exon	41498933	41499093	.	+	.	gene_id "XLOC_188283"; transcript_id "Novel_002615"; exon_number "1"; gene_type "
Dt_chr5	Cufflinks	exon	41499452	41500675	.	+	.	gene_id "XLOC_188283"; transcript_id "Novel_002615"; exon_number "2"; gene_type "
Dt_chr5	Cufflinks	exon	44562594	44562912	.	+	.	gene_id "XLOC_188444"; transcript_id "Novel_002616"; exon_number "1"; gene_type "
Dt_chr5	Cufflinks	exon	44563702	44563849	.	+	.	gene_id "XLOC_188444"; transcript_id "Novel_002616"; exon_number "2"; gene_type "
Dt_chr5	Cufflinks	exon	44564433	44564545	.	+	.	gene_id "XLOC_188444"; transcript_id "Novel_002616"; exon_number "3"; gene_type "
Dt_chr5	Cufflinks	exon	44564890	44564966	.	+	.	gene_id "XLOC_188444"; transcript_id "Novel_002616"; exon_number "4"; gene_type "
Dt_chr5	Cufflinks	exon	44565171	44565250	.	+	.	gene_id "XLOC_188444"; transcript_id "Novel_002616"; exon_number "5"; gene_type "
Dt_chr5	Cufflinks	exon	44565744	44565804	.	+	.	gene_id "XLOC_188444"; transcript_id "Novel_002616"; exon_number "6"; gene_type "
Dt_chr5	Cufflinks	exon	44566558	44566625	.	+	.	gene_id "XLOC_188444"; transcript_id "Novel_002616"; exon_number "7"; gene_type "
Dt_chr5	Cufflinks	exon	44566746	44566827	.	+	.	gene_id "XLOC_188444"; transcript_id "Novel_002616"; exon_number "8"; gene_type "
Dt_chr5	Cufflinks	exon	44567402	44567488	.	+	.	gene_id "XLOC_188444"; transcript_id "Novel_002616"; exon_number "9"; gene_type "
Dt_chr5	Cufflinks	exon	44567565	44567642	.	+	.	gene_id "XLOC_188444"; transcript_id "Novel_002616"; exon_number "10"; gene_type
Dt_chr5	Cufflinks	exon	44568027	44568146	.	+	.	gene_id "XLOC_188444"; transcript_id "Novel_002616"; exon_number "11"; gene_type
Dt_chr5	Cufflinks	exon	44568910	44569199	.	+	.	gene_id "XLOC_188444"; transcript_id "Novel_002616"; exon_number "12"; gene_type
Dt_chr5	Cufflinks	exon	47983110	47983810	.	+	.	gene_id "XLOC_188616"; transcript_id "Novel_002617"; exon_number "1"; gene_type "
Dt_chr5	Cufflinks	exon	47983901	47984038	.	+	.	gene_id "XLOC_188616"; transcript_id "Novel_002617"; exon_number "2"; gene_type "
Dt_chr5	Cufflinks	exon	49251854	49251921	.	+	.	gene_id "XLOC_188733"; transcript_id "Novel_002618"; exon_number "1"; gene_type "
Dt_chr5	Cufflinks	exon	49252394	49255863	.	+	.	gene_id "XLOC_188733"; transcript_id "Novel_002618"; exon_number "2"; gene_type "
Dt_chr5	Cufflinks	exon	49256007	49256072	.	+	.	gene_id "XLOC_188733"; transcript_id "Novel_002618"; exon_number "3"; gene_type "
Dt_chr5	Cufflinks	exon	49256192	49256290	.	+	.	gene_id "XLOC_188733"; transcript_id "Novel_002618"; exon_number "4"; gene_type "
Dt_chr5	Cufflinks	exon	49256400	49257962	.	+	.	gene_id "XLOC_188733"; transcript_id "Novel_002618"; exon_number "5"; gene_type "
Dt_chr5	Cufflinks	exon	49251856	49251921	.	+	.	gene_id "XLOC_188733"; transcript_id "Novel_002619"; exon_number "1"; gene_type "
Dt_chr5	Cufflinks	exon	49252394	49255863	.	+	.	gene_id "XLOC_188733"; transcript_id "Novel_002619"; exon_number "2"; gene_type "
Dt_chr5	Cufflinks	exon	49256007	49256072	.	+	.	gene_id "XLOC_188733"; transcript_id "Novel_002619"; exon_number "3"; gene_type "
Dt_chr5	Cufflinks	exon	49256192	49256290	.	+	.	gene_id "XLOC_188733"; transcript_id "Novel_002619"; exon_number "4"; gene_type "
Dt_chr5	Cufflinks	exon	49256400	49256511	.	+	.	gene_id "XLOC_188733"; transcript_id "Novel_002619"; exon_number "5"; gene_type "
Dt_chr5	Cufflinks	exon	49259362	49260157	.	+	.	gene_id "XLOC_188733"; transcript_id "Novel_002619"; exon_number "6"; gene_type "
Dt_chr5	Cufflinks	exon	49251929	49255863	.	+	.	gene_id "XLOC_188733"; transcript_id "Novel_002620"; exon_number "1"; gene_type "

Dt_chr5	Cufflinks	exon	49256007	49256072	.	+	.	gene_id "XLOC_188733"; transcript_id "Novel_002620"; exon_number "2"; gene_type "
Dt_chr5	Cufflinks	exon	49256192	49256290	.	+	.	gene_id "XLOC_188733"; transcript_id "Novel_002620"; exon_number "3"; gene_type "
Dt_chr5	Cufflinks	exon	49256400	49257962	.	+	.	gene_id "XLOC_188733"; transcript_id "Novel_002620"; exon_number "4"; gene_type "
Dt_chr5	Cufflinks	exon	49932967	49933104	.	+	.	gene_id "XLOC_188802"; transcript_id "Novel_002621"; exon_number "1"; gene_type "
Dt_chr5	Cufflinks	exon	49933632	49934928	.	+	.	gene_id "XLOC_188802"; transcript_id "Novel_002621"; exon_number "2"; gene_type "
Dt_chr5	Cufflinks	exon	51780199	51780305	.	+	.	gene_id "XLOC_189014"; transcript_id "Novel_002622"; exon_number "1"; gene_type "
Dt_chr5	Cufflinks	exon	51780468	51780841	.	+	.	gene_id "XLOC_189014"; transcript_id "Novel_002622"; exon_number "2"; gene_type "
Dt_chr5	Cufflinks	exon	51781046	51783164	.	+	.	gene_id "XLOC_189014"; transcript_id "Novel_002622"; exon_number "3"; gene_type "
Dt_chr5	Cufflinks	exon	53339900	53340015	.	+	.	gene_id "XLOC_189197"; transcript_id "Novel_002623"; exon_number "1"; gene_type "
Dt_chr5	Cufflinks	exon	53340143	53340237	.	+	.	gene_id "XLOC_189197"; transcript_id "Novel_002623"; exon_number "2"; gene_type "
Dt_chr5	Cufflinks	exon	53341888	53343495	.	+	.	gene_id "XLOC_189197"; transcript_id "Novel_002623"; exon_number "3"; gene_type "
Dt_chr5	Cufflinks	exon	53343586	53344769	.	+	.	gene_id "XLOC_189197"; transcript_id "Novel_002623"; exon_number "4"; gene_type "
Dt_chr5	Cufflinks	exon	53339900	53340015	.	+	.	gene_id "XLOC_189197"; transcript_id "Novel_002624"; exon_number "1"; gene_type "
Dt_chr5	Cufflinks	exon	53340143	53340237	.	+	.	gene_id "XLOC_189197"; transcript_id "Novel_002624"; exon_number "2"; gene_type "
Dt_chr5	Cufflinks	exon	53341888	53342976	.	+	.	gene_id "XLOC_189197"; transcript_id "Novel_002624"; exon_number "3"; gene_type "
Dt_chr5	Cufflinks	exon	53343052	53343495	.	+	.	gene_id "XLOC_189197"; transcript_id "Novel_002624"; exon_number "4"; gene_type "
Dt_chr5	Cufflinks	exon	53343586	53344769	.	+	.	gene_id "XLOC_189197"; transcript_id "Novel_002624"; exon_number "5"; gene_type "
Dt_chr5	Cufflinks	exon	57782392	57782949	.	+	.	gene_id "XLOC_189377"; transcript_id "Novel_002625"; exon_number "1"; gene_type "
Dt_chr5	Cufflinks	exon	57784008	57784251	.	+	.	gene_id "XLOC_189377"; transcript_id "Novel_002625"; exon_number "2"; gene_type "
Dt_chr5	Cufflinks	exon	4781005	4781301	.	-	.	gene_id "XLOC_190054"; transcript_id "Novel_002626"; exon_number "1"; gene_type "
Dt_chr5	Cufflinks	exon	4781405	4784153	.	-	.	gene_id "XLOC_190054"; transcript_id "Novel_002626"; exon_number "2"; gene_type "
Dt_chr5	Cufflinks	exon	5034863	5035112	.	-	.	gene_id "XLOC_190073"; transcript_id "Novel_002627"; exon_number "1"; gene_type "
Dt_chr5	Cufflinks	exon	5035854	5035901	.	-	.	gene_id "XLOC_190073"; transcript_id "Novel_002627"; exon_number "2"; gene_type "
Dt_chr5	Cufflinks	exon	5036032	5036097	.	-	.	gene_id "XLOC_190073"; transcript_id "Novel_002627"; exon_number "3"; gene_type "
Dt_chr5	Cufflinks	exon	5036868	5037069	.	-	.	gene_id "XLOC_190073"; transcript_id "Novel_002627"; exon_number "4"; gene_type "
Dt_chr5	Cufflinks	exon	5034863	5035112	.	-	.	gene_id "XLOC_190073"; transcript_id "Novel_002628"; exon_number "1"; gene_type "
Dt_chr5	Cufflinks	exon	5035854	5035901	.	-	.	gene_id "XLOC_190073"; transcript_id "Novel_002628"; exon_number "2"; gene_type "
Dt_chr5	Cufflinks	exon	5036025	5036097	.	-	.	gene_id "XLOC_190073"; transcript_id "Novel_002628"; exon_number "3"; gene_type "
Dt_chr5	Cufflinks	exon	5036868	5037069	.	-	.	gene_id "XLOC_190073"; transcript_id "Novel_002628"; exon_number "4"; gene_type "
Dt_chr5	Cufflinks	exon	5174849	5175847	.	-	.	gene_id "XLOC_190080"; transcript_id "Novel_002629"; exon_number "1"; gene_type "
Dt_chr5	Cufflinks	exon	5176038	5176263	.	-	.	gene_id "XLOC_190080"; transcript_id "Novel_002629"; exon_number "2"; gene_type "
Dt_chr5	Cufflinks	exon	5176340	5176794	.	-	.	gene_id "XLOC_190080"; transcript_id "Novel_002629"; exon_number "3"; gene_type "
Dt_chr5	Cufflinks	exon	5178197	5178516	.	-	.	gene_id "XLOC_190080"; transcript_id "Novel_002629"; exon_number "4"; gene_type "
Dt_chr5	Cufflinks	exon	5935657	5936005	.	-	.	gene_id "XLOC_190125"; transcript_id "Novel_002630"; exon_number "1"; gene_type "
Dt_chr5	Cufflinks	exon	6620831	6622678	.	-	.	gene_id "XLOC_190188"; transcript_id "Novel_002631"; exon_number "1"; gene_type "

Dt_chr5	Cufflinks	exon	6623124	6623149	.	-	.	gene_id "XLOC_190188"; transcript_id "Novel_002631"; exon_number "2"; gene_type "
Dt_chr5	Cufflinks	exon	6623558	6623887	.	-	.	gene_id "XLOC_190188"; transcript_id "Novel_002631"; exon_number "3"; gene_type "
Dt_chr5	Cufflinks	exon	6620831	6622678	.	-	.	gene_id "XLOC_190188"; transcript_id "Novel_002632"; exon_number "1"; gene_type "
Dt_chr5	Cufflinks	exon	6623558	6623887	.	-	.	gene_id "XLOC_190188"; transcript_id "Novel_002632"; exon_number "2"; gene_type "
Dt_chr5	Cufflinks	exon	7248813	7250263	.	-	.	gene_id "XLOC_190308"; transcript_id "Novel_002633"; exon_number "1"; gene_type "
Dt_chr5	Cufflinks	exon	7250366	7250436	.	-	.	gene_id "XLOC_190308"; transcript_id "Novel_002633"; exon_number "2"; gene_type "
Dt_chr5	Cufflinks	exon	7250682	7250760	.	-	.	gene_id "XLOC_190308"; transcript_id "Novel_002633"; exon_number "3"; gene_type "
Dt_chr5	Cufflinks	exon	7250838	7250879	.	-	.	gene_id "XLOC_190308"; transcript_id "Novel_002633"; exon_number "4"; gene_type "
Dt_chr5	Cufflinks	exon	7250972	7251025	.	-	.	gene_id "XLOC_190308"; transcript_id "Novel_002633"; exon_number "5"; gene_type "
Dt_chr5	Cufflinks	exon	7251129	7251161	.	-	.	gene_id "XLOC_190308"; transcript_id "Novel_002633"; exon_number "6"; gene_type "
Dt_chr5	Cufflinks	exon	7251264	7251344	.	-	.	gene_id "XLOC_190308"; transcript_id "Novel_002633"; exon_number "7"; gene_type "
Dt_chr5	Cufflinks	exon	7251416	7251502	.	-	.	gene_id "XLOC_190308"; transcript_id "Novel_002633"; exon_number "8"; gene_type "
Dt_chr5	Cufflinks	exon	7251594	7251669	.	-	.	gene_id "XLOC_190308"; transcript_id "Novel_002633"; exon_number "9"; gene_type "
Dt_chr5	Cufflinks	exon	7251757	7251965	.	-	.	gene_id "XLOC_190308"; transcript_id "Novel_002633"; exon_number "10"; gene_type
Dt_chr5	Cufflinks	exon	7252040	7252162	.	-	.	gene_id "XLOC_190308"; transcript_id "Novel_002633"; exon_number "11"; gene_type
Dt_chr5	Cufflinks	exon	7252288	7252400	.	-	.	gene_id "XLOC_190308"; transcript_id "Novel_002633"; exon_number "12"; gene_type
Dt_chr5	Cufflinks	exon	7253357	7253468	.	-	.	gene_id "XLOC_190308"; transcript_id "Novel_002633"; exon_number "13"; gene_type
Dt_chr5	Cufflinks	exon	7257911	7258150	.	-	.	gene_id "XLOC_190308"; transcript_id "Novel_002633"; exon_number "14"; gene_type
Dt_chr5	Cufflinks	exon	7248923	7250263	.	-	.	gene_id "XLOC_190308"; transcript_id "Novel_002634"; exon_number "1"; gene_type "
Dt_chr5	Cufflinks	exon	7250366	7250436	.	-	.	gene_id "XLOC_190308"; transcript_id "Novel_002634"; exon_number "2"; gene_type "
Dt_chr5	Cufflinks	exon	7250682	7250760	.	-	.	gene_id "XLOC_190308"; transcript_id "Novel_002634"; exon_number "3"; gene_type "
Dt_chr5	Cufflinks	exon	7250838	7250879	.	-	.	gene_id "XLOC_190308"; transcript_id "Novel_002634"; exon_number "4"; gene_type "
Dt_chr5	Cufflinks	exon	7250972	7251025	.	-	.	gene_id "XLOC_190308"; transcript_id "Novel_002634"; exon_number "5"; gene_type "
Dt_chr5	Cufflinks	exon	7251129	7251161	.	-	.	gene_id "XLOC_190308"; transcript_id "Novel_002634"; exon_number "6"; gene_type "
Dt_chr5	Cufflinks	exon	7251264	7251344	.	-	.	gene_id "XLOC_190308"; transcript_id "Novel_002634"; exon_number "7"; gene_type "
Dt_chr5	Cufflinks	exon	7251416	7251669	.	-	.	gene_id "XLOC_190308"; transcript_id "Novel_002634"; exon_number "8"; gene_type "
Dt_chr5	Cufflinks	exon	7251757	7252162	.	-	.	gene_id "XLOC_190308"; transcript_id "Novel_002634"; exon_number "9"; gene_type "
Dt_chr5	Cufflinks	exon	7252288	7252400	.	-	.	gene_id "XLOC_190308"; transcript_id "Novel_002634"; exon_number "10"; gene_type
Dt_chr5	Cufflinks	exon	7253357	7253468	.	-	.	gene_id "XLOC_190308"; transcript_id "Novel_002634"; exon_number "11"; gene_type
Dt_chr5	Cufflinks	exon	7257911	7258240	.	-	.	gene_id "XLOC_190308"; transcript_id "Novel_002634"; exon_number "12"; gene_type
Dt_chr5	Cufflinks	exon	12420152	12420516	.	-	.	gene_id "XLOC_191151"; transcript_id "Novel_002635"; exon_number "1"; gene_type "
Dt_chr5	Cufflinks	exon	12420615	12420726	.	-	.	gene_id "XLOC_191151"; transcript_id "Novel_002635"; exon_number "2"; gene_type "
Dt_chr5	Cufflinks	exon	12420908	12421171	.	-	.	gene_id "XLOC_191151"; transcript_id "Novel_002635"; exon_number "3"; gene_type "
Dt_chr5	Cufflinks	exon	12421701	12421803	.	-	.	gene_id "XLOC_191151"; transcript_id "Novel_002635"; exon_number "4"; gene_type "
Dt_chr5	Cufflinks	exon	12422390	12422468	.	-	.	gene_id "XLOC_191151"; transcript_id "Novel_002635"; exon_number "5"; gene_type "

Dt_chr5	Cufflinks	exon	12422757	12422892	.	-	.	gene_id "XLOC_191151"; transcript_id "Novel_002635"; exon_number "6"; gene_type "
Dt_chr5	Cufflinks	exon	12479142	12481606	.	-	.	gene_id "XLOC_191170"; transcript_id "Novel_002636"; exon_number "1"; gene_type "
Dt_chr5	Cufflinks	exon	12481724	12481858	.	-	.	gene_id "XLOC_191170"; transcript_id "Novel_002636"; exon_number "2"; gene_type "
Dt_chr5	Cufflinks	exon	12483547	12483998	.	-	.	gene_id "XLOC_191170"; transcript_id "Novel_002636"; exon_number "3"; gene_type "
Dt_chr5	Cufflinks	exon	12479142	12481858	.	-	.	gene_id "XLOC_191170"; transcript_id "Novel_002637"; exon_number "1"; gene_type "
Dt_chr5	Cufflinks	exon	12483547	12483998	.	-	.	gene_id "XLOC_191170"; transcript_id "Novel_002637"; exon_number "2"; gene_type "
Dt_chr5	Cufflinks	exon	12479563	12481606	.	-	.	gene_id "XLOC_191170"; transcript_id "Novel_002638"; exon_number "1"; gene_type "
Dt_chr5	Cufflinks	exon	12481724	12481858	.	-	.	gene_id "XLOC_191170"; transcript_id "Novel_002638"; exon_number "2"; gene_type "
Dt_chr5	Cufflinks	exon	12483067	12483232	.	-	.	gene_id "XLOC_191170"; transcript_id "Novel_002638"; exon_number "3"; gene_type "
Dt_chr5	Cufflinks	exon	12483547	12483793	.	-	.	gene_id "XLOC_191170"; transcript_id "Novel_002638"; exon_number "4"; gene_type "
Dt_chr5	Cufflinks	exon	12501164	12502087	.	-	.	gene_id "XLOC_191173"; transcript_id "Novel_002639"; exon_number "1"; gene_type "
Dt_chr5	Cufflinks	exon	12503120	12503238	.	-	.	gene_id "XLOC_191173"; transcript_id "Novel_002639"; exon_number "2"; gene_type "
Dt_chr5	Cufflinks	exon	12504056	12504631	.	-	.	gene_id "XLOC_191173"; transcript_id "Novel_002639"; exon_number "3"; gene_type "
Dt_chr5	Cufflinks	exon	13022261	13022379	.	-	.	gene_id "XLOC_191276"; transcript_id "Novel_002640"; exon_number "1"; gene_type "
Dt_chr5	Cufflinks	exon	13022518	13022742	.	-	.	gene_id "XLOC_191276"; transcript_id "Novel_002640"; exon_number "2"; gene_type "
Dt_chr5	Cufflinks	exon	13476689	13476696	.	-	.	gene_id "XLOC_191370"; transcript_id "Novel_002641"; exon_number "1"; gene_type "
Dt_chr5	Cufflinks	exon	13476786	13477193	.	-	.	gene_id "XLOC_191370"; transcript_id "Novel_002641"; exon_number "2"; gene_type "
Dt_chr5	Cufflinks	exon	13555405	13555888	.	-	.	gene_id "XLOC_191381"; transcript_id "Novel_002642"; exon_number "1"; gene_type "
Dt_chr5	Cufflinks	exon	13555985	13556050	.	-	.	gene_id "XLOC_191381"; transcript_id "Novel_002642"; exon_number "2"; gene_type "
Dt_chr5	Cufflinks	exon	13556144	13558132	.	-	.	gene_id "XLOC_191381"; transcript_id "Novel_002642"; exon_number "3"; gene_type "
Dt_chr5	Cufflinks	exon	13558681	13559045	.	-	.	gene_id "XLOC_191381"; transcript_id "Novel_002642"; exon_number "4"; gene_type "
Dt_chr5	Cufflinks	exon	13555405	13555899	.	-	.	gene_id "XLOC_191381"; transcript_id "Novel_002643"; exon_number "1"; gene_type "
Dt_chr5	Cufflinks	exon	13555985	13556050	.	-	.	gene_id "XLOC_191381"; transcript_id "Novel_002643"; exon_number "2"; gene_type "
Dt_chr5	Cufflinks	exon	13556144	13558132	.	-	.	gene_id "XLOC_191381"; transcript_id "Novel_002643"; exon_number "3"; gene_type "
Dt_chr5	Cufflinks	exon	13558681	13559045	.	-	.	gene_id "XLOC_191381"; transcript_id "Novel_002643"; exon_number "4"; gene_type "
Dt_chr5	Cufflinks	exon	14757027	14757314	.	-	.	gene_id "XLOC_191582"; transcript_id "Novel_002644"; exon_number "1"; gene_type "
Dt_chr5	Cufflinks	exon	14757404	14758078	.	-	.	gene_id "XLOC_191582"; transcript_id "Novel_002644"; exon_number "2"; gene_type "
Dt_chr5	Cufflinks	exon	15978656	15978799	.	-	.	gene_id "XLOC_191674"; transcript_id "Novel_002645"; exon_number "1"; gene_type "
Dt_chr5	Cufflinks	exon	15978913	15978950	.	-	.	gene_id "XLOC_191674"; transcript_id "Novel_002645"; exon_number "2"; gene_type "
Dt_chr5	Cufflinks	exon	15979190	15979265	.	-	.	gene_id "XLOC_191674"; transcript_id "Novel_002645"; exon_number "3"; gene_type "
Dt_chr5	Cufflinks	exon	15979664	15979768	.	-	.	gene_id "XLOC_191674"; transcript_id "Novel_002645"; exon_number "4"; gene_type "
Dt_chr5	Cufflinks	exon	15980032	15980084	.	-	.	gene_id "XLOC_191674"; transcript_id "Novel_002645"; exon_number "5"; gene_type "
Dt_chr5	Cufflinks	exon	15980160	15980893	.	-	.	gene_id "XLOC_191674"; transcript_id "Novel_002645"; exon_number "6"; gene_type "
Dt_chr5	Cufflinks	exon	15981628	15982014	.	-	.	gene_id "XLOC_191674"; transcript_id "Novel_002645"; exon_number "7"; gene_type "
Dt_chr5	Cufflinks	exon	17361327	17361768	.	-	.	gene_id "XLOC_191908"; transcript_id "Novel_002646"; exon_number "1"; gene_type "

Dt_chr5	Cufflinks	exon	17362852	17362929	.	-	.	gene_id "XLOC_191908"; transcript_id "Novel_002646"; exon_number "2"; gene_type "
Dt_chr5	Cufflinks	exon	17364098	17364155	.	-	.	gene_id "XLOC_191908"; transcript_id "Novel_002646"; exon_number "3"; gene_type "
Dt_chr5	Cufflinks	exon	17364304	17364422	.	-	.	gene_id "XLOC_191908"; transcript_id "Novel_002646"; exon_number "4"; gene_type "
Dt_chr5	Cufflinks	exon	17364533	17364786	.	-	.	gene_id "XLOC_191908"; transcript_id "Novel_002646"; exon_number "5"; gene_type "
Dt_chr5	Cufflinks	exon	21769210	21769720	.	-	.	gene_id "XLOC_192382"; transcript_id "Novel_002647"; exon_number "1"; gene_type "
Dt_chr5	Cufflinks	exon	21770206	21770370	.	-	.	gene_id "XLOC_192382"; transcript_id "Novel_002647"; exon_number "2"; gene_type "
Dt_chr5	Cufflinks	exon	24818219	24818414	.	-	.	gene_id "XLOC_192600"; transcript_id "Novel_002648"; exon_number "1"; gene_type "
Dt_chr5	Cufflinks	exon	24818692	24818763	.	-	.	gene_id "XLOC_192600"; transcript_id "Novel_002648"; exon_number "2"; gene_type "
Dt_chr5	Cufflinks	exon	24818878	24818985	.	-	.	gene_id "XLOC_192600"; transcript_id "Novel_002648"; exon_number "3"; gene_type "
Dt_chr5	Cufflinks	exon	24819264	24819352	.	-	.	gene_id "XLOC_192600"; transcript_id "Novel_002648"; exon_number "4"; gene_type "
Dt_chr5	Cufflinks	exon	24819912	24820356	.	-	.	gene_id "XLOC_192600"; transcript_id "Novel_002648"; exon_number "5"; gene_type "
Dt_chr5	Cufflinks	exon	25860562	25860640	.	-	.	gene_id "XLOC_192712"; transcript_id "Novel_002649"; exon_number "1"; gene_type "
Dt_chr5	Cufflinks	exon	25861131	25861280	.	-	.	gene_id "XLOC_192712"; transcript_id "Novel_002649"; exon_number "2"; gene_type "
Dt_chr5	Cufflinks	exon	28400024	28400296	.	-	.	gene_id "XLOC_192887"; transcript_id "Novel_002650"; exon_number "1"; gene_type "
Dt_chr5	Cufflinks	exon	28404617	28404829	.	-	.	gene_id "XLOC_192887"; transcript_id "Novel_002650"; exon_number "2"; gene_type "
Dt_chr5	Cufflinks	exon	28404907	28404978	.	-	.	gene_id "XLOC_192887"; transcript_id "Novel_002650"; exon_number "3"; gene_type "
Dt_chr5	Cufflinks	exon	28405060	28405321	.	-	.	gene_id "XLOC_192887"; transcript_id "Novel_002650"; exon_number "4"; gene_type "
Dt_chr5	Cufflinks	exon	28404572	28404829	.	-	.	gene_id "XLOC_192887"; transcript_id "Novel_002651"; exon_number "1"; gene_type "
Dt_chr5	Cufflinks	exon	28404907	28404978	.	-	.	gene_id "XLOC_192887"; transcript_id "Novel_002651"; exon_number "2"; gene_type "
Dt_chr5	Cufflinks	exon	28405060	28405317	.	-	.	gene_id "XLOC_192887"; transcript_id "Novel_002651"; exon_number "3"; gene_type "
Dt_chr5	Cufflinks	exon	30767249	30767470	.	-	.	gene_id "XLOC_193002"; transcript_id "Novel_002652"; exon_number "1"; gene_type "
Dt_chr5	Cufflinks	exon	30767630	30767681	.	-	.	gene_id "XLOC_193002"; transcript_id "Novel_002652"; exon_number "2"; gene_type "
Dt_chr5	Cufflinks	exon	30768715	30769870	.	-	.	gene_id "XLOC_193002"; transcript_id "Novel_002652"; exon_number "3"; gene_type "
Dt_chr5	Cufflinks	exon	30770365	30770472	.	-	.	gene_id "XLOC_193002"; transcript_id "Novel_002652"; exon_number "4"; gene_type "
Dt_chr5	Cufflinks	exon	30770566	30770683	.	-	.	gene_id "XLOC_193002"; transcript_id "Novel_002652"; exon_number "5"; gene_type "
Dt_chr5	Cufflinks	exon	31664290	31664332	.	-	.	gene_id "XLOC_193072"; transcript_id "Novel_002653"; exon_number "1"; gene_type "
Dt_chr5	Cufflinks	exon	31664508	31664682	.	-	.	gene_id "XLOC_193072"; transcript_id "Novel_002653"; exon_number "2"; gene_type "
Dt_chr5	Cufflinks	exon	35206908	35208015	.	-	.	gene_id "XLOC_193374"; transcript_id "Novel_002654"; exon_number "1"; gene_type "
Dt_chr5	Cufflinks	exon	35208138	35209462	.	-	.	gene_id "XLOC_193374"; transcript_id "Novel_002654"; exon_number "2"; gene_type "
Dt_chr5	Cufflinks	exon	38347282	38347934	.	-	.	gene_id "XLOC_193601"; transcript_id "Novel_002655"; exon_number "1"; gene_type "
Dt_chr5	Cufflinks	exon	38349032	38349055	.	-	.	gene_id "XLOC_193601"; transcript_id "Novel_002655"; exon_number "2"; gene_type "
Dt_chr5	Cufflinks	exon	38616293	38619318	.	-	.	gene_id "XLOC_193617"; transcript_id "Novel_002656"; exon_number "1"; gene_type "
Dt_chr5	Cufflinks	exon	38620189	38620448	.	-	.	gene_id "XLOC_193617"; transcript_id "Novel_002656"; exon_number "2"; gene_type "
Dt_chr5	Cufflinks	exon	39863678	39864036	.	-	.	gene_id "XLOC_193711"; transcript_id "Novel_002657"; exon_number "1"; gene_type "
Dt_chr5	Cufflinks	exon	39864150	39864224	.	-	.	gene_id "XLOC_193711"; transcript_id "Novel_002657"; exon_number "2"; gene_type "

Dt_chr5	Cufflinks	exon	39864491	39864882	.	-	.	gene_id "XLOC_193711"; transcript_id "Novel_002657"; exon_number "3"; gene_type "
Dt_chr5	Cufflinks	exon	39865337	39865472	.	-	.	gene_id "XLOC_193711"; transcript_id "Novel_002657"; exon_number "4"; gene_type "
Dt_chr5	Cufflinks	exon	39865669	39865782	.	-	.	gene_id "XLOC_193711"; transcript_id "Novel_002657"; exon_number "5"; gene_type "
Dt_chr5	Cufflinks	exon	39865864	39865929	.	-	.	gene_id "XLOC_193711"; transcript_id "Novel_002657"; exon_number "6"; gene_type "
Dt_chr5	Cufflinks	exon	39866034	39866133	.	-	.	gene_id "XLOC_193711"; transcript_id "Novel_002657"; exon_number "7"; gene_type "
Dt_chr5	Cufflinks	exon	39866222	39866271	.	-	.	gene_id "XLOC_193711"; transcript_id "Novel_002657"; exon_number "8"; gene_type "
Dt_chr5	Cufflinks	exon	39866372	39866473	.	-	.	gene_id "XLOC_193711"; transcript_id "Novel_002657"; exon_number "9"; gene_type "
Dt_chr5	Cufflinks	exon	39866604	39866681	.	-	.	gene_id "XLOC_193711"; transcript_id "Novel_002657"; exon_number "10"; gene_type
Dt_chr5	Cufflinks	exon	39866799	39867065	.	-	.	gene_id "XLOC_193711"; transcript_id "Novel_002657"; exon_number "11"; gene_type
Dt_chr5	Cufflinks	exon	39867267	39867366	.	-	.	gene_id "XLOC_193711"; transcript_id "Novel_002657"; exon_number "12"; gene_type
Dt_chr5	Cufflinks	exon	39867469	39868128	.	-	.	gene_id "XLOC_193711"; transcript_id "Novel_002657"; exon_number "13"; gene_type
Dt_chr5	Cufflinks	exon	39863803	39864036	.	-	.	gene_id "XLOC_193711"; transcript_id "Novel_002658"; exon_number "1"; gene_type "
Dt_chr5	Cufflinks	exon	39864150	39864224	.	-	.	gene_id "XLOC_193711"; transcript_id "Novel_002658"; exon_number "2"; gene_type "
Dt_chr5	Cufflinks	exon	39864491	39864882	.	-	.	gene_id "XLOC_193711"; transcript_id "Novel_002658"; exon_number "3"; gene_type "
Dt_chr5	Cufflinks	exon	39865337	39865472	.	-	.	gene_id "XLOC_193711"; transcript_id "Novel_002658"; exon_number "4"; gene_type "
Dt_chr5	Cufflinks	exon	39865669	39865782	.	-	.	gene_id "XLOC_193711"; transcript_id "Novel_002658"; exon_number "5"; gene_type "
Dt_chr5	Cufflinks	exon	39865864	39865929	.	-	.	gene_id "XLOC_193711"; transcript_id "Novel_002658"; exon_number "6"; gene_type "
Dt_chr5	Cufflinks	exon	39866034	39866133	.	-	.	gene_id "XLOC_193711"; transcript_id "Novel_002658"; exon_number "7"; gene_type "
Dt_chr5	Cufflinks	exon	39866222	39866271	.	-	.	gene_id "XLOC_193711"; transcript_id "Novel_002658"; exon_number "8"; gene_type "
Dt_chr5	Cufflinks	exon	39866372	39866473	.	-	.	gene_id "XLOC_193711"; transcript_id "Novel_002658"; exon_number "9"; gene_type "
Dt_chr5	Cufflinks	exon	39866604	39866681	.	-	.	gene_id "XLOC_193711"; transcript_id "Novel_002658"; exon_number "10"; gene_type
Dt_chr5	Cufflinks	exon	39866799	39867065	.	-	.	gene_id "XLOC_193711"; transcript_id "Novel_002658"; exon_number "11"; gene_type
Dt_chr5	Cufflinks	exon	39867267	39867366	.	-	.	gene_id "XLOC_193711"; transcript_id "Novel_002658"; exon_number "12"; gene_type
Dt_chr5	Cufflinks	exon	39867469	39867757	.	-	.	gene_id "XLOC_193711"; transcript_id "Novel_002658"; exon_number "13"; gene_type
Dt_chr5	Cufflinks	exon	39868027	39868507	.	-	.	gene_id "XLOC_193711"; transcript_id "Novel_002658"; exon_number "14"; gene_type
Dt_chr5	Cufflinks	exon	39863803	39864036	.	-	.	gene_id "XLOC_193711"; transcript_id "Novel_002659"; exon_number "1"; gene_type "
Dt_chr5	Cufflinks	exon	39864150	39864224	.	-	.	gene_id "XLOC_193711"; transcript_id "Novel_002659"; exon_number "2"; gene_type "
Dt_chr5	Cufflinks	exon	39864491	39864882	.	-	.	gene_id "XLOC_193711"; transcript_id "Novel_002659"; exon_number "3"; gene_type "
Dt_chr5	Cufflinks	exon	39865337	39865472	.	-	.	gene_id "XLOC_193711"; transcript_id "Novel_002659"; exon_number "4"; gene_type "
Dt_chr5	Cufflinks	exon	39865669	39865782	.	-	.	gene_id "XLOC_193711"; transcript_id "Novel_002659"; exon_number "5"; gene_type "
Dt_chr5	Cufflinks	exon	39865864	39865929	.	-	.	gene_id "XLOC_193711"; transcript_id "Novel_002659"; exon_number "6"; gene_type "
Dt_chr5	Cufflinks	exon	39866034	39866120	.	-	.	gene_id "XLOC_193711"; transcript_id "Novel_002659"; exon_number "7"; gene_type "
Dt_chr5	Cufflinks	exon	39866222	39866271	.	-	.	gene_id "XLOC_193711"; transcript_id "Novel_002659"; exon_number "8"; gene_type "
Dt_chr5	Cufflinks	exon	39866372	39866473	.	-	.	gene_id "XLOC_193711"; transcript_id "Novel_002659"; exon_number "9"; gene_type "
Dt_chr5	Cufflinks	exon	39866604	39866681	.	-	.	gene_id "XLOC_193711"; transcript_id "Novel_002659"; exon_number "10"; gene_type

Dt_chr5	Cufflinks	exon	39866799	39867065	.	-	.	gene_id "XLOC_193711"; transcript_id "Novel_002659"; exon_number "11"; gene_type
Dt_chr5	Cufflinks	exon	39867267	39867366	.	-	.	gene_id "XLOC_193711"; transcript_id "Novel_002659"; exon_number "12"; gene_type
Dt_chr5	Cufflinks	exon	39867469	39867757	.	-	.	gene_id "XLOC_193711"; transcript_id "Novel_002659"; exon_number "13"; gene_type
Dt_chr5	Cufflinks	exon	39868027	39868507	.	-	.	gene_id "XLOC_193711"; transcript_id "Novel_002659"; exon_number "14"; gene_type
Dt_chr5	Cufflinks	exon	40242634	40243299	.	-	.	gene_id "XLOC_193718"; transcript_id "Novel_002660"; exon_number "1"; gene_type "
Dt_chr5	Cufflinks	exon	40243416	40243572	.	-	.	gene_id "XLOC_193718"; transcript_id "Novel_002660"; exon_number "2"; gene_type "
Dt_chr5	Cufflinks	exon	40243719	40243791	.	-	.	gene_id "XLOC_193718"; transcript_id "Novel_002660"; exon_number "3"; gene_type "
Dt_chr5	Cufflinks	exon	40244395	40244485	.	-	.	gene_id "XLOC_193718"; transcript_id "Novel_002660"; exon_number "4"; gene_type "
Dt_chr5	Cufflinks	exon	40244568	40244883	.	-	.	gene_id "XLOC_193718"; transcript_id "Novel_002660"; exon_number "5"; gene_type "
Dt_chr5	Cufflinks	exon	41301265	41301736	.	-	.	gene_id "XLOC_193805"; transcript_id "Novel_002661"; exon_number "1"; gene_type "
Dt_chr5	Cufflinks	exon	41301828	41301926	.	-	.	gene_id "XLOC_193805"; transcript_id "Novel_002661"; exon_number "2"; gene_type "
Dt_chr5	Cufflinks	exon	41302060	41302164	.	-	.	gene_id "XLOC_193805"; transcript_id "Novel_002661"; exon_number "3"; gene_type "
Dt_chr5	Cufflinks	exon	41302255	41302347	.	-	.	gene_id "XLOC_193805"; transcript_id "Novel_002661"; exon_number "4"; gene_type "
Dt_chr5	Cufflinks	exon	41302443	41302535	.	-	.	gene_id "XLOC_193805"; transcript_id "Novel_002661"; exon_number "5"; gene_type "
Dt_chr5	Cufflinks	exon	41302618	41302671	.	-	.	gene_id "XLOC_193805"; transcript_id "Novel_002661"; exon_number "6"; gene_type "
Dt_chr5	Cufflinks	exon	41302790	41302891	.	-	.	gene_id "XLOC_193805"; transcript_id "Novel_002661"; exon_number "7"; gene_type "
Dt_chr5	Cufflinks	exon	41303046	41303120	.	-	.	gene_id "XLOC_193805"; transcript_id "Novel_002661"; exon_number "8"; gene_type "
Dt_chr5	Cufflinks	exon	41303272	41303906	.	-	.	gene_id "XLOC_193805"; transcript_id "Novel_002661"; exon_number "9"; gene_type "
Dt_chr5	Cufflinks	exon	41301380	41301736	.	-	.	gene_id "XLOC_193805"; transcript_id "Novel_002662"; exon_number "1"; gene_type "
Dt_chr5	Cufflinks	exon	41301828	41301926	.	-	.	gene_id "XLOC_193805"; transcript_id "Novel_002662"; exon_number "2"; gene_type "
Dt_chr5	Cufflinks	exon	41302060	41302174	.	-	.	gene_id "XLOC_193805"; transcript_id "Novel_002662"; exon_number "3"; gene_type "
Dt_chr5	Cufflinks	exon	41302255	41302347	.	-	.	gene_id "XLOC_193805"; transcript_id "Novel_002662"; exon_number "4"; gene_type "
Dt_chr5	Cufflinks	exon	41302443	41302535	.	-	.	gene_id "XLOC_193805"; transcript_id "Novel_002662"; exon_number "5"; gene_type "
Dt_chr5	Cufflinks	exon	41302618	41302671	.	-	.	gene_id "XLOC_193805"; transcript_id "Novel_002662"; exon_number "6"; gene_type "
Dt_chr5	Cufflinks	exon	41302790	41302891	.	-	.	gene_id "XLOC_193805"; transcript_id "Novel_002662"; exon_number "7"; gene_type "
Dt_chr5	Cufflinks	exon	41303046	41303120	.	-	.	gene_id "XLOC_193805"; transcript_id "Novel_002662"; exon_number "8"; gene_type "
Dt_chr5	Cufflinks	exon	41303272	41303895	.	-	.	gene_id "XLOC_193805"; transcript_id "Novel_002662"; exon_number "9"; gene_type "
Dt_chr5	Cufflinks	exon	41676257	41677760	.	-	.	gene_id "XLOC_193824"; transcript_id "Novel_002663"; exon_number "1"; gene_type "
Dt_chr5	Cufflinks	exon	41679947	41680205	.	-	.	gene_id "XLOC_193824"; transcript_id "Novel_002663"; exon_number "2"; gene_type "
Dt_chr5	Cufflinks	exon	43033914	43034197	.	-	.	gene_id "XLOC_193913"; transcript_id "Novel_002664"; exon_number "1"; gene_type "
Dt_chr5	Cufflinks	exon	43034338	43034435	.	-	.	gene_id "XLOC_193913"; transcript_id "Novel_002664"; exon_number "2"; gene_type "
Dt_chr5	Cufflinks	exon	43034741	43034921	.	-	.	gene_id "XLOC_193913"; transcript_id "Novel_002664"; exon_number "3"; gene_type "
Dt_chr5	Cufflinks	exon	43035007	43035084	.	-	.	gene_id "XLOC_193913"; transcript_id "Novel_002664"; exon_number "4"; gene_type "
Dt_chr5	Cufflinks	exon	43035520	43035648	.	-	.	gene_id "XLOC_193913"; transcript_id "Novel_002664"; exon_number "5"; gene_type "
Dt_chr5	Cufflinks	exon	47665063	47669565	.	-	.	gene_id "XLOC_194169"; transcript_id "Novel_002665"; exon_number "1"; gene_type "

Dt_chr5	Cufflinks	exon	47670769	47671110	.	-	.	gene_id "XLOC_194169"; transcript_id "Novel_002665"; exon_number "2"; gene_type "
Dt_chr5	Cufflinks	exon	49873293	49874266	.	-	.	gene_id "XLOC_194349"; transcript_id "Novel_002666"; exon_number "1"; gene_type "
Dt_chr5	Cufflinks	exon	49874451	49874853	.	-	.	gene_id "XLOC_194349"; transcript_id "Novel_002666"; exon_number "2"; gene_type "
Dt_chr5	Cufflinks	exon	50959840	50960877	.	-	.	gene_id "XLOC_194457"; transcript_id "Novel_002667"; exon_number "1"; gene_type "
Dt_chr5	Cufflinks	exon	50960971	50961135	.	-	.	gene_id "XLOC_194457"; transcript_id "Novel_002667"; exon_number "2"; gene_type "
Dt_chr5	Cufflinks	exon	54369101	54370336	.	-	.	gene_id "XLOC_194732"; transcript_id "Novel_002668"; exon_number "1"; gene_type "
Dt_chr5	Cufflinks	exon	54375602	54375655	.	-	.	gene_id "XLOC_194732"; transcript_id "Novel_002668"; exon_number "2"; gene_type "
Dt_chr5	Cufflinks	exon	54376021	54376030	.	-	.	gene_id "XLOC_194732"; transcript_id "Novel_002668"; exon_number "3"; gene_type "
Dt_chr5	Cufflinks	exon	54369101	54370336	.	-	.	gene_id "XLOC_194732"; transcript_id "Novel_002669"; exon_number "1"; gene_type "
Dt_chr5	Cufflinks	exon	54375602	54376081	.	-	.	gene_id "XLOC_194732"; transcript_id "Novel_002669"; exon_number "2"; gene_type "
Dt_chr5	Cufflinks	exon	54374297	54374925	.	-	.	gene_id "XLOC_194732"; transcript_id "Novel_002670"; exon_number "1"; gene_type "
Dt_chr5	Cufflinks	exon	54375602	54375655	.	-	.	gene_id "XLOC_194732"; transcript_id "Novel_002670"; exon_number "2"; gene_type "
Dt_chr5	Cufflinks	exon	54376021	54376064	.	-	.	gene_id "XLOC_194732"; transcript_id "Novel_002670"; exon_number "3"; gene_type "
Dt_chr6	Cufflinks	exon	221019	221109	.	+	.	gene_id "XLOC_195391"; transcript_id "Novel_002671"; exon_number "1"; gene_type "
Dt_chr6	Cufflinks	exon	221225	221356	.	+	.	gene_id "XLOC_195391"; transcript_id "Novel_002671"; exon_number "2"; gene_type "
Dt_chr6	Cufflinks	exon	222308	222893	.	+	.	gene_id "XLOC_195391"; transcript_id "Novel_002671"; exon_number "3"; gene_type "
Dt_chr6	Cufflinks	exon	221357	221358	.	+	.	gene_id "XLOC_195391"; transcript_id "Novel_002672"; exon_number "1"; gene_type "
Dt_chr6	Cufflinks	exon	222310	222958	.	+	.	gene_id "XLOC_195391"; transcript_id "Novel_002672"; exon_number "2"; gene_type "
Dt_chr6	Cufflinks	exon	785243	786119	.	+	.	gene_id "XLOC_195444"; transcript_id "Novel_002673"; exon_number "1"; gene_type "
Dt_chr6	Cufflinks	exon	786202	786562	.	+	.	gene_id "XLOC_195444"; transcript_id "Novel_002673"; exon_number "2"; gene_type "
Dt_chr6	Cufflinks	exon	790053	791534	.	+	.	gene_id "XLOC_195444"; transcript_id "Novel_002673"; exon_number "3"; gene_type "
Dt_chr6	Cufflinks	exon	785243	785771	.	+	.	gene_id "XLOC_195444"; transcript_id "Novel_002674"; exon_number "1"; gene_type "
Dt_chr6	Cufflinks	exon	786070	786119	.	+	.	gene_id "XLOC_195444"; transcript_id "Novel_002674"; exon_number "2"; gene_type "
Dt_chr6	Cufflinks	exon	786202	786562	.	+	.	gene_id "XLOC_195444"; transcript_id "Novel_002674"; exon_number "3"; gene_type "
Dt_chr6	Cufflinks	exon	790053	791534	.	+	.	gene_id "XLOC_195444"; transcript_id "Novel_002674"; exon_number "4"; gene_type "
Dt_chr6	Cufflinks	exon	785265	785771	.	+	.	gene_id "XLOC_195444"; transcript_id "Novel_002675"; exon_number "1"; gene_type "
Dt_chr6	Cufflinks	exon	786075	786119	.	+	.	gene_id "XLOC_195444"; transcript_id "Novel_002675"; exon_number "2"; gene_type "
Dt_chr6	Cufflinks	exon	786202	786562	.	+	.	gene_id "XLOC_195444"; transcript_id "Novel_002675"; exon_number "3"; gene_type "
Dt_chr6	Cufflinks	exon	790053	791568	.	+	.	gene_id "XLOC_195444"; transcript_id "Novel_002675"; exon_number "4"; gene_type "
Dt_chr6	Cufflinks	exon	2057808	2057981	.	+	.	gene_id "XLOC_195569"; transcript_id "Novel_002676"; exon_number "1"; gene_type "
Dt_chr6	Cufflinks	exon	2058167	2058897	.	+	.	gene_id "XLOC_195569"; transcript_id "Novel_002676"; exon_number "2"; gene_type "
Dt_chr6	Cufflinks	exon	2240700	2240993	.	+	.	gene_id "XLOC_195579"; transcript_id "Novel_002677"; exon_number "1"; gene_type "
Dt_chr6	Cufflinks	exon	2241082	2241504	.	+	.	gene_id "XLOC_195579"; transcript_id "Novel_002677"; exon_number "2"; gene_type "
Dt_chr6	Cufflinks	exon	2705901	2706133	.	+	.	gene_id "XLOC_195640"; transcript_id "Novel_002678"; exon_number "1"; gene_type "
Dt_chr6	Cufflinks	exon	2706346	2706401	.	+	.	gene_id "XLOC_195640"; transcript_id "Novel_002678"; exon_number "2"; gene_type "

Dt_chr6	Cufflinks	exon	2706586	2706656	.	+	.	gene_id "XLOC_195640"; transcript_id "Novel_002678"; exon_number "3"; gene_type "
Dt_chr6	Cufflinks	exon	2707315	2707405	.	+	.	gene_id "XLOC_195640"; transcript_id "Novel_002678"; exon_number "4"; gene_type "
Dt_chr6	Cufflinks	exon	2707513	2707624	.	+	.	gene_id "XLOC_195640"; transcript_id "Novel_002678"; exon_number "5"; gene_type "
Dt_chr6	Cufflinks	exon	2708041	2709155	.	+	.	gene_id "XLOC_195640"; transcript_id "Novel_002678"; exon_number "6"; gene_type "
Dt_chr6	Cufflinks	exon	2706093	2706133	.	+	.	gene_id "XLOC_195640"; transcript_id "Novel_002679"; exon_number "1"; gene_type "
Dt_chr6	Cufflinks	exon	2706346	2706401	.	+	.	gene_id "XLOC_195640"; transcript_id "Novel_002679"; exon_number "2"; gene_type "
Dt_chr6	Cufflinks	exon	2706586	2707405	.	+	.	gene_id "XLOC_195640"; transcript_id "Novel_002679"; exon_number "3"; gene_type "
Dt_chr6	Cufflinks	exon	2707513	2707624	.	+	.	gene_id "XLOC_195640"; transcript_id "Novel_002679"; exon_number "4"; gene_type "
Dt_chr6	Cufflinks	exon	2708041	2709137	.	+	.	gene_id "XLOC_195640"; transcript_id "Novel_002679"; exon_number "5"; gene_type "
Dt_chr6	Cufflinks	exon	4228758	4228826	.	+	.	gene_id "XLOC_195865"; transcript_id "Novel_002680"; exon_number "1"; gene_type "
Dt_chr6	Cufflinks	exon	4229300	4229339	.	+	.	gene_id "XLOC_195865"; transcript_id "Novel_002680"; exon_number "2"; gene_type "
Dt_chr6	Cufflinks	exon	4229625	4229713	.	+	.	gene_id "XLOC_195865"; transcript_id "Novel_002680"; exon_number "3"; gene_type "
Dt_chr6	Cufflinks	exon	4229827	4229909	.	+	.	gene_id "XLOC_195865"; transcript_id "Novel_002680"; exon_number "4"; gene_type "
Dt_chr6	Cufflinks	exon	4229998	4230264	.	+	.	gene_id "XLOC_195865"; transcript_id "Novel_002680"; exon_number "5"; gene_type "
Dt_chr6	Cufflinks	exon	6211292	6211727	.	+	.	gene_id "XLOC_196198"; transcript_id "Novel_002681"; exon_number "1"; gene_type "
Dt_chr6	Cufflinks	exon	6211855	6212680	.	+	.	gene_id "XLOC_196198"; transcript_id "Novel_002681"; exon_number "2"; gene_type "
Dt_chr6	Cufflinks	exon	6356582	6356693	.	+	.	gene_id "XLOC_196218"; transcript_id "Novel_002682"; exon_number "1"; gene_type "
Dt_chr6	Cufflinks	exon	6357187	6357323	.	+	.	gene_id "XLOC_196218"; transcript_id "Novel_002682"; exon_number "2"; gene_type "
Dt_chr6	Cufflinks	exon	6364491	6364639	.	+	.	gene_id "XLOC_196218"; transcript_id "Novel_002682"; exon_number "3"; gene_type "
Dt_chr6	Cufflinks	exon	6365102	6365188	.	+	.	gene_id "XLOC_196218"; transcript_id "Novel_002682"; exon_number "4"; gene_type "
Dt_chr6	Cufflinks	exon	6365478	6365636	.	+	.	gene_id "XLOC_196218"; transcript_id "Novel_002682"; exon_number "5"; gene_type "
Dt_chr6	Cufflinks	exon	6365876	6366696	.	+	.	gene_id "XLOC_196218"; transcript_id "Novel_002682"; exon_number "6"; gene_type "
Dt_chr6	Cufflinks	exon	6710988	6713196	.	+	.	gene_id "XLOC_196299"; transcript_id "Novel_002683"; exon_number "1"; gene_type "
Dt_chr6	Cufflinks	exon	6713340	6714237	.	+	.	gene_id "XLOC_196299"; transcript_id "Novel_002683"; exon_number "2"; gene_type "
Dt_chr6	Cufflinks	exon	7411601	7412759	.	+	.	gene_id "XLOC_196429"; transcript_id "Novel_002684"; exon_number "1"; gene_type "
Dt_chr6	Cufflinks	exon	8000199	8000298	.	+	.	gene_id "XLOC_196511"; transcript_id "Novel_002685"; exon_number "1"; gene_type "
Dt_chr6	Cufflinks	exon	8000821	8002269	.	+	.	gene_id "XLOC_196511"; transcript_id "Novel_002685"; exon_number "2"; gene_type "
Dt_chr6	Cufflinks	exon	8755583	8755980	.	+	.	gene_id "XLOC_196614"; transcript_id "Novel_002686"; exon_number "1"; gene_type "
Dt_chr6	Cufflinks	exon	8763310	8763860	.	+	.	gene_id "XLOC_196614"; transcript_id "Novel_002686"; exon_number "2"; gene_type "
Dt_chr6	Cufflinks	exon	9418591	9418689	.	+	.	gene_id "XLOC_196732"; transcript_id "Novel_002687"; exon_number "1"; gene_type "
Dt_chr6	Cufflinks	exon	9419729	9419846	.	+	.	gene_id "XLOC_196732"; transcript_id "Novel_002687"; exon_number "2"; gene_type "
Dt_chr6	Cufflinks	exon	9420827	9420866	.	+	.	gene_id "XLOC_196732"; transcript_id "Novel_002687"; exon_number "3"; gene_type "
Dt_chr6	Cufflinks	exon	9421019	9421114	.	+	.	gene_id "XLOC_196732"; transcript_id "Novel_002687"; exon_number "4"; gene_type "
Dt_chr6	Cufflinks	exon	9421727	9421923	.	+	.	gene_id "XLOC_196732"; transcript_id "Novel_002687"; exon_number "5"; gene_type "
Dt_chr6	Cufflinks	exon	9422054	9422140	.	+	.	gene_id "XLOC_196732"; transcript_id "Novel_002687"; exon_number "6"; gene_type "

Dt_chr6	Cufflinks	exon	9422250	9422496	.	+	.	gene_id "XLOC_196732"; transcript_id "Novel_002687"; exon_number "7"; gene_type "
Dt_chr6	Cufflinks	exon	9418591	9418689	.	+	.	gene_id "XLOC_196732"; transcript_id "Novel_002688"; exon_number "1"; gene_type "
Dt_chr6	Cufflinks	exon	9419729	9419846	.	+	.	gene_id "XLOC_196732"; transcript_id "Novel_002688"; exon_number "2"; gene_type "
Dt_chr6	Cufflinks	exon	9420827	9420866	.	+	.	gene_id "XLOC_196732"; transcript_id "Novel_002688"; exon_number "3"; gene_type "
Dt_chr6	Cufflinks	exon	9421019	9421114	.	+	.	gene_id "XLOC_196732"; transcript_id "Novel_002688"; exon_number "4"; gene_type "
Dt_chr6	Cufflinks	exon	9421727	9421923	.	+	.	gene_id "XLOC_196732"; transcript_id "Novel_002688"; exon_number "5"; gene_type "
Dt_chr6	Cufflinks	exon	9422054	9422140	.	+	.	gene_id "XLOC_196732"; transcript_id "Novel_002688"; exon_number "6"; gene_type "
Dt_chr6	Cufflinks	exon	9422247	9422905	.	+	.	gene_id "XLOC_196732"; transcript_id "Novel_002688"; exon_number "7"; gene_type "
Dt_chr6	Cufflinks	exon	9711525	9711750	.	+	.	gene_id "XLOC_196790"; transcript_id "Novel_002689"; exon_number "1"; gene_type "
Dt_chr6	Cufflinks	exon	9712012	9712063	.	+	.	gene_id "XLOC_196790"; transcript_id "Novel_002689"; exon_number "2"; gene_type "
Dt_chr6	Cufflinks	exon	9712368	9712982	.	+	.	gene_id "XLOC_196790"; transcript_id "Novel_002689"; exon_number "3"; gene_type "
Dt_chr6	Cufflinks	exon	9711525	9711750	.	+	.	gene_id "XLOC_196790"; transcript_id "Novel_002690"; exon_number "1"; gene_type "
Dt_chr6	Cufflinks	exon	9712012	9712063	.	+	.	gene_id "XLOC_196790"; transcript_id "Novel_002690"; exon_number "2"; gene_type "
Dt_chr6	Cufflinks	exon	9712368	9712444	.	+	.	gene_id "XLOC_196790"; transcript_id "Novel_002690"; exon_number "3"; gene_type "
Dt_chr6	Cufflinks	exon	9712536	9712669	.	+	.	gene_id "XLOC_196790"; transcript_id "Novel_002690"; exon_number "4"; gene_type "
Dt_chr6	Cufflinks	exon	9712893	9712982	.	+	.	gene_id "XLOC_196790"; transcript_id "Novel_002690"; exon_number "5"; gene_type "
Dt_chr6	Cufflinks	exon	9996695	9997425	.	+	.	gene_id "XLOC_196824"; transcript_id "Novel_002691"; exon_number "1"; gene_type "
Dt_chr6	Cufflinks	exon	9997704	9997826	.	+	.	gene_id "XLOC_196824"; transcript_id "Novel_002691"; exon_number "2"; gene_type "
Dt_chr6	Cufflinks	exon	9998617	9998862	.	+	.	gene_id "XLOC_196824"; transcript_id "Novel_002691"; exon_number "3"; gene_type "
Dt_chr6	Cufflinks	exon	12205339	12205403	.	+	.	gene_id "XLOC_197108"; transcript_id "Novel_002692"; exon_number "1"; gene_type "
Dt_chr6	Cufflinks	exon	12205504	12205597	.	+	.	gene_id "XLOC_197108"; transcript_id "Novel_002692"; exon_number "2"; gene_type "
Dt_chr6	Cufflinks	exon	12205808	12205917	.	+	.	gene_id "XLOC_197108"; transcript_id "Novel_002692"; exon_number "3"; gene_type "
Dt_chr6	Cufflinks	exon	12337001	12337247	.	+	.	gene_id "XLOC_197121"; transcript_id "Novel_002693"; exon_number "1"; gene_type "
Dt_chr6	Cufflinks	exon	12337350	12337445	.	+	.	gene_id "XLOC_197121"; transcript_id "Novel_002693"; exon_number "2"; gene_type "
Dt_chr6	Cufflinks	exon	12337655	12337712	.	+	.	gene_id "XLOC_197121"; transcript_id "Novel_002693"; exon_number "3"; gene_type "
Dt_chr6	Cufflinks	exon	13641866	13642533	.	+	.	gene_id "XLOC_197338"; transcript_id "Novel_002694"; exon_number "1"; gene_type "
Dt_chr6	Cufflinks	exon	13642896	13643877	.	+	.	gene_id "XLOC_197338"; transcript_id "Novel_002694"; exon_number "2"; gene_type "
Dt_chr6	Cufflinks	exon	14276296	14276606	.	+	.	gene_id "XLOC_197433"; transcript_id "Novel_002695"; exon_number "1"; gene_type "
Dt_chr6	Cufflinks	exon	14277210	14278278	.	+	.	gene_id "XLOC_197433"; transcript_id "Novel_002695"; exon_number "2"; gene_type "
Dt_chr6	Cufflinks	exon	14278947	14279390	.	+	.	gene_id "XLOC_197433"; transcript_id "Novel_002695"; exon_number "3"; gene_type "
Dt_chr6	Cufflinks	exon	14280411	14280466	.	+	.	gene_id "XLOC_197433"; transcript_id "Novel_002695"; exon_number "4"; gene_type "
Dt_chr6	Cufflinks	exon	14276357	14276606	.	+	.	gene_id "XLOC_197433"; transcript_id "Novel_002696"; exon_number "1"; gene_type "
Dt_chr6	Cufflinks	exon	14276950	14277074	.	+	.	gene_id "XLOC_197433"; transcript_id "Novel_002696"; exon_number "2"; gene_type "
Dt_chr6	Cufflinks	exon	14277210	14278278	.	+	.	gene_id "XLOC_197433"; transcript_id "Novel_002696"; exon_number "3"; gene_type "
Dt_chr6	Cufflinks	exon	14278947	14279390	.	+	.	gene_id "XLOC_197433"; transcript_id "Novel_002696"; exon_number "4"; gene_type "

Dt_chr6	Cufflinks	exon	14280411	14281485	.	+	.	gene_id "XLOC_197433"; transcript_id "Novel_002696"; exon_number "5"; gene_type "
Dt_chr6	Cufflinks	exon	14276357	14276606	.	+	.	gene_id "XLOC_197433"; transcript_id "Novel_002697"; exon_number "1"; gene_type "
Dt_chr6	Cufflinks	exon	14276950	14276988	.	+	.	gene_id "XLOC_197433"; transcript_id "Novel_002697"; exon_number "2"; gene_type "
Dt_chr6	Cufflinks	exon	14277210	14278278	.	+	.	gene_id "XLOC_197433"; transcript_id "Novel_002697"; exon_number "3"; gene_type "
Dt_chr6	Cufflinks	exon	14278947	14279390	.	+	.	gene_id "XLOC_197433"; transcript_id "Novel_002697"; exon_number "4"; gene_type "
Dt_chr6	Cufflinks	exon	14280411	14281485	.	+	.	gene_id "XLOC_197433"; transcript_id "Novel_002697"; exon_number "5"; gene_type "
Dt_chr6	Cufflinks	exon	14276569	14276606	.	+	.	gene_id "XLOC_197433"; transcript_id "Novel_002698"; exon_number "1"; gene_type "
Dt_chr6	Cufflinks	exon	14276950	14278278	.	+	.	gene_id "XLOC_197433"; transcript_id "Novel_002698"; exon_number "2"; gene_type "
Dt_chr6	Cufflinks	exon	14278947	14279390	.	+	.	gene_id "XLOC_197433"; transcript_id "Novel_002698"; exon_number "3"; gene_type "
Dt_chr6	Cufflinks	exon	14280411	14281465	.	+	.	gene_id "XLOC_197433"; transcript_id "Novel_002698"; exon_number "4"; gene_type "
Dt_chr6	Cufflinks	exon	14360079	14360269	.	+	.	gene_id "XLOC_197441"; transcript_id "Novel_002699"; exon_number "1"; gene_type "
Dt_chr6	Cufflinks	exon	14360390	14361969	.	+	.	gene_id "XLOC_197441"; transcript_id "Novel_002699"; exon_number "2"; gene_type "
Dt_chr6	Cufflinks	exon	14905530	14907903	.	+	.	gene_id "XLOC_197530"; transcript_id "Novel_002700"; exon_number "1"; gene_type "
Dt_chr6	Cufflinks	exon	14907992	14908254	.	+	.	gene_id "XLOC_197530"; transcript_id "Novel_002700"; exon_number "2"; gene_type "
Dt_chr6	Cufflinks	exon	14906044	14906047	.	+	.	gene_id "XLOC_197530"; transcript_id "Novel_002701"; exon_number "1"; gene_type "
Dt_chr6	Cufflinks	exon	14906155	14906236	.	+	.	gene_id "XLOC_197530"; transcript_id "Novel_002701"; exon_number "2"; gene_type "
Dt_chr6	Cufflinks	exon	14906327	14906423	.	+	.	gene_id "XLOC_197530"; transcript_id "Novel_002701"; exon_number "3"; gene_type "
Dt_chr6	Cufflinks	exon	14906588	14906663	.	+	.	gene_id "XLOC_197530"; transcript_id "Novel_002701"; exon_number "4"; gene_type "
Dt_chr6	Cufflinks	exon	14906757	14906815	.	+	.	gene_id "XLOC_197530"; transcript_id "Novel_002701"; exon_number "5"; gene_type "
Dt_chr6	Cufflinks	exon	14906927	14907049	.	+	.	gene_id "XLOC_197530"; transcript_id "Novel_002701"; exon_number "6"; gene_type "
Dt_chr6	Cufflinks	exon	14907636	14907693	.	+	.	gene_id "XLOC_197530"; transcript_id "Novel_002701"; exon_number "7"; gene_type "
Dt_chr6	Cufflinks	exon	14907818	14907903	.	+	.	gene_id "XLOC_197530"; transcript_id "Novel_002701"; exon_number "8"; gene_type "
Dt_chr6	Cufflinks	exon	14907992	14908563	.	+	.	gene_id "XLOC_197530"; transcript_id "Novel_002701"; exon_number "9"; gene_type "
Dt_chr6	Cufflinks	exon	14906741	14907693	.	+	.	gene_id "XLOC_197530"; transcript_id "Novel_002702"; exon_number "1"; gene_type "
Dt_chr6	Cufflinks	exon	14907818	14907903	.	+	.	gene_id "XLOC_197530"; transcript_id "Novel_002702"; exon_number "2"; gene_type "
Dt_chr6	Cufflinks	exon	14907992	14908444	.	+	.	gene_id "XLOC_197530"; transcript_id "Novel_002702"; exon_number "3"; gene_type "
Dt_chr6	Cufflinks	exon	20833472	20833698	.	+	.	gene_id "XLOC_198188"; transcript_id "Novel_002703"; exon_number "1"; gene_type "
Dt_chr6	Cufflinks	exon	23729612	23730620	.	+	.	gene_id "XLOC_198408"; transcript_id "Novel_002704"; exon_number "1"; gene_type "
Dt_chr6	Cufflinks	exon	23731033	23731143	.	+	.	gene_id "XLOC_198408"; transcript_id "Novel_002704"; exon_number "2"; gene_type "
Dt_chr6	Cufflinks	exon	23731580	23731647	.	+	.	gene_id "XLOC_198408"; transcript_id "Novel_002704"; exon_number "3"; gene_type "
Dt_chr6	Cufflinks	exon	23731789	23732619	.	+	.	gene_id "XLOC_198408"; transcript_id "Novel_002704"; exon_number "4"; gene_type "
Dt_chr6	Cufflinks	exon	23730215	23730466	.	+	.	gene_id "XLOC_198408"; transcript_id "Novel_002705"; exon_number "1"; gene_type "
Dt_chr6	Cufflinks	exon	23730553	23730620	.	+	.	gene_id "XLOC_198408"; transcript_id "Novel_002705"; exon_number "2"; gene_type "
Dt_chr6	Cufflinks	exon	23731033	23731143	.	+	.	gene_id "XLOC_198408"; transcript_id "Novel_002705"; exon_number "3"; gene_type "
Dt_chr6	Cufflinks	exon	23731561	23731647	.	+	.	gene_id "XLOC_198408"; transcript_id "Novel_002705"; exon_number "4"; gene_type "

Dt_chr6	Cufflinks	exon	23731789	23732769	.	+	.	gene_id "XLOC_198408"; transcript_id "Novel_002705"; exon_number "5"; gene_type "
Dt_chr6	Cufflinks	exon	23730974	23731647	.	+	.	gene_id "XLOC_198408"; transcript_id "Novel_002706"; exon_number "1"; gene_type "
Dt_chr6	Cufflinks	exon	23731789	23732957	.	+	.	gene_id "XLOC_198408"; transcript_id "Novel_002706"; exon_number "2"; gene_type "
Dt_chr6	Cufflinks	exon	24146642	24146814	.	+	.	gene_id "XLOC_198431"; transcript_id "Novel_002707"; exon_number "1"; gene_type "
Dt_chr6	Cufflinks	exon	24147836	24148225	.	+	.	gene_id "XLOC_198431"; transcript_id "Novel_002707"; exon_number "2"; gene_type "
Dt_chr6	Cufflinks	exon	24226495	24226713	.	+	.	gene_id "XLOC_198436"; transcript_id "Novel_002708"; exon_number "1"; gene_type "
Dt_chr6	Cufflinks	exon	24227556	24227646	.	+	.	gene_id "XLOC_198436"; transcript_id "Novel_002708"; exon_number "2"; gene_type "
Dt_chr6	Cufflinks	exon	24227769	24227896	.	+	.	gene_id "XLOC_198436"; transcript_id "Novel_002708"; exon_number "3"; gene_type "
Dt_chr6	Cufflinks	exon	24228270	24228392	.	+	.	gene_id "XLOC_198436"; transcript_id "Novel_002708"; exon_number "4"; gene_type "
Dt_chr6	Cufflinks	exon	24228510	24228598	.	+	.	gene_id "XLOC_198436"; transcript_id "Novel_002708"; exon_number "5"; gene_type "
Dt_chr6	Cufflinks	exon	24228708	24228828	.	+	.	gene_id "XLOC_198436"; transcript_id "Novel_002708"; exon_number "6"; gene_type "
Dt_chr6	Cufflinks	exon	24228940	24229254	.	+	.	gene_id "XLOC_198436"; transcript_id "Novel_002708"; exon_number "7"; gene_type "
Dt_chr6	Cufflinks	exon	24229348	24229605	.	+	.	gene_id "XLOC_198436"; transcript_id "Novel_002709"; exon_number "1"; gene_type "
Dt_chr6	Cufflinks	exon	24229685	24229881	.	+	.	gene_id "XLOC_198436"; transcript_id "Novel_002709"; exon_number "2"; gene_type "
Dt_chr6	Cufflinks	exon	24230148	24230476	.	+	.	gene_id "XLOC_198436"; transcript_id "Novel_002709"; exon_number "3"; gene_type "
Dt_chr6	Cufflinks	exon	24229557	24229881	.	+	.	gene_id "XLOC_198436"; transcript_id "Novel_002710"; exon_number "1"; gene_type "
Dt_chr6	Cufflinks	exon	24230148	24230599	.	+	.	gene_id "XLOC_198436"; transcript_id "Novel_002710"; exon_number "2"; gene_type "
Dt_chr6	Cufflinks	exon	24236861	24237046	.	+	.	gene_id "XLOC_198441"; transcript_id "Novel_002711"; exon_number "1"; gene_type "
Dt_chr6	Cufflinks	exon	24237374	24237542	.	+	.	gene_id "XLOC_198441"; transcript_id "Novel_002711"; exon_number "2"; gene_type "
Dt_chr6	Cufflinks	exon	24237653	24237761	.	+	.	gene_id "XLOC_198441"; transcript_id "Novel_002711"; exon_number "3"; gene_type "
Dt_chr6	Cufflinks	exon	24238647	24238729	.	+	.	gene_id "XLOC_198441"; transcript_id "Novel_002711"; exon_number "4"; gene_type "
Dt_chr6	Cufflinks	exon	24238809	24238871	.	+	.	gene_id "XLOC_198441"; transcript_id "Novel_002711"; exon_number "5"; gene_type "
Dt_chr6	Cufflinks	exon	24238973	24239552	.	+	.	gene_id "XLOC_198441"; transcript_id "Novel_002711"; exon_number "6"; gene_type "
Dt_chr6	Cufflinks	exon	30463076	30466924	.	+	.	gene_id "XLOC_198868"; transcript_id "Novel_002712"; exon_number "1"; gene_type "
Dt_chr6	Cufflinks	exon	30467023	30467620	.	+	.	gene_id "XLOC_198868"; transcript_id "Novel_002712"; exon_number "2"; gene_type "
Dt_chr6	Cufflinks	exon	34678408	34678512	.	+	.	gene_id "XLOC_199088"; transcript_id "Novel_002713"; exon_number "1"; gene_type "
Dt_chr6	Cufflinks	exon	34678598	34678799	.	+	.	gene_id "XLOC_199088"; transcript_id "Novel_002713"; exon_number "2"; gene_type "
Dt_chr6	Cufflinks	exon	35489250	35489494	.	+	.	gene_id "XLOC_199145"; transcript_id "Novel_002714"; exon_number "1"; gene_type "
Dt_chr6	Cufflinks	exon	35489722	35489795	.	+	.	gene_id "XLOC_199145"; transcript_id "Novel_002714"; exon_number "2"; gene_type "
Dt_chr6	Cufflinks	exon	35490075	35490763	.	+	.	gene_id "XLOC_199145"; transcript_id "Novel_002714"; exon_number "3"; gene_type "
Dt_chr6	Cufflinks	exon	35491030	35491134	.	+	.	gene_id "XLOC_199145"; transcript_id "Novel_002714"; exon_number "4"; gene_type "
Dt_chr6	Cufflinks	exon	35491596	35492505	.	+	.	gene_id "XLOC_199145"; transcript_id "Novel_002714"; exon_number "5"; gene_type "
Dt_chr6	Cufflinks	exon	35489307	35489494	.	+	.	gene_id "XLOC_199145"; transcript_id "Novel_002715"; exon_number "1"; gene_type "
Dt_chr6	Cufflinks	exon	35489765	35489795	.	+	.	gene_id "XLOC_199145"; transcript_id "Novel_002715"; exon_number "2"; gene_type "
Dt_chr6	Cufflinks	exon	35490075	35490763	.	+	.	gene_id "XLOC_199145"; transcript_id "Novel_002715"; exon_number "3"; gene_type "

Dt_chr6	Cufflinks	exon	35491030	35491134	.	+	.	gene_id "XLOC_199145"; transcript_id "Novel_002715"; exon_number "4"; gene_type "
Dt_chr6	Cufflinks	exon	35491596	35492504	.	+	.	gene_id "XLOC_199145"; transcript_id "Novel_002715"; exon_number "5"; gene_type "
Dt_chr6	Cufflinks	exon	35489307	35489494	.	+	.	gene_id "XLOC_199145"; transcript_id "Novel_002716"; exon_number "1"; gene_type "
Dt_chr6	Cufflinks	exon	35489722	35489795	.	+	.	gene_id "XLOC_199145"; transcript_id "Novel_002716"; exon_number "2"; gene_type "
Dt_chr6	Cufflinks	exon	35490075	35490108	.	+	.	gene_id "XLOC_199145"; transcript_id "Novel_002716"; exon_number "3"; gene_type "
Dt_chr6	Cufflinks	exon	35490711	35490763	.	+	.	gene_id "XLOC_199145"; transcript_id "Novel_002716"; exon_number "4"; gene_type "
Dt_chr6	Cufflinks	exon	35491030	35491134	.	+	.	gene_id "XLOC_199145"; transcript_id "Novel_002716"; exon_number "5"; gene_type "
Dt_chr6	Cufflinks	exon	35491596	35492515	.	+	.	gene_id "XLOC_199145"; transcript_id "Novel_002716"; exon_number "6"; gene_type "
Dt_chr6	Cufflinks	exon	35489529	35490763	.	+	.	gene_id "XLOC_199145"; transcript_id "Novel_002717"; exon_number "1"; gene_type "
Dt_chr6	Cufflinks	exon	35491030	35491134	.	+	.	gene_id "XLOC_199145"; transcript_id "Novel_002717"; exon_number "2"; gene_type "
Dt_chr6	Cufflinks	exon	35491596	35492505	.	+	.	gene_id "XLOC_199145"; transcript_id "Novel_002717"; exon_number "3"; gene_type "
Dt_chr6	Cufflinks	exon	37564819	37565074	.	+	.	gene_id "XLOC_199305"; transcript_id "Novel_002718"; exon_number "1"; gene_type "
Dt_chr6	Cufflinks	exon	37566350	37566491	.	+	.	gene_id "XLOC_199305"; transcript_id "Novel_002718"; exon_number "2"; gene_type "
Dt_chr6	Cufflinks	exon	37564819	37565074	.	+	.	gene_id "XLOC_199305"; transcript_id "Novel_002719"; exon_number "1"; gene_type "
Dt_chr6	Cufflinks	exon	37566361	37566491	.	+	.	gene_id "XLOC_199305"; transcript_id "Novel_002719"; exon_number "2"; gene_type "
Dt_chr6	Cufflinks	exon	43347792	43348577	.	+	.	gene_id "XLOC_199737"; transcript_id "Novel_002720"; exon_number "1"; gene_type "
Dt_chr6	Cufflinks	exon	43348689	43348770	.	+	.	gene_id "XLOC_199737"; transcript_id "Novel_002720"; exon_number "2"; gene_type "
Dt_chr6	Cufflinks	exon	43348895	43348939	.	+	.	gene_id "XLOC_199737"; transcript_id "Novel_002720"; exon_number "3"; gene_type "
Dt_chr6	Cufflinks	exon	43349049	43349198	.	+	.	gene_id "XLOC_199737"; transcript_id "Novel_002720"; exon_number "4"; gene_type "
Dt_chr6	Cufflinks	exon	43349283	43349541	.	+	.	gene_id "XLOC_199737"; transcript_id "Novel_002720"; exon_number "5"; gene_type "
Dt_chr6	Cufflinks	exon	43349643	43349776	.	+	.	gene_id "XLOC_199737"; transcript_id "Novel_002720"; exon_number "6"; gene_type "
Dt_chr6	Cufflinks	exon	43349908	43350063	.	+	.	gene_id "XLOC_199737"; transcript_id "Novel_002720"; exon_number "7"; gene_type "
Dt_chr6	Cufflinks	exon	43350450	43350529	.	+	.	gene_id "XLOC_199737"; transcript_id "Novel_002720"; exon_number "8"; gene_type "
Dt_chr6	Cufflinks	exon	43351801	43352155	.	+	.	gene_id "XLOC_199737"; transcript_id "Novel_002720"; exon_number "9"; gene_type "
Dt_chr6	Cufflinks	exon	43347800	43348339	.	+	.	gene_id "XLOC_199737"; transcript_id "Novel_002721"; exon_number "1"; gene_type "
Dt_chr6	Cufflinks	exon	43348423	43348577	.	+	.	gene_id "XLOC_199737"; transcript_id "Novel_002721"; exon_number "2"; gene_type "
Dt_chr6	Cufflinks	exon	43348689	43348770	.	+	.	gene_id "XLOC_199737"; transcript_id "Novel_002721"; exon_number "3"; gene_type "
Dt_chr6	Cufflinks	exon	43348895	43348939	.	+	.	gene_id "XLOC_199737"; transcript_id "Novel_002721"; exon_number "4"; gene_type "
Dt_chr6	Cufflinks	exon	43349049	43349198	.	+	.	gene_id "XLOC_199737"; transcript_id "Novel_002721"; exon_number "5"; gene_type "
Dt_chr6	Cufflinks	exon	43349283	43349541	.	+	.	gene_id "XLOC_199737"; transcript_id "Novel_002721"; exon_number "6"; gene_type "
Dt_chr6	Cufflinks	exon	43349643	43349776	.	+	.	gene_id "XLOC_199737"; transcript_id "Novel_002721"; exon_number "7"; gene_type "
Dt_chr6	Cufflinks	exon	43349908	43350259	.	+	.	gene_id "XLOC_199737"; transcript_id "Novel_002721"; exon_number "8"; gene_type "
Dt_chr6	Cufflinks	exon	43347800	43348339	.	+	.	gene_id "XLOC_199737"; transcript_id "Novel_002722"; exon_number "1"; gene_type "
Dt_chr6	Cufflinks	exon	43348423	43348577	.	+	.	gene_id "XLOC_199737"; transcript_id "Novel_002722"; exon_number "2"; gene_type "
Dt_chr6	Cufflinks	exon	43348689	43348770	.	+	.	gene_id "XLOC_199737"; transcript_id "Novel_002722"; exon_number "3"; gene_type "

Dt_chr6	Cufflinks	exon	43348895	43348939	.	+	.	gene_id "XLOC_199737"; transcript_id "Novel_002722"; exon_number "4"; gene_type "
Dt_chr6	Cufflinks	exon	43349049	43349198	.	+	.	gene_id "XLOC_199737"; transcript_id "Novel_002722"; exon_number "5"; gene_type "
Dt_chr6	Cufflinks	exon	43349283	43349541	.	+	.	gene_id "XLOC_199737"; transcript_id "Novel_002722"; exon_number "6"; gene_type "
Dt_chr6	Cufflinks	exon	43349643	43349776	.	+	.	gene_id "XLOC_199737"; transcript_id "Novel_002722"; exon_number "7"; gene_type "
Dt_chr6	Cufflinks	exon	43349908	43350063	.	+	.	gene_id "XLOC_199737"; transcript_id "Novel_002722"; exon_number "8"; gene_type "
Dt_chr6	Cufflinks	exon	43350450	43350529	.	+	.	gene_id "XLOC_199737"; transcript_id "Novel_002722"; exon_number "9"; gene_type "
Dt_chr6	Cufflinks	exon	43351801	43352145	.	+	.	gene_id "XLOC_199737"; transcript_id "Novel_002722"; exon_number "10"; gene_type
Dt_chr6	Cufflinks	exon	43598205	43598801	.	+	.	gene_id "XLOC_199757"; transcript_id "Novel_002723"; exon_number "1"; gene_type "
Dt_chr6	Cufflinks	exon	43600231	43600279	.	+	.	gene_id "XLOC_199757"; transcript_id "Novel_002723"; exon_number "2"; gene_type "
Dt_chr6	Cufflinks	exon	44068451	44068562	.	+	.	gene_id "XLOC_199785"; transcript_id "Novel_002724"; exon_number "1"; gene_type "
Dt_chr6	Cufflinks	exon	44068726	44069001	.	+	.	gene_id "XLOC_199785"; transcript_id "Novel_002724"; exon_number "2"; gene_type "
Dt_chr6	Cufflinks	exon	44069406	44069969	.	+	.	gene_id "XLOC_199785"; transcript_id "Novel_002724"; exon_number "3"; gene_type "
Dt_chr6	Cufflinks	exon	1054877	1055167	.	-	.	gene_id "XLOC_199915"; transcript_id "Novel_002725"; exon_number "1"; gene_type "
Dt_chr6	Cufflinks	exon	1333129	1333543	.	-	.	gene_id "XLOC_199930"; transcript_id "Novel_002726"; exon_number "1"; gene_type "
Dt_chr6	Cufflinks	exon	1334658	1335047	.	-	.	gene_id "XLOC_199930"; transcript_id "Novel_002726"; exon_number "2"; gene_type "
Dt_chr6	Cufflinks	exon	1335178	1335339	.	-	.	gene_id "XLOC_199930"; transcript_id "Novel_002726"; exon_number "3"; gene_type "
Dt_chr6	Cufflinks	exon	1334357	1335047	.	-	.	gene_id "XLOC_199930"; transcript_id "Novel_002727"; exon_number "1"; gene_type "
Dt_chr6	Cufflinks	exon	1335178	1335339	.	-	.	gene_id "XLOC_199930"; transcript_id "Novel_002727"; exon_number "2"; gene_type "
Dt_chr6	Cufflinks	exon	2971210	2972233	.	-	.	gene_id "XLOC_200045"; transcript_id "Novel_002728"; exon_number "1"; gene_type "
Dt_chr6	Cufflinks	exon	2974590	2974817	.	-	.	gene_id "XLOC_200045"; transcript_id "Novel_002728"; exon_number "2"; gene_type "
Dt_chr6	Cufflinks	exon	2975037	2975185	.	-	.	gene_id "XLOC_200045"; transcript_id "Novel_002728"; exon_number "3"; gene_type "
Dt_chr6	Cufflinks	exon	2975788	2975968	.	-	.	gene_id "XLOC_200045"; transcript_id "Novel_002728"; exon_number "4"; gene_type "
Dt_chr6	Cufflinks	exon	2976224	2976430	.	-	.	gene_id "XLOC_200045"; transcript_id "Novel_002728"; exon_number "5"; gene_type "
Dt_chr6	Cufflinks	exon	2977718	2978134	.	-	.	gene_id "XLOC_200045"; transcript_id "Novel_002728"; exon_number "6"; gene_type "
Dt_chr6	Cufflinks	exon	4035516	4036196	.	-	.	gene_id "XLOC_200260"; transcript_id "Novel_002729"; exon_number "1"; gene_type "
Dt_chr6	Cufflinks	exon	4036393	4036608	.	-	.	gene_id "XLOC_200260"; transcript_id "Novel_002729"; exon_number "2"; gene_type "
Dt_chr6	Cufflinks	exon	4036749	4036946	.	-	.	gene_id "XLOC_200260"; transcript_id "Novel_002729"; exon_number "3"; gene_type "
Dt_chr6	Cufflinks	exon	4037126	4037304	.	-	.	gene_id "XLOC_200260"; transcript_id "Novel_002729"; exon_number "4"; gene_type "
Dt_chr6	Cufflinks	exon	4035567	4036196	.	-	.	gene_id "XLOC_200260"; transcript_id "Novel_002730"; exon_number "1"; gene_type "
Dt_chr6	Cufflinks	exon	4036393	4036946	.	-	.	gene_id "XLOC_200260"; transcript_id "Novel_002730"; exon_number "2"; gene_type "
Dt_chr6	Cufflinks	exon	4037126	4037312	.	-	.	gene_id "XLOC_200260"; transcript_id "Novel_002730"; exon_number "3"; gene_type "
Dt_chr6	Cufflinks	exon	4723330	4724414	.	-	.	gene_id "XLOC_200378"; transcript_id "Novel_002731"; exon_number "1"; gene_type "
Dt_chr6	Cufflinks	exon	4725008	4725137	.	-	.	gene_id "XLOC_200378"; transcript_id "Novel_002731"; exon_number "2"; gene_type "
Dt_chr6	Cufflinks	exon	4725245	4725521	.	-	.	gene_id "XLOC_200378"; transcript_id "Novel_002731"; exon_number "3"; gene_type "
Dt_chr6	Cufflinks	exon	4725625	4725673	.	-	.	gene_id "XLOC_200378"; transcript_id "Novel_002731"; exon_number "4"; gene_type "

Dt_chr6	Cufflinks	exon	4725759	4725843	.	-	.	gene_id "XLOC_200378"; transcript_id "Novel_002731"; exon_number "5"; gene_type "
Dt_chr6	Cufflinks	exon	5424204	5425161	.	-	.	gene_id "XLOC_200490"; transcript_id "Novel_002732"; exon_number "1"; gene_type "
Dt_chr6	Cufflinks	exon	5425354	5426985	.	-	.	gene_id "XLOC_200490"; transcript_id "Novel_002732"; exon_number "2"; gene_type "
Dt_chr6	Cufflinks	exon	5427129	5427169	.	-	.	gene_id "XLOC_200490"; transcript_id "Novel_002732"; exon_number "3"; gene_type "
Dt_chr6	Cufflinks	exon	5427672	5427691	.	-	.	gene_id "XLOC_200490"; transcript_id "Novel_002732"; exon_number "4"; gene_type "
Dt_chr6	Cufflinks	exon	5424230	5425161	.	-	.	gene_id "XLOC_200490"; transcript_id "Novel_002733"; exon_number "1"; gene_type "
Dt_chr6	Cufflinks	exon	5425354	5426985	.	-	.	gene_id "XLOC_200490"; transcript_id "Novel_002733"; exon_number "2"; gene_type "
Dt_chr6	Cufflinks	exon	5427129	5427604	.	-	.	gene_id "XLOC_200490"; transcript_id "Novel_002733"; exon_number "3"; gene_type "
Dt_chr6	Cufflinks	exon	13786205	13786511	.	-	.	gene_id "XLOC_201850"; transcript_id "Novel_002734"; exon_number "1"; gene_type "
Dt_chr6	Cufflinks	exon	13786627	13787422	.	-	.	gene_id "XLOC_201850"; transcript_id "Novel_002734"; exon_number "2"; gene_type "
Dt_chr6	Cufflinks	exon	14847708	14848876	.	-	.	gene_id "XLOC_202027"; transcript_id "Novel_002735"; exon_number "1"; gene_type "
Dt_chr6	Cufflinks	exon	14849009	14849062	.	-	.	gene_id "XLOC_202027"; transcript_id "Novel_002735"; exon_number "2"; gene_type "
Dt_chr6	Cufflinks	exon	15532149	15532380	.	-	.	gene_id "XLOC_202147"; transcript_id "Novel_002736"; exon_number "1"; gene_type "
Dt_chr6	Cufflinks	exon	15648304	15648933	.	-	.	gene_id "XLOC_202156"; transcript_id "Novel_002737"; exon_number "1"; gene_type "
Dt_chr6	Cufflinks	exon	15649233	15649382	.	-	.	gene_id "XLOC_202156"; transcript_id "Novel_002737"; exon_number "2"; gene_type "
Dt_chr6	Cufflinks	exon	17103001	17104213	.	-	.	gene_id "XLOC_202379"; transcript_id "Novel_002738"; exon_number "1"; gene_type "
Dt_chr6	Cufflinks	exon	17104654	17104838	.	-	.	gene_id "XLOC_202379"; transcript_id "Novel_002738"; exon_number "2"; gene_type "
Dt_chr6	Cufflinks	exon	18313604	18314334	.	-	.	gene_id "XLOC_202542"; transcript_id "Novel_002739"; exon_number "1"; gene_type "
Dt_chr6	Cufflinks	exon	18314587	18314768	.	-	.	gene_id "XLOC_202542"; transcript_id "Novel_002739"; exon_number "2"; gene_type "
Dt_chr6	Cufflinks	exon	18314951	18315028	.	-	.	gene_id "XLOC_202542"; transcript_id "Novel_002739"; exon_number "3"; gene_type "
Dt_chr6	Cufflinks	exon	18315103	18315174	.	-	.	gene_id "XLOC_202542"; transcript_id "Novel_002739"; exon_number "4"; gene_type "
Dt_chr6	Cufflinks	exon	18315779	18315850	.	-	.	gene_id "XLOC_202542"; transcript_id "Novel_002739"; exon_number "5"; gene_type "
Dt_chr6	Cufflinks	exon	18315995	18316401	.	-	.	gene_id "XLOC_202542"; transcript_id "Novel_002739"; exon_number "6"; gene_type "
Dt_chr6	Cufflinks	exon	21926471	21928364	.	-	.	gene_id "XLOC_202876"; transcript_id "Novel_002740"; exon_number "1"; gene_type "
Dt_chr6	Cufflinks	exon	21929185	21930151	.	-	.	gene_id "XLOC_202876"; transcript_id "Novel_002740"; exon_number "2"; gene_type "
Dt_chr6	Cufflinks	exon	23995917	23996176	.	-	.	gene_id "XLOC_203032"; transcript_id "Novel_002741"; exon_number "1"; gene_type "
Dt_chr6	Cufflinks	exon	24150017	24150403	.	-	.	gene_id "XLOC_203051"; transcript_id "Novel_002742"; exon_number "1"; gene_type "
Dt_chr6	Cufflinks	exon	24151864	24152728	.	-	.	gene_id "XLOC_203051"; transcript_id "Novel_002742"; exon_number "2"; gene_type "
Dt_chr6	Cufflinks	exon	24152957	24153152	.	-	.	gene_id "XLOC_203051"; transcript_id "Novel_002742"; exon_number "3"; gene_type "
Dt_chr6	Cufflinks	exon	24154004	24154472	.	-	.	gene_id "XLOC_203051"; transcript_id "Novel_002742"; exon_number "4"; gene_type "
Dt_chr6	Cufflinks	exon	26188190	26188663	.	-	.	gene_id "XLOC_203247"; transcript_id "Novel_002743"; exon_number "1"; gene_type "
Dt_chr6	Cufflinks	exon	26189020	26189127	.	-	.	gene_id "XLOC_203247"; transcript_id "Novel_002743"; exon_number "2"; gene_type "
Dt_chr6	Cufflinks	exon	26190585	26190629	.	-	.	gene_id "XLOC_203247"; transcript_id "Novel_002743"; exon_number "3"; gene_type "
Dt_chr6	Cufflinks	exon	26190789	26191058	.	-	.	gene_id "XLOC_203247"; transcript_id "Novel_002743"; exon_number "4"; gene_type "
Dt_chr6	Cufflinks	exon	30382541	30383142	.	-	.	gene_id "XLOC_203465"; transcript_id "Novel_002744"; exon_number "1"; gene_type "

Dt_chr6	Cufflinks	exon	30384421	30384561	.	-	.	gene_id "XLOC_203465"; transcript_id "Novel_002744"; exon_number "2"; gene_type "
Dt_chr6	Cufflinks	exon	30384678	30384809	.	-	.	gene_id "XLOC_203465"; transcript_id "Novel_002744"; exon_number "3"; gene_type "
Dt_chr6	Cufflinks	exon	30384913	30385164	.	-	.	gene_id "XLOC_203465"; transcript_id "Novel_002744"; exon_number "4"; gene_type "
Dt_chr6	Cufflinks	exon	30383087	30384561	.	-	.	gene_id "XLOC_203465"; transcript_id "Novel_002745"; exon_number "1"; gene_type "
Dt_chr6	Cufflinks	exon	30384678	30384813	.	-	.	gene_id "XLOC_203465"; transcript_id "Novel_002745"; exon_number "2"; gene_type "
Dt_chr6	Cufflinks	exon	30384913	30385158	.	-	.	gene_id "XLOC_203465"; transcript_id "Novel_002745"; exon_number "3"; gene_type "
Dt_chr6	Cufflinks	exon	30526574	30530027	.	-	.	gene_id "XLOC_203479"; transcript_id "Novel_002746"; exon_number "1"; gene_type "
Dt_chr6	Cufflinks	exon	30530163	30530346	.	-	.	gene_id "XLOC_203479"; transcript_id "Novel_002746"; exon_number "2"; gene_type "
Dt_chr6	Cufflinks	exon	30526592	30529659	.	-	.	gene_id "XLOC_203479"; transcript_id "Novel_002747"; exon_number "1"; gene_type "
Dt_chr6	Cufflinks	exon	30529756	30530027	.	-	.	gene_id "XLOC_203479"; transcript_id "Novel_002747"; exon_number "2"; gene_type "
Dt_chr6	Cufflinks	exon	30530163	30530265	.	-	.	gene_id "XLOC_203479"; transcript_id "Novel_002747"; exon_number "3"; gene_type "
Dt_chr6	Cufflinks	exon	30526660	30526980	.	-	.	gene_id "XLOC_203479"; transcript_id "Novel_002748"; exon_number "1"; gene_type "
Dt_chr6	Cufflinks	exon	30529279	30529344	.	-	.	gene_id "XLOC_203479"; transcript_id "Novel_002748"; exon_number "2"; gene_type "
Dt_chr6	Cufflinks	exon	30529429	30530027	.	-	.	gene_id "XLOC_203479"; transcript_id "Novel_002748"; exon_number "3"; gene_type "
Dt_chr6	Cufflinks	exon	30530163	30530346	.	-	.	gene_id "XLOC_203479"; transcript_id "Novel_002748"; exon_number "4"; gene_type "
Dt_chr6	Cufflinks	exon	32302798	32303345	.	-	.	gene_id "XLOC_203560"; transcript_id "Novel_002749"; exon_number "1"; gene_type "
Dt_chr6	Cufflinks	exon	32303751	32305278	.	-	.	gene_id "XLOC_203560"; transcript_id "Novel_002749"; exon_number "2"; gene_type "
Dt_chr6	Cufflinks	exon	32305747	32305826	.	-	.	gene_id "XLOC_203560"; transcript_id "Novel_002749"; exon_number "3"; gene_type "
Dt_chr6	Cufflinks	exon	32306873	32307013	.	-	.	gene_id "XLOC_203560"; transcript_id "Novel_002749"; exon_number "4"; gene_type "
Dt_chr6	Cufflinks	exon	32302865	32303345	.	-	.	gene_id "XLOC_203560"; transcript_id "Novel_002750"; exon_number "1"; gene_type "
Dt_chr6	Cufflinks	exon	32303751	32303875	.	-	.	gene_id "XLOC_203560"; transcript_id "Novel_002750"; exon_number "2"; gene_type "
Dt_chr6	Cufflinks	exon	32305212	32305278	.	-	.	gene_id "XLOC_203560"; transcript_id "Novel_002750"; exon_number "3"; gene_type "
Dt_chr6	Cufflinks	exon	32305747	32307001	.	-	.	gene_id "XLOC_203560"; transcript_id "Novel_002750"; exon_number "4"; gene_type "
Dt_chr6	Cufflinks	exon	32602019	32602301	.	-	.	gene_id "XLOC_203569"; transcript_id "Novel_002751"; exon_number "1"; gene_type "
Dt_chr6	Cufflinks	exon	32602629	32602899	.	-	.	gene_id "XLOC_203569"; transcript_id "Novel_002751"; exon_number "2"; gene_type "
Dt_chr6	Cufflinks	exon	33571730	33571983	.	-	.	gene_id "XLOC_203640"; transcript_id "Novel_002752"; exon_number "1"; gene_type "
Dt_chr6	Cufflinks	exon	33572231	33572293	.	-	.	gene_id "XLOC_203640"; transcript_id "Novel_002752"; exon_number "2"; gene_type "
Dt_chr6	Cufflinks	exon	33572417	33572509	.	-	.	gene_id "XLOC_203640"; transcript_id "Novel_002752"; exon_number "3"; gene_type "
Dt_chr6	Cufflinks	exon	33573210	33573267	.	-	.	gene_id "XLOC_203640"; transcript_id "Novel_002752"; exon_number "4"; gene_type "
Dt_chr6	Cufflinks	exon	33573884	33575299	.	-	.	gene_id "XLOC_203640"; transcript_id "Novel_002752"; exon_number "5"; gene_type "
Dt_chr6	Cufflinks	exon	35854915	35855220	.	-	.	gene_id "XLOC_203750"; transcript_id "Novel_002753"; exon_number "1"; gene_type "
Dt_chr6	Cufflinks	exon	35856043	35856207	.	-	.	gene_id "XLOC_203750"; transcript_id "Novel_002753"; exon_number "2"; gene_type "
Dt_chr6	Cufflinks	exon	36800928	36801164	.	-	.	gene_id "XLOC_203822"; transcript_id "Novel_002754"; exon_number "1"; gene_type "
Dt_chr6	Cufflinks	exon	37716998	37717242	.	-	.	gene_id "XLOC_203890"; transcript_id "Novel_002755"; exon_number "1"; gene_type "
Dt_chr6	Cufflinks	exon	38025655	38028647	.	-	.	gene_id "XLOC_203902"; transcript_id "Novel_002756"; exon_number "1"; gene_type "

Dt_chr6	Cufflinks	exon	38029185	38029337	.	-	.	gene_id "XLOC_203902"; transcript_id "Novel_002756"; exon_number "2"; gene_type "
Dt_chr6	Cufflinks	exon	38026113	38028629	.	-	.	gene_id "XLOC_203902"; transcript_id "Novel_002757"; exon_number "1"; gene_type "
Dt_chr6	Cufflinks	exon	38029185	38029339	.	-	.	gene_id "XLOC_203902"; transcript_id "Novel_002757"; exon_number "2"; gene_type "
Dt_chr6	Cufflinks	exon	38412374	38412932	.	-	.	gene_id "XLOC_203925"; transcript_id "Novel_002758"; exon_number "1"; gene_type "
Dt_chr6	Cufflinks	exon	38414148	38414233	.	-	.	gene_id "XLOC_203925"; transcript_id "Novel_002758"; exon_number "2"; gene_type "
Dt_chr6	Cufflinks	exon	39975192	39975544	.	-	.	gene_id "XLOC_204017"; transcript_id "Novel_002759"; exon_number "1"; gene_type "
Dt_chr6	Cufflinks	exon	40800177	40804704	.	-	.	gene_id "XLOC_204074"; transcript_id "Novel_002760"; exon_number "1"; gene_type "
Dt_chr6	Cufflinks	exon	40804827	40804997	.	-	.	gene_id "XLOC_204074"; transcript_id "Novel_002760"; exon_number "2"; gene_type "
Dt_chr6	Cufflinks	exon	40969703	40970015	.	-	.	gene_id "XLOC_204088"; transcript_id "Novel_002761"; exon_number "1"; gene_type "
Dt_chr6	Cufflinks	exon	40970214	40970315	.	-	.	gene_id "XLOC_204088"; transcript_id "Novel_002761"; exon_number "2"; gene_type "
Dt_chr6	Cufflinks	exon	40970496	40970630	.	-	.	gene_id "XLOC_204088"; transcript_id "Novel_002761"; exon_number "3"; gene_type "
Dt_chr6	Cufflinks	exon	41742386	41742575	.	-	.	gene_id "XLOC_204199"; transcript_id "Novel_002762"; exon_number "1"; gene_type "
Dt_chr6	Cufflinks	exon	41743101	41743176	.	-	.	gene_id "XLOC_204199"; transcript_id "Novel_002762"; exon_number "2"; gene_type "
Dt_chr6	Cufflinks	exon	41743275	41743514	.	-	.	gene_id "XLOC_204199"; transcript_id "Novel_002762"; exon_number "3"; gene_type "
Dt_chr6	Cufflinks	exon	41783125	41783506	.	-	.	gene_id "XLOC_204201"; transcript_id "Novel_002763"; exon_number "1"; gene_type "
Dt_chr6	Cufflinks	exon	41783624	41783721	.	-	.	gene_id "XLOC_204201"; transcript_id "Novel_002763"; exon_number "2"; gene_type "
Dt_chr7	Cufflinks	exon	658766	658895	.	+	.	gene_id "XLOC_204586"; transcript_id "Novel_002764"; exon_number "1"; gene_type "
Dt_chr7	Cufflinks	exon	659024	659078	.	+	.	gene_id "XLOC_204586"; transcript_id "Novel_002764"; exon_number "2"; gene_type "
Dt_chr7	Cufflinks	exon	659227	659524	.	+	.	gene_id "XLOC_204586"; transcript_id "Novel_002764"; exon_number "3"; gene_type "
Dt_chr7	Cufflinks	exon	1683986	1684810	.	+	.	gene_id "XLOC_204654"; transcript_id "Novel_002765"; exon_number "1"; gene_type "
Dt_chr7	Cufflinks	exon	1684885	1685052	.	+	.	gene_id "XLOC_204654"; transcript_id "Novel_002765"; exon_number "2"; gene_type "
Dt_chr7	Cufflinks	exon	1685169	1685375	.	+	.	gene_id "XLOC_204654"; transcript_id "Novel_002765"; exon_number "3"; gene_type "
Dt_chr7	Cufflinks	exon	1685477	1686284	.	+	.	gene_id "XLOC_204654"; transcript_id "Novel_002765"; exon_number "4"; gene_type "
Dt_chr7	Cufflinks	exon	1686979	1687374	.	+	.	gene_id "XLOC_204655"; transcript_id "Novel_002766"; exon_number "1"; gene_type "
Dt_chr7	Cufflinks	exon	1687460	1687941	.	+	.	gene_id "XLOC_204655"; transcript_id "Novel_002766"; exon_number "2"; gene_type "
Dt_chr7	Cufflinks	exon	2549083	2549977	.	+	.	gene_id "XLOC_204798"; transcript_id "Novel_002767"; exon_number "1"; gene_type "
Dt_chr7	Cufflinks	exon	2550150	2550248	.	+	.	gene_id "XLOC_204798"; transcript_id "Novel_002767"; exon_number "2"; gene_type "
Dt_chr7	Cufflinks	exon	2550389	2550505	.	+	.	gene_id "XLOC_204798"; transcript_id "Novel_002767"; exon_number "3"; gene_type "
Dt_chr7	Cufflinks	exon	2550612	2550672	.	+	.	gene_id "XLOC_204798"; transcript_id "Novel_002767"; exon_number "4"; gene_type "
Dt_chr7	Cufflinks	exon	2551157	2551281	.	+	.	gene_id "XLOC_204798"; transcript_id "Novel_002767"; exon_number "5"; gene_type "
Dt_chr7	Cufflinks	exon	2551688	2552062	.	+	.	gene_id "XLOC_204798"; transcript_id "Novel_002767"; exon_number "6"; gene_type "
Dt_chr7	Cufflinks	exon	2754319	2754798	.	+	.	gene_id "XLOC_204811"; transcript_id "Novel_002768"; exon_number "1"; gene_type "
Dt_chr7	Cufflinks	exon	2754997	2755438	.	+	.	gene_id "XLOC_204811"; transcript_id "Novel_002768"; exon_number "2"; gene_type "
Dt_chr7	Cufflinks	exon	2946875	2947130	.	+	.	gene_id "XLOC_204849"; transcript_id "Novel_002769"; exon_number "1"; gene_type "
Dt_chr7	Cufflinks	exon	2947899	2948039	.	+	.	gene_id "XLOC_204849"; transcript_id "Novel_002769"; exon_number "2"; gene_type "

Dt_chr7	Cufflinks	exon	2948619	2948751	.	+	.	gene_id "XLOC_204849"; transcript_id "Novel_002769"; exon_number "3"; gene_type "
Dt_chr7	Cufflinks	exon	2948916	2948987	.	+	.	gene_id "XLOC_204849"; transcript_id "Novel_002769"; exon_number "4"; gene_type "
Dt_chr7	Cufflinks	exon	2949084	2949381	.	+	.	gene_id "XLOC_204849"; transcript_id "Novel_002769"; exon_number "5"; gene_type "
Dt_chr7	Cufflinks	exon	2946880	2947130	.	+	.	gene_id "XLOC_204849"; transcript_id "Novel_002770"; exon_number "1"; gene_type "
Dt_chr7	Cufflinks	exon	2947899	2948039	.	+	.	gene_id "XLOC_204849"; transcript_id "Novel_002770"; exon_number "2"; gene_type "
Dt_chr7	Cufflinks	exon	2948619	2948751	.	+	.	gene_id "XLOC_204849"; transcript_id "Novel_002770"; exon_number "3"; gene_type "
Dt_chr7	Cufflinks	exon	2948916	2948987	.	+	.	gene_id "XLOC_204849"; transcript_id "Novel_002770"; exon_number "4"; gene_type "
Dt_chr7	Cufflinks	exon	2949084	2949227	.	+	.	gene_id "XLOC_204849"; transcript_id "Novel_002770"; exon_number "5"; gene_type "
Dt_chr7	Cufflinks	exon	2949325	2949396	.	+	.	gene_id "XLOC_204849"; transcript_id "Novel_002770"; exon_number "6"; gene_type "
Dt_chr7	Cufflinks	exon	2949561	2949626	.	+	.	gene_id "XLOC_204849"; transcript_id "Novel_002770"; exon_number "7"; gene_type "
Dt_chr7	Cufflinks	exon	2949767	2949837	.	+	.	gene_id "XLOC_204849"; transcript_id "Novel_002770"; exon_number "8"; gene_type "
Dt_chr7	Cufflinks	exon	2949966	2950106	.	+	.	gene_id "XLOC_204849"; transcript_id "Novel_002770"; exon_number "9"; gene_type "
Dt_chr7	Cufflinks	exon	2950236	2950355	.	+	.	gene_id "XLOC_204849"; transcript_id "Novel_002770"; exon_number "10"; gene_type
Dt_chr7	Cufflinks	exon	2946880	2947130	.	+	.	gene_id "XLOC_204849"; transcript_id "Novel_002771"; exon_number "1"; gene_type "
Dt_chr7	Cufflinks	exon	2947899	2948039	.	+	.	gene_id "XLOC_204849"; transcript_id "Novel_002771"; exon_number "2"; gene_type "
Dt_chr7	Cufflinks	exon	2948619	2948751	.	+	.	gene_id "XLOC_204849"; transcript_id "Novel_002771"; exon_number "3"; gene_type "
Dt_chr7	Cufflinks	exon	2948916	2948987	.	+	.	gene_id "XLOC_204849"; transcript_id "Novel_002771"; exon_number "4"; gene_type "
Dt_chr7	Cufflinks	exon	2949084	2949227	.	+	.	gene_id "XLOC_204849"; transcript_id "Novel_002771"; exon_number "5"; gene_type "
Dt_chr7	Cufflinks	exon	2949325	2949396	.	+	.	gene_id "XLOC_204849"; transcript_id "Novel_002771"; exon_number "6"; gene_type "
Dt_chr7	Cufflinks	exon	2949561	2949626	.	+	.	gene_id "XLOC_204849"; transcript_id "Novel_002771"; exon_number "7"; gene_type "
Dt_chr7	Cufflinks	exon	2949767	2950106	.	+	.	gene_id "XLOC_204849"; transcript_id "Novel_002771"; exon_number "8"; gene_type "
Dt_chr7	Cufflinks	exon	2950236	2950355	.	+	.	gene_id "XLOC_204849"; transcript_id "Novel_002771"; exon_number "9"; gene_type "
Dt_chr7	Cufflinks	exon	2950450	2951185	.	+	.	gene_id "XLOC_204851"; transcript_id "Novel_002772"; exon_number "1"; gene_type "
Dt_chr7	Cufflinks	exon	2951287	2952089	.	+	.	gene_id "XLOC_204851"; transcript_id "Novel_002772"; exon_number "2"; gene_type "
Dt_chr7	Cufflinks	exon	3190500	3191071	.	+	.	gene_id "XLOC_204897"; transcript_id "Novel_002773"; exon_number "1"; gene_type "
Dt_chr7	Cufflinks	exon	3191160	3191245	.	+	.	gene_id "XLOC_204897"; transcript_id "Novel_002773"; exon_number "2"; gene_type "
Dt_chr7	Cufflinks	exon	3191361	3191467	.	+	.	gene_id "XLOC_204897"; transcript_id "Novel_002773"; exon_number "3"; gene_type "
Dt_chr7	Cufflinks	exon	3191572	3191727	.	+	.	gene_id "XLOC_204897"; transcript_id "Novel_002773"; exon_number "4"; gene_type "
Dt_chr7	Cufflinks	exon	3191938	3192024	.	+	.	gene_id "XLOC_204897"; transcript_id "Novel_002773"; exon_number "5"; gene_type "
Dt_chr7	Cufflinks	exon	3192266	3192363	.	+	.	gene_id "XLOC_204897"; transcript_id "Novel_002773"; exon_number "6"; gene_type "
Dt_chr7	Cufflinks	exon	3193574	3193661	.	+	.	gene_id "XLOC_204897"; transcript_id "Novel_002773"; exon_number "7"; gene_type "
Dt_chr7	Cufflinks	exon	3194419	3194744	.	+	.	gene_id "XLOC_204897"; transcript_id "Novel_002773"; exon_number "8"; gene_type "
Dt_chr7	Cufflinks	exon	3190500	3190883	.	+	.	gene_id "XLOC_204897"; transcript_id "Novel_002774"; exon_number "1"; gene_type "
Dt_chr7	Cufflinks	exon	3190997	3191071	.	+	.	gene_id "XLOC_204897"; transcript_id "Novel_002774"; exon_number "2"; gene_type "
Dt_chr7	Cufflinks	exon	3191160	3191245	.	+	.	gene_id "XLOC_204897"; transcript_id "Novel_002774"; exon_number "3"; gene_type "

Dt_chr7	Cufflinks	exon	3191361	3191467	.	+	.	gene_id "XLOC_204897"; transcript_id "Novel_002774"; exon_number "4"; gene_type "
Dt_chr7	Cufflinks	exon	3191572	3191727	.	+	.	gene_id "XLOC_204897"; transcript_id "Novel_002774"; exon_number "5"; gene_type "
Dt_chr7	Cufflinks	exon	3191938	3192024	.	+	.	gene_id "XLOC_204897"; transcript_id "Novel_002774"; exon_number "6"; gene_type "
Dt_chr7	Cufflinks	exon	3192266	3192363	.	+	.	gene_id "XLOC_204897"; transcript_id "Novel_002774"; exon_number "7"; gene_type "
Dt_chr7	Cufflinks	exon	3193574	3193661	.	+	.	gene_id "XLOC_204897"; transcript_id "Novel_002774"; exon_number "8"; gene_type "
Dt_chr7	Cufflinks	exon	3194419	3194744	.	+	.	gene_id "XLOC_204897"; transcript_id "Novel_002774"; exon_number "9"; gene_type "
Dt_chr7	Cufflinks	exon	3190562	3191071	.	+	.	gene_id "XLOC_204897"; transcript_id "Novel_002775"; exon_number "1"; gene_type "
Dt_chr7	Cufflinks	exon	3191160	3191245	.	+	.	gene_id "XLOC_204897"; transcript_id "Novel_002775"; exon_number "2"; gene_type "
Dt_chr7	Cufflinks	exon	3191361	3191594	.	+	.	gene_id "XLOC_204897"; transcript_id "Novel_002775"; exon_number "3"; gene_type "
Dt_chr7	Cufflinks	exon	3191783	3192024	.	+	.	gene_id "XLOC_204897"; transcript_id "Novel_002776"; exon_number "1"; gene_type "
Dt_chr7	Cufflinks	exon	3192266	3192363	.	+	.	gene_id "XLOC_204897"; transcript_id "Novel_002776"; exon_number "2"; gene_type "
Dt_chr7	Cufflinks	exon	3193574	3193661	.	+	.	gene_id "XLOC_204897"; transcript_id "Novel_002776"; exon_number "3"; gene_type "
Dt_chr7	Cufflinks	exon	3194419	3194920	.	+	.	gene_id "XLOC_204897"; transcript_id "Novel_002776"; exon_number "4"; gene_type "
Dt_chr7	Cufflinks	exon	3191984	3192363	.	+	.	gene_id "XLOC_204897"; transcript_id "Novel_002777"; exon_number "1"; gene_type "
Dt_chr7	Cufflinks	exon	3193574	3193661	.	+	.	gene_id "XLOC_204897"; transcript_id "Novel_002777"; exon_number "2"; gene_type "
Dt_chr7	Cufflinks	exon	3194419	3194850	.	+	.	gene_id "XLOC_204897"; transcript_id "Novel_002777"; exon_number "3"; gene_type "
Dt_chr7	Cufflinks	exon	3416686	3417375	.	+	.	gene_id "XLOC_204956"; transcript_id "Novel_002778"; exon_number "1"; gene_type "
Dt_chr7	Cufflinks	exon	3417685	3417802	.	+	.	gene_id "XLOC_204956"; transcript_id "Novel_002778"; exon_number "2"; gene_type "
Dt_chr7	Cufflinks	exon	3418094	3418314	.	+	.	gene_id "XLOC_204956"; transcript_id "Novel_002778"; exon_number "3"; gene_type "
Dt_chr7	Cufflinks	exon	3418917	3419141	.	+	.	gene_id "XLOC_204956"; transcript_id "Novel_002778"; exon_number "4"; gene_type "
Dt_chr7	Cufflinks	exon	3419242	3419380	.	+	.	gene_id "XLOC_204956"; transcript_id "Novel_002778"; exon_number "5"; gene_type "
Dt_chr7	Cufflinks	exon	3419482	3420288	.	+	.	gene_id "XLOC_204956"; transcript_id "Novel_002778"; exon_number "6"; gene_type "
Dt_chr7	Cufflinks	exon	3416691	3417375	.	+	.	gene_id "XLOC_204956"; transcript_id "Novel_002779"; exon_number "1"; gene_type "
Dt_chr7	Cufflinks	exon	3417685	3417802	.	+	.	gene_id "XLOC_204956"; transcript_id "Novel_002779"; exon_number "2"; gene_type "
Dt_chr7	Cufflinks	exon	3418094	3418314	.	+	.	gene_id "XLOC_204956"; transcript_id "Novel_002779"; exon_number "3"; gene_type "
Dt_chr7	Cufflinks	exon	3418917	3419368	.	+	.	gene_id "XLOC_204956"; transcript_id "Novel_002779"; exon_number "4"; gene_type "
Dt_chr7	Cufflinks	exon	3524532	3524585	.	+	.	gene_id "XLOC_204984"; transcript_id "Novel_002780"; exon_number "1"; gene_type "
Dt_chr7	Cufflinks	exon	3525612	3526625	.	+	.	gene_id "XLOC_204984"; transcript_id "Novel_002780"; exon_number "2"; gene_type "
Dt_chr7	Cufflinks	exon	3602776	3602993	.	+	.	gene_id "XLOC_205010"; transcript_id "Novel_002781"; exon_number "1"; gene_type "
Dt_chr7	Cufflinks	exon	3603423	3604272	.	+	.	gene_id "XLOC_205010"; transcript_id "Novel_002781"; exon_number "2"; gene_type "
Dt_chr7	Cufflinks	exon	3604371	3604540	.	+	.	gene_id "XLOC_205010"; transcript_id "Novel_002781"; exon_number "3"; gene_type "
Dt_chr7	Cufflinks	exon	3604792	3604848	.	+	.	gene_id "XLOC_205010"; transcript_id "Novel_002781"; exon_number "4"; gene_type "
Dt_chr7	Cufflinks	exon	3602808	3602993	.	+	.	gene_id "XLOC_205010"; transcript_id "Novel_002782"; exon_number "1"; gene_type "
Dt_chr7	Cufflinks	exon	3603423	3604272	.	+	.	gene_id "XLOC_205010"; transcript_id "Novel_002782"; exon_number "2"; gene_type "
Dt_chr7	Cufflinks	exon	3604371	3604540	.	+	.	gene_id "XLOC_205010"; transcript_id "Novel_002782"; exon_number "3"; gene_type "

Dt_chr7	Cufflinks	exon	3604772	3604860	.	+	.	gene_id "XLOC_205010"; transcript_id "Novel_002782"; exon_number "4"; gene_type "
Dt_chr7	Cufflinks	exon	3602876	3602993	.	+	.	gene_id "XLOC_205010"; transcript_id "Novel_002783"; exon_number "1"; gene_type "
Dt_chr7	Cufflinks	exon	3603426	3604272	.	+	.	gene_id "XLOC_205010"; transcript_id "Novel_002783"; exon_number "2"; gene_type "
Dt_chr7	Cufflinks	exon	3604371	3604540	.	+	.	gene_id "XLOC_205010"; transcript_id "Novel_002783"; exon_number "3"; gene_type "
Dt_chr7	Cufflinks	exon	3604792	3604877	.	+	.	gene_id "XLOC_205010"; transcript_id "Novel_002783"; exon_number "4"; gene_type "
Dt_chr7	Cufflinks	exon	3605016	3605065	.	+	.	gene_id "XLOC_205010"; transcript_id "Novel_002783"; exon_number "5"; gene_type "
Dt_chr7	Cufflinks	exon	3605338	3605405	.	+	.	gene_id "XLOC_205010"; transcript_id "Novel_002783"; exon_number "6"; gene_type "
Dt_chr7	Cufflinks	exon	3605994	3606078	.	+	.	gene_id "XLOC_205010"; transcript_id "Novel_002783"; exon_number "7"; gene_type "
Dt_chr7	Cufflinks	exon	3780894	3781259	.	+	.	gene_id "XLOC_205045"; transcript_id "Novel_002784"; exon_number "1"; gene_type "
Dt_chr7	Cufflinks	exon	3782149	3783314	.	+	.	gene_id "XLOC_205045"; transcript_id "Novel_002784"; exon_number "2"; gene_type "
Dt_chr7	Cufflinks	exon	3930351	3930515	.	+	.	gene_id "XLOC_205069"; transcript_id "Novel_002785"; exon_number "1"; gene_type "
Dt_chr7	Cufflinks	exon	3931170	3931750	.	+	.	gene_id "XLOC_205069"; transcript_id "Novel_002785"; exon_number "2"; gene_type "
Dt_chr7	Cufflinks	exon	3931934	3932034	.	+	.	gene_id "XLOC_205069"; transcript_id "Novel_002785"; exon_number "3"; gene_type "
Dt_chr7	Cufflinks	exon	3932428	3932511	.	+	.	gene_id "XLOC_205069"; transcript_id "Novel_002785"; exon_number "4"; gene_type "
Dt_chr7	Cufflinks	exon	3932611	3932721	.	+	.	gene_id "XLOC_205069"; transcript_id "Novel_002785"; exon_number "5"; gene_type "
Dt_chr7	Cufflinks	exon	3932840	3933203	.	+	.	gene_id "XLOC_205069"; transcript_id "Novel_002785"; exon_number "6"; gene_type "
Dt_chr7	Cufflinks	exon	3933979	3934283	.	+	.	gene_id "XLOC_205072"; transcript_id "Novel_002786"; exon_number "1"; gene_type "
Dt_chr7	Cufflinks	exon	3934364	3934421	.	+	.	gene_id "XLOC_205072"; transcript_id "Novel_002786"; exon_number "2"; gene_type "
Dt_chr7	Cufflinks	exon	3934745	3934812	.	+	.	gene_id "XLOC_205072"; transcript_id "Novel_002786"; exon_number "3"; gene_type "
Dt_chr7	Cufflinks	exon	3934912	3935091	.	+	.	gene_id "XLOC_205072"; transcript_id "Novel_002786"; exon_number "4"; gene_type "
Dt_chr7	Cufflinks	exon	3935316	3935412	.	+	.	gene_id "XLOC_205072"; transcript_id "Novel_002786"; exon_number "5"; gene_type "
Dt_chr7	Cufflinks	exon	3936155	3936264	.	+	.	gene_id "XLOC_205072"; transcript_id "Novel_002786"; exon_number "6"; gene_type "
Dt_chr7	Cufflinks	exon	3936355	3936488	.	+	.	gene_id "XLOC_205072"; transcript_id "Novel_002786"; exon_number "7"; gene_type "
Dt_chr7	Cufflinks	exon	3936570	3936751	.	+	.	gene_id "XLOC_205072"; transcript_id "Novel_002786"; exon_number "8"; gene_type "
Dt_chr7	Cufflinks	exon	3937011	3937442	.	+	.	gene_id "XLOC_205072"; transcript_id "Novel_002786"; exon_number "9"; gene_type "
Dt_chr7	Cufflinks	exon	3934287	3934421	.	+	.	gene_id "XLOC_205072"; transcript_id "Novel_002787"; exon_number "1"; gene_type "
Dt_chr7	Cufflinks	exon	3934745	3934812	.	+	.	gene_id "XLOC_205072"; transcript_id "Novel_002787"; exon_number "2"; gene_type "
Dt_chr7	Cufflinks	exon	3934912	3935091	.	+	.	gene_id "XLOC_205072"; transcript_id "Novel_002787"; exon_number "3"; gene_type "
Dt_chr7	Cufflinks	exon	3935312	3935395	.	+	.	gene_id "XLOC_205072"; transcript_id "Novel_002787"; exon_number "4"; gene_type "
Dt_chr7	Cufflinks	exon	3935171	3935412	.	+	.	gene_id "XLOC_205072"; transcript_id "Novel_002788"; exon_number "1"; gene_type "
Dt_chr7	Cufflinks	exon	3936155	3936264	.	+	.	gene_id "XLOC_205072"; transcript_id "Novel_002788"; exon_number "2"; gene_type "
Dt_chr7	Cufflinks	exon	3936355	3936488	.	+	.	gene_id "XLOC_205072"; transcript_id "Novel_002788"; exon_number "3"; gene_type "
Dt_chr7	Cufflinks	exon	3936570	3936751	.	+	.	gene_id "XLOC_205072"; transcript_id "Novel_002788"; exon_number "4"; gene_type "
Dt_chr7	Cufflinks	exon	3937011	3937307	.	+	.	gene_id "XLOC_205072"; transcript_id "Novel_002788"; exon_number "5"; gene_type "
Dt_chr7	Cufflinks	exon	4149028	4149829	.	+	.	gene_id "XLOC_205115"; transcript_id "Novel_002789"; exon_number "1"; gene_type "

Dt_chr7	Cufflinks	exon	4149930	4151533	.	+	.	gene_id "XLOC_205115"; transcript_id "Novel_002789"; exon_number "2"; gene_type "
Dt_chr7	Cufflinks	exon	4210559	4211534	.	+	.	gene_id "XLOC_205129"; transcript_id "Novel_002790"; exon_number "1"; gene_type "
Dt_chr7	Cufflinks	exon	4253961	4254260	.	+	.	gene_id "XLOC_205135"; transcript_id "Novel_002791"; exon_number "1"; gene_type "
Dt_chr7	Cufflinks	exon	4254364	4254421	.	+	.	gene_id "XLOC_205135"; transcript_id "Novel_002791"; exon_number "2"; gene_type "
Dt_chr7	Cufflinks	exon	4254516	4254620	.	+	.	gene_id "XLOC_205135"; transcript_id "Novel_002791"; exon_number "3"; gene_type "
Dt_chr7	Cufflinks	exon	4255262	4255319	.	+	.	gene_id "XLOC_205135"; transcript_id "Novel_002791"; exon_number "4"; gene_type "
Dt_chr7	Cufflinks	exon	4255415	4255590	.	+	.	gene_id "XLOC_205135"; transcript_id "Novel_002791"; exon_number "5"; gene_type "
Dt_chr7	Cufflinks	exon	4255716	4255776	.	+	.	gene_id "XLOC_205135"; transcript_id "Novel_002791"; exon_number "6"; gene_type "
Dt_chr7	Cufflinks	exon	4256139	4256906	.	+	.	gene_id "XLOC_205135"; transcript_id "Novel_002791"; exon_number "7"; gene_type "
Dt_chr7	Cufflinks	exon	4253998	4254260	.	+	.	gene_id "XLOC_205135"; transcript_id "Novel_002792"; exon_number "1"; gene_type "
Dt_chr7	Cufflinks	exon	4254364	4254421	.	+	.	gene_id "XLOC_205135"; transcript_id "Novel_002792"; exon_number "2"; gene_type "
Dt_chr7	Cufflinks	exon	4254516	4255359	.	+	.	gene_id "XLOC_205135"; transcript_id "Novel_002792"; exon_number "3"; gene_type "
Dt_chr7	Cufflinks	exon	4269509	4269672	.	+	.	gene_id "XLOC_205137"; transcript_id "Novel_002793"; exon_number "1"; gene_type "
Dt_chr7	Cufflinks	exon	4269786	4269959	.	+	.	gene_id "XLOC_205137"; transcript_id "Novel_002793"; exon_number "2"; gene_type "
Dt_chr7	Cufflinks	exon	4270057	4270327	.	+	.	gene_id "XLOC_205137"; transcript_id "Novel_002793"; exon_number "3"; gene_type "
Dt_chr7	Cufflinks	exon	4270984	4270998	.	+	.	gene_id "XLOC_205137"; transcript_id "Novel_002793"; exon_number "4"; gene_type "
Dt_chr7	Cufflinks	exon	4269645	4269959	.	+	.	gene_id "XLOC_205137"; transcript_id "Novel_002794"; exon_number "1"; gene_type "
Dt_chr7	Cufflinks	exon	4270057	4270327	.	+	.	gene_id "XLOC_205137"; transcript_id "Novel_002794"; exon_number "2"; gene_type "
Dt_chr7	Cufflinks	exon	4270984	4271131	.	+	.	gene_id "XLOC_205137"; transcript_id "Novel_002794"; exon_number "3"; gene_type "
Dt_chr7	Cufflinks	exon	4283378	4283596	.	+	.	gene_id "XLOC_205139"; transcript_id "Novel_002795"; exon_number "1"; gene_type "
Dt_chr7	Cufflinks	exon	4303573	4303576	.	+	.	gene_id "XLOC_205142"; transcript_id "Novel_002796"; exon_number "1"; gene_type "
Dt_chr7	Cufflinks	exon	4303848	4303946	.	+	.	gene_id "XLOC_205142"; transcript_id "Novel_002796"; exon_number "2"; gene_type "
Dt_chr7	Cufflinks	exon	4324105	4324293	.	+	.	gene_id "XLOC_205142"; transcript_id "Novel_002796"; exon_number "3"; gene_type "
Dt_chr7	Cufflinks	exon	4324438	4324516	.	+	.	gene_id "XLOC_205142"; transcript_id "Novel_002796"; exon_number "4"; gene_type "
Dt_chr7	Cufflinks	exon	4324590	4325015	.	+	.	gene_id "XLOC_205142"; transcript_id "Novel_002796"; exon_number "5"; gene_type "
Dt_chr7	Cufflinks	exon	4350156	4350386	.	+	.	gene_id "XLOC_205146"; transcript_id "Novel_002797"; exon_number "1"; gene_type "
Dt_chr7	Cufflinks	exon	4350614	4351221	.	+	.	gene_id "XLOC_205146"; transcript_id "Novel_002797"; exon_number "2"; gene_type "
Dt_chr7	Cufflinks	exon	4351313	4351921	.	+	.	gene_id "XLOC_205146"; transcript_id "Novel_002797"; exon_number "3"; gene_type "
Dt_chr7	Cufflinks	exon	4390547	4391354	.	+	.	gene_id "XLOC_205158"; transcript_id "Novel_002798"; exon_number "1"; gene_type "
Dt_chr7	Cufflinks	exon	4537989	4538418	.	+	.	gene_id "XLOC_205183"; transcript_id "Novel_002799"; exon_number "1"; gene_type "
Dt_chr7	Cufflinks	exon	4538543	4539145	.	+	.	gene_id "XLOC_205183"; transcript_id "Novel_002799"; exon_number "2"; gene_type "
Dt_chr7	Cufflinks	exon	4539227	4539369	.	+	.	gene_id "XLOC_205183"; transcript_id "Novel_002799"; exon_number "3"; gene_type "
Dt_chr7	Cufflinks	exon	4539703	4540319	.	+	.	gene_id "XLOC_205183"; transcript_id "Novel_002799"; exon_number "4"; gene_type "
Dt_chr7	Cufflinks	exon	4538058	4538418	.	+	.	gene_id "XLOC_205183"; transcript_id "Novel_002800"; exon_number "1"; gene_type "
Dt_chr7	Cufflinks	exon	4538491	4538512	.	+	.	gene_id "XLOC_205183"; transcript_id "Novel_002800"; exon_number "2"; gene_type "

Dt_chr7	Cufflinks	exon	4594558	4595065	.	+	.	gene_id "XLOC_205192"; transcript_id "Novel_002801"; exon_number "1"; gene_type "
Dt_chr7	Cufflinks	exon	4595757	4596905	.	+	.	gene_id "XLOC_205192"; transcript_id "Novel_002801"; exon_number "2"; gene_type "
Dt_chr7	Cufflinks	exon	4604784	4605320	.	+	.	gene_id "XLOC_205192"; transcript_id "Novel_002802"; exon_number "1"; gene_type "
Dt_chr7	Cufflinks	exon	4606005	4607055	.	+	.	gene_id "XLOC_205192"; transcript_id "Novel_002802"; exon_number "2"; gene_type "
Dt_chr7	Cufflinks	exon	4729771	4730405	.	+	.	gene_id "XLOC_205218"; transcript_id "Novel_002803"; exon_number "1"; gene_type "
Dt_chr7	Cufflinks	exon	4731652	4731706	.	+	.	gene_id "XLOC_205218"; transcript_id "Novel_002803"; exon_number "2"; gene_type "
Dt_chr7	Cufflinks	exon	4731823	4731946	.	+	.	gene_id "XLOC_205218"; transcript_id "Novel_002803"; exon_number "3"; gene_type "
Dt_chr7	Cufflinks	exon	4732188	4732284	.	+	.	gene_id "XLOC_205218"; transcript_id "Novel_002803"; exon_number "4"; gene_type "
Dt_chr7	Cufflinks	exon	4732365	4732401	.	+	.	gene_id "XLOC_205218"; transcript_id "Novel_002803"; exon_number "5"; gene_type "
Dt_chr7	Cufflinks	exon	4732492	4732596	.	+	.	gene_id "XLOC_205218"; transcript_id "Novel_002803"; exon_number "6"; gene_type "
Dt_chr7	Cufflinks	exon	4732681	4732738	.	+	.	gene_id "XLOC_205218"; transcript_id "Novel_002803"; exon_number "7"; gene_type "
Dt_chr7	Cufflinks	exon	4733483	4733602	.	+	.	gene_id "XLOC_205218"; transcript_id "Novel_002803"; exon_number "8"; gene_type "
Dt_chr7	Cufflinks	exon	4733918	4733980	.	+	.	gene_id "XLOC_205218"; transcript_id "Novel_002803"; exon_number "9"; gene_type "
Dt_chr7	Cufflinks	exon	4734306	4734424	.	+	.	gene_id "XLOC_205218"; transcript_id "Novel_002803"; exon_number "10"; gene_type
Dt_chr7	Cufflinks	exon	4734520	4734647	.	+	.	gene_id "XLOC_205218"; transcript_id "Novel_002803"; exon_number "11"; gene_type
Dt_chr7	Cufflinks	exon	4734768	4735763	.	+	.	gene_id "XLOC_205218"; transcript_id "Novel_002803"; exon_number "12"; gene_type
Dt_chr7	Cufflinks	exon	4735841	4735971	.	+	.	gene_id "XLOC_205218"; transcript_id "Novel_002803"; exon_number "13"; gene_type
Dt_chr7	Cufflinks	exon	4736437	4736510	.	+	.	gene_id "XLOC_205218"; transcript_id "Novel_002803"; exon_number "14"; gene_type
Dt_chr7	Cufflinks	exon	4736638	4736805	.	+	.	gene_id "XLOC_205218"; transcript_id "Novel_002803"; exon_number "15"; gene_type
Dt_chr7	Cufflinks	exon	4737440	4737568	.	+	.	gene_id "XLOC_205218"; transcript_id "Novel_002803"; exon_number "16"; gene_type
Dt_chr7	Cufflinks	exon	4737650	4737932	.	+	.	gene_id "XLOC_205218"; transcript_id "Novel_002803"; exon_number "17"; gene_type
Dt_chr7	Cufflinks	exon	4738200	4738438	.	+	.	gene_id "XLOC_205218"; transcript_id "Novel_002803"; exon_number "18"; gene_type
Dt_chr7	Cufflinks	exon	4738554	4739672	.	+	.	gene_id "XLOC_205218"; transcript_id "Novel_002803"; exon_number "19"; gene_type
Dt_chr7	Cufflinks	exon	4789816	4790032	.	+	.	gene_id "XLOC_205239"; transcript_id "Novel_002804"; exon_number "1"; gene_type "
Dt_chr7	Cufflinks	exon	4790182	4791021	.	+	.	gene_id "XLOC_205239"; transcript_id "Novel_002804"; exon_number "2"; gene_type "
Dt_chr7	Cufflinks	exon	4831873	4832394	.	+	.	gene_id "XLOC_205245"; transcript_id "Novel_002805"; exon_number "1"; gene_type "
Dt_chr7	Cufflinks	exon	4832475	4833168	.	+	.	gene_id "XLOC_205245"; transcript_id "Novel_002805"; exon_number "2"; gene_type "
Dt_chr7	Cufflinks	exon	4833266	4833477	.	+	.	gene_id "XLOC_205245"; transcript_id "Novel_002805"; exon_number "3"; gene_type "
Dt_chr7	Cufflinks	exon	4840048	4840625	.	+	.	gene_id "XLOC_205245"; transcript_id "Novel_002806"; exon_number "1"; gene_type "
Dt_chr7	Cufflinks	exon	4840999	4841864	.	+	.	gene_id "XLOC_205245"; transcript_id "Novel_002806"; exon_number "2"; gene_type "
Dt_chr7	Cufflinks	exon	4908879	4909405	.	+	.	gene_id "XLOC_205253"; transcript_id "Novel_002807"; exon_number "1"; gene_type "
Dt_chr7	Cufflinks	exon	4910062	4910137	.	+	.	gene_id "XLOC_205253"; transcript_id "Novel_002807"; exon_number "2"; gene_type "
Dt_chr7	Cufflinks	exon	4910472	4910560	.	+	.	gene_id "XLOC_205253"; transcript_id "Novel_002807"; exon_number "3"; gene_type "
Dt_chr7	Cufflinks	exon	4910875	4910964	.	+	.	gene_id "XLOC_205253"; transcript_id "Novel_002807"; exon_number "4"; gene_type "
Dt_chr7	Cufflinks	exon	4911605	4911649	.	+	.	gene_id "XLOC_205253"; transcript_id "Novel_002807"; exon_number "5"; gene_type "

Dt_chr7	Cufflinks	exon	4911737	4911807	.	+	.	gene_id "XLOC_205253"; transcript_id "Novel_002807"; exon_number "6"; gene_type "
Dt_chr7	Cufflinks	exon	4912017	4912155	.	+	.	gene_id "XLOC_205253"; transcript_id "Novel_002807"; exon_number "7"; gene_type "
Dt_chr7	Cufflinks	exon	4912611	4912719	.	+	.	gene_id "XLOC_205253"; transcript_id "Novel_002807"; exon_number "8"; gene_type "
Dt_chr7	Cufflinks	exon	4912944	4913580	.	+	.	gene_id "XLOC_205253"; transcript_id "Novel_002807"; exon_number "9"; gene_type "
Dt_chr7	Cufflinks	exon	4910686	4911649	.	+	.	gene_id "XLOC_205253"; transcript_id "Novel_002808"; exon_number "1"; gene_type "
Dt_chr7	Cufflinks	exon	4911737	4911807	.	+	.	gene_id "XLOC_205253"; transcript_id "Novel_002808"; exon_number "2"; gene_type "
Dt_chr7	Cufflinks	exon	4912017	4912155	.	+	.	gene_id "XLOC_205253"; transcript_id "Novel_002808"; exon_number "3"; gene_type "
Dt_chr7	Cufflinks	exon	4912631	4912719	.	+	.	gene_id "XLOC_205253"; transcript_id "Novel_002808"; exon_number "4"; gene_type "
Dt_chr7	Cufflinks	exon	4912944	4913368	.	+	.	gene_id "XLOC_205253"; transcript_id "Novel_002808"; exon_number "5"; gene_type "
Dt_chr7	Cufflinks	exon	4971854	4972745	.	+	.	gene_id "XLOC_205261"; transcript_id "Novel_002809"; exon_number "1"; gene_type "
Dt_chr7	Cufflinks	exon	4972854	4973894	.	+	.	gene_id "XLOC_205261"; transcript_id "Novel_002809"; exon_number "2"; gene_type "
Dt_chr7	Cufflinks	exon	4971854	4972745	.	+	.	gene_id "XLOC_205261"; transcript_id "Novel_002810"; exon_number "1"; gene_type "
Dt_chr7	Cufflinks	exon	4972854	4972925	.	+	.	gene_id "XLOC_205261"; transcript_id "Novel_002810"; exon_number "2"; gene_type "
Dt_chr7	Cufflinks	exon	4973021	4973059	.	+	.	gene_id "XLOC_205261"; transcript_id "Novel_002810"; exon_number "3"; gene_type "
Dt_chr7	Cufflinks	exon	4974484	4974878	.	+	.	gene_id "XLOC_205261"; transcript_id "Novel_002810"; exon_number "4"; gene_type "
Dt_chr7	Cufflinks	exon	4975017	4975369	.	+	.	gene_id "XLOC_205261"; transcript_id "Novel_002810"; exon_number "5"; gene_type "
Dt_chr7	Cufflinks	exon	4971861	4972745	.	+	.	gene_id "XLOC_205261"; transcript_id "Novel_002811"; exon_number "1"; gene_type "
Dt_chr7	Cufflinks	exon	4972854	4972925	.	+	.	gene_id "XLOC_205261"; transcript_id "Novel_002811"; exon_number "2"; gene_type "
Dt_chr7	Cufflinks	exon	4973021	4975370	.	+	.	gene_id "XLOC_205261"; transcript_id "Novel_002811"; exon_number "3"; gene_type "
Dt_chr7	Cufflinks	exon	5067385	5067495	.	+	.	gene_id "XLOC_205270"; transcript_id "Novel_002812"; exon_number "1"; gene_type "
Dt_chr7	Cufflinks	exon	5067640	5067714	.	+	.	gene_id "XLOC_205270"; transcript_id "Novel_002812"; exon_number "2"; gene_type "
Dt_chr7	Cufflinks	exon	5068631	5068737	.	+	.	gene_id "XLOC_205270"; transcript_id "Novel_002812"; exon_number "3"; gene_type "
Dt_chr7	Cufflinks	exon	5068940	5069207	.	+	.	gene_id "XLOC_205270"; transcript_id "Novel_002812"; exon_number "4"; gene_type "
Dt_chr7	Cufflinks	exon	5069526	5069670	.	+	.	gene_id "XLOC_205270"; transcript_id "Novel_002812"; exon_number "5"; gene_type "
Dt_chr7	Cufflinks	exon	5072178	5072752	.	+	.	gene_id "XLOC_205270"; transcript_id "Novel_002812"; exon_number "6"; gene_type "
Dt_chr7	Cufflinks	exon	5067467	5067495	.	+	.	gene_id "XLOC_205270"; transcript_id "Novel_002813"; exon_number "1"; gene_type "
Dt_chr7	Cufflinks	exon	5067640	5067714	.	+	.	gene_id "XLOC_205270"; transcript_id "Novel_002813"; exon_number "2"; gene_type "
Dt_chr7	Cufflinks	exon	5068631	5068737	.	+	.	gene_id "XLOC_205270"; transcript_id "Novel_002813"; exon_number "3"; gene_type "
Dt_chr7	Cufflinks	exon	5068940	5069207	.	+	.	gene_id "XLOC_205270"; transcript_id "Novel_002813"; exon_number "4"; gene_type "
Dt_chr7	Cufflinks	exon	5069526	5070141	.	+	.	gene_id "XLOC_205270"; transcript_id "Novel_002813"; exon_number "5"; gene_type "
Dt_chr7	Cufflinks	exon	5067666	5067714	.	+	.	gene_id "XLOC_205270"; transcript_id "Novel_002814"; exon_number "1"; gene_type "
Dt_chr7	Cufflinks	exon	5068631	5068737	.	+	.	gene_id "XLOC_205270"; transcript_id "Novel_002814"; exon_number "2"; gene_type "
Dt_chr7	Cufflinks	exon	5068940	5069881	.	+	.	gene_id "XLOC_205270"; transcript_id "Novel_002814"; exon_number "3"; gene_type "
Dt_chr7	Cufflinks	exon	5068307	5068737	.	+	.	gene_id "XLOC_205270"; transcript_id "Novel_002815"; exon_number "1"; gene_type "
Dt_chr7	Cufflinks	exon	5068940	5069207	.	+	.	gene_id "XLOC_205270"; transcript_id "Novel_002815"; exon_number "2"; gene_type "

Dt_chr7	Cufflinks	exon	5069526	5069670	.	+	.	gene_id "XLOC_205270"; transcript_id "Novel_002815"; exon_number "3"; gene_type "
Dt_chr7	Cufflinks	exon	5072178	5072643	.	+	.	gene_id "XLOC_205270"; transcript_id "Novel_002815"; exon_number "4"; gene_type "
Dt_chr7	Cufflinks	exon	5107621	5107654	.	+	.	gene_id "XLOC_205276"; transcript_id "Novel_002816"; exon_number "1"; gene_type "
Dt_chr7	Cufflinks	exon	5107734	5107862	.	+	.	gene_id "XLOC_205276"; transcript_id "Novel_002816"; exon_number "2"; gene_type "
Dt_chr7	Cufflinks	exon	5107995	5108234	.	+	.	gene_id "XLOC_205276"; transcript_id "Novel_002816"; exon_number "3"; gene_type "
Dt_chr7	Cufflinks	exon	5108312	5108444	.	+	.	gene_id "XLOC_205276"; transcript_id "Novel_002816"; exon_number "4"; gene_type "
Dt_chr7	Cufflinks	exon	5123339	5123991	.	+	.	gene_id "XLOC_205281"; transcript_id "Novel_002817"; exon_number "1"; gene_type "
Dt_chr7	Cufflinks	exon	5124089	5124782	.	+	.	gene_id "XLOC_205281"; transcript_id "Novel_002817"; exon_number "2"; gene_type "
Dt_chr7	Cufflinks	exon	5124912	5125007	.	+	.	gene_id "XLOC_205281"; transcript_id "Novel_002817"; exon_number "3"; gene_type "
Dt_chr7	Cufflinks	exon	5125128	5125193	.	+	.	gene_id "XLOC_205281"; transcript_id "Novel_002817"; exon_number "4"; gene_type "
Dt_chr7	Cufflinks	exon	5126667	5127041	.	+	.	gene_id "XLOC_205281"; transcript_id "Novel_002817"; exon_number "5"; gene_type "
Dt_chr7	Cufflinks	exon	5127121	5127625	.	+	.	gene_id "XLOC_205281"; transcript_id "Novel_002817"; exon_number "6"; gene_type "
Dt_chr7	Cufflinks	exon	5124903	5125007	.	+	.	gene_id "XLOC_205281"; transcript_id "Novel_002818"; exon_number "1"; gene_type "
Dt_chr7	Cufflinks	exon	5125128	5125193	.	+	.	gene_id "XLOC_205281"; transcript_id "Novel_002818"; exon_number "2"; gene_type "
Dt_chr7	Cufflinks	exon	5126667	5127041	.	+	.	gene_id "XLOC_205281"; transcript_id "Novel_002818"; exon_number "3"; gene_type "
Dt_chr7	Cufflinks	exon	5127121	5127625	.	+	.	gene_id "XLOC_205281"; transcript_id "Novel_002818"; exon_number "4"; gene_type "
Dt_chr7	Cufflinks	exon	5157449	5157487	.	+	.	gene_id "XLOC_205291"; transcript_id "Novel_002819"; exon_number "1"; gene_type "
Dt_chr7	Cufflinks	exon	5157894	5158439	.	+	.	gene_id "XLOC_205291"; transcript_id "Novel_002819"; exon_number "2"; gene_type "
Dt_chr7	Cufflinks	exon	5158536	5158619	.	+	.	gene_id "XLOC_205291"; transcript_id "Novel_002819"; exon_number "3"; gene_type "
Dt_chr7	Cufflinks	exon	5158726	5159390	.	+	.	gene_id "XLOC_205291"; transcript_id "Novel_002819"; exon_number "4"; gene_type "
Dt_chr7	Cufflinks	exon	5157789	5158439	.	+	.	gene_id "XLOC_205291"; transcript_id "Novel_002820"; exon_number "1"; gene_type "
Dt_chr7	Cufflinks	exon	5158536	5158665	.	+	.	gene_id "XLOC_205291"; transcript_id "Novel_002820"; exon_number "2"; gene_type "
Dt_chr7	Cufflinks	exon	5161586	5161980	.	+	.	gene_id "XLOC_205292"; transcript_id "Novel_002821"; exon_number "1"; gene_type "
Dt_chr7	Cufflinks	exon	5162079	5162342	.	+	.	gene_id "XLOC_205292"; transcript_id "Novel_002821"; exon_number "2"; gene_type "
Dt_chr7	Cufflinks	exon	5162710	5163006	.	+	.	gene_id "XLOC_205292"; transcript_id "Novel_002821"; exon_number "3"; gene_type "
Dt_chr7	Cufflinks	exon	5161955	5162829	.	+	.	gene_id "XLOC_205292"; transcript_id "Novel_002822"; exon_number "1"; gene_type "
Dt_chr7	Cufflinks	exon	5163467	5163526	.	+	.	gene_id "XLOC_205292"; transcript_id "Novel_002822"; exon_number "2"; gene_type "
Dt_chr7	Cufflinks	exon	5164887	5164927	.	+	.	gene_id "XLOC_205292"; transcript_id "Novel_002822"; exon_number "3"; gene_type "
Dt_chr7	Cufflinks	exon	5165049	5165126	.	+	.	gene_id "XLOC_205292"; transcript_id "Novel_002822"; exon_number "4"; gene_type "
Dt_chr7	Cufflinks	exon	5165231	5165315	.	+	.	gene_id "XLOC_205292"; transcript_id "Novel_002822"; exon_number "5"; gene_type "
Dt_chr7	Cufflinks	exon	5165854	5166012	.	+	.	gene_id "XLOC_205292"; transcript_id "Novel_002822"; exon_number "6"; gene_type "
Dt_chr7	Cufflinks	exon	5167016	5167595	.	+	.	gene_id "XLOC_205292"; transcript_id "Novel_002822"; exon_number "7"; gene_type "
Dt_chr7	Cufflinks	exon	5197737	5200037	.	+	.	gene_id "XLOC_205304"; transcript_id "Novel_002823"; exon_number "1"; gene_type "
Dt_chr7	Cufflinks	exon	5200267	5200337	.	+	.	gene_id "XLOC_205304"; transcript_id "Novel_002823"; exon_number "2"; gene_type "
Dt_chr7	Cufflinks	exon	5200544	5200677	.	+	.	gene_id "XLOC_205304"; transcript_id "Novel_002823"; exon_number "3"; gene_type "

Dt_chr7	Cufflinks	exon	5200764	5200793	.	+	.	gene_id "XLOC_205304"; transcript_id "Novel_002823"; exon_number "4"; gene_type "
Dt_chr7	Cufflinks	exon	5197737	5200037	.	+	.	gene_id "XLOC_205304"; transcript_id "Novel_002824"; exon_number "1"; gene_type "
Dt_chr7	Cufflinks	exon	5200267	5200337	.	+	.	gene_id "XLOC_205304"; transcript_id "Novel_002824"; exon_number "2"; gene_type "
Dt_chr7	Cufflinks	exon	5200544	5201116	.	+	.	gene_id "XLOC_205304"; transcript_id "Novel_002824"; exon_number "3"; gene_type "
Dt_chr7	Cufflinks	exon	5201422	5201595	.	+	.	gene_id "XLOC_205304"; transcript_id "Novel_002824"; exon_number "4"; gene_type "
Dt_chr7	Cufflinks	exon	5201669	5202024	.	+	.	gene_id "XLOC_205304"; transcript_id "Novel_002824"; exon_number "5"; gene_type "
Dt_chr7	Cufflinks	exon	5319990	5320137	.	+	.	gene_id "XLOC_205310"; transcript_id "Novel_002825"; exon_number "1"; gene_type "
Dt_chr7	Cufflinks	exon	5320248	5321152	.	+	.	gene_id "XLOC_205310"; transcript_id "Novel_002825"; exon_number "2"; gene_type "
Dt_chr7	Cufflinks	exon	5322115	5322227	.	+	.	gene_id "XLOC_205311"; transcript_id "Novel_002826"; exon_number "1"; gene_type "
Dt_chr7	Cufflinks	exon	5322306	5322441	.	+	.	gene_id "XLOC_205311"; transcript_id "Novel_002826"; exon_number "2"; gene_type "
Dt_chr7	Cufflinks	exon	5376784	5380019	.	+	.	gene_id "XLOC_205320"; transcript_id "Novel_002827"; exon_number "1"; gene_type "
Dt_chr7	Cufflinks	exon	5380114	5380530	.	+	.	gene_id "XLOC_205320"; transcript_id "Novel_002827"; exon_number "2"; gene_type "
Dt_chr7	Cufflinks	exon	5510507	5510892	.	+	.	gene_id "XLOC_205329"; transcript_id "Novel_002828"; exon_number "1"; gene_type "
Dt_chr7	Cufflinks	exon	5511115	5511687	.	+	.	gene_id "XLOC_205329"; transcript_id "Novel_002828"; exon_number "2"; gene_type "
Dt_chr7	Cufflinks	exon	5511777	5512893	.	+	.	gene_id "XLOC_205329"; transcript_id "Novel_002828"; exon_number "3"; gene_type "
Dt_chr7	Cufflinks	exon	5653317	5653378	.	+	.	gene_id "XLOC_205348"; transcript_id "Novel_002829"; exon_number "1"; gene_type "
Dt_chr7	Cufflinks	exon	5653742	5653858	.	+	.	gene_id "XLOC_205348"; transcript_id "Novel_002829"; exon_number "2"; gene_type "
Dt_chr7	Cufflinks	exon	5675933	5676127	.	+	.	gene_id "XLOC_205348"; transcript_id "Novel_002829"; exon_number "3"; gene_type "
Dt_chr7	Cufflinks	exon	5676247	5676474	.	+	.	gene_id "XLOC_205348"; transcript_id "Novel_002829"; exon_number "4"; gene_type "
Dt_chr7	Cufflinks	exon	5674320	5674580	.	+	.	gene_id "XLOC_205348"; transcript_id "Novel_002830"; exon_number "1"; gene_type "
Dt_chr7	Cufflinks	exon	5674949	5675065	.	+	.	gene_id "XLOC_205348"; transcript_id "Novel_002830"; exon_number "2"; gene_type "
Dt_chr7	Cufflinks	exon	5675933	5676127	.	+	.	gene_id "XLOC_205348"; transcript_id "Novel_002830"; exon_number "3"; gene_type "
Dt_chr7	Cufflinks	exon	5676247	5676485	.	+	.	gene_id "XLOC_205348"; transcript_id "Novel_002830"; exon_number "4"; gene_type "
Dt_chr7	Cufflinks	exon	5674320	5674580	.	+	.	gene_id "XLOC_205348"; transcript_id "Novel_002831"; exon_number "1"; gene_type "
Dt_chr7	Cufflinks	exon	5674949	5675065	.	+	.	gene_id "XLOC_205348"; transcript_id "Novel_002831"; exon_number "2"; gene_type "
Dt_chr7	Cufflinks	exon	5675933	5676127	.	+	.	gene_id "XLOC_205348"; transcript_id "Novel_002831"; exon_number "3"; gene_type "
Dt_chr7	Cufflinks	exon	5676250	5676485	.	+	.	gene_id "XLOC_205348"; transcript_id "Novel_002831"; exon_number "4"; gene_type "
Dt_chr7	Cufflinks	exon	5675083	5676127	.	+	.	gene_id "XLOC_205348"; transcript_id "Novel_002832"; exon_number "1"; gene_type "
Dt_chr7	Cufflinks	exon	5676247	5676573	.	+	.	gene_id "XLOC_205348"; transcript_id "Novel_002832"; exon_number "2"; gene_type "
Dt_chr7	Cufflinks	exon	5656488	5656837	.	+	.	gene_id "XLOC_205351"; transcript_id "Novel_002833"; exon_number "1"; gene_type "
Dt_chr7	Cufflinks	exon	5657066	5657225	.	+	.	gene_id "XLOC_205351"; transcript_id "Novel_002833"; exon_number "2"; gene_type "
Dt_chr7	Cufflinks	exon	5678431	5678468	.	+	.	gene_id "XLOC_205351"; transcript_id "Novel_002833"; exon_number "3"; gene_type "
Dt_chr7	Cufflinks	exon	5678607	5679039	.	+	.	gene_id "XLOC_205351"; transcript_id "Novel_002833"; exon_number "4"; gene_type "
Dt_chr7	Cufflinks	exon	5656889	5657225	.	+	.	gene_id "XLOC_205351"; transcript_id "Novel_002834"; exon_number "1"; gene_type "
Dt_chr7	Cufflinks	exon	5678431	5678468	.	+	.	gene_id "XLOC_205351"; transcript_id "Novel_002834"; exon_number "2"; gene_type "

Dt_chr7	Cufflinks	exon	5678607	5679036	.	+	.	gene_id "XLOC_205351";	transcript_id "Novel_002834";	exon_number "3";	gene_type "
Dt_chr7	Cufflinks	exon	5677412	5677637	.	+	.	gene_id "XLOC_205351";	transcript_id "Novel_002835";	exon_number "1";	gene_type "
Dt_chr7	Cufflinks	exon	5677911	5678084	.	+	.	gene_id "XLOC_205351";	transcript_id "Novel_002835";	exon_number "2";	gene_type "
Dt_chr7	Cufflinks	exon	5678271	5678468	.	+	.	gene_id "XLOC_205351";	transcript_id "Novel_002835";	exon_number "3";	gene_type "
Dt_chr7	Cufflinks	exon	5678607	5679036	.	+	.	gene_id "XLOC_205351";	transcript_id "Novel_002835";	exon_number "4";	gene_type "
Dt_chr7	Cufflinks	exon	5677556	5678084	.	+	.	gene_id "XLOC_205351";	transcript_id "Novel_002836";	exon_number "1";	gene_type "
Dt_chr7	Cufflinks	exon	5678271	5678468	.	+	.	gene_id "XLOC_205351";	transcript_id "Novel_002836";	exon_number "2";	gene_type "
Dt_chr7	Cufflinks	exon	5678607	5679129	.	+	.	gene_id "XLOC_205351";	transcript_id "Novel_002836";	exon_number "3";	gene_type "
Dt_chr7	Cufflinks	exon	5671867	5671956	.	+	.	gene_id "XLOC_205353";	transcript_id "Novel_002837";	exon_number "1";	gene_type "
Dt_chr7	Cufflinks	exon	5672295	5672370	.	+	.	gene_id "XLOC_205353";	transcript_id "Novel_002837";	exon_number "2";	gene_type "
Dt_chr7	Cufflinks	exon	5672476	5672982	.	+	.	gene_id "XLOC_205353";	transcript_id "Novel_002837";	exon_number "3";	gene_type "
Dt_chr7	Cufflinks	exon	5748782	5749233	.	+	.	gene_id "XLOC_205379";	transcript_id "Novel_002838";	exon_number "1";	gene_type "
Dt_chr7	Cufflinks	exon	5749869	5750185	.	+	.	gene_id "XLOC_205379";	transcript_id "Novel_002838";	exon_number "2";	gene_type "
Dt_chr7	Cufflinks	exon	5778365	5778922	.	+	.	gene_id "XLOC_205383";	transcript_id "Novel_002839";	exon_number "1";	gene_type "
Dt_chr7	Cufflinks	exon	5779009	5780363	.	+	.	gene_id "XLOC_205383";	transcript_id "Novel_002839";	exon_number "2";	gene_type "
Dt_chr7	Cufflinks	exon	5780522	5780756	.	+	.	gene_id "XLOC_205383";	transcript_id "Novel_002839";	exon_number "3";	gene_type "
Dt_chr7	Cufflinks	exon	5781186	5782001	.	+	.	gene_id "XLOC_205383";	transcript_id "Novel_002839";	exon_number "4";	gene_type "
Dt_chr7	Cufflinks	exon	5797435	5798875	.	+	.	gene_id "XLOC_205389";	transcript_id "Novel_002840";	exon_number "1";	gene_type "
Dt_chr7	Cufflinks	exon	5798966	5801773	.	+	.	gene_id "XLOC_205389";	transcript_id "Novel_002840";	exon_number "2";	gene_type "
Dt_chr7	Cufflinks	exon	5797441	5798316	.	+	.	gene_id "XLOC_205389";	transcript_id "Novel_002841";	exon_number "1";	gene_type "
Dt_chr7	Cufflinks	exon	5798444	5799447	.	+	.	gene_id "XLOC_205389";	transcript_id "Novel_002841";	exon_number "2";	gene_type "
Dt_chr7	Cufflinks	exon	5797445	5799970	.	+	.	gene_id "XLOC_205389";	transcript_id "Novel_002842";	exon_number "1";	gene_type "
Dt_chr7	Cufflinks	exon	5800055	5800267	.	+	.	gene_id "XLOC_205389";	transcript_id "Novel_002842";	exon_number "2";	gene_type "
Dt_chr7	Cufflinks	exon	5801412	5801458	.	+	.	gene_id "XLOC_205389";	transcript_id "Novel_002842";	exon_number "3";	gene_type "
Dt_chr7	Cufflinks	exon	5908247	5908997	.	+	.	gene_id "XLOC_205416";	transcript_id "Novel_002843";	exon_number "1";	gene_type "
Dt_chr7	Cufflinks	exon	5909089	5909287	.	+	.	gene_id "XLOC_205416";	transcript_id "Novel_002843";	exon_number "2";	gene_type "
Dt_chr7	Cufflinks	exon	5908361	5908452	.	+	.	gene_id "XLOC_205416";	transcript_id "Novel_002844";	exon_number "1";	gene_type "
Dt_chr7	Cufflinks	exon	5908530	5908983	.	+	.	gene_id "XLOC_205416";	transcript_id "Novel_002844";	exon_number "2";	gene_type "
Dt_chr7	Cufflinks	exon	5908366	5908791	.	+	.	gene_id "XLOC_205416";	transcript_id "Novel_002845";	exon_number "1";	gene_type "
Dt_chr7	Cufflinks	exon	5908920	5908997	.	+	.	gene_id "XLOC_205416";	transcript_id "Novel_002845";	exon_number "2";	gene_type "
Dt_chr7	Cufflinks	exon	5909089	5910148	.	+	.	gene_id "XLOC_205416";	transcript_id "Novel_002845";	exon_number "3";	gene_type "
Dt_chr7	Cufflinks	exon	5908370	5908452	.	+	.	gene_id "XLOC_205416";	transcript_id "Novel_002846";	exon_number "1";	gene_type "
Dt_chr7	Cufflinks	exon	5908530	5908791	.	+	.	gene_id "XLOC_205416";	transcript_id "Novel_002846";	exon_number "2";	gene_type "
Dt_chr7	Cufflinks	exon	5908920	5908941	.	+	.	gene_id "XLOC_205416";	transcript_id "Novel_002846";	exon_number "3";	gene_type "
Dt_chr7	Cufflinks	exon	5910206	5910610	.	+	.	gene_id "XLOC_205416";	transcript_id "Novel_002847";	exon_number "1";	gene_type "

Dt_chr7	Cufflinks	exon	5910716	5910836	.	+	.	gene_id "XLOC_205416"; transcript_id "Novel_002847"; exon_number "2"; gene_type "
Dt_chr7	Cufflinks	exon	5910914	5910982	.	+	.	gene_id "XLOC_205416"; transcript_id "Novel_002847"; exon_number "3"; gene_type "
Dt_chr7	Cufflinks	exon	5910217	5910610	.	+	.	gene_id "XLOC_205416"; transcript_id "Novel_002848"; exon_number "1"; gene_type "
Dt_chr7	Cufflinks	exon	5910716	5911057	.	+	.	gene_id "XLOC_205416"; transcript_id "Novel_002848"; exon_number "2"; gene_type "
Dt_chr7	Cufflinks	exon	5916506	5916530	.	+	.	gene_id "XLOC_205417"; transcript_id "Novel_002849"; exon_number "1"; gene_type "
Dt_chr7	Cufflinks	exon	5916606	5917165	.	+	.	gene_id "XLOC_205417"; transcript_id "Novel_002849"; exon_number "2"; gene_type "
Dt_chr7	Cufflinks	exon	7070054	7070547	.	+	.	gene_id "XLOC_205638"; transcript_id "Novel_002850"; exon_number "1"; gene_type "
Dt_chr7	Cufflinks	exon	7071343	7071412	.	+	.	gene_id "XLOC_205638"; transcript_id "Novel_002850"; exon_number "2"; gene_type "
Dt_chr7	Cufflinks	exon	7071533	7071705	.	+	.	gene_id "XLOC_205638"; transcript_id "Novel_002850"; exon_number "3"; gene_type "
Dt_chr7	Cufflinks	exon	7072133	7072307	.	+	.	gene_id "XLOC_205638"; transcript_id "Novel_002850"; exon_number "4"; gene_type "
Dt_chr7	Cufflinks	exon	7072650	7072816	.	+	.	gene_id "XLOC_205638"; transcript_id "Novel_002850"; exon_number "5"; gene_type "
Dt_chr7	Cufflinks	exon	7072934	7073153	.	+	.	gene_id "XLOC_205638"; transcript_id "Novel_002850"; exon_number "6"; gene_type "
Dt_chr7	Cufflinks	exon	7073250	7073354	.	+	.	gene_id "XLOC_205638"; transcript_id "Novel_002850"; exon_number "7"; gene_type "
Dt_chr7	Cufflinks	exon	7073622	7073774	.	+	.	gene_id "XLOC_205638"; transcript_id "Novel_002850"; exon_number "8"; gene_type "
Dt_chr7	Cufflinks	exon	7073912	7074643	.	+	.	gene_id "XLOC_205638"; transcript_id "Novel_002850"; exon_number "9"; gene_type "
Dt_chr7	Cufflinks	exon	7408083	7408223	.	+	.	gene_id "XLOC_205693"; transcript_id "Novel_002851"; exon_number "1"; gene_type "
Dt_chr7	Cufflinks	exon	7408692	7408973	.	+	.	gene_id "XLOC_205693"; transcript_id "Novel_002851"; exon_number "2"; gene_type "
Dt_chr7	Cufflinks	exon	7409059	7409130	.	+	.	gene_id "XLOC_205693"; transcript_id "Novel_002851"; exon_number "3"; gene_type "
Dt_chr7	Cufflinks	exon	7409295	7409362	.	+	.	gene_id "XLOC_205693"; transcript_id "Novel_002851"; exon_number "4"; gene_type "
Dt_chr7	Cufflinks	exon	7409556	7409706	.	+	.	gene_id "XLOC_205693"; transcript_id "Novel_002851"; exon_number "5"; gene_type "
Dt_chr7	Cufflinks	exon	7409793	7409873	.	+	.	gene_id "XLOC_205693"; transcript_id "Novel_002851"; exon_number "6"; gene_type "
Dt_chr7	Cufflinks	exon	7409962	7410051	.	+	.	gene_id "XLOC_205693"; transcript_id "Novel_002851"; exon_number "7"; gene_type "
Dt_chr7	Cufflinks	exon	7410163	7410945	.	+	.	gene_id "XLOC_205693"; transcript_id "Novel_002851"; exon_number "8"; gene_type "
Dt_chr7	Cufflinks	exon	7570436	7570674	.	+	.	gene_id "XLOC_205723"; transcript_id "Novel_002852"; exon_number "1"; gene_type "
Dt_chr7	Cufflinks	exon	7570761	7571395	.	+	.	gene_id "XLOC_205723"; transcript_id "Novel_002852"; exon_number "2"; gene_type "
Dt_chr7	Cufflinks	exon	8236556	8236731	.	+	.	gene_id "XLOC_205840"; transcript_id "Novel_002853"; exon_number "1"; gene_type "
Dt_chr7	Cufflinks	exon	8236966	8237117	.	+	.	gene_id "XLOC_205840"; transcript_id "Novel_002853"; exon_number "2"; gene_type "
Dt_chr7	Cufflinks	exon	8238093	8238137	.	+	.	gene_id "XLOC_205840"; transcript_id "Novel_002853"; exon_number "3"; gene_type "
Dt_chr7	Cufflinks	exon	8239061	8241158	.	+	.	gene_id "XLOC_205840"; transcript_id "Novel_002853"; exon_number "4"; gene_type "
Dt_chr7	Cufflinks	exon	8236620	8236731	.	+	.	gene_id "XLOC_205840"; transcript_id "Novel_002854"; exon_number "1"; gene_type "
Dt_chr7	Cufflinks	exon	8238093	8238137	.	+	.	gene_id "XLOC_205840"; transcript_id "Novel_002854"; exon_number "2"; gene_type "
Dt_chr7	Cufflinks	exon	8239061	8241576	.	+	.	gene_id "XLOC_205840"; transcript_id "Novel_002854"; exon_number "3"; gene_type "
Dt_chr7	Cufflinks	exon	8443566	8445031	.	+	.	gene_id "XLOC_205879"; transcript_id "Novel_002855"; exon_number "1"; gene_type "
Dt_chr7	Cufflinks	exon	8445509	8446045	.	+	.	gene_id "XLOC_205879"; transcript_id "Novel_002855"; exon_number "2"; gene_type "
Dt_chr7	Cufflinks	exon	8443570	8445031	.	+	.	gene_id "XLOC_205879"; transcript_id "Novel_002856"; exon_number "1"; gene_type "

Dt_chr7	Cufflinks	exon	8445668	8446279	.	+	.	gene_id "XLOC_205879"; transcript_id "Novel_002856"; exon_number "2"; gene_type "
Dt_chr7	Cufflinks	exon	8480800	8481927	.	+	.	gene_id "XLOC_205881"; transcript_id "Novel_002857"; exon_number "1"; gene_type "
Dt_chr7	Cufflinks	exon	8482121	8482908	.	+	.	gene_id "XLOC_205881"; transcript_id "Novel_002857"; exon_number "2"; gene_type "
Dt_chr7	Cufflinks	exon	8873639	8873897	.	+	.	gene_id "XLOC_205933"; transcript_id "Novel_002858"; exon_number "1"; gene_type "
Dt_chr7	Cufflinks	exon	8964589	8965061	.	+	.	gene_id "XLOC_205941"; transcript_id "Novel_002859"; exon_number "1"; gene_type "
Dt_chr7	Cufflinks	exon	8965162	8965356	.	+	.	gene_id "XLOC_205941"; transcript_id "Novel_002859"; exon_number "2"; gene_type "
Dt_chr7	Cufflinks	exon	8969874	8970110	.	+	.	gene_id "XLOC_205941"; transcript_id "Novel_002859"; exon_number "3"; gene_type "
Dt_chr7	Cufflinks	exon	8964605	8965061	.	+	.	gene_id "XLOC_205941"; transcript_id "Novel_002860"; exon_number "1"; gene_type "
Dt_chr7	Cufflinks	exon	8965162	8965356	.	+	.	gene_id "XLOC_205941"; transcript_id "Novel_002860"; exon_number "2"; gene_type "
Dt_chr7	Cufflinks	exon	8969874	8969996	.	+	.	gene_id "XLOC_205941"; transcript_id "Novel_002860"; exon_number "3"; gene_type "
Dt_chr7	Cufflinks	exon	8970791	8970996	.	+	.	gene_id "XLOC_205941"; transcript_id "Novel_002860"; exon_number "4"; gene_type "
Dt_chr7	Cufflinks	exon	8971080	8971778	.	+	.	gene_id "XLOC_205941"; transcript_id "Novel_002860"; exon_number "5"; gene_type "
Dt_chr7	Cufflinks	exon	8964605	8965061	.	+	.	gene_id "XLOC_205941"; transcript_id "Novel_002861"; exon_number "1"; gene_type "
Dt_chr7	Cufflinks	exon	8965162	8965356	.	+	.	gene_id "XLOC_205941"; transcript_id "Novel_002861"; exon_number "2"; gene_type "
Dt_chr7	Cufflinks	exon	8969874	8969996	.	+	.	gene_id "XLOC_205941"; transcript_id "Novel_002861"; exon_number "3"; gene_type "
Dt_chr7	Cufflinks	exon	8970791	8971778	.	+	.	gene_id "XLOC_205941"; transcript_id "Novel_002861"; exon_number "4"; gene_type "
Dt_chr7	Cufflinks	exon	8969835	8969996	.	+	.	gene_id "XLOC_205941"; transcript_id "Novel_002862"; exon_number "1"; gene_type "
Dt_chr7	Cufflinks	exon	8970791	8970996	.	+	.	gene_id "XLOC_205941"; transcript_id "Novel_002862"; exon_number "2"; gene_type "
Dt_chr7	Cufflinks	exon	8971080	8971688	.	+	.	gene_id "XLOC_205941"; transcript_id "Novel_002862"; exon_number "3"; gene_type "
Dt_chr7	Cufflinks	exon	9111690	9111997	.	+	.	gene_id "XLOC_205973"; transcript_id "Novel_002863"; exon_number "1"; gene_type "
Dt_chr7	Cufflinks	exon	9112098	9112167	.	+	.	gene_id "XLOC_205973"; transcript_id "Novel_002863"; exon_number "2"; gene_type "
Dt_chr7	Cufflinks	exon	9204890	9205079	.	+	.	gene_id "XLOC_205985"; transcript_id "Novel_002864"; exon_number "1"; gene_type "
Dt_chr7	Cufflinks	exon	9205645	9205828	.	+	.	gene_id "XLOC_205985"; transcript_id "Novel_002864"; exon_number "2"; gene_type "
Dt_chr7	Cufflinks	exon	9206037	9206076	.	+	.	gene_id "XLOC_205985"; transcript_id "Novel_002864"; exon_number "3"; gene_type "
Dt_chr7	Cufflinks	exon	9206357	9206407	.	+	.	gene_id "XLOC_205985"; transcript_id "Novel_002864"; exon_number "4"; gene_type "
Dt_chr7	Cufflinks	exon	9207698	9207783	.	+	.	gene_id "XLOC_205985"; transcript_id "Novel_002864"; exon_number "5"; gene_type "
Dt_chr7	Cufflinks	exon	9208468	9208605	.	+	.	gene_id "XLOC_205985"; transcript_id "Novel_002864"; exon_number "6"; gene_type "
Dt_chr7	Cufflinks	exon	9208735	9208885	.	+	.	gene_id "XLOC_205985"; transcript_id "Novel_002864"; exon_number "7"; gene_type "
Dt_chr7	Cufflinks	exon	9209076	9209836	.	+	.	gene_id "XLOC_205985"; transcript_id "Novel_002864"; exon_number "8"; gene_type "
Dt_chr7	Cufflinks	exon	9204906	9205079	.	+	.	gene_id "XLOC_205985"; transcript_id "Novel_002865"; exon_number "1"; gene_type "
Dt_chr7	Cufflinks	exon	9205645	9205828	.	+	.	gene_id "XLOC_205985"; transcript_id "Novel_002865"; exon_number "2"; gene_type "
Dt_chr7	Cufflinks	exon	9206037	9206076	.	+	.	gene_id "XLOC_205985"; transcript_id "Novel_002865"; exon_number "3"; gene_type "
Dt_chr7	Cufflinks	exon	9206932	9206982	.	+	.	gene_id "XLOC_205985"; transcript_id "Novel_002865"; exon_number "4"; gene_type "
Dt_chr7	Cufflinks	exon	9207698	9207783	.	+	.	gene_id "XLOC_205985"; transcript_id "Novel_002865"; exon_number "5"; gene_type "
Dt_chr7	Cufflinks	exon	9208468	9208605	.	+	.	gene_id "XLOC_205985"; transcript_id "Novel_002865"; exon_number "6"; gene_type "

Dt_chr7	Cufflinks	exon	9208735	9208885	.	+	.	gene_id "XLOC_205985"; transcript_id "Novel_002865"; exon_number "7"; gene_type "
Dt_chr7	Cufflinks	exon	9209076	9209409	.	+	.	gene_id "XLOC_205985"; transcript_id "Novel_002865"; exon_number "8"; gene_type "
Dt_chr7	Cufflinks	exon	9210015	9210505	.	+	.	gene_id "XLOC_205985"; transcript_id "Novel_002866"; exon_number "1"; gene_type "
Dt_chr7	Cufflinks	exon	9210580	9210666	.	+	.	gene_id "XLOC_205985"; transcript_id "Novel_002866"; exon_number "2"; gene_type "
Dt_chr7	Cufflinks	exon	9210775	9210879	.	+	.	gene_id "XLOC_205985"; transcript_id "Novel_002866"; exon_number "3"; gene_type "
Dt_chr7	Cufflinks	exon	9210960	9211059	.	+	.	gene_id "XLOC_205985"; transcript_id "Novel_002866"; exon_number "4"; gene_type "
Dt_chr7	Cufflinks	exon	9211155	9211246	.	+	.	gene_id "XLOC_205985"; transcript_id "Novel_002866"; exon_number "5"; gene_type "
Dt_chr7	Cufflinks	exon	9211330	9211410	.	+	.	gene_id "XLOC_205985"; transcript_id "Novel_002866"; exon_number "6"; gene_type "
Dt_chr7	Cufflinks	exon	9211509	9211577	.	+	.	gene_id "XLOC_205985"; transcript_id "Novel_002866"; exon_number "7"; gene_type "
Dt_chr7	Cufflinks	exon	9211938	9212404	.	+	.	gene_id "XLOC_205985"; transcript_id "Novel_002866"; exon_number "8"; gene_type "
Dt_chr7	Cufflinks	exon	9210127	9210505	.	+	.	gene_id "XLOC_205985"; transcript_id "Novel_002867"; exon_number "1"; gene_type "
Dt_chr7	Cufflinks	exon	9210580	9210666	.	+	.	gene_id "XLOC_205985"; transcript_id "Novel_002867"; exon_number "2"; gene_type "
Dt_chr7	Cufflinks	exon	9210775	9211059	.	+	.	gene_id "XLOC_205985"; transcript_id "Novel_002867"; exon_number "3"; gene_type "
Dt_chr7	Cufflinks	exon	9211155	9211246	.	+	.	gene_id "XLOC_205985"; transcript_id "Novel_002867"; exon_number "4"; gene_type "
Dt_chr7	Cufflinks	exon	9211330	9211410	.	+	.	gene_id "XLOC_205985"; transcript_id "Novel_002867"; exon_number "5"; gene_type "
Dt_chr7	Cufflinks	exon	9211509	9211577	.	+	.	gene_id "XLOC_205985"; transcript_id "Novel_002867"; exon_number "6"; gene_type "
Dt_chr7	Cufflinks	exon	9211938	9212501	.	+	.	gene_id "XLOC_205985"; transcript_id "Novel_002867"; exon_number "7"; gene_type "
Dt_chr7	Cufflinks	exon	9210272	9210505	.	+	.	gene_id "XLOC_205985"; transcript_id "Novel_002868"; exon_number "1"; gene_type "
Dt_chr7	Cufflinks	exon	9210580	9210666	.	+	.	gene_id "XLOC_205985"; transcript_id "Novel_002868"; exon_number "2"; gene_type "
Dt_chr7	Cufflinks	exon	9210764	9211059	.	+	.	gene_id "XLOC_205985"; transcript_id "Novel_002868"; exon_number "3"; gene_type "
Dt_chr7	Cufflinks	exon	9211181	9211246	.	+	.	gene_id "XLOC_205985"; transcript_id "Novel_002868"; exon_number "4"; gene_type "
Dt_chr7	Cufflinks	exon	9211330	9211410	.	+	.	gene_id "XLOC_205985"; transcript_id "Novel_002868"; exon_number "5"; gene_type "
Dt_chr7	Cufflinks	exon	9211509	9211577	.	+	.	gene_id "XLOC_205985"; transcript_id "Novel_002868"; exon_number "6"; gene_type "
Dt_chr7	Cufflinks	exon	9211938	9212412	.	+	.	gene_id "XLOC_205985"; transcript_id "Novel_002868"; exon_number "7"; gene_type "
Dt_chr7	Cufflinks	exon	9210344	9210666	.	+	.	gene_id "XLOC_205985"; transcript_id "Novel_002869"; exon_number "1"; gene_type "
Dt_chr7	Cufflinks	exon	9210775	9211059	.	+	.	gene_id "XLOC_205985"; transcript_id "Novel_002869"; exon_number "2"; gene_type "
Dt_chr7	Cufflinks	exon	9211155	9211246	.	+	.	gene_id "XLOC_205985"; transcript_id "Novel_002869"; exon_number "3"; gene_type "
Dt_chr7	Cufflinks	exon	9211330	9211410	.	+	.	gene_id "XLOC_205985"; transcript_id "Novel_002869"; exon_number "4"; gene_type "
Dt_chr7	Cufflinks	exon	9211509	9211577	.	+	.	gene_id "XLOC_205985"; transcript_id "Novel_002869"; exon_number "5"; gene_type "
Dt_chr7	Cufflinks	exon	9211938	9212404	.	+	.	gene_id "XLOC_205985"; transcript_id "Novel_002869"; exon_number "6"; gene_type "
Dt_chr7	Cufflinks	exon	9488323	9488686	.	+	.	gene_id "XLOC_206027"; transcript_id "Novel_002870"; exon_number "1"; gene_type "
Dt_chr7	Cufflinks	exon	9489871	9491461	.	+	.	gene_id "XLOC_206027"; transcript_id "Novel_002870"; exon_number "2"; gene_type "
Dt_chr7	Cufflinks	exon	10051243	10052104	.	+	.	gene_id "XLOC_206125"; transcript_id "Novel_002871"; exon_number "1"; gene_type "
Dt_chr7	Cufflinks	exon	10052187	10055510	.	+	.	gene_id "XLOC_206125"; transcript_id "Novel_002871"; exon_number "2"; gene_type "
Dt_chr7	Cufflinks	exon	10051243	10051918	.	+	.	gene_id "XLOC_206125"; transcript_id "Novel_002872"; exon_number "1"; gene_type "

Dt_chr7	Cufflinks	exon	10052051	10052104	.	+	.	gene_id "XLOC_206125"; transcript_id "Novel_002872"; exon_number "2"; gene_type "
Dt_chr7	Cufflinks	exon	10052187	10055510	.	+	.	gene_id "XLOC_206125"; transcript_id "Novel_002872"; exon_number "3"; gene_type "
Dt_chr7	Cufflinks	exon	10751828	10752378	.	+	.	gene_id "XLOC_206219"; transcript_id "Novel_002873"; exon_number "1"; gene_type "
Dt_chr7	Cufflinks	exon	10752972	10753826	.	+	.	gene_id "XLOC_206219"; transcript_id "Novel_002873"; exon_number "2"; gene_type "
Dt_chr7	Cufflinks	exon	11830997	11831025	.	+	.	gene_id "XLOC_206371"; transcript_id "Novel_002874"; exon_number "1"; gene_type "
Dt_chr7	Cufflinks	exon	11831538	11831921	.	+	.	gene_id "XLOC_206371"; transcript_id "Novel_002874"; exon_number "2"; gene_type "
Dt_chr7	Cufflinks	exon	13945930	13953017	.	+	.	gene_id "XLOC_206651"; transcript_id "Novel_002875"; exon_number "1"; gene_type "
Dt_chr7	Cufflinks	exon	13954045	13954687	.	+	.	gene_id "XLOC_206651"; transcript_id "Novel_002875"; exon_number "2"; gene_type "
Dt_chr7	Cufflinks	exon	14309968	14310098	.	+	.	gene_id "XLOC_206695"; transcript_id "Novel_002876"; exon_number "1"; gene_type "
Dt_chr7	Cufflinks	exon	14311087	14311223	.	+	.	gene_id "XLOC_206695"; transcript_id "Novel_002876"; exon_number "2"; gene_type "
Dt_chr7	Cufflinks	exon	14311486	14311570	.	+	.	gene_id "XLOC_206695"; transcript_id "Novel_002876"; exon_number "3"; gene_type "
Dt_chr7	Cufflinks	exon	14311690	14311762	.	+	.	gene_id "XLOC_206695"; transcript_id "Novel_002876"; exon_number "4"; gene_type "
Dt_chr7	Cufflinks	exon	14312301	14312398	.	+	.	gene_id "XLOC_206695"; transcript_id "Novel_002876"; exon_number "5"; gene_type "
Dt_chr7	Cufflinks	exon	14312570	14312662	.	+	.	gene_id "XLOC_206695"; transcript_id "Novel_002876"; exon_number "6"; gene_type "
Dt_chr7	Cufflinks	exon	14312807	14312880	.	+	.	gene_id "XLOC_206695"; transcript_id "Novel_002876"; exon_number "7"; gene_type "
Dt_chr7	Cufflinks	exon	14312980	14313018	.	+	.	gene_id "XLOC_206695"; transcript_id "Novel_002876"; exon_number "8"; gene_type "
Dt_chr7	Cufflinks	exon	14313102	14313190	.	+	.	gene_id "XLOC_206695"; transcript_id "Novel_002876"; exon_number "9"; gene_type "
Dt_chr7	Cufflinks	exon	14313267	14313343	.	+	.	gene_id "XLOC_206695"; transcript_id "Novel_002876"; exon_number "10"; gene_type
Dt_chr7	Cufflinks	exon	14313443	14313517	.	+	.	gene_id "XLOC_206695"; transcript_id "Novel_002876"; exon_number "11"; gene_type
Dt_chr7	Cufflinks	exon	14313772	14313819	.	+	.	gene_id "XLOC_206695"; transcript_id "Novel_002876"; exon_number "12"; gene_type
Dt_chr7	Cufflinks	exon	14313912	14313995	.	+	.	gene_id "XLOC_206695"; transcript_id "Novel_002876"; exon_number "13"; gene_type
Dt_chr7	Cufflinks	exon	14314085	14314172	.	+	.	gene_id "XLOC_206695"; transcript_id "Novel_002876"; exon_number "14"; gene_type
Dt_chr7	Cufflinks	exon	14314249	14314327	.	+	.	gene_id "XLOC_206695"; transcript_id "Novel_002876"; exon_number "15"; gene_type
Dt_chr7	Cufflinks	exon	14314713	14314803	.	+	.	gene_id "XLOC_206695"; transcript_id "Novel_002876"; exon_number "16"; gene_type
Dt_chr7	Cufflinks	exon	14314893	14314971	.	+	.	gene_id "XLOC_206695"; transcript_id "Novel_002876"; exon_number "17"; gene_type
Dt_chr7	Cufflinks	exon	14315174	14315289	.	+	.	gene_id "XLOC_206695"; transcript_id "Novel_002876"; exon_number "18"; gene_type
Dt_chr7	Cufflinks	exon	14315495	14315824	.	+	.	gene_id "XLOC_206695"; transcript_id "Novel_002876"; exon_number "19"; gene_type
Dt_chr7	Cufflinks	exon	18851429	18851615	.	+	.	gene_id "XLOC_207152"; transcript_id "Novel_002877"; exon_number "1"; gene_type "
Dt_chr7	Cufflinks	exon	18851736	18851886	.	+	.	gene_id "XLOC_207152"; transcript_id "Novel_002877"; exon_number "2"; gene_type "
Dt_chr7	Cufflinks	exon	18924211	18924393	.	+	.	gene_id "XLOC_207168"; transcript_id "Novel_002878"; exon_number "1"; gene_type "
Dt_chr7	Cufflinks	exon	18924487	18924547	.	+	.	gene_id "XLOC_207168"; transcript_id "Novel_002878"; exon_number "2"; gene_type "
Dt_chr7	Cufflinks	exon	18925293	18927376	.	+	.	gene_id "XLOC_207168"; transcript_id "Novel_002878"; exon_number "3"; gene_type "
Dt_chr7	Cufflinks	exon	20496077	20496334	.	+	.	gene_id "XLOC_207364"; transcript_id "Novel_002879"; exon_number "1"; gene_type "
Dt_chr7	Cufflinks	exon	20499046	20501133	.	+	.	gene_id "XLOC_207364"; transcript_id "Novel_002879"; exon_number "2"; gene_type "
Dt_chr7	Cufflinks	exon	23268196	23268439	.	+	.	gene_id "XLOC_207642"; transcript_id "Novel_002880"; exon_number "1"; gene_type "

Dt_chr7	Cufflinks	exon	23269068	23269155	.	+	.	gene_id "XLOC_207642"; transcript_id "Novel_002880"; exon_number "2"; gene_type "
Dt_chr7	Cufflinks	exon	23269401	23269799	.	+	.	gene_id "XLOC_207642"; transcript_id "Novel_002880"; exon_number "3"; gene_type "
Dt_chr7	Cufflinks	exon	23270012	23270713	.	+	.	gene_id "XLOC_207642"; transcript_id "Novel_002880"; exon_number "4"; gene_type "
Dt_chr7	Cufflinks	exon	23268245	23268439	.	+	.	gene_id "XLOC_207642"; transcript_id "Novel_002881"; exon_number "1"; gene_type "
Dt_chr7	Cufflinks	exon	23269068	23269105	.	+	.	gene_id "XLOC_207642"; transcript_id "Novel_002881"; exon_number "2"; gene_type "
Dt_chr7	Cufflinks	exon	23269401	23269799	.	+	.	gene_id "XLOC_207642"; transcript_id "Novel_002881"; exon_number "3"; gene_type "
Dt_chr7	Cufflinks	exon	23270012	23270336	.	+	.	gene_id "XLOC_207642"; transcript_id "Novel_002881"; exon_number "4"; gene_type "
Dt_chr7	Cufflinks	exon	23271035	23271709	.	+	.	gene_id "XLOC_207642"; transcript_id "Novel_002881"; exon_number "5"; gene_type "
Dt_chr7	Cufflinks	exon	23268247	23268439	.	+	.	gene_id "XLOC_207642"; transcript_id "Novel_002882"; exon_number "1"; gene_type "
Dt_chr7	Cufflinks	exon	23269068	23269799	.	+	.	gene_id "XLOC_207642"; transcript_id "Novel_002882"; exon_number "2"; gene_type "
Dt_chr7	Cufflinks	exon	23270012	23270766	.	+	.	gene_id "XLOC_207642"; transcript_id "Novel_002882"; exon_number "3"; gene_type "
Dt_chr7	Cufflinks	exon	23268247	23268439	.	+	.	gene_id "XLOC_207642"; transcript_id "Novel_002883"; exon_number "1"; gene_type "
Dt_chr7	Cufflinks	exon	23269068	23269105	.	+	.	gene_id "XLOC_207642"; transcript_id "Novel_002883"; exon_number "2"; gene_type "
Dt_chr7	Cufflinks	exon	23269428	23269799	.	+	.	gene_id "XLOC_207642"; transcript_id "Novel_002883"; exon_number "3"; gene_type "
Dt_chr7	Cufflinks	exon	23270012	23270895	.	+	.	gene_id "XLOC_207642"; transcript_id "Novel_002883"; exon_number "4"; gene_type "
Dt_chr7	Cufflinks	exon	26905860	26906510	.	+	.	gene_id "XLOC_207968"; transcript_id "Novel_002884"; exon_number "1"; gene_type "
Dt_chr7	Cufflinks	exon	26909879	26910430	.	+	.	gene_id "XLOC_207968"; transcript_id "Novel_002884"; exon_number "2"; gene_type "
Dt_chr7	Cufflinks	exon	26910489	26910730	.	+	.	gene_id "XLOC_207968"; transcript_id "Novel_002885"; exon_number "1"; gene_type "
Dt_chr7	Cufflinks	exon	26910814	26910942	.	+	.	gene_id "XLOC_207968"; transcript_id "Novel_002885"; exon_number "2"; gene_type "
Dt_chr7	Cufflinks	exon	26911077	26911796	.	+	.	gene_id "XLOC_207968"; transcript_id "Novel_002885"; exon_number "3"; gene_type "
Dt_chr7	Cufflinks	exon	30209224	30209321	.	+	.	gene_id "XLOC_208225"; transcript_id "Novel_002886"; exon_number "1"; gene_type "
Dt_chr7	Cufflinks	exon	30209674	30209748	.	+	.	gene_id "XLOC_208225"; transcript_id "Novel_002886"; exon_number "2"; gene_type "
Dt_chr7	Cufflinks	exon	30210259	30210340	.	+	.	gene_id "XLOC_208225"; transcript_id "Novel_002886"; exon_number "3"; gene_type "
Dt_chr7	Cufflinks	exon	30211027	30211112	.	+	.	gene_id "XLOC_208225"; transcript_id "Novel_002886"; exon_number "4"; gene_type "
Dt_chr7	Cufflinks	exon	30211213	30211298	.	+	.	gene_id "XLOC_208225"; transcript_id "Novel_002886"; exon_number "5"; gene_type "
Dt_chr7	Cufflinks	exon	30217239	30218624	.	+	.	gene_id "XLOC_208225"; transcript_id "Novel_002886"; exon_number "6"; gene_type "
Dt_chr7	Cufflinks	exon	30209232	30209321	.	+	.	gene_id "XLOC_208225"; transcript_id "Novel_002887"; exon_number "1"; gene_type "
Dt_chr7	Cufflinks	exon	30209674	30209748	.	+	.	gene_id "XLOC_208225"; transcript_id "Novel_002887"; exon_number "2"; gene_type "
Dt_chr7	Cufflinks	exon	30210259	30210340	.	+	.	gene_id "XLOC_208225"; transcript_id "Novel_002887"; exon_number "3"; gene_type "
Dt_chr7	Cufflinks	exon	30211027	30211112	.	+	.	gene_id "XLOC_208225"; transcript_id "Novel_002887"; exon_number "4"; gene_type "
Dt_chr7	Cufflinks	exon	30211213	30211448	.	+	.	gene_id "XLOC_208225"; transcript_id "Novel_002887"; exon_number "5"; gene_type "
Dt_chr7	Cufflinks	exon	30209232	30209321	.	+	.	gene_id "XLOC_208225"; transcript_id "Novel_002888"; exon_number "1"; gene_type "
Dt_chr7	Cufflinks	exon	30209674	30209748	.	+	.	gene_id "XLOC_208225"; transcript_id "Novel_002888"; exon_number "2"; gene_type "
Dt_chr7	Cufflinks	exon	30210259	30211112	.	+	.	gene_id "XLOC_208225"; transcript_id "Novel_002888"; exon_number "3"; gene_type "
Dt_chr7	Cufflinks	exon	30211213	30211298	.	+	.	gene_id "XLOC_208225"; transcript_id "Novel_002888"; exon_number "4"; gene_type "

Dt_chr7	Cufflinks	exon	30217239	30217771	.	+	.	gene_id "XLOC_208225"; transcript_id "Novel_002888"; exon_number "5"; gene_type "
Dt_chr7	Cufflinks	exon	30209232	30209321	.	+	.	gene_id "XLOC_208225"; transcript_id "Novel_002889"; exon_number "1"; gene_type "
Dt_chr7	Cufflinks	exon	30209674	30209748	.	+	.	gene_id "XLOC_208225"; transcript_id "Novel_002889"; exon_number "2"; gene_type "
Dt_chr7	Cufflinks	exon	30210259	30210285	.	+	.	gene_id "XLOC_208225"; transcript_id "Novel_002889"; exon_number "3"; gene_type "
Dt_chr7	Cufflinks	exon	30210998	30211112	.	+	.	gene_id "XLOC_208225"; transcript_id "Novel_002889"; exon_number "4"; gene_type "
Dt_chr7	Cufflinks	exon	30211213	30211298	.	+	.	gene_id "XLOC_208225"; transcript_id "Novel_002889"; exon_number "5"; gene_type "
Dt_chr7	Cufflinks	exon	30217239	30217779	.	+	.	gene_id "XLOC_208225"; transcript_id "Novel_002889"; exon_number "6"; gene_type "
Dt_chr7	Cufflinks	exon	30209238	30209321	.	+	.	gene_id "XLOC_208225"; transcript_id "Novel_002890"; exon_number "1"; gene_type "
Dt_chr7	Cufflinks	exon	30209674	30211112	.	+	.	gene_id "XLOC_208225"; transcript_id "Novel_002890"; exon_number "2"; gene_type "
Dt_chr7	Cufflinks	exon	30211213	30211566	.	+	.	gene_id "XLOC_208225"; transcript_id "Novel_002890"; exon_number "3"; gene_type "
Dt_chr7	Cufflinks	exon	30209240	30209321	.	+	.	gene_id "XLOC_208225"; transcript_id "Novel_002891"; exon_number "1"; gene_type "
Dt_chr7	Cufflinks	exon	30209674	30211122	.	+	.	gene_id "XLOC_208225"; transcript_id "Novel_002891"; exon_number "2"; gene_type "
Dt_chr7	Cufflinks	exon	33683751	33684047	.	+	.	gene_id "XLOC_208451"; transcript_id "Novel_002892"; exon_number "1"; gene_type "
Dt_chr7	Cufflinks	exon	33684157	33684273	.	+	.	gene_id "XLOC_208451"; transcript_id "Novel_002892"; exon_number "2"; gene_type "
Dt_chr7	Cufflinks	exon	33684456	33684705	.	+	.	gene_id "XLOC_208451"; transcript_id "Novel_002892"; exon_number "3"; gene_type "
Dt_chr7	Cufflinks	exon	33683755	33684047	.	+	.	gene_id "XLOC_208451"; transcript_id "Novel_002893"; exon_number "1"; gene_type "
Dt_chr7	Cufflinks	exon	33684157	33685637	.	+	.	gene_id "XLOC_208451"; transcript_id "Novel_002893"; exon_number "2"; gene_type "
Dt_chr7	Cufflinks	exon	34104155	34104448	.	+	.	gene_id "XLOC_208471"; transcript_id "Novel_002894"; exon_number "1"; gene_type "
Dt_chr7	Cufflinks	exon	34104571	34104645	.	+	.	gene_id "XLOC_208471"; transcript_id "Novel_002894"; exon_number "2"; gene_type "
Dt_chr7	Cufflinks	exon	34105040	34105141	.	+	.	gene_id "XLOC_208471"; transcript_id "Novel_002894"; exon_number "3"; gene_type "
Dt_chr7	Cufflinks	exon	34105248	34105833	.	+	.	gene_id "XLOC_208471"; transcript_id "Novel_002894"; exon_number "4"; gene_type "
Dt_chr7	Cufflinks	exon	34105913	34106011	.	+	.	gene_id "XLOC_208471"; transcript_id "Novel_002894"; exon_number "5"; gene_type "
Dt_chr7	Cufflinks	exon	34106102	34106885	.	+	.	gene_id "XLOC_208471"; transcript_id "Novel_002894"; exon_number "6"; gene_type "
Dt_chr7	Cufflinks	exon	34104157	34105141	.	+	.	gene_id "XLOC_208471"; transcript_id "Novel_002895"; exon_number "1"; gene_type "
Dt_chr7	Cufflinks	exon	34105248	34105301	.	+	.	gene_id "XLOC_208471"; transcript_id "Novel_002895"; exon_number "2"; gene_type "
Dt_chr7	Cufflinks	exon	34105389	34105481	.	+	.	gene_id "XLOC_208471"; transcript_id "Novel_002895"; exon_number "3"; gene_type "
Dt_chr7	Cufflinks	exon	34105553	34105645	.	+	.	gene_id "XLOC_208471"; transcript_id "Novel_002895"; exon_number "4"; gene_type "
Dt_chr7	Cufflinks	exon	34105729	34105780	.	+	.	gene_id "XLOC_208471"; transcript_id "Novel_002895"; exon_number "5"; gene_type "
Dt_chr7	Cufflinks	exon	36597697	36597902	.	+	.	gene_id "XLOC_208714"; transcript_id "Novel_002896"; exon_number "1"; gene_type "
Dt_chr7	Cufflinks	exon	36599756	36599978	.	+	.	gene_id "XLOC_208714"; transcript_id "Novel_002896"; exon_number "2"; gene_type "
Dt_chr7	Cufflinks	exon	36600579	36600877	.	+	.	gene_id "XLOC_208714"; transcript_id "Novel_002896"; exon_number "3"; gene_type "
Dt_chr7	Cufflinks	exon	36600993	36601798	.	+	.	gene_id "XLOC_208714"; transcript_id "Novel_002896"; exon_number "4"; gene_type "
Dt_chr7	Cufflinks	exon	37624147	37625456	.	+	.	gene_id "XLOC_208843"; transcript_id "Novel_002897"; exon_number "1"; gene_type "
Dt_chr7	Cufflinks	exon	37748613	37748747	.	+	.	gene_id "XLOC_208848"; transcript_id "Novel_002898"; exon_number "1"; gene_type "
Dt_chr7	Cufflinks	exon	37749265	37749391	.	+	.	gene_id "XLOC_208848"; transcript_id "Novel_002898"; exon_number "2"; gene_type "

Dt_chr7	Cufflinks	exon	37749482	37749554	.	+	.	gene_id "XLOC_208848"; transcript_id "Novel_002898"; exon_number "3"; gene_type "
Dt_chr7	Cufflinks	exon	37749962	37750716	.	+	.	gene_id "XLOC_208848"; transcript_id "Novel_002898"; exon_number "4"; gene_type "
Dt_chr7	Cufflinks	exon	41335926	41336745	.	+	.	gene_id "XLOC_209086"; transcript_id "Novel_002899"; exon_number "1"; gene_type "
Dt_chr7	Cufflinks	exon	41336857	41336946	.	+	.	gene_id "XLOC_209086"; transcript_id "Novel_002899"; exon_number "2"; gene_type "
Dt_chr7	Cufflinks	exon	41337595	41337660	.	+	.	gene_id "XLOC_209086"; transcript_id "Novel_002899"; exon_number "3"; gene_type "
Dt_chr7	Cufflinks	exon	41337744	41337874	.	+	.	gene_id "XLOC_209086"; transcript_id "Novel_002899"; exon_number "4"; gene_type "
Dt_chr7	Cufflinks	exon	41335937	41336946	.	+	.	gene_id "XLOC_209086"; transcript_id "Novel_002900"; exon_number "1"; gene_type "
Dt_chr7	Cufflinks	exon	41337595	41337992	.	+	.	gene_id "XLOC_209086"; transcript_id "Novel_002900"; exon_number "2"; gene_type "
Dt_chr7	Cufflinks	exon	43298749	43298789	.	+	.	gene_id "XLOC_209191"; transcript_id "Novel_002901"; exon_number "1"; gene_type "
Dt_chr7	Cufflinks	exon	43298870	43299378	.	+	.	gene_id "XLOC_209191"; transcript_id "Novel_002901"; exon_number "2"; gene_type "
Dt_chr7	Cufflinks	exon	789369	790124	.	-	.	gene_id "XLOC_209491"; transcript_id "Novel_002902"; exon_number "1"; gene_type "
Dt_chr7	Cufflinks	exon	791356	791689	.	-	.	gene_id "XLOC_209491"; transcript_id "Novel_002902"; exon_number "2"; gene_type "
Dt_chr7	Cufflinks	exon	1046703	1046866	.	-	.	gene_id "XLOC_209507"; transcript_id "Novel_002903"; exon_number "1"; gene_type "
Dt_chr7	Cufflinks	exon	1047320	1047383	.	-	.	gene_id "XLOC_209507"; transcript_id "Novel_002903"; exon_number "2"; gene_type "
Dt_chr7	Cufflinks	exon	1047464	1047541	.	-	.	gene_id "XLOC_209507"; transcript_id "Novel_002903"; exon_number "3"; gene_type "
Dt_chr7	Cufflinks	exon	1047612	1047671	.	-	.	gene_id "XLOC_209507"; transcript_id "Novel_002903"; exon_number "4"; gene_type "
Dt_chr7	Cufflinks	exon	1047851	1047925	.	-	.	gene_id "XLOC_209507"; transcript_id "Novel_002903"; exon_number "5"; gene_type "
Dt_chr7	Cufflinks	exon	1048030	1048259	.	-	.	gene_id "XLOC_209507"; transcript_id "Novel_002903"; exon_number "6"; gene_type "
Dt_chr7	Cufflinks	exon	1053188	1053299	.	-	.	gene_id "XLOC_209507"; transcript_id "Novel_002903"; exon_number "7"; gene_type "
Dt_chr7	Cufflinks	exon	1693734	1694633	.	-	.	gene_id "XLOC_209546"; transcript_id "Novel_002904"; exon_number "1"; gene_type "
Dt_chr7	Cufflinks	exon	1694759	1695075	.	-	.	gene_id "XLOC_209546"; transcript_id "Novel_002904"; exon_number "2"; gene_type "
Dt_chr7	Cufflinks	exon	1695330	1695397	.	-	.	gene_id "XLOC_209546"; transcript_id "Novel_002904"; exon_number "3"; gene_type "
Dt_chr7	Cufflinks	exon	1695605	1695697	.	-	.	gene_id "XLOC_209546"; transcript_id "Novel_002904"; exon_number "4"; gene_type "
Dt_chr7	Cufflinks	exon	1695839	1696049	.	-	.	gene_id "XLOC_209546"; transcript_id "Novel_002904"; exon_number "5"; gene_type "
Dt_chr7	Cufflinks	exon	1748405	1748966	.	-	.	gene_id "XLOC_209559"; transcript_id "Novel_002905"; exon_number "1"; gene_type "
Dt_chr7	Cufflinks	exon	1749094	1749177	.	-	.	gene_id "XLOC_209559"; transcript_id "Novel_002905"; exon_number "2"; gene_type "
Dt_chr7	Cufflinks	exon	1749520	1749590	.	-	.	gene_id "XLOC_209559"; transcript_id "Novel_002905"; exon_number "3"; gene_type "
Dt_chr7	Cufflinks	exon	1749938	1750080	.	-	.	gene_id "XLOC_209559"; transcript_id "Novel_002905"; exon_number "4"; gene_type "
Dt_chr7	Cufflinks	exon	1750194	1750286	.	-	.	gene_id "XLOC_209559"; transcript_id "Novel_002905"; exon_number "5"; gene_type "
Dt_chr7	Cufflinks	exon	1750427	1750480	.	-	.	gene_id "XLOC_209559"; transcript_id "Novel_002905"; exon_number "6"; gene_type "
Dt_chr7	Cufflinks	exon	1750693	1750855	.	-	.	gene_id "XLOC_209559"; transcript_id "Novel_002905"; exon_number "7"; gene_type "
Dt_chr7	Cufflinks	exon	1751103	1751137	.	-	.	gene_id "XLOC_209559"; transcript_id "Novel_002905"; exon_number "8"; gene_type "
Dt_chr7	Cufflinks	exon	1751372	1751554	.	-	.	gene_id "XLOC_209559"; transcript_id "Novel_002905"; exon_number "9"; gene_type "
Dt_chr7	Cufflinks	exon	1751692	1751843	.	-	.	gene_id "XLOC_209559"; transcript_id "Novel_002905"; exon_number "10"; gene_type
Dt_chr7	Cufflinks	exon	1752011	1752127	.	-	.	gene_id "XLOC_209559"; transcript_id "Novel_002905"; exon_number "11"; gene_type

Dt_chr7	Cufflinks	exon	1752334	1752438	.	-	.	gene_id "XLOC_209559"; transcript_id "Novel_002905"; exon_number "12"; gene_type
Dt_chr7	Cufflinks	exon	1752597	1752755	.	-	.	gene_id "XLOC_209559"; transcript_id "Novel_002905"; exon_number "13"; gene_type
Dt_chr7	Cufflinks	exon	1753735	1754008	.	-	.	gene_id "XLOC_209559"; transcript_id "Novel_002905"; exon_number "14"; gene_type
Dt_chr7	Cufflinks	exon	1748694	1748966	.	-	.	gene_id "XLOC_209559"; transcript_id "Novel_002906"; exon_number "1"; gene_type "
Dt_chr7	Cufflinks	exon	1749094	1749177	.	-	.	gene_id "XLOC_209559"; transcript_id "Novel_002906"; exon_number "2"; gene_type "
Dt_chr7	Cufflinks	exon	1749520	1750080	.	-	.	gene_id "XLOC_209559"; transcript_id "Novel_002906"; exon_number "3"; gene_type "
Dt_chr7	Cufflinks	exon	1750194	1750286	.	-	.	gene_id "XLOC_209559"; transcript_id "Novel_002906"; exon_number "4"; gene_type "
Dt_chr7	Cufflinks	exon	1750427	1750480	.	-	.	gene_id "XLOC_209559"; transcript_id "Novel_002906"; exon_number "5"; gene_type "
Dt_chr7	Cufflinks	exon	1750693	1750855	.	-	.	gene_id "XLOC_209559"; transcript_id "Novel_002906"; exon_number "6"; gene_type "
Dt_chr7	Cufflinks	exon	1751103	1751137	.	-	.	gene_id "XLOC_209559"; transcript_id "Novel_002906"; exon_number "7"; gene_type "
Dt_chr7	Cufflinks	exon	1751372	1751554	.	-	.	gene_id "XLOC_209559"; transcript_id "Novel_002906"; exon_number "8"; gene_type "
Dt_chr7	Cufflinks	exon	1751692	1751843	.	-	.	gene_id "XLOC_209559"; transcript_id "Novel_002906"; exon_number "9"; gene_type "
Dt_chr7	Cufflinks	exon	1752011	1752127	.	-	.	gene_id "XLOC_209559"; transcript_id "Novel_002906"; exon_number "10"; gene_type
Dt_chr7	Cufflinks	exon	1752334	1753650	.	-	.	gene_id "XLOC_209559"; transcript_id "Novel_002906"; exon_number "11"; gene_type
Dt_chr7	Cufflinks	exon	2986727	2987473	.	-	.	gene_id "XLOC_209763"; transcript_id "Novel_002907"; exon_number "1"; gene_type "
Dt_chr7	Cufflinks	exon	2987990	2988485	.	-	.	gene_id "XLOC_209763"; transcript_id "Novel_002907"; exon_number "2"; gene_type "
Dt_chr7	Cufflinks	exon	3280069	3280408	.	-	.	gene_id "XLOC_209827"; transcript_id "Novel_002908"; exon_number "1"; gene_type "
Dt_chr7	Cufflinks	exon	3280489	3280535	.	-	.	gene_id "XLOC_209827"; transcript_id "Novel_002908"; exon_number "2"; gene_type "
Dt_chr7	Cufflinks	exon	3280634	3280770	.	-	.	gene_id "XLOC_209827"; transcript_id "Novel_002908"; exon_number "3"; gene_type "
Dt_chr7	Cufflinks	exon	3280899	3280942	.	-	.	gene_id "XLOC_209827"; transcript_id "Novel_002908"; exon_number "4"; gene_type "
Dt_chr7	Cufflinks	exon	3421259	3421841	.	-	.	gene_id "XLOC_209854"; transcript_id "Novel_002909"; exon_number "1"; gene_type "
Dt_chr7	Cufflinks	exon	3422127	3422327	.	-	.	gene_id "XLOC_209854"; transcript_id "Novel_002909"; exon_number "2"; gene_type "
Dt_chr7	Cufflinks	exon	3422438	3424259	.	-	.	gene_id "XLOC_209854"; transcript_id "Novel_002909"; exon_number "3"; gene_type "
Dt_chr7	Cufflinks	exon	3424337	3424610	.	-	.	gene_id "XLOC_209854"; transcript_id "Novel_002909"; exon_number "4"; gene_type "
Dt_chr7	Cufflinks	exon	3426418	3428131	.	-	.	gene_id "XLOC_209854"; transcript_id "Novel_002909"; exon_number "5"; gene_type "
Dt_chr7	Cufflinks	exon	3424196	3424610	.	-	.	gene_id "XLOC_209854"; transcript_id "Novel_002910"; exon_number "1"; gene_type "
Dt_chr7	Cufflinks	exon	3426418	3428143	.	-	.	gene_id "XLOC_209854"; transcript_id "Novel_002910"; exon_number "2"; gene_type "
Dt_chr7	Cufflinks	exon	3722297	3723392	.	-	.	gene_id "XLOC_209906"; transcript_id "Novel_002911"; exon_number "1"; gene_type "
Dt_chr7	Cufflinks	exon	3723559	3723889	.	-	.	gene_id "XLOC_209906"; transcript_id "Novel_002911"; exon_number "2"; gene_type "
Dt_chr7	Cufflinks	exon	3722558	3722793	.	-	.	gene_id "XLOC_209906"; transcript_id "Novel_002912"; exon_number "1"; gene_type "
Dt_chr7	Cufflinks	exon	3723270	3723392	.	-	.	gene_id "XLOC_209906"; transcript_id "Novel_002912"; exon_number "2"; gene_type "
Dt_chr7	Cufflinks	exon	3723559	3723844	.	-	.	gene_id "XLOC_209906"; transcript_id "Novel_002912"; exon_number "3"; gene_type "
Dt_chr7	Cufflinks	exon	3746336	3747258	.	-	.	gene_id "XLOC_209921"; transcript_id "Novel_002913"; exon_number "1"; gene_type "
Dt_chr7	Cufflinks	exon	3747343	3748125	.	-	.	gene_id "XLOC_209921"; transcript_id "Novel_002913"; exon_number "2"; gene_type "
Dt_chr7	Cufflinks	exon	3748209	3748346	.	-	.	gene_id "XLOC_209921"; transcript_id "Novel_002913"; exon_number "3"; gene_type "

Dt_chr7	Cufflinks	exon	3748461	3748533	.	-	.	gene_id "XLOC_209921"; transcript_id "Novel_002913"; exon_number "4"; gene_type "
Dt_chr7	Cufflinks	exon	3748628	3749476	.	-	.	gene_id "XLOC_209921"; transcript_id "Novel_002913"; exon_number "5"; gene_type "
Dt_chr7	Cufflinks	exon	3751405	3751499	.	-	.	gene_id "XLOC_209921"; transcript_id "Novel_002913"; exon_number "6"; gene_type "
Dt_chr7	Cufflinks	exon	3751608	3751670	.	-	.	gene_id "XLOC_209921"; transcript_id "Novel_002913"; exon_number "7"; gene_type "
Dt_chr7	Cufflinks	exon	3752320	3752630	.	-	.	gene_id "XLOC_209921"; transcript_id "Novel_002913"; exon_number "8"; gene_type "
Dt_chr7	Cufflinks	exon	3880893	3881156	.	-	.	gene_id "XLOC_209942"; transcript_id "Novel_002914"; exon_number "1"; gene_type "
Dt_chr7	Cufflinks	exon	3881259	3881417	.	-	.	gene_id "XLOC_209942"; transcript_id "Novel_002914"; exon_number "2"; gene_type "
Dt_chr7	Cufflinks	exon	3881513	3881872	.	-	.	gene_id "XLOC_209942"; transcript_id "Novel_002914"; exon_number "3"; gene_type "
Dt_chr7	Cufflinks	exon	3880926	3881417	.	-	.	gene_id "XLOC_209942"; transcript_id "Novel_002915"; exon_number "1"; gene_type "
Dt_chr7	Cufflinks	exon	3881513	3881692	.	-	.	gene_id "XLOC_209942"; transcript_id "Novel_002915"; exon_number "2"; gene_type "
Dt_chr7	Cufflinks	exon	3888525	3888891	.	-	.	gene_id "XLOC_209944"; transcript_id "Novel_002916"; exon_number "1"; gene_type "
Dt_chr7	Cufflinks	exon	3889012	3889417	.	-	.	gene_id "XLOC_209944"; transcript_id "Novel_002916"; exon_number "2"; gene_type "
Dt_chr7	Cufflinks	exon	3889529	3890023	.	-	.	gene_id "XLOC_209944"; transcript_id "Novel_002916"; exon_number "3"; gene_type "
Dt_chr7	Cufflinks	exon	3888526	3888891	.	-	.	gene_id "XLOC_209944"; transcript_id "Novel_002917"; exon_number "1"; gene_type "
Dt_chr7	Cufflinks	exon	3889012	3889164	.	-	.	gene_id "XLOC_209944"; transcript_id "Novel_002917"; exon_number "2"; gene_type "
Dt_chr7	Cufflinks	exon	3889280	3889417	.	-	.	gene_id "XLOC_209944"; transcript_id "Novel_002917"; exon_number "3"; gene_type "
Dt_chr7	Cufflinks	exon	3889529	3889950	.	-	.	gene_id "XLOC_209944"; transcript_id "Novel_002917"; exon_number "4"; gene_type "
Dt_chr7	Cufflinks	exon	3888526	3888891	.	-	.	gene_id "XLOC_209944"; transcript_id "Novel_002918"; exon_number "1"; gene_type "
Dt_chr7	Cufflinks	exon	3889012	3889164	.	-	.	gene_id "XLOC_209944"; transcript_id "Novel_002918"; exon_number "2"; gene_type "
Dt_chr7	Cufflinks	exon	3889293	3889417	.	-	.	gene_id "XLOC_209944"; transcript_id "Novel_002918"; exon_number "3"; gene_type "
Dt_chr7	Cufflinks	exon	3889529	3890002	.	-	.	gene_id "XLOC_209944"; transcript_id "Novel_002918"; exon_number "4"; gene_type "
Dt_chr7	Cufflinks	exon	3888983	3889164	.	-	.	gene_id "XLOC_209944"; transcript_id "Novel_002919"; exon_number "1"; gene_type "
Dt_chr7	Cufflinks	exon	3889280	3889417	.	-	.	gene_id "XLOC_209944"; transcript_id "Novel_002919"; exon_number "2"; gene_type "
Dt_chr7	Cufflinks	exon	3889529	3890002	.	-	.	gene_id "XLOC_209944"; transcript_id "Novel_002919"; exon_number "3"; gene_type "
Dt_chr7	Cufflinks	exon	3911978	3912654	.	-	.	gene_id "XLOC_209950"; transcript_id "Novel_002920"; exon_number "1"; gene_type "
Dt_chr7	Cufflinks	exon	3912818	3912883	.	-	.	gene_id "XLOC_209950"; transcript_id "Novel_002920"; exon_number "2"; gene_type "
Dt_chr7	Cufflinks	exon	3913386	3913688	.	-	.	gene_id "XLOC_209950"; transcript_id "Novel_002920"; exon_number "3"; gene_type "
Dt_chr7	Cufflinks	exon	3914043	3914163	.	-	.	gene_id "XLOC_209950"; transcript_id "Novel_002920"; exon_number "4"; gene_type "
Dt_chr7	Cufflinks	exon	3914389	3914469	.	-	.	gene_id "XLOC_209950"; transcript_id "Novel_002920"; exon_number "5"; gene_type "
Dt_chr7	Cufflinks	exon	3914564	3914900	.	-	.	gene_id "XLOC_209950"; transcript_id "Novel_002920"; exon_number "6"; gene_type "
Dt_chr7	Cufflinks	exon	3950417	3952041	.	-	.	gene_id "XLOC_209957"; transcript_id "Novel_002921"; exon_number "1"; gene_type "
Dt_chr7	Cufflinks	exon	3952123	3953816	.	-	.	gene_id "XLOC_209957"; transcript_id "Novel_002921"; exon_number "2"; gene_type "
Dt_chr7	Cufflinks	exon	4005843	4006452	.	-	.	gene_id "XLOC_209967"; transcript_id "Novel_002922"; exon_number "1"; gene_type "
Dt_chr7	Cufflinks	exon	4006801	4007058	.	-	.	gene_id "XLOC_209967"; transcript_id "Novel_002922"; exon_number "2"; gene_type "
Dt_chr7	Cufflinks	exon	4007431	4007473	.	-	.	gene_id "XLOC_209967"; transcript_id "Novel_002922"; exon_number "3"; gene_type "

Dt_chr7	Cufflinks	exon	4007569	4007693	.	-	.	gene_id "XLOC_209967"; transcript_id "Novel_002922"; exon_number "4"; gene_type "
Dt_chr7	Cufflinks	exon	4007788	4007877	.	-	.	gene_id "XLOC_209967"; transcript_id "Novel_002922"; exon_number "5"; gene_type "
Dt_chr7	Cufflinks	exon	4008165	4008679	.	-	.	gene_id "XLOC_209967"; transcript_id "Novel_002922"; exon_number "6"; gene_type "
Dt_chr7	Cufflinks	exon	4006067	4006452	.	-	.	gene_id "XLOC_209967"; transcript_id "Novel_002923"; exon_number "1"; gene_type "
Dt_chr7	Cufflinks	exon	4006801	4007058	.	-	.	gene_id "XLOC_209967"; transcript_id "Novel_002923"; exon_number "2"; gene_type "
Dt_chr7	Cufflinks	exon	4007431	4007473	.	-	.	gene_id "XLOC_209967"; transcript_id "Novel_002923"; exon_number "3"; gene_type "
Dt_chr7	Cufflinks	exon	4007569	4007693	.	-	.	gene_id "XLOC_209967"; transcript_id "Novel_002923"; exon_number "4"; gene_type "
Dt_chr7	Cufflinks	exon	4007788	4007877	.	-	.	gene_id "XLOC_209967"; transcript_id "Novel_002923"; exon_number "5"; gene_type "
Dt_chr7	Cufflinks	exon	4008393	4008659	.	-	.	gene_id "XLOC_209967"; transcript_id "Novel_002923"; exon_number "6"; gene_type "
Dt_chr7	Cufflinks	exon	4006098	4006452	.	-	.	gene_id "XLOC_209967"; transcript_id "Novel_002924"; exon_number "1"; gene_type "
Dt_chr7	Cufflinks	exon	4006801	4007397	.	-	.	gene_id "XLOC_209967"; transcript_id "Novel_002924"; exon_number "2"; gene_type "
Dt_chr7	Cufflinks	exon	4031139	4031651	.	-	.	gene_id "XLOC_209972"; transcript_id "Novel_002925"; exon_number "1"; gene_type "
Dt_chr7	Cufflinks	exon	4032301	4032344	.	-	.	gene_id "XLOC_209972"; transcript_id "Novel_002925"; exon_number "2"; gene_type "
Dt_chr7	Cufflinks	exon	4184846	4185847	.	-	.	gene_id "XLOC_210011"; transcript_id "Novel_002926"; exon_number "1"; gene_type "
Dt_chr7	Cufflinks	exon	4185970	4186074	.	-	.	gene_id "XLOC_210011"; transcript_id "Novel_002926"; exon_number "2"; gene_type "
Dt_chr7	Cufflinks	exon	4186171	4186183	.	-	.	gene_id "XLOC_210011"; transcript_id "Novel_002926"; exon_number "3"; gene_type "
Dt_chr7	Cufflinks	exon	4186356	4186452	.	-	.	gene_id "XLOC_210011"; transcript_id "Novel_002927"; exon_number "1"; gene_type "
Dt_chr7	Cufflinks	exon	4187115	4187347	.	-	.	gene_id "XLOC_210011"; transcript_id "Novel_002927"; exon_number "2"; gene_type "
Dt_chr7	Cufflinks	exon	4188246	4188437	.	-	.	gene_id "XLOC_210011"; transcript_id "Novel_002927"; exon_number "3"; gene_type "
Dt_chr7	Cufflinks	exon	4188568	4188748	.	-	.	gene_id "XLOC_210011"; transcript_id "Novel_002927"; exon_number "4"; gene_type "
Dt_chr7	Cufflinks	exon	4188835	4189034	.	-	.	gene_id "XLOC_210011"; transcript_id "Novel_002927"; exon_number "5"; gene_type "
Dt_chr7	Cufflinks	exon	4189476	4190219	.	-	.	gene_id "XLOC_210011"; transcript_id "Novel_002927"; exon_number "6"; gene_type "
Dt_chr7	Cufflinks	exon	4187394	4188437	.	-	.	gene_id "XLOC_210011"; transcript_id "Novel_002928"; exon_number "1"; gene_type "
Dt_chr7	Cufflinks	exon	4188568	4188748	.	-	.	gene_id "XLOC_210011"; transcript_id "Novel_002928"; exon_number "2"; gene_type "
Dt_chr7	Cufflinks	exon	4188835	4189034	.	-	.	gene_id "XLOC_210011"; transcript_id "Novel_002928"; exon_number "3"; gene_type "
Dt_chr7	Cufflinks	exon	4189476	4190202	.	-	.	gene_id "XLOC_210011"; transcript_id "Novel_002928"; exon_number "4"; gene_type "
Dt_chr7	Cufflinks	exon	4280770	4281718	.	-	.	gene_id "XLOC_210027"; transcript_id "Novel_002929"; exon_number "1"; gene_type "
Dt_chr7	Cufflinks	exon	4454151	4454292	.	-	.	gene_id "XLOC_210055"; transcript_id "Novel_002930"; exon_number "1"; gene_type "
Dt_chr7	Cufflinks	exon	4454467	4454594	.	-	.	gene_id "XLOC_210055"; transcript_id "Novel_002930"; exon_number "2"; gene_type "
Dt_chr7	Cufflinks	exon	4801824	4803300	.	-	.	gene_id "XLOC_210124"; transcript_id "Novel_002931"; exon_number "1"; gene_type "
Dt_chr7	Cufflinks	exon	4803804	4804979	.	-	.	gene_id "XLOC_210124"; transcript_id "Novel_002931"; exon_number "2"; gene_type "
Dt_chr7	Cufflinks	exon	5034898	5035686	.	-	.	gene_id "XLOC_210165"; transcript_id "Novel_002932"; exon_number "1"; gene_type "
Dt_chr7	Cufflinks	exon	5035777	5035873	.	-	.	gene_id "XLOC_210165"; transcript_id "Novel_002932"; exon_number "2"; gene_type "
Dt_chr7	Cufflinks	exon	5035971	5036118	.	-	.	gene_id "XLOC_210165"; transcript_id "Novel_002932"; exon_number "3"; gene_type "
Dt_chr7	Cufflinks	exon	5036252	5036635	.	-	.	gene_id "XLOC_210165"; transcript_id "Novel_002932"; exon_number "4"; gene_type "

Dt_chr7	Cufflinks	exon	5034898	5035873	.	-	.	gene_id "XLOC_210165"; transcript_id "Novel_002933"; exon_number "1"; gene_type "
Dt_chr7	Cufflinks	exon	5035971	5036118	.	-	.	gene_id "XLOC_210165"; transcript_id "Novel_002933"; exon_number "2"; gene_type "
Dt_chr7	Cufflinks	exon	5036252	5036635	.	-	.	gene_id "XLOC_210165"; transcript_id "Novel_002933"; exon_number "3"; gene_type "
Dt_chr7	Cufflinks	exon	5063431	5064177	.	-	.	gene_id "XLOC_210168"; transcript_id "Novel_002934"; exon_number "1"; gene_type "
Dt_chr7	Cufflinks	exon	5064272	5064313	.	-	.	gene_id "XLOC_210168"; transcript_id "Novel_002934"; exon_number "2"; gene_type "
Dt_chr7	Cufflinks	exon	5064397	5064498	.	-	.	gene_id "XLOC_210168"; transcript_id "Novel_002934"; exon_number "3"; gene_type "
Dt_chr7	Cufflinks	exon	5064582	5064680	.	-	.	gene_id "XLOC_210168"; transcript_id "Novel_002934"; exon_number "4"; gene_type "
Dt_chr7	Cufflinks	exon	5065077	5065137	.	-	.	gene_id "XLOC_210168"; transcript_id "Novel_002934"; exon_number "5"; gene_type "
Dt_chr7	Cufflinks	exon	5065286	5065407	.	-	.	gene_id "XLOC_210168"; transcript_id "Novel_002934"; exon_number "6"; gene_type "
Dt_chr7	Cufflinks	exon	5065507	5065592	.	-	.	gene_id "XLOC_210168"; transcript_id "Novel_002934"; exon_number "7"; gene_type "
Dt_chr7	Cufflinks	exon	5065748	5065832	.	-	.	gene_id "XLOC_210168"; transcript_id "Novel_002934"; exon_number "8"; gene_type "
Dt_chr7	Cufflinks	exon	5066026	5066532	.	-	.	gene_id "XLOC_210168"; transcript_id "Novel_002934"; exon_number "9"; gene_type "
Dt_chr7	Cufflinks	exon	5066633	5066701	.	-	.	gene_id "XLOC_210168"; transcript_id "Novel_002934"; exon_number "10"; gene_type
Dt_chr7	Cufflinks	exon	5066809	5066889	.	-	.	gene_id "XLOC_210168"; transcript_id "Novel_002934"; exon_number "11"; gene_type
Dt_chr7	Cufflinks	exon	5066980	5067172	.	-	.	gene_id "XLOC_210168"; transcript_id "Novel_002934"; exon_number "12"; gene_type
Dt_chr7	Cufflinks	exon	5073249	5073449	.	-	.	gene_id "XLOC_210170"; transcript_id "Novel_002935"; exon_number "1"; gene_type "
Dt_chr7	Cufflinks	exon	5073915	5074033	.	-	.	gene_id "XLOC_210170"; transcript_id "Novel_002935"; exon_number "2"; gene_type "
Dt_chr7	Cufflinks	exon	5074375	5074426	.	-	.	gene_id "XLOC_210170"; transcript_id "Novel_002935"; exon_number "3"; gene_type "
Dt_chr7	Cufflinks	exon	5180464	5181095	.	-	.	gene_id "XLOC_210177"; transcript_id "Novel_002936"; exon_number "1"; gene_type "
Dt_chr7	Cufflinks	exon	5181388	5181512	.	-	.	gene_id "XLOC_210177"; transcript_id "Novel_002936"; exon_number "2"; gene_type "
Dt_chr7	Cufflinks	exon	5181988	5182048	.	-	.	gene_id "XLOC_210177"; transcript_id "Novel_002936"; exon_number "3"; gene_type "
Dt_chr7	Cufflinks	exon	5182157	5182273	.	-	.	gene_id "XLOC_210177"; transcript_id "Novel_002936"; exon_number "4"; gene_type "
Dt_chr7	Cufflinks	exon	5182415	5182513	.	-	.	gene_id "XLOC_210177"; transcript_id "Novel_002936"; exon_number "5"; gene_type "
Dt_chr7	Cufflinks	exon	5182687	5183834	.	-	.	gene_id "XLOC_210177"; transcript_id "Novel_002936"; exon_number "6"; gene_type "
Dt_chr7	Cufflinks	exon	5180636	5181095	.	-	.	gene_id "XLOC_210177"; transcript_id "Novel_002937"; exon_number "1"; gene_type "
Dt_chr7	Cufflinks	exon	5181388	5181528	.	-	.	gene_id "XLOC_210177"; transcript_id "Novel_002937"; exon_number "2"; gene_type "
Dt_chr7	Cufflinks	exon	5181988	5182048	.	-	.	gene_id "XLOC_210177"; transcript_id "Novel_002937"; exon_number "3"; gene_type "
Dt_chr7	Cufflinks	exon	5182157	5182273	.	-	.	gene_id "XLOC_210177"; transcript_id "Novel_002937"; exon_number "4"; gene_type "
Dt_chr7	Cufflinks	exon	5182415	5182513	.	-	.	gene_id "XLOC_210177"; transcript_id "Novel_002937"; exon_number "5"; gene_type "
Dt_chr7	Cufflinks	exon	5182687	5183812	.	-	.	gene_id "XLOC_210177"; transcript_id "Novel_002937"; exon_number "6"; gene_type "
Dt_chr7	Cufflinks	exon	5232848	5233290	.	-	.	gene_id "XLOC_210184"; transcript_id "Novel_002938"; exon_number "1"; gene_type "
Dt_chr7	Cufflinks	exon	5233376	5233456	.	-	.	gene_id "XLOC_210184"; transcript_id "Novel_002938"; exon_number "2"; gene_type "
Dt_chr7	Cufflinks	exon	5233578	5233653	.	-	.	gene_id "XLOC_210184"; transcript_id "Novel_002938"; exon_number "3"; gene_type "
Dt_chr7	Cufflinks	exon	5233785	5233833	.	-	.	gene_id "XLOC_210184"; transcript_id "Novel_002938"; exon_number "4"; gene_type "
Dt_chr7	Cufflinks	exon	5234265	5234388	.	-	.	gene_id "XLOC_210184"; transcript_id "Novel_002938"; exon_number "5"; gene_type "

Dt_chr7	Cufflinks	exon	5456095	5456594	.	-	.	gene_id	"XLOC_210208";	transcript_id	"Novel_002939";	exon_number	"1";	gene_type	"
Dt_chr7	Cufflinks	exon	5456687	5456882	.	-	.	gene_id	"XLOC_210208";	transcript_id	"Novel_002939";	exon_number	"2";	gene_type	"
Dt_chr7	Cufflinks	exon	5512102	5513302	.	-	.	gene_id	"XLOC_210216";	transcript_id	"Novel_002940";	exon_number	"1";	gene_type	"
Dt_chr7	Cufflinks	exon	5513384	5513513	.	-	.	gene_id	"XLOC_210216";	transcript_id	"Novel_002940";	exon_number	"2";	gene_type	"
Dt_chr7	Cufflinks	exon	5513778	5514010	.	-	.	gene_id	"XLOC_210216";	transcript_id	"Novel_002940";	exon_number	"3";	gene_type	"
Dt_chr7	Cufflinks	exon	5514110	5514147	.	-	.	gene_id	"XLOC_210216";	transcript_id	"Novel_002940";	exon_number	"4";	gene_type	"
Dt_chr7	Cufflinks	exon	5514233	5514572	.	-	.	gene_id	"XLOC_210216";	transcript_id	"Novel_002940";	exon_number	"5";	gene_type	"
Dt_chr7	Cufflinks	exon	5512988	5513302	.	-	.	gene_id	"XLOC_210216";	transcript_id	"Novel_002941";	exon_number	"1";	gene_type	"
Dt_chr7	Cufflinks	exon	5513384	5513513	.	-	.	gene_id	"XLOC_210216";	transcript_id	"Novel_002941";	exon_number	"2";	gene_type	"
Dt_chr7	Cufflinks	exon	5513778	5514010	.	-	.	gene_id	"XLOC_210216";	transcript_id	"Novel_002941";	exon_number	"3";	gene_type	"
Dt_chr7	Cufflinks	exon	5514110	5514557	.	-	.	gene_id	"XLOC_210216";	transcript_id	"Novel_002941";	exon_number	"4";	gene_type	"
Dt_chr7	Cufflinks	exon	5512988	5513302	.	-	.	gene_id	"XLOC_210216";	transcript_id	"Novel_002942";	exon_number	"1";	gene_type	"
Dt_chr7	Cufflinks	exon	5513384	5513513	.	-	.	gene_id	"XLOC_210216";	transcript_id	"Novel_002942";	exon_number	"2";	gene_type	"
Dt_chr7	Cufflinks	exon	5513778	5514557	.	-	.	gene_id	"XLOC_210216";	transcript_id	"Novel_002942";	exon_number	"3";	gene_type	"
Dt_chr7	Cufflinks	exon	5512994	5513513	.	-	.	gene_id	"XLOC_210216";	transcript_id	"Novel_002943";	exon_number	"1";	gene_type	"
Dt_chr7	Cufflinks	exon	5513778	5514010	.	-	.	gene_id	"XLOC_210216";	transcript_id	"Novel_002943";	exon_number	"2";	gene_type	"
Dt_chr7	Cufflinks	exon	5514110	5514147	.	-	.	gene_id	"XLOC_210216";	transcript_id	"Novel_002943";	exon_number	"3";	gene_type	"
Dt_chr7	Cufflinks	exon	5514233	5514519	.	-	.	gene_id	"XLOC_210216";	transcript_id	"Novel_002943";	exon_number	"4";	gene_type	"
Dt_chr7	Cufflinks	exon	5533650	5533842	.	-	.	gene_id	"XLOC_210217";	transcript_id	"Novel_002944";	exon_number	"1";	gene_type	"
Dt_chr7	Cufflinks	exon	5534110	5534194	.	-	.	gene_id	"XLOC_210217";	transcript_id	"Novel_002944";	exon_number	"2";	gene_type	"
Dt_chr7	Cufflinks	exon	5534509	5535125	.	-	.	gene_id	"XLOC_210217";	transcript_id	"Novel_002944";	exon_number	"3";	gene_type	"
Dt_chr7	Cufflinks	exon	5535449	5535885	.	-	.	gene_id	"XLOC_210217";	transcript_id	"Novel_002944";	exon_number	"4";	gene_type	"
Dt_chr7	Cufflinks	exon	5537686	5537731	.	-	.	gene_id	"XLOC_210226";	transcript_id	"Novel_002945";	exon_number	"1";	gene_type	"
Dt_chr7	Cufflinks	exon	5538029	5538196	.	-	.	gene_id	"XLOC_210226";	transcript_id	"Novel_002945";	exon_number	"2";	gene_type	"
Dt_chr7	Cufflinks	exon	5538027	5538718	.	-	.	gene_id	"XLOC_210226";	transcript_id	"Novel_002946";	exon_number	"1";	gene_type	"
Dt_chr7	Cufflinks	exon	5539140	5539233	.	-	.	gene_id	"XLOC_210226";	transcript_id	"Novel_002946";	exon_number	"2";	gene_type	"
Dt_chr7	Cufflinks	exon	5542001	5542531	.	-	.	gene_id	"XLOC_210229";	transcript_id	"Novel_002947";	exon_number	"1";	gene_type	"
Dt_chr7	Cufflinks	exon	5543194	5543502	.	-	.	gene_id	"XLOC_210229";	transcript_id	"Novel_002947";	exon_number	"2";	gene_type	"
Dt_chr7	Cufflinks	exon	5626486	5627326	.	-	.	gene_id	"XLOC_210245";	transcript_id	"Novel_002948";	exon_number	"1";	gene_type	"
Dt_chr7	Cufflinks	exon	5627398	5627445	.	-	.	gene_id	"XLOC_210245";	transcript_id	"Novel_002948";	exon_number	"2";	gene_type	"
Dt_chr7	Cufflinks	exon	5627568	5628347	.	-	.	gene_id	"XLOC_210245";	transcript_id	"Novel_002948";	exon_number	"3";	gene_type	"
Dt_chr7	Cufflinks	exon	5658783	5660918	.	-	.	gene_id	"XLOC_210251";	transcript_id	"Novel_002949";	exon_number	"1";	gene_type	"
Dt_chr7	Cufflinks	exon	5867716	5868507	.	-	.	gene_id	"XLOC_210301";	transcript_id	"Novel_002950";	exon_number	"1";	gene_type	"
Dt_chr7	Cufflinks	exon	5868597	5869621	.	-	.	gene_id	"XLOC_210301";	transcript_id	"Novel_002950";	exon_number	"2";	gene_type	"
Dt_chr7	Cufflinks	exon	5870001	5870391	.	-	.	gene_id	"XLOC_210301";	transcript_id	"Novel_002950";	exon_number	"3";	gene_type	"

Dt_chr7	Cufflinks	exon	5870975	5871087	.	-	.	gene_id "XLOC_210301"; transcript_id "Novel_002950"; exon_number "4"; gene_type "
Dt_chr7	Cufflinks	exon	5868381	5868507	.	-	.	gene_id "XLOC_210301"; transcript_id "Novel_002951"; exon_number "1"; gene_type "
Dt_chr7	Cufflinks	exon	5868597	5868949	.	-	.	gene_id "XLOC_210301"; transcript_id "Novel_002951"; exon_number "2"; gene_type "
Dt_chr7	Cufflinks	exon	5869355	5869377	.	-	.	gene_id "XLOC_210301"; transcript_id "Novel_002951"; exon_number "3"; gene_type "
Dt_chr7	Cufflinks	exon	5869325	5870391	.	-	.	gene_id "XLOC_210301"; transcript_id "Novel_002952"; exon_number "1"; gene_type "
Dt_chr7	Cufflinks	exon	5870975	5871071	.	-	.	gene_id "XLOC_210301"; transcript_id "Novel_002952"; exon_number "2"; gene_type "
Dt_chr7	Cufflinks	exon	5998878	5999565	.	-	.	gene_id "XLOC_210326"; transcript_id "Novel_002953"; exon_number "1"; gene_type "
Dt_chr7	Cufflinks	exon	7356429	7356474	.	-	.	gene_id "XLOC_210550"; transcript_id "Novel_002954"; exon_number "1"; gene_type "
Dt_chr7	Cufflinks	exon	7357226	7357289	.	-	.	gene_id "XLOC_210550"; transcript_id "Novel_002954"; exon_number "2"; gene_type "
Dt_chr7	Cufflinks	exon	7357382	7357428	.	-	.	gene_id "XLOC_210550"; transcript_id "Novel_002954"; exon_number "3"; gene_type "
Dt_chr7	Cufflinks	exon	7358313	7358463	.	-	.	gene_id "XLOC_210550"; transcript_id "Novel_002954"; exon_number "4"; gene_type "
Dt_chr7	Cufflinks	exon	7357177	7357289	.	-	.	gene_id "XLOC_210550"; transcript_id "Novel_002955"; exon_number "1"; gene_type "
Dt_chr7	Cufflinks	exon	7357382	7357428	.	-	.	gene_id "XLOC_210550"; transcript_id "Novel_002955"; exon_number "2"; gene_type "
Dt_chr7	Cufflinks	exon	7358313	7358460	.	-	.	gene_id "XLOC_210550"; transcript_id "Novel_002955"; exon_number "3"; gene_type "
Dt_chr7	Cufflinks	exon	7390992	7391850	.	-	.	gene_id "XLOC_210555"; transcript_id "Novel_002956"; exon_number "1"; gene_type "
Dt_chr7	Cufflinks	exon	7391922	7392530	.	-	.	gene_id "XLOC_210555"; transcript_id "Novel_002956"; exon_number "2"; gene_type "
Dt_chr7	Cufflinks	exon	8853333	8853925	.	-	.	gene_id "XLOC_210808"; transcript_id "Novel_002957"; exon_number "1"; gene_type "
Dt_chr7	Cufflinks	exon	8854025	8854592	.	-	.	gene_id "XLOC_210808"; transcript_id "Novel_002957"; exon_number "2"; gene_type "
Dt_chr7	Cufflinks	exon	8853333	8853925	.	-	.	gene_id "XLOC_210808"; transcript_id "Novel_002958"; exon_number "1"; gene_type "
Dt_chr7	Cufflinks	exon	8854025	8854499	.	-	.	gene_id "XLOC_210808"; transcript_id "Novel_002958"; exon_number "2"; gene_type "
Dt_chr7	Cufflinks	exon	8854643	8854687	.	-	.	gene_id "XLOC_210808"; transcript_id "Novel_002958"; exon_number "3"; gene_type "
Dt_chr7	Cufflinks	exon	8854008	8854496	.	-	.	gene_id "XLOC_210808"; transcript_id "Novel_002959"; exon_number "1"; gene_type "
Dt_chr7	Cufflinks	exon	8854643	8854670	.	-	.	gene_id "XLOC_210808"; transcript_id "Novel_002959"; exon_number "2"; gene_type "
Dt_chr7	Cufflinks	exon	9125956	9126332	.	-	.	gene_id "XLOC_210851"; transcript_id "Novel_002960"; exon_number "1"; gene_type "
Dt_chr7	Cufflinks	exon	9126472	9128405	.	-	.	gene_id "XLOC_210851"; transcript_id "Novel_002960"; exon_number "2"; gene_type "
Dt_chr7	Cufflinks	exon	9128888	9129356	.	-	.	gene_id "XLOC_210851"; transcript_id "Novel_002960"; exon_number "3"; gene_type "
Dt_chr7	Cufflinks	exon	9125956	9126332	.	-	.	gene_id "XLOC_210851"; transcript_id "Novel_002961"; exon_number "1"; gene_type "
Dt_chr7	Cufflinks	exon	9126472	9129356	.	-	.	gene_id "XLOC_210851"; transcript_id "Novel_002961"; exon_number "2"; gene_type "
Dt_chr7	Cufflinks	exon	9180139	9181041	.	-	.	gene_id "XLOC_210860"; transcript_id "Novel_002962"; exon_number "1"; gene_type "
Dt_chr7	Cufflinks	exon	9181134	9181318	.	-	.	gene_id "XLOC_210860"; transcript_id "Novel_002962"; exon_number "2"; gene_type "
Dt_chr7	Cufflinks	exon	9181449	9181665	.	-	.	gene_id "XLOC_210860"; transcript_id "Novel_002962"; exon_number "3"; gene_type "
Dt_chr7	Cufflinks	exon	9182229	9184199	.	-	.	gene_id "XLOC_210860"; transcript_id "Novel_002962"; exon_number "4"; gene_type "
Dt_chr7	Cufflinks	exon	9275241	9275936	.	-	.	gene_id "XLOC_210865"; transcript_id "Novel_002963"; exon_number "1"; gene_type "
Dt_chr7	Cufflinks	exon	9276176	9276313	.	-	.	gene_id "XLOC_210865"; transcript_id "Novel_002963"; exon_number "2"; gene_type "
Dt_chr7	Cufflinks	exon	9276441	9276627	.	-	.	gene_id "XLOC_210865"; transcript_id "Novel_002963"; exon_number "3"; gene_type "

Dt_chr7	Cufflinks	exon	9275241	9275936	.	-	.	gene_id "XLOC_210865"; transcript_id "Novel_002964"; exon_number "1"; gene_type "
Dt_chr7	Cufflinks	exon	9276176	9276316	.	-	.	gene_id "XLOC_210865"; transcript_id "Novel_002964"; exon_number "2"; gene_type "
Dt_chr7	Cufflinks	exon	9276441	9276529	.	-	.	gene_id "XLOC_210865"; transcript_id "Novel_002964"; exon_number "3"; gene_type "
Dt_chr7	Cufflinks	exon	9276623	9276648	.	-	.	gene_id "XLOC_210865"; transcript_id "Novel_002964"; exon_number "4"; gene_type "
Dt_chr7	Cufflinks	exon	9275241	9275936	.	-	.	gene_id "XLOC_210865"; transcript_id "Novel_002965"; exon_number "1"; gene_type "
Dt_chr7	Cufflinks	exon	9276176	9276313	.	-	.	gene_id "XLOC_210865"; transcript_id "Novel_002965"; exon_number "2"; gene_type "
Dt_chr7	Cufflinks	exon	9276441	9276536	.	-	.	gene_id "XLOC_210865"; transcript_id "Novel_002965"; exon_number "3"; gene_type "
Dt_chr7	Cufflinks	exon	9276623	9276737	.	-	.	gene_id "XLOC_210865"; transcript_id "Novel_002965"; exon_number "4"; gene_type "
Dt_chr7	Cufflinks	exon	9275252	9275936	.	-	.	gene_id "XLOC_210865"; transcript_id "Novel_002966"; exon_number "1"; gene_type "
Dt_chr7	Cufflinks	exon	9276176	9276313	.	-	.	gene_id "XLOC_210865"; transcript_id "Novel_002966"; exon_number "2"; gene_type "
Dt_chr7	Cufflinks	exon	9276441	9276529	.	-	.	gene_id "XLOC_210865"; transcript_id "Novel_002966"; exon_number "3"; gene_type "
Dt_chr7	Cufflinks	exon	9276623	9276646	.	-	.	gene_id "XLOC_210865"; transcript_id "Novel_002966"; exon_number "4"; gene_type "
Dt_chr7	Cufflinks	exon	9275306	9275936	.	-	.	gene_id "XLOC_210865"; transcript_id "Novel_002967"; exon_number "1"; gene_type "
Dt_chr7	Cufflinks	exon	9276176	9276316	.	-	.	gene_id "XLOC_210865"; transcript_id "Novel_002967"; exon_number "2"; gene_type "
Dt_chr7	Cufflinks	exon	9276441	9276678	.	-	.	gene_id "XLOC_210865"; transcript_id "Novel_002967"; exon_number "3"; gene_type "
Dt_chr7	Cufflinks	exon	9275321	9275936	.	-	.	gene_id "XLOC_210865"; transcript_id "Novel_002968"; exon_number "1"; gene_type "
Dt_chr7	Cufflinks	exon	9276176	9276692	.	-	.	gene_id "XLOC_210865"; transcript_id "Novel_002968"; exon_number "2"; gene_type "
Dt_chr7	Cufflinks	exon	9308194	9308937	.	-	.	gene_id "XLOC_210869"; transcript_id "Novel_002969"; exon_number "1"; gene_type "
Dt_chr7	Cufflinks	exon	9309592	9310105	.	-	.	gene_id "XLOC_210869"; transcript_id "Novel_002969"; exon_number "2"; gene_type "
Dt_chr7	Cufflinks	exon	9315850	9317653	.	-	.	gene_id "XLOC_210873"; transcript_id "Novel_002970"; exon_number "1"; gene_type "
Dt_chr7	Cufflinks	exon	9317988	9319075	.	-	.	gene_id "XLOC_210873"; transcript_id "Novel_002970"; exon_number "2"; gene_type "
Dt_chr7	Cufflinks	exon	10670092	10673336	.	-	.	gene_id "XLOC_211037"; transcript_id "Novel_002971"; exon_number "1"; gene_type "
Dt_chr7	Cufflinks	exon	10673750	10673906	.	-	.	gene_id "XLOC_211037"; transcript_id "Novel_002971"; exon_number "2"; gene_type "
Dt_chr7	Cufflinks	exon	10670911	10673339	.	-	.	gene_id "XLOC_211037"; transcript_id "Novel_002972"; exon_number "1"; gene_type "
Dt_chr7	Cufflinks	exon	10673750	10673915	.	-	.	gene_id "XLOC_211037"; transcript_id "Novel_002972"; exon_number "2"; gene_type "
Dt_chr7	Cufflinks	exon	11819694	11820046	.	-	.	gene_id "XLOC_211216"; transcript_id "Novel_002973"; exon_number "1"; gene_type "
Dt_chr7	Cufflinks	exon	11820175	11821004	.	-	.	gene_id "XLOC_211216"; transcript_id "Novel_002973"; exon_number "2"; gene_type "
Dt_chr7	Cufflinks	exon	11821281	11821458	.	-	.	gene_id "XLOC_211216"; transcript_id "Novel_002973"; exon_number "3"; gene_type "
Dt_chr7	Cufflinks	exon	11821543	11821982	.	-	.	gene_id "XLOC_211216"; transcript_id "Novel_002973"; exon_number "4"; gene_type "
Dt_chr7	Cufflinks	exon	11819706	11820046	.	-	.	gene_id "XLOC_211216"; transcript_id "Novel_002974"; exon_number "1"; gene_type "
Dt_chr7	Cufflinks	exon	11820175	11820303	.	-	.	gene_id "XLOC_211216"; transcript_id "Novel_002974"; exon_number "2"; gene_type "
Dt_chr7	Cufflinks	exon	11820538	11821004	.	-	.	gene_id "XLOC_211216"; transcript_id "Novel_002974"; exon_number "3"; gene_type "
Dt_chr7	Cufflinks	exon	11821281	11821458	.	-	.	gene_id "XLOC_211216"; transcript_id "Novel_002974"; exon_number "4"; gene_type "
Dt_chr7	Cufflinks	exon	11821543	11822015	.	-	.	gene_id "XLOC_211216"; transcript_id "Novel_002974"; exon_number "5"; gene_type "
Dt_chr7	Cufflinks	exon	11819707	11820046	.	-	.	gene_id "XLOC_211216"; transcript_id "Novel_002975"; exon_number "1"; gene_type "

Dt_chr7	Cufflinks	exon	11820175	11820303	.	-	.	gene_id "XLOC_211216"; transcript_id "Novel_002975"; exon_number "2"; gene_type "
Dt_chr7	Cufflinks	exon	11820538	11821004	.	-	.	gene_id "XLOC_211216"; transcript_id "Novel_002975"; exon_number "3"; gene_type "
Dt_chr7	Cufflinks	exon	11821281	11821999	.	-	.	gene_id "XLOC_211216"; transcript_id "Novel_002975"; exon_number "4"; gene_type "
Dt_chr7	Cufflinks	exon	12431642	12432796	.	-	.	gene_id "XLOC_211305"; transcript_id "Novel_002976"; exon_number "1"; gene_type "
Dt_chr7	Cufflinks	exon	12433688	12433814	.	-	.	gene_id "XLOC_211305"; transcript_id "Novel_002976"; exon_number "2"; gene_type "
Dt_chr7	Cufflinks	exon	15082085	15083579	.	-	.	gene_id "XLOC_211617"; transcript_id "Novel_002977"; exon_number "1"; gene_type "
Dt_chr7	Cufflinks	exon	15084240	15084293	.	-	.	gene_id "XLOC_211617"; transcript_id "Novel_002977"; exon_number "2"; gene_type "
Dt_chr7	Cufflinks	exon	15084377	15085507	.	-	.	gene_id "XLOC_211617"; transcript_id "Novel_002977"; exon_number "3"; gene_type "
Dt_chr7	Cufflinks	exon	15082542	15083579	.	-	.	gene_id "XLOC_211617"; transcript_id "Novel_002978"; exon_number "1"; gene_type "
Dt_chr7	Cufflinks	exon	15084240	15084293	.	-	.	gene_id "XLOC_211617"; transcript_id "Novel_002978"; exon_number "2"; gene_type "
Dt_chr7	Cufflinks	exon	15084377	15084421	.	-	.	gene_id "XLOC_211617"; transcript_id "Novel_002978"; exon_number "3"; gene_type "
Dt_chr7	Cufflinks	exon	15085663	15086047	.	-	.	gene_id "XLOC_211617"; transcript_id "Novel_002978"; exon_number "4"; gene_type "
Dt_chr7	Cufflinks	exon	16499077	16499468	.	-	.	gene_id "XLOC_211759"; transcript_id "Novel_002979"; exon_number "1"; gene_type "
Dt_chr7	Cufflinks	exon	16499731	16499835	.	-	.	gene_id "XLOC_211759"; transcript_id "Novel_002979"; exon_number "2"; gene_type "
Dt_chr7	Cufflinks	exon	16499912	16499971	.	-	.	gene_id "XLOC_211759"; transcript_id "Novel_002979"; exon_number "3"; gene_type "
Dt_chr7	Cufflinks	exon	16500066	16500177	.	-	.	gene_id "XLOC_211759"; transcript_id "Novel_002979"; exon_number "4"; gene_type "
Dt_chr7	Cufflinks	exon	16502934	16503290	.	-	.	gene_id "XLOC_211759"; transcript_id "Novel_002979"; exon_number "5"; gene_type "
Dt_chr7	Cufflinks	exon	16503531	16503568	.	-	.	gene_id "XLOC_211759"; transcript_id "Novel_002979"; exon_number "6"; gene_type "
Dt_chr7	Cufflinks	exon	16504544	16504580	.	-	.	gene_id "XLOC_211759"; transcript_id "Novel_002979"; exon_number "7"; gene_type "
Dt_chr7	Cufflinks	exon	16499140	16499468	.	-	.	gene_id "XLOC_211759"; transcript_id "Novel_002980"; exon_number "1"; gene_type "
Dt_chr7	Cufflinks	exon	16499731	16499835	.	-	.	gene_id "XLOC_211759"; transcript_id "Novel_002980"; exon_number "2"; gene_type "
Dt_chr7	Cufflinks	exon	16499912	16499971	.	-	.	gene_id "XLOC_211759"; transcript_id "Novel_002980"; exon_number "3"; gene_type "
Dt_chr7	Cufflinks	exon	16500066	16500177	.	-	.	gene_id "XLOC_211759"; transcript_id "Novel_002980"; exon_number "4"; gene_type "
Dt_chr7	Cufflinks	exon	16502934	16503290	.	-	.	gene_id "XLOC_211759"; transcript_id "Novel_002980"; exon_number "5"; gene_type "
Dt_chr7	Cufflinks	exon	16504544	16504582	.	-	.	gene_id "XLOC_211759"; transcript_id "Novel_002980"; exon_number "6"; gene_type "
Dt_chr7	Cufflinks	exon	16499176	16499468	.	-	.	gene_id "XLOC_211759"; transcript_id "Novel_002981"; exon_number "1"; gene_type "
Dt_chr7	Cufflinks	exon	16499731	16499835	.	-	.	gene_id "XLOC_211759"; transcript_id "Novel_002981"; exon_number "2"; gene_type "
Dt_chr7	Cufflinks	exon	16499912	16499971	.	-	.	gene_id "XLOC_211759"; transcript_id "Novel_002981"; exon_number "3"; gene_type "
Dt_chr7	Cufflinks	exon	16500066	16500177	.	-	.	gene_id "XLOC_211759"; transcript_id "Novel_002981"; exon_number "4"; gene_type "
Dt_chr7	Cufflinks	exon	16502934	16503398	.	-	.	gene_id "XLOC_211759"; transcript_id "Novel_002981"; exon_number "5"; gene_type "
Dt_chr7	Cufflinks	exon	22507308	22508490	.	-	.	gene_id "XLOC_212344"; transcript_id "Novel_002982"; exon_number "1"; gene_type "
Dt_chr7	Cufflinks	exon	22508725	22508787	.	-	.	gene_id "XLOC_212344"; transcript_id "Novel_002982"; exon_number "2"; gene_type "
Dt_chr7	Cufflinks	exon	22510064	22510351	.	-	.	gene_id "XLOC_212344"; transcript_id "Novel_002982"; exon_number "3"; gene_type "
Dt_chr7	Cufflinks	exon	23473155	23473480	.	-	.	gene_id "XLOC_212457"; transcript_id "Novel_002983"; exon_number "1"; gene_type "
Dt_chr7	Cufflinks	exon	23474422	23474906	.	-	.	gene_id "XLOC_212457"; transcript_id "Novel_002983"; exon_number "2"; gene_type "

Dt_chr7	Cufflinks	exon	23504576	23504819	.	-	.	gene_id "XLOC_212465"; transcript_id "Novel_002984"; exon_number "1"; gene_type "
Dt_chr7	Cufflinks	exon	23524132	23525531	.	-	.	gene_id "XLOC_212469"; transcript_id "Novel_002985"; exon_number "1"; gene_type "
Dt_chr7	Cufflinks	exon	23525677	23526367	.	-	.	gene_id "XLOC_212469"; transcript_id "Novel_002985"; exon_number "2"; gene_type "
Dt_chr7	Cufflinks	exon	23655919	23656554	.	-	.	gene_id "XLOC_212502"; transcript_id "Novel_002986"; exon_number "1"; gene_type "
Dt_chr7	Cufflinks	exon	23656955	23657098	.	-	.	gene_id "XLOC_212502"; transcript_id "Novel_002986"; exon_number "2"; gene_type "
Dt_chr7	Cufflinks	exon	23657374	23657457	.	-	.	gene_id "XLOC_212502"; transcript_id "Novel_002986"; exon_number "3"; gene_type "
Dt_chr7	Cufflinks	exon	23657805	23658133	.	-	.	gene_id "XLOC_212502"; transcript_id "Novel_002986"; exon_number "4"; gene_type "
Dt_chr7	Cufflinks	exon	23658222	23658304	.	-	.	gene_id "XLOC_212502"; transcript_id "Novel_002986"; exon_number "5"; gene_type "
Dt_chr7	Cufflinks	exon	23658735	23658883	.	-	.	gene_id "XLOC_212502"; transcript_id "Novel_002986"; exon_number "6"; gene_type "
Dt_chr7	Cufflinks	exon	23658958	23659064	.	-	.	gene_id "XLOC_212502"; transcript_id "Novel_002986"; exon_number "7"; gene_type "
Dt_chr7	Cufflinks	exon	23659208	23659297	.	-	.	gene_id "XLOC_212502"; transcript_id "Novel_002986"; exon_number "8"; gene_type "
Dt_chr7	Cufflinks	exon	23659377	23659505	.	-	.	gene_id "XLOC_212502"; transcript_id "Novel_002986"; exon_number "9"; gene_type "
Dt_chr7	Cufflinks	exon	23659700	23659867	.	-	.	gene_id "XLOC_212502"; transcript_id "Novel_002986"; exon_number "10"; gene_type
Dt_chr7	Cufflinks	exon	23659976	23660073	.	-	.	gene_id "XLOC_212502"; transcript_id "Novel_002986"; exon_number "11"; gene_type
Dt_chr7	Cufflinks	exon	23655921	23656554	.	-	.	gene_id "XLOC_212502"; transcript_id "Novel_002987"; exon_number "1"; gene_type "
Dt_chr7	Cufflinks	exon	23656955	23657098	.	-	.	gene_id "XLOC_212502"; transcript_id "Novel_002987"; exon_number "2"; gene_type "
Dt_chr7	Cufflinks	exon	23657374	23657457	.	-	.	gene_id "XLOC_212502"; transcript_id "Novel_002987"; exon_number "3"; gene_type "
Dt_chr7	Cufflinks	exon	23657805	23658344	.	-	.	gene_id "XLOC_212502"; transcript_id "Novel_002987"; exon_number "4"; gene_type "
Dt_chr7	Cufflinks	exon	23660205	23660898	.	-	.	gene_id "XLOC_212502"; transcript_id "Novel_002988"; exon_number "1"; gene_type "
Dt_chr7	Cufflinks	exon	23662562	23662962	.	-	.	gene_id "XLOC_212502"; transcript_id "Novel_002988"; exon_number "2"; gene_type "
Dt_chr7	Cufflinks	exon	23663101	23663175	.	-	.	gene_id "XLOC_212503"; transcript_id "Novel_002989"; exon_number "1"; gene_type "
Dt_chr7	Cufflinks	exon	23663270	23663446	.	-	.	gene_id "XLOC_212503"; transcript_id "Novel_002989"; exon_number "2"; gene_type "
Dt_chr7	Cufflinks	exon	23663159	23663729	.	-	.	gene_id "XLOC_212503"; transcript_id "Novel_002990"; exon_number "1"; gene_type "
Dt_chr7	Cufflinks	exon	23663825	23664871	.	-	.	gene_id "XLOC_212503"; transcript_id "Novel_002990"; exon_number "2"; gene_type "
Dt_chr7	Cufflinks	exon	27670106	27671132	.	-	.	gene_id "XLOC_212821"; transcript_id "Novel_002991"; exon_number "1"; gene_type "
Dt_chr7	Cufflinks	exon	27673680	27673963	.	-	.	gene_id "XLOC_212821"; transcript_id "Novel_002991"; exon_number "2"; gene_type "
Dt_chr7	Cufflinks	exon	28716647	28716881	.	-	.	gene_id "XLOC_212878"; transcript_id "Novel_002992"; exon_number "1"; gene_type "
Dt_chr7	Cufflinks	exon	28716970	28717031	.	-	.	gene_id "XLOC_212878"; transcript_id "Novel_002992"; exon_number "2"; gene_type "
Dt_chr7	Cufflinks	exon	28721741	28721789	.	-	.	gene_id "XLOC_212878"; transcript_id "Novel_002992"; exon_number "3"; gene_type "
Dt_chr7	Cufflinks	exon	28717740	28721518	.	-	.	gene_id "XLOC_212878"; transcript_id "Novel_002993"; exon_number "1"; gene_type "
Dt_chr7	Cufflinks	exon	28721741	28721835	.	-	.	gene_id "XLOC_212878"; transcript_id "Novel_002993"; exon_number "2"; gene_type "
Dt_chr7	Cufflinks	exon	29781117	29781193	.	-	.	gene_id "XLOC_212964"; transcript_id "Novel_002994"; exon_number "1"; gene_type "
Dt_chr7	Cufflinks	exon	29781282	29781760	.	-	.	gene_id "XLOC_212964"; transcript_id "Novel_002994"; exon_number "2"; gene_type "
Dt_chr7	Cufflinks	exon	29880076	29880571	.	-	.	gene_id "XLOC_212971"; transcript_id "Novel_002995"; exon_number "1"; gene_type "
Dt_chr7	Cufflinks	exon	29881781	29882078	.	-	.	gene_id "XLOC_212971"; transcript_id "Novel_002995"; exon_number "2"; gene_type "

Dt_chr7	Cufflinks	exon	29882167	29882241	.	-	.	gene_id "XLOC_212971"; transcript_id "Novel_002995"; exon_number "3"; gene_type "
Dt_chr7	Cufflinks	exon	29881015	29882078	.	-	.	gene_id "XLOC_212971"; transcript_id "Novel_002996"; exon_number "1"; gene_type "
Dt_chr7	Cufflinks	exon	29882167	29882241	.	-	.	gene_id "XLOC_212971"; transcript_id "Novel_002996"; exon_number "2"; gene_type "
Dt_chr7	Cufflinks	exon	31781297	31781502	.	-	.	gene_id "XLOC_213081"; transcript_id "Novel_002997"; exon_number "1"; gene_type "
Dt_chr7	Cufflinks	exon	34436921	34437419	.	-	.	gene_id "XLOC_213233"; transcript_id "Novel_002998"; exon_number "1"; gene_type "
Dt_chr7	Cufflinks	exon	34437600	34437698	.	-	.	gene_id "XLOC_213233"; transcript_id "Novel_002998"; exon_number "2"; gene_type "
Dt_chr7	Cufflinks	exon	34437801	34437854	.	-	.	gene_id "XLOC_213233"; transcript_id "Novel_002998"; exon_number "3"; gene_type "
Dt_chr7	Cufflinks	exon	34438102	34438165	.	-	.	gene_id "XLOC_213233"; transcript_id "Novel_002998"; exon_number "4"; gene_type "
Dt_chr7	Cufflinks	exon	34438416	34438453	.	-	.	gene_id "XLOC_213233"; transcript_id "Novel_002998"; exon_number "5"; gene_type "
Dt_chr7	Cufflinks	exon	34438548	34438606	.	-	.	gene_id "XLOC_213233"; transcript_id "Novel_002998"; exon_number "6"; gene_type "
Dt_chr7	Cufflinks	exon	34438696	34438735	.	-	.	gene_id "XLOC_213233"; transcript_id "Novel_002998"; exon_number "7"; gene_type "
Dt_chr7	Cufflinks	exon	34438903	34438978	.	-	.	gene_id "XLOC_213233"; transcript_id "Novel_002998"; exon_number "8"; gene_type "
Dt_chr7	Cufflinks	exon	34439063	34439314	.	-	.	gene_id "XLOC_213233"; transcript_id "Novel_002998"; exon_number "9"; gene_type "
Dt_chr7	Cufflinks	exon	36751971	36752172	.	-	.	gene_id "XLOC_213417"; transcript_id "Novel_002999"; exon_number "1"; gene_type "
Dt_chr7	Cufflinks	exon	36840906	36842328	.	-	.	gene_id "XLOC_213436"; transcript_id "Novel_003000"; exon_number "1"; gene_type "
Dt_chr7	Cufflinks	exon	36842694	36842762	.	-	.	gene_id "XLOC_213436"; transcript_id "Novel_003000"; exon_number "2"; gene_type "
Dt_chr7	Cufflinks	exon	36842984	36843193	.	-	.	gene_id "XLOC_213436"; transcript_id "Novel_003000"; exon_number "3"; gene_type "
Dt_chr7	Cufflinks	exon	36840912	36842328	.	-	.	gene_id "XLOC_213436"; transcript_id "Novel_003001"; exon_number "1"; gene_type "
Dt_chr7	Cufflinks	exon	36842694	36842845	.	-	.	gene_id "XLOC_213436"; transcript_id "Novel_003001"; exon_number "2"; gene_type "
Dt_chr7	Cufflinks	exon	36951290	36951914	.	-	.	gene_id "XLOC_213451"; transcript_id "Novel_003002"; exon_number "1"; gene_type "
Dt_chr7	Cufflinks	exon	36952125	36952221	.	-	.	gene_id "XLOC_213451"; transcript_id "Novel_003002"; exon_number "2"; gene_type "
Dt_chr7	Cufflinks	exon	36952640	36952784	.	-	.	gene_id "XLOC_213451"; transcript_id "Novel_003002"; exon_number "3"; gene_type "
Dt_chr7	Cufflinks	exon	38525351	38526217	.	-	.	gene_id "XLOC_213569"; transcript_id "Novel_003003"; exon_number "1"; gene_type "
Dt_chr7	Cufflinks	exon	38526448	38529472	.	-	.	gene_id "XLOC_213569"; transcript_id "Novel_003003"; exon_number "2"; gene_type "
Dt_chr7	Cufflinks	exon	40811127	40811341	.	-	.	gene_id "XLOC_213698"; transcript_id "Novel_003004"; exon_number "1"; gene_type "
Dt_chr7	Cufflinks	exon	41086168	41086408	.	-	.	gene_id "XLOC_213721"; transcript_id "Novel_003005"; exon_number "1"; gene_type "
Dt_chr7	Cufflinks	exon	43429353	43430455	.	-	.	gene_id "XLOC_213865"; transcript_id "Novel_003006"; exon_number "1"; gene_type "
Dt_chr7	Cufflinks	exon	43430550	43430598	.	-	.	gene_id "XLOC_213865"; transcript_id "Novel_003006"; exon_number "2"; gene_type "
Dt_chr7	Cufflinks	exon	43430672	43431050	.	-	.	gene_id "XLOC_213865"; transcript_id "Novel_003006"; exon_number "3"; gene_type "
Dt_chr7	Cufflinks	exon	43430405	43430455	.	-	.	gene_id "XLOC_213865"; transcript_id "Novel_003007"; exon_number "1"; gene_type "
Dt_chr7	Cufflinks	exon	43430550	43431048	.	-	.	gene_id "XLOC_213865"; transcript_id "Novel_003007"; exon_number "2"; gene_type "
Dt_chr7	Cufflinks	exon	44897219	44897716	.	-	.	gene_id "XLOC_213937"; transcript_id "Novel_003008"; exon_number "1"; gene_type "
Dt_chr7	Cufflinks	exon	44897862	44897915	.	-	.	gene_id "XLOC_213937"; transcript_id "Novel_003008"; exon_number "2"; gene_type "
Dt_chr7	Cufflinks	exon	44898390	44898480	.	-	.	gene_id "XLOC_213937"; transcript_id "Novel_003008"; exon_number "3"; gene_type "
Dt_chr7	Cufflinks	exon	44898747	44899025	.	-	.	gene_id "XLOC_213937"; transcript_id "Novel_003008"; exon_number "4"; gene_type "

Dt_chr7	Cufflinks	exon	44897231	44897915	.	-	.	gene_id "XLOC_213937"; transcript_id "Novel_003009"; exon_number "1"; gene_type "
Dt_chr7	Cufflinks	exon	44898390	44898480	.	-	.	gene_id "XLOC_213937"; transcript_id "Novel_003009"; exon_number "2"; gene_type "
Dt_chr7	Cufflinks	exon	44898747	44899044	.	-	.	gene_id "XLOC_213937"; transcript_id "Novel_003009"; exon_number "3"; gene_type "
Dt_chr8	Cufflinks	exon	1545392	1547745	.	+	.	gene_id "XLOC_214284"; transcript_id "Novel_003010"; exon_number "1"; gene_type "
Dt_chr8	Cufflinks	exon	1547835	1547923	.	+	.	gene_id "XLOC_214284"; transcript_id "Novel_003010"; exon_number "2"; gene_type "
Dt_chr8	Cufflinks	exon	1548253	1548514	.	+	.	gene_id "XLOC_214284"; transcript_id "Novel_003010"; exon_number "3"; gene_type "
Dt_chr8	Cufflinks	exon	1548697	1548953	.	+	.	gene_id "XLOC_214284"; transcript_id "Novel_003010"; exon_number "4"; gene_type "
Dt_chr8	Cufflinks	exon	1549287	1550080	.	+	.	gene_id "XLOC_214284"; transcript_id "Novel_003010"; exon_number "5"; gene_type "
Dt_chr8	Cufflinks	exon	1547567	1547745	.	+	.	gene_id "XLOC_214284"; transcript_id "Novel_003011"; exon_number "1"; gene_type "
Dt_chr8	Cufflinks	exon	1547835	1548514	.	+	.	gene_id "XLOC_214284"; transcript_id "Novel_003011"; exon_number "2"; gene_type "
Dt_chr8	Cufflinks	exon	1548702	1548953	.	+	.	gene_id "XLOC_214284"; transcript_id "Novel_003011"; exon_number "3"; gene_type "
Dt_chr8	Cufflinks	exon	1549287	1550158	.	+	.	gene_id "XLOC_214284"; transcript_id "Novel_003011"; exon_number "4"; gene_type "
Dt_chr8	Cufflinks	exon	2621909	2623396	.	+	.	gene_id "XLOC_214412"; transcript_id "Novel_003012"; exon_number "1"; gene_type "
Dt_chr8	Cufflinks	exon	13567141	13567410	.	+	.	gene_id "XLOC_215153"; transcript_id "Novel_003013"; exon_number "1"; gene_type "
Dt_chr8	Cufflinks	exon	13568214	13570425	.	+	.	gene_id "XLOC_215153"; transcript_id "Novel_003013"; exon_number "2"; gene_type "
Dt_chr8	Cufflinks	exon	17494886	17495021	.	+	.	gene_id "XLOC_215346"; transcript_id "Novel_003014"; exon_number "1"; gene_type "
Dt_chr8	Cufflinks	exon	17495121	17498617	.	+	.	gene_id "XLOC_215346"; transcript_id "Novel_003014"; exon_number "2"; gene_type "
Dt_chr8	Cufflinks	exon	19202278	19202464	.	+	.	gene_id "XLOC_215429"; transcript_id "Novel_003015"; exon_number "1"; gene_type "
Dt_chr8	Cufflinks	exon	19202563	19202627	.	+	.	gene_id "XLOC_215429"; transcript_id "Novel_003015"; exon_number "2"; gene_type "
Dt_chr8	Cufflinks	exon	20706641	20706799	.	+	.	gene_id "XLOC_215513"; transcript_id "Novel_003016"; exon_number "1"; gene_type "
Dt_chr8	Cufflinks	exon	20706896	20706952	.	+	.	gene_id "XLOC_215513"; transcript_id "Novel_003016"; exon_number "2"; gene_type "
Dt_chr8	Cufflinks	exon	20707994	20710484	.	+	.	gene_id "XLOC_215513"; transcript_id "Novel_003016"; exon_number "3"; gene_type "
Dt_chr8	Cufflinks	exon	21833082	21834099	.	+	.	gene_id "XLOC_215607"; transcript_id "Novel_003017"; exon_number "1"; gene_type "
Dt_chr8	Cufflinks	exon	21834399	21837328	.	+	.	gene_id "XLOC_215607"; transcript_id "Novel_003017"; exon_number "2"; gene_type "
Dt_chr8	Cufflinks	exon	22736084	22736250	.	+	.	gene_id "XLOC_215657"; transcript_id "Novel_003018"; exon_number "1"; gene_type "
Dt_chr8	Cufflinks	exon	22736334	22736800	.	+	.	gene_id "XLOC_215657"; transcript_id "Novel_003018"; exon_number "2"; gene_type "
Dt_chr8	Cufflinks	exon	24140735	24141247	.	+	.	gene_id "XLOC_215809"; transcript_id "Novel_003019"; exon_number "1"; gene_type "
Dt_chr8	Cufflinks	exon	24141790	24142012	.	+	.	gene_id "XLOC_215809"; transcript_id "Novel_003019"; exon_number "2"; gene_type "
Dt_chr8	Cufflinks	exon	24142128	24142207	.	+	.	gene_id "XLOC_215809"; transcript_id "Novel_003019"; exon_number "3"; gene_type "
Dt_chr8	Cufflinks	exon	24143280	24143436	.	+	.	gene_id "XLOC_215809"; transcript_id "Novel_003019"; exon_number "4"; gene_type "
Dt_chr8	Cufflinks	exon	24143518	24143864	.	+	.	gene_id "XLOC_215809"; transcript_id "Novel_003019"; exon_number "5"; gene_type "
Dt_chr8	Cufflinks	exon	24140868	24141046	.	+	.	gene_id "XLOC_215809"; transcript_id "Novel_003020"; exon_number "1"; gene_type "
Dt_chr8	Cufflinks	exon	24141790	24142012	.	+	.	gene_id "XLOC_215809"; transcript_id "Novel_003020"; exon_number "2"; gene_type "
Dt_chr8	Cufflinks	exon	24142128	24142207	.	+	.	gene_id "XLOC_215809"; transcript_id "Novel_003020"; exon_number "3"; gene_type "
Dt_chr8	Cufflinks	exon	24143280	24143436	.	+	.	gene_id "XLOC_215809"; transcript_id "Novel_003020"; exon_number "4"; gene_type "

Dt_chr8	Cufflinks	exon	24143518	24143803	.	+	.	gene_id	"XLOC_215809";	transcript_id	"Novel_003020";	exon_number	"5";	gene_type	"
Dt_chr8	Cufflinks	exon	24142503	24143436	.	+	.	gene_id	"XLOC_215809";	transcript_id	"Novel_003021";	exon_number	"1";	gene_type	"
Dt_chr8	Cufflinks	exon	24143518	24143529	.	+	.	gene_id	"XLOC_215809";	transcript_id	"Novel_003021";	exon_number	"2";	gene_type	"
Dt_chr8	Cufflinks	exon	30392677	30393097	.	+	.	gene_id	"XLOC_216152";	transcript_id	"Novel_003022";	exon_number	"1";	gene_type	"
Dt_chr8	Cufflinks	exon	30393638	30393717	.	+	.	gene_id	"XLOC_216152";	transcript_id	"Novel_003022";	exon_number	"2";	gene_type	"
Dt_chr8	Cufflinks	exon	30394057	30394104	.	+	.	gene_id	"XLOC_216152";	transcript_id	"Novel_003022";	exon_number	"3";	gene_type	"
Dt_chr8	Cufflinks	exon	30394176	30394406	.	+	.	gene_id	"XLOC_216152";	transcript_id	"Novel_003022";	exon_number	"4";	gene_type	"
Dt_chr8	Cufflinks	exon	30393499	30393717	.	+	.	gene_id	"XLOC_216152";	transcript_id	"Novel_003023";	exon_number	"1";	gene_type	"
Dt_chr8	Cufflinks	exon	30394057	30394104	.	+	.	gene_id	"XLOC_216152";	transcript_id	"Novel_003023";	exon_number	"2";	gene_type	"
Dt_chr8	Cufflinks	exon	30394176	30394396	.	+	.	gene_id	"XLOC_216152";	transcript_id	"Novel_003023";	exon_number	"3";	gene_type	"
Dt_chr8	Cufflinks	exon	30401173	30401495	.	+	.	gene_id	"XLOC_216154";	transcript_id	"Novel_003024";	exon_number	"1";	gene_type	"
Dt_chr8	Cufflinks	exon	30401583	30401664	.	+	.	gene_id	"XLOC_216154";	transcript_id	"Novel_003024";	exon_number	"2";	gene_type	"
Dt_chr8	Cufflinks	exon	30407601	30407708	.	+	.	gene_id	"XLOC_216154";	transcript_id	"Novel_003024";	exon_number	"3";	gene_type	"
Dt_chr8	Cufflinks	exon	30407794	30407861	.	+	.	gene_id	"XLOC_216154";	transcript_id	"Novel_003024";	exon_number	"4";	gene_type	"
Dt_chr8	Cufflinks	exon	30408427	30410050	.	+	.	gene_id	"XLOC_216154";	transcript_id	"Novel_003024";	exon_number	"5";	gene_type	"
Dt_chr8	Cufflinks	exon	30401173	30401495	.	+	.	gene_id	"XLOC_216154";	transcript_id	"Novel_003025";	exon_number	"1";	gene_type	"
Dt_chr8	Cufflinks	exon	30401583	30401664	.	+	.	gene_id	"XLOC_216154";	transcript_id	"Novel_003025";	exon_number	"2";	gene_type	"
Dt_chr8	Cufflinks	exon	30407601	30407708	.	+	.	gene_id	"XLOC_216154";	transcript_id	"Novel_003025";	exon_number	"3";	gene_type	"
Dt_chr8	Cufflinks	exon	30407794	30407899	.	+	.	gene_id	"XLOC_216154";	transcript_id	"Novel_003025";	exon_number	"4";	gene_type	"
Dt_chr8	Cufflinks	exon	30408427	30410050	.	+	.	gene_id	"XLOC_216154";	transcript_id	"Novel_003025";	exon_number	"5";	gene_type	"
Dt_chr8	Cufflinks	exon	34028250	34028470	.	+	.	gene_id	"XLOC_216443";	transcript_id	"Novel_003026";	exon_number	"1";	gene_type	"
Dt_chr8	Cufflinks	exon	34412803	34412992	.	+	.	gene_id	"XLOC_216481";	transcript_id	"Novel_003027";	exon_number	"1";	gene_type	"
Dt_chr8	Cufflinks	exon	34414051	34414128	.	+	.	gene_id	"XLOC_216481";	transcript_id	"Novel_003027";	exon_number	"2";	gene_type	"
Dt_chr8	Cufflinks	exon	34414595	34414812	.	+	.	gene_id	"XLOC_216481";	transcript_id	"Novel_003027";	exon_number	"3";	gene_type	"
Dt_chr8	Cufflinks	exon	35169200	35169371	.	+	.	gene_id	"XLOC_216549";	transcript_id	"Novel_003028";	exon_number	"1";	gene_type	"
Dt_chr8	Cufflinks	exon	35169601	35169684	.	+	.	gene_id	"XLOC_216549";	transcript_id	"Novel_003028";	exon_number	"2";	gene_type	"
Dt_chr8	Cufflinks	exon	35737053	35737508	.	+	.	gene_id	"XLOC_216596";	transcript_id	"Novel_003029";	exon_number	"1";	gene_type	"
Dt_chr8	Cufflinks	exon	35739384	35739497	.	+	.	gene_id	"XLOC_216596";	transcript_id	"Novel_003029";	exon_number	"2";	gene_type	"
Dt_chr8	Cufflinks	exon	35740134	35740529	.	+	.	gene_id	"XLOC_216596";	transcript_id	"Novel_003029";	exon_number	"3";	gene_type	"
Dt_chr8	Cufflinks	exon	35741249	35741317	.	+	.	gene_id	"XLOC_216596";	transcript_id	"Novel_003029";	exon_number	"4";	gene_type	"
Dt_chr8	Cufflinks	exon	35741422	35741493	.	+	.	gene_id	"XLOC_216596";	transcript_id	"Novel_003029";	exon_number	"5";	gene_type	"
Dt_chr8	Cufflinks	exon	35741594	35741722	.	+	.	gene_id	"XLOC_216596";	transcript_id	"Novel_003029";	exon_number	"6";	gene_type	"
Dt_chr8	Cufflinks	exon	35741823	35741903	.	+	.	gene_id	"XLOC_216596";	transcript_id	"Novel_003029";	exon_number	"7";	gene_type	"
Dt_chr8	Cufflinks	exon	35742131	35742326	.	+	.	gene_id	"XLOC_216596";	transcript_id	"Novel_003029";	exon_number	"8";	gene_type	"
Dt_chr8	Cufflinks	exon	35742837	35743363	.	+	.	gene_id	"XLOC_216596";	transcript_id	"Novel_003029";	exon_number	"9";	gene_type	"

Dt_chr8	Cufflinks	exon	36054219	36054490	.	+	.	gene_id "XLOC_216624"; transcript_id "Novel_003030"; exon_number "1"; gene_type "
Dt_chr8	Cufflinks	exon	36055415	36055455	.	+	.	gene_id "XLOC_216624"; transcript_id "Novel_003030"; exon_number "2"; gene_type "
Dt_chr8	Cufflinks	exon	36055562	36055649	.	+	.	gene_id "XLOC_216624"; transcript_id "Novel_003030"; exon_number "3"; gene_type "
Dt_chr8	Cufflinks	exon	36055800	36055886	.	+	.	gene_id "XLOC_216624"; transcript_id "Novel_003030"; exon_number "4"; gene_type "
Dt_chr8	Cufflinks	exon	36056122	36056168	.	+	.	gene_id "XLOC_216624"; transcript_id "Novel_003030"; exon_number "5"; gene_type "
Dt_chr8	Cufflinks	exon	37291873	37292952	.	+	.	gene_id "XLOC_216728"; transcript_id "Novel_003031"; exon_number "1"; gene_type "
Dt_chr8	Cufflinks	exon	37293557	37294105	.	+	.	gene_id "XLOC_216728"; transcript_id "Novel_003031"; exon_number "2"; gene_type "
Dt_chr8	Cufflinks	exon	38613551	38613785	.	+	.	gene_id "XLOC_216860"; transcript_id "Novel_003032"; exon_number "1"; gene_type "
Dt_chr8	Cufflinks	exon	38616273	38616550	.	+	.	gene_id "XLOC_216860"; transcript_id "Novel_003032"; exon_number "2"; gene_type "
Dt_chr8	Cufflinks	exon	38632184	38632684	.	+	.	gene_id "XLOC_216866"; transcript_id "Novel_003033"; exon_number "1"; gene_type "
Dt_chr8	Cufflinks	exon	38632889	38633773	.	+	.	gene_id "XLOC_216866"; transcript_id "Novel_003033"; exon_number "2"; gene_type "
Dt_chr8	Cufflinks	exon	38633867	38634157	.	+	.	gene_id "XLOC_216866"; transcript_id "Novel_003033"; exon_number "3"; gene_type "
Dt_chr8	Cufflinks	exon	38632189	38632684	.	+	.	gene_id "XLOC_216866"; transcript_id "Novel_003034"; exon_number "1"; gene_type "
Dt_chr8	Cufflinks	exon	38632889	38633388	.	+	.	gene_id "XLOC_216866"; transcript_id "Novel_003034"; exon_number "2"; gene_type "
Dt_chr8	Cufflinks	exon	38633489	38633626	.	+	.	gene_id "XLOC_216866"; transcript_id "Novel_003034"; exon_number "3"; gene_type "
Dt_chr8	Cufflinks	exon	38633715	38633773	.	+	.	gene_id "XLOC_216866"; transcript_id "Novel_003034"; exon_number "4"; gene_type "
Dt_chr8	Cufflinks	exon	38633867	38634225	.	+	.	gene_id "XLOC_216866"; transcript_id "Novel_003034"; exon_number "5"; gene_type "
Dt_chr8	Cufflinks	exon	39152731	39153005	.	+	.	gene_id "XLOC_216920"; transcript_id "Novel_003035"; exon_number "1"; gene_type "
Dt_chr8	Cufflinks	exon	39153080	39153180	.	+	.	gene_id "XLOC_216920"; transcript_id "Novel_003035"; exon_number "2"; gene_type "
Dt_chr8	Cufflinks	exon	40770584	40770809	.	+	.	gene_id "XLOC_217126"; transcript_id "Novel_003036"; exon_number "1"; gene_type "
Dt_chr8	Cufflinks	exon	43619659	43619745	.	+	.	gene_id "XLOC_217447"; transcript_id "Novel_003037"; exon_number "1"; gene_type "
Dt_chr8	Cufflinks	exon	43620084	43628973	.	+	.	gene_id "XLOC_217447"; transcript_id "Novel_003037"; exon_number "2"; gene_type "
Dt_chr8	Cufflinks	exon	43619661	43619745	.	+	.	gene_id "XLOC_217447"; transcript_id "Novel_003038"; exon_number "1"; gene_type "
Dt_chr8	Cufflinks	exon	43620084	43628365	.	+	.	gene_id "XLOC_217447"; transcript_id "Novel_003038"; exon_number "2"; gene_type "
Dt_chr8	Cufflinks	exon	43628596	43629104	.	+	.	gene_id "XLOC_217447"; transcript_id "Novel_003038"; exon_number "3"; gene_type "
Dt_chr8	Cufflinks	exon	43886633	43886657	.	+	.	gene_id "XLOC_217480"; transcript_id "Novel_003039"; exon_number "1"; gene_type "
Dt_chr8	Cufflinks	exon	43886782	43886829	.	+	.	gene_id "XLOC_217480"; transcript_id "Novel_003039"; exon_number "2"; gene_type "
Dt_chr8	Cufflinks	exon	43887099	43887154	.	+	.	gene_id "XLOC_217480"; transcript_id "Novel_003039"; exon_number "3"; gene_type "
Dt_chr8	Cufflinks	exon	43887298	43889067	.	+	.	gene_id "XLOC_217480"; transcript_id "Novel_003039"; exon_number "4"; gene_type "
Dt_chr8	Cufflinks	exon	44589844	44590676	.	+	.	gene_id "XLOC_217583"; transcript_id "Novel_003040"; exon_number "1"; gene_type "
Dt_chr8	Cufflinks	exon	44590767	44591192	.	+	.	gene_id "XLOC_217583"; transcript_id "Novel_003040"; exon_number "2"; gene_type "
Dt_chr8	Cufflinks	exon	44589944	44590676	.	+	.	gene_id "XLOC_217583"; transcript_id "Novel_003041"; exon_number "1"; gene_type "
Dt_chr8	Cufflinks	exon	44590767	44591112	.	+	.	gene_id "XLOC_217583"; transcript_id "Novel_003041"; exon_number "2"; gene_type "
Dt_chr8	Cufflinks	exon	44591191	44591239	.	+	.	gene_id "XLOC_217583"; transcript_id "Novel_003041"; exon_number "3"; gene_type "
Dt_chr8	Cufflinks	exon	44591328	44591399	.	+	.	gene_id "XLOC_217583"; transcript_id "Novel_003041"; exon_number "4"; gene_type "

Dt_chr8	Cufflinks	exon	44591498	44591627	.	+	.	gene_id "XLOC_217583"; transcript_id "Novel_003041"; exon_number "5"; gene_type "
Dt_chr8	Cufflinks	exon	44591728	44592188	.	+	.	gene_id "XLOC_217583"; transcript_id "Novel_003041"; exon_number "6"; gene_type "
Dt_chr8	Cufflinks	exon	44592280	44592384	.	+	.	gene_id "XLOC_217583"; transcript_id "Novel_003041"; exon_number "7"; gene_type "
Dt_chr8	Cufflinks	exon	44592493	44592537	.	+	.	gene_id "XLOC_217583"; transcript_id "Novel_003041"; exon_number "8"; gene_type "
Dt_chr8	Cufflinks	exon	44592624	44592773	.	+	.	gene_id "XLOC_217583"; transcript_id "Novel_003041"; exon_number "9"; gene_type "
Dt_chr8	Cufflinks	exon	44592875	44593153	.	+	.	gene_id "XLOC_217583"; transcript_id "Novel_003041"; exon_number "10"; gene_type
Dt_chr8	Cufflinks	exon	44593233	44593369	.	+	.	gene_id "XLOC_217583"; transcript_id "Novel_003041"; exon_number "11"; gene_type
Dt_chr8	Cufflinks	exon	44593460	44593668	.	+	.	gene_id "XLOC_217583"; transcript_id "Novel_003041"; exon_number "12"; gene_type
Dt_chr8	Cufflinks	exon	44589968	44590676	.	+	.	gene_id "XLOC_217583"; transcript_id "Novel_003042"; exon_number "1"; gene_type "
Dt_chr8	Cufflinks	exon	44590767	44591112	.	+	.	gene_id "XLOC_217583"; transcript_id "Novel_003042"; exon_number "2"; gene_type "
Dt_chr8	Cufflinks	exon	44591191	44591239	.	+	.	gene_id "XLOC_217583"; transcript_id "Novel_003042"; exon_number "3"; gene_type "
Dt_chr8	Cufflinks	exon	44591328	44591399	.	+	.	gene_id "XLOC_217583"; transcript_id "Novel_003042"; exon_number "4"; gene_type "
Dt_chr8	Cufflinks	exon	44591498	44591627	.	+	.	gene_id "XLOC_217583"; transcript_id "Novel_003042"; exon_number "5"; gene_type "
Dt_chr8	Cufflinks	exon	44591728	44591848	.	+	.	gene_id "XLOC_217583"; transcript_id "Novel_003042"; exon_number "6"; gene_type "
Dt_chr8	Cufflinks	exon	44591930	44592188	.	+	.	gene_id "XLOC_217583"; transcript_id "Novel_003042"; exon_number "7"; gene_type "
Dt_chr8	Cufflinks	exon	44592280	44592384	.	+	.	gene_id "XLOC_217583"; transcript_id "Novel_003042"; exon_number "8"; gene_type "
Dt_chr8	Cufflinks	exon	44592493	44592537	.	+	.	gene_id "XLOC_217583"; transcript_id "Novel_003042"; exon_number "9"; gene_type "
Dt_chr8	Cufflinks	exon	44592624	44592773	.	+	.	gene_id "XLOC_217583"; transcript_id "Novel_003042"; exon_number "10"; gene_type
Dt_chr8	Cufflinks	exon	44592875	44593153	.	+	.	gene_id "XLOC_217583"; transcript_id "Novel_003042"; exon_number "11"; gene_type
Dt_chr8	Cufflinks	exon	44593233	44593369	.	+	.	gene_id "XLOC_217583"; transcript_id "Novel_003042"; exon_number "12"; gene_type
Dt_chr8	Cufflinks	exon	44593460	44593670	.	+	.	gene_id "XLOC_217583"; transcript_id "Novel_003042"; exon_number "13"; gene_type
Dt_chr8	Cufflinks	exon	45463160	45463545	.	+	.	gene_id "XLOC_217691"; transcript_id "Novel_003043"; exon_number "1"; gene_type "
Dt_chr8	Cufflinks	exon	45833768	45834246	.	+	.	gene_id "XLOC_217741"; transcript_id "Novel_003044"; exon_number "1"; gene_type "
Dt_chr8	Cufflinks	exon	45834331	45834625	.	+	.	gene_id "XLOC_217741"; transcript_id "Novel_003044"; exon_number "2"; gene_type "
Dt_chr8	Cufflinks	exon	45834937	45835357	.	+	.	gene_id "XLOC_217741"; transcript_id "Novel_003044"; exon_number "3"; gene_type "
Dt_chr8	Cufflinks	exon	45835485	45835533	.	+	.	gene_id "XLOC_217741"; transcript_id "Novel_003044"; exon_number "4"; gene_type "
Dt_chr8	Cufflinks	exon	45835632	45835703	.	+	.	gene_id "XLOC_217741"; transcript_id "Novel_003044"; exon_number "5"; gene_type "
Dt_chr8	Cufflinks	exon	45835995	45836124	.	+	.	gene_id "XLOC_217741"; transcript_id "Novel_003044"; exon_number "6"; gene_type "
Dt_chr8	Cufflinks	exon	45836707	45836827	.	+	.	gene_id "XLOC_217741"; transcript_id "Novel_003044"; exon_number "7"; gene_type "
Dt_chr8	Cufflinks	exon	45836908	45837157	.	+	.	gene_id "XLOC_217741"; transcript_id "Novel_003044"; exon_number "8"; gene_type "
Dt_chr8	Cufflinks	exon	45837248	45837352	.	+	.	gene_id "XLOC_217741"; transcript_id "Novel_003044"; exon_number "9"; gene_type "
Dt_chr8	Cufflinks	exon	45837455	45837499	.	+	.	gene_id "XLOC_217741"; transcript_id "Novel_003044"; exon_number "10"; gene_type
Dt_chr8	Cufflinks	exon	45837590	45837742	.	+	.	gene_id "XLOC_217741"; transcript_id "Novel_003044"; exon_number "11"; gene_type
Dt_chr8	Cufflinks	exon	45837853	45838131	.	+	.	gene_id "XLOC_217741"; transcript_id "Novel_003044"; exon_number "12"; gene_type
Dt_chr8	Cufflinks	exon	45838488	45838624	.	+	.	gene_id "XLOC_217741"; transcript_id "Novel_003044"; exon_number "13"; gene_type

Dt_chr8	Cufflinks	exon	45838756	45839213	.	+	.	gene_id "XLOC_217741"; transcript_id "Novel_003044"; exon_number "14"; gene_type
Dt_chr8	Cufflinks	exon	46740357	46740486	.	+	.	gene_id "XLOC_217841"; transcript_id "Novel_003045"; exon_number "1"; gene_type "
Dt_chr8	Cufflinks	exon	46741748	46741866	.	+	.	gene_id "XLOC_217841"; transcript_id "Novel_003045"; exon_number "2"; gene_type "
Dt_chr8	Cufflinks	exon	46742450	46742982	.	+	.	gene_id "XLOC_217841"; transcript_id "Novel_003045"; exon_number "3"; gene_type "
Dt_chr8	Cufflinks	exon	46743088	46743544	.	+	.	gene_id "XLOC_217841"; transcript_id "Novel_003045"; exon_number "4"; gene_type "
Dt_chr8	Cufflinks	exon	46740428	46740486	.	+	.	gene_id "XLOC_217841"; transcript_id "Novel_003046"; exon_number "1"; gene_type "
Dt_chr8	Cufflinks	exon	46741748	46741866	.	+	.	gene_id "XLOC_217841"; transcript_id "Novel_003046"; exon_number "2"; gene_type "
Dt_chr8	Cufflinks	exon	46742450	46743121	.	+	.	gene_id "XLOC_217841"; transcript_id "Novel_003046"; exon_number "3"; gene_type "
Dt_chr8	Cufflinks	exon	48155578	48157381	.	+	.	gene_id "XLOC_218027"; transcript_id "Novel_003047"; exon_number "1"; gene_type "
Dt_chr8	Cufflinks	exon	48157588	48159162	.	+	.	gene_id "XLOC_218027"; transcript_id "Novel_003047"; exon_number "2"; gene_type "
Dt_chr8	Cufflinks	exon	49032336	49032781	.	+	.	gene_id "XLOC_218168"; transcript_id "Novel_003048"; exon_number "1"; gene_type "
Dt_chr8	Cufflinks	exon	49032894	49033031	.	+	.	gene_id "XLOC_218168"; transcript_id "Novel_003048"; exon_number "2"; gene_type "
Dt_chr8	Cufflinks	exon	49033124	49033565	.	+	.	gene_id "XLOC_218168"; transcript_id "Novel_003048"; exon_number "3"; gene_type "
Dt_chr8	Cufflinks	exon	49419843	49419946	.	+	.	gene_id "XLOC_218243"; transcript_id "Novel_003049"; exon_number "1"; gene_type "
Dt_chr8	Cufflinks	exon	49420188	49420381	.	+	.	gene_id "XLOC_218243"; transcript_id "Novel_003049"; exon_number "2"; gene_type "
Dt_chr8	Cufflinks	exon	49420489	49421236	.	+	.	gene_id "XLOC_218243"; transcript_id "Novel_003049"; exon_number "3"; gene_type "
Dt_chr8	Cufflinks	exon	49419848	49419946	.	+	.	gene_id "XLOC_218243"; transcript_id "Novel_003050"; exon_number "1"; gene_type "
Dt_chr8	Cufflinks	exon	49420188	49420381	.	+	.	gene_id "XLOC_218243"; transcript_id "Novel_003050"; exon_number "2"; gene_type "
Dt_chr8	Cufflinks	exon	49420489	49420656	.	+	.	gene_id "XLOC_218243"; transcript_id "Novel_003050"; exon_number "3"; gene_type "
Dt_chr8	Cufflinks	exon	49421250	49421343	.	+	.	gene_id "XLOC_218243"; transcript_id "Novel_003050"; exon_number "4"; gene_type "
Dt_chr8	Cufflinks	exon	49421889	49422646	.	+	.	gene_id "XLOC_218243"; transcript_id "Novel_003050"; exon_number "5"; gene_type "
Dt_chr8	Cufflinks	exon	49419852	49419946	.	+	.	gene_id "XLOC_218243"; transcript_id "Novel_003051"; exon_number "1"; gene_type "
Dt_chr8	Cufflinks	exon	49420188	49420381	.	+	.	gene_id "XLOC_218243"; transcript_id "Novel_003051"; exon_number "2"; gene_type "
Dt_chr8	Cufflinks	exon	49420489	49420656	.	+	.	gene_id "XLOC_218243"; transcript_id "Novel_003051"; exon_number "3"; gene_type "
Dt_chr8	Cufflinks	exon	49421250	49421343	.	+	.	gene_id "XLOC_218243"; transcript_id "Novel_003051"; exon_number "4"; gene_type "
Dt_chr8	Cufflinks	exon	49421889	49422083	.	+	.	gene_id "XLOC_218243"; transcript_id "Novel_003051"; exon_number "5"; gene_type "
Dt_chr8	Cufflinks	exon	49422886	49423582	.	+	.	gene_id "XLOC_218243"; transcript_id "Novel_003051"; exon_number "6"; gene_type "
Dt_chr8	Cufflinks	exon	49437264	49437333	.	+	.	gene_id "XLOC_218252"; transcript_id "Novel_003052"; exon_number "1"; gene_type "
Dt_chr8	Cufflinks	exon	49437456	49437648	.	+	.	gene_id "XLOC_218252"; transcript_id "Novel_003052"; exon_number "2"; gene_type "
Dt_chr8	Cufflinks	exon	49945530	49947866	.	+	.	gene_id "XLOC_218352"; transcript_id "Novel_003053"; exon_number "1"; gene_type "
Dt_chr8	Cufflinks	exon	50239815	50240346	.	+	.	gene_id "XLOC_218399"; transcript_id "Novel_003054"; exon_number "1"; gene_type "
Dt_chr8	Cufflinks	exon	50448946	50449085	.	+	.	gene_id "XLOC_218439"; transcript_id "Novel_003055"; exon_number "1"; gene_type "
Dt_chr8	Cufflinks	exon	50449169	50449264	.	+	.	gene_id "XLOC_218439"; transcript_id "Novel_003055"; exon_number "2"; gene_type "
Dt_chr8	Cufflinks	exon	50449367	50449453	.	+	.	gene_id "XLOC_218439"; transcript_id "Novel_003055"; exon_number "3"; gene_type "
Dt_chr8	Cufflinks	exon	50450215	50450807	.	+	.	gene_id "XLOC_218439"; transcript_id "Novel_003055"; exon_number "4"; gene_type "

Dt_chr8	Cufflinks	exon	50451861	50451926	.	+	.	gene_id "XLOC_218439"; transcript_id "Novel_003055"; exon_number "5"; gene_type "
Dt_chr8	Cufflinks	exon	50452050	50452445	.	+	.	gene_id "XLOC_218439"; transcript_id "Novel_003055"; exon_number "6"; gene_type "
Dt_chr8	Cufflinks	exon	50448971	50449085	.	+	.	gene_id "XLOC_218439"; transcript_id "Novel_003056"; exon_number "1"; gene_type "
Dt_chr8	Cufflinks	exon	50449169	50449264	.	+	.	gene_id "XLOC_218439"; transcript_id "Novel_003056"; exon_number "2"; gene_type "
Dt_chr8	Cufflinks	exon	50449367	50449453	.	+	.	gene_id "XLOC_218439"; transcript_id "Novel_003056"; exon_number "3"; gene_type "
Dt_chr8	Cufflinks	exon	50450215	50451926	.	+	.	gene_id "XLOC_218439"; transcript_id "Novel_003056"; exon_number "4"; gene_type "
Dt_chr8	Cufflinks	exon	50452050	50452353	.	+	.	gene_id "XLOC_218439"; transcript_id "Novel_003056"; exon_number "5"; gene_type "
Dt_chr8	Cufflinks	exon	50596854	50597014	.	+	.	gene_id "XLOC_218474"; transcript_id "Novel_003057"; exon_number "1"; gene_type "
Dt_chr8	Cufflinks	exon	50598160	50598576	.	+	.	gene_id "XLOC_218474"; transcript_id "Novel_003057"; exon_number "2"; gene_type "
Dt_chr8	Cufflinks	exon	50604067	50604535	.	+	.	gene_id "XLOC_218477"; transcript_id "Novel_003058"; exon_number "1"; gene_type "
Dt_chr8	Cufflinks	exon	50605904	50606683	.	+	.	gene_id "XLOC_218477"; transcript_id "Novel_003058"; exon_number "2"; gene_type "
Dt_chr8	Cufflinks	exon	50606774	50607960	.	+	.	gene_id "XLOC_218477"; transcript_id "Novel_003058"; exon_number "3"; gene_type "
Dt_chr8	Cufflinks	exon	51970362	51971313	.	+	.	gene_id "XLOC_218625"; transcript_id "Novel_003059"; exon_number "1"; gene_type "
Dt_chr8	Cufflinks	exon	51971473	51971557	.	+	.	gene_id "XLOC_218625"; transcript_id "Novel_003059"; exon_number "2"; gene_type "
Dt_chr8	Cufflinks	exon	51972634	51972833	.	+	.	gene_id "XLOC_218625"; transcript_id "Novel_003059"; exon_number "3"; gene_type "
Dt_chr8	Cufflinks	exon	51972923	51972943	.	+	.	gene_id "XLOC_218625"; transcript_id "Novel_003059"; exon_number "4"; gene_type "
Dt_chr8	Cufflinks	exon	51970440	51971313	.	+	.	gene_id "XLOC_218625"; transcript_id "Novel_003060"; exon_number "1"; gene_type "
Dt_chr8	Cufflinks	exon	51971473	51971557	.	+	.	gene_id "XLOC_218625"; transcript_id "Novel_003060"; exon_number "2"; gene_type "
Dt_chr8	Cufflinks	exon	51972634	51972908	.	+	.	gene_id "XLOC_218625"; transcript_id "Novel_003060"; exon_number "3"; gene_type "
Dt_chr8	Cufflinks	exon	53139228	53139638	.	+	.	gene_id "XLOC_218774"; transcript_id "Novel_003061"; exon_number "1"; gene_type "
Dt_chr8	Cufflinks	exon	53139765	53139911	.	+	.	gene_id "XLOC_218774"; transcript_id "Novel_003061"; exon_number "2"; gene_type "
Dt_chr8	Cufflinks	exon	53140222	53140253	.	+	.	gene_id "XLOC_218774"; transcript_id "Novel_003061"; exon_number "3"; gene_type "
Dt_chr8	Cufflinks	exon	53139950	53140343	.	+	.	gene_id "XLOC_218774"; transcript_id "Novel_003062"; exon_number "1"; gene_type "
Dt_chr8	Cufflinks	exon	53140429	53140809	.	+	.	gene_id "XLOC_218774"; transcript_id "Novel_003062"; exon_number "2"; gene_type "
Dt_chr8	Cufflinks	exon	53140914	53140924	.	+	.	gene_id "XLOC_218774"; transcript_id "Novel_003063"; exon_number "1"; gene_type "
Dt_chr8	Cufflinks	exon	53141299	53142450	.	+	.	gene_id "XLOC_218774"; transcript_id "Novel_003063"; exon_number "2"; gene_type "
Dt_chr8	Cufflinks	exon	54764626	54765470	.	+	.	gene_id "XLOC_219004"; transcript_id "Novel_003064"; exon_number "1"; gene_type "
Dt_chr8	Cufflinks	exon	54765577	54766651	.	+	.	gene_id "XLOC_219004"; transcript_id "Novel_003064"; exon_number "2"; gene_type "
Dt_chr8	Cufflinks	exon	56614623	56614827	.	+	.	gene_id "XLOC_219288"; transcript_id "Novel_003065"; exon_number "1"; gene_type "
Dt_chr8	Cufflinks	exon	56615296	56615467	.	+	.	gene_id "XLOC_219288"; transcript_id "Novel_003065"; exon_number "2"; gene_type "
Dt_chr8	Cufflinks	exon	56615695	56615811	.	+	.	gene_id "XLOC_219288"; transcript_id "Novel_003065"; exon_number "3"; gene_type "
Dt_chr8	Cufflinks	exon	56616077	56616498	.	+	.	gene_id "XLOC_219288"; transcript_id "Novel_003065"; exon_number "4"; gene_type "
Dt_chr8	Cufflinks	exon	56614651	56614827	.	+	.	gene_id "XLOC_219288"; transcript_id "Novel_003066"; exon_number "1"; gene_type "
Dt_chr8	Cufflinks	exon	56615366	56615467	.	+	.	gene_id "XLOC_219288"; transcript_id "Novel_003066"; exon_number "2"; gene_type "
Dt_chr8	Cufflinks	exon	56615695	56615811	.	+	.	gene_id "XLOC_219288"; transcript_id "Novel_003066"; exon_number "3"; gene_type "

Dt_chr8	Cufflinks	exon	56616077	56616483	.	+	.	gene_id "XLOC_219288"; transcript_id "Novel_003066"; exon_number "4"; gene_type "
Dt_chr8	Cufflinks	exon	902574	903033	.	-	.	gene_id "XLOC_219463"; transcript_id "Novel_003067"; exon_number "1"; gene_type "
Dt_chr8	Cufflinks	exon	903668	903698	.	-	.	gene_id "XLOC_219463"; transcript_id "Novel_003067"; exon_number "2"; gene_type "
Dt_chr8	Cufflinks	exon	902631	904028	.	-	.	gene_id "XLOC_219463"; transcript_id "Novel_003068"; exon_number "1"; gene_type "
Dt_chr8	Cufflinks	exon	904518	904701	.	-	.	gene_id "XLOC_219463"; transcript_id "Novel_003068"; exon_number "2"; gene_type "
Dt_chr8	Cufflinks	exon	908872	909847	.	-	.	gene_id "XLOC_219464"; transcript_id "Novel_003069"; exon_number "1"; gene_type "
Dt_chr8	Cufflinks	exon	910008	910192	.	-	.	gene_id "XLOC_219464"; transcript_id "Novel_003069"; exon_number "2"; gene_type "
Dt_chr8	Cufflinks	exon	910391	910806	.	-	.	gene_id "XLOC_219464"; transcript_id "Novel_003069"; exon_number "3"; gene_type "
Dt_chr8	Cufflinks	exon	911063	911262	.	-	.	gene_id "XLOC_219464"; transcript_id "Novel_003069"; exon_number "4"; gene_type "
Dt_chr8	Cufflinks	exon	911346	911417	.	-	.	gene_id "XLOC_219464"; transcript_id "Novel_003069"; exon_number "5"; gene_type "
Dt_chr8	Cufflinks	exon	911946	912125	.	-	.	gene_id "XLOC_219464"; transcript_id "Novel_003069"; exon_number "6"; gene_type "
Dt_chr8	Cufflinks	exon	912334	912452	.	-	.	gene_id "XLOC_219464"; transcript_id "Novel_003069"; exon_number "7"; gene_type "
Dt_chr8	Cufflinks	exon	912619	912722	.	-	.	gene_id "XLOC_219464"; transcript_id "Novel_003069"; exon_number "8"; gene_type "
Dt_chr8	Cufflinks	exon	2632666	2632856	.	-	.	gene_id "XLOC_219645"; transcript_id "Novel_003070"; exon_number "1"; gene_type "
Dt_chr8	Cufflinks	exon	2633290	2633362	.	-	.	gene_id "XLOC_219645"; transcript_id "Novel_003070"; exon_number "2"; gene_type "
Dt_chr8	Cufflinks	exon	2633674	2634025	.	-	.	gene_id "XLOC_219645"; transcript_id "Novel_003070"; exon_number "3"; gene_type "
Dt_chr8	Cufflinks	exon	2634137	2634186	.	-	.	gene_id "XLOC_219645"; transcript_id "Novel_003070"; exon_number "4"; gene_type "
Dt_chr8	Cufflinks	exon	2634479	2634913	.	-	.	gene_id "XLOC_219645"; transcript_id "Novel_003070"; exon_number "5"; gene_type "
Dt_chr8	Cufflinks	exon	3431000	3431448	.	-	.	gene_id "XLOC_219711"; transcript_id "Novel_003071"; exon_number "1"; gene_type "
Dt_chr8	Cufflinks	exon	3431553	3431605	.	-	.	gene_id "XLOC_219711"; transcript_id "Novel_003071"; exon_number "2"; gene_type "
Dt_chr8	Cufflinks	exon	3431688	3432007	.	-	.	gene_id "XLOC_219711"; transcript_id "Novel_003071"; exon_number "3"; gene_type "
Dt_chr8	Cufflinks	exon	3432543	3432591	.	-	.	gene_id "XLOC_219711"; transcript_id "Novel_003071"; exon_number "4"; gene_type "
Dt_chr8	Cufflinks	exon	3432707	3432808	.	-	.	gene_id "XLOC_219711"; transcript_id "Novel_003071"; exon_number "5"; gene_type "
Dt_chr8	Cufflinks	exon	6525327	6525729	.	-	.	gene_id "XLOC_220048"; transcript_id "Novel_003072"; exon_number "1"; gene_type "
Dt_chr8	Cufflinks	exon	6525903	6525943	.	-	.	gene_id "XLOC_220048"; transcript_id "Novel_003072"; exon_number "2"; gene_type "
Dt_chr8	Cufflinks	exon	6526113	6526617	.	-	.	gene_id "XLOC_220048"; transcript_id "Novel_003072"; exon_number "3"; gene_type "
Dt_chr8	Cufflinks	exon	6526921	6527394	.	-	.	gene_id "XLOC_220048"; transcript_id "Novel_003072"; exon_number "4"; gene_type "
Dt_chr8	Cufflinks	exon	6528100	6528214	.	-	.	gene_id "XLOC_220048"; transcript_id "Novel_003072"; exon_number "5"; gene_type "
Dt_chr8	Cufflinks	exon	6528485	6528848	.	-	.	gene_id "XLOC_220048"; transcript_id "Novel_003072"; exon_number "6"; gene_type "
Dt_chr8	Cufflinks	exon	9130901	9131470	.	-	.	gene_id "XLOC_220199"; transcript_id "Novel_003073"; exon_number "1"; gene_type "
Dt_chr8	Cufflinks	exon	9131696	9132348	.	-	.	gene_id "XLOC_220199"; transcript_id "Novel_003073"; exon_number "2"; gene_type "
Dt_chr8	Cufflinks	exon	10346823	10347061	.	-	.	gene_id "XLOC_220265"; transcript_id "Novel_003074"; exon_number "1"; gene_type "
Dt_chr8	Cufflinks	exon	13485143	13485193	.	-	.	gene_id "XLOC_220418"; transcript_id "Novel_003075"; exon_number "1"; gene_type "
Dt_chr8	Cufflinks	exon	13486151	13486194	.	-	.	gene_id "XLOC_220418"; transcript_id "Novel_003075"; exon_number "2"; gene_type "
Dt_chr8	Cufflinks	exon	13486282	13486386	.	-	.	gene_id "XLOC_220418"; transcript_id "Novel_003075"; exon_number "3"; gene_type "

Dt_chr8	Cufflinks	exon	13485143	13485193	.	-	.	gene_id "XLOC_220418"; transcript_id "Novel_003076"; exon_number "1"; gene_type "
Dt_chr8	Cufflinks	exon	13486151	13486194	.	-	.	gene_id "XLOC_220418"; transcript_id "Novel_003076"; exon_number "2"; gene_type "
Dt_chr8	Cufflinks	exon	13486282	13486355	.	-	.	gene_id "XLOC_220418"; transcript_id "Novel_003076"; exon_number "3"; gene_type "
Dt_chr8	Cufflinks	exon	13486454	13486485	.	-	.	gene_id "XLOC_220418"; transcript_id "Novel_003076"; exon_number "4"; gene_type "
Dt_chr8	Cufflinks	exon	13905417	13905683	.	-	.	gene_id "XLOC_220445"; transcript_id "Novel_003077"; exon_number "1"; gene_type "
Dt_chr8	Cufflinks	exon	20064096	20065422	.	-	.	gene_id "XLOC_220745"; transcript_id "Novel_003078"; exon_number "1"; gene_type "
Dt_chr8	Cufflinks	exon	20065629	20067382	.	-	.	gene_id "XLOC_220745"; transcript_id "Novel_003078"; exon_number "2"; gene_type "
Dt_chr8	Cufflinks	exon	20251819	20255194	.	-	.	gene_id "XLOC_220754"; transcript_id "Novel_003079"; exon_number "1"; gene_type "
Dt_chr8	Cufflinks	exon	20255430	20256752	.	-	.	gene_id "XLOC_220754"; transcript_id "Novel_003079"; exon_number "2"; gene_type "
Dt_chr8	Cufflinks	exon	20251952	20252068	.	-	.	gene_id "XLOC_220754"; transcript_id "Novel_003080"; exon_number "1"; gene_type "
Dt_chr8	Cufflinks	exon	20255089	20255194	.	-	.	gene_id "XLOC_220754"; transcript_id "Novel_003080"; exon_number "2"; gene_type "
Dt_chr8	Cufflinks	exon	20255430	20256741	.	-	.	gene_id "XLOC_220754"; transcript_id "Novel_003080"; exon_number "3"; gene_type "
Dt_chr8	Cufflinks	exon	23459661	23460258	.	-	.	gene_id "XLOC_220982"; transcript_id "Novel_003081"; exon_number "1"; gene_type "
Dt_chr8	Cufflinks	exon	23460355	23460549	.	-	.	gene_id "XLOC_220982"; transcript_id "Novel_003081"; exon_number "2"; gene_type "
Dt_chr8	Cufflinks	exon	23460827	23461776	.	-	.	gene_id "XLOC_220982"; transcript_id "Novel_003081"; exon_number "3"; gene_type "
Dt_chr8	Cufflinks	exon	23461821	23463945	.	-	.	gene_id "XLOC_220982"; transcript_id "Novel_003082"; exon_number "1"; gene_type "
Dt_chr8	Cufflinks	exon	23464024	23465463	.	-	.	gene_id "XLOC_220982"; transcript_id "Novel_003082"; exon_number "2"; gene_type "
Dt_chr8	Cufflinks	exon	23465835	23465891	.	-	.	gene_id "XLOC_220982"; transcript_id "Novel_003082"; exon_number "3"; gene_type "
Dt_chr8	Cufflinks	exon	23465981	23466048	.	-	.	gene_id "XLOC_220982"; transcript_id "Novel_003082"; exon_number "4"; gene_type "
Dt_chr8	Cufflinks	exon	23466208	23466357	.	-	.	gene_id "XLOC_220982"; transcript_id "Novel_003082"; exon_number "5"; gene_type "
Dt_chr8	Cufflinks	exon	23466460	23466593	.	-	.	gene_id "XLOC_220982"; transcript_id "Novel_003082"; exon_number "6"; gene_type "
Dt_chr8	Cufflinks	exon	23466758	23466997	.	-	.	gene_id "XLOC_220982"; transcript_id "Novel_003082"; exon_number "7"; gene_type "
Dt_chr8	Cufflinks	exon	23467071	23467093	.	-	.	gene_id "XLOC_220982"; transcript_id "Novel_003082"; exon_number "8"; gene_type "
Dt_chr8	Cufflinks	exon	23461875	23463945	.	-	.	gene_id "XLOC_220982"; transcript_id "Novel_003083"; exon_number "1"; gene_type "
Dt_chr8	Cufflinks	exon	23464024	23465463	.	-	.	gene_id "XLOC_220982"; transcript_id "Novel_003083"; exon_number "2"; gene_type "
Dt_chr8	Cufflinks	exon	23465835	23465891	.	-	.	gene_id "XLOC_220982"; transcript_id "Novel_003083"; exon_number "3"; gene_type "
Dt_chr8	Cufflinks	exon	23465981	23466048	.	-	.	gene_id "XLOC_220982"; transcript_id "Novel_003083"; exon_number "4"; gene_type "
Dt_chr8	Cufflinks	exon	23466208	23466357	.	-	.	gene_id "XLOC_220982"; transcript_id "Novel_003083"; exon_number "5"; gene_type "
Dt_chr8	Cufflinks	exon	23466460	23467306	.	-	.	gene_id "XLOC_220982"; transcript_id "Novel_003083"; exon_number "6"; gene_type "
Dt_chr8	Cufflinks	exon	23461896	23463945	.	-	.	gene_id "XLOC_220982"; transcript_id "Novel_003084"; exon_number "1"; gene_type "
Dt_chr8	Cufflinks	exon	23464024	23465463	.	-	.	gene_id "XLOC_220982"; transcript_id "Novel_003084"; exon_number "2"; gene_type "
Dt_chr8	Cufflinks	exon	23465835	23465891	.	-	.	gene_id "XLOC_220982"; transcript_id "Novel_003084"; exon_number "3"; gene_type "
Dt_chr8	Cufflinks	exon	23465981	23466048	.	-	.	gene_id "XLOC_220982"; transcript_id "Novel_003084"; exon_number "4"; gene_type "
Dt_chr8	Cufflinks	exon	23466208	23467149	.	-	.	gene_id "XLOC_220982"; transcript_id "Novel_003084"; exon_number "5"; gene_type "
Dt_chr8	Cufflinks	exon	23889039	23889789	.	-	.	gene_id "XLOC_221040"; transcript_id "Novel_003085"; exon_number "1"; gene_type "

Dt_chr8	Cufflinks	exon	23890043	23890192	.	-	.	gene_id "XLOC_221040"; transcript_id "Novel_003085"; exon_number "2"; gene_type "
Dt_chr8	Cufflinks	exon	23911519	23912043	.	-	.	gene_id "XLOC_221042"; transcript_id "Novel_003086"; exon_number "1"; gene_type "
Dt_chr8	Cufflinks	exon	23913508	23913536	.	-	.	gene_id "XLOC_221042"; transcript_id "Novel_003086"; exon_number "2"; gene_type "
Dt_chr8	Cufflinks	exon	23935124	23935444	.	-	.	gene_id "XLOC_221052"; transcript_id "Novel_003087"; exon_number "1"; gene_type "
Dt_chr8	Cufflinks	exon	23935780	23935912	.	-	.	gene_id "XLOC_221052"; transcript_id "Novel_003087"; exon_number "2"; gene_type "
Dt_chr8	Cufflinks	exon	23935999	23936047	.	-	.	gene_id "XLOC_221052"; transcript_id "Novel_003087"; exon_number "3"; gene_type "
Dt_chr8	Cufflinks	exon	23937971	23938077	.	-	.	gene_id "XLOC_221052"; transcript_id "Novel_003087"; exon_number "4"; gene_type "
Dt_chr8	Cufflinks	exon	26233105	26234093	.	-	.	gene_id "XLOC_221187"; transcript_id "Novel_003088"; exon_number "1"; gene_type "
Dt_chr8	Cufflinks	exon	26234209	26234278	.	-	.	gene_id "XLOC_221187"; transcript_id "Novel_003088"; exon_number "2"; gene_type "
Dt_chr8	Cufflinks	exon	29128049	29128273	.	-	.	gene_id "XLOC_221318"; transcript_id "Novel_003089"; exon_number "1"; gene_type "
Dt_chr8	Cufflinks	exon	29129941	29130042	.	-	.	gene_id "XLOC_221318"; transcript_id "Novel_003089"; exon_number "2"; gene_type "
Dt_chr8	Cufflinks	exon	29973012	29973928	.	-	.	gene_id "XLOC_221342"; transcript_id "Novel_003090"; exon_number "1"; gene_type "
Dt_chr8	Cufflinks	exon	29974387	29974611	.	-	.	gene_id "XLOC_221342"; transcript_id "Novel_003090"; exon_number "2"; gene_type "
Dt_chr8	Cufflinks	exon	30398449	30398739	.	-	.	gene_id "XLOC_221376"; transcript_id "Novel_003091"; exon_number "1"; gene_type "
Dt_chr8	Cufflinks	exon	30398823	30398938	.	-	.	gene_id "XLOC_221376"; transcript_id "Novel_003091"; exon_number "2"; gene_type "
Dt_chr8	Cufflinks	exon	30399672	30399929	.	-	.	gene_id "XLOC_221376"; transcript_id "Novel_003091"; exon_number "3"; gene_type "
Dt_chr8	Cufflinks	exon	33017628	33018255	.	-	.	gene_id "XLOC_221578"; transcript_id "Novel_003092"; exon_number "1"; gene_type "
Dt_chr8	Cufflinks	exon	33018363	33018635	.	-	.	gene_id "XLOC_221578"; transcript_id "Novel_003092"; exon_number "2"; gene_type "
Dt_chr8	Cufflinks	exon	33652290	33653600	.	-	.	gene_id "XLOC_221618"; transcript_id "Novel_003093"; exon_number "1"; gene_type "
Dt_chr8	Cufflinks	exon	33655625	33655983	.	-	.	gene_id "XLOC_221618"; transcript_id "Novel_003093"; exon_number "2"; gene_type "
Dt_chr8	Cufflinks	exon	33663297	33663566	.	-	.	gene_id "XLOC_221620"; transcript_id "Novel_003094"; exon_number "1"; gene_type "
Dt_chr8	Cufflinks	exon	33663648	33663685	.	-	.	gene_id "XLOC_221620"; transcript_id "Novel_003094"; exon_number "2"; gene_type "
Dt_chr8	Cufflinks	exon	33663756	33664160	.	-	.	gene_id "XLOC_221620"; transcript_id "Novel_003094"; exon_number "3"; gene_type "
Dt_chr8	Cufflinks	exon	33664232	33664515	.	-	.	gene_id "XLOC_221620"; transcript_id "Novel_003094"; exon_number "4"; gene_type "
Dt_chr8	Cufflinks	exon	33664624	33664708	.	-	.	gene_id "XLOC_221620"; transcript_id "Novel_003094"; exon_number "5"; gene_type "
Dt_chr8	Cufflinks	exon	33663365	33664515	.	-	.	gene_id "XLOC_221620"; transcript_id "Novel_003095"; exon_number "1"; gene_type "
Dt_chr8	Cufflinks	exon	33664624	33664785	.	-	.	gene_id "XLOC_221620"; transcript_id "Novel_003095"; exon_number "2"; gene_type "
Dt_chr8	Cufflinks	exon	34937550	34938464	.	-	.	gene_id "XLOC_221733"; transcript_id "Novel_003096"; exon_number "1"; gene_type "
Dt_chr8	Cufflinks	exon	35559765	35560043	.	-	.	gene_id "XLOC_221779"; transcript_id "Novel_003097"; exon_number "1"; gene_type "
Dt_chr8	Cufflinks	exon	35561068	35561174	.	-	.	gene_id "XLOC_221779"; transcript_id "Novel_003097"; exon_number "2"; gene_type "
Dt_chr8	Cufflinks	exon	35562118	35562196	.	-	.	gene_id "XLOC_221779"; transcript_id "Novel_003097"; exon_number "3"; gene_type "
Dt_chr8	Cufflinks	exon	35562404	35562481	.	-	.	gene_id "XLOC_221779"; transcript_id "Novel_003097"; exon_number "4"; gene_type "
Dt_chr8	Cufflinks	exon	35562633	35563049	.	-	.	gene_id "XLOC_221779"; transcript_id "Novel_003097"; exon_number "5"; gene_type "
Dt_chr8	Cufflinks	exon	35559767	35560043	.	-	.	gene_id "XLOC_221779"; transcript_id "Novel_003098"; exon_number "1"; gene_type "
Dt_chr8	Cufflinks	exon	35561068	35562196	.	-	.	gene_id "XLOC_221779"; transcript_id "Novel_003098"; exon_number "2"; gene_type "

Dt_chr8	Cufflinks	exon	35562404	35562481	.	-	.	gene_id "XLOC_221779"; transcript_id "Novel_003098"; exon_number "3"; gene_type "
Dt_chr8	Cufflinks	exon	35562633	35563022	.	-	.	gene_id "XLOC_221779"; transcript_id "Novel_003098"; exon_number "4"; gene_type "
Dt_chr8	Cufflinks	exon	35614365	35615835	.	-	.	gene_id "XLOC_221788"; transcript_id "Novel_003099"; exon_number "1"; gene_type "
Dt_chr8	Cufflinks	exon	35616016	35616127	.	-	.	gene_id "XLOC_221788"; transcript_id "Novel_003099"; exon_number "2"; gene_type "
Dt_chr8	Cufflinks	exon	35616254	35616445	.	-	.	gene_id "XLOC_221788"; transcript_id "Novel_003099"; exon_number "3"; gene_type "
Dt_chr8	Cufflinks	exon	35616704	35616729	.	-	.	gene_id "XLOC_221788"; transcript_id "Novel_003099"; exon_number "4"; gene_type "
Dt_chr8	Cufflinks	exon	35617016	35617742	.	-	.	gene_id "XLOC_221788"; transcript_id "Novel_003099"; exon_number "5"; gene_type "
Dt_chr8	Cufflinks	exon	35614365	35615835	.	-	.	gene_id "XLOC_221788"; transcript_id "Novel_003100"; exon_number "1"; gene_type "
Dt_chr8	Cufflinks	exon	35616016	35616127	.	-	.	gene_id "XLOC_221788"; transcript_id "Novel_003100"; exon_number "2"; gene_type "
Dt_chr8	Cufflinks	exon	35616254	35616729	.	-	.	gene_id "XLOC_221788"; transcript_id "Novel_003100"; exon_number "3"; gene_type "
Dt_chr8	Cufflinks	exon	35617016	35617742	.	-	.	gene_id "XLOC_221788"; transcript_id "Novel_003100"; exon_number "4"; gene_type "
Dt_chr8	Cufflinks	exon	35615921	35616127	.	-	.	gene_id "XLOC_221788"; transcript_id "Novel_003101"; exon_number "1"; gene_type "
Dt_chr8	Cufflinks	exon	35616254	35616445	.	-	.	gene_id "XLOC_221788"; transcript_id "Novel_003101"; exon_number "2"; gene_type "
Dt_chr8	Cufflinks	exon	35616635	35616729	.	-	.	gene_id "XLOC_221788"; transcript_id "Novel_003101"; exon_number "3"; gene_type "
Dt_chr8	Cufflinks	exon	35617016	35617274	.	-	.	gene_id "XLOC_221788"; transcript_id "Novel_003101"; exon_number "4"; gene_type "
Dt_chr8	Cufflinks	exon	36699819	36699900	.	-	.	gene_id "XLOC_221892"; transcript_id "Novel_003102"; exon_number "1"; gene_type "
Dt_chr8	Cufflinks	exon	36775550	36775683	.	-	.	gene_id "XLOC_221892"; transcript_id "Novel_003102"; exon_number "2"; gene_type "
Dt_chr8	Cufflinks	exon	36776655	36776714	.	-	.	gene_id "XLOC_221892"; transcript_id "Novel_003102"; exon_number "3"; gene_type "
Dt_chr8	Cufflinks	exon	36777095	36777238	.	-	.	gene_id "XLOC_221892"; transcript_id "Novel_003102"; exon_number "4"; gene_type "
Dt_chr8	Cufflinks	exon	36774144	36776714	.	-	.	gene_id "XLOC_221892"; transcript_id "Novel_003103"; exon_number "1"; gene_type "
Dt_chr8	Cufflinks	exon	36777095	36777259	.	-	.	gene_id "XLOC_221892"; transcript_id "Novel_003103"; exon_number "2"; gene_type "
Dt_chr8	Cufflinks	exon	36774144	36774740	.	-	.	gene_id "XLOC_221892"; transcript_id "Novel_003104"; exon_number "1"; gene_type "
Dt_chr8	Cufflinks	exon	36774906	36775010	.	-	.	gene_id "XLOC_221892"; transcript_id "Novel_003104"; exon_number "2"; gene_type "
Dt_chr8	Cufflinks	exon	36776655	36776714	.	-	.	gene_id "XLOC_221892"; transcript_id "Novel_003104"; exon_number "3"; gene_type "
Dt_chr8	Cufflinks	exon	36777095	36777259	.	-	.	gene_id "XLOC_221892"; transcript_id "Novel_003104"; exon_number "4"; gene_type "
Dt_chr8	Cufflinks	exon	36774318	36775010	.	-	.	gene_id "XLOC_221892"; transcript_id "Novel_003105"; exon_number "1"; gene_type "
Dt_chr8	Cufflinks	exon	36776655	36776714	.	-	.	gene_id "XLOC_221892"; transcript_id "Novel_003105"; exon_number "2"; gene_type "
Dt_chr8	Cufflinks	exon	36777095	36777246	.	-	.	gene_id "XLOC_221892"; transcript_id "Novel_003105"; exon_number "3"; gene_type "
Dt_chr8	Cufflinks	exon	38685776	38686112	.	-	.	gene_id "XLOC_222103"; transcript_id "Novel_003106"; exon_number "1"; gene_type "
Dt_chr8	Cufflinks	exon	38686293	38686454	.	-	.	gene_id "XLOC_222103"; transcript_id "Novel_003106"; exon_number "2"; gene_type "
Dt_chr8	Cufflinks	exon	38686876	38686915	.	-	.	gene_id "XLOC_222103"; transcript_id "Novel_003106"; exon_number "3"; gene_type "
Dt_chr8	Cufflinks	exon	38687329	38687406	.	-	.	gene_id "XLOC_222103"; transcript_id "Novel_003106"; exon_number "4"; gene_type "
Dt_chr8	Cufflinks	exon	38687545	38687643	.	-	.	gene_id "XLOC_222103"; transcript_id "Novel_003106"; exon_number "5"; gene_type "
Dt_chr8	Cufflinks	exon	38687774	38687815	.	-	.	gene_id "XLOC_222103"; transcript_id "Novel_003106"; exon_number "6"; gene_type "
Dt_chr8	Cufflinks	exon	38687997	38688045	.	-	.	gene_id "XLOC_222103"; transcript_id "Novel_003106"; exon_number "7"; gene_type "

Dt_chr8	Cufflinks	exon	38688203	38688322	.	-	.	gene_id "XLOC_222103"; transcript_id "Novel_003106"; exon_number "8"; gene_type "
Dt_chr8	Cufflinks	exon	38685779	38686112	.	-	.	gene_id "XLOC_222103"; transcript_id "Novel_003107"; exon_number "1"; gene_type "
Dt_chr8	Cufflinks	exon	38686293	38686454	.	-	.	gene_id "XLOC_222103"; transcript_id "Novel_003107"; exon_number "2"; gene_type "
Dt_chr8	Cufflinks	exon	38686876	38686915	.	-	.	gene_id "XLOC_222103"; transcript_id "Novel_003107"; exon_number "3"; gene_type "
Dt_chr8	Cufflinks	exon	38687329	38687406	.	-	.	gene_id "XLOC_222103"; transcript_id "Novel_003107"; exon_number "4"; gene_type "
Dt_chr8	Cufflinks	exon	38687545	38687643	.	-	.	gene_id "XLOC_222103"; transcript_id "Novel_003107"; exon_number "5"; gene_type "
Dt_chr8	Cufflinks	exon	38687774	38687815	.	-	.	gene_id "XLOC_222103"; transcript_id "Novel_003107"; exon_number "6"; gene_type "
Dt_chr8	Cufflinks	exon	38687997	38688045	.	-	.	gene_id "XLOC_222103"; transcript_id "Novel_003107"; exon_number "7"; gene_type "
Dt_chr8	Cufflinks	exon	38688276	38688395	.	-	.	gene_id "XLOC_222103"; transcript_id "Novel_003107"; exon_number "8"; gene_type "
Dt_chr8	Cufflinks	exon	38685859	38686112	.	-	.	gene_id "XLOC_222103"; transcript_id "Novel_003108"; exon_number "1"; gene_type "
Dt_chr8	Cufflinks	exon	38686293	38686454	.	-	.	gene_id "XLOC_222103"; transcript_id "Novel_003108"; exon_number "2"; gene_type "
Dt_chr8	Cufflinks	exon	38686876	38686915	.	-	.	gene_id "XLOC_222103"; transcript_id "Novel_003108"; exon_number "3"; gene_type "
Dt_chr8	Cufflinks	exon	38687329	38687406	.	-	.	gene_id "XLOC_222103"; transcript_id "Novel_003108"; exon_number "4"; gene_type "
Dt_chr8	Cufflinks	exon	38687545	38687645	.	-	.	gene_id "XLOC_222103"; transcript_id "Novel_003108"; exon_number "5"; gene_type "
Dt_chr8	Cufflinks	exon	39185625	39185869	.	-	.	gene_id "XLOC_222196"; transcript_id "Novel_003109"; exon_number "1"; gene_type "
Dt_chr8	Cufflinks	exon	39186336	39186413	.	-	.	gene_id "XLOC_222196"; transcript_id "Novel_003109"; exon_number "2"; gene_type "
Dt_chr8	Cufflinks	exon	39187504	39187774	.	-	.	gene_id "XLOC_222196"; transcript_id "Novel_003109"; exon_number "3"; gene_type "
Dt_chr8	Cufflinks	exon	40190358	40190693	.	-	.	gene_id "XLOC_222330"; transcript_id "Novel_003110"; exon_number "1"; gene_type "
Dt_chr8	Cufflinks	exon	40191188	40191248	.	-	.	gene_id "XLOC_222330"; transcript_id "Novel_003110"; exon_number "2"; gene_type "
Dt_chr8	Cufflinks	exon	43469609	43469727	.	-	.	gene_id "XLOC_222706"; transcript_id "Novel_003111"; exon_number "1"; gene_type "
Dt_chr8	Cufflinks	exon	43470397	43470498	.	-	.	gene_id "XLOC_222706"; transcript_id "Novel_003111"; exon_number "2"; gene_type "
Dt_chr8	Cufflinks	exon	43470629	43470676	.	-	.	gene_id "XLOC_222706"; transcript_id "Novel_003111"; exon_number "3"; gene_type "
Dt_chr8	Cufflinks	exon	44899279	44899295	.	-	.	gene_id "XLOC_222903"; transcript_id "Novel_003112"; exon_number "1"; gene_type "
Dt_chr8	Cufflinks	exon	44899409	44899520	.	-	.	gene_id "XLOC_222903"; transcript_id "Novel_003112"; exon_number "2"; gene_type "
Dt_chr8	Cufflinks	exon	44899699	44899894	.	-	.	gene_id "XLOC_222903"; transcript_id "Novel_003112"; exon_number "3"; gene_type "
Dt_chr8	Cufflinks	exon	45394866	45395684	.	-	.	gene_id "XLOC_222962"; transcript_id "Novel_003113"; exon_number "1"; gene_type "
Dt_chr8	Cufflinks	exon	45395765	45396196	.	-	.	gene_id "XLOC_222962"; transcript_id "Novel_003113"; exon_number "2"; gene_type "
Dt_chr8	Cufflinks	exon	46729993	46730313	.	-	.	gene_id "XLOC_223135"; transcript_id "Novel_003114"; exon_number "1"; gene_type "
Dt_chr8	Cufflinks	exon	46730649	46730709	.	-	.	gene_id "XLOC_223135"; transcript_id "Novel_003114"; exon_number "2"; gene_type "
Dt_chr8	Cufflinks	exon	46731698	46731716	.	-	.	gene_id "XLOC_223135"; transcript_id "Novel_003114"; exon_number "3"; gene_type "
Dt_chr8	Cufflinks	exon	46732345	46732506	.	-	.	gene_id "XLOC_223135"; transcript_id "Novel_003114"; exon_number "4"; gene_type "
Dt_chr8	Cufflinks	exon	46732573	46732851	.	-	.	gene_id "XLOC_223135"; transcript_id "Novel_003114"; exon_number "5"; gene_type "
Dt_chr8	Cufflinks	exon	46729993	46730313	.	-	.	gene_id "XLOC_223135"; transcript_id "Novel_003115"; exon_number "1"; gene_type "
Dt_chr8	Cufflinks	exon	46730649	46730709	.	-	.	gene_id "XLOC_223135"; transcript_id "Novel_003115"; exon_number "2"; gene_type "
Dt_chr8	Cufflinks	exon	46731698	46731716	.	-	.	gene_id "XLOC_223135"; transcript_id "Novel_003115"; exon_number "3"; gene_type "

Dt_chr8	Cufflinks	exon	46732345	46732484	.	-	.	gene_id "XLOC_223135"; transcript_id "Novel_003115"; exon_number "4"; gene_type "
Dt_chr8	Cufflinks	exon	46732573	46732851	.	-	.	gene_id "XLOC_223135"; transcript_id "Novel_003115"; exon_number "5"; gene_type "
Dt_chr8	Cufflinks	exon	46879417	46879670	.	-	.	gene_id "XLOC_223153"; transcript_id "Novel_003116"; exon_number "1"; gene_type "
Dt_chr8	Cufflinks	exon	47081337	47082109	.	-	.	gene_id "XLOC_223188"; transcript_id "Novel_003117"; exon_number "1"; gene_type "
Dt_chr8	Cufflinks	exon	47082657	47082788	.	-	.	gene_id "XLOC_223188"; transcript_id "Novel_003117"; exon_number "2"; gene_type "
Dt_chr8	Cufflinks	exon	47082946	47083095	.	-	.	gene_id "XLOC_223188"; transcript_id "Novel_003117"; exon_number "3"; gene_type "
Dt_chr8	Cufflinks	exon	47083602	47083757	.	-	.	gene_id "XLOC_223188"; transcript_id "Novel_003117"; exon_number "4"; gene_type "
Dt_chr8	Cufflinks	exon	47084049	47084172	.	-	.	gene_id "XLOC_223188"; transcript_id "Novel_003118"; exon_number "1"; gene_type "
Dt_chr8	Cufflinks	exon	47084396	47084491	.	-	.	gene_id "XLOC_223188"; transcript_id "Novel_003118"; exon_number "2"; gene_type "
Dt_chr8	Cufflinks	exon	47084672	47084712	.	-	.	gene_id "XLOC_223188"; transcript_id "Novel_003118"; exon_number "3"; gene_type "
Dt_chr8	Cufflinks	exon	47344044	47344517	.	-	.	gene_id "XLOC_223231"; transcript_id "Novel_003119"; exon_number "1"; gene_type "
Dt_chr8	Cufflinks	exon	47344586	47344640	.	-	.	gene_id "XLOC_223231"; transcript_id "Novel_003119"; exon_number "2"; gene_type "
Dt_chr8	Cufflinks	exon	47344761	47344864	.	-	.	gene_id "XLOC_223231"; transcript_id "Novel_003119"; exon_number "3"; gene_type "
Dt_chr8	Cufflinks	exon	47346415	47346734	.	-	.	gene_id "XLOC_223231"; transcript_id "Novel_003119"; exon_number "4"; gene_type "
Dt_chr8	Cufflinks	exon	47609114	47609211	.	-	.	gene_id "XLOC_223270"; transcript_id "Novel_003120"; exon_number "1"; gene_type "
Dt_chr8	Cufflinks	exon	47609527	47609772	.	-	.	gene_id "XLOC_223270"; transcript_id "Novel_003120"; exon_number "2"; gene_type "
Dt_chr8	Cufflinks	exon	47609861	47609933	.	-	.	gene_id "XLOC_223270"; transcript_id "Novel_003120"; exon_number "3"; gene_type "
Dt_chr8	Cufflinks	exon	47610378	47610445	.	-	.	gene_id "XLOC_223270"; transcript_id "Novel_003120"; exon_number "4"; gene_type "
Dt_chr8	Cufflinks	exon	47610568	47610655	.	-	.	gene_id "XLOC_223270"; transcript_id "Novel_003120"; exon_number "5"; gene_type "
Dt_chr8	Cufflinks	exon	47611414	47611488	.	-	.	gene_id "XLOC_223270"; transcript_id "Novel_003120"; exon_number "6"; gene_type "
Dt_chr8	Cufflinks	exon	47611626	47611665	.	-	.	gene_id "XLOC_223270"; transcript_id "Novel_003120"; exon_number "7"; gene_type "
Dt_chr8	Cufflinks	exon	47611802	47611898	.	-	.	gene_id "XLOC_223270"; transcript_id "Novel_003120"; exon_number "8"; gene_type "
Dt_chr8	Cufflinks	exon	47612270	47612377	.	-	.	gene_id "XLOC_223270"; transcript_id "Novel_003120"; exon_number "9"; gene_type "
Dt_chr8	Cufflinks	exon	47612596	47612691	.	-	.	gene_id "XLOC_223270"; transcript_id "Novel_003120"; exon_number "10"; gene_type
Dt_chr8	Cufflinks	exon	47612812	47612970	.	-	.	gene_id "XLOC_223270"; transcript_id "Novel_003120"; exon_number "11"; gene_type
Dt_chr8	Cufflinks	exon	47609168	47609772	.	-	.	gene_id "XLOC_223270"; transcript_id "Novel_003121"; exon_number "1"; gene_type "
Dt_chr8	Cufflinks	exon	47609861	47609933	.	-	.	gene_id "XLOC_223270"; transcript_id "Novel_003121"; exon_number "2"; gene_type "
Dt_chr8	Cufflinks	exon	47610378	47610445	.	-	.	gene_id "XLOC_223270"; transcript_id "Novel_003121"; exon_number "3"; gene_type "
Dt_chr8	Cufflinks	exon	47610568	47610655	.	-	.	gene_id "XLOC_223270"; transcript_id "Novel_003121"; exon_number "4"; gene_type "
Dt_chr8	Cufflinks	exon	47611414	47611488	.	-	.	gene_id "XLOC_223270"; transcript_id "Novel_003121"; exon_number "5"; gene_type "
Dt_chr8	Cufflinks	exon	47611626	47611665	.	-	.	gene_id "XLOC_223270"; transcript_id "Novel_003121"; exon_number "6"; gene_type "
Dt_chr8	Cufflinks	exon	47611802	47611898	.	-	.	gene_id "XLOC_223270"; transcript_id "Novel_003121"; exon_number "7"; gene_type "
Dt_chr8	Cufflinks	exon	47612270	47612377	.	-	.	gene_id "XLOC_223270"; transcript_id "Novel_003121"; exon_number "8"; gene_type "
Dt_chr8	Cufflinks	exon	47612596	47612691	.	-	.	gene_id "XLOC_223270"; transcript_id "Novel_003121"; exon_number "9"; gene_type "
Dt_chr8	Cufflinks	exon	47612812	47613016	.	-	.	gene_id "XLOC_223270"; transcript_id "Novel_003121"; exon_number "10"; gene_type

Dt_chr8	Cufflinks	exon	47730884	47731221	.	-	.	gene_id "XLOC_223286"; transcript_id "Novel_003122"; exon_number "1"; gene_type "
Dt_chr8	Cufflinks	exon	47731840	47731905	.	-	.	gene_id "XLOC_223286"; transcript_id "Novel_003122"; exon_number "2"; gene_type "
Dt_chr8	Cufflinks	exon	48530206	48532602	.	-	.	gene_id "XLOC_223364"; transcript_id "Novel_003123"; exon_number "1"; gene_type "
Dt_chr8	Cufflinks	exon	50581467	50582606	.	-	.	gene_id "XLOC_223695"; transcript_id "Novel_003124"; exon_number "1"; gene_type "
Dt_chr8	Cufflinks	exon	50582687	50582853	.	-	.	gene_id "XLOC_223695"; transcript_id "Novel_003124"; exon_number "2"; gene_type "
Dt_chr8	Cufflinks	exon	50583956	50584792	.	-	.	gene_id "XLOC_223695"; transcript_id "Novel_003124"; exon_number "3"; gene_type "
Dt_chr8	Cufflinks	exon	50585902	50586405	.	-	.	gene_id "XLOC_223697"; transcript_id "Novel_003125"; exon_number "1"; gene_type "
Dt_chr8	Cufflinks	exon	50587039	50587202	.	-	.	gene_id "XLOC_223697"; transcript_id "Novel_003125"; exon_number "2"; gene_type "
Dt_chr8	Cufflinks	exon	50587471	50587564	.	-	.	gene_id "XLOC_223697"; transcript_id "Novel_003125"; exon_number "3"; gene_type "
Dt_chr8	Cufflinks	exon	50587646	50587774	.	-	.	gene_id "XLOC_223697"; transcript_id "Novel_003125"; exon_number "4"; gene_type "
Dt_chr8	Cufflinks	exon	50587846	50587933	.	-	.	gene_id "XLOC_223697"; transcript_id "Novel_003125"; exon_number "5"; gene_type "
Dt_chr8	Cufflinks	exon	50588499	50588596	.	-	.	gene_id "XLOC_223697"; transcript_id "Novel_003125"; exon_number "6"; gene_type "
Dt_chr8	Cufflinks	exon	50588709	50588827	.	-	.	gene_id "XLOC_223697"; transcript_id "Novel_003125"; exon_number "7"; gene_type "
Dt_chr8	Cufflinks	exon	50589197	50589306	.	-	.	gene_id "XLOC_223697"; transcript_id "Novel_003125"; exon_number "8"; gene_type "
Dt_chr8	Cufflinks	exon	50589401	50589607	.	-	.	gene_id "XLOC_223697"; transcript_id "Novel_003125"; exon_number "9"; gene_type "
Dt_chr8	Cufflinks	exon	50589027	50589306	.	-	.	gene_id "XLOC_223697"; transcript_id "Novel_003126"; exon_number "1"; gene_type "
Dt_chr8	Cufflinks	exon	50589401	50589611	.	-	.	gene_id "XLOC_223697"; transcript_id "Novel_003126"; exon_number "2"; gene_type "
Dt_chr8	Cufflinks	exon	52001579	52002360	.	-	.	gene_id "XLOC_223835"; transcript_id "Novel_003127"; exon_number "1"; gene_type "
Dt_chr8	Cufflinks	exon	52002436	52002594	.	-	.	gene_id "XLOC_223835"; transcript_id "Novel_003127"; exon_number "2"; gene_type "
Dt_chr8	Cufflinks	exon	52002700	52002798	.	-	.	gene_id "XLOC_223835"; transcript_id "Novel_003127"; exon_number "3"; gene_type "
Dt_chr8	Cufflinks	exon	52002975	52003228	.	-	.	gene_id "XLOC_223835"; transcript_id "Novel_003127"; exon_number "4"; gene_type "
Dt_chr8	Cufflinks	exon	52003631	52004358	.	-	.	gene_id "XLOC_223835"; transcript_id "Novel_003127"; exon_number "5"; gene_type "
Dt_chr8	Cufflinks	exon	52136042	52137565	.	-	.	gene_id "XLOC_223857"; transcript_id "Novel_003128"; exon_number "1"; gene_type "
Dt_chr8	Cufflinks	exon	52140447	52140525	.	-	.	gene_id "XLOC_223857"; transcript_id "Novel_003128"; exon_number "2"; gene_type "
Dt_chr8	Cufflinks	exon	52140703	52141248	.	-	.	gene_id "XLOC_223857"; transcript_id "Novel_003128"; exon_number "3"; gene_type "
Dt_chr8	Cufflinks	exon	52136152	52137565	.	-	.	gene_id "XLOC_223857"; transcript_id "Novel_003129"; exon_number "1"; gene_type "
Dt_chr8	Cufflinks	exon	52140447	52140517	.	-	.	gene_id "XLOC_223857"; transcript_id "Novel_003129"; exon_number "2"; gene_type "
Dt_chr8	Cufflinks	exon	52140703	52141193	.	-	.	gene_id "XLOC_223857"; transcript_id "Novel_003129"; exon_number "3"; gene_type "
Dt_chr8	Cufflinks	exon	52710862	52712522	.	-	.	gene_id "XLOC_223970"; transcript_id "Novel_003130"; exon_number "1"; gene_type "
Dt_chr8	Cufflinks	exon	52718415	52718848	.	-	.	gene_id "XLOC_223970"; transcript_id "Novel_003130"; exon_number "2"; gene_type "
Dt_chr8	Cufflinks	exon	53298304	53299050	.	-	.	gene_id "XLOC_224059"; transcript_id "Novel_003131"; exon_number "1"; gene_type "
Dt_chr8	Cufflinks	exon	53299860	53300075	.	-	.	gene_id "XLOC_224059"; transcript_id "Novel_003131"; exon_number "2"; gene_type "
Dt_chr8	Cufflinks	exon	53300799	53301155	.	-	.	gene_id "XLOC_224059"; transcript_id "Novel_003131"; exon_number "3"; gene_type "
Dt_chr8	Cufflinks	exon	53861245	53861600	.	-	.	gene_id "XLOC_224139"; transcript_id "Novel_003132"; exon_number "1"; gene_type "
Dt_chr8	Cufflinks	exon	53861697	53862171	.	-	.	gene_id "XLOC_224139"; transcript_id "Novel_003132"; exon_number "2"; gene_type "

Dt_chr8	Cufflinks	exon	53882034	53882295	.	-	.	gene_id "XLOC_224142"; transcript_id "Novel_003133"; exon_number "1"; gene_type "
Dt_chr8	Cufflinks	exon	53882425	53882803	.	-	.	gene_id "XLOC_224142"; transcript_id "Novel_003133"; exon_number "2"; gene_type "
Dt_chr8	Cufflinks	exon	53882923	53883414	.	-	.	gene_id "XLOC_224142"; transcript_id "Novel_003133"; exon_number "3"; gene_type "
Dt_chr8	Cufflinks	exon	53978787	53979143	.	-	.	gene_id "XLOC_224164"; transcript_id "Novel_003134"; exon_number "1"; gene_type "
Dt_chr8	Cufflinks	exon	53980738	53980983	.	-	.	gene_id "XLOC_224164"; transcript_id "Novel_003134"; exon_number "2"; gene_type "
Dt_chr8	Cufflinks	exon	53978787	53978920	.	-	.	gene_id "XLOC_224164"; transcript_id "Novel_003135"; exon_number "1"; gene_type "
Dt_chr8	Cufflinks	exon	53979088	53979143	.	-	.	gene_id "XLOC_224164"; transcript_id "Novel_003135"; exon_number "2"; gene_type "
Dt_chr8	Cufflinks	exon	53980738	53980983	.	-	.	gene_id "XLOC_224164"; transcript_id "Novel_003135"; exon_number "3"; gene_type "
Dt_chr8	Cufflinks	exon	55171355	55172557	.	-	.	gene_id "XLOC_224322"; transcript_id "Novel_003136"; exon_number "1"; gene_type "
Dt_chr8	Cufflinks	exon	55172639	55172697	.	-	.	gene_id "XLOC_224322"; transcript_id "Novel_003136"; exon_number "2"; gene_type "
Dt_chr8	Cufflinks	exon	55173371	55173689	.	-	.	gene_id "XLOC_224322"; transcript_id "Novel_003136"; exon_number "3"; gene_type "
Dt_chr8	Cufflinks	exon	55174045	55174383	.	-	.	gene_id "XLOC_224322"; transcript_id "Novel_003136"; exon_number "4"; gene_type "
Dt_chr8	Cufflinks	exon	55651545	55651955	.	-	.	gene_id "XLOC_224421"; transcript_id "Novel_003137"; exon_number "1"; gene_type "
Dt_chr8	Cufflinks	exon	55652046	55652260	.	-	.	gene_id "XLOC_224421"; transcript_id "Novel_003137"; exon_number "2"; gene_type "
Dt_chr9	Cufflinks	exon	947726	947885	.	+	.	gene_id "XLOC_224815"; transcript_id "Novel_003138"; exon_number "1"; gene_type "
Dt_chr9	Cufflinks	exon	948185	948238	.	+	.	gene_id "XLOC_224815"; transcript_id "Novel_003138"; exon_number "2"; gene_type "
Dt_chr9	Cufflinks	exon	948912	950450	.	+	.	gene_id "XLOC_224815"; transcript_id "Novel_003138"; exon_number "3"; gene_type "
Dt_chr9	Cufflinks	exon	947730	947885	.	+	.	gene_id "XLOC_224815"; transcript_id "Novel_003139"; exon_number "1"; gene_type "
Dt_chr9	Cufflinks	exon	948185	948238	.	+	.	gene_id "XLOC_224815"; transcript_id "Novel_003139"; exon_number "2"; gene_type "
Dt_chr9	Cufflinks	exon	948912	949061	.	+	.	gene_id "XLOC_224815"; transcript_id "Novel_003139"; exon_number "3"; gene_type "
Dt_chr9	Cufflinks	exon	949704	949783	.	+	.	gene_id "XLOC_224815"; transcript_id "Novel_003139"; exon_number "4"; gene_type "
Dt_chr9	Cufflinks	exon	1129342	1129674	.	+	.	gene_id "XLOC_224839"; transcript_id "Novel_003140"; exon_number "1"; gene_type "
Dt_chr9	Cufflinks	exon	1130291	1130412	.	+	.	gene_id "XLOC_224839"; transcript_id "Novel_003140"; exon_number "2"; gene_type "
Dt_chr9	Cufflinks	exon	1130509	1130663	.	+	.	gene_id "XLOC_224839"; transcript_id "Novel_003140"; exon_number "3"; gene_type "
Dt_chr9	Cufflinks	exon	1130737	1130887	.	+	.	gene_id "XLOC_224839"; transcript_id "Novel_003140"; exon_number "4"; gene_type "
Dt_chr9	Cufflinks	exon	1131257	1131291	.	+	.	gene_id "XLOC_224839"; transcript_id "Novel_003140"; exon_number "5"; gene_type "
Dt_chr9	Cufflinks	exon	1131988	1132063	.	+	.	gene_id "XLOC_224839"; transcript_id "Novel_003140"; exon_number "6"; gene_type "
Dt_chr9	Cufflinks	exon	1132152	1132215	.	+	.	gene_id "XLOC_224839"; transcript_id "Novel_003140"; exon_number "7"; gene_type "
Dt_chr9	Cufflinks	exon	1132369	1132559	.	+	.	gene_id "XLOC_224839"; transcript_id "Novel_003140"; exon_number "8"; gene_type "
Dt_chr9	Cufflinks	exon	1133004	1133258	.	+	.	gene_id "XLOC_224839"; transcript_id "Novel_003140"; exon_number "9"; gene_type "
Dt_chr9	Cufflinks	exon	1133616	1133717	.	+	.	gene_id "XLOC_224839"; transcript_id "Novel_003140"; exon_number "10"; gene_type
Dt_chr9	Cufflinks	exon	1133859	1133892	.	+	.	gene_id "XLOC_224839"; transcript_id "Novel_003140"; exon_number "11"; gene_type
Dt_chr9	Cufflinks	exon	1129472	1129674	.	+	.	gene_id "XLOC_224839"; transcript_id "Novel_003141"; exon_number "1"; gene_type "
Dt_chr9	Cufflinks	exon	1130291	1130412	.	+	.	gene_id "XLOC_224839"; transcript_id "Novel_003141"; exon_number "2"; gene_type "
Dt_chr9	Cufflinks	exon	1130509	1130663	.	+	.	gene_id "XLOC_224839"; transcript_id "Novel_003141"; exon_number "3"; gene_type "

Dt_chr9	Cufflinks	exon	1130737	1130887	.	+	.	gene_id "XLOC_224839"; transcript_id "Novel_003141"; exon_number "4"; gene_type "
Dt_chr9	Cufflinks	exon	1131257	1131291	.	+	.	gene_id "XLOC_224839"; transcript_id "Novel_003141"; exon_number "5"; gene_type "
Dt_chr9	Cufflinks	exon	1131988	1132063	.	+	.	gene_id "XLOC_224839"; transcript_id "Novel_003141"; exon_number "6"; gene_type "
Dt_chr9	Cufflinks	exon	1132152	1132215	.	+	.	gene_id "XLOC_224839"; transcript_id "Novel_003141"; exon_number "7"; gene_type "
Dt_chr9	Cufflinks	exon	1132369	1132559	.	+	.	gene_id "XLOC_224839"; transcript_id "Novel_003141"; exon_number "8"; gene_type "
Dt_chr9	Cufflinks	exon	1133004	1133258	.	+	.	gene_id "XLOC_224839"; transcript_id "Novel_003141"; exon_number "9"; gene_type "
Dt_chr9	Cufflinks	exon	1133616	1133769	.	+	.	gene_id "XLOC_224839"; transcript_id "Novel_003141"; exon_number "10"; gene_type
Dt_chr9	Cufflinks	exon	1130337	1130412	.	+	.	gene_id "XLOC_224839"; transcript_id "Novel_003142"; exon_number "1"; gene_type "
Dt_chr9	Cufflinks	exon	1130509	1130663	.	+	.	gene_id "XLOC_224839"; transcript_id "Novel_003142"; exon_number "2"; gene_type "
Dt_chr9	Cufflinks	exon	1130737	1131751	.	+	.	gene_id "XLOC_224839"; transcript_id "Novel_003142"; exon_number "3"; gene_type "
Dt_chr9	Cufflinks	exon	1248035	1249169	.	+	.	gene_id "XLOC_224855"; transcript_id "Novel_003143"; exon_number "1"; gene_type "
Dt_chr9	Cufflinks	exon	1249274	1249320	.	+	.	gene_id "XLOC_224855"; transcript_id "Novel_003143"; exon_number "2"; gene_type "
Dt_chr9	Cufflinks	exon	2904413	2904459	.	+	.	gene_id "XLOC_225025"; transcript_id "Novel_003144"; exon_number "1"; gene_type "
Dt_chr9	Cufflinks	exon	2904565	2904692	.	+	.	gene_id "XLOC_225025"; transcript_id "Novel_003144"; exon_number "2"; gene_type "
Dt_chr9	Cufflinks	exon	2905896	2905943	.	+	.	gene_id "XLOC_225025"; transcript_id "Novel_003144"; exon_number "3"; gene_type "
Dt_chr9	Cufflinks	exon	2906669	2907073	.	+	.	gene_id "XLOC_225025"; transcript_id "Novel_003144"; exon_number "4"; gene_type "
Dt_chr9	Cufflinks	exon	2904508	2904692	.	+	.	gene_id "XLOC_225025"; transcript_id "Novel_003145"; exon_number "1"; gene_type "
Dt_chr9	Cufflinks	exon	2905896	2905943	.	+	.	gene_id "XLOC_225025"; transcript_id "Novel_003145"; exon_number "2"; gene_type "
Dt_chr9	Cufflinks	exon	2906669	2907032	.	+	.	gene_id "XLOC_225025"; transcript_id "Novel_003145"; exon_number "3"; gene_type "
Dt_chr9	Cufflinks	exon	2943865	2947134	.	+	.	gene_id "XLOC_225032"; transcript_id "Novel_003146"; exon_number "1"; gene_type "
Dt_chr9	Cufflinks	exon	7604631	7604858	.	+	.	gene_id "XLOC_225380"; transcript_id "Novel_003147"; exon_number "1"; gene_type "
Dt_chr9	Cufflinks	exon	8279021	8279296	.	+	.	gene_id "XLOC_225441"; transcript_id "Novel_003148"; exon_number "1"; gene_type "
Dt_chr9	Cufflinks	exon	8644216	8644513	.	+	.	gene_id "XLOC_225485"; transcript_id "Novel_003149"; exon_number "1"; gene_type "
Dt_chr9	Cufflinks	exon	17968861	17969207	.	+	.	gene_id "XLOC_225990"; transcript_id "Novel_003150"; exon_number "1"; gene_type "
Dt_chr9	Cufflinks	exon	17969845	17969898	.	+	.	gene_id "XLOC_225990"; transcript_id "Novel_003150"; exon_number "2"; gene_type "
Dt_chr9	Cufflinks	exon	17970209	17970364	.	+	.	gene_id "XLOC_225990"; transcript_id "Novel_003150"; exon_number "3"; gene_type "
Dt_chr9	Cufflinks	exon	17970772	17970817	.	+	.	gene_id "XLOC_225990"; transcript_id "Novel_003150"; exon_number "4"; gene_type "
Dt_chr9	Cufflinks	exon	17971151	17971175	.	+	.	gene_id "XLOC_225990"; transcript_id "Novel_003150"; exon_number "5"; gene_type "
Dt_chr9	Cufflinks	exon	23225768	23225871	.	+	.	gene_id "XLOC_226315"; transcript_id "Novel_003151"; exon_number "1"; gene_type "
Dt_chr9	Cufflinks	exon	23226190	23226295	.	+	.	gene_id "XLOC_226315"; transcript_id "Novel_003151"; exon_number "2"; gene_type "
Dt_chr9	Cufflinks	exon	23226431	23226483	.	+	.	gene_id "XLOC_226315"; transcript_id "Novel_003151"; exon_number "3"; gene_type "
Dt_chr9	Cufflinks	exon	23226581	23226662	.	+	.	gene_id "XLOC_226315"; transcript_id "Novel_003151"; exon_number "4"; gene_type "
Dt_chr9	Cufflinks	exon	23227208	23227273	.	+	.	gene_id "XLOC_226315"; transcript_id "Novel_003151"; exon_number "5"; gene_type "
Dt_chr9	Cufflinks	exon	23227388	23227455	.	+	.	gene_id "XLOC_226315"; transcript_id "Novel_003151"; exon_number "6"; gene_type "
Dt_chr9	Cufflinks	exon	23227582	23227800	.	+	.	gene_id "XLOC_226315"; transcript_id "Novel_003151"; exon_number "7"; gene_type "

Dt_chr9	Cufflinks	exon	23228013	23228505	.	+	.	gene_id "XLOC_226315"; transcript_id "Novel_003151"; exon_number "8"; gene_type "
Dt_chr9	Cufflinks	exon	23229011	23229280	.	+	.	gene_id "XLOC_226315"; transcript_id "Novel_003151"; exon_number "9"; gene_type "
Dt_chr9	Cufflinks	exon	23230069	23230555	.	+	.	gene_id "XLOC_226315"; transcript_id "Novel_003151"; exon_number "10"; gene_type
Dt_chr9	Cufflinks	exon	23228566	23229280	.	+	.	gene_id "XLOC_226315"; transcript_id "Novel_003152"; exon_number "1"; gene_type "
Dt_chr9	Cufflinks	exon	23230069	23230769	.	+	.	gene_id "XLOC_226315"; transcript_id "Novel_003152"; exon_number "2"; gene_type "
Dt_chr9	Cufflinks	exon	23228570	23228692	.	+	.	gene_id "XLOC_226315"; transcript_id "Novel_003153"; exon_number "1"; gene_type "
Dt_chr9	Cufflinks	exon	23229011	23229280	.	+	.	gene_id "XLOC_226315"; transcript_id "Novel_003153"; exon_number "2"; gene_type "
Dt_chr9	Cufflinks	exon	23230069	23230775	.	+	.	gene_id "XLOC_226315"; transcript_id "Novel_003153"; exon_number "3"; gene_type "
Dt_chr9	Cufflinks	exon	24279840	24280206	.	+	.	gene_id "XLOC_226383"; transcript_id "Novel_003154"; exon_number "1"; gene_type "
Dt_chr9	Cufflinks	exon	24280414	24281803	.	+	.	gene_id "XLOC_226383"; transcript_id "Novel_003154"; exon_number "2"; gene_type "
Dt_chr9	Cufflinks	exon	24279958	24281371	.	+	.	gene_id "XLOC_226383"; transcript_id "Novel_003155"; exon_number "1"; gene_type "
Dt_chr9	Cufflinks	exon	24281504	24281814	.	+	.	gene_id "XLOC_226383"; transcript_id "Novel_003155"; exon_number "2"; gene_type "
Dt_chr9	Cufflinks	exon	24946790	24946996	.	+	.	gene_id "XLOC_226402"; transcript_id "Novel_003156"; exon_number "1"; gene_type "
Dt_chr9	Cufflinks	exon	24963319	24963511	.	+	.	gene_id "XLOC_226410"; transcript_id "Novel_003157"; exon_number "1"; gene_type "
Dt_chr9	Cufflinks	exon	24963601	24963739	.	+	.	gene_id "XLOC_226410"; transcript_id "Novel_003157"; exon_number "2"; gene_type "
Dt_chr9	Cufflinks	exon	24963922	24964109	.	+	.	gene_id "XLOC_226410"; transcript_id "Novel_003157"; exon_number "3"; gene_type "
Dt_chr9	Cufflinks	exon	24964264	24964804	.	+	.	gene_id "XLOC_226410"; transcript_id "Novel_003157"; exon_number "4"; gene_type "
Dt_chr9	Cufflinks	exon	24964874	24965231	.	+	.	gene_id "XLOC_226410"; transcript_id "Novel_003157"; exon_number "5"; gene_type "
Dt_chr9	Cufflinks	exon	24964267	24964540	.	+	.	gene_id "XLOC_226410"; transcript_id "Novel_003158"; exon_number "1"; gene_type "
Dt_chr9	Cufflinks	exon	24964608	24965140	.	+	.	gene_id "XLOC_226410"; transcript_id "Novel_003158"; exon_number "2"; gene_type "
Dt_chr9	Cufflinks	exon	24985488	24985606	.	+	.	gene_id "XLOC_226412"; transcript_id "Novel_003159"; exon_number "1"; gene_type "
Dt_chr9	Cufflinks	exon	24985800	24986359	.	+	.	gene_id "XLOC_226412"; transcript_id "Novel_003159"; exon_number "2"; gene_type "
Dt_chr9	Cufflinks	exon	24986529	24986606	.	+	.	gene_id "XLOC_226412"; transcript_id "Novel_003159"; exon_number "3"; gene_type "
Dt_chr9	Cufflinks	exon	24986727	24986802	.	+	.	gene_id "XLOC_226412"; transcript_id "Novel_003159"; exon_number "4"; gene_type "
Dt_chr9	Cufflinks	exon	24986903	24986925	.	+	.	gene_id "XLOC_226412"; transcript_id "Novel_003159"; exon_number "5"; gene_type "
Dt_chr9	Cufflinks	exon	24987045	24987145	.	+	.	gene_id "XLOC_226412"; transcript_id "Novel_003159"; exon_number "6"; gene_type "
Dt_chr9	Cufflinks	exon	24987691	24987785	.	+	.	gene_id "XLOC_226412"; transcript_id "Novel_003159"; exon_number "7"; gene_type "
Dt_chr9	Cufflinks	exon	24987996	24988064	.	+	.	gene_id "XLOC_226412"; transcript_id "Novel_003159"; exon_number "8"; gene_type "
Dt_chr9	Cufflinks	exon	24993342	24993411	.	+	.	gene_id "XLOC_226412"; transcript_id "Novel_003159"; exon_number "9"; gene_type "
Dt_chr9	Cufflinks	exon	24993898	24993961	.	+	.	gene_id "XLOC_226412"; transcript_id "Novel_003159"; exon_number "10"; gene_type
Dt_chr9	Cufflinks	exon	24994052	24994092	.	+	.	gene_id "XLOC_226412"; transcript_id "Novel_003159"; exon_number "11"; gene_type
Dt_chr9	Cufflinks	exon	24994212	24994296	.	+	.	gene_id "XLOC_226412"; transcript_id "Novel_003159"; exon_number "12"; gene_type
Dt_chr9	Cufflinks	exon	24994568	24994630	.	+	.	gene_id "XLOC_226412"; transcript_id "Novel_003159"; exon_number "13"; gene_type
Dt_chr9	Cufflinks	exon	24994765	24994823	.	+	.	gene_id "XLOC_226412"; transcript_id "Novel_003159"; exon_number "14"; gene_type
Dt_chr9	Cufflinks	exon	24995025	24995077	.	+	.	gene_id "XLOC_226412"; transcript_id "Novel_003159"; exon_number "15"; gene_type

Dt_chr9	Cufflinks	exon	24995295	24995367	.	+	.	gene_id "XLOC_226412"; transcript_id "Novel_003159"; exon_number "16"; gene_type
Dt_chr9	Cufflinks	exon	24995763	24996607	.	+	.	gene_id "XLOC_226412"; transcript_id "Novel_003159"; exon_number "17"; gene_type
Dt_chr9	Cufflinks	exon	24985595	24986359	.	+	.	gene_id "XLOC_226412"; transcript_id "Novel_003160"; exon_number "1"; gene_type "
Dt_chr9	Cufflinks	exon	24986529	24986606	.	+	.	gene_id "XLOC_226412"; transcript_id "Novel_003160"; exon_number "2"; gene_type "
Dt_chr9	Cufflinks	exon	24986727	24986802	.	+	.	gene_id "XLOC_226412"; transcript_id "Novel_003160"; exon_number "3"; gene_type "
Dt_chr9	Cufflinks	exon	24986903	24986925	.	+	.	gene_id "XLOC_226412"; transcript_id "Novel_003160"; exon_number "4"; gene_type "
Dt_chr9	Cufflinks	exon	24987045	24987145	.	+	.	gene_id "XLOC_226412"; transcript_id "Novel_003160"; exon_number "5"; gene_type "
Dt_chr9	Cufflinks	exon	24987691	24987785	.	+	.	gene_id "XLOC_226412"; transcript_id "Novel_003160"; exon_number "6"; gene_type "
Dt_chr9	Cufflinks	exon	24987996	24988064	.	+	.	gene_id "XLOC_226412"; transcript_id "Novel_003160"; exon_number "7"; gene_type "
Dt_chr9	Cufflinks	exon	24993342	24993411	.	+	.	gene_id "XLOC_226412"; transcript_id "Novel_003160"; exon_number "8"; gene_type "
Dt_chr9	Cufflinks	exon	24993898	24993961	.	+	.	gene_id "XLOC_226412"; transcript_id "Novel_003160"; exon_number "9"; gene_type "
Dt_chr9	Cufflinks	exon	24994052	24994092	.	+	.	gene_id "XLOC_226412"; transcript_id "Novel_003160"; exon_number "10"; gene_type
Dt_chr9	Cufflinks	exon	24994212	24994296	.	+	.	gene_id "XLOC_226412"; transcript_id "Novel_003160"; exon_number "11"; gene_type
Dt_chr9	Cufflinks	exon	24994568	24994630	.	+	.	gene_id "XLOC_226412"; transcript_id "Novel_003160"; exon_number "12"; gene_type
Dt_chr9	Cufflinks	exon	24994765	24994823	.	+	.	gene_id "XLOC_226412"; transcript_id "Novel_003160"; exon_number "13"; gene_type
Dt_chr9	Cufflinks	exon	24995025	24995077	.	+	.	gene_id "XLOC_226412"; transcript_id "Novel_003160"; exon_number "14"; gene_type
Dt_chr9	Cufflinks	exon	24995295	24995367	.	+	.	gene_id "XLOC_226412"; transcript_id "Novel_003160"; exon_number "15"; gene_type
Dt_chr9	Cufflinks	exon	24995763	24996607	.	+	.	gene_id "XLOC_226412"; transcript_id "Novel_003160"; exon_number "16"; gene_type
Dt_chr9	Cufflinks	exon	25062838	25063033	.	+	.	gene_id "XLOC_226417"; transcript_id "Novel_003161"; exon_number "1"; gene_type "
Dt_chr9	Cufflinks	exon	25063113	25063189	.	+	.	gene_id "XLOC_226417"; transcript_id "Novel_003161"; exon_number "2"; gene_type "
Dt_chr9	Cufflinks	exon	25081072	25081463	.	+	.	gene_id "XLOC_226419"; transcript_id "Novel_003162"; exon_number "1"; gene_type "
Dt_chr9	Cufflinks	exon	25081670	25081754	.	+	.	gene_id "XLOC_226419"; transcript_id "Novel_003162"; exon_number "2"; gene_type "
Dt_chr9	Cufflinks	exon	25082252	25082547	.	+	.	gene_id "XLOC_226419"; transcript_id "Novel_003162"; exon_number "3"; gene_type "
Dt_chr9	Cufflinks	exon	25082862	25084217	.	+	.	gene_id "XLOC_226419"; transcript_id "Novel_003162"; exon_number "4"; gene_type "
Dt_chr9	Cufflinks	exon	25081072	25081463	.	+	.	gene_id "XLOC_226419"; transcript_id "Novel_003163"; exon_number "1"; gene_type "
Dt_chr9	Cufflinks	exon	25081670	25081754	.	+	.	gene_id "XLOC_226419"; transcript_id "Novel_003163"; exon_number "2"; gene_type "
Dt_chr9	Cufflinks	exon	25082252	25083247	.	+	.	gene_id "XLOC_226419"; transcript_id "Novel_003163"; exon_number "3"; gene_type "
Dt_chr9	Cufflinks	exon	25083515	25083641	.	+	.	gene_id "XLOC_226419"; transcript_id "Novel_003163"; exon_number "4"; gene_type "
Dt_chr9	Cufflinks	exon	25083755	25084217	.	+	.	gene_id "XLOC_226419"; transcript_id "Novel_003163"; exon_number "5"; gene_type "
Dt_chr9	Cufflinks	exon	25364029	25364131	.	+	.	gene_id "XLOC_226445"; transcript_id "Novel_003164"; exon_number "1"; gene_type "
Dt_chr9	Cufflinks	exon	25364522	25366770	.	+	.	gene_id "XLOC_226445"; transcript_id "Novel_003164"; exon_number "2"; gene_type "
Dt_chr9	Cufflinks	exon	25520024	25520135	.	+	.	gene_id "XLOC_226454"; transcript_id "Novel_003165"; exon_number "1"; gene_type "
Dt_chr9	Cufflinks	exon	25520218	25520883	.	+	.	gene_id "XLOC_226454"; transcript_id "Novel_003165"; exon_number "2"; gene_type "
Dt_chr9	Cufflinks	exon	25521938	25522058	.	+	.	gene_id "XLOC_226454"; transcript_id "Novel_003165"; exon_number "3"; gene_type "
Dt_chr9	Cufflinks	exon	25522150	25522408	.	+	.	gene_id "XLOC_226454"; transcript_id "Novel_003165"; exon_number "4"; gene_type "

Dt_chr9	Cufflinks	exon	25522503	25522549	.	+	.	gene_id "XLOC_226454"; transcript_id "Novel_003165"; exon_number "5"; gene_type "
Dt_chr9	Cufflinks	exon	25522496	25523081	.	+	.	gene_id "XLOC_226454"; transcript_id "Novel_003166"; exon_number "1"; gene_type "
Dt_chr9	Cufflinks	exon	25523191	25524454	.	+	.	gene_id "XLOC_226454"; transcript_id "Novel_003166"; exon_number "2"; gene_type "
Dt_chr9	Cufflinks	exon	25522671	25523324	.	+	.	gene_id "XLOC_226454"; transcript_id "Novel_003167"; exon_number "1"; gene_type "
Dt_chr9	Cufflinks	exon	25524018	25524488	.	+	.	gene_id "XLOC_226454"; transcript_id "Novel_003167"; exon_number "2"; gene_type "
Dt_chr9	Cufflinks	exon	28297982	28298014	.	+	.	gene_id "XLOC_226637"; transcript_id "Novel_003168"; exon_number "1"; gene_type "
Dt_chr9	Cufflinks	exon	28298103	28301234	.	+	.	gene_id "XLOC_226637"; transcript_id "Novel_003168"; exon_number "2"; gene_type "
Dt_chr9	Cufflinks	exon	30605427	30605721	.	+	.	gene_id "XLOC_226817"; transcript_id "Novel_003169"; exon_number "1"; gene_type "
Dt_chr9	Cufflinks	exon	30605814	30605903	.	+	.	gene_id "XLOC_226817"; transcript_id "Novel_003169"; exon_number "2"; gene_type "
Dt_chr9	Cufflinks	exon	30608055	30608344	.	+	.	gene_id "XLOC_226817"; transcript_id "Novel_003169"; exon_number "3"; gene_type "
Dt_chr9	Cufflinks	exon	31414806	31415060	.	+	.	gene_id "XLOC_226854"; transcript_id "Novel_003170"; exon_number "1"; gene_type "
Dt_chr9	Cufflinks	exon	31415593	31417766	.	+	.	gene_id "XLOC_226854"; transcript_id "Novel_003170"; exon_number "2"; gene_type "
Dt_chr9	Cufflinks	exon	36488703	36488743	.	+	.	gene_id "XLOC_227423"; transcript_id "Novel_003171"; exon_number "1"; gene_type "
Dt_chr9	Cufflinks	exon	36488835	36488883	.	+	.	gene_id "XLOC_227423"; transcript_id "Novel_003171"; exon_number "2"; gene_type "
Dt_chr9	Cufflinks	exon	36489570	36489654	.	+	.	gene_id "XLOC_227423"; transcript_id "Novel_003171"; exon_number "3"; gene_type "
Dt_chr9	Cufflinks	exon	36490175	36490231	.	+	.	gene_id "XLOC_227423"; transcript_id "Novel_003171"; exon_number "4"; gene_type "
Dt_chr9	Cufflinks	exon	36490465	36490765	.	+	.	gene_id "XLOC_227423"; transcript_id "Novel_003171"; exon_number "5"; gene_type "
Dt_chr9	Cufflinks	exon	37370638	37371629	.	+	.	gene_id "XLOC_227551"; transcript_id "Novel_003172"; exon_number "1"; gene_type "
Dt_chr9	Cufflinks	exon	37371707	37372288	.	+	.	gene_id "XLOC_227551"; transcript_id "Novel_003172"; exon_number "2"; gene_type "
Dt_chr9	Cufflinks	exon	39400013	39400484	.	+	.	gene_id "XLOC_227749"; transcript_id "Novel_003173"; exon_number "1"; gene_type "
Dt_chr9	Cufflinks	exon	39400732	39401613	.	+	.	gene_id "XLOC_227749"; transcript_id "Novel_003173"; exon_number "2"; gene_type "
Dt_chr9	Cufflinks	exon	41178912	41179000	.	+	.	gene_id "XLOC_227993"; transcript_id "Novel_003174"; exon_number "1"; gene_type "
Dt_chr9	Cufflinks	exon	41179455	41179568	.	+	.	gene_id "XLOC_227993"; transcript_id "Novel_003174"; exon_number "2"; gene_type "
Dt_chr9	Cufflinks	exon	41181210	41181818	.	+	.	gene_id "XLOC_227993"; transcript_id "Novel_003174"; exon_number "3"; gene_type "
Dt_chr9	Cufflinks	exon	41179345	41179568	.	+	.	gene_id "XLOC_227993"; transcript_id "Novel_003175"; exon_number "1"; gene_type "
Dt_chr9	Cufflinks	exon	41181210	41181818	.	+	.	gene_id "XLOC_227993"; transcript_id "Novel_003175"; exon_number "2"; gene_type "
Dt_chr9	Cufflinks	exon	41180260	41180511	.	+	.	gene_id "XLOC_227993"; transcript_id "Novel_003176"; exon_number "1"; gene_type "
Dt_chr9	Cufflinks	exon	41181210	41181724	.	+	.	gene_id "XLOC_227993"; transcript_id "Novel_003176"; exon_number "2"; gene_type "
Dt_chr9	Cufflinks	exon	41198220	41198767	.	+	.	gene_id "XLOC_228003"; transcript_id "Novel_003177"; exon_number "1"; gene_type "
Dt_chr9	Cufflinks	exon	41199594	41199980	.	+	.	gene_id "XLOC_228003"; transcript_id "Novel_003177"; exon_number "2"; gene_type "
Dt_chr9	Cufflinks	exon	41200058	41200619	.	+	.	gene_id "XLOC_228003"; transcript_id "Novel_003177"; exon_number "3"; gene_type "
Dt_chr9	Cufflinks	exon	41443957	41444086	.	+	.	gene_id "XLOC_228057"; transcript_id "Novel_003178"; exon_number "1"; gene_type "
Dt_chr9	Cufflinks	exon	41445187	41445270	.	+	.	gene_id "XLOC_228057"; transcript_id "Novel_003178"; exon_number "2"; gene_type "
Dt_chr9	Cufflinks	exon	41445823	41445896	.	+	.	gene_id "XLOC_228057"; transcript_id "Novel_003178"; exon_number "3"; gene_type "
Dt_chr9	Cufflinks	exon	41445975	41446824	.	+	.	gene_id "XLOC_228057"; transcript_id "Novel_003178"; exon_number "4"; gene_type "

Dt_chr9	Cufflinks	exon	41443990	41444086	.	+	.	gene_id "XLOC_228057"; transcript_id "Novel_003179"; exon_number "1"; gene_type "
Dt_chr9	Cufflinks	exon	41445178	41445270	.	+	.	gene_id "XLOC_228057"; transcript_id "Novel_003179"; exon_number "2"; gene_type "
Dt_chr9	Cufflinks	exon	41445823	41445896	.	+	.	gene_id "XLOC_228057"; transcript_id "Novel_003179"; exon_number "3"; gene_type "
Dt_chr9	Cufflinks	exon	41445975	41446824	.	+	.	gene_id "XLOC_228057"; transcript_id "Novel_003179"; exon_number "4"; gene_type "
Dt_chr9	Cufflinks	exon	41870534	41870630	.	+	.	gene_id "XLOC_228124"; transcript_id "Novel_003180"; exon_number "1"; gene_type "
Dt_chr9	Cufflinks	exon	41870730	41870803	.	+	.	gene_id "XLOC_228124"; transcript_id "Novel_003180"; exon_number "2"; gene_type "
Dt_chr9	Cufflinks	exon	41870896	41870943	.	+	.	gene_id "XLOC_228124"; transcript_id "Novel_003180"; exon_number "3"; gene_type "
Dt_chr9	Cufflinks	exon	41872085	41872306	.	+	.	gene_id "XLOC_228124"; transcript_id "Novel_003180"; exon_number "4"; gene_type "
Dt_chr9	Cufflinks	exon	41872610	41873490	.	+	.	gene_id "XLOC_228124"; transcript_id "Novel_003180"; exon_number "5"; gene_type "
Dt_chr9	Cufflinks	exon	41873567	41874867	.	+	.	gene_id "XLOC_228124"; transcript_id "Novel_003180"; exon_number "6"; gene_type "
Dt_chr9	Cufflinks	exon	44617528	44617793	.	+	.	gene_id "XLOC_228518"; transcript_id "Novel_003181"; exon_number "1"; gene_type "
Dt_chr9	Cufflinks	exon	44619058	44620316	.	+	.	gene_id "XLOC_228518"; transcript_id "Novel_003181"; exon_number "2"; gene_type "
Dt_chr9	Cufflinks	exon	46976087	46976264	.	+	.	gene_id "XLOC_228811"; transcript_id "Novel_003182"; exon_number "1"; gene_type "
Dt_chr9	Cufflinks	exon	46977115	46977199	.	+	.	gene_id "XLOC_228811"; transcript_id "Novel_003182"; exon_number "2"; gene_type "
Dt_chr9	Cufflinks	exon	46977280	46979206	.	+	.	gene_id "XLOC_228811"; transcript_id "Novel_003182"; exon_number "3"; gene_type "
Dt_chr9	Cufflinks	exon	47517559	47517806	.	+	.	gene_id "XLOC_228901"; transcript_id "Novel_003183"; exon_number "1"; gene_type "
Dt_chr9	Cufflinks	exon	47518471	47518564	.	+	.	gene_id "XLOC_228901"; transcript_id "Novel_003183"; exon_number "2"; gene_type "
Dt_chr9	Cufflinks	exon	47518709	47519238	.	+	.	gene_id "XLOC_228901"; transcript_id "Novel_003183"; exon_number "3"; gene_type "
Dt_chr9	Cufflinks	exon	47517560	47517806	.	+	.	gene_id "XLOC_228901"; transcript_id "Novel_003184"; exon_number "1"; gene_type "
Dt_chr9	Cufflinks	exon	47518471	47518564	.	+	.	gene_id "XLOC_228901"; transcript_id "Novel_003184"; exon_number "2"; gene_type "
Dt_chr9	Cufflinks	exon	47518709	47518885	.	+	.	gene_id "XLOC_228901"; transcript_id "Novel_003184"; exon_number "3"; gene_type "
Dt_chr9	Cufflinks	exon	47519239	47519932	.	+	.	gene_id "XLOC_228901"; transcript_id "Novel_003184"; exon_number "4"; gene_type "
Dt_chr9	Cufflinks	exon	47520468	47521613	.	+	.	gene_id "XLOC_228901"; transcript_id "Novel_003184"; exon_number "5"; gene_type "
Dt_chr9	Cufflinks	exon	47580156	47580311	.	+	.	gene_id "XLOC_228906"; transcript_id "Novel_003185"; exon_number "1"; gene_type "
Dt_chr9	Cufflinks	exon	47580690	47580944	.	+	.	gene_id "XLOC_228906"; transcript_id "Novel_003185"; exon_number "2"; gene_type "
Dt_chr9	Cufflinks	exon	47906945	47907461	.	+	.	gene_id "XLOC_228953"; transcript_id "Novel_003186"; exon_number "1"; gene_type "
Dt_chr9	Cufflinks	exon	47907553	47907635	.	+	.	gene_id "XLOC_228953"; transcript_id "Novel_003186"; exon_number "2"; gene_type "
Dt_chr9	Cufflinks	exon	47907730	47907929	.	+	.	gene_id "XLOC_228953"; transcript_id "Novel_003186"; exon_number "3"; gene_type "
Dt_chr9	Cufflinks	exon	47908204	47908851	.	+	.	gene_id "XLOC_228953"; transcript_id "Novel_003186"; exon_number "4"; gene_type "
Dt_chr9	Cufflinks	exon	47914744	47915138	.	+	.	gene_id "XLOC_228961"; transcript_id "Novel_003187"; exon_number "1"; gene_type "
Dt_chr9	Cufflinks	exon	47915231	47915295	.	+	.	gene_id "XLOC_228961"; transcript_id "Novel_003187"; exon_number "2"; gene_type "
Dt_chr9	Cufflinks	exon	47915413	47915503	.	+	.	gene_id "XLOC_228962"; transcript_id "Novel_003188"; exon_number "1"; gene_type "
Dt_chr9	Cufflinks	exon	47915883	47916040	.	+	.	gene_id "XLOC_228962"; transcript_id "Novel_003188"; exon_number "2"; gene_type "
Dt_chr9	Cufflinks	exon	47916120	47916155	.	+	.	gene_id "XLOC_228962"; transcript_id "Novel_003188"; exon_number "3"; gene_type "
Dt_chr9	Cufflinks	exon	47916349	47916436	.	+	.	gene_id "XLOC_228963"; transcript_id "Novel_003189"; exon_number "1"; gene_type "

Dt_chr9	Cufflinks	exon	47916529	47916809	.	+	.	gene_id "XLOC_228963"; transcript_id "Novel_003189"; exon_number "2"; gene_type "
Dt_chr9	Cufflinks	exon	47980414	47980527	.	+	.	gene_id "XLOC_228971"; transcript_id "Novel_003190"; exon_number "1"; gene_type "
Dt_chr9	Cufflinks	exon	47980664	47980735	.	+	.	gene_id "XLOC_228971"; transcript_id "Novel_003190"; exon_number "2"; gene_type "
Dt_chr9	Cufflinks	exon	47980840	47980916	.	+	.	gene_id "XLOC_228971"; transcript_id "Novel_003190"; exon_number "3"; gene_type "
Dt_chr9	Cufflinks	exon	47980989	47981079	.	+	.	gene_id "XLOC_228971"; transcript_id "Novel_003190"; exon_number "4"; gene_type "
Dt_chr9	Cufflinks	exon	47981184	47981471	.	+	.	gene_id "XLOC_228971"; transcript_id "Novel_003190"; exon_number "5"; gene_type "
Dt_chr9	Cufflinks	exon	48042479	48042905	.	+	.	gene_id "XLOC_228984"; transcript_id "Novel_003191"; exon_number "1"; gene_type "
Dt_chr9	Cufflinks	exon	48043064	48043311	.	+	.	gene_id "XLOC_228984"; transcript_id "Novel_003191"; exon_number "2"; gene_type "
Dt_chr9	Cufflinks	exon	48042606	48042905	.	+	.	gene_id "XLOC_228984"; transcript_id "Novel_003192"; exon_number "1"; gene_type "
Dt_chr9	Cufflinks	exon	48043064	48043138	.	+	.	gene_id "XLOC_228984"; transcript_id "Novel_003192"; exon_number "2"; gene_type "
Dt_chr9	Cufflinks	exon	48043471	48043572	.	+	.	gene_id "XLOC_228984"; transcript_id "Novel_003192"; exon_number "3"; gene_type "
Dt_chr9	Cufflinks	exon	48043833	48043886	.	+	.	gene_id "XLOC_228984"; transcript_id "Novel_003192"; exon_number "4"; gene_type "
Dt_chr9	Cufflinks	exon	48043965	48044057	.	+	.	gene_id "XLOC_228984"; transcript_id "Novel_003192"; exon_number "5"; gene_type "
Dt_chr9	Cufflinks	exon	48044137	48044229	.	+	.	gene_id "XLOC_228984"; transcript_id "Novel_003192"; exon_number "6"; gene_type "
Dt_chr9	Cufflinks	exon	48044308	48044412	.	+	.	gene_id "XLOC_228984"; transcript_id "Novel_003192"; exon_number "7"; gene_type "
Dt_chr9	Cufflinks	exon	48044528	48045523	.	+	.	gene_id "XLOC_228984"; transcript_id "Novel_003192"; exon_number "8"; gene_type "
Dt_chr9	Cufflinks	exon	48042610	48042905	.	+	.	gene_id "XLOC_228984"; transcript_id "Novel_003193"; exon_number "1"; gene_type "
Dt_chr9	Cufflinks	exon	48043064	48043138	.	+	.	gene_id "XLOC_228984"; transcript_id "Novel_003193"; exon_number "2"; gene_type "
Dt_chr9	Cufflinks	exon	48043471	48043572	.	+	.	gene_id "XLOC_228984"; transcript_id "Novel_003193"; exon_number "3"; gene_type "
Dt_chr9	Cufflinks	exon	48043833	48043886	.	+	.	gene_id "XLOC_228984"; transcript_id "Novel_003193"; exon_number "4"; gene_type "
Dt_chr9	Cufflinks	exon	48043965	48044057	.	+	.	gene_id "XLOC_228984"; transcript_id "Novel_003193"; exon_number "5"; gene_type "
Dt_chr9	Cufflinks	exon	48044137	48044387	.	+	.	gene_id "XLOC_228984"; transcript_id "Novel_003193"; exon_number "6"; gene_type "
Dt_chr9	Cufflinks	exon	48111430	48112134	.	+	.	gene_id "XLOC_228997"; transcript_id "Novel_003194"; exon_number "1"; gene_type "
Dt_chr9	Cufflinks	exon	48112235	48113209	.	+	.	gene_id "XLOC_228997"; transcript_id "Novel_003194"; exon_number "2"; gene_type "
Dt_chr9	Cufflinks	exon	48538494	48538757	.	+	.	gene_id "XLOC_229075"; transcript_id "Novel_003195"; exon_number "1"; gene_type "
Dt_chr9	Cufflinks	exon	48538850	48540128	.	+	.	gene_id "XLOC_229075"; transcript_id "Novel_003195"; exon_number "2"; gene_type "
Dt_chr9	Cufflinks	exon	49854131	49854988	.	+	.	gene_id "XLOC_229251"; transcript_id "Novel_003196"; exon_number "1"; gene_type "
Dt_chr9	Cufflinks	exon	51178390	51178600	.	+	.	gene_id "XLOC_229465"; transcript_id "Novel_003197"; exon_number "1"; gene_type "
Dt_chr9	Cufflinks	exon	51178737	51178920	.	+	.	gene_id "XLOC_229465"; transcript_id "Novel_003197"; exon_number "2"; gene_type "
Dt_chr9	Cufflinks	exon	51180264	51180790	.	+	.	gene_id "XLOC_229465"; transcript_id "Novel_003197"; exon_number "3"; gene_type "
Dt_chr9	Cufflinks	exon	51178664	51178920	.	+	.	gene_id "XLOC_229465"; transcript_id "Novel_003198"; exon_number "1"; gene_type "
Dt_chr9	Cufflinks	exon	51180264	51180790	.	+	.	gene_id "XLOC_229465"; transcript_id "Novel_003198"; exon_number "2"; gene_type "
Dt_chr9	Cufflinks	exon	51238218	51240019	.	+	.	gene_id "XLOC_229473"; transcript_id "Novel_003199"; exon_number "1"; gene_type "
Dt_chr9	Cufflinks	exon	51240226	51243329	.	+	.	gene_id "XLOC_229473"; transcript_id "Novel_003199"; exon_number "2"; gene_type "
Dt_chr9	Cufflinks	exon	51995686	51996008	.	+	.	gene_id "XLOC_229565"; transcript_id "Novel_003200"; exon_number "1"; gene_type "

Dt_chr9	Cufflinks	exon	51996102	51996193	.	+	.	gene_id "XLOC_229565"; transcript_id "Novel_003200"; exon_number "2"; gene_type "
Dt_chr9	Cufflinks	exon	51996302	51996964	.	+	.	gene_id "XLOC_229565"; transcript_id "Novel_003200"; exon_number "3"; gene_type "
Dt_chr9	Cufflinks	exon	52332567	52333148	.	+	.	gene_id "XLOC_229614"; transcript_id "Novel_003201"; exon_number "1"; gene_type "
Dt_chr9	Cufflinks	exon	52333255	52333395	.	+	.	gene_id "XLOC_229614"; transcript_id "Novel_003201"; exon_number "2"; gene_type "
Dt_chr9	Cufflinks	exon	52333518	52333637	.	+	.	gene_id "XLOC_229614"; transcript_id "Novel_003201"; exon_number "3"; gene_type "
Dt_chr9	Cufflinks	exon	52333787	52333882	.	+	.	gene_id "XLOC_229614"; transcript_id "Novel_003201"; exon_number "4"; gene_type "
Dt_chr9	Cufflinks	exon	52333965	52334508	.	+	.	gene_id "XLOC_229614"; transcript_id "Novel_003201"; exon_number "5"; gene_type "
Dt_chr9	Cufflinks	exon	52332567	52333637	.	+	.	gene_id "XLOC_229614"; transcript_id "Novel_003202"; exon_number "1"; gene_type "
Dt_chr9	Cufflinks	exon	52333787	52333882	.	+	.	gene_id "XLOC_229614"; transcript_id "Novel_003202"; exon_number "2"; gene_type "
Dt_chr9	Cufflinks	exon	52333965	52334508	.	+	.	gene_id "XLOC_229614"; transcript_id "Novel_003202"; exon_number "3"; gene_type "
Dt_chr9	Cufflinks	exon	52332657	52333148	.	+	.	gene_id "XLOC_229614"; transcript_id "Novel_003203"; exon_number "1"; gene_type "
Dt_chr9	Cufflinks	exon	52333343	52333395	.	+	.	gene_id "XLOC_229614"; transcript_id "Novel_003203"; exon_number "2"; gene_type "
Dt_chr9	Cufflinks	exon	52333518	52333637	.	+	.	gene_id "XLOC_229614"; transcript_id "Novel_003203"; exon_number "3"; gene_type "
Dt_chr9	Cufflinks	exon	52333787	52333882	.	+	.	gene_id "XLOC_229614"; transcript_id "Novel_003203"; exon_number "4"; gene_type "
Dt_chr9	Cufflinks	exon	52333965	52334368	.	+	.	gene_id "XLOC_229614"; transcript_id "Novel_003203"; exon_number "5"; gene_type "
Dt_chr9	Cufflinks	exon	52762064	52762345	.	+	.	gene_id "XLOC_229657"; transcript_id "Novel_003204"; exon_number "1"; gene_type "
Dt_chr9	Cufflinks	exon	52762858	52762955	.	+	.	gene_id "XLOC_229657"; transcript_id "Novel_003204"; exon_number "2"; gene_type "
Dt_chr9	Cufflinks	exon	52763051	52763081	.	+	.	gene_id "XLOC_229657"; transcript_id "Novel_003204"; exon_number "3"; gene_type "
Dt_chr9	Cufflinks	exon	52763320	52763419	.	+	.	gene_id "XLOC_229657"; transcript_id "Novel_003204"; exon_number "4"; gene_type "
Dt_chr9	Cufflinks	exon	52763547	52763617	.	+	.	gene_id "XLOC_229657"; transcript_id "Novel_003204"; exon_number "5"; gene_type "
Dt_chr9	Cufflinks	exon	52763749	52763892	.	+	.	gene_id "XLOC_229657"; transcript_id "Novel_003204"; exon_number "6"; gene_type "
Dt_chr9	Cufflinks	exon	52763989	52764738	.	+	.	gene_id "XLOC_229657"; transcript_id "Novel_003204"; exon_number "7"; gene_type "
Dt_chr9	Cufflinks	exon	52935561	52935753	.	+	.	gene_id "XLOC_229690"; transcript_id "Novel_003205"; exon_number "1"; gene_type "
Dt_chr9	Cufflinks	exon	52936034	52937526	.	+	.	gene_id "XLOC_229690"; transcript_id "Novel_003205"; exon_number "2"; gene_type "
Dt_chr9	Cufflinks	exon	53926943	53927357	.	+	.	gene_id "XLOC_229909"; transcript_id "Novel_003206"; exon_number "1"; gene_type "
Dt_chr9	Cufflinks	exon	53927693	53928625	.	+	.	gene_id "XLOC_229909"; transcript_id "Novel_003206"; exon_number "2"; gene_type "
Dt_chr9	Cufflinks	exon	55836478	55837171	.	+	.	gene_id "XLOC_230276"; transcript_id "Novel_003207"; exon_number "1"; gene_type "
Dt_chr9	Cufflinks	exon	56111194	56111435	.	+	.	gene_id "XLOC_230311"; transcript_id "Novel_003208"; exon_number "1"; gene_type "
Dt_chr9	Cufflinks	exon	56111783	56111899	.	+	.	gene_id "XLOC_230311"; transcript_id "Novel_003208"; exon_number "2"; gene_type "
Dt_chr9	Cufflinks	exon	56112167	56112205	.	+	.	gene_id "XLOC_230311"; transcript_id "Novel_003208"; exon_number "3"; gene_type "
Dt_chr9	Cufflinks	exon	56112801	56112903	.	+	.	gene_id "XLOC_230311"; transcript_id "Novel_003209"; exon_number "1"; gene_type "
Dt_chr9	Cufflinks	exon	56113001	56113297	.	+	.	gene_id "XLOC_230311"; transcript_id "Novel_003209"; exon_number "2"; gene_type "
Dt_chr9	Cufflinks	exon	56267914	56268180	.	+	.	gene_id "XLOC_230344"; transcript_id "Novel_003210"; exon_number "1"; gene_type "
Dt_chr9	Cufflinks	exon	56268959	56271281	.	+	.	gene_id "XLOC_230344"; transcript_id "Novel_003210"; exon_number "2"; gene_type "
Dt_chr9	Cufflinks	exon	56267943	56269519	.	+	.	gene_id "XLOC_230344"; transcript_id "Novel_003211"; exon_number "1"; gene_type "

Dt_chr9	Cufflinks	exon	56269947	56271328	.	+	.	gene_id "XLOC_230344"; transcript_id "Novel_003211"; exon_number "2"; gene_type "
Dt_chr9	Cufflinks	exon	56267943	56268173	.	+	.	gene_id "XLOC_230344"; transcript_id "Novel_003212"; exon_number "1"; gene_type "
Dt_chr9	Cufflinks	exon	56268959	56269519	.	+	.	gene_id "XLOC_230344"; transcript_id "Novel_003212"; exon_number "2"; gene_type "
Dt_chr9	Cufflinks	exon	56269947	56271328	.	+	.	gene_id "XLOC_230344"; transcript_id "Novel_003212"; exon_number "3"; gene_type "
Dt_chr9	Cufflinks	exon	56267943	56268180	.	+	.	gene_id "XLOC_230344"; transcript_id "Novel_003213"; exon_number "1"; gene_type "
Dt_chr9	Cufflinks	exon	56268959	56269519	.	+	.	gene_id "XLOC_230344"; transcript_id "Novel_003213"; exon_number "2"; gene_type "
Dt_chr9	Cufflinks	exon	56269947	56271328	.	+	.	gene_id "XLOC_230344"; transcript_id "Novel_003213"; exon_number "3"; gene_type "
Dt_chr9	Cufflinks	exon	56536451	56536847	.	+	.	gene_id "XLOC_230392"; transcript_id "Novel_003214"; exon_number "1"; gene_type "
Dt_chr9	Cufflinks	exon	56537020	56537315	.	+	.	gene_id "XLOC_230392"; transcript_id "Novel_003214"; exon_number "2"; gene_type "
Dt_chr9	Cufflinks	exon	56537498	56538091	.	+	.	gene_id "XLOC_230392"; transcript_id "Novel_003214"; exon_number "3"; gene_type "
Dt_chr9	Cufflinks	exon	56889261	56889402	.	+	.	gene_id "XLOC_230424"; transcript_id "Novel_003215"; exon_number "1"; gene_type "
Dt_chr9	Cufflinks	exon	56890190	56890281	.	+	.	gene_id "XLOC_230424"; transcript_id "Novel_003215"; exon_number "2"; gene_type "
Dt_chr9	Cufflinks	exon	56890946	56891977	.	+	.	gene_id "XLOC_230424"; transcript_id "Novel_003215"; exon_number "3"; gene_type "
Dt_chr9	Cufflinks	exon	56889294	56889402	.	+	.	gene_id "XLOC_230424"; transcript_id "Novel_003216"; exon_number "1"; gene_type "
Dt_chr9	Cufflinks	exon	56890190	56890456	.	+	.	gene_id "XLOC_230424"; transcript_id "Novel_003216"; exon_number "2"; gene_type "
Dt_chr9	Cufflinks	exon	56890946	56891731	.	+	.	gene_id "XLOC_230424"; transcript_id "Novel_003216"; exon_number "3"; gene_type "
Dt_chr9	Cufflinks	exon	57155514	57155548	.	+	.	gene_id "XLOC_230458"; transcript_id "Novel_003217"; exon_number "1"; gene_type "
Dt_chr9	Cufflinks	exon	57155924	57156025	.	+	.	gene_id "XLOC_230458"; transcript_id "Novel_003217"; exon_number "2"; gene_type "
Dt_chr9	Cufflinks	exon	57156140	57156273	.	+	.	gene_id "XLOC_230458"; transcript_id "Novel_003217"; exon_number "3"; gene_type "
Dt_chr9	Cufflinks	exon	57156855	57157168	.	+	.	gene_id "XLOC_230458"; transcript_id "Novel_003217"; exon_number "4"; gene_type "
Dt_chr9	Cufflinks	exon	57768910	57769280	.	+	.	gene_id "XLOC_230586"; transcript_id "Novel_003218"; exon_number "1"; gene_type "
Dt_chr9	Cufflinks	exon	57769368	57769900	.	+	.	gene_id "XLOC_230586"; transcript_id "Novel_003218"; exon_number "2"; gene_type "
Dt_chr9	Cufflinks	exon	57825940	57826272	.	+	.	gene_id "XLOC_230590"; transcript_id "Novel_003219"; exon_number "1"; gene_type "
Dt_chr9	Cufflinks	exon	57827468	57827669	.	+	.	gene_id "XLOC_230590"; transcript_id "Novel_003219"; exon_number "2"; gene_type "
Dt_chr9	Cufflinks	exon	57827911	57828020	.	+	.	gene_id "XLOC_230590"; transcript_id "Novel_003219"; exon_number "3"; gene_type "
Dt_chr9	Cufflinks	exon	57828107	57828181	.	+	.	gene_id "XLOC_230590"; transcript_id "Novel_003219"; exon_number "4"; gene_type "
Dt_chr9	Cufflinks	exon	57829083	57829253	.	+	.	gene_id "XLOC_230590"; transcript_id "Novel_003219"; exon_number "5"; gene_type "
Dt_chr9	Cufflinks	exon	57829334	57829924	.	+	.	gene_id "XLOC_230590"; transcript_id "Novel_003219"; exon_number "6"; gene_type "
Dt_chr9	Cufflinks	exon	57826374	57828020	.	+	.	gene_id "XLOC_230590"; transcript_id "Novel_003220"; exon_number "1"; gene_type "
Dt_chr9	Cufflinks	exon	57828107	57828181	.	+	.	gene_id "XLOC_230590"; transcript_id "Novel_003220"; exon_number "2"; gene_type "
Dt_chr9	Cufflinks	exon	57829083	57829253	.	+	.	gene_id "XLOC_230590"; transcript_id "Novel_003220"; exon_number "3"; gene_type "
Dt_chr9	Cufflinks	exon	57829334	57830159	.	+	.	gene_id "XLOC_230590"; transcript_id "Novel_003220"; exon_number "4"; gene_type "
Dt_chr9	Cufflinks	exon	57828687	57829253	.	+	.	gene_id "XLOC_230590"; transcript_id "Novel_003221"; exon_number "1"; gene_type "
Dt_chr9	Cufflinks	exon	57829334	57829870	.	+	.	gene_id "XLOC_230590"; transcript_id "Novel_003221"; exon_number "2"; gene_type "
Dt_chr9	Cufflinks	exon	57895480	57895681	.	+	.	gene_id "XLOC_230605"; transcript_id "Novel_003222"; exon_number "1"; gene_type "

Dt_chr9	Cufflinks	exon	59229190	59229231	.	+	.	gene_id "XLOC_230844"; transcript_id "Novel_003223"; exon_number "1"; gene_type "
Dt_chr9	Cufflinks	exon	59229780	59229959	.	+	.	gene_id "XLOC_230844"; transcript_id "Novel_003223"; exon_number "2"; gene_type "
Dt_chr9	Cufflinks	exon	59230093	59230140	.	+	.	gene_id "XLOC_230844"; transcript_id "Novel_003223"; exon_number "3"; gene_type "
Dt_chr9	Cufflinks	exon	59230229	59230291	.	+	.	gene_id "XLOC_230844"; transcript_id "Novel_003223"; exon_number "4"; gene_type "
Dt_chr9	Cufflinks	exon	59230541	59230594	.	+	.	gene_id "XLOC_230844"; transcript_id "Novel_003223"; exon_number "5"; gene_type "
Dt_chr9	Cufflinks	exon	59230698	59230784	.	+	.	gene_id "XLOC_230844"; transcript_id "Novel_003223"; exon_number "6"; gene_type "
Dt_chr9	Cufflinks	exon	59230904	59231522	.	+	.	gene_id "XLOC_230844"; transcript_id "Novel_003223"; exon_number "7"; gene_type "
Dt_chr9	Cufflinks	exon	59750136	59751285	.	+	.	gene_id "XLOC_230933"; transcript_id "Novel_003224"; exon_number "1"; gene_type "
Dt_chr9	Cufflinks	exon	59752015	59752312	.	+	.	gene_id "XLOC_230933"; transcript_id "Novel_003224"; exon_number "2"; gene_type "
Dt_chr9	Cufflinks	exon	60040501	60041169	.	+	.	gene_id "XLOC_230994"; transcript_id "Novel_003225"; exon_number "1"; gene_type "
Dt_chr9	Cufflinks	exon	60041738	60042007	.	+	.	gene_id "XLOC_230994"; transcript_id "Novel_003225"; exon_number "2"; gene_type "
Dt_chr9	Cufflinks	exon	60042195	60043086	.	+	.	gene_id "XLOC_230994"; transcript_id "Novel_003225"; exon_number "3"; gene_type "
Dt_chr9	Cufflinks	exon	60555508	60555618	.	+	.	gene_id "XLOC_231097"; transcript_id "Novel_003226"; exon_number "1"; gene_type "
Dt_chr9	Cufflinks	exon	60556217	60558144	.	+	.	gene_id "XLOC_231097"; transcript_id "Novel_003226"; exon_number "2"; gene_type "
Dt_chr9	Cufflinks	exon	61087712	61088180	.	+	.	gene_id "XLOC_231209"; transcript_id "Novel_003227"; exon_number "1"; gene_type "
Dt_chr9	Cufflinks	exon	61088369	61088415	.	+	.	gene_id "XLOC_231209"; transcript_id "Novel_003227"; exon_number "2"; gene_type "
Dt_chr9	Cufflinks	exon	61088972	61089093	.	+	.	gene_id "XLOC_231209"; transcript_id "Novel_003227"; exon_number "3"; gene_type "
Dt_chr9	Cufflinks	exon	61089175	61089286	.	+	.	gene_id "XLOC_231209"; transcript_id "Novel_003227"; exon_number "4"; gene_type "
Dt_chr9	Cufflinks	exon	61089373	61089478	.	+	.	gene_id "XLOC_231209"; transcript_id "Novel_003227"; exon_number "5"; gene_type "
Dt_chr9	Cufflinks	exon	61089763	61089954	.	+	.	gene_id "XLOC_231209"; transcript_id "Novel_003227"; exon_number "6"; gene_type "
Dt_chr9	Cufflinks	exon	61090122	61091264	.	+	.	gene_id "XLOC_231209"; transcript_id "Novel_003227"; exon_number "7"; gene_type "
Dt_chr9	Cufflinks	exon	61089681	61089954	.	+	.	gene_id "XLOC_231209"; transcript_id "Novel_003228"; exon_number "1"; gene_type "
Dt_chr9	Cufflinks	exon	61090122	61090420	.	+	.	gene_id "XLOC_231209"; transcript_id "Novel_003228"; exon_number "2"; gene_type "
Dt_chr9	Cufflinks	exon	61180240	61180347	.	+	.	gene_id "XLOC_231230"; transcript_id "Novel_003229"; exon_number "1"; gene_type "
Dt_chr9	Cufflinks	exon	61181062	61183437	.	+	.	gene_id "XLOC_231230"; transcript_id "Novel_003229"; exon_number "2"; gene_type "
Dt_chr9	Cufflinks	exon	61180505	61180720	.	+	.	gene_id "XLOC_231230"; transcript_id "Novel_003230"; exon_number "1"; gene_type "
Dt_chr9	Cufflinks	exon	61181062	61183217	.	+	.	gene_id "XLOC_231230"; transcript_id "Novel_003230"; exon_number "2"; gene_type "
Dt_chr9	Cufflinks	exon	62030128	62030642	.	+	.	gene_id "XLOC_231345"; transcript_id "Novel_003231"; exon_number "1"; gene_type "
Dt_chr9	Cufflinks	exon	62031300	62031791	.	+	.	gene_id "XLOC_231345"; transcript_id "Novel_003231"; exon_number "2"; gene_type "
Dt_chr9	Cufflinks	exon	63586392	63586664	.	+	.	gene_id "XLOC_231672"; transcript_id "Novel_003232"; exon_number "1"; gene_type "
Dt_chr9	Cufflinks	exon	63586960	63589163	.	+	.	gene_id "XLOC_231672"; transcript_id "Novel_003232"; exon_number "2"; gene_type "
Dt_chr9	Cufflinks	exon	63589272	63589328	.	+	.	gene_id "XLOC_231672"; transcript_id "Novel_003232"; exon_number "3"; gene_type "
Dt_chr9	Cufflinks	exon	63590044	63590927	.	+	.	gene_id "XLOC_231672"; transcript_id "Novel_003232"; exon_number "4"; gene_type "
Dt_chr9	Cufflinks	exon	63586392	63586664	.	+	.	gene_id "XLOC_231672"; transcript_id "Novel_003233"; exon_number "1"; gene_type "
Dt_chr9	Cufflinks	exon	63586960	63589163	.	+	.	gene_id "XLOC_231672"; transcript_id "Novel_003233"; exon_number "2"; gene_type "

Dt_chr9	Cufflinks	exon	63589275	63589328	.	+	.	gene_id "XLOC_231672"; transcript_id "Novel_003233"; exon_number "3"; gene_type "
Dt_chr9	Cufflinks	exon	63590044	63590927	.	+	.	gene_id "XLOC_231672"; transcript_id "Novel_003233"; exon_number "4"; gene_type "
Dt_chr9	Cufflinks	exon	63766713	63768036	.	+	.	gene_id "XLOC_231698"; transcript_id "Novel_003234"; exon_number "1"; gene_type "
Dt_chr9	Cufflinks	exon	63768491	63768642	.	+	.	gene_id "XLOC_231698"; transcript_id "Novel_003234"; exon_number "2"; gene_type "
Dt_chr9	Cufflinks	exon	63768998	63769505	.	+	.	gene_id "XLOC_231698"; transcript_id "Novel_003234"; exon_number "3"; gene_type "
Dt_chr9	Cufflinks	exon	64132469	64132968	.	+	.	gene_id "XLOC_231771"; transcript_id "Novel_003235"; exon_number "1"; gene_type "
Dt_chr9	Cufflinks	exon	64133092	64135129	.	+	.	gene_id "XLOC_231771"; transcript_id "Novel_003235"; exon_number "2"; gene_type "
Dt_chr9	Cufflinks	exon	64132488	64132645	.	+	.	gene_id "XLOC_231771"; transcript_id "Novel_003236"; exon_number "1"; gene_type "
Dt_chr9	Cufflinks	exon	64132776	64134350	.	+	.	gene_id "XLOC_231771"; transcript_id "Novel_003236"; exon_number "2"; gene_type "
Dt_chr9	Cufflinks	exon	64132488	64132527	.	+	.	gene_id "XLOC_231771"; transcript_id "Novel_003237"; exon_number "1"; gene_type "
Dt_chr9	Cufflinks	exon	64133092	64134357	.	+	.	gene_id "XLOC_231771"; transcript_id "Novel_003237"; exon_number "2"; gene_type "
Dt_chr9	Cufflinks	exon	64604474	64605398	.	+	.	gene_id "XLOC_231862"; transcript_id "Novel_003238"; exon_number "1"; gene_type "
Dt_chr9	Cufflinks	exon	64605472	64606358	.	+	.	gene_id "XLOC_231862"; transcript_id "Novel_003238"; exon_number "2"; gene_type "
Dt_chr9	Cufflinks	exon	65359050	65359090	.	+	.	gene_id "XLOC_231993"; transcript_id "Novel_003239"; exon_number "1"; gene_type "
Dt_chr9	Cufflinks	exon	65359186	65359565	.	+	.	gene_id "XLOC_231993"; transcript_id "Novel_003239"; exon_number "2"; gene_type "
Dt_chr9	Cufflinks	exon	65359669	65360664	.	+	.	gene_id "XLOC_231993"; transcript_id "Novel_003239"; exon_number "3"; gene_type "
Dt_chr9	Cufflinks	exon	65916185	65916415	.	+	.	gene_id "XLOC_232095"; transcript_id "Novel_003240"; exon_number "1"; gene_type "
Dt_chr9	Cufflinks	exon	65916892	65917980	.	+	.	gene_id "XLOC_232095"; transcript_id "Novel_003240"; exon_number "2"; gene_type "
Dt_chr9	Cufflinks	exon	65918351	65919120	.	+	.	gene_id "XLOC_232095"; transcript_id "Novel_003240"; exon_number "3"; gene_type "
Dt_chr9	Cufflinks	exon	66490853	66491200	.	+	.	gene_id "XLOC_232209"; transcript_id "Novel_003241"; exon_number "1"; gene_type "
Dt_chr9	Cufflinks	exon	66491984	66492057	.	+	.	gene_id "XLOC_232209"; transcript_id "Novel_003241"; exon_number "2"; gene_type "
Dt_chr9	Cufflinks	exon	66492159	66492423	.	+	.	gene_id "XLOC_232209"; transcript_id "Novel_003241"; exon_number "3"; gene_type "
Dt_chr9	Cufflinks	exon	66492790	66492921	.	+	.	gene_id "XLOC_232209"; transcript_id "Novel_003241"; exon_number "4"; gene_type "
Dt_chr9	Cufflinks	exon	66493081	66493216	.	+	.	gene_id "XLOC_232209"; transcript_id "Novel_003241"; exon_number "5"; gene_type "
Dt_chr9	Cufflinks	exon	66493294	66493785	.	+	.	gene_id "XLOC_232209"; transcript_id "Novel_003241"; exon_number "6"; gene_type "
Dt_chr9	Cufflinks	exon	67906471	67906704	.	+	.	gene_id "XLOC_232482"; transcript_id "Novel_003242"; exon_number "1"; gene_type "
Dt_chr9	Cufflinks	exon	67907293	67908738	.	+	.	gene_id "XLOC_232482"; transcript_id "Novel_003242"; exon_number "2"; gene_type "
Dt_chr9	Cufflinks	exon	69275022	69275249	.	+	.	gene_id "XLOC_232694"; transcript_id "Novel_003243"; exon_number "1"; gene_type "
Dt_chr9	Cufflinks	exon	69275392	69275446	.	+	.	gene_id "XLOC_232694"; transcript_id "Novel_003243"; exon_number "2"; gene_type "
Dt_chr9	Cufflinks	exon	69275533	69275655	.	+	.	gene_id "XLOC_232694"; transcript_id "Novel_003243"; exon_number "3"; gene_type "
Dt_chr9	Cufflinks	exon	69275972	69276028	.	+	.	gene_id "XLOC_232694"; transcript_id "Novel_003243"; exon_number "4"; gene_type "
Dt_chr9	Cufflinks	exon	69276230	69276624	.	+	.	gene_id "XLOC_232694"; transcript_id "Novel_003243"; exon_number "5"; gene_type "
Dt_chr9	Cufflinks	exon	69674751	69674889	.	+	.	gene_id "XLOC_232767"; transcript_id "Novel_003244"; exon_number "1"; gene_type "
Dt_chr9	Cufflinks	exon	69674998	69675224	.	+	.	gene_id "XLOC_232767"; transcript_id "Novel_003244"; exon_number "2"; gene_type "
Dt_chr9	Cufflinks	exon	1339512	1339709	.	-	.	gene_id "XLOC_233158"; transcript_id "Novel_003245"; exon_number "1"; gene_type "

Dt_chr9	Cufflinks	exon	1339814	1339917	.	-	.	gene_id "XLOC_233158"; transcript_id "Novel_003245"; exon_number "2"; gene_type "
Dt_chr9	Cufflinks	exon	1340199	1340303	.	-	.	gene_id "XLOC_233158"; transcript_id "Novel_003245"; exon_number "3"; gene_type "
Dt_chr9	Cufflinks	exon	2235215	2235866	.	-	.	gene_id "XLOC_233247"; transcript_id "Novel_003246"; exon_number "1"; gene_type "
Dt_chr9	Cufflinks	exon	2236814	2236985	.	-	.	gene_id "XLOC_233247"; transcript_id "Novel_003246"; exon_number "2"; gene_type "
Dt_chr9	Cufflinks	exon	3124094	3124553	.	-	.	gene_id "XLOC_233321"; transcript_id "Novel_003247"; exon_number "1"; gene_type "
Dt_chr9	Cufflinks	exon	3125752	3125852	.	-	.	gene_id "XLOC_233321"; transcript_id "Novel_003247"; exon_number "2"; gene_type "
Dt_chr9	Cufflinks	exon	3126494	3126545	.	-	.	gene_id "XLOC_233321"; transcript_id "Novel_003247"; exon_number "3"; gene_type "
Dt_chr9	Cufflinks	exon	3126680	3127560	.	-	.	gene_id "XLOC_233321"; transcript_id "Novel_003247"; exon_number "4"; gene_type "
Dt_chr9	Cufflinks	exon	3124111	3124553	.	-	.	gene_id "XLOC_233321"; transcript_id "Novel_003248"; exon_number "1"; gene_type "
Dt_chr9	Cufflinks	exon	3125752	3125852	.	-	.	gene_id "XLOC_233321"; transcript_id "Novel_003248"; exon_number "2"; gene_type "
Dt_chr9	Cufflinks	exon	3126494	3126545	.	-	.	gene_id "XLOC_233321"; transcript_id "Novel_003248"; exon_number "3"; gene_type "
Dt_chr9	Cufflinks	exon	3126680	3126815	.	-	.	gene_id "XLOC_233321"; transcript_id "Novel_003248"; exon_number "4"; gene_type "
Dt_chr9	Cufflinks	exon	3127052	3127611	.	-	.	gene_id "XLOC_233321"; transcript_id "Novel_003248"; exon_number "5"; gene_type "
Dt_chr9	Cufflinks	exon	3124236	3124553	.	-	.	gene_id "XLOC_233321"; transcript_id "Novel_003249"; exon_number "1"; gene_type "
Dt_chr9	Cufflinks	exon	3125752	3125852	.	-	.	gene_id "XLOC_233321"; transcript_id "Novel_003249"; exon_number "2"; gene_type "
Dt_chr9	Cufflinks	exon	3126494	3126545	.	-	.	gene_id "XLOC_233321"; transcript_id "Novel_003249"; exon_number "3"; gene_type "
Dt_chr9	Cufflinks	exon	3126680	3126815	.	-	.	gene_id "XLOC_233321"; transcript_id "Novel_003249"; exon_number "4"; gene_type "
Dt_chr9	Cufflinks	exon	3127052	3127170	.	-	.	gene_id "XLOC_233321"; transcript_id "Novel_003249"; exon_number "5"; gene_type "
Dt_chr9	Cufflinks	exon	3127271	3127538	.	-	.	gene_id "XLOC_233321"; transcript_id "Novel_003249"; exon_number "6"; gene_type "
Dt_chr9	Cufflinks	exon	3125729	3125852	.	-	.	gene_id "XLOC_233321"; transcript_id "Novel_003250"; exon_number "1"; gene_type "
Dt_chr9	Cufflinks	exon	3126494	3126545	.	-	.	gene_id "XLOC_233321"; transcript_id "Novel_003250"; exon_number "2"; gene_type "
Dt_chr9	Cufflinks	exon	3126680	3126826	.	-	.	gene_id "XLOC_233321"; transcript_id "Novel_003250"; exon_number "3"; gene_type "
Dt_chr9	Cufflinks	exon	3127052	3127560	.	-	.	gene_id "XLOC_233321"; transcript_id "Novel_003250"; exon_number "4"; gene_type "
Dt_chr9	Cufflinks	exon	3126270	3126545	.	-	.	gene_id "XLOC_233321"; transcript_id "Novel_003251"; exon_number "1"; gene_type "
Dt_chr9	Cufflinks	exon	3126680	3126815	.	-	.	gene_id "XLOC_233321"; transcript_id "Novel_003251"; exon_number "2"; gene_type "
Dt_chr9	Cufflinks	exon	3127052	3127555	.	-	.	gene_id "XLOC_233321"; transcript_id "Novel_003251"; exon_number "3"; gene_type "
Dt_chr9	Cufflinks	exon	3138553	3139055	.	-	.	gene_id "XLOC_233325"; transcript_id "Novel_003252"; exon_number "1"; gene_type "
Dt_chr9	Cufflinks	exon	3140007	3140150	.	-	.	gene_id "XLOC_233325"; transcript_id "Novel_003252"; exon_number "2"; gene_type "
Dt_chr9	Cufflinks	exon	3143886	3144628	.	-	.	gene_id "XLOC_233326"; transcript_id "Novel_003253"; exon_number "1"; gene_type "
Dt_chr9	Cufflinks	exon	3145313	3147364	.	-	.	gene_id "XLOC_233326"; transcript_id "Novel_003253"; exon_number "2"; gene_type "
Dt_chr9	Cufflinks	exon	3143886	3144628	.	-	.	gene_id "XLOC_233326"; transcript_id "Novel_003254"; exon_number "1"; gene_type "
Dt_chr9	Cufflinks	exon	3145313	3146577	.	-	.	gene_id "XLOC_233326"; transcript_id "Novel_003254"; exon_number "2"; gene_type "
Dt_chr9	Cufflinks	exon	3147259	3147391	.	-	.	gene_id "XLOC_233326"; transcript_id "Novel_003254"; exon_number "3"; gene_type "
Dt_chr9	Cufflinks	exon	3143888	3144628	.	-	.	gene_id "XLOC_233326"; transcript_id "Novel_003255"; exon_number "1"; gene_type "
Dt_chr9	Cufflinks	exon	3145541	3146577	.	-	.	gene_id "XLOC_233326"; transcript_id "Novel_003255"; exon_number "2"; gene_type "

Dt_chr9	Cufflinks	exon	3147259	3147388	.	-	.	gene_id "XLOC_233326"; transcript_id "Novel_003255"; exon_number "3"; gene_type "
Dt_chr9	Cufflinks	exon	3144119	3146577	.	-	.	gene_id "XLOC_233326"; transcript_id "Novel_003256"; exon_number "1"; gene_type "
Dt_chr9	Cufflinks	exon	3147259	3147366	.	-	.	gene_id "XLOC_233326"; transcript_id "Novel_003256"; exon_number "2"; gene_type "
Dt_chr9	Cufflinks	exon	4634617	4636017	.	-	.	gene_id "XLOC_233528"; transcript_id "Novel_003257"; exon_number "1"; gene_type "
Dt_chr9	Cufflinks	exon	4793867	4793906	.	-	.	gene_id "XLOC_233533"; transcript_id "Novel_003258"; exon_number "1"; gene_type "
Dt_chr9	Cufflinks	exon	4794013	4794126	.	-	.	gene_id "XLOC_233533"; transcript_id "Novel_003258"; exon_number "2"; gene_type "
Dt_chr9	Cufflinks	exon	4794225	4794356	.	-	.	gene_id "XLOC_233533"; transcript_id "Novel_003258"; exon_number "3"; gene_type "
Dt_chr9	Cufflinks	exon	4794640	4794668	.	-	.	gene_id "XLOC_233533"; transcript_id "Novel_003258"; exon_number "4"; gene_type "
Dt_chr9	Cufflinks	exon	10611119	10611972	.	-	.	gene_id "XLOC_233949"; transcript_id "Novel_003259"; exon_number "1"; gene_type "
Dt_chr9	Cufflinks	exon	10612190	10612502	.	-	.	gene_id "XLOC_233949"; transcript_id "Novel_003259"; exon_number "2"; gene_type "
Dt_chr9	Cufflinks	exon	10613137	10613443	.	-	.	gene_id "XLOC_233949"; transcript_id "Novel_003259"; exon_number "3"; gene_type "
Dt_chr9	Cufflinks	exon	10614164	10614294	.	-	.	gene_id "XLOC_233949"; transcript_id "Novel_003259"; exon_number "4"; gene_type "
Dt_chr9	Cufflinks	exon	10614648	10615005	.	-	.	gene_id "XLOC_233949"; transcript_id "Novel_003259"; exon_number "5"; gene_type "
Dt_chr9	Cufflinks	exon	10615698	10615816	.	-	.	gene_id "XLOC_233949"; transcript_id "Novel_003259"; exon_number "6"; gene_type "
Dt_chr9	Cufflinks	exon	10616056	10616202	.	-	.	gene_id "XLOC_233949"; transcript_id "Novel_003259"; exon_number "7"; gene_type "
Dt_chr9	Cufflinks	exon	10611134	10611972	.	-	.	gene_id "XLOC_233949"; transcript_id "Novel_003260"; exon_number "1"; gene_type "
Dt_chr9	Cufflinks	exon	10612190	10612502	.	-	.	gene_id "XLOC_233949"; transcript_id "Novel_003260"; exon_number "2"; gene_type "
Dt_chr9	Cufflinks	exon	10613137	10613629	.	-	.	gene_id "XLOC_233949"; transcript_id "Novel_003260"; exon_number "3"; gene_type "
Dt_chr9	Cufflinks	exon	10611165	10611485	.	-	.	gene_id "XLOC_233949"; transcript_id "Novel_003261"; exon_number "1"; gene_type "
Dt_chr9	Cufflinks	exon	10611854	10611972	.	-	.	gene_id "XLOC_233949"; transcript_id "Novel_003261"; exon_number "2"; gene_type "
Dt_chr9	Cufflinks	exon	10612190	10612502	.	-	.	gene_id "XLOC_233949"; transcript_id "Novel_003261"; exon_number "3"; gene_type "
Dt_chr9	Cufflinks	exon	10613137	10613443	.	-	.	gene_id "XLOC_233949"; transcript_id "Novel_003261"; exon_number "4"; gene_type "
Dt_chr9	Cufflinks	exon	10614164	10614294	.	-	.	gene_id "XLOC_233949"; transcript_id "Novel_003261"; exon_number "5"; gene_type "
Dt_chr9	Cufflinks	exon	10614648	10615648	.	-	.	gene_id "XLOC_233949"; transcript_id "Novel_003261"; exon_number "6"; gene_type "
Dt_chr9	Cufflinks	exon	10613080	10613443	.	-	.	gene_id "XLOC_233949"; transcript_id "Novel_003262"; exon_number "1"; gene_type "
Dt_chr9	Cufflinks	exon	10614164	10614294	.	-	.	gene_id "XLOC_233949"; transcript_id "Novel_003262"; exon_number "2"; gene_type "
Dt_chr9	Cufflinks	exon	10614648	10615816	.	-	.	gene_id "XLOC_233949"; transcript_id "Novel_003262"; exon_number "3"; gene_type "
Dt_chr9	Cufflinks	exon	10616056	10616202	.	-	.	gene_id "XLOC_233949"; transcript_id "Novel_003262"; exon_number "4"; gene_type "
Dt_chr9	Cufflinks	exon	10613969	10614294	.	-	.	gene_id "XLOC_233949"; transcript_id "Novel_003263"; exon_number "1"; gene_type "
Dt_chr9	Cufflinks	exon	10614648	10615005	.	-	.	gene_id "XLOC_233949"; transcript_id "Novel_003263"; exon_number "2"; gene_type "
Dt_chr9	Cufflinks	exon	10616056	10616156	.	-	.	gene_id "XLOC_233949"; transcript_id "Novel_003263"; exon_number "3"; gene_type "
Dt_chr9	Cufflinks	exon	12291543	12292825	.	-	.	gene_id "XLOC_234019"; transcript_id "Novel_003264"; exon_number "1"; gene_type "
Dt_chr9	Cufflinks	exon	14578736	14579150	.	-	.	gene_id "XLOC_234110"; transcript_id "Novel_003265"; exon_number "1"; gene_type "
Dt_chr9	Cufflinks	exon	14579253	14579321	.	-	.	gene_id "XLOC_234110"; transcript_id "Novel_003265"; exon_number "2"; gene_type "
Dt_chr9	Cufflinks	exon	14579428	14579541	.	-	.	gene_id "XLOC_234110"; transcript_id "Novel_003265"; exon_number "3"; gene_type "

Dt_chr9	Cufflinks	exon	14579640	14579848	.	-	.	gene_id "XLOC_234110"; transcript_id "Novel_003265"; exon_number "4"; gene_type "
Dt_chr9	Cufflinks	exon	17955760	17960043	.	-	.	gene_id "XLOC_234265"; transcript_id "Novel_003266"; exon_number "1"; gene_type "
Dt_chr9	Cufflinks	exon	17963902	17964260	.	-	.	gene_id "XLOC_234265"; transcript_id "Novel_003266"; exon_number "2"; gene_type "
Dt_chr9	Cufflinks	exon	17964368	17964721	.	-	.	gene_id "XLOC_234265"; transcript_id "Novel_003266"; exon_number "3"; gene_type "
Dt_chr9	Cufflinks	exon	17964962	17965219	.	-	.	gene_id "XLOC_234265"; transcript_id "Novel_003266"; exon_number "4"; gene_type "
Dt_chr9	Cufflinks	exon	17955799	17964260	.	-	.	gene_id "XLOC_234265"; transcript_id "Novel_003267"; exon_number "1"; gene_type "
Dt_chr9	Cufflinks	exon	17964339	17964721	.	-	.	gene_id "XLOC_234265"; transcript_id "Novel_003267"; exon_number "2"; gene_type "
Dt_chr9	Cufflinks	exon	17964962	17965223	.	-	.	gene_id "XLOC_234265"; transcript_id "Novel_003267"; exon_number "3"; gene_type "
Dt_chr9	Cufflinks	exon	19087511	19087861	.	-	.	gene_id "XLOC_234325"; transcript_id "Novel_003268"; exon_number "1"; gene_type "
Dt_chr9	Cufflinks	exon	19088738	19088868	.	-	.	gene_id "XLOC_234325"; transcript_id "Novel_003268"; exon_number "2"; gene_type "
Dt_chr9	Cufflinks	exon	19299828	19299948	.	-	.	gene_id "XLOC_234337"; transcript_id "Novel_003269"; exon_number "1"; gene_type "
Dt_chr9	Cufflinks	exon	19300466	19300563	.	-	.	gene_id "XLOC_234337"; transcript_id "Novel_003269"; exon_number "2"; gene_type "
Dt_chr9	Cufflinks	exon	22561772	22562048	.	-	.	gene_id "XLOC_234536"; transcript_id "Novel_003270"; exon_number "1"; gene_type "
Dt_chr9	Cufflinks	exon	25243294	25243560	.	-	.	gene_id "XLOC_234693"; transcript_id "Novel_003271"; exon_number "1"; gene_type "
Dt_chr9	Cufflinks	exon	25244302	25244587	.	-	.	gene_id "XLOC_234693"; transcript_id "Novel_003271"; exon_number "2"; gene_type "
Dt_chr9	Cufflinks	exon	25245296	25245490	.	-	.	gene_id "XLOC_234693"; transcript_id "Novel_003271"; exon_number "3"; gene_type "
Dt_chr9	Cufflinks	exon	25245637	25246083	.	-	.	gene_id "XLOC_234693"; transcript_id "Novel_003271"; exon_number "4"; gene_type "
Dt_chr9	Cufflinks	exon	25243295	25243560	.	-	.	gene_id "XLOC_234693"; transcript_id "Novel_003272"; exon_number "1"; gene_type "
Dt_chr9	Cufflinks	exon	25244302	25244587	.	-	.	gene_id "XLOC_234693"; transcript_id "Novel_003272"; exon_number "2"; gene_type "
Dt_chr9	Cufflinks	exon	25245296	25245540	.	-	.	gene_id "XLOC_234693"; transcript_id "Novel_003272"; exon_number "3"; gene_type "
Dt_chr9	Cufflinks	exon	25245637	25245979	.	-	.	gene_id "XLOC_234693"; transcript_id "Novel_003272"; exon_number "4"; gene_type "
Dt_chr9	Cufflinks	exon	25246110	25246229	.	-	.	gene_id "XLOC_234693"; transcript_id "Novel_003272"; exon_number "5"; gene_type "
Dt_chr9	Cufflinks	exon	25437305	25437847	.	-	.	gene_id "XLOC_234708"; transcript_id "Novel_003273"; exon_number "1"; gene_type "
Dt_chr9	Cufflinks	exon	25437975	25438068	.	-	.	gene_id "XLOC_234708"; transcript_id "Novel_003273"; exon_number "2"; gene_type "
Dt_chr9	Cufflinks	exon	27777868	27777981	.	-	.	gene_id "XLOC_234848"; transcript_id "Novel_003274"; exon_number "1"; gene_type "
Dt_chr9	Cufflinks	exon	27778586	27778762	.	-	.	gene_id "XLOC_234848"; transcript_id "Novel_003274"; exon_number "2"; gene_type "
Dt_chr9	Cufflinks	exon	28122123	28122822	.	-	.	gene_id "XLOC_234877"; transcript_id "Novel_003275"; exon_number "1"; gene_type "
Dt_chr9	Cufflinks	exon	28122930	28123661	.	-	.	gene_id "XLOC_234877"; transcript_id "Novel_003275"; exon_number "2"; gene_type "
Dt_chr9	Cufflinks	exon	28566123	28566366	.	-	.	gene_id "XLOC_234925"; transcript_id "Novel_003276"; exon_number "1"; gene_type "
Dt_chr9	Cufflinks	exon	29367747	29368306	.	-	.	gene_id "XLOC_234977"; transcript_id "Novel_003277"; exon_number "1"; gene_type "
Dt_chr9	Cufflinks	exon	29371866	29372023	.	-	.	gene_id "XLOC_234977"; transcript_id "Novel_003277"; exon_number "2"; gene_type "
Dt_chr9	Cufflinks	exon	29372113	29372708	.	-	.	gene_id "XLOC_234977"; transcript_id "Novel_003277"; exon_number "3"; gene_type "
Dt_chr9	Cufflinks	exon	31026354	31026867	.	-	.	gene_id "XLOC_235067"; transcript_id "Novel_003278"; exon_number "1"; gene_type "
Dt_chr9	Cufflinks	exon	31027672	31027706	.	-	.	gene_id "XLOC_235067"; transcript_id "Novel_003278"; exon_number "2"; gene_type "
Dt_chr9	Cufflinks	exon	31562382	31562766	.	-	.	gene_id "XLOC_235094"; transcript_id "Novel_003279"; exon_number "1"; gene_type "

Dt_chr9	Cufflinks	exon	31562848	31563276	.	-	.	gene_id "XLOC_235094"; transcript_id "Novel_003279"; exon_number "2"; gene_type "
Dt_chr9	Cufflinks	exon	31563377	31563421	.	-	.	gene_id "XLOC_235094"; transcript_id "Novel_003279"; exon_number "3"; gene_type "
Dt_chr9	Cufflinks	exon	31563498	31563602	.	-	.	gene_id "XLOC_235094"; transcript_id "Novel_003279"; exon_number "4"; gene_type "
Dt_chr9	Cufflinks	exon	31563703	31563961	.	-	.	gene_id "XLOC_235094"; transcript_id "Novel_003279"; exon_number "5"; gene_type "
Dt_chr9	Cufflinks	exon	31564048	31564168	.	-	.	gene_id "XLOC_235094"; transcript_id "Novel_003279"; exon_number "6"; gene_type "
Dt_chr9	Cufflinks	exon	31564244	31564373	.	-	.	gene_id "XLOC_235094"; transcript_id "Novel_003279"; exon_number "7"; gene_type "
Dt_chr9	Cufflinks	exon	31564465	31564536	.	-	.	gene_id "XLOC_235094"; transcript_id "Novel_003279"; exon_number "8"; gene_type "
Dt_chr9	Cufflinks	exon	31564615	31564663	.	-	.	gene_id "XLOC_235094"; transcript_id "Novel_003279"; exon_number "9"; gene_type "
Dt_chr9	Cufflinks	exon	31564750	31565853	.	-	.	gene_id "XLOC_235094"; transcript_id "Novel_003279"; exon_number "10"; gene_type
Dt_chr9	Cufflinks	exon	31562386	31562766	.	-	.	gene_id "XLOC_235094"; transcript_id "Novel_003280"; exon_number "1"; gene_type "
Dt_chr9	Cufflinks	exon	31562848	31563276	.	-	.	gene_id "XLOC_235094"; transcript_id "Novel_003280"; exon_number "2"; gene_type "
Dt_chr9	Cufflinks	exon	31563377	31563421	.	-	.	gene_id "XLOC_235094"; transcript_id "Novel_003280"; exon_number "3"; gene_type "
Dt_chr9	Cufflinks	exon	31563498	31563602	.	-	.	gene_id "XLOC_235094"; transcript_id "Novel_003280"; exon_number "4"; gene_type "
Dt_chr9	Cufflinks	exon	31563703	31563961	.	-	.	gene_id "XLOC_235094"; transcript_id "Novel_003280"; exon_number "5"; gene_type "
Dt_chr9	Cufflinks	exon	31564048	31564373	.	-	.	gene_id "XLOC_235094"; transcript_id "Novel_003280"; exon_number "6"; gene_type "
Dt_chr9	Cufflinks	exon	31564465	31564536	.	-	.	gene_id "XLOC_235094"; transcript_id "Novel_003280"; exon_number "7"; gene_type "
Dt_chr9	Cufflinks	exon	31564615	31564663	.	-	.	gene_id "XLOC_235094"; transcript_id "Novel_003280"; exon_number "8"; gene_type "
Dt_chr9	Cufflinks	exon	31564750	31565071	.	-	.	gene_id "XLOC_235094"; transcript_id "Novel_003280"; exon_number "9"; gene_type "
Dt_chr9	Cufflinks	exon	31565148	31565450	.	-	.	gene_id "XLOC_235094"; transcript_id "Novel_003280"; exon_number "10"; gene_type
Dt_chr9	Cufflinks	exon	33124972	33125663	.	-	.	gene_id "XLOC_235266"; transcript_id "Novel_003281"; exon_number "1"; gene_type "
Dt_chr9	Cufflinks	exon	33125753	33126089	.	-	.	gene_id "XLOC_235266"; transcript_id "Novel_003281"; exon_number "2"; gene_type "
Dt_chr9	Cufflinks	exon	33126194	33126321	.	-	.	gene_id "XLOC_235266"; transcript_id "Novel_003281"; exon_number "3"; gene_type "
Dt_chr9	Cufflinks	exon	33126590	33127033	.	-	.	gene_id "XLOC_235266"; transcript_id "Novel_003281"; exon_number "4"; gene_type "
Dt_chr9	Cufflinks	exon	33127106	33127196	.	-	.	gene_id "XLOC_235266"; transcript_id "Novel_003281"; exon_number "5"; gene_type "
Dt_chr9	Cufflinks	exon	33127280	33127389	.	-	.	gene_id "XLOC_235266"; transcript_id "Novel_003281"; exon_number "6"; gene_type "
Dt_chr9	Cufflinks	exon	33127661	33127770	.	-	.	gene_id "XLOC_235266"; transcript_id "Novel_003281"; exon_number "7"; gene_type "
Dt_chr9	Cufflinks	exon	33127890	33127992	.	-	.	gene_id "XLOC_235266"; transcript_id "Novel_003281"; exon_number "8"; gene_type "
Dt_chr9	Cufflinks	exon	33128393	33128437	.	-	.	gene_id "XLOC_235266"; transcript_id "Novel_003281"; exon_number "9"; gene_type "
Dt_chr9	Cufflinks	exon	33128553	33128677	.	-	.	gene_id "XLOC_235266"; transcript_id "Novel_003281"; exon_number "10"; gene_type
Dt_chr9	Cufflinks	exon	33128795	33128865	.	-	.	gene_id "XLOC_235266"; transcript_id "Novel_003281"; exon_number "11"; gene_type
Dt_chr9	Cufflinks	exon	33128966	33129120	.	-	.	gene_id "XLOC_235266"; transcript_id "Novel_003281"; exon_number "12"; gene_type
Dt_chr9	Cufflinks	exon	33125002	33125663	.	-	.	gene_id "XLOC_235266"; transcript_id "Novel_003282"; exon_number "1"; gene_type "
Dt_chr9	Cufflinks	exon	33125753	33126089	.	-	.	gene_id "XLOC_235266"; transcript_id "Novel_003282"; exon_number "2"; gene_type "
Dt_chr9	Cufflinks	exon	33126194	33126321	.	-	.	gene_id "XLOC_235266"; transcript_id "Novel_003282"; exon_number "3"; gene_type "
Dt_chr9	Cufflinks	exon	33126590	33127033	.	-	.	gene_id "XLOC_235266"; transcript_id "Novel_003282"; exon_number "4"; gene_type "

Dt_chr9	Cufflinks	exon	33127106	33127196	.	-	.	gene_id "XLOC_235266"; transcript_id "Novel_003282"; exon_number "5"; gene_type "
Dt_chr9	Cufflinks	exon	33127280	33127389	.	-	.	gene_id "XLOC_235266"; transcript_id "Novel_003282"; exon_number "6"; gene_type "
Dt_chr9	Cufflinks	exon	33127661	33127770	.	-	.	gene_id "XLOC_235266"; transcript_id "Novel_003282"; exon_number "7"; gene_type "
Dt_chr9	Cufflinks	exon	33127890	33128187	.	-	.	gene_id "XLOC_235266"; transcript_id "Novel_003282"; exon_number "8"; gene_type "
Dt_chr9	Cufflinks	exon	33128299	33128437	.	-	.	gene_id "XLOC_235266"; transcript_id "Novel_003283"; exon_number "1"; gene_type "
Dt_chr9	Cufflinks	exon	33128553	33128677	.	-	.	gene_id "XLOC_235266"; transcript_id "Novel_003283"; exon_number "2"; gene_type "
Dt_chr9	Cufflinks	exon	33128795	33128865	.	-	.	gene_id "XLOC_235266"; transcript_id "Novel_003283"; exon_number "3"; gene_type "
Dt_chr9	Cufflinks	exon	33128966	33129133	.	-	.	gene_id "XLOC_235266"; transcript_id "Novel_003283"; exon_number "4"; gene_type "
Dt_chr9	Cufflinks	exon	38752441	38752955	.	-	.	gene_id "XLOC_235838"; transcript_id "Novel_003284"; exon_number "1"; gene_type "
Dt_chr9	Cufflinks	exon	38753113	38753224	.	-	.	gene_id "XLOC_235838"; transcript_id "Novel_003284"; exon_number "2"; gene_type "
Dt_chr9	Cufflinks	exon	41134863	41135076	.	-	.	gene_id "XLOC_236128"; transcript_id "Novel_003285"; exon_number "1"; gene_type "
Dt_chr9	Cufflinks	exon	41135176	41135242	.	-	.	gene_id "XLOC_236128"; transcript_id "Novel_003285"; exon_number "2"; gene_type "
Dt_chr9	Cufflinks	exon	41135881	41135983	.	-	.	gene_id "XLOC_236128"; transcript_id "Novel_003285"; exon_number "3"; gene_type "
Dt_chr9	Cufflinks	exon	41136297	41136383	.	-	.	gene_id "XLOC_236128"; transcript_id "Novel_003285"; exon_number "4"; gene_type "
Dt_chr9	Cufflinks	exon	41136486	41136711	.	-	.	gene_id "XLOC_236128"; transcript_id "Novel_003285"; exon_number "5"; gene_type "
Dt_chr9	Cufflinks	exon	41456695	41457542	.	-	.	gene_id "XLOC_236188"; transcript_id "Novel_003286"; exon_number "1"; gene_type "
Dt_chr9	Cufflinks	exon	42413637	42414198	.	-	.	gene_id "XLOC_236339"; transcript_id "Novel_003287"; exon_number "1"; gene_type "
Dt_chr9	Cufflinks	exon	42418097	42418243	.	-	.	gene_id "XLOC_236339"; transcript_id "Novel_003287"; exon_number "2"; gene_type "
Dt_chr9	Cufflinks	exon	42418329	42418382	.	-	.	gene_id "XLOC_236339"; transcript_id "Novel_003287"; exon_number "3"; gene_type "
Dt_chr9	Cufflinks	exon	42418676	42418840	.	-	.	gene_id "XLOC_236339"; transcript_id "Novel_003287"; exon_number "4"; gene_type "
Dt_chr9	Cufflinks	exon	42419323	42419535	.	-	.	gene_id "XLOC_236339"; transcript_id "Novel_003287"; exon_number "5"; gene_type "
Dt_chr9	Cufflinks	exon	42419653	42419739	.	-	.	gene_id "XLOC_236339"; transcript_id "Novel_003287"; exon_number "6"; gene_type "
Dt_chr9	Cufflinks	exon	42419827	42420445	.	-	.	gene_id "XLOC_236339"; transcript_id "Novel_003287"; exon_number "7"; gene_type "
Dt_chr9	Cufflinks	exon	45675313	45678017	.	-	.	gene_id "XLOC_236639"; transcript_id "Novel_003288"; exon_number "1"; gene_type "
Dt_chr9	Cufflinks	exon	45679058	45679330	.	-	.	gene_id "XLOC_236639"; transcript_id "Novel_003288"; exon_number "2"; gene_type "
Dt_chr9	Cufflinks	exon	46541626	46542332	.	-	.	gene_id "XLOC_236744"; transcript_id "Novel_003289"; exon_number "1"; gene_type "
Dt_chr9	Cufflinks	exon	46543537	46544039	.	-	.	gene_id "XLOC_236744"; transcript_id "Novel_003289"; exon_number "2"; gene_type "
Dt_chr9	Cufflinks	exon	46575436	46575491	.	-	.	gene_id "XLOC_236749"; transcript_id "Novel_003290"; exon_number "1"; gene_type "
Dt_chr9	Cufflinks	exon	46575620	46575712	.	-	.	gene_id "XLOC_236749"; transcript_id "Novel_003290"; exon_number "2"; gene_type "
Dt_chr9	Cufflinks	exon	46575804	46576514	.	-	.	gene_id "XLOC_236749"; transcript_id "Novel_003290"; exon_number "3"; gene_type "
Dt_chr9	Cufflinks	exon	46641125	46644146	.	-	.	gene_id "XLOC_236756"; transcript_id "Novel_003291"; exon_number "1"; gene_type "
Dt_chr9	Cufflinks	exon	46644245	46644401	.	-	.	gene_id "XLOC_236756"; transcript_id "Novel_003291"; exon_number "2"; gene_type "
Dt_chr9	Cufflinks	exon	46644694	46644817	.	-	.	gene_id "XLOC_236756"; transcript_id "Novel_003291"; exon_number "3"; gene_type "
Dt_chr9	Cufflinks	exon	46777948	46778287	.	-	.	gene_id "XLOC_236778"; transcript_id "Novel_003292"; exon_number "1"; gene_type "
Dt_chr9	Cufflinks	exon	46778494	46778566	.	-	.	gene_id "XLOC_236778"; transcript_id "Novel_003292"; exon_number "2"; gene_type "

Dt_chr9	Cufflinks	exon	46778723	46779207	.	-	.	gene_id "XLOC_236778"; transcript_id "Novel_003292"; exon_number "3"; gene_type "
Dt_chr9	Cufflinks	exon	46779314	46779336	.	-	.	gene_id "XLOC_236778"; transcript_id "Novel_003292"; exon_number "4"; gene_type "
Dt_chr9	Cufflinks	exon	46779420	46779589	.	-	.	gene_id "XLOC_236778"; transcript_id "Novel_003292"; exon_number "5"; gene_type "
Dt_chr9	Cufflinks	exon	46777952	46778566	.	-	.	gene_id "XLOC_236778"; transcript_id "Novel_003293"; exon_number "1"; gene_type "
Dt_chr9	Cufflinks	exon	46778723	46779207	.	-	.	gene_id "XLOC_236778"; transcript_id "Novel_003293"; exon_number "2"; gene_type "
Dt_chr9	Cufflinks	exon	46779314	46779336	.	-	.	gene_id "XLOC_236778"; transcript_id "Novel_003293"; exon_number "3"; gene_type "
Dt_chr9	Cufflinks	exon	46779420	46779591	.	-	.	gene_id "XLOC_236778"; transcript_id "Novel_003293"; exon_number "4"; gene_type "
Dt_chr9	Cufflinks	exon	46777954	46778566	.	-	.	gene_id "XLOC_236778"; transcript_id "Novel_003294"; exon_number "1"; gene_type "
Dt_chr9	Cufflinks	exon	46778723	46778793	.	-	.	gene_id "XLOC_236778"; transcript_id "Novel_003294"; exon_number "2"; gene_type "
Dt_chr9	Cufflinks	exon	46779102	46779207	.	-	.	gene_id "XLOC_236778"; transcript_id "Novel_003294"; exon_number "3"; gene_type "
Dt_chr9	Cufflinks	exon	46779314	46779336	.	-	.	gene_id "XLOC_236778"; transcript_id "Novel_003294"; exon_number "4"; gene_type "
Dt_chr9	Cufflinks	exon	46779420	46779599	.	-	.	gene_id "XLOC_236778"; transcript_id "Novel_003294"; exon_number "5"; gene_type "
Dt_chr9	Cufflinks	exon	46777954	46778287	.	-	.	gene_id "XLOC_236778"; transcript_id "Novel_003295"; exon_number "1"; gene_type "
Dt_chr9	Cufflinks	exon	46778494	46778566	.	-	.	gene_id "XLOC_236778"; transcript_id "Novel_003295"; exon_number "2"; gene_type "
Dt_chr9	Cufflinks	exon	46778723	46778793	.	-	.	gene_id "XLOC_236778"; transcript_id "Novel_003295"; exon_number "3"; gene_type "
Dt_chr9	Cufflinks	exon	46779102	46779207	.	-	.	gene_id "XLOC_236778"; transcript_id "Novel_003295"; exon_number "4"; gene_type "
Dt_chr9	Cufflinks	exon	46779314	46779336	.	-	.	gene_id "XLOC_236778"; transcript_id "Novel_003295"; exon_number "5"; gene_type "
Dt_chr9	Cufflinks	exon	46779420	46779599	.	-	.	gene_id "XLOC_236778"; transcript_id "Novel_003295"; exon_number "6"; gene_type "
Dt_chr9	Cufflinks	exon	46803151	46803234	.	-	.	gene_id "XLOC_236787"; transcript_id "Novel_003296"; exon_number "1"; gene_type "
Dt_chr9	Cufflinks	exon	46803440	46803477	.	-	.	gene_id "XLOC_236787"; transcript_id "Novel_003296"; exon_number "2"; gene_type "
Dt_chr9	Cufflinks	exon	46803982	46804006	.	-	.	gene_id "XLOC_236787"; transcript_id "Novel_003296"; exon_number "3"; gene_type "
Dt_chr9	Cufflinks	exon	46804131	46804217	.	-	.	gene_id "XLOC_236787"; transcript_id "Novel_003296"; exon_number "4"; gene_type "
Dt_chr9	Cufflinks	exon	46804324	46804376	.	-	.	gene_id "XLOC_236787"; transcript_id "Novel_003296"; exon_number "5"; gene_type "
Dt_chr9	Cufflinks	exon	46803152	46803234	.	-	.	gene_id "XLOC_236787"; transcript_id "Novel_003297"; exon_number "1"; gene_type "
Dt_chr9	Cufflinks	exon	46803440	46803477	.	-	.	gene_id "XLOC_236787"; transcript_id "Novel_003297"; exon_number "2"; gene_type "
Dt_chr9	Cufflinks	exon	46803982	46804006	.	-	.	gene_id "XLOC_236787"; transcript_id "Novel_003297"; exon_number "3"; gene_type "
Dt_chr9	Cufflinks	exon	46804131	46804217	.	-	.	gene_id "XLOC_236787"; transcript_id "Novel_003297"; exon_number "4"; gene_type "
Dt_chr9	Cufflinks	exon	46804321	46804501	.	-	.	gene_id "XLOC_236787"; transcript_id "Novel_003297"; exon_number "5"; gene_type "
Dt_chr9	Cufflinks	exon	47696921	47697273	.	-	.	gene_id "XLOC_236903"; transcript_id "Novel_003298"; exon_number "1"; gene_type "
Dt_chr9	Cufflinks	exon	47697707	47697942	.	-	.	gene_id "XLOC_236903"; transcript_id "Novel_003298"; exon_number "2"; gene_type "
Dt_chr9	Cufflinks	exon	47700686	47701947	.	-	.	gene_id "XLOC_236906"; transcript_id "Novel_003299"; exon_number "1"; gene_type "
Dt_chr9	Cufflinks	exon	47702374	47702452	.	-	.	gene_id "XLOC_236906"; transcript_id "Novel_003299"; exon_number "2"; gene_type "
Dt_chr9	Cufflinks	exon	47702618	47704002	.	-	.	gene_id "XLOC_236906"; transcript_id "Novel_003299"; exon_number "3"; gene_type "
Dt_chr9	Cufflinks	exon	47704091	47704159	.	-	.	gene_id "XLOC_236906"; transcript_id "Novel_003299"; exon_number "4"; gene_type "
Dt_chr9	Cufflinks	exon	47704489	47704554	.	-	.	gene_id "XLOC_236906"; transcript_id "Novel_003299"; exon_number "5"; gene_type "

Dt_chr9	Cufflinks	exon	47704633	47704776	.	-	.	gene_id "XLOC_236906"; transcript_id "Novel_003299"; exon_number "6"; gene_type "
Dt_chr9	Cufflinks	exon	47705288	47705384	.	-	.	gene_id "XLOC_236906"; transcript_id "Novel_003299"; exon_number "7"; gene_type "
Dt_chr9	Cufflinks	exon	47705536	47705634	.	-	.	gene_id "XLOC_236906"; transcript_id "Novel_003299"; exon_number "8"; gene_type "
Dt_chr9	Cufflinks	exon	47705960	47706082	.	-	.	gene_id "XLOC_236906"; transcript_id "Novel_003299"; exon_number "9"; gene_type "
Dt_chr9	Cufflinks	exon	47706190	47706932	.	-	.	gene_id "XLOC_236906"; transcript_id "Novel_003299"; exon_number "10"; gene_type
Dt_chr9	Cufflinks	exon	47701561	47701811	.	-	.	gene_id "XLOC_236906"; transcript_id "Novel_003300"; exon_number "1"; gene_type "
Dt_chr9	Cufflinks	exon	47701881	47701947	.	-	.	gene_id "XLOC_236906"; transcript_id "Novel_003300"; exon_number "2"; gene_type "
Dt_chr9	Cufflinks	exon	47702374	47702452	.	-	.	gene_id "XLOC_236906"; transcript_id "Novel_003300"; exon_number "3"; gene_type "
Dt_chr9	Cufflinks	exon	47702618	47703156	.	-	.	gene_id "XLOC_236906"; transcript_id "Novel_003300"; exon_number "4"; gene_type "
Dt_chr9	Cufflinks	exon	47703895	47704002	.	-	.	gene_id "XLOC_236906"; transcript_id "Novel_003300"; exon_number "5"; gene_type "
Dt_chr9	Cufflinks	exon	47704091	47704159	.	-	.	gene_id "XLOC_236906"; transcript_id "Novel_003300"; exon_number "6"; gene_type "
Dt_chr9	Cufflinks	exon	47704489	47704554	.	-	.	gene_id "XLOC_236906"; transcript_id "Novel_003300"; exon_number "7"; gene_type "
Dt_chr9	Cufflinks	exon	47704633	47704776	.	-	.	gene_id "XLOC_236906"; transcript_id "Novel_003300"; exon_number "8"; gene_type "
Dt_chr9	Cufflinks	exon	47705288	47705384	.	-	.	gene_id "XLOC_236906"; transcript_id "Novel_003300"; exon_number "9"; gene_type "
Dt_chr9	Cufflinks	exon	47705536	47705634	.	-	.	gene_id "XLOC_236906"; transcript_id "Novel_003300"; exon_number "10"; gene_type
Dt_chr9	Cufflinks	exon	47705960	47706082	.	-	.	gene_id "XLOC_236906"; transcript_id "Novel_003300"; exon_number "11"; gene_type
Dt_chr9	Cufflinks	exon	47706190	47706958	.	-	.	gene_id "XLOC_236906"; transcript_id "Novel_003300"; exon_number "12"; gene_type
Dt_chr9	Cufflinks	exon	47705042	47705384	.	-	.	gene_id "XLOC_236906"; transcript_id "Novel_003301"; exon_number "1"; gene_type "
Dt_chr9	Cufflinks	exon	47705536	47705634	.	-	.	gene_id "XLOC_236906"; transcript_id "Novel_003301"; exon_number "2"; gene_type "
Dt_chr9	Cufflinks	exon	47705960	47706082	.	-	.	gene_id "XLOC_236906"; transcript_id "Novel_003301"; exon_number "3"; gene_type "
Dt_chr9	Cufflinks	exon	47706190	47706932	.	-	.	gene_id "XLOC_236906"; transcript_id "Novel_003301"; exon_number "4"; gene_type "
Dt_chr9	Cufflinks	exon	47714261	47714558	.	-	.	gene_id "XLOC_236908"; transcript_id "Novel_003302"; exon_number "1"; gene_type "
Dt_chr9	Cufflinks	exon	47714882	47714976	.	-	.	gene_id "XLOC_236908"; transcript_id "Novel_003302"; exon_number "2"; gene_type "
Dt_chr9	Cufflinks	exon	47715272	47715303	.	-	.	gene_id "XLOC_236908"; transcript_id "Novel_003302"; exon_number "3"; gene_type "
Dt_chr9	Cufflinks	exon	47715530	47715591	.	-	.	gene_id "XLOC_236908"; transcript_id "Novel_003302"; exon_number "4"; gene_type "
Dt_chr9	Cufflinks	exon	47715760	47715875	.	-	.	gene_id "XLOC_236908"; transcript_id "Novel_003302"; exon_number "5"; gene_type "
Dt_chr9	Cufflinks	exon	47715982	47716218	.	-	.	gene_id "XLOC_236908"; transcript_id "Novel_003302"; exon_number "6"; gene_type "
Dt_chr9	Cufflinks	exon	47714316	47714558	.	-	.	gene_id "XLOC_236908"; transcript_id "Novel_003303"; exon_number "1"; gene_type "
Dt_chr9	Cufflinks	exon	47714882	47715149	.	-	.	gene_id "XLOC_236908"; transcript_id "Novel_003303"; exon_number "2"; gene_type "
Dt_chr9	Cufflinks	exon	47787930	47788424	.	-	.	gene_id "XLOC_236919"; transcript_id "Novel_003304"; exon_number "1"; gene_type "
Dt_chr9	Cufflinks	exon	47789538	47790438	.	-	.	gene_id "XLOC_236919"; transcript_id "Novel_003304"; exon_number "2"; gene_type "
Dt_chr9	Cufflinks	exon	47790545	47790674	.	-	.	gene_id "XLOC_236919"; transcript_id "Novel_003304"; exon_number "3"; gene_type "
Dt_chr9	Cufflinks	exon	47788015	47788424	.	-	.	gene_id "XLOC_236919"; transcript_id "Novel_003305"; exon_number "1"; gene_type "
Dt_chr9	Cufflinks	exon	47789538	47790708	.	-	.	gene_id "XLOC_236919"; transcript_id "Novel_003305"; exon_number "2"; gene_type "
Dt_chr9	Cufflinks	exon	47818400	47818998	.	-	.	gene_id "XLOC_236927"; transcript_id "Novel_003306"; exon_number "1"; gene_type "

Dt_chr9	Cufflinks	exon	47819970	47819997	.	-	.	gene_id "XLOC_236927"; transcript_id "Novel_003306"; exon_number "2"; gene_type "
Dt_chr9	Cufflinks	exon	47818400	47818998	.	-	.	gene_id "XLOC_236927"; transcript_id "Novel_003307"; exon_number "1"; gene_type "
Dt_chr9	Cufflinks	exon	47825939	47825966	.	-	.	gene_id "XLOC_236927"; transcript_id "Novel_003307"; exon_number "2"; gene_type "
Dt_chr9	Cufflinks	exon	47818484	47818998	.	-	.	gene_id "XLOC_236927"; transcript_id "Novel_003308"; exon_number "1"; gene_type "
Dt_chr9	Cufflinks	exon	47832656	47832671	.	-	.	gene_id "XLOC_236927"; transcript_id "Novel_003308"; exon_number "2"; gene_type "
Dt_chr9	Cufflinks	exon	47824757	47825516	.	-	.	gene_id "XLOC_236927"; transcript_id "Novel_003309"; exon_number "1"; gene_type "
Dt_chr9	Cufflinks	exon	47825939	47826977	.	-	.	gene_id "XLOC_236927"; transcript_id "Novel_003309"; exon_number "2"; gene_type "
Dt_chr9	Cufflinks	exon	47939196	47939555	.	-	.	gene_id "XLOC_236945"; transcript_id "Novel_003310"; exon_number "1"; gene_type "
Dt_chr9	Cufflinks	exon	47939790	47939898	.	-	.	gene_id "XLOC_236945"; transcript_id "Novel_003310"; exon_number "2"; gene_type "
Dt_chr9	Cufflinks	exon	47940147	47940208	.	-	.	gene_id "XLOC_236945"; transcript_id "Novel_003310"; exon_number "3"; gene_type "
Dt_chr9	Cufflinks	exon	47940292	47940300	.	-	.	gene_id "XLOC_236945"; transcript_id "Novel_003310"; exon_number "4"; gene_type "
Dt_chr9	Cufflinks	exon	47940370	47940390	.	-	.	gene_id "XLOC_236945"; transcript_id "Novel_003311"; exon_number "1"; gene_type "
Dt_chr9	Cufflinks	exon	47940466	47940570	.	-	.	gene_id "XLOC_236945"; transcript_id "Novel_003311"; exon_number "2"; gene_type "
Dt_chr9	Cufflinks	exon	47940793	47940872	.	-	.	gene_id "XLOC_236945"; transcript_id "Novel_003311"; exon_number "3"; gene_type "
Dt_chr9	Cufflinks	exon	47941089	47941161	.	-	.	gene_id "XLOC_236945"; transcript_id "Novel_003311"; exon_number "4"; gene_type "
Dt_chr9	Cufflinks	exon	47941264	47941353	.	-	.	gene_id "XLOC_236945"; transcript_id "Novel_003311"; exon_number "5"; gene_type "
Dt_chr9	Cufflinks	exon	47941850	47941912	.	-	.	gene_id "XLOC_236945"; transcript_id "Novel_003311"; exon_number "6"; gene_type "
Dt_chr9	Cufflinks	exon	47942941	47942992	.	-	.	gene_id "XLOC_236945"; transcript_id "Novel_003311"; exon_number "7"; gene_type "
Dt_chr9	Cufflinks	exon	47943541	47943641	.	-	.	gene_id "XLOC_236945"; transcript_id "Novel_003311"; exon_number "8"; gene_type "
Dt_chr9	Cufflinks	exon	47943984	47944083	.	-	.	gene_id "XLOC_236945"; transcript_id "Novel_003311"; exon_number "9"; gene_type "
Dt_chr9	Cufflinks	exon	47944700	47944763	.	-	.	gene_id "XLOC_236945"; transcript_id "Novel_003311"; exon_number "10"; gene_type
Dt_chr9	Cufflinks	exon	47945532	47945570	.	-	.	gene_id "XLOC_236945"; transcript_id "Novel_003311"; exon_number "11"; gene_type
Dt_chr9	Cufflinks	exon	47940535	47940872	.	-	.	gene_id "XLOC_236945"; transcript_id "Novel_003312"; exon_number "1"; gene_type "
Dt_chr9	Cufflinks	exon	47941089	47941161	.	-	.	gene_id "XLOC_236945"; transcript_id "Novel_003312"; exon_number "2"; gene_type "
Dt_chr9	Cufflinks	exon	47941264	47941353	.	-	.	gene_id "XLOC_236945"; transcript_id "Novel_003312"; exon_number "3"; gene_type "
Dt_chr9	Cufflinks	exon	47941850	47941912	.	-	.	gene_id "XLOC_236945"; transcript_id "Novel_003312"; exon_number "4"; gene_type "
Dt_chr9	Cufflinks	exon	47942941	47942992	.	-	.	gene_id "XLOC_236945"; transcript_id "Novel_003312"; exon_number "5"; gene_type "
Dt_chr9	Cufflinks	exon	47943541	47943641	.	-	.	gene_id "XLOC_236945"; transcript_id "Novel_003312"; exon_number "6"; gene_type "
Dt_chr9	Cufflinks	exon	47943984	47944083	.	-	.	gene_id "XLOC_236945"; transcript_id "Novel_003312"; exon_number "7"; gene_type "
Dt_chr9	Cufflinks	exon	47944700	47944763	.	-	.	gene_id "XLOC_236945"; transcript_id "Novel_003312"; exon_number "8"; gene_type "
Dt_chr9	Cufflinks	exon	47945532	47945673	.	-	.	gene_id "XLOC_236945"; transcript_id "Novel_003312"; exon_number "9"; gene_type "
Dt_chr9	Cufflinks	exon	47941104	47941161	.	-	.	gene_id "XLOC_236945"; transcript_id "Novel_003313"; exon_number "1"; gene_type "
Dt_chr9	Cufflinks	exon	47941264	47941353	.	-	.	gene_id "XLOC_236945"; transcript_id "Novel_003313"; exon_number "2"; gene_type "
Dt_chr9	Cufflinks	exon	47941850	47941912	.	-	.	gene_id "XLOC_236945"; transcript_id "Novel_003313"; exon_number "3"; gene_type "
Dt_chr9	Cufflinks	exon	47942941	47942992	.	-	.	gene_id "XLOC_236945"; transcript_id "Novel_003313"; exon_number "4"; gene_type "

Dt_chr9	Cufflinks	exon	47943541	47943693	.	-	.	gene_id "XLOC_236945"; transcript_id "Novel_003313"; exon_number "5"; gene_type "
Dt_chr9	Cufflinks	exon	47941121	47941161	.	-	.	gene_id "XLOC_236945"; transcript_id "Novel_003314"; exon_number "1"; gene_type "
Dt_chr9	Cufflinks	exon	47941264	47941353	.	-	.	gene_id "XLOC_236945"; transcript_id "Novel_003314"; exon_number "2"; gene_type "
Dt_chr9	Cufflinks	exon	47941850	47941962	.	-	.	gene_id "XLOC_236945"; transcript_id "Novel_003314"; exon_number "3"; gene_type "
Dt_chr9	Cufflinks	exon	48018876	48019516	.	-	.	gene_id "XLOC_236968"; transcript_id "Novel_003315"; exon_number "1"; gene_type "
Dt_chr9	Cufflinks	exon	48019603	48019933	.	-	.	gene_id "XLOC_236968"; transcript_id "Novel_003315"; exon_number "2"; gene_type "
Dt_chr9	Cufflinks	exon	48019012	48019516	.	-	.	gene_id "XLOC_236968"; transcript_id "Novel_003316"; exon_number "1"; gene_type "
Dt_chr9	Cufflinks	exon	48019603	48019719	.	-	.	gene_id "XLOC_236968"; transcript_id "Novel_003316"; exon_number "2"; gene_type "
Dt_chr9	Cufflinks	exon	48019814	48020155	.	-	.	gene_id "XLOC_236968"; transcript_id "Novel_003316"; exon_number "3"; gene_type "
Dt_chr9	Cufflinks	exon	48048473	48048499	.	-	.	gene_id "XLOC_236974"; transcript_id "Novel_003317"; exon_number "1"; gene_type "
Dt_chr9	Cufflinks	exon	48048607	48048691	.	-	.	gene_id "XLOC_236974"; transcript_id "Novel_003317"; exon_number "2"; gene_type "
Dt_chr9	Cufflinks	exon	48049227	48049487	.	-	.	gene_id "XLOC_236974"; transcript_id "Novel_003317"; exon_number "3"; gene_type "
Dt_chr9	Cufflinks	exon	48049605	48050131	.	-	.	gene_id "XLOC_236974"; transcript_id "Novel_003317"; exon_number "4"; gene_type "
Dt_chr9	Cufflinks	exon	48177150	48178003	.	-	.	gene_id "XLOC_237006"; transcript_id "Novel_003318"; exon_number "1"; gene_type "
Dt_chr9	Cufflinks	exon	48178839	48179115	.	-	.	gene_id "XLOC_237006"; transcript_id "Novel_003318"; exon_number "2"; gene_type "
Dt_chr9	Cufflinks	exon	48179210	48179449	.	-	.	gene_id "XLOC_237006"; transcript_id "Novel_003318"; exon_number "3"; gene_type "
Dt_chr9	Cufflinks	exon	48180193	48180289	.	-	.	gene_id "XLOC_237006"; transcript_id "Novel_003318"; exon_number "4"; gene_type "
Dt_chr9	Cufflinks	exon	48180396	48180803	.	-	.	gene_id "XLOC_237006"; transcript_id "Novel_003318"; exon_number "5"; gene_type "
Dt_chr9	Cufflinks	exon	48177413	48178003	.	-	.	gene_id "XLOC_237006"; transcript_id "Novel_003319"; exon_number "1"; gene_type "
Dt_chr9	Cufflinks	exon	48178839	48179449	.	-	.	gene_id "XLOC_237006"; transcript_id "Novel_003319"; exon_number "2"; gene_type "
Dt_chr9	Cufflinks	exon	48180193	48180289	.	-	.	gene_id "XLOC_237006"; transcript_id "Novel_003319"; exon_number "3"; gene_type "
Dt_chr9	Cufflinks	exon	48180396	48180802	.	-	.	gene_id "XLOC_237006"; transcript_id "Novel_003319"; exon_number "4"; gene_type "
Dt_chr9	Cufflinks	exon	48305506	48305646	.	-	.	gene_id "XLOC_237024"; transcript_id "Novel_003320"; exon_number "1"; gene_type "
Dt_chr9	Cufflinks	exon	48305989	48307016	.	-	.	gene_id "XLOC_237024"; transcript_id "Novel_003320"; exon_number "2"; gene_type "
Dt_chr9	Cufflinks	exon	48383922	48385227	.	-	.	gene_id "XLOC_237043"; transcript_id "Novel_003321"; exon_number "1"; gene_type "
Dt_chr9	Cufflinks	exon	48385553	48385722	.	-	.	gene_id "XLOC_237043"; transcript_id "Novel_003321"; exon_number "2"; gene_type "
Dt_chr9	Cufflinks	exon	48384304	48384377	.	-	.	gene_id "XLOC_237043"; transcript_id "Novel_003322"; exon_number "1"; gene_type "
Dt_chr9	Cufflinks	exon	48384524	48385227	.	-	.	gene_id "XLOC_237043"; transcript_id "Novel_003322"; exon_number "2"; gene_type "
Dt_chr9	Cufflinks	exon	48385553	48385731	.	-	.	gene_id "XLOC_237043"; transcript_id "Novel_003322"; exon_number "3"; gene_type "
Dt_chr9	Cufflinks	exon	48384341	48384377	.	-	.	gene_id "XLOC_237043"; transcript_id "Novel_003323"; exon_number "1"; gene_type "
Dt_chr9	Cufflinks	exon	48384524	48385626	.	-	.	gene_id "XLOC_237043"; transcript_id "Novel_003323"; exon_number "2"; gene_type "
Dt_chr9	Cufflinks	exon	48988501	48989157	.	-	.	gene_id "XLOC_237099"; transcript_id "Novel_003324"; exon_number "1"; gene_type "
Dt_chr9	Cufflinks	exon	48989272	48989329	.	-	.	gene_id "XLOC_237099"; transcript_id "Novel_003324"; exon_number "2"; gene_type "
Dt_chr9	Cufflinks	exon	48989935	48989971	.	-	.	gene_id "XLOC_237099"; transcript_id "Novel_003324"; exon_number "3"; gene_type "
Dt_chr9	Cufflinks	exon	48990096	48990895	.	-	.	gene_id "XLOC_237099"; transcript_id "Novel_003324"; exon_number "4"; gene_type "

Dt_chr9	Cufflinks	exon	48991426	48991467	.	-	.	gene_id "XLOC_237099"; transcript_id "Novel_003324"; exon_number "5"; gene_type "
Dt_chr9	Cufflinks	exon	48991696	48992089	.	-	.	gene_id "XLOC_237099"; transcript_id "Novel_003324"; exon_number "6"; gene_type "
Dt_chr9	Cufflinks	exon	50233780	50236428	.	-	.	gene_id "XLOC_237268"; transcript_id "Novel_003325"; exon_number "1"; gene_type "
Dt_chr9	Cufflinks	exon	50237560	50237608	.	-	.	gene_id "XLOC_237268"; transcript_id "Novel_003325"; exon_number "2"; gene_type "
Dt_chr9	Cufflinks	exon	50238601	50238798	.	-	.	gene_id "XLOC_237268"; transcript_id "Novel_003325"; exon_number "3"; gene_type "
Dt_chr9	Cufflinks	exon	50267478	50268375	.	-	.	gene_id "XLOC_237273"; transcript_id "Novel_003326"; exon_number "1"; gene_type "
Dt_chr9	Cufflinks	exon	50590644	50590906	.	-	.	gene_id "XLOC_237316"; transcript_id "Novel_003327"; exon_number "1"; gene_type "
Dt_chr9	Cufflinks	exon	50591012	50591138	.	-	.	gene_id "XLOC_237316"; transcript_id "Novel_003327"; exon_number "2"; gene_type "
Dt_chr9	Cufflinks	exon	50591227	50591382	.	-	.	gene_id "XLOC_237316"; transcript_id "Novel_003327"; exon_number "3"; gene_type "
Dt_chr9	Cufflinks	exon	50592114	50592305	.	-	.	gene_id "XLOC_237316"; transcript_id "Novel_003327"; exon_number "4"; gene_type "
Dt_chr9	Cufflinks	exon	53056622	53057278	.	-	.	gene_id "XLOC_237794"; transcript_id "Novel_003328"; exon_number "1"; gene_type "
Dt_chr9	Cufflinks	exon	53057906	53057993	.	-	.	gene_id "XLOC_237794"; transcript_id "Novel_003328"; exon_number "2"; gene_type "
Dt_chr9	Cufflinks	exon	53058223	53058630	.	-	.	gene_id "XLOC_237794"; transcript_id "Novel_003328"; exon_number "3"; gene_type "
Dt_chr9	Cufflinks	exon	53056764	53057278	.	-	.	gene_id "XLOC_237794"; transcript_id "Novel_003329"; exon_number "1"; gene_type "
Dt_chr9	Cufflinks	exon	53057906	53057993	.	-	.	gene_id "XLOC_237794"; transcript_id "Novel_003329"; exon_number "2"; gene_type "
Dt_chr9	Cufflinks	exon	53058223	53058410	.	-	.	gene_id "XLOC_237794"; transcript_id "Novel_003329"; exon_number "3"; gene_type "
Dt_chr9	Cufflinks	exon	53058559	53059043	.	-	.	gene_id "XLOC_237794"; transcript_id "Novel_003329"; exon_number "4"; gene_type "
Dt_chr9	Cufflinks	exon	53059103	53059464	.	-	.	gene_id "XLOC_237794"; transcript_id "Novel_003330"; exon_number "1"; gene_type "
Dt_chr9	Cufflinks	exon	53059925	53060390	.	-	.	gene_id "XLOC_237794"; transcript_id "Novel_003330"; exon_number "2"; gene_type "
Dt_chr9	Cufflinks	exon	53059609	53060009	.	-	.	gene_id "XLOC_237794"; transcript_id "Novel_003331"; exon_number "1"; gene_type "
Dt_chr9	Cufflinks	exon	53060087	53060390	.	-	.	gene_id "XLOC_237794"; transcript_id "Novel_003331"; exon_number "2"; gene_type "
Dt_chr9	Cufflinks	exon	53850654	53850678	.	-	.	gene_id "XLOC_237944"; transcript_id "Novel_003332"; exon_number "1"; gene_type "
Dt_chr9	Cufflinks	exon	53850760	53851118	.	-	.	gene_id "XLOC_237944"; transcript_id "Novel_003332"; exon_number "2"; gene_type "
Dt_chr9	Cufflinks	exon	53851749	53851847	.	-	.	gene_id "XLOC_237944"; transcript_id "Novel_003332"; exon_number "3"; gene_type "
Dt_chr9	Cufflinks	exon	53852007	53852169	.	-	.	gene_id "XLOC_237944"; transcript_id "Novel_003332"; exon_number "4"; gene_type "
Dt_chr9	Cufflinks	exon	53852278	53852356	.	-	.	gene_id "XLOC_237944"; transcript_id "Novel_003332"; exon_number "5"; gene_type "
Dt_chr9	Cufflinks	exon	53852561	53853926	.	-	.	gene_id "XLOC_237944"; transcript_id "Novel_003332"; exon_number "6"; gene_type "
Dt_chr9	Cufflinks	exon	53850654	53850665	.	-	.	gene_id "XLOC_237944"; transcript_id "Novel_003333"; exon_number "1"; gene_type "
Dt_chr9	Cufflinks	exon	53850760	53851118	.	-	.	gene_id "XLOC_237944"; transcript_id "Novel_003333"; exon_number "2"; gene_type "
Dt_chr9	Cufflinks	exon	53851749	53851847	.	-	.	gene_id "XLOC_237944"; transcript_id "Novel_003333"; exon_number "3"; gene_type "
Dt_chr9	Cufflinks	exon	53852007	53852169	.	-	.	gene_id "XLOC_237944"; transcript_id "Novel_003333"; exon_number "4"; gene_type "
Dt_chr9	Cufflinks	exon	53852278	53852356	.	-	.	gene_id "XLOC_237944"; transcript_id "Novel_003333"; exon_number "5"; gene_type "
Dt_chr9	Cufflinks	exon	53852561	53853926	.	-	.	gene_id "XLOC_237944"; transcript_id "Novel_003333"; exon_number "6"; gene_type "
Dt_chr9	Cufflinks	exon	53850654	53851118	.	-	.	gene_id "XLOC_237944"; transcript_id "Novel_003334"; exon_number "1"; gene_type "
Dt_chr9	Cufflinks	exon	53851749	53851847	.	-	.	gene_id "XLOC_237944"; transcript_id "Novel_003334"; exon_number "2"; gene_type "

Dt_chr9	Cufflinks	exon	53852007	53852169	.	-	.	gene_id "XLOC_237944"; transcript_id "Novel_003334"; exon_number "3"; gene_type "
Dt_chr9	Cufflinks	exon	53852278	53852356	.	-	.	gene_id "XLOC_237944"; transcript_id "Novel_003334"; exon_number "4"; gene_type "
Dt_chr9	Cufflinks	exon	53852561	53853926	.	-	.	gene_id "XLOC_237944"; transcript_id "Novel_003334"; exon_number "5"; gene_type "
Dt_chr9	Cufflinks	exon	53898317	53898370	.	-	.	gene_id "XLOC_237948"; transcript_id "Novel_003335"; exon_number "1"; gene_type "
Dt_chr9	Cufflinks	exon	53898554	53898625	.	-	.	gene_id "XLOC_237948"; transcript_id "Novel_003335"; exon_number "2"; gene_type "
Dt_chr9	Cufflinks	exon	53898758	53898885	.	-	.	gene_id "XLOC_237948"; transcript_id "Novel_003335"; exon_number "3"; gene_type "
Dt_chr9	Cufflinks	exon	53898972	53899088	.	-	.	gene_id "XLOC_237948"; transcript_id "Novel_003335"; exon_number "4"; gene_type "
Dt_chr9	Cufflinks	exon	53899233	53899353	.	-	.	gene_id "XLOC_237948"; transcript_id "Novel_003335"; exon_number "5"; gene_type "
Dt_chr9	Cufflinks	exon	53899451	53899776	.	-	.	gene_id "XLOC_237948"; transcript_id "Novel_003335"; exon_number "6"; gene_type "
Dt_chr9	Cufflinks	exon	53899904	53900137	.	-	.	gene_id "XLOC_237948"; transcript_id "Novel_003335"; exon_number "7"; gene_type "
Dt_chr9	Cufflinks	exon	53898545	53898625	.	-	.	gene_id "XLOC_237948"; transcript_id "Novel_003336"; exon_number "1"; gene_type "
Dt_chr9	Cufflinks	exon	53898758	53898885	.	-	.	gene_id "XLOC_237948"; transcript_id "Novel_003336"; exon_number "2"; gene_type "
Dt_chr9	Cufflinks	exon	53898972	53899080	.	-	.	gene_id "XLOC_237948"; transcript_id "Novel_003336"; exon_number "3"; gene_type "
Dt_chr9	Cufflinks	exon	53899233	53899353	.	-	.	gene_id "XLOC_237948"; transcript_id "Novel_003336"; exon_number "4"; gene_type "
Dt_chr9	Cufflinks	exon	53899451	53899776	.	-	.	gene_id "XLOC_237948"; transcript_id "Novel_003336"; exon_number "5"; gene_type "
Dt_chr9	Cufflinks	exon	53899904	53900139	.	-	.	gene_id "XLOC_237948"; transcript_id "Novel_003336"; exon_number "6"; gene_type "
Dt_chr9	Cufflinks	exon	53932696	53933228	.	-	.	gene_id "XLOC_237958"; transcript_id "Novel_003337"; exon_number "1"; gene_type "
Dt_chr9	Cufflinks	exon	53959507	53960417	.	-	.	gene_id "XLOC_237962"; transcript_id "Novel_003338"; exon_number "1"; gene_type "
Dt_chr9	Cufflinks	exon	53960811	53960949	.	-	.	gene_id "XLOC_237962"; transcript_id "Novel_003338"; exon_number "2"; gene_type "
Dt_chr9	Cufflinks	exon	53961511	53961646	.	-	.	gene_id "XLOC_237962"; transcript_id "Novel_003338"; exon_number "3"; gene_type "
Dt_chr9	Cufflinks	exon	53962223	53962486	.	-	.	gene_id "XLOC_237962"; transcript_id "Novel_003338"; exon_number "4"; gene_type "
Dt_chr9	Cufflinks	exon	53959804	53959870	.	-	.	gene_id "XLOC_237962"; transcript_id "Novel_003339"; exon_number "1"; gene_type "
Dt_chr9	Cufflinks	exon	53960266	53960417	.	-	.	gene_id "XLOC_237962"; transcript_id "Novel_003339"; exon_number "2"; gene_type "
Dt_chr9	Cufflinks	exon	53960811	53960939	.	-	.	gene_id "XLOC_237962"; transcript_id "Novel_003339"; exon_number "3"; gene_type "
Dt_chr9	Cufflinks	exon	53961511	53961646	.	-	.	gene_id "XLOC_237962"; transcript_id "Novel_003339"; exon_number "4"; gene_type "
Dt_chr9	Cufflinks	exon	53962223	53962463	.	-	.	gene_id "XLOC_237962"; transcript_id "Novel_003339"; exon_number "5"; gene_type "
Dt_chr9	Cufflinks	exon	54112211	54112654	.	-	.	gene_id "XLOC_237987"; transcript_id "Novel_003340"; exon_number "1"; gene_type "
Dt_chr9	Cufflinks	exon	54112777	54112948	.	-	.	gene_id "XLOC_237987"; transcript_id "Novel_003340"; exon_number "2"; gene_type "
Dt_chr9	Cufflinks	exon	54113146	54113329	.	-	.	gene_id "XLOC_237987"; transcript_id "Novel_003340"; exon_number "3"; gene_type "
Dt_chr9	Cufflinks	exon	54113412	54113657	.	-	.	gene_id "XLOC_237987"; transcript_id "Novel_003340"; exon_number "4"; gene_type "
Dt_chr9	Cufflinks	exon	54113958	54114689	.	-	.	gene_id "XLOC_237987"; transcript_id "Novel_003340"; exon_number "5"; gene_type "
Dt_chr9	Cufflinks	exon	54112300	54112654	.	-	.	gene_id "XLOC_237987"; transcript_id "Novel_003341"; exon_number "1"; gene_type "
Dt_chr9	Cufflinks	exon	54112777	54112948	.	-	.	gene_id "XLOC_237987"; transcript_id "Novel_003341"; exon_number "2"; gene_type "
Dt_chr9	Cufflinks	exon	54113146	54113657	.	-	.	gene_id "XLOC_237987"; transcript_id "Novel_003341"; exon_number "3"; gene_type "
Dt_chr9	Cufflinks	exon	54113958	54114676	.	-	.	gene_id "XLOC_237987"; transcript_id "Novel_003341"; exon_number "4"; gene_type "

Dt_chr9	Cufflinks	exon	54132682	54133551	.	-	.	gene_id "XLOC_237989"; transcript_id "Novel_003342"; exon_number "1"; gene_type "
Dt_chr9	Cufflinks	exon	54133723	54134048	.	-	.	gene_id "XLOC_237989"; transcript_id "Novel_003342"; exon_number "2"; gene_type "
Dt_chr9	Cufflinks	exon	54134179	54134278	.	-	.	gene_id "XLOC_237989"; transcript_id "Novel_003342"; exon_number "3"; gene_type "
Dt_chr9	Cufflinks	exon	54134373	54134403	.	-	.	gene_id "XLOC_237989"; transcript_id "Novel_003342"; exon_number "4"; gene_type "
Dt_chr9	Cufflinks	exon	54134514	54134611	.	-	.	gene_id "XLOC_237989"; transcript_id "Novel_003342"; exon_number "5"; gene_type "
Dt_chr9	Cufflinks	exon	54134705	54135144	.	-	.	gene_id "XLOC_237989"; transcript_id "Novel_003342"; exon_number "6"; gene_type "
Dt_chr9	Cufflinks	exon	54132682	54133551	.	-	.	gene_id "XLOC_237989"; transcript_id "Novel_003343"; exon_number "1"; gene_type "
Dt_chr9	Cufflinks	exon	54133723	54133866	.	-	.	gene_id "XLOC_237989"; transcript_id "Novel_003343"; exon_number "2"; gene_type "
Dt_chr9	Cufflinks	exon	54133978	54134048	.	-	.	gene_id "XLOC_237989"; transcript_id "Novel_003343"; exon_number "3"; gene_type "
Dt_chr9	Cufflinks	exon	54134179	54134278	.	-	.	gene_id "XLOC_237989"; transcript_id "Novel_003343"; exon_number "4"; gene_type "
Dt_chr9	Cufflinks	exon	54134373	54134403	.	-	.	gene_id "XLOC_237989"; transcript_id "Novel_003343"; exon_number "5"; gene_type "
Dt_chr9	Cufflinks	exon	54134514	54134611	.	-	.	gene_id "XLOC_237989"; transcript_id "Novel_003343"; exon_number "6"; gene_type "
Dt_chr9	Cufflinks	exon	54134705	54135144	.	-	.	gene_id "XLOC_237989"; transcript_id "Novel_003343"; exon_number "7"; gene_type "
Dt_chr9	Cufflinks	exon	54132689	54133551	.	-	.	gene_id "XLOC_237989"; transcript_id "Novel_003344"; exon_number "1"; gene_type "
Dt_chr9	Cufflinks	exon	54133723	54134048	.	-	.	gene_id "XLOC_237989"; transcript_id "Novel_003344"; exon_number "2"; gene_type "
Dt_chr9	Cufflinks	exon	54134179	54134278	.	-	.	gene_id "XLOC_237989"; transcript_id "Novel_003344"; exon_number "3"; gene_type "
Dt_chr9	Cufflinks	exon	54134373	54134403	.	-	.	gene_id "XLOC_237989"; transcript_id "Novel_003344"; exon_number "4"; gene_type "
Dt_chr9	Cufflinks	exon	54134514	54135023	.	-	.	gene_id "XLOC_237989"; transcript_id "Novel_003344"; exon_number "5"; gene_type "
Dt_chr9	Cufflinks	exon	54934320	54935522	.	-	.	gene_id "XLOC_238139"; transcript_id "Novel_003345"; exon_number "1"; gene_type "
Dt_chr9	Cufflinks	exon	54935631	54936837	.	-	.	gene_id "XLOC_238139"; transcript_id "Novel_003345"; exon_number "2"; gene_type "
Dt_chr9	Cufflinks	exon	55057762	55058322	.	-	.	gene_id "XLOC_238155"; transcript_id "Novel_003346"; exon_number "1"; gene_type "
Dt_chr9	Cufflinks	exon	55058914	55059637	.	-	.	gene_id "XLOC_238155"; transcript_id "Novel_003346"; exon_number "2"; gene_type "
Dt_chr9	Cufflinks	exon	55735848	55736747	.	-	.	gene_id "XLOC_238283"; transcript_id "Novel_003347"; exon_number "1"; gene_type "
Dt_chr9	Cufflinks	exon	55736838	55737047	.	-	.	gene_id "XLOC_238283"; transcript_id "Novel_003347"; exon_number "2"; gene_type "
Dt_chr9	Cufflinks	exon	57747703	57748213	.	-	.	gene_id "XLOC_238625"; transcript_id "Novel_003348"; exon_number "1"; gene_type "
Dt_chr9	Cufflinks	exon	57748320	57749084	.	-	.	gene_id "XLOC_238625"; transcript_id "Novel_003348"; exon_number "2"; gene_type "
Dt_chr9	Cufflinks	exon	57747703	57748213	.	-	.	gene_id "XLOC_238625"; transcript_id "Novel_003349"; exon_number "1"; gene_type "
Dt_chr9	Cufflinks	exon	57748339	57748419	.	-	.	gene_id "XLOC_238625"; transcript_id "Novel_003349"; exon_number "2"; gene_type "
Dt_chr9	Cufflinks	exon	57748535	57749084	.	-	.	gene_id "XLOC_238625"; transcript_id "Novel_003349"; exon_number "3"; gene_type "
Dt_chr9	Cufflinks	exon	57747736	57748213	.	-	.	gene_id "XLOC_238625"; transcript_id "Novel_003350"; exon_number "1"; gene_type "
Dt_chr9	Cufflinks	exon	57748320	57748419	.	-	.	gene_id "XLOC_238625"; transcript_id "Novel_003350"; exon_number "2"; gene_type "
Dt_chr9	Cufflinks	exon	57748535	57749113	.	-	.	gene_id "XLOC_238625"; transcript_id "Novel_003350"; exon_number "3"; gene_type "
Dt_chr9	Cufflinks	exon	57757543	57758494	.	-	.	gene_id "XLOC_238628"; transcript_id "Novel_003351"; exon_number "1"; gene_type "
Dt_chr9	Cufflinks	exon	57759034	57759132	.	-	.	gene_id "XLOC_238628"; transcript_id "Novel_003351"; exon_number "2"; gene_type "
Dt_chr9	Cufflinks	exon	57759272	57759352	.	-	.	gene_id "XLOC_238628"; transcript_id "Novel_003351"; exon_number "3"; gene_type "

Dt_chr9	Cufflinks	exon	57760180	57760268	.	-	.	gene_id "XLOC_238628"; transcript_id "Novel_003351"; exon_number "4"; gene_type "
Dt_chr9	Cufflinks	exon	57760480	57760530	.	-	.	gene_id "XLOC_238628"; transcript_id "Novel_003351"; exon_number "5"; gene_type "
Dt_chr9	Cufflinks	exon	57760626	57760739	.	-	.	gene_id "XLOC_238628"; transcript_id "Novel_003351"; exon_number "6"; gene_type "
Dt_chr9	Cufflinks	exon	57760881	57761119	.	-	.	gene_id "XLOC_238628"; transcript_id "Novel_003351"; exon_number "7"; gene_type "
Dt_chr9	Cufflinks	exon	57757543	57758494	.	-	.	gene_id "XLOC_238628"; transcript_id "Novel_003352"; exon_number "1"; gene_type "
Dt_chr9	Cufflinks	exon	57759034	57759132	.	-	.	gene_id "XLOC_238628"; transcript_id "Novel_003352"; exon_number "2"; gene_type "
Dt_chr9	Cufflinks	exon	57759272	57759352	.	-	.	gene_id "XLOC_238628"; transcript_id "Novel_003352"; exon_number "3"; gene_type "
Dt_chr9	Cufflinks	exon	57760180	57760271	.	-	.	gene_id "XLOC_238628"; transcript_id "Novel_003352"; exon_number "4"; gene_type "
Dt_chr9	Cufflinks	exon	57760480	57760530	.	-	.	gene_id "XLOC_238628"; transcript_id "Novel_003352"; exon_number "5"; gene_type "
Dt_chr9	Cufflinks	exon	57760626	57760739	.	-	.	gene_id "XLOC_238628"; transcript_id "Novel_003352"; exon_number "6"; gene_type "
Dt_chr9	Cufflinks	exon	57760881	57761119	.	-	.	gene_id "XLOC_238628"; transcript_id "Novel_003352"; exon_number "7"; gene_type "
Dt_chr9	Cufflinks	exon	57765928	57766776	.	-	.	gene_id "XLOC_238632"; transcript_id "Novel_003353"; exon_number "1"; gene_type "
Dt_chr9	Cufflinks	exon	57830768	57832825	.	-	.	gene_id "XLOC_238647"; transcript_id "Novel_003354"; exon_number "1"; gene_type "
Dt_chr9	Cufflinks	exon	59963402	59964565	.	-	.	gene_id "XLOC_239007"; transcript_id "Novel_003355"; exon_number "1"; gene_type "
Dt_chr9	Cufflinks	exon	59965608	59965675	.	-	.	gene_id "XLOC_239007"; transcript_id "Novel_003355"; exon_number "2"; gene_type "
Dt_chr9	Cufflinks	exon	59968016	59968055	.	-	.	gene_id "XLOC_239007"; transcript_id "Novel_003355"; exon_number "3"; gene_type "
Dt_chr9	Cufflinks	exon	59969470	59970346	.	-	.	gene_id "XLOC_239007"; transcript_id "Novel_003355"; exon_number "4"; gene_type "
Dt_chr9	Cufflinks	exon	61542038	61543196	.	-	.	gene_id "XLOC_239276"; transcript_id "Novel_003356"; exon_number "1"; gene_type "
Dt_chr9	Cufflinks	exon	61544908	61544953	.	-	.	gene_id "XLOC_239276"; transcript_id "Novel_003356"; exon_number "2"; gene_type "
Dt_chr9	Cufflinks	exon	61545083	61545238	.	-	.	gene_id "XLOC_239276"; transcript_id "Novel_003356"; exon_number "3"; gene_type "
Dt_chr9	Cufflinks	exon	61545338	61545425	.	-	.	gene_id "XLOC_239276"; transcript_id "Novel_003356"; exon_number "4"; gene_type "
Dt_chr9	Cufflinks	exon	61542038	61543196	.	-	.	gene_id "XLOC_239276"; transcript_id "Novel_003357"; exon_number "1"; gene_type "
Dt_chr9	Cufflinks	exon	61544908	61544953	.	-	.	gene_id "XLOC_239276"; transcript_id "Novel_003357"; exon_number "2"; gene_type "
Dt_chr9	Cufflinks	exon	61545133	61545238	.	-	.	gene_id "XLOC_239276"; transcript_id "Novel_003357"; exon_number "3"; gene_type "
Dt_chr9	Cufflinks	exon	61545338	61545425	.	-	.	gene_id "XLOC_239276"; transcript_id "Novel_003357"; exon_number "4"; gene_type "
Dt_chr9	Cufflinks	exon	61542038	61543196	.	-	.	gene_id "XLOC_239276"; transcript_id "Novel_003358"; exon_number "1"; gene_type "
Dt_chr9	Cufflinks	exon	61544908	61544953	.	-	.	gene_id "XLOC_239276"; transcript_id "Novel_003358"; exon_number "2"; gene_type "
Dt_chr9	Cufflinks	exon	61545083	61545261	.	-	.	gene_id "XLOC_239276"; transcript_id "Novel_003358"; exon_number "3"; gene_type "
Dt_chr9	Cufflinks	exon	61545338	61545427	.	-	.	gene_id "XLOC_239276"; transcript_id "Novel_003358"; exon_number "4"; gene_type "
Dt_chr9	Cufflinks	exon	61542038	61543196	.	-	.	gene_id "XLOC_239276"; transcript_id "Novel_003359"; exon_number "1"; gene_type "
Dt_chr9	Cufflinks	exon	61544908	61544953	.	-	.	gene_id "XLOC_239276"; transcript_id "Novel_003359"; exon_number "2"; gene_type "
Dt_chr9	Cufflinks	exon	61545338	61545427	.	-	.	gene_id "XLOC_239276"; transcript_id "Novel_003359"; exon_number "3"; gene_type "
Dt_chr9	Cufflinks	exon	61542038	61543196	.	-	.	gene_id "XLOC_239276"; transcript_id "Novel_003360"; exon_number "1"; gene_type "
Dt_chr9	Cufflinks	exon	61544908	61544953	.	-	.	gene_id "XLOC_239276"; transcript_id "Novel_003360"; exon_number "2"; gene_type "
Dt_chr9	Cufflinks	exon	61545295	61545427	.	-	.	gene_id "XLOC_239276"; transcript_id "Novel_003360"; exon_number "3"; gene_type "

Dt_chr9	Cufflinks	exon	61542045	61543196	.	-	.	gene_id "XLOC_239276"; transcript_id "Novel_003361"; exon_number "1"; gene_type "
Dt_chr9	Cufflinks	exon	61544908	61544953	.	-	.	gene_id "XLOC_239276"; transcript_id "Novel_003361"; exon_number "2"; gene_type "
Dt_chr9	Cufflinks	exon	61545083	61545417	.	-	.	gene_id "XLOC_239276"; transcript_id "Novel_003361"; exon_number "3"; gene_type "
Dt_chr9	Cufflinks	exon	61542152	61543196	.	-	.	gene_id "XLOC_239276"; transcript_id "Novel_003362"; exon_number "1"; gene_type "
Dt_chr9	Cufflinks	exon	61544908	61544953	.	-	.	gene_id "XLOC_239276"; transcript_id "Novel_003362"; exon_number "2"; gene_type "
Dt_chr9	Cufflinks	exon	61545133	61545261	.	-	.	gene_id "XLOC_239276"; transcript_id "Novel_003362"; exon_number "3"; gene_type "
Dt_chr9	Cufflinks	exon	61545338	61545422	.	-	.	gene_id "XLOC_239276"; transcript_id "Novel_003362"; exon_number "4"; gene_type "
Dt_chr9	Cufflinks	exon	61760724	61761235	.	-	.	gene_id "XLOC_239317"; transcript_id "Novel_003363"; exon_number "1"; gene_type "
Dt_chr9	Cufflinks	exon	61761425	61761627	.	-	.	gene_id "XLOC_239317"; transcript_id "Novel_003363"; exon_number "2"; gene_type "
Dt_chr9	Cufflinks	exon	61761809	61761916	.	-	.	gene_id "XLOC_239317"; transcript_id "Novel_003363"; exon_number "3"; gene_type "
Dt_chr9	Cufflinks	exon	61760724	61761627	.	-	.	gene_id "XLOC_239317"; transcript_id "Novel_003364"; exon_number "1"; gene_type "
Dt_chr9	Cufflinks	exon	61761809	61761916	.	-	.	gene_id "XLOC_239317"; transcript_id "Novel_003364"; exon_number "2"; gene_type "
Dt_chr9	Cufflinks	exon	64151679	64152213	.	-	.	gene_id "XLOC_239804"; transcript_id "Novel_003365"; exon_number "1"; gene_type "
Dt_chr9	Cufflinks	exon	64152466	64153189	.	-	.	gene_id "XLOC_239804"; transcript_id "Novel_003365"; exon_number "2"; gene_type "
Dt_chr9	Cufflinks	exon	64245306	64246097	.	-	.	gene_id "XLOC_239818"; transcript_id "Novel_003366"; exon_number "1"; gene_type "
Dt_chr9	Cufflinks	exon	64246324	64246412	.	-	.	gene_id "XLOC_239818"; transcript_id "Novel_003366"; exon_number "2"; gene_type "
Dt_chr9	Cufflinks	exon	64246503	64246955	.	-	.	gene_id "XLOC_239818"; transcript_id "Novel_003366"; exon_number "3"; gene_type "
Dt_chr9	Cufflinks	exon	64434052	64434455	.	-	.	gene_id "XLOC_239849"; transcript_id "Novel_003367"; exon_number "1"; gene_type "
Dt_chr9	Cufflinks	exon	64434555	64435191	.	-	.	gene_id "XLOC_239849"; transcript_id "Novel_003367"; exon_number "2"; gene_type "
Dt_chr9	Cufflinks	exon	64910195	64911099	.	-	.	gene_id "XLOC_239951"; transcript_id "Novel_003368"; exon_number "1"; gene_type "
Dt_chr9	Cufflinks	exon	64911424	64911778	.	-	.	gene_id "XLOC_239951"; transcript_id "Novel_003368"; exon_number "2"; gene_type "
Dt_chr9	Cufflinks	exon	64911896	64911991	.	-	.	gene_id "XLOC_239951"; transcript_id "Novel_003368"; exon_number "3"; gene_type "
Dt_chr9	Cufflinks	exon	64912117	64912177	.	-	.	gene_id "XLOC_239951"; transcript_id "Novel_003368"; exon_number "4"; gene_type "
Dt_chr9	Cufflinks	exon	64912297	64912488	.	-	.	gene_id "XLOC_239951"; transcript_id "Novel_003368"; exon_number "5"; gene_type "
Dt_chr9	Cufflinks	exon	65639890	65640099	.	-	.	gene_id "XLOC_240080"; transcript_id "Novel_003369"; exon_number "1"; gene_type "
Dt_chr9	Cufflinks	exon	65640674	65640754	.	-	.	gene_id "XLOC_240080"; transcript_id "Novel_003369"; exon_number "2"; gene_type "
Dt_chr9	Cufflinks	exon	67865770	67866609	.	-	.	gene_id "XLOC_240489"; transcript_id "Novel_003370"; exon_number "1"; gene_type "
Dt_chr9	Cufflinks	exon	67867667	67867793	.	-	.	gene_id "XLOC_240489"; transcript_id "Novel_003370"; exon_number "2"; gene_type "
Dt_chr9	Cufflinks	exon	67868345	67868803	.	-	.	gene_id "XLOC_240489"; transcript_id "Novel_003370"; exon_number "3"; gene_type "
Dt_chr9	Cufflinks	exon	67867039	67867793	.	-	.	gene_id "XLOC_240489"; transcript_id "Novel_003371"; exon_number "1"; gene_type "
Dt_chr9	Cufflinks	exon	67868345	67868803	.	-	.	gene_id "XLOC_240489"; transcript_id "Novel_003371"; exon_number "2"; gene_type "
Dt_chr9	Cufflinks	exon	68136385	68136773	.	-	.	gene_id "XLOC_240515"; transcript_id "Novel_003372"; exon_number "1"; gene_type "
Dt_chr9	Cufflinks	exon	68137576	68137740	.	-	.	gene_id "XLOC_240515"; transcript_id "Novel_003372"; exon_number "2"; gene_type "
Dt_chr9	Cufflinks	exon	68138375	68138503	.	-	.	gene_id "XLOC_240515"; transcript_id "Novel_003372"; exon_number "3"; gene_type "
Dt_chr9	Cufflinks	exon	68139499	68139594	.	-	.	gene_id "XLOC_240515"; transcript_id "Novel_003372"; exon_number "4"; gene_type "

Dt_chr9	Cufflinks	exon	68139704	68139760	.	-	.	gene_id "XLOC_240515"; transcript_id "Novel_003372"; exon_number "5"; gene_type "
Dt_chr9	Cufflinks	exon	68139848	68139991	.	-	.	gene_id "XLOC_240515"; transcript_id "Novel_003372"; exon_number "6"; gene_type "
Dt_chr9	Cufflinks	exon	68145530	68145604	.	-	.	gene_id "XLOC_240515"; transcript_id "Novel_003372"; exon_number "7"; gene_type "
Dt_chr9	Cufflinks	exon	68146218	68146370	.	-	.	gene_id "XLOC_240515"; transcript_id "Novel_003372"; exon_number "8"; gene_type "
Dt_chr9	Cufflinks	exon	68146441	68146518	.	-	.	gene_id "XLOC_240515"; transcript_id "Novel_003372"; exon_number "9"; gene_type "
Dt_chr9	Cufflinks	exon	68146608	68146763	.	-	.	gene_id "XLOC_240515"; transcript_id "Novel_003372"; exon_number "10"; gene_type
Dt_chr9	Cufflinks	exon	68146869	68147027	.	-	.	gene_id "XLOC_240515"; transcript_id "Novel_003372"; exon_number "11"; gene_type
Dt_chr9	Cufflinks	exon	68147119	68147723	.	-	.	gene_id "XLOC_240515"; transcript_id "Novel_003372"; exon_number "12"; gene_type
Dt_chr9	Cufflinks	exon	68144387	68145604	.	-	.	gene_id "XLOC_240515"; transcript_id "Novel_003373"; exon_number "1"; gene_type "
Dt_chr9	Cufflinks	exon	68146218	68146518	.	-	.	gene_id "XLOC_240515"; transcript_id "Novel_003373"; exon_number "2"; gene_type "
Dt_chr9	Cufflinks	exon	68146608	68147884	.	-	.	gene_id "XLOC_240515"; transcript_id "Novel_003373"; exon_number "3"; gene_type "
Dt_chr9	Cufflinks	exon	68144388	68145604	.	-	.	gene_id "XLOC_240515"; transcript_id "Novel_003374"; exon_number "1"; gene_type "
Dt_chr9	Cufflinks	exon	68146218	68146370	.	-	.	gene_id "XLOC_240515"; transcript_id "Novel_003374"; exon_number "2"; gene_type "
Dt_chr9	Cufflinks	exon	68146441	68146518	.	-	.	gene_id "XLOC_240515"; transcript_id "Novel_003374"; exon_number "3"; gene_type "
Dt_chr9	Cufflinks	exon	68146608	68146763	.	-	.	gene_id "XLOC_240515"; transcript_id "Novel_003374"; exon_number "4"; gene_type "
Dt_chr9	Cufflinks	exon	68146869	68147027	.	-	.	gene_id "XLOC_240515"; transcript_id "Novel_003374"; exon_number "5"; gene_type "
Dt_chr9	Cufflinks	exon	68147119	68147850	.	-	.	gene_id "XLOC_240515"; transcript_id "Novel_003374"; exon_number "6"; gene_type "
Dt_chr9	Cufflinks	exon	68298646	68299122	.	-	.	gene_id "XLOC_240555"; transcript_id "Novel_003375"; exon_number "1"; gene_type "
Dt_chr9	Cufflinks	exon	68299541	68299796	.	-	.	gene_id "XLOC_240555"; transcript_id "Novel_003375"; exon_number "2"; gene_type "
Dt_chr9	Cufflinks	exon	68301137	68302231	.	-	.	gene_id "XLOC_240555"; transcript_id "Novel_003375"; exon_number "3"; gene_type "
Dt_chr9	Cufflinks	exon	68337650	68338120	.	-	.	gene_id "XLOC_240568"; transcript_id "Novel_003376"; exon_number "1"; gene_type "
Dt_chr9	Cufflinks	exon	68338218	68338328	.	-	.	gene_id "XLOC_240568"; transcript_id "Novel_003376"; exon_number "2"; gene_type "
Dt_chr9	Cufflinks	exon	68338581	68338980	.	-	.	gene_id "XLOC_240568"; transcript_id "Novel_003376"; exon_number "3"; gene_type "
Dt_chr9	Cufflinks	exon	68337919	68338120	.	-	.	gene_id "XLOC_240568"; transcript_id "Novel_003377"; exon_number "1"; gene_type "
Dt_chr9	Cufflinks	exon	68338218	68338328	.	-	.	gene_id "XLOC_240568"; transcript_id "Novel_003377"; exon_number "2"; gene_type "
Dt_chr9	Cufflinks	exon	68338581	68338683	.	-	.	gene_id "XLOC_240568"; transcript_id "Novel_003377"; exon_number "3"; gene_type "
Dt_chr9	Cufflinks	exon	68338896	68338960	.	-	.	gene_id "XLOC_240568"; transcript_id "Novel_003377"; exon_number "4"; gene_type "
Dt_chr9	Cufflinks	exon	68339074	68339168	.	-	.	gene_id "XLOC_240568"; transcript_id "Novel_003377"; exon_number "5"; gene_type "
Dt_chr9	Cufflinks	exon	68339105	68339162	.	-	.	gene_id "XLOC_240568"; transcript_id "Novel_003378"; exon_number "1"; gene_type "
Dt_chr9	Cufflinks	exon	68339293	68339469	.	-	.	gene_id "XLOC_240568"; transcript_id "Novel_003378"; exon_number "2"; gene_type "
Dt_chr9	Cufflinks	exon	68339737	68339886	.	-	.	gene_id "XLOC_240568"; transcript_id "Novel_003378"; exon_number "3"; gene_type "
Dt_chr9	Cufflinks	exon	68340415	68340625	.	-	.	gene_id "XLOC_240568"; transcript_id "Novel_003378"; exon_number "4"; gene_type "
Dt_chr9	Cufflinks	exon	68490228	68490921	.	-	.	gene_id "XLOC_240592"; transcript_id "Novel_003379"; exon_number "1"; gene_type "
Dt_chr9	Cufflinks	exon	68491003	68491175	.	-	.	gene_id "XLOC_240592"; transcript_id "Novel_003379"; exon_number "2"; gene_type "
Dt_chr9	Cufflinks	exon	68491274	68491864	.	-	.	gene_id "XLOC_240592"; transcript_id "Novel_003379"; exon_number "3"; gene_type "

Dt_chr9	Cufflinks	exon	68490576	68491175	.	-	.	gene_id "XLOC_240592"; transcript_id "Novel_003380"; exon_number "1"; gene_type "
Dt_chr9	Cufflinks	exon	68491274	68491854	.	-	.	gene_id "XLOC_240592"; transcript_id "Novel_003380"; exon_number "2"; gene_type "
Dt_chr9	Cufflinks	exon	69167677	69168213	.	-	.	gene_id "XLOC_240665"; transcript_id "Novel_003381"; exon_number "1"; gene_type "
Dt_chr9	Cufflinks	exon	69171855	69176249	.	-	.	gene_id "XLOC_240665"; transcript_id "Novel_003381"; exon_number "2"; gene_type "
Dt_chr9	Cufflinks	exon	69406371	69408175	.	-	.	gene_id "XLOC_240685"; transcript_id "Novel_003382"; exon_number "1"; gene_type "
Dt_chr9	Cufflinks	exon	69413188	69413355	.	-	.	gene_id "XLOC_240685"; transcript_id "Novel_003382"; exon_number "2"; gene_type "
Dt_chr9	Cufflinks	exon	69522109	69522323	.	-	.	gene_id "XLOC_240706"; transcript_id "Novel_003383"; exon_number "1"; gene_type "
scaffold1	Cufflinks	exon	147550	148463	.	+	.	gene_id "XLOC_241507"; transcript_id "Novel_003384"; exon_number "1"; gene_type "
scaffold1	Cufflinks	exon	149159	149354	.	+	.	gene_id "XLOC_241507"; transcript_id "Novel_003384"; exon_number "2"; gene_type "
scaffold1	Cufflinks	exon	150038	150162	.	+	.	gene_id "XLOC_241507"; transcript_id "Novel_003384"; exon_number "3"; gene_type "
scaffold1	Cufflinks	exon	150248	150406	.	+	.	gene_id "XLOC_241507"; transcript_id "Novel_003384"; exon_number "4"; gene_type "
scaffold1	Cufflinks	exon	150493	150983	.	+	.	gene_id "XLOC_241507"; transcript_id "Novel_003384"; exon_number "5"; gene_type "
scaffold1	Cufflinks	exon	148771	149354	.	+	.	gene_id "XLOC_241507"; transcript_id "Novel_003385"; exon_number "1"; gene_type "
scaffold1	Cufflinks	exon	150038	150162	.	+	.	gene_id "XLOC_241507"; transcript_id "Novel_003385"; exon_number "2"; gene_type "
scaffold1	Cufflinks	exon	150248	150406	.	+	.	gene_id "XLOC_241507"; transcript_id "Novel_003385"; exon_number "3"; gene_type "
scaffold1	Cufflinks	exon	150493	151016	.	+	.	gene_id "XLOC_241507"; transcript_id "Novel_003385"; exon_number "4"; gene_type "
scaffold1	Cufflinks	exon	330025	330786	.	+	.	gene_id "XLOC_241534"; transcript_id "Novel_003386"; exon_number "1"; gene_type "
scaffold1	Cufflinks	exon	332088	332423	.	+	.	gene_id "XLOC_241534"; transcript_id "Novel_003386"; exon_number "2"; gene_type "
scaffold1	Cufflinks	exon	400514	400717	.	+	.	gene_id "XLOC_241544"; transcript_id "Novel_003387"; exon_number "1"; gene_type "
scaffold1	Cufflinks	exon	400804	400904	.	+	.	gene_id "XLOC_241544"; transcript_id "Novel_003387"; exon_number "2"; gene_type "
scaffold1	Cufflinks	exon	401320	401435	.	+	.	gene_id "XLOC_241544"; transcript_id "Novel_003387"; exon_number "3"; gene_type "
scaffold1	Cufflinks	exon	401528	401627	.	+	.	gene_id "XLOC_241544"; transcript_id "Novel_003387"; exon_number "4"; gene_type "
scaffold1	Cufflinks	exon	401949	402095	.	+	.	gene_id "XLOC_241544"; transcript_id "Novel_003387"; exon_number "5"; gene_type "
scaffold1	Cufflinks	exon	402166	402226	.	+	.	gene_id "XLOC_241544"; transcript_id "Novel_003387"; exon_number "6"; gene_type "
scaffold1	Cufflinks	exon	402304	402401	.	+	.	gene_id "XLOC_241544"; transcript_id "Novel_003387"; exon_number "7"; gene_type "
scaffold1	Cufflinks	exon	402791	402933	.	+	.	gene_id "XLOC_241544"; transcript_id "Novel_003387"; exon_number "8"; gene_type "
scaffold1	Cufflinks	exon	403011	403094	.	+	.	gene_id "XLOC_241544"; transcript_id "Novel_003387"; exon_number "9"; gene_type "
scaffold1	Cufflinks	exon	403177	403266	.	+	.	gene_id "XLOC_241544"; transcript_id "Novel_003387"; exon_number "10"; gene_type
scaffold1	Cufflinks	exon	403374	403616	.	+	.	gene_id "XLOC_241544"; transcript_id "Novel_003387"; exon_number "11"; gene_type
scaffold1	Cufflinks	exon	806755	807156	.	+	.	gene_id "XLOC_241607"; transcript_id "Novel_003388"; exon_number "1"; gene_type "
scaffold1	Cufflinks	exon	807336	807476	.	+	.	gene_id "XLOC_241607"; transcript_id "Novel_003388"; exon_number "2"; gene_type "
scaffold1	Cufflinks	exon	807593	807751	.	+	.	gene_id "XLOC_241607"; transcript_id "Novel_003388"; exon_number "3"; gene_type "
scaffold1	Cufflinks	exon	808292	809699	.	+	.	gene_id "XLOC_241607"; transcript_id "Novel_003388"; exon_number "4"; gene_type "
scaffold1	Cufflinks	exon	1313643	1313809	.	+	.	gene_id "XLOC_241674"; transcript_id "Novel_003389"; exon_number "1"; gene_type "
scaffold1	Cufflinks	exon	1314975	1316167	.	+	.	gene_id "XLOC_241674"; transcript_id "Novel_003389"; exon_number "2"; gene_type "

scaffold1Cufflinks	exon	1940691	1940968	.	+	.	gene_id "XLOC_241783"; transcript_id "Novel_003390"; exon_number "1"; gene_type "
scaffold1Cufflinks	exon	1941825	1942073	.	+	.	gene_id "XLOC_241783"; transcript_id "Novel_003390"; exon_number "2"; gene_type "
scaffold1Cufflinks	exon	1943122	1943647	.	+	.	gene_id "XLOC_241783"; transcript_id "Novel_003390"; exon_number "3"; gene_type "
scaffold1Cufflinks	exon	1940777	1940968	.	+	.	gene_id "XLOC_241783"; transcript_id "Novel_003391"; exon_number "1"; gene_type "
scaffold1Cufflinks	exon	1941825	1942105	.	+	.	gene_id "XLOC_241783"; transcript_id "Novel_003391"; exon_number "2"; gene_type "
scaffold1Cufflinks	exon	1941623	1942073	.	+	.	gene_id "XLOC_241783"; transcript_id "Novel_003392"; exon_number "1"; gene_type "
scaffold1Cufflinks	exon	1943122	1943507	.	+	.	gene_id "XLOC_241783"; transcript_id "Novel_003392"; exon_number "2"; gene_type "
scaffold1Cufflinks	exon	482124	484540	.	-	.	gene_id "XLOC_241872"; transcript_id "Novel_003393"; exon_number "1"; gene_type "
scaffold1Cufflinks	exon	484665	484805	.	-	.	gene_id "XLOC_241872"; transcript_id "Novel_003393"; exon_number "2"; gene_type "
scaffold1Cufflinks	exon	484982	485401	.	-	.	gene_id "XLOC_241872"; transcript_id "Novel_003393"; exon_number "3"; gene_type "
scaffold1Cufflinks	exon	483260	483850	.	-	.	gene_id "XLOC_241872"; transcript_id "Novel_003394"; exon_number "1"; gene_type "
scaffold1Cufflinks	exon	484382	484540	.	-	.	gene_id "XLOC_241872"; transcript_id "Novel_003394"; exon_number "2"; gene_type "
scaffold1Cufflinks	exon	484665	484805	.	-	.	gene_id "XLOC_241872"; transcript_id "Novel_003394"; exon_number "3"; gene_type "
scaffold1Cufflinks	exon	484982	485382	.	-	.	gene_id "XLOC_241872"; transcript_id "Novel_003394"; exon_number "4"; gene_type "
scaffold1Cufflinks	exon	966900	967524	.	-	.	gene_id "XLOC_241938"; transcript_id "Novel_003395"; exon_number "1"; gene_type "
scaffold1Cufflinks	exon	968884	969613	.	-	.	gene_id "XLOC_241938"; transcript_id "Novel_003395"; exon_number "2"; gene_type "
scaffold1Cufflinks	exon	1109024	1109561	.	-	.	gene_id "XLOC_241951"; transcript_id "Novel_003396"; exon_number "1"; gene_type "
scaffold1Cufflinks	exon	1109648	1109806	.	-	.	gene_id "XLOC_241951"; transcript_id "Novel_003396"; exon_number "2"; gene_type "
scaffold1Cufflinks	exon	1109892	1110016	.	-	.	gene_id "XLOC_241951"; transcript_id "Novel_003396"; exon_number "3"; gene_type "
scaffold1Cufflinks	exon	1110700	1110895	.	-	.	gene_id "XLOC_241951"; transcript_id "Novel_003396"; exon_number "4"; gene_type "
scaffold1Cufflinks	exon	1111591	1111723	.	-	.	gene_id "XLOC_241951"; transcript_id "Novel_003396"; exon_number "5"; gene_type "
scaffold1Cufflinks	exon	1111817	1112537	.	-	.	gene_id "XLOC_241951"; transcript_id "Novel_003396"; exon_number "6"; gene_type "
scaffold1Cufflinks	exon	211800	212236	.	+	.	gene_id "XLOC_242326"; transcript_id "Novel_003397"; exon_number "1"; gene_type "
scaffold1Cufflinks	exon	213578	213832	.	+	.	gene_id "XLOC_242326"; transcript_id "Novel_003397"; exon_number "2"; gene_type "
scaffold1Cufflinks	exon	213999	214521	.	+	.	gene_id "XLOC_242326"; transcript_id "Novel_003397"; exon_number "3"; gene_type "
scaffold1Cufflinks	exon	354876	355197	.	+	.	gene_id "XLOC_242358"; transcript_id "Novel_003398"; exon_number "1"; gene_type "
scaffold1Cufflinks	exon	355307	355733	.	+	.	gene_id "XLOC_242358"; transcript_id "Novel_003398"; exon_number "2"; gene_type "
scaffold1Cufflinks	exon	19	488	.	-	.	gene_id "XLOC_242477"; transcript_id "Novel_003399"; exon_number "1"; gene_type "
scaffold1Cufflinks	exon	585	673	.	-	.	gene_id "XLOC_242477"; transcript_id "Novel_003399"; exon_number "2"; gene_type "
scaffold1Cufflinks	exon	990	1111	.	-	.	gene_id "XLOC_242477"; transcript_id "Novel_003399"; exon_number "3"; gene_type "
scaffold1Cufflinks	exon	1608	1714	.	-	.	gene_id "XLOC_242477"; transcript_id "Novel_003399"; exon_number "4"; gene_type "
scaffold1Cufflinks	exon	1789	1859	.	-	.	gene_id "XLOC_242477"; transcript_id "Novel_003399"; exon_number "5"; gene_type "
scaffold1Cufflinks	exon	2197	2290	.	-	.	gene_id "XLOC_242477"; transcript_id "Novel_003399"; exon_number "6"; gene_type "
scaffold1Cufflinks	exon	2376	2530	.	-	.	gene_id "XLOC_242477"; transcript_id "Novel_003399"; exon_number "7"; gene_type "
scaffold1Cufflinks	exon	2623	2730	.	-	.	gene_id "XLOC_242477"; transcript_id "Novel_003399"; exon_number "8"; gene_type "

scaffold1Cufflinks	exon	2974	3295	.	-	.	gene_id "XLOC_242477"; transcript_id "Novel_003399"; exon_number "9"; gene_type "
scaffold1Cufflinks	exon	1526	1714	.	-	.	gene_id "XLOC_242477"; transcript_id "Novel_003400"; exon_number "1"; gene_type "
scaffold1Cufflinks	exon	1789	1859	.	-	.	gene_id "XLOC_242477"; transcript_id "Novel_003400"; exon_number "2"; gene_type "
scaffold1Cufflinks	exon	2197	2290	.	-	.	gene_id "XLOC_242477"; transcript_id "Novel_003400"; exon_number "3"; gene_type "
scaffold1Cufflinks	exon	2376	2530	.	-	.	gene_id "XLOC_242477"; transcript_id "Novel_003400"; exon_number "4"; gene_type "
scaffold1Cufflinks	exon	2623	2730	.	-	.	gene_id "XLOC_242477"; transcript_id "Novel_003400"; exon_number "5"; gene_type "
scaffold1Cufflinks	exon	2974	3262	.	-	.	gene_id "XLOC_242477"; transcript_id "Novel_003400"; exon_number "6"; gene_type "
scaffold1Cufflinks	exon	395	610	.	+	.	gene_id "XLOC_242656"; transcript_id "Novel_003401"; exon_number "1"; gene_type "
scaffold1Cufflinks	exon	54	288	.	+	.	gene_id "XLOC_242677"; transcript_id "Novel_003402"; exon_number "1"; gene_type "
scaffold1Cufflinks	exon	2260	2751	.	+	.	gene_id "XLOC_242679"; transcript_id "Novel_003403"; exon_number "1"; gene_type "
scaffold1Cufflinks	exon	2943	3010	.	+	.	gene_id "XLOC_242679"; transcript_id "Novel_003403"; exon_number "2"; gene_type "
scaffold1Cufflinks	exon	3145	3300	.	+	.	gene_id "XLOC_242679"; transcript_id "Novel_003403"; exon_number "3"; gene_type "
scaffold1Cufflinks	exon	2814	3010	.	+	.	gene_id "XLOC_242679"; transcript_id "Novel_003404"; exon_number "1"; gene_type "
scaffold1Cufflinks	exon	3145	3306	.	+	.	gene_id "XLOC_242679"; transcript_id "Novel_003404"; exon_number "2"; gene_type "
scaffold1Cufflinks	exon	1247	1527	.	+	.	gene_id "XLOC_242680"; transcript_id "Novel_003405"; exon_number "1"; gene_type "
scaffold1Cufflinks	exon	196310	196517	.	+	.	gene_id "XLOC_242713"; transcript_id "Novel_003406"; exon_number "1"; gene_type "
scaffold1Cufflinks	exon	196614	196796	.	+	.	gene_id "XLOC_242713"; transcript_id "Novel_003406"; exon_number "2"; gene_type "
scaffold1Cufflinks	exon	727	1210	.	-	.	gene_id "XLOC_242837"; transcript_id "Novel_003407"; exon_number "1"; gene_type "
scaffold1Cufflinks	exon	1599	1775	.	-	.	gene_id "XLOC_242837"; transcript_id "Novel_003407"; exon_number "2"; gene_type "
scaffold1Cufflinks	exon	2683	3339	.	-	.	gene_id "XLOC_242837"; transcript_id "Novel_003407"; exon_number "3"; gene_type "
scaffold1Cufflinks	exon	491	805	.	-	.	gene_id "XLOC_242977"; transcript_id "Novel_003408"; exon_number "1"; gene_type "
scaffold1Cufflinks	exon	228329	228984	.	-	.	gene_id "XLOC_243044"; transcript_id "Novel_003409"; exon_number "1"; gene_type "
scaffold1Cufflinks	exon	230801	230891	.	-	.	gene_id "XLOC_243044"; transcript_id "Novel_003409"; exon_number "2"; gene_type "
scaffold1Cufflinks	exon	231023	231070	.	-	.	gene_id "XLOC_243044"; transcript_id "Novel_003409"; exon_number "3"; gene_type "
scaffold1Cufflinks	exon	231235	231412	.	-	.	gene_id "XLOC_243044"; transcript_id "Novel_003409"; exon_number "4"; gene_type "
scaffold1Cufflinks	exon	228329	228984	.	-	.	gene_id "XLOC_243044"; transcript_id "Novel_003410"; exon_number "1"; gene_type "
scaffold1Cufflinks	exon	230801	230906	.	-	.	gene_id "XLOC_243044"; transcript_id "Novel_003410"; exon_number "2"; gene_type "
scaffold1Cufflinks	exon	231023	231070	.	-	.	gene_id "XLOC_243044"; transcript_id "Novel_003410"; exon_number "3"; gene_type "
scaffold1Cufflinks	exon	231235	231412	.	-	.	gene_id "XLOC_243044"; transcript_id "Novel_003410"; exon_number "4"; gene_type "
scaffold1Cufflinks	exon	532102	532597	.	-	.	gene_id "XLOC_243080"; transcript_id "Novel_003411"; exon_number "1"; gene_type "
scaffold1Cufflinks	exon	532890	533737	.	-	.	gene_id "XLOC_243080"; transcript_id "Novel_003411"; exon_number "2"; gene_type "
scaffold1Cufflinks	exon	465637	466267	.	-	.	gene_id "XLOC_243274"; transcript_id "Novel_003412"; exon_number "1"; gene_type "
scaffold1Cufflinks	exon	466389	466451	.	-	.	gene_id "XLOC_243274"; transcript_id "Novel_003412"; exon_number "2"; gene_type "
scaffold1Cufflinks	exon	466549	466930	.	-	.	gene_id "XLOC_243274"; transcript_id "Novel_003412"; exon_number "3"; gene_type "
scaffold1Cufflinks	exon	465637	466290	.	-	.	gene_id "XLOC_243274"; transcript_id "Novel_003413"; exon_number "1"; gene_type "

scaffold1	Cufflinks	exon	466389	466451	.	-	.	gene_id "XLOC_243274"; transcript_id "Novel_003413"; exon_number "2"; gene_type "
scaffold1	Cufflinks	exon	466549	466622	.	-	.	gene_id "XLOC_243274"; transcript_id "Novel_003413"; exon_number "3"; gene_type "
scaffold1	Cufflinks	exon	466695	466930	.	-	.	gene_id "XLOC_243274"; transcript_id "Novel_003413"; exon_number "4"; gene_type "
scaffold1	Cufflinks	exon	465767	466622	.	-	.	gene_id "XLOC_243274"; transcript_id "Novel_003414"; exon_number "1"; gene_type "
scaffold1	Cufflinks	exon	466695	466930	.	-	.	gene_id "XLOC_243274"; transcript_id "Novel_003414"; exon_number "2"; gene_type "
scaffold1	Cufflinks	exon	465767	466290	.	-	.	gene_id "XLOC_243274"; transcript_id "Novel_003415"; exon_number "1"; gene_type "
scaffold1	Cufflinks	exon	466389	466451	.	-	.	gene_id "XLOC_243274"; transcript_id "Novel_003415"; exon_number "2"; gene_type "
scaffold1	Cufflinks	exon	466549	466930	.	-	.	gene_id "XLOC_243274"; transcript_id "Novel_003415"; exon_number "3"; gene_type "
scaffold1	Cufflinks	exon	465856	466267	.	-	.	gene_id "XLOC_243274"; transcript_id "Novel_003416"; exon_number "1"; gene_type "
scaffold1	Cufflinks	exon	466389	466451	.	-	.	gene_id "XLOC_243274"; transcript_id "Novel_003416"; exon_number "2"; gene_type "
scaffold1	Cufflinks	exon	466549	466622	.	-	.	gene_id "XLOC_243274"; transcript_id "Novel_003416"; exon_number "3"; gene_type "
scaffold1	Cufflinks	exon	466695	466969	.	-	.	gene_id "XLOC_243274"; transcript_id "Novel_003416"; exon_number "4"; gene_type "
scaffold1	Cufflinks	exon	105514	105646	.	+	.	gene_id "XLOC_243406"; transcript_id "Novel_003417"; exon_number "1"; gene_type "
scaffold1	Cufflinks	exon	105750	105810	.	+	.	gene_id "XLOC_243406"; transcript_id "Novel_003417"; exon_number "2"; gene_type "
scaffold1	Cufflinks	exon	105935	106027	.	+	.	gene_id "XLOC_243406"; transcript_id "Novel_003417"; exon_number "3"; gene_type "
scaffold1	Cufflinks	exon	106394	106590	.	+	.	gene_id "XLOC_243406"; transcript_id "Novel_003417"; exon_number "4"; gene_type "
scaffold1	Cufflinks	exon	106697	107294	.	+	.	gene_id "XLOC_243406"; transcript_id "Novel_003417"; exon_number "5"; gene_type "
scaffold1	Cufflinks	exon	105539	105650	.	+	.	gene_id "XLOC_243406"; transcript_id "Novel_003418"; exon_number "1"; gene_type "
scaffold1	Cufflinks	exon	105750	105810	.	+	.	gene_id "XLOC_243406"; transcript_id "Novel_003418"; exon_number "2"; gene_type "
scaffold1	Cufflinks	exon	105935	106027	.	+	.	gene_id "XLOC_243406"; transcript_id "Novel_003418"; exon_number "3"; gene_type "
scaffold1	Cufflinks	exon	106394	106590	.	+	.	gene_id "XLOC_243406"; transcript_id "Novel_003418"; exon_number "4"; gene_type "
scaffold1	Cufflinks	exon	106697	106712	.	+	.	gene_id "XLOC_243406"; transcript_id "Novel_003418"; exon_number "5"; gene_type "
scaffold1	Cufflinks	exon	106767	106802	.	+	.	gene_id "XLOC_243406"; transcript_id "Novel_003419"; exon_number "1"; gene_type "
scaffold1	Cufflinks	exon	108296	108442	.	+	.	gene_id "XLOC_243406"; transcript_id "Novel_003419"; exon_number "2"; gene_type "
scaffold1	Cufflinks	exon	109146	109502	.	+	.	gene_id "XLOC_243406"; transcript_id "Novel_003419"; exon_number "3"; gene_type "
scaffold1	Cufflinks	exon	72029	72900	.	-	.	gene_id "XLOC_243463"; transcript_id "Novel_003420"; exon_number "1"; gene_type "
scaffold1	Cufflinks	exon	73354	73547	.	-	.	gene_id "XLOC_243463"; transcript_id "Novel_003420"; exon_number "2"; gene_type "
scaffold1	Cufflinks	exon	168978	170881	.	-	.	gene_id "XLOC_243469"; transcript_id "Novel_003421"; exon_number "1"; gene_type "
scaffold1	Cufflinks	exon	270581	270730	.	-	.	gene_id "XLOC_243481"; transcript_id "Novel_003422"; exon_number "1"; gene_type "
scaffold1	Cufflinks	exon	270812	271423	.	-	.	gene_id "XLOC_243481"; transcript_id "Novel_003422"; exon_number "2"; gene_type "
scaffold1	Cufflinks	exon	272636	273037	.	-	.	gene_id "XLOC_243481"; transcript_id "Novel_003422"; exon_number "3"; gene_type "
scaffold1	Cufflinks	exon	273343	273550	.	-	.	gene_id "XLOC_243481"; transcript_id "Novel_003422"; exon_number "4"; gene_type "
scaffold1	Cufflinks	exon	540737	540767	.	-	.	gene_id "XLOC_243510"; transcript_id "Novel_003423"; exon_number "1"; gene_type "
scaffold1	Cufflinks	exon	541178	542270	.	-	.	gene_id "XLOC_243510"; transcript_id "Novel_003423"; exon_number "2"; gene_type "
scaffold1	Cufflinks	exon	569321	569496	.	-	.	gene_id "XLOC_243512"; transcript_id "Novel_003424"; exon_number "1"; gene_type "

scaffold1Cufflinks	exon	569643	569693	.	-	.	gene_id "XLOC_243512"; transcript_id "Novel_003424"; exon_number "2"; gene_type "
scaffold1Cufflinks	exon	297670	298863	.	-	.	gene_id "XLOC_243645"; transcript_id "Novel_003425"; exon_number "1"; gene_type "
scaffold1Cufflinks	exon	300072	300279	.	-	.	gene_id "XLOC_243645"; transcript_id "Novel_003425"; exon_number "2"; gene_type "
scaffold1Cufflinks	exon	209451	209896	.	+	.	gene_id "XLOC_243849"; transcript_id "Novel_003426"; exon_number "1"; gene_type "
scaffold1Cufflinks	exon	210064	210154	.	+	.	gene_id "XLOC_243849"; transcript_id "Novel_003426"; exon_number "2"; gene_type "
scaffold1Cufflinks	exon	210687	210756	.	+	.	gene_id "XLOC_243849"; transcript_id "Novel_003426"; exon_number "3"; gene_type "
scaffold1Cufflinks	exon	210851	210946	.	+	.	gene_id "XLOC_243849"; transcript_id "Novel_003426"; exon_number "4"; gene_type "
scaffold1Cufflinks	exon	211500	211834	.	+	.	gene_id "XLOC_243849"; transcript_id "Novel_003426"; exon_number "5"; gene_type "
scaffold1Cufflinks	exon	211927	212047	.	+	.	gene_id "XLOC_243849"; transcript_id "Novel_003426"; exon_number "6"; gene_type "
scaffold1Cufflinks	exon	212176	212446	.	+	.	gene_id "XLOC_243849"; transcript_id "Novel_003426"; exon_number "7"; gene_type "
scaffold1Cufflinks	exon	416545	417703	.	-	.	gene_id "XLOC_243942"; transcript_id "Novel_003427"; exon_number "1"; gene_type "
scaffold1Cufflinks	exon	417888	418944	.	-	.	gene_id "XLOC_243942"; transcript_id "Novel_003427"; exon_number "2"; gene_type "
scaffold1Cufflinks	exon	417332	417698	.	-	.	gene_id "XLOC_243942"; transcript_id "Novel_003428"; exon_number "1"; gene_type "
scaffold1Cufflinks	exon	417888	419018	.	-	.	gene_id "XLOC_243942"; transcript_id "Novel_003428"; exon_number "2"; gene_type "
scaffold1Cufflinks	exon	260055	260795	.	+	.	gene_id "XLOC_244223"; transcript_id "Novel_003429"; exon_number "1"; gene_type "
scaffold1Cufflinks	exon	261582	262256	.	+	.	gene_id "XLOC_244223"; transcript_id "Novel_003429"; exon_number "2"; gene_type "
scaffold1Cufflinks	exon	432253	434175	.	+	.	gene_id "XLOC_244253"; transcript_id "Novel_003430"; exon_number "1"; gene_type "
scaffold1Cufflinks	exon	179941	180959	.	-	.	gene_id "XLOC_244276"; transcript_id "Novel_003431"; exon_number "1"; gene_type "
scaffold1Cufflinks	exon	181765	181931	.	-	.	gene_id "XLOC_244276"; transcript_id "Novel_003431"; exon_number "2"; gene_type "
scaffold1Cufflinks	exon	182079	182262	.	-	.	gene_id "XLOC_244276"; transcript_id "Novel_003431"; exon_number "3"; gene_type "
scaffold1Cufflinks	exon	182362	182439	.	-	.	gene_id "XLOC_244276"; transcript_id "Novel_003431"; exon_number "4"; gene_type "
scaffold1Cufflinks	exon	182530	182598	.	-	.	gene_id "XLOC_244276"; transcript_id "Novel_003431"; exon_number "5"; gene_type "
scaffold1Cufflinks	exon	183355	183482	.	-	.	gene_id "XLOC_244278"; transcript_id "Novel_003432"; exon_number "1"; gene_type "
scaffold1Cufflinks	exon	183962	184380	.	-	.	gene_id "XLOC_244278"; transcript_id "Novel_003432"; exon_number "2"; gene_type "
scaffold1Cufflinks	exon	300072	301437	.	-	.	gene_id "XLOC_244286"; transcript_id "Novel_003433"; exon_number "1"; gene_type "
scaffold1Cufflinks	exon	301539	301753	.	-	.	gene_id "XLOC_244286"; transcript_id "Novel_003433"; exon_number "2"; gene_type "
scaffold1Cufflinks	exon	301674	302790	.	-	.	gene_id "XLOC_244286"; transcript_id "Novel_003434"; exon_number "1"; gene_type "
scaffold1Cufflinks	exon	303888	304676	.	-	.	gene_id "XLOC_244286"; transcript_id "Novel_003434"; exon_number "2"; gene_type "
scaffold1Cufflinks	exon	321202	323413	.	-	.	gene_id "XLOC_244292"; transcript_id "Novel_003435"; exon_number "1"; gene_type "
scaffold1Cufflinks	exon	323831	323951	.	-	.	gene_id "XLOC_244292"; transcript_id "Novel_003435"; exon_number "2"; gene_type "
scaffold1Cufflinks	exon	324031	324081	.	-	.	gene_id "XLOC_244292"; transcript_id "Novel_003435"; exon_number "3"; gene_type "
scaffold1Cufflinks	exon	324179	324257	.	-	.	gene_id "XLOC_244292"; transcript_id "Novel_003435"; exon_number "4"; gene_type "
scaffold1Cufflinks	exon	324352	324455	.	-	.	gene_id "XLOC_244292"; transcript_id "Novel_003435"; exon_number "5"; gene_type "
scaffold1Cufflinks	exon	324543	324624	.	-	.	gene_id "XLOC_244292"; transcript_id "Novel_003435"; exon_number "6"; gene_type "
scaffold1Cufflinks	exon	324733	324853	.	-	.	gene_id "XLOC_244292"; transcript_id "Novel_003435"; exon_number "7"; gene_type "

scaffold1	Cufflinks	exon	324943	325102	.	-	.	gene_id "XLOC_244292"; transcript_id "Novel_003435"; exon_number "8"; gene_type "
scaffold1	Cufflinks	exon	325219	325280	.	-	.	gene_id "XLOC_244292"; transcript_id "Novel_003435"; exon_number "9"; gene_type "
scaffold1	Cufflinks	exon	325980	326139	.	-	.	gene_id "XLOC_244292"; transcript_id "Novel_003435"; exon_number "10"; gene_type
scaffold1	Cufflinks	exon	326291	326664	.	-	.	gene_id "XLOC_244292"; transcript_id "Novel_003435"; exon_number "11"; gene_type
scaffold1	Cufflinks	exon	321291	322210	.	-	.	gene_id "XLOC_244292"; transcript_id "Novel_003436"; exon_number "1"; gene_type "
scaffold1	Cufflinks	exon	323831	323951	.	-	.	gene_id "XLOC_244292"; transcript_id "Novel_003436"; exon_number "2"; gene_type "
scaffold1	Cufflinks	exon	324031	324081	.	-	.	gene_id "XLOC_244292"; transcript_id "Novel_003436"; exon_number "3"; gene_type "
scaffold1	Cufflinks	exon	324179	324257	.	-	.	gene_id "XLOC_244292"; transcript_id "Novel_003436"; exon_number "4"; gene_type "
scaffold1	Cufflinks	exon	324352	324455	.	-	.	gene_id "XLOC_244292"; transcript_id "Novel_003436"; exon_number "5"; gene_type "
scaffold1	Cufflinks	exon	324543	324624	.	-	.	gene_id "XLOC_244292"; transcript_id "Novel_003436"; exon_number "6"; gene_type "
scaffold1	Cufflinks	exon	324733	324853	.	-	.	gene_id "XLOC_244292"; transcript_id "Novel_003436"; exon_number "7"; gene_type "
scaffold1	Cufflinks	exon	324943	325102	.	-	.	gene_id "XLOC_244292"; transcript_id "Novel_003436"; exon_number "8"; gene_type "
scaffold1	Cufflinks	exon	325219	325280	.	-	.	gene_id "XLOC_244292"; transcript_id "Novel_003436"; exon_number "9"; gene_type "
scaffold1	Cufflinks	exon	325980	326156	.	-	.	gene_id "XLOC_244292"; transcript_id "Novel_003436"; exon_number "10"; gene_type
scaffold1	Cufflinks	exon	326291	326681	.	-	.	gene_id "XLOC_244292"; transcript_id "Novel_003436"; exon_number "11"; gene_type
scaffold1	Cufflinks	exon	322326	323951	.	-	.	gene_id "XLOC_244292"; transcript_id "Novel_003437"; exon_number "1"; gene_type "
scaffold1	Cufflinks	exon	324031	324081	.	-	.	gene_id "XLOC_244292"; transcript_id "Novel_003437"; exon_number "2"; gene_type "
scaffold1	Cufflinks	exon	324179	324257	.	-	.	gene_id "XLOC_244292"; transcript_id "Novel_003437"; exon_number "3"; gene_type "
scaffold1	Cufflinks	exon	324352	324455	.	-	.	gene_id "XLOC_244292"; transcript_id "Novel_003437"; exon_number "4"; gene_type "
scaffold1	Cufflinks	exon	324543	324624	.	-	.	gene_id "XLOC_244292"; transcript_id "Novel_003437"; exon_number "5"; gene_type "
scaffold1	Cufflinks	exon	324733	324853	.	-	.	gene_id "XLOC_244292"; transcript_id "Novel_003437"; exon_number "6"; gene_type "
scaffold1	Cufflinks	exon	324943	325102	.	-	.	gene_id "XLOC_244292"; transcript_id "Novel_003437"; exon_number "7"; gene_type "
scaffold1	Cufflinks	exon	325219	325280	.	-	.	gene_id "XLOC_244292"; transcript_id "Novel_003437"; exon_number "8"; gene_type "
scaffold1	Cufflinks	exon	325980	326139	.	-	.	gene_id "XLOC_244292"; transcript_id "Novel_003437"; exon_number "9"; gene_type "
scaffold1	Cufflinks	exon	326291	326664	.	-	.	gene_id "XLOC_244292"; transcript_id "Novel_003437"; exon_number "10"; gene_type
scaffold1	Cufflinks	exon	322326	323951	.	-	.	gene_id "XLOC_244292"; transcript_id "Novel_003438"; exon_number "1"; gene_type "
scaffold1	Cufflinks	exon	324031	324081	.	-	.	gene_id "XLOC_244292"; transcript_id "Novel_003438"; exon_number "2"; gene_type "
scaffold1	Cufflinks	exon	324179	324257	.	-	.	gene_id "XLOC_244292"; transcript_id "Novel_003438"; exon_number "3"; gene_type "
scaffold1	Cufflinks	exon	324352	324455	.	-	.	gene_id "XLOC_244292"; transcript_id "Novel_003438"; exon_number "4"; gene_type "
scaffold1	Cufflinks	exon	324543	324624	.	-	.	gene_id "XLOC_244292"; transcript_id "Novel_003438"; exon_number "5"; gene_type "
scaffold1	Cufflinks	exon	324733	324853	.	-	.	gene_id "XLOC_244292"; transcript_id "Novel_003438"; exon_number "6"; gene_type "
scaffold1	Cufflinks	exon	324943	325102	.	-	.	gene_id "XLOC_244292"; transcript_id "Novel_003438"; exon_number "7"; gene_type "
scaffold1	Cufflinks	exon	325219	325280	.	-	.	gene_id "XLOC_244292"; transcript_id "Novel_003438"; exon_number "8"; gene_type "
scaffold1	Cufflinks	exon	325980	326156	.	-	.	gene_id "XLOC_244292"; transcript_id "Novel_003438"; exon_number "9"; gene_type "
scaffold1	Cufflinks	exon	326291	326664	.	-	.	gene_id "XLOC_244292"; transcript_id "Novel_003438"; exon_number "10"; gene_type

scaffold1	Cufflinks	exon	82670	83112	.	+	.	gene_id "XLOC_244522"; transcript_id "Novel_003439"; exon_number "1"; gene_type "
scaffold1	Cufflinks	exon	83206	83504	.	+	.	gene_id "XLOC_244522"; transcript_id "Novel_003439"; exon_number "2"; gene_type "
scaffold1	Cufflinks	exon	83585	83725	.	+	.	gene_id "XLOC_244522"; transcript_id "Novel_003439"; exon_number "3"; gene_type "
scaffold1	Cufflinks	exon	83814	84093	.	+	.	gene_id "XLOC_244522"; transcript_id "Novel_003439"; exon_number "4"; gene_type "
scaffold1	Cufflinks	exon	89489	89950	.	+	.	gene_id "XLOC_244525"; transcript_id "Novel_003440"; exon_number "1"; gene_type "
scaffold1	Cufflinks	exon	90039	90560	.	+	.	gene_id "XLOC_244525"; transcript_id "Novel_003440"; exon_number "2"; gene_type "
scaffold1	Cufflinks	exon	90649	90876	.	+	.	gene_id "XLOC_244525"; transcript_id "Novel_003440"; exon_number "3"; gene_type "
scaffold1	Cufflinks	exon	16027	16088	.	-	.	gene_id "XLOC_244549"; transcript_id "Novel_003441"; exon_number "1"; gene_type "
scaffold1	Cufflinks	exon	21302	21376	.	-	.	gene_id "XLOC_244549"; transcript_id "Novel_003441"; exon_number "2"; gene_type "
scaffold1	Cufflinks	exon	21467	21563	.	-	.	gene_id "XLOC_244549"; transcript_id "Novel_003441"; exon_number "3"; gene_type "
scaffold1	Cufflinks	exon	287065	287626	.	-	.	gene_id "XLOC_244572"; transcript_id "Novel_003442"; exon_number "1"; gene_type "
scaffold1	Cufflinks	exon	288077	289495	.	-	.	gene_id "XLOC_244572"; transcript_id "Novel_003442"; exon_number "2"; gene_type "
scaffold1	Cufflinks	exon	289775	289921	.	-	.	gene_id "XLOC_244572"; transcript_id "Novel_003442"; exon_number "3"; gene_type "
scaffold1	Cufflinks	exon	290205	290351	.	-	.	gene_id "XLOC_244572"; transcript_id "Novel_003442"; exon_number "4"; gene_type "
scaffold1	Cufflinks	exon	290638	290784	.	-	.	gene_id "XLOC_244572"; transcript_id "Novel_003442"; exon_number "5"; gene_type "
scaffold1	Cufflinks	exon	291166	291492	.	-	.	gene_id "XLOC_244572"; transcript_id "Novel_003442"; exon_number "6"; gene_type "
scaffold1	Cufflinks	exon	291753	294051	.	-	.	gene_id "XLOC_244572"; transcript_id "Novel_003442"; exon_number "7"; gene_type "
scaffold1	Cufflinks	exon	344208	345539	.	-	.	gene_id "XLOC_244579"; transcript_id "Novel_003443"; exon_number "1"; gene_type "
scaffold1	Cufflinks	exon	346768	347647	.	-	.	gene_id "XLOC_244579"; transcript_id "Novel_003443"; exon_number "2"; gene_type "
scaffold1	Cufflinks	exon	347736	348318	.	-	.	gene_id "XLOC_244579"; transcript_id "Novel_003443"; exon_number "3"; gene_type "
scaffold1	Cufflinks	exon	346817	346868	.	-	.	gene_id "XLOC_244579"; transcript_id "Novel_003444"; exon_number "1"; gene_type "
scaffold1	Cufflinks	exon	347736	348314	.	-	.	gene_id "XLOC_244579"; transcript_id "Novel_003444"; exon_number "2"; gene_type "
scaffold1	Cufflinks	exon	353686	353770	.	-	.	gene_id "XLOC_244581"; transcript_id "Novel_003445"; exon_number "1"; gene_type "
scaffold1	Cufflinks	exon	461842	462826	.	-	.	gene_id "XLOC_244581"; transcript_id "Novel_003445"; exon_number "2"; gene_type "
scaffold1	Cufflinks	exon	370425	370716	.	-	.	gene_id "XLOC_244587"; transcript_id "Novel_003446"; exon_number "1"; gene_type "
scaffold1	Cufflinks	exon	371123	372078	.	-	.	gene_id "XLOC_244587"; transcript_id "Novel_003446"; exon_number "2"; gene_type "
scaffold1	Cufflinks	exon	464471	464950	.	+	.	gene_id "XLOC_244684"; transcript_id "Novel_003447"; exon_number "1"; gene_type "
scaffold1	Cufflinks	exon	465541	466086	.	+	.	gene_id "XLOC_244684"; transcript_id "Novel_003447"; exon_number "2"; gene_type "
scaffold1	Cufflinks	exon	464477	464514	.	+	.	gene_id "XLOC_244684"; transcript_id "Novel_003448"; exon_number "1"; gene_type "
scaffold1	Cufflinks	exon	464866	466086	.	+	.	gene_id "XLOC_244684"; transcript_id "Novel_003448"; exon_number "2"; gene_type "
scaffold1	Cufflinks	exon	135521	136053	.	-	.	gene_id "XLOC_244704"; transcript_id "Novel_003449"; exon_number "1"; gene_type "
scaffold1	Cufflinks	exon	136836	136968	.	-	.	gene_id "XLOC_244704"; transcript_id "Novel_003449"; exon_number "2"; gene_type "
scaffold1	Cufflinks	exon	12234	12487	.	-	.	gene_id "XLOC_244819"; transcript_id "Novel_003450"; exon_number "1"; gene_type "
scaffold1	Cufflinks	exon	557184	557807	.	-	.	gene_id "XLOC_245090"; transcript_id "Novel_003451"; exon_number "1"; gene_type "
scaffold1	Cufflinks	exon	559346	561404	.	-	.	gene_id "XLOC_245090"; transcript_id "Novel_003451"; exon_number "2"; gene_type "

scaffold1Cufflinks	exon	557184	557807	.	-	.	gene_id "XLOC_245090"; transcript_id "Novel_003452"; exon_number "1"; gene_type "
scaffold1Cufflinks	exon	559346	561272	.	-	.	gene_id "XLOC_245090"; transcript_id "Novel_003452"; exon_number "2"; gene_type "
scaffold1Cufflinks	exon	561362	561461	.	-	.	gene_id "XLOC_245090"; transcript_id "Novel_003452"; exon_number "3"; gene_type "
scaffold1Cufflinks	exon	558021	561272	.	-	.	gene_id "XLOC_245090"; transcript_id "Novel_003453"; exon_number "1"; gene_type "
scaffold1Cufflinks	exon	561362	561460	.	-	.	gene_id "XLOC_245090"; transcript_id "Novel_003453"; exon_number "2"; gene_type "
scaffold1Cufflinks	exon	2	503	.	-	.	gene_id "XLOC_245098"; transcript_id "Novel_003454"; exon_number "1"; gene_type "
scaffold1Cufflinks	exon	974	1064	.	-	.	gene_id "XLOC_245098"; transcript_id "Novel_003454"; exon_number "2"; gene_type "
scaffold1Cufflinks	exon	1331	1383	.	-	.	gene_id "XLOC_245098"; transcript_id "Novel_003454"; exon_number "3"; gene_type "
scaffold1Cufflinks	exon	1984	2017	.	-	.	gene_id "XLOC_245098"; transcript_id "Novel_003454"; exon_number "4"; gene_type "
scaffold1Cufflinks	exon	2296	2455	.	-	.	gene_id "XLOC_245098"; transcript_id "Novel_003454"; exon_number "5"; gene_type "
scaffold1Cufflinks	exon	2594	2782	.	-	.	gene_id "XLOC_245098"; transcript_id "Novel_003454"; exon_number "6"; gene_type "
scaffold1Cufflinks	exon	2	503	.	-	.	gene_id "XLOC_245098"; transcript_id "Novel_003455"; exon_number "1"; gene_type "
scaffold1Cufflinks	exon	974	1064	.	-	.	gene_id "XLOC_245098"; transcript_id "Novel_003455"; exon_number "2"; gene_type "
scaffold1Cufflinks	exon	1331	2455	.	-	.	gene_id "XLOC_245098"; transcript_id "Novel_003455"; exon_number "3"; gene_type "
scaffold1Cufflinks	exon	2594	2782	.	-	.	gene_id "XLOC_245098"; transcript_id "Novel_003455"; exon_number "4"; gene_type "
scaffold1Cufflinks	exon	7	503	.	-	.	gene_id "XLOC_245098"; transcript_id "Novel_003456"; exon_number "1"; gene_type "
scaffold1Cufflinks	exon	974	1064	.	-	.	gene_id "XLOC_245098"; transcript_id "Novel_003456"; exon_number "2"; gene_type "
scaffold1Cufflinks	exon	1331	2017	.	-	.	gene_id "XLOC_245098"; transcript_id "Novel_003456"; exon_number "3"; gene_type "
scaffold1Cufflinks	exon	2296	2455	.	-	.	gene_id "XLOC_245098"; transcript_id "Novel_003456"; exon_number "4"; gene_type "
scaffold1Cufflinks	exon	2594	2782	.	-	.	gene_id "XLOC_245098"; transcript_id "Novel_003456"; exon_number "5"; gene_type "
scaffold1Cufflinks	exon	16	503	.	-	.	gene_id "XLOC_245098"; transcript_id "Novel_003457"; exon_number "1"; gene_type "
scaffold1Cufflinks	exon	974	1064	.	-	.	gene_id "XLOC_245098"; transcript_id "Novel_003457"; exon_number "2"; gene_type "
scaffold1Cufflinks	exon	1331	1383	.	-	.	gene_id "XLOC_245098"; transcript_id "Novel_003457"; exon_number "3"; gene_type "
scaffold1Cufflinks	exon	1727	2455	.	-	.	gene_id "XLOC_245098"; transcript_id "Novel_003457"; exon_number "4"; gene_type "
scaffold1Cufflinks	exon	2594	2781	.	-	.	gene_id "XLOC_245098"; transcript_id "Novel_003457"; exon_number "5"; gene_type "
scaffold1Cufflinks	exon	6	2474	.	-	.	gene_id "XLOC_245176"; transcript_id "Novel_003458"; exon_number "1"; gene_type "
scaffold1Cufflinks	exon	2557	2639	.	-	.	gene_id "XLOC_245176"; transcript_id "Novel_003458"; exon_number "2"; gene_type "
scaffold1Cufflinks	exon	13	298	.	-	.	gene_id "XLOC_245177"; transcript_id "Novel_003459"; exon_number "1"; gene_type "
scaffold1Cufflinks	exon	996	2642	.	-	.	gene_id "XLOC_245177"; transcript_id "Novel_003459"; exon_number "2"; gene_type "
scaffold1Cufflinks	exon	43	121	.	-	.	gene_id "XLOC_245177"; transcript_id "Novel_003460"; exon_number "1"; gene_type "
scaffold1Cufflinks	exon	197	298	.	-	.	gene_id "XLOC_245177"; transcript_id "Novel_003460"; exon_number "2"; gene_type "
scaffold1Cufflinks	exon	996	2635	.	-	.	gene_id "XLOC_245177"; transcript_id "Novel_003460"; exon_number "3"; gene_type "
scaffold1Cufflinks	exon	365945	366735	.	+	.	gene_id "XLOC_245234"; transcript_id "Novel_003461"; exon_number "1"; gene_type "
scaffold1Cufflinks	exon	366863	367090	.	+	.	gene_id "XLOC_245234"; transcript_id "Novel_003461"; exon_number "2"; gene_type "
scaffold1Cufflinks	exon	5	1081	.	-	.	gene_id "XLOC_245342"; transcript_id "Novel_003462"; exon_number "1"; gene_type "

scaffold1	Cufflinks	exon	1236	1578	.	-	.	gene_id	"XLOC_245342";	transcript_id	"Novel_003462";	exon_number	"2";	gene_type	"
scaffold1	Cufflinks	exon	1658	1756	.	-	.	gene_id	"XLOC_245342";	transcript_id	"Novel_003462";	exon_number	"3";	gene_type	"
scaffold1	Cufflinks	exon	2035	2152	.	-	.	gene_id	"XLOC_245342";	transcript_id	"Novel_003462";	exon_number	"4";	gene_type	"
scaffold1	Cufflinks	exon	2233	2318	.	-	.	gene_id	"XLOC_245342";	transcript_id	"Novel_003462";	exon_number	"5";	gene_type	"
scaffold1	Cufflinks	exon	2408	2476	.	-	.	gene_id	"XLOC_245342";	transcript_id	"Novel_003462";	exon_number	"6";	gene_type	"
scaffold1	Cufflinks	exon	2556	2620	.	-	.	gene_id	"XLOC_245342";	transcript_id	"Novel_003462";	exon_number	"7";	gene_type	"
scaffold1	Cufflinks	exon	16	1086	.	-	.	gene_id	"XLOC_245342";	transcript_id	"Novel_003463";	exon_number	"1";	gene_type	"
scaffold1	Cufflinks	exon	1236	1578	.	-	.	gene_id	"XLOC_245342";	transcript_id	"Novel_003463";	exon_number	"2";	gene_type	"
scaffold1	Cufflinks	exon	1658	1756	.	-	.	gene_id	"XLOC_245342";	transcript_id	"Novel_003463";	exon_number	"3";	gene_type	"
scaffold1	Cufflinks	exon	2035	2152	.	-	.	gene_id	"XLOC_245342";	transcript_id	"Novel_003463";	exon_number	"4";	gene_type	"
scaffold1	Cufflinks	exon	2233	2318	.	-	.	gene_id	"XLOC_245342";	transcript_id	"Novel_003463";	exon_number	"5";	gene_type	"
scaffold1	Cufflinks	exon	2408	2476	.	-	.	gene_id	"XLOC_245342";	transcript_id	"Novel_003463";	exon_number	"6";	gene_type	"
scaffold1	Cufflinks	exon	2556	2619	.	-	.	gene_id	"XLOC_245342";	transcript_id	"Novel_003463";	exon_number	"7";	gene_type	"
scaffold1	Cufflinks	exon	14	158	.	-	.	gene_id	"XLOC_245369";	transcript_id	"Novel_003464";	exon_number	"1";	gene_type	"
scaffold1	Cufflinks	exon	263	332	.	-	.	gene_id	"XLOC_245369";	transcript_id	"Novel_003464";	exon_number	"2";	gene_type	"
scaffold1	Cufflinks	exon	660	816	.	-	.	gene_id	"XLOC_245369";	transcript_id	"Novel_003464";	exon_number	"3";	gene_type	"
scaffold1	Cufflinks	exon	2120	2288	.	-	.	gene_id	"XLOC_245369";	transcript_id	"Novel_003464";	exon_number	"4";	gene_type	"
scaffold1	Cufflinks	exon	14	158	.	-	.	gene_id	"XLOC_245369";	transcript_id	"Novel_003465";	exon_number	"1";	gene_type	"
scaffold1	Cufflinks	exon	263	332	.	-	.	gene_id	"XLOC_245369";	transcript_id	"Novel_003465";	exon_number	"2";	gene_type	"
scaffold1	Cufflinks	exon	2120	2311	.	-	.	gene_id	"XLOC_245369";	transcript_id	"Novel_003465";	exon_number	"3";	gene_type	"
scaffold1	Cufflinks	exon	380936	381050	.	+	.	gene_id	"XLOC_245413";	transcript_id	"Novel_003466";	exon_number	"1";	gene_type	"
scaffold1	Cufflinks	exon	381433	382105	.	+	.	gene_id	"XLOC_245413";	transcript_id	"Novel_003466";	exon_number	"2";	gene_type	"
scaffold1	Cufflinks	exon	382348	382411	.	+	.	gene_id	"XLOC_245413";	transcript_id	"Novel_003466";	exon_number	"3";	gene_type	"
scaffold1	Cufflinks	exon	382504	382557	.	+	.	gene_id	"XLOC_245413";	transcript_id	"Novel_003466";	exon_number	"4";	gene_type	"
scaffold1	Cufflinks	exon	382811	382888	.	+	.	gene_id	"XLOC_245413";	transcript_id	"Novel_003466";	exon_number	"5";	gene_type	"
scaffold1	Cufflinks	exon	383844	383907	.	+	.	gene_id	"XLOC_245413";	transcript_id	"Novel_003466";	exon_number	"6";	gene_type	"
scaffold1	Cufflinks	exon	384100	384133	.	+	.	gene_id	"XLOC_245413";	transcript_id	"Novel_003466";	exon_number	"7";	gene_type	"
scaffold1	Cufflinks	exon	478664	478760	.	+	.	gene_id	"XLOC_245434";	transcript_id	"Novel_003467";	exon_number	"1";	gene_type	"
scaffold1	Cufflinks	exon	478943	479029	.	+	.	gene_id	"XLOC_245434";	transcript_id	"Novel_003467";	exon_number	"2";	gene_type	"
scaffold1	Cufflinks	exon	479126	479179	.	+	.	gene_id	"XLOC_245434";	transcript_id	"Novel_003467";	exon_number	"3";	gene_type	"
scaffold1	Cufflinks	exon	480046	482827	.	+	.	gene_id	"XLOC_245434";	transcript_id	"Novel_003467";	exon_number	"4";	gene_type	"
scaffold1	Cufflinks	exon	483052	483512	.	+	.	gene_id	"XLOC_245434";	transcript_id	"Novel_003467";	exon_number	"5";	gene_type	"
scaffold1	Cufflinks	exon	478664	478760	.	+	.	gene_id	"XLOC_245434";	transcript_id	"Novel_003468";	exon_number	"1";	gene_type	"
scaffold1	Cufflinks	exon	478943	479029	.	+	.	gene_id	"XLOC_245434";	transcript_id	"Novel_003468";	exon_number	"2";	gene_type	"
scaffold1	Cufflinks	exon	479126	483512	.	+	.	gene_id	"XLOC_245434";	transcript_id	"Novel_003468";	exon_number	"3";	gene_type	"

scaffold1Cufflinks	exon	765004	765850	.	+	.	gene_id "XLOC_245640"; transcript_id "Novel_003469"; exon_number "1"; gene_type "
scaffold1Cufflinks	exon	766759	767500	.	+	.	gene_id "XLOC_245640"; transcript_id "Novel_003469"; exon_number "2"; gene_type "
scaffold1Cufflinks	exon	409576	410086	.	-	.	gene_id "XLOC_245864"; transcript_id "Novel_003470"; exon_number "1"; gene_type "
scaffold1Cufflinks	exon	410164	410304	.	-	.	gene_id "XLOC_245864"; transcript_id "Novel_003470"; exon_number "2"; gene_type "
scaffold1Cufflinks	exon	411216	411338	.	-	.	gene_id "XLOC_245864"; transcript_id "Novel_003470"; exon_number "3"; gene_type "
scaffold1Cufflinks	exon	411988	412050	.	-	.	gene_id "XLOC_245864"; transcript_id "Novel_003470"; exon_number "4"; gene_type "
scaffold1Cufflinks	exon	412145	412220	.	-	.	gene_id "XLOC_245864"; transcript_id "Novel_003470"; exon_number "5"; gene_type "
scaffold1Cufflinks	exon	409609	410086	.	-	.	gene_id "XLOC_245864"; transcript_id "Novel_003471"; exon_number "1"; gene_type "
scaffold1Cufflinks	exon	411216	411338	.	-	.	gene_id "XLOC_245864"; transcript_id "Novel_003471"; exon_number "2"; gene_type "
scaffold1Cufflinks	exon	411988	412050	.	-	.	gene_id "XLOC_245864"; transcript_id "Novel_003471"; exon_number "3"; gene_type "
scaffold1Cufflinks	exon	412145	412207	.	-	.	gene_id "XLOC_245864"; transcript_id "Novel_003471"; exon_number "4"; gene_type "
scaffold1Cufflinks	exon	42821	44521	.	-	.	gene_id "XLOC_246174"; transcript_id "Novel_003472"; exon_number "1"; gene_type "
scaffold1Cufflinks	exon	21	121	.	+	.	gene_id "XLOC_246290"; transcript_id "Novel_003473"; exon_number "1"; gene_type "
scaffold1Cufflinks	exon	553	2325	.	+	.	gene_id "XLOC_246290"; transcript_id "Novel_003473"; exon_number "2"; gene_type "
scaffold1Cufflinks	exon	142825	143058	.	+	.	gene_id "XLOC_246447"; transcript_id "Novel_003474"; exon_number "1"; gene_type "
scaffold1Cufflinks	exon	143268	143360	.	+	.	gene_id "XLOC_246447"; transcript_id "Novel_003474"; exon_number "2"; gene_type "
scaffold1Cufflinks	exon	143481	143546	.	+	.	gene_id "XLOC_246447"; transcript_id "Novel_003474"; exon_number "3"; gene_type "
scaffold1Cufflinks	exon	28	90	.	+	.	gene_id "XLOC_246666"; transcript_id "Novel_003475"; exon_number "1"; gene_type "
scaffold1Cufflinks	exon	1682	1750	.	+	.	gene_id "XLOC_246666"; transcript_id "Novel_003475"; exon_number "2"; gene_type "
scaffold1Cufflinks	exon	1859	2023	.	+	.	gene_id "XLOC_246666"; transcript_id "Novel_003475"; exon_number "3"; gene_type "
scaffold1Cufflinks	exon	113890	114166	.	+	.	gene_id "XLOC_246684"; transcript_id "Novel_003476"; exon_number "1"; gene_type "
scaffold1Cufflinks	exon	114310	115309	.	+	.	gene_id "XLOC_246684"; transcript_id "Novel_003476"; exon_number "2"; gene_type "
scaffold1Cufflinks	exon	437312	437463	.	+	.	gene_id "XLOC_246706"; transcript_id "Novel_003477"; exon_number "1"; gene_type "
scaffold1Cufflinks	exon	437543	437987	.	+	.	gene_id "XLOC_246706"; transcript_id "Novel_003477"; exon_number "2"; gene_type "
scaffold1Cufflinks	exon	438192	438253	.	+	.	gene_id "XLOC_246706"; transcript_id "Novel_003477"; exon_number "3"; gene_type "
scaffold1Cufflinks	exon	438343	438417	.	+	.	gene_id "XLOC_246706"; transcript_id "Novel_003477"; exon_number "4"; gene_type "
scaffold1Cufflinks	exon	438521	438686	.	+	.	gene_id "XLOC_246706"; transcript_id "Novel_003477"; exon_number "5"; gene_type "
scaffold1Cufflinks	exon	438768	439426	.	+	.	gene_id "XLOC_246706"; transcript_id "Novel_003477"; exon_number "6"; gene_type "
scaffold1Cufflinks	exon	439775	440022	.	+	.	gene_id "XLOC_246707"; transcript_id "Novel_003478"; exon_number "1"; gene_type "
scaffold1Cufflinks	exon	440169	440325	.	+	.	gene_id "XLOC_246707"; transcript_id "Novel_003478"; exon_number "2"; gene_type "
scaffold1Cufflinks	exon	440442	440629	.	+	.	gene_id "XLOC_246707"; transcript_id "Novel_003478"; exon_number "3"; gene_type "
scaffold1Cufflinks	exon	747101	747167	.	+	.	gene_id "XLOC_246748"; transcript_id "Novel_003479"; exon_number "1"; gene_type "
scaffold1Cufflinks	exon	747260	747925	.	+	.	gene_id "XLOC_246748"; transcript_id "Novel_003479"; exon_number "2"; gene_type "
scaffold1Cufflinks	exon	782526	782886	.	+	.	gene_id "XLOC_246758"; transcript_id "Novel_003480"; exon_number "1"; gene_type "
scaffold1Cufflinks	exon	783703	786082	.	+	.	gene_id "XLOC_246758"; transcript_id "Novel_003480"; exon_number "2"; gene_type "

scaffold1	Cufflinks	exon	969112	969391	.	+	.	gene_id "XLOC_246781"; transcript_id "Novel_003481"; exon_number "1"; gene_type "
scaffold1	Cufflinks	exon	970196	970232	.	+	.	gene_id "XLOC_246781"; transcript_id "Novel_003481"; exon_number "2"; gene_type "
scaffold1	Cufflinks	exon	1177332	1177705	.	+	.	gene_id "XLOC_246814"; transcript_id "Novel_003482"; exon_number "1"; gene_type "
scaffold1	Cufflinks	exon	1178601	1178890	.	+	.	gene_id "XLOC_246814"; transcript_id "Novel_003482"; exon_number "2"; gene_type "
scaffold1	Cufflinks	exon	1178965	1179156	.	+	.	gene_id "XLOC_246814"; transcript_id "Novel_003482"; exon_number "3"; gene_type "
scaffold1	Cufflinks	exon	1177372	1177705	.	+	.	gene_id "XLOC_246814"; transcript_id "Novel_003483"; exon_number "1"; gene_type "
scaffold1	Cufflinks	exon	1178601	1178846	.	+	.	gene_id "XLOC_246814"; transcript_id "Novel_003483"; exon_number "2"; gene_type "
scaffold1	Cufflinks	exon	1178965	1179201	.	+	.	gene_id "XLOC_246814"; transcript_id "Novel_003483"; exon_number "3"; gene_type "
scaffold1	Cufflinks	exon	1179432	1179784	.	+	.	gene_id "XLOC_246814"; transcript_id "Novel_003484"; exon_number "1"; gene_type "
scaffold1	Cufflinks	exon	1179898	1180911	.	+	.	gene_id "XLOC_246814"; transcript_id "Novel_003484"; exon_number "2"; gene_type "
scaffold1	Cufflinks	exon	1304803	1304906	.	+	.	gene_id "XLOC_246829"; transcript_id "Novel_003485"; exon_number "1"; gene_type "
scaffold1	Cufflinks	exon	1305031	1305156	.	+	.	gene_id "XLOC_246829"; transcript_id "Novel_003485"; exon_number "2"; gene_type "
scaffold1	Cufflinks	exon	1305793	1305883	.	+	.	gene_id "XLOC_246829"; transcript_id "Novel_003485"; exon_number "3"; gene_type "
scaffold1	Cufflinks	exon	1306176	1306562	.	+	.	gene_id "XLOC_246829"; transcript_id "Novel_003485"; exon_number "4"; gene_type "
scaffold1	Cufflinks	exon	1421955	1422375	.	+	.	gene_id "XLOC_246839"; transcript_id "Novel_003486"; exon_number "1"; gene_type "
scaffold1	Cufflinks	exon	1422967	1423307	.	+	.	gene_id "XLOC_246839"; transcript_id "Novel_003486"; exon_number "2"; gene_type "
scaffold1	Cufflinks	exon	1594982	1595134	.	+	.	gene_id "XLOC_246870"; transcript_id "Novel_003487"; exon_number "1"; gene_type "
scaffold1	Cufflinks	exon	1595276	1596056	.	+	.	gene_id "XLOC_246870"; transcript_id "Novel_003487"; exon_number "2"; gene_type "
scaffold1	Cufflinks	exon	1596155	1596251	.	+	.	gene_id "XLOC_246870"; transcript_id "Novel_003487"; exon_number "3"; gene_type "
scaffold1	Cufflinks	exon	1596342	1597180	.	+	.	gene_id "XLOC_246870"; transcript_id "Novel_003487"; exon_number "4"; gene_type "
scaffold1	Cufflinks	exon	1594982	1595134	.	+	.	gene_id "XLOC_246870"; transcript_id "Novel_003488"; exon_number "1"; gene_type "
scaffold1	Cufflinks	exon	1595276	1595751	.	+	.	gene_id "XLOC_246870"; transcript_id "Novel_003488"; exon_number "2"; gene_type "
scaffold1	Cufflinks	exon	1595884	1596056	.	+	.	gene_id "XLOC_246870"; transcript_id "Novel_003488"; exon_number "3"; gene_type "
scaffold1	Cufflinks	exon	1596155	1596251	.	+	.	gene_id "XLOC_246870"; transcript_id "Novel_003488"; exon_number "4"; gene_type "
scaffold1	Cufflinks	exon	1596342	1597180	.	+	.	gene_id "XLOC_246870"; transcript_id "Novel_003488"; exon_number "5"; gene_type "
scaffold1	Cufflinks	exon	1682020	1682168	.	+	.	gene_id "XLOC_246870"; transcript_id "Novel_003489"; exon_number "1"; gene_type "
scaffold1	Cufflinks	exon	1682241	1682337	.	+	.	gene_id "XLOC_246870"; transcript_id "Novel_003489"; exon_number "2"; gene_type "
scaffold1	Cufflinks	exon	1682421	1682574	.	+	.	gene_id "XLOC_246870"; transcript_id "Novel_003489"; exon_number "3"; gene_type "
scaffold1	Cufflinks	exon	1682737	1683182	.	+	.	gene_id "XLOC_246870"; transcript_id "Novel_003489"; exon_number "4"; gene_type "
scaffold1	Cufflinks	exon	1844747	1845500	.	+	.	gene_id "XLOC_246907"; transcript_id "Novel_003490"; exon_number "1"; gene_type "
scaffold1	Cufflinks	exon	1846039	1846365	.	+	.	gene_id "XLOC_246908"; transcript_id "Novel_003491"; exon_number "1"; gene_type "
scaffold1	Cufflinks	exon	86583	87385	.	-	.	gene_id "XLOC_246939"; transcript_id "Novel_003492"; exon_number "1"; gene_type "
scaffold1	Cufflinks	exon	90597	91205	.	-	.	gene_id "XLOC_246939"; transcript_id "Novel_003492"; exon_number "2"; gene_type "
scaffold1	Cufflinks	exon	288482	288509	.	-	.	gene_id "XLOC_246969"; transcript_id "Novel_003493"; exon_number "1"; gene_type "
scaffold1	Cufflinks	exon	288638	288749	.	-	.	gene_id "XLOC_246969"; transcript_id "Novel_003493"; exon_number "2"; gene_type "

scaffold1	Cufflinks	exon	288848	289098	.	-	.	gene_id "XLOC_246969"; transcript_id "Novel_003493"; exon_number "3"; gene_type "
scaffold1	Cufflinks	exon	348124	348217	.	-	.	gene_id "XLOC_246981"; transcript_id "Novel_003494"; exon_number "1"; gene_type "
scaffold1	Cufflinks	exon	348756	349009	.	-	.	gene_id "XLOC_246981"; transcript_id "Novel_003494"; exon_number "2"; gene_type "
scaffold1	Cufflinks	exon	350524	350595	.	-	.	gene_id "XLOC_246981"; transcript_id "Novel_003494"; exon_number "3"; gene_type "
scaffold1	Cufflinks	exon	350686	350772	.	-	.	gene_id "XLOC_246981"; transcript_id "Novel_003494"; exon_number "4"; gene_type "
scaffold1	Cufflinks	exon	350877	351050	.	-	.	gene_id "XLOC_246981"; transcript_id "Novel_003494"; exon_number "5"; gene_type "
scaffold1	Cufflinks	exon	520451	520569	.	-	.	gene_id "XLOC_246990"; transcript_id "Novel_003495"; exon_number "1"; gene_type "
scaffold1	Cufflinks	exon	521845	521955	.	-	.	gene_id "XLOC_246990"; transcript_id "Novel_003495"; exon_number "2"; gene_type "
scaffold1	Cufflinks	exon	522458	523323	.	-	.	gene_id "XLOC_246990"; transcript_id "Novel_003495"; exon_number "3"; gene_type "
scaffold1	Cufflinks	exon	644965	645973	.	-	.	gene_id "XLOC_247027"; transcript_id "Novel_003496"; exon_number "1"; gene_type "
scaffold1	Cufflinks	exon	649074	649167	.	-	.	gene_id "XLOC_247027"; transcript_id "Novel_003496"; exon_number "2"; gene_type "
scaffold1	Cufflinks	exon	645570	645984	.	-	.	gene_id "XLOC_247027"; transcript_id "Novel_003497"; exon_number "1"; gene_type "
scaffold1	Cufflinks	exon	649074	649194	.	-	.	gene_id "XLOC_247027"; transcript_id "Novel_003497"; exon_number "2"; gene_type "
scaffold1	Cufflinks	exon	757996	759071	.	-	.	gene_id "XLOC_247036"; transcript_id "Novel_003498"; exon_number "1"; gene_type "
scaffold1	Cufflinks	exon	759184	759255	.	-	.	gene_id "XLOC_247036"; transcript_id "Novel_003498"; exon_number "2"; gene_type "
scaffold1	Cufflinks	exon	759367	760164	.	-	.	gene_id "XLOC_247036"; transcript_id "Novel_003498"; exon_number "3"; gene_type "
scaffold1	Cufflinks	exon	761459	761610	.	-	.	gene_id "XLOC_247036"; transcript_id "Novel_003498"; exon_number "4"; gene_type "
scaffold1	Cufflinks	exon	758736	759728	.	-	.	gene_id "XLOC_247036"; transcript_id "Novel_003499"; exon_number "1"; gene_type "
scaffold1	Cufflinks	exon	759954	760164	.	-	.	gene_id "XLOC_247036"; transcript_id "Novel_003499"; exon_number "2"; gene_type "
scaffold1	Cufflinks	exon	761459	761571	.	-	.	gene_id "XLOC_247036"; transcript_id "Novel_003499"; exon_number "3"; gene_type "
scaffold1	Cufflinks	exon	1480864	1481695	.	-	.	gene_id "XLOC_247079"; transcript_id "Novel_003500"; exon_number "1"; gene_type "
scaffold1	Cufflinks	exon	1731948	1732274	.	-	.	gene_id "XLOC_247099"; transcript_id "Novel_003501"; exon_number "1"; gene_type "
scaffold1	Cufflinks	exon	1732955	1733032	.	-	.	gene_id "XLOC_247099"; transcript_id "Novel_003501"; exon_number "2"; gene_type "
scaffold1	Cufflinks	exon	1733127	1733173	.	-	.	gene_id "XLOC_247099"; transcript_id "Novel_003501"; exon_number "3"; gene_type "
scaffold1	Cufflinks	exon	1733260	1733439	.	-	.	gene_id "XLOC_247099"; transcript_id "Novel_003501"; exon_number "4"; gene_type "
scaffold1	Cufflinks	exon	1733644	1733769	.	-	.	gene_id "XLOC_247099"; transcript_id "Novel_003501"; exon_number "5"; gene_type "
scaffold1	Cufflinks	exon	1734019	1734105	.	-	.	gene_id "XLOC_247099"; transcript_id "Novel_003501"; exon_number "6"; gene_type "
scaffold1	Cufflinks	exon	1734347	1734744	.	-	.	gene_id "XLOC_247099"; transcript_id "Novel_003501"; exon_number "7"; gene_type "
scaffold1	Cufflinks	exon	478656	478867	.	-	.	gene_id "XLOC_247265"; transcript_id "Novel_003502"; exon_number "1"; gene_type "
scaffold1	Cufflinks	exon	509155	511119	.	+	.	gene_id "XLOC_247429"; transcript_id "Novel_003503"; exon_number "1"; gene_type "
scaffold1	Cufflinks	exon	513646	513972	.	+	.	gene_id "XLOC_247429"; transcript_id "Novel_003503"; exon_number "2"; gene_type "
scaffold1	Cufflinks	exon	514062	514126	.	+	.	gene_id "XLOC_247429"; transcript_id "Novel_003503"; exon_number "3"; gene_type "
scaffold1	Cufflinks	exon	438573	438974	.	-	.	gene_id "XLOC_247499"; transcript_id "Novel_003504"; exon_number "1"; gene_type "
scaffold1	Cufflinks	exon	439058	440285	.	-	.	gene_id "XLOC_247499"; transcript_id "Novel_003504"; exon_number "2"; gene_type "
scaffold1	Cufflinks	exon	33140	33313	.	+	.	gene_id "XLOC_247544"; transcript_id "Novel_003505"; exon_number "1"; gene_type "

scaffold1Cufflinks	exon	33641	34261	.	+	.	gene_id "XLOC_247544"; transcript_id "Novel_003505"; exon_number "2"; gene_type "
scaffold1Cufflinks	exon	404940	405511	.	+	.	gene_id "XLOC_247590"; transcript_id "Novel_003506"; exon_number "1"; gene_type "
scaffold1Cufflinks	exon	131688	131968	.	+	.	gene_id "XLOC_247746"; transcript_id "Novel_003507"; exon_number "1"; gene_type "
scaffold1Cufflinks	exon	150316	152178	.	+	.	gene_id "XLOC_247746"; transcript_id "Novel_003507"; exon_number "2"; gene_type "
scaffold1Cufflinks	exon	154016	154265	.	+	.	gene_id "XLOC_247746"; transcript_id "Novel_003507"; exon_number "3"; gene_type "
scaffold1Cufflinks	exon	131690	131968	.	+	.	gene_id "XLOC_247746"; transcript_id "Novel_003508"; exon_number "1"; gene_type "
scaffold1Cufflinks	exon	150316	152563	.	+	.	gene_id "XLOC_247746"; transcript_id "Novel_003508"; exon_number "2"; gene_type "
scaffold1Cufflinks	exon	133675	133953	.	+	.	gene_id "XLOC_247746"; transcript_id "Novel_003509"; exon_number "1"; gene_type "
scaffold1Cufflinks	exon	150316	152178	.	+	.	gene_id "XLOC_247746"; transcript_id "Novel_003509"; exon_number "2"; gene_type "
scaffold1Cufflinks	exon	154016	154255	.	+	.	gene_id "XLOC_247746"; transcript_id "Novel_003509"; exon_number "3"; gene_type "
scaffold1Cufflinks	exon	305499	305979	.	+	.	gene_id "XLOC_247777"; transcript_id "Novel_003510"; exon_number "1"; gene_type "
scaffold1Cufflinks	exon	306086	306298	.	+	.	gene_id "XLOC_247777"; transcript_id "Novel_003510"; exon_number "2"; gene_type "
scaffold1Cufflinks	exon	307312	307337	.	+	.	gene_id "XLOC_247777"; transcript_id "Novel_003510"; exon_number "3"; gene_type "
scaffold1Cufflinks	exon	307412	307648	.	+	.	gene_id "XLOC_247777"; transcript_id "Novel_003510"; exon_number "4"; gene_type "
scaffold1Cufflinks	exon	307288	307337	.	+	.	gene_id "XLOC_247777"; transcript_id "Novel_003511"; exon_number "1"; gene_type "
scaffold1Cufflinks	exon	307412	307648	.	+	.	gene_id "XLOC_247777"; transcript_id "Novel_003511"; exon_number "2"; gene_type "
scaffold1Cufflinks	exon	31284	32051	.	-	.	gene_id "XLOC_247803"; transcript_id "Novel_003512"; exon_number "1"; gene_type "
scaffold1Cufflinks	exon	32631	32914	.	-	.	gene_id "XLOC_247803"; transcript_id "Novel_003512"; exon_number "2"; gene_type "
scaffold1Cufflinks	exon	33001	33365	.	-	.	gene_id "XLOC_247803"; transcript_id "Novel_003512"; exon_number "3"; gene_type "
scaffold1Cufflinks	exon	453642	454766	.	-	.	gene_id "XLOC_247847"; transcript_id "Novel_003513"; exon_number "1"; gene_type "
scaffold1Cufflinks	exon	456945	457136	.	-	.	gene_id "XLOC_247847"; transcript_id "Novel_003513"; exon_number "2"; gene_type "
scaffold1Cufflinks	exon	445209	445417	.	-	.	gene_id "XLOC_247848"; transcript_id "Novel_003514"; exon_number "1"; gene_type "
scaffold1Cufflinks	exon	20	234	.	-	.	gene_id "XLOC_247944"; transcript_id "Novel_003515"; exon_number "1"; gene_type "
scaffold1Cufflinks	exon	320	1287	.	-	.	gene_id "XLOC_247944"; transcript_id "Novel_003515"; exon_number "2"; gene_type "
scaffold1Cufflinks	exon	1728	1881	.	-	.	gene_id "XLOC_247944"; transcript_id "Novel_003515"; exon_number "3"; gene_type "
scaffold1Cufflinks	exon	20	561	.	-	.	gene_id "XLOC_247944"; transcript_id "Novel_003516"; exon_number "1"; gene_type "
scaffold1Cufflinks	exon	1202	1287	.	-	.	gene_id "XLOC_247944"; transcript_id "Novel_003516"; exon_number "2"; gene_type "
scaffold1Cufflinks	exon	1728	1881	.	-	.	gene_id "XLOC_247944"; transcript_id "Novel_003516"; exon_number "3"; gene_type "
scaffold1Cufflinks	exon	48	83	.	-	.	gene_id "XLOC_247944"; transcript_id "Novel_003517"; exon_number "1"; gene_type "
scaffold1Cufflinks	exon	172	234	.	-	.	gene_id "XLOC_247944"; transcript_id "Novel_003517"; exon_number "2"; gene_type "
scaffold1Cufflinks	exon	320	392	.	-	.	gene_id "XLOC_247944"; transcript_id "Novel_003517"; exon_number "3"; gene_type "
scaffold1Cufflinks	exon	497	561	.	-	.	gene_id "XLOC_247944"; transcript_id "Novel_003517"; exon_number "4"; gene_type "
scaffold1Cufflinks	exon	1202	1287	.	-	.	gene_id "XLOC_247944"; transcript_id "Novel_003517"; exon_number "5"; gene_type "
scaffold1Cufflinks	exon	1728	1840	.	-	.	gene_id "XLOC_247944"; transcript_id "Novel_003517"; exon_number "6"; gene_type "
scaffold1Cufflinks	exon	206606	206806	.	+	.	gene_id "XLOC_248132"; transcript_id "Novel_003518"; exon_number "1"; gene_type "

scaffold1Cufflinks	exon	232695	233968	.	-	.	gene_id "XLOC_248145"; transcript_id "Novel_003519"; exon_number "1"; gene_type "
scaffold1Cufflinks	exon	236071	236198	.	-	.	gene_id "XLOC_248145"; transcript_id "Novel_003519"; exon_number "2"; gene_type "
scaffold1Cufflinks	exon	236289	236654	.	-	.	gene_id "XLOC_248145"; transcript_id "Novel_003519"; exon_number "3"; gene_type "
scaffold1Cufflinks	exon	233496	233968	.	-	.	gene_id "XLOC_248145"; transcript_id "Novel_003520"; exon_number "1"; gene_type "
scaffold1Cufflinks	exon	236071	236198	.	-	.	gene_id "XLOC_248145"; transcript_id "Novel_003520"; exon_number "2"; gene_type "
scaffold1Cufflinks	exon	236289	236389	.	-	.	gene_id "XLOC_248145"; transcript_id "Novel_003520"; exon_number "3"; gene_type "
scaffold1Cufflinks	exon	236465	236648	.	-	.	gene_id "XLOC_248145"; transcript_id "Novel_003520"; exon_number "4"; gene_type "
scaffold1Cufflinks	exon	234546	236198	.	-	.	gene_id "XLOC_248145"; transcript_id "Novel_003521"; exon_number "1"; gene_type "
scaffold1Cufflinks	exon	236289	236389	.	-	.	gene_id "XLOC_248145"; transcript_id "Novel_003521"; exon_number "2"; gene_type "
scaffold1Cufflinks	exon	236465	236626	.	-	.	gene_id "XLOC_248145"; transcript_id "Novel_003521"; exon_number "3"; gene_type "
scaffold1Cufflinks	exon	1622865	1623396	.	-	.	gene_id "XLOC_248744"; transcript_id "Novel_003522"; exon_number "1"; gene_type "
scaffold1Cufflinks	exon	1624538	1625683	.	-	.	gene_id "XLOC_248744"; transcript_id "Novel_003522"; exon_number "2"; gene_type "
scaffold1Cufflinks	exon	1622865	1623378	.	-	.	gene_id "XLOC_248744"; transcript_id "Novel_003523"; exon_number "1"; gene_type "
scaffold1Cufflinks	exon	1624538	1625683	.	-	.	gene_id "XLOC_248744"; transcript_id "Novel_003523"; exon_number "2"; gene_type "
scaffold1Cufflinks	exon	193468	193634	.	-	.	gene_id "XLOC_248938"; transcript_id "Novel_003524"; exon_number "1"; gene_type "
scaffold1Cufflinks	exon	194048	194236	.	-	.	gene_id "XLOC_248938"; transcript_id "Novel_003524"; exon_number "2"; gene_type "
scaffold1Cufflinks	exon	194330	194480	.	-	.	gene_id "XLOC_248938"; transcript_id "Novel_003524"; exon_number "3"; gene_type "
scaffold1Cufflinks	exon	93368	94084	.	+	.	gene_id "XLOC_249006"; transcript_id "Novel_003525"; exon_number "1"; gene_type "
scaffold1Cufflinks	exon	154263	154410	.	+	.	gene_id "XLOC_249014"; transcript_id "Novel_003526"; exon_number "1"; gene_type "
scaffold1Cufflinks	exon	154503	154633	.	+	.	gene_id "XLOC_249014"; transcript_id "Novel_003526"; exon_number "2"; gene_type "
scaffold1Cufflinks	exon	155335	155462	.	+	.	gene_id "XLOC_249014"; transcript_id "Novel_003526"; exon_number "3"; gene_type "
scaffold1Cufflinks	exon	155543	155647	.	+	.	gene_id "XLOC_249014"; transcript_id "Novel_003526"; exon_number "4"; gene_type "
scaffold1Cufflinks	exon	156064	156416	.	+	.	gene_id "XLOC_249014"; transcript_id "Novel_003526"; exon_number "5"; gene_type "
scaffold1Cufflinks	exon	331842	332250	.	+	.	gene_id "XLOC_249572"; transcript_id "Novel_003527"; exon_number "1"; gene_type "
scaffold1Cufflinks	exon	344723	344947	.	+	.	gene_id "XLOC_249577"; transcript_id "Novel_003528"; exon_number "1"; gene_type "
scaffold1Cufflinks	exon	345801	346884	.	+	.	gene_id "XLOC_249577"; transcript_id "Novel_003528"; exon_number "2"; gene_type "
scaffold1Cufflinks	exon	226844	227086	.	+	.	gene_id "XLOC_249661"; transcript_id "Novel_003529"; exon_number "1"; gene_type "
scaffold1Cufflinks	exon	227170	227938	.	+	.	gene_id "XLOC_249661"; transcript_id "Novel_003529"; exon_number "2"; gene_type "
scaffold1Cufflinks	exon	53844	54054	.	+	.	gene_id "XLOC_249752"; transcript_id "Novel_003530"; exon_number "1"; gene_type "
scaffold1Cufflinks	exon	72576	72840	.	+	.	gene_id "XLOC_249757"; transcript_id "Novel_003531"; exon_number "1"; gene_type "
scaffold1Cufflinks	exon	1581435	1582797	.	+	.	gene_id "XLOC_250053"; transcript_id "Novel_003532"; exon_number "1"; gene_type "
scaffold1Cufflinks	exon	1582960	1583997	.	+	.	gene_id "XLOC_250053"; transcript_id "Novel_003532"; exon_number "2"; gene_type "
scaffold1Cufflinks	exon	1581442	1581569	.	+	.	gene_id "XLOC_250053"; transcript_id "Novel_003533"; exon_number "1"; gene_type "
scaffold1Cufflinks	exon	1581892	1582797	.	+	.	gene_id "XLOC_250053"; transcript_id "Novel_003533"; exon_number "2"; gene_type "
scaffold1Cufflinks	exon	1582960	1583946	.	+	.	gene_id "XLOC_250053"; transcript_id "Novel_003533"; exon_number "3"; gene_type "

scaffold1	Cufflinks	exon	310438	312742	.	-	.	gene_id "XLOC_250107"; transcript_id "Novel_003534"; exon_number "1"; gene_type "
scaffold1	Cufflinks	exon	312849	313482	.	-	.	gene_id "XLOC_250107"; transcript_id "Novel_003534"; exon_number "2"; gene_type "
scaffold1	Cufflinks	exon	1443046	1444353	.	-	.	gene_id "XLOC_250224"; transcript_id "Novel_003535"; exon_number "1"; gene_type "
scaffold1	Cufflinks	exon	1444473	1445464	.	-	.	gene_id "XLOC_250224"; transcript_id "Novel_003535"; exon_number "2"; gene_type "
scaffold1	Cufflinks	exon	1443046	1444353	.	-	.	gene_id "XLOC_250224"; transcript_id "Novel_003536"; exon_number "1"; gene_type "
scaffold1	Cufflinks	exon	1444473	1444836	.	-	.	gene_id "XLOC_250224"; transcript_id "Novel_003536"; exon_number "2"; gene_type "
scaffold1	Cufflinks	exon	1445188	1445464	.	-	.	gene_id "XLOC_250224"; transcript_id "Novel_003536"; exon_number "3"; gene_type "
scaffold1	Cufflinks	exon	1443214	1444353	.	-	.	gene_id "XLOC_250224"; transcript_id "Novel_003537"; exon_number "1"; gene_type "
scaffold1	Cufflinks	exon	1444473	1444832	.	-	.	gene_id "XLOC_250224"; transcript_id "Novel_003537"; exon_number "2"; gene_type "
scaffold1	Cufflinks	exon	1445188	1445304	.	-	.	gene_id "XLOC_250224"; transcript_id "Novel_003537"; exon_number "3"; gene_type "
scaffold1	Cufflinks	exon	27277	29140	.	-	.	gene_id "XLOC_250282"; transcript_id "Novel_003538"; exon_number "1"; gene_type "
scaffold1	Cufflinks	exon	29683	29770	.	-	.	gene_id "XLOC_250282"; transcript_id "Novel_003538"; exon_number "2"; gene_type "
scaffold1	Cufflinks	exon	29871	29966	.	-	.	gene_id "XLOC_250282"; transcript_id "Novel_003538"; exon_number "3"; gene_type "
scaffold1	Cufflinks	exon	30247	30397	.	-	.	gene_id "XLOC_250282"; transcript_id "Novel_003538"; exon_number "4"; gene_type "
scaffold1	Cufflinks	exon	30908	30987	.	-	.	gene_id "XLOC_250282"; transcript_id "Novel_003538"; exon_number "5"; gene_type "
scaffold1	Cufflinks	exon	31226	31401	.	-	.	gene_id "XLOC_250282"; transcript_id "Novel_003538"; exon_number "6"; gene_type "
scaffold1	Cufflinks	exon	31619	31910	.	-	.	gene_id "XLOC_250282"; transcript_id "Novel_003538"; exon_number "7"; gene_type "
scaffold1	Cufflinks	exon	27277	29140	.	-	.	gene_id "XLOC_250282"; transcript_id "Novel_003539"; exon_number "1"; gene_type "
scaffold1	Cufflinks	exon	29683	29770	.	-	.	gene_id "XLOC_250282"; transcript_id "Novel_003539"; exon_number "2"; gene_type "
scaffold1	Cufflinks	exon	29871	29966	.	-	.	gene_id "XLOC_250282"; transcript_id "Novel_003539"; exon_number "3"; gene_type "
scaffold1	Cufflinks	exon	30247	30397	.	-	.	gene_id "XLOC_250282"; transcript_id "Novel_003539"; exon_number "4"; gene_type "
scaffold1	Cufflinks	exon	30465	30524	.	-	.	gene_id "XLOC_250282"; transcript_id "Novel_003539"; exon_number "5"; gene_type "
scaffold1	Cufflinks	exon	30908	30987	.	-	.	gene_id "XLOC_250282"; transcript_id "Novel_003539"; exon_number "6"; gene_type "
scaffold1	Cufflinks	exon	31226	31401	.	-	.	gene_id "XLOC_250282"; transcript_id "Novel_003539"; exon_number "7"; gene_type "
scaffold1	Cufflinks	exon	31619	31910	.	-	.	gene_id "XLOC_250282"; transcript_id "Novel_003539"; exon_number "8"; gene_type "
scaffold1	Cufflinks	exon	27296	29140	.	-	.	gene_id "XLOC_250282"; transcript_id "Novel_003540"; exon_number "1"; gene_type "
scaffold1	Cufflinks	exon	29399	29556	.	-	.	gene_id "XLOC_250282"; transcript_id "Novel_003540"; exon_number "2"; gene_type "
scaffold1	Cufflinks	exon	29683	29770	.	-	.	gene_id "XLOC_250282"; transcript_id "Novel_003540"; exon_number "3"; gene_type "
scaffold1	Cufflinks	exon	29871	29966	.	-	.	gene_id "XLOC_250282"; transcript_id "Novel_003540"; exon_number "4"; gene_type "
scaffold1	Cufflinks	exon	30247	30397	.	-	.	gene_id "XLOC_250282"; transcript_id "Novel_003540"; exon_number "5"; gene_type "
scaffold1	Cufflinks	exon	30908	30987	.	-	.	gene_id "XLOC_250282"; transcript_id "Novel_003540"; exon_number "6"; gene_type "
scaffold1	Cufflinks	exon	31226	31401	.	-	.	gene_id "XLOC_250282"; transcript_id "Novel_003540"; exon_number "7"; gene_type "
scaffold1	Cufflinks	exon	31619	31908	.	-	.	gene_id "XLOC_250282"; transcript_id "Novel_003540"; exon_number "8"; gene_type "
scaffold1	Cufflinks	exon	28667	29770	.	-	.	gene_id "XLOC_250282"; transcript_id "Novel_003541"; exon_number "1"; gene_type "
scaffold1	Cufflinks	exon	29871	29966	.	-	.	gene_id "XLOC_250282"; transcript_id "Novel_003541"; exon_number "2"; gene_type "

scaffold1	Cufflinks	exon	30247	30397	.	-	.	gene_id "XLOC_250282"; transcript_id "Novel_003541"; exon_number "3"; gene_type "
scaffold1	Cufflinks	exon	30465	30524	.	-	.	gene_id "XLOC_250282"; transcript_id "Novel_003541"; exon_number "4"; gene_type "
scaffold1	Cufflinks	exon	30908	30987	.	-	.	gene_id "XLOC_250282"; transcript_id "Novel_003541"; exon_number "5"; gene_type "
scaffold1	Cufflinks	exon	31226	31401	.	-	.	gene_id "XLOC_250282"; transcript_id "Novel_003541"; exon_number "6"; gene_type "
scaffold1	Cufflinks	exon	31619	31913	.	-	.	gene_id "XLOC_250282"; transcript_id "Novel_003541"; exon_number "7"; gene_type "
scaffold1	Cufflinks	exon	28814	29140	.	-	.	gene_id "XLOC_250282"; transcript_id "Novel_003542"; exon_number "1"; gene_type "
scaffold1	Cufflinks	exon	29399	29770	.	-	.	gene_id "XLOC_250282"; transcript_id "Novel_003542"; exon_number "2"; gene_type "
scaffold1	Cufflinks	exon	29871	29966	.	-	.	gene_id "XLOC_250282"; transcript_id "Novel_003542"; exon_number "3"; gene_type "
scaffold1	Cufflinks	exon	30247	30397	.	-	.	gene_id "XLOC_250282"; transcript_id "Novel_003542"; exon_number "4"; gene_type "
scaffold1	Cufflinks	exon	30465	30524	.	-	.	gene_id "XLOC_250282"; transcript_id "Novel_003542"; exon_number "5"; gene_type "
scaffold1	Cufflinks	exon	30908	30987	.	-	.	gene_id "XLOC_250282"; transcript_id "Novel_003542"; exon_number "6"; gene_type "
scaffold1	Cufflinks	exon	31226	31401	.	-	.	gene_id "XLOC_250282"; transcript_id "Novel_003542"; exon_number "7"; gene_type "
scaffold1	Cufflinks	exon	31619	31914	.	-	.	gene_id "XLOC_250282"; transcript_id "Novel_003542"; exon_number "8"; gene_type "
scaffold1	Cufflinks	exon	220872	221196	.	+	.	gene_id "XLOC_250331"; transcript_id "Novel_003543"; exon_number "1"; gene_type "
scaffold1	Cufflinks	exon	221278	221303	.	+	.	gene_id "XLOC_250331"; transcript_id "Novel_003543"; exon_number "2"; gene_type "
scaffold1	Cufflinks	exon	222078	222167	.	+	.	gene_id "XLOC_250331"; transcript_id "Novel_003543"; exon_number "3"; gene_type "
scaffold1	Cufflinks	exon	222266	222354	.	+	.	gene_id "XLOC_250331"; transcript_id "Novel_003543"; exon_number "4"; gene_type "
scaffold1	Cufflinks	exon	223111	223579	.	+	.	gene_id "XLOC_250331"; transcript_id "Novel_003543"; exon_number "5"; gene_type "
scaffold1	Cufflinks	exon	74556	74786	.	+	.	gene_id "XLOC_250494"; transcript_id "Novel_003544"; exon_number "1"; gene_type "
scaffold1	Cufflinks	exon	74879	75068	.	+	.	gene_id "XLOC_250494"; transcript_id "Novel_003544"; exon_number "2"; gene_type "
scaffold1	Cufflinks	exon	75983	76126	.	+	.	gene_id "XLOC_250494"; transcript_id "Novel_003544"; exon_number "3"; gene_type "
scaffold1	Cufflinks	exon	1711721	1712102	.	+	.	gene_id "XLOC_250706"; transcript_id "Novel_003545"; exon_number "1"; gene_type "
scaffold1	Cufflinks	exon	1712857	1712986	.	+	.	gene_id "XLOC_250706"; transcript_id "Novel_003545"; exon_number "2"; gene_type "
scaffold1	Cufflinks	exon	1713742	1713879	.	+	.	gene_id "XLOC_250706"; transcript_id "Novel_003545"; exon_number "3"; gene_type "
scaffold1	Cufflinks	exon	1714273	1714605	.	+	.	gene_id "XLOC_250706"; transcript_id "Novel_003545"; exon_number "4"; gene_type "
scaffold1	Cufflinks	exon	1714964	1715147	.	+	.	gene_id "XLOC_250706"; transcript_id "Novel_003545"; exon_number "5"; gene_type "
scaffold1	Cufflinks	exon	1715236	1715937	.	+	.	gene_id "XLOC_250706"; transcript_id "Novel_003545"; exon_number "6"; gene_type "
scaffold1	Cufflinks	exon	1711832	1712102	.	+	.	gene_id "XLOC_250706"; transcript_id "Novel_003546"; exon_number "1"; gene_type "
scaffold1	Cufflinks	exon	1712857	1714605	.	+	.	gene_id "XLOC_250706"; transcript_id "Novel_003546"; exon_number "2"; gene_type "
scaffold1	Cufflinks	exon	1714964	1715147	.	+	.	gene_id "XLOC_250706"; transcript_id "Novel_003546"; exon_number "3"; gene_type "
scaffold1	Cufflinks	exon	1715236	1715906	.	+	.	gene_id "XLOC_250706"; transcript_id "Novel_003546"; exon_number "4"; gene_type "
scaffold1	Cufflinks	exon	1712567	1712986	.	+	.	gene_id "XLOC_250706"; transcript_id "Novel_003547"; exon_number "1"; gene_type "
scaffold1	Cufflinks	exon	1713742	1713879	.	+	.	gene_id "XLOC_250706"; transcript_id "Novel_003547"; exon_number "2"; gene_type "
scaffold1	Cufflinks	exon	1714273	1714605	.	+	.	gene_id "XLOC_250706"; transcript_id "Novel_003547"; exon_number "3"; gene_type "
scaffold1	Cufflinks	exon	1714964	1715147	.	+	.	gene_id "XLOC_250706"; transcript_id "Novel_003547"; exon_number "4"; gene_type "

scaffold1	Cufflinks	exon	1715236	1715906	.	+	.	gene_id "XLOC_250706"; transcript_id "Novel_003547"; exon_number "5"; gene_type "
scaffold1	Cufflinks	exon	1270570	1272465	.	-	.	gene_id "XLOC_250903"; transcript_id "Novel_003548"; exon_number "1"; gene_type "
scaffold1	Cufflinks	exon	1272560	1273419	.	-	.	gene_id "XLOC_250903"; transcript_id "Novel_003548"; exon_number "2"; gene_type "
scaffold1	Cufflinks	exon	1270570	1272465	.	-	.	gene_id "XLOC_250903"; transcript_id "Novel_003549"; exon_number "1"; gene_type "
scaffold1	Cufflinks	exon	1272587	1273419	.	-	.	gene_id "XLOC_250903"; transcript_id "Novel_003549"; exon_number "2"; gene_type "
scaffold1	Cufflinks	exon	190353	193039	.	-	.	gene_id "XLOC_251109"; transcript_id "Novel_003550"; exon_number "1"; gene_type "
scaffold1	Cufflinks	exon	193231	193385	.	-	.	gene_id "XLOC_251109"; transcript_id "Novel_003550"; exon_number "2"; gene_type "
scaffold1	Cufflinks	exon	193592	195480	.	-	.	gene_id "XLOC_251109"; transcript_id "Novel_003550"; exon_number "3"; gene_type "
scaffold1	Cufflinks	exon	190368	192675	.	-	.	gene_id "XLOC_251109"; transcript_id "Novel_003551"; exon_number "1"; gene_type "
scaffold1	Cufflinks	exon	192764	195384	.	-	.	gene_id "XLOC_251109"; transcript_id "Novel_003551"; exon_number "2"; gene_type "
scaffold1	Cufflinks	exon	190368	193039	.	-	.	gene_id "XLOC_251109"; transcript_id "Novel_003552"; exon_number "1"; gene_type "
scaffold1	Cufflinks	exon	193231	195384	.	-	.	gene_id "XLOC_251109"; transcript_id "Novel_003552"; exon_number "2"; gene_type "
scaffold1	Cufflinks	exon	190391	193385	.	-	.	gene_id "XLOC_251109"; transcript_id "Novel_003553"; exon_number "1"; gene_type "
scaffold1	Cufflinks	exon	193592	195401	.	-	.	gene_id "XLOC_251109"; transcript_id "Novel_003553"; exon_number "2"; gene_type "
scaffold1	Cufflinks	exon	352543	353255	.	-	.	gene_id "XLOC_251142"; transcript_id "Novel_003554"; exon_number "1"; gene_type "
scaffold1	Cufflinks	exon	353347	353460	.	-	.	gene_id "XLOC_251142"; transcript_id "Novel_003554"; exon_number "2"; gene_type "
scaffold1	Cufflinks	exon	237835	238061	.	+	.	gene_id "XLOC_251455"; transcript_id "Novel_003555"; exon_number "1"; gene_type "
scaffold1	Cufflinks	exon	238767	240193	.	+	.	gene_id "XLOC_251455"; transcript_id "Novel_003555"; exon_number "2"; gene_type "
scaffold1	Cufflinks	exon	345306	345922	.	-	.	gene_id "XLOC_251548"; transcript_id "Novel_003556"; exon_number "1"; gene_type "
scaffold1	Cufflinks	exon	347247	348124	.	-	.	gene_id "XLOC_251548"; transcript_id "Novel_003556"; exon_number "2"; gene_type "
scaffold1	Cufflinks	exon	122479	122895	.	-	.	gene_id "XLOC_251904"; transcript_id "Novel_003557"; exon_number "1"; gene_type "
scaffold1	Cufflinks	exon	122980	123075	.	-	.	gene_id "XLOC_251904"; transcript_id "Novel_003557"; exon_number "2"; gene_type "
scaffold1	Cufflinks	exon	123713	123888	.	-	.	gene_id "XLOC_251904"; transcript_id "Novel_003557"; exon_number "3"; gene_type "
scaffold1	Cufflinks	exon	124379	124493	.	-	.	gene_id "XLOC_251904"; transcript_id "Novel_003557"; exon_number "4"; gene_type "
scaffold1	Cufflinks	exon	125339	125664	.	-	.	gene_id "XLOC_251904"; transcript_id "Novel_003557"; exon_number "5"; gene_type "
scaffold1	Cufflinks	exon	76325	79224	.	-	.	gene_id "XLOC_251974"; transcript_id "Novel_003558"; exon_number "1"; gene_type "
scaffold1	Cufflinks	exon	79866	81800	.	-	.	gene_id "XLOC_251974"; transcript_id "Novel_003558"; exon_number "2"; gene_type "
scaffold1	Cufflinks	exon	76739	77646	.	-	.	gene_id "XLOC_251974"; transcript_id "Novel_003559"; exon_number "1"; gene_type "
scaffold1	Cufflinks	exon	77900	79224	.	-	.	gene_id "XLOC_251974"; transcript_id "Novel_003559"; exon_number "2"; gene_type "
scaffold1	Cufflinks	exon	79866	81621	.	-	.	gene_id "XLOC_251974"; transcript_id "Novel_003559"; exon_number "3"; gene_type "
scaffold1	Cufflinks	exon	76739	77646	.	-	.	gene_id "XLOC_251974"; transcript_id "Novel_003560"; exon_number "1"; gene_type "
scaffold1	Cufflinks	exon	77900	79224	.	-	.	gene_id "XLOC_251974"; transcript_id "Novel_003560"; exon_number "2"; gene_type "
scaffold1	Cufflinks	exon	79866	81155	.	-	.	gene_id "XLOC_251974"; transcript_id "Novel_003560"; exon_number "3"; gene_type "
scaffold1	Cufflinks	exon	81249	81621	.	-	.	gene_id "XLOC_251974"; transcript_id "Novel_003560"; exon_number "4"; gene_type "
scaffold1	Cufflinks	exon	43579	43792	.	+	.	gene_id "XLOC_251996"; transcript_id "Novel_003561"; exon_number "1"; gene_type "

scaffold1Cufflinks	exon	44067	44101	.	+	.	gene_id "XLOC_251996"; transcript_id "Novel_003561"; exon_number "2"; gene_type "
scaffold1Cufflinks	exon	70637	70848	.	+	.	gene_id "XLOC_252001"; transcript_id "Novel_003562"; exon_number "1"; gene_type "
scaffold1Cufflinks	exon	70935	70997	.	+	.	gene_id "XLOC_252001"; transcript_id "Novel_003562"; exon_number "2"; gene_type "
scaffold1Cufflinks	exon	71080	71195	.	+	.	gene_id "XLOC_252001"; transcript_id "Novel_003562"; exon_number "3"; gene_type "
scaffold1Cufflinks	exon	71272	71330	.	+	.	gene_id "XLOC_252001"; transcript_id "Novel_003562"; exon_number "4"; gene_type "
scaffold1Cufflinks	exon	71418	71920	.	+	.	gene_id "XLOC_252001"; transcript_id "Novel_003562"; exon_number "5"; gene_type "
scaffold1Cufflinks	exon	335644	336709	.	-	.	gene_id "XLOC_252057"; transcript_id "Novel_003563"; exon_number "1"; gene_type "
scaffold1Cufflinks	exon	336949	337010	.	-	.	gene_id "XLOC_252057"; transcript_id "Novel_003563"; exon_number "2"; gene_type "
scaffold1Cufflinks	exon	337363	337402	.	-	.	gene_id "XLOC_252057"; transcript_id "Novel_003563"; exon_number "3"; gene_type "
scaffold1Cufflinks	exon	337515	338417	.	-	.	gene_id "XLOC_252057"; transcript_id "Novel_003563"; exon_number "4"; gene_type "
scaffold1Cufflinks	exon	338715	338784	.	-	.	gene_id "XLOC_252057"; transcript_id "Novel_003563"; exon_number "5"; gene_type "
scaffold1Cufflinks	exon	339021	339173	.	-	.	gene_id "XLOC_252057"; transcript_id "Novel_003563"; exon_number "6"; gene_type "
scaffold1Cufflinks	exon	339668	339844	.	-	.	gene_id "XLOC_252057"; transcript_id "Novel_003563"; exon_number "7"; gene_type "
scaffold1Cufflinks	exon	340023	340268	.	-	.	gene_id "XLOC_252057"; transcript_id "Novel_003563"; exon_number "8"; gene_type "
scaffold1Cufflinks	exon	336656	336709	.	-	.	gene_id "XLOC_252057"; transcript_id "Novel_003564"; exon_number "1"; gene_type "
scaffold1Cufflinks	exon	336949	337010	.	-	.	gene_id "XLOC_252057"; transcript_id "Novel_003564"; exon_number "2"; gene_type "
scaffold1Cufflinks	exon	337363	337402	.	-	.	gene_id "XLOC_252057"; transcript_id "Novel_003564"; exon_number "3"; gene_type "
scaffold1Cufflinks	exon	337515	337628	.	-	.	gene_id "XLOC_252057"; transcript_id "Novel_003564"; exon_number "4"; gene_type "
scaffold1Cufflinks	exon	338259	338417	.	-	.	gene_id "XLOC_252057"; transcript_id "Novel_003564"; exon_number "5"; gene_type "
scaffold1Cufflinks	exon	338715	338784	.	-	.	gene_id "XLOC_252057"; transcript_id "Novel_003564"; exon_number "6"; gene_type "
scaffold1Cufflinks	exon	339021	339173	.	-	.	gene_id "XLOC_252057"; transcript_id "Novel_003564"; exon_number "7"; gene_type "
scaffold1Cufflinks	exon	339668	339844	.	-	.	gene_id "XLOC_252057"; transcript_id "Novel_003564"; exon_number "8"; gene_type "
scaffold1Cufflinks	exon	340023	340264	.	-	.	gene_id "XLOC_252057"; transcript_id "Novel_003564"; exon_number "9"; gene_type "
scaffold1Cufflinks	exon	338829	339173	.	-	.	gene_id "XLOC_252057"; transcript_id "Novel_003565"; exon_number "1"; gene_type "
scaffold1Cufflinks	exon	339668	340264	.	-	.	gene_id "XLOC_252057"; transcript_id "Novel_003565"; exon_number "2"; gene_type "
scaffold1Cufflinks	exon	231161	231531	.	-	.	gene_id "XLOC_252106"; transcript_id "Novel_003566"; exon_number "1"; gene_type "
scaffold1Cufflinks	exon	231613	231747	.	-	.	gene_id "XLOC_252106"; transcript_id "Novel_003566"; exon_number "2"; gene_type "
scaffold1Cufflinks	exon	233402	233544	.	-	.	gene_id "XLOC_252106"; transcript_id "Novel_003566"; exon_number "3"; gene_type "
scaffold1Cufflinks	exon	234284	234724	.	-	.	gene_id "XLOC_252106"; transcript_id "Novel_003566"; exon_number "4"; gene_type "
scaffold1Cufflinks	exon	233189	233544	.	-	.	gene_id "XLOC_252106"; transcript_id "Novel_003567"; exon_number "1"; gene_type "
scaffold1Cufflinks	exon	234284	234723	.	-	.	gene_id "XLOC_252106"; transcript_id "Novel_003567"; exon_number "2"; gene_type "
scaffold1Cufflinks	exon	91395	91860	.	+	.	gene_id "XLOC_252119"; transcript_id "Novel_003568"; exon_number "1"; gene_type "
scaffold1Cufflinks	exon	91965	92234	.	+	.	gene_id "XLOC_252119"; transcript_id "Novel_003568"; exon_number "2"; gene_type "
scaffold1Cufflinks	exon	92792	93798	.	+	.	gene_id "XLOC_252119"; transcript_id "Novel_003568"; exon_number "3"; gene_type "
scaffold1Cufflinks	exon	332165	332276	.	+	.	gene_id "XLOC_252248"; transcript_id "Novel_003569"; exon_number "1"; gene_type "

scaffold1	Cufflinks	exon	332487	332540	.	+	.	gene_id "XLOC_252248"; transcript_id "Novel_003569"; exon_number "2"; gene_type "
scaffold1	Cufflinks	exon	332952	333301	.	+	.	gene_id "XLOC_252248"; transcript_id "Novel_003569"; exon_number "3"; gene_type "
scaffold1	Cufflinks	exon	333434	335415	.	+	.	gene_id "XLOC_252248"; transcript_id "Novel_003569"; exon_number "4"; gene_type "
scaffold1	Cufflinks	exon	335838	336157	.	+	.	gene_id "XLOC_252248"; transcript_id "Novel_003569"; exon_number "5"; gene_type "
scaffold1	Cufflinks	exon	332165	332276	.	+	.	gene_id "XLOC_252248"; transcript_id "Novel_003570"; exon_number "1"; gene_type "
scaffold1	Cufflinks	exon	332487	332540	.	+	.	gene_id "XLOC_252248"; transcript_id "Novel_003570"; exon_number "2"; gene_type "
scaffold1	Cufflinks	exon	332952	333301	.	+	.	gene_id "XLOC_252248"; transcript_id "Novel_003570"; exon_number "3"; gene_type "
scaffold1	Cufflinks	exon	337488	339695	.	+	.	gene_id "XLOC_252248"; transcript_id "Novel_003570"; exon_number "4"; gene_type "
scaffold1	Cufflinks	exon	332165	332276	.	+	.	gene_id "XLOC_252248"; transcript_id "Novel_003571"; exon_number "1"; gene_type "
scaffold1	Cufflinks	exon	332487	332540	.	+	.	gene_id "XLOC_252248"; transcript_id "Novel_003571"; exon_number "2"; gene_type "
scaffold1	Cufflinks	exon	332952	333301	.	+	.	gene_id "XLOC_252248"; transcript_id "Novel_003571"; exon_number "3"; gene_type "
scaffold1	Cufflinks	exon	337488	339469	.	+	.	gene_id "XLOC_252248"; transcript_id "Novel_003571"; exon_number "4"; gene_type "
scaffold1	Cufflinks	exon	339890	340432	.	+	.	gene_id "XLOC_252248"; transcript_id "Novel_003571"; exon_number "5"; gene_type "
scaffold1	Cufflinks	exon	332284	332540	.	+	.	gene_id "XLOC_252248"; transcript_id "Novel_003572"; exon_number "1"; gene_type "
scaffold1	Cufflinks	exon	332952	333301	.	+	.	gene_id "XLOC_252248"; transcript_id "Novel_003572"; exon_number "2"; gene_type "
scaffold1	Cufflinks	exon	337488	339469	.	+	.	gene_id "XLOC_252248"; transcript_id "Novel_003572"; exon_number "3"; gene_type "
scaffold1	Cufflinks	exon	339890	340046	.	+	.	gene_id "XLOC_252248"; transcript_id "Novel_003572"; exon_number "4"; gene_type "
scaffold1	Cufflinks	exon	332347	332540	.	+	.	gene_id "XLOC_252248"; transcript_id "Novel_003573"; exon_number "1"; gene_type "
scaffold1	Cufflinks	exon	332952	333301	.	+	.	gene_id "XLOC_252248"; transcript_id "Novel_003573"; exon_number "2"; gene_type "
scaffold1	Cufflinks	exon	333434	335415	.	+	.	gene_id "XLOC_252248"; transcript_id "Novel_003573"; exon_number "3"; gene_type "
scaffold1	Cufflinks	exon	339890	340028	.	+	.	gene_id "XLOC_252248"; transcript_id "Novel_003573"; exon_number "4"; gene_type "
scaffold1	Cufflinks	exon	673343	674273	.	-	.	gene_id "XLOC_252521"; transcript_id "Novel_003574"; exon_number "1"; gene_type "
scaffold1	Cufflinks	exon	674741	674856	.	-	.	gene_id "XLOC_252521"; transcript_id "Novel_003574"; exon_number "2"; gene_type "
scaffold1	Cufflinks	exon	675470	675622	.	-	.	gene_id "XLOC_252521"; transcript_id "Novel_003574"; exon_number "3"; gene_type "
scaffold1	Cufflinks	exon	675764	675803	.	-	.	gene_id "XLOC_252521"; transcript_id "Novel_003574"; exon_number "4"; gene_type "
scaffold1	Cufflinks	exon	1133746	1134176	.	-	.	gene_id "XLOC_252548"; transcript_id "Novel_003575"; exon_number "1"; gene_type "
scaffold1	Cufflinks	exon	1134690	1135276	.	-	.	gene_id "XLOC_252548"; transcript_id "Novel_003575"; exon_number "2"; gene_type "
scaffold1	Cufflinks	exon	1135387	1135716	.	-	.	gene_id "XLOC_252548"; transcript_id "Novel_003575"; exon_number "3"; gene_type "
scaffold1	Cufflinks	exon	1133840	1134176	.	-	.	gene_id "XLOC_252548"; transcript_id "Novel_003576"; exon_number "1"; gene_type "
scaffold1	Cufflinks	exon	1134690	1134872	.	-	.	gene_id "XLOC_252548"; transcript_id "Novel_003576"; exon_number "2"; gene_type "
scaffold1	Cufflinks	exon	1134967	1135095	.	-	.	gene_id "XLOC_252548"; transcript_id "Novel_003576"; exon_number "3"; gene_type "
scaffold1	Cufflinks	exon	1135187	1135276	.	-	.	gene_id "XLOC_252548"; transcript_id "Novel_003576"; exon_number "4"; gene_type "
scaffold1	Cufflinks	exon	1135387	1136112	.	-	.	gene_id "XLOC_252548"; transcript_id "Novel_003576"; exon_number "5"; gene_type "
scaffold1	Cufflinks	exon	167223	168812	.	+	.	gene_id "XLOC_252759"; transcript_id "Novel_003577"; exon_number "1"; gene_type "
scaffold1	Cufflinks	exon	168939	169031	.	+	.	gene_id "XLOC_252759"; transcript_id "Novel_003577"; exon_number "2"; gene_type "

scaffold1	Cufflinks	exon	169118	169657	.	+	.	gene_id "XLOC_252759"; transcript_id "Novel_003577"; exon_number "3"; gene_type "
scaffold1	Cufflinks	exon	104726	105512	.	-	.	gene_id "XLOC_252807"; transcript_id "Novel_003578"; exon_number "1"; gene_type "
scaffold1	Cufflinks	exon	105944	105973	.	-	.	gene_id "XLOC_252807"; transcript_id "Novel_003578"; exon_number "2"; gene_type "
scaffold1	Cufflinks	exon	106705	106740	.	-	.	gene_id "XLOC_252807"; transcript_id "Novel_003578"; exon_number "3"; gene_type "
scaffold1	Cufflinks	exon	106962	107141	.	-	.	gene_id "XLOC_252807"; transcript_id "Novel_003578"; exon_number "4"; gene_type "
scaffold1	Cufflinks	exon	107860	108192	.	-	.	gene_id "XLOC_252807"; transcript_id "Novel_003578"; exon_number "5"; gene_type "
scaffold1	Cufflinks	exon	108367	108585	.	-	.	gene_id "XLOC_252807"; transcript_id "Novel_003578"; exon_number "6"; gene_type "
scaffold1	Cufflinks	exon	205557	206493	.	-	.	gene_id "XLOC_252841"; transcript_id "Novel_003579"; exon_number "1"; gene_type "
scaffold1	Cufflinks	exon	207513	207542	.	-	.	gene_id "XLOC_252841"; transcript_id "Novel_003579"; exon_number "2"; gene_type "
scaffold1	Cufflinks	exon	208068	208115	.	-	.	gene_id "XLOC_252841"; transcript_id "Novel_003579"; exon_number "3"; gene_type "
scaffold1	Cufflinks	exon	208187	208213	.	-	.	gene_id "XLOC_252841"; transcript_id "Novel_003579"; exon_number "4"; gene_type "
scaffold1	Cufflinks	exon	208291	208515	.	-	.	gene_id "XLOC_252841"; transcript_id "Novel_003579"; exon_number "5"; gene_type "
scaffold1	Cufflinks	exon	208631	208932	.	-	.	gene_id "XLOC_252841"; transcript_id "Novel_003579"; exon_number "6"; gene_type "
scaffold1	Cufflinks	exon	209138	209263	.	-	.	gene_id "XLOC_252841"; transcript_id "Novel_003579"; exon_number "7"; gene_type "
scaffold1	Cufflinks	exon	205724	206493	.	-	.	gene_id "XLOC_252841"; transcript_id "Novel_003580"; exon_number "1"; gene_type "
scaffold1	Cufflinks	exon	207513	207542	.	-	.	gene_id "XLOC_252841"; transcript_id "Novel_003580"; exon_number "2"; gene_type "
scaffold1	Cufflinks	exon	208068	208115	.	-	.	gene_id "XLOC_252841"; transcript_id "Novel_003580"; exon_number "3"; gene_type "
scaffold1	Cufflinks	exon	208187	208213	.	-	.	gene_id "XLOC_252841"; transcript_id "Novel_003580"; exon_number "4"; gene_type "
scaffold1	Cufflinks	exon	208291	208932	.	-	.	gene_id "XLOC_252841"; transcript_id "Novel_003580"; exon_number "5"; gene_type "
scaffold1	Cufflinks	exon	209138	209275	.	-	.	gene_id "XLOC_252841"; transcript_id "Novel_003580"; exon_number "6"; gene_type "
scaffold1	Cufflinks	exon	222606	223069	.	-	.	gene_id "XLOC_252841"; transcript_id "Novel_003581"; exon_number "1"; gene_type "
scaffold1	Cufflinks	exon	223677	223706	.	-	.	gene_id "XLOC_252841"; transcript_id "Novel_003581"; exon_number "2"; gene_type "
scaffold1	Cufflinks	exon	224313	224357	.	-	.	gene_id "XLOC_252841"; transcript_id "Novel_003581"; exon_number "3"; gene_type "
scaffold1	Cufflinks	exon	224625	224651	.	-	.	gene_id "XLOC_252841"; transcript_id "Novel_003581"; exon_number "4"; gene_type "
scaffold1	Cufflinks	exon	224729	224953	.	-	.	gene_id "XLOC_252841"; transcript_id "Novel_003581"; exon_number "5"; gene_type "
scaffold1	Cufflinks	exon	225054	225349	.	-	.	gene_id "XLOC_252841"; transcript_id "Novel_003581"; exon_number "6"; gene_type "
scaffold1	Cufflinks	exon	232058	232075	.	-	.	gene_id "XLOC_252841"; transcript_id "Novel_003581"; exon_number "7"; gene_type "
scaffold1	Cufflinks	exon	222612	223069	.	-	.	gene_id "XLOC_252841"; transcript_id "Novel_003582"; exon_number "1"; gene_type "
scaffold1	Cufflinks	exon	223677	223706	.	-	.	gene_id "XLOC_252841"; transcript_id "Novel_003582"; exon_number "2"; gene_type "
scaffold1	Cufflinks	exon	224313	224357	.	-	.	gene_id "XLOC_252841"; transcript_id "Novel_003582"; exon_number "3"; gene_type "
scaffold1	Cufflinks	exon	224429	224455	.	-	.	gene_id "XLOC_252841"; transcript_id "Novel_003582"; exon_number "4"; gene_type "
scaffold1	Cufflinks	exon	224729	224953	.	-	.	gene_id "XLOC_252841"; transcript_id "Novel_003582"; exon_number "5"; gene_type "
scaffold1	Cufflinks	exon	225054	225169	.	-	.	gene_id "XLOC_252841"; transcript_id "Novel_003582"; exon_number "6"; gene_type "
scaffold1	Cufflinks	exon	231705	231723	.	-	.	gene_id "XLOC_252841"; transcript_id "Novel_003582"; exon_number "7"; gene_type "
scaffold1	Cufflinks	exon	222621	223069	.	-	.	gene_id "XLOC_252841"; transcript_id "Novel_003583"; exon_number "1"; gene_type "

scaffold1Cufflinks	exon	223677	223706	.	-	.	gene_id "XLOC_252841"; transcript_id "Novel_003583"; exon_number "2"; gene_type "
scaffold1Cufflinks	exon	224313	224357	.	-	.	gene_id "XLOC_252841"; transcript_id "Novel_003583"; exon_number "3"; gene_type "
scaffold1Cufflinks	exon	224429	224455	.	-	.	gene_id "XLOC_252841"; transcript_id "Novel_003583"; exon_number "4"; gene_type "
scaffold1Cufflinks	exon	224729	224953	.	-	.	gene_id "XLOC_252841"; transcript_id "Novel_003583"; exon_number "5"; gene_type "
scaffold1Cufflinks	exon	225054	225349	.	-	.	gene_id "XLOC_252841"; transcript_id "Novel_003583"; exon_number "6"; gene_type "
scaffold1Cufflinks	exon	225560	225825	.	-	.	gene_id "XLOC_252841"; transcript_id "Novel_003583"; exon_number "7"; gene_type "
scaffold1Cufflinks	exon	254391	254781	.	-	.	gene_id "XLOC_252858"; transcript_id "Novel_003584"; exon_number "1"; gene_type "
scaffold1Cufflinks	exon	257072	257277	.	-	.	gene_id "XLOC_252858"; transcript_id "Novel_003584"; exon_number "2"; gene_type "
scaffold1Cufflinks	exon	257378	257554	.	-	.	gene_id "XLOC_252858"; transcript_id "Novel_003584"; exon_number "3"; gene_type "
scaffold1Cufflinks	exon	295839	296219	.	-	.	gene_id "XLOC_252869"; transcript_id "Novel_003585"; exon_number "1"; gene_type "
scaffold1Cufflinks	exon	296299	296365	.	-	.	gene_id "XLOC_252869"; transcript_id "Novel_003585"; exon_number "2"; gene_type "
scaffold1Cufflinks	exon	298404	298506	.	-	.	gene_id "XLOC_252869"; transcript_id "Novel_003585"; exon_number "3"; gene_type "
scaffold1Cufflinks	exon	298605	298691	.	-	.	gene_id "XLOC_252869"; transcript_id "Novel_003585"; exon_number "4"; gene_type "
scaffold1Cufflinks	exon	298795	298949	.	-	.	gene_id "XLOC_252869"; transcript_id "Novel_003585"; exon_number "5"; gene_type "
scaffold1Cufflinks	exon	185753	186251	.	+	.	gene_id "XLOC_253169"; transcript_id "Novel_003586"; exon_number "1"; gene_type "
scaffold1Cufflinks	exon	186324	186403	.	+	.	gene_id "XLOC_253169"; transcript_id "Novel_003586"; exon_number "2"; gene_type "
scaffold1Cufflinks	exon	186497	186632	.	+	.	gene_id "XLOC_253169"; transcript_id "Novel_003586"; exon_number "3"; gene_type "
scaffold1Cufflinks	exon	186748	186897	.	+	.	gene_id "XLOC_253169"; transcript_id "Novel_003586"; exon_number "4"; gene_type "
scaffold1Cufflinks	exon	187014	187481	.	+	.	gene_id "XLOC_253169"; transcript_id "Novel_003586"; exon_number "5"; gene_type "
scaffold1Cufflinks	exon	844911	844967	.	+	.	gene_id "XLOC_253403"; transcript_id "Novel_003587"; exon_number "1"; gene_type "
scaffold1Cufflinks	exon	846499	847076	.	+	.	gene_id "XLOC_253403"; transcript_id "Novel_003587"; exon_number "2"; gene_type "
scaffold1Cufflinks	exon	847665	848238	.	+	.	gene_id "XLOC_253403"; transcript_id "Novel_003587"; exon_number "3"; gene_type "
scaffold1Cufflinks	exon	890183	890251	.	+	.	gene_id "XLOC_253412"; transcript_id "Novel_003588"; exon_number "1"; gene_type "
scaffold1Cufflinks	exon	891114	891331	.	+	.	gene_id "XLOC_253412"; transcript_id "Novel_003588"; exon_number "2"; gene_type "
scaffold1Cufflinks	exon	1091908	1093344	.	+	.	gene_id "XLOC_253441"; transcript_id "Novel_003589"; exon_number "1"; gene_type "
scaffold1Cufflinks	exon	1093803	1094404	.	+	.	gene_id "XLOC_253441"; transcript_id "Novel_003589"; exon_number "2"; gene_type "
scaffold1Cufflinks	exon	1091956	1093344	.	+	.	gene_id "XLOC_253441"; transcript_id "Novel_003590"; exon_number "1"; gene_type "
scaffold1Cufflinks	exon	1093803	1093833	.	+	.	gene_id "XLOC_253441"; transcript_id "Novel_003590"; exon_number "2"; gene_type "
scaffold1Cufflinks	exon	1093926	1094314	.	+	.	gene_id "XLOC_253441"; transcript_id "Novel_003590"; exon_number "3"; gene_type "
scaffold1Cufflinks	exon	1328965	1329167	.	+	.	gene_id "XLOC_253470"; transcript_id "Novel_003591"; exon_number "1"; gene_type "
scaffold1Cufflinks	exon	1575392	1575928	.	+	.	gene_id "XLOC_253522"; transcript_id "Novel_003592"; exon_number "1"; gene_type "
scaffold1Cufflinks	exon	1576002	1576249	.	+	.	gene_id "XLOC_253522"; transcript_id "Novel_003592"; exon_number "2"; gene_type "
scaffold1Cufflinks	exon	1576327	1577031	.	+	.	gene_id "XLOC_253522"; transcript_id "Novel_003592"; exon_number "3"; gene_type "
scaffold1Cufflinks	exon	1575392	1575928	.	+	.	gene_id "XLOC_253522"; transcript_id "Novel_003593"; exon_number "1"; gene_type "
scaffold1Cufflinks	exon	1576002	1576249	.	+	.	gene_id "XLOC_253522"; transcript_id "Novel_003593"; exon_number "2"; gene_type "

scaffold1	Cufflinks	exon	1576327	1576645	.	+	.	gene_id "XLOC_253522"; transcript_id "Novel_003593"; exon_number "3"; gene_type "
scaffold1	Cufflinks	exon	1576717	1577031	.	+	.	gene_id "XLOC_253522"; transcript_id "Novel_003593"; exon_number "4"; gene_type "
scaffold1	Cufflinks	exon	854257	854733	.	-	.	gene_id "XLOC_253648"; transcript_id "Novel_003594"; exon_number "1"; gene_type "
scaffold1	Cufflinks	exon	855460	856007	.	-	.	gene_id "XLOC_253648"; transcript_id "Novel_003594"; exon_number "2"; gene_type "
scaffold1	Cufflinks	exon	856811	857173	.	-	.	gene_id "XLOC_253648"; transcript_id "Novel_003594"; exon_number "3"; gene_type "
scaffold1	Cufflinks	exon	854259	854733	.	-	.	gene_id "XLOC_253648"; transcript_id "Novel_003595"; exon_number "1"; gene_type "
scaffold1	Cufflinks	exon	855460	856735	.	-	.	gene_id "XLOC_253648"; transcript_id "Novel_003595"; exon_number "2"; gene_type "
scaffold1	Cufflinks	exon	1342872	1344560	.	-	.	gene_id "XLOC_253731"; transcript_id "Novel_003596"; exon_number "1"; gene_type "
scaffold1	Cufflinks	exon	1344693	1344836	.	-	.	gene_id "XLOC_253731"; transcript_id "Novel_003596"; exon_number "2"; gene_type "
scaffold1	Cufflinks	exon	1344928	1346048	.	-	.	gene_id "XLOC_253731"; transcript_id "Novel_003596"; exon_number "3"; gene_type "
scaffold1	Cufflinks	exon	44444	44733	.	-	.	gene_id "XLOC_253920"; transcript_id "Novel_003597"; exon_number "1"; gene_type "
scaffold1	Cufflinks	exon	44978	45050	.	-	.	gene_id "XLOC_253920"; transcript_id "Novel_003597"; exon_number "2"; gene_type "
scaffold1	Cufflinks	exon	45422	45846	.	-	.	gene_id "XLOC_253920"; transcript_id "Novel_003597"; exon_number "3"; gene_type "
scaffold1	Cufflinks	exon	256950	258746	.	-	.	gene_id "XLOC_253977"; transcript_id "Novel_003598"; exon_number "1"; gene_type "
scaffold1	Cufflinks	exon	259063	259835	.	-	.	gene_id "XLOC_253977"; transcript_id "Novel_003598"; exon_number "2"; gene_type "
scaffold1	Cufflinks	exon	326366	329211	.	+	.	gene_id "XLOC_254057"; transcript_id "Novel_003599"; exon_number "1"; gene_type "
scaffold1	Cufflinks	exon	330127	331717	.	+	.	gene_id "XLOC_254057"; transcript_id "Novel_003599"; exon_number "2"; gene_type "
scaffold1	Cufflinks	exon	236122	237350	.	-	.	gene_id "XLOC_254195"; transcript_id "Novel_003600"; exon_number "1"; gene_type "
scaffold1	Cufflinks	exon	238113	238147	.	-	.	gene_id "XLOC_254195"; transcript_id "Novel_003600"; exon_number "2"; gene_type "
scaffold1	Cufflinks	exon	1197723	1198791	.	+	.	gene_id "XLOC_254305"; transcript_id "Novel_003601"; exon_number "1"; gene_type "
scaffold1	Cufflinks	exon	1198869	1198995	.	+	.	gene_id "XLOC_254305"; transcript_id "Novel_003601"; exon_number "2"; gene_type "
scaffold1	Cufflinks	exon	1074677	1076666	.	-	.	gene_id "XLOC_254568"; transcript_id "Novel_003602"; exon_number "1"; gene_type "
scaffold1	Cufflinks	exon	1077856	1078370	.	-	.	gene_id "XLOC_254568"; transcript_id "Novel_003602"; exon_number "2"; gene_type "
scaffold1	Cufflinks	exon	1079235	1079630	.	-	.	gene_id "XLOC_254568"; transcript_id "Novel_003602"; exon_number "3"; gene_type "
scaffold1	Cufflinks	exon	1079716	1079757	.	-	.	gene_id "XLOC_254568"; transcript_id "Novel_003602"; exon_number "4"; gene_type "
scaffold1	Cufflinks	exon	1079891	1080193	.	-	.	gene_id "XLOC_254568"; transcript_id "Novel_003602"; exon_number "5"; gene_type "
scaffold1	Cufflinks	exon	1080304	1080493	.	-	.	gene_id "XLOC_254568"; transcript_id "Novel_003602"; exon_number "6"; gene_type "
scaffold1	Cufflinks	exon	1080874	1080964	.	-	.	gene_id "XLOC_254568"; transcript_id "Novel_003602"; exon_number "7"; gene_type "
scaffold1	Cufflinks	exon	1082569	1082615	.	-	.	gene_id "XLOC_254568"; transcript_id "Novel_003602"; exon_number "8"; gene_type "
scaffold1	Cufflinks	exon	1083316	1083752	.	-	.	gene_id "XLOC_254568"; transcript_id "Novel_003602"; exon_number "9"; gene_type "
scaffold1	Cufflinks	exon	1074677	1076666	.	-	.	gene_id "XLOC_254568"; transcript_id "Novel_003603"; exon_number "1"; gene_type "
scaffold1	Cufflinks	exon	1077856	1078370	.	-	.	gene_id "XLOC_254568"; transcript_id "Novel_003603"; exon_number "2"; gene_type "
scaffold1	Cufflinks	exon	1079235	1079630	.	-	.	gene_id "XLOC_254568"; transcript_id "Novel_003603"; exon_number "3"; gene_type "
scaffold1	Cufflinks	exon	1079716	1080193	.	-	.	gene_id "XLOC_254568"; transcript_id "Novel_003603"; exon_number "4"; gene_type "
scaffold1	Cufflinks	exon	1080304	1080449	.	-	.	gene_id "XLOC_254568"; transcript_id "Novel_003603"; exon_number "5"; gene_type "

scaffold1Cufflinks	exon	1080874	1080964	.	-	.	gene_id "XLOC_254568"; transcript_id "Novel_003603"; exon_number "6"; gene_type "
scaffold1Cufflinks	exon	1082569	1082615	.	-	.	gene_id "XLOC_254568"; transcript_id "Novel_003603"; exon_number "7"; gene_type "
scaffold1Cufflinks	exon	1083316	1083752	.	-	.	gene_id "XLOC_254568"; transcript_id "Novel_003603"; exon_number "8"; gene_type "
scaffold1Cufflinks	exon	1185302	1186936	.	-	.	gene_id "XLOC_254586"; transcript_id "Novel_003604"; exon_number "1"; gene_type "
scaffold1Cufflinks	exon	1187288	1187390	.	-	.	gene_id "XLOC_254586"; transcript_id "Novel_003604"; exon_number "2"; gene_type "
scaffold1Cufflinks	exon	1310467	1310978	.	-	.	gene_id "XLOC_254613"; transcript_id "Novel_003605"; exon_number "1"; gene_type "
scaffold1Cufflinks	exon	1311688	1311763	.	-	.	gene_id "XLOC_254613"; transcript_id "Novel_003605"; exon_number "2"; gene_type "
scaffold1Cufflinks	exon	1312131	1312548	.	-	.	gene_id "XLOC_254613"; transcript_id "Novel_003605"; exon_number "3"; gene_type "
scaffold1Cufflinks	exon	1336419	1336620	.	-	.	gene_id "XLOC_254615"; transcript_id "Novel_003606"; exon_number "1"; gene_type "
scaffold1Cufflinks	exon	1336718	1336978	.	-	.	gene_id "XLOC_254615"; transcript_id "Novel_003606"; exon_number "2"; gene_type "
scaffold1Cufflinks	exon	158924	159312	.	-	.	gene_id "XLOC_254806"; transcript_id "Novel_003607"; exon_number "1"; gene_type "
scaffold1Cufflinks	exon	159417	159605	.	-	.	gene_id "XLOC_254806"; transcript_id "Novel_003607"; exon_number "2"; gene_type "
scaffold1Cufflinks	exon	159744	159861	.	-	.	gene_id "XLOC_254806"; transcript_id "Novel_003607"; exon_number "3"; gene_type "
scaffold1Cufflinks	exon	159954	160604	.	-	.	gene_id "XLOC_254806"; transcript_id "Novel_003607"; exon_number "4"; gene_type "
scaffold1Cufflinks	exon	160795	160958	.	-	.	gene_id "XLOC_254806"; transcript_id "Novel_003607"; exon_number "5"; gene_type "
scaffold1Cufflinks	exon	161058	161910	.	-	.	gene_id "XLOC_254806"; transcript_id "Novel_003607"; exon_number "6"; gene_type "
scaffold1Cufflinks	exon	179262	180186	.	-	.	gene_id "XLOC_254977"; transcript_id "Novel_003608"; exon_number "1"; gene_type "
scaffold1Cufflinks	exon	181262	181712	.	-	.	gene_id "XLOC_254977"; transcript_id "Novel_003608"; exon_number "2"; gene_type "
scaffold1Cufflinks	exon	70840	71084	.	+	.	gene_id "XLOC_255113"; transcript_id "Novel_003609"; exon_number "1"; gene_type "
scaffold1Cufflinks	exon	72192	72451	.	+	.	gene_id "XLOC_255113"; transcript_id "Novel_003609"; exon_number "2"; gene_type "
scaffold1Cufflinks	exon	72855	73183	.	+	.	gene_id "XLOC_255113"; transcript_id "Novel_003609"; exon_number "3"; gene_type "
scaffold1Cufflinks	exon	70870	71084	.	+	.	gene_id "XLOC_255113"; transcript_id "Novel_003610"; exon_number "1"; gene_type "
scaffold1Cufflinks	exon	72192	72374	.	+	.	gene_id "XLOC_255113"; transcript_id "Novel_003610"; exon_number "2"; gene_type "
scaffold1Cufflinks	exon	77555	77631	.	+	.	gene_id "XLOC_255113"; transcript_id "Novel_003610"; exon_number "3"; gene_type "
scaffold1Cufflinks	exon	78084	78719	.	+	.	gene_id "XLOC_255113"; transcript_id "Novel_003610"; exon_number "4"; gene_type "
scaffold1Cufflinks	exon	70870	71084	.	+	.	gene_id "XLOC_255113"; transcript_id "Novel_003611"; exon_number "1"; gene_type "
scaffold1Cufflinks	exon	72192	72338	.	+	.	gene_id "XLOC_255113"; transcript_id "Novel_003611"; exon_number "2"; gene_type "
scaffold1Cufflinks	exon	77519	77631	.	+	.	gene_id "XLOC_255113"; transcript_id "Novel_003611"; exon_number "3"; gene_type "
scaffold1Cufflinks	exon	78084	78719	.	+	.	gene_id "XLOC_255113"; transcript_id "Novel_003611"; exon_number "4"; gene_type "
scaffold1Cufflinks	exon	76024	76159	.	+	.	gene_id "XLOC_255113"; transcript_id "Novel_003612"; exon_number "1"; gene_type "
scaffold1Cufflinks	exon	77372	77631	.	+	.	gene_id "XLOC_255113"; transcript_id "Novel_003612"; exon_number "2"; gene_type "
scaffold1Cufflinks	exon	78084	78719	.	+	.	gene_id "XLOC_255113"; transcript_id "Novel_003612"; exon_number "3"; gene_type "
scaffold1Cufflinks	exon	222418	222487	.	-	.	gene_id "XLOC_255173"; transcript_id "Novel_003613"; exon_number "1"; gene_type "
scaffold1Cufflinks	exon	222611	222729	.	-	.	gene_id "XLOC_255173"; transcript_id "Novel_003613"; exon_number "2"; gene_type "
scaffold1Cufflinks	exon	222819	222912	.	-	.	gene_id "XLOC_255173"; transcript_id "Novel_003613"; exon_number "3"; gene_type "

scaffold1	Cufflinks	exon	43778	44755	.	-	.	gene_id "XLOC_255510"; transcript_id "Novel_003614"; exon_number "1"; gene_type "
scaffold1	Cufflinks	exon	44843	46512	.	-	.	gene_id "XLOC_255510"; transcript_id "Novel_003614"; exon_number "2"; gene_type "
scaffold1	Cufflinks	exon	43778	44744	.	-	.	gene_id "XLOC_255510"; transcript_id "Novel_003615"; exon_number "1"; gene_type "
scaffold1	Cufflinks	exon	44843	46512	.	-	.	gene_id "XLOC_255510"; transcript_id "Novel_003615"; exon_number "2"; gene_type "
scaffold1	Cufflinks	exon	43778	45064	.	-	.	gene_id "XLOC_255510"; transcript_id "Novel_003616"; exon_number "1"; gene_type "
scaffold1	Cufflinks	exon	46874	46962	.	-	.	gene_id "XLOC_255510"; transcript_id "Novel_003616"; exon_number "2"; gene_type "
scaffold1	Cufflinks	exon	47053	47123	.	-	.	gene_id "XLOC_255510"; transcript_id "Novel_003616"; exon_number "3"; gene_type "
scaffold1	Cufflinks	exon	47545	47624	.	-	.	gene_id "XLOC_255510"; transcript_id "Novel_003616"; exon_number "4"; gene_type "
scaffold1	Cufflinks	exon	47715	47945	.	-	.	gene_id "XLOC_255510"; transcript_id "Novel_003616"; exon_number "5"; gene_type "
scaffold1	Cufflinks	exon	43856	44744	.	-	.	gene_id "XLOC_255510"; transcript_id "Novel_003617"; exon_number "1"; gene_type "
scaffold1	Cufflinks	exon	44843	45067	.	-	.	gene_id "XLOC_255510"; transcript_id "Novel_003617"; exon_number "2"; gene_type "
scaffold1	Cufflinks	exon	46874	46962	.	-	.	gene_id "XLOC_255510"; transcript_id "Novel_003617"; exon_number "3"; gene_type "
scaffold1	Cufflinks	exon	47053	47123	.	-	.	gene_id "XLOC_255510"; transcript_id "Novel_003617"; exon_number "4"; gene_type "
scaffold1	Cufflinks	exon	47545	47624	.	-	.	gene_id "XLOC_255510"; transcript_id "Novel_003617"; exon_number "5"; gene_type "
scaffold1	Cufflinks	exon	47715	47923	.	-	.	gene_id "XLOC_255510"; transcript_id "Novel_003617"; exon_number "6"; gene_type "
scaffold1	Cufflinks	exon	46598	46962	.	-	.	gene_id "XLOC_255510"; transcript_id "Novel_003618"; exon_number "1"; gene_type "
scaffold1	Cufflinks	exon	47053	47123	.	-	.	gene_id "XLOC_255510"; transcript_id "Novel_003618"; exon_number "2"; gene_type "
scaffold1	Cufflinks	exon	47545	47624	.	-	.	gene_id "XLOC_255510"; transcript_id "Novel_003618"; exon_number "3"; gene_type "
scaffold1	Cufflinks	exon	47715	47913	.	-	.	gene_id "XLOC_255510"; transcript_id "Novel_003618"; exon_number "4"; gene_type "
scaffold1	Cufflinks	exon	46603	46962	.	-	.	gene_id "XLOC_255510"; transcript_id "Novel_003619"; exon_number "1"; gene_type "
scaffold1	Cufflinks	exon	47053	47624	.	-	.	gene_id "XLOC_255510"; transcript_id "Novel_003619"; exon_number "2"; gene_type "
scaffold1	Cufflinks	exon	47715	47923	.	-	.	gene_id "XLOC_255510"; transcript_id "Novel_003619"; exon_number "3"; gene_type "
scaffold1	Cufflinks	exon	131259	132458	.	-	.	gene_id "XLOC_255892"; transcript_id "Novel_003620"; exon_number "1"; gene_type "
scaffold1	Cufflinks	exon	132615	132707	.	-	.	gene_id "XLOC_255892"; transcript_id "Novel_003620"; exon_number "2"; gene_type "
scaffold1	Cufflinks	exon	132890	132998	.	-	.	gene_id "XLOC_255892"; transcript_id "Novel_003620"; exon_number "3"; gene_type "
scaffold1	Cufflinks	exon	82379	83570	.	+	.	gene_id "XLOC_256203"; transcript_id "Novel_003621"; exon_number "1"; gene_type "
scaffold1	Cufflinks	exon	14092	14510	.	-	.	gene_id "XLOC_256246"; transcript_id "Novel_003622"; exon_number "1"; gene_type "
scaffold1	Cufflinks	exon	497593	497853	.	-	.	gene_id "XLOC_256703"; transcript_id "Novel_003623"; exon_number "1"; gene_type "
scaffold1	Cufflinks	exon	1666	2605	.	-	.	gene_id "XLOC_257048"; transcript_id "Novel_003624"; exon_number "1"; gene_type "
scaffold1	Cufflinks	exon	2780	3105	.	-	.	gene_id "XLOC_257048"; transcript_id "Novel_003624"; exon_number "2"; gene_type "
scaffold1	Cufflinks	exon	3229	3328	.	-	.	gene_id "XLOC_257048"; transcript_id "Novel_003624"; exon_number "3"; gene_type "
scaffold1	Cufflinks	exon	3423	3990	.	-	.	gene_id "XLOC_257048"; transcript_id "Novel_003624"; exon_number "4"; gene_type "
scaffold1	Cufflinks	exon	1686	2605	.	-	.	gene_id "XLOC_257048"; transcript_id "Novel_003625"; exon_number "1"; gene_type "
scaffold1	Cufflinks	exon	2780	3105	.	-	.	gene_id "XLOC_257048"; transcript_id "Novel_003625"; exon_number "2"; gene_type "
scaffold1	Cufflinks	exon	3229	3328	.	-	.	gene_id "XLOC_257048"; transcript_id "Novel_003625"; exon_number "3"; gene_type "

scaffold1	Cufflinks	exon	3423	3453	.	-	.	gene_id "XLOC_257048"; transcript_id "Novel_003625"; exon_number "4"; gene_type "
scaffold1	Cufflinks	exon	3564	3661	.	-	.	gene_id "XLOC_257048"; transcript_id "Novel_003625"; exon_number "5"; gene_type "
scaffold1	Cufflinks	exon	3755	3995	.	-	.	gene_id "XLOC_257048"; transcript_id "Novel_003625"; exon_number "6"; gene_type "
scaffold1	Cufflinks	exon	1704	2605	.	-	.	gene_id "XLOC_257048"; transcript_id "Novel_003626"; exon_number "1"; gene_type "
scaffold1	Cufflinks	exon	2780	3105	.	-	.	gene_id "XLOC_257048"; transcript_id "Novel_003626"; exon_number "2"; gene_type "
scaffold1	Cufflinks	exon	3229	3328	.	-	.	gene_id "XLOC_257048"; transcript_id "Novel_003626"; exon_number "3"; gene_type "
scaffold1	Cufflinks	exon	3423	3453	.	-	.	gene_id "XLOC_257048"; transcript_id "Novel_003626"; exon_number "4"; gene_type "
scaffold1	Cufflinks	exon	3564	4097	.	-	.	gene_id "XLOC_257048"; transcript_id "Novel_003626"; exon_number "5"; gene_type "
scaffold1	Cufflinks	exon	1796	2605	.	-	.	gene_id "XLOC_257048"; transcript_id "Novel_003627"; exon_number "1"; gene_type "
scaffold1	Cufflinks	exon	2780	2923	.	-	.	gene_id "XLOC_257048"; transcript_id "Novel_003627"; exon_number "2"; gene_type "
scaffold1	Cufflinks	exon	3035	3105	.	-	.	gene_id "XLOC_257048"; transcript_id "Novel_003627"; exon_number "3"; gene_type "
scaffold1	Cufflinks	exon	3229	3328	.	-	.	gene_id "XLOC_257048"; transcript_id "Novel_003627"; exon_number "4"; gene_type "
scaffold1	Cufflinks	exon	3423	3453	.	-	.	gene_id "XLOC_257048"; transcript_id "Novel_003627"; exon_number "5"; gene_type "
scaffold1	Cufflinks	exon	3564	3661	.	-	.	gene_id "XLOC_257048"; transcript_id "Novel_003627"; exon_number "6"; gene_type "
scaffold1	Cufflinks	exon	3755	4493	.	-	.	gene_id "XLOC_257048"; transcript_id "Novel_003627"; exon_number "7"; gene_type "
scaffold1	Cufflinks	exon	204929	206124	.	+	.	gene_id "XLOC_257133"; transcript_id "Novel_003628"; exon_number "1"; gene_type "
scaffold1	Cufflinks	exon	206226	206365	.	+	.	gene_id "XLOC_257133"; transcript_id "Novel_003628"; exon_number "2"; gene_type "
scaffold1	Cufflinks	exon	206951	207032	.	+	.	gene_id "XLOC_257133"; transcript_id "Novel_003628"; exon_number "3"; gene_type "
scaffold1	Cufflinks	exon	207144	207557	.	+	.	gene_id "XLOC_257133"; transcript_id "Novel_003628"; exon_number "4"; gene_type "
scaffold1	Cufflinks	exon	204934	205007	.	+	.	gene_id "XLOC_257133"; transcript_id "Novel_003629"; exon_number "1"; gene_type "
scaffold1	Cufflinks	exon	205328	205396	.	+	.	gene_id "XLOC_257133"; transcript_id "Novel_003629"; exon_number "2"; gene_type "
scaffold1	Cufflinks	exon	206024	206124	.	+	.	gene_id "XLOC_257133"; transcript_id "Novel_003629"; exon_number "3"; gene_type "
scaffold1	Cufflinks	exon	206226	206365	.	+	.	gene_id "XLOC_257133"; transcript_id "Novel_003629"; exon_number "4"; gene_type "
scaffold1	Cufflinks	exon	206951	207032	.	+	.	gene_id "XLOC_257133"; transcript_id "Novel_003629"; exon_number "5"; gene_type "
scaffold1	Cufflinks	exon	207144	207965	.	+	.	gene_id "XLOC_257133"; transcript_id "Novel_003629"; exon_number "6"; gene_type "
scaffold1	Cufflinks	exon	706704	706727	.	+	.	gene_id "XLOC_257215"; transcript_id "Novel_003630"; exon_number "1"; gene_type "
scaffold1	Cufflinks	exon	709004	709210	.	+	.	gene_id "XLOC_257215"; transcript_id "Novel_003630"; exon_number "2"; gene_type "
scaffold1	Cufflinks	exon	710142	710234	.	+	.	gene_id "XLOC_257215"; transcript_id "Novel_003630"; exon_number "3"; gene_type "
scaffold1	Cufflinks	exon	710637	711728	.	+	.	gene_id "XLOC_257215"; transcript_id "Novel_003630"; exon_number "4"; gene_type "
scaffold1	Cufflinks	exon	707659	709210	.	+	.	gene_id "XLOC_257215"; transcript_id "Novel_003631"; exon_number "1"; gene_type "
scaffold1	Cufflinks	exon	710142	710234	.	+	.	gene_id "XLOC_257215"; transcript_id "Novel_003631"; exon_number "2"; gene_type "
scaffold1	Cufflinks	exon	710637	711405	.	+	.	gene_id "XLOC_257215"; transcript_id "Novel_003631"; exon_number "3"; gene_type "
scaffold1	Cufflinks	exon	710058	710234	.	+	.	gene_id "XLOC_257215"; transcript_id "Novel_003632"; exon_number "1"; gene_type "
scaffold1	Cufflinks	exon	710637	711588	.	+	.	gene_id "XLOC_257215"; transcript_id "Novel_003632"; exon_number "2"; gene_type "
scaffold1	Cufflinks	exon	1179805	1179910	.	+	.	gene_id "XLOC_257303"; transcript_id "Novel_003633"; exon_number "1"; gene_type "

scaffold1Cufflinks	exon	1180003	1180265	.	+	.	gene_id "XLOC_257303"; transcript_id "Novel_003633"; exon_number "2"; gene_type "
scaffold1Cufflinks	exon	151769	153278	.	-	.	gene_id "XLOC_257428"; transcript_id "Novel_003634"; exon_number "1"; gene_type "
scaffold1Cufflinks	exon	153671	154231	.	-	.	gene_id "XLOC_257428"; transcript_id "Novel_003634"; exon_number "2"; gene_type "
scaffold1Cufflinks	exon	548168	548921	.	-	.	gene_id "XLOC_257510"; transcript_id "Novel_003635"; exon_number "1"; gene_type "
scaffold1Cufflinks	exon	161699	161926	.	-	.	gene_id "XLOC_257913"; transcript_id "Novel_003636"; exon_number "1"; gene_type "
scaffold1Cufflinks	exon	237079	238587	.	+	.	gene_id "XLOC_257977"; transcript_id "Novel_003637"; exon_number "1"; gene_type "
scaffold1Cufflinks	exon	238884	240317	.	+	.	gene_id "XLOC_257977"; transcript_id "Novel_003637"; exon_number "2"; gene_type "
scaffold1Cufflinks	exon	31184	31368	.	-	.	gene_id "XLOC_258091"; transcript_id "Novel_003638"; exon_number "1"; gene_type "
scaffold1Cufflinks	exon	31477	31567	.	-	.	gene_id "XLOC_258091"; transcript_id "Novel_003638"; exon_number "2"; gene_type "
scaffold1Cufflinks	exon	31673	31780	.	-	.	gene_id "XLOC_258091"; transcript_id "Novel_003638"; exon_number "3"; gene_type "
scaffold1Cufflinks	exon	32318	32483	.	-	.	gene_id "XLOC_258091"; transcript_id "Novel_003638"; exon_number "4"; gene_type "
scaffold1Cufflinks	exon	31190	31368	.	-	.	gene_id "XLOC_258091"; transcript_id "Novel_003639"; exon_number "1"; gene_type "
scaffold1Cufflinks	exon	31477	31567	.	-	.	gene_id "XLOC_258091"; transcript_id "Novel_003639"; exon_number "2"; gene_type "
scaffold1Cufflinks	exon	31673	31890	.	-	.	gene_id "XLOC_258091"; transcript_id "Novel_003639"; exon_number "3"; gene_type "
scaffold1Cufflinks	exon	20114	20362	.	+	.	gene_id "XLOC_258148"; transcript_id "Novel_003640"; exon_number "1"; gene_type "
scaffold1Cufflinks	exon	20464	20548	.	+	.	gene_id "XLOC_258148"; transcript_id "Novel_003640"; exon_number "2"; gene_type "
scaffold1Cufflinks	exon	20731	21419	.	+	.	gene_id "XLOC_258148"; transcript_id "Novel_003640"; exon_number "3"; gene_type "
scaffold1Cufflinks	exon	20147	20362	.	+	.	gene_id "XLOC_258148"; transcript_id "Novel_003641"; exon_number "1"; gene_type "
scaffold1Cufflinks	exon	20464	20548	.	+	.	gene_id "XLOC_258148"; transcript_id "Novel_003641"; exon_number "2"; gene_type "
scaffold1Cufflinks	exon	20731	20877	.	+	.	gene_id "XLOC_258148"; transcript_id "Novel_003641"; exon_number "3"; gene_type "
scaffold1Cufflinks	exon	21451	21675	.	+	.	gene_id "XLOC_258148"; transcript_id "Novel_003641"; exon_number "4"; gene_type "
scaffold1Cufflinks	exon	21794	21808	.	+	.	gene_id "XLOC_258148"; transcript_id "Novel_003641"; exon_number "5"; gene_type "
scaffold1Cufflinks	exon	21924	21976	.	+	.	gene_id "XLOC_258148"; transcript_id "Novel_003642"; exon_number "1"; gene_type "
scaffold1Cufflinks	exon	22101	22324	.	+	.	gene_id "XLOC_258148"; transcript_id "Novel_003642"; exon_number "2"; gene_type "
scaffold1Cufflinks	exon	22583	22720	.	+	.	gene_id "XLOC_258148"; transcript_id "Novel_003642"; exon_number "3"; gene_type "
scaffold1Cufflinks	exon	22877	24669	.	+	.	gene_id "XLOC_258148"; transcript_id "Novel_003642"; exon_number "4"; gene_type "
scaffold1Cufflinks	exon	230376	230562	.	+	.	gene_id "XLOC_258163"; transcript_id "Novel_003643"; exon_number "1"; gene_type "
scaffold1Cufflinks	exon	230659	230702	.	+	.	gene_id "XLOC_258163"; transcript_id "Novel_003643"; exon_number "2"; gene_type "
scaffold1Cufflinks	exon	230791	230886	.	+	.	gene_id "XLOC_258163"; transcript_id "Novel_003643"; exon_number "3"; gene_type "
scaffold1Cufflinks	exon	231208	231522	.	+	.	gene_id "XLOC_258163"; transcript_id "Novel_003643"; exon_number "4"; gene_type "
scaffold1Cufflinks	exon	230383	230702	.	+	.	gene_id "XLOC_258163"; transcript_id "Novel_003644"; exon_number "1"; gene_type "
scaffold1Cufflinks	exon	230791	230886	.	+	.	gene_id "XLOC_258163"; transcript_id "Novel_003644"; exon_number "2"; gene_type "
scaffold1Cufflinks	exon	231208	231499	.	+	.	gene_id "XLOC_258163"; transcript_id "Novel_003644"; exon_number "3"; gene_type "
scaffold1Cufflinks	exon	231542	231891	.	+	.	gene_id "XLOC_258163"; transcript_id "Novel_003645"; exon_number "1"; gene_type "
scaffold1Cufflinks	exon	231986	232163	.	+	.	gene_id "XLOC_258163"; transcript_id "Novel_003645"; exon_number "2"; gene_type "

scaffold1Cufflinks	exon	232247	232290	.	+	.	gene_id "XLOC_258163"; transcript_id "Novel_003645"; exon_number "3"; gene_type "
scaffold1Cufflinks	exon	232444	232657	.	+	.	gene_id "XLOC_258163"; transcript_id "Novel_003645"; exon_number "4"; gene_type "
scaffold1Cufflinks	exon	232885	233481	.	+	.	gene_id "XLOC_258163"; transcript_id "Novel_003645"; exon_number "5"; gene_type "
scaffold1Cufflinks	exon	231823	232163	.	+	.	gene_id "XLOC_258163"; transcript_id "Novel_003646"; exon_number "1"; gene_type "
scaffold1Cufflinks	exon	232247	232290	.	+	.	gene_id "XLOC_258163"; transcript_id "Novel_003646"; exon_number "2"; gene_type "
scaffold1Cufflinks	exon	232444	232657	.	+	.	gene_id "XLOC_258163"; transcript_id "Novel_003646"; exon_number "3"; gene_type "
scaffold1Cufflinks	exon	232885	233611	.	+	.	gene_id "XLOC_258163"; transcript_id "Novel_003646"; exon_number "4"; gene_type "
scaffold1Cufflinks	exon	231985	232163	.	+	.	gene_id "XLOC_258163"; transcript_id "Novel_003647"; exon_number "1"; gene_type "
scaffold1Cufflinks	exon	232247	232657	.	+	.	gene_id "XLOC_258163"; transcript_id "Novel_003647"; exon_number "2"; gene_type "
scaffold1Cufflinks	exon	232885	233276	.	+	.	gene_id "XLOC_258163"; transcript_id "Novel_003647"; exon_number "3"; gene_type "
scaffold1Cufflinks	exon	258867	258969	.	+	.	gene_id "XLOC_258168"; transcript_id "Novel_003648"; exon_number "1"; gene_type "
scaffold1Cufflinks	exon	259053	259159	.	+	.	gene_id "XLOC_258168"; transcript_id "Novel_003648"; exon_number "2"; gene_type "
scaffold1Cufflinks	exon	260245	260323	.	+	.	gene_id "XLOC_258168"; transcript_id "Novel_003648"; exon_number "3"; gene_type "
scaffold1Cufflinks	exon	260421	261409	.	+	.	gene_id "XLOC_258168"; transcript_id "Novel_003648"; exon_number "4"; gene_type "
scaffold1Cufflinks	exon	285755	286741	.	+	.	gene_id "XLOC_258172"; transcript_id "Novel_003649"; exon_number "1"; gene_type "
scaffold1Cufflinks	exon	286886	287477	.	+	.	gene_id "XLOC_258172"; transcript_id "Novel_003649"; exon_number "2"; gene_type "
scaffold1Cufflinks	exon	161210	162704	.	-	.	gene_id "XLOC_258221"; transcript_id "Novel_003650"; exon_number "1"; gene_type "
scaffold1Cufflinks	exon	162942	162967	.	-	.	gene_id "XLOC_258221"; transcript_id "Novel_003650"; exon_number "2"; gene_type "
scaffold1Cufflinks	exon	165291	167480	.	-	.	gene_id "XLOC_258222"; transcript_id "Novel_003651"; exon_number "1"; gene_type "
scaffold1Cufflinks	exon	167565	167708	.	-	.	gene_id "XLOC_258222"; transcript_id "Novel_003651"; exon_number "2"; gene_type "
scaffold1Cufflinks	exon	167861	167963	.	-	.	gene_id "XLOC_258222"; transcript_id "Novel_003651"; exon_number "3"; gene_type "
scaffold1Cufflinks	exon	168056	168166	.	-	.	gene_id "XLOC_258222"; transcript_id "Novel_003651"; exon_number "4"; gene_type "
scaffold1Cufflinks	exon	168265	169568	.	-	.	gene_id "XLOC_258222"; transcript_id "Novel_003651"; exon_number "5"; gene_type "
scaffold1Cufflinks	exon	189164	189884	.	-	.	gene_id "XLOC_258229"; transcript_id "Novel_003652"; exon_number "1"; gene_type "
scaffold1Cufflinks	exon	189973	190074	.	-	.	gene_id "XLOC_258229"; transcript_id "Novel_003652"; exon_number "2"; gene_type "
scaffold1Cufflinks	exon	190177	190407	.	-	.	gene_id "XLOC_258229"; transcript_id "Novel_003652"; exon_number "3"; gene_type "
scaffold1Cufflinks	exon	190501	190588	.	-	.	gene_id "XLOC_258229"; transcript_id "Novel_003652"; exon_number "4"; gene_type "
scaffold1Cufflinks	exon	190719	190807	.	-	.	gene_id "XLOC_258229"; transcript_id "Novel_003652"; exon_number "5"; gene_type "
scaffold1Cufflinks	exon	190901	190966	.	-	.	gene_id "XLOC_258229"; transcript_id "Novel_003652"; exon_number "6"; gene_type "
scaffold1Cufflinks	exon	191068	192854	.	-	.	gene_id "XLOC_258229"; transcript_id "Novel_003652"; exon_number "7"; gene_type "
scaffold1Cufflinks	exon	189164	189884	.	-	.	gene_id "XLOC_258229"; transcript_id "Novel_003653"; exon_number "1"; gene_type "
scaffold1Cufflinks	exon	189973	190074	.	-	.	gene_id "XLOC_258229"; transcript_id "Novel_003653"; exon_number "2"; gene_type "
scaffold1Cufflinks	exon	190177	190407	.	-	.	gene_id "XLOC_258229"; transcript_id "Novel_003653"; exon_number "3"; gene_type "
scaffold1Cufflinks	exon	190501	190588	.	-	.	gene_id "XLOC_258229"; transcript_id "Novel_003653"; exon_number "4"; gene_type "
scaffold1Cufflinks	exon	190719	190807	.	-	.	gene_id "XLOC_258229"; transcript_id "Novel_003653"; exon_number "5"; gene_type "

scaffold1	Cufflinks	exon	190901	190966	.	-	.	gene_id "XLOC_258229"; transcript_id "Novel_003653"; exon_number "6"; gene_type "
scaffold1	Cufflinks	exon	191068	191716	.	-	.	gene_id "XLOC_258229"; transcript_id "Novel_003653"; exon_number "7"; gene_type "
scaffold1	Cufflinks	exon	192040	192854	.	-	.	gene_id "XLOC_258229"; transcript_id "Novel_003653"; exon_number "8"; gene_type "
scaffold1	Cufflinks	exon	241059	241258	.	-	.	gene_id "XLOC_258232"; transcript_id "Novel_003654"; exon_number "1"; gene_type "
scaffold1	Cufflinks	exon	241346	241569	.	-	.	gene_id "XLOC_258232"; transcript_id "Novel_003654"; exon_number "2"; gene_type "
scaffold1	Cufflinks	exon	301280	302160	.	-	.	gene_id "XLOC_258241"; transcript_id "Novel_003655"; exon_number "1"; gene_type "
scaffold1	Cufflinks	exon	302251	302518	.	-	.	gene_id "XLOC_258241"; transcript_id "Novel_003655"; exon_number "2"; gene_type "
scaffold1	Cufflinks	exon	309540	309991	.	-	.	gene_id "XLOC_258245"; transcript_id "Novel_003656"; exon_number "1"; gene_type "
scaffold1	Cufflinks	exon	310682	311290	.	-	.	gene_id "XLOC_258245"; transcript_id "Novel_003656"; exon_number "2"; gene_type "
scaffold1	Cufflinks	exon	322487	323874	.	-	.	gene_id "XLOC_258248"; transcript_id "Novel_003657"; exon_number "1"; gene_type "
scaffold1	Cufflinks	exon	323997	324593	.	-	.	gene_id "XLOC_258248"; transcript_id "Novel_003657"; exon_number "2"; gene_type "
scaffold1	Cufflinks	exon	323533	323573	.	-	.	gene_id "XLOC_258248"; transcript_id "Novel_003658"; exon_number "1"; gene_type "
scaffold1	Cufflinks	exon	323678	323874	.	-	.	gene_id "XLOC_258248"; transcript_id "Novel_003658"; exon_number "2"; gene_type "
scaffold1	Cufflinks	exon	323997	324564	.	-	.	gene_id "XLOC_258248"; transcript_id "Novel_003658"; exon_number "3"; gene_type "
scaffold1	Cufflinks	exon	235953	237617	.	-	.	gene_id "XLOC_258379"; transcript_id "Novel_003659"; exon_number "1"; gene_type "
scaffold1	Cufflinks	exon	299699	299786	.	-	.	gene_id "XLOC_258433"; transcript_id "Novel_003660"; exon_number "1"; gene_type "
scaffold1	Cufflinks	exon	304831	304874	.	-	.	gene_id "XLOC_258433"; transcript_id "Novel_003660"; exon_number "2"; gene_type "
scaffold1	Cufflinks	exon	305005	305052	.	-	.	gene_id "XLOC_258433"; transcript_id "Novel_003660"; exon_number "3"; gene_type "
scaffold1	Cufflinks	exon	305141	305203	.	-	.	gene_id "XLOC_258433"; transcript_id "Novel_003660"; exon_number "4"; gene_type "
scaffold1	Cufflinks	exon	305396	305461	.	-	.	gene_id "XLOC_258433"; transcript_id "Novel_003660"; exon_number "5"; gene_type "
scaffold1	Cufflinks	exon	310589	310657	.	-	.	gene_id "XLOC_258433"; transcript_id "Novel_003660"; exon_number "6"; gene_type "
scaffold1	Cufflinks	exon	311484	311555	.	-	.	gene_id "XLOC_258433"; transcript_id "Novel_003660"; exon_number "7"; gene_type "
scaffold1	Cufflinks	exon	311647	311718	.	-	.	gene_id "XLOC_258433"; transcript_id "Novel_003660"; exon_number "8"; gene_type "
scaffold1	Cufflinks	exon	311825	311896	.	-	.	gene_id "XLOC_258433"; transcript_id "Novel_003660"; exon_number "9"; gene_type "
scaffold1	Cufflinks	exon	311998	312069	.	-	.	gene_id "XLOC_258433"; transcript_id "Novel_003660"; exon_number "10"; gene_type
scaffold1	Cufflinks	exon	312155	312819	.	-	.	gene_id "XLOC_258433"; transcript_id "Novel_003660"; exon_number "11"; gene_type
scaffold1	Cufflinks	exon	312792	312843	.	-	.	gene_id "XLOC_258433"; transcript_id "Novel_003661"; exon_number "1"; gene_type "
scaffold1	Cufflinks	exon	312952	313023	.	-	.	gene_id "XLOC_258433"; transcript_id "Novel_003661"; exon_number "2"; gene_type "
scaffold1	Cufflinks	exon	313411	313582	.	-	.	gene_id "XLOC_258433"; transcript_id "Novel_003661"; exon_number "3"; gene_type "
scaffold1	Cufflinks	exon	314238	314419	.	-	.	gene_id "XLOC_258433"; transcript_id "Novel_003661"; exon_number "4"; gene_type "
scaffold1	Cufflinks	exon	220327	220556	.	+	.	gene_id "XLOC_258592"; transcript_id "Novel_003662"; exon_number "1"; gene_type "
scaffold1	Cufflinks	exon	238538	238805	.	+	.	gene_id "XLOC_258666"; transcript_id "Novel_003663"; exon_number "1"; gene_type "
scaffold1	Cufflinks	exon	115422	116121	.	-	.	gene_id "XLOC_258690"; transcript_id "Novel_003664"; exon_number "1"; gene_type "
scaffold1	Cufflinks	exon	191700	192550	.	-	.	gene_id "XLOC_258695"; transcript_id "Novel_003665"; exon_number "1"; gene_type "
scaffold1	Cufflinks	exon	193301	193341	.	-	.	gene_id "XLOC_258695"; transcript_id "Novel_003665"; exon_number "2"; gene_type "

scaffold1	Cufflinks	exon	193707	194807	.	-	.	gene_id "XLOC_258695"; transcript_id "Novel_003665"; exon_number "3"; gene_type "
scaffold1	Cufflinks	exon	258054	258374	.	-	.	gene_id "XLOC_258706"; transcript_id "Novel_003666"; exon_number "1"; gene_type "
scaffold1	Cufflinks	exon	260608	260685	.	-	.	gene_id "XLOC_258706"; transcript_id "Novel_003666"; exon_number "2"; gene_type "
scaffold1	Cufflinks	exon	263473	263714	.	-	.	gene_id "XLOC_258707"; transcript_id "Novel_003667"; exon_number "1"; gene_type "
scaffold1	Cufflinks	exon	264789	265887	.	-	.	gene_id "XLOC_258707"; transcript_id "Novel_003667"; exon_number "2"; gene_type "
scaffold1	Cufflinks	exon	266107	266728	.	-	.	gene_id "XLOC_258707"; transcript_id "Novel_003667"; exon_number "3"; gene_type "
scaffold1	Cufflinks	exon	264408	265887	.	-	.	gene_id "XLOC_258707"; transcript_id "Novel_003668"; exon_number "1"; gene_type "
scaffold1	Cufflinks	exon	266107	266688	.	-	.	gene_id "XLOC_258707"; transcript_id "Novel_003668"; exon_number "2"; gene_type "
scaffold1	Cufflinks	exon	272041	272290	.	-	.	gene_id "XLOC_258711"; transcript_id "Novel_003669"; exon_number "1"; gene_type "
scaffold1	Cufflinks	exon	273052	273390	.	-	.	gene_id "XLOC_258711"; transcript_id "Novel_003669"; exon_number "2"; gene_type "
scaffold1	Cufflinks	exon	273935	274042	.	-	.	gene_id "XLOC_258711"; transcript_id "Novel_003670"; exon_number "1"; gene_type "
scaffold1	Cufflinks	exon	274130	274330	.	-	.	gene_id "XLOC_258711"; transcript_id "Novel_003670"; exon_number "2"; gene_type "
scaffold2	Cufflinks	exon	263060	264840	.	+	.	gene_id "XLOC_259187"; transcript_id "Novel_003671"; exon_number "1"; gene_type "
scaffold2	Cufflinks	exon	265448	266074	.	+	.	gene_id "XLOC_259187"; transcript_id "Novel_003671"; exon_number "2"; gene_type "
scaffold2	Cufflinks	exon	18067	18775	.	-	.	gene_id "XLOC_259313"; transcript_id "Novel_003672"; exon_number "1"; gene_type "
scaffold2	Cufflinks	exon	18866	19414	.	-	.	gene_id "XLOC_259313"; transcript_id "Novel_003672"; exon_number "2"; gene_type "
scaffold2	Cufflinks	exon	79224	79730	.	+	.	gene_id "XLOC_259415"; transcript_id "Novel_003673"; exon_number "1"; gene_type "
scaffold2	Cufflinks	exon	79869	79931	.	+	.	gene_id "XLOC_259415"; transcript_id "Novel_003673"; exon_number "2"; gene_type "
scaffold2	Cufflinks	exon	80049	80123	.	+	.	gene_id "XLOC_259415"; transcript_id "Novel_003673"; exon_number "3"; gene_type "
scaffold2	Cufflinks	exon	80236	80851	.	+	.	gene_id "XLOC_259415"; transcript_id "Novel_003673"; exon_number "4"; gene_type "
scaffold2	Cufflinks	exon	281148	281449	.	+	.	gene_id "XLOC_259452"; transcript_id "Novel_003674"; exon_number "1"; gene_type "
scaffold2	Cufflinks	exon	281539	281608	.	+	.	gene_id "XLOC_259452"; transcript_id "Novel_003674"; exon_number "2"; gene_type "
scaffold2	Cufflinks	exon	285700	285749	.	+	.	gene_id "XLOC_259452"; transcript_id "Novel_003674"; exon_number "3"; gene_type "
scaffold2	Cufflinks	exon	286606	286646	.	+	.	gene_id "XLOC_259452"; transcript_id "Novel_003674"; exon_number "4"; gene_type "
scaffold2	Cufflinks	exon	287333	288561	.	+	.	gene_id "XLOC_259452"; transcript_id "Novel_003674"; exon_number "5"; gene_type "
scaffold2	Cufflinks	exon	283361	283793	.	+	.	gene_id "XLOC_259452"; transcript_id "Novel_003675"; exon_number "1"; gene_type "
scaffold2	Cufflinks	exon	285700	285749	.	+	.	gene_id "XLOC_259452"; transcript_id "Novel_003675"; exon_number "2"; gene_type "
scaffold2	Cufflinks	exon	286606	286646	.	+	.	gene_id "XLOC_259452"; transcript_id "Novel_003675"; exon_number "3"; gene_type "
scaffold2	Cufflinks	exon	287333	288337	.	+	.	gene_id "XLOC_259452"; transcript_id "Novel_003675"; exon_number "4"; gene_type "
scaffold2	Cufflinks	exon	119190	119412	.	-	.	gene_id "XLOC_259566"; transcript_id "Novel_003676"; exon_number "1"; gene_type "
scaffold2	Cufflinks	exon	119599	119666	.	-	.	gene_id "XLOC_259566"; transcript_id "Novel_003676"; exon_number "2"; gene_type "
scaffold2	Cufflinks	exon	120174	120271	.	-	.	gene_id "XLOC_259566"; transcript_id "Novel_003676"; exon_number "3"; gene_type "
scaffold2	Cufflinks	exon	120850	120911	.	-	.	gene_id "XLOC_259566"; transcript_id "Novel_003676"; exon_number "4"; gene_type "
scaffold2	Cufflinks	exon	121021	121164	.	-	.	gene_id "XLOC_259566"; transcript_id "Novel_003676"; exon_number "5"; gene_type "
scaffold2	Cufflinks	exon	121393	121757	.	-	.	gene_id "XLOC_259566"; transcript_id "Novel_003676"; exon_number "6"; gene_type "

scaffold2	Cufflinks	exon	290639	291248	.	-	.	gene_id "XLOC_259576"; transcript_id "Novel_003677"; exon_number "1"; gene_type "
scaffold2	Cufflinks	exon	291627	291716	.	-	.	gene_id "XLOC_259576"; transcript_id "Novel_003677"; exon_number "2"; gene_type "
scaffold2	Cufflinks	exon	291840	292168	.	-	.	gene_id "XLOC_259576"; transcript_id "Novel_003677"; exon_number "3"; gene_type "
scaffold2	Cufflinks	exon	292340	292499	.	-	.	gene_id "XLOC_259576"; transcript_id "Novel_003677"; exon_number "4"; gene_type "
[truncated: 433,956 more chars]
